# From rivers to ocean basins: The role of ocean barriers and philopatry in the genetic structuring of a cosmopolitan coastal predator

**DOI:** 10.1002/ece3.9837

**Published:** 2023-02-22

**Authors:** Floriaan Devloo‐Delva, Christopher P. Burridge, Peter M. Kyne, Juerg M. Brunnschweiler, Demian D. Chapman, Patricia Charvet, Xiao Chen, Geremy Cliff, Ryan Daly, J. Marcus Drymon, Mario Espinoza, Daniel Fernando, Laura Garcia Barcia, Kerstin Glaus, Blanca I. González‐Garza, Michael I. Grant, Rasanthi M. Gunasekera, Sebastian Hernandez, Susumu Hyodo, Rima W. Jabado, Sébastien Jaquemet, Grant Johnson, James T. Ketchum, Hélène Magalon, James R. Marthick, Frederik H. Mollen, Stefano Mona, Gavin J. P. Naylor, John E. G. Nevill, Nicole M. Phillips, Richard D. Pillans, Bautisse D. Postaire, Amy F. Smoothey, Katsunori Tachihara, Bree J. Tillet, Jorge A. Valerio‐Vargas, Pierre Feutry

**Affiliations:** ^1^ Oceans and Atmosphere, CSIRO Hobart Tasmania Australia; ^2^ Quantitative Marine Science, Institute for Marine and Antarctic Studies, University of Tasmania Hobart Tasmania Australia; ^3^ Discipline of Biological Sciences, School of Natural Sciences University of Tasmania Hobart Tasmania Australia; ^4^ Research Institute for the Environment and Livelihoods Charles Darwin University Darwin Northern Territory Australia; ^5^ Independent Researcher Zurich Switzerland; ^6^ Department of Biological Sciences Florida International University North Miami Florida USA; ^7^ Programa de Pós‐graduação em Sistemática, Uso e Conservação da Biodiversidade Universidade Federal do Ceará (PPGSis ‐ UFC) Fortaleza Brazil; ^8^ College of Veterinary Medicine South China Agricultural University Guangzhou China; ^9^ KwaZulu‐Natal Sharks Board, Umhlanga 4320, South Africa and School of Life Sciences University of KwaZulu‐Natal Durban South Africa; ^10^ Oceanographic Research Institute, South African Association for Marine Biological Research, Point Durban South Africa; ^11^ South African Institute for Aquatic Biodiversity Mkhanda South Africa; ^12^ Coastal Research and Extension Center Mississippi State University Biloxi Mississippi USA; ^13^ Mississippi‐Alabama Sea Grant Consortium Ocean Springs Mississippi USA; ^14^ Centro de Investigación en Ciencias del Mar y Limnología & Escuela de Biología Universidad de Costa Rica, San Pedro de Montes de Oca San José Costa Rica; ^15^ Blue Resources Trust Colombo Sri Lanka; ^16^ Faculty of Science, Technology and Environment, School of Marine Studies The University of the South Pacific Suva Fiji; ^17^ Pelagios‐Kakunja La Paz Mexico; ^18^ College of Science and Engineering, Centre for Sustainable Tropical Fisheries and Aquaculture James Cook University Townsville Queensland Australia; ^19^ Biomolecular Laboratory, Center for International Programs Universidad VERITAS San José Costa Rica; ^20^ Sala de Colecciones, Facultad de Ciencias del Mar Universidad Católica del Norte Coquimbo Chile; ^21^ Laboratory of Physiology, Atmosphere and Ocean Research Institute University of Tokyo Kashiwa, Chiba Japan; ^22^ Elasmo Project Dubai United Arab Emirates; ^23^ UMR ENTROPIE (Université de La Réunion, Université de Nouvelle‐Calédonie, IRD, CNRS, IFREMER), Faculté des Sciences et Technologies Université de La Réunion Cedex 09, La Réunion France; ^24^ Department of Industry, Tourism and Trade, Aquatic Resource Research Unit Darwin Northern Territory Australia; ^25^ MigraMar Olema California USA; ^26^ Menzies Institute for Medical Research University of Tasmania Hobart Tasmania Australia; ^27^ Elasmobranch Research Bonheiden Belgium; ^28^ Institut de Systématique, Evolution, Biodiversité, ISYEB (UMR 7205), Muséum National d'Histoire Naturelle, CNRS, Sorbonne Université, EPHE Université des Antilles Paris France; ^29^ EPHE PSL Research University Paris France; ^30^ Florida Museum of Natural History University of Florida Gainesville Florida USA; ^31^ Environment Seychelles Victoria Seychelles; ^32^ School of Biological, Environmental and Earth Sciences The University of Southern Mississippi Hattiesburg Mississippi USA; ^33^ Oceans and Atmosphere, CSIRO Dutton Park Queensland Australia; ^34^ NSW Department of Primary Industries, Fisheries Research Sydney Institute of Marine Science Mosman New South Wales Australia; ^35^ Laboratory of Fisheries Biology and Coral Reef Studies, Faculty of Science University of Ryukyus, Nishihara Okinawa Japan; ^36^ Translational Research Institute, University of Queensland Diamantina Institute Brisbane Queensland Australia

**Keywords:** close‐kin, DArTseq, DNA forensics, genetic connectivity, mitogenome, provenance

## Abstract

The Bull Shark (*Carcharhinus leucas*) faces varying levels of exploitation around the world due to its coastal distribution. Information regarding population connectivity is crucial to evaluate its conservation status and local fishing impacts. In this study, we sampled 922 putative Bull Sharks from 19 locations in the first global assessment of population structure of this cosmopolitan species. Using a recently developed DNA‐capture approach (DArTcap), samples were genotyped for 3400 nuclear markers. Additionally, full mitochondrial genomes of 384 Indo‐Pacific samples were sequenced. Reproductive isolation was found between and across ocean basins (eastern Pacific, western Atlantic, eastern Atlantic, Indo‐West Pacific) with distinct island populations in Japan and Fiji. Bull Sharks appear to maintain gene flow using shallow coastal waters as dispersal corridors, whereas large oceanic distances and historical land‐bridges act as barriers. Females tend to return to the same area for reproduction, making them more susceptible to local threats and an important focus for management actions. Given these behaviors, the exploitation of Bull Sharks from insular populations, such as Japan and Fiji, may instigate local decline that cannot readily be replenished by immigration, which can in turn affect ecosystem dynamics and functions. These data also supported the development of a genetic panel to ascertain the population of origin, which will be useful in monitoring the trade of fisheries products and assessing population‐level impacts of this harvest.

## Graphical abstract

In this study, 922 putative Bull Sharks were obtained from 19 locations for the first global assessment of population structure of this cosmopolitan coastal species. Nuclear DNA identified distinct populations in each ocean basin, with distinct island populations in Japan and Fiji. While nuclear DNA indicated that the Indo‐West Pacific is well‐connected, mitochondrial DNA—only inherited from the mother—showed that females return to the same locations for breeding.

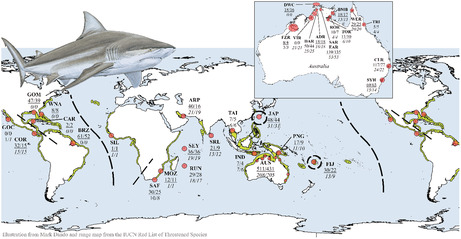

## 1 INTRODUCTION

Understanding the population structure of a species and the barriers that disrupt dispersal is important to accurately assess the global conservation status and manage the risk of local extinction. This is especially true for species of commercial importance (Begg et al., 1999) or conservation concern (Moritz, 1994), which are impacted disproportionally by anthropogenic or environmental pressures. Dispersal can promote genetic connectivity across patches of suitable habitat (Ronce, 2007), but physical barriers and behaviors can ultimately limit gene flow, even when dispersal potential is high, and result in demographically independent populations (Waples & Gaggiotti, 2006). When genetically isolated populations are reduced in size due to unsustainable harvest, there is a risk of inbreeding depression and the loss of genetic diversity, without the chance to be ‘rescued’ by individuals dispersing from adjacent populations, increasing the likelihood of population extinctions (Frankham et al., 2017).

In marine taxa, gene flow tends to be restricted by environmental or biogeographic barriers, movement ecologies, and habitat preferences (Bowen et al., 2016; Dudgeon et al., 2012; Hirschfeld et al., 2021; Rocha et al., 2007). In large‐bodied coastal species with global distributions, such as marine turtles, cetaceans, and many elasmobranchs (sharks and rays), large‐scale marine biogeographic barriers shape population genetic structure (Dutton et al., 1999; Fontaine et al., 2007). For example, the Scalloped Hammerhead (*Sphyrna lewini*) shows genetic connectivity along the continental margins, yet limited gene flow across the East Pacific barrier, the Mid‐Atlantic barrier, and the Isthmus of Panama (Daly‐Engel et al., 2012; Green, Appleyard, et al., 2022). The permeability of environmental barriers, such as the Indo‐Australian Archipelago, changes across time and space, and consequently determines the observed distribution of genetic variation (Cowman & Bellwood, 2013). The effect of these ocean barriers on the spatial structuring of populations is essential knowledge for management, given that threats, such as overfishing and habitat modification, should be assessed and managed at biologically relevant spatial scales. For vagile marine taxa this often requires cooperative strategies between nations (e.g., International Whaling Commission, Indian Ocean Tuna Commission, and Western and Central Pacific Fisheries Commission).

Delineation of population structure in species with high mobilities and large population sizes have recently been improved with the use of genomic data (Layton et al., 2020; Luikart et al., 2019; Oleksiak & Rajora, 2020; Ovenden et al., 2018). Complexity‐reduction genome‐scan methods, such as Diversity Arrays Technology sequencing (DArTseq, Jaccoud et al., 2001) and targeted DNA‐capture approaches, including Rapture and DArTcap (Ali et al., 2016; Feutry et al., 2020), have been widely used to assess genetic diversity and reproductive connectivity in natural populations (e.g., Green et al., 2019; Komoroske et al., 2019). Recently, these methods have also been applied to DNA forensics or traceability studies in nonmodel species, with the objective to identify species, sex, provenance, and close‐kin relationships (e.g., Arenas et al., 2017; Feutry et al., 2017; Nielsen et al., 2012; Stovall et al., 2018). Moreover, in taxa with slow mitochondrial DNA (mtDNA) mutation rates, such as elasmobranchs and marine turtles (Avise et al., 1992; Martin et al., 1992), the sequencing of full mitochondrial genomes (mitogenomes) instead of single genes has improved the fine‐scale resolution of matrilineal population structure (Feutry et al., 2014).

The Bull Shark (*Carcharhinus leucas*) is a cosmopolitan species that occupies tropical, subtropical, and temperate coastal waters and has an important ecological role in freshwater, estuarine, and marine environments (Matich et al., 2011; Smoothey et al., 2019; Trystram et al., 2017). This species experiences variable degrees of exploitation within its range and is assessed as Vulnerable on the IUCN Red List of Threatened Species (Rigby et al., 2021). The main threats affecting Bull Sharks are small‐ and large‐scale fisheries for meat and fins (Glaus et al., 2015; Holmes et al., 2009), and shark control programs which directly target this species (Blaison et al., 2015; Dudley & Simpfendorfer, 2006; Niella et al., 2021). Because of its global catch, the Bull Shark is also frequently found in the international shark fin and meat trade, raising questions regarding the origin and trade routes of the fished products (Cardeñosa et al., 2022; Clarke et al., 2006; Fields et al., 2018). Unsustainable exploitation may result in population declines and negative ecosystem impacts (Ferretti et al., 2010; MacNeil et al., 2020).

The Bull Shark has shown capacity for long‐distance coastal movement (Brunnschweiler et al., 2010; Daly et al., 2014; Espinoza et al., 2016, 2021; Heupel et al., 2015; Lea et al., 2015) and genetic connectivity along continental shelves (Glaus et al., 2020; Pirog et al., 2019; Testerman, 2014). However, females exhibit reproductive philopatry to estuarine habitats at small spatial scales (~100 km; Karl et al., 2011; Sandoval Laurrabaquio‐Alvarado et al., 2021; Tillett et al., 2012). Genetic connectivity of Bull Sharks has been studied within specific regions, such as the western Atlantic (Karl et al., 2011; Sandoval Laurrabaquio‐Alvarado et al., 2019, 2021) and the Indo‐West Pacific (Deng et al., 2019; Glaus et al., 2020; Kitamura et al., 1996; Pirog et al., 2019; Tillett et al., 2012). Yet, no studies have investigated genetic connectivity across its global distribution, which can provide crucial data to assess the population‐level impacts of threats and identify the origin of fisheries products.

This study aims to apply genomic techniques (full mitogenomes and nuclear Single Nucleotide Polymorphisms or SNPs) to investigate the population genetic structure of Bull Sharks at global and local scales. We hypothesize that gene flow is limited by large‐scale biogeographic barriers, but that connectivity occurs along continuous coastlines. We further evaluate the forensic power of genomic data to assign sample provenance and establish a diagnostic SNP panel to aid monitoring the origin and trade of fisheries products and assess population‐level impacts of this trade. To achieve these goals, we analyze a global set of samples with three different genomic sequencing approaches (DArTseq, DArTcap, and mitogenomes), which allows us to assess population structure and gene flow across putative barriers for dispersal (such as open‐ocean expanses, strong temperature gradients, and historical land‐bridges). At a smaller spatial scale, contemporary reproductive connectivity is estimated by examining the spatial distribution of closely related individuals, such as full siblings and cross‐cohort half siblings.

## 2 MATERIALS AND METHODS

### 2.1 Sample collection and DNA extraction

**FIGURE 1:**
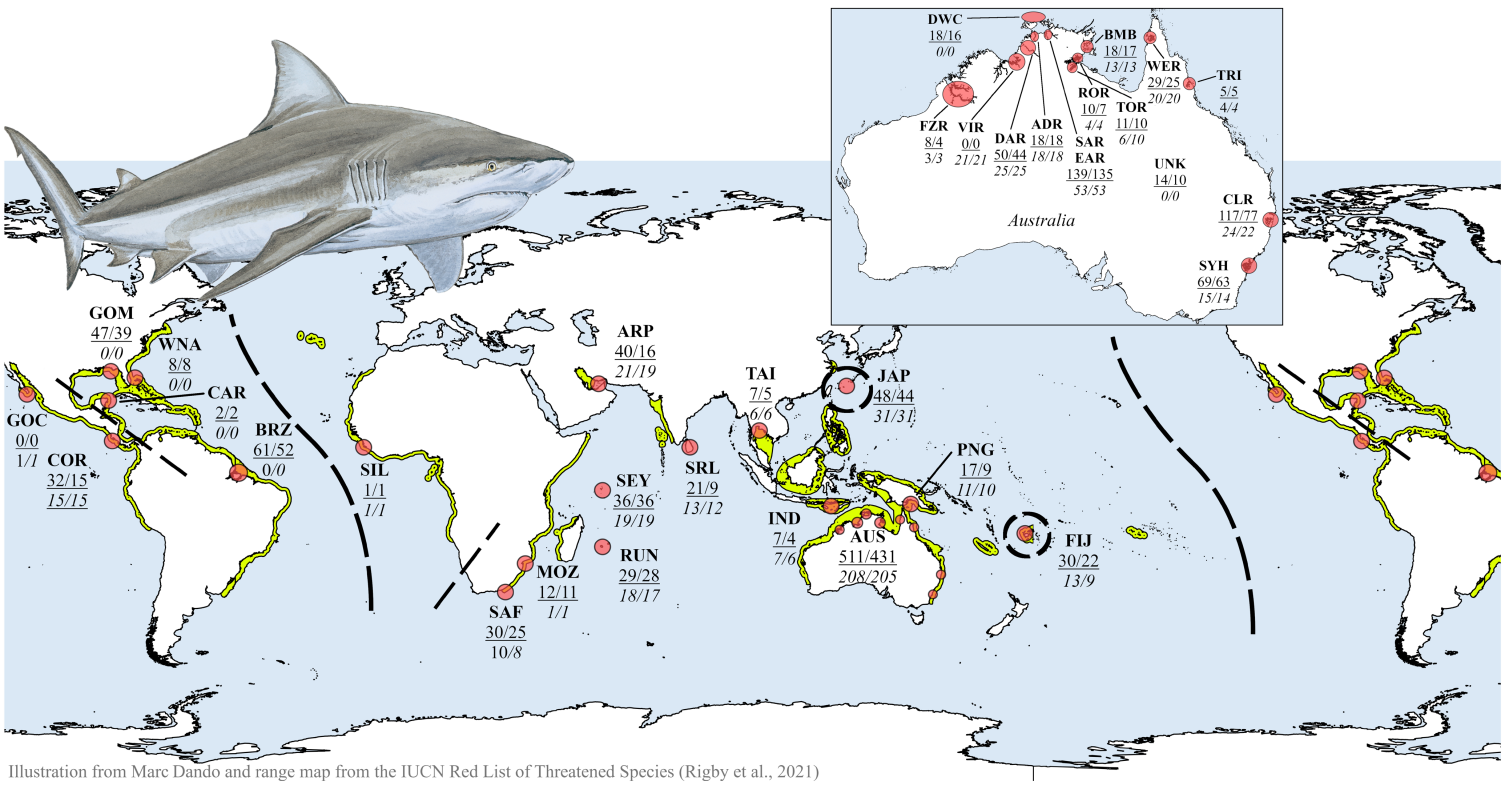
Map indicating the *Carcharhinus leucas* sampling locations with red circles and the known species range distribution in yellow. The sample sizes for the SNP data are underlined with the number of samples before/after data filtering. The sample sizes for the mitogenome data are in *italics* with the number of samples before/after data filtering. Putative barriers for gene flow are indicated by dashed lines. GOC, Gulf of California; COR, Costa Rica; BRZ, Brazil; CAR, Caribbean Sea; GOM, Gulf of Mexico; WNA, Western North Atlantic; SIL, Sierra Leone; SAF, South Africa; MOZ, Mozambique; RUN, Réunion Island; SEY, Seychelles; ARP, Arabian Peninsula; SRL, Sri Lanka; TAI, Thailand; IND, Indonesia; PNG, Papua New Guinea; AUS, Australia; JAP, Japan; and FIJ, Fiji. Australian sampling locations were presented as an additional inset: FZR, Fitzroy River; VIR, Victoria River; DAR, Daly River; ADR, Adelaide River; DWC, Darwin Coastal; SAR, South Alligator River; EAR, East Alligator River; BMB, Blue Mud Bay; ROR, Roper River; TOR, Towns River; WER, Wenlock River; TRI, Trinity Inlet; CLR, Clarence River; SYH, Sydney Harbor; and UNK, Australian fisheries samples from unknown origin.

A total of 922 putative Bull Shark samples (muscle or fin clip) were collected between 1980 and 2019 from 19 different countries or water bodies (termed ‘sampling locations’) around the globe in all major ocean basin regions: the eastern Pacific (E‐PAC), western Atlantic (W‐ATL), eastern Atlantic (E‐ATL), and the Indo‐West Pacific (IWP; Figure 1). Samples from Brazil, eastern Indian Ocean (E‐IO), Fiji, and numerous Australian samples were sourced from previously published genetic studies (*n* = 175; Glaus et al., 2020; Karl et al., 2011; Pirog et al., 2019; Tillett et al., 2012) while all other samples were novel. The samples from Japan included 10 individuals from the aquarium (Okinawa, locally sourced animals) with known pedigree and 38 wild‐caught samples from the Urauchi River. Each sampling location had an approximately equal sex ratio, and total length (TL) ranged from 26.4 (in utero) to 406.0 cm (Supporting Information section 2). Sharks smaller than 150 cm TL (68% of all individuals) were considered juveniles with limited dispersal capacity (Heupel et al., 2015; Pillans & Franklin, 2004; Simpfendorfer & Milward, 1993). DNA was extracted using the Qiagen Blood and Tissue kit following the standard protocol (Qiagen Inc., Valencia, California, USA). DNA quality and quantity was assessed on a 1% agarose gel, stained with SYBR safe (Invitrogen, USA), and with the NanoDrop ND‐8000 spectrophotometer (Thermo Fisher Scientific, Waltham, Massachusetts, USA).

### 2.2 SNP bait design and DArTcap genotyping

Initially, 188 samples with a minimum of 10 samples per location were genotyped following the DArTseq approach (according to Feutry et al., 2017). De novo SNP calling was performed with a proprietary software (DArTsoft14). The resulting markers were filtered using the ‘filter_rad’ function, as implemented in *radiator* v1.1.5 (Gosselin et al., 2020). Filtering thresholds were chosen based on the empirical distribution of each filtering parameter (see Supporting Information sections 4–6). Briefly, the data were filtered for: (1) low DArT reproducibility based on replicated libraries (technical replicates), (2) monomorphic markers, (3) high degree of missing data, high levels of inferred heterozygosity, and low sequencing coverage per individual, (4) low minor allele count, (5) low and high SNP sequencing coverage to avoid unreliable SNP calls or paralogous sequences, (6) high missing data per SNP, (7) too many SNPs per sequence due to suboptimal sequence clustering, (8) short‐distance linkage by keeping one SNP per sequence, (9) individual DNA contamination based on high proportion of heterozygous SNPs, (10) duplicated samples, and (11) departure from Hardy–Weinberg equilibrium. We further filtered out RAD‐tags that were too short (<60 bp), with low complexity (>6 nucleotide repeats), or with high cluster size (>10) to allow efficient capture with the RNA baits. Of the markers satisfying the filtering criteria, 3200 population genetic markers were randomly selected for DNA‐capture bait development. Secondly, the unfiltered data were run through the ‘sexy_markers’ function from *radiator* (Devloo‐Delva et al., 2022) to identify polymorphic sex‐linked markers (putatively located on sex chromosomes), which were subsequently included in the DNA capture panel. One biotinylated RNA MYbait (Arbor Bioscience, USA) was synthesized per RAD‐tag. DArTcap hybridization and washing followed the MYbaits standard protocol (https://arborbiosci.com/wp‐content/uploads/2020/08/myBaits_v5.0_Manual.pdf). The DArTcap‐enriched libraries were sequenced on a HiSeq 2500 (Illumina, San Diego, California, USA) as described by Feutry et al. (2020).

### 2.3 DArTcap SNP and individual filtering

Overall, 1014 sample libraries, including 92 technical replicates, were genotyped with the DArTcap protocol. Additional filtering was performed to remove any unspecific enrichment or low‐quality loci. This filtering was performed with the ‘filter_rad’ function as described previously (see Supporting Information sections 7–12). Sex‐linked markers were removed prior to population genetic analyses.

The filtering steps were applied with different thresholds to six different subsets of the data (Table 1). A hierarchical approach was used for the clustering methods (e.g., Vähä et al., 2007). The first DArTcap data set was filtered under less stringent thresholds (e.g., allow more missing data and higher levels of heterozygosity) to permit the identification of species that were not Bull Shark (DATA1: 922 sharks). The second dataset only contained confirmed Bull Sharks (DATA2: 868 sharks). Since unequal sample sizes can introduce bias in clustering algorithms (Foster et al., 2018; Puechmaille, 2016), in the third dataset, all sampling locations were included but Australia was represented by a random subset of 60 individuals (DATA3: 430 sharks). The fourth and fifth datasets are comprised of sampling locations in the W‐ATL and IWP, respectively (DATA4: 117 sharks; DATA5: 732 sharks). Lastly, within the IWP, samples from Japan and Fiji were removed and 60 Australian samples were again randomly selected to equalize the sample sizes and investigate unbiased signals of more subtle structure (DATA6: 221 sharks).

**TABLE 1:** Description of the different *Carcharhinus leucas* datasets used in this study.

Data set	Genotyping method	Data description	Usage	Before filtering	After filtering
DArTseq1	DArTseq	Full global dataset including replicates and other species	For species identification (allow more missing data and higher levels of heterozygosity)	218 sharks; 250,945 SNPs	218 sharks; 28,418 SNPs
DArTseq2	DArTseq	Full global dataset with only Bull Shark samples	For DArTcap marker selection	182 sharks; 250,945 SNPs	149 sharks; 17,029 SNPs
DATA1	DArTcap	Full global dataset including replicates and other species	For species identification (allow more missing data and higher levels of heterozygosity)	1014 sharks; 37,537 SNPs	1014 sharks; 5053 SNPs
DATA2	DArTcap	Global dataset with confirmed Bull Sharks	For genetic diversity, F_ST_, and provenance assignment	868 sharks; 37,537 SNPs	769 sharks; 3409 SNPs
DATA3	DArTcap	Global Bull Shark dataset with a subset of 60 samples from Australia	Equalized sample size to allow unbiased clustering analyses	430 sharks; 37,537 SNPs	382 sharks; 1849 SNPs
DATA4	DArTcap	All western Atlantic Bull Shark samples	For genetic diversity estimates without bias from population structure	117 sharks; 37,537 SNPs	91 sharks; 931 SNPs
DATA5	DArTcap	All Indo‐West Pacific Bull Shark samples	For genetic diversity estimates without bias from population structure	732 sharks; 37,537 SNPs	635 sharks; 3416 SNPs
DATA6	DArTcap	IWP data with a subset of 60 samples from Australia	Equalized sample size to allow unbiased clustering analyses	221 sharks; 37,537 SNPs	189 sharks; 1785 SNPs

*Note*: The data generated by DArTseq and DArTcap genotyping were subdivided to account for unequal samples sizes and hierarchical clustering. The number of sharks and SNPs before and after quality filtering are presented.

### 2.4 Mitogenome amplification and sequencing

Samples from the E‐ATL, IWP, and E‐PAC were used to investigate their matrilineal evolutionary history (sample sizes indicated in Figure 1). The full mitochondrial genomes of 384 putative Bull Sharks were amplified with two primer pairs (A and B fragments; Supporting Information section 13.1). Polymerase chain reactions (PCR) were performed in 30 μL volumes, following the standard proofreading Takara LA Taq protocol (Takara, Otsu, Shiga, Japan). PCR conditions were set to 1 min at 94°C for initial denaturation, then 40 cycles of denaturation (94°C, 30 s), annealing (55°C, 30 s), and extension (68°C, 10 min); concluded with a 10 min extension at 72°C. PCR products were cleaned following the Agencourt AMPure XP magnetic bead protocol (Beckman Coulter Inc., Indianapolis, Indiana, USA). Amplicons were quantified with a NanoDrop 8000 Spectrophotometer and the purified A and B fragments were pooled at equimolar concentrations for each individual. Subsequently, these amplicons were simultaneously fragmented and barcoded with the Nextera XT DNA Sample Preparation kits and 96 sample Nextera Index kit (Illumina). The libraries were quantified with the Qubit dsDNA BR assay kit (Life Technologies, Carlsbad, California, USA) and normalized. Libraries were then pooled and sequenced on a MiSeq desktop sequencer using the 2 × 250 bp paired‐end reads MiSeq reagent kit v2 (Illumina).

### 2.5 Mitogenome assembly and alignment

Demultiplexed fastq files were imported into Geneious prime software v2020.1 (Biomatters Ltd., Auckland, New Zealand) and the reads were paired. The Nextera adapters were removed, and the reads were quality trimmed at a phred score < 20 for a k‐mer of 20 using the BBDuk tool as implemented in Geneious. Reads shorter than 60 bp after trimming were discarded from subsequent analyses. Merged reads for each individual were then mapped onto a previously published Bull Shark reference sequence (Chen et al., 2015) using “Map to Reference” tool in Geneious with the “high sensitivity” parameters and 10 iterations. The majority rule consensus (>50% of mapped reads for any single SNP, insertion, or deletion) for each shark was exported. All mitogenome sequences were aligned with the ‘multiple align’ tool and the MUSCLE algorithm (Edgar, 2004).

### 2.6 Species identification

The mitogenomes were blasted (*megablast*) in Geneious against the GenBank nr/nt database. All sequences with a match of identity ≥98% to a database entry were assigned as that species. Samples without mitogenome information (only DArTcap genotypes, i.e., DATA1) were assigned to species using a principal component analysis (PCA) as implemented in *adegenet* v2.1.1 (Supporting Information section 7; Jombart & Ahmed, 2011). Here, the species identified based on mitogenomes served as a baseline to detect species clusters from PCA. To provide certainty, individuals needed to cluster on multiple PC axes with a mitochondrial‐verified species. Where this was not possible, we marked the species as ‘unknown’. Only samples identified as Bull Shark were included in further analyses.

### 2.7 Genetic diversity

Allelic richness (A_r_), observed heterozygosity (H_O_), and unbiased expected heterozygosity (H_E_) were calculated for each sampling location with *n* > 1 using the R package *diveRsity* v1.9.90 (Keenan et al., 2013). Heterozygosity was calculated across the global dataset (including monomorphic and polymorphic SNPs) to yield an unbiased estimate of H_O_ (see Schmidt et al., 2021). Inbreeding coefficients (F_IS_) were calculated with *hierfstat* v0.04–22 (Goudet, 2005), using 1000 bootstraps to determine a 95% confidence interval. Mitochondrial nucleotide (π_mt_) and haplotype (h) diversities per location, and parsimony haplotype networks were calculated with the *pegas* v0.14 package (Paradis, 2010). All analyses were performed in R 4.0.2 (R Core Team, 2020) and provided in the Supporting Information.

### 2.8 Population structure

Fixation indices (mtDNA: sequence‐based Φ_ST_, SNPs: F_ST_) were calculated between all sampling locations (*n* ≥ 1) and between/across ocean basins with the ‘popStructTest’ function in the *strataG* v2.4.905 package (Archer et al., 2017), and their significance assessed by 1000 permutations. Next, dimensionality‐reduction clustering analyses were conducted with *adegenet* (PCA and Discriminant Analysis of Principal Components, DAPC; Jombart & Ahmed, 2011; Jombart et al., 2010). Individuals were grouped using the successive K‐means algorithm implemented in the ‘find.clusters’ function. The goodness of fit, determined by the Bayesian information criterion (BIC), was employed to find the best number of clusters (K). To avoid overfitting, the optimal number of principal components was selected through cross‐validation with a 10% hold‐out set and 1000 replicates for all DAPC analyses, where individuals were grouped according to their sample location.

### 2.9 Provenance assignment

The provenance assignment success of the DArTcap markers was tested with a*sssignPOP* v. 1.2.2 (Chen, Marschall, et al., 2018) and *rubias* v.0.3.2 (Anderson et al., 2008; Moran & Anderson, 2019). Assignment accuracy was tested with *assignPOP*, using both the Monte‐Carlo and K‐fold cross‐validation procedures to test the assignment of a hold‐out data set with 1000 iterations. We tested power of the markers by selecting a subset of loci with the highest F_ST_ values (5%, 10%, 50%, and 100% of all loci) to train the assignment model. Similarly, the assignment accuracy of simulated mixed groups, based on a reference leave‐one‐out dataset, was evaluated with *rubias* (Anderson et al., 2008). Known simulated proportions for each reporting unit were compared with the numbers estimated by *rubias*. Populations with a sample size of one (i.e., Sierra Leone, eastern Atlantic) were excluded from these analyses. We also examined the minimum number of informative markers needed to assign provenance by subsampling 5–500 markers based on loading contributions of each principal component from the DAPC analysis and testing the assignment accuracy with *rubias*.

### 2.10 Kinship assignment

To investigate fine‐scale contemporary connectivity, close‐kin relationships were examined in each identified genetic cluster with >100 individuals to allow accurate allele frequency estimation, namely W‐ATL and IWP. Kinship was tested using a log‐likelihood‐ratio (LLR) approach developed by Bravington et al. (2016) and applied by Hillary et al. (2018) and Feutry et al. (2020). A statistical threshold was set to reduce the number of false positive detections (i.e., more distantly‐related kin) due to the large number of pairwise comparisons. Replicated or recaptured individuals were already visually identified with the ‘filter_rad’ function based on the number of loci with the same genotype for each pair of individuals (<10% genotypic difference).

## 3 RESULTS

### 3.1 SNP genotyping, baiting success, and data filtering

One sample from Papua New Guinea (PNG) failed DArTseq library construction, resulting in 187 samples for DArTseq sequencing and genotyping. An average of 2,182,162 reads (of 69 bp length) per sample were obtained from the sequencing and a total of 250,945 SNPs, located on 168,810 unique RAD‐tags, were called by the DArTsoft14 program. Data filtering discarded 233,916 SNPs and 33 sharks, leaving a total of 17,029 high‐quality SNPs. Of those, we randomly selected 3200 loci with at least one SNP (Supporting Information sections 4–6). We also identified 469 sex‐linked markers (three Y‐linked and 466 X‐linked markers; Supporting Information section 4.2). Two Y‐ and 208 polymorphic X‐linked sequences were included for DArTcap bait design.

Five samples failed DArTcap library construction. On average, 583,809 reads per sample were obtained from the DArTcap sequencing. After sequence clustering and SNP calling, we obtained 37,537 SNPs found on 26,335 RAD‐tags. Quality‐filtering was applied to six subsets of the DArTcap data (Table 1): the full data set (DATA1: 1014 sharks including replicates and other species; 5053 SNPs), the global dataset with confirmed Bull Sharks (DATA2: 769 sharks; 3409 SNPs), the global dataset with equalized samples sizes (DATA3: 382 sharks; 1849 SNPs), and regional Bull Shark data sets: W‐ATL data (DATA4: 91 sharks; 931 SNPs), IWP data (DATA5: 635 sharks; 3416 SNPs), and IWP data with equalized samples size (DATA6: 189 sharks; 1785 SNPs).

### 3.2 Mitogenome sequencing and assembly

The mitogenome was sequenced for 384 individuals with an average of 49,766 reads. Six individuals had low sequence coverage (<5000 mapped reads and mtDNA regions with no sequence coverage relative to the reference genome) and were subsequently removed from analyses (from South Africa, Sri Lanka, PNG, and Australia: Clarence River and Wenlock River). All reads that mapped to the reference genome were checked for ambiguous base calls at an 85% threshold (where a base needed to be present in >85% of mapped reads to be called unambiguous) to detect DNA cross‐contamination, barcode slippage, or heteroplasmy. Seven individuals that had ambiguous base calls were removed due to signs of DNA contamination (originating from South Africa, PNG, Seychelles, Réunion, and Australia). Overall, the mitogenome length was 16,707–16,708 bp (Supporting Information section 13).

### 3.3 Species identification

Mitogenome sequencing revealed 11 individuals from the Arabian Peninsula, Réunion, Sri Lanka, PNG, Costa Rica, and Fiji that were not Bull Shark: Pigeye Shark (*Carcharhinus amboinensis*, *n* = 5), Spinner Shark (*Carcharhinus brevipinna*, *n* = 1), Graceful Shark (*Carcharhinus amblyrhynchoides*, *n* = 1), Gray Reef Shark (*Carcharhinus amblyrhynchos*, *n* = 1), Pacific Smalltail Shark (*Carcharhinus cerdale*, *n* = 1), Dusky Shark (*Carcharhinus obscurus*, *n* = 1), and Speartooth Shark (*Glyphis glyphis*, *n* = 1). Furthermore, in the PCA analysis carried out on DATA1 an additional 54 individuals grouped with one of these species and another four individuals were neither these nor Bull Shark (Supporting Information section 7.8). Consequently, these individuals were omitted, and 769 confirmed Bull Sharks were retained in the filtered DArTcap data (DATA2) and 361 in the filtered mitogenome data.

### 3.4 Global genetic diversity

More than half the DArTseq and DArTcap (DATA2) markers were monomorphic at almost all locations (Table 2 and Supporting Information section 5.6). In both genotyping protocols, the H_O_ for the E‐PAC (H_O_ = 0.036 in Costa Rica) and the W‐ATL (H_O_ range = 0.048–0.052) were lower than those for the E‐ATL and IWP (H_O_ range = 0.056–0.069; Table 2). Most sampling locations exhibited significantly positive inbreeding coefficients (Table 2), but this was most pronounced in the E‐PAC (Costa Rica) and IWP (South Africa, Mozambique, and Fiji; F_IS_ = 0.041–0.068).

**TABLE 2:**
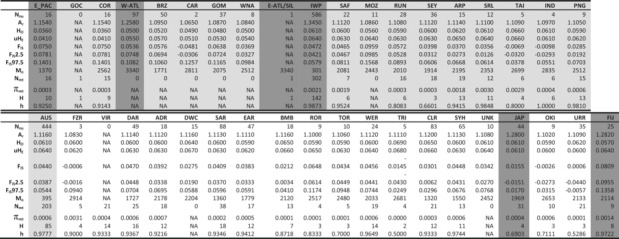
Description of the genetic variation for the full *Carcharhinus leucas* data set (DATA2: 769 sharks; 3409 SNPs) and the full mitochondrial genomes (361 sharks; 16,708 bp).

A total of 165 polymorphic sites were identified across all 361 mitogenome sequences, with an average nucleotide diversity of 0.001 and a haplotype diversity of 0.890 (Supporting Information section 13.3.1). Mitochondrial nucleotide diversities per sampling location ranged from 0.0001 in northern Australia (e.g., Roper and Towns rivers) to 0.003 in Sri Lanka (Table 2). Of the 165 polymorphic sites, most were only present within the IWP group (Supporting Information section 13.3.4). Haplotype diversities were high in almost all sampling locations (h range = 0.80–0.99), with the lowest values in the Seychelles, Trinity Inlet (Australia), and Japan (h = 0.50–0.70).

### 3.5 Population structure

**FIGURE 2:**
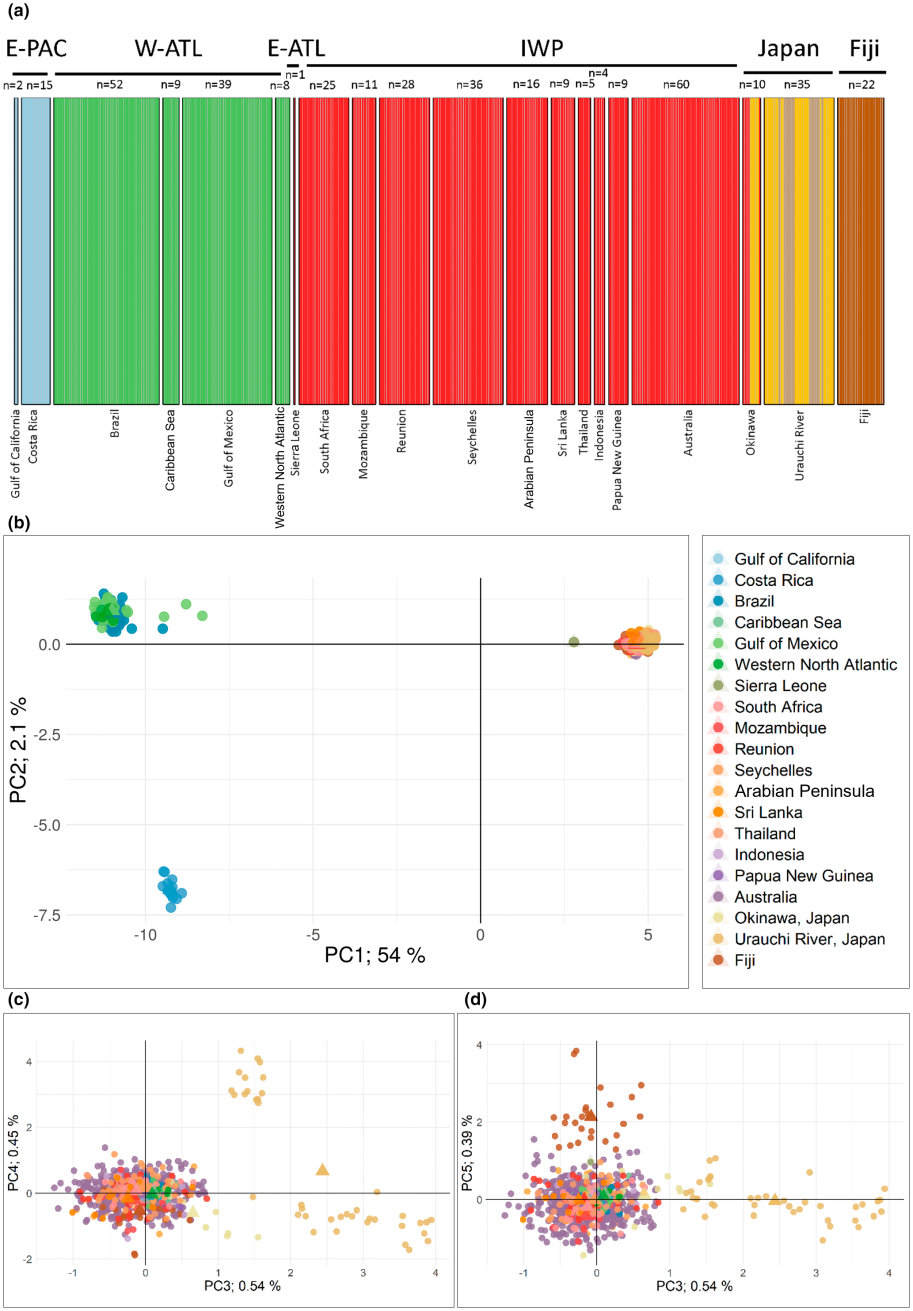
Population clustering analysis for the global *Carcharhinus leucas* dataset with a subsample of Australian sharks (DATA3: 382 sharks; 1849 SNPs). Panel a shows the Discriminant Analysis of Principal Components (DAPC) assignment barplot for K = 6 and 51 principal components (PC). Each bar represents an individual and is colored according to the posterior membership probabilities. Panels b–d represent the principal component analysis (PCA) scatterplot, where each point represents an individual shark, triangles indicate the mean PCA score per sampling location, and colors represent the sampling country or oceanographic location. (b) PCA scatterplot with PC1 on the x‐axis and PC2 on the y‐axis. (c) PCA scatterplot with PC3 on the x‐axis and PC4 on the y‐axis. (d) PCA scatterplot with PC3 on the x‐axis and PC5 on the y‐axis.

Based on the nuclear DNA data (F_ST_, DAPC, and PCA), we identified four major genetic clusters and additional hierarchical structure within these clusters (Figure 2, Table 3). The DAPC indicated that the optimal number of clusters for DATA3 (i.e., equal sample sizes) was K = 3–4 (Supporting Information section 9.9). These clusters corresponded to the E‐PAC, W‐ATL, E‐ATL, and IWP/Japan/Fiji (F_ST_ = 0.10–0.51, *p* < .001; Table 3). Further investigation within the latter group (i.e., DATA5; Supporting Information section 11.9) revealed further clustering into three groups corresponding to IWP, Japan, and Fiji (Figure 2). Here, the F_ST_ values ranged from 0.06 between Japan and Fiji to <0.02 among IWP locations (*p* < .001). We saw high divergence between the two Japan locations (Okinawa and Urauchi River; Figure 2c; F_ST_ = 0.03, *p* < .001), where individuals from Okinawa clustered closer to the IWP group and individuals from the Urauchi River were split into two groups. While the DAPC grouped the E‐ATL (one individual from Sierra Leone) with the IWP cluster, F_ST_ showed this location as distinctly different (Table 3). Nuclear differentiation between locations in the W‐ATL were small (F_ST_ < 0.007), although the DAPC and PCA analyses showed signs of differentiation between the southern (Brazil) and the northern locations (Gulf of Mexico and Western North Atlantic; Supporting Information section 10). Overall, these results indicated significant genetic differentiation of the E‐PAC, southern W‐ATL, northern W‐ATL, E‐ATL, IWP, Japan, and Fiji.

**TABLE 3:** Pairwise comparison of fixation indices for *Carcharhinus leucas*.

	E‐PAC		W‐ATL				E‐ATL	IWP																								Japan		Fiji
																	Australia																	
	GOC	COR	BRZ	CAR	GOM	WNA	SIL	SAF	MOZ	RUN	SEY	ARP	SRL	TAI	IND	PNG	FZR	VIR	DAR	ADR	DWC	SAR	EAR	BMB	ROR	TOR	WER	TRI	CLR	SYH	UNK	OKI	URR	FIJ
GOC		0.124	NA	NA	NA	NA	NA	0.788	NA	0.968	*0.967	0.804	0.664	0.677	0.951	0.931	0.649	*0.951	*0.934	0.923	NA	*0.977	0.944	*0.988	0.988	0.985	*0.932	1.000	*0.966	0.936	NA	0.937	0.989	0.848
COR	NA		NA	NA	NA	NA	0.958	*0.913	NA	*0.969	*0.968	*0.877	*0.832	*0.887	*0.965	*0.951	*0.891	*0.959	*0.947	*0.944	NA	*0.975	*0.956	*0.979	*0.973	*0.974	*0.949	*0.975	*0.968	*0.954	NA	*0.957	0.981	*0.925
BRZ	*0.220	*0.237		NA	NA	NA	NA	NA	NA	NA	NA	NA	NA	NA	NA	NA	NA	NA	NA	NA	NA	NA	NA	NA	NA	NA	NA	NA	NA	NA	NA	NA	NA	NA
CAR	*0.229	*0.248	*0.007		NA	NA	NA	NA	NA	NA	NA	NA	NA	NA	NA	NA	NA	NA	NA	NA	NA	NA	NA	NA	NA	NA	NA	NA	NA	NA	NA	NA	NA	NA
GOM	*0.225	*0.243	*0.004	*0.006		NA	NA	NA	NA	NA	NA	NA	NA	NA	NA	NA	NA	NA	NA	NA	NA	NA	NA	NA	NA	NA	NA	NA	NA	NA	NA	NA	NA	NA
WNA	*0.229	*0.251	*0.005	*0.003	*0.004		NA	NA	NA	NA	NA	NA	NA	NA	NA	NA	NA	NA	NA	NA	NA	NA	NA	NA	NA	NA	NA	NA	NA	NA	NA	NA	NA	NA
SIL	NA	NA	0.467	0.473	0.473	0.476		0.788	NA	0.968	0.967	*0.802	0.662	0.676	0.950	0.930	0.646	*0.950	*0.933	0.922	NA	*0.977	0.943	0.988	0.988	0.985	0.931	1.000	0.966	0.935	NA	0.937	*0.989	0.846
SAF	*0.478	*0.497	*0.472	*0.473	*0.476	*0.479	0.098		NA	0.211	*0.159	*0.590	0.113	0.048	0.747	*0.782	0.276	*0.828	*0.813	*0.784	NA	*0.897	*0.808	*0.846	*0.736	*0.757	*0.800	*0.741	*0.849	*0.781	NA	*0.777	*0.884	*0.643
MOZ	*0.489	*0.508	*0.481	*0.482	*0.485	*0.488	0.107	*0.004		NA	NA	NA	NA	NA	NA	NA	NA	NA	NA	NA	NA	NA	NA	NA	NA	NA	NA	NA	NA	NA	NA	NA	NA	NA
RUN	*0.485	*0.502	*0.476	*0.478	*0.481	*0.483	0.104	*0.002	*0.006		*0.606	*0.776	*0.456	*0.520	*0.941	*0.919	*0.713	*0.933	*0.915	*0.911	NA	*0.958	*0.929	*0.963	*0.953	*0.954	*0.918	*0.956	*0.946	*0.925	NA	*0.931	*0.967	*0.864
SEY	*0.486	*0.502	*0.476	*0.478	*0.481	*0.483	0.107	*0.002	*0.006	*0.003		*0.776	*0.395	*0.399	*0.937	*0.916	*0.678	*0.929	*0.912	*0.907	NA	*0.955	*0.925	*0.959	*0.949	*0.950	*0.915	*0.952	*0.943	*0.922	NA	*0.928	*0.964	*0.862
ARP	*0.479	*0.496	*0.470	*0.472	*0.475	*0.477	0.102	*0.002	*0.005	*0.002	*0.002		*0.292	*0.387	0.135	*0.286	*0.203	*0.322	*0.252	*0.224	NA	*0.483	*0.256	*0.392	0.256	*0.287	*0.227	0.228	*0.362	*0.272	NA	*0.256	*0.326	*0.219
SRL	*0.492	*0.507	*0.480	*0.481	*0.484	*0.487	0.107	*0.003	*0.006	*0.003	*0.005	0.001		0.000	*0.391	*0.504	0.000	*0.563	*0.552	*0.500	NA	*0.692	*0.524	*0.561	*0.404	*0.434	*0.520	*0.393	*0.585	*0.490	NA	*0.476	*0.610	*0.334
TAI	0.492	*0.509	*0.480	*0.482	*0.484	*0.487	0.122	*0.011	*0.013	*0.010	*0.012	*0.009	*0.011		*0.550	*0.648	0.030	*0.713	*0.691	*0.646	NA	*0.825	*0.677	*0.729	*0.544	*0.580	*0.666	*0.539	*0.741	*0.638	NA	*0.621	*0.780	*0.451
IND	0.478	*0.497	*0.468	*0.470	*0.473	*0.475	0.102	*0.009	*0.013	*0.012	*0.014	*0.012	*0.014	*0.020		*0.424	*0.345	*0.463	*0.320	*0.255	NA	*0.705	*0.389	*0.762	*0.651	*0.673	*0.308	*0.629	*0.571	*0.409	NA	*0.425	*0.660	*0.321
PNG	*0.483	*0.497	*0.471	*0.472	*0.475	*0.478	0.108	*0.004	*0.012	*0.005	*0.006	*0.006	*0.006	*0.013	*0.015		*0.405	*0.290	*0.245	*0.153	NA	*0.486	*0.277	*0.475	*0.343	*0.380	*0.257	*0.429	*0.427	*0.116	NA	*0.411	*0.639	*0.339
FZR	NA	*0.512	*0.483	*0.483	*0.487	*0.489	NA	0.002	0.006	0.003	*0.007	0.005	*0.009	0.016	0.017	0.005		*0.439	*0.433	*0.375	NA	*0.611	*0.404	*0.469	0.197	*0.256	0.405	0.288	*0.543	*0.399	NA	*0.431	*0.666	*0.210
VIR	*0.483	*0.497	*0.472	*0.473	*0.476	*0.479	0.105	0.001	*0.005	*0.004	*0.004	*0.003	*0.004	*0.011	*0.014	*0.006	0.004		*0.063	0.039	NA	*0.042	0.030	*0.133	0.009	0.073	*0.069	*0.502	*0.440	*0.317	NA	*0.501	*0.667	*0.410
DAR	*0.485	*0.500	*0.476	*0.477	*0.480	*0.483	0.103	*0.001	*0.006	*0.002	*0.002	*0.002	*0.003	*0.011	*0.012	*0.003	*0.006	*0.002		0.025	NA	*0.206	0.005	*0.268	0.145	*0.194	*0.029	*0.377	*0.366	*0.259	NA	*0.386	*0.514	*0.358
ADR	*0.485	*0.501	*0.475	*0.477	*0.479	*0.482	0.108	*0.002	*0.005	*0.002	*0.002	0.001	*0.003	*0.011	*0.009	*0.004	0.001	*0.003	*0.001		NA	*0.223	0.028	*0.234	0.110	*0.156	*0.018	*0.335	*0.338	*0.184	NA	*0.356	*0.514	*0.312
DWC	*0.475	*0.493	*0.468	*0.469	*0.472	*0.475	0.099	0.001	*0.006	*0.004	*0.003	*0.003	*0.005	*0.012	*0.011	*0.005	0.003	0.001	*0.002	*0.002		NA	NA	NA	NA	NA	NA	NA	NA	NA	NA	NA	NA	NA
SAR	*0.485	*0.500	*0.475	*0.476	*0.479	*0.482	0.105	0.001	*0.005	*0.003	*0.002	*0.002	*0.003	*0.009	*0.012	*0.004	*0.004	*0.002	*0.001	*0.001	*0.002		*0.148	*0.147	0.062	*0.148	*0.233	*0.720	*0.603	*0.527	NA	*0.687	*0.802	*0.594
EAR	*0.483	*0.499	*0.475	*0.476	*0.479	*0.482	0.104	*0.001	*0.006	*0.003	*0.002	*0.002	*0.002	*0.011	*0.013	*0.004	*0.004	*0.002	*0.001	0.001	*0.002	0.000		*0.245	0.103	0.163	*0.044	*0.449	*0.411	*0.294	NA	*0.435	*0.615	*0.363
BMB	*0.486	*0.502	*0.476	*0.477	*0.480	*0.483	0.109	*0.005	*0.008	*0.005	*0.006	*0.006	*0.007	*0.013	*0.016	*0.008	*0.009	*0.006	*0.005	*0.004	*0.006	*0.005	*0.005		*0.296	*0.331	*0.236	*0.873	*0.646	*0.528	NA	*0.691	*0.883	*0.512
ROR	*0.481	*0.502	*0.475	*0.476	*0.479	*0.482	0.098	*0.002	0.006	*0.004	*0.004	*0.003	0.003	*0.010	*0.013	*0.006	*0.006	*0.004	*0.002	0.003	0.003	*0.002	0.002	*0.006		0.000	*0.132	*0.911	*0.578	*0.394	NA	*0.574	*0.880	*0.326
TOR	*0.495	*0.508	*0.481	*0.483	*0.485	*0.489	0.114	*0.006	*0.011	*0.007	*0.006	*0.004	*0.008	*0.015	*0.019	*0.010	0.006	*0.007	*0.006	*0.005	*0.005	*0.005	*0.005	*0.008	0.005		*0.175	*0.885	*0.597	*0.428	NA	*0.598	*0.879	*0.367
WER	*0.488	*0.503	*0.477	*0.478	*0.481	*0.484	0.111	*0.003	*0.008	*0.002	*0.003	*0.002	*0.003	*0.012	*0.014	*0.005	0.004	*0.004	*0.002	*0.002	0.002	*0.001	*0.001	*0.006	*0.004	*0.006		*0.357	*0.348	*0.255	NA	*0.378	*0.529	*0.328
TRI	0.478	*0.498	*0.470	*0.472	*0.475	*0.477	0.103	*0.004	0.010	*0.007	*0.006	*0.005	*0.011	0.013	0.013	0.008	0.002	0.006	0.002	*0.006	0.003	*0.004	0.002	0.007	0.002	0.010	0.004		0.000	0.186	NA	*0.551	*0.882	0.000
CLR	*0.473	*0.488	*0.465	*0.466	*0.469	*0.472	0.103	*0.002	*0.005	*0.003	*0.003	*0.002	*0.004	*0.010	*0.012	*0.005	*0.004	*0.003	*0.002	*0.001	*0.002	*0.001	*0.001	*0.005	*0.003	*0.005	*0.002	*0.004		*0.188	NA	*0.570	*0.750	*0.178
SYH	*0.485	*0.500	*0.475	*0.476	*0.479	*0.482	0.104	*0.002	*0.005	*0.003	*0.003	*0.002	*0.003	*0.011	*0.013	*0.004	0.003	*0.002	*0.001	0.000	0.002	*0.001	*0.001	*0.005	*0.003	*0.005	*0.002	*0.004	*0.001		NA	*0.446	*0.666	*0.178
UNK	*0.484	*0.499	*0.473	*0.474	*0.477	*0.480	0.104	*0.003	*0.008	*0.005	*0.004	*0.005	*0.006	*0.015	*0.013	*0.009	0.005	*0.005	*0.003	0.003	0.003	*0.002	*0.002	*0.007	*0.004	0.005	*0.004	0.003	*0.002	*0.002		NA	NA	NA
OKI	*0.507	*0.516	*0.486	*0.488	*0.490	*0.494	0.125	*0.017	*0.024	*0.017	*0.018	*0.018	*0.023	*0.028	*0.023	*0.022	*0.018	*0.019	*0.016	*0.017	*0.018	*0.017	*0.017	*0.021	*0.019	*0.023	*0.018	*0.019	*0.016	*0.017	*0.016		*0.555	*0.611
URR	*0.509	*0.519	*0.492	*0.493	*0.496	*0.499	0.142	*0.021	*0.026	*0.023	*0.020	*0.021	*0.025	*0.033	*0.034	*0.024	*0.023	*0.024	*0.022	*0.021	*0.021	*0.021	*0.021	*0.027	*0.024	*0.028	*0.021	*0.024	*0.021	*0.022	*0.021	*0.032		*0.389
FIJ	*0.499	*0.514	*0.486	*0.487	*0.491	*0.493	0.116	*0.018	*0.023	*0.017	*0.017	*0.016	*0.019	*0.027	*0.026	*0.019	*0.017	*0.018	*0.018	*0.017	*0.019	*0.017	*0.016	*0.020	*0.019	*0.021	*0.018	*0.022	*0.016	*0.017	*0.018	*0.032	*0.037	

*Note*: F_ST_ values are presented in the bottom diagonal and sequence‐based Φ_ST_ are in the top diagonal. ‘NA’ values indicate that no or too few samples were available to estimate genetic differentiation. Asterisks (*) indicate statistical significance of *p* < .05. No pairwise comparisons were significant after Bonferroni correction. Fixation indices are colored from high (red) to medium (yellow) to low (blue) values. GOC, Gulf of California; COR, Costa Rica; BRZ, Brazil; CAR, Caribbean Sea; GOM, Gulf of Mexico; WNA, Western North Atlantic; SIL, Sierra Leone; SAF, South Africa; MOZ, Mozambique; RUN, Réunion Island; SEY, Seychelles; ARP, Arabian Peninsula; SRL, Sri Lanka; TAI, Thailand; IND, Indonesia; PNG, Papua New Guinea; FZR, Fitzroy River; VIR, Victoria River; DAR, Daly River; ADR, Adelaide River; DWC, Darwin Coastal; SAR, South Alligator River; EAR, East Alligator River; BMB, Blue Mud Bay; ROR, Roper River; TOR, Towns River; WER, Wenlock River; TRI, Trinity Inlet; CLR, Clarence River; SYH, Sydney Harbor; and UNK, Australian fisheries samples from unknown origin; OKI, Churaumi Aquarium, Okinawa; URR, Urauchi River; and FIJ, Fiji.

**FIGURE 3:**
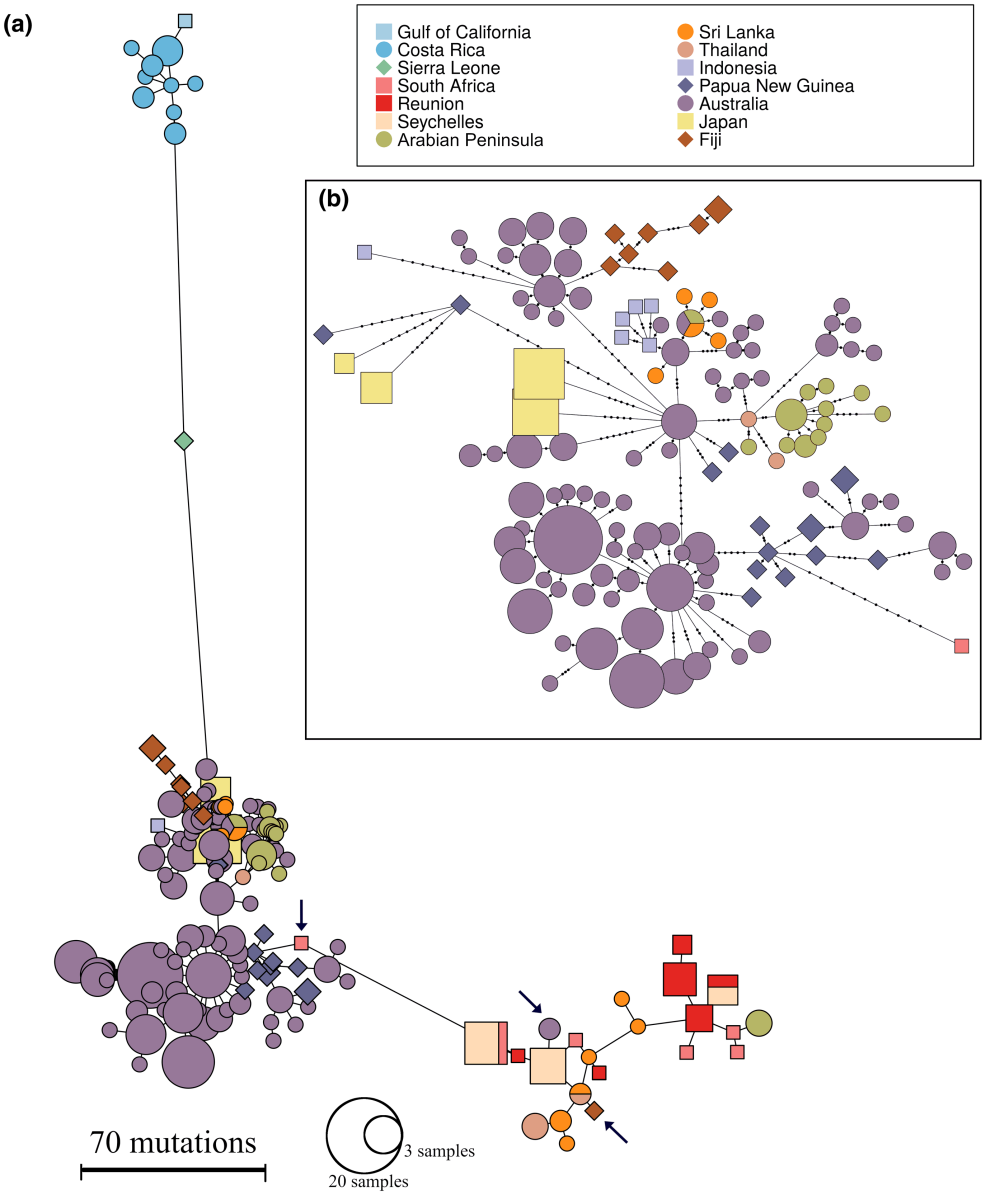
*Carcharhinus leucas* mtDNA haplotype network, based on the full mitogenome (16,707–16,708 bp). Panel a presents the network for all 361 sharks from the eastern Pacific, eastern Atlantic, Indo‐West Pacific, Japan, and Fiji. The distance between haplotypes reflects the number of mutations between them. Panel b shows the ‘eastern Indian Ocean/western Pacific/Japan/Fiji’ cluster in detail (285 sharks). Here, the number of mutations between haplotypes are represented by small black dots. In panels a and b, the size of the shape is equivalent to the square root of the number of individuals that share this haplotype. The color and shape of each haplotype corresponds to the sampling location where they were found. The three black arrows indicate haplotypes that represent recent maternal movement between haplotype clusters.

The mitogenome distance‐based Φ_ST_ values showed high differentiation between and across ocean basins (Φ_ST_ = 0.67–0.99, *p* < .001; Table 3) and the haplotype network demonstrated four major clusters (Figure 3): E‐PAC, E‐ATL, western Indian Ocean (W‐IO), and the E‐IO and western Pacific combined (E‐IO/W‐PAC). All haplotypes, except four, were unique to a single sampling location (Figure 3). Within the Indian Ocean, differentiation was strong between the W‐IO and E‐IO locations (Φ_ST_ = 0.75–0.96, *p* < .001), while the northern Indian Ocean locations (N‐IO; i.e., Arabian Peninsula, Sri Lanka, and Thailand) showed intermediate divergence (Φ_ST_ = 0.46–0.52, *p* < .001; Supporting Information section 13.5.3). The latter is demonstrated in the haplotype network with individuals from the N‐IO split between the W‐IO and E‐IO/W‐PAC haplogroups. Between the W‐IO and E‐IO/W‐PAC groups, the haplotypes of several individuals indicated matrilineal movement at an evolutionary‐recent time scale: two haplotypes, sampled from the Fitzroy River (Australia) and Fiji, clustered with the W‐IO group and one haplotype from South Africa grouped with the E‐IO/W‐PAC group (indicated in Figure 3). Haplotypes sampled from Japan (*n* = 4) and Fiji (*n* = 8) grouped with the E‐IO/W‐PAC cluster, where the Japanese haplotypes did not correspond to the three Japanese clusters identified with the nuclear SNPs. Within Australia, the mitochondrial Φ_ST_ showed structure between western (Fitzroy River), northern (Victoria River, Daly River, Adelaide River, Alligator Rivers—encompassing the South Alligator River and East Alligator River—Blue Mud Bay, Roper River, Towns River, Wenlock River), and eastern Australian sites (Trinity Inlet, Clarence River, and Sydney Harbor; Table 3). The haplotype network of all Australian individuals indicated three major haplotype clusters with no obvious geographic pattern, yet most haplotypes were unique to a sampling site (Supporting Information section 13.4.2.3).

### 3.6 Provenance assignment

**FIGURE 4:**
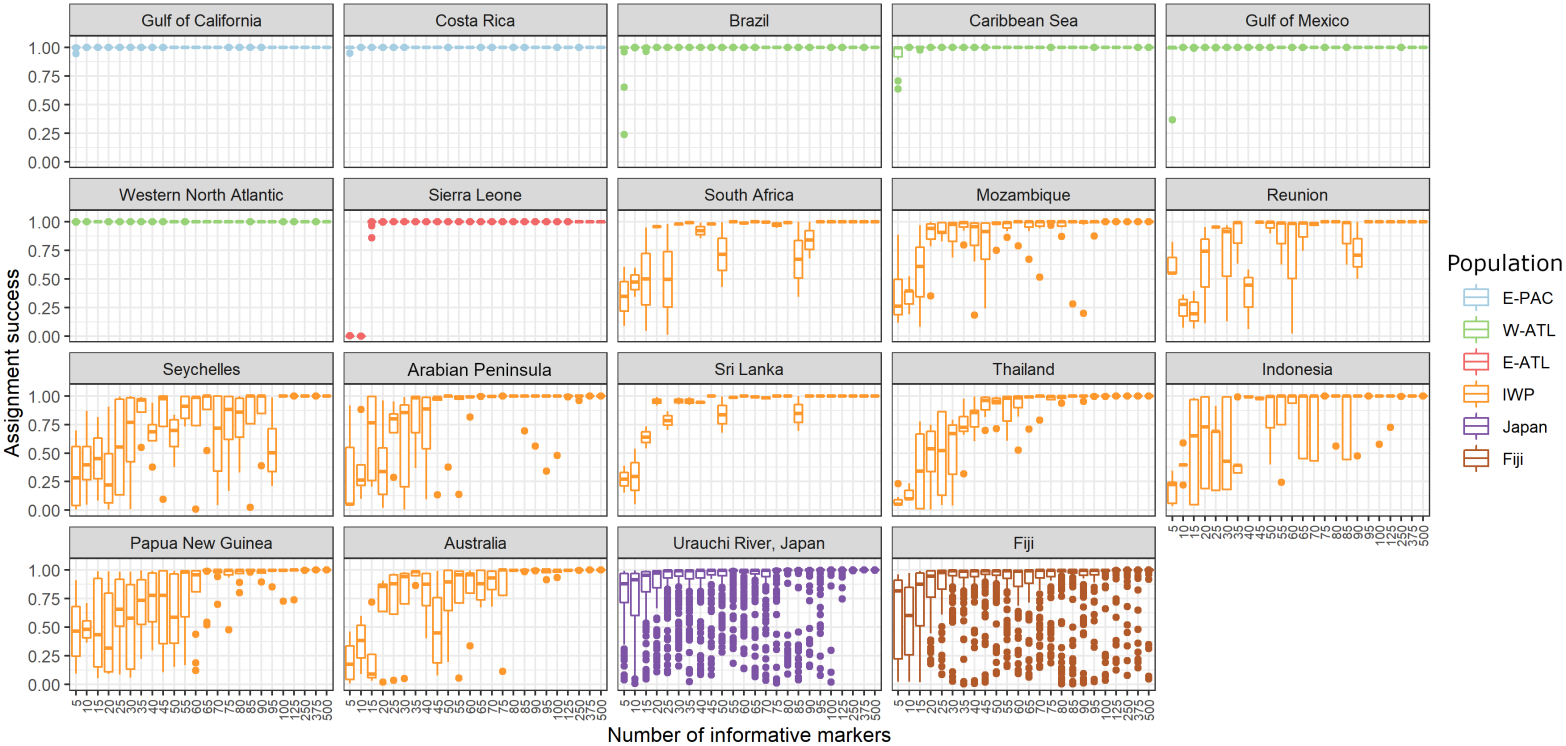
Reassignment success of *Carcharhinus leucas* respective to the number of informative markers to assign simulated mixtures to their population of origin (E‐PAC = eastern Pacific; W‐ATL = western Atlantic; E‐ATL = eastern Atlantic; IWP = Indo‐West Pacific; Japan = Urauchi River, Japan; and Fiji). The simulated mixtures are based on a leave‐one‐out resampling method in *rubias*. The assignment success was tested based on 5–500 informative markers that were selected according to their high DAPC loadings contribution from the *adegenet* package.

After population structure was identified with the nuclear DArTcap markers, we tested the accuracy to assign provenance to a hold‐out data set (Figure 4). Using the marker contributions of the DAPC analysis, we found that at least 250–500 highly differentiating markers were required for 100% provenance assignment accuracy (Supporting Information section 8.10.9). Individuals from Japan and Fiji were unlikely to be assigned to the inferred genetic cluster with less than 500 markers. A minimum of 50–100 markers were needed to obtain a reliable assignment (>80%) to each inferred population and only 5–50 markers were required to differentiate individuals from the E‐PAC, W‐PAC, and E‐ATL, but sites within the IWP required up to 100 markers (Figure 4).

### 3.7 Individual and kinship assignment

In total, 102 duplicate individuals were identified in the dataset. Most represented the technical replicates that were purposely included, but we also detected six recaptured sharks from Sydney Harbor, Japan, and Fiji (time‐at‐liberty = 38–741 days). These recaptures were always assigned to the same age‐cohort where length data were available, thus providing confidence in the length‐at‐age function from Tillett et al. (2011). Within the W‐ATL, we performed 4095 pairwise comparisons and found two full‐sibling pairs (FSPs) in Brazil. The IWP revealed more kin pairs due to the large number of pairwise comparisons (201,295). Overall, we found 18 full‐sibling pairs (FSPs), all within the same sampling locations (Brazil, Réunion, Indonesia, Fiji, and within Australia: Daly, South Alligator, East Alligator, Towns, and Clarence rivers), and 40 half‐sibling pairs (HSPs). Of the 40 HSPs, 32 were found within the same locations (Seychelles, Réunion, PNG, and within Australia: Sydney Harbor, South Alligator, East Alligator, Wenlock, and Clarence rivers), where 10 and 8 HSPs had the same and different haplotypes, respectively; 14 pairs had missing haplotype information. Eight HSPs were distributed between rivers within northern Australia (Daly River/South Alligator River, *n* = 4; Adelaide River/South Alligator River, *n* = 1; South Alligator River/East Alligator River, *n* = 2; and Towns River/Wenlock River, *n* = 1). Seven of those eight ‘cross‐river’ HSPs were juveniles (<150 cm TL) from different age cohorts, and six pairs had different mitochondrial haplotypes.

## 4 DISCUSSION

This study provides the first global assessment of genetic population structure of the Bull Shark, a cosmopolitan coastal predator, using thousands of SNP markers and full mitogenome data. We identify distinct genetic divergence driven by significant biogeographic barriers and philopatric behavior. These results further facilitate the development of a SNP panel for species, sex, provenance, and kinship identification.

### 4.1 Global population structure across ocean barriers

Population structure in large coastal sharks tends to be driven by environmental barriers, movement ecology, and habitat preference (Dudgeon et al., 2012; Hirschfeld et al., 2021). The DArTcap results revealed four global genetic clusters (E‐PAC, W‐ATL, E‐ATL, and IWP), with Japan and Fiji each supporting additional isolated Bull Shark populations. Our results concur with previous studies in the Western Atlantic and Indo‐Pacific regions, which showed genetic differentiation between the W‐ATL, IWP, and Fiji using microsatellite data (Pirog et al., 2019; Testerman, 2014). We show that the E‐PAC is strongly differentiated from the W‐ATL, likely coinciding with the closure of the Isthmus of Panama (~3 Myr; Knowlton et al., 1993; O'Dea et al., 2016). Previously, Bull Shark connectivity has been suggested between the E‐PAC and W‐ATL, based on microsatellite data, through the Panama Canal (Pirog et al., 2019; Testerman, 2014), but our results indicate these populations are demographically isolated and that gene flow through the Panama Canal is negligible, if existent at all. Rather, we believe the differences between our results are likely due to higher power delivered by thousands of SNPs compared to fewer microsatellites (e.g., Green et al., 2019; Layton et al., 2020). In addition, the latter can suffer from homoplasy caused by high mutation rates (i.e., identical by state, not identical by descent; Estoup et al., 2002).

The greater divergence between the W‐ATL and E‐ATL, compared to the E‐ATL and IWP, demonstrates that the Mid Atlantic Barrier (an open ocean distance barrier) forms a stronger and more enduring barrier than the Benguela Upwelling System (a thermal barrier). The permeability of the latter barrier may fluctuate over time due to oscillations in climate (Hirschfeld et al., 2021). Fiji and New Caledonia have previously been identified as differentiated island populations, although New Caledonia showed less differentiation than Fiji from other sites (Glaus et al., 2020; Pirog et al., 2019). This was also attributed to the long distances across deep water trenches and is consistent with our results for Fiji. Similarly, individuals from Japan could be isolated by deep water trenches (>500 m deep, e.g., Okinawa Trough), since the Bull Shark generally prefers shallow waters (<160 m; Rigby et al., 2021). Yet, sea levels between Japan and continental Asia were low until 17 Kyr ago (Voris, 2000), although the high latitudinal location of Japan may have formed a thermal barrier at that time. Additionally, historical land‐bridges such as the Taiwan Strait, Tokara Strait, or Tsushima/Korea Strait could have restricted dispersal between Japan and the IWP (Chen, Wang, et al., 2018; Yin et al., 2009).

Historical land‐bridges also play a potential role for the genetic structuring of Bull Sharks across northern Australia. Despite a lack of structure inferred from the nuclear markers, the mitogenome Φ_ST_ differentiation showed three separate clusters (western, northern, and eastern Australia). Here, the Torres Strait land‐bridge between Australia and Papua New Guinea has been implicated for genetic structuring in a variety of marine taxa across this region (Mirams et al., 2011). This signal from the Bull Shark haplotype network is less structured, and may indicate either recent separation or historical isolation followed by gene flow (e.g., Puckridge et al., 2013).

Our study shows strong influences of land‐bridges, long ocean distances, cold upwelling or high latitudes, and deep‐water trenches on the genetic population structure of the Bull Shark. We hypothesize that these barriers will equally affect elasmobranch species with similar dispersal capacity, distribution, and habitat use. To date, the global population structure of only a few large‐bodied shark species has been investigated (e.g., Galapagos Shark, *C. galapagensis*; Sandbar Shark, *C. plumbeus*; and Scalloped Hammerhead, *S. lewini*; Daly‐Engel et al., 2012; Pazmiño et al., 2018; Portnoy et al., 2010). Known biogeographic barriers have similarly affected the population structure of these species (reviewed in Hirschfeld et al., 2021). For example, the Galapagos Shark, which occupies insular habitats, shows limited gene flow across oceanic barriers, such as the East Pacific Barrier (Pazmiño et al., 2018). Similarly, both the Scalloped Hammerhead and Sandbar Shark exhibited increased connectivity along continuous coastlines within ocean basins but showed significant divergence due to the East Pacific Barrier and closure of the Isthmus of Panama (Daly‐Engel et al., 2012; Portnoy et al., 2010). Consequently, future studies could use our results as a foundation to construct hypotheses for other exploited and threatened species with similar vagility, ecology, and life‐history characteristics (such as the Spinner Shark, the Graceful Shark, or the Dusky Shark).

### 4.2 Fine‐scale structure and sex‐biased dispersal

Minor nuclear differentiation was detected between the southern and northern W‐ATL locations. This concurs with other Bull Shark studies showing strong mitochondrial and weak nuclear population structure in the W‐ATL (Karl et al., 2011; Sandoval Laurrabaquio‐Alvarado et al., 2021) and that long‐distance movement is rare in the Gulf of Mexico (Carlson et al., 2010). This would suggest that a weak or recent barrier to dispersal may be present in this region. Such a barrier can be attributed to oceanographic features (e.g., Caribbean Current) or reproductive asynchrony caused by seasonal differences between the hemispheres (Carrillo et al., 2015, 2017; Castro, 2011; Jensen, 1976). Similarly, genetic differentiation may be present in the IWP (as suggested by Pirog et al., 2019), but our ability to detect significant nuclear differences is constrained by high gene flow and/or recent separation (Bailleul et al., 2018; Waples, 1998). A different set of genetic markers (e.g., whole genome analysis) combined with alternative approaches, like telemetry or parasitology, may provide a new perspective on population structure within the IWP (reviewed in Green, Simpfendorfer, & Devloo‐Delva, 2022).

Within the IWP, the mitogenome results demonstrated maternal structure at a finer spatial scale than the SNP results, supporting the growing evidence of long‐term female philopatry and male‐biased dispersal in Bull Sharks (Karl et al., 2011; Sandoval Laurrabaquio‐Alvarado et al., 2019; Tillett et al., 2012) and other elasmobranchs (reviewed in Chapman et al., 2015; Phillips et al., 2021). Specifically, the mtDNA shows a separation between the W‐IO and E‐IO/W‐PAC, not evident from the nuclear DNA, and greater differentiation of the E‐ATL. The presence of haplotypes from multiple distinct mitochondrial lineages in South Africa, Sri Lanka, Thailand, Fiji, and the Fitzroy River (Australia) represents signals of secondary female contact after historical isolation or low ongoing female gene flow. However, we see that almost every haplotype is unique to a sampling location (except for locations within Australia). This could indicate that each location has experienced sufficient female isolation for the mitogenome to mutate (i.e., complete lineage sorting), and/or is a consequence of high haplotype diversity as a result of sequencing complete mitogenomes. In addition, demographic events affect the non‐recombining haploid mtDNA and recombining diploid nuclear DNA differently (Heist, 2012; Lawson Handley & Perrin, 2007), and, therefore, the mito‐nuclear discordance may not necessarily be driven by sex‐biased dispersal (Toews & Brelsford, 2012).

The spatial distribution of close‐kin, and the cross‐cohort HSPs in particular, is valuable to identify contemporary dispersal and sex bias therein (e.g.,Feutry et al., 2017, 2020). In the IWP, we found that all FSPs and most HSPs were sampled from the same sampling location. The 32 same‐river, cross‐cohort HSPs had an equal likelihood of being maternally (same haplotype) or paternally (different haplotype) related, indicating contemporary biparental philopatry to a finer scale than detected by conventional population genetic methods (Tillett et al., 2012). However, the seven cross‐river, cross‐cohort HSPs in northern Australia were most likely paternally related, suggesting a bias toward male dispersal between rivers. This unconfounded result supports the mito‐nuclear findings and suggests that male‐biased dispersal may be occurring at a broader scale, yet this needs to be confirmed with larger samples sizes.

Within Japan, the SNP data grouped the individuals into three separate clusters, with individuals from Okinawa clustering more closely with the IWP and those from the Urauchi River forming two separate groups. The sharks from the Churaumi Aquarium, Okinawa were originally sourced from Japanese waters near Okinawa Island and several individuals were known to be related. The close similarity of the Okinawa group to the IWP cluster may represent ongoing genetic connectivity or recent colonization, where the strong nuclear differentiation may be caused by an accumulation of mutations at the edge of a range expansion due to the more pronounced effect of drift on a small and recently established population (Peischl et al., 2013). This divergence may be reinforced through high relatedness within a small population and seasonal differences in parturition, which would also explain why we observed multiple genetic clusters within the Urauchi River. The sample size within Japan was too small to accurately estimate kinship; thus, family structure due to extremely small population size cannot be excluded (e.g., Devloo‐Delva et al., 2019; Feutry et al., 2017). Similarly, the Fiji population contains many related individuals (see Glaus et al., 2020), yet these were not removed from our analyses as they could signify an artifact of a small population size (Waples & Anderson, 2017). Mitochondrial haplotypes from individuals in Japan and Fiji are part of the E‐IO/W‐PAC haplogroup, supporting that these are recently established populations.

### 4.3 Species, sex, provenance, and kinship identification

Increasingly, there is need to monitor wild populations and the global trade of wildlife products through DNA forensics (Cardeñosa et al., 2022). Our DArTcap panel of 3400 SNPs can identify species, sex, provenance, and kinship, which allows future monitoring of multiple demographic aspects at low cost (~AU$15 per sample; see Feutry et al., 2020). We were able to identify most of the individuals to species‐level using the DArTseq and DArTcap data (i.e., Bull Shark, Pigeye Shark, Spinner Shark, Graceful Shark, Gray Reef Shark, Smalltail Shark, Dusky Shark, and Speartooth Shark), validated alongside mitochondrial species verification and a complete mtDNA reference database. Given the global scale of shark product trade (Clarke et al., 2006) and the morphological similarity between juveniles of many carcharhinid species, a selection of these SNP markers could be developed into a rapid tool for species identification of various shark products (Johri et al., 2019; Liu et al., 2017). While the species composition of shark fin trade has been studied using mtDNA (Cardeñosa et al., 2022; Fields et al., 2018), some species, like the Galapagos Shark and Dusky Shark, show mitochondrial introgression (Corrigan et al., 2017; Naylor et al., 2012). Here, the use of nuclear SNPs can resolve the ability to separate species with such introgression or incomplete lineage sorting (e.g., Kyne & Feutry, 2017; Liu et al., 2017). Moreover, the presence of sex‐linked markers, specifically Y‐linked markers, in the DArTseq data allowed us to identify the genetic sex of our samples when the visual sex information was missing. Unfortunately, the RNA baits failed to capture these markers appropriately with DArTcap sequencing, although future studies could assess sex ratios from fisheries or trade samples by redesigning the RNA baits for these Y‐linked markers (e.g., Stovall et al., 2018).

The DArTcap panel also shows great promise and power to assign individuals to their respective populations (ocean‐basin scale), and we estimated that a minimum of 100 markers are needed to achieve an accuracy of >80%. Few studies have specifically investigated the power of genetic markers to assign samples from traded shark products to their population of origin, and, to date, most forensic studies of shark trade have employed mtDNA to identify broad geographic origins (e.g., Cardeñosa et al., 2021; Chapman et al., 2009; Fields et al., 2020). Here, by genetically characterizing the source populations with 769 sharks and 3409 SNPs, the regional contributions of Bull Shark products to the global trade market can be estimated in future studies and highlight key regions of harvest to inform management actions. Furthermore, our results suggest a diagnostic SNP panel of 100–500 markers could be designed to identify species, sex, and provenance from tissue samples. New genotyping technologies, such as DNA microarrays, may provide a more cost‐efficient method for monitoring of Bull Shark exploitation (e.g., Arenas et al., 2017; Wenne et al., 2016). However, this panel needs further testing prior to use as an enforcement tool. This will involve obtaining additional samples from undersampled locations (e.g., E‐PAC and E‐ATL) and testing the assignment accuracy on these newly acquired samples.

Finally, we were able to identify close‐kin relationships with the selected DArTcap markers. These relationships are important to estimate contemporary dispersal patterns (Feutry et al., 2017, 2020) or total adult abundance of a population (Bravington et al., 2016). This application requires a large sample size (relative to the total population size) and sufficient biological information (such as sex, age‐at‐length, and age‐at‐maturity), which can pose a challenge in studies of rare or threatened species. While the number of kin was limited in this dataset, the identified kin pairs provided insights into local demographic and genetic connectivity in Australia.

### 4.4 Conservation and management considerations

Coastal shark species, such as the Bull Shark, are particularly susceptible to anthropogenic pressures such as fishing, shark control programs, and habitat modification, due to their close proximity to human populations (Knip et al., 2010). These species fulfill an important role in maintaining the ecosystem dynamics and functions (Ferretti et al., 2010), where a reduction in the abundance of top predators may lead to cascading effects in the food web and ecosystem health (e.g., Dudley & Simpfendorfer, 2006). Understanding the patterns of reproductive isolation of coastal sharks is essential to assess the impact of these threats at the population level (Frankham et al., 2017). Given our results of large‐scale gene flow, we advocate for international collaboration to monitor the impacts on dispersing individuals (predominantly males) as they maintain the gene flow and genetic diversity between populations, and likely experience threats across many different countries. Similarly, coastal development near nursery areas may affect philopatric females disproportionally, with potential consequences for a wider region if other countries depend on recruitment from those nurseries. In addition, genetically isolated and small island populations, such as Japan and Fiji, currently face a number of threats, such as targets or bycatch in fisheries (Glaus et al., 2015) and require close monitoring as even low levels of catch may cause a population reduction. Any decline in abundance is unlikely to be replenished by migration from neighboring populations. Overall, this study suggests that coastal species with comparable characteristics exhibit similar dispersal patterns, and consequently, risk and vulnerability assessments and management actions should be considered at the smallest spatial scales, in accordance with the philopatric behaviors of these species, in order to prevent local depletion.

## AUTHOR CONTRIBUTIONS

**Floriaan Devloo‐Delva:** Conceptualization (equal); data curation (lead); formal analysis (lead); funding acquisition (equal); investigation (lead); methodology (lead); project administration (supporting); resources (supporting); visualization (lead); writing – original draft (lead); writing – review and editing (equal). **Christopher Burridge:** Conceptualization (equal); funding acquisition (equal); investigation (supporting); project administration (equal); supervision (equal); writing – review and editing (equal). **Peter Kyne:** Conceptualization (equal); funding acquisition (equal); project administration (equal); resources (equal); supervision (equal); writing – review and editing (equal). **Juerg Brunnschweiler:** Resources (equal); writing – review and editing (equal). **Demian Chapman:** Resources (equal); writing – review and editing (equal). **Patricia Charvet:** Resources (equal); writing – review and editing (equal). **Xiao Chen:** Resources (equal); writing – review and editing (equal). **Geremy Cliff:** Resources (equal); writing – review and editing (equal). **Ryan Daly:** Resources (equal); writing – review and editing (equal). **J. Marcus Drymon:** Resources (equal); writing – review and editing (equal). **Mario Espinoza:** Resources (equal); writing – review and editing (equal). **Daniel Fernando:** Resources (equal); writing – review and editing (equal). **Laura Garcia Barcia:** Resources (equal); writing – review and editing (equal). **Kerstin Glaus:** Resources (equal); writing – review and editing (equal). **Blanca I. Gonzalez‐Garza:** Resources (equal); writing – review and editing (equal). **Michael Grant:** Resources (equal); writing – review and editing (equal). **Rasanthi M. Gunasekera:** Investigation (equal); project administration (supporting); writing – review and editing (equal). **Sebastian Hernandez:** Resources (equal); writing – review and editing (equal). **Susumu Hyodo:** Resources (equal); writing – review and editing (equal). **Rima W. Jabado:** Resources (equal); writing – review and editing (equal). **Sébastien Jaquemet:** Resources (equal); writing – review and editing (equal). **Grant Johnson:** Resources (equal); writing – review and editing (equal). **James T. Ketchum:** Resources (equal); writing – review and editing (equal). **Hélène Magalon:** Funding acquisition (equal); resources (equal); writing – review and editing (equal). **James R. Marthick:** Investigation (equal); writing – review and editing (equal). **Frederik Mollen:** Resources (equal); writing – review and editing (equal). **Stefano Mona:** Conceptualization (equal); funding acquisition (equal); supervision (supporting); writing – review and editing (equal). **Gavin J.P. Naylor:** Conceptualization (supporting); resources (equal); writing – review and editing (equal). **John E.G. Nevill:** Resources (equal); writing – review and editing (equal). **Nicole M. Phillips:** Resources (equal); writing – review and editing (equal). **Richard D. Pillans:** Funding acquisition (equal); resources (equal); writing – review and editing (equal). **Bautisse D. Postaire:** Conceptualization (equal); data curation (supporting); funding acquisition (supporting); writing – review and editing (equal). **Amy F. Smoothey:** Resources (equal); writing – review and editing (equal). **Katsunori Tachihara:** Resources (equal); writing – review and editing (equal). **Bree J. Tillett:** Resources (equal); writing – review and editing (equal). **Jorge A. Valerio‐Vargas:** Resources (equal); writing – review and editing (equal). **Pierre Feutry:** Conceptualization (equal); funding acquisition (equal); investigation (supporting); project administration (lead); supervision (lead); writing – review and editing (equal).

## CONFLICT OF INTEREST STATEMENT

We note no conflict or competing interests among the authors in relation to the information provided in this manuscript.

### OPEN RESEARCH BADGES

This article has earned an Open Data badge for making publicly available the digitally‐shareable data necessary to reproduce the reported results. The data is available at [https://doi.org/10.5061/dryad.4qrfj6qdg].

## Supporting information


Data S1.

## Data Availability

Genetic data: Mitochondrial genomes are uploaded to GenBank (accession numbers: OP006762‐OP007122). Mitochondrial DNA alignment and SNP genotypes are available on DataDryad (https://doi.org/10.5061/dryad.4qrfj6qdg). Sample metadata: Sample metadata and Rmarkdown document, which included all performed analyses discussed in this study, are available on DataDryad (https://doi.org/10.5061/dryad.4qrfj6qdg). Benefit‐Sharing Statement: Benefits Generated: A research collaboration was developed with scientists from the countries providing genetic samples, all collaborators are included as co‐authors, the results of research have been shared with the provider communities and the broader scientific community (see above), and the research addresses a priority concern, in this case the conservation of the Bull Shark. In Australia and Papua New Guinea, we consulted with the Indigenous communities providing the biodiversity resources and worked with members of the communities to collect samples based on local knowledge. More broadly, our group is committed to international scientific partnerships, as well as institutional capacity building.
