# Supplementary material for: From rivers to ocean basins: The role of ocean barriers and philopatry in the genetic structuring of a cosmopolitan coastal predator
Source: Ecol Evol. 2023 Feb 22;13(2):e9837. doi: 10.1002/ece3.9837 (PMC9944188; doi:10.1002/ece3.9837)
Supplement: Supplementary file 1 — Data S1. [file ECE3-13-e9837-s001.pdf]

Ecology and Evolution
Open Access

# Supplemental Information for:

## From rivers to ocean basins: the role of ocean barriers and philopatry in the genetic structuring of a cosmopolitan marine predator

Floriaan Devloo-Delva*, Christopher P. Burridge, Peter M. Kyne, Juerg M. Brunnschweiler, Demian D. Chapman, Patricia Charvet, Xiao Chen, Geremy Cliff, Ryan Daly, J. Marcus Drymon, Mario Espinoza, Daniel Fernando, Laura Garcia Barcia, Kerstin Glaus, Blanca Idalia González Garza, Michael I Grant, Rasanthi M. Gunasekera, Sebastian I. Hernandez, Susumu Hyodo, Rima W. Jabado, Sébastien Jaquemet, Grant Johnson, James T. Ketchum, Hélène Magalon, James R. Marthick, Frederik H. Mollen, Stefano Mona, Gavin J.P. Naylor, John E.G. Nevill, Nicole M. Phillips, Richard D. Pillans, Bautisse D. Postaire, Amy F. Smoothey, Katsunori Tachihara, Bree J. Tillet, Jorge A. Valerio-Vargas, and Pierre Feutry

* *Author produced this Rmarkdown*

December, 2022

# Contents

**1 Background** **5**

**2 Sampling map and metadata** **6**
2.1 Visual sex ID . . . . . . . . . . . . . . . . . . . . . . . . . . . . . . . . . . . . . 6
2.2 Total lengths . . . . . . . . . . . . . . . . . . . . . . . . . . . . . . . . . . . . . 7
2.3 Aproximate Length-at-age . . . . . . . . . . . . . . . . . . . . . . . . . . . . . . 8
2.3.1 Catch date . . . . . . . . . . . . . . . . . . . . . . . . . . . . . . . . . . . 9
2.3.2 Age . . . . . . . . . . . . . . . . . . . . . . . . . . . . . . . . . . . . . . . 10
2.3.3 Cohort . . . . . . . . . . . . . . . . . . . . . . . . . . . . . . . . . . . . . 11

**3 Load packages** **12**

**4 DArTseq - All data** **15**
4.1 Set strata . . . . . . . . . . . . . . . . . . . . . . . . . . . . . . . . . . . . . . . 15
4.2 Find sex-linked markers . . . . . . . . . . . . . . . . . . . . . . . . . . . . . . . 15
4.3 Filter data . . . . . . . . . . . . . . . . . . . . . . . . . . . . . . . . . . . . . . 23
4.4 Save data . . . . . . . . . . . . . . . . . . . . . . . . . . . . . . . . . . . . . . . 24
4.5 Load data . . . . . . . . . . . . . . . . . . . . . . . . . . . . . . . . . . . . . . . 25
4.6 Remove sex-linked markers . . . . . . . . . . . . . . . . . . . . . . . . . . . . . 25
4.7 PCA to identify species . . . . . . . . . . . . . . . . . . . . . . . . . . . . . . . 26

**5 DArTseq - Only Bull Shark** **32**
5.1 Set strata . . . . . . . . . . . . . . . . . . . . . . . . . . . . . . . . . . . . . . . 32
5.2 Filter data . . . . . . . . . . . . . . . . . . . . . . . . . . . . . . . . . . . . . . 32

5.3 Save data . . . 32
5.4 Load data . . . 33
5.5 Remove sex-linked markers . . . 34
5.6 Genetic diversity . . . 34
5.6.1 Number of monomorphic markers per population . . . 37
5.6.2 Nucleotide diversity and inbreeding . . . 37
5.7 Genetic differentiation . . . 39
5.8 PCA . . . 43
5.9 DAPC . . . 48
5.9.1 Optimal number of clusters with neutral markers . . . 48
5.9.2 Cross-validation to identify the optimal number of PCs . . . 49
5.9.3 DAPC barplot . . . 51
5.9.4 DAPC scatterplot . . . 56
5.9.5 DAPC scatterplot - FLO . . . 65

**6 DArTseq - marker selection 71**
6.1 Sex-linked markers . . . 71
6.2 Popgen markers . . . 71
6.2.1 Load data . . . 71
6.2.2 Extra filters . . . 71
6.3 Test selected markers . . . 73
6.3.1 Load data . . . 74
6.3.2 Genetic diversity . . . 75
6.3.3 Genetic differentiation . . . 80
6.3.4 PCA . . . 84

**7 DATA1: DArTcap - All data 89**
7.1 Set strata . . . 89
7.2 Test baiting success . . . 89
7.3 Find sex-linked markers . . . 89
7.4 Filter data . . . 98
7.5 Save data . . . 99
7.6 Load data . . . 100
7.7 Remove sex-linked markers . . . 100
7.8 PCA to identify species . . . 101

**8 DATA2: DArTcap - Only Bull Shark 109**
8.1 Set strata . . . 109
8.2 Filter data . . . 109
8.2.1 Remove extra low and high heterozygous individuals . . . 110
8.3 Save data . . . 110
8.4 Load data . . . 110
8.5 Remove sex-linked markers . . . 111
8.6 Genetic diversity . . . 112
8.6.1 Number of monomorphic markers per population . . . 119
8.6.2 Nucleotide diversity and inbreeding . . . 120
8.7 Genetic differentiation . . . 124
8.7.1 Fst per location . . . 124
8.7.2 Fst per Site . . . 136
8.7.3 Fst per Region . . . 140
8.8 PCA . . . 142
8.9 DAPC . . . 146
8.9.1 Optimal number of clusters with neutral markers . . . 146
8.9.2 Cross-validation to identify the optimal number of PCs . . . 147
8.9.3 DAPC barplot . . . 149
8.9.4 DAPC scatterplot . . . 154
8.9.5 DAPC scatterplot - FLO . . . 163
8.10 Provenance assignment . . . 168
8.10.1 Per Site . . . 168

8.10.2 Per region . . . 175
8.10.3 Test number of informative markers chosen by DAPC contribution . . . 181
8.11 Sex-specific connectivity . . . 185
8.11.1 Sex-linked markers . . . 185
8.11.2 Individual-based tests by Site . . . 206
8.11.3 Individual-based tests by Region . . . 220

**9 DATA3: DArTcap - All data, subset Australia 232**
9.1 Set strata . . . 232
9.2 Filter data . . . 232
9.2.1 Remove extra low and high heterozygous individuals . . . 233
9.3 Save data . . . 233
9.4 Load data . . . 234
9.5 Remove sex-linked markers . . . 235
9.6 Genetic diversity . . . 235
9.6.1 Number of monomorphic markers per population . . . 238
9.6.2 Nucleotide diversity and inbreeding . . . 238
9.7 Genetic differentiation . . . 240
9.8 PCA . . . 249
9.9 DAPC . . . 254
9.9.1 Optimal number of clusters with neutral markers . . . 254
9.9.2 Cross-validation to identify the optimal number of PCs . . . 255
9.9.3 DAPC barplot . . . 257
9.9.4 DAPC scatterplot . . . 262
9.9.5 DAPC scatterplot - FLO . . . 271

**10 DATA4: DArTcap - West-Atlantic 277**
10.1 Set strata . . . 277
10.2 Filter data . . . 277
10.2.1 Remove extra low and high heterozygous individuals . . . 278
10.3 Save data . . . 278
10.4 Load data . . . 278
10.5 Remove sex-linked markers . . . 279
10.6 Genetic diversity . . . 280
10.6.1 Number of monomorphic markers per population . . . 280
10.6.2 Nucleotide diversity and inbreeding . . . 281
10.7 Genetic differentiation . . . 283
10.8 PCA . . . 285
10.9 DAPC . . . 289
10.9.1 Optimal number of clusters with neutral markers . . . 289
10.9.2 Cross-validation to identify the optimal number of PCs . . . 290
10.9.3 DAPC barplot . . . 292
10.9.4 DAPC scatterplot . . . 297
10.9.5 DAPC scatterplot - FLO . . . 306

**11 DATA5: DArTcap - Indo - West Pacific 312**
11.1 Set strata . . . 312
11.2 Filter data . . . 312
11.2.1 Remove extra low and high heterozygous individuals . . . 313
11.3 Save data . . . 313
11.4 Load data . . . 314
11.5 Remove sex-linked markers . . . 314
11.6 Genetic diversity . . . 315
11.6.1 Number of monomorphic markers per population . . . 318
11.6.2 Nucleotide diversity and inbreeding . . . 318
11.7 Genetic differentiation . . . 320
11.8 PCA . . . 328
11.9 DAPC . . . 332
11.9.1 Optimal number of clusters with neutral markers . . . 332

11.9.2 Cross-validation to identify the optimal number of PCs . . . 333
11.9.3 DAPC barplot . . . 335
11.9.4 DAPC scatterplot . . . 340
11.9.5 DAPC scatterplot - FLO . . . 349

**12 DATA6: DArTcap - Indo - West Pacific: no Japan or Fiji and subset from Australia 355**
12.1 Set strata . . . 355
12.2 Filter data . . . 355
12.2.1 Remove extra low and high heterozygous individuals . . . 356
12.3 Save data . . . 356
12.4 Load data . . . 356
12.5 Remove sex-linked markers . . . 357
12.6 Genetic diversity . . . 358
12.6.1 Number of monomorphic markers per population . . . 359
12.6.2 Nucleotide diversity and inbreeding . . . 360
12.7 Genetic differentiation . . . 361
12.8 PCA . . . 363
12.9 DAPC . . . 367
12.9.1 Optimal number of clusters with neutral markers . . . 367
12.9.2 Cross-validation to identify the optimal number of PCs . . . 368
12.9.3 DAPC barplot . . . 370
12.9.4 DAPC scatterplot . . . 375
12.9.5 DAPC scatterplot - FLO . . . 384

**13 Mitogenome analyses 390**
13.1 Sequencing primers . . . 390
13.2 Preparing the mitogenome data files . . . 390
13.2.1 MultiDNA format . . . 390
13.2.2 Genind format . . . 390
13.2.3 Add metadata . . . 390
13.2.4 Gtypes format . . . 391
13.2.5 Haplotype format . . . 391
13.2.6 Save data . . . 392
13.2.7 Load data . . . 392
13.3 Genetic diversity . . . 394
13.3.1 Overall diversity . . . 394
13.3.2 Diversity per location . . . 401
13.3.3 Diversity per Site . . . 418
13.3.4 Diversity per region . . . 425
13.4 Haplotype network . . . 431
13.4.1 Rarefaction . . . 431
13.4.2 Network . . . 433
13.5 Genetic differentiation . . . 462
13.5.1 PHIst per Location . . . 462
13.5.2 PHIst per Site . . . 467
13.5.3 PHIst per region . . . 469
13.6 PCA . . . 472

**14 References 474**

**15 Citations for packages 474**

**16 Session Info 476**

---

# 1 Background

This Rmarkdown document described the data-filtering and analysis steps for assessing the population structure of the Bull Shark (*Carcharhinus leucas*) in Devloo-Delva et al. Basically, we followed a step-wise procedure to analyse the data at different hierarchies and with different subsets. Each time the data was sub-sampled, we re-filtered the data:

1. Hundred-eighty-eight sharks, with 32 technical replicates, were sequenced and genotyped according to the DArTseq protocol (see Feutry et al. 2017) to yield 250,950 single nucleotide polymorphisms (SNPs).
    - The raw DArTseq data was investigated to find putative sex-linked markers (i.e. markers located on the sex chromosomes), as these markers can assign the genetic sex and could potentially bias the population structure analyses (see Benestan et al. 2017).
    - The data was loosely filtered to identify incorrect species in our data using a simple principal component analysis.
    - The data with only confirmed Bull Sharks was used to select approximately 3400 SNPs to design targeted capture probes for DArTcap genotyping (see Feutry et al. 2020).
    - We also investigated population structure and genetic diversity within Australia, where sample sizes were largest.
2. We analysed the resulting DArTcap SNPs for 940 individuals and an additional 74 technical replicates.
    - The raw DArTcap data was again tested to identify sex-linked markers.
    - We applied a loose data quality filtering to identify incorrect species.
    - The data with only Bull Sharks was analysed for genetic diversity and population structure
3. Subsequently, we randomly subsampled 60 individuals from Australia to reduce the bias that was caused by extreme unequal sample sizes (see Puechmaille 2016; Foster et al. 2018).
4. Within the West-Atlantic population, we looked for signals of (sub)structure that could have been hidden due to the extreme differentiation from other populations.
5. Within the Indo-West Pacific (IWP) population, we looked for signals of (sub)structure that could have been hidden due to the extreme differentiation from other populations. In addition, we looked for close-kin relations within the IWP, given the large sample size.
6. Within Australia, we investigated the potential that rivers or regions form fine-scale structure.
7. Lastly, we amplified the full mitochondrial genome (mitogenome) of 361 Bull Sharks from the eastern Atlantic and Indo-Pacific regions.
    - Genetic diversity
    - Haplotype networks
    - Genetic differentiation

# 2 Sampling map and metadata

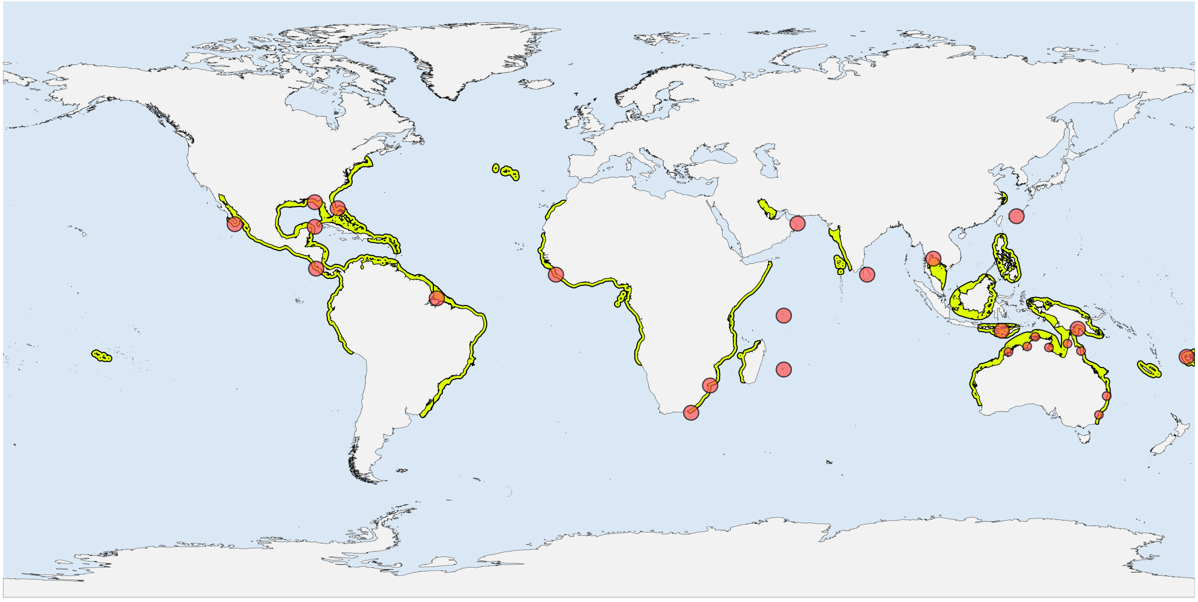

## 2.1 Visual sex ID

```
metafile <- "1.BullShark_AllSamples_metadata.csv"
meta <- readr::read_csv(metafile) %>%
  dplyr::filter(SpeciesID_genetic == "Carcharhinus leucas")
pop.levels <- c("Gulf of California", "Costa Rica" , "Caribbean Sea",
                "Gulf of Mexico", "Western North Atlantic", "Brazil",
                "Sierra Leone", "South Africa", "Mozambique", "Reunion" ,
                "Seychelles", "Arabian Sea", "Sri Lanka", "Thailand",
                "Indonesia", "Papua New Guinea" , "Australia", "Japan", "Fiji")
meta$Site <- factor(meta$Site, levels = pop.levels)
meta$Date_sampled <- as.Date(meta$Date_sampled, "%d/%m/%Y")
```

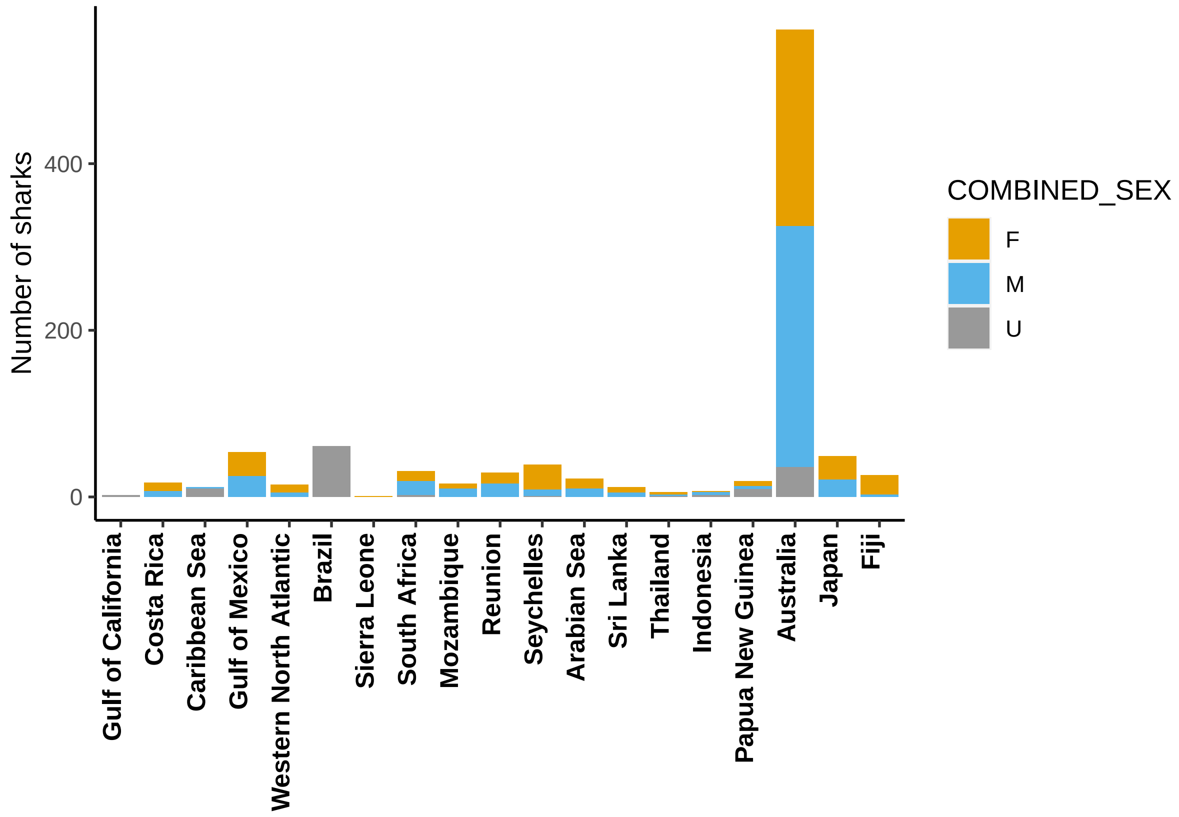


## 2.2 Total lengths

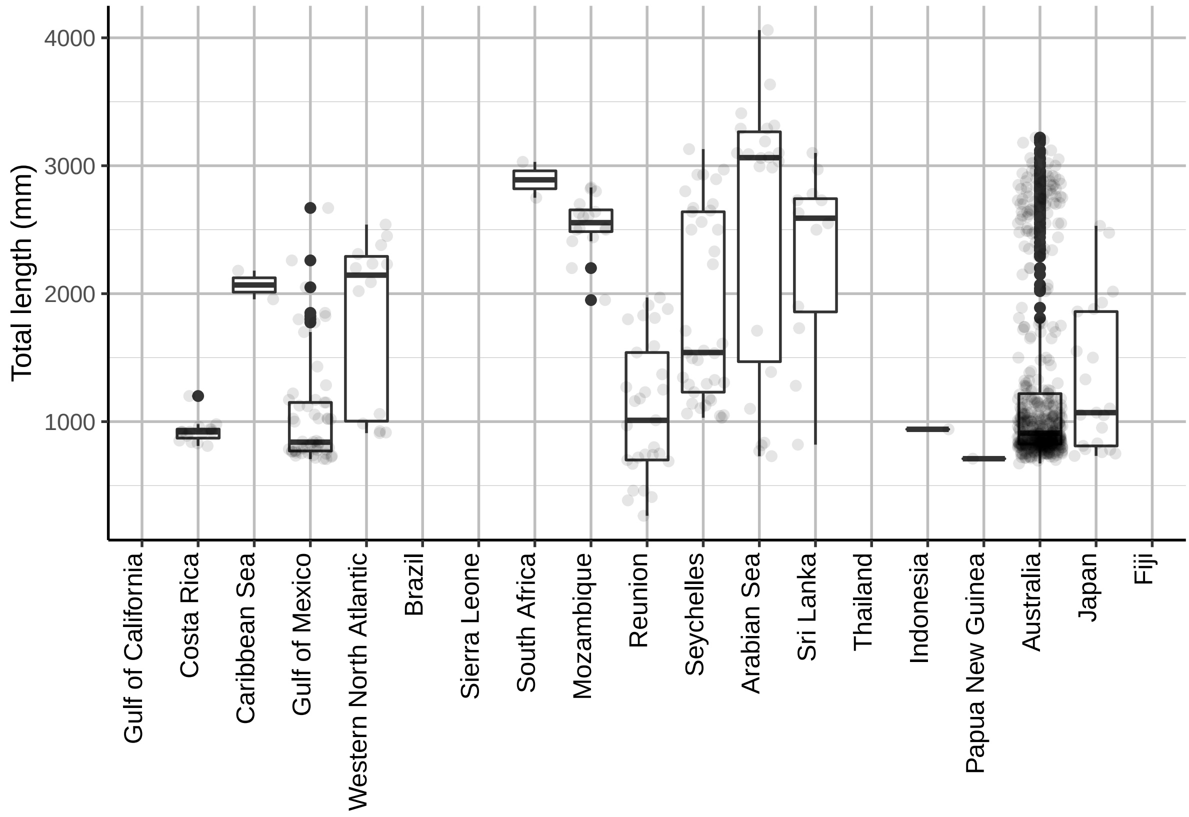

## 2.3 Aproximate Length-at-age

Based on length-at-age function from Tillet et al. (2011).

"Model-averaged growth parameters for these length-at-age data are theoretical asymptotic length (Linf) (s.e.) = 3119 (9.803)mm and a growth coefficient (k) = 0.158 year-1 respectively. Age at maturity was 9.5 years and like pig-eye sharks, bull sharks are long-lived with a predicted longevity of >27 years (Table 2). Juvenile growth rate was 186 mm/year for the first five years and then slowed to 45 mm/year post-maturity."

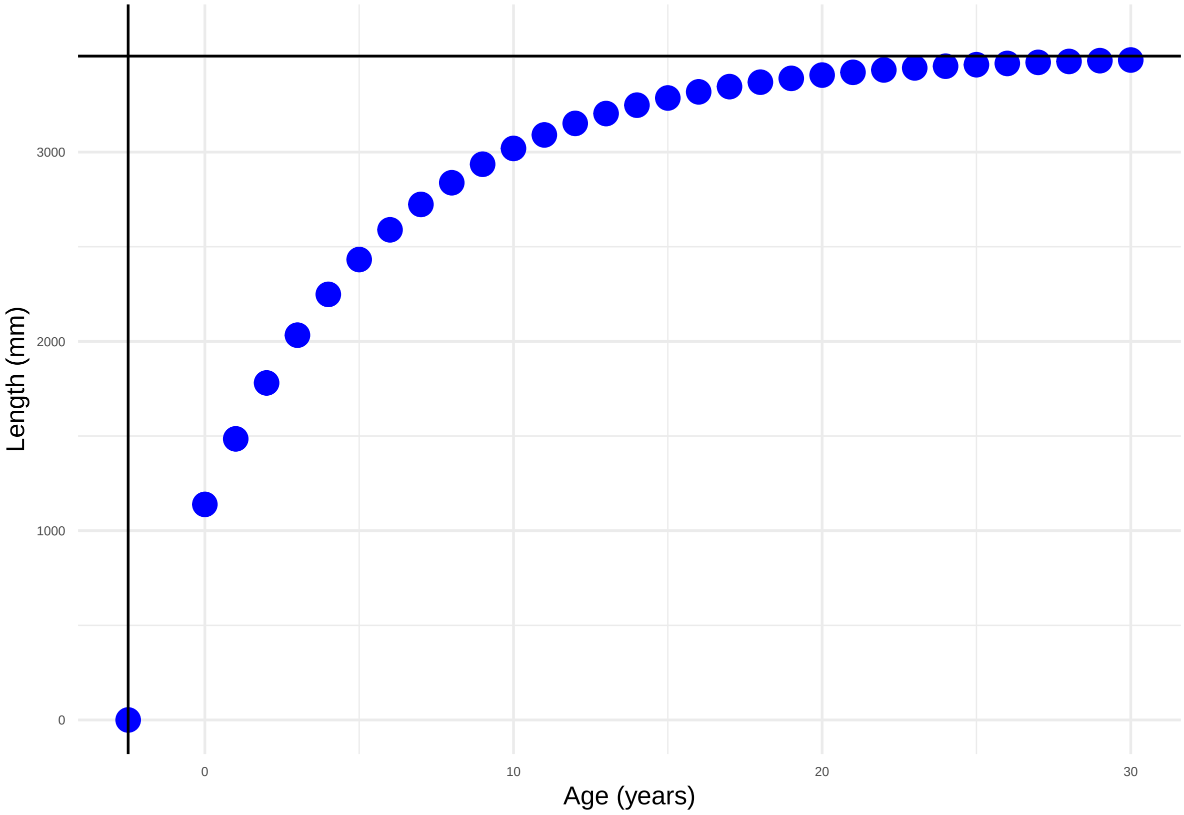

### 2.3.1 Catch date

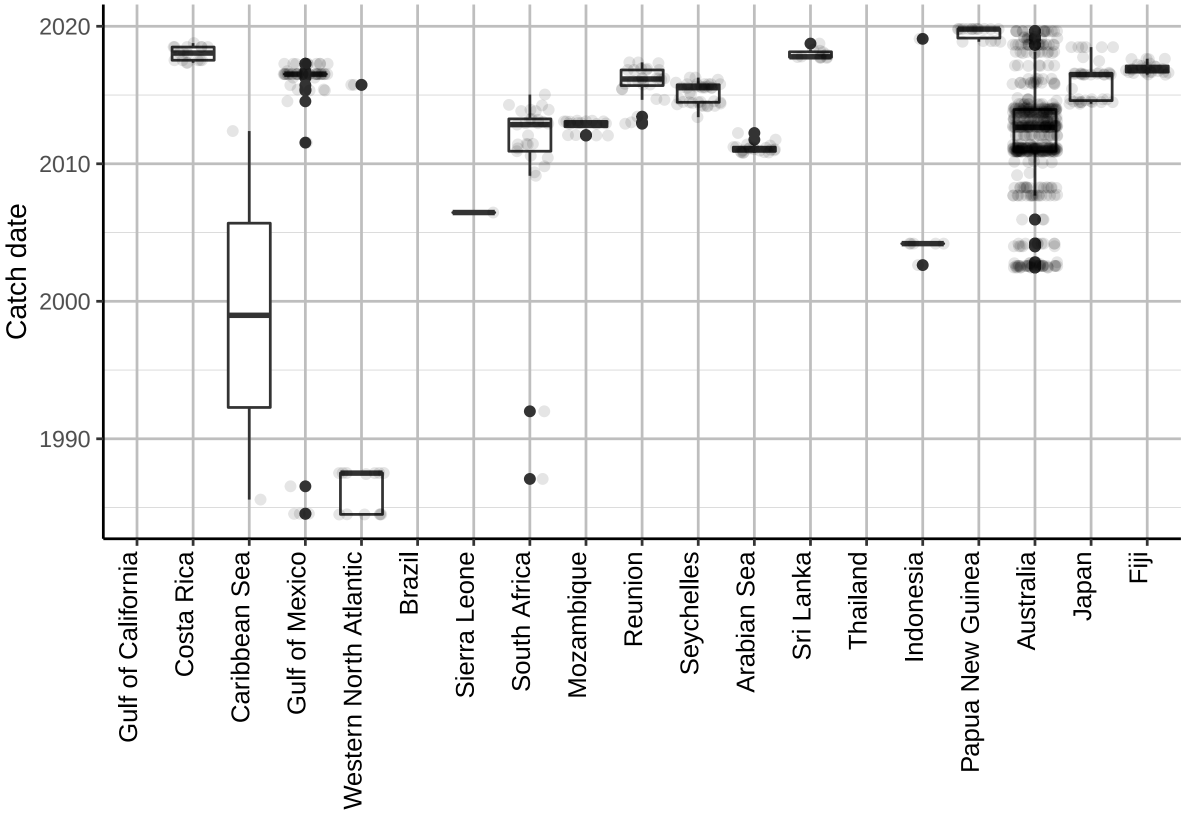


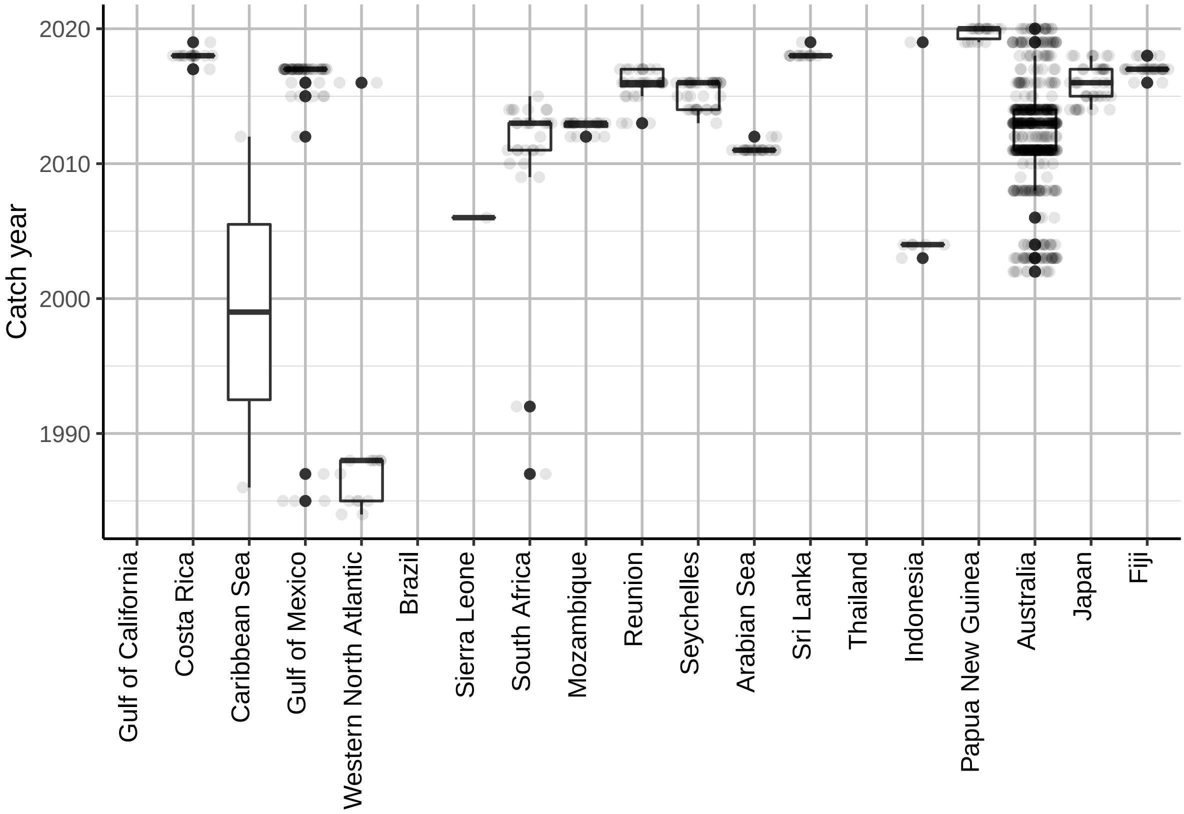

### 2.3.2 Age

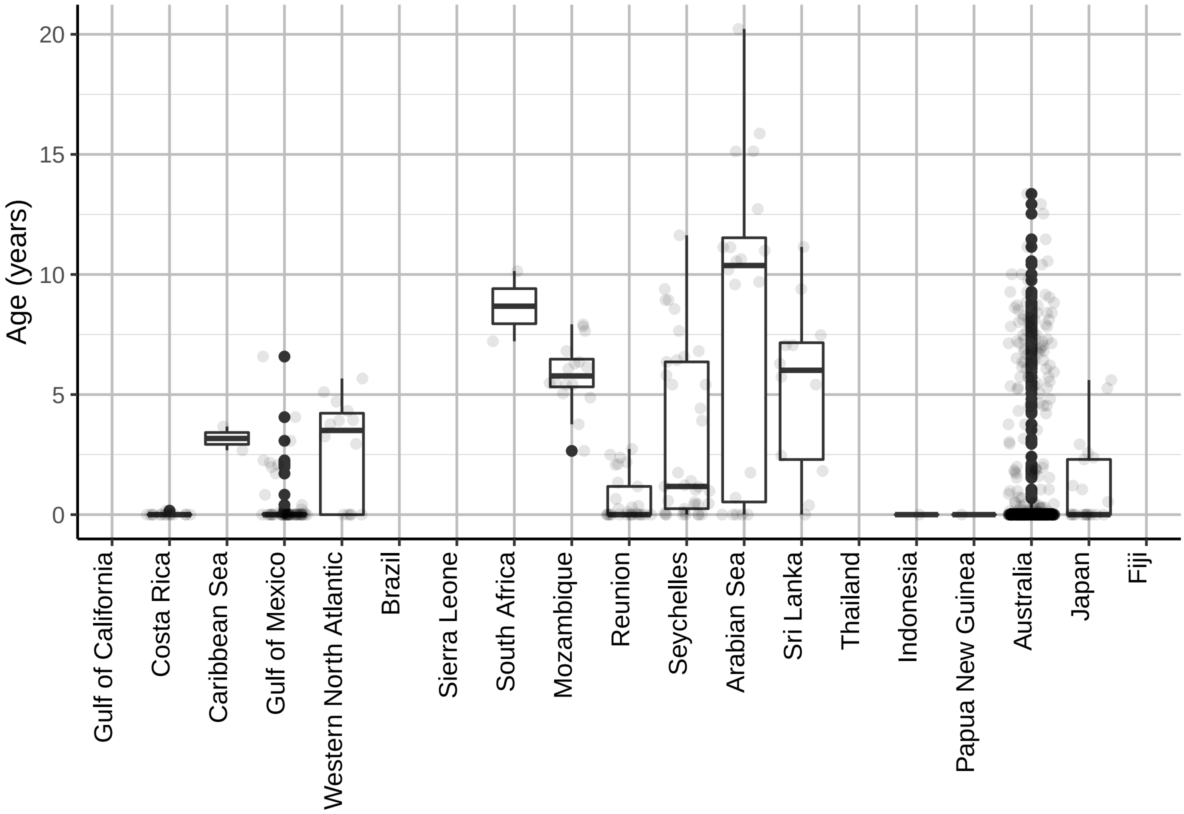


```
## [1] 4060
## [1] 2
```

### 2.3.3 Cohort

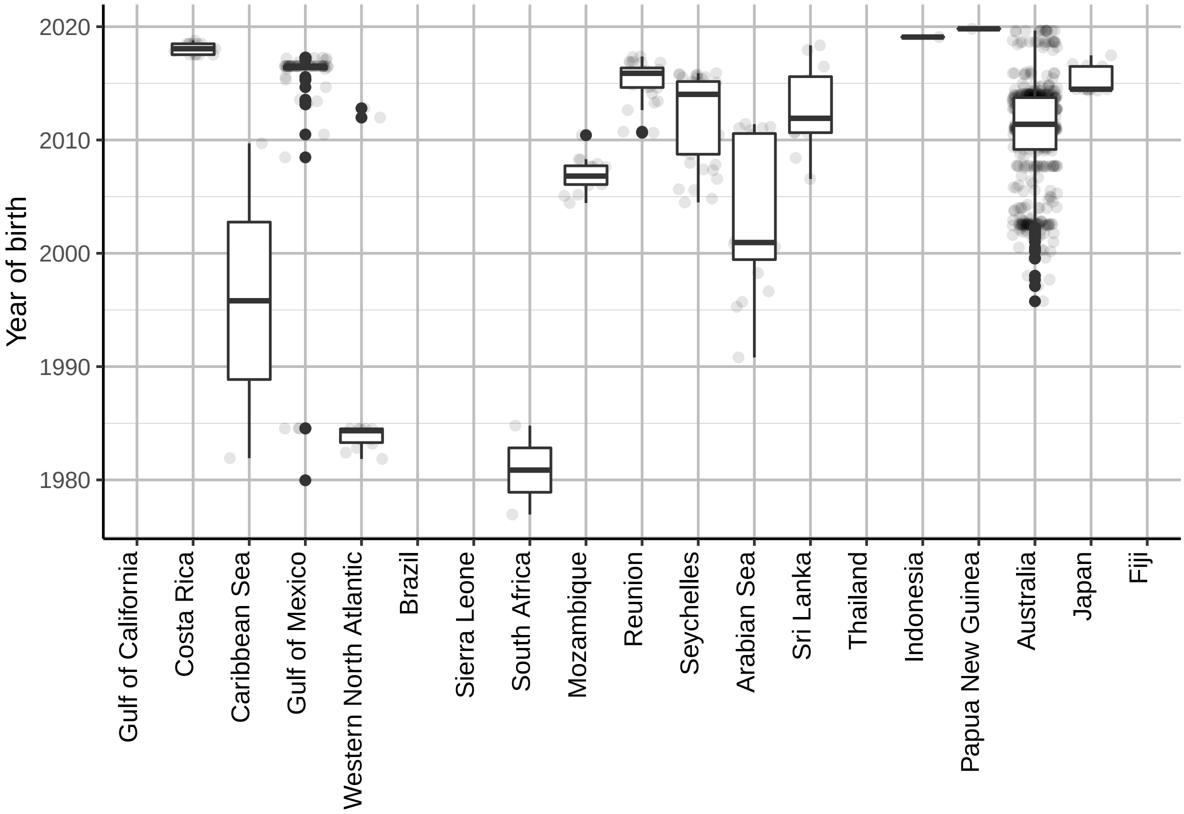

Year of birth
2020
2010
2000
1990
1980
Gulf of California
Costa Rica
Caribbean Sea
Gulf of Mexico
Western North Atlantic
Brazil
Sierra Leone
South Africa
Mozambique
Reunion
Seychelles
Arabian Sea
Sri Lanka
Thailand
Indonesia
Papua New Guinea
Australia
Japan
Fiji


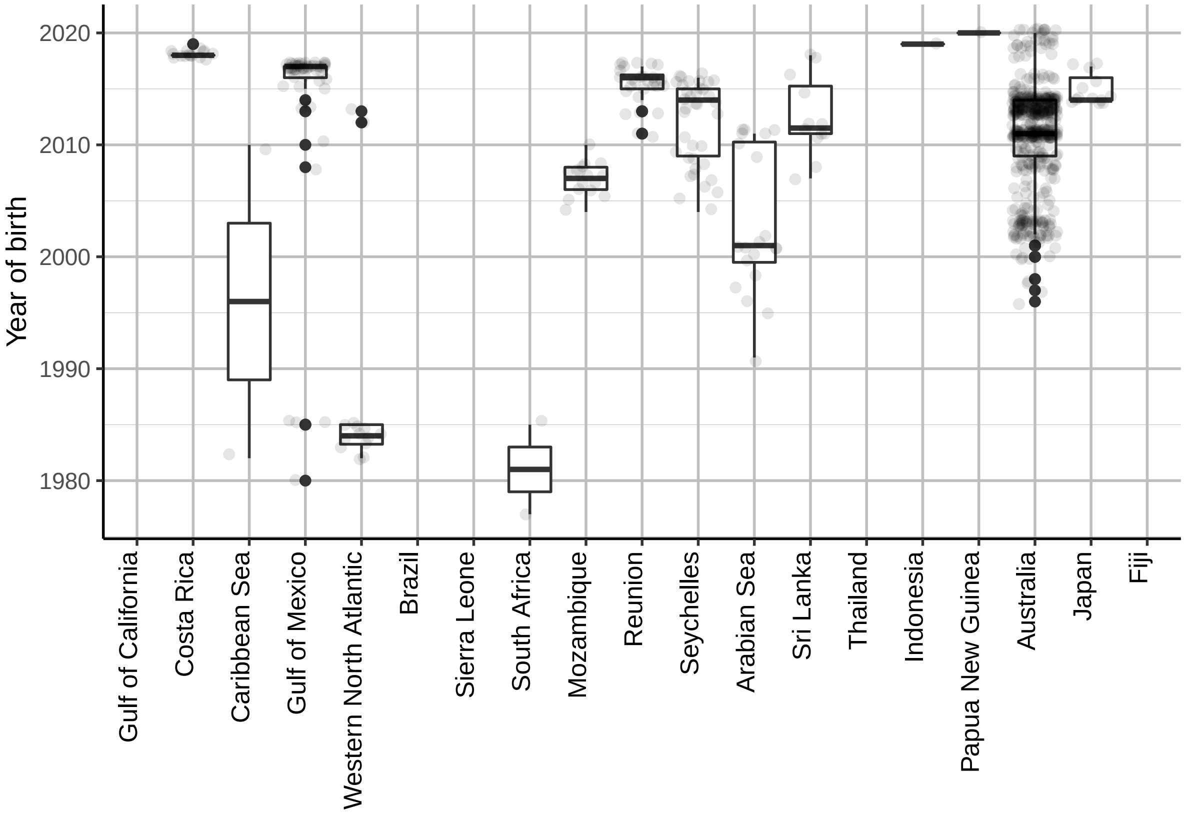

Year of birth
2020
2010
2000
1990
1980
Gulf of California
Costa Rica
Caribbean Sea
Gulf of Mexico
Western North Atlantic
Brazil
Sierra Leone
South Africa
Mozambique
Reunion
Seychelles
Arabian Sea
Sri Lanka
Thailand
Indonesia
Papua New Guinea
Australia
Japan
Fiji

# 3 Load packages

```r
library(radiator)
library(ggplot2)
library(dplyr)
library(tidyverse)
library(ade4)
library(adegenet)
library(ape)
library(apex)
library(assigner)
library(assignPOP)
library(dartR)
library(fossil)
library(haplotypes)
library(data.table)
library(ggplot2)
library(grid)
library(gridExtra)
library(gtools)
# library(HWxtest)
library(kableExtra)
library(knitr)
library(marmap)
library(magrittr)
library(mvbutils)
library(OutFLANK)
library(pegas)
library(qvalue)
library(rubias)
library(strataG)
library(vegan)
source("Filtering funtions.R")


#set colours

colours.prior <- adegenet::funky(10)

colours.4 <- adegenet::funky(4)
names(colours.4) <- c("Brazil","Caribbean Sea","Gulf of Mexico",
                      "Western North Atlantic")

colours.6 <- adegenet::funky(6)
names(colours.6) <- c("E-PAC","W-ATL","E-ATL","IWP", "Japan","Fiji")

colours.8 <- adegenet::funky(8)
names(colours.8) <- c("E-PAC","E-ATL", "W-IO",  "N-IO", "E-IO",  "W-PAC",
                      "Japan", "Fiji")

colours.10 <- adegenet::funky(10)
names(colours.10) <- c("South Africa", "Mozambique", "Reunion" , "Seychelles",
                       "Arabian Sea", "Sri Lanka", "Thailand", "Indonesia",
                       "Papua New Guinea" , "Australia")

colours.14 <- adegenet::funky(14)
names(colours.14) <- c("Gulf of California","Costa Rica", "Sierra Leone",
                       "South Africa","Reunion","Seychelles","Arabian Sea",
```

```
                         "Sri Lanka","Thailand","Indonesia","Papua New Guinea",
                         "Australia","Japan","Fiji")

colours.18 <- adegenet::funky(18)
names(colours.18) <- c("Costa Rica","Brazil", "Caribbean Sea", "Gulf of Mexico",
                         "Western North Atlantic",  "Sierra Leone", "South Africa",
                         "Mozambique", "Reunion","Seychelles","Arabian Sea",
                         "Sri Lanka","Thailand","Indonesia","Papua New Guinea",
                         "Australia","Japan","Fiji")

colours.13 <- adegenet::funky(13)
names(colours.13) <- c("Fitzroy River","Victoria River","Daly River","Adelaide River",
                         "South Alligator River","East Alligator River","Blue mud Bay",
                         "Roper River","Towns River", "Wenlock River","Trinity inlet",
                         "Clarence River","Sydney Harbour")


colours.26 <- adegenet::funky(26)
names(colours.26) <- c("South Africa", "Mozambique", "Reunion" , "Seychelles",
                         "Arabian Sea", "Sri Lanka", "Thailand", "Indonesia",
                         "Papua New Guinea" , "Fitzroy River", "Daly River",
                         "Adelaide River","Darwin Coastal",  "South Alligator River",
                         "East Alligator River","Blue mud Bay", "Roper River",
                         "Towns River","Unknown","Wenlock River","Trinity inlet",
                         "Clarence River", "Sydney Harbour","Okinawa", "Urauchi River",
                         "Fiji")

colours.27 <- adegenet::funky(27)
names(colours.27) <- c("Gulf of California","Costa Rica", "Sierra Leone",
                         "South Africa","Reunion","Seychelles","Arabian Sea",
                         "Sri Lanka","Thailand","Indonesia","Papua New Guinea",
                         "Fitzroy River", "Victoria River","Daly River",
                         "Adelaide River","South Alligator River", "East Alligator River",
                         "Blue mud Bay", "Roper River","Towns River",
                         "Wenlock River","Trinity inlet","Clarence River",
                         "Sydney Harbour","Okinawa", "Urauchi River", "Fiji")

colours.29 <- adegenet::funky(29)
names(colours.29) <- c("Brazil","Caribbean Sea","Gulf of Mexico",
                         "Western North Atlantic", "Sierra Leone", "South Africa",
                         "Mozambique", "Reunion" , "Seychelles", "Arabian Sea",
                         "Sri Lanka", "Thailand", "Indonesia", "Papua New Guinea" ,
                         "Fitzroy River", "Daly River", "Adelaide River",
                         "South Alligator River", "East Alligator River","Blue mud Bay",
                         "Roper River","Towns River","Wenlock River","Trinity inlet",
                         "Clarence River", "Sydney Harbour","Okinawa", "Urauchi River",
                         "Fiji")

colours.32 <- adegenet::funky(32)
names(colours.32) <- c("Costa Rica" , "Brazil","Caribbean Sea","Gulf of Mexico",
                         "Western North Atlantic", "Sierra Leone", "South Africa",
                         "Mozambique", "Reunion" , "Seychelles", "Arabian Sea",
                         "Sri Lanka", "Thailand", "Indonesia", "Papua New Guinea" ,
                         "Fitzroy River", "Daly River", "Adelaide River","Darwin Coastal",
                         "South Alligator River", "East Alligator River","Blue mud Bay",
                         "Roper River","Towns River","Unknown","Wenlock River",
                         "Trinity inlet","Clarence River", "Sydney Harbour", "Okinawa",
                         "Urauchi River", "Fiji")
```

# 4 DArTseq - All data

We sequenced 188 putative Bull Shark samples with the DArTseq protocol (according to Feutry et al. 2017). Thirty-two sample replicates were included to test the reproducibility of the genotyping.

These DArTseq samples were used to identify sex-linked markers, species and population structure, and subsequently select ~35000 markers for capture probe design.

## 4.1 Set strata

One sample failed library construction

```
data.file.snp <- "DArTseq_SNP_OrderAppendix_2_DCarcha19-4454.csv"
targetid <- readr::read_tsv("DArTseq_target.id.tsv")

strata <- readr::read_csv(metafile) %>%
  dplyr::left_join(targetid, by = "INDIVIDUALS") %>%
  dplyr::filter(!is.na(TARGET_ID)) %>%
  dplyr::mutate(STRATA = Site2)
strata$INDIVIDUALS <- with(strata, make.unique(as.character(INDIVIDUALS)))
# readr::write_tsv(strata, path = "Bull_shark_DArTseq_strata.tsv")
```

## 4.2 Find sex-linked markers

Sex markers are identified as per Devloo-Delva et al. (2022 preprint).

```
data <- "DArTseq_SNP_OrderAppendix_2_DCarcha19-4454.csv"
silicodata <- "DArTseq_SILICO_OrderAppendix_1_DCarcha19-4454.csv"
strata <- radiator::read_strata("Bull_shark_DArTseq_strata.tsv",
                                keep.two = FALSE)$strata
strata %<>% dplyr::rename(POP = "STRATA") %>%
  dplyr::rename(STRATA = "Sex")

bull_shark <- radiator::sexy_markers(
  data = data,
  silicodata = silicodata,
  strata = strata,
  interactive.filter = TRUE)
save(bull_shark, file = "Sex_results.Rdata")
```

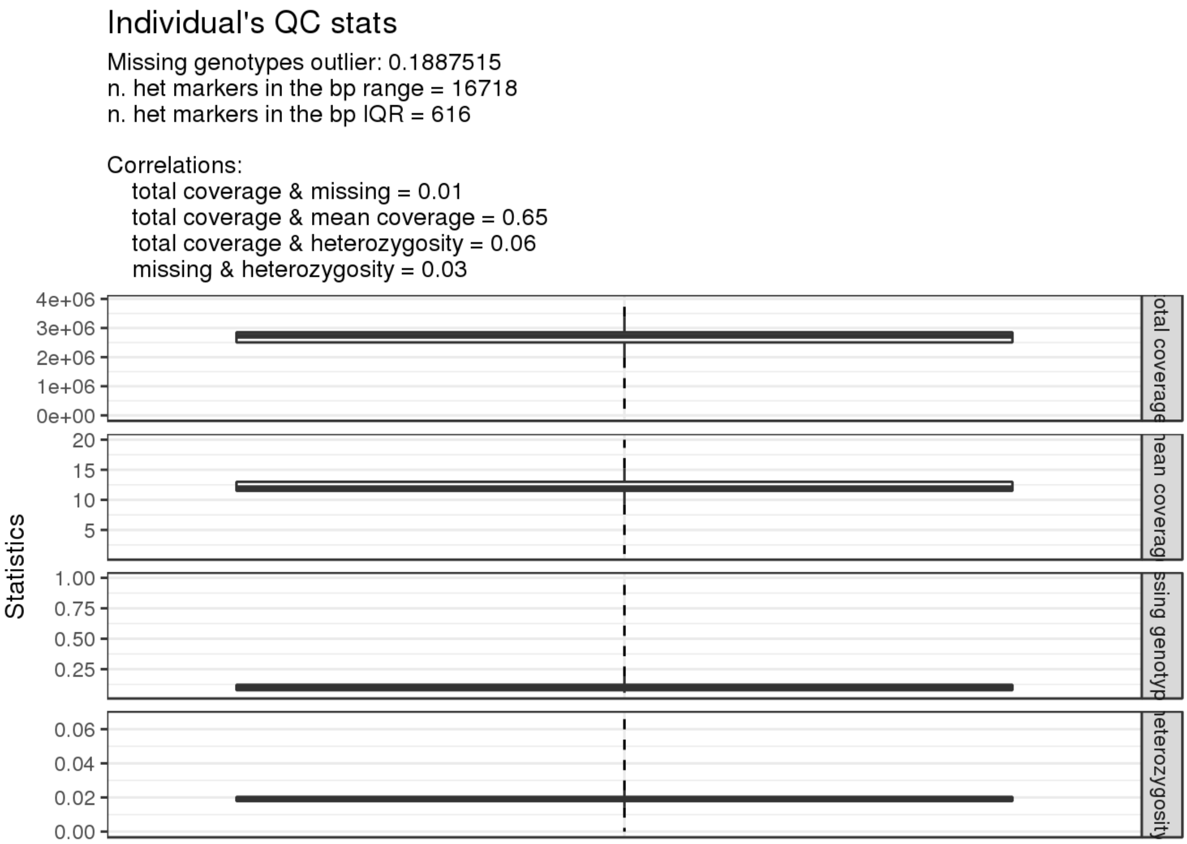
Individual's QC stats
Missing genotypes outlier: 0.1887515
n. het markers in the bp range = 16718
n. het markers in the bp IQR = 616
Correlations:
total coverage & missing = 0.01
total coverage & mean coverage = 0.65
total coverage & heterozygosity = 0.06
missing & heterozygosity = 0.03
Statistics
4e+06
3e+06
2e+06
1e+06
0e+00
otal coverage
20
15
10
5
nean coverag
1.00
0.75
0.50
0.25
ssing genotyp
0.06
0.04
0.02
0.00
eterozygosity

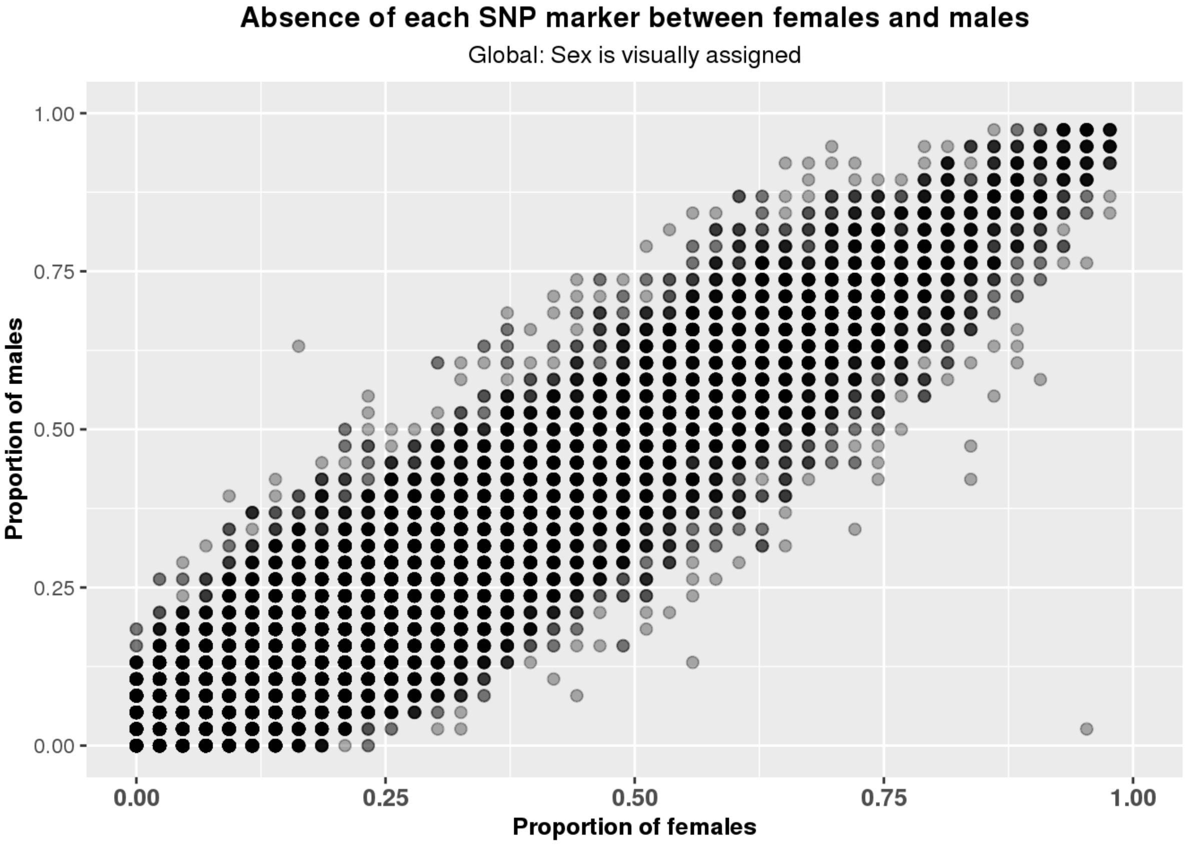
Absence of each SNP marker between females and males
Global: Sex is visually assigned
Proportion of males
1.00
0.75
0.50
0.25
0.00
0.00
0.25
0.50
0.75
1.00
Proportion of females

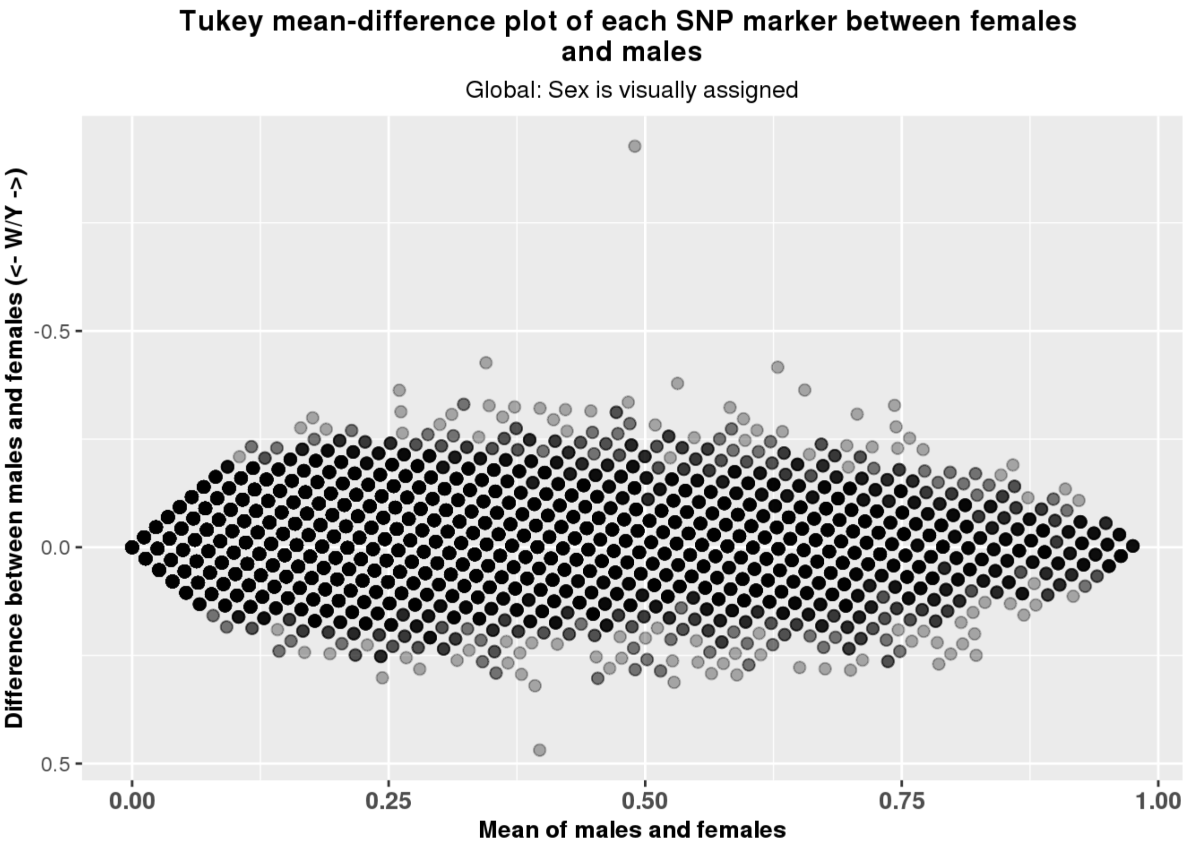
Tukey mean-difference plot of each SNP marker between females and males
Global: Sex is visually assigned
Difference between males and females (<- W/Y ->)
-0.5
0.0
0.5
0.00
0.25
0.50
0.75
1.00
Mean of males and females

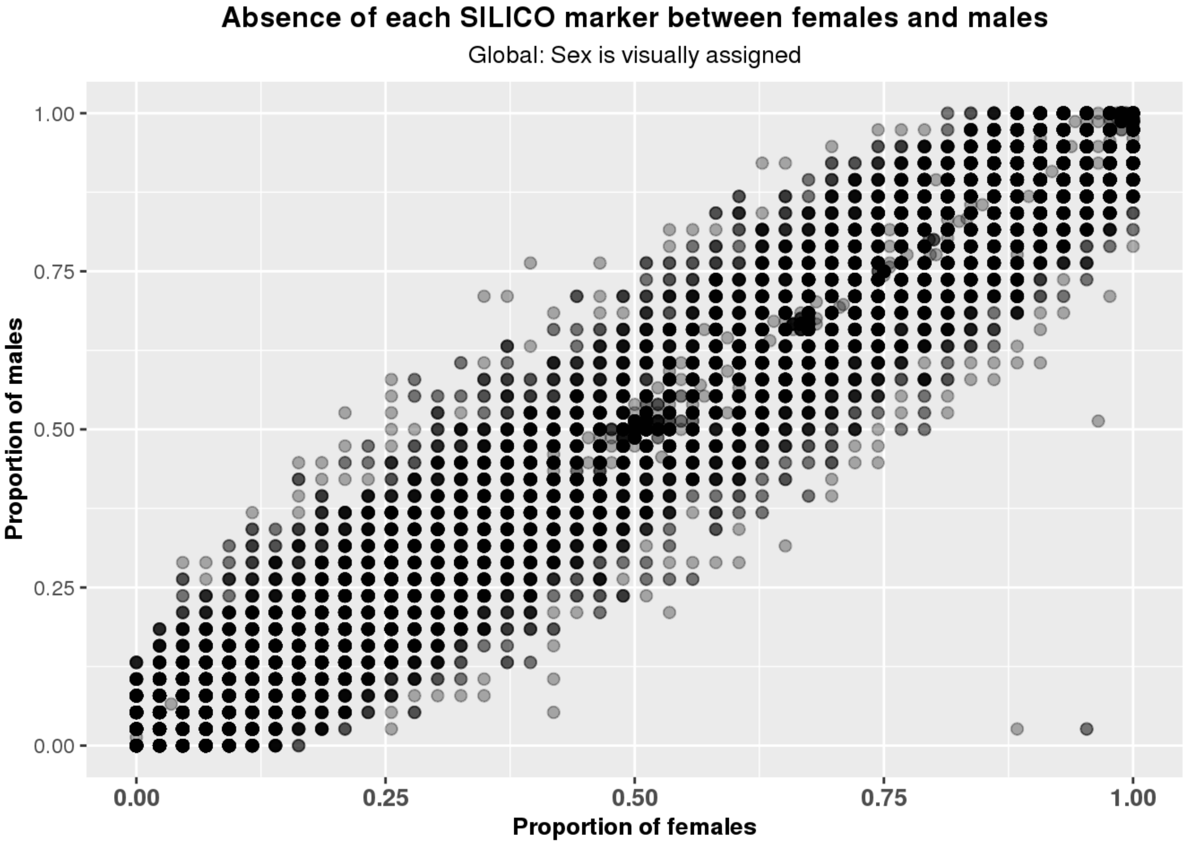
Absence of each SILICO marker between females and males
Global: Sex is visually assigned
Proportion of males
1.00
0.75
0.50
0.25
0.00
0.00
0.25
0.50
0.75
1.00
Proportion of females

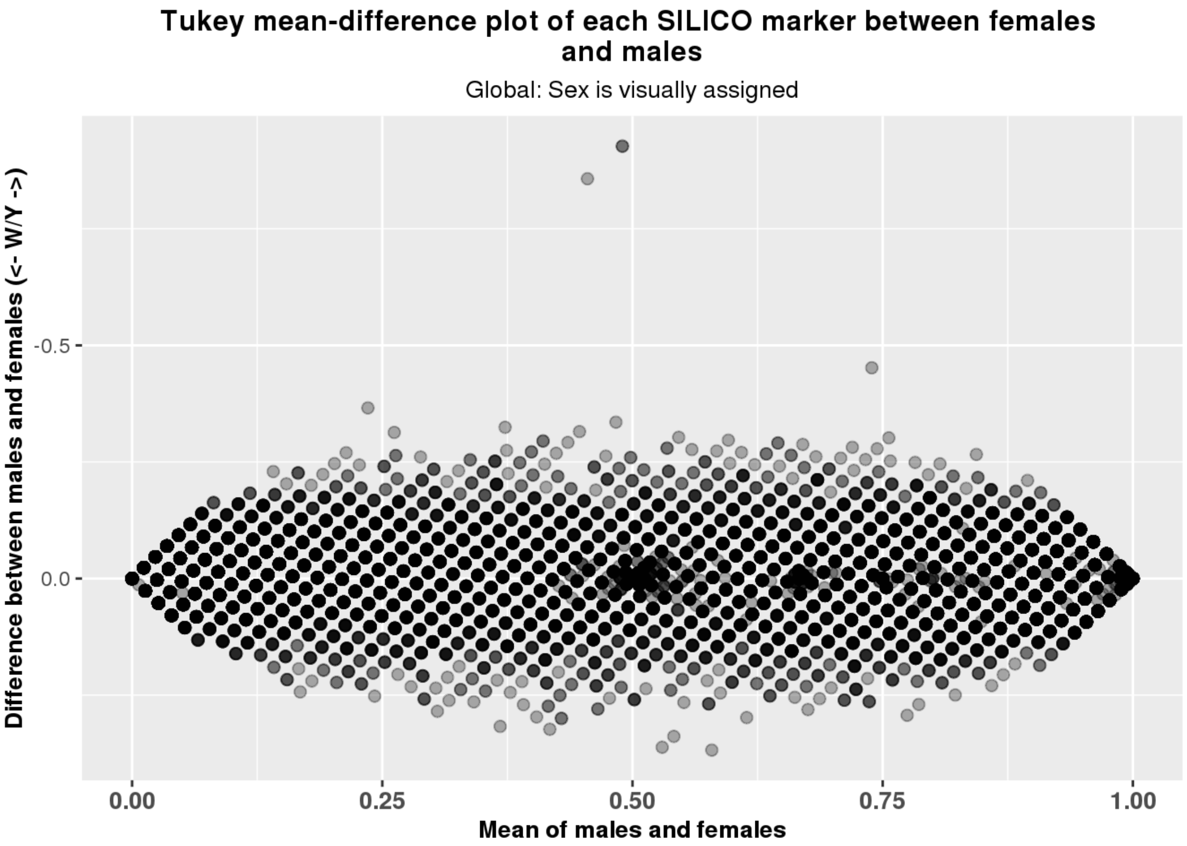
Tukey mean-difference plot of each SILICO marker between females and males
Global: Sex is visually assigned
Difference between males and females (<- W/Y ->)
-0.5
0.0
0.00
0.25
0.50
0.75
1.00
Mean of males and females

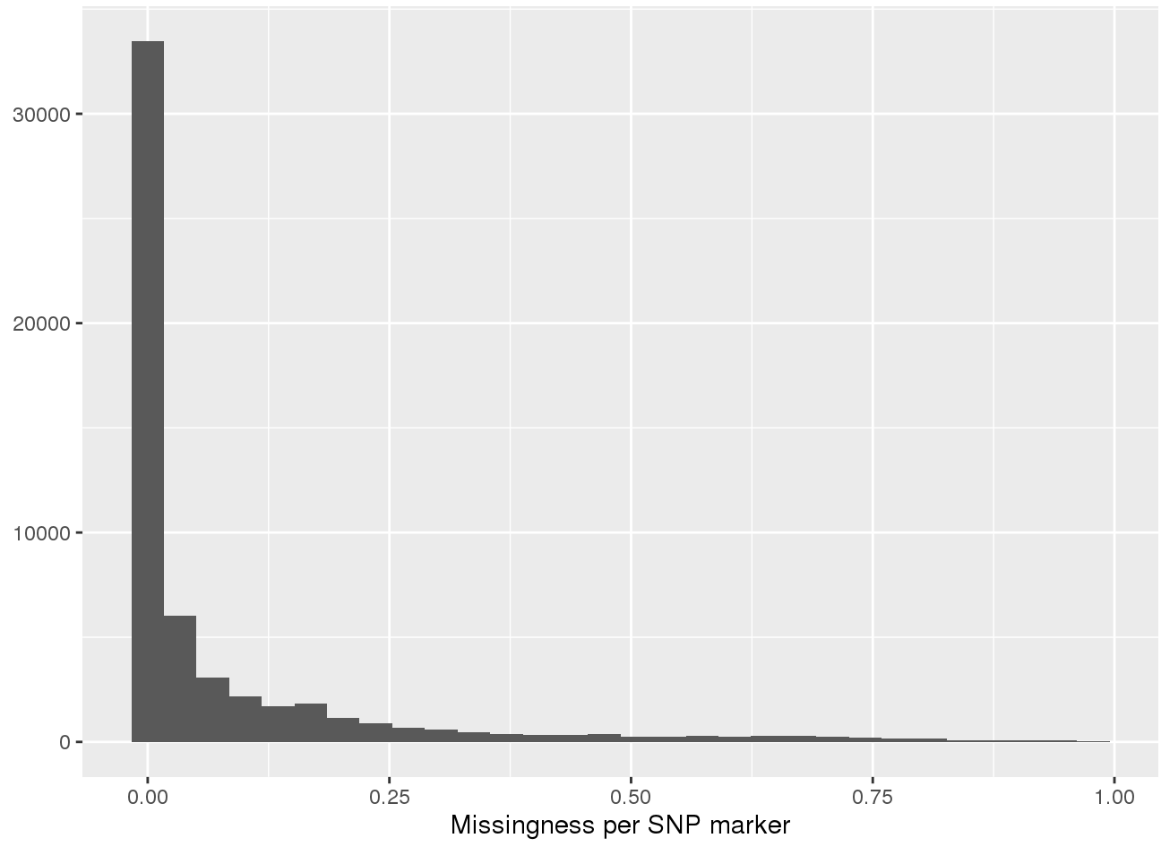
30000
20000
10000
0
0.00
0.25
0.50
0.75
1.00
Missingness per SNP marker

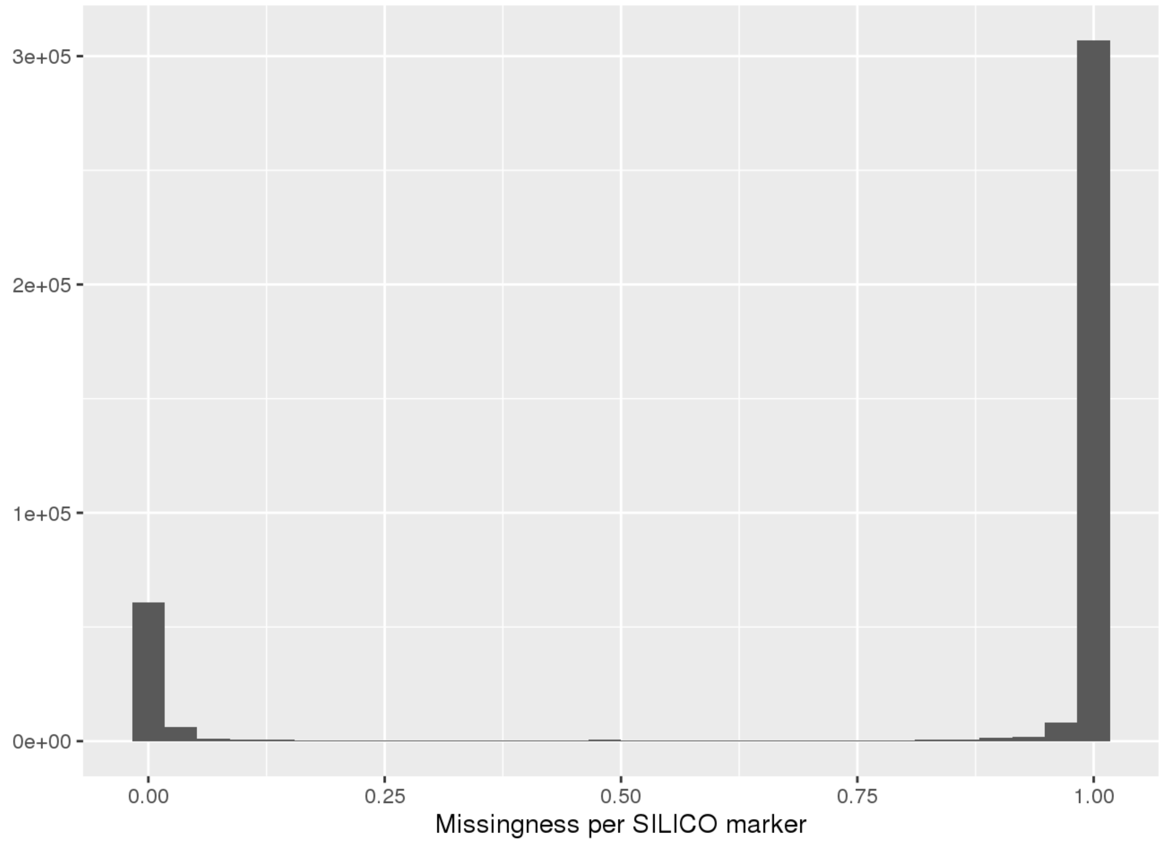
3e+05
2e+05
1e+05
0e+00
0.00
0.25
0.50
0.75
1.00
Missingness per SILICO marker

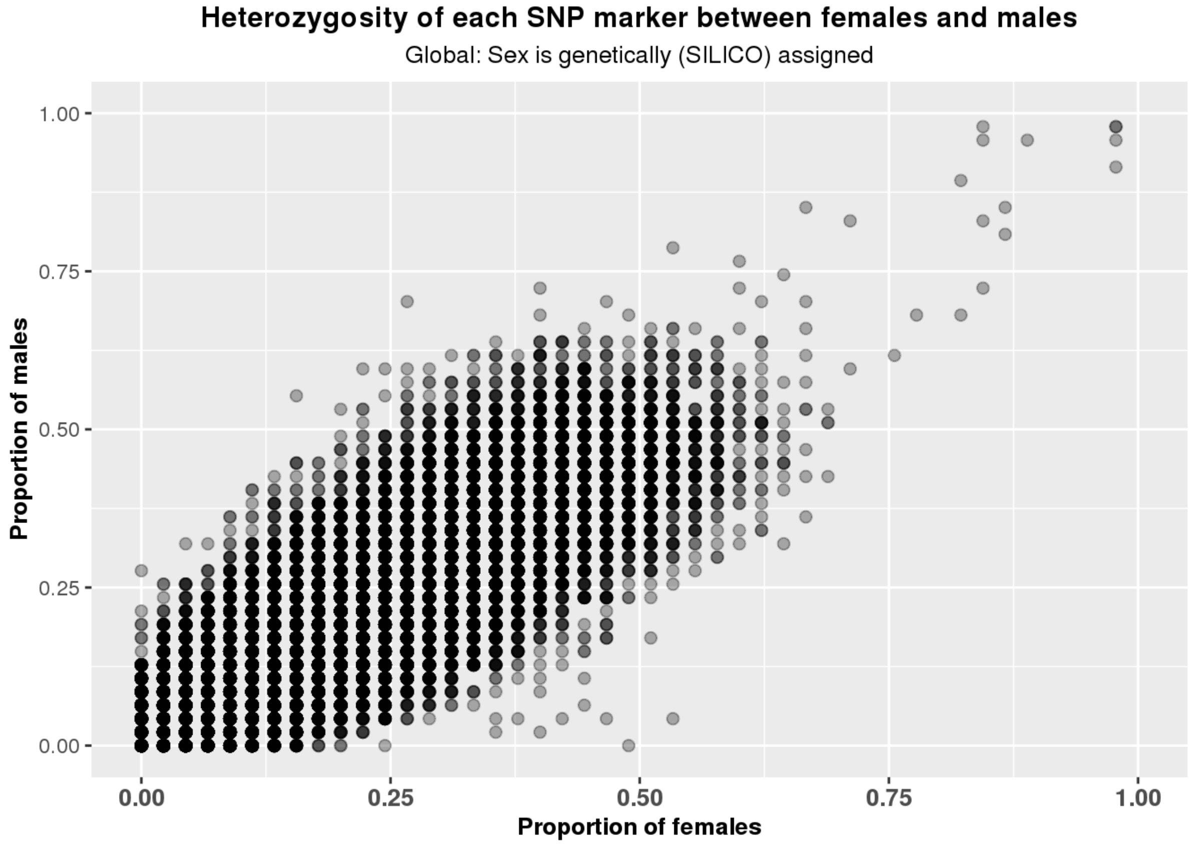
Heterozygosity of each SNP marker between females and males
Global: Sex is genetically (SILICO) assigned
Proportion of males
1.00
0.75
0.50
0.25
0.00
0.00
0.25
0.50
0.75
1.00
Proportion of females

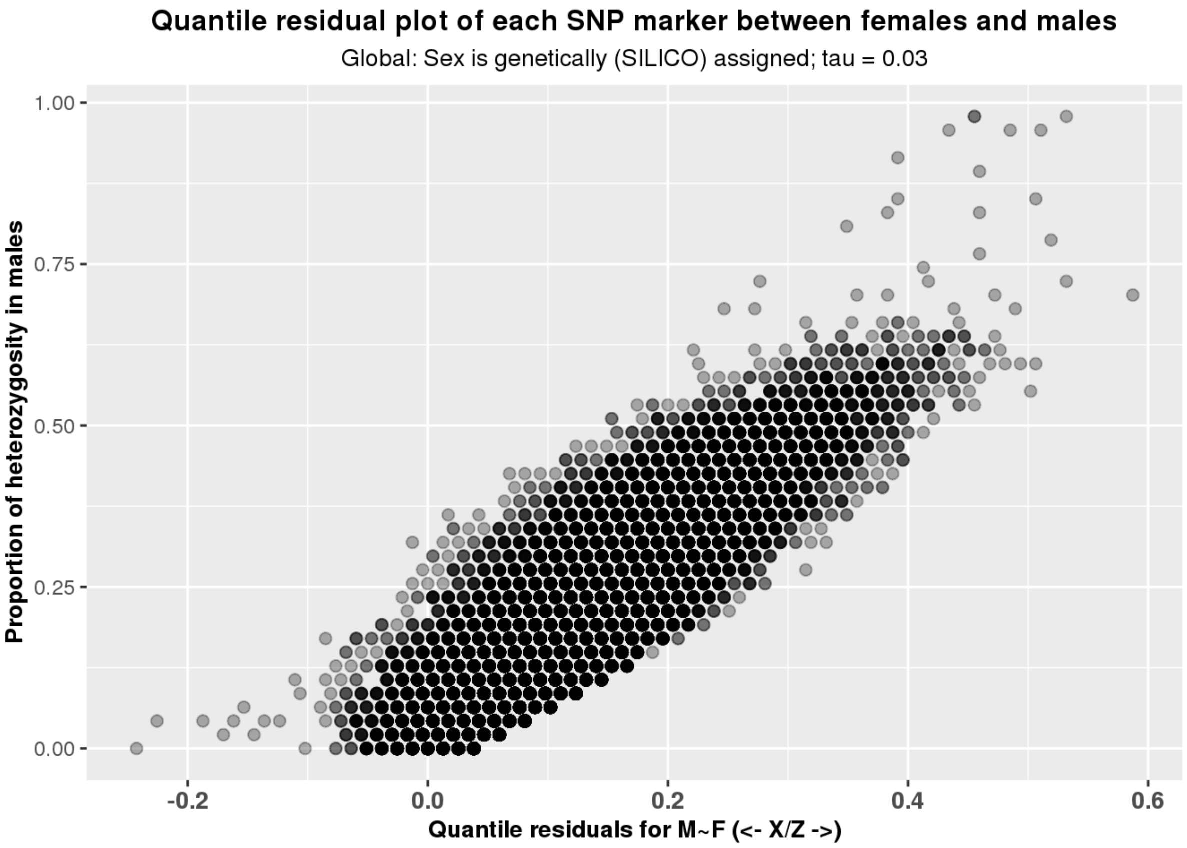
Quantile residual plot of each SNP marker between females and males
Global: Sex is genetically (SILICO) assigned; tau = 0.03
Proportion of heterozygosity in males
1.00
0.75
0.50
0.25
0.00
-0.2
0.0
0.2
0.4
0.6
Quantile residuals for M~F (<- X/Z ->)

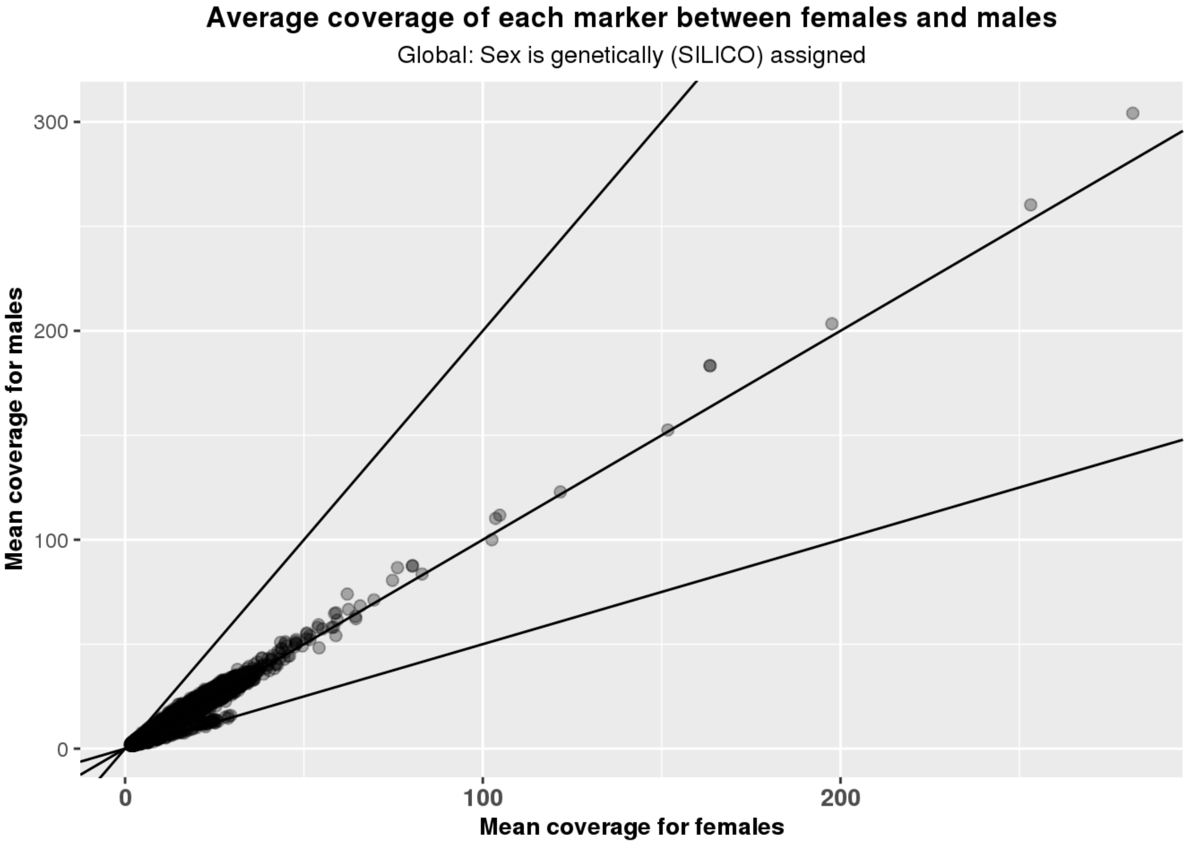
Average coverage of each marker between females and males
Global: Sex is genetically (SILICO) assigned
Mean coverage for males
300
200
100
0
100
200
Mean coverage for females

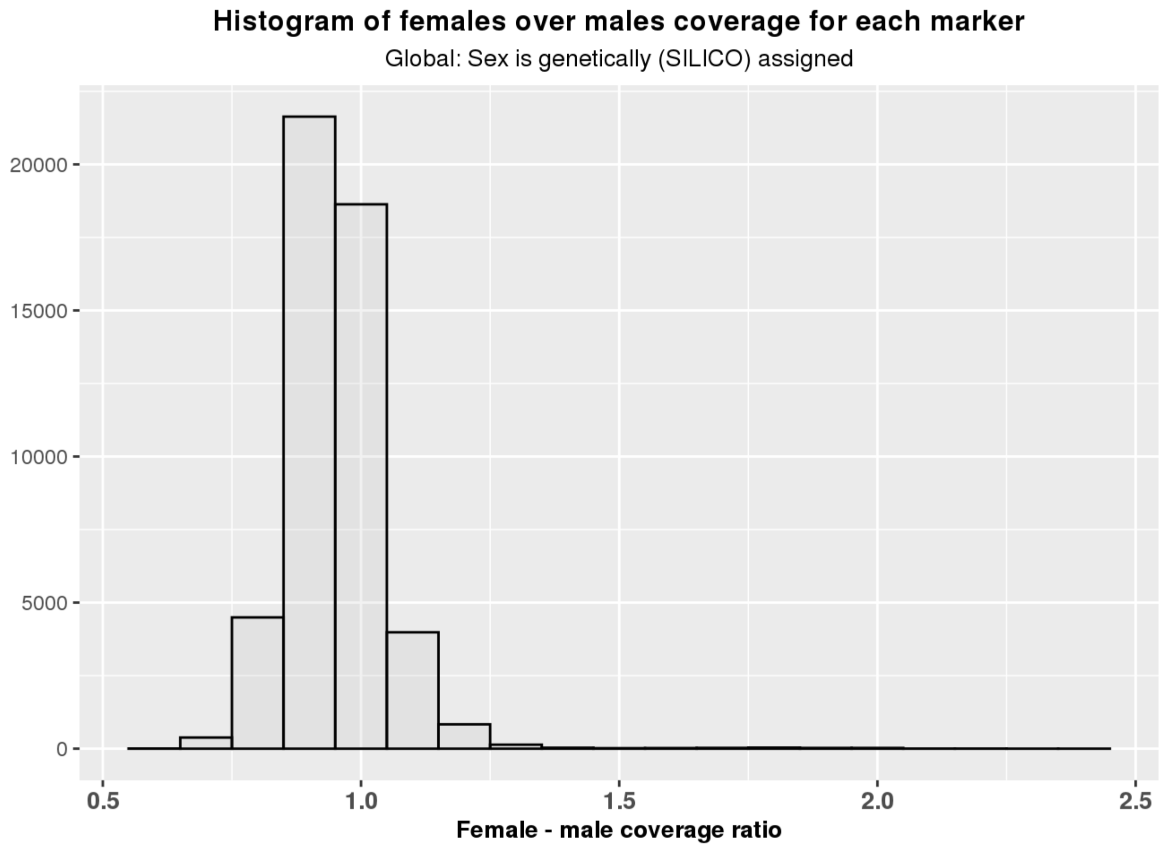
Histogram of females over males coverage for each marker
Global: Sex is genetically (SILICO) assigned
20000
15000
10000
5000
0
0.5
1.0
1.5
2.0
2.5
Female - male coverage ratio

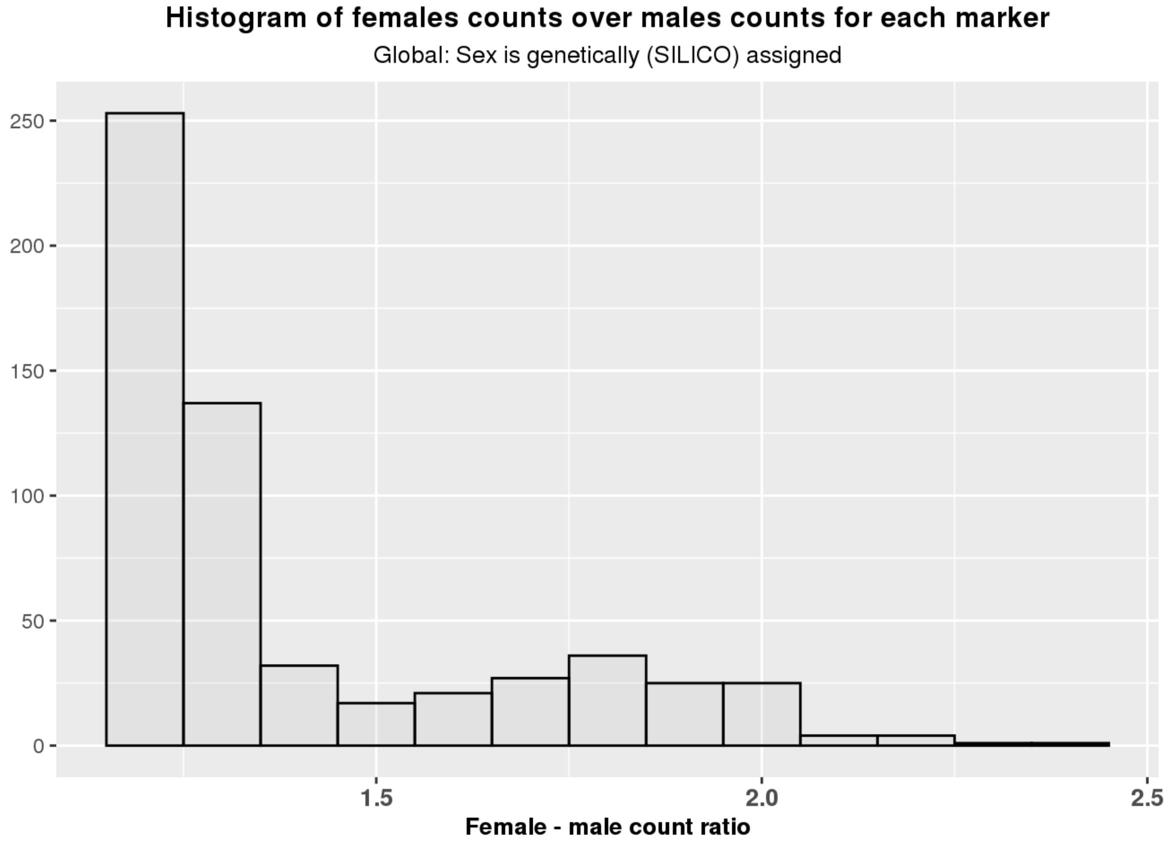
Histogram of females counts over males counts for each marker
Global: Sex is genetically (SILICO) assigned
250
200
150
100
50
0
1.5
2.0
2.5
Female - male count ratio

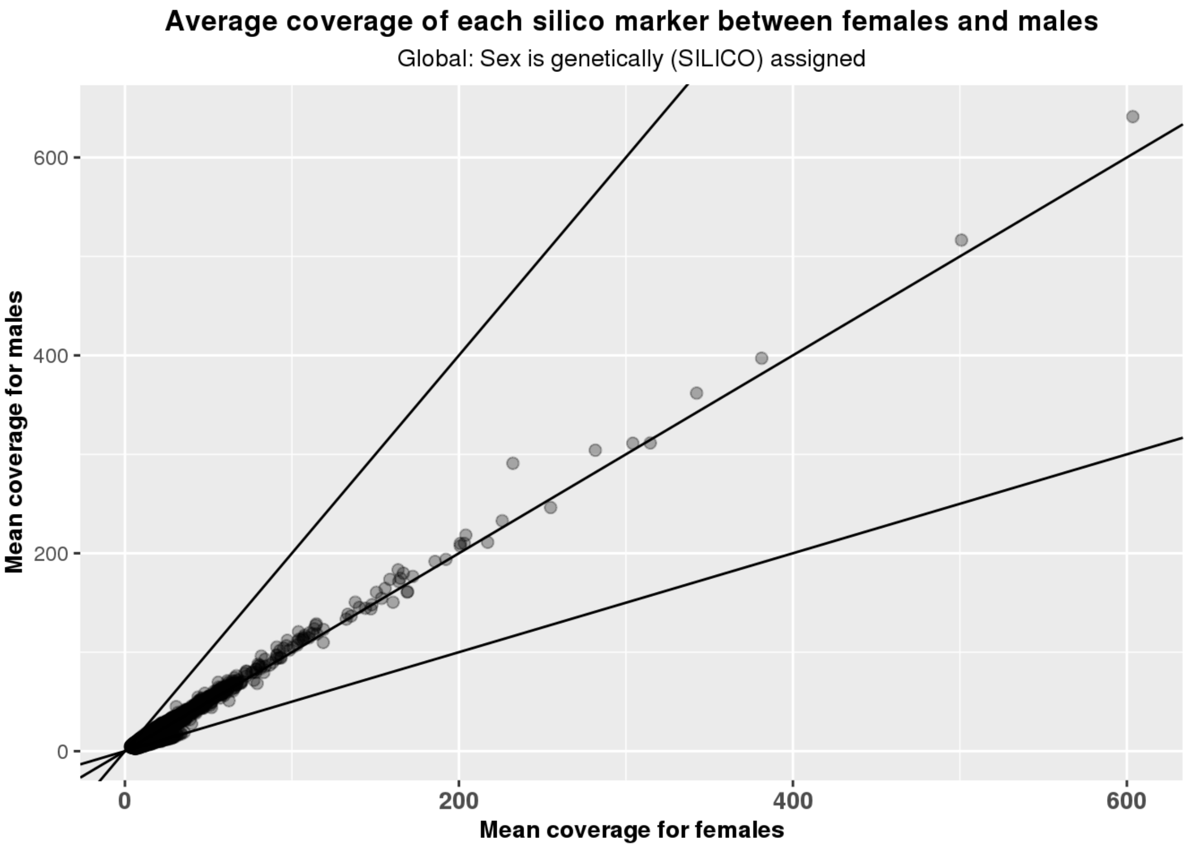
Average coverage of each silico marker between females and males
Global: Sex is genetically (SILICO) assigned
Mean coverage for males
0
200
400
600
Mean coverage for females

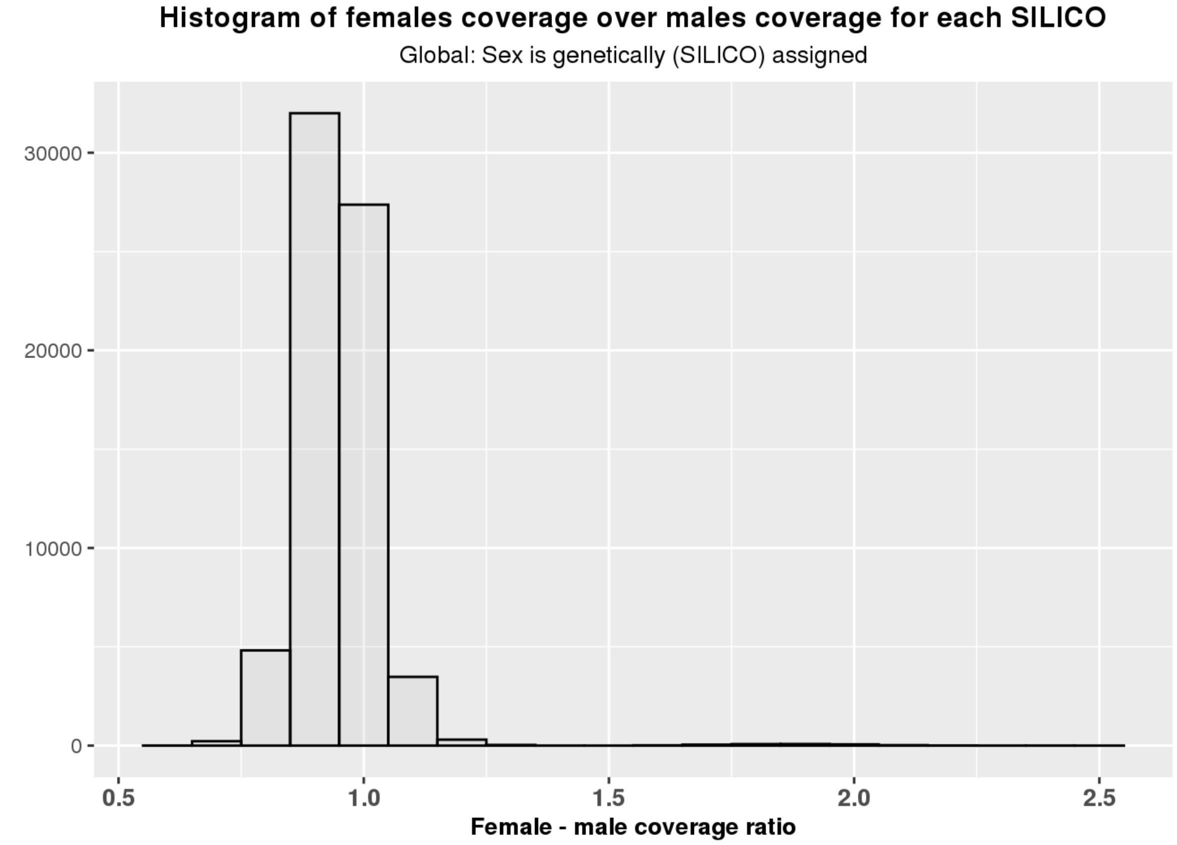
Histogram of females coverage over males coverage for each SILICO
Global: Sex is genetically (SILICO) assigned
0
10000
20000
30000
0.5
1.0
1.5
2.0
2.5
Female - male coverage ratio

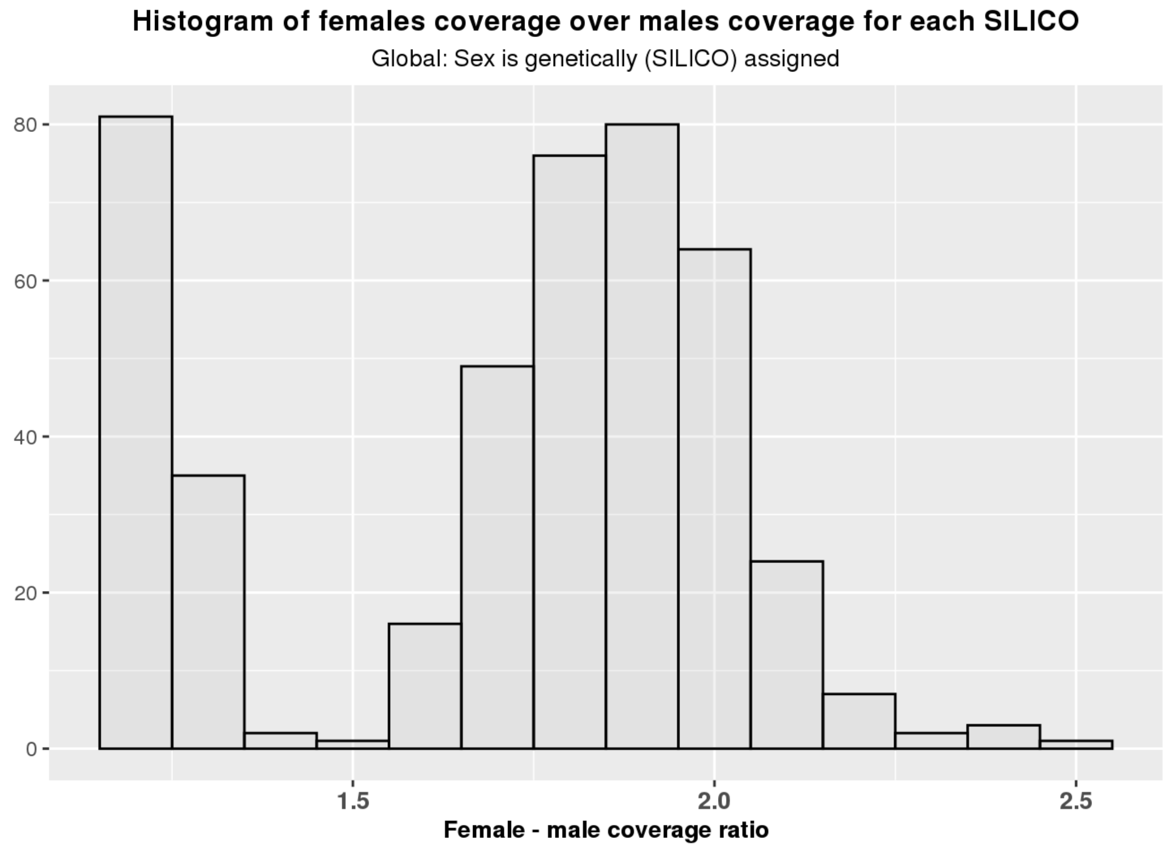


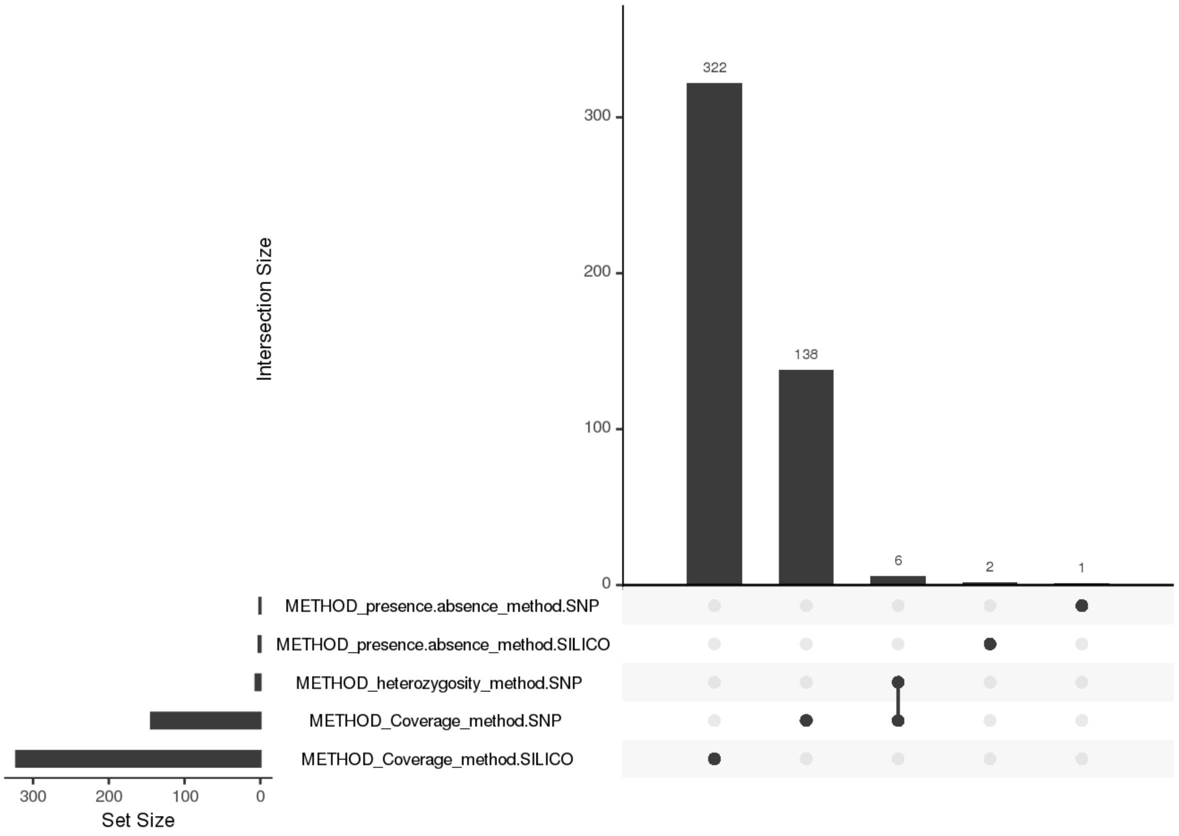


## 4.3 Filter data

First we filter all data with a soft filter (SF) to allow for species identification.

1. Marker Reproducibility: 0.98
2. Individual missingness: n
3. Individual Het - low: n
4. Individual Het - high: n
5. Individual total coverage - low: 7,000
6. Individual total coverage - high: 10,000,000
7. Marker MAC - low: 14
8. Marker coverage - low: 5
9. Marker coverage - high:39
10. Marker maximum missing proportion: 0.1
11. Marker snp position on locus: n
12. Max 3 SNPs per locus
13. 1 SNP per locus according to mac
14. no LD filter
15. Individual Het - low: n
16. Individual Het - high: n
17. Duplicated individuals: n
18. no filter on HWE

```
dartseq.data <- "DArTseq_SNP_OrderAppendix_2_DCarcha19-4454.csv"
strata <- "Bull_shark_DArTseq_strata.tsv"
BSsnp <- radiator::filter_rad(dartseq.data, strata,
                              parallel.core = 1)
BS.dartseq.SF.gds <- BSsnp$gds
BS.dartseq.SF.tidy <- BSsnp$output$tidy.data
print(BS.dartseq.SF.gds, expand=TRUE, all=TRUE, attribute=TRUE,
      attribute.trim=FALSE)
save(BS.dartseq.SF.tidy, file = "DArTseq_ALL_SF_objects.Rdata")
```

Table 1:

| FILTERS | VALUES | BEFORE | BLACKLIST |
|---|---|---|---|
| Filter DArT reproducibility | 0.98 | 219 / 18 / 1 / 168810 / 250945 | 0 / 0 / 0 / 9754 / 19242 |
| Filter monomorphic markers | NA | 219 / 18 / 1 / 159056 / 231703 | 0 / 0 / 0 / 0 / 0 |
| Filter markers in common | NA | 219 / 18 / 1 / 159056 / 231703 | 0 / 0 / 0 / 70113 / 110685 |
| Filter individuals based on total coverage | 7000 1e+07 | 219 / 18 / 1 / 88943 / 121018 | 1 / 0 / 0 / 0 / 0 |
| Filter monomorphic markers | NA | 218 / 18 / 1 / 88943 / 121018 | 0 / 0 / 0 / 5 / 11 |
| Filter MAC | 14 | 218 / 18 / 1 / 88938 / 121007 | 0 / 0 / 0 / 34884 / 61921 |
| Filter coverage min / max | 5-39 | 218 / 18 / 1 / 54054 / 59086 | 0 / 0 / 0 / 14506 / 14976 |
| Filter genotyping | 0.1 | 218 / 18 / 1 / 39548 / 44110 | 0 / 0 / 0 / 11105 / 13720 |
| Filter SNPs position on the read | all | 218 / 18 / 1 / 28443 / 30390 | 0 / 0 / 0 / 0 / 0 |
| Filter markers snp number | 3 | 218 / 18 / 1 / 28443 / 30390 | 0 / 0 / 0 / 25 / 123 |
| Filter short ld | mac | 218 / 18 / 1 / 28418 / 30267 | 0 / 0 / 0 / 0 / 1849 |
| detect mixed genomes | 0 1 | 218 / 18 / 1 / 28418 / 28418 | 0 / 0 / 0 / 0 / 0 |
| detect duplicate genomes | 0 | 218 / 18 / 1 / 28418 / 28418 | 0 / 0 / 0 / 0 / 0 |
| Filter HWE | NA | 218 / 18 / 1 / 28418 / 28418 | 0 / 0 / 0 / 0 / 0 |

## 4.4 Save data

```
BSconvert <- radiator::genomic_converter(BS.dartseq.SF.gds,output = "genlight")
BS.dartseq.SF.gl <- BSconvert$genlight


markers.meta <- radiator::extract_markers_metadata(BS.dartseq.SF.gds, whitelist = TRUE)
individuals.meta <- radiator::extract_individuals_metadata(BS.dartseq.SF.gds, whitelist = TRUE)
BS.dartseq.SF.gl$other$ind.metrics <- individuals.meta
BS.dartseq.SF.gl$other$loc.metrics <- markers.meta

pop.levels <- c("Costa Rica" , "Brazil","Caribbean Sea","Gulf of Mexico",
                "Western North Atlantic", "Sierra Leone", "South Africa",
                "Mozambique", "Reunion" , "Seychelles", "Arabian Sea",
                "Sri Lanka", "Thailand", "Indonesia", "Papua New Guinea" ,
                "King Sound","Fitzroy River", "Durack River", "Daly River",
```

```
                "Adelaide River",   "South Alligator River", "East Alligator River",
                "Blue mud Bay", "Roper River","Towns River", "Skardon River",
                "Wenlock River","Trinity inlet","Clarence River", "Sydney Harbour",
                "Japan", "Fiji")
BS.dartseq.SF.gl$pop <- factor(BS.dartseq.SF.gl$other$ind.metrics$pop,
                               levels = pop.levels)

save(BS.dartseq.SF.tidy, BS.dartseq.SF.gl, file = "DArTseq_ALL_SF_objects.Rdata")
```

## 4.5 Load data

```
load("DArTseq_ALL_SF_objects.Rdata")
pop.levels <- c("Costa Rica" , "Brazil","Caribbean Sea","Gulf of Mexico",
                "Western North Atlantic", "Sierra Leone", "South Africa",
                "Mozambique", "Reunion" , "Seychelles", "Arabian Sea", "Sri Lanka",
                "Thailand", "Indonesia", "Papua New Guinea" , "Australia",
                "Japan", "Fiji")
BS.dartseq.SF.gl$pop <- factor(BS.dartseq.SF.gl$other$ind.metrics$Site)
BS.dartseq.SF.gl <- BS.dartseq.SF.gl[order(BS.dartseq.SF.gl$pop,
                                           BS.dartseq.SF.gl$ind.names)]

adegenet::nInd(BS.dartseq.SF.gl) # 218
adegenet::nLoc(BS.dartseq.SF.gl) # 28418
summary(BS.dartseq.SF.gl$pop)
sum(duplicated(stringr::str_match(pattern = "-\\s*(.*?)\\s*-",
                                  string =  BS.dartseq.SF.gl$ind.names)[,2])) # 32 replicate left
```

```
## [1] 218
## [1] 28418
##            Arabian Sea              Australia                 Brazil
##                     13                     62                     13
##          Caribbean Sea             Costa Rica                   Fiji
##                      2                     10                     11
##         Gulf of Mexico              Indonesia                  Japan
##                     13                      6                     14
##             Mozambique       Papua New Guinea                Reunion
##                      9                      6                     11
##             Seychelles           Sierra Leone           South Africa
##                     10                      2                     10
##              Sri Lanka               Thailand Western North Atlantic
##                     10                      8                      8
## [1] 32
```

## 4.6 Remove sex-linked markers

```
load("Sex_results.Rdata")
sum(BS.dartseq.SF.gl$loc.names %in% bull_shark$sexy.summary$CLONE_ID ) #84
sex.markers <- bull_shark$sexy.summary$CLONE_ID

locnames <- BS.dartseq.SF.gl$loc.names
BS.dartseq.SF.gl <- BS.dartseq.SF.gl[,!locnames %in% sex.markers]
BS.dartseq.SF.gl$other$loc.metrics <- BS.dartseq.SF.gl$other$loc.metrics[!locnames %in%
                                                                          sex.markers,]

sum(sex.markers %in% unique(BS.dartseq.SF.tidy$LOCUS)) # 84
BS.dartseq.SF.tidy <- BS.dartseq.SF.tidy[!BS.dartseq.SF.tidy$LOCUS %in%
                                           sex.markers,]
```

```
## [1] 84
## [1] 112
```

### 4.7 PCA to identify species

```
pca1 <- adegenet::glPca(BS.dartseq.SF.gl, nf = 4, parallel = TRUE,
                        n.cores = parallel::detectCores() - 1)
save(pca1, file = "DArTseq_SF_speciesID_PCA.Rdata")
```

```
load("DArTseq_SF_speciesID_PCA.Rdata")

BS.pca.scores <- as.data.frame(pca1$scores)
BS.pca.scores$pop <- adegenet::pop(BS.dartseq.SF.gl)
BS.pca.scores$species <- BS.dartseq.SF.gl$other$ind.metrics$SpeciesID_genetic
cols <- adegenet::funky(adegenet::nPop(BS.dartseq.SF.gl))


p <- ggplot2::ggplot(BS.pca.scores, ggplot2::aes(x = PC1, y = PC2, colour = pop,
                                                 group = species)) +
  ggplot2::geom_point(aes(shape = species), size = 2) +
  ggplot2::scale_color_manual(values = cols) +
  ggplot2::scale_shape_manual(values = c(1,2,3,4,5,6,8,9)) +
  ggplot2::geom_hline(yintercept = 0) +
  ggplot2::geom_vline(xintercept = 0) +
  ggplot2::theme(panel.background = ggplot2::element_rect(fill = "white",
                                                          colour = "white"),
                 panel.grid.major = ggplot2::element_line(size = 0.5,
                                                          linetype = 'solid',
                                                          colour = "grey"),
                 panel.grid.minor = ggplot2::element_line(size = 0.1,
                                                          linetype = 'solid',
                                                          colour = "grey"),
                 legend.text = ggplot2::element_text(size = 7),
                 legend.position = "right",
                 panel.border = element_rect(colour = "black",
                                             fill = NA, size = 1.5)) +
  ggplot2::guides(shape = ggplot2::guide_legend(ncol = 1),
                  col = ggplot2::guide_legend(ncol = 2),
                  override.aes = list(size = 0.5))
p
```

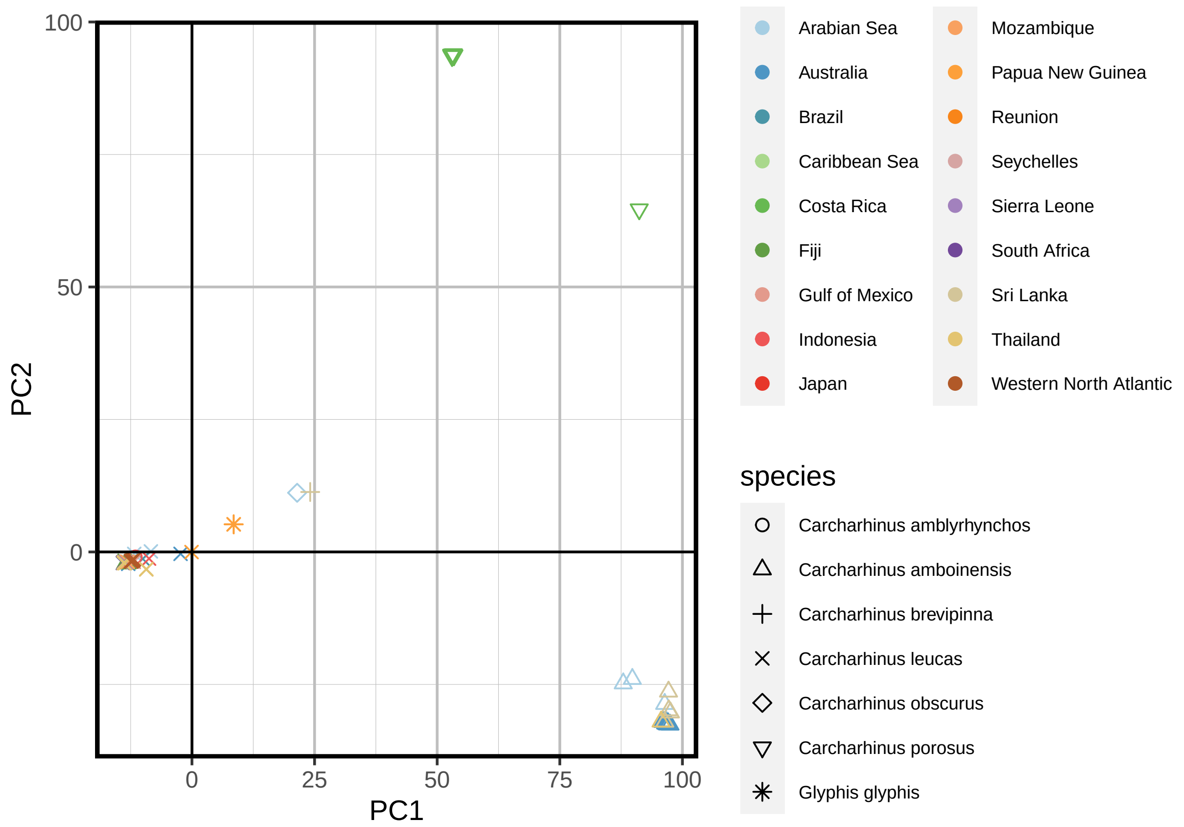


```
ggplot2::ggsave(p, filename = "DArTseq_SF_SpeciesID_PCA1.png",
                width = 30, height = 15,units = "cm")


p <- ggplot2::ggplot(BS.pca.scores, ggplot2::aes(x = PC1, y = PC3, colour = pop,
                                 group = species)) +
  ggplot2::geom_point(aes(shape = species), size = 2) +
  ggplot2::scale_color_manual(values = cols) +
  ggplot2::scale_shape_manual(values = c(1,2,3,4,5,6,8,9)) +
  ggplot2::geom_hline(yintercept = 0) +
  ggplot2::geom_vline(xintercept = 0) +
  ggplot2::theme(panel.background = ggplot2::element_rect(fill = "white",
                                                     colour = "white"),
                 panel.grid.major = ggplot2::element_line(size = 0.5,
                                                     linetype = 'solid',
                                                     colour = "grey"),
                 panel.grid.minor = ggplot2::element_line(size = 0.1,
                                                     linetype = 'solid',
                                                     colour = "grey"),
                 legend.text = ggplot2::element_text(size = 7),
                 legend.position = "right",
                 panel.border = element_rect(colour = "black", fill = NA,
                                          size = 1.5)) +
  ggplot2::guides(shape = ggplot2::guide_legend(ncol = 1),
                  col = ggplot2::guide_legend(ncol = 2),
                  override.aes = list(size = 0.5))
p
```

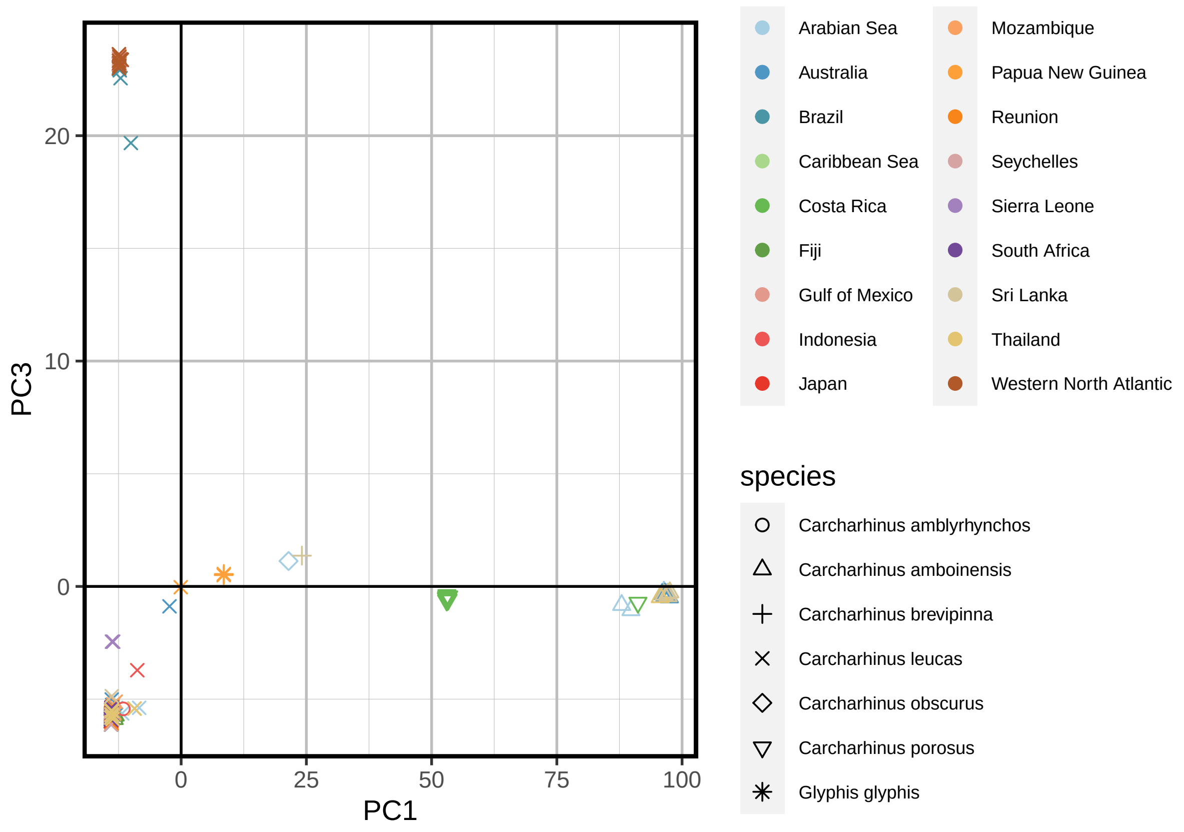


```
ggplot2::ggsave(p, filename = "DArTseq_SF_SpeciesID_PCA2.png", width = 30,
                height = 15,units = "cm")

p <- ggplot2::ggplot(BS.pca.scores, ggplot2::aes(x = PC2, y = PC3, colour = pop,
                                                 group = species)) +
  ggplot2::geom_point(aes(shape = species), size = 2) +
  ggplot2::scale_color_manual(values = cols) +
  ggplot2::scale_shape_manual(values = c(1,2,3,4,5,6,8,9)) +
  ggplot2::geom_hline(yintercept = 0) +
  ggplot2::geom_vline(xintercept = 0) +
  ggplot2::theme(panel.background = ggplot2::element_rect(fill = "white",
                                                          colour = "white"),
                 panel.grid.major = ggplot2::element_line(size = 0.5,
                                                          linetype = 'solid',
                                                          colour = "grey"),
                 panel.grid.minor = ggplot2::element_line(size = 0.1,
                                                          linetype = 'solid',
                                                          colour = "grey"),
                 legend.text = ggplot2::element_text(size = 7),
                 legend.position = "right",
                 panel.border = element_rect(colour = "black",
                                             fill = NA, size = 1.5)) +
  ggplot2::guides(shape = ggplot2::guide_legend(ncol = 1),
                  col = ggplot2::guide_legend(ncol = 2),
                  override.aes = list(size = 0.5))
p
```

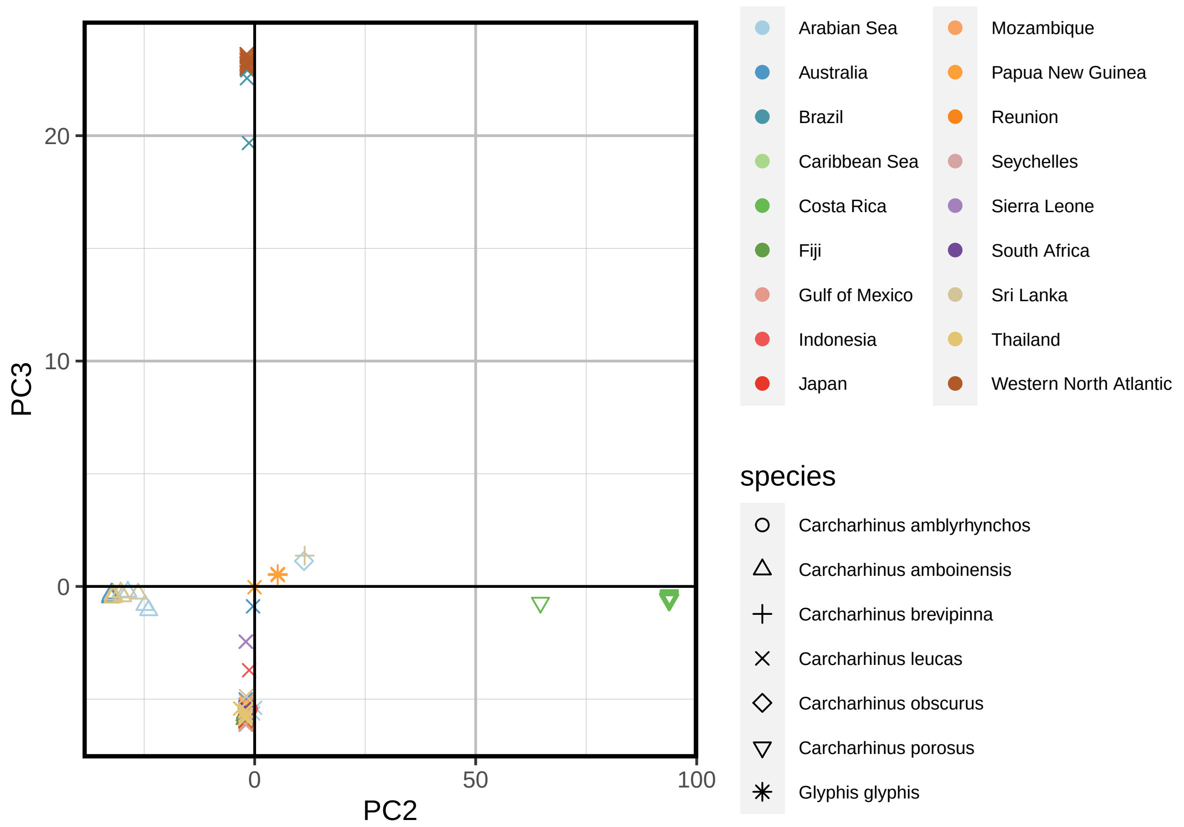


```
ggplot2::ggsave(p, filename = "DArTseq_SF_SpeciesID_PCA3.png",
                width = 30, height = 15,units = "cm")


p <- ggplot2::ggplot(BS.pca.scores, ggplot2::aes(x = PC3, y = PC4, colour = pop,
                                                  group = species)) +
  ggplot2::geom_point(aes(shape = species), size = 2) +
  ggplot2::scale_color_manual(values = cols) +
  ggplot2::scale_shape_manual(values = c(1,2,3,4,5,6,8,9)) +
  ggplot2::geom_hline(yintercept = 0) +
  ggplot2::geom_vline(xintercept = 0) +
  ggplot2::theme(panel.background = ggplot2::element_rect(fill = "white",
                                                          colour = "white"),
                 panel.grid.major = ggplot2::element_line(size = 0.5,
                                                          linetype = 'solid',
                                                          colour = "grey"),
                 panel.grid.minor = ggplot2::element_line(size = 0.1,
                                                          linetype = 'solid',
                                                          colour = "grey"),
                 legend.text = ggplot2::element_text(size = 7),
                 legend.position = "right",
                 panel.border = element_rect(colour = "black",
                                             fill = NA, size = 1.5)) +
  ggplot2::guides(shape = ggplot2::guide_legend(ncol = 1),
                  col = ggplot2::guide_legend(ncol = 2),
                  override.aes = list(size = 0.5))
p
```

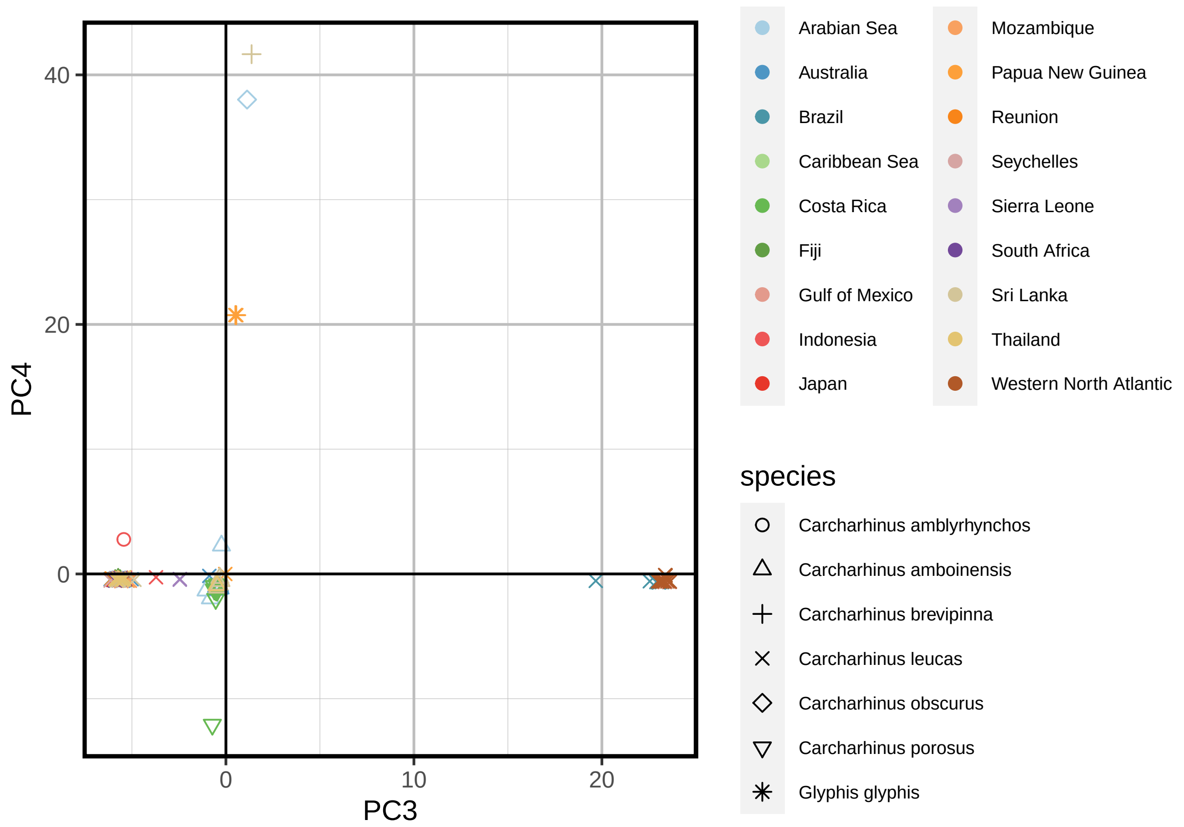


```r
ggplot2::ggsave(p, filename = "DArTseq_SF_SpeciesID_PCA4.png",
                width = 30, height = 15,units = "cm")

p <- ggplot2::ggplot(BS.pca.scores, ggplot2::aes(x = PC2, y = PC4, colour = pop,
                                                 group = species)) +
  ggplot2::geom_point(aes(shape = species), size = 2) +
  ggplot2::scale_color_manual(values = cols) +
  ggplot2::scale_shape_manual(values = c(1,2,3,4,5,6,8,9)) +
  ggplot2::geom_hline(yintercept = 0) +
  ggplot2::geom_vline(xintercept = 0) +
  ggplot2::theme(panel.background = ggplot2::element_rect(fill = "white",
                                                          colour = "white"),
                 panel.grid.major = ggplot2::element_line(size = 0.5,
                                                          linetype = 'solid',
                                                          colour = "grey"),
                 panel.grid.minor = ggplot2::element_line(size = 0.1,
                                                          linetype = 'solid',
                                                          colour = "grey"),
                 legend.text = ggplot2::element_text(size = 7),
                 legend.position = "right",
                 panel.border = element_rect(colour = "black",
                                             fill = NA, size = 1.5)) +
  ggplot2::guides(shape = ggplot2::guide_legend(ncol = 1),
                  col = ggplot2::guide_legend(ncol = 2),
                  override.aes = list(size = 0.5))
p
```

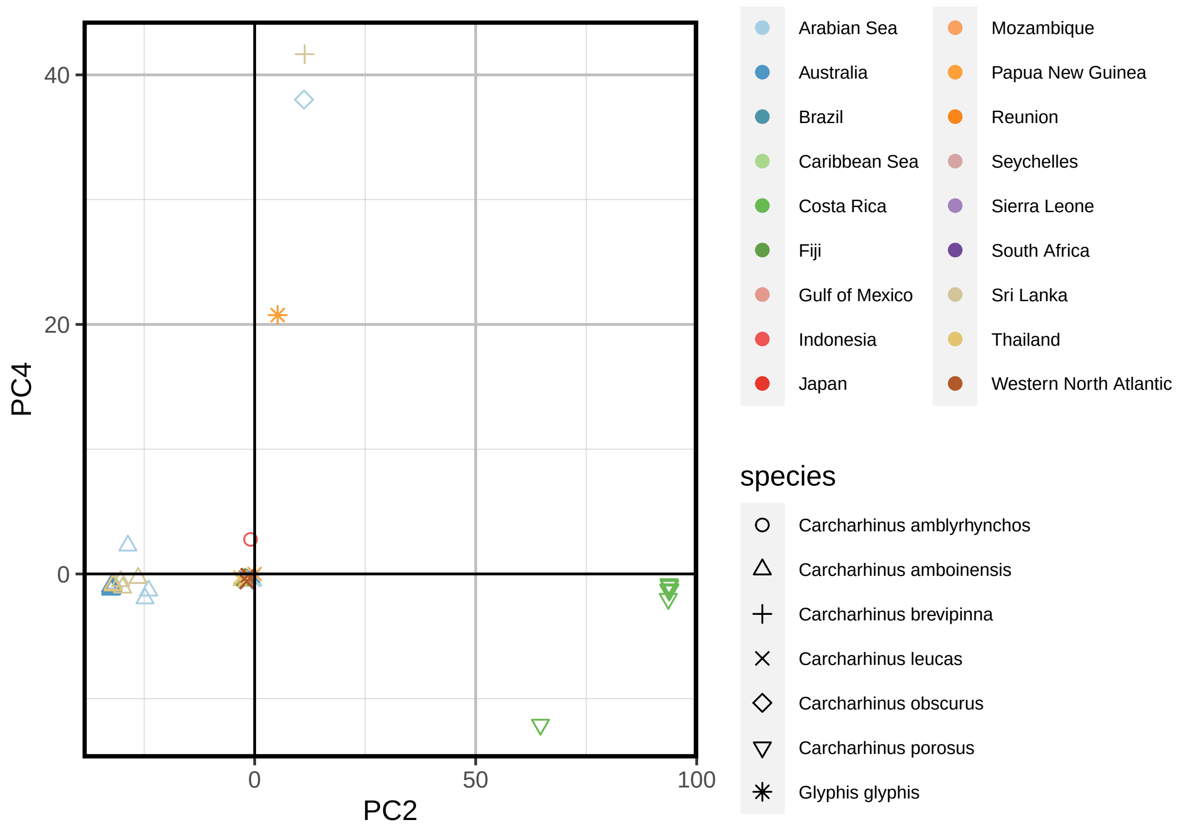


```
ggplot2::ggsave(p, filename = "DArTseq_SF_SpeciesID_PCA4.png",
                width = 30, height = 15,units = "cm")
```

---

# 5 DArTseq - Only Bull Shark

## 5.1 Set strata

```
strata <- radiator::read_strata("Bull_shark_DArTseq_strata.tsv",
                                keep.two = FALSE)$strata %>%
  dplyr:: filter(SpeciesID_genetic == "Carcharhinus leucas")
# readr::write_tsv(strata, path = "Bull_shark_DArTseq_strata2.tsv")
```

## 5.2 Filter data

1. Marker Reproducibility: 0.98
2. Individual missingness: 0.2
3. Individual Het - low: 0
4. Individual Het - high: 0.1
5. Individual total coverage - low: 7,000
6. Individual total coverage - high: 10,000,000
7. Marker MAC - low: 4
8. Marker coverage - low: 5
9. Marker coverage - high:39
10. Marker maximum missing proportion: 0.1
11. Marker snp position on locus: n
12. Max 3 SNPs per locus
13. 1 SNP per locus according to mac
14. no LD filter
15. Individual Het - low: 0
16. Individual Het - high: 0.132
17. Duplicated individuals: 0.1
18. Filter on HWE: 2 pop, 0.01

```
dartseq.data <- "DArTseq_SNP_OrderAppendix_2_DCarcha19-4454.csv"
strata <- "Bull_shark_DArTseq_strata2.tsv"
BSsnp <- radiator::filter_rad(dartseq.data, strata,
                              parallel.core = 1)
BS.dartseq.gds <- BSsnp$gds
BS.dartseq.tidy <- BSsnp$output$tidy.data
print(BS.dartseq.gds, expand=TRUE, all=TRUE, attribute=TRUE,
      attribute.trim=FALSE)
save(BS.dartseq.tidy, file = "DArTseq_ALL_objects2.Rdata")
```

Table 2:

| FILTERS | VALUES | BEFORE | BLACKLIST |
|---|---|---|---|
| Filter DArT reproducibility | 0.98 | 182 / 29 / 1 / 168810 / 250945 | 0 / 0 / 0 / 9754 / 19242 |
| Filter monomorphic markers | NA | 182 / 29 / 1 / 159056 / 231703 | 0 / 0 / 0 / 97626 / 156667 |
| Filter markers in common | NA | 182 / 29 / 1 / 61430 / 75036 | 0 / 0 / 0 / 12699 / 15156 |
| Filter individuals based on missingness | 0.2 | 182 / 29 / 1 / 48731 / 59880 | 4 / 0 / 0 / 0 / 0 |
| Filter individuals based on heterozygosity | 0 0.1 | 178 / 29 / 1 / 48731 / 59880 | 1 / 0 / 0 / 0 / 0 |
| Filter individuals based on total coverage | 7000 1e+07 | 177 / 29 / 1 / 48731 / 59880 | 0 / 0 / 0 / 0 / 0 |
| Filter monomorphic markers | NA | 177 / 29 / 1 / 48731 / 59880 | 0 / 0 / 0 / 2510 / 3417 |
| Filter MAC | 4 | 177 / 29 / 1 / 46221 / 56463 | 0 / 0 / 0 / 25024 / 33343 |
| Filter coverage min / max | 5-39 | 177 / 29 / 1 / 21197 / 23120 | 0 / 0 / 0 / 2311 / 2382 |
| Filter genotyping | 0.1 | 177 / 29 / 1 / 18886 / 20738 | 0 / 0 / 0 / 266 / 393 |
| Filter SNPs position on the read | all | 177 / 29 / 1 / 18620 / 20345 | 0 / 0 / 0 / 0 / 0 |
| Filter markers snp number | 1 | 177 / 29 / 1 / 18620 / 20345 | 0 / 0 / 0 / 1590 / 3315 |
| Filter short ld | not filtering | 177 / 29 / 1 / 17030 / 17030 | 0 / 0 / 0 / 0 / 0 |
| detect mixed genomes | 0 0.132 | 177 / 29 / 1 / 17030 / 17030 | 5 / 0 / 0 / 0 / 0 |
| Filter monomorphic markers | NA | 172 / 29 / 1 / 17030 / 17030 | 0 / 0 / 0 / 0 / 0 |
| detect duplicate genomes | 0.1 | 172 / 29 / 1 / 17030 / 17030 | 23 / 0 / 0 / 0 / 0 |
| Filter monomorphic markers | NA | 149 / 29 / 1 / 17030 / 17030 | 0 / 0 / 0 / 0 / 0 |
| Filter HWE | 20.01 | 149 / 29 / 1 / 17030 / 17030 | 0 / 0 / 0 / 1 / 1 |

## 5.3 Save data

```r
BSconvert <- radiator::genomic_converter(BS.dartseq.gds,
                                         output = c("genlight", "stockr",
                                                    "pcadapt", "gtypes"))

BS.dartseq.gl <- BSconvert$genlight
BS.dartseq.stkr <- BSconvert$stockr
BS.dartseq.pc <- BSconvert$pcadapt
BS.dartseq.gt <- BSconvert$gtypes

markers.meta <- radiator::extract_markers_metadata(BS.dartseq.gds, whitelist = TRUE)
individuals.meta <- radiator::extract_individuals_metadata(BS.dartseq.gds,
                                                           whitelist = TRUE)
BS.dartseq.gl$other$ind.metrics <- individuals.meta
BS.dartseq.gl$other$loc.metrics <- markers.meta
BS.dartseq.gt@schemes <- individuals.meta

pop.levels <- c("Brazil","Caribbean Sea","Gulf of Mexico",  "Western North Atlantic",
                "Sierra Leone", "South Africa", "Mozambique", "Reunion" ,
                "Seychelles", "Arabian Sea", "Sri Lanka", "Thailand", "Indonesia",
                "Papua New Guinea" , "Fitzroy River", "Daly River", "Adelaide River",
                "South Alligator River", "East Alligator River","Blue mud Bay",
                "Roper River","Towns River","Wenlock River","Trinity inlet","Clarence River",
                "Sydney Harbour","Okinawa", "Urauchi River", "Fiji")
BS.dartseq.gl$pop <- factor(stringr::str_replace_all(
  string = BS.dartseq.gl$other$ind.metrics$STRATA,
  pattern = "_", replacement = " "))
BS.dartseq.gl$pop <- factor(BS.dartseq.gl$pop, levels = pop.levels)


BS.dartseq.gl <- BS.dartseq.gl[order(BS.dartseq.gl$pop, BS.dartseq.gl$ind.names),]

pop.levels1 <- c("Brazil","Caribbean Sea","Gulf of Mexico",  "Western North Atlantic",
                 "Sierra Leone", "South Africa", "Mozambique", "Reunion" , "Seychelles",
                 "Arabian Sea", "Sri Lanka", "Thailand", "Indonesia", "Papua New Guinea" ,
                 "Australia", "Japan", "Fiji")
BS.dartseq.tidy$POP_ID <- factor(BS.dartseq.tidy$Site, levels = pop.levels1)
BS.dartseq.tidy <- BS.dartseq.tidy[order(BS.dartseq.tidy$POP_ID,
                                         BS.dartseq.tidy$INDIVIDUALS),]

save(BS.dartseq.tidy, BS.dartseq.gl, BS.dartseq.stkr, BS.dartseq.pc,BS.dartseq.gt,
     markers.meta, individuals.meta, file = "DArTseq_ALL_objects2.Rdata")
# save(BS.dartseq.gl, BS.dartseq.stkr, BS.dartseq.pc,BS.dartseq.gt,
# markers.meta, individuals.meta, file = "DArTseq_ALL_objects3.Rdata")
# save(BS.dartseq.tidy,
#      file = "DArTseq_ALL_objects4.Rdata")
# object.size(BS.dartseq.tidy)  #2,033,649,160 bytes
```

### 5.4 Load data

```r
# load("DArTseq_ALL_objects2.Rdata")
load("DArTseq_ALL_objects3.Rdata")
adegenet::nInd(BS.dartseq.gl) # 149
adegenet::nLoc(BS.dartseq.gl) # 16989
summary(BS.dartseq.gl$pop)
sum(duplicated(stringr::str_match(pattern = "-\\s*(.*?)\\s*-",
                                  string =  BS.dartseq.gl$ind.names)[,2])) # 0 replicate left
```

```
## [1] 149
## [1] 16989
##                 Brazil           Caribbean Sea          Gulf of Mexico
##                     11                       2                      11
## Western North Atlantic           Sierra Leone           South Africa
##                      8                       1                       9
##             Mozambique                 Reunion             Seychelles
##                      8                       9                      10
##            Arabian Sea               Sri Lanka               Thailand
##                      6                       4                       6
##              Indonesia        Papua New Guinea              Australia
##                      4                       3                      37
##                  Japan                    Fiji
##                     12                       8
## [1] 0
```

## 5.5 Remove sex-linked markers

```
load("Sex_results.Rdata")
sum(BS.dartseq.gl$loc.names %in% bull_shark$sexy.summary$CLONE_ID ) #24
sex.markers <- bull_shark$sexy.summary$CLONE_ID

locnames <- BS.dartseq.gl$loc.names
BS.dartseq.gl <- BS.dartseq.gl[,!locnames %in% bull_shark$sexy.summary$CLONE_ID]
BS.dartseq.gl$other$loc.metrics <-
  BS.dartseq.gl$other$loc.metrics[!locnames %in%
                                    bull_shark$sexy.summary$CLONE_ID,]

sum(sex.markers %in% unique(BS.dartseq.tidy$LOCUS)) # 24
BS.dartseq.tidy <- BS.dartseq.tidy[!BS.dartseq.tidy$LOCUS %in% sex.markers,]

locus.gt <- stringr::str_match(pattern = "__\\s*(.*?)\\s*__",
                               string =  BS.dartseq.gt@data$locus)[,2]
BS.dartseq.gt <- BS.dartseq.gt[,!locus.gt %in% sex.markers, , drop = TRUE]
```

## 5.6 Genetic diversity

```
set.seed(124)
gl <- BS.dartseq.gl
pop.levels <- c("Brazil","Caribbean Sea","Gulf of Mexico",  "Western North Atlantic",
                "South Africa", "Mozambique", "Reunion" , "Seychelles", "Arabian Sea",
                "Sri Lanka", "Thailand", "Indonesia", "Papua New Guinea" , "Australia",
                "Japan", "Fiji")
gl <- gl[gl$pop != "Sierra Leone"] #only 1 sample
gl$pop <- factor(gl$other$ind.metrics$Site, levels = pop.levels)
gl <- gl[order(gl$pop, gl$ind.names)]

gl2gpop(gl, filename = "BS_dartseq_all_genepop2.txt")
bastat <- diveRsity::basicStats(infile = "BS_dartseq_all_genepop2.txt",
                                 outfile = "BS_dartseq_all_BasicStat_output.txt",
                                fis_ci = TRUE,ar_ci = TRUE, fis_boots = 1000,
                                ar_boots = 1000,mc_reps = 1000,
                                rarefaction = FALSE, ar_alpha = 0.05, fis_alpha = 0.05)
length(bastat$main_tab) #16
gendiv <- data.frame(
  "Brazil" = bastat$main_tab[[1]]$overall,
  "Caribbean Sea" = bastat$main_tab[[2]]$overall,
```

```r
  "Gulf of Mexico" = bastat$main_tab[[3]]$overall,
  "Western North Atlantic" = bastat$main_tab[[4]]$overall,
  "South Africa" = bastat$main_tab[[5]]$overall,
  "Mozambique" = bastat$main_tab[[6]]$overall,
  "Reunion" = bastat$main_tab[[7]]$overall,
  "Seychelles" = bastat$main_tab[[8]]$overall,
  "Arabian Sea" = bastat$main_tab[[9]]$overall,
  "Sri Lanka" = bastat$main_tab[[10]]$overall,
  "Thailand" = bastat$main_tab[[11]]$overall,
  "Indonesia" = bastat$main_tab[[12]]$overall,
  "Papua New Guinea" = bastat$main_tab[[13]]$overall,
  "Australia" = bastat$main_tab[[14]]$overall,
  "Japan" = bastat$main_tab[[15]]$overall,
  "Fiji" = bastat$main_tab[[16]]$overall,
  check.names = FALSE)
row.names(gendiv) <- rownames(bastat$main_tab[[1]])

df <- as.data.frame(gl)
df[df == "0"] <- "11"
df[df == "1"] <- "12"
df[df == "2"] <- "22"
gen.gi <- adegenet::df2genind(df, ploidy = 2,ncode = 2, pop = gl$pop)
ris_basic <- hierfstat::basic.stats(gen.gi)
fis.hierfstat <- apply(ris_basic$Fis, 2, mean, na.rm=T)
fis.CI <- hierfstat::boot.ppfis(gen.gi, nboot = 1000, quant = c(0.025,0.975),
                                diploid = TRUE,dig = 4)
df <- data.frame(fis.hierfstat,fis.CI.low = fis.CI$fis.ci[,1],
                 fis.CI.high = fis.CI$fis.ci[,2])
df <- as.data.frame(t(df))
gendiv <- rbind(gendiv, df)

readr::write_tsv(gendiv, file = "DArTseq_ALL_gendiv2.txt")
save(gendiv, file = "DArTseq_ALL_gendiv2.Rdata")
```

```r
load("DArTseq_ALL_gendiv2.Rdata")
shortnames <- c("BRZ", "CAR", "GOM", "WNA", "SAF", "MOZ", "RUN" , "SEY",
                "ARS", "SRL", "TAI", "IND", "PNG" , "AUS", "JAP", "FIJ")
knitr::kable(gendiv, col.names = shortnames,caption = "", digits = 4) %>%
  kableExtra::kable_styling(full_width = FALSE,font_size = 5,
                            latex_options = c( "hold_position")) %>%
  kableExtra::landscape()
```

Table 3:

| | BRZ | CAR | GOM | WNA | SAF | MOZ | RUN | SEY | ARS | SRL | TAI | IND | PNG | AUS | JAP | FIJ |
|---|---|---|---|---|---|---|---|---|---|---|---|---|---|---|---|---|
| ar | 1.1500 | 1.1150 | 1.1440 | 1.1430 | 1.1890 | 1.1810 | 1.1880 | 1.1910 | 1.1830 | 1.1810 | 1.1890 | 1.1660 | 1.1750 | 1.1970 | 1.1880 | 1.1810 |
| size | 10.9420 | 1.9890 | 10.9430 | 7.9550 | 8.9680 | 7.8580 | 8.9650 | 9.9550 | 5.9610 | 3.9880 | 5.9790 | 3.9840 | 2.9920 | 36.8550 | 11.9630 | 7.9390 |
| obs_het | 0.0840 | 0.0800 | 0.0810 | 0.0820 | 0.1060 | 0.1020 | 0.1050 | 0.1070 | 0.1050 | 0.1060 | 0.1120 | 0.1060 | 0.1160 | 0.1050 | 0.1080 | 0.1020 |
| exp_het | 0.0840 | 0.0620 | 0.0810 | 0.0800 | 0.1050 | 0.1040 | 0.1040 | 0.1050 | 0.1010 | 0.0960 | 0.1010 | 0.0890 | 0.0930 | 0.1090 | 0.1010 | 0.1010 |
| uexp_het | 0.0880 | 0.0830 | 0.0840 | 0.0850 | 0.1110 | 0.1110 | 0.1100 | 0.1110 | 0.1110 | 0.1100 | 0.1100 | 0.1020 | 0.1120 | 0.1100 | 0.1050 | 0.1080 |
| fis | -0.0120 | -0.2960 | -0.0160 | -0.0340 | -0.0230 | -0.0030 | -0.0290 | -0.0240 | -0.0460 | -0.1140 | -0.1000 | -0.1840 | -0.2310 | 0.0270 | -0.0600 | -0.0220 |
| hwe_glb | 1.0000 | 1.0000 | 1.0000 | 1.0000 | 1.0000 | 1.0000 | 1.0000 | 1.0000 | 1.0000 | 1.0000 | 1.0000 | 1.0000 | 1.0000 | 1.0000 | 1.0000 | 1.0000 |
| hwe_hom | 1.0000 | 1.0000 | 1.0000 | 1.0000 | 1.0000 | 1.0000 | 1.0000 | 1.0000 | 1.0000 | 1.0000 | 1.0000 | 1.0000 | 1.0000 | 1.0000 | 1.0000 | 1.0000 |
| hwe_het | 1.0000 | 1.0000 | 1.0000 | 1.0000 | 1.0000 | 1.0000 | 1.0000 | 1.0000 | 1.0000 | 1.0000 | 1.0000 | 1.0000 | 1.0000 | 1.0000 | 1.0000 | 1.0000 |
| fis_lo | -0.0870 | -1.0000 | -0.0910 | -0.1340 | -0.1220 | -0.1180 | -0.1250 | -0.1080 | -0.2000 | -0.3520 | -0.3050 | -0.5580 | -1.0000 | 0.0080 | -0.1390 | -0.1480 |
| fis_hi | -0.0240 | -0.2960 | -0.0280 | -0.0460 | -0.0350 | -0.0120 | -0.0400 | -0.0360 | -0.0590 | -0.1140 | -0.1080 | -0.1840 | -0.2310 | 0.0200 | -0.0670 | -0.0350 |
| ar_lo | 1.0770 | 1.0710 | 1.0750 | 1.0740 | 1.1000 | 1.0680 | 1.0990 | 1.1020 | 1.0990 | 1.0970 | 1.1000 | 1.0970 | 1.1020 | 1.1070 | 1.1010 | 1.0900 |
| ar_hi | 1.1610 | 1.1530 | 1.1560 | 1.1590 | 1.2060 | 1.2060 | 1.2050 | 1.2070 | 1.2070 | 1.2050 | 1.2190 | 1.2040 | 1.2150 | 1.2130 | 1.2100 | 1.2020 |
| fis.hierfstat | 0.0326 | -0.0480 | 0.0283 | 0.0277 | 0.0312 | 0.0576 | 0.0261 | 0.0246 | 0.0360 | 0.0111 | -0.0176 | -0.0627 | -0.0662 | 0.0399 | -0.0190 | 0.0388 |
| fis.CI.low | 0.0359 | 0.0230 | 0.0293 | 0.0315 | 0.0379 | 0.0755 | 0.0340 | 0.0270 | 0.0468 | 0.0264 | -0.0295 | -0.0660 | -0.0571 | 0.0432 | -0.0378 | 0.0523 |
| fis.CI.high | 0.0552 | 0.0754 | 0.0501 | 0.0572 | 0.0571 | 0.0963 | 0.0526 | 0.0450 | 0.0711 | 0.0551 | -0.0072 | -0.0334 | -0.0233 | 0.0529 | -0.0203 | 0.0720 |

### 5.6.1 Number of monomorphic markers per population

```
pop.levels <- c("Brazil","Caribbean Sea","Gulf of Mexico", "Western North Atlantic",
                "Sierra Leone", "South Africa", "Mozambique", "Reunion" , "Seychelles",
                "Arabian Sea", "Sri Lanka", "Thailand", "Indonesia", "Papua New Guinea",
                "Australia", "Japan", "Fiji")
BS.dartseq.gl$pop <- factor(BS.dartseq.gl$other$ind.metrics$Site, levels = pop.levels)
BS.dartseq.gl <- BS.dartseq.gl[order(BS.dartseq.gl$pop, BS.dartseq.gl$ind.names)]

monomorphics <- c()
for (i in 1:length(pop.levels)) {
  x <- BS.dartseq.gl[BS.dartseq.gl$pop == pop.levels[i]]
  x2 <- dartR::gl.filter.monomorphs(x, verbose = 0)
  locs <-  adegenet::nLoc(x) - adegenet::nLoc(x2)
  monomorphics[i] <- locs
}
names(monomorphics) <- pop.levels
shortnames <- c("BRZ", "CAR", "GOM", "WNA","SIL", "SAF", "MOZ", "RUN" , "SEY",
                "ARS", "SRL", "TAI", "IND", "PNG" , "AUS", "JAP", "FIJ")
knitr::kable(t(as.table(monomorphics)), col.names = shortnames, digits = 4,
             caption = "") %>%
  kableExtra::kable_styling(full_width = FALSE,font_size = 5,
                            latex_options = c( "hold_position"))
```

Table 4:

| BRZ | CAR | GOM | WNA | SIL | SAF | MOZ | RUN | SEY | ARS | SRL | TAI | IND | PNG | AUS | JAP | FIJ |
|---|---|---|---|---|---|---|---|---|---|---|---|---|---|---|---|---|
| 9909 | 12609 | 10058 | 10432 | 16440 | 9502 | 9943 | 9712 | 9348 | 10818 | 11947 | 10945 | 12709 | 12884 | 5415 | 9821 | 10483 |

### 5.6.2 Nucleotide diversity and inbreeding

```
pi.sum <- radiator::pi(
  data = BS.dartseq.tidy,
  read.length = 70,
  parallel.core = parallel::detectCores() - 1,
  path.folder = NULL,
  verbose = TRUE
)

FIS.sum<- radiator::ibdg_fh(data = BS.dartseq.tidy,
        path.folder = NULL, verbose = TRUE)

save(pi.sum, FIS.sum, file = "DArTseq_ALL_pi_FIS_Sites2.Rdata")
# boxplot.pi <- pi.sum$boxplot.pi
# fh.box.plot <- FIS.sum$fh.box.plot
# save(boxplot.pi, fh.box.plot, file = "DArTseq_ALL_pi_FIS_Sites3.Rdata")
```

```
load("DArTseq_ALL_pi_FIS_Sites3.Rdata")
boxplot.pi
```

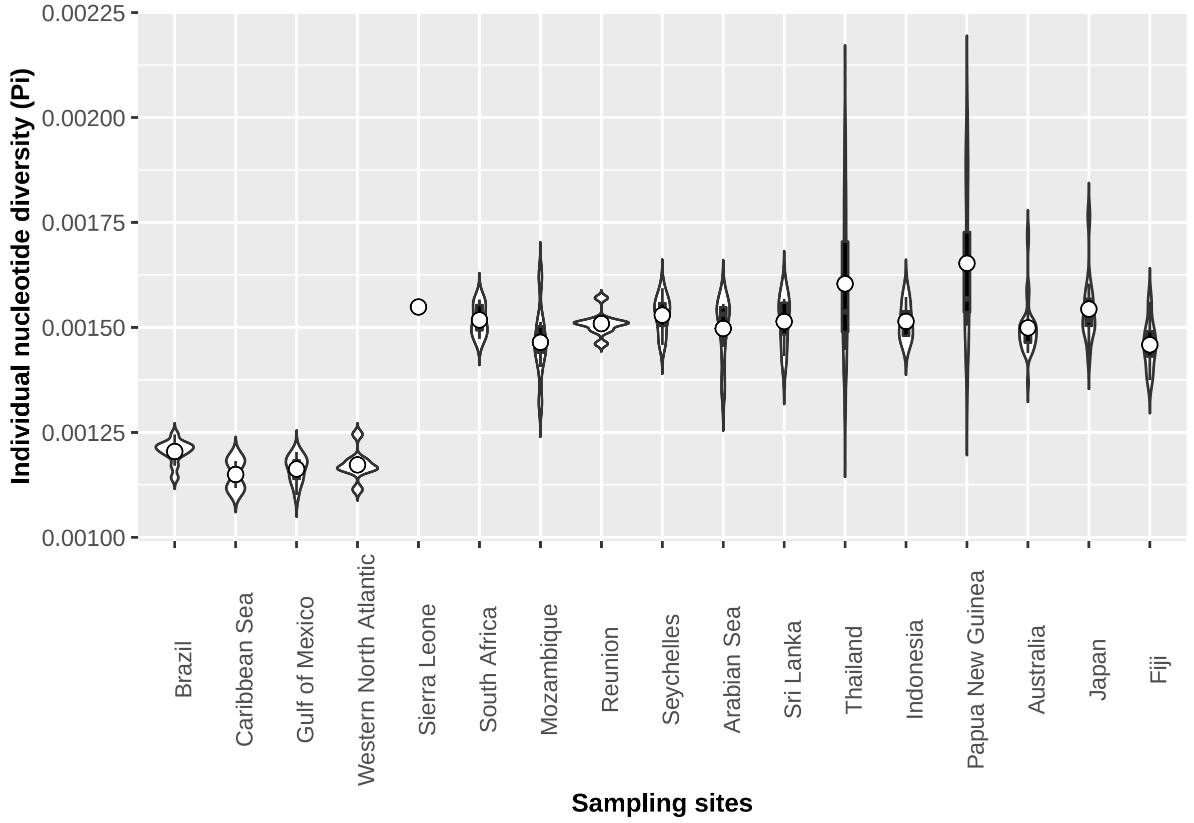


```
fh.box.plot
```

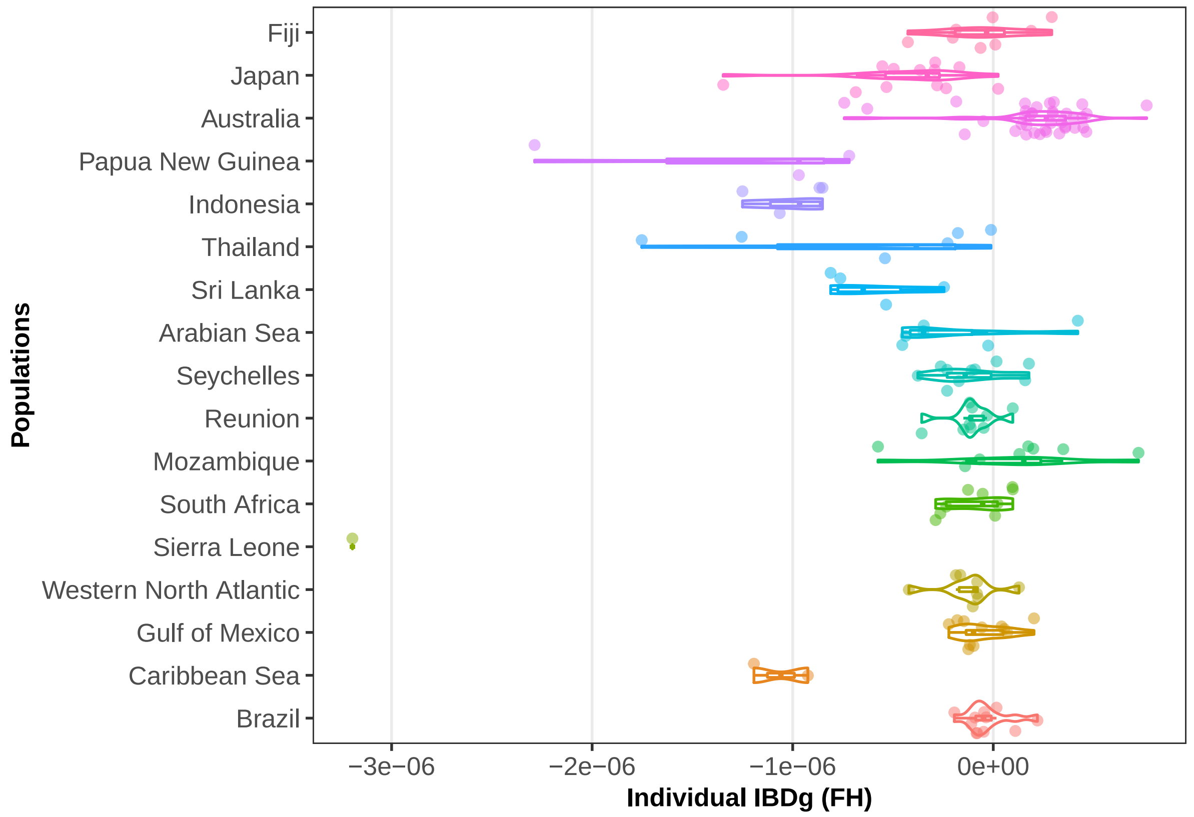


```
# pi.sum$boxplot.pi
# FIS.sum$fh.box.plot
```

## 5.7 Genetic differentiation

```
Fst.gt <- strataG::popStructTest(
    BS.dartseq.gt,
    nrep = 1000,
    stats = "all",
    type = "pairwise",
    keep.null = FALSE,
    quietly = TRUE,
    nb.cores =  parallel::detectCores() - 1,
    write.output = FALSE
  )
save(Fst.gt, file = "DArTseq_ALL_FST.Rdata")
readr::write_tsv(Fst.gt$pairwise$result, path = "DArTseq_ALL_FST.txt")
```

```
load("DArTseq_ALL_FST.Rdata")
kableExtra::kbl(Fst.gt$pairwise$result[order(Fst.gt$pairwise$result$Fst,
                                             decreasing = TRUE),
                                       -c(1:4)], digits = 4,
  caption = "Pairwise Fst for 16989 DArTseq loci between the all sites",
  longtable = TRUE, booktabs = T) %>%
  kableExtra::kable_styling(full_width = FALSE,font_size = 5,
                            latex_options = c("repeat_header", "hold_position")) %>%
  kableExtra::landscape()
```

Table 5: Pairwise Fst for 16989 DArTseq loci between the all sites

| | Chi2 | Chi2.p.val | D | D.p.val | Fis | Fis.p.val | Fst | Fst.p.val | F'st | F'st.p.val | Gst | Gst.p.val | G'st | G'st.p.val | G"st | G"st.p.val |
|---|---|---|---|---|---|---|---|---|---|---|---|---|---|---|---|---|
| Gulf_of_Mexico (11) v. Papua_New_Guinea (2) | 77033.084 | 0.0120 | 0.1109 | 0.0120 | -5.2515 | 1.0000 | 0.6747 | 0.0451 | 0.7753 | 0.0451 | 0.6907 | 0.0622 | 0.8012 | 0.0622 | 0.8824 | 0.0622 |
| Gulf_of_Mexico (11) v. Indonesia (4) | 93596.844 | 0.0010 | 0.1191 | 0.0010 | -2.7267 | 1.0000 | 0.6733 | 0.0011 | 0.7762 | 0.0011 | 0.5738 | 0.0011 | 0.7147 | 0.0011 | 0.8187 | 0.0011 |
| Papua_New_Guinea (2) v. Western_North_Atlantic (8) | 61638.548 | 0.0240 | 0.1104 | 0.0240 | -6.3798 | 1.0000 | 0.6713 | 0.0745 | 0.7803 | 0.0745 | 0.7239 | 0.0811 | 0.8241 | 0.0811 | 0.8980 | 0.0811 |
| Indonesia (4) v. Western_North_Atlantic (8) | 76570.194 | 0.0030 | 0.1185 | 0.0030 | -3.0371 | 1.0000 | 0.6695 | 0.0033 | 0.7780 | 0.0033 | 0.5893 | 0.0033 | 0.7263 | 0.0033 | 0.8278 | 0.0033 |
| Gulf_of_Mexico (11) v. Thailand (4) | 92623.543 | 0.0010 | 0.1141 | 0.0010 | -2.1662 | 1.0000 | 0.6642 | 0.0011 | 0.7678 | 0.0011 | 0.5473 | 0.0011 | 0.6931 | 0.0011 | 0.8016 | 0.0011 |
| Gulf_of_Mexico (11) v. Sri_Lanka (4) | 92859.069 | 0.0020 | 0.1144 | 0.0020 | -2.0099 | 1.0000 | 0.6614 | 0.0023 | 0.7655 | 0.0023 | 0.5382 | 0.0023 | 0.6865 | 0.0023 | 0.7962 | 0.0023 |
| Brazil (11) v. Indonesia (4) | 90951.536 | 0.0010 | 0.1171 | 0.0010 | -2.4811 | 1.0000 | 0.6608 | 0.0012 | 0.7656 | 0.0012 | 0.5577 | 0.0012 | 0.7010 | 0.0012 | 0.8080 | 0.0012 |
| Brazil (11) v. Papua_New_Guinea (2) | 74133.916 | 0.0130 | 0.1090 | 0.0130 | -4.5918 | 1.0000 | 0.6606 | 0.0491 | 0.7636 | 0.0491 | 0.6659 | 0.0522 | 0.7829 | 0.0522 | 0.8697 | 0.0522 |
| Thailand (4) v. Western_North_Atlantic (8) | 75824.367 | 0.0030 | 0.1136 | 0.0030 | -2.4040 | 1.0000 | 0.6593 | 0.0033 | 0.7691 | 0.0033 | 0.5607 | 0.0033 | 0.7036 | 0.0033 | 0.8101 | 0.0033 |
| Fiji (8) v. Gulf_of_Mexico (11) | 121032.342 | 0.0010 | 0.1201 | 0.0010 | -1.3537 | 1.0000 | 0.6562 | 0.0010 | 0.7674 | 0.0010 | 0.5119 | 0.0010 | 0.6660 | 0.0010 | 0.7791 | 0.0010 |
| Sri_Lanka (4) v. Western_North_Atlantic (8) | 75994.828 | 0.0030 | 0.1139 | 0.0030 | -2.2407 | 1.0000 | 0.6560 | 0.0032 | 0.7668 | 0.0032 | 0.5515 | 0.0032 | 0.6970 | 0.0032 | 0.8047 | 0.0032 |
| Gulf_of_Mexico (11) v. Japan (11) | 140297.363 | 0.0010 | 0.1201 | 0.0010 | -1.0983 | 1.0000 | 0.6528 | 0.0010 | 0.7624 | 0.0010 | 0.5043 | 0.0010 | 0.6594 | 0.0010 | 0.7736 | 0.0010 |
| Brazil (11) v. Thailand (4) | 89962.341 | 0.0020 | 0.1121 | 0.0020 | -2.0210 | 1.0000 | 0.6518 | 0.0023 | 0.7573 | 0.0023 | 0.5335 | 0.0024 | 0.6810 | 0.0024 | 0.7920 | 0.0024 |
| Arabian_Sea (6) v. Gulf_of_Mexico (11) | 106824.672 | 0.0010 | 0.1146 | 0.0010 | -1.2937 | 1.0000 | 0.6515 | 0.0010 | 0.7631 | 0.0010 | 0.5039 | 0.0010 | 0.6587 | 0.0010 | 0.7730 | 0.0010 |
| Fiji (8) v. Western_North_Atlantic (8) | 102162.153 | 0.0010 | 0.1194 | 0.0010 | -1.4973 | 1.0000 | 0.6503 | 0.0010 | 0.7624 | 0.0010 | 0.5195 | 0.0010 | 0.6720 | 0.0010 | 0.7842 | 0.0010 |
| Brazil (11) v. Sri_Lanka (4) | 90297.515 | 0.0020 | 0.1126 | 0.0020 | -1.8575 | 1.0000 | 0.6491 | 0.0023 | 0.7552 | 0.0023 | 0.5243 | 0.0023 | 0.6743 | 0.0023 | 0.7864 | 0.0023 |
| Japan (11) v. Western_North_Atlantic (8) | 120651.330 | 0.0010 | 0.1196 | 0.0010 | -1.1916 | 1.0000 | 0.6476 | 0.0010 | 0.7597 | 0.0010 | 0.5099 | 0.0010 | 0.6638 | 0.0010 | 0.7773 | 0.0010 |
| Gulf_of_Mexico (11) v. Mozambique (8) | 119009.537 | 0.0010 | 0.1159 | 0.0010 | -1.0355 | 1.0000 | 0.6474 | 0.0010 | 0.7648 | 0.0010 | 0.4962 | 0.0010 | 0.6520 | 0.0010 | 0.7674 | 0.0010 |
| Caribbean_Sea (2) v. Indonesia (4) | 39028.741 | 0.0779 | 0.1119 | 0.0779 | -19.5630 | 1.0000 | 0.6471 | 0.1615 | 0.7609 | 0.1898 | 0.8856 | 0.2274 | 0.9300 | 0.2274 | 0.9629 | 0.2274 |
| Gulf_of_Mexico (11) v. Reunion (9) | 126641.833 | 0.0010 | 0.1167 | 0.0010 | -0.9377 | 1.0000 | 0.6467 | 0.0010 | 0.7573 | 0.0010 | 0.4936 | 0.0010 | 0.6504 | 0.0010 | 0.7659 | 0.0010 |
| Brazil (11) v. Fiji (8) | 118513.284 | 0.0010 | 0.1180 | 0.0010 | -1.2409 | 1.0000 | 0.6458 | 0.0010 | 0.7584 | 0.0010 | 0.4996 | 0.0010 | 0.6548 | 0.0010 | 0.7698 | 0.0010 |
| Gulf_of_Mexico (11) v. South_Africa (9) | 126189.816 | 0.0010 | 0.1156 | 0.0010 | -0.8796 | 1.0000 | 0.6448 | 0.0010 | 0.7550 | 0.0010 | 0.4904 | 0.0010 | 0.6471 | 0.0010 | 0.7632 | 0.0010 |
| Arabian_Sea (6) v. Western_North_Atlantic (8) | 88918.136 | 0.0010 | 0.1141 | 0.0010 | -1.4101 | 1.0000 | 0.6447 | 0.0010 | 0.7573 | 0.0010 | 0.5112 | 0.0010 | 0.6645 | 0.0010 | 0.7780 | 0.0010 |
| Caribbean_Sea (2) v. Sierra_Leone (1) | 18745.883 | 0.3227 | 0.0679 | 0.3227 | NA | NA | 0.6446 | 0.3227 | 0.7406 | 0.3227 | NA | NA | NA | NA | NA | NA |
| Brazil (11) v. Japan (11) | 137727.301 | 0.0010 | 0.1183 | 0.0010 | -1.0138 | 1.0000 | 0.6435 | 0.0010 | 0.7541 | 0.0010 | 0.4928 | 0.0010 | 0.6488 | 0.0010 | 0.7647 | 0.0010 |
| Gulf_of_Mexico (11) v. Seychelles (10) | 132727.138 | 0.0010 | 0.1168 | 0.0010 | -0.7989 | 1.0000 | 0.6419 | 0.0010 | 0.7543 | 0.0010 | 0.4868 | 0.0010 | 0.6449 | 0.0010 | 0.7612 | 0.0010 |
| Mozambique (8) v. Western_North_Atlantic (8) | 100386.634 | 0.0010 | 0.1152 | 0.0010 | -1.1268 | 1.0000 | 0.6406 | 0.0010 | 0.7538 | 0.0010 | 0.5017 | 0.0010 | 0.6564 | 0.0010 | 0.7712 | 0.0010 |
| Arabian_Sea (6) v. Brazil (11) | 104258.428 | 0.0030 | 0.1126 | 0.0030 | -1.1959 | 1.0000 | 0.6403 | 0.0030 | 0.7529 | 0.0030 | 0.4915 | 0.0030 | 0.6473 | 0.0030 | 0.7635 | 0.0030 |
| Reunion (9) v. Western_North_Atlantic (8) | 107588.086 | 0.0010 | 0.1161 | 0.0010 | -1.0175 | 1.0000 | 0.6400 | 0.0010 | 0.7536 | 0.0010 | 0.4985 | 0.0010 | 0.6542 | 0.0010 | 0.7692 | 0.0010 |
| South_Africa (9) v. Western_North_Atlantic (8) | 107195.063 | 0.0010 | 0.1149 | 0.0010 | -0.9684 | 1.0000 | 0.6380 | 0.0010 | 0.7515 | 0.0010 | 0.4952 | 0.0010 | 0.6510 | 0.0010 | 0.7666 | 0.0010 |
| Caribbean_Sea (2) v. Papua_New_Guinea (2) | 27164.723 | 0.3656 | 0.1045 | 0.3656 | NA | NA | 0.6374 | 0.3656 | 0.7505 | 0.3656 | NA | NA | NA | NA | NA | NA |
| Brazil (11) v. Mozambique (8) | 116483.030 | 0.0010 | 0.1139 | 0.0010 | -0.9406 | 1.0000 | 0.6370 | 0.0010 | 0.7556 | 0.0010 | 0.4841 | 0.0010 | 0.6408 | 0.0010 | 0.7580 | 0.0010 |
| Brazil (11) v. Reunion (9) | 124081.652 | 0.0010 | 0.1148 | 0.0010 | -0.8638 | 1.0000 | 0.6367 | 0.0010 | 0.7485 | 0.0010 | 0.4822 | 0.0010 | 0.6397 | 0.0010 | 0.7569 | 0.0010 |
| Seychelles (10) v. Western_North_Atlantic (8) | 113326.862 | 0.0010 | 0.1161 | 0.0010 | -0.8798 | 1.0000 | 0.6350 | 0.0010 | 0.7495 | 0.0010 | 0.4911 | 0.0010 | 0.6482 | 0.0010 | 0.7641 | 0.0010 |
| Brazil (11) v. South_Africa (9) | 123642.988 | 0.0010 | 0.1137 | 0.0010 | -0.8041 | 1.0000 | 0.6348 | 0.0010 | 0.7461 | 0.0010 | 0.4788 | 0.0010 | 0.6363 | 0.0010 | 0.7541 | 0.0010 |
| Brazil (11) v. Seychelles (10) | 130161.195 | 0.0010 | 0.1149 | 0.0010 | -0.7239 | 1.0000 | 0.6322 | 0.0010 | 0.7456 | 0.0010 | 0.4754 | 0.0010 | 0.6341 | 0.0010 | 0.7520 | 0.0010 |
| Caribbean_Sea (2) v. Japan (11) | 76592.764 | 0.0100 | 0.1129 | 0.0100 | -2.8319 | 1.0000 | 0.6276 | 0.0450 | 0.7462 | 0.0465 | 0.6239 | 0.0529 | 0.7528 | 0.0529 | 0.8478 | 0.0529 |
| Caribbean_Sea (2) v. Thailand (4) | 38513.630 | 0.0749 | 0.1072 | 0.0749 | -10.0183 | 1.0000 | 0.6272 | 0.1638 | 0.7437 | 0.1638 | 0.8095 | 0.1638 | 0.8810 | 0.1638 | 0.9342 | 0.1638 |
| Caribbean_Sea (2) v. Fiji (8) | 60456.741 | 0.0290 | 0.1127 | 0.0290 | -3.6470 | 1.0000 | 0.6238 | 0.2071 | 0.7489 | 0.2071 | 0.6573 | 0.2071 | 0.7774 | 0.2071 | 0.8657 | 0.2071 |
| Australia (34) v. Gulf_of_Mexico (11) | 266279.436 | 0.0010 | 0.1163 | 0.0010 | -0.2294 | 1.0000 | 0.6203 | 0.0010 | 0.7371 | 0.0010 | 0.4736 | 0.0010 | 0.6329 | 0.0010 | 0.7509 | 0.0010 |
| Caribbean_Sea (2) v. Sri_Lanka (4) | 38450.471 | 0.0719 | 0.1072 | 0.0719 | -8.2881 | 1.0000 | 0.6193 | 0.1319 | 0.7368 | 0.1319 | 0.7833 | 0.1319 | 0.8640 | 0.1319 | 0.9237 | 0.1319 |
| Australia (34) v. Western_North_Atlantic (8) | 242136.802 | 0.0010 | 0.1157 | 0.0010 | -0.2818 | 1.0000 | 0.6158 | 0.0010 | 0.7332 | 0.0010 | 0.4751 | 0.0010 | 0.6340 | 0.0010 | 0.7518 | 0.0010 |
| Australia (34) v. Brazil (11) | 262424.982 | 0.0010 | 0.1144 | 0.0010 | -0.1938 | 1.0000 | 0.6134 | 0.0010 | 0.7302 | 0.0010 | 0.4632 | 0.0010 | 0.6228 | 0.0010 | 0.7422 | 0.0010 |
| Caribbean_Sea (2) v. Reunion (9) | 65164.107 | 0.0160 | 0.1093 | 0.0160 | -2.4310 | 1.0000 | 0.6119 | 0.0645 | 0.7340 | 0.0684 | 0.6031 | 0.0684 | 0.7370 | 0.0684 | 0.8359 | 0.0684 |
| Caribbean_Sea (2) v. Mozambique (8) | 59068.619 | 0.0260 | 0.1086 | 0.0260 | -2.7066 | 1.0000 | 0.6098 | 0.3171 | 0.7470 | 0.3171 | 0.6169 | 0.3171 | 0.7469 | 0.3171 | 0.8435 | 0.3171 |
| Arabian_Sea (6) v. Caribbean_Sea (2) | 49216.891 | 0.0559 | 0.1075 | 0.0559 | -3.6443 | 1.0000 | 0.6094 | 0.1842 | 0.7275 | 0.1842 | 0.6560 | 0.2036 | 0.7760 | 0.2036 | 0.8647 | 0.2036 |
| Caribbean_Sea (2) v. South_Africa (9) | 64987.779 | 0.0170 | 0.1082 | 0.0170 | -2.2823 | 1.0000 | 0.6094 | 0.0592 | 0.7309 | 0.0592 | 0.5952 | 0.0632 | 0.7306 | 0.0632 | 0.8311 | 0.0632 |
| Caribbean_Sea (2) v. Seychelles (10) | 70039.173 | 0.0110 | 0.1096 | 0.0110 | -2.0491 | 1.0000 | 0.6071 | 0.0466 | 0.7311 | 0.0466 | 0.5816 | 0.0514 | 0.7208 | 0.0514 | 0.8235 | 0.0514 |
| Australia (34) v. Caribbean_Sea (2) | 184513.747 | 0.0010 | 0.1092 | 0.0010 | -0.8685 | 1.0000 | 0.6017 | 0.0050 | 0.7207 | 0.0050 | 0.5161 | 0.0052 | 0.6683 | 0.0052 | 0.7812 | 0.0052 |
| Papua_New_Guinea (2) v. Sierra_Leone (1) | 6416.767 | 0.3227 | 0.0042 | 0.3227 | NA | NA | 0.0929 | 0.3227 | 0.1126 | 0.3227 | NA | NA | NA | NA | NA | NA |
| Indonesia (4) v. Japan (11) | 11178.826 | 0.0010 | 0.0025 | 0.0010 | -3.8365 | 0.9918 | 0.0750 | 0.0010 | 0.0897 | 0.0010 | 0.1905 | 0.0041 | 0.2406 | 0.0041 | 0.3621 | 0.0041 |
| Fiji (8) v. Indonesia (4) | 10448.990 | 0.0020 | 0.0029 | 0.0020 | -4.2258 | 1.0000 | 0.0732 | 0.0020 | 0.0875 | 0.0020 | 0.2201 | 0.0021 | 0.2747 | 0.0021 | 0.4055 | 0.0021 |
| Fiji (8) v. Japan (11) | 13523.753 | 0.0010 | 0.0024 | 0.0010 | -2.7437 | 1.0000 | 0.0679 | 0.0010 | 0.0814 | 0.0010 | 0.1088 | 0.0010 | 0.1440 | 0.0010 | 0.2279 | 0.0010 |

Table 5: Pairwise Fst for 16989 DArTseq loci between the all sites *(continued)*

| | Chi2 | Chi2.p.val | D | D.p.val | Fis | Fis.p.val | Fst | Fst.p.val | F'st | F'st.p.val | Gst | Gst.p.val | G'st | G'st.p.val | G"st | G"st.p.val |
|---|---|---|---|---|---|---|---|---|---|---|---|---|---|---|---|---|
| Indonesia (4) v. Papua_New_Guinea (2) | 6781.410 | 0.2028 | 0.0023 | 0.2028 | NA | NA | 0.0480 | 0.0972 | 0.0573 | 0.0972 | NA | NA | NA | NA | NA | NA |
| Indonesia (4) v. Thailand (4) | 7570.074 | 0.0320 | 0.0019 | 0.0320 | -6.8647 | 1.0000 | 0.0461 | 0.0331 | 0.0552 | 0.0331 | 0.3524 | 0.0342 | 0.4141 | 0.0342 | 0.5668 | 0.0342 |
| Japan (11) v. Thailand (4) | 9733.776 | 0.0030 | 0.0018 | 0.0120 | -3.5281 | 0.8671 | 0.0440 | 0.0111 | 0.0526 | 0.0111 | 0.1636 | 0.0184 | 0.2091 | 0.0153 | 0.3203 | 0.0164 |
| Indonesia (4) v. Sri_Lanka (4) | 7684.223 | 0.0240 | 0.0019 | 0.1029 | -6.2934 | 1.0000 | 0.0437 | 0.0247 | 0.0525 | 0.0247 | 0.3330 | 0.0254 | 0.3951 | 0.0254 | 0.5462 | 0.0254 |
| Indonesia (4) v. Reunion (9) | 9002.416 | 0.0020 | 0.0018 | 0.0020 | -3.6602 | 1.0000 | 0.0421 | 0.0020 | 0.0507 | 0.0021 | 0.1785 | 0.0021 | 0.2266 | 0.0021 | 0.3437 | 0.0021 |
| Japan (11) v. Reunion (9) | 11453.845 | 0.0010 | 0.0015 | 0.0010 | -2.5427 | 1.0000 | 0.0405 | 0.0010 | 0.0487 | 0.0010 | 0.0847 | 0.0010 | 0.1133 | 0.0010 | 0.1826 | 0.0010 |
| Indonesia (4) v. Seychelles (10) | 9067.995 | 0.0030 | 0.0018 | 0.0030 | -3.4077 | 1.0000 | 0.0404 | 0.0030 | 0.0489 | 0.0030 | 0.1642 | 0.0030 | 0.2102 | 0.0030 | 0.3215 | 0.0030 |
| Japan (11) v. Sri_Lanka (4) | 9650.543 | 0.0010 | 0.0016 | 0.0220 | -3.4005 | 0.6767 | 0.0403 | 0.0112 | 0.0482 | 0.0112 | 0.1570 | 0.0300 | 0.2018 | 0.0248 | 0.3101 | 0.0248 |
| Japan (11) v. Papua_New_Guinea (2) | 8561.875 | 0.0330 | 0.0021 | 0.1568 | -6.5902 | 0.8100 | 0.0403 | 0.0538 | 0.0481 | 0.0538 | 0.3820 | 0.3300 | 0.4454 | 0.3300 | 0.5987 | 0.3300 |
| Japan (11) v. Seychelles (10) | 11592.099 | 0.0010 | 0.0015 | 0.0010 | -2.4653 | 1.0000 | 0.0389 | 0.0010 | 0.0468 | 0.0010 | 0.0786 | 0.0010 | 0.1057 | 0.0010 | 0.1708 | 0.0010 |
| Indonesia (4) v. South_Africa (9) | 8789.531 | 0.0050 | 0.0017 | 0.0050 | -3.6000 | 1.0000 | 0.0381 | 0.0050 | 0.0459 | 0.0050 | 0.1744 | 0.0050 | 0.2217 | 0.0050 | 0.3373 | 0.0050 |
| Fiji (8) v. Reunion (9) | 10665.486 | 0.0010 | 0.0015 | 0.0010 | -2.6226 | 1.0000 | 0.0379 | 0.0010 | 0.0457 | 0.0010 | 0.0980 | 0.0010 | 0.1303 | 0.0010 | 0.2080 | 0.0010 |
| Arabian_Sea (6) v. Japan (11) | 10309.704 | 0.0010 | 0.0015 | 0.0050 | -2.8363 | 0.8881 | 0.0376 | 0.0040 | 0.0452 | 0.0040 | 0.1087 | 0.0080 | 0.1436 | 0.0060 | 0.2276 | 0.0060 |
| Fiji (8) v. Thailand (4) | 8865.162 | 0.0050 | 0.0018 | 0.0050 | -3.8772 | 1.0000 | 0.0374 | 0.0051 | 0.0448 | 0.0051 | 0.1897 | 0.0052 | 0.2396 | 0.0052 | 0.3608 | 0.0052 |
| Fiji (8) v. Seychelles (10) | 10839.719 | 0.0010 | 0.0016 | 0.0010 | -2.5154 | 1.0000 | 0.0372 | 0.0010 | 0.0450 | 0.0010 | 0.0911 | 0.0010 | 0.1219 | 0.0010 | 0.1952 | 0.0010 |
| Japan (11) v. Mozambique (8) | 10712.336 | 0.0010 | 0.0014 | 0.0010 | -2.6315 | 0.9960 | 0.0361 | 0.0010 | 0.0439 | 0.0010 | 0.0903 | 0.0010 | 0.1203 | 0.0010 | 0.1932 | 0.0010 |
| Fiji (8) v. Sri_Lanka (4) | 9100.450 | 0.0010 | 0.0018 | 0.0010 | -3.6561 | 0.9713 | 0.0359 | 0.0010 | 0.0431 | 0.0010 | 0.1808 | 0.0010 | 0.2301 | 0.0010 | 0.3480 | 0.0010 |
| Fiji (8) v. Mozambique (8) | 10160.898 | 0.0010 | 0.0015 | 0.0010 | -2.7350 | 1.0000 | 0.0358 | 0.0010 | 0.0437 | 0.0010 | 0.1063 | 0.0010 | 0.1407 | 0.0010 | 0.2232 | 0.0010 |
| Arabian_Sea (6) v. Indonesia (4) | 8113.920 | 0.0050 | 0.0018 | 0.0090 | -4.4368 | 1.0000 | 0.0358 | 0.0051 | 0.0431 | 0.0051 | 0.2269 | 0.0052 | 0.2815 | 0.0052 | 0.4144 | 0.0052 |
| Indonesia (4) v. Mozambique (8) | 8461.219 | 0.0030 | 0.0017 | 0.0030 | -3.8955 | 1.0000 | 0.0352 | 0.0030 | 0.0432 | 0.0033 | 0.1914 | 0.0033 | 0.2412 | 0.0033 | 0.3631 | 0.0033 |
| Japan (11) v. South_Africa (9) | 10864.284 | 0.0010 | 0.0014 | 0.0010 | -2.5325 | 0.9980 | 0.0351 | 0.0010 | 0.0421 | 0.0010 | 0.0816 | 0.0010 | 0.1092 | 0.0010 | 0.1764 | 0.0010 |
| Fiji (8) v. Papua_New_Guinea (2) | 8199.940 | 0.0340 | 0.0024 | 0.0340 | -8.0103 | 1.0000 | 0.0347 | 0.1288 | 0.0416 | 0.1288 | 0.4471 | 0.4416 | 0.5107 | 0.4416 | 0.6619 | 0.4416 |
| Fiji (8) v. South_Africa (9) | 10212.562 | 0.0010 | 0.0014 | 0.0010 | -2.6095 | 1.0000 | 0.0338 | 0.0010 | 0.0407 | 0.0010 | 0.0955 | 0.0010 | 0.1271 | 0.0010 | 0.2033 | 0.0010 |
| Arabian_Sea (6) v. Fiji (8) | 9533.093 | 0.0010 | 0.0015 | 0.0010 | -3.0109 | 1.0000 | 0.0332 | 0.0010 | 0.0403 | 0.0010 | 0.1263 | 0.0010 | 0.1655 | 0.0010 | 0.2591 | 0.0010 |
| Australia (34) v. Indonesia (4) | 10470.950 | 0.0010 | 0.0015 | 0.0010 | -2.3894 | 1.0000 | 0.0329 | 0.0010 | 0.0396 | 0.0010 | 0.1031 | 0.0010 | 0.1361 | 0.0010 | 0.2169 | 0.0010 |
| Australia (34) v. Fiji (8) | 13038.738 | 0.0010 | 0.0013 | 0.0010 | -1.9589 | 1.0000 | 0.0327 | 0.0010 | 0.0395 | 0.0010 | 0.0566 | 0.0010 | 0.0771 | 0.0010 | 0.1265 | 0.0010 |
| Australia (34) v. Japan (11) | 13998.770 | 0.0010 | 0.0012 | 0.0010 | -1.9736 | 1.0000 | 0.0319 | 0.0010 | 0.0384 | 0.0010 | 0.0471 | 0.0010 | 0.0644 | 0.0010 | 0.1065 | 0.0010 |
| Reunion (9) v. Thailand (4) | 7436.247 | 0.0989 | 0.0011 | 0.0869 | -3.3835 | 0.9254 | 0.0082 | 0.0935 | 0.0099 | 0.0935 | 0.1514 | 0.0867 | 0.1944 | 0.0887 | 0.3003 | 0.0887 |
| Reunion (9) v. Seychelles (10) | 8193.729 | 0.0020 | 0.0007 | 0.0080 | -2.3873 | 0.9960 | 0.0081 | 0.0060 | 0.0098 | 0.0060 | 0.0682 | 0.0060 | 0.0922 | 0.0060 | 0.1502 | 0.0060 |
| Australia (34) v. Reunion (9) | 9163.755 | 0.0010 | 0.0005 | 0.0010 | -1.9225 | 0.9990 | 0.0075 | 0.0010 | 0.0090 | 0.0010 | 0.0389 | 0.0010 | 0.0534 | 0.0010 | 0.0888 | 0.0010 |
| Reunion (9) v. Sri_Lanka (4) | 7667.716 | 0.0969 | 0.0011 | 0.1089 | -3.1964 | 0.6727 | 0.0072 | 0.1330 | 0.0087 | 0.1340 | 0.1439 | 0.1692 | 0.1860 | 0.1601 | 0.2884 | 0.1672 |
| Mozambique (8) v. Reunion (9) | 8036.435 | 0.0040 | 0.0008 | 0.0020 | -2.5285 | 0.9880 | 0.0072 | 0.0050 | 0.0087 | 0.0050 | 0.0809 | 0.0040 | 0.1083 | 0.0040 | 0.1750 | 0.0040 |
| Brazil (11) v. Western_North_Atlantic (8) | 5969.634 | 0.0010 | 0.0003 | 0.0010 | -5.7343 | 1.0000 | 0.0069 | 0.0010 | 0.0079 | 0.0010 | 0.1470 | 0.0010 | 0.1769 | 0.0010 | 0.2824 | 0.0010 |
| Reunion (9) v. South_Africa (9) | 7939.288 | 0.0170 | 0.0007 | 0.0080 | -2.4433 | 0.9930 | 0.0065 | 0.0110 | 0.0079 | 0.0110 | 0.0724 | 0.0110 | 0.0974 | 0.0110 | 0.1584 | 0.0110 |
| Seychelles (10) v. Thailand (4) | 7243.604 | 0.1888 | 0.0010 | 0.1059 | -3.2235 | 0.9239 | 0.0059 | 0.1131 | 0.0071 | 0.1131 | 0.1400 | 0.0891 | 0.1810 | 0.0911 | 0.2816 | 0.0891 |
| Seychelles (10) v. South_Africa (9) | 7830.997 | 0.0150 | 0.0006 | 0.0060 | -2.3823 | 0.9910 | 0.0054 | 0.0090 | 0.0065 | 0.0090 | 0.0667 | 0.0080 | 0.0902 | 0.0080 | 0.1471 | 0.0080 |
| Arabian_Sea (6) v. Reunion (9) | 7743.722 | 0.0699 | 0.0008 | 0.1429 | -2.7339 | 0.6593 | 0.0049 | 0.1948 | 0.0060 | 0.1948 | 0.0981 | 0.2168 | 0.1301 | 0.2098 | 0.2078 | 0.2118 |
| Caribbean_Sea (2) v. Western_North_Atlantic (8) | 4466.898 | 0.2947 | 0.0009 | 0.0460 | 28.5591 | 1.0000 | 0.0043 | 0.4340 | 0.0050 | 1.0000 | 1.5682 | 1.0000 | 1.4871 | 1.0000 | 1.1897 | 1.0000 |
| Brazil (11) v. Gulf_of_Mexico (11) | 6086.428 | 0.0010 | 0.0002 | 0.0170 | -5.1049 | 0.9960 | 0.0041 | 0.0050 | 0.0047 | 0.0050 | 0.1126 | 0.0050 | 0.1373 | 0.0050 | 0.2246 | 0.0050 |
| Papua_New_Guinea (2) v. Reunion (9) | 7257.257 | 0.1179 | 0.0017 | 0.4466 | -5.9832 | 0.2185 | 0.0039 | 0.4290 | 0.0047 | 0.4408 | 0.3588 | 0.6975 | 0.4221 | 0.5798 | 0.5747 | 0.5798 |
| Australia (34) v. Seychelles (10) | 8496.485 | 0.0010 | 0.0004 | 0.0020 | -1.8963 | 0.7323 | 0.0035 | 0.0060 | 0.0043 | 0.0060 | 0.0336 | 0.0050 | 0.0463 | 0.0050 | 0.0773 | 0.0050 |
| South_Africa (9) v. Sri_Lanka (4) | 7270.440 | 0.0220 | 0.0010 | 0.0460 | -3.1960 | 0.5207 | 0.0034 | 0.0535 | 0.0041 | 0.0535 | 0.1420 | 0.1312 | 0.1837 | 0.1140 | 0.2852 | 0.1241 |
| Mozambique (8) v. Seychelles (10) | 7676.401 | 0.1708 | 0.0006 | 0.1039 | -2.4553 | 0.7952 | 0.0033 | 0.1898 | 0.0040 | 0.1918 | 0.0733 | 0.1279 | 0.0986 | 0.1319 | 0.1601 | 0.1299 |
| Seychelles (10) v. Sri_Lanka (4) | 7383.298 | 0.1638 | 0.0010 | 0.2408 | -3.0516 | 0.6100 | 0.0031 | 0.2910 | 0.0038 | 0.2910 | 0.1322 | 0.3280 | 0.1721 | 0.3320 | 0.2688 | 0.3320 |
| Papua_New_Guinea (2) v. Thailand (4) | 6091.084 | 0.1299 | 0.0017 | 0.4565 | NA | NA | 0.0024 | 0.2379 | 0.0029 | 0.3265 | NA | NA | NA | NA | NA | NA |
| Australia (34) v. Mozambique (8) | 8236.530 | 0.0010 | 0.0004 | 0.0180 | -1.9434 | 0.1419 | 0.0023 | 0.0020 | 0.0028 | 0.0020 | 0.0411 | 0.0020 | 0.0563 | 0.0020 | 0.0935 | 0.0020 |
| Sri_Lanka (4) v. Thailand (4) | 6497.360 | 0.1149 | 0.0011 | 0.2398 | -5.5253 | 0.7436 | 0.0022 | 0.2587 | 0.0027 | 0.2413 | 0.2885 | 0.2925 | 0.3478 | 0.2925 | 0.4938 | 0.2925 |
| Australia (34) v. South_Africa (9) | 8263.583 | 0.0010 | 0.0004 | 0.0040 | -1.9207 | 0.6953 | 0.0022 | 0.0040 | 0.0027 | 0.0040 | 0.0361 | 0.0060 | 0.0497 | 0.0060 | 0.0828 | 0.0060 |
| Australia (34) v. Thailand (4) | 7765.556 | 0.1978 | 0.0008 | 0.1538 | -2.2952 | 1.0000 | 0.0020 | 0.1064 | 0.0024 | 0.1074 | 0.0832 | 0.0171 | 0.1108 | 0.0211 | 0.1791 | 0.0211 |
| Australia (34) v. Sri_Lanka (4) | 8208.993 | 0.0180 | 0.0008 | 0.1698 | -2.1999 | 0.1518 | 0.0019 | 0.1052 | 0.0023 | 0.1052 | 0.0797 | 0.3578 | 0.1068 | 0.2905 | 0.1727 | 0.3146 |
| Arabian_Sea (6) v. Seychelles (10) | 7435.985 | 0.3037 | 0.0007 | 0.4146 | -2.6269 | 0.4625 | 0.0018 | 0.3686 | 0.0022 | 0.3686 | 0.0894 | 0.3746 | 0.1193 | 0.3736 | 0.1915 | 0.3736 |
| Mozambique (8) v. South_Africa (9) | 7504.197 | 0.0529 | 0.0007 | 0.0140 | -2.5352 | 0.8931 | 0.0017 | 0.0709 | 0.0021 | 0.0719 | 0.0784 | 0.0410 | 0.1049 | 0.0410 | 0.1700 | 0.0410 |
| South_Africa (9) v. Thailand (4) | 7002.891 | 0.3307 | 0.0009 | 0.1758 | -3.3554 | 0.9898 | 0.0017 | 0.2444 | 0.0020 | 0.2444 | 0.1472 | 0.0530 | 0.1892 | 0.0591 | 0.2933 | 0.0591 |

Table 5: Pairwise Fst for 16989 DArTseq loci between the all sites *(continued)*

| | Chi2 | Chi2.p.val | D | D.p.val | Fis | Fis.p.val | Fst | Fst.p.val | F'st | F'st.p.val | Gst | Gst.p.val | G'st | G'st.p.val | G"st | G"st.p.val |
|---|---|---|---|---|---|---|---|---|---|---|---|---|---|---|---|---|
| Caribbean_Sea (2) v. Gulf_of_Mexico (11) | 4659.761 | 0.5754 | 0.0008 | 0.2348 | 95.8315 | 1.0000 | 0.0011 | 1.0000 | 0.0013 | 1.0000 | 1.1312 | 1.0000 | 1.1146 | 1.0000 | 1.0538 | 1.0000 |
| Papua_New_Guinea (2) v. Sri_Lanka (4) | 6164.749 | 0.3277 | 0.0017 | 0.8621 | -26.8706 | 0.3073 | 0.0007 | 0.4283 | 0.0008 | 0.3458 | 0.7616 | 1.0000 | 0.7981 | 1.0000 | 0.8854 | 1.0000 |
| Gulf_of_Mexico (11) v. Western_North_Atlantic (8) | 5176.118 | 0.4765 | 0.0002 | 0.3506 | -6.4515 | 0.6280 | 0.0003 | 0.4400 | 0.0003 | 0.4400 | 0.1584 | 0.4280 | 0.1888 | 0.4270 | 0.2997 | 0.4260 |
| Brazil (11) v. Caribbean_Sea (2) | 4866.404 | 0.7882 | 0.0009 | 0.2078 | -211.9907 | 1.0000 | 0.0002 | 1.0000 | 0.0003 | 1.0000 | 0.9505 | 1.0000 | 0.9564 | 1.0000 | 0.9777 | 1.0000 |
| Mozambique (8) v. Thailand (4) | 6935.491 | 0.5385 | 0.0009 | 0.5554 | -3.6041 | 0.6087 | 0.0002 | 0.4400 | 0.0003 | 0.4400 | 0.1636 | 0.7027 | 0.2084 | 0.7224 | 0.3197 | 0.7169 |
| Mozambique (8) v. Sri_Lanka (4) | 7159.624 | 0.2448 | 0.0010 | 0.3407 | -3.3991 | 0.1049 | 0.0002 | 0.4591 | 0.0003 | 0.4591 | 0.1557 | 0.8559 | 0.2000 | 0.8485 | 0.3078 | 0.8485 |
| Arabian_Sea (6) v. Australia (34) | 7994.027 | 0.0180 | 0.0005 | 0.1898 | -2.0228 | 0.0030 | 0.0002 | 0.4585 | 0.0002 | 0.4585 | 0.0515 | 0.7403 | 0.0703 | 0.6853 | 0.1158 | 0.7153 |
| Papua_New_Guinea (2) v. Seychelles (10) | 7000.832 | 0.3327 | 0.0016 | 0.6054 | -5.3888 | 0.2640 | 0.0001 | 0.4757 | 0.0001 | 0.6190 | 0.3286 | 0.8080 | 0.3913 | 0.8080 | 0.5418 | 0.8080 |
| Papua_New_Guinea (2) v. South_Africa (9) | 6953.554 | 0.1668 | 0.0016 | 0.7572 | -5.8186 | 0.1702 | -0.0004 | 0.6738 | -0.0005 | 0.6935 | 0.3509 | 1.0000 | 0.4139 | 1.0000 | 0.5661 | 1.0000 |
| Arabian_Sea (6) v. South_Africa (9) | 7177.547 | 0.6054 | 0.0007 | 0.3876 | -2.7254 | 0.0450 | -0.0008 | 0.7333 | -0.0010 | 0.7333 | 0.0950 | 0.8052 | 0.1261 | 0.7922 | 0.2019 | 0.7982 |
| Arabian_Sea (6) v. Sri_Lanka (4) | 6876.948 | 0.3447 | 0.0011 | 0.1269 | -3.8635 | 0.4256 | -0.0009 | 0.6264 | -0.0011 | 0.6264 | 0.1880 | 0.6730 | 0.2380 | 0.6730 | 0.3586 | 0.6730 |
| Arabian_Sea (6) v. Thailand (4) | 6678.836 | 0.6434 | 0.0010 | 0.4326 | -4.0768 | 0.9396 | -0.0011 | 0.6703 | -0.0013 | 0.6703 | 0.1961 | 0.3857 | 0.2464 | 0.4179 | 0.3700 | 0.4068 |
| Arabian_Sea (6) v. Mozambique (8) | 7148.821 | 0.8262 | 0.0007 | 0.7922 | -2.8570 | 0.0160 | -0.0022 | 0.9451 | -0.0028 | 0.9461 | 0.1051 | 0.9820 | 0.1387 | 0.9780 | 0.2206 | 0.9790 |
| Australia (34) v. Papua_New_Guinea (2) | 7672.289 | 0.4126 | 0.0015 | 0.9291 | -3.1099 | 0.0230 | -0.0036 | 0.8812 | -0.0043 | 0.8812 | 0.1955 | 1.0000 | 0.2463 | 1.0000 | 0.3695 | 1.0000 |
| Mozambique (8) v. Papua_New_Guinea (2) | 6753.842 | 0.7932 | 0.0016 | 1.0000 | -6.7316 | 0.6102 | -0.0077 | 1.0000 | -0.0094 | 1.0000 | 0.3916 | 1.0000 | 0.4548 | 1.0000 | 0.6082 | 1.0000 |
| Arabian_Sea (6) v. Papua_New_Guinea (2) | 6374.787 | 0.8841 | 0.0016 | 1.0000 | -9.1102 | 0.3263 | -0.0081 | 1.0000 | -0.0098 | 1.0000 | 0.4828 | 1.0000 | 0.5444 | 1.0000 | 0.6928 | 1.0000 |
| Arabian_Sea (6) v. Sierra_Leone (1) | 9707.152 | 0.1518 | 0.0051 | 0.1518 | NA | NA | NA | NA | NA | NA | NA | NA | NA | NA | NA | NA |
| Australia (34) v. Sierra_Leone (1) | 24824.983 | 0.0340 | 0.0053 | 0.0340 | NA | NA | NA | NA | NA | NA | NA | NA | NA | NA | NA | NA |
| Brazil (11) v. Sierra_Leone (1) | 55309.051 | 0.0819 | 0.0714 | 0.0819 | NA | NA | NA | NA | NA | NA | NA | NA | NA | NA | NA | NA |
| Fiji (8) v. Sierra_Leone (1) | 12245.299 | 0.1279 | 0.0061 | 0.1279 | NA | NA | NA | NA | NA | NA | NA | NA | NA | NA | NA | NA |
| Gulf_of_Mexico (11) v. Sierra_Leone (1) | 58236.780 | 0.0789 | 0.0724 | 0.0789 | NA | NA | NA | NA | NA | NA | NA | NA | NA | NA | NA | NA |
| Indonesia (4) v. Sierra_Leone (1) | 9125.677 | 0.2088 | 0.0061 | 0.2088 | NA | NA | NA | NA | NA | NA | NA | NA | NA | NA | NA | NA |
| Japan (11) v. Sierra_Leone (1) | 14485.463 | 0.0839 | 0.0060 | 0.0839 | NA | NA | NA | NA | NA | NA | NA | NA | NA | NA | NA | NA |
| Mozambique (8) v. Sierra_Leone (1) | 11045.550 | 0.1279 | 0.0051 | 0.1279 | NA | NA | NA | NA | NA | NA | NA | NA | NA | NA | NA | NA |
| Reunion (9) v. Sierra_Leone (1) | 12026.058 | 0.1059 | 0.0053 | 0.1059 | NA | NA | NA | NA | NA | NA | NA | NA | NA | NA | NA | NA |
| Seychelles (10) v. Sierra_Leone (1) | 12316.464 | 0.0819 | 0.0053 | 0.0819 | NA | NA | NA | NA | NA | NA | NA | NA | NA | NA | NA | NA |
| Sierra_Leone (1) v. South_Africa (9) | 11819.383 | 0.1129 | 0.0051 | 0.1129 | NA | NA | NA | NA | NA | NA | NA | NA | NA | NA | NA | NA |
| Sierra_Leone (1) v. Sri_Lanka (4) | 8331.470 | 0.2088 | 0.0050 | 0.2088 | NA | NA | NA | NA | NA | NA | NA | NA | NA | NA | NA | NA |
| Sierra_Leone (1) v. Thailand (4) | 8268.943 | 0.2218 | 0.0047 | 0.2218 | NA | NA | NA | NA | NA | NA | NA | NA | NA | NA | NA | NA |
| Sierra_Leone (1) v. Western_North_Atlantic (8) | 46417.112 | 0.1239 | 0.0722 | 0.1239 | NA | NA | NA | NA | NA | NA | NA | NA | NA | NA | NA | NA |

## 5.8 PCA

```
pca1 <- adegenet::glPca(BS.dartseq.gl, nf = 5, parallel = TRUE,
                        n.cores = parallel::detectCores() - 1)
save(pca1, file = "DArTseq_ALL_PCA2.Rdata")
```

```
load("DArTseq_ALL_PCA2.Rdata")
load("DArTseq_ALL_objects3.Rdata")

pop.levels <- c("Brazil","Caribbean Sea","Gulf of Mexico",  "Western North Atlantic",
                "Sierra Leone", "South Africa", "Mozambique", "Reunion" ,
                "Seychelles", "Arabian Sea", "Sri Lanka", "Thailand", "Indonesia",
                "Papua New Guinea" , "Fitzroy River", "Daly River", "Adelaide River",
                "South Alligator River", "East Alligator River","Blue mud Bay",
                "Roper River","Towns River","Wenlock River","Trinity inlet","Clarence River",
                "Sydney Harbour", "Okinawa", "Urauchi River", "Fiji")
BS.dartseq.gl$pop <- factor(BS.dartseq.gl$other$ind.metrics$Site2,
                            levels = pop.levels)

BS.pca.scores <- as.data.frame(pca1$scores)
BS.pca.scores$pop <- adegenet::pop(BS.dartseq.gl)
cols <- adegenet::funky(nPop(BS.dartseq.gl))


p <-
  ggplot2::ggplot(BS.pca.scores, ggplot2::aes(x = PC1, y = PC2, colour = pop)) +
  ggplot2::geom_point(size = 2) + ggplot2::scale_color_manual(values = cols) +
  ggplot2::geom_hline(yintercept = 0) + ggplot2::geom_vline(xintercept = 0) +
  ggplot2::theme_minimal() +
  ggplot2::theme(
    axis.text = ggplot2::element_text(size = 5),
    axis.title.x = ggplot2::element_text(size = 10),
    axis.title.y = ggplot2::element_text(size = 10),
    legend.text = ggplot2::element_text(size = 7)
  )
p
```

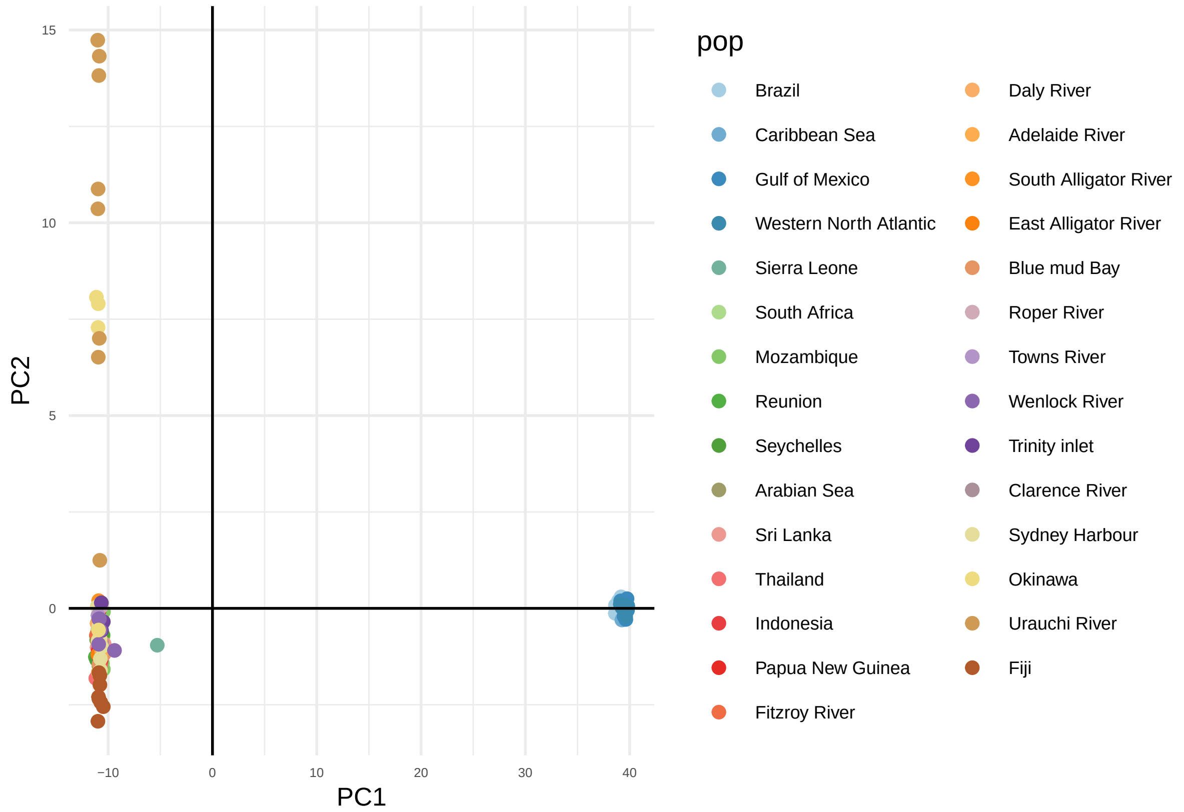


```
ggplot2::ggsave(p, filename = "DArTseq_ALL_PCA1.png", width = 30,
                height = 15,units = "cm")



p <-
  ggplot2::ggplot(BS.pca.scores, ggplot2::aes(x = PC1, y = PC3, colour = pop)) +
  ggplot2::geom_point(size = 2) + ggplot2::scale_color_manual(values = cols) +
  ggplot2::geom_hline(yintercept = 0) + ggplot2::geom_vline(xintercept = 0) +
  ggplot2::theme_minimal() +
  ggplot2::theme(
    axis.text = ggplot2::element_text(size = 5),
    axis.title.x = ggplot2::element_text(size = 10),
    axis.title.y = ggplot2::element_text(size = 10),
    legend.text = ggplot2::element_text(size = 7)
  )
p
```

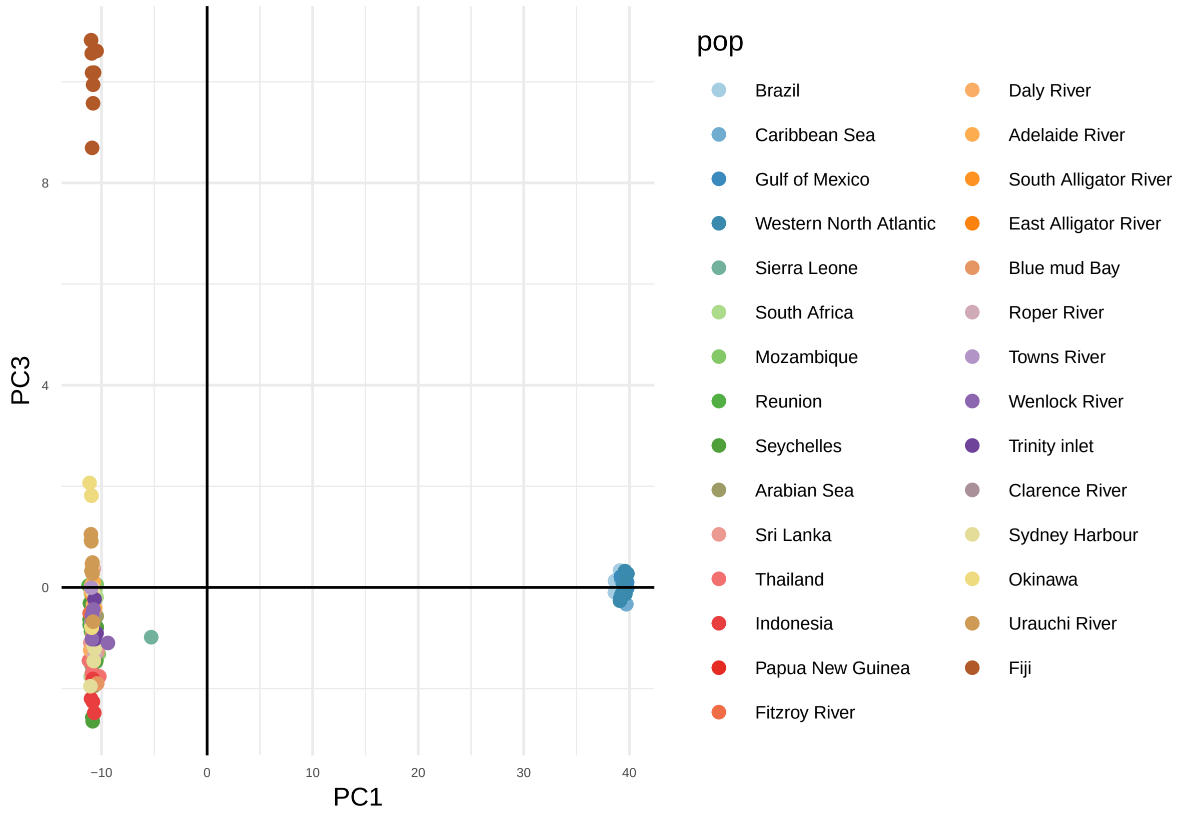


```r
ggplot2::ggsave(p, filename = "DArTseq_ALL_PCA2.png", width = 30, height = 15,
                units = "cm")

p <-
  ggplot2::ggplot(BS.pca.scores, ggplot2::aes(x = PC2, y = PC3, colour = pop)) +
  ggplot2::geom_point(size = 2) + ggplot2::scale_color_manual(values = cols) +
  ggplot2::geom_hline(yintercept = 0) + ggplot2::geom_vline(xintercept = 0) +
  ggplot2::theme_minimal() +
  ggplot2::theme(
    axis.text = ggplot2::element_text(size = 5),
    axis.title.x = ggplot2::element_text(size = 10),
    axis.title.y = ggplot2::element_text(size = 10),
    legend.text = ggplot2::element_text(size = 7)
  )
p
```

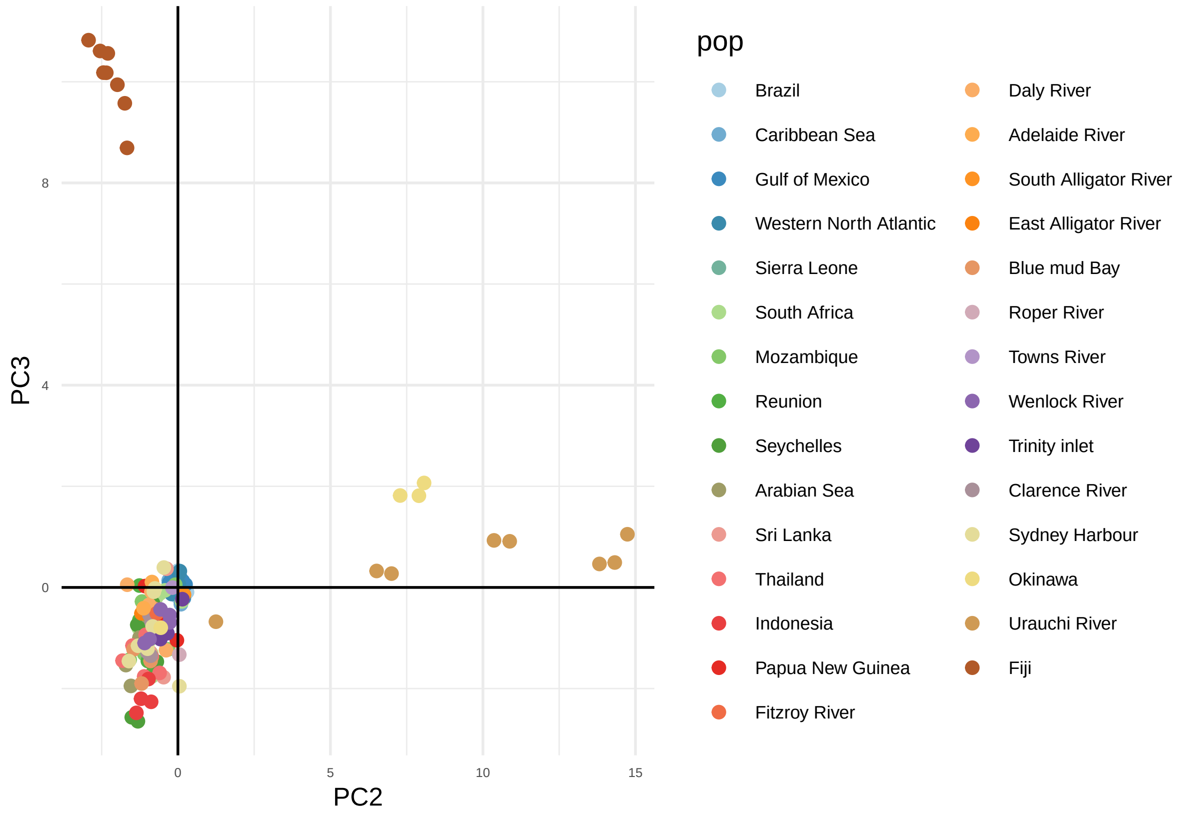


```
ggplot2::ggsave(p, filename = "DArTseq_ALL_PCA3.png", width = 30, height = 15,
                units = "cm")


p <-
  ggplot2::ggplot(BS.pca.scores, ggplot2::aes(x = PC3, y = PC4, colour = pop)) +
  ggplot2::geom_point(size = 2) + ggplot2::scale_color_manual(values = cols) +
  ggplot2::geom_hline(yintercept = 0) + ggplot2::geom_vline(xintercept = 0) +
  ggplot2::theme_minimal() +
  ggplot2::theme(
    axis.text = ggplot2::element_text(size = 5),
    axis.title.x = ggplot2::element_text(size = 10),
    axis.title.y = ggplot2::element_text(size = 10),
    legend.text = ggplot2::element_text(size = 7)
  )
p
```

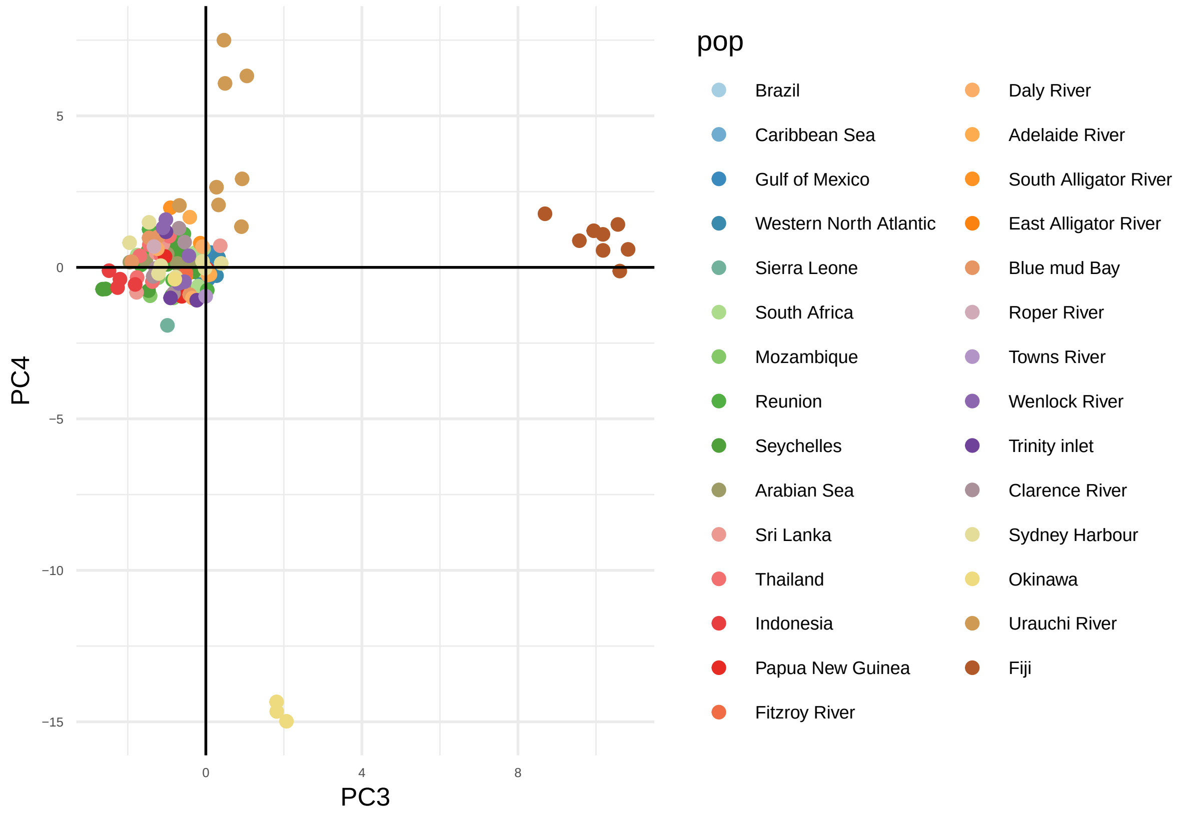


```
ggplot2::ggsave(p, filename = "DArTseq_ALL_PCA4.png", width = 30, height = 15,
                units = "cm")

p <-
  ggplot2::ggplot(BS.pca.scores, ggplot2::aes(x = PC3, y = PC5, colour = pop)) +
  ggplot2::geom_point(size = 2) + ggplot2::scale_color_manual(values = cols) +
  ggplot2::geom_hline(yintercept = 0) + ggplot2::geom_vline(xintercept = 0) +
  ggplot2::theme_minimal() +
  ggplot2::theme(
    axis.text = ggplot2::element_text(size = 5),
    axis.title.x = ggplot2::element_text(size = 10),
    axis.title.y = ggplot2::element_text(size = 10),
    legend.text = ggplot2::element_text(size = 7)
  )
p
```

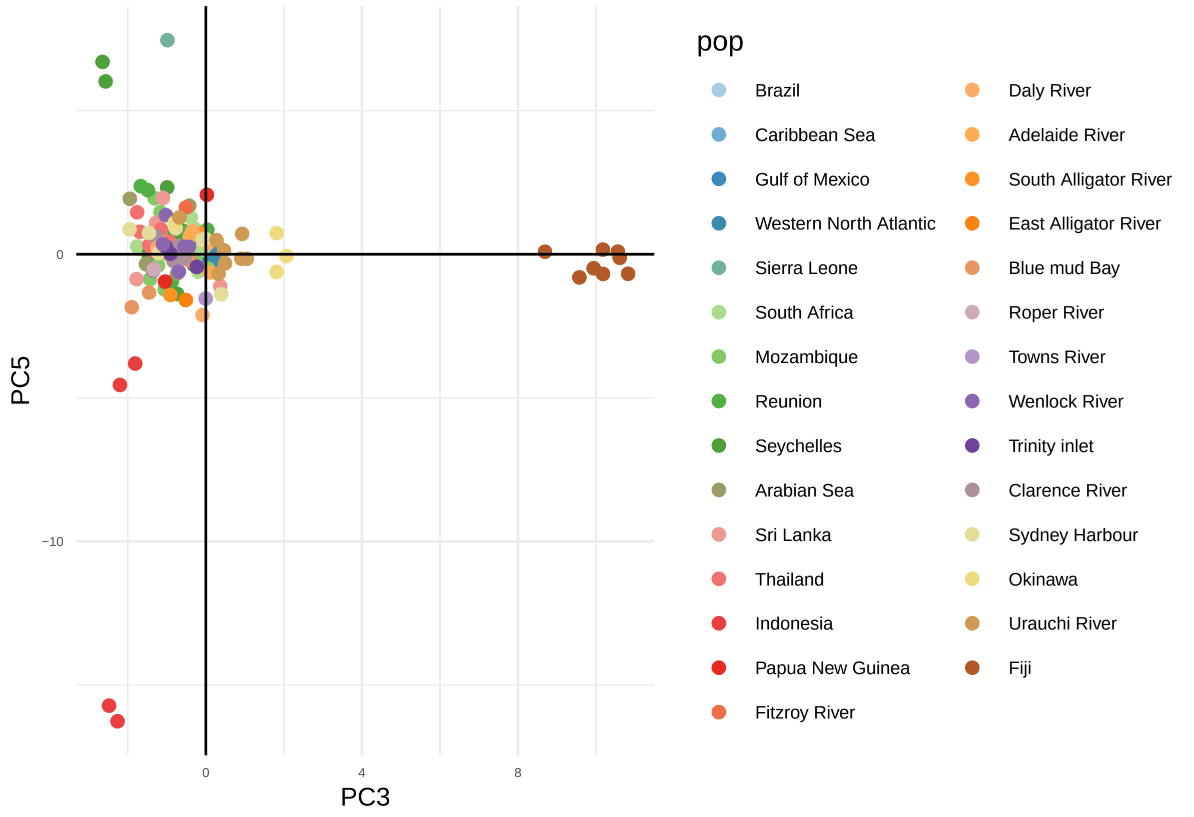


```
ggplot2::ggsave(p, filename = "DArTseq_ALL_PCA5.png", width = 30, height = 15,
                units = "cm")
```

## 5.9 DAPC

### 5.9.1 Optimal number of clusters with neutral markers

```
set.seed(124)
grp <-
  adegenet::find.clusters(
    BS.dartseq.gl,
    max.n.clust = 18,
    n.pca = adegenet::nInd(BS.dartseq.gl) / 3,
    stat = "BIC",
    parallel = TRUE,
    n.cores = parallel::detectCores() - 1
  )

save(grp, file = "DArTseq_ALL_grp_.Rdata")
```

```
load("DArTseq_ALL_grp_.Rdata")

y <- as.numeric(grp$Kstat)
x <- 1:18
data <- data.frame(x,y,stringsAsFactors = F)
plot <- ggplot2::ggplot(data, ggplot2::aes(x,y)) +
  ggplot2::geom_point(size = 5, shape = 1, color = "blue") +
  ggplot2::geom_line(size = 1,color = "blue") +
  ggplot2::scale_x_continuous(name = waiver(),
                              breaks = seq(from = 0,
                                           to = nrow(BS.dartseq.gl) - 1, by = 1)) +
```

```
  ggplot2::labs(subtitle = "",
                y = "Bayesian Information Criterion",
                x = "Number of clusters",
                title = "",
                caption = "") +
  ggplot2::theme_classic() +
  ggplot2::theme(
    axis.text = ggplot2::element_text(size = 5),
    axis.title.x = ggplot2::element_text(size = 20,vjust = 0, hjust = 0.5),
    axis.title.y = ggplot2::element_text(size = 20,vjust = 2, hjust = 0.5))
print(plot)
```

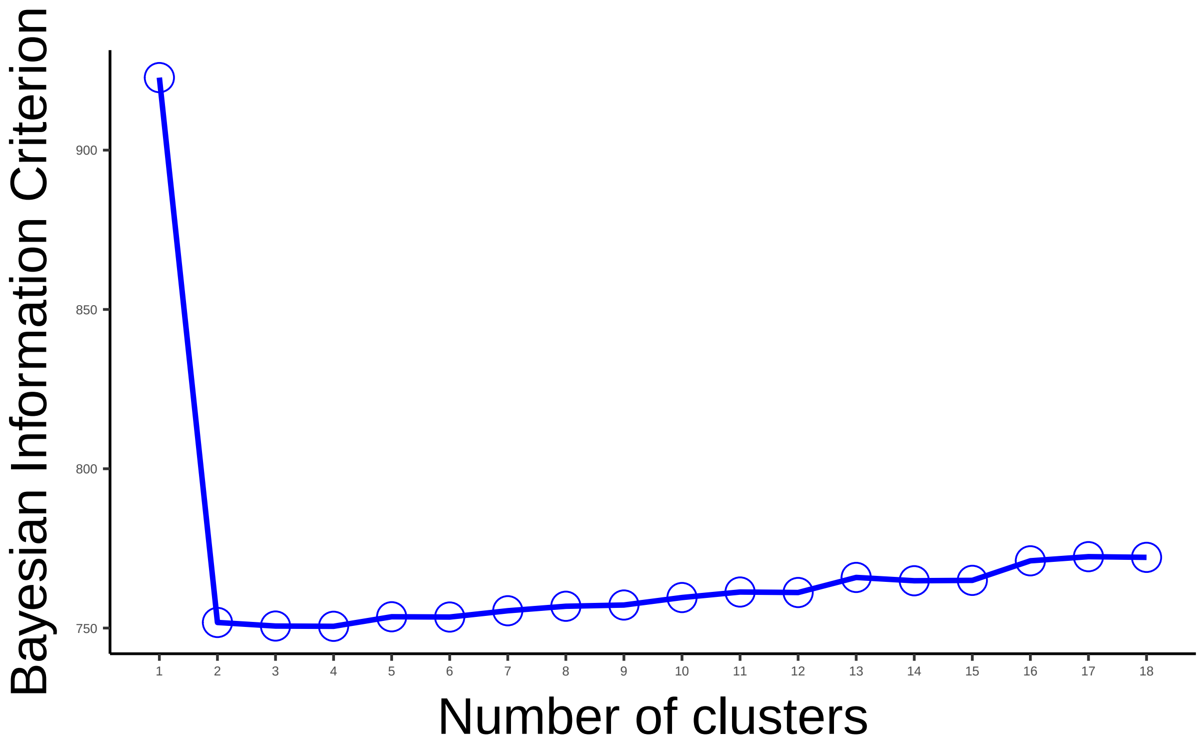


```
ggplot2::ggsave(plot, filename = "DArTseq_ALL_grp.png", width = 30, height = 15,
                units = "cm")
```

### 5.9.2 Cross-validation to identify the optimal number of PCs

Uses a training - holdout set of individuals to check how reliable individuals can be assigned.

```
set.seed(124)
x <- BS.dartseq.gl
x <- x[x$pop != "Sierra Leone",]
pop.levels <- c("Brazil","Caribbean Sea","Gulf of Mexico",  "Western North Atlantic",
                "Sierra Leone", "South Africa", "Mozambique", "Reunion" , "Seychelles",
                "Arabian Sea", "Sri Lanka", "Thailand", "Indonesia", "Papua New Guinea",
                "Australia", "Japan", "Fiji")
x$pop <- factor(x$other$ind.metrics$Site, levels = pop.levels)
x <- x[x$pop != "Sierra Leone"] #only 1 sample


xval <-
  adegenet::xvalDapc(
```

```r
  tab(x, NA.method = "mean"),
  adegenet::pop(x),
  n.pca = seq(1, adegenet::nInd(x), by = 20),
  training.set = 0.9,
  result = "groupMean",
  center = TRUE,
  scale = FALSE,
  n.rep = 1000,
  parallel = "multicore",
  ncpus = 1,
  xval.plot = TRUE
  )
print(xval[2:6])
PC <- as.numeric(xval$`Number of PCs Achieving Lowest MSE`)

#refine the analysis
set.seed(124)
png(file="DArTseq_ALL_xval.png")
xval <-
  adegenet::xvalDapc(
  tab(x, NA.method = "mean"),
  adegenet::pop(x),
  n.pca = (PC - 15):(PC + 15),
  n.rep = 1000,
  parallel = "multicore",
  ncpus = 1,
  xval.plot = TRUE
  )
dev.off()
save(xval, file = "DArTseq_ALL_xval.rdata")
```

```r
load("DArTseq_ALL_xval.rdata")
print(xval[2:6])
PC <- as.numeric(xval$`Number of PCs Achieving Lowest MSE`)
```

```
## $`Median and Confidence Interval for Random Chance`
##       2.5%        50%      97.5%
## 0.03180112 0.06023563 0.10943031
##
## $`Mean Successful Assignment by Number of PCs of PCA`
##         6         7         8         9        10        11        12        13
## 0.3334479 0.3421860 0.3338213 0.3383668 0.3321606 0.3407525 0.3438592 0.3456094
##        14        15        16        17        18        19        20        21
## 0.3487801 0.3492491 0.3410468 0.3400176 0.3382993 0.3396637 0.3410831 0.3390233
##        22        23        24        25        26        27        28        29
## 0.3431461 0.3447303 0.3461724 0.3434242 0.3417871 0.3413665 0.3411261 0.3391184
##        30        31        32        33        34        35        36
## 0.3435539 0.3440634 0.3433089 0.3476013 0.3457444 0.3356826 0.3372854
##
## $`Number of PCs Achieving Highest Mean Success`
## [1] "15"
##
## $`Root Mean Squared Error by Number of PCs of PCA`
##         6         7         8         9        10        11        12        13
## 0.6675354 0.6591730 0.6674186 0.6629926 0.6691257 0.6606877 0.6575962 0.6558610
##        14        15        16        17        18        19        20        21
## 0.6527248 0.6521758 0.6603002 0.6613992 0.6631257 0.6618169 0.6602382 0.6624344
##        22        23        24        25        26        27        28        29
```

```
## 0.6582304 0.6566346 0.6554321 0.6580812 0.6597661 0.6600890 0.6603060 0.6625241
##        30        31        32        33        34        35        36
## 0.6581730 0.6575802 0.6584071 0.6542000 0.6559334 0.6660468 0.6644172
##
## $`Number of PCs Achieving Lowest MSE`
## [1] "15"
```

```
dapc.all.object.names <- c()
for (K in 2:10) {
  set.seed(124)
  grp <- adegenet::find.clusters(BS.dartseq.gl, n.clust = K, n.pca = PC)
  set.seed(124)
  dapc.all.neutral <- adegenet::dapc(BS.dartseq.gl, grp$grp, n.da = K - 1,
                                     n.pca = PC)
  assign(paste0("dapc.all.", K),value = dapc.all.neutral)
  dapc.all.object.names <- c(dapc.all.object.names,
                             paste0("dapc.all.", K))
}
save(list = dapc.all.object.names, dapc.all.object.names,
     file = "DArTseq_ALL_DAPC2.Rdata")
```

### 5.9.3 DAPC barplot

Group individuals according to DAPC posterior membership.

```
load("DArTseq_ALL_DAPC2.Rdata")
for (K in 2:10) {
  colour <- colours.prior[1:K]
  dapc <- get(dapc.all.object.names[K - 1])
  post <- as.matrix(dapc$posterior)
  colnames(post) <- paste0("Group", 1:nlevels(dapc$grp))
  locations <- BS.dartseq.gl$pop

  plot.dapc.FDD(x = post,
                locations = locations,
                colour = colour,
                region.lwd = 1,
                plotTitle = paste0("DAPC: all Bull Sharks - subset Australia\n for K=",
                                   K, " & PC=", PC, sep = ""))
  dev.print(
    device = png,
    file = paste0("DArTseq_ALL_DAPC_barplot_K", K, ".png"),
    res = 300,
    width = 30,
    height = 15,
    units = "cm")
}
```

# DAPC: all Bull Sharks − subset Australia for K=2 & PC=15

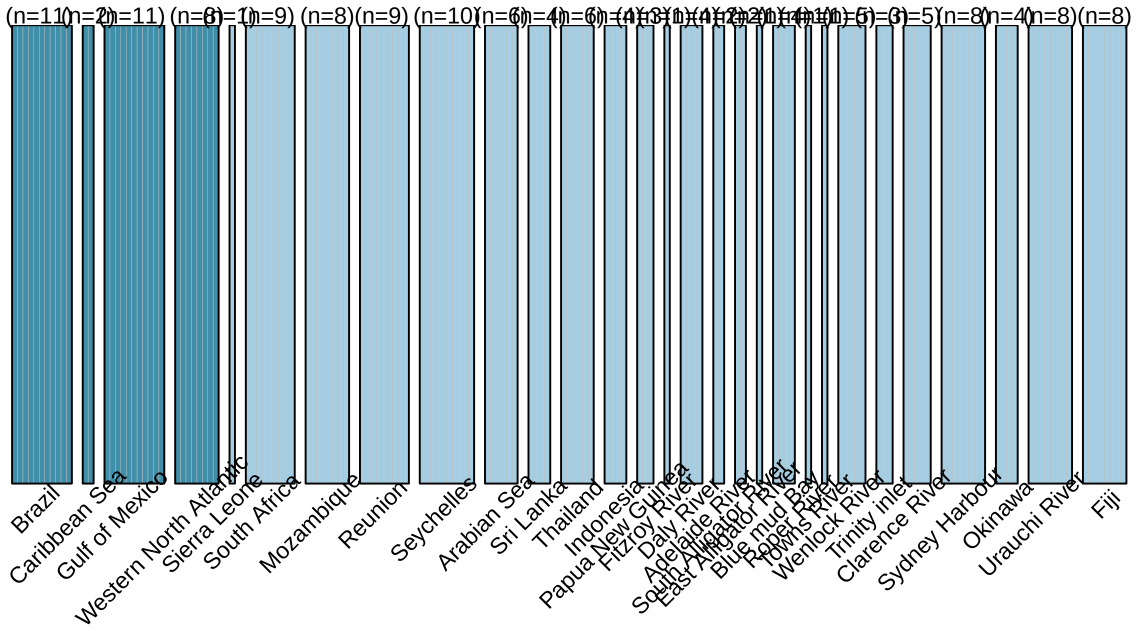


# DAPC: all Bull Sharks − subset Australia for K=3 & PC=15

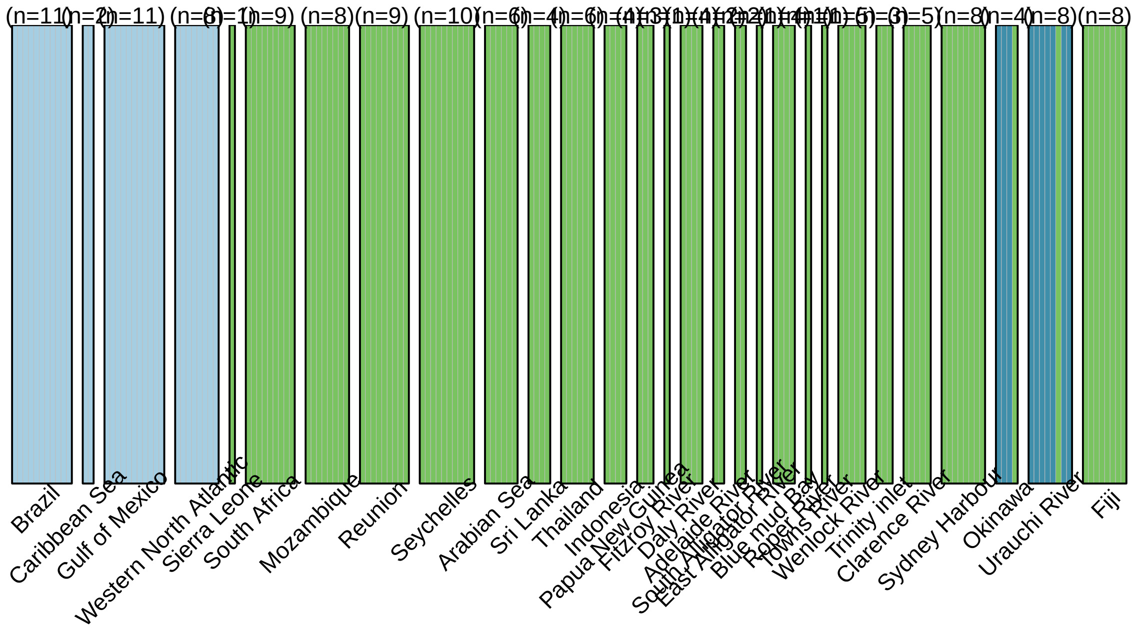

## DAPC: all Bull Sharks − subset Australia for K=4 & PC=15

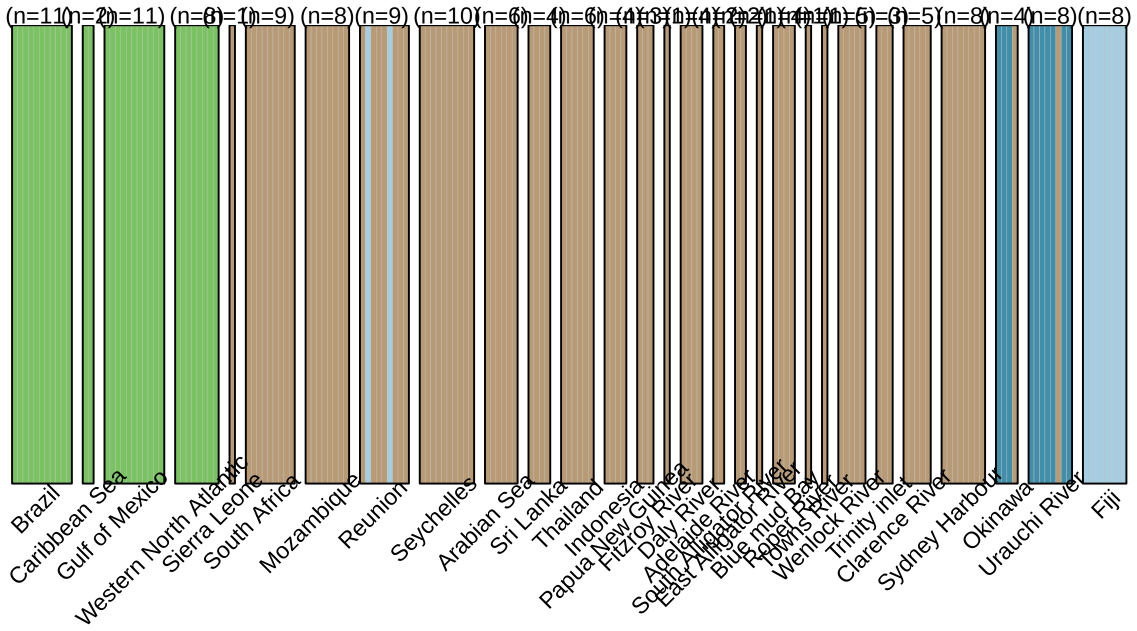


## DAPC: all Bull Sharks − subset Australia for K=5 & PC=15

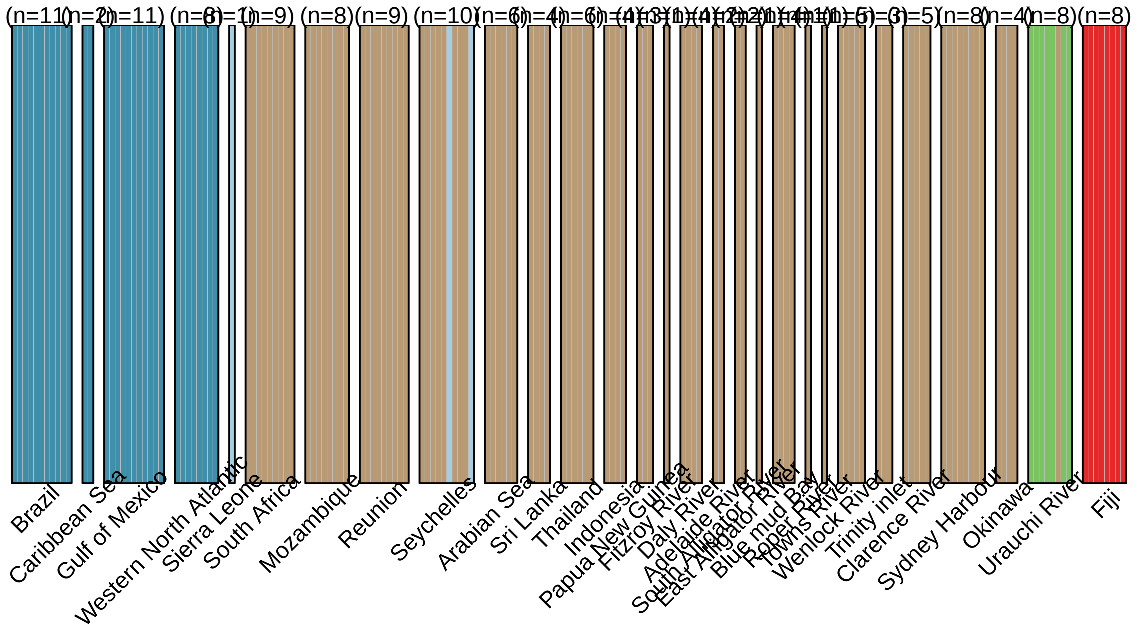

## DAPC: all Bull Sharks − subset Australia for K=6 & PC=15

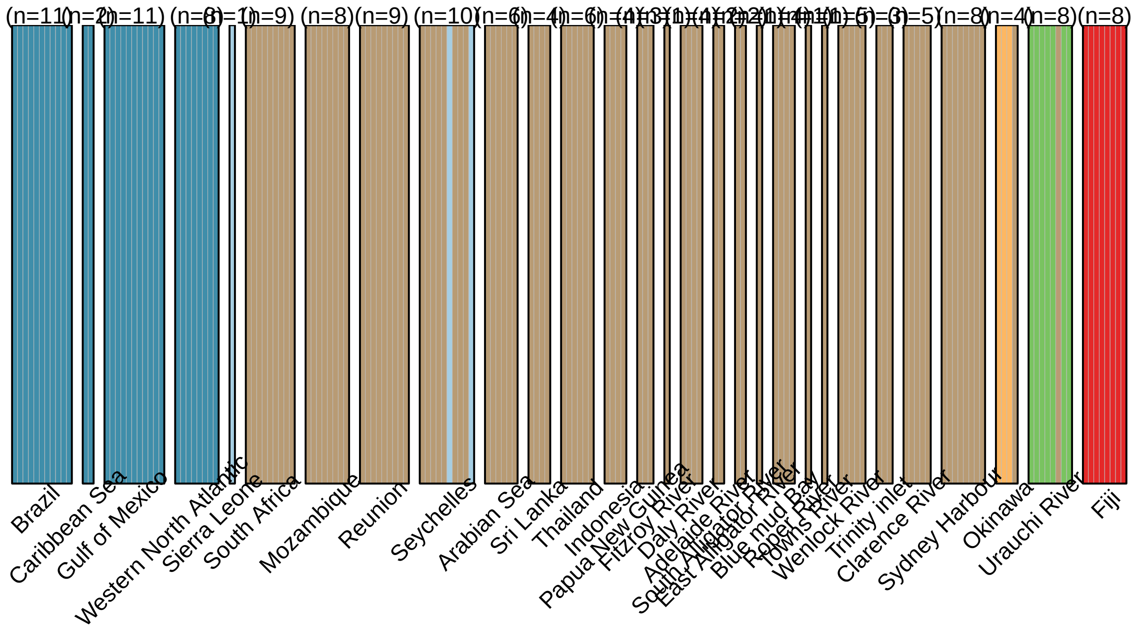


## DAPC: all Bull Sharks − subset Australia for K=7 & PC=15

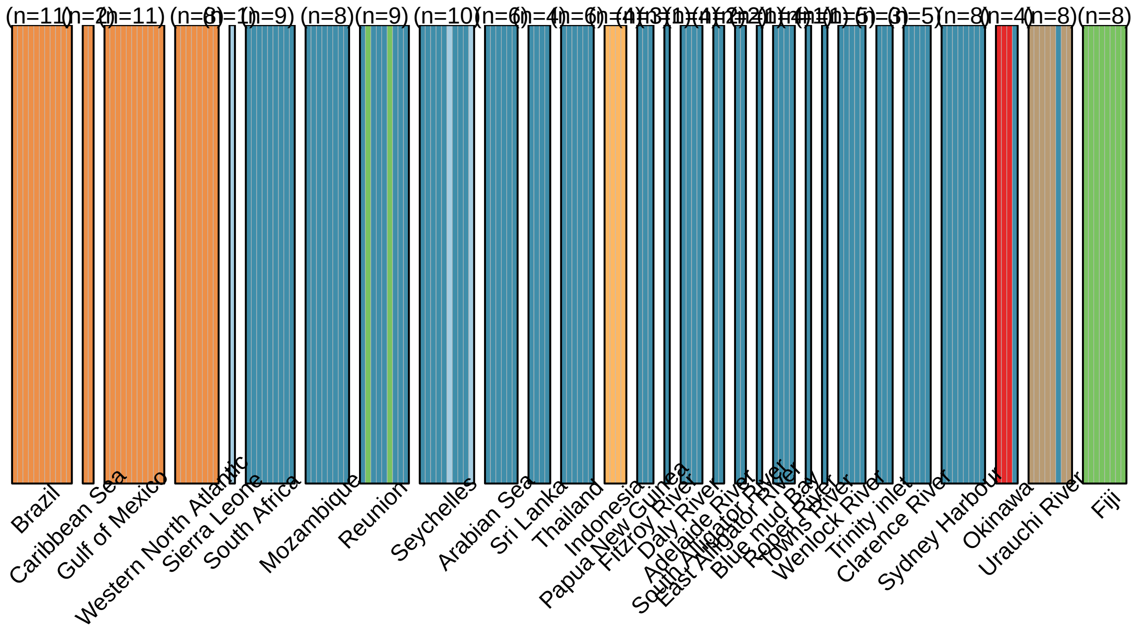

## DAPC: all Bull Sharks − subset Australia for K=8 & PC=15

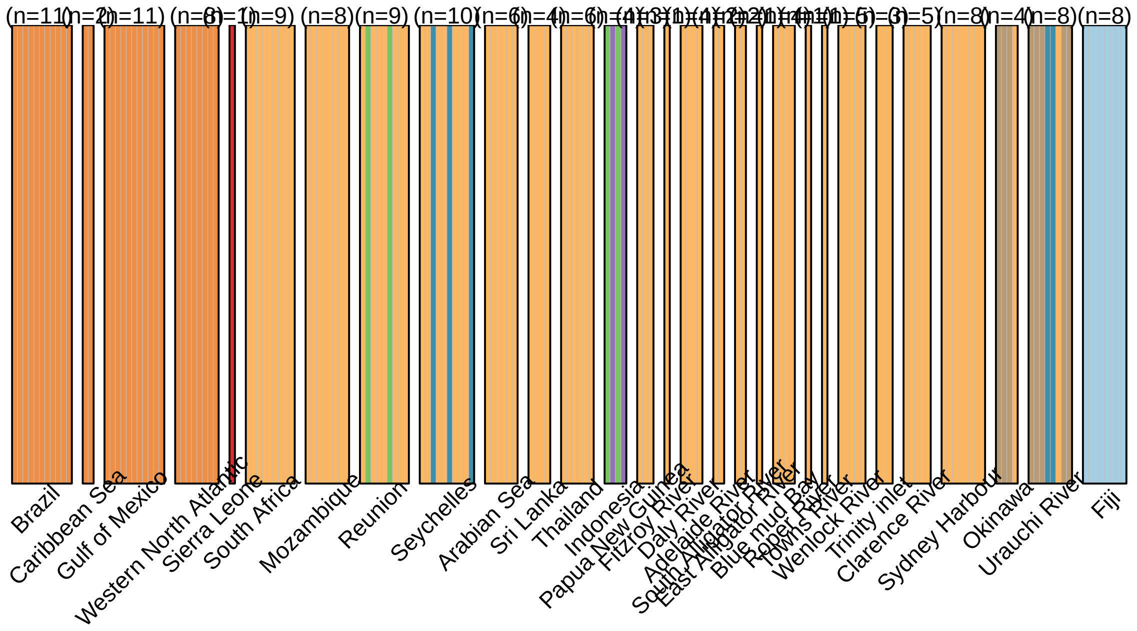


## DAPC: all Bull Sharks − subset Australia for K=9 & PC=15

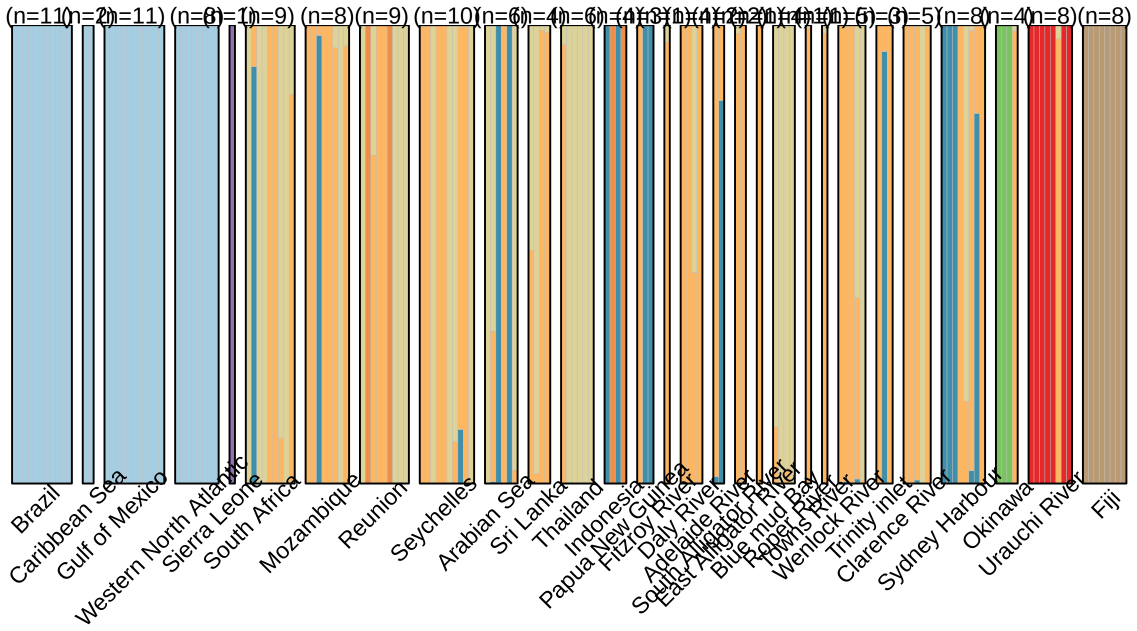

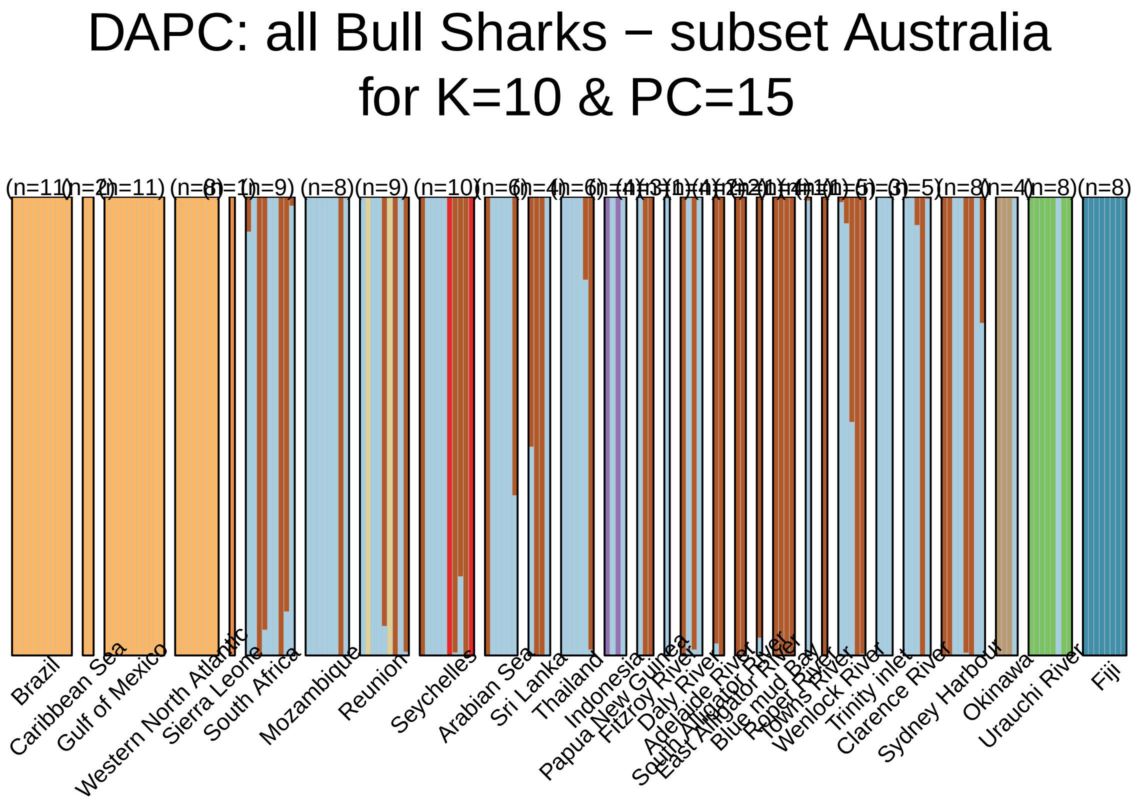


### 5.9.4 DAPC scatterplot

```r
load("DArTseq_ALL_DAPC2.Rdata")

for (K in 3:10) {
  dapc <- get(dapc.all.object.names[K - 1])
  if (K == 2) {
    posi.leg <- "bottomright"
  } else {posi.leg <- "bottomleft"}
  dapc.plot <- ade4::scatter(dapc, grp = pop(BS.dartseq.gl), cex = 2,
                             legend = TRUE, col = colours.29,clabel = FALSE,
                             posi.leg = posi.leg, scree.pca = FALSE,
                             posi.pca = "topleft", cleg = 0.5, xax = 1,
                             yax = 2, inset.solid = 0.3)
  dev.print(
    device = png,
    file = paste0("DArTseq_ALL_DAPC_scatterplot_K", K, ".png"),
    width = 30,
    height = 15,
    units = "cm",
    res = 300
  )
}
```

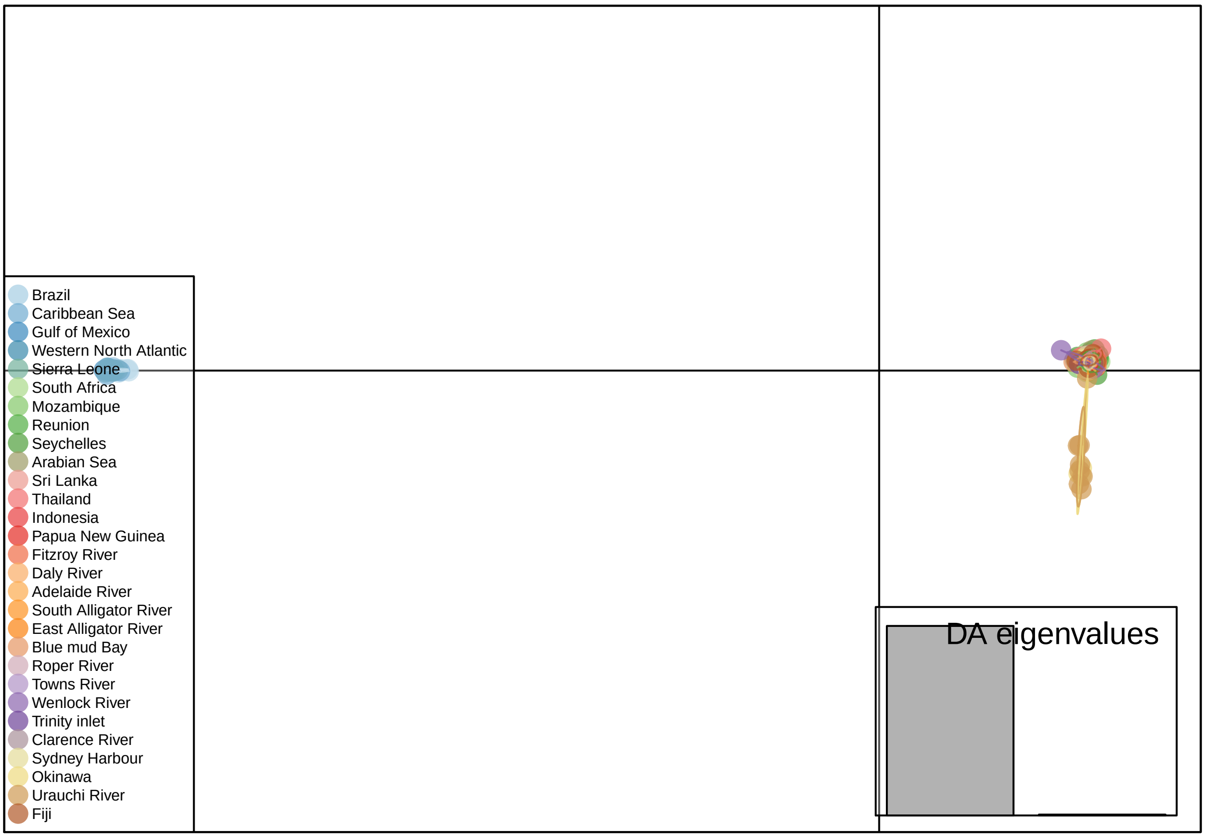
Brazil
Caribbean Sea
Gulf of Mexico
Western North Atlantic
Sierra Leone
South Africa
Mozambique
Reunion
Seychelles
Arabian Sea
Sri Lanka
Thailand
Indonesia
Papua New Guinea
Fitzroy River
Daly River
Adelaide River
South Alligator River
East Alligator River
Blue mud Bay
Roper River
Towns River
Wenlock River
Trinity inlet
Clarence River
Sydney Harbour
Okinawa
Urauchi River
Fiji
DA eigenvalues

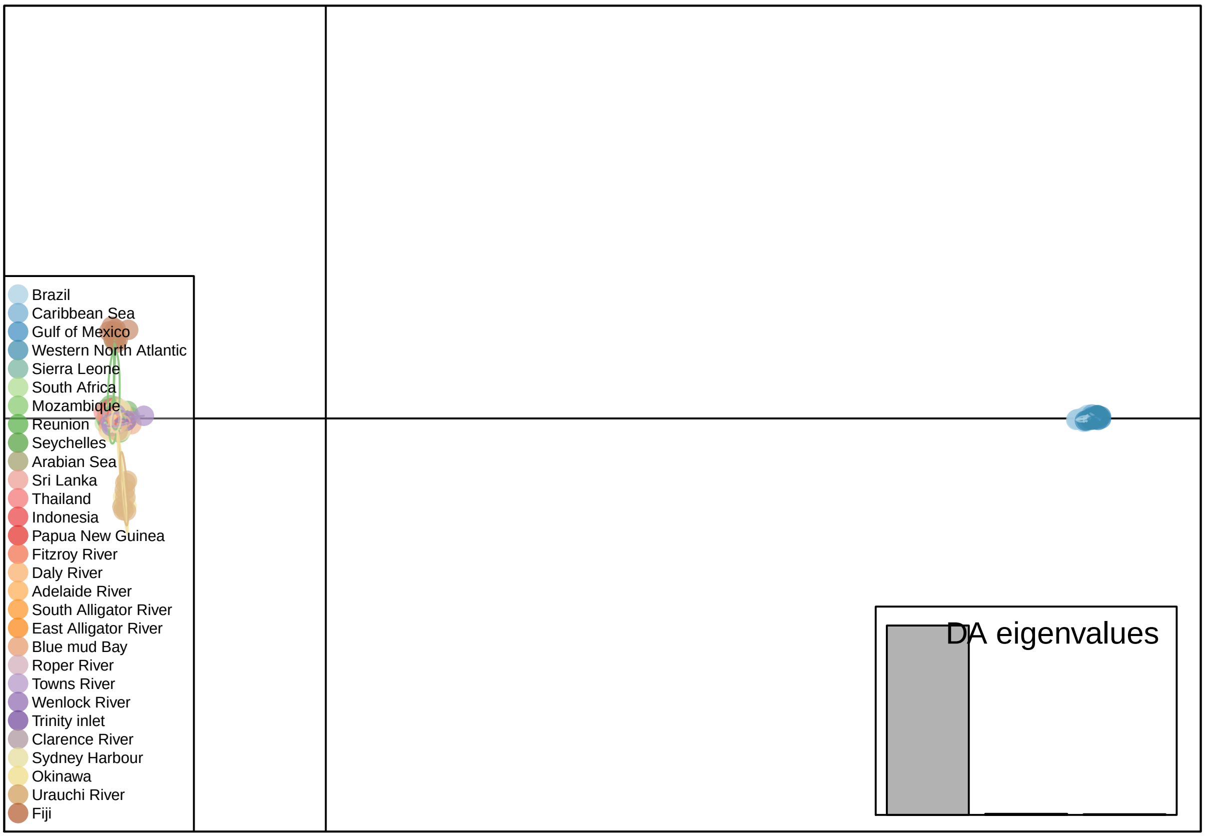
Brazil
Caribbean Sea
Gulf of Mexico
Western North Atlantic
Sierra Leone
South Africa
Mozambique
Reunion
Seychelles
Arabian Sea
Sri Lanka
Thailand
Indonesia
Papua New Guinea
Fitzroy River
Daly River
Adelaide River
South Alligator River
East Alligator River
Blue mud Bay
Roper River
Towns River
Wenlock River
Trinity inlet
Clarence River
Sydney Harbour
Okinawa
Urauchi River
Fiji
DA eigenvalues

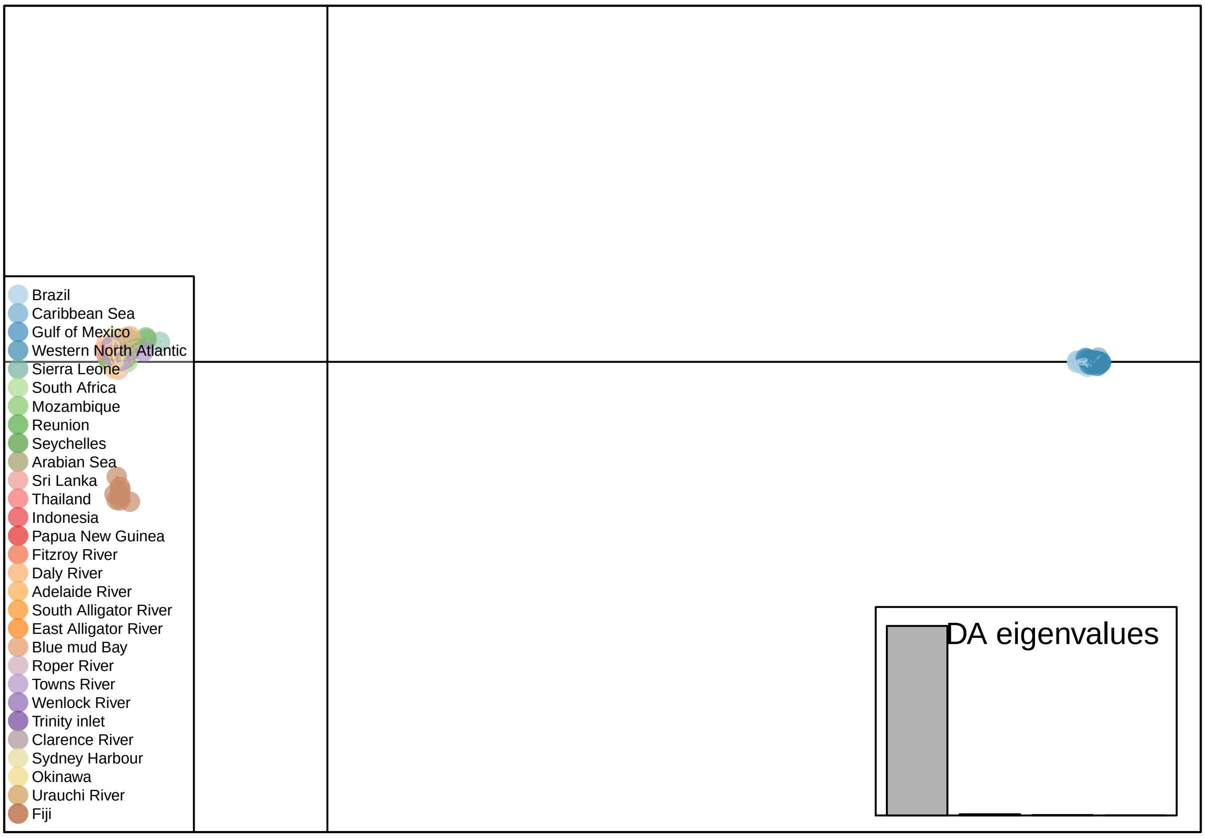
Brazil
Caribbean Sea
Gulf of Mexico
Western North Atlantic
Sierra Leone
South Africa
Mozambique
Reunion
Seychelles
Arabian Sea
Sri Lanka
Thailand
Indonesia
Papua New Guinea
Fitzroy River
Daly River
Adelaide River
South Alligator River
East Alligator River
Blue mud Bay
Roper River
Towns River
Wenlock River
Trinity inlet
Clarence River
Sydney Harbour
Okinawa
Urauchi River
Fiji
DA eigenvalues

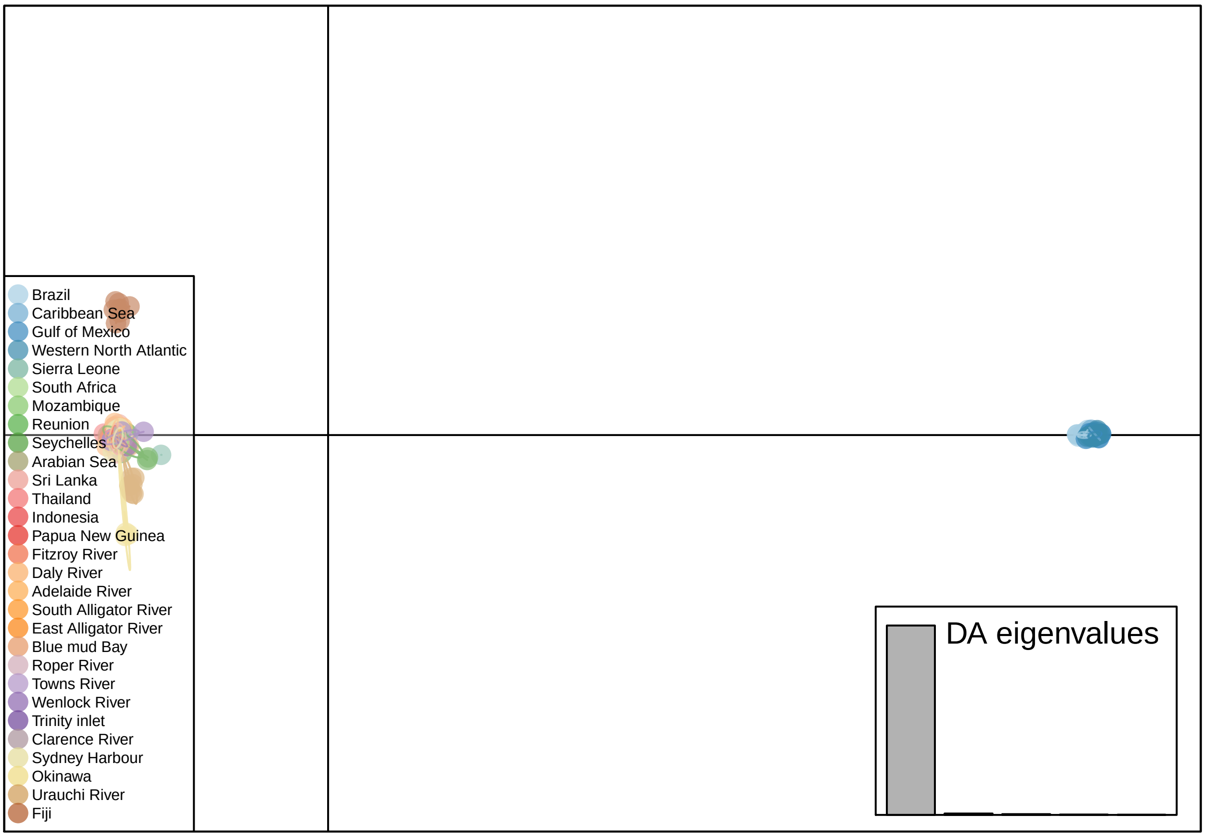
Brazil
Caribbean Sea
Gulf of Mexico
Western North Atlantic
Sierra Leone
South Africa
Mozambique
Reunion
Seychelles
Arabian Sea
Sri Lanka
Thailand
Indonesia
Papua New Guinea
Fitzroy River
Daly River
Adelaide River
South Alligator River
East Alligator River
Blue mud Bay
Roper River
Towns River
Wenlock River
Trinity inlet
Clarence River
Sydney Harbour
Okinawa
Urauchi River
Fiji
DA eigenvalues

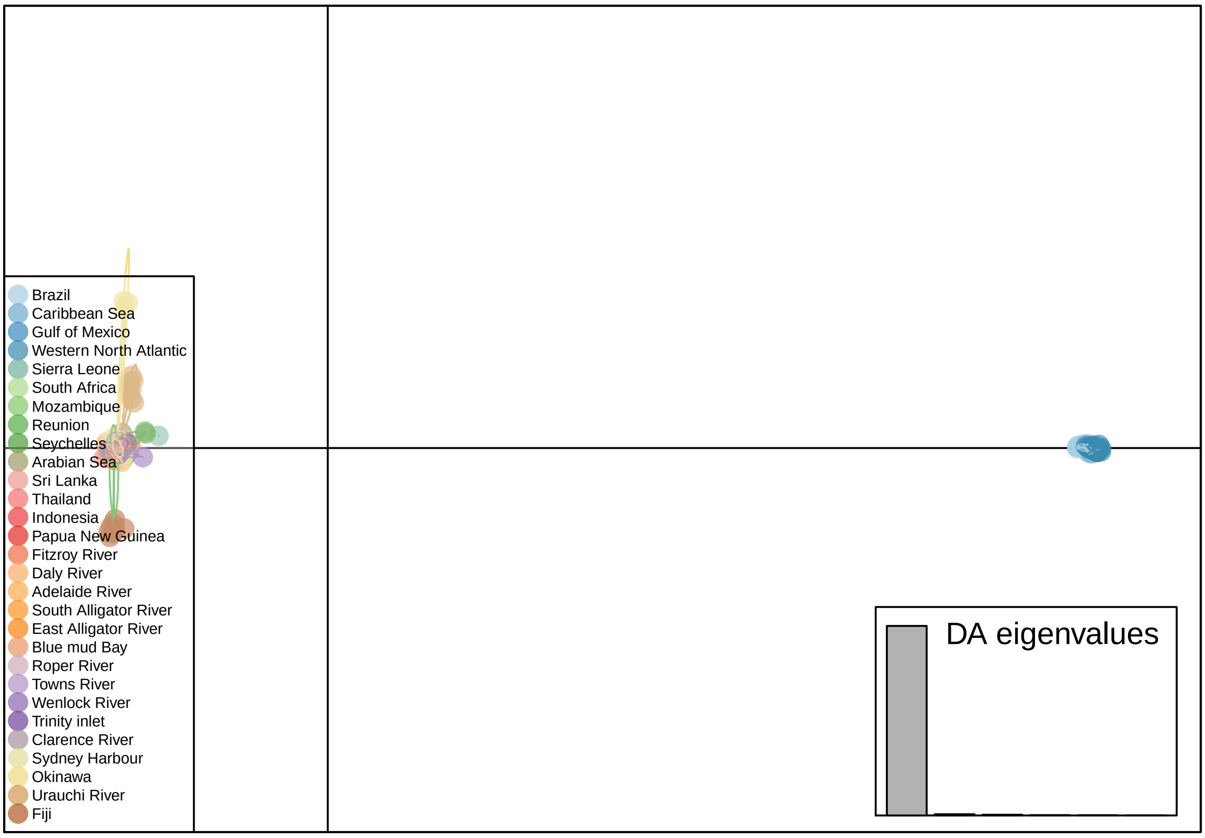
Brazil
Caribbean Sea
Gulf of Mexico
Western North Atlantic
Sierra Leone
South Africa
Mozambique
Reunion
Seychelles
Arabian Sea
Sri Lanka
Thailand
Indonesia
Papua New Guinea
Fitzroy River
Daly River
Adelaide River
South Alligator River
East Alligator River
Blue mud Bay
Roper River
Towns River
Wenlock River
Trinity inlet
Clarence River
Sydney Harbour
Okinawa
Urauchi River
Fiji
DA eigenvalues

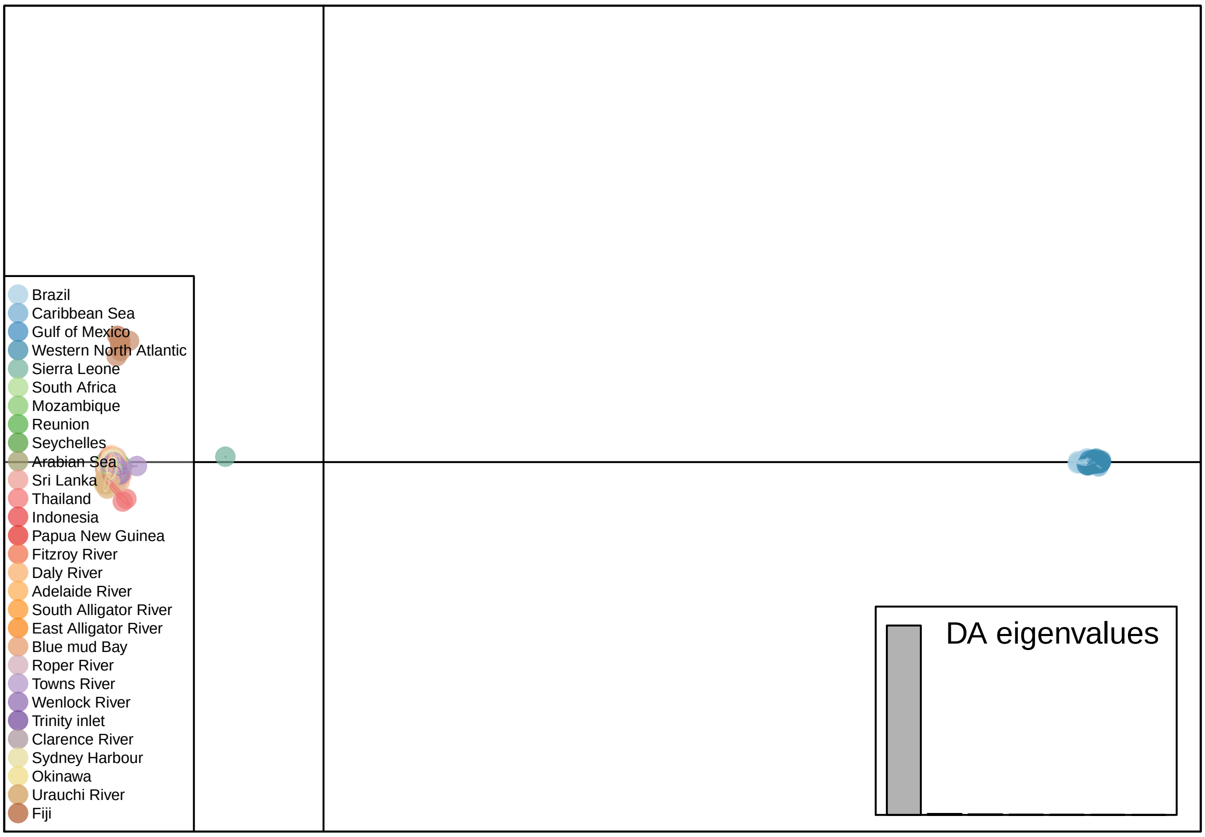
Brazil
Caribbean Sea
Gulf of Mexico
Western North Atlantic
Sierra Leone
South Africa
Mozambique
Reunion
Seychelles
Arabian Sea
Sri Lanka
Thailand
Indonesia
Papua New Guinea
Fitzroy River
Daly River
Adelaide River
South Alligator River
East Alligator River
Blue mud Bay
Roper River
Towns River
Wenlock River
Trinity inlet
Clarence River
Sydney Harbour
Okinawa
Urauchi River
Fiji
DA eigenvalues

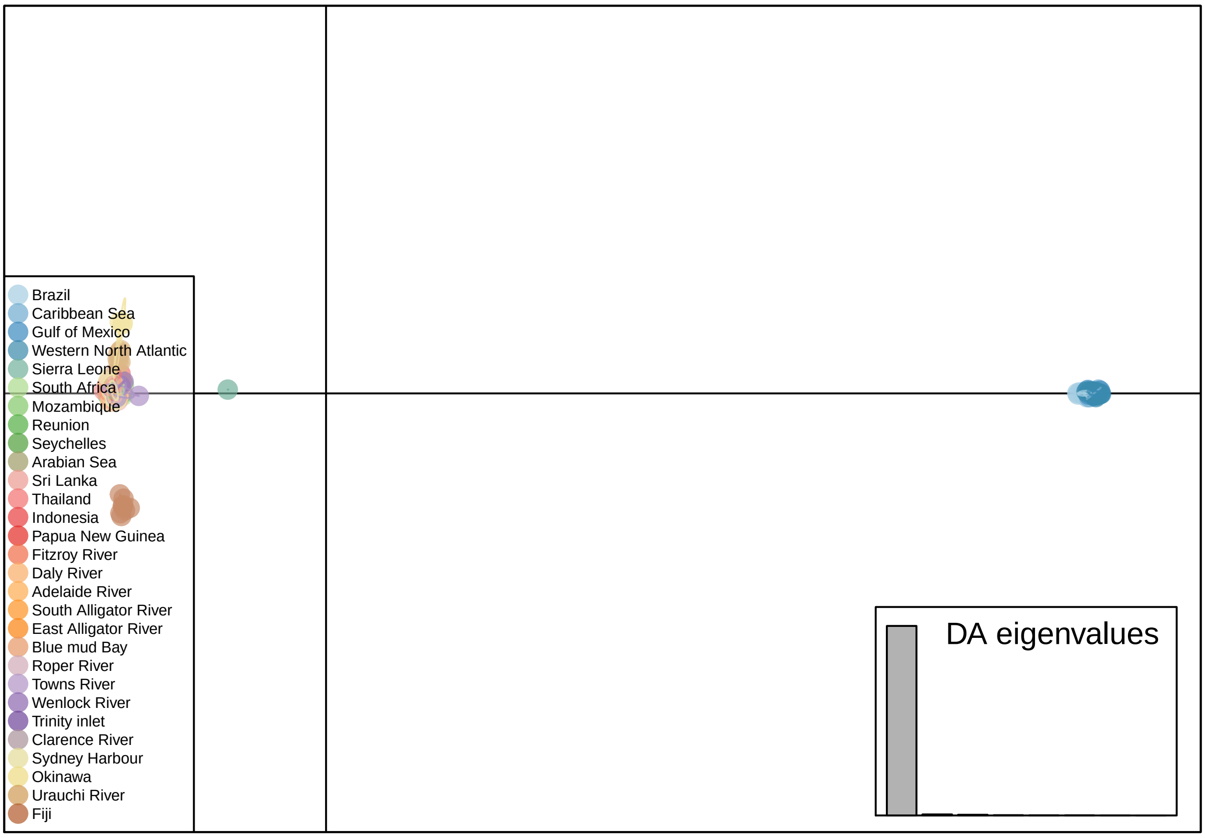


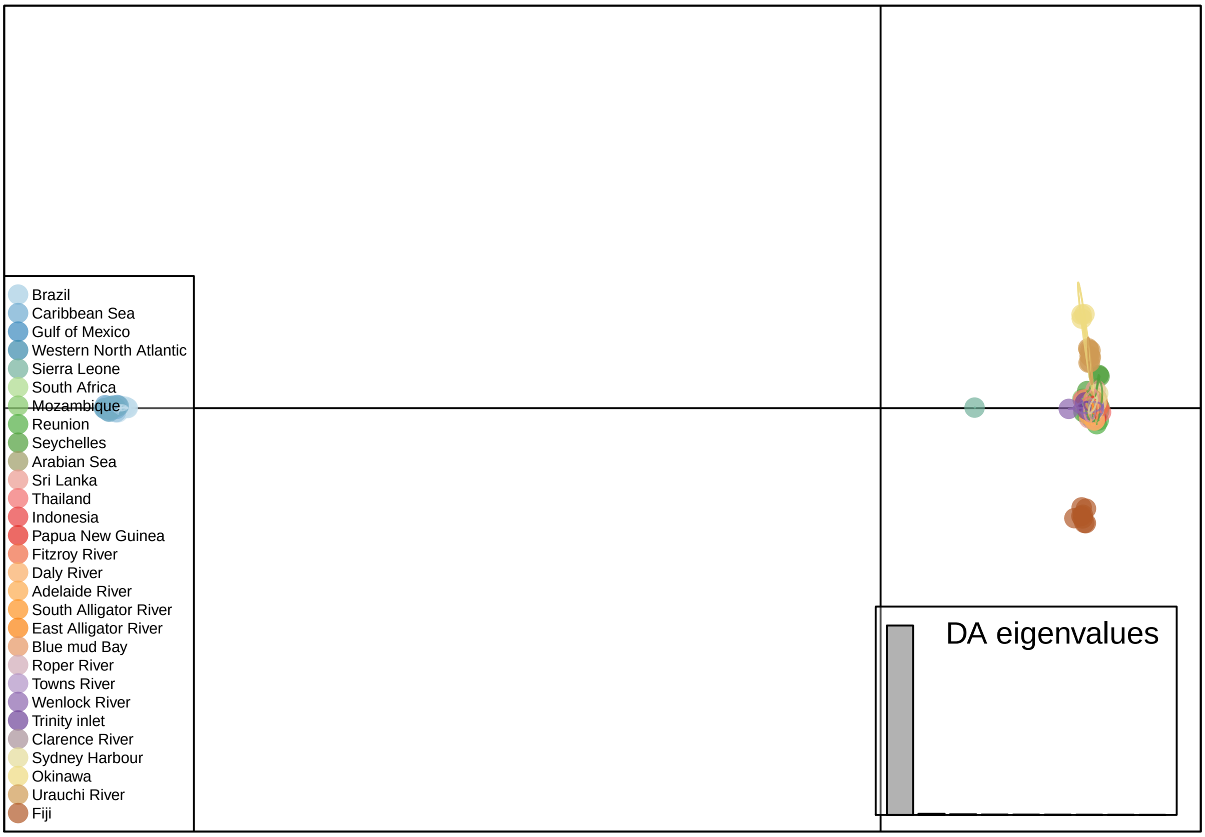


```r
for (K in 2:10) {
  colour <- colours.prior[1:K]
  dapc <- get(dapc.all.object.names[K - 1])
  dapc.plot <- ade4::scatter(dapc, cex = 2, legend = TRUE, col = colour,
                             clabel = FALSE, posi.leg = "bottomleft",
```

```
                                 scree.pca = FALSE, posi.pca = "topleft",
                                 cleg = 0.75, xax = 1, yax = 2, inset.solid = 0.3)
  dev.print(
    device = png,
    file = paste0("DArTseq_ALL_DAPC_scatterplot_pergroups_K",
                  K,".png"),
    width = 30,
    height = 15,
    units = "cm",
    res = 300
  )
}
```

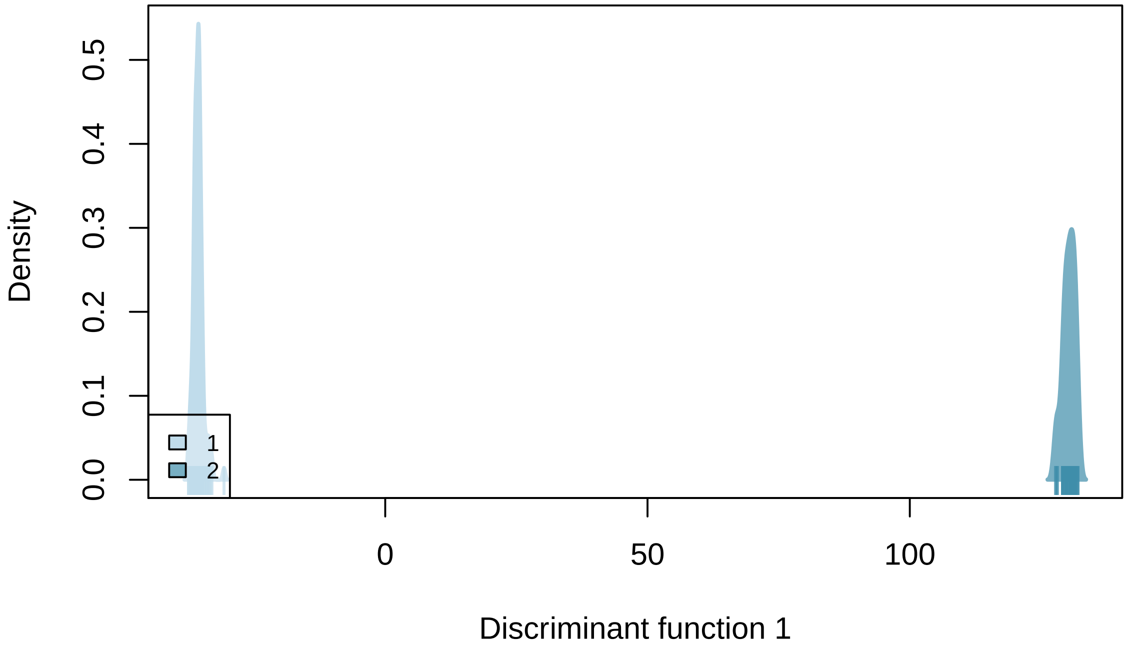

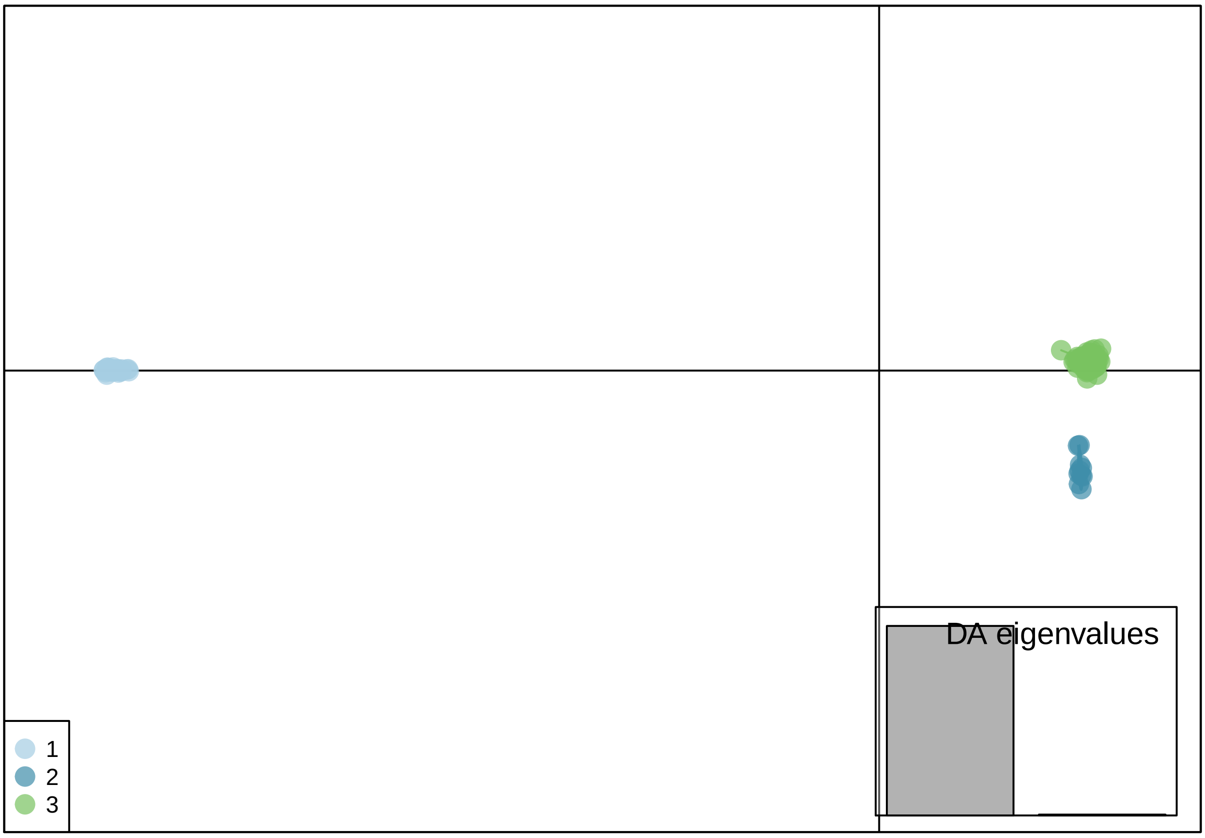
DA eigenvalues
1
2
3

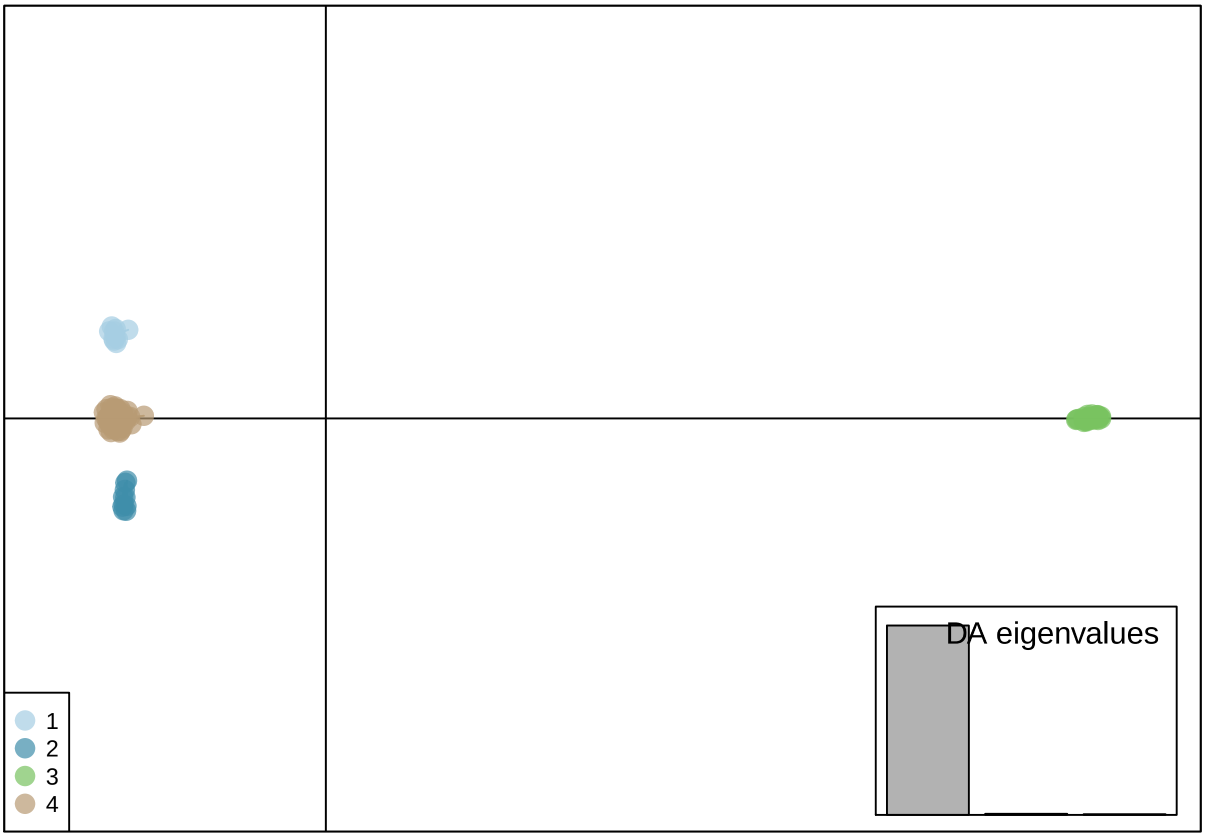
DA eigenvalues
1
2
3
4

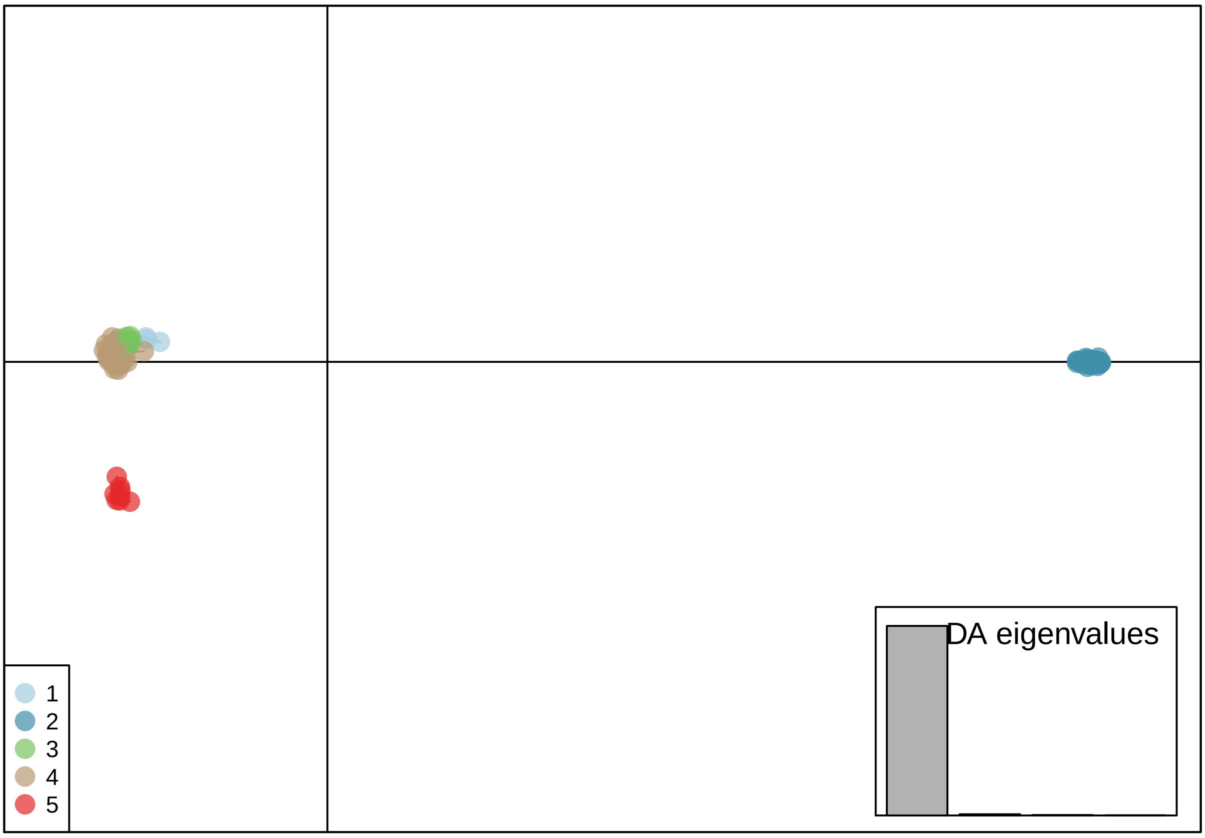
DA eigenvalues
1
2
3
4
5

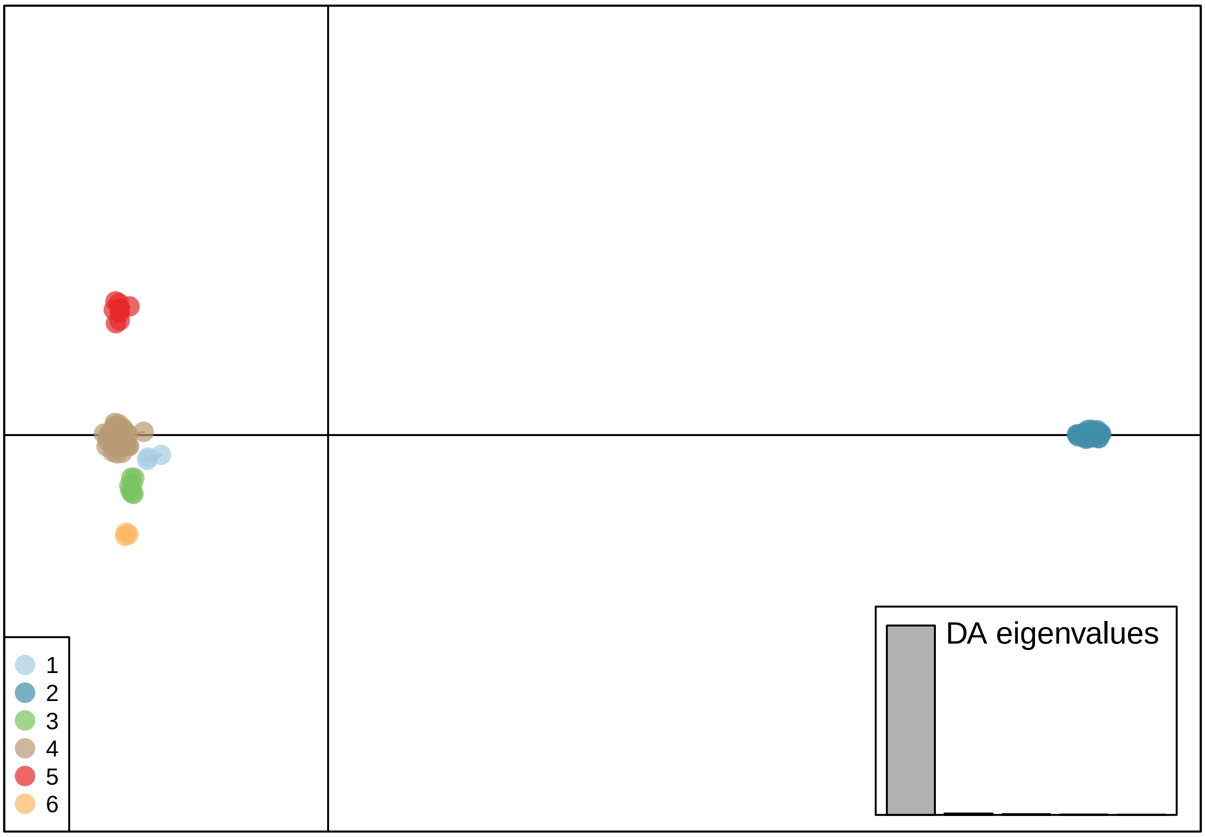
DA eigenvalues
1
2
3
4
5
6

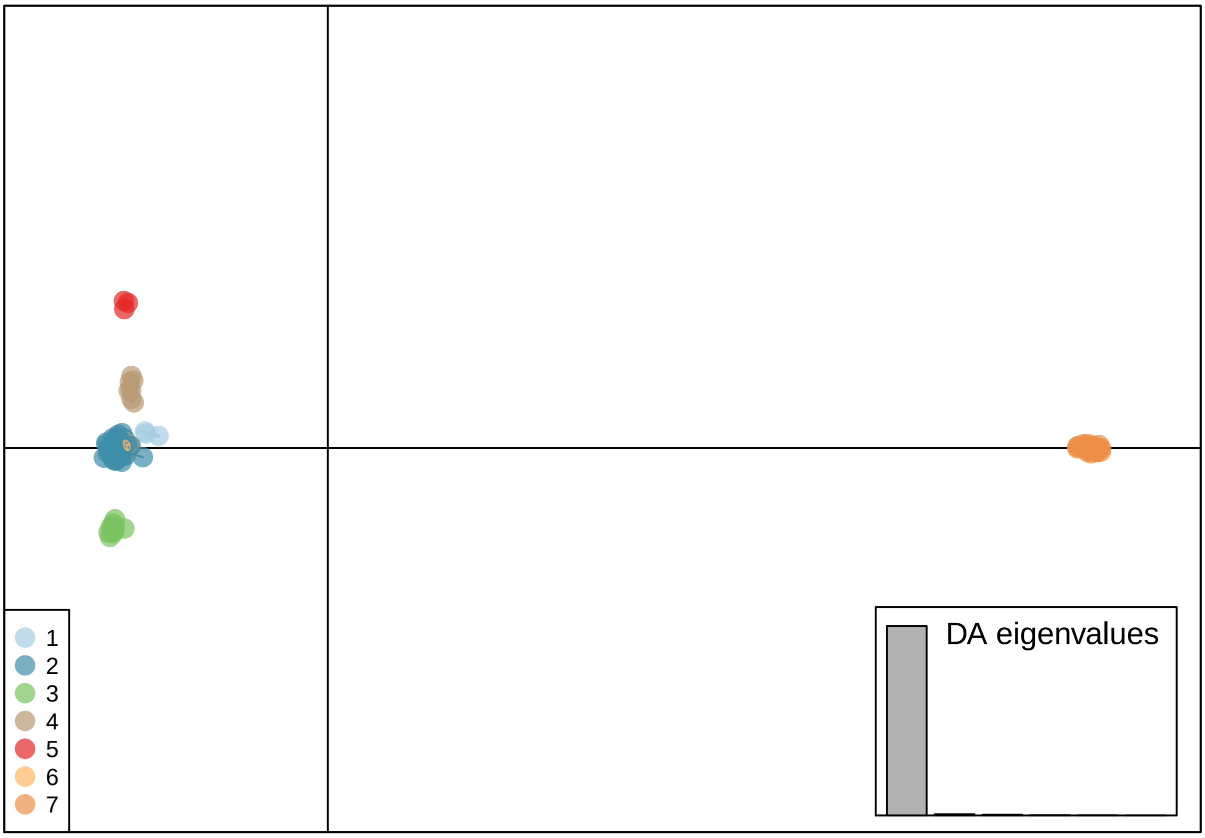
1
2
3
4
5
6
7
DA eigenvalues

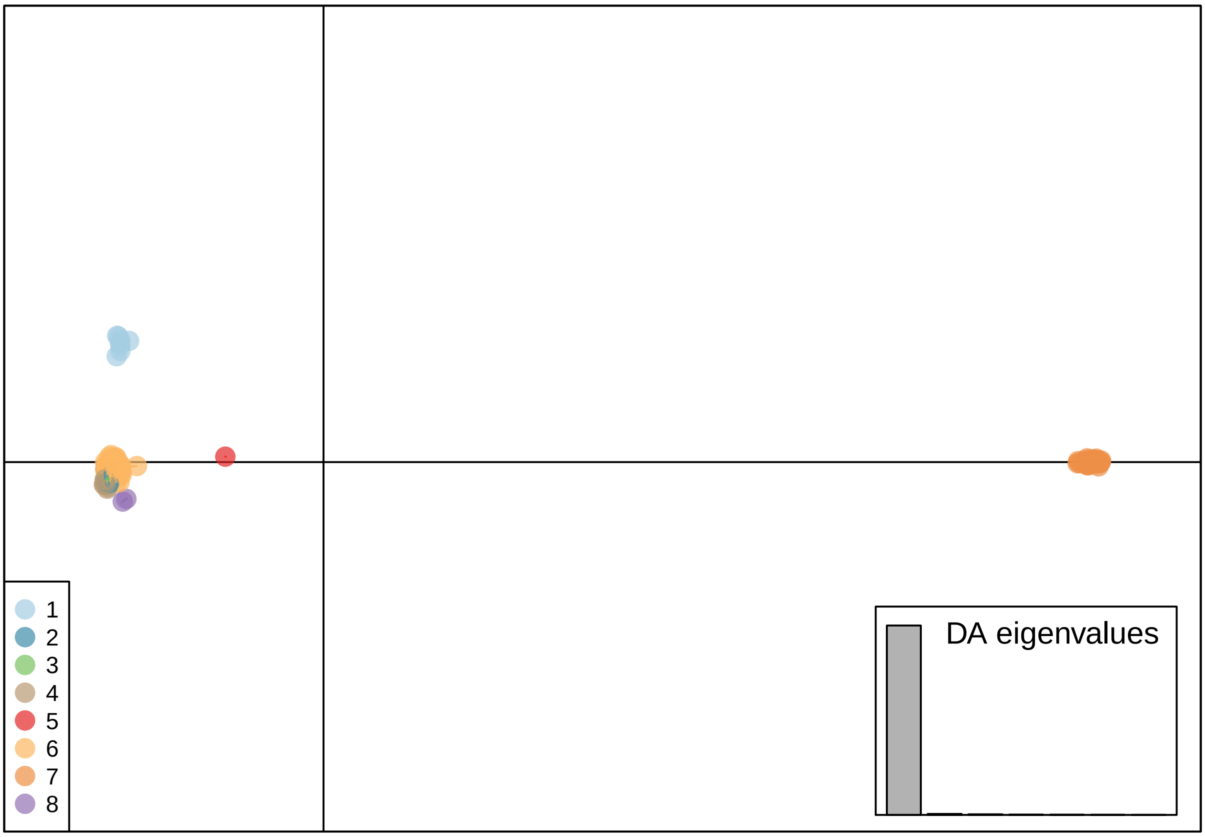
1
2
3
4
5
6
7
8
DA eigenvalues

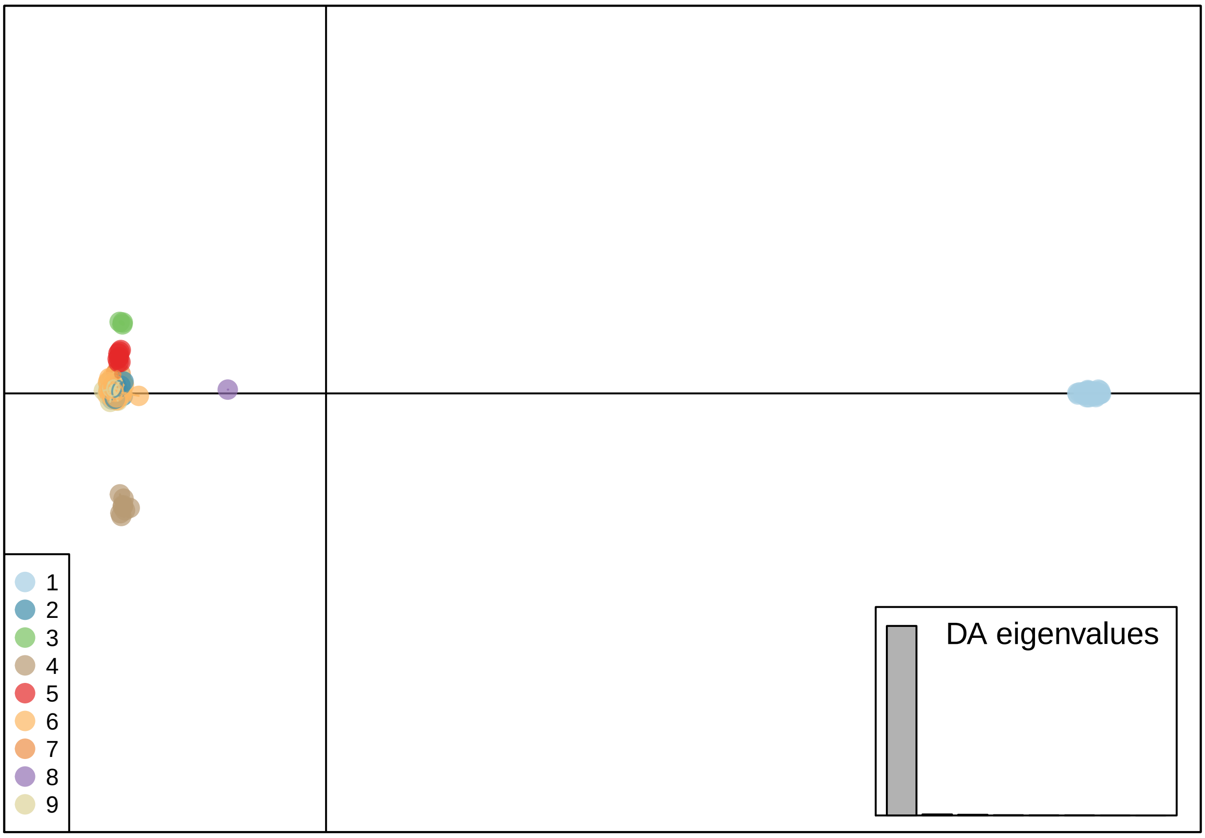


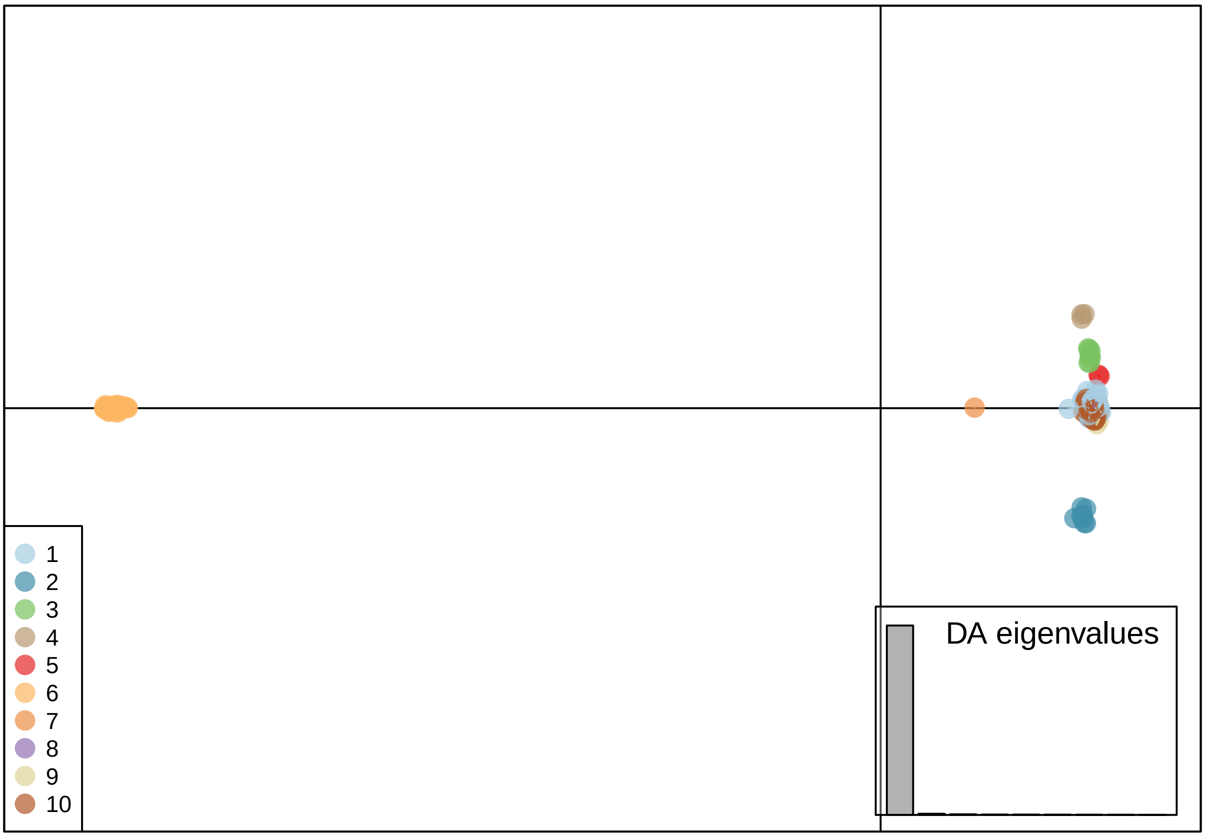


### 5.9.5 DAPC scatterplot - FLO

This code will *colour* individuals according to *sampling location* and **group** individuals according to DAPC **posterior membership**.

```r
for (K in 3:10) {
  dapc <- get(dapc.all.object.names[K - 1])
  x <- dapc$ind.coord[,1]
  y <- dapc$ind.coord[,2]
  group <- paste("Group", as.numeric(dapc$assign))
  pop <- BS.dartseq.gl$pop
  df <- data.frame(x, y, group, pop)
  colnames(df) <- c("x","y","group","pop")
  gg <- merge(df,aggregate(cbind(mean.x = x,mean.y = y)~group,df,mean),
              by = "group")

  plot <- ggplot2::ggplot(gg, ggplot2::aes(x,y,color = pop)) +
    ggplot2::geom_point(size = 5) +
    ggplot2::geom_point(ggplot2::aes(x = mean.x, y = mean.y),size = 5, shape = 17,
                        color = "black") +
    ggplot2::geom_segment(ggplot2::aes(x = mean.x, y = mean.y, xend = x, yend = y)) +
    ggplot2::scale_colour_manual(values = colours.29) +
    ggplot2::scale_fill_manual(values = colours.29) +
    ggplot2::geom_hline(yintercept = 0) +
    ggplot2::geom_vline(xintercept = 0) +
    ggplot2::labs(subtitle = "",
        y = "PC2",
        x = "PC1",
        title = paste0("K=",K," & PC=",PC, sep = ""),
        caption = "") +
    ggplot2::theme_minimal() +
    ggplot2::theme(
      axis.text = ggplot2::element_text(size = 5),
      axis.title.x = ggplot2::element_text(size = 10),
      axis.title.y = ggplot2::element_text(size = 10),
      legend.text = ggplot2::element_text(size = 7))
  print(plot)
  ggplot2::ggsave(plot, filename = paste0("DArTseq_ALL_DAPC_scatterplot2_K", K, ".png"),
                  width = 30, height = 15,units = "cm")
}
```

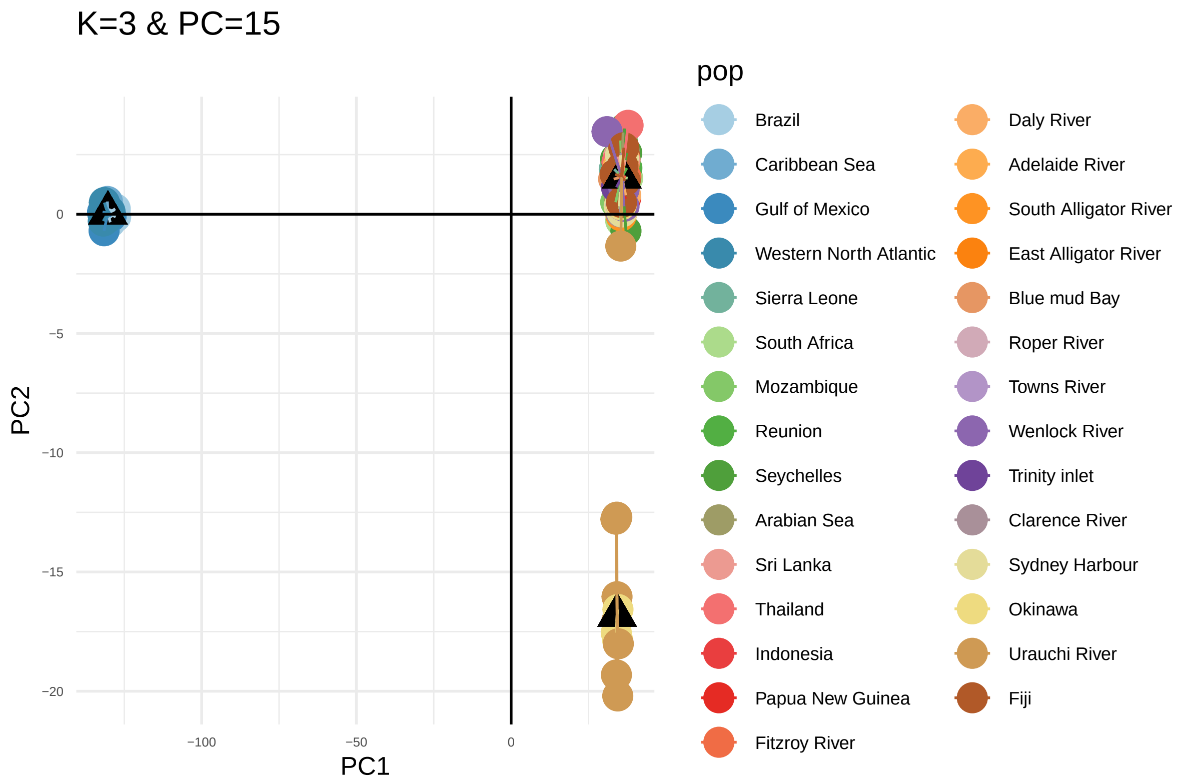
K=3 & PC=15
pop
Brazil
Caribbean Sea
Gulf of Mexico
Western North Atlantic
Sierra Leone
South Africa
Mozambique
Reunion
Seychelles
Arabian Sea
Sri Lanka
Thailand
Indonesia
Papua New Guinea
Fitzroy River
Daly River
Adelaide River
South Alligator River
East Alligator River
Blue mud Bay
Roper River
Towns River
Wenlock River
Trinity inlet
Clarence River
Sydney Harbour
Okinawa
Urauchi River
Fiji
PC2
PC1
0
−5
−10
−15
−20
−100
−50
0

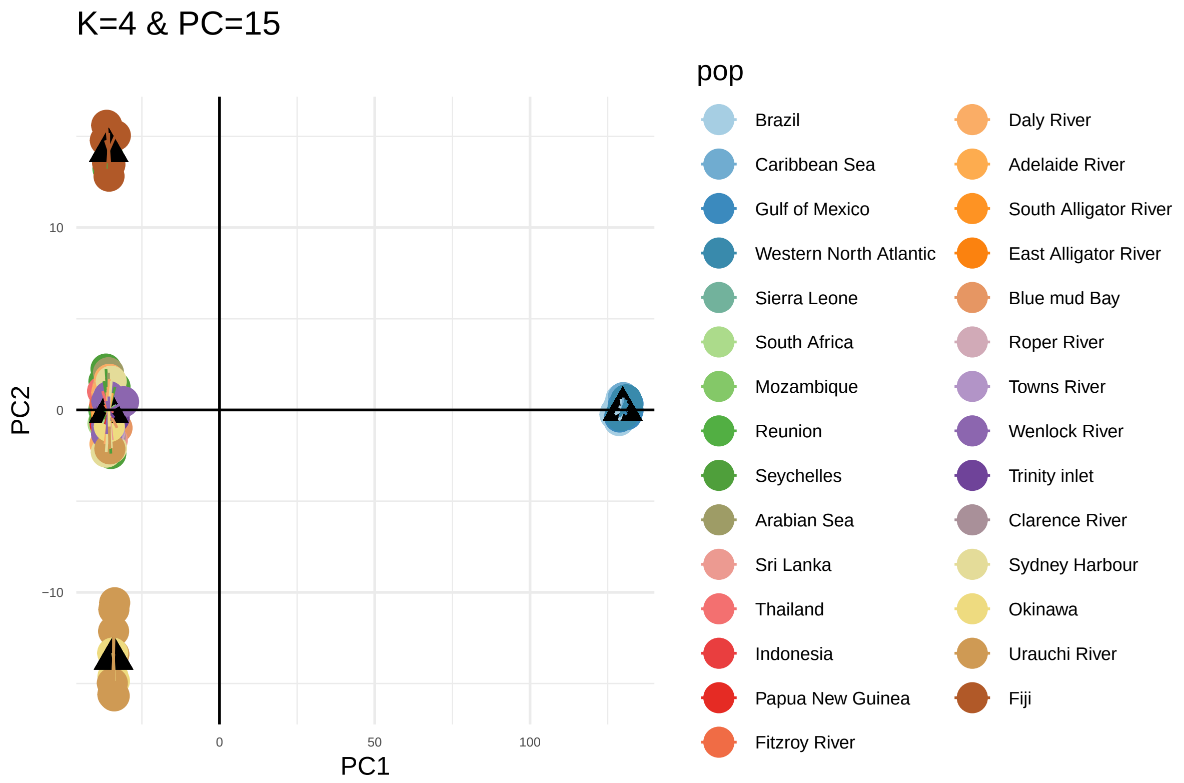
K=4 & PC=15
pop
Brazil
Caribbean Sea
Gulf of Mexico
Western North Atlantic
Sierra Leone
South Africa
Mozambique
Reunion
Seychelles
Arabian Sea
Sri Lanka
Thailand
Indonesia
Papua New Guinea
Fitzroy River
Daly River
Adelaide River
South Alligator River
East Alligator River
Blue mud Bay
Roper River
Towns River
Wenlock River
Trinity inlet
Clarence River
Sydney Harbour
Okinawa
Urauchi River
Fiji
PC2
PC1
10
0
−10
0
50
100

## K=5 & PC=15

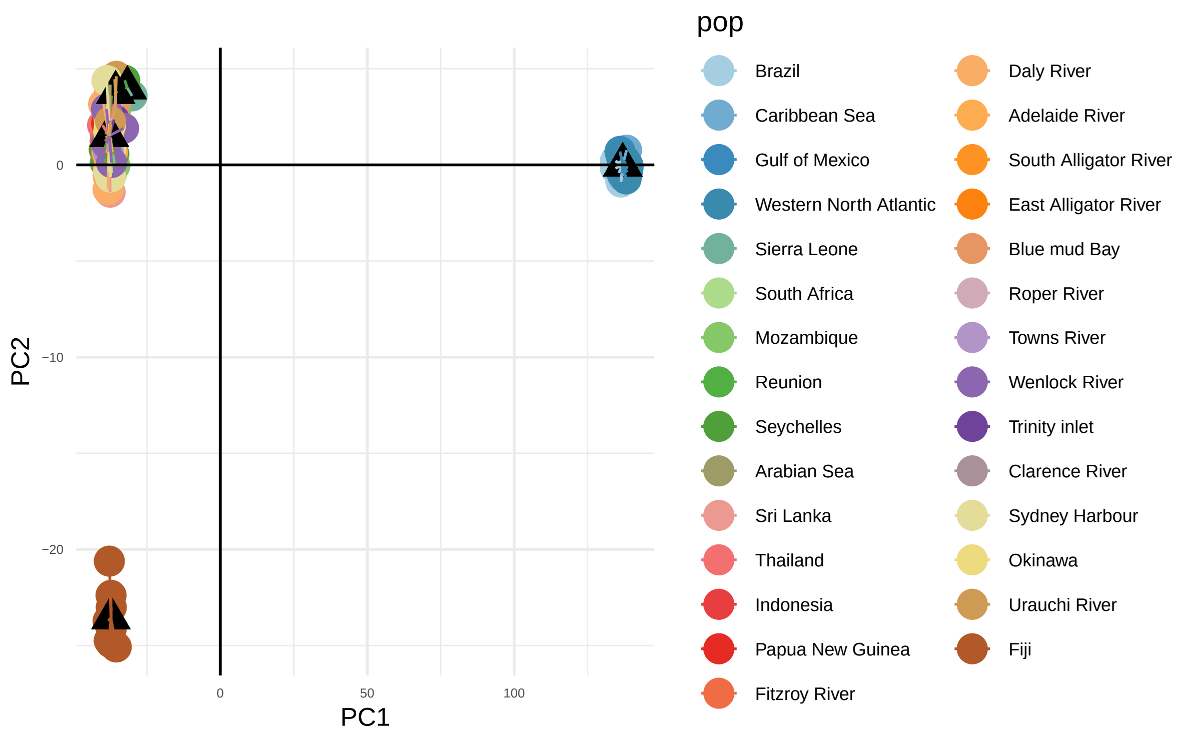


## K=6 & PC=15

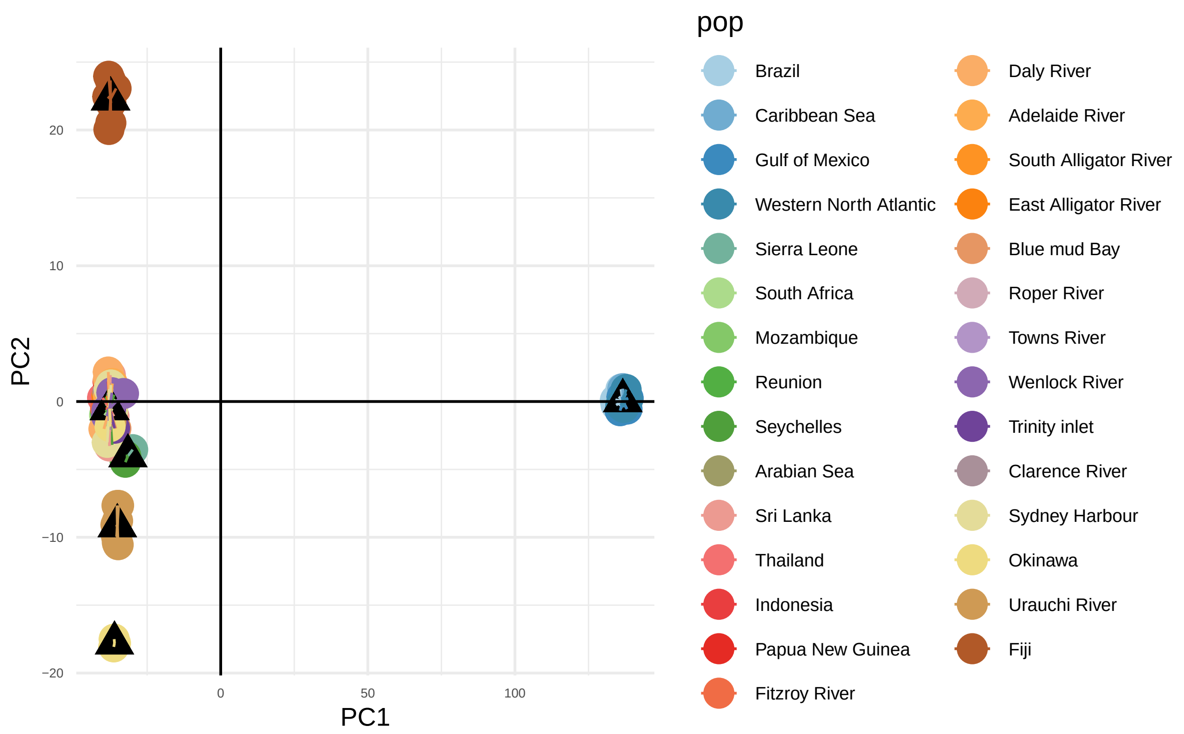

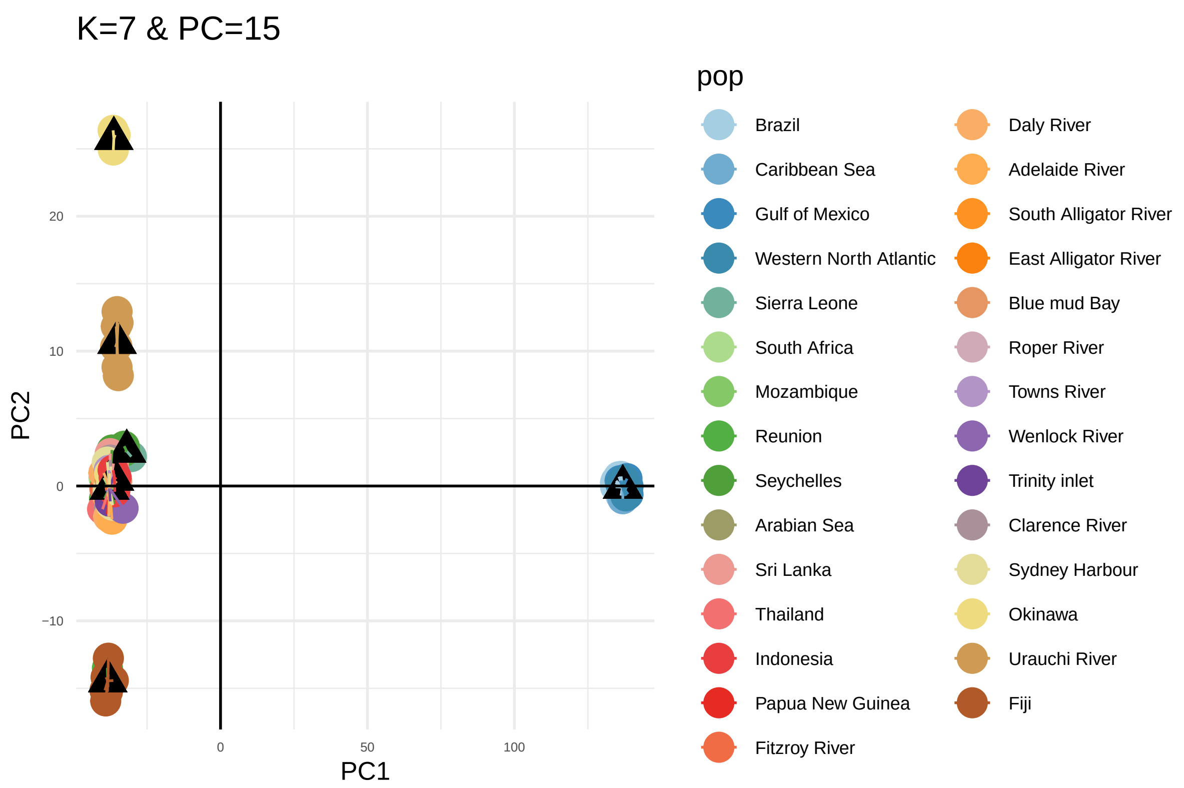
K=7 & PC=15
pop
Brazil
Caribbean Sea
Gulf of Mexico
Western North Atlantic
Sierra Leone
South Africa
Mozambique
Reunion
Seychelles
Arabian Sea
Sri Lanka
Thailand
Indonesia
Papua New Guinea
Fitzroy River
Daly River
Adelaide River
South Alligator River
East Alligator River
Blue mud Bay
Roper River
Towns River
Wenlock River
Trinity inlet
Clarence River
Sydney Harbour
Okinawa
Urauchi River
Fiji
PC2
20
10
0
−10
0
50
100
PC1

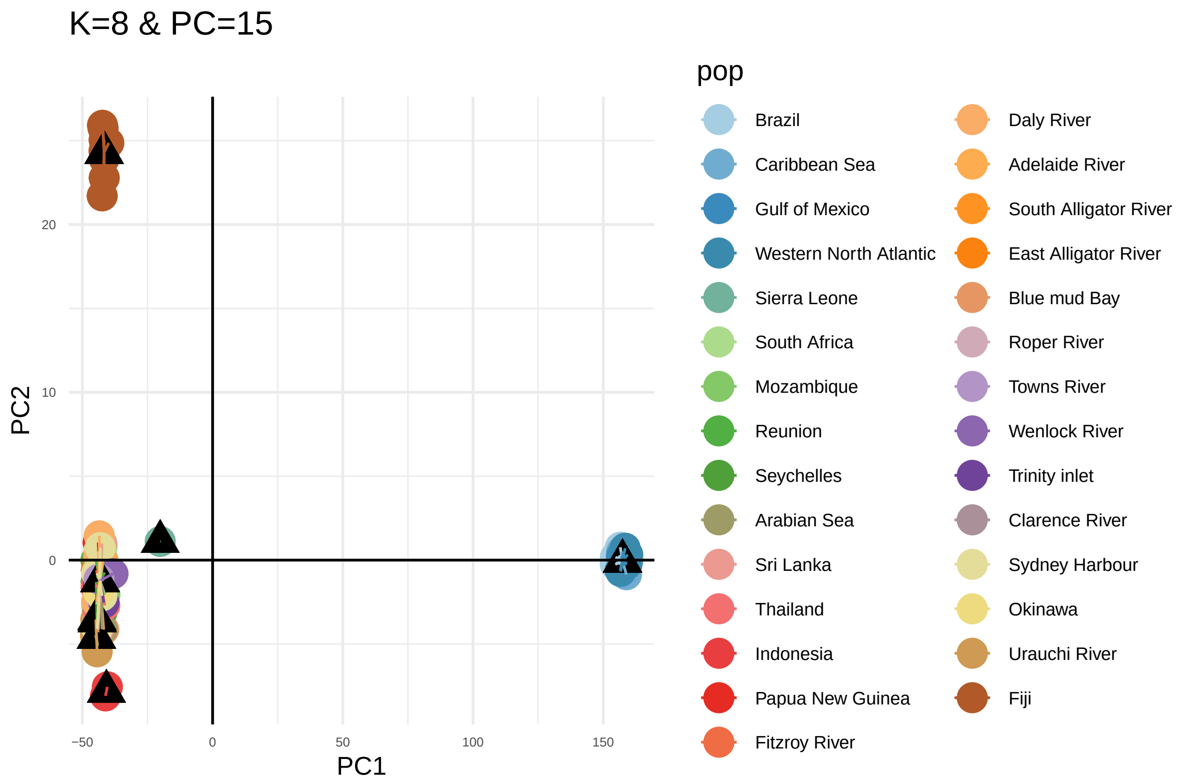
K=8 & PC=15
pop
Brazil
Caribbean Sea
Gulf of Mexico
Western North Atlantic
Sierra Leone
South Africa
Mozambique
Reunion
Seychelles
Arabian Sea
Sri Lanka
Thailand
Indonesia
Papua New Guinea
Fitzroy River
Daly River
Adelaide River
South Alligator River
East Alligator River
Blue mud Bay
Roper River
Towns River
Wenlock River
Trinity inlet
Clarence River
Sydney Harbour
Okinawa
Urauchi River
Fiji
PC2
20
10
0
−50
0
50
100
150
PC1

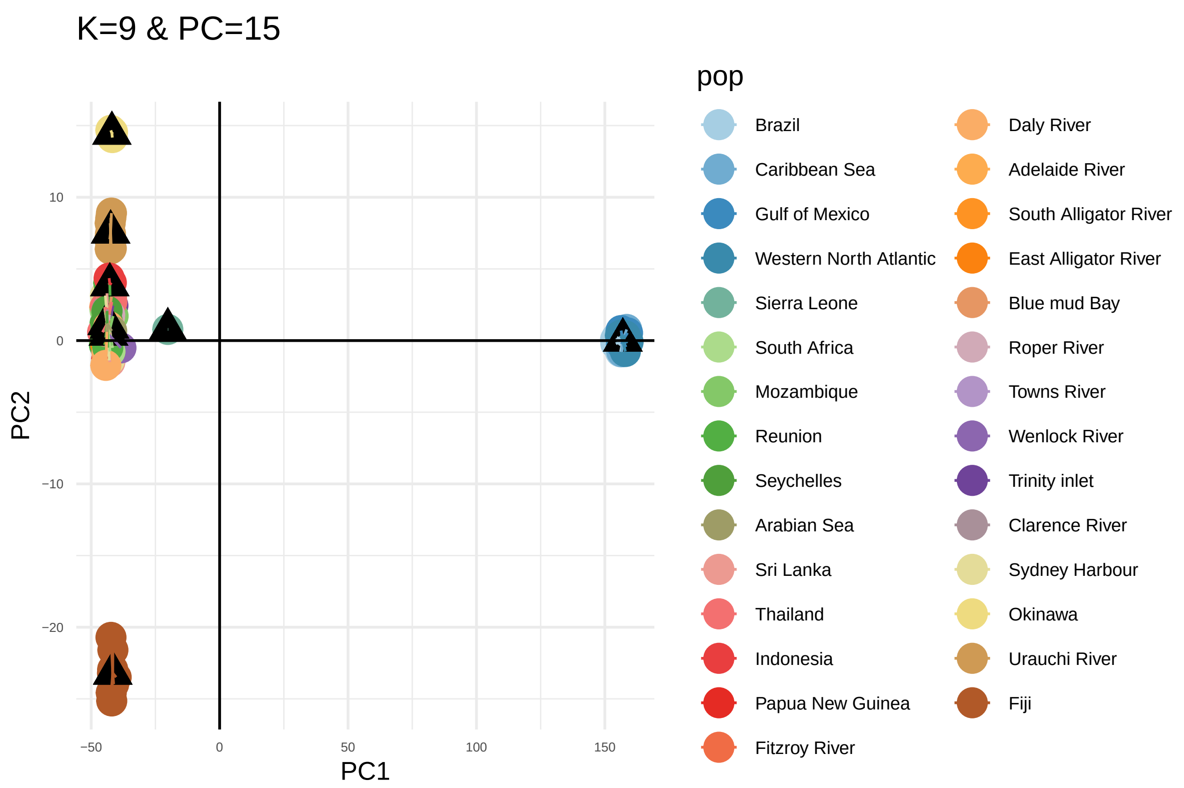
K=9 & PC=15
pop
Brazil
Caribbean Sea
Gulf of Mexico
Western North Atlantic
Sierra Leone
South Africa
Mozambique
Reunion
Seychelles
Arabian Sea
Sri Lanka
Thailand
Indonesia
Papua New Guinea
Fitzroy River
Daly River
Adelaide River
South Alligator River
East Alligator River
Blue mud Bay
Roper River
Towns River
Wenlock River
Trinity inlet
Clarence River
Sydney Harbour
Okinawa
Urauchi River
Fiji
PC1
PC2
−50
0
50
100
150
10
0
−10
−20

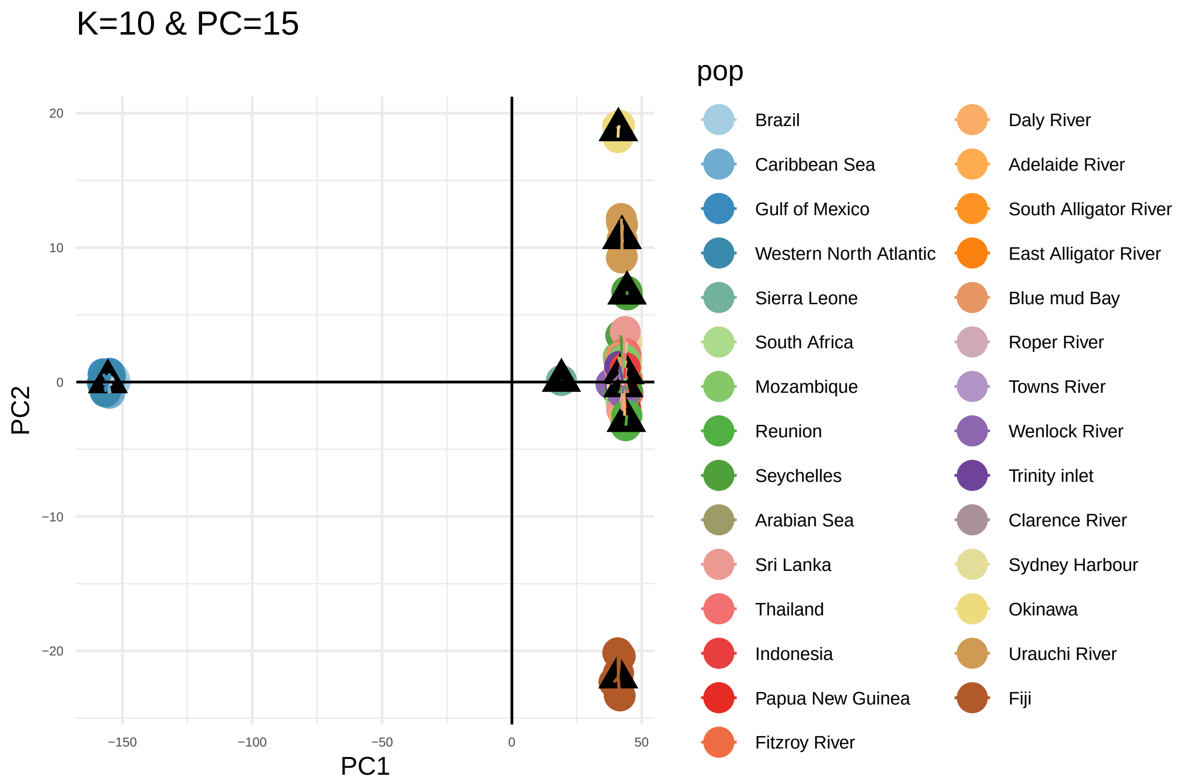
K=10 & PC=15
pop
Brazil
Caribbean Sea
Gulf of Mexico
Western North Atlantic
Sierra Leone
South Africa
Mozambique
Reunion
Seychelles
Arabian Sea
Sri Lanka
Thailand
Indonesia
Papua New Guinea
Fitzroy River
Daly River
Adelaide River
South Alligator River
East Alligator River
Blue mud Bay
Roper River
Towns River
Wenlock River
Trinity inlet
Clarence River
Sydney Harbour
Okinawa
Urauchi River
Fiji
PC1
PC2
−150
−100
−50
0
50
20
10
0
−10
−20

# 6 DArTseq - marker selection

## 6.1 Sex-linked markers

```
load("Sex_results.Rdata")
bull_shark_sex.markers <- bull_shark$sexy.summary[bull_shark$sexy.summary$METHOD %in%
                          c("presence/absence_method-SNP",
                            "presence/absence_method-SILICO",
                            "heterozygosity_method-SNP",
                            "Coverage_method-SNP"),]
dim(bull_shark_sex.markers) #210 SLMs

knitr::kable(
  bull_shark_sex.markers[c(20:25),],
  caption = "210 sex-linked markers with a SNP from DArTseq data for bait design.") %>%
  kableExtra::kable_styling(full_width = FALSE,font_size = 5,
                            latex_options = c("hold_position"))

sex.markers <- bull_shark_sex.markers$CLONE_ID
```

```
## [1] 172   5
```

Table 6: 210 sex-linked markers with a SNP from DArTseq data for bait design.

| SEX_MARKERS | CLONE_ID | METHOD | MARKER_TYPE | SEQUENCE |
|---|---|---|---|---|
| CHROM_1___100203043___49 | 100203043 | Coverage_method-SNP | Homogametic_sex-marker | TGCAGCTTCCCCCCCAGTTGCATTTGCAGGGTGTGCAGCT |
| CHROM_1___100203980___10 | 100203980 | Coverage_method-SNP | Homogametic_sex-marker | TGCAGGAATTYTAAATCCAAAATAATTGCTTTCAGGTGGAA |
| CHROM_1___100204589___28 | 100204589 | Coverage_method-SNP | Homogametic_sex-marker | TGCAGGAGTCACTTTTGCCTCGTGATCARAAACAGTTTGG |
| CHROM_1___100204774___22 | 100204774 | Coverage_method-SNP | Homogametic_sex-marker | TGCAGGATCACAACTGTGTGCAYGTGCCTGTGTGTGTGCG |
| CHROM_1___100204975___7 | 100204975 | Coverage_method-SNP | Homogametic_sex-marker | TGCAGGAYTCATGCAGCCACTGCAACAGAGGTCCGAAGTT |
| CHROM_1___100205908___20 | 100205908 | Coverage_method-SNP | Homogametic_sex-marker | TGCAGGCTGGGTTTGTAACTSATGCACAAGGACTGAGGTC |

## 6.2 Popgen markers

### 6.2.1 Load data

```
load("DArTseq_ALL_objects3.Rdata")
nInd(BS.dartseq.gl) # 149
nLoc(BS.dartseq.gl) # 16989
```

```
## [1] 149
## [1] 16989
```

### 6.2.2 Extra filters

```
snp <- BS.dartseq.gl$other$loc.metrics

# CLUSTER SIZE
ggplot2::qplot(x = snp$CLUSTER_SIZE, binwidth = 1, main = "Cluster size")
```

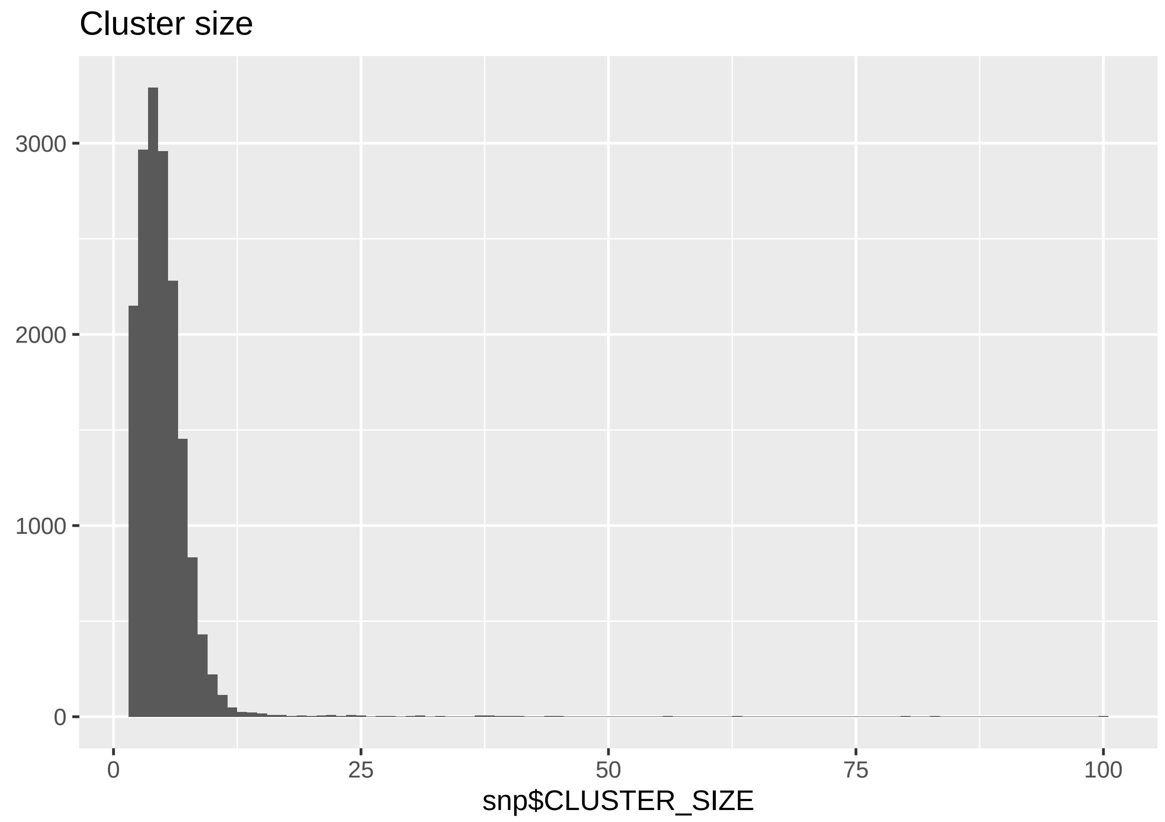


```
snp <- dplyr::filter(snp, CLUSTER_SIZE < 10)

# SEQUENCE LENGTH
seq.length <- sapply(X = snp$SEQUENCE, nchar, simplify = TRUE)
summary(seq.length)
snp <- dplyr::filter(snp, seq.length > 60)

# LOW-COMPLEXITY
res <- c()
for (i in 1:length(snp$SEQUENCE)) {
  seqsVector <- snp$SEQUENCE[i]
  lC_nucl <- c()
  for (nucl in c("A", "T", "G", "C")) {
    lC_nucl = c(lC_nucl,
                Biostrings::longestConsecutive(seqsVector, nucl))
  }
  res <- c(res, max(lC_nucl))
}
ggplot2::qplot(x = res, binwidth = 1, main = "Nucleotide repeats",
               xlab = "Longest consecutive repeats")
```

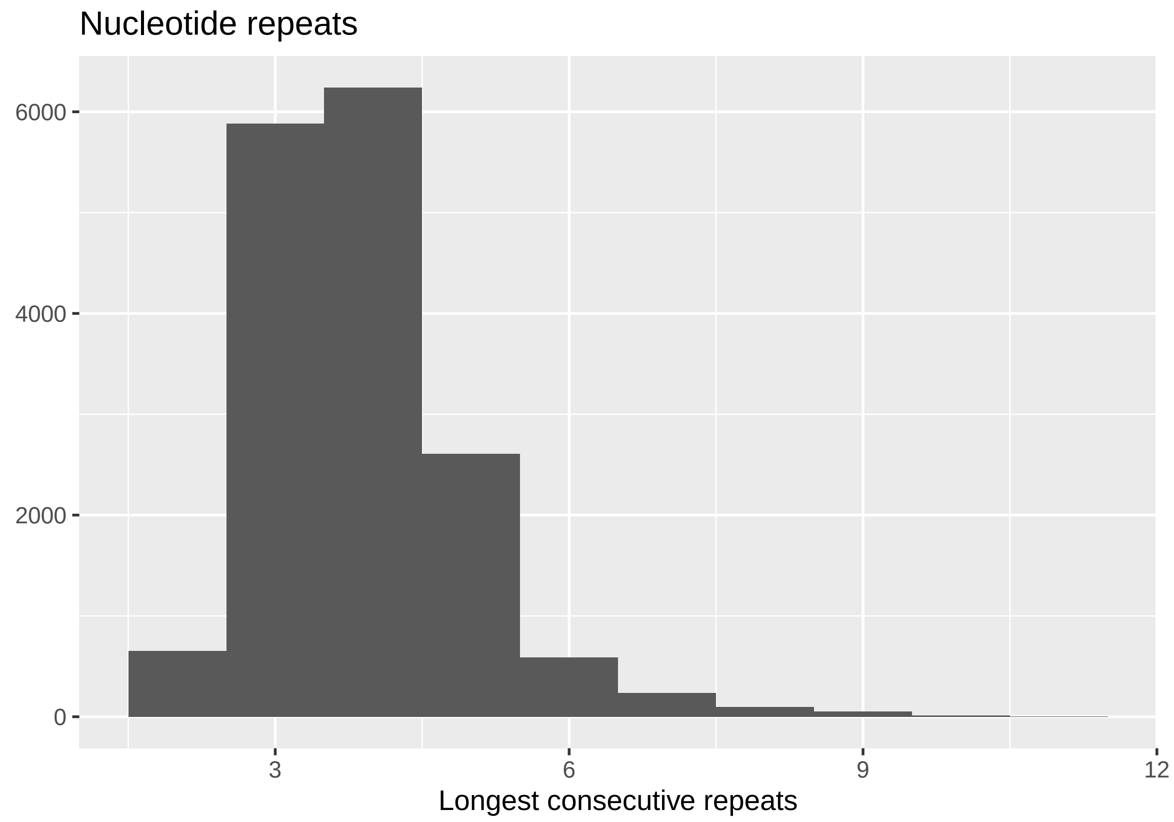


```
snp <- dplyr::filter(snp, res < 6)

snp <- dplyr::distinct(snp, LOCUS, .keep_all = TRUE) %>%
  dplyr::rename(CLONE_ID = LOCUS, AlleleSequence = SEQUENCE)
dim(snp)
snp <- snp[snp$MARKERS %in% sample(snp$MARKERS, 3200,replace = FALSE),]
snp %<>% dplyr::select(c("CLONE_ID", "AlleleSequence"))
head(snp)
# readr::write_tsv(snp, "Selected_sex-markers_for_baits.tsv")
```

```
##    Min. 1st Qu.  Median    Mean 3rd Qu.    Max.
##      69      69      69      69      69      69
## [1] 15381    36
## # A tibble: 6 x 2
##   CLONE_ID  AlleleSequence
##   <chr>     <chr>
## 1 100190323 TGCAGAGTGCCTCAGAGAGATTCACACTGAAACTCAGTGGGCAGAGCCTATTCCTGCTGAAGAGGCT~
## 2 100190357 TGCAGTGAAGTTATTGTGAAAATCCCCTAGTTGCCACACACTGGTGTCTGTTCAGGTACATGAGGGA~
## 3 100190483 TGCAGAAAAGCAGACATTTAAGACAAGATTTGAACTGGCGGGTGCATGAGCGGTTCAGCAGGAATGC~
## 4 100190716 TGCAGAAAGAATGGATTGCAAGGACATAGCTACAAGTATTGCTTGGGTTAAAGAGAAAATTAGCCAC~
## 5 100190719 TGCAGAAAGACAGTTTAAACAACAAGGTCGAATGCAACATTTCACAGTTGCATGAGCGGTTCAGCAG~
## 6 100190991 TGCAGAAATTCGTGTGTTAGACCTGCCGGGAGACAGCAAACCGAAAGGGTCGGGATTAAACAGGCAG~
```

## 6.3 Test selected markers

```
baits <- readr::read_tsv("Total_selected_markers.tsv")

dartseq.data <- "DArTseq_SNP_OrderAppendix_2_DCarcha19-4454.csv"
strata <- "Bull_shark_DArTseq_strata.tsv"
BSsnp <- radiator::read_dart(dartseq.data, strata,tidy.dart = FALSE,
                              parallel.core = 1)
```

```r
markers.meta <- radiator::extract_markers_metadata(BSsnp, whitelist = TRUE)
individuals.meta <- radiator::extract_individuals_metadata(BSsnp, whitelist = TRUE)
BSconvert <- radiator::genomic_converter(BSsnp ,output = "tidy")
BS.dartseq.raw.tidy <- BSconvert$tidy.data
save(BS.dartseq.raw.tidy,markers.meta, individuals.meta,
     file = "DArTseq_Raw_tidy.Rdata")
load("DArTseq_Raw_tidy.Rdata")

length(unique(BS.dartseq.raw.tidy$INDIVIDUALS)) #219
length(unique(BS.dartseq.raw.tidy$MARKERS)) #250945

BS.dartseq.baits.tidy <- BS.dartseq.raw.tidy[BS.dartseq.raw.tidy$MARKERS %in%
                                               baits$AlelleID &
                                               BS.dartseq.raw.tidy$INDIVIDUALS %in%
                                               BS.dartseq.gl$ind.names,]
length(unique(BS.dartseq.baits.tidy$INDIVIDUALS)) #142
length(unique(BS.dartseq.baits.tidy$MARKERS)) #3409

Convert <- radiator::genomic_converter(BS.dartseq.baits.tidy,
                                       output = c("genlight","gtypes"))
BS.dartseq.baits.gl <- Convert$genlight
BS.dartseq.baits.gt <- Convert$gtypes

save(BS.dartseq.baits.tidy,BS.dartseq.baits.gl, BS.dartseq.baits.gt,
     individuals.meta, markers.meta, file = "DArTseq_baits.Rdata")
```

### 6.3.1 Load data

```r
baits <- readr::read_tsv("Total_selected_markers.tsv")
load("DArTseq_baits.Rdata")
pop.levels <- c("Brazil", "Caribbean Sea", "Gulf of Mexico", "Western North Atlantic",
                "Sierra Leone", "South Africa", "Mozambique", "Reunion" , "Seychelles",
                "Arabian Sea", "Sri Lanka", "Thailand", "Indonesia", "Papua New Guinea",
                "Australia", "Japan", "Fiji")
BS.dartseq.baits.gl$pop <- factor(BS.dartseq.baits.gl$other$ind.metrics$Site,
                                  levels = pop.levels)
BS.dartseq.baits.gl <- BS.dartseq.baits.gl[order(BS.dartseq.baits.gl$pop,
                                                 BS.dartseq.baits.gl$ind.names),]

BS.dartseq.baits.tidy$POP_ID <- factor(BS.dartseq.baits.tidy$Site, levels = pop.levels)
BS.dartseq.baits.tidy <- BS.dartseq.baits.tidy[order(
  BS.dartseq.baits.tidy$POP_ID, BS.dartseq.baits.tidy$INDIVIDUALS),]

adegenet::nInd(BS.dartseq.baits.gl) # 142
adegenet::nLoc(BS.dartseq.baits.gl) # 3409
summary(BS.dartseq.baits.gl$pop)
sum(duplicated(BS.dartseq.baits.gl$other$ind.metrics$`Genetic code`))# 0 replicate left
sum(baits$CloneID %in% BS.dartseq.baits.gl$other$loc.metrics$LOCUS) #3409
```

```
## [1] 142
## [1] 3409
##                 Brazil          Caribbean Sea         Gulf of Mexico
##                     11                      2                     11
## Western North Atlantic           Sierra Leone           South Africa
##                      8                      1                      9
##             Mozambique                Reunion             Seychelles
##                      8                      9                     10
```

```
##          Arabian Sea            Sri Lanka             Thailand
##                    6                    4                    4
##            Indonesia     Papua New Guinea            Australia
##                    4                    2                   34
##                Japan                 Fiji
##                   11                    8
## [1] 0
## [1] 3409
```

### 6.3.2 Genetic diversity

```
set.seed(124)
gl <- BS.dartseq.baits.gl
pop.levels <- c("Brazil","Caribbean Sea","Gulf of Mexico",  "Western North Atlantic",
                "South Africa", "Mozambique", "Reunion" , "Seychelles", "Arabian Sea",
                "Sri Lanka", "Thailand", "Indonesia", "Papua New Guinea" , "Australia",
                "Japan", "Fiji")
gl <- gl[gl$pop != "Sierra Leone"] #only 1 sample
gl$pop <- factor(gl$other$ind.metrics$Site, levels = pop.levels)
gl <- gl[order(gl$pop, gl$ind.names)]

gl2gpop(gl, filename = "BS_dartseq_all_baits_genepop.txt")
bastat <- diveRsity::basicStats(infile = "BS_dartseq_all_baits_genepop.txt",
                                 outfile = "BS_dartseq_all_baits_BasicStat_output.txt",
                                fis_ci = TRUE,ar_ci = TRUE, fis_boots = 1000,
                                ar_boots = 1000,mc_reps = 1000,
                                rarefaction = FALSE, ar_alpha = 0.05,
                                fis_alpha = 0.05)
length(bastat$main_tab) #16
gendiv <- data.frame(
  "Brazil" = bastat$main_tab[[1]]$overall,
  "Caribbean Sea" = bastat$main_tab[[2]]$overall,
  "Gulf of Mexico" = bastat$main_tab[[3]]$overall,
  "Western North Atlantic" = bastat$main_tab[[4]]$overall,
  "South Africa" = bastat$main_tab[[5]]$overall,
  "Mozambique" = bastat$main_tab[[6]]$overall,
  "Reunion" = bastat$main_tab[[7]]$overall,
  "Seychelles" = bastat$main_tab[[8]]$overall,
  "Arabian Sea" = bastat$main_tab[[9]]$overall,
  "Sri Lanka" = bastat$main_tab[[10]]$overall,
  "Thailand" = bastat$main_tab[[11]]$overall,
  "Indonesia" = bastat$main_tab[[12]]$overall,
  "Papua New Guinea" = bastat$main_tab[[13]]$overall,
  "Australia" = bastat$main_tab[[14]]$overall,
  "Japan" = bastat$main_tab[[15]]$overall,
  "Fiji" = bastat$main_tab[[16]]$overall,
  check.names = FALSE)
row.names(gendiv) <- rownames(bastat$main_tab[[1]])

df <- as.data.frame(gl)
df[df == "0"] <- "11"
df[df == "1"] <- "12"
df[df == "2"] <- "22"
gen.gi <- adegenet::df2genind(df, ploidy = 2,ncode = 2, pop = gl$pop)
ris_basic <- hierfstat::basic.stats(gen.gi)
fis.hierfstat <- apply(ris_basic$Fis, 2, mean, na.rm=T)
fis.CI <- hierfstat::boot.ppfis(gen.gi, nboot = 1000, quant = c(0.025,0.975),
                                 diploid = TRUE,dig = 4)
```

```r
df <- data.frame(fis.hierfstat,fis.CI.low = fis.CI$fis.ci[,1],
                 fis.CI.high = fis.CI$fis.ci[,2])
df <- as.data.frame(t(df))
gendiv <- rbind(gendiv, df)

readr::write_tsv(gendiv, path = "DArTseq_ALL_baits_gendiv.txt")
save(gendiv, file = "DArTseq_ALL_baits_gendiv.Rdata")
```

```r
load("DArTseq_ALL_baits_gendiv.Rdata")
shortnames <- c("BRZ", "CAR", "GOM", "WNA", "SAF", "MOZ", "RUN" , "SEY",
                "ARS", "SRL", "TAI", "IND", "PNG" , "AUS", "JAP", "FIJ")
knitr::kable(gendiv, col.names = shortnames, digits = 4,caption = "") %>%
  kableExtra::kable_styling(full_width = FALSE,font_size = 5,
                            latex_options = c( "hold_position")) %>%
  kableExtra::landscape()
```

Table 7:

| | BRZ | CAR | GOM | WNA | SAF | MOZ | RUN | SEY | ARS | SRL | TAI | IND | PNG | AUS | JAP | FIJ |
|---|---|---|---|---|---|---|---|---|---|---|---|---|---|---|---|---|
| ar | 1.0740 | 1.0440 | 1.0700 | 1.0710 | 1.1120 | 1.0690 | 1.1100 | 1.1110 | 1.1040 | 1.1020 | 1.0970 | 1.0930 | 1.0720 | 1.1160 | 1.1070 | 1.0990 |
| size | 10.7760 | 1.9490 | 10.7670 | 7.8190 | 8.8250 | 7.2800 | 8.7800 | 9.8020 | 5.8420 | 3.9550 | 3.9320 | 3.9270 | 1.9720 | 33.2310 | 10.8050 | 7.6950 |
| obs_het | 0.0420 | 0.0380 | 0.0400 | 0.0420 | 0.0620 | 0.0570 | 0.0620 | 0.0610 | 0.0600 | 0.0620 | 0.0590 | 0.0590 | 0.0600 | 0.0610 | 0.0600 | 0.0610 |
| exp_het | 0.0480 | 0.0350 | 0.0460 | 0.0460 | 0.0670 | 0.0650 | 0.0670 | 0.0650 | 0.0630 | 0.0590 | 0.0580 | 0.0560 | 0.0440 | 0.0690 | 0.0630 | 0.0660 |
| uexp_het | 0.0490 | 0.0440 | 0.0470 | 0.0480 | 0.0710 | 0.0700 | 0.0710 | 0.0680 | 0.0690 | 0.0670 | 0.0670 | 0.0630 | 0.0560 | 0.0700 | 0.0660 | 0.0710 |
| fis | 0.0500 | -0.1950 | 0.0540 | 0.0210 | 0.0390 | 0.0620 | 0.0370 | 0.0280 | 0.0170 | -0.0780 | -0.0560 | -0.0840 | -0.4200 | 0.0800 | 0.0210 | 0.0370 |
| hwe_glb | 1.0000 | 1.0000 | 1.0000 | 1.0000 | 1.0000 | 1.0000 | 1.0000 | 1.0000 | 1.0000 | 1.0000 | 1.0000 | 1.0000 | 1.0000 | 1.0000 | 1.0000 | 1.0000 |
| hwe_hom | 1.0000 | 1.0000 | 1.0000 | 1.0000 | 1.0000 | 1.0000 | 1.0000 | 1.0000 | 1.0000 | 1.0000 | 1.0000 | 1.0000 | 1.0000 | 1.0000 | 1.0000 | 1.0000 |
| hwe_het | 1.0000 | 1.0000 | 1.0000 | 1.0000 | 1.0000 | 1.0000 | 1.0000 | 1.0000 | 1.0000 | 1.0000 | 1.0000 | 1.0000 | 1.0000 | 1.0000 | 1.0000 | 1.0000 |
| fis_lo | -0.0220 | -1.0000 | -0.0150 | -0.0940 | -0.0790 | -0.0660 | -0.0600 | -0.0510 | -0.1690 | -0.3350 | -0.3090 | -0.4580 | -1.0000 | 0.0580 | -0.0710 | -0.0810 |
| fis_hi | 0.0450 | -0.1950 | 0.0490 | 0.0150 | 0.0310 | 0.0570 | 0.0290 | 0.0170 | 0.0130 | -0.0780 | -0.0560 | -0.0840 | -0.4200 | 0.0820 | 0.0200 | 0.0260 |
| ar_lo | 1.0170 | 0.9970 | 1.0130 | 1.0160 | 1.0370 | 0.9010 | 1.0320 | 1.0370 | 0.9570 | 1.0450 | 1.0350 | 1.0350 | 1.0380 | 1.0530 | 1.0380 | 0.9280 |
| ar_hi | 1.0850 | 1.0770 | 1.0800 | 1.0870 | 1.1310 | 1.1230 | 1.1250 | 1.1240 | 1.1270 | 1.1230 | 1.1210 | 1.1260 | 1.0970 | 1.1290 | 1.1230 | 1.1230 |
| fis.hierfstat | 0.0936 | 0.0449 | 0.0984 | 0.0818 | 0.0920 | 0.1246 | 0.0907 | 0.0761 | 0.0977 | 0.0439 | 0.0655 | 0.0356 | -0.2143 | 0.0939 | 0.0653 | 0.0983 |
| fis.CI.low | 0.1159 | 0.0766 | 0.1240 | 0.0964 | 0.1003 | 0.1669 | 0.1049 | 0.0844 | 0.1185 | 0.0455 | 0.0812 | 0.0359 | -0.2006 | 0.1174 | 0.0673 | 0.1138 |
| fis.CI.high | 0.1781 | 0.2514 | 0.1894 | 0.1719 | 0.1583 | 0.2337 | 0.1634 | 0.1433 | 0.1907 | 0.1377 | 0.1707 | 0.1338 | -0.0408 | 0.1517 | 0.1230 | 0.1763 |

```r
pop.levels <- c("Brazil","Caribbean Sea","Gulf of Mexico",  "Western North Atlantic",
                "Sierra Leone", "South Africa", "Mozambique", "Reunion" , "Seychelles",
                "Arabian Sea", "Sri Lanka", "Thailand", "Indonesia", "Papua New Guinea",
                "Australia", "Japan", "Fiji")
BS.dartseq.baits.gl$pop <- factor(BS.dartseq.baits.gl$other$ind.metrics$Site,
                                  levels = pop.levels)
BS.dartseq.baits.gl <- BS.dartseq.baits.gl[order(BS.dartseq.baits.gl$pop,
                                                 BS.dartseq.baits.gl$ind.names)]

monomorphics <- c()
for (i in 1:length(pop.levels)) {
  x <- BS.dartseq.baits.gl[BS.dartseq.baits.gl$pop == pop.levels[i]]
  x2 <- dartR::gl.filter.monomorphs(x, verbose = 0)
  locs <-  adegenet::nLoc(x) - adegenet::nLoc(x2)
  monomorphics[i] <- locs
}
names(monomorphics) <- pop.levels
shortnames <- c("BRZ", "CAR", "GOM", "WNA","SIL", "SAF", "MOZ", "RUN" , "SEY",
                "ARS", "SRL", "TAI", "IND", "PNG" , "AUS", "JAP", "FIJ")
knitr::kable(t(as.table(monomorphics)), col.names = shortnames, digits = 4,
             caption = "") %>%
  kableExtra::kable_styling(full_width = FALSE,font_size = 5,
                            latex_options = c( "hold_position"))
```

Table 8:

| BRZ | CAR | GOM | WNA | SIL | SAF | MOZ | RUN | SEY | ARS | SRL | TAI | IND | PNG | AUS | JAP | FIJ |
|---|---|---|---|---|---|---|---|---|---|---|---|---|---|---|---|---|
| 2531 | 2966 | 2546 | 2610 | 3343 | 2426 | 2526 | 2458 | 2425 | 2616 | 2797 | 2798 | 2856 | 3143 | 1742 | 2429 | 2521 |

#### 6.3.2.1 Number of monomorphic markers per population

```r
pi.sum <- radiator::pi(
  data = BS.dartseq.baits.tidy,
  read.length = 70,
  parallel.core = parallel::detectCores() - 1,
  path.folder = NULL,
  verbose = TRUE
)

FIS.sum<- radiator::ibdg_fh(data = BS.dartseq.baits.tidy,
        path.folder = NULL, verbose = TRUE)

save(pi.sum, FIS.sum, file = "DArTcap_ALL_baits_pi_FIS_Sites2.Rdata")
```

```r
load("DArTcap_ALL_pi_FIS_Sites2.Rdata")
pi.sum$boxplot.pi
```

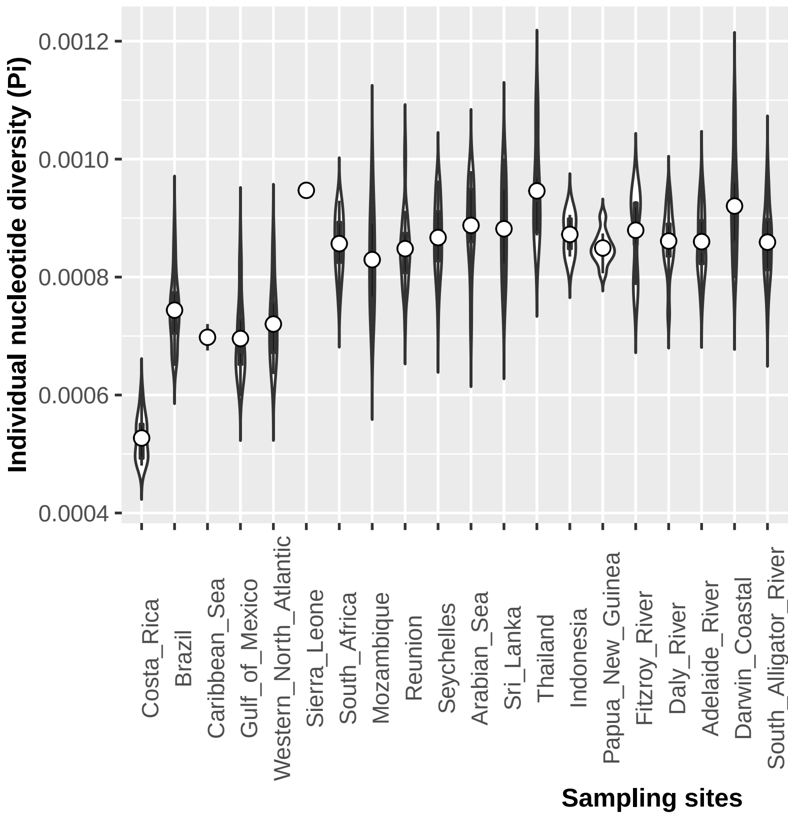


#### 6.3.2.2 Nucleotide diversity and inbreeding

```
FIS.sum$fh.box.plot
```

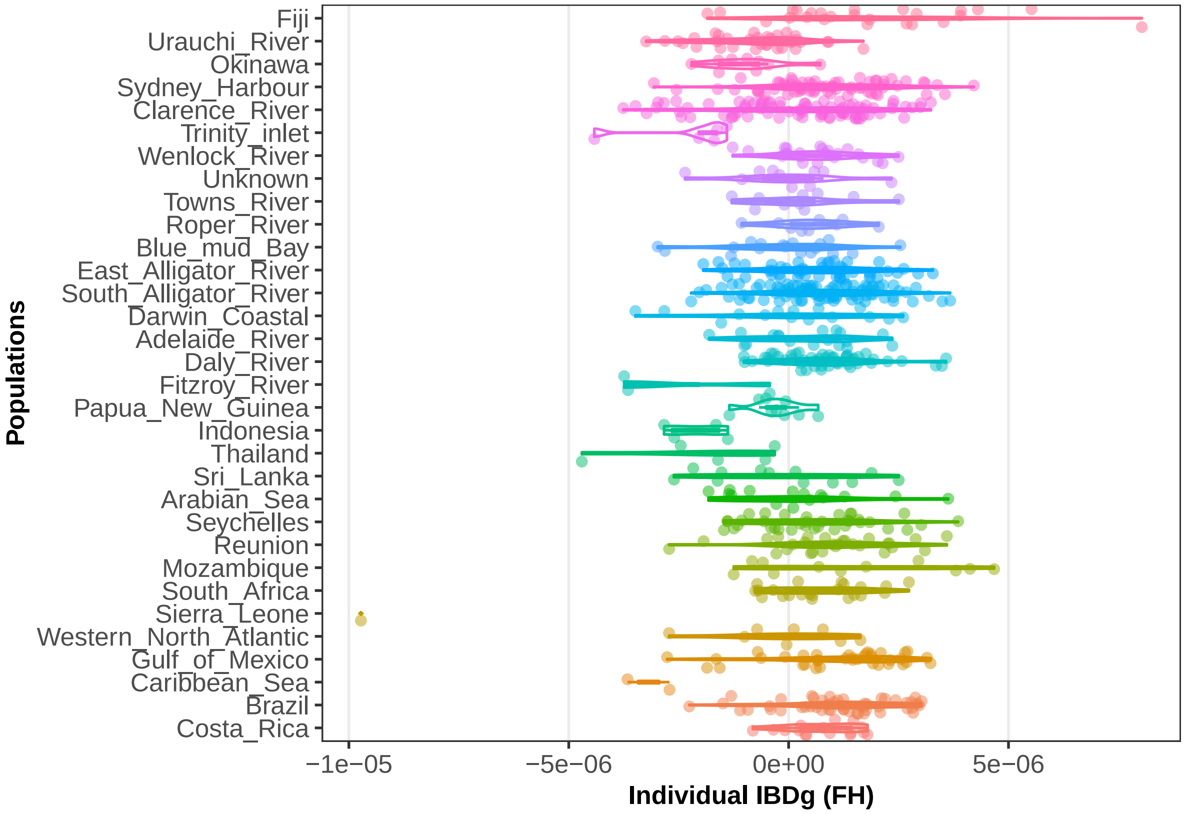

### 6.3.3 Genetic differentiation

```
strata <- as.character(BS.dartseq.baits.gt@schemes$Site)
names(strata) <- BS.dartseq.baits.gt@schemes$id
strataG::setStrata(BS.dartseq.baits.gt) <- strata

Fst.gt <- strataG::popStructTest(
    BS.dartseq.baits.gt,
    nrep = 1000,
    stats = "all",
    type = "pairwise",
    keep.null = FALSE,
    quietly = TRUE,
    nb.cores =  parallel::detectCores() - 1,
    write.output = FALSE
  )
save(Fst.gt, file = "DArTseq_ALL_baits_FST.Rdata")
readr::write_tsv(Fst.gt$pairwise$result, path = "DArTseq_ALL_baits_FST.txt")
```

```
load("DArTseq_ALL_baits_FST.Rdata")
kableExtra::kbl(Fst.gt$pairwise$result[order(Fst.gt$pairwise$result$Fst,
                                        decreasing = TRUE),
                                   -c(1:4)], digits = 4,
  caption = "Pairwise Fst for 3409 DArTseq baits between the all sites",
  longtable = TRUE, booktabs = T) %>%
  kableExtra::kable_styling(full_width = FALSE,font_size = 5,
                         latex_options = c("repeat_header", "hold_position")) %>%
  kableExtra::landscape()
```

Table 9: Pairwise Fst for 3409 DArTseq baits between the all sites

| | Chi2 | Chi2.p.val | D | D.p.val | Fis | Fis.p.val | Fst | Fst.p.val | F’st | F’st.p.val | Gst | Gst.p.val | G’st | G’st.p.val | G”st | G”st.p.val |
|---|---|---|---|---|---|---|---|---|---|---|---|---|---|---|---|---|
| Gulf of Mexico (11) v. Papua New Guinea (2) | 8102.5340 | 0.0100 | 0.0101 | 0.0100 | NA | NA | 0.6089 | 0.2041 | 0.6433 | 0.2041 | NA | NA | NA | NA | NA | NA |
| Papua New Guinea (2) v. Western North Atlantic (8) | 6372.1345 | 0.0230 | 0.0103 | 0.0230 | NA | NA | 0.5985 | 0.1917 | NA | NA | NA | NA | NA | NA | NA | NA |
| Gulf of Mexico (11) v. Indonesia (4) | 9532.0578 | 0.0030 | 0.0103 | 0.0030 | NA | NA | 0.5965 | 0.0043 | 0.6344 | 0.0043 | NA | NA | NA | NA | NA | NA |
| Gulf of Mexico (11) v. Thailand (4) | 9686.5999 | 0.0020 | 0.0104 | 0.0020 | NA | NA | 0.5960 | 0.0026 | 0.6339 | 0.0026 | NA | NA | NA | NA | NA | NA |
| Gulf of Mexico (11) v. Sri Lanka (4) | 9509.0294 | 0.0040 | 0.0100 | 0.0040 | NA | NA | 0.5872 | 0.0059 | 0.6231 | 0.0059 | NA | NA | NA | NA | NA | NA |
| Indonesia (4) v. Western North Atlantic (8) | 7763.2194 | 0.0030 | 0.0104 | 0.0030 | NA | NA | 0.5870 | 0.0041 | 0.6357 | 0.0041 | NA | NA | NA | NA | NA | NA |
| Brazil (11) v. Indonesia (4) | 9236.5349 | 0.0020 | 0.0101 | 0.0020 | NA | NA | 0.5842 | 0.0023 | 0.6223 | 0.0023 | NA | NA | NA | NA | NA | NA |
| Thailand (4) v. Western North Atlantic (8) | 7829.6372 | 0.0060 | 0.0104 | 0.0060 | NA | NA | 0.5842 | 0.0078 | 0.6334 | 0.0083 | NA | NA | NA | NA | NA | NA |
| Brazil (11) v. Thailand (4) | 9378.6255 | 0.0010 | 0.0101 | 0.0010 | NA | NA | 0.5828 | 0.0012 | 0.6208 | 0.0012 | NA | NA | NA | NA | NA | NA |
| Japan (11) v. Western North Atlantic (8) | 12623.2524 | 0.0010 | 0.0116 | 0.0010 | NA | NA | 0.5824 | 0.0010 | 0.6272 | 0.0010 | NA | NA | NA | NA | NA | NA |
| Brazil (11) v. Japan (11) | 14386.7049 | 0.0010 | 0.0112 | 0.0010 | NA | NA | 0.5807 | 0.0010 | 0.6242 | 0.0010 | NA | NA | NA | NA | NA | NA |
| Sri Lanka (4) v. Western North Atlantic (8) | 7713.8626 | 0.0030 | 0.0100 | 0.0030 | NA | NA | 0.5763 | 0.0047 | 0.6248 | 0.0053 | NA | NA | NA | NA | NA | NA |
| Arabian Sea (6) v. Gulf of Mexico (11) | 10961.0694 | 0.0010 | 0.0101 | 0.0010 | NA | NA | 0.5759 | 0.0010 | 0.6230 | 0.0011 | NA | NA | NA | NA | NA | NA |
| Fiji (8) v. Gulf of Mexico (11) | 12403.9837 | 0.0010 | 0.0107 | 0.0010 | NA | NA | 0.5750 | 0.0010 | 0.6222 | 0.0010 | NA | NA | NA | NA | NA | NA |
| Brazil (11) v. Sri Lanka (4) | 9218.8942 | 0.0010 | 0.0098 | 0.0010 | NA | NA | 0.5739 | 0.0014 | 0.6099 | 0.0014 | NA | NA | NA | NA | NA | NA |
| Gulf of Mexico (11) v. Reunion (9) | 13151.6388 | 0.0010 | 0.0108 | 0.0010 | NA | NA | 0.5708 | 0.0010 | NA | NA | NA | NA | NA | NA | NA | NA |
| Gulf of Mexico (11) v. Seychelles (10) | 13436.3933 | 0.0010 | 0.0104 | 0.0010 | NA | NA | 0.5684 | 0.0010 | NA | NA | NA | NA | NA | NA | NA | NA |
| Gulf of Mexico (11) v. Mozambique (8) | 11723.3601 | 0.0010 | 0.0103 | 0.0010 | NA | NA | 0.5680 | 0.0010 | NA | NA | NA | NA | NA | NA | NA | NA |
| Brazil (11) v. Fiji (8) | 12117.7180 | 0.0010 | 0.0105 | 0.0010 | NA | NA | 0.5640 | 0.0010 | 0.6163 | 0.0010 | NA | NA | NA | NA | NA | NA |
| Arabian Sea (6) v. Brazil (11) | 10647.2547 | 0.0010 | 0.0098 | 0.0010 | NA | NA | 0.5639 | 0.0010 | 0.6109 | 0.0011 | NA | NA | NA | NA | NA | NA |
| Arabian Sea (6) v. Western North Atlantic (8) | 9079.0498 | 0.0010 | 0.0102 | 0.0010 | NA | NA | 0.5639 | 0.0011 | 0.6112 | 0.0011 | NA | NA | NA | NA | NA | NA |
| Fiji (8) v. Western North Atlantic (8) | 10387.0276 | 0.0020 | 0.0108 | 0.0020 | NA | NA | 0.5629 | 0.0025 | 0.6101 | 0.0025 | NA | NA | NA | NA | NA | NA |
| Caribbean Sea (2) v. Papua New Guinea (2) | 2790.0714 | 0.3467 | 0.0099 | 0.3467 | NA | NA | 0.5614 | 0.3467 | 0.6059 | 0.3467 | NA | NA | NA | NA | NA | NA |
| Brazil (11) v. Reunion (9) | 12855.1300 | 0.0010 | 0.0106 | 0.0010 | NA | NA | 0.5601 | 0.0010 | 0.6077 | 0.0010 | NA | NA | NA | NA | NA | NA |
| Reunion (9) v. Western North Atlantic (8) | 11106.9875 | 0.0010 | 0.0109 | 0.0010 | NA | NA | 0.5593 | 0.0010 | 0.6058 | 0.0010 | NA | NA | NA | NA | NA | NA |
| Brazil (11) v. Seychelles (10) | 13186.7411 | 0.0010 | 0.0102 | 0.0010 | NA | NA | 0.5580 | 0.0010 | 0.6036 | 0.0010 | NA | NA | NA | NA | NA | NA |
| Seychelles (10) v. Western North Atlantic (8) | 11468.7198 | 0.0010 | 0.0105 | 0.0010 | NA | NA | 0.5580 | 0.0010 | 0.6030 | 0.0010 | NA | NA | NA | NA | NA | NA |
| Brazil (11) v. Mozambique (8) | 11470.4265 | 0.0010 | 0.0101 | 0.0010 | NA | NA | 0.5572 | 0.0010 | NA | NA | NA | NA | NA | NA | NA | NA |
| Brazil (11) v. South Africa (9) | 12589.1098 | 0.0010 | 0.0099 | 0.0010 | NA | NA | 0.5559 | 0.0010 | NA | NA | NA | NA | NA | NA | NA | NA |
| Mozambique (8) v. Western North Atlantic (8) | 9811.4527 | 0.0030 | 0.0104 | 0.0030 | NA | NA | 0.5557 | 0.0031 | 0.6040 | 0.0031 | NA | NA | NA | NA | NA | NA |
| Caribbean Sea (2) v. Indonesia (4) | 3907.0225 | 0.0609 | 0.0102 | 0.0609 | NA | NA | 0.5553 | 1.0000 | NA | NA | NA | NA | NA | NA | NA | NA |
| South Africa (9) v. Western North Atlantic (8) | 10865.6475 | 0.0010 | 0.0101 | 0.0010 | NA | NA | 0.5550 | 0.0010 | 0.6007 | 0.0010 | NA | NA | NA | NA | NA | NA |
| Caribbean Sea (2) v. Thailand (4) | 3880.0798 | 0.0689 | 0.0101 | 0.0689 | NA | NA | 0.5384 | 0.3670 | 0.5877 | 0.5433 | NA | NA | NA | NA | NA | NA |
| Australia (34) v. Gulf of Mexico (11) | 26372.2394 | 0.0010 | 0.0104 | 0.0010 | NA | NA | 0.5380 | 0.0010 | 0.5800 | 0.0010 | NA | NA | NA | NA | NA | NA |
| Caribbean Sea (2) v. Sri Lanka (4) | 3833.6746 | 0.0749 | 0.0097 | 0.0749 | NA | NA | 0.5343 | 0.1820 | 0.5785 | 0.5474 | NA | NA | NA | NA | NA | NA |
| Australia (34) v. Western North Atlantic (8) | 24019.0389 | 0.0010 | 0.0105 | 0.0010 | NA | NA | 0.5321 | 0.0011 | 0.5738 | 0.0011 | NA | NA | NA | NA | NA | NA |
| Australia (34) v. Brazil (11) | 25908.7124 | 0.0010 | 0.0102 | 0.0010 | NA | NA | 0.5298 | 0.0010 | 0.5714 | 0.0010 | NA | NA | NA | NA | NA | NA |
| Caribbean Sea (2) v. Seychelles (10) | 6931.6802 | 0.0120 | 0.0101 | 0.0120 | NA | NA | 0.5239 | 0.1121 | NA | NA | NA | NA | NA | NA | NA | NA |
| Caribbean Sea (2) v. Fiji (8) | 5943.5605 | 0.0230 | 0.0105 | 0.0230 | NA | NA | 0.5210 | 0.2771 | NA | NA | NA | NA | NA | NA | NA | NA |
| Arabian Sea (6) v. Caribbean Sea (2) | 4912.1344 | 0.0390 | 0.0098 | 0.0390 | NA | NA | 0.5185 | 0.1418 | 0.5600 | 0.1418 | NA | NA | NA | NA | NA | NA |
| Caribbean Sea (2) v. Reunion (9) | 6511.0696 | 0.0170 | 0.0105 | 0.0170 | NA | NA | 0.5178 | 0.4146 | NA | NA | NA | NA | NA | NA | NA | NA |
| Caribbean Sea (2) v. Mozambique (8) | 5511.7323 | 0.0170 | 0.0101 | 0.0170 | NA | NA | 0.5124 | 1.0000 | NA | NA | NA | NA | NA | NA | NA | NA |
| Australia (34) v. Caribbean Sea (2) | 17826.7556 | 0.0030 | 0.0102 | 0.0030 | NA | NA | 0.5097 | 0.2727 | 0.5486 | 0.2727 | NA | NA | NA | NA | NA | NA |
| Papua New Guinea (2) v. Sierra Leone (1) | 869.8667 | 0.3327 | 0.0008 | 0.3327 | NA | NA | 0.1103 | 0.3327 | 0.1184 | 0.3327 | NA | NA | NA | NA | NA | NA |
| Indonesia (4) v. Japan (11) | 1900.8212 | 0.0010 | 0.0004 | 0.0010 | NA | NA | 0.0777 | 0.0010 | NA | NA | NA | NA | NA | NA | NA | NA |
| Fiji (8) v. Japan (11) | 2290.7442 | 0.0010 | 0.0003 | 0.0010 | -23.8431 | 1.0000 | 0.0641 | 0.0010 | 0.0698 | 0.0011 | 0.4458 | 0.0012 | 0.4713 | 0.0012 | 0.6343 | 0.0012 |
| Fiji (8) v. Indonesia (4) | 1659.3082 | 0.0020 | 0.0004 | 0.0020 | NA | NA | 0.0595 | 0.0021 | 0.0647 | 0.0021 | NA | NA | NA | NA | NA | NA |
| Indonesia (4) v. Papua New Guinea (2) | 1051.8778 | 0.2108 | 0.0005 | 0.2108 | NA | NA | 0.0537 | 0.1734 | 0.0579 | 0.1734 | NA | NA | NA | NA | NA | NA |
| Japan (11) v. Sri Lanka (4) | 1678.9484 | 0.0010 | 0.0003 | 0.0140 | NA | NA | 0.0492 | 0.0050 | 0.0529 | 0.0053 | NA | NA | NA | NA | NA | NA |
| Japan (11) v. Thailand (4) | 1683.9072 | 0.0030 | 0.0003 | 0.0080 | NA | NA | 0.0468 | 0.0150 | 0.0504 | 0.0164 | NA | NA | NA | NA | NA | NA |
| Japan (11) v. Mozambique (8) | 1958.8523 | 0.0010 | 0.0003 | 0.0010 | NA | NA | 0.0462 | 0.0010 | 0.0523 | 0.0012 | NA | NA | NA | NA | NA | NA |
| Indonesia (4) v. Thailand (4) | 1197.2271 | 0.0559 | 0.0003 | 0.1469 | 13.5058 | 0.9760 | 0.0458 | 0.0559 | 0.0498 | 0.0559 | 29.4957 | 0.0345 | 25.9063 | 0.0345 | 1.8167 | 0.0345 |
| Japan (11) v. Papua New Guinea (2) | 1417.8562 | 0.0430 | 0.0004 | 0.0659 | NA | NA | 0.0432 | 0.0994 | 0.0464 | 0.0994 | NA | NA | NA | NA | NA | NA |

Table 9: Pairwise Fst for 3409 DArTseq baits between the all sites *(continued)*

| | Chi2 | Chi2.p.val | D | D.p.val | Fis | Fis.p.val | Fst | Fst.p.val | F'st | F'st.p.val | Gst | Gst.p.val | G'st | G'st.p.val | G"st | G"st.p.val |
|---|---|---|---|---|---|---|---|---|---|---|---|---|---|---|---|---|
| Japan (11) v. South Africa (9) | 2001.5867 | 0.0010 | 0.0002 | 0.0010 | NA | NA | 0.0405 | 0.0010 | 0.0442 | 0.0019 | NA | NA | NA | NA | NA | NA |
| Fiji (8) v. Seychelles (10) | 2046.0674 | 0.0020 | 0.0002 | 0.0030 | -21.7300 | 1.0000 | 0.0395 | 0.0021 | 0.0434 | 0.0021 | 0.4257 | 0.0021 | 0.4515 | 0.0021 | 0.6152 | 0.0021 |
| Arabian Sea (6) v. Japan (11) | 1790.6767 | 0.0010 | 0.0002 | 0.0010 | NA | NA | 0.0381 | 0.0010 | 0.0415 | 0.0019 | NA | NA | NA | NA | NA | NA |
| Japan (11) v. Reunion (9) | 2005.6853 | 0.0010 | 0.0003 | 0.0010 | -20.2818 | 0.9955 | 0.0374 | 0.0011 | 0.0409 | 0.0011 | 0.3750 | 0.0022 | 0.3999 | 0.0022 | 0.5636 | 0.0022 |
| Japan (11) v. Seychelles (10) | 2009.7485 | 0.0010 | 0.0002 | 0.0010 | NA | NA | 0.0360 | 0.0010 | 0.0393 | 0.0012 | NA | NA | NA | NA | NA | NA |
| Fiji (8) v. Mozambique (8) | 1891.5201 | 0.0010 | 0.0003 | 0.0010 | -34.8132 | 0.9978 | 0.0358 | 0.0010 | 0.0413 | 0.0011 | 0.5894 | 0.0011 | 0.6124 | 0.0011 | 0.7561 | 0.0011 |
| Indonesia (4) v. Mozambique (8) | 1451.4379 | 0.0270 | 0.0003 | 0.0509 | NA | NA | 0.0344 | 0.0183 | NA | NA | NA | NA | NA | NA | NA | NA |
| Fiji (8) v. Thailand (4) | 1527.9697 | 0.0070 | 0.0003 | 0.0090 | NA | NA | 0.0336 | 0.0078 | 0.0365 | 0.0079 | NA | NA | NA | NA | NA | NA |
| Indonesia (4) v. Seychelles (10) | 1509.6262 | 0.0150 | 0.0003 | 0.0170 | NA | NA | 0.0333 | 0.0101 | NA | NA | NA | NA | NA | NA | NA | NA |
| Australia (34) v. Fiji (8) | 3012.8187 | 0.0010 | 0.0002 | 0.0010 | -9.1806 | 0.9909 | 0.0328 | 0.0013 | 0.0357 | 0.0013 | 0.1827 | 0.0013 | 0.2022 | 0.0013 | 0.3254 | 0.0013 |
| Fiji (8) v. Sri Lanka (4) | 1519.3088 | 0.0060 | 0.0003 | 0.0060 | NA | NA | 0.0325 | 0.0063 | 0.0352 | 0.0069 | NA | NA | NA | NA | NA | NA |
| Australia (34) v. Japan (11) | 2806.5855 | 0.0010 | 0.0002 | 0.0010 | -9.1646 | 1.0000 | 0.0324 | 0.0010 | 0.0350 | 0.0010 | 0.1477 | 0.0015 | 0.1638 | 0.0015 | 0.2714 | 0.0015 |
| Fiji (8) v. South Africa (9) | 1921.1801 | 0.0010 | 0.0002 | 0.0010 | NA | NA | 0.0307 | 0.0010 | 0.0338 | 0.0011 | NA | NA | NA | NA | NA | NA |
| Fiji (8) v. Reunion (9) | 1952.9401 | 0.0010 | 0.0002 | 0.0010 | -22.7665 | 1.0000 | 0.0306 | 0.0010 | 0.0338 | 0.0010 | 0.4456 | 0.0010 | 0.4716 | 0.0010 | 0.6345 | 0.0010 |
| Indonesia (4) v. Sri Lanka (4) | 1130.9851 | 0.1888 | 0.0002 | 0.1588 | NA | NA | 0.0294 | 0.1588 | NA | NA | NA | NA | NA | NA | NA | NA |
| Australia (34) v. Indonesia (4) | 2314.2974 | 0.0010 | 0.0002 | 0.0010 | NA | NA | 0.0280 | 0.0011 | 0.0302 | 0.0011 | NA | NA | NA | NA | NA | NA |
| Arabian Sea (6) v. Indonesia (4) | 1292.8201 | 0.0839 | 0.0003 | 0.0859 | NA | NA | 0.0262 | 0.0967 | 0.0285 | 0.1067 | NA | NA | NA | NA | NA | NA |
| Indonesia (4) v. South Africa (9) | 1474.5715 | 0.0270 | 0.0002 | 0.1409 | 81.9077 | 0.9967 | 0.0260 | 0.0142 | 0.0288 | 0.0153 | 1.2958 | 0.0077 | 1.2727 | 0.0077 | 1.1188 | 0.0077 |
| Arabian Sea (6) v. Fiji (8) | 1629.1213 | 0.0020 | 0.0002 | 0.0040 | -57.6854 | 0.9706 | 0.0217 | 0.0020 | 0.0243 | 0.0025 | 0.7147 | 0.0240 | 0.7322 | 0.0240 | 0.8438 | 0.0240 |
| Sri Lanka (4) v. Thailand (4) | 1107.5346 | 0.0210 | 0.0002 | 0.0849 | NA | NA | 0.0124 | 0.0839 | 0.0135 | 0.0591 | NA | NA | NA | NA | NA | NA |
| Australia (34) v. Mozambique (8) | 2276.3729 | 0.0060 | 0.0001 | 0.5115 | -10.0857 | 1.0000 | 0.0120 | 0.0018 | 0.0132 | 0.0018 | 0.2040 | 0.0018 | 0.2242 | 0.0018 | 0.3556 | 0.0018 |
| Mozambique (8) v. South Africa (9) | 1594.8721 | 0.0260 | 0.0002 | 0.0150 | NA | NA | 0.0086 | 0.0270 | 0.0095 | 0.0227 | NA | NA | NA | NA | NA | NA |
| Australia (34) v. Sri Lanka (4) | 2030.6818 | 0.1818 | 0.0001 | 0.6024 | -18.2684 | 0.7222 | 0.0078 | 0.0812 | 0.0084 | 0.0812 | 0.4224 | 0.3137 | 0.4472 | 0.3235 | 0.6114 | 0.3235 |
| South Africa (9) v. Sri Lanka (4) | 1397.1209 | 0.1329 | 0.0002 | 0.0100 | NA | NA | 0.0076 | 0.1418 | NA | NA | NA | NA | NA | NA | NA | NA |
| Seychelles (10) v. Sri Lanka (4) | 1374.9997 | 0.1209 | 0.0002 | 0.2158 | NA | NA | 0.0071 | 0.1636 | 0.0076 | 0.1604 | NA | NA | NA | NA | NA | NA |
| Reunion (9) v. Seychelles (10) | 1565.7084 | 0.0759 | 0.0001 | 0.0220 | -19.6474 | 0.7569 | 0.0067 | 0.0921 | 0.0074 | 0.0921 | 0.3668 | 0.1410 | 0.3917 | 0.1398 | 0.5550 | 0.1398 |
| Caribbean Sea (2) v. Gulf of Mexico (11) | 981.1163 | 0.0869 | 0.0001 | 0.3277 | NA | NA | 0.0062 | 0.2432 | NA | NA | NA | NA | NA | NA | NA | NA |
| Seychelles (10) v. South Africa (9) | 1590.4783 | 0.0929 | 0.0001 | 0.0040 | NA | NA | 0.0056 | 0.1139 | 0.0061 | 0.1196 | NA | NA | NA | NA | NA | NA |
| Mozambique (8) v. Sri Lanka (4) | 1294.3686 | 0.3127 | 0.0002 | 0.1419 | NA | NA | 0.0056 | 0.2304 | 0.0060 | 0.2382 | NA | NA | NA | NA | NA | NA |
| Mozambique (8) v. Seychelles (10) | 1553.9683 | 0.1269 | 0.0001 | 0.4156 | NA | NA | 0.0042 | 0.1998 | 0.0046 | 0.2159 | NA | NA | NA | NA | NA | NA |
| Mozambique (8) v. Thailand (4) | 1279.2778 | 0.4456 | 0.0002 | 0.2358 | NA | NA | 0.0039 | 0.2745 | 0.0043 | 0.2714 | NA | NA | NA | NA | NA | NA |
| Gulf of Mexico (11) v. Western North Atlantic (8) | 1150.4714 | 0.1019 | 0.0000 | 0.1349 | NA | NA | 0.0039 | 0.1556 | NA | NA | NA | NA | NA | NA | NA | NA |
| Australia (34) v. Reunion (9) | 2090.7826 | 0.1758 | 0.0001 | 0.2268 | -8.9360 | 0.1864 | 0.0036 | 0.0833 | 0.0040 | 0.0833 | 0.1479 | 0.3111 | 0.1644 | 0.2853 | 0.2721 | 0.2995 |
| Seychelles (10) v. Thailand (4) | 1308.7435 | 0.3596 | 0.0002 | 0.1928 | NA | NA | 0.0036 | 0.2876 | 0.0039 | 0.3096 | NA | NA | NA | NA | NA | NA |
| Brazil (11) v. Western North Atlantic (8) | 1174.3975 | 0.1608 | 0.0000 | 0.3377 | NA | NA | 0.0027 | 0.2397 | NA | NA | NA | NA | NA | NA | NA | NA |
| Australia (34) v. South Africa (9) | 2305.7262 | 0.0030 | 0.0001 | 0.0849 | NA | NA | 0.0025 | 0.1309 | 0.0027 | 0.1310 | NA | NA | NA | NA | NA | NA |
| Australia (34) v. Seychelles (10) | 2176.7156 | 0.0420 | 0.0001 | 0.0350 | -8.6955 | 0.9422 | 0.0020 | 0.1359 | 0.0022 | 0.1361 | 0.1355 | 0.0766 | 0.1507 | 0.0797 | 0.2521 | 0.0797 |
| Reunion (9) v. Sri Lanka (4) | 1309.9356 | 0.3127 | 0.0002 | 0.1638 | 123.7838 | 0.6395 | 0.0016 | 0.3636 | 0.0018 | 0.3636 | 1.1848 | 0.3416 | 1.1707 | 0.3416 | 1.0781 | 0.3416 |
| Australia (34) v. Thailand (4) | 1935.2647 | 0.4885 | 0.0001 | 0.6274 | NA | NA | 0.0008 | 0.3957 | 0.0009 | 0.3951 | NA | NA | NA | NA | NA | NA |
| Mozambique (8) v. Reunion (9) | 1478.8841 | 0.3816 | 0.0001 | 0.2977 | -29.0749 | 1.0000 | 0.0005 | 0.4842 | 0.0005 | 0.4842 | 0.5147 | 0.0011 | 0.5388 | 0.0011 | 0.6955 | 0.0011 |
| Brazil (11) v. Gulf of Mexico (11) | 1208.1742 | 0.2987 | 0.0000 | 0.1798 | NA | NA | 0.0004 | 0.4380 | 0.0004 | 0.4758 | NA | NA | NA | NA | NA | NA |
| Arabian Sea (6) v. Australia (34) | 2043.3687 | 0.2957 | 0.0000 | 0.9960 | -11.2122 | 0.5175 | 0.0003 | 0.4555 | 0.0003 | 0.4577 | 0.2357 | 0.4330 | 0.2572 | 0.4412 | 0.3989 | 0.4371 |
| Arabian Sea (6) v. South Africa (9) | 1424.3239 | 0.5145 | 0.0001 | 0.3057 | NA | NA | 0.0002 | 0.4975 | 0.0003 | 0.5249 | NA | NA | NA | NA | NA | NA |
| South Africa (9) v. Thailand (4) | 1269.4545 | 0.8352 | 0.0002 | 0.4356 | 106.7048 | 0.4233 | -0.0012 | 0.5501 | -0.0013 | 0.5501 | 1.2200 | 0.5300 | 1.2033 | 0.5276 | 1.0916 | 0.5264 |
| Caribbean Sea (2) v. Western North Atlantic (8) | 805.3188 | 0.7173 | 0.0001 | 0.3816 | NA | NA | -0.0015 | 0.5933 | NA | NA | NA | NA | NA | NA | NA | NA |
| Arabian Sea (6) v. Sri Lanka (4) | 1145.9785 | 0.8292 | 0.0002 | 0.3387 | NA | NA | -0.0047 | 0.7815 | -0.0051 | 0.7753 | NA | NA | NA | NA | NA | NA |
| Arabian Sea (6) v. Seychelles (10) | 1400.2838 | 0.5425 | 0.0001 | 0.9261 | NA | NA | -0.0049 | 0.8142 | -0.0054 | 0.8054 | NA | NA | NA | NA | NA | NA |
| Arabian Sea (6) v. Reunion (9) | 1333.2114 | 0.6194 | 0.0001 | 0.5774 | -41.6492 | 0.3902 | -0.0057 | 0.7993 | -0.0063 | 0.7975 | 0.6194 | 0.6545 | 0.6404 | 0.6590 | 0.7779 | 0.6579 |
| Arabian Sea (6) v. Thailand (4) | 1115.7016 | 0.9660 | 0.0001 | 0.9451 | NA | NA | -0.0062 | 0.8847 | -0.0067 | 0.8951 | NA | NA | NA | NA | NA | NA |
| Arabian Sea (6) v. Mozambique (8) | 1279.5434 | 0.9840 | 0.0001 | 0.9720 | NA | NA | -0.0107 | 0.9736 | -0.0124 | 0.9853 | NA | NA | NA | NA | NA | NA |
| Arabian Sea (6) v. Papua New Guinea (2) | 1029.1868 | 0.8621 | 0.0003 | 0.7962 | NA | NA | -0.0137 | 0.9339 | -0.0148 | 0.9339 | NA | NA | NA | NA | NA | NA |
| Mozambique (8) v. Papua New Guinea (2) | 1064.4045 | 0.8751 | 0.0003 | 0.6214 | NA | NA | -0.0199 | 1.0000 | -0.0216 | 1.0000 | NA | NA | NA | NA | NA | NA |
| Arabian Sea (6) v. Sierra Leone (1) | 1366.8318 | 0.1439 | 0.0008 | 0.3047 | NA | NA | NA | NA | NA | NA | NA | NA | NA | NA | NA | NA |

Table 9: Pairwise Fst for 3409 DArTseq baits between the all sites *(continued)*

| | Chi2 | Chi2.p.val | D | D.p.val | Fis | Fis.p.val | Fst | Fst.p.val | F'st | F'st.p.val | Gst | Gst.p.val | G'st | G'st.p.val | G"st | G"st.p.val |
|---|---|---|---|---|---|---|---|---|---|---|---|---|---|---|---|---|
| Australia (34) v. Papua New Guinea (2) | 1696.0948 | 0.9181 | 0.0002 | 0.7053 | NA | NA | NA | NA | NA | NA | NA | NA | NA | NA | NA | NA |
| Australia (34) v. Sierra Leone (1) | 3623.3691 | 0.0290 | 0.0008 | 0.0290 | NA | NA | NA | NA | NA | NA | NA | NA | NA | NA | NA | NA |
| Brazil (11) v. Caribbean Sea (2) | 875.3034 | 0.8432 | 0.0001 | 0.4276 | NA | NA | NA | NA | NA | NA | NA | NA | NA | NA | NA | NA |
| Brazil (11) v. Papua New Guinea (2) | 7747.9949 | 0.0130 | 0.0099 | 0.0130 | NA | NA | NA | NA | NA | NA | NA | NA | NA | NA | NA | NA |
| Brazil (11) v. Sierra Leone (1) | 6002.3001 | 0.0919 | 0.0065 | 0.0919 | NA | NA | NA | NA | NA | NA | NA | NA | NA | NA | NA | NA |
| Caribbean Sea (2) v. Japan (11) | 7843.8015 | 0.0110 | 0.0112 | 0.0110 | NA | NA | NA | NA | NA | NA | NA | NA | NA | NA | NA | NA |
| Caribbean Sea (2) v. Sierra Leone (1) | 1968.9750 | 0.3516 | 0.0064 | 0.3516 | NA | NA | NA | NA | NA | NA | NA | NA | NA | NA | NA | NA |
| Caribbean Sea (2) v. South Africa (9) | 6487.9082 | 0.0130 | 0.0099 | 0.0130 | NA | NA | NA | NA | NA | NA | NA | NA | NA | NA | NA | NA |
| Fiji (8) v. Papua New Guinea (2) | 1236.9172 | 0.2418 | 0.0004 | 0.0709 | NA | NA | NA | NA | NA | NA | NA | NA | NA | NA | NA | NA |
| Fiji (8) v. Sierra Leone (1) | 1668.9862 | 0.0879 | 0.0010 | 0.0879 | NA | NA | NA | NA | NA | NA | NA | NA | NA | NA | NA | NA |
| Gulf of Mexico (11) v. Japan (11) | 14700.8676 | 0.0010 | 0.0114 | 0.0010 | NA | NA | NA | NA | NA | NA | NA | NA | NA | NA | NA | NA |
| Gulf of Mexico (11) v. Sierra Leone (1) | 6326.3966 | 0.0769 | 0.0065 | 0.0769 | NA | NA | NA | NA | NA | NA | NA | NA | NA | NA | NA | NA |
| Gulf of Mexico (11) v. South Africa (9) | 12895.7845 | 0.0010 | 0.0101 | 0.0010 | NA | NA | NA | NA | NA | NA | NA | NA | NA | NA | NA | NA |
| Indonesia (4) v. Reunion (9) | 1491.6003 | 0.0280 | 0.0003 | 0.0230 | NA | NA | NA | NA | NA | NA | NA | NA | NA | NA | NA | NA |
| Indonesia (4) v. Sierra Leone (1) | 1182.9341 | 0.2208 | 0.0008 | 0.2208 | NA | NA | NA | NA | NA | NA | NA | NA | NA | NA | NA | NA |
| Japan (11) v. Sierra Leone (1) | 2129.3009 | 0.0879 | 0.0010 | 0.0879 | NA | NA | NA | NA | NA | NA | NA | NA | NA | NA | NA | NA |
| Mozambique (8) v. Sierra Leone (1) | 1552.4615 | 0.1139 | 0.0009 | 0.1139 | NA | NA | NA | NA | NA | NA | NA | NA | NA | NA | NA | NA |
| Papua New Guinea (2) v. Reunion (9) | 1132.4893 | 0.8432 | 0.0003 | 0.4246 | NA | NA | NA | NA | NA | NA | NA | NA | NA | NA | NA | NA |
| Papua New Guinea (2) v. Seychelles (10) | 1233.2102 | 0.4236 | 0.0003 | 0.4216 | NA | NA | NA | NA | NA | NA | NA | NA | NA | NA | NA | NA |
| Papua New Guinea (2) v. South Africa (9) | 1088.8191 | 0.9790 | 0.0002 | 0.9031 | NA | NA | NA | NA | NA | NA | NA | NA | NA | NA | NA | NA |
| Papua New Guinea (2) v. Sri Lanka (4) | 906.5828 | 0.4565 | 0.0003 | 0.3187 | NA | NA | NA | NA | NA | NA | NA | NA | NA | NA | NA | NA |
| Papua New Guinea (2) v. Thailand (4) | 899.6582 | 0.8062 | 0.0003 | 0.4665 | NA | NA | NA | NA | NA | NA | NA | NA | NA | NA | NA | NA |
| Reunion (9) v. Sierra Leone (1) | 1677.6190 | 0.0969 | 0.0009 | 0.0969 | NA | NA | NA | NA | NA | NA | NA | NA | NA | NA | NA | NA |
| Reunion (9) v. South Africa (9) | 1586.2125 | 0.0799 | 0.0001 | 0.0160 | NA | NA | NA | NA | NA | NA | NA | NA | NA | NA | NA | NA |
| Reunion (9) v. Thailand (4) | 1307.6413 | 0.3137 | 0.0002 | 0.2308 | NA | NA | NA | NA | NA | NA | NA | NA | NA | NA | NA | NA |
| Seychelles (10) v. Sierra Leone (1) | 1803.8697 | 0.0859 | 0.0008 | 0.0859 | NA | NA | NA | NA | NA | NA | NA | NA | NA | NA | NA | NA |
| Sierra Leone (1) v. South Africa (9) | 1691.0264 | 0.0929 | 0.0008 | 0.0929 | NA | NA | NA | NA | NA | NA | NA | NA | NA | NA | NA | NA |
| Sierra Leone (1) v. Sri Lanka (4) | 1113.7442 | 0.2068 | 0.0008 | 0.2068 | NA | NA | NA | NA | NA | NA | NA | NA | NA | NA | NA | NA |
| Sierra Leone (1) v. Thailand (4) | 1135.4761 | 0.2068 | 0.0008 | 0.2068 | NA | NA | NA | NA | NA | NA | NA | NA | NA | NA | NA | NA |
| Sierra Leone (1) v. Western North Atlantic (8) | 4968.4411 | 0.1059 | 0.0066 | 0.1059 | NA | NA | NA | NA | NA | NA | NA | NA | NA | NA | NA | NA |

### 6.3.4 PCA

```
pca1 <- adegenet::glPca(BS.dartseq.baits.gl, nf = 5, parallel = TRUE,
                        n.cores = parallel::detectCores() - 1)
save(pca1, file = "DArTseq_ALL_baits_PCA.Rdata")
```

```
load("DArTseq_ALL_baits_PCA.Rdata")
pop.levels <- c("Costa Rica", "Brazil", "Caribbean Sea", "Gulf of Mexico",
                "Western North Atlantic", "Sierra Leone", "South Africa",
                "Mozambique", "Reunion" , "Seychelles", "Arabian Sea", "Sri Lanka",
                "Thailand", "Indonesia", "Papua New Guinea" , "Australia",
                "Japan", "Fiji")
BS.dartseq.baits.gl$pop <- factor(BS.dartseq.baits.gl$other$ind.metrics$Site,
                                  levels = pop.levels)

BS.pca.scores <- as.data.frame(pca1$scores)
BS.pca.scores$pop <- pop(BS.dartseq.baits.gl)
cols <- adegenet::funky(nPop(BS.dartseq.baits.gl))


p <- ggplot2::ggplot(BS.pca.scores, ggplot2::aes(x = PC1, y = PC2, colour = pop)) +
  ggplot2::geom_point(size = 2) + ggplot2::scale_color_manual(values = cols) +
  ggplot2::geom_hline(yintercept = 0) + ggplot2::geom_vline(xintercept = 0) +
  ggplot2::theme_minimal() +
  ggplot2::theme(
    axis.text = ggplot2::element_text(size = 5),
    axis.title.x = ggplot2::element_text(size = 10),
    axis.title.y = ggplot2::element_text(size = 10),
    legend.text = ggplot2::element_text(size = 7)
  )
p
```

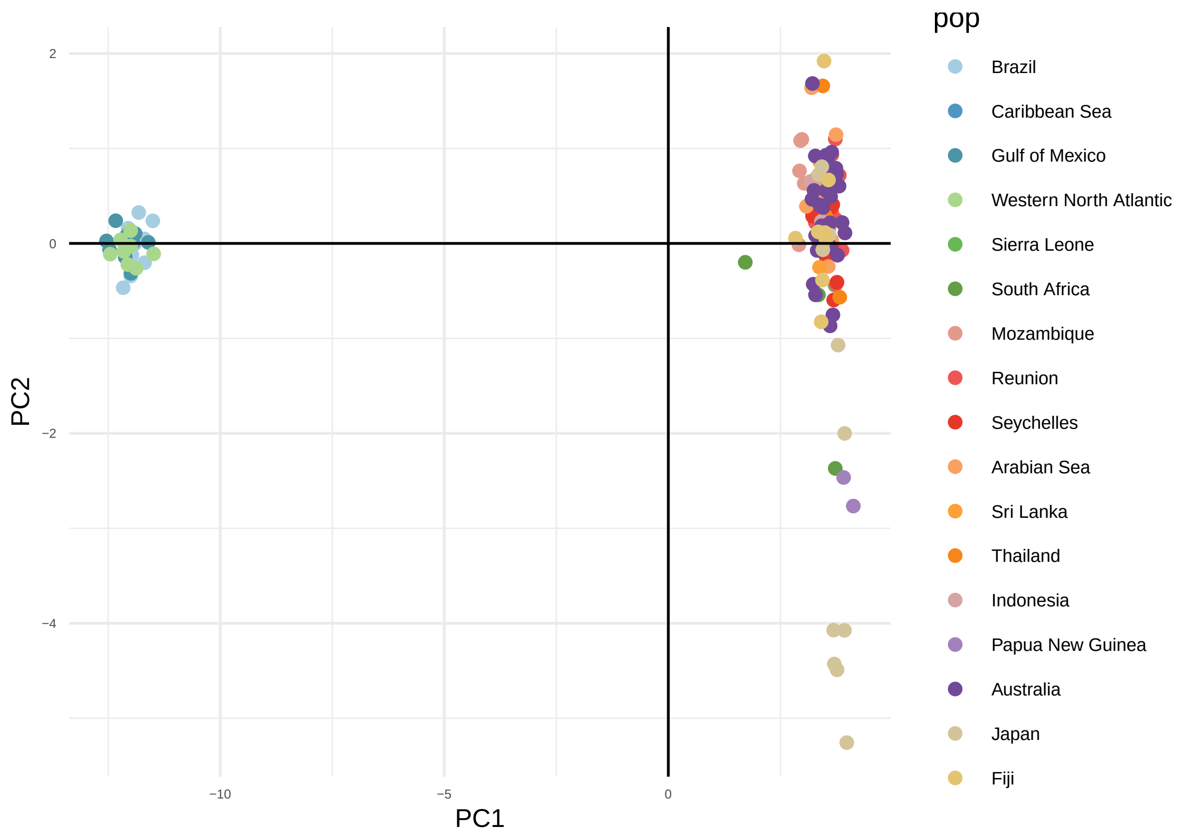

```r
ggplot2::ggsave(p, filename = "DArTseq_ALL_baits_PCA1.png", width = 30,
                height = 15,units = "cm")


p <-
  ggplot2::ggplot(BS.pca.scores, ggplot2::aes(x = PC1, y = PC3, colour = pop)) +
  ggplot2::geom_point(size = 2) + ggplot2::scale_color_manual(values = cols) +
  ggplot2::geom_hline(yintercept = 0) + ggplot2::geom_vline(xintercept = 0) +
  ggplot2::theme_minimal() +
  ggplot2::theme(
    axis.text = ggplot2::element_text(size = 5),
    axis.title.x = ggplot2::element_text(size = 10),
    axis.title.y = ggplot2::element_text(size = 10),
    legend.text = ggplot2::element_text(size = 7)
  )
p
```

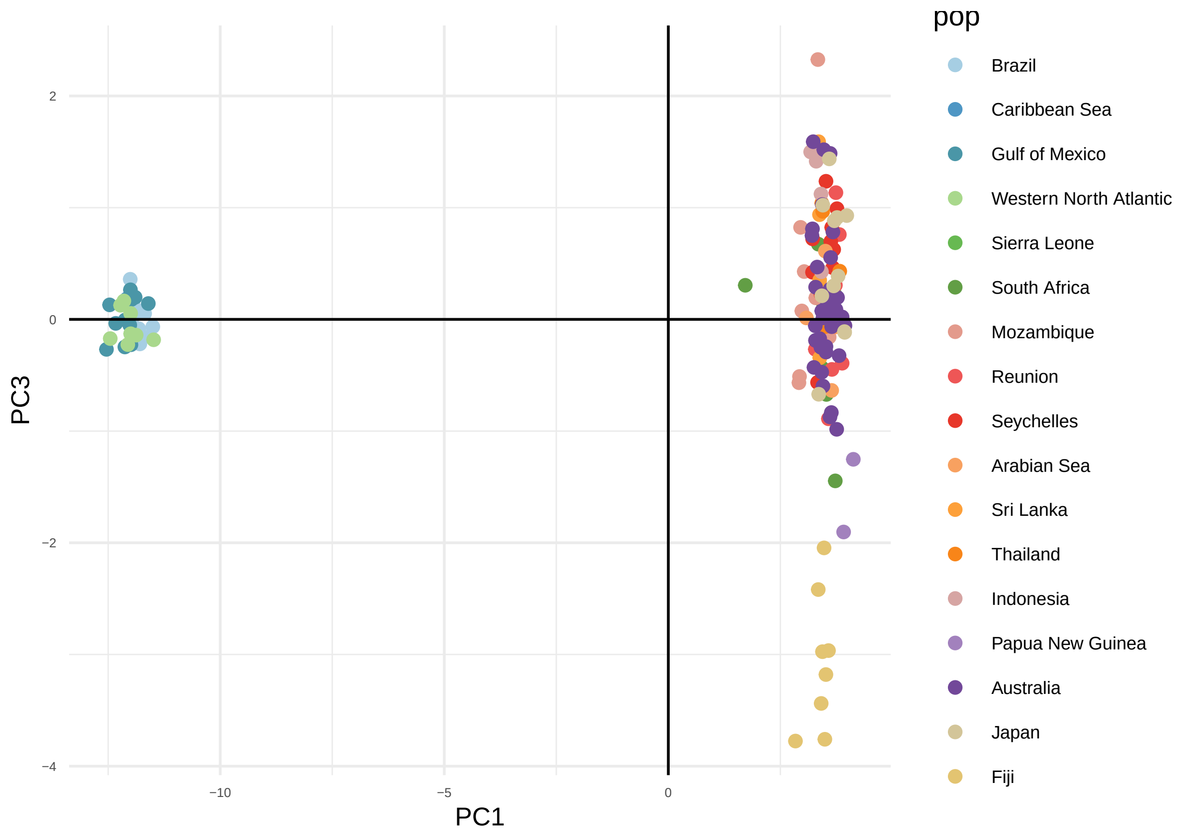


```r
ggplot2::ggsave(p, filename = "DArTseq_ALL_baits_PCA2.png", width = 30,
                height = 15,units = "cm")

p <-
  ggplot2::ggplot(BS.pca.scores, ggplot2::aes(x = PC2, y = PC3, colour = pop)) +
  ggplot2::geom_point(size = 2) + ggplot2::scale_color_manual(values = cols) +
  ggplot2::geom_hline(yintercept = 0) + ggplot2::geom_vline(xintercept = 0) +
  ggplot2::theme_minimal() +
  ggplot2::theme(
    axis.text = ggplot2::element_text(size = 5),
    axis.title.x = ggplot2::element_text(size = 10),
    axis.title.y = ggplot2::element_text(size = 10),
    legend.text = ggplot2::element_text(size = 7)
  )
```

```
p
```

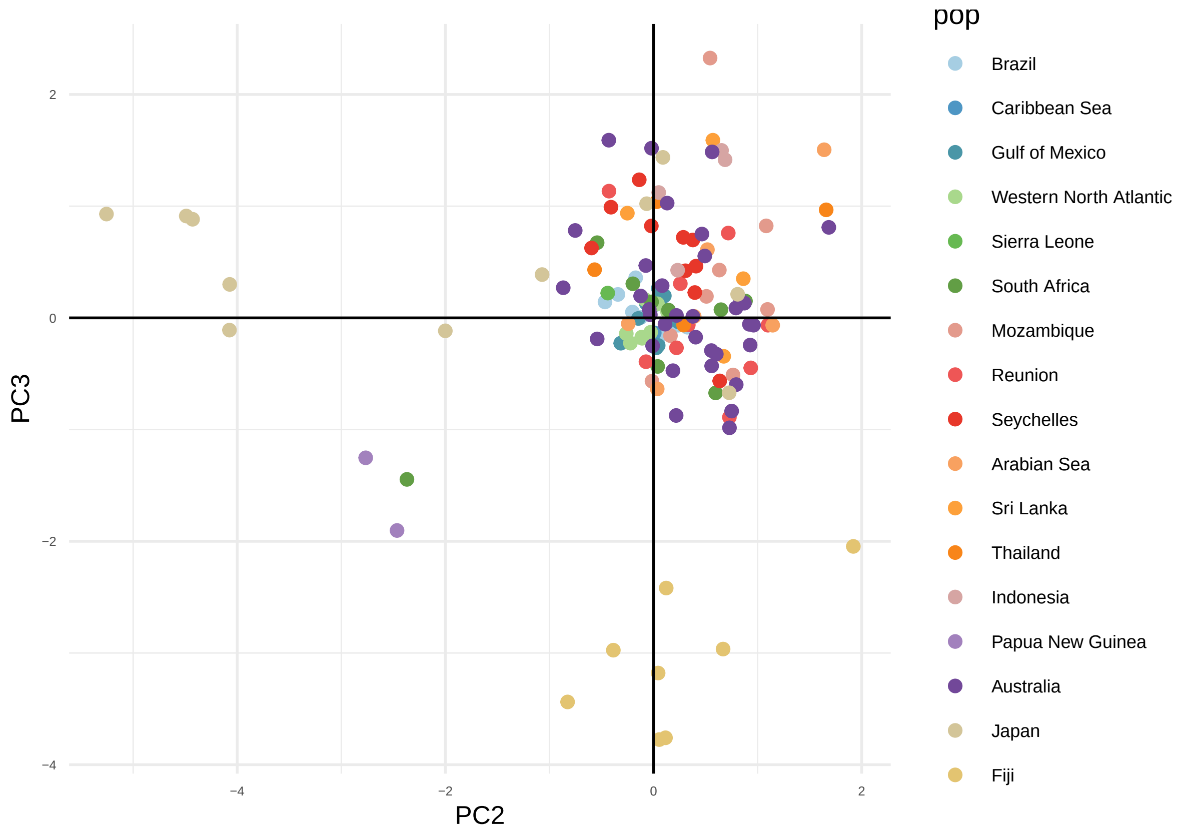


```
ggplot2::ggsave(p, filename = "DArTseq_ALL_baits_PCA3.png", width = 30,
                height = 15,units = "cm")


p <-
  ggplot2::ggplot(BS.pca.scores, ggplot2::aes(x = PC3, y = PC4, colour = pop)) +
  ggplot2::geom_point(size = 2) + ggplot2::scale_color_manual(values = cols) +
  ggplot2::geom_hline(yintercept = 0) + ggplot2::geom_vline(xintercept = 0) +
  ggplot2::theme_minimal() +
  ggplot2::theme(
    axis.text = ggplot2::element_text(size = 5),
    axis.title.x = ggplot2::element_text(size = 10),
    axis.title.y = ggplot2::element_text(size = 10),
    legend.text = ggplot2::element_text(size = 7)
  )
p
```

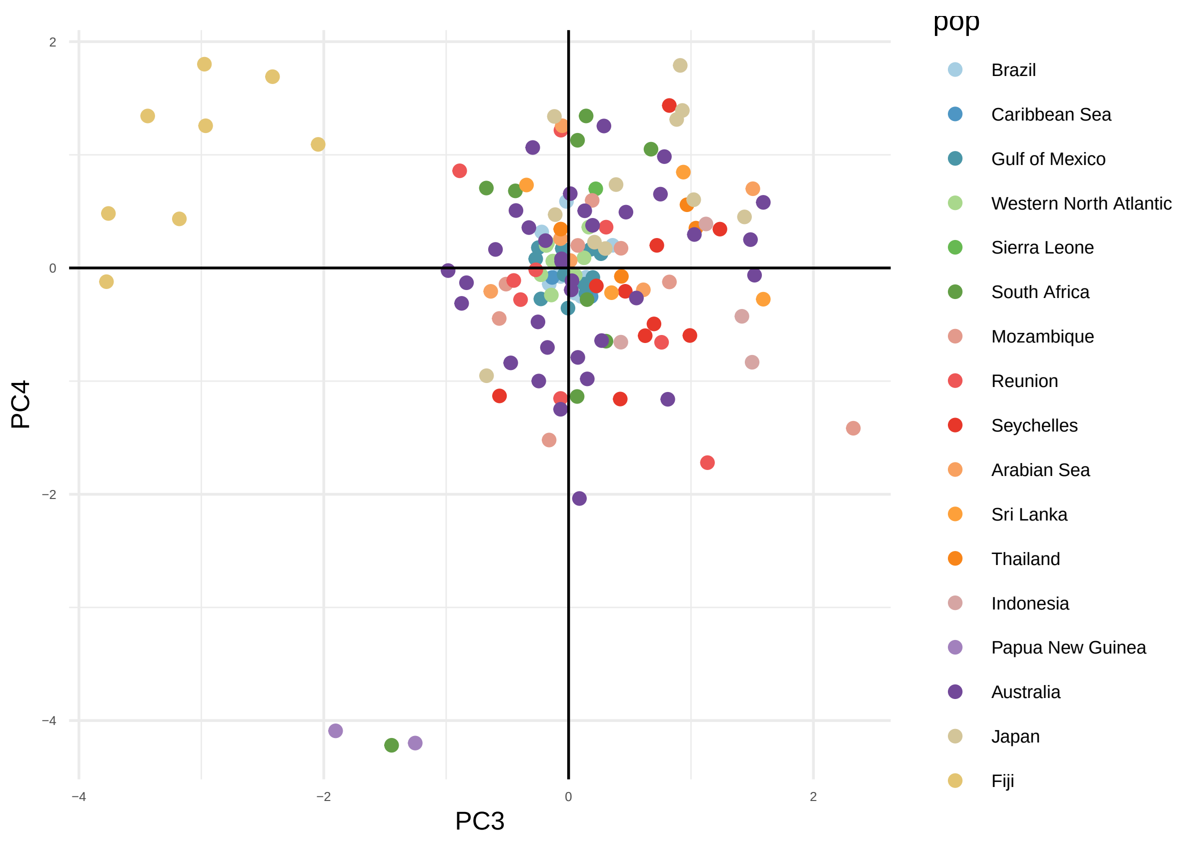


```r
ggplot2::ggsave(p, filename = "DArTseq_ALL_baits_PCA4.png", width = 30,
                height = 15,units = "cm")

p <-
  ggplot2::ggplot(BS.pca.scores, ggplot2::aes(x = PC3, y = PC5, colour = pop)) +
  ggplot2::geom_point(size = 2) + ggplot2::scale_color_manual(values = cols) +
  ggplot2::geom_hline(yintercept = 0) + ggplot2::geom_vline(xintercept = 0) +
  ggplot2::theme_minimal() +
  ggplot2::theme(
    axis.text = ggplot2::element_text(size = 5),
    axis.title.x = ggplot2::element_text(size = 10),
    axis.title.y = ggplot2::element_text(size = 10),
    legend.text = ggplot2::element_text(size = 7)
  )
p
```

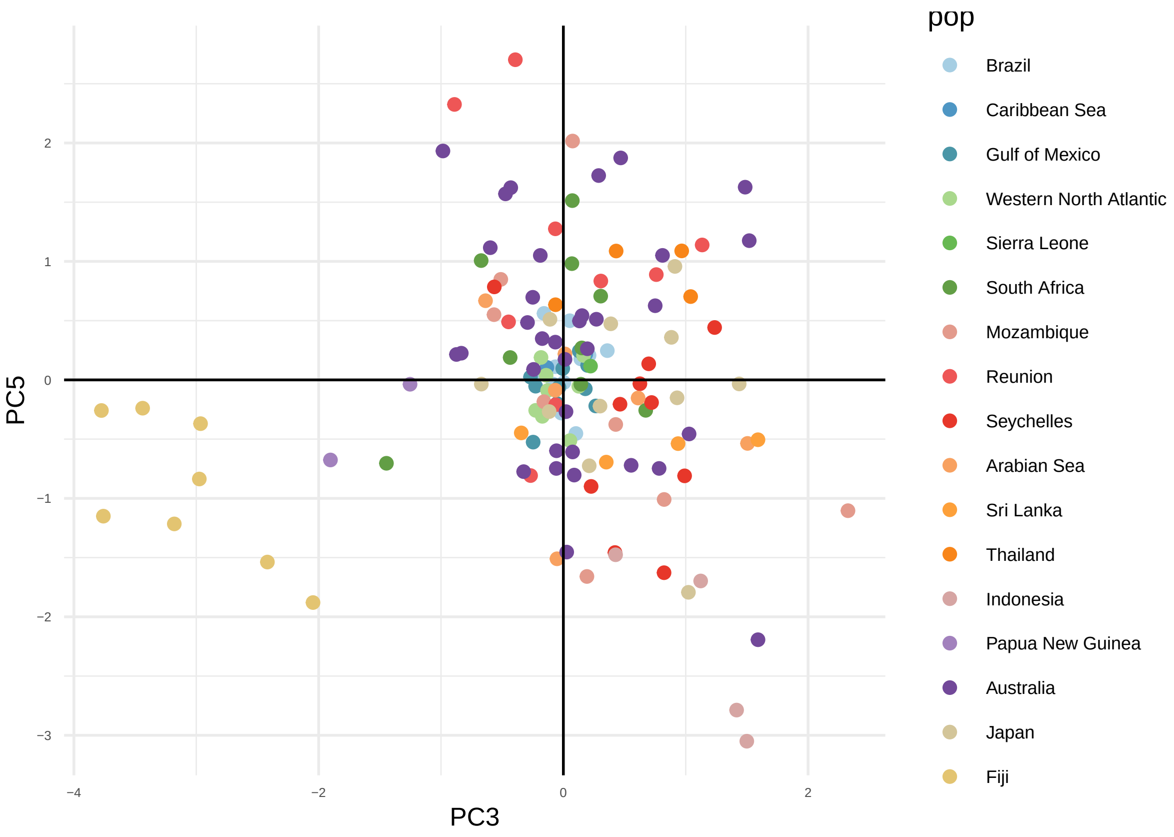


```
ggplot2::ggsave(p, filename = "DArTseq_ALL_baits_PCA5.png", width = 30,
                height = 15,units = "cm")
```

---

# 7 DATA1: DArTcap - All data

5 samples failed library construction

## 7.1 Set strata

```
targetid <- readr::read_tsv("DArTcap_target_id.tsv")

strata <- readr::read_csv(metafile) %>%
  dplyr::left_join(targetid, by = "INDIVIDUALS") %>%
  dplyr::filter(!is.na(TARGET_ID)) %>%
  dplyr::mutate(STRATA = Site2)
strata$INDIVIDUALS <- with(strata, make.unique(as.character(INDIVIDUALS)))
# readr::write_tsv(strata, path = "Bull_shark_DArTcap_strata.tsv")
```

## 7.2 Test baiting success

```
baits <- readr::read_tsv("Total_selected_markers.tsv")
```

```
dartcap.data <- "SEQ_SNPs_counts_0_Target_merge.csv"
strata <- "Bull_shark_DArTcap_strata.tsv"
BSsnp <- radiator::read_dart(data = dartcap.data, strata = strata,tidy.dart = TRUE,
                             parallel.core = parallel::detectCores() - 1)
BS.dartcap.raw.tidy <- BSsnp$tidy.data
length(unique(BS.dartcap.raw.tidy$INDIVIDUALS)) #942
length(unique(BS.dartcap.raw.tidy$MARKERS)) #19549
save(BSsnp, file = "DArTcap_Raw_tidy.Rdata")
sum(unique(baits$CloneID) %in% unique(BS.dartcap.raw.tidy$LOCUS)) #453
```

## 7.3 Find sex-linked markers

```
data <- "SEQ_SNPs_counts_0_Target_merge.csv"
silicodata <- NULL
strata <- radiator::read_strata("Bull_shark_DArTcap_strata.tsv",
                                keep.two = FALSE)$strata
strata %<>% dplyr::rename(POP = "STRATA") %>%
  dplyr::rename(STRATA = "Sex")

sex.results <- radiator::sexy_markers(
  data = data,
  silicodata = silicodata,
  strata = strata,
  interactive.filter = TRUE)
```

Individual's QC stats

Missing genotypes outlier: 0.26892325
n. het markers in the bp range = 4435
n. het markers in the bp IQR = 109

Correlations:
total coverage & missing = −0.02
total coverage & mean coverage = 0.82
total coverage & heterozygosity = 0.03
missing & heterozygosity = −0.14

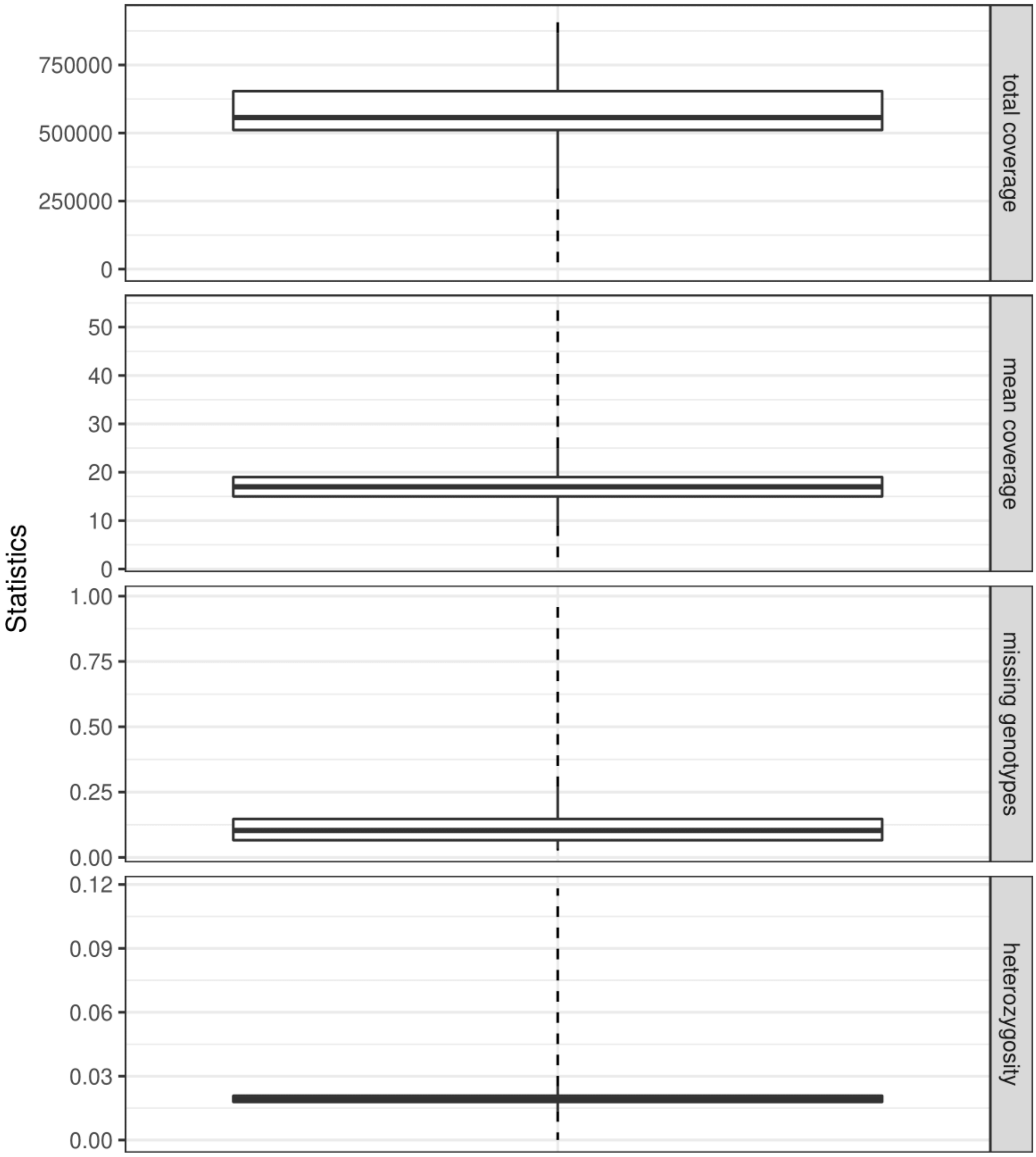

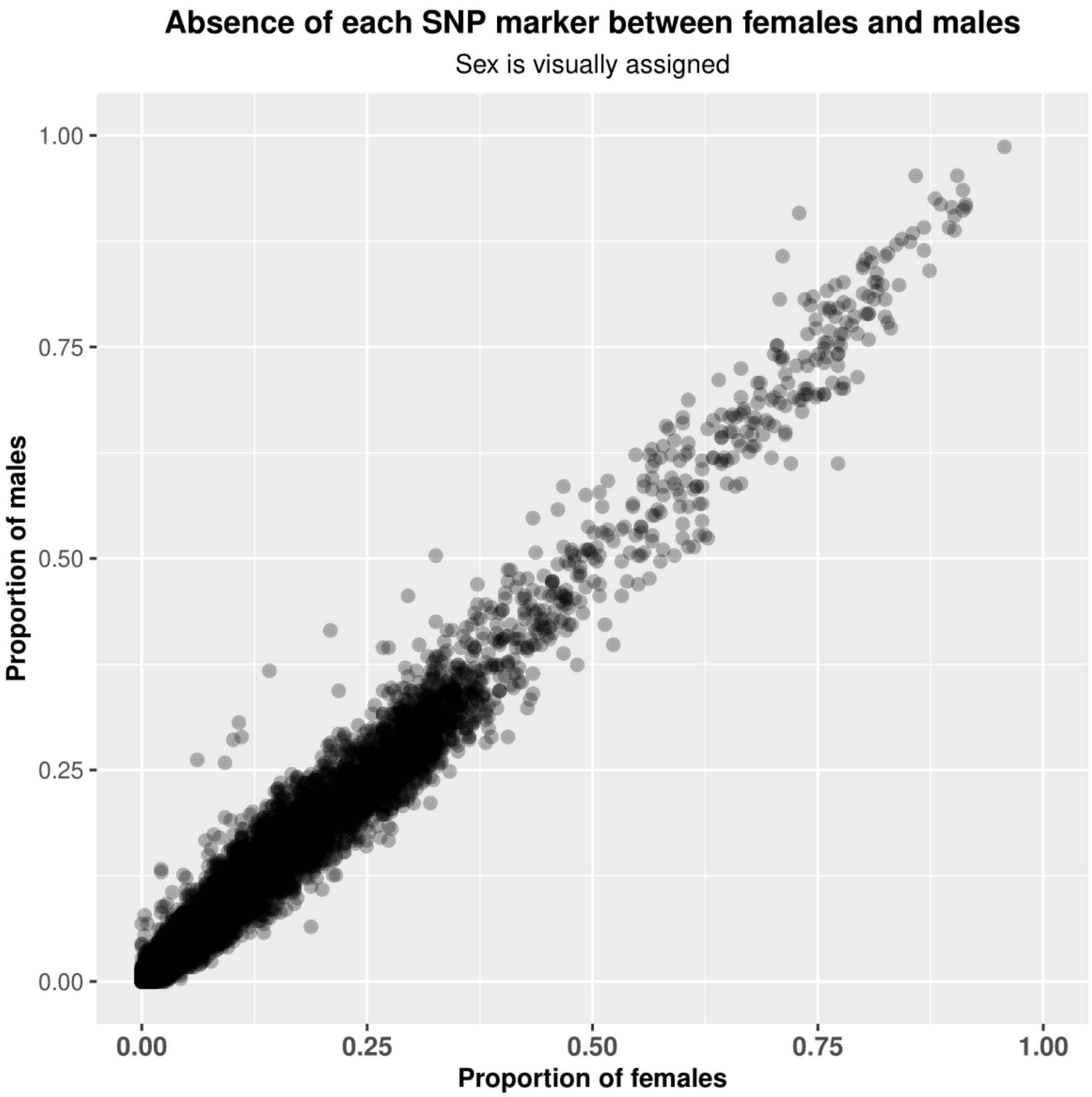
Absence of each SNP marker between females and males
Sex is visually assigned
Proportion of males
Proportion of females
0.00
0.25
0.50
0.75
1.00
0.00
0.25
0.50
0.75
1.00

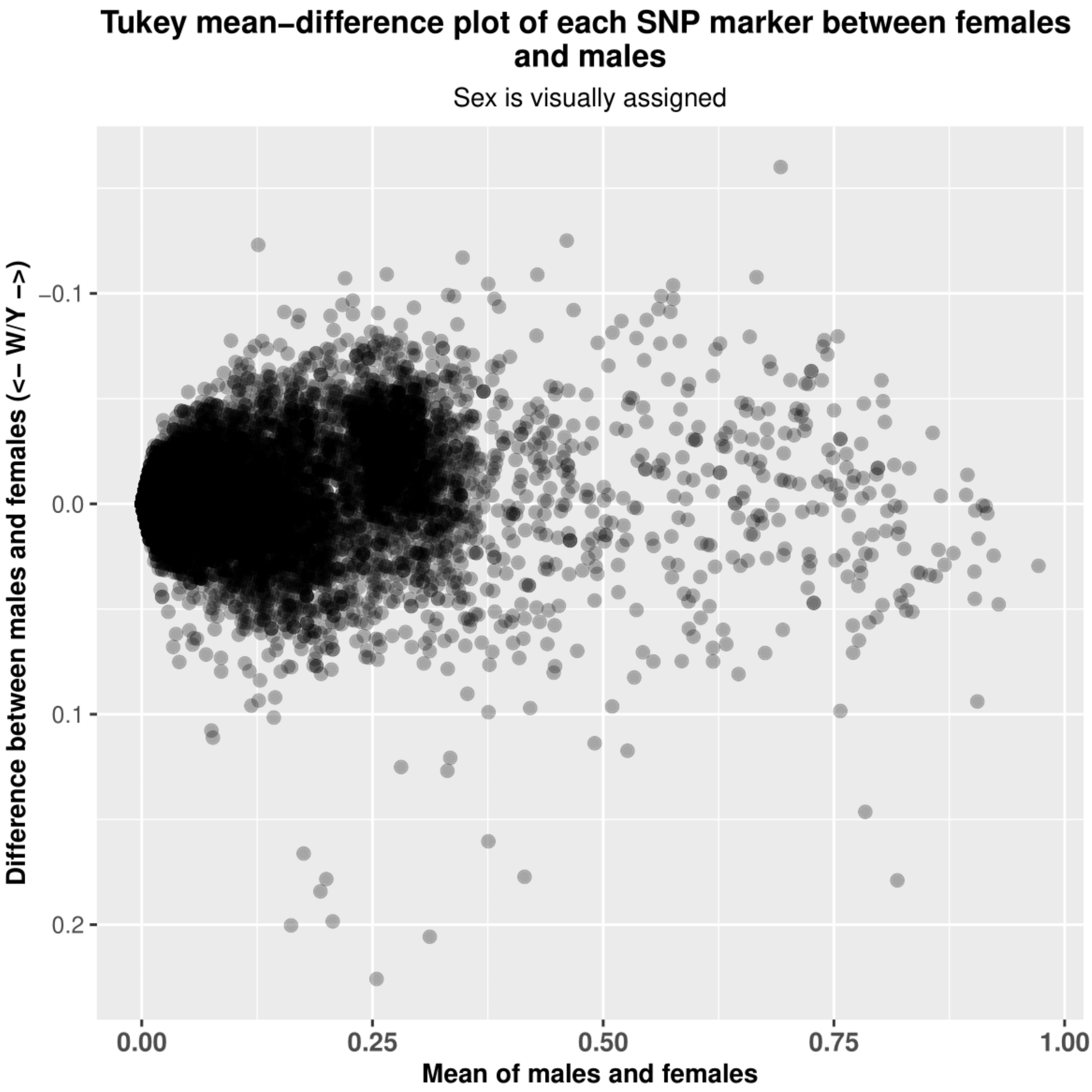

Tukey mean−difference plot of each SNP marker between females and males
Sex is visually assigned
Difference between males and females (<− W/Y −>)
−0.1
0.0
0.1
0.2
0.00
0.25
0.50
0.75
1.00
Mean of males and females

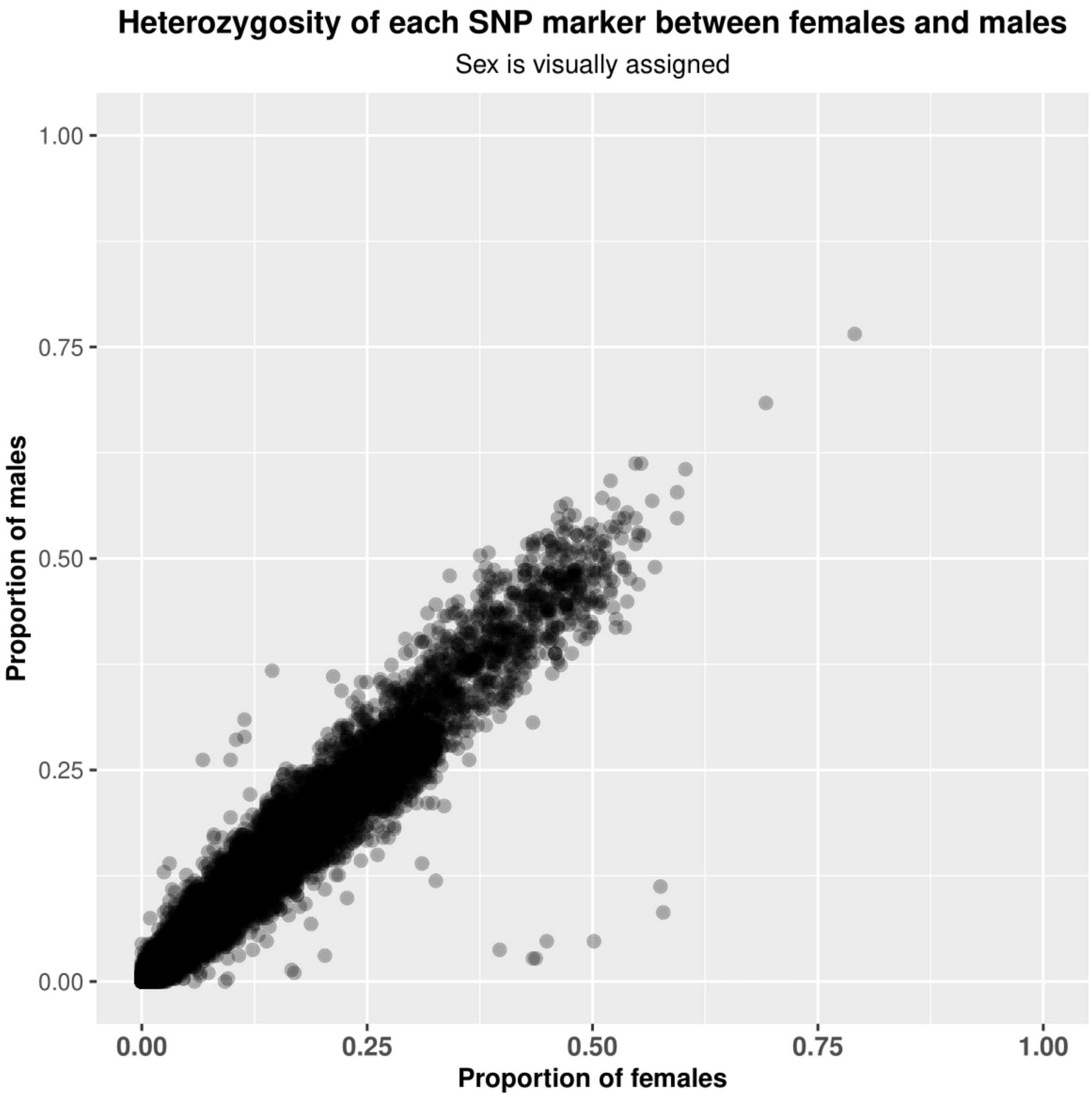

Heterozygosity of each SNP marker between females and males
Sex is visually assigned
1.00
0.75
0.50
0.25
0.00
Proportion of males
0.00
0.25
0.50
0.75
1.00
Proportion of females

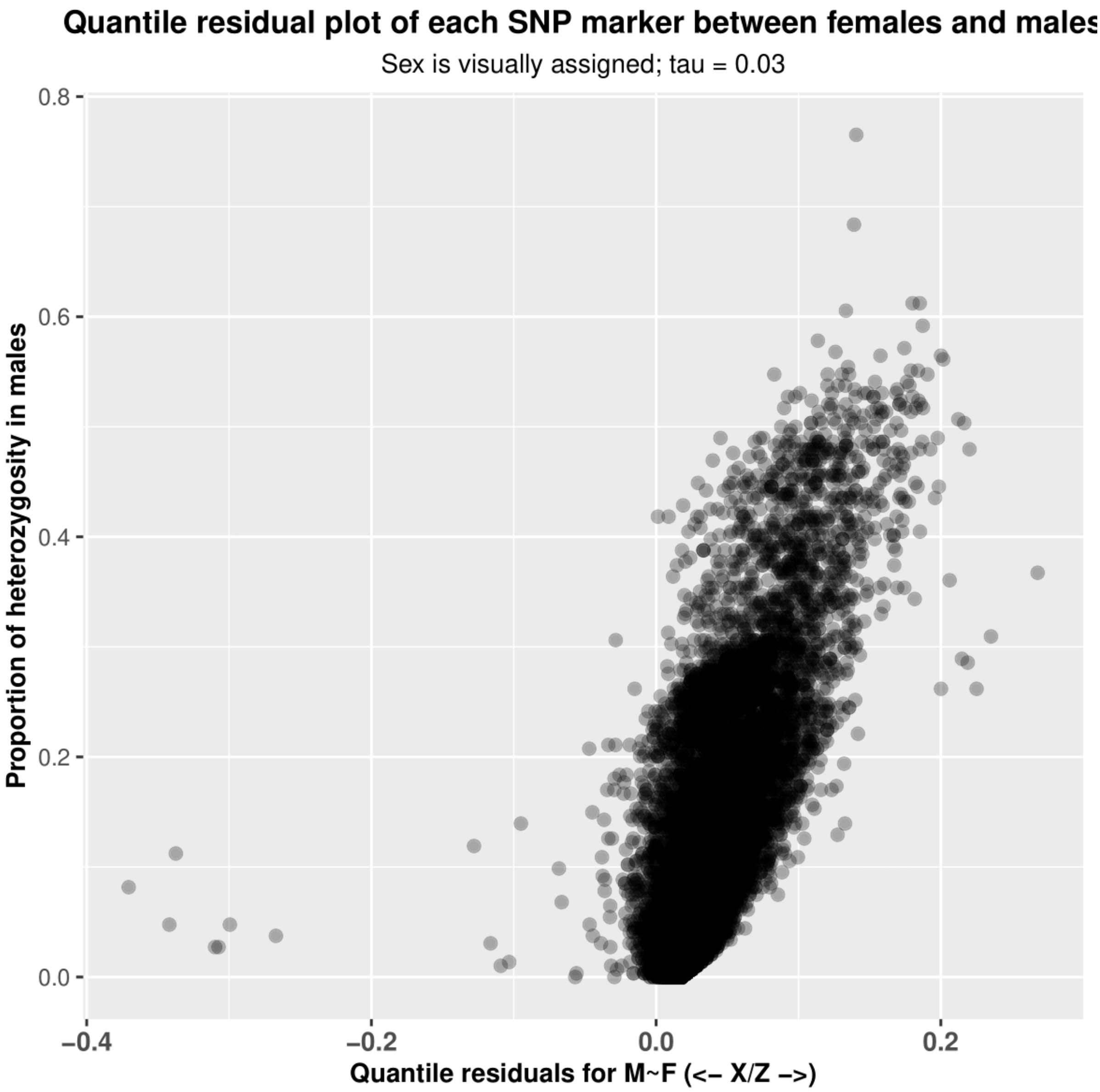
Quantile residual plot of each SNP marker between females and males
Sex is visually assigned; tau = 0.03
Proportion of heterozygosity in males
0.8
0.6
0.4
0.2
0.0
–0.4
–0.2
0.0
0.2
Quantile residuals for M~F (<– X/Z –>)

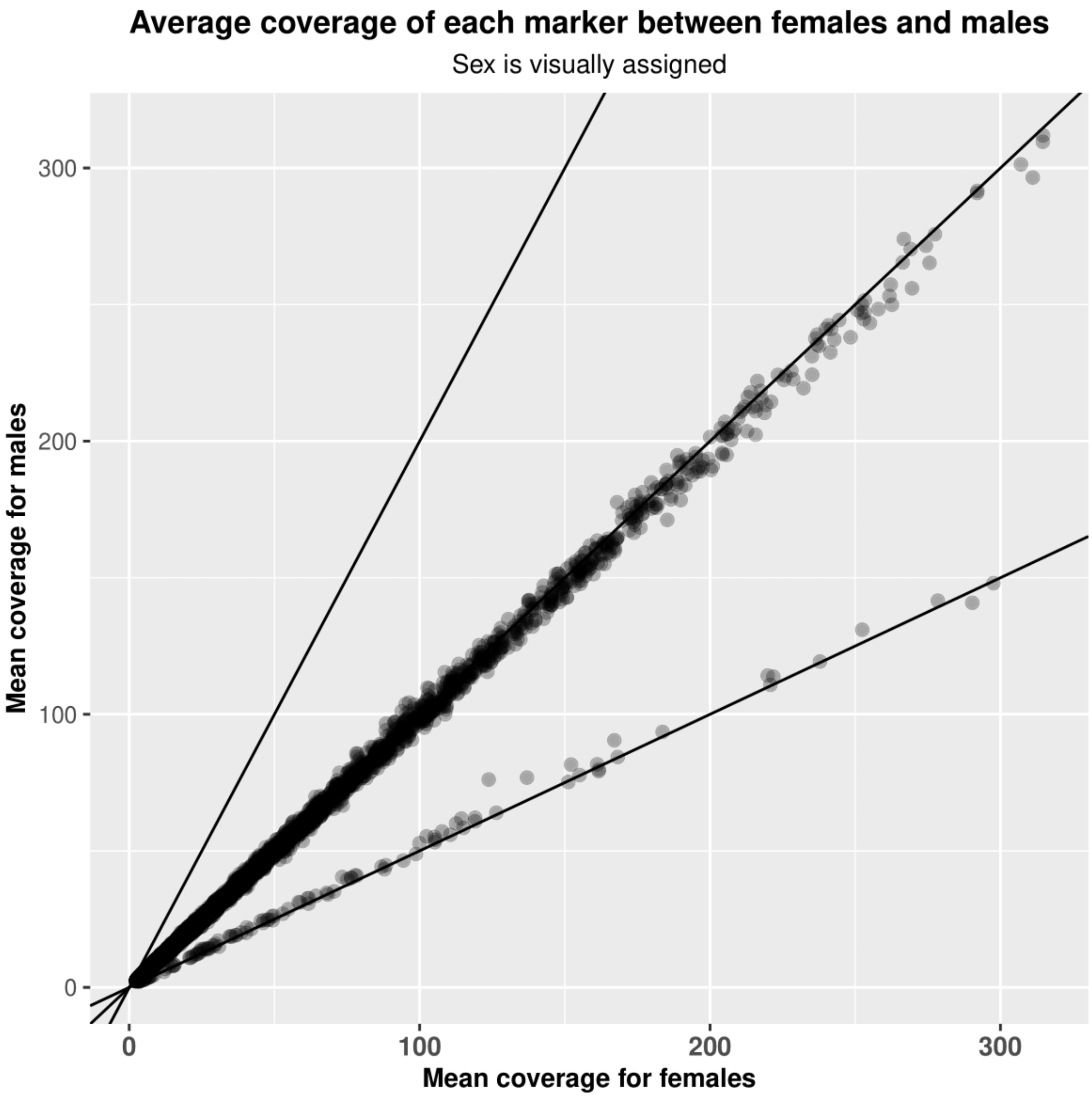
Average coverage of each marker between females and males
Sex is visually assigned
Mean coverage for males
Mean coverage for females
0
100
200
300
0
100
200
300

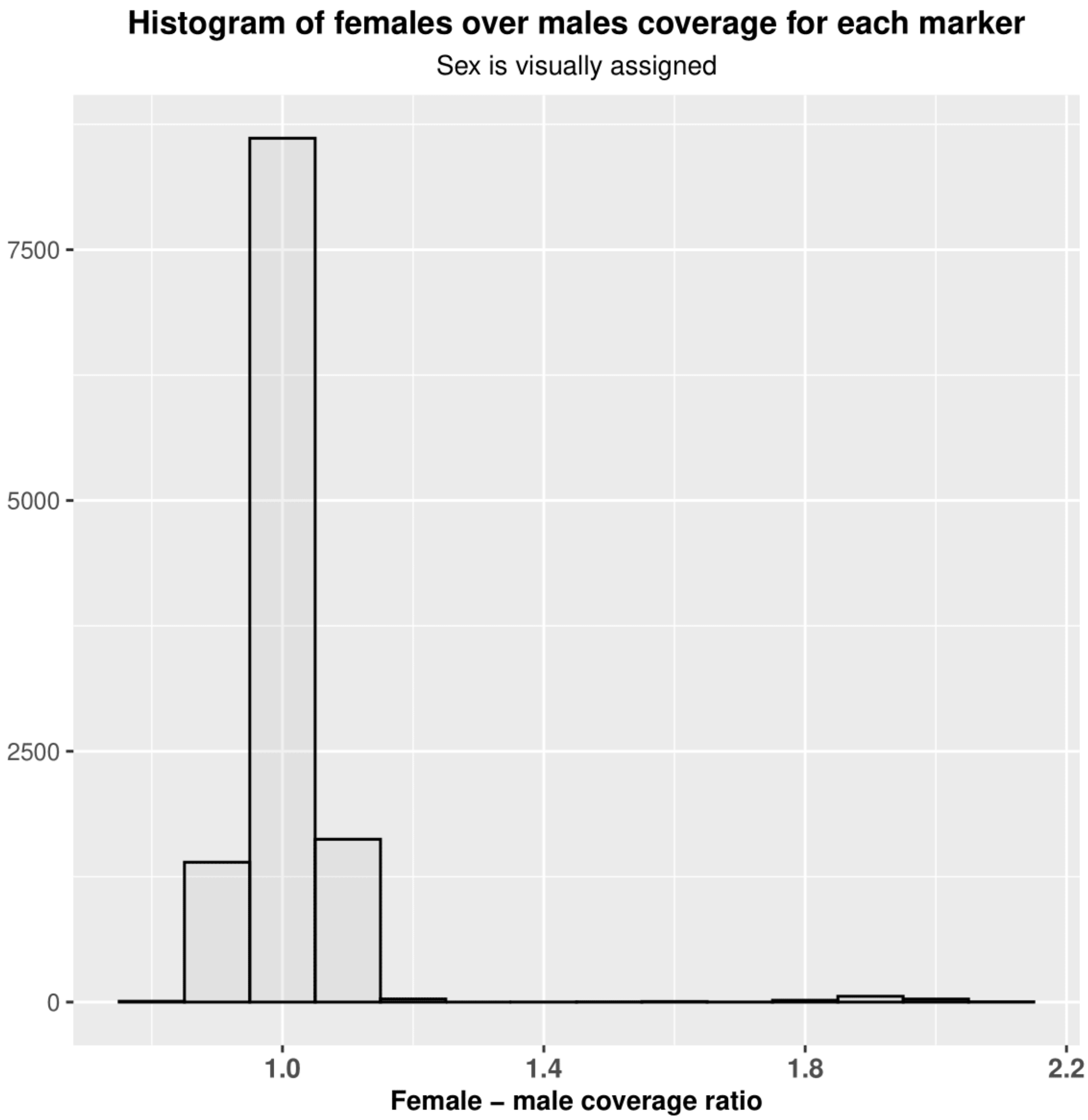
Histogram of females over males coverage for each marker
Sex is visually assigned
7500
5000
2500
0
1.0
1.4
1.8
2.2
Female – male coverage ratio

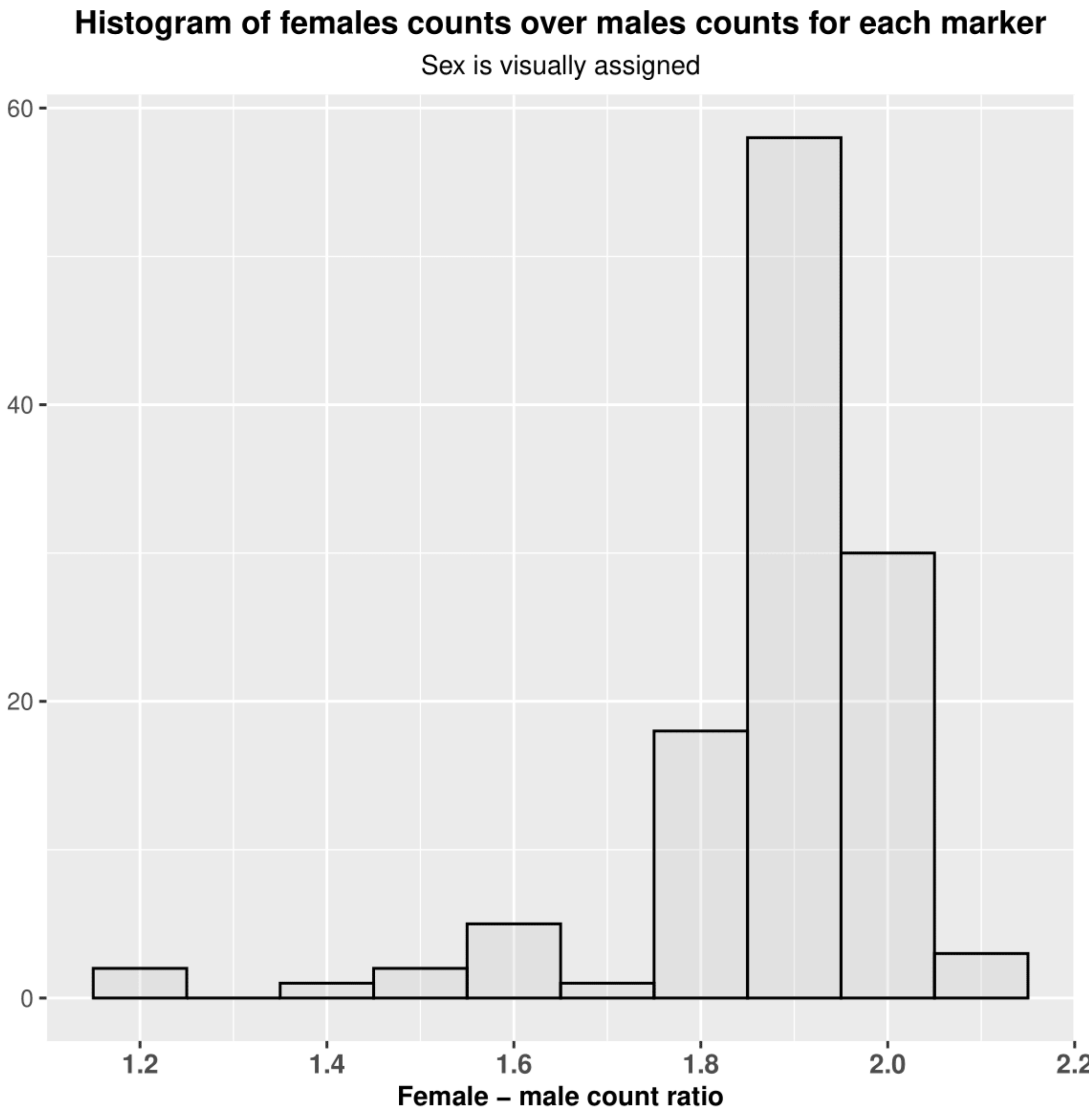
Histogram of females counts over males counts for each marker
Sex is visually assigned
60
40
20
0
1.2
1.4
1.6
1.8
2.0
2.2
Female – male count ratio

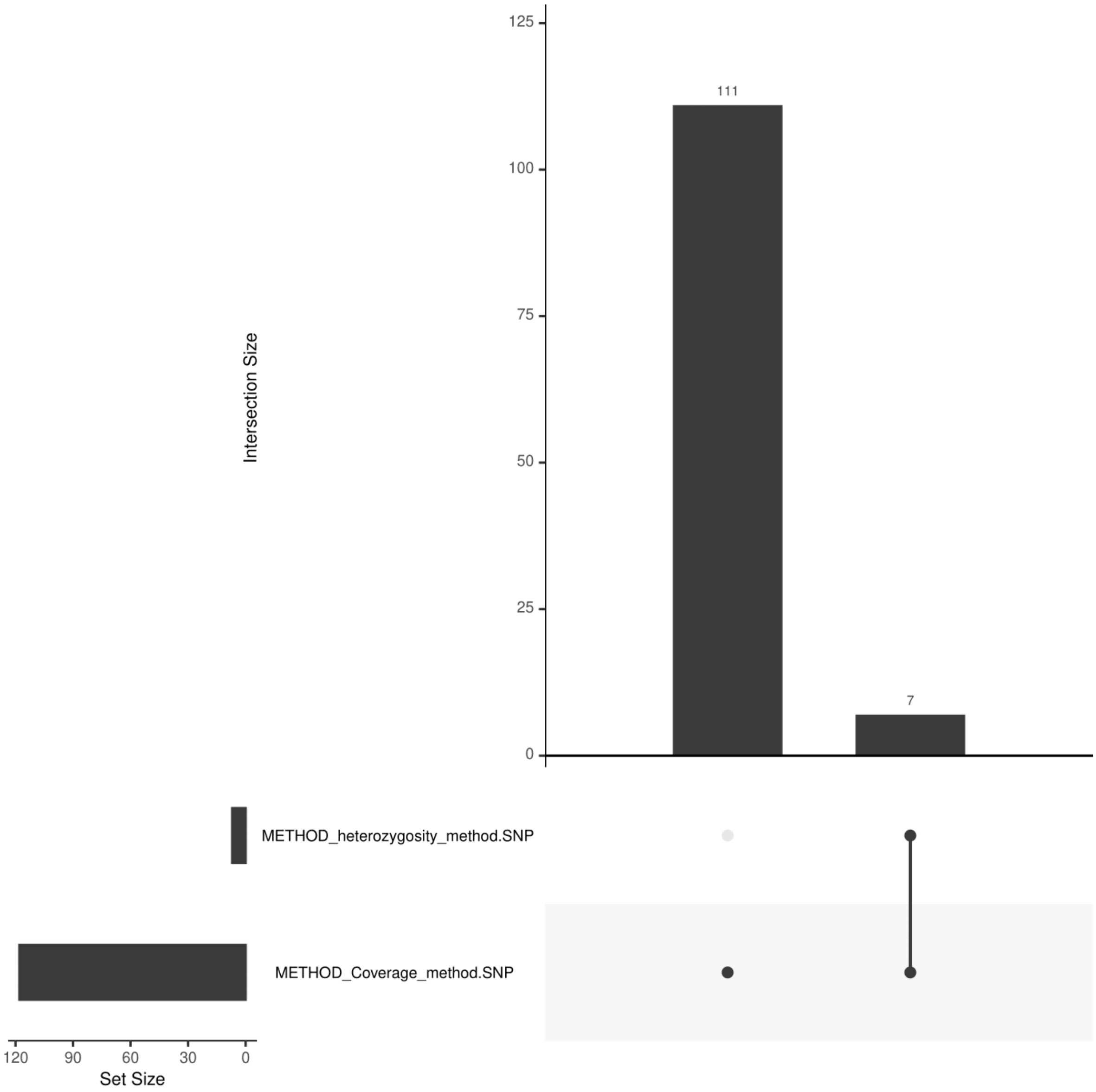


## 7.4 Filter data

1. Marker Reproducibility: 0.98
2. Individual missingness: 0.75•
3. Individual Het - low: n
4. Individual Het - high: n
5. Individual total coverage - low: 10,000
6. Individual total coverage - high: 1,000,000
7. Marker MAC - low: 4
8. Marker coverage - low: 4
9. Marker coverage - high:33
10. Marker maximum missing proportion: 0.1
11. Marker snp position on locus: n
12. Max 3 SNPs per locus
13. 1 SNP per locus according to mac
14. no LD filter
15. Individual Het - low: n
16. Individual Het - high: n
17. Duplicated individuals: n

18. No filter on HWE

```
dartcap.data <- "SEQ_SNPs_counts_0_Target_merge.csv"
strata <- "Bull_shark_DArTcap_strata.tsv"
BSsnp <- radiator::filter_rad(dartcap.data, strata,
                              parallel.core = parallel::detectCores() - 1)

BS.dartcap.SF.gds <- BSsnp$gds
BS.dartcap.SF.tidy <- BSsnp$output$tidy.data
print(BS.dartcap.SF.gds, expand = TRUE, all = TRUE, attribute = TRUE,
      attribute.trim = FALSE)
```

Table 10:

| FILTERS | VALUES | BEFORE | BLACKLIST |
|---|---|---|---|
| Filter DArT reproducibility | 0.98 | 1014 / 34 / 1 / 26335 / 37537 | 0 / 0 / 0 / 1043 / 2250 |
| Filter monomorphic markers | NA | 1014 / 34 / 1 / 25292 / 35287 | 0 / 0 / 0 / 0 / 0 |
| Filter markers in common | NA | 1014 / 34 / 1 / 25292 / 35287 | 0 / 0 / 0 / 10291 / 17213 |
| Filter individuals based on missingness | 0.75 | 1014 / 34 / 1 / 15001 / 18074 | 8 / 0 / 0 / 0 / 0 |
| Filter individuals based on total coverage | 10000 1e+06 | 1006 / 34 / 1 / 15001 / 18074 | 5 / 0 / 0 / 0 / 0 |
| Filter monomorphic markers | NA | 1001 / 34 / 1 / 15001 / 18074 | 0 / 0 / 0 / 1306 / 1879 |
| Filter MAC | 4 | 1001 / 34 / 1 / 13695 / 16195 | 0 / 0 / 0 / 2050 / 3069 |
| Filter coverage min / max | 4-33 | 1001 / 34 / 1 / 11645 / 13126 | 0 / 0 / 0 / 5384 / 5816 |
| Filter genotyping | 0.1 | 1001 / 34 / 1 / 6261 / 7310 | 0 / 0 / 0 / 1166 / 1285 |
| Filter SNPs position on the read | all | 1001 / 34 / 1 / 5095 / 6025 | 0 / 0 / 0 / 0 / 0 |
| Filter markers snp number | 3 | 1001 / 34 / 1 / 5095 / 6025 | 0 / 0 / 0 / 33 / 143 |
| Filter short ld | mac | 1001 / 34 / 1 / 5062 / 5882 | 0 / 0 / 0 / 0 / 820 |
| detect mixed genomes | 0 1 | 1001 / 34 / 1 / 5062 / 5062 | 0 / 0 / 0 / 0 / 0 |
| detect duplicate genomes | 0 | 1001 / 34 / 1 / 5062 / 5062 | 0 / 0 / 0 / 0 / 0 |
| Filter HWE | NA | 1001 / 34 / 1 / 5062 / 5062 | 0 / 0 / 0 / 0 / 0 |

## 7.5 Save data

```
Convert <- radiator::genomic_converter(BS.dartcap.SF.gds,
                                       output = c("genlight", "stockr",
                                                  "pcadapt", "gtypes"))
BS.dartcap.SF.gl <- Convert$genlight
BS.dartcap.SF.stkr <- Convert$stockr
BS.dartcap.SF.pc <- Convert$pcadapt
BS.dartcap.SF.gt <- Convert$gtypes


markers.meta <- radiator::extract_markers_metadata(BS.dartcap.SF.gds, whitelist = TRUE)
individuals.meta <- radiator::extract_individuals_metadata(BS.dartcap.SF.gds, whitelist = TRUE)
BS.dartcap.SF.gl$other$ind.metrics <- individuals.meta
BS.dartcap.SF.gl$other$loc.metrics <- markers.meta
BS.dartcap.SF.gt@schemes <- individuals.meta

pop.levels <- c("Costa Rica" , "Brazil","Caribbean Sea","Gulf of Mexico",
                "Western North Atlantic", "Sierra Leone", "South Africa",
                "Mozambique", "Reunion" , "Seychelles", "Arabian Sea", "Sri Lanka",
                "Thailand", "Indonesia", "Papua New Guinea" , "Fitzroy River",
                "Durack River", "Daly River", "Adelaide River","Darwin Coastal",
                "South Alligator River", "East Alligator River","Blue mud Bay",
                "Roper River","Towns River","Unknown","Wenlock River","Trinity inlet",
                "Clarence River", "Sydney Harbour", "Okinawa", "Urauchi River", "Fiji")
BS.dartcap.SF.gl$pop <- factor(BS.dartcap.SF.gl$other$ind.metrics$Site2,
                               levels = pop.levels)
BS.dartcap.SF.gl <- BS.dartcap.SF.gl[order(BS.dartcap.SF.gl$pop,
                                           BS.dartcap.SF.gl$ind.names)]

save(BS.dartcap.SF.tidy, BS.dartcap.SF.gl, BS.dartcap.SF.stkr,
     BS.dartcap.SF.pc,BS.dartcap.SF.gt, markers.meta, individuals.meta,
     file = "DArTcap_ALL_SF_objects.Rdata")
```

## 7.6 Load data

```
load("DArTcap_ALL_SF_objects.Rdata")
pop.levels <- c("Costa Rica" , "Brazil","Caribbean Sea","Gulf of Mexico",
                "Western North Atlantic", "Sierra Leone", "South Africa",
                "Mozambique", "Reunion" , "Seychelles", "Arabian Sea", "Sri Lanka",
                "Thailand", "Indonesia", "Papua New Guinea" , "Fitzroy River",
                "Durack River", "Daly River", "Adelaide River","Darwin Coastal",
                "South Alligator River", "East Alligator River","Blue mud Bay",
                "Roper River","Towns River","Unknown","Wenlock River","Trinity inlet",
                "Clarence River", "Sydney Harbour", "Okinawa", "Urauchi River", "Fiji")

BS.dartcap.SF.gl$pop <- factor(BS.dartcap.SF.gl$other$ind.metrics$Site2,
                              levels = pop.levels)
BS.dartcap.SF.gl <- BS.dartcap.SF.gl[order(BS.dartcap.SF.gl$pop,
                                          BS.dartcap.SF.gl$ind.names)]

adegenet::nInd(BS.dartcap.SF.gl) # 1001
adegenet::nLoc(BS.dartcap.SF.gl) # 5065
summary(BS.dartcap.SF.gl$pop)
sum(duplicated(stringr::str_replace_all(pattern = "\\.1", replacement = "",
                                   string =  BS.dartcap.SF.gl$ind.names))) # 79 replicate left
sum(baits$CloneID %in% BS.dartcap.SF.gl$other$loc.metrics$LOCUS) #60
```

```
## [1] 1001
## [1] 5062
##            Costa Rica                 Brazil          Caribbean Sea
##                    34                     60                      2
##        Gulf of Mexico Western North Atlantic           Sierra Leone
##                    52                      9                      1
##          South Africa             Mozambique                Reunion
##                    31                     14                     29
##            Seychelles            Arabian Sea              Sri Lanka
##                    42                     43                     23
##              Thailand              Indonesia       Papua New Guinea
##                     8                      8                     17
##         Fitzroy River           Durack River             Daly River
##                     7                      4                     52
##        Adelaide River         Darwin Coastal  South Alligator River
##                    23                     18                     95
##  East Alligator River           Blue mud Bay            Roper River
##                    55                     18                      9
##           Towns River                Unknown          Wenlock River
##                    13                     12                     32
##         Trinity inlet         Clarence River         Sydney Harbour
##                     5                    123                     78
##               Okinawa          Urauchi River                   Fiji
##                    10                     42                     32
## [1] 79
## [1] 60
```

## 7.7 Remove sex-linked markers

```
load("Sex_results.Rdata")
sum(BS.dartcap.SF.gl$loc.names %in% bull_shark$sexy.summary$CLONE_ID ) #12
sex.markers <-  bull_shark$sexy.summary$CLONE_ID

locnames <- BS.dartcap.SF.gl$loc.names
```

```
BS.dartcap.SF.gl <- BS.dartcap.SF.gl[,!locnames %in% sex.markers]
BS.dartcap.SF.gl$other$loc.metrics <- BS.dartcap.SF.gl$other$loc.metrics[!locnames %in%
                                                                          sex.markers,]

sum(sex.markers %in% unique(BS.dartcap.SF.tidy$LOCUS)) # 12
BS.dartcap.SF.tidy <- BS.dartcap.SF.tidy[!BS.dartcap.SF.tidy$LOCUS %in%
                                            sex.markers,]
```

```
## [1] 12
## [1] 18
```

## 7.8 PCA to identify species

```
pca1 <- adegenet::glPca(BS.dartcap.SF.gl, nf = 8, parallel = TRUE,
                        n.cores = parallel::detectCores() - 1)
save(pca1, file = "DArTcap_SF_SpeciesID_PCA.Rdata")
```

```
load("DArTcap_SF_SpeciesID_PCA.Rdata")

BS.pca.scores <- as.data.frame(pca1$scores)
BS.pca.scores$pop <- pop(BS.dartcap.SF.gl)
BS.pca.scores$species <- BS.dartcap.SF.gl$other$ind.metrics$SpeciesID_genetic
cols <- adegenet::funky(nPop(BS.dartcap.SF.gl))


p <- ggplot2::ggplot(BS.pca.scores, ggplot2::aes(x = PC1, y = PC2, colour = pop,
                                                 group = species)) +
  ggplot2::geom_point(aes(shape = species), size = 2) +
  ggplot2::scale_color_manual(values = cols) +
  ggplot2::scale_shape_manual(values = c(1,2,3,4,5,6,8,9)) +
  ggplot2::geom_hline(yintercept = 0) +
  ggplot2::geom_vline(xintercept = 0) +
  ggplot2::theme(panel.background = ggplot2::element_rect(fill = "white",
                                                          colour = "white"),
                 panel.grid.major = ggplot2::element_line(size = 0.5,
                                                          linetype = 'solid',
                                                          colour = "grey"),
                 panel.grid.minor = ggplot2::element_line(size = 0.1,
                                                          linetype = 'solid',
                                                          colour = "grey"),
                 legend.text = ggplot2::element_text(size = 7),
                 legend.position = "right",
                 panel.border = element_rect(colour = "black", fill = NA,
                                             size = 1.5)) +
  ggplot2::guides(shape = ggplot2::guide_legend(ncol = 2),
                  col = ggplot2::guide_legend(ncol = 3),
                  override.aes = list(size = 0.5))
p
```

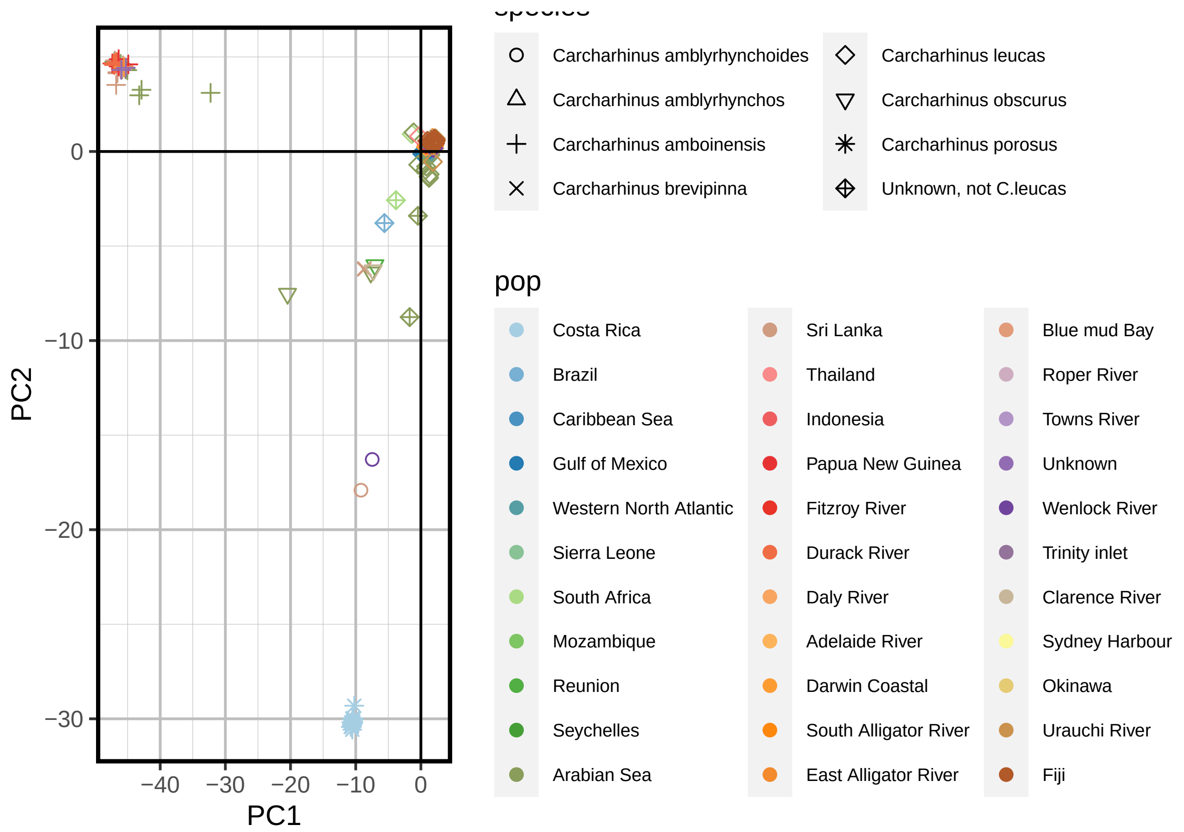


```
ggplot2::ggsave(p, filename = "DArTcap_SF_SpeciesID_PCA1.png",
                width = 30, height = 15,units = "cm")
```

```
p <- ggplot2::ggplot(BS.pca.scores, ggplot2::aes(x = PC1, y = PC3, colour = pop)) +
  ggplot2::geom_point(aes(shape = species),size = 2) +
  ggplot2::scale_color_manual(values = cols) +
  ggplot2::scale_shape_manual(values = c(1,2,3,4,5,6,8,9)) +
  ggplot2::geom_hline(yintercept = 0) + ggplot2::geom_vline(xintercept = 0) +
  ggplot2::theme(panel.background = ggplot2::element_rect(fill = "white",
                                                          colour = "white"),
                 panel.grid.major = ggplot2::element_line(size = 0.5,
                                                          linetype = 'solid',
                                                          colour = "grey"),
                 panel.grid.minor = ggplot2::element_line(size = 0.1,
                                                          linetype = 'solid',
                                                          colour = "grey"),
                 legend.text = ggplot2::element_text(size = 7),
                 legend.position = "right",
                 panel.border = element_rect(colour = "black",
                                             fill = NA, size = 1.5)) +
  ggplot2::guides(shape = ggplot2::guide_legend(ncol = 2),
                  col = ggplot2::guide_legend(ncol = 3),
                  override.aes = list(size = 0.5))
p
```

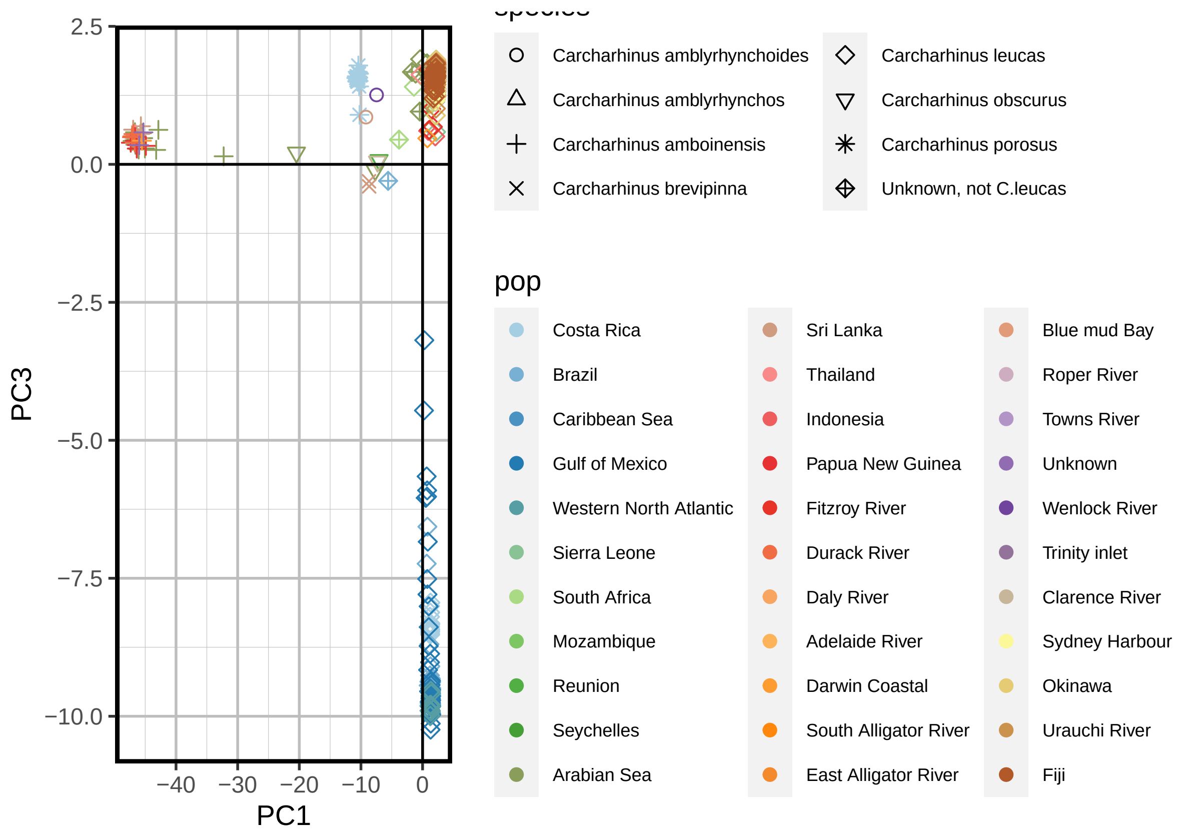


```r
ggplot2::ggsave(p, filename = "DArTcap_SF_SpeciesID_PCA2.png",
                width = 30, height = 15,units = "cm")

p <- ggplot2::ggplot(BS.pca.scores, ggplot2::aes(x = PC2, y = PC3, colour = pop)) +
  ggplot2::geom_point(aes(shape = species),size = 2) +
  ggplot2::scale_color_manual(values = cols) +
  ggplot2::scale_shape_manual(values = c(1,2,3,4,5,6,8,9)) +
  ggplot2::geom_hline(yintercept = 0) + ggplot2::geom_vline(xintercept = 0) +
  ggplot2::theme(panel.background = ggplot2::element_rect(fill = "white",
                                                          colour = "white"),
                 panel.grid.major = ggplot2::element_line(size = 0.5,
                                                          linetype = 'solid',
                                                          colour = "grey"),
                 panel.grid.minor = ggplot2::element_line(size = 0.1,
                                                          linetype = 'solid',
                                                          colour = "grey"),
                 legend.text = ggplot2::element_text(size = 7),
                 legend.position = "right",
                 panel.border = element_rect(colour = "black", fill = NA, size = 1.5)) +
  ggplot2::guides(shape = ggplot2::guide_legend(ncol = 2),
                  col = ggplot2::guide_legend(ncol = 3),
                  override.aes = list(size = 0.5))
p
```

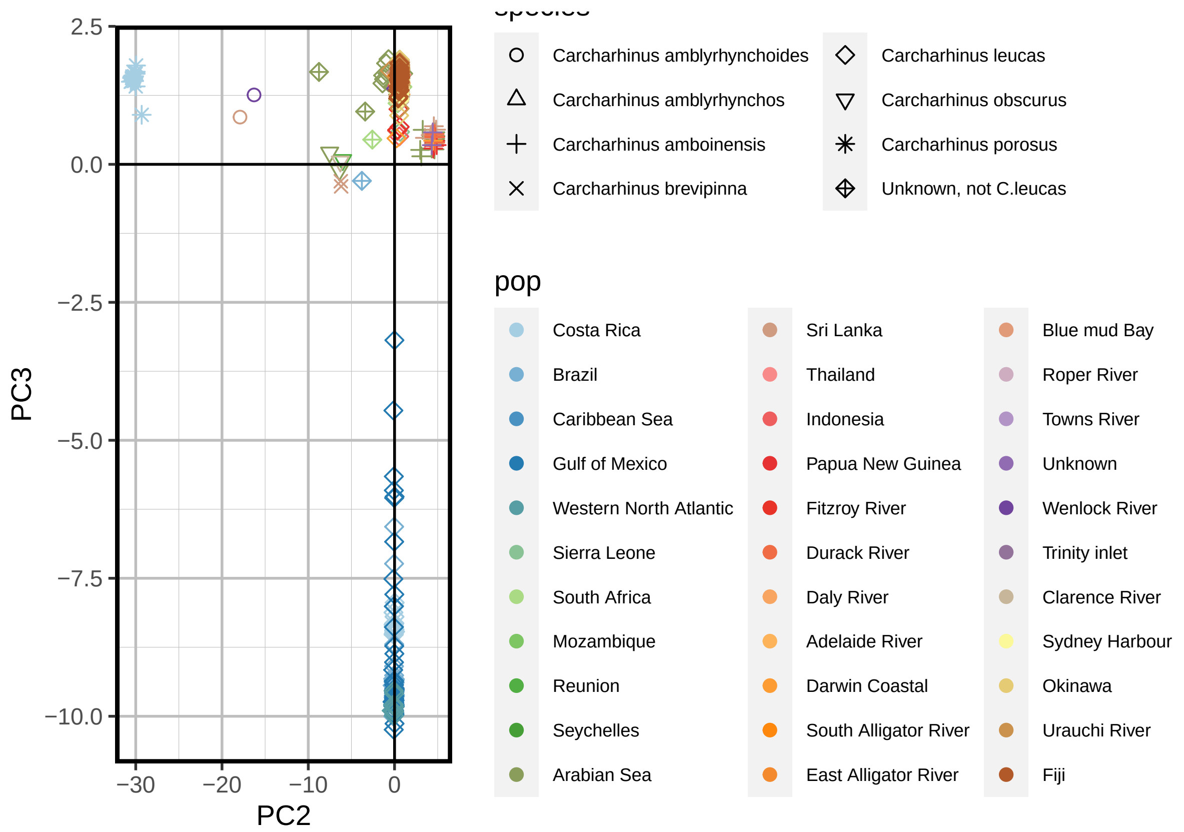


```
ggplot2::ggsave(p, filename = "DArTcap_SF_SpeciesID_PCA3.png",
                width = 30, height = 15,units = "cm")


p <- ggplot2::ggplot(BS.pca.scores, ggplot2::aes(x = PC3, y = PC4, colour = pop)) +
  ggplot2::geom_point(aes(shape = species),size = 2) +
  ggplot2::scale_color_manual(values = cols) +
  ggplot2::scale_shape_manual(values = c(1,2,3,4,5,6,8,9)) +
  ggplot2::geom_hline(yintercept = 0) + ggplot2::geom_vline(xintercept = 0) +
  ggplot2::theme(panel.background = ggplot2::element_rect(fill = "white",
                                                        colour = "white"),
                 panel.grid.major = ggplot2::element_line(size = 0.5,
                                                        linetype = 'solid',
                                                        colour = "grey"),
                 panel.grid.minor = ggplot2::element_line(size = 0.1,
                                                        linetype = 'solid',
                                                        colour = "grey"),
                 legend.text = ggplot2::element_text(size = 7),
                 legend.position = "right",
                 panel.border = element_rect(colour = "black", fill = NA, size = 1.5)) +
  ggplot2::guides(shape = ggplot2::guide_legend(ncol = 2),
                  col = ggplot2::guide_legend(ncol = 3),
                  override.aes = list(size = 0.5))
p
```

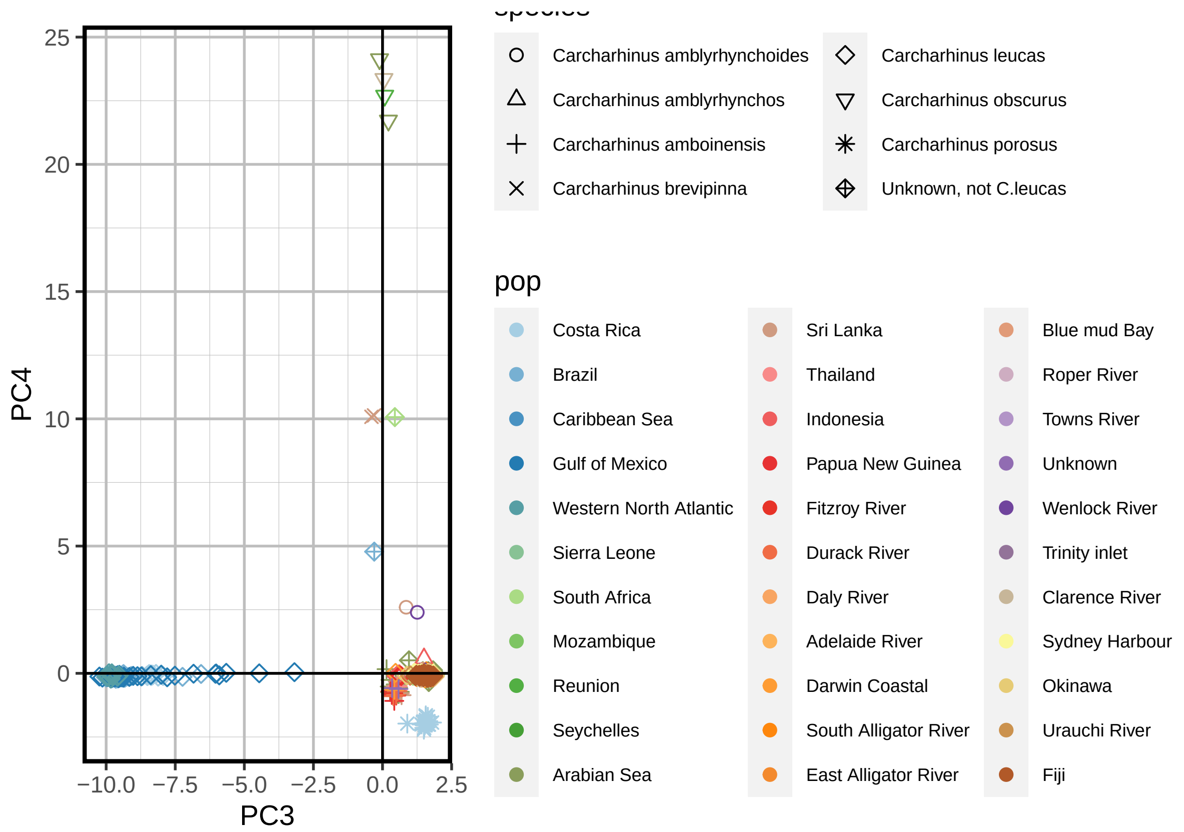


```
ggplot2::ggsave(p, filename = "DArTcap_SF_SpeciesID_PCA4.png",
                width = 30, height = 15,units = "cm")

p <- ggplot2::ggplot(BS.pca.scores, ggplot2::aes(x = PC2, y = PC4, colour = pop)) +
  ggplot2::geom_point(aes(shape = species),size = 2) +
  ggplot2::scale_color_manual(values = cols) +
  ggplot2::scale_shape_manual(values = c(1,2,3,4,5,6,8,9)) +
  ggplot2::geom_hline(yintercept = 0) + ggplot2::geom_vline(xintercept = 0) +
  ggplot2::theme(panel.background = ggplot2::element_rect(fill = "white",
                                                          colour = "white"),
                 panel.grid.major = ggplot2::element_line(size = 0.5,
                                                          linetype = 'solid',
                                                          colour = "grey"),
                 panel.grid.minor = ggplot2::element_line(size = 0.1,
                                                          linetype = 'solid',
                                                          colour = "grey"),
                 legend.text = ggplot2::element_text(size = 7),
                 legend.position = "right",
                 panel.border = element_rect(colour = "black", fill = NA,
                                             size = 1.5)) +
  ggplot2::guides(shape = ggplot2::guide_legend(ncol = 2),
                  col = ggplot2::guide_legend(ncol = 3),
                  override.aes = list(size = 0.5))
p
```

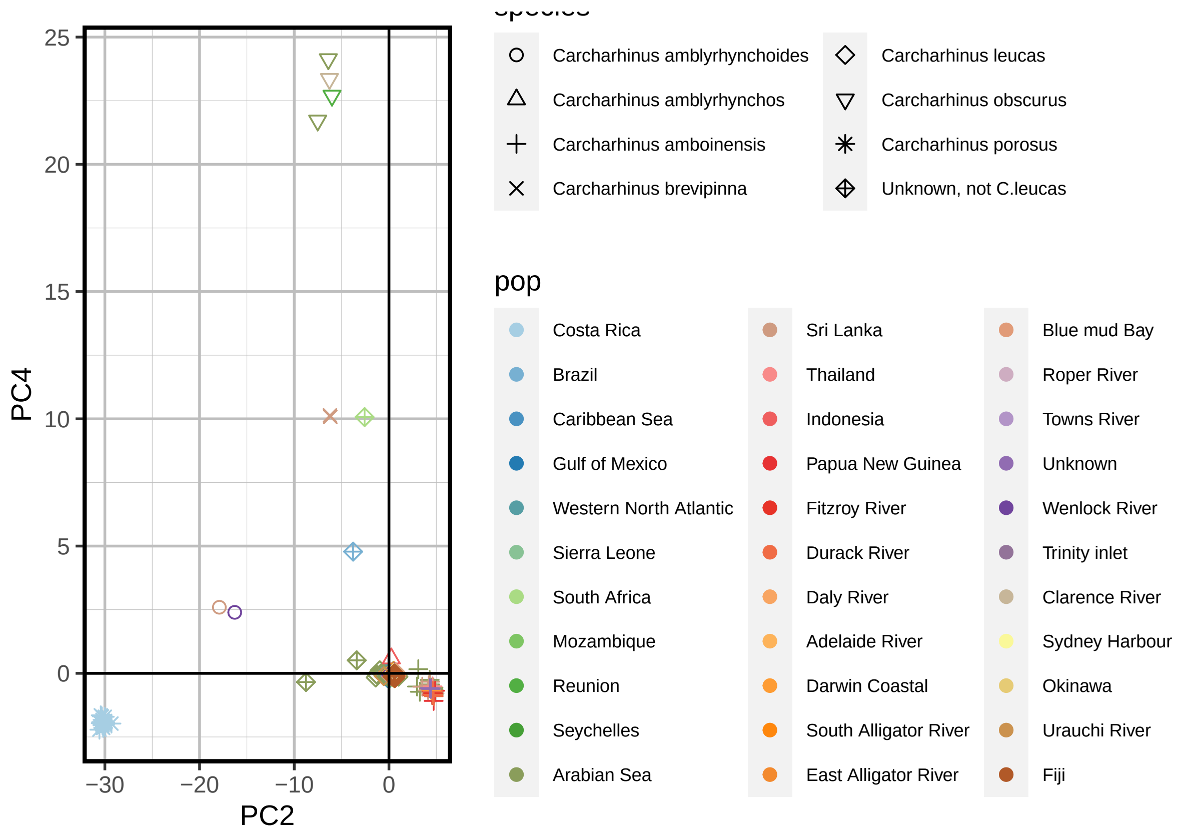


```
ggplot2::ggsave(p, filename = "DArTcap_SF_SpeciesID_PCA5.png",
                width = 30, height = 15,units = "cm")


p <- ggplot2::ggplot(BS.pca.scores, ggplot2::aes(x = PC1, y = PC6, colour = pop)) +
  ggplot2::geom_point(aes(shape = species),size = 2) +
  ggplot2::scale_color_manual(values = cols) +
  ggplot2::scale_shape_manual(values = c(1,2,3,4,5,6,8,9)) +
  ggplot2::geom_hline(yintercept = 0) + ggplot2::geom_vline(xintercept = 0) +
  ggplot2::theme(panel.background = ggplot2::element_rect(fill = "white",
                                                          colour = "white"),
                 panel.grid.major = ggplot2::element_line(size = 0.5,
                                                          linetype = 'solid',
                                                          colour = "grey"),
                 panel.grid.minor = ggplot2::element_line(size = 0.1,
                                                          linetype = 'solid',
                                                          colour = "grey"),
                 legend.text = ggplot2::element_text(size = 7),
                 legend.position = "right",
                 panel.border = element_rect(colour = "black", fill = NA,
                                             size = 1.5)) +
  ggplot2::guides(shape = ggplot2::guide_legend(ncol = 2),
                  col = ggplot2::guide_legend(ncol = 3),
                  override.aes = list(size = 0.5))
p
```

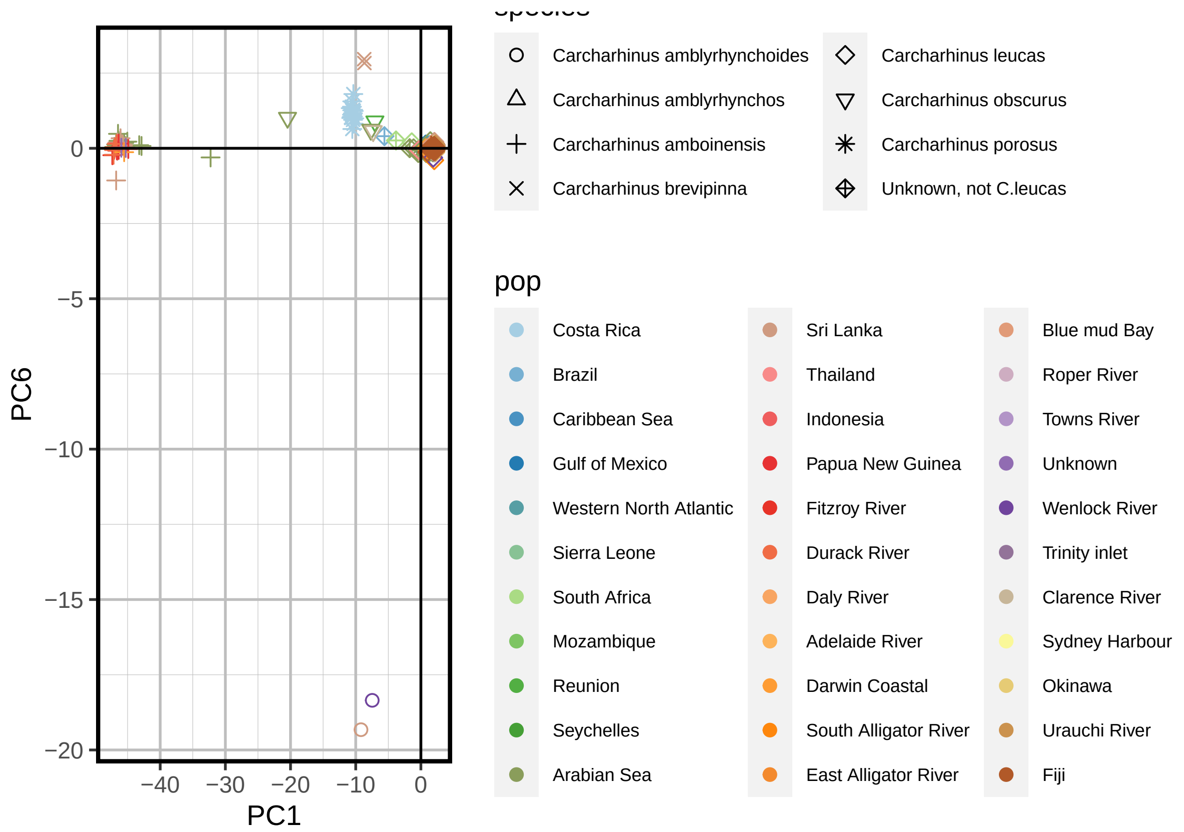


```
ggplot2::ggsave(p, filename = "DArTcap_SF_SpeciesID_PCA6.png",
                width = 30, height = 15,units = "cm")

p <- ggplot2::ggplot(BS.pca.scores, ggplot2::aes(x = PC2, y = PC6, colour = pop)) +
  ggplot2::geom_point(aes(shape = species),size = 2) +
  ggplot2::scale_color_manual(values = cols) +
  ggplot2::scale_shape_manual(values = c(1,2,3,4,5,6,8,9)) +
  ggplot2::geom_hline(yintercept = 0) + ggplot2::geom_vline(xintercept = 0) +
  ggplot2::theme(panel.background = ggplot2::element_rect(fill = "white",
                                                          colour = "white"),
                 panel.grid.major = ggplot2::element_line(size = 0.5,
                                                          linetype = 'solid',
                                                          colour = "grey"),
                 panel.grid.minor = ggplot2::element_line(size = 0.1,
                                                          linetype = 'solid',
                                                          colour = "grey"),
                 legend.text = ggplot2::element_text(size = 7),
                 legend.position = "right",
                 panel.border = element_rect(colour = "black",
                                             fill = NA, size = 1.5)) +
  ggplot2::guides(shape = ggplot2::guide_legend(ncol = 2),
                  col = ggplot2::guide_legend(ncol = 3),
                  override.aes = list(size = 0.5))
p
```

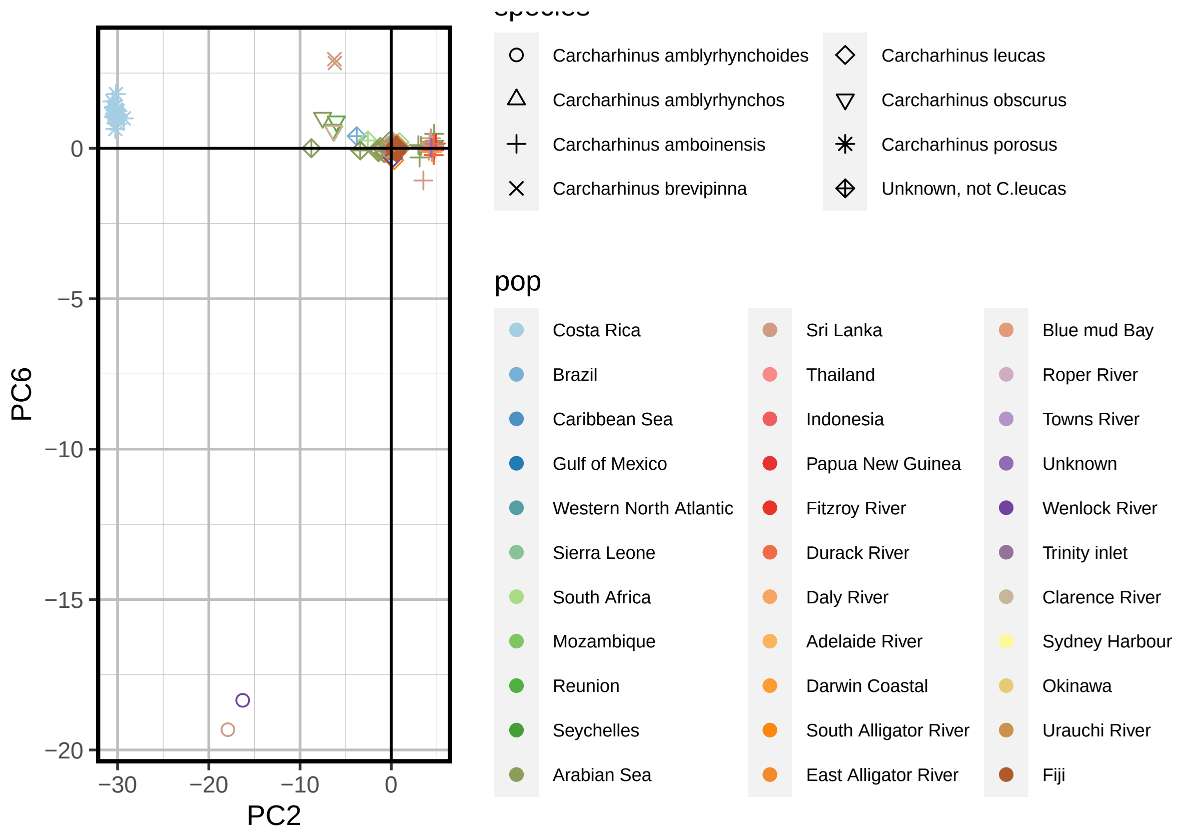


```
ggplot2::ggsave(p, filename = "DArTcap_SF_SpeciesID_PCA7.png",
                width = 30, height = 15,units = "cm")
```

---

# 8 DATA2: DArTcap - Only Bull Shark

## 8.1 Set strata

```
strata <- radiator::read_strata("Bull_shark_DArTcap_strata.tsv",
                                keep.two = FALSE)$strata %>%
  dplyr:: filter(SpeciesID_genetic == "Carcharhinus leucas")
readr::write_tsv(strata, path = "Bull_shark_DArTcap_strata2.tsv")
```

1. Marker Reproducibility: 0.98
2. Individual missingness: 0.35
3. Individual Het - low: 0
4. Individual Het - high: 0.05
5. Individual total coverage - low: 10,000
6. Individual total coverage - high: 1,000,000
7. Marker MAC - low: 4
8. Marker coverage - low: 4
9. Marker coverage - high:60
10. Marker maximum missing proportion: 0.1
11. Marker snp position on locus: n
12. Max 3 SNPs per locus
13. 1 SNP per locus according to mac
14. no LD filter
15. Individual Het - low: 0.031
16. Individual Het - high: 0.08
17. Duplicated individuals: n
18. Filter on HWE: 4 pop, 0.001

## 8.2 Filter data

```
dartcap.data <- "SEQ_SNPs_counts_0_Target_merge.csv"
strata <- "Bull_shark_DArTcap_strata2.tsv"
BSsnp <- radiator::filter_rad(dartcap.data, strata,
                              parallel.core = 1)
BS.dartcap.gds <- BSsnp$gds
BS.dartcap.tidy <- BSsnp$output$tidy.data
print(BS.dartcap.gds, expand=TRUE, all = TRUE, attribute=TRUE,
      attribute.trim = FALSE)

save(BS.dartcap.tidy, file = "DArTcap_ALL_objects.Rdata")
```

Table 11:

| FILTERS | VALUES | BEFORE | BLACKLIST |
|---|---|---|---|
| Filter DArT reproducibility | 0.98 | 942 / 32 / 1 / 26335 / 37537 | 0 / 0 / 0 / 1043 / 2250 |
| Filter monomorphic markers | NA | 942 / 32 / 1 / 25292 / 35287 | 0 / 0 / 0 / 10550 / 17988 |
| Filter markers in common | NA | 942 / 32 / 1 / 14742 / 17299 | 0 / 0 / 0 / 1710 / 2139 |
| Filter individuals based on missingness | 0.35 | 942 / 32 / 1 / 13032 / 15160 | 20 / 0 / 0 / 0 / 0 |
| Filter individuals based on heterozygosity | 0 0.05 | 922 / 32 / 1 / 13032 / 15160 | 10 / 0 / 0 / 0 / 0 |
| Filter individuals based on total coverage | 10000 1e+06 | 912 / 32 / 1 / 13032 / 15160 | 4 / 0 / 0 / 0 / 0 |
| Filter monomorphic markers | NA | 908 / 32 / 1 / 13032 / 15160 | 0 / 0 / 0 / 2115 / 2820 |
| Filter MAC | 4 | 908 / 32 / 1 / 10917 / 12340 | 0 / 0 / 0 / 5725 / 6709 |
| Filter coverage min / max | 4-60 | 908 / 32 / 1 / 5192 / 5631 | 0 / 0 / 0 / 1367 / 1436 |
| Filter genotyping | 0.1 | 908 / 32 / 1 / 3825 / 4195 | 0 / 0 / 0 / 367 / 407 |
| Filter SNPs position on the read | all | 908 / 32 / 1 / 3458 / 3788 | 0 / 0 / 0 / 0 / 0 |
| Filter markers snp number | 3 | 908 / 32 / 1 / 3458 / 3788 | 0 / 0 / 0 / 4 / 17 |
| Filter short ld | mac | 908 / 32 / 1 / 3454 / 3771 | 0 / 0 / 0 / 0 / 317 |
| detect mixed genomes | 0.031 0.08 | 908 / 32 / 1 / 3454 / 3454 | 44 / 0 / 0 / 0 / 0 |
| Filter monomorphic markers | NA | 864 / 32 / 1 / 3454 / 3454 | 0 / 0 / 0 / 0 / 0 |
| detect duplicate genomes | 0.1 | 864 / 32 / 1 / 3454 / 3454 | 83 / 0 / 0 / 0 / 0 |
| Filter monomorphic markers | NA | 781 / 32 / 1 / 3454 / 3454 | 0 / 0 / 0 / 0 / 0 |
| Filter HWE | 40.001 | 781 / 32 / 1 / 3454 / 3454 | 0 / 0 / 0 / 11 / 11 |

### 8.2.1 Remove extra low and high heterozygous individuals

Because the average heterozygosity per sampling location is not even, certain individuals cannot be excluded without removing 'good' individuals.

```
rm <-  c("CL-ARS021-F","CL-GOM025-F.1","CL-GOM012-F","CL-SAF014-M","CL-SAF002-F",
         "CL-BRZ038-U","CLF003-F","CL-GOM011-F","CL-SAF015-F","CL-SAF003-M",
         "CL-PNG005-U","CLCR056-M","CLCR047.1","CLCR055-M","CLCR066-M")

BS.dartcap.tidy <- BS.dartcap.tidy[!BS.dartcap.tidy$INDIVIDUALS %in% rm,]
length(unique(BS.dartcap.tidy$INDIVIDUALS)) # 769
```

## 8.3 Save data

```
Convert <- radiator::genomic_converter(BS.dartcap.tidy,
                                       output = c("genlight", "stockr", "pcadapt", "gtypes", "rubias
BS.dartcap.gl <- Convert$genlight
BS.dartcap.stkr <- Convert$stockr
BS.dartcap.pc <- Convert$pcadapt
BS.dartcap.gt <- Convert$gtypes
BS.dartcap.rubias <- Convert$rubias



markers.meta <- radiator::extract_markers_metadata(BS.dartcap.gds, whitelist = TRUE)
individuals.meta <- radiator::extract_individuals_metadata(BS.dartcap.gds, whitelist = TRUE)
individuals.meta <- individuals.meta[individuals.meta$INDIVIDUALS %in%
                                       BS.dartcap.gl$ind.names,]
BS.dartcap.gl$other$ind.metrics <- individuals.meta
BS.dartcap.gl$other$loc.metrics <- markers.meta
BS.dartcap.gt@schemes <- individuals.meta

pop.levels <- c("Costa Rica" , "Brazil","Caribbean Sea","Gulf of Mexico",
                "Western North Atlantic", "Sierra Leone", "South Africa",
                "Mozambique", "Reunion" , "Seychelles", "Arabian Sea",
                "Sri Lanka", "Thailand", "Indonesia", "Papua New Guinea" ,
                "Fitzroy River", "Daly River", "Adelaide River","Darwin Coastal",
                "South Alligator River", "East Alligator River","Blue mud Bay",
                "Roper River","Towns River","Unknown","Wenlock River","Trinity inlet",
                "Clarence River", "Sydney Harbour", "Okinawa", "Urauchi River", "Fiji")
BS.dartcap.gl$pop <- factor(BS.dartcap.gl$other$ind.metrics$Site2, levels = pop.levels)
BS.dartcap.gl <- BS.dartcap.gl[order(BS.dartcap.gl$pop, BS.dartcap.gl$ind.names)]
BS.dartcap.rubias$repunit <- factor(BS.dartcap.rubias$repunit, levels = pop.levels)

save(BS.dartcap.tidy, BS.dartcap.gl, BS.dartcap.stkr, BS.dartcap.pc,BS.dartcap.gt,
     BS.dartcap.rubias,
     markers.meta, individuals.meta, file = "DArTcap_ALL_objects.Rdata")
```

## 8.4 Load data

```
load("DArTcap_ALL_objects.Rdata")
pop.levels <- c("Costa Rica" , "Brazil","Caribbean Sea","Gulf of Mexico",
                "Western North Atlantic", "Sierra Leone", "South Africa",
                "Mozambique", "Reunion" , "Seychelles", "Arabian Sea", "Sri Lanka",
                "Thailand", "Indonesia", "Papua New Guinea" , "Fitzroy River",
                "Daly River", "Adelaide River","Darwin Coastal",  "South Alligator River",
                "East Alligator River","Blue mud Bay", "Roper River","Towns River",
                "Unknown","Wenlock River","Trinity inlet","Clarence River",
```

```
                "Sydney Harbour", "Okinawa", "Urauchi River", "Fiji")


BS.dartcap.gl$pop <- factor(BS.dartcap.gl$other$ind.metrics$Site2,
                            levels = pop.levels)
BS.dartcap.gl <- BS.dartcap.gl[order(BS.dartcap.gl$pop, BS.dartcap.gl$ind.names)]

pop.levels1 <- stringr::str_replace_all(pop.levels, " ", "_")
BS.dartcap.tidy$POP_ID <- factor(BS.dartcap.tidy$POP_ID, levels = pop.levels1)
BS.dartcap.tidy <- BS.dartcap.tidy[order(BS.dartcap.tidy$POP_ID,
                                         BS.dartcap.tidy$INDIVIDUALS),]

adegenet::nInd(BS.dartcap.gl) # 769
adegenet::nLoc(BS.dartcap.gl) # 3443
summary(BS.dartcap.gl$pop)
sum(duplicated(BS.dartcap.gl$other$ind.metrics$`Genetic code`))# 0 replicate left
sum(baits$CloneID %in% BS.dartcap.gl$other$loc.metrics$LOCUS) #78
```

```
## [1] 769
## [1] 3443
##            Costa Rica                  Brazil          Caribbean Sea
##                    16                      50                      2
##        Gulf of Mexico Western North Atlantic           Sierra Leone
##                    37                       8                      1
##          South Africa              Mozambique               Reunion
##                    22                      11                     28
##            Seychelles             Arabian Sea              Sri Lanka
##                    36                      15                     12
##              Thailand               Indonesia       Papua New Guinea
##                     5                       4                      9
##         Fitzroy River             Daly River          Adelaide River
##                     3                      49                     18
##       Darwin Coastal  South Alligator River   East Alligator River
##                    15                      88                     47
##          Blue mud Bay             Roper River            Towns River
##                    18                       9                     10
##               Unknown           Wenlock River          Trinity inlet
##                    10                      24                      5
##       Clarence River          Sydney Harbour                Okinawa
##                    83                      65                      9
##         Urauchi River                    Fiji
##                    35                      25
## [1] 0
## [1] 78
```

## 8.5 Remove sex-linked markers

```
load("Sex-linked_marker_DArTcap.Rdata")
load("Sex_results.Rdata")
sum(bull_shark$sexy.summary$CLONE_ID %in% SLM$sexy.summary$CLONE_ID) #33
sum(SLM$sexy.summary$CLONE_ID %in% bull_shark$sexy.summary$CLONE_ID) #23
sex.markers <- unique(c(bull_shark$sexy.summary$CLONE_ID, SLM$sexy.summary$CLONE_ID))

sum(BS.dartcap.gl$loc.names %in% sex.markers) #34
locnames <- BS.dartcap.gl$loc.names
BS.dartcap.gl <- BS.dartcap.gl[,!locnames %in% sex.markers]
BS.dartcap.gl$other$loc.metrics <- BS.dartcap.gl$other$loc.metrics[!locnames %in%
```

```
                                                                        sex.markers,]

sum(sex.markers %in% unique(BS.dartcap.tidy$LOCUS)) # 34
BS.dartcap.tidy <- BS.dartcap.tidy[!BS.dartcap.tidy$LOCUS %in% sex.markers,]

locus.gt <- stringr::str_match(pattern = "__\\s*(.*?)\\s*__",
                                string =  BS.dartcap.gt@data$locus)[,2]
BS.dartcap.gt <- BS.dartcap.gt[,!locus.gt %in% sex.markers, , drop = TRUE]

locus.rubias <- stringr::str_match(pattern = "__\\s*(.*?)\\s*__",
                                    string =  colnames(BS.dartcap.rubias))[,2]
BS.dartcap.rubias <- BS.dartcap.rubias[,!locus.rubias %in% sex.markers]
```

```
## [1] 33
## [1] 23
## [1] 34
## [1] 34
```

## 8.6 Genetic diversity

```
set.seed(124)
pop.levels <- c("Costa Rica" , "Brazil","Caribbean Sea","Gulf of Mexico",
                "Western North Atlantic", "South Africa", "Mozambique", "Reunion" ,
                "Seychelles", "Arabian Sea", "Sri Lanka", "Thailand",
                "Indonesia", "Papua New Guinea" , "Fitzroy River", "Daly River",
                "Adelaide River","Darwin Coastal",  "South Alligator River",
                "East Alligator River","Blue mud Bay", "Roper River","Towns River",
                "Unknown","Wenlock River","Trinity inlet","Clarence River",
                "Sydney Harbour", "Urauchi River","Okinawa", "Fiji")

gl <- BS.dartcap.gl
gl <- gl[gl$pop != "Sierra Leone"] #only 1 sample
gl$pop <- factor(gl$other$ind.metrics$Site2, levels = pop.levels)
gl <- gl[order(gl$pop,gl$ind.names)]

gl2gpop(gl, filename = "BS_dartcap_all_genepop.txt")
bastat <-diveRsity::basicStats(infile = "BS_dartcap_all_genepop.txt",
                                outfile = "BS_dartcap_all_BasicStat_output.txt",
                                fis_ci = TRUE,ar_ci = TRUE, fis_boots = 1000,
                                ar_boots = 1000,mc_reps = 1000,
                                rarefaction = FALSE, ar_alpha = 0.05,
                                fis_alpha = 0.05)

length(bastat$main_tab)#31
gendiv <- data.frame(
  "Costa Rica" = bastat$main_tab[[1]]$overall,
  "Brazil" = bastat$main_tab[[2]]$overall,
  "Caribbean Sea" = bastat$main_tab[[3]]$overall,
  "Gulf of Mexico" = bastat$main_tab[[4]]$overall,
  "Western North Atlantic" = bastat$main_tab[[5]]$overall,
  "South Africa" = bastat$main_tab[[6]]$overall,
  "Mozambique" = bastat$main_tab[[7]]$overall,
  "Reunion" = bastat$main_tab[[8]]$overall,
  "Seychelles" = bastat$main_tab[[9]]$overall,
  "Arabian Sea" = bastat$main_tab[[10]]$overall,
  "Sri Lanka" = bastat$main_tab[[11]]$overall,
  "Thailand" = bastat$main_tab[[12]]$overall,
```

```r
  "Indonesia" = bastat$main_tab[[13]]$overall,
  "Papua New Guinea" = bastat$main_tab[[14]]$overall,
  "Fitzroy River" = bastat$main_tab[[15]]$overall,
  "Daly River" = bastat$main_tab[[16]]$overall,
  "Adelaide River" = bastat$main_tab[[17]]$overall,
  "Darwin Coastal" = bastat$main_tab[[18]]$overall,
  "South Alligator River" = bastat$main_tab[[19]]$overall,
  "East Alligator River" = bastat$main_tab[[20]]$overall,
  "Blue mud Bay" = bastat$main_tab[[21]]$overall,
  "Roper River" = bastat$main_tab[[22]]$overall,
  "Towns River" = bastat$main_tab[[23]]$overall,
  "Unknown" = bastat$main_tab[[24]]$overall,
  "Wenlock River" = bastat$main_tab[[25]]$overall,
  "Trinity inlet" = bastat$main_tab[[26]]$overall,
  "Clarence River" = bastat$main_tab[[27]]$overall,
  "Sydney Harbour" = bastat$main_tab[[28]]$overall,
  "Urauchi River" = bastat$main_tab[[29]]$overall,
  "Okinawa" = bastat$main_tab[[30]]$overall,
  "Fiji" = bastat$main_tab[[31]]$overall,
  check.names = FALSE)
row.names(gendiv) <- rownames(bastat$main_tab[[1]])

df <- as.data.frame(gl)
df[df == "0"] <- "11"
df[df == "1"] <- "12"
df[df == "2"] <- "22"
gen.gi <- adegenet::df2genind(df, ploidy = 2,ncode = 2, pop = gl$pop)
ris_basic <- hierfstat::basic.stats(gen.gi)
fis.hierfstat <- apply(ris_basic$Fis, 2, mean, na.rm=T)
fis.CI <- hierfstat::boot.ppfis(gen.gi, nboot = 1000, quant = c(0.025,0.975),
                               diploid = TRUE,dig = 4)
df <- data.frame(fis.hierfstat,fis.CI.low = fis.CI$fis.ci[,1],
                 fis.CI.high = fis.CI$fis.ci[,2])
df <- as.data.frame(t(df))
gendiv <- rbind(gendiv, df)

readr::write_tsv(gendiv, path = "DArTcap_ALL_gendiv.txt")
save(gendiv, file = "DArTcap_ALL_gendiv.Rdata")


pop.levels <- c("Costa Rica" , "Brazil", "Caribbean Sea", "Gulf of Mexico",
                "Western North Atlantic", "South Africa", "Mozambique", "Reunion" ,
                "Seychelles", "Arabian Sea", "Sri Lanka", "Thailand", "Indonesia",
                "Papua New Guinea" , "Australia", "Japan", "Fiji")
gl <- BS.dartcap.gl
gl <- gl[gl$pop != "Sierra Leone"] #only 1 sample
gl$pop <- factor(gl$other$ind.metrics$Site, levels = pop.levels)
gl <- gl[order(gl$pop,gl$ind.names)]

gl2gpop(gl, filename = "BS_dartcap_all_Sites_genepop.txt")
bastat <-diveRsity::basicStats(infile = "BS_dartcap_all_Sites_genepop.txt",
                               outfile = "BS_dartcap_all_Sites_BasicStat_output.txt",
                               fis_ci = TRUE,ar_ci = TRUE, fis_boots = 1000,
                               ar_boots = 1000,mc_reps = 1000,
                               rarefaction = FALSE, ar_alpha = 0.05,
                               fis_alpha = 0.05)
```

```r
length(bastat$main_tab)#17
gendiv <- data.frame(
  "Costa Rica" = bastat$main_tab[[1]]$overall,
  "Brazil" = bastat$main_tab[[2]]$overall,
  "Caribbean Sea" = bastat$main_tab[[3]]$overall,
  "Gulf of Mexico" = bastat$main_tab[[4]]$overall,
  "Western North Atlantic" = bastat$main_tab[[5]]$overall,
  "South Africa" = bastat$main_tab[[6]]$overall,
  "Mozambique" = bastat$main_tab[[7]]$overall,
  "Reunion" = bastat$main_tab[[8]]$overall,
  "Seychelles" = bastat$main_tab[[9]]$overall,
  "Arabian Sea" = bastat$main_tab[[10]]$overall,
  "Sri Lanka" = bastat$main_tab[[11]]$overall,
  "Thailand" = bastat$main_tab[[12]]$overall,
  "Indonesia" = bastat$main_tab[[13]]$overall,
  "Papua New Guinea" = bastat$main_tab[[14]]$overall,
  "Australia" = bastat$main_tab[[15]]$overall,
  "Japan" = bastat$main_tab[[16]]$overall,
  "Fiji" = bastat$main_tab[[17]]$overall,
  check.names = FALSE)
row.names(gendiv) <- rownames(bastat$main_tab[[1]])

df <- as.data.frame(gl)
df[df == "0"] <- "11"
df[df == "1"] <- "12"
df[df == "2"] <- "22"
gen.gi <- adegenet::df2genind(df, ploidy = 2,ncode = 2, pop = gl$pop)
ris_basic <- hierfstat::basic.stats(gen.gi)
fis.hierfstat <- apply(ris_basic$Fis, 2, mean, na.rm=T)
fis.CI <- hierfstat::boot.ppfis(gen.gi, nboot = 1000, quant = c(0.025,0.975),
                                diploid = TRUE,dig = 4)
df <- data.frame(fis.hierfstat,fis.CI.low = fis.CI$fis.ci[,1],
                 fis.CI.high = fis.CI$fis.ci[,2])
df <- as.data.frame(t(df))
gendiv <- rbind(gendiv, df)
readr::write_tsv(gendiv, path = "DArTcap_ALL_Sites_gendiv.txt")
save(gendiv, file = "DArTcap_ALL_Sites_gendiv.Rdata")


gl <- BS.dartcap.gl
gl <- gl[!gl$other$ind.metrics$Region == "E-ATL",] #only 1 sample
gl$other$ind.metrics$Region[gl$other$ind.metrics$Region %in%
                              c("W-IO","N-IO","E-IO","W-PAC","E-IO/W-PAC")] <- "IWP"
pop.levels <- c("E-PAC","W-ATL","IWP", "Japan","Fiji")
gl$pop <- factor(gl$other$ind.metrics$Region, levels = pop.levels)
gl <- gl[order(gl$pop, gl$ind.names)]

gl2gpop(gl, filename = "BS_dartcap_all_Regions_genepop.txt")
bastat <-diveRsity::basicStats(infile = "BS_dartcap_all_Regions_genepop.txt",
                               outfile = "BS_dartcap_all_Regions_BasicStat_output.txt",
                               fis_ci = TRUE,ar_ci = TRUE, fis_boots = 1000,
                               ar_boots = 1000,mc_reps = 1000,
                               rarefaction = FALSE, ar_alpha = 0.05,
                               fis_alpha = 0.05)

length(bastat$main_tab)#5
gendiv <- data.frame(
```

```r
  "E-PAC" = bastat$main_tab[[1]]$overall,
  "W-ATL" = bastat$main_tab[[2]]$overall,
  "IWP" = bastat$main_tab[[3]]$overall,
  "Japan" = bastat$main_tab[[4]]$overall,
  "Fiji" = bastat$main_tab[[5]]$overall,
  check.names = FALSE)
row.names(gendiv) <- rownames(bastat$main_tab[[1]])

df <- as.data.frame(gl)
df[df == "0"] <- "11"
df[df == "1"] <- "12"
df[df == "2"] <- "22"
gen.gi <- adegenet::df2genind(df, ploidy = 2,ncode = 2, pop = gl$pop)
ris_basic <- hierfstat::basic.stats(gen.gi)
fis.hierfstat <- apply(ris_basic$Fis, 2, mean, na.rm=T)
fis.CI <- hierfstat::boot.ppfis(gen.gi, nboot = 1000, quant = c(0.025,0.975),
                                diploid = TRUE,dig = 4)
df <- data.frame(fis.hierfstat,fis.CI.low = fis.CI$fis.ci[,1],
                 fis.CI.high = fis.CI$fis.ci[,2])
df <- as.data.frame(t(df))
gendiv <- rbind(gendiv, df)
readr::write_tsv(gendiv, path = "DArTcap_ALL_Regions_gendiv.txt")
save(gendiv, file = "DArTcap_ALL_Regions_gendiv.Rdata")
```

```r
load("DArTcap_ALL_gendiv.Rdata")

shortnames <- c("COR","BRZ", "CAR", "GOM", "WNA", "SAF", "MOZ", "RUN" , "SEY",
                "ARS", "SRL", "TAI", "IND", "PNG" , "FZR", "DAR")
knitr::kable(gendiv[,1:16], col.names = shortnames, digits = 4,
             caption = "Table1A") %>%
  kableExtra::kable_styling(full_width = FALSE,font_size = 5,
                            latex_options = c( "hold_position")) %>%
  kableExtra::landscape()

shortnames <- c("ADR","DWC","SAR", "EAR","BMB", "ROR","TOR","UNK","WER","TRI",
                "CLR", "SYH", "URR","OKI", "FIJ")
knitr::kable(gendiv[,17:31], col.names = shortnames, digits = 4,
             caption = "Table1B") %>%
  kableExtra::kable_styling(full_width = FALSE,font_size = 5,
                            latex_options = c( "hold_position")) %>%
  kableExtra::landscape()


load("DArTcap_ALL_Sites_gendiv.Rdata")
shortnames <- c("COR","BRZ", "CAR", "GOM", "WNA", "SAF", "MOZ", "RUN" , "SEY",
                "ARS", "SRL", "TAI", "IND", "PNG" , "AUS", "JAP", "FIJ")
knitr::kable(gendiv, col.names = shortnames, digits = 4,
             caption = "Table2") %>%
  kableExtra::kable_styling(full_width = FALSE,font_size = 5,
                            latex_options = c( "hold_position")) %>%
  kableExtra::landscape()

load("DArTcap_ALL_Regions_gendiv.Rdata")
shortnames <- c("E-PAC","W-ATL","IWP", "Japan","Fiji")
knitr::kable(gendiv, col.names = shortnames, digits = 4,
             caption = "Table3") %>%
  kableExtra::kable_styling(full_width = FALSE,font_size = 5,
                            latex_options = c( "hold_position"))
```

Table 12: Table1A

| | COR | BRZ | CAR | GOM | WNA | SAF | MOZ | RUN | SEY | ARS | SRL | TAI | IND | PNG | FZR | DAR |
|---|---|---|---|---|---|---|---|---|---|---|---|---|---|---|---|---|
| ar | 1.0620 | 1.0950 | 1.0650 | 1.0870 | 1.0840 | 1.1120 | 1.0860 | 1.1080 | 1.1120 | 1.1140 | 1.1100 | 1.1090 | 1.0970 | 1.1050 | 1.0830 | 1.1140 |
| size | 15.6540 | 49.0870 | 1.9750 | 36.0400 | 7.8830 | 21.7470 | 10.2580 | 27.3810 | 35.4700 | 14.8430 | 11.8850 | 4.9650 | 3.9590 | 8.9060 | 2.9130 | 48.5170 |
| obs_het | 0.0360 | 0.0520 | 0.0490 | 0.0480 | 0.0500 | 0.0600 | 0.0560 | 0.0590 | 0.0600 | 0.0620 | 0.0610 | 0.0660 | 0.0610 | 0.0590 | 0.0600 | 0.0600 |
| exp_het | 0.0390 | 0.0560 | 0.0380 | 0.0530 | 0.0500 | 0.0620 | 0.0600 | 0.0620 | 0.0620 | 0.0630 | 0.0610 | 0.0590 | 0.0540 | 0.0580 | 0.0520 | 0.0630 |
| uexp_het | 0.0410 | 0.0570 | 0.0510 | 0.0530 | 0.0540 | 0.0630 | 0.0640 | 0.0630 | 0.0630 | 0.0650 | 0.0640 | 0.0660 | 0.0610 | 0.0620 | 0.0620 | 0.0630 |
| fis | 0.0450 | 0.0480 | -0.2930 | 0.0500 | -0.0250 | 0.0240 | 0.0520 | 0.0400 | 0.0260 | 0.0040 | -0.0050 | -0.1080 | -0.1360 | -0.0270 | -0.1750 | 0.0370 |
| hwe_glb | 1.0000 | 1.0000 | 1.0000 | 1.0000 | 1.0000 | 1.0000 | 1.0000 | 1.0000 | 1.0000 | 1.0000 | 1.0000 | 1.0000 | 1.0000 | 1.0000 | 1.0000 | 1.0000 |
| hwe_hom | 1.0000 | 1.0000 | 1.0000 | 1.0000 | 1.0000 | 1.0000 | 1.0000 | 1.0000 | 1.0000 | 1.0000 | 1.0000 | 1.0000 | 1.0000 | 1.0000 | 1.0000 | 1.0000 |
| hwe_het | 1.0000 | 1.0000 | 1.0000 | 1.0000 | 1.0000 | 1.0000 | 1.0000 | 1.0000 | 1.0000 | 1.0000 | 1.0000 | 1.0000 | 1.0000 | 1.0000 | 1.0000 | 1.0000 |
| fis_lo | -0.0030 | 0.0340 | -1.0000 | 0.0310 | -0.1350 | -0.0100 | -0.0410 | 0.0110 | 0.0030 | -0.0490 | -0.0770 | -0.3690 | -0.5470 | -0.1240 | -1.0000 | 0.0200 |
| fis_hi | 0.0430 | 0.0520 | -0.2930 | 0.0540 | -0.0360 | 0.0190 | 0.0440 | 0.0370 | 0.0250 | -0.0030 | -0.0150 | -0.1080 | -0.1360 | -0.0370 | -0.1750 | 0.0370 |
| ar_lo | 1.0150 | 1.0850 | 1.0330 | 1.0660 | 1.0290 | 1.0530 | 0.9640 | 1.0490 | 1.0600 | 1.0520 | 1.0490 | 1.0520 | 1.0470 | 1.0450 | 1.0060 | 1.1010 |
| ar_hi | 1.0720 | 1.1060 | 1.0920 | 1.0990 | 1.1020 | 1.1230 | 1.1190 | 1.1220 | 1.1220 | 1.1280 | 1.1300 | 1.1290 | 1.1190 | 1.1180 | 1.1180 | 1.1240 |
| fis.hierfstat | 0.0750 | 0.0576 | -0.0481 | 0.0638 | 0.0369 | 0.0465 | 0.0959 | 0.0572 | 0.0398 | 0.0370 | 0.0356 | -0.0069 | -0.0098 | 0.0285 | -0.0006 | 0.0470 |
| fis.CI.low | 0.0787 | 0.0694 | -0.0306 | 0.0724 | 0.0327 | 0.0467 | 0.0985 | 0.0528 | 0.0312 | 0.0273 | 0.0126 | -0.0320 | -0.0293 | 0.0192 | -0.0016 | 0.0448 |
| fis.CI.high | 0.1376 | 0.1060 | 0.1257 | 0.1165 | 0.0984 | 0.0811 | 0.1568 | 0.0893 | 0.0606 | 0.0668 | 0.0614 | 0.0378 | 0.0551 | 0.0703 | 0.0940 | 0.0704 |

Table 13: Table1B

| | ADR | DWC | SAR | EAR | BMB | ROR | TOR | UNK | WER | TRI | CLR | SYH | URR | OKI | FIJ |
|---|---|---|---|---|---|---|---|---|---|---|---|---|---|---|---|
| ar | 1.1120 | 1.1160 | 1.1130 | 1.1110 | 1.1160 | 1.1000 | 1.1060 | 1.1080 | 1.1120 | 1.1110 | 1.1200 | 1.1130 | 1.1090 | 1.1020 | 1.0960 |
| size | 17.8180 | 14.8180 | 87.0540 | 46.5490 | 17.7220 | 8.6150 | 9.8140 | 9.8220 | 23.6920 | 4.9410 | 81.9800 | 64.0920 | 34.5690 | 8.9080 | 23.5170 |
| obs_het | 0.0600 | 0.0640 | 0.0600 | 0.0590 | 0.0650 | 0.0590 | 0.0590 | 0.0610 | 0.0600 | 0.0690 | 0.0650 | 0.0600 | 0.0620 | 0.0590 | 0.0570 |
| exp_het | 0.0610 | 0.0640 | 0.0620 | 0.0610 | 0.0640 | 0.0610 | 0.0600 | 0.0610 | 0.0620 | 0.0610 | 0.0650 | 0.0630 | 0.0590 | 0.0560 | 0.0630 |
| uexp_het | 0.0630 | 0.0670 | 0.0630 | 0.0620 | 0.0660 | 0.0650 | 0.0630 | 0.0640 | 0.0640 | 0.0680 | 0.0660 | 0.0630 | 0.0600 | 0.0600 | 0.0640 |
| fis | 0.0120 | -0.0060 | 0.0350 | 0.0280 | -0.0060 | 0.0090 | -0.0060 | -0.0150 | 0.0250 | -0.1150 | 0.0240 | 0.0370 | -0.0130 | -0.0570 | 0.0610 |
| hwe_glb | 1.0000 | 1.0000 | 1.0000 | 1.0000 | 1.0000 | 1.0000 | 1.0000 | 1.0000 | 1.0000 | 1.0000 | 1.0000 | 1.0000 | 1.0000 | 1.0000 | 1.0000 |
| hwe_hom | 1.0000 | 1.0000 | 1.0000 | 1.0000 | 1.0000 | 1.0000 | 1.0000 | 1.0000 | 1.0000 | 1.0000 | 1.0000 | 1.0000 | 1.0000 | 1.0000 | 1.0000 |
| hwe_het | 1.0000 | 1.0000 | 1.0000 | 1.0000 | 1.0000 | 1.0000 | 1.0000 | 1.0000 | 1.0000 | 1.0000 | 1.0000 | 1.0000 | 1.0000 | 1.0000 | 1.0000 |
| fis_lo | -0.0310 | -0.0580 | 0.0260 | 0.0110 | -0.0540 | -0.0960 | -0.0920 | -0.0980 | -0.0090 | -0.3880 | 0.0110 | 0.0240 | -0.0400 | -0.1890 | 0.0260 |
| fis_hi | 0.0050 | -0.0110 | 0.0360 | 0.0260 | -0.0110 | 0.0020 | -0.0140 | -0.0240 | 0.0220 | -0.1150 | 0.0250 | 0.0380 | -0.0180 | -0.0620 | 0.0650 |
| ar_lo | 1.0510 | 1.0470 | 1.1040 | 1.1000 | 1.0530 | 1.0310 | 1.0470 | 1.0430 | 1.0500 | 1.0510 | 1.1090 | 1.1010 | 1.0850 | 1.0450 | 1.0220 |
| ar_hi | 1.1240 | 1.1330 | 1.1240 | 1.1220 | 1.1340 | 1.1200 | 1.1220 | 1.1210 | 1.1220 | 1.1330 | 1.1310 | 1.1250 | 1.1210 | 1.1220 | 1.1190 |
| fis.hierfstat | 0.0392 | 0.0275 | 0.0409 | 0.0383 | 0.0212 | 0.0648 | 0.0434 | 0.0342 | 0.0456 | -0.0145 | 0.0301 | 0.0448 | 0.0006 | -0.0026 | 0.0809 |
| fis.CI.low | 0.0338 | 0.0190 | 0.0370 | 0.0333 | 0.0034 | 0.0614 | 0.0449 | 0.0270 | 0.0441 | -0.0430 | 0.0062 | 0.0431 | -0.0440 | -0.0273 | 0.0954 |
| fis.CI.high | 0.0695 | 0.0588 | 0.0596 | 0.0591 | 0.0410 | 0.1174 | 0.0948 | 0.0768 | 0.0744 | 0.0249 | 0.0296 | 0.0676 | -0.0057 | 0.0315 | 0.1351 |

Table 14: Table2

| | COR | BRZ | CAR | GOM | WNA | SAF | MOZ | RUN | SEY | ARS | SRL | TAI | IND | PNG | AUS | JAP | FIJ |
|---|---|---|---|---|---|---|---|---|---|---|---|---|---|---|---|---|---|
| ar | 1.0610 | 1.0950 | 1.0640 | 1.0860 | 1.0850 | 1.1120 | 1.0870 | 1.1090 | 1.1120 | 1.1140 | 1.1110 | 1.1100 | 1.0960 | 1.1050 | 1.1160 | 1.1100 | 1.0960 |
| size | 15.6540 | 49.0870 | 1.9750 | 36.0400 | 7.8830 | 21.7470 | 10.2580 | 27.3810 | 35.4700 | 14.8430 | 11.8850 | 4.9650 | 3.9590 | 8.9060 | 438.3460 | 43.4770 | 23.5170 |
| obs_het | 0.0360 | 0.0520 | 0.0490 | 0.0480 | 0.0500 | 0.0600 | 0.0560 | 0.0590 | 0.0600 | 0.0620 | 0.0610 | 0.0660 | 0.0610 | 0.0590 | 0.0610 | 0.0610 | 0.0570 |
| exp_het | 0.0390 | 0.0560 | 0.0380 | 0.0530 | 0.0500 | 0.0620 | 0.0600 | 0.0620 | 0.0620 | 0.0630 | 0.0610 | 0.0590 | 0.0540 | 0.0580 | 0.0640 | 0.0610 | 0.0630 |
| uexp_het | 0.0410 | 0.0570 | 0.0510 | 0.0530 | 0.0540 | 0.0630 | 0.0640 | 0.0630 | 0.0630 | 0.0650 | 0.0640 | 0.0660 | 0.0610 | 0.0620 | 0.0640 | 0.0610 | 0.0640 |
| fis | 0.0450 | 0.0480 | -0.2930 | 0.0500 | -0.0250 | 0.0240 | 0.0520 | 0.0400 | 0.0260 | 0.0040 | -0.0050 | -0.1080 | -0.1360 | -0.0270 | 0.0430 | 0.0040 | 0.0610 |
| hwe_glb | 1.0000 | 1.0000 | 1.0000 | 1.0000 | 1.0000 | 1.0000 | 1.0000 | 1.0000 | 1.0000 | 1.0000 | 1.0000 | 1.0000 | 1.0000 | 1.0000 | 0.0000 | 1.0000 | 1.0000 |
| hwe_hom | 1.0000 | 1.0000 | 1.0000 | 1.0000 | 1.0000 | 1.0000 | 1.0000 | 1.0000 | 1.0000 | 1.0000 | 1.0000 | 1.0000 | 1.0000 | 1.0000 | 1.0000 | 1.0000 | 1.0000 |
| hwe_het | 1.0000 | 1.0000 | 1.0000 | 1.0000 | 1.0000 | 1.0000 | 1.0000 | 1.0000 | 1.0000 | 1.0000 | 1.0000 | 1.0000 | 1.0000 | 1.0000 | 0.0000 | 1.0000 | 1.0000 |
| fis_lo | -0.0090 | 0.0330 | -1.0000 | 0.0310 | -0.1450 | -0.0090 | -0.0350 | 0.0100 | 0.0020 | -0.0480 | -0.0770 | -0.3680 | -0.5470 | -0.1260 | 0.0370 | -0.0170 | 0.0260 |
| fis_hi | 0.0440 | 0.0520 | -0.2930 | 0.0530 | -0.0340 | 0.0180 | 0.0450 | 0.0370 | 0.0250 | -0.0030 | -0.0150 | -0.1080 | -0.1360 | -0.0370 | 0.0430 | 0.0020 | 0.0640 |
| ar_lo | 1.0130 | 1.0850 | 1.0330 | 1.0360 | 1.0290 | 1.0510 | 0.9460 | 1.0460 | 1.0600 | 1.0480 | 1.0490 | 1.0520 | 1.0470 | 1.0430 | 1.1050 | 1.0550 | 1.0220 |
| ar_hi | 1.0710 | 1.1070 | 1.0920 | 1.0990 | 1.1020 | 1.1220 | 1.1190 | 1.1210 | 1.1210 | 1.1280 | 1.1280 | 1.1290 | 1.1190 | 1.1180 | 1.1270 | 1.1220 | 1.1190 |
| fis.hierfstat | 0.0750 | 0.0576 | -0.0481 | 0.0638 | 0.0369 | 0.0465 | 0.0959 | 0.0572 | 0.0398 | 0.0370 | 0.0356 | -0.0069 | -0.0098 | 0.0285 | 0.0440 | 0.0155 | 0.0809 |
| fis.CI.low | 0.0785 | 0.0687 | -0.0302 | 0.0736 | 0.0321 | 0.0454 | 0.1015 | 0.0530 | 0.0328 | 0.0261 | 0.0130 | -0.0302 | -0.0282 | 0.0165 | 0.0387 | -0.0142 | 0.0969 |
| fis.CI.high | 0.1389 | 0.1058 | 0.1227 | 0.1164 | 0.0998 | 0.0787 | 0.1564 | 0.0888 | 0.0611 | 0.0675 | 0.0610 | 0.0374 | 0.0545 | 0.0702 | 0.0544 | 0.0171 | 0.1351 |

Table 15: Table3

| | E-PAC | W-ATL | IWP | Japan | Fiji |
|---|---|---|---|---|---|
| ar | 1.1540 | 1.2580 | 1.3450 | 1.2800 | 1.2820 |
| size | 15.6540 | 94.9850 | 577.7590 | 43.4770 | 23.5170 |
| obs_het | 0.0360 | 0.0500 | 0.0610 | 0.0610 | 0.0570 |
| exp_het | 0.0390 | 0.0550 | 0.0640 | 0.0610 | 0.0630 |
| uexp_het | 0.0410 | 0.0550 | 0.0640 | 0.0610 | 0.0640 |
| fis | 0.0450 | 0.0490 | 0.0460 | 0.0040 | 0.0610 |
| hwe_glb | 1.0000 | 1.0000 | 0.0000 | 1.0000 | 1.0000 |
| hwe_hom | 1.0000 | 1.0000 | 1.0000 | 1.0000 | 1.0000 |
| hwe_het | 1.0000 | 1.0000 | 0.0000 | 1.0000 | 1.0000 |
| fis_lo | -0.0070 | 0.0410 | 0.0400 | -0.0180 | 0.0270 |
| fis_hi | 0.0440 | 0.0530 | 0.0460 | 0.0030 | 0.0630 |
| ar_lo | 1.1380 | 1.2350 | 1.3290 | 1.2540 | 1.2520 |
| ar_hi | 1.1690 | 1.2790 | 1.3600 | 1.3030 | 1.3090 |
| fis.hierfstat | 0.0750 | 0.0536 | 0.0472 | 0.0155 | 0.0809 |
| fis.CI.low | 0.0781 | 0.0748 | 0.0421 | -0.0151 | 0.0955 |
| fis.CI.high | 0.1401 | 0.1082 | 0.0579 | 0.0170 | 0.1358 |

### 8.6.1 Number of monomorphic markers per population

```
pop.levels <- c("Costa Rica" , "Brazil","Caribbean Sea","Gulf of Mexico",
                "Western North Atlantic", "Sierra Leone", "South Africa",
                "Mozambique", "Reunion" , "Seychelles", "Arabian Sea",
                "Sri Lanka", "Thailand", "Indonesia", "Papua New Guinea" ,
                "Fitzroy River", "Daly River", "Adelaide River","Darwin Coastal",
                "South Alligator River", "East Alligator River","Blue mud Bay",
                "Roper River","Towns River","Unknown","Wenlock River","Trinity inlet",
                "Clarence River", "Sydney Harbour","Okinawa", "Urauchi River", "Fiji")
BS.dartcap.gl$pop <- factor(BS.dartcap.gl$other$ind.metrics$Site2, levels = pop.levels)
monomorphics <- c()
for (i in 1:length(pop.levels)) {
  x <- BS.dartcap.gl[BS.dartcap.gl$pop == pop.levels[i]]
  x2 <- dartR::gl.filter.monomorphs(x, verbose = 0)
  locs <-  adegenet::nLoc(x) - adegenet::nLoc(x2)
  monomorphics[i] <- locs
}
names(monomorphics) <- pop.levels
shortnames <- c("COR","BRZ", "CAR", "GOM", "WNA", "SIL","SAF", "MOZ", "RUN" ,
                "SEY", "ARS", "SRL", "TAI", "IND", "PNG" , "FZR", "DAR", "ADR",
                "DWC",  "SAR", "EAR","BMB", "ROR","TOR","UNK","WER","TRI","CLR",
                "SYH", "URR","OKI", "FIJ")
knitr::kable(t(as.table(monomorphics)), col.names = shortnames, digits = 4,
             caption = "") %>%
  kableExtra::kable_styling(full_width = FALSE,font_size = 5,
                            latex_options = c( "hold_position"))



pop.levels <- c("Costa Rica" , "Brazil","Caribbean Sea", "Gulf of Mexico",
                "Western North Atlantic", "Sierra Leone", "South Africa",
                "Mozambique", "Reunion" , "Seychelles", "Arabian Sea", "Sri Lanka",
                "Thailand", "Indonesia", "Papua New Guinea" , "Australia", "Japan",
                "Fiji")
BS.dartcap.gl$pop <- factor(BS.dartcap.gl$other$ind.metrics$Site, levels = pop.levels)
monomorphics <- c()
for (i in 1:length(pop.levels)) {
  x <- BS.dartcap.gl[BS.dartcap.gl$pop == pop.levels[i]]
  x2 <- dartR::gl.filter.monomorphs(x, verbose = 0)
  locs <-  adegenet::nLoc(x) - adegenet::nLoc(x2)
  monomorphics[i] <- locs
}
names(monomorphics) <- pop.levels
```

```r
shortnames <- c("COR","BRZ", "CAR", "GOM", "WNA","SIL", "SAF", "MOZ", "RUN" ,
                "SEY", "ARS", "SRL", "TAI", "IND", "PNG" , "AUS", "JAP", "FIJ")
knitr::kable(t(as.table(monomorphics)), col.names = shortnames, digits = 4,
             caption = "") %>%
  kableExtra::kable_styling(full_width = FALSE,font_size = 5,
                            latex_options = c( "hold_position"))



pop.levels <- c("E-PAC","W-ATL","E-ATL","W-IO","N-IO","E-IO","W-PAC","E-IO/W-PAC",
                "Japan","Fiji")
strata <- BS.dartcap.gl$other$ind.metrics$Region
strata[strata %in% c("W-IO","N-IO","E-IO","W-PAC","E-IO/W-PAC")] <- "IWP"
pop.levels <- c("E-PAC","W-ATL","E-ATL","IWP", "Japan","Fiji")
BS.dartcap.gl$pop <- factor(strata, levels = pop.levels)
monomorphics <- c()
for (i in 1:length(pop.levels)) {
  x <- BS.dartcap.gl[BS.dartcap.gl$pop == pop.levels[i]]
  x2 <- dartR::gl.filter.monomorphs(x, verbose = 0)
  locs <-  adegenet::nLoc(x) - adegenet::nLoc(x2)
  monomorphics[i] <- locs
}
names(monomorphics) <- pop.levels
shortnames <- c("E-PAC","W-ATL","E-ATL","IWP", "Japan","Fiji")
knitr::kable(t(as.table(monomorphics)), col.names = shortnames, digits = 4,
             caption = "") %>%
  kableExtra::kable_styling(full_width = FALSE,font_size = 5,
                            latex_options = c( "hold_position"))
```

Table 16:

| COR | BRZ | CAR | GOM | WNA | SIL | SAF | MOZ | RUN | SEY | ARS | SRL | TAI | IND | PNG | FZR | DAR | ADR | DWC | SA |
|---|---|---|---|---|---|---|---|---|---|---|---|---|---|---|---|---|---|---|---|
| 2562 | 1771 | 2811 | 2075 | 2512 | 3340 | 2081 | 2443 | 2010 | 1914 | 2195 | 2353 | 2699 | 2835 | 2512 | 2914 | 1727 | 2178 | 2204 | 136 |

Table 17:

| COR | BRZ | CAR | GOM | WNA | SIL | SAF | MOZ | RUN | SEY | ARS | SRL | TAI | IND | PNG | AUS | JAP | FIJ |
|---|---|---|---|---|---|---|---|---|---|---|---|---|---|---|---|---|---|
| 2562 | 1771 | 2811 | 2075 | 2512 | 3340 | 2081 | 2443 | 2010 | 1914 | 2195 | 2353 | 2699 | 2835 | 2512 | 395 | 1969 | 2114 |

Table 18:

| E-PAC | W-ATL | E-ATL | IWP | Japan | Fiji |
|---|---|---|---|---|---|
| 2562 | 1370 | 3340 | 301 | 1969 | 2114 |

### 8.6.2 Nucleotide diversity and inbreeding

```r
pi.sum <- radiator::pi(
  data = BS.dartcap.tidy,
  read.length = 70,
  parallel.core = parallel::detectCores() - 1,
  path.folder = NULL,
  verbose = TRUE
)


BS.dartcap.tidy2 <- BS.dartcap.tidy
BS.dartcap.tidy2$POP_ID <- as.character(BS.dartcap.tidy2$POP_ID)
unique(BS.dartcap.tidy2$POP_ID)
```

```
BS.dartcap.tidy2$POP_ID[BS.dartcap.tidy2$POP_ID %in% c("Fitzroy_River",
                                                       "Daly_River", "Adelaide_River",
                                                       "Darwin_Coastal",
                                                       "South_Alligator_River",
                                                       "East_Alligator_River",
                                                       "Blue_mud_Bay", "Roper_River",
                                                       "Towns_River","Unknown",
                                                       "Wenlock_River","Trinity_inlet",
                                                       "Clarence_River",
                                                       "Sydney_Harbour")] <- "Australia"
BS.dartcap.tidy2$POP_ID[BS.dartcap.tidy2$POP_ID %in% c("Okinawa",
                                                       "Urauchi_River")] <- "Japan"

pop.levels <- c("Costa_Rica" , "Brazil","Caribbean_Sea", "Gulf_of_Mexico",
                "Western_North_Atlantic", "Sierra_Leone", "South_Africa",
                "Mozambique", "Reunion" , "Seychelles", "Arabian_Sea",
                "Sri_Lanka", "Thailand", "Indonesia", "Papua_New_Guinea" ,
                "Australia", "Japan", "Fiji")
BS.dartcap.tidy2$POP_ID <- factor(BS.dartcap.tidy2$POP_ID, levels = pop.levels)
BS.dartcap.tidy2 <- BS.dartcap.tidy2[order(BS.dartcap.tidy2$POP_ID,
                                           BS.dartcap.tidy2$INDIVIDUALS),]

pi.sum.site <- radiator::pi(
  data = BS.dartcap.tidy2,
  read.length = 70,
  parallel.core = parallel::detectCores() - 1,
  path.folder = NULL,
  verbose = TRUE
)


BS.dartcap.tidy3 <- BS.dartcap.tidy2
BS.dartcap.tidy3$POP_ID <- as.character(BS.dartcap.tidy3$POP_ID)
BS.dartcap.tidy3$POP_ID[BS.dartcap.tidy3$POP_ID %in% c("Costa_Rica")] <- "E-PAC"
BS.dartcap.tidy3$POP_ID[BS.dartcap.tidy3$POP_ID %in% c("Brazil","Caribbean_Sea",
                                                       "Gulf_of_Mexico",
                                                       "Western_North_Atlantic")] <- "W-ATL"
BS.dartcap.tidy3$POP_ID[BS.dartcap.tidy3$POP_ID %in% c("Sierra_Leone")] <- "E-ATL"
BS.dartcap.tidy3$POP_ID[BS.dartcap.tidy3$POP_ID %in% c("South_Africa", "Mozambique",
                                                       "Reunion" , "Seychelles",
                                                       "Arabian_Sea", "Sri_Lanka",
                                                       "Thailand", "Indonesia",
                                                       "Papua_New_Guinea" ,
                                                       "Australia")] <- "IWP"
BS.dartcap.tidy3$POP_ID[BS.dartcap.tidy3$POP_ID %in% c("Okinawa",
                                                       "Urauchi_River")] <- "Japan"
pop.levels <- c("E-PAC", "W-ATL", "E-ATL", "IWP", "Japan", "Fiji")
BS.dartcap.tidy3$POP_ID <- factor(BS.dartcap.tidy3$POP_ID, levels = pop.levels)
BS.dartcap.tidy3 <- BS.dartcap.tidy3[order(BS.dartcap.tidy3$POP_ID,
                                           BS.dartcap.tidy3$INDIVIDUALS),]

pi.sum.Region <- radiator::pi(
  data = BS.dartcap.tidy3,
  read.length = 70,
  parallel.core = parallel::detectCores() - 1,
  path.folder = NULL,
  verbose = TRUE
```

```
)

FIS.sum<- radiator::ibdg_fh(data = BS.dartcap.tidy,
        path.folder = NULL, verbose = TRUE)

save(pi.sum,pi.sum.site,pi.sum.Region, FIS.sum,
     file = "DArTcap_ALL_pi_FIS_Sites2.Rdata")
```

```
load("DArTcap_ALL_pi_FIS_Sites2.Rdata")
pi.sum$boxplot.pi
```

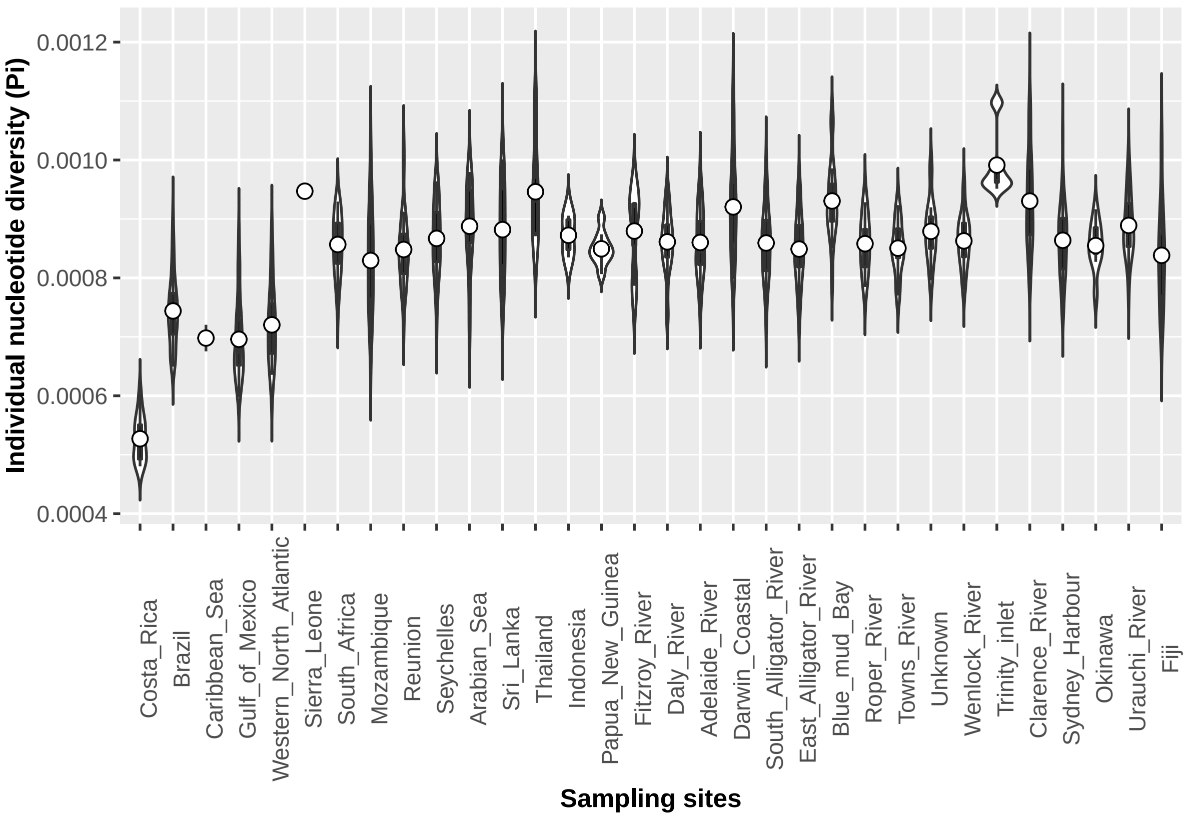


```
pi.sum.site$boxplot.pi
```

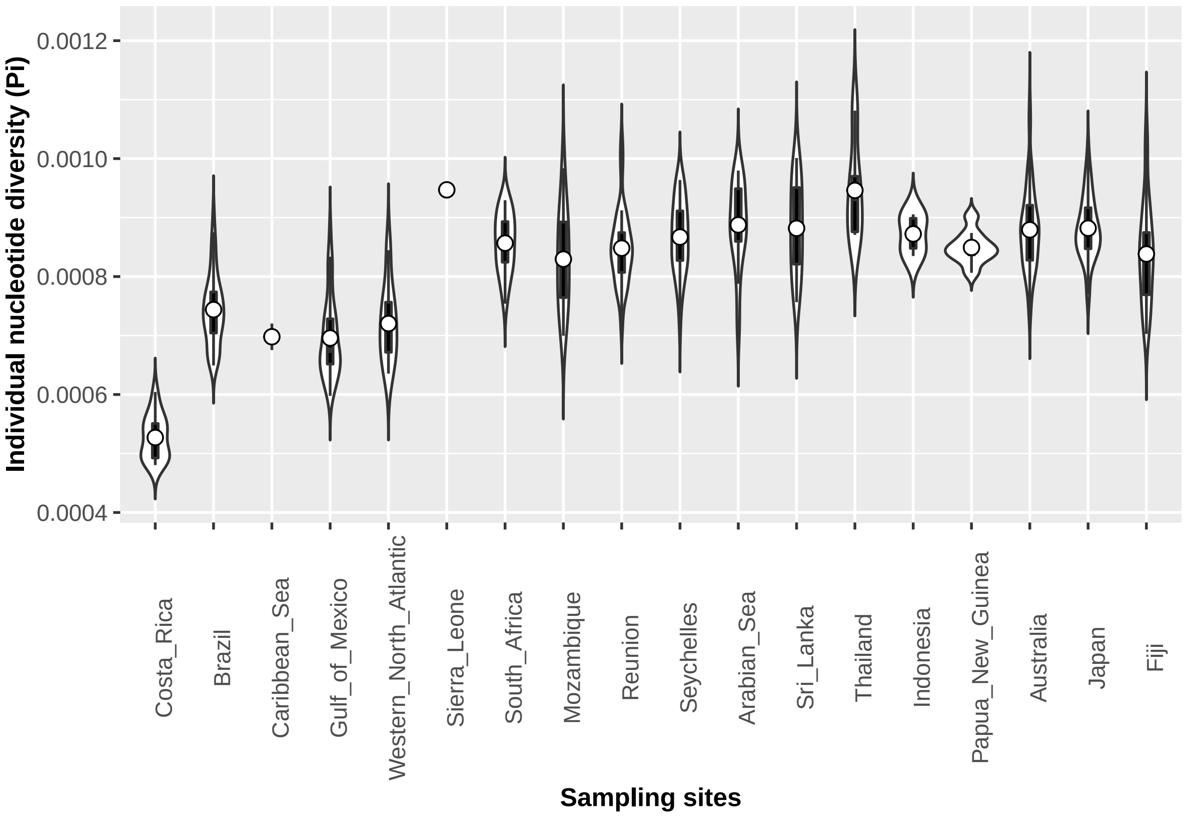


```
pi.sum.Region$boxplot.pi
```

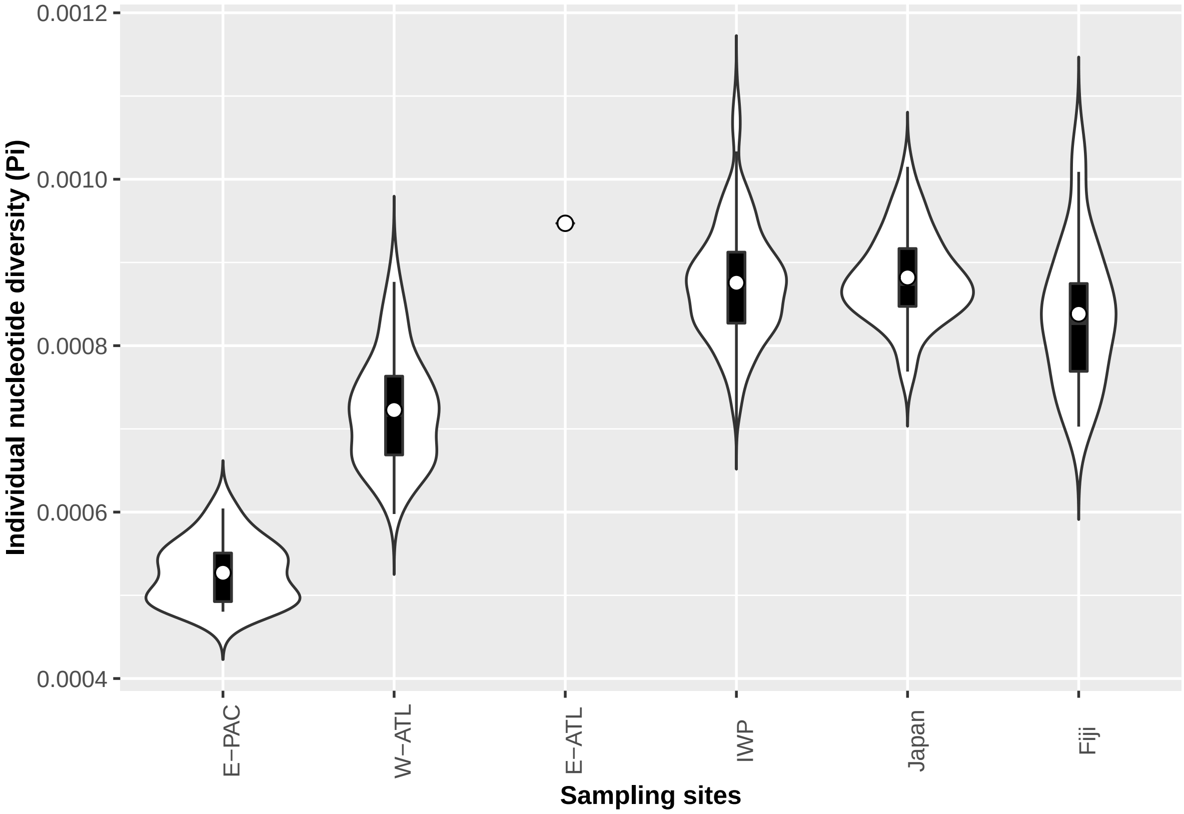


```
FIS.sum$fh.box.plot
```

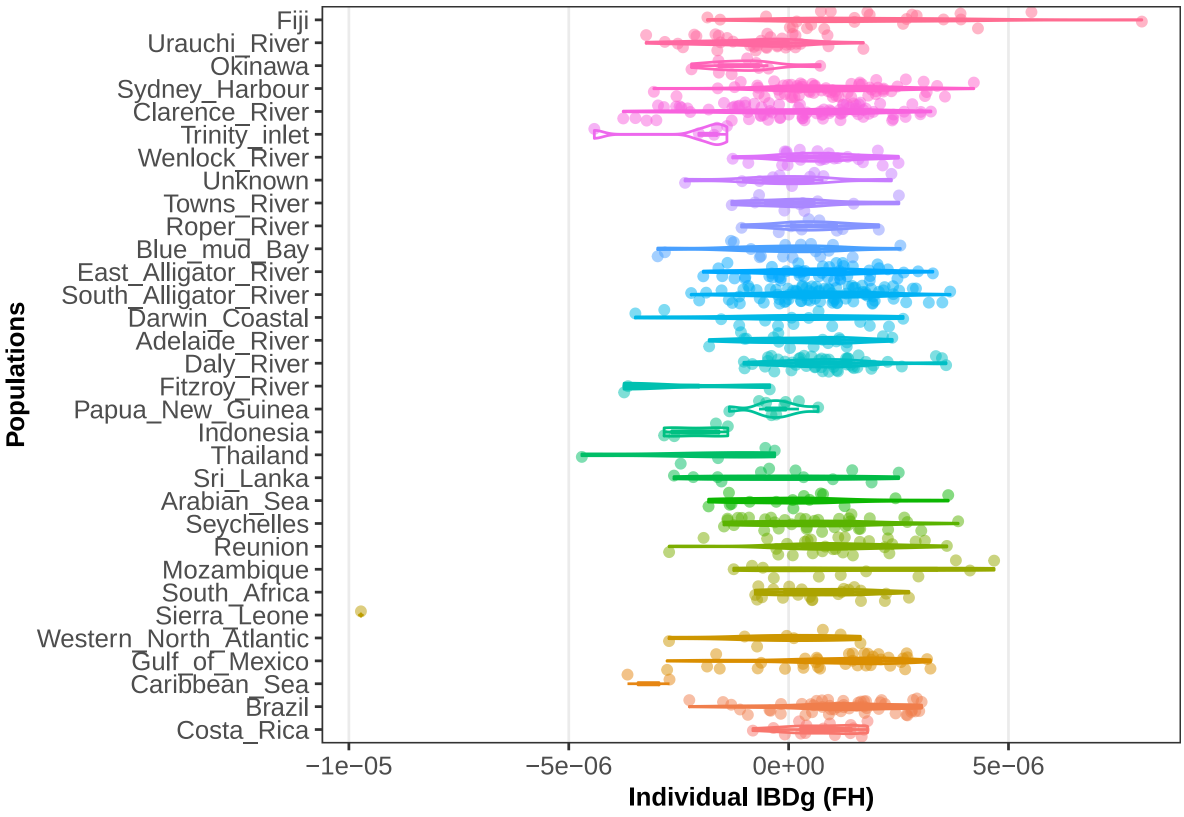


## 8.7 Genetic differentiation

### 8.7.1 Fst per location

```
Fst.gt <- strataG::popStructTest(
    BS.dartcap.gt,
    nrep = 1000,
    stats = "all",
    type = "pairwise",
    keep.null = FALSE,
    quietly = TRUE,
    nb.cores =  parallel::detectCores() - 1,
    write.output = FALSE
  )
save(Fst.gt, file = "DArTcap_ALL_FST_Sites2.Rdata")
readr::write_tsv(Fst.gt$pairwise$result, path = "DArTcap_ALL_FST_Sites2.txt")
```

```
load("DArTcap_ALL_FST_Sites2.Rdata")
kableExtra::kbl(
  Fst.gt$pairwise$result[order(Fst.gt$pairwise$result$Fst,
                                          decreasing = TRUE),
                                    -c(1:4)], digits = 4,
  caption = "Pairwise Fst for 3443 DArTcap loci between the all locations",
  longtable = TRUE, booktabs = TRUE) %>%
  kableExtra::kable_styling(full_width = FALSE,font_size = 5,
                           latex_options = c("repeat_header", "hold_position")) %>%
  kableExtra::landscape()

pop.levels1 <- c("Costa_Rica","Brazil","Caribbean_Sea","Gulf_of_Mexico",
                 "Western_North_Atlantic","Sierra_Leone","South_Africa",
                 "Mozambique","Reunion","Seychelles","Arabian_Sea", "Sri_Lanka",
```

```r
                 "Thailand", "Indonesia", "Papua_New_Guinea" , "Fitzroy_River",
                 "Daly_River", "Adelaide_River","Darwin_Coastal",
                 "South_Alligator_River","East_Alligator_River","Blue_mud_Bay",
                 "Roper_River","Towns_River","Unknown","Wenlock_River",
                 "Trinity_inlet","Clarence_River","Sydney_Harbour","Okinawa",
                 "Urauchi_River","Fiji")

m2 <- Fst.gt$pairwise$result[order(match(
  Fst.gt$pairwise$result$strata.1,pop.levels1)),c(1,2,11)]
Gdf <- igraph::graph.data.frame(m2, directed = FALSE)
m2.1 <- igraph::as_adjacency_matrix(Gdf, names = TRUE, sparse = FALSE,
                                    attr = "Fst", type = 'lower')

m2 <- Fst.gt$pairwise$result[order(match(
  Fst.gt$pairwise$result$strata.1,pop.levels1)),c(1,2,12)]
Gdf <- igraph::graph.data.frame(m2, directed = FALSE)
m2.2 <- igraph::as_adjacency_matrix(Gdf, names = TRUE, sparse = FALSE,
                                    attr = "Fst.p.val", type = 'lower')
m2$Fst.p.adj <- p.adjust(m2$Fst.p.val, "bonferroni")
Gdf <- igraph::graph.data.frame(m2, directed = FALSE)
m2.3 <- igraph::as_adjacency_matrix(Gdf, names = TRUE, sparse = FALSE,
                                    attr = "Fst.p.adj", type = 'lower')

write.csv(m2.1, file = "DArTcap_ALL_FST_Sites2.csv")
write.csv(m2.2, file = "DArTcap_ALL_FST_Pval_Sites2.csv")
write.csv(m2.3, file = "DArTcap_ALL_FST_Pval_bonferroni_Sites2.csv")
```

Table 19: Pairwise Fst for 3443 DArTcap loci between the all locations

| | Chi2 | Chi2.p.val | D | D.p.val | Fis | Fis.p.val | Fst | Fst.p.val | F'st | F'st.p.val | Gst | Gst.p.val | G'st | G'st.p.val | G"st | G"st |
|---|---|---|---|---|---|---|---|---|---|---|---|---|---|---|---|---|
| Costa_Rica (16) v. Fitzroy_River (3) | 15244.4823 | 0.0010 | 0.0170 | 0.0010 | NA | NA | 0.7109 | 0.0094 | 0.7494 | 0.0094 | NA | NA | NA | NA | NA | |
| Costa_Rica (16) v. Indonesia (4) | 16273.1465 | 0.0010 | 0.0174 | 0.0010 | NA | NA | 0.7066 | 0.0016 | 0.7426 | 0.0016 | NA | NA | NA | NA | NA | |
| Costa_Rica (16) v. Sierra_Leone (1) | 11158.8583 | 0.0629 | 0.0114 | 0.0629 | NA | NA | 0.7013 | 1.0000 | 0.7320 | 1.0000 | NA | NA | NA | NA | NA | |
| Costa_Rica (16) v. Okinawa (9) | 21164.4339 | 0.0010 | 0.0181 | 0.0010 | -31.4871 | 1.0000 | 0.6968 | 0.0010 | 0.7370 | 0.0010 | 0.7755 | 0.0010 | 0.8074 | 0.0010 | 0.8915 | 0 |
| Costa_Rica (16) v. Trinity_inlet (5) | 17650.9703 | 0.0010 | 0.0177 | 0.0010 | -108.5163 | 1.0000 | 0.6964 | 0.0013 | 0.7348 | 0.0013 | 0.9277 | 0.0013 | 0.9390 | 0.0013 | 0.9684 | 0 |
| Costa_Rica (16) v. Thailand (5) | 17395.3981 | 0.0010 | 0.0171 | 0.0010 | -128.4526 | 1.0000 | 0.6957 | 0.0013 | 0.7321 | 0.0013 | 0.9375 | 0.0013 | 0.9472 | 0.0013 | 0.9728 | 0 |
| Costa_Rica (16) v. Papua_New_Guinea (9) | 20872.6332 | 0.0010 | 0.0172 | 0.0010 | -25.9715 | 1.0000 | 0.6888 | 0.0010 | 0.7293 | 0.0010 | 0.7465 | 0.0010 | 0.7815 | 0.0010 | 0.8749 | 0 |
| Costa_Rica (16) v. Roper_River (9) | 20692.4019 | 0.0010 | 0.0174 | 0.0010 | -25.5909 | 1.0000 | 0.6838 | 0.0010 | 0.7415 | 0.0010 | 0.7435 | 0.0010 | 0.7791 | 0.0010 | 0.8733 | 0 |
| Costa_Rica (16) v. Towns_River (10) | 21787.7844 | 0.0010 | 0.0173 | 0.0010 | -22.4141 | 1.0000 | 0.6835 | 0.0010 | 0.7295 | 0.0010 | 0.7190 | 0.0010 | 0.7573 | 0.0010 | 0.8588 | 0 |
| Costa_Rica (16) v. Unknown (10) | 21738.0344 | 0.0010 | 0.0172 | 0.0010 | -21.6159 | 1.0000 | 0.6812 | 0.0010 | 0.7267 | 0.0010 | 0.7125 | 0.0010 | 0.7515 | 0.0010 | 0.8549 | 0 |
| Gulf_of_Mexico (37) v. Okinawa (9) | 37928.8235 | 0.0010 | 0.0212 | 0.0010 | -13.0773 | 1.0000 | 0.6797 | 0.0010 | 0.7218 | 0.0010 | 0.6376 | 0.0010 | 0.6881 | 0.0010 | 0.8095 | 0 |
| Gulf_of_Mexico (37) v. Indonesia (4) | 30354.5133 | 0.0010 | 0.0206 | 0.0010 | NA | NA | 0.6790 | 0.0019 | 0.7194 | 0.0019 | NA | NA | NA | NA | NA | |
| Costa_Rica (16) v. Mozambique (11) | 21911.2511 | 0.0010 | 0.0171 | 0.0010 | -21.4465 | 1.0000 | 0.6766 | 0.0010 | 0.7455 | 0.0010 | 0.7102 | 0.0010 | 0.7492 | 0.0010 | 0.8533 | 0 |
| Costa_Rica (16) v. Sri_Lanka (12) | 23381.6592 | 0.0010 | 0.0171 | 0.0010 | -18.2470 | 1.0000 | 0.6765 | 0.0010 | 0.7186 | 0.0010 | 0.6784 | 0.0010 | 0.7208 | 0.0010 | 0.8336 | 0 |
| Gulf_of_Mexico (37) v. Trinity_inlet (5) | 32845.5244 | 0.0010 | 0.0208 | 0.0010 | -18.3811 | 1.0000 | 0.6758 | 0.0013 | 0.7174 | 0.0013 | 0.7219 | 0.0013 | 0.7640 | 0.0013 | 0.8629 | 0 |
| Gulf_of_Mexico (37) v. Papua_New_Guinea (9) | 37442.1062 | 0.0010 | 0.0202 | 0.0010 | -11.4281 | 1.0000 | 0.6735 | 0.0010 | 0.7158 | 0.0010 | 0.6173 | 0.0010 | 0.6692 | 0.0010 | 0.7955 | 0 |
| Gulf_of_Mexico (37) v. Thailand (5) | 32159.8344 | 0.0010 | 0.0201 | 0.0010 | -19.0984 | 1.0000 | 0.6734 | 0.0013 | 0.7138 | 0.0013 | 0.7257 | 0.0014 | 0.7666 | 0.0014 | 0.8648 | 0 |
| Gulf_of_Mexico (37) v. Urauchi_River (35) | 64776.6129 | 0.0010 | 0.0216 | 0.0010 | -7.4585 | 1.0000 | 0.6733 | 0.0010 | 0.7220 | 0.0010 | 0.5440 | 0.0010 | 0.6027 | 0.0010 | 0.7427 | 0 |
| Gulf_of_Mexico (37) v. Towns_River (10) | 38728.3190 | 0.0010 | 0.0203 | 0.0010 | -10.8329 | 1.0000 | 0.6721 | 0.0010 | 0.7175 | 0.0010 | 0.6035 | 0.0010 | 0.6570 | 0.0010 | 0.7861 | 0 |
| Gulf_of_Mexico (37) v. Roper_River (9) | 37383.6943 | 0.0010 | 0.0205 | 0.0010 | -11.3633 | 1.0000 | 0.6713 | 0.0010 | 0.7232 | 0.0010 | 0.6151 | 0.0010 | 0.6677 | 0.0010 | 0.7942 | 0 |
| Gulf_of_Mexico (37) v. Unknown (10) | 38750.4443 | 0.0010 | 0.0203 | 0.0010 | -10.4049 | 1.0000 | 0.6707 | 0.0010 | 0.7158 | 0.0010 | 0.5978 | 0.0010 | 0.6518 | 0.0010 | 0.7821 | 0 |
| Arabian_Sea (15) v. Costa_Rica (16) | 25971.5113 | 0.0010 | 0.0172 | 0.0010 | -14.4992 | 1.0000 | 0.6703 | 0.0010 | 0.7190 | 0.0010 | 0.6412 | 0.0010 | 0.6874 | 0.0010 | 0.8095 | 0 |
| Okinawa (9) v. Western_North_Atlantic (8) | 15535.9050 | 0.0010 | 0.0207 | 0.0010 | -39.0563 | 1.0000 | 0.6700 | 0.0010 | 0.7181 | 0.0010 | 0.8290 | 0.0010 | 0.8561 | 0.0010 | 0.9213 | 0 |
| Fitzroy_River (3) v. Western_North_Atlantic (8) | 9762.9230 | 0.0110 | 0.0199 | 0.0110 | NA | NA | 0.6699 | 0.0171 | 0.7229 | 0.0183 | NA | NA | NA | NA | NA | |
| Indonesia (4) v. Western_North_Atlantic (8) | 10796.1481 | 0.0010 | 0.0201 | 0.0010 | NA | NA | 0.6695 | 0.0011 | 0.7210 | 0.0011 | NA | NA | NA | NA | NA | |
| Costa_Rica (16) v. Urauchi_River (35) | 42234.0313 | 0.0010 | 0.0184 | 0.0010 | -12.5734 | 1.0000 | 0.6693 | 0.0010 | 0.7189 | 0.0010 | 0.6113 | 0.0010 | 0.6604 | 0.0010 | 0.7892 | 0 |
| Gulf_of_Mexico (37) v. Sri_Lanka (12) | 40959.6725 | 0.0010 | 0.0201 | 0.0010 | -9.3431 | 1.0000 | 0.6690 | 0.0010 | 0.7122 | 0.0010 | 0.5772 | 0.0010 | 0.6327 | 0.0010 | 0.7671 | 0 |
| Adelaide_River (18) v. Costa_Rica (16) | 28352.4697 | 0.0010 | 0.0172 | 0.0010 | -13.8701 | 1.0000 | 0.6689 | 0.0010 | 0.7167 | 0.0010 | 0.6302 | 0.0010 | 0.6772 | 0.0010 | 0.8020 | 0 |
| Gulf_of_Mexico (37) v. Mozambique (11) | 38936.2039 | 0.0010 | 0.0202 | 0.0010 | -10.5682 | 1.0000 | 0.6678 | 0.0010 | 0.7265 | 0.0010 | 0.5984 | 0.0010 | 0.6521 | 0.0010 | 0.7823 | 0 |
| Arabian_Sea (15) v. Gulf_of_Mexico (37) | 44493.1758 | 0.0010 | 0.0203 | 0.0010 | -7.9916 | 1.0000 | 0.6674 | 0.0010 | 0.7306 | 0.0010 | 0.5559 | 0.0010 | 0.6134 | 0.0010 | 0.7515 | 0 |
| Adelaide_River (18) v. Gulf_of_Mexico (37) | 47395.8479 | 0.0010 | 0.0203 | 0.0010 | -7.7838 | 1.0000 | 0.6671 | 0.0010 | 0.7290 | 0.0010 | 0.5500 | 0.0010 | 0.6076 | 0.0010 | 0.7469 | 0 |
| Gulf_of_Mexico (37) v. South_Africa (22) | 51302.5655 | 0.0010 | 0.0203 | 0.0010 | -7.0620 | 1.0000 | 0.6645 | 0.0010 | 0.7110 | 0.0010 | 0.5385 | 0.0010 | 0.5969 | 0.0010 | 0.7380 | 0 |
| Gulf_of_Mexico (37) v. Wenlock_River (24) | 53455.4440 | 0.0010 | 0.0205 | 0.0010 | -6.8025 | 1.0000 | 0.6643 | 0.0010 | 0.7121 | 0.0010 | 0.5356 | 0.0010 | 0.5944 | 0.0010 | 0.7358 | 0 |
| Caribbean_Sea (2) v. Okinawa (9) | 9746.0586 | 0.0180 | 0.0200 | 0.0180 | 8.3392 | 1.0000 | 0.6641 | 0.0249 | 0.7174 | 0.0353 | 3.6652 | 0.4674 | 3.0121 | 0.4674 | 1.4313 | 0 |
| Urauchi_River (35) v. Western_North_Atlantic (8) | 37504.7555 | 0.0010 | 0.0212 | 0.0010 | -12.9267 | 1.0000 | 0.6640 | 0.0010 | 0.7085 | 0.0010 | 0.6469 | 0.0010 | 0.6969 | 0.0010 | 0.8159 | 0 |
| Costa_Rica (16) v. Darwin_Coastal (15) | 25781.4259 | 0.0010 | 0.0171 | 0.0010 | -14.2060 | 1.0000 | 0.6638 | 0.0010 | 0.7095 | 0.0010 | 0.6325 | 0.0010 | 0.6794 | 0.0010 | 0.8036 | 0 |
| Fiji (25) v. Gulf_of_Mexico (37) | 53347.0294 | 0.0010 | 0.0208 | 0.0010 | -7.6557 | 1.0000 | 0.6636 | 0.0010 | 0.7246 | 0.0010 | 0.5421 | 0.0010 | 0.6007 | 0.0010 | 0.7411 | 0 |
| Blue_mud_Bay (18) v. Gulf_of_Mexico (37) | 47552.7908 | 0.0010 | 0.0204 | 0.0010 | -7.3318 | 1.0000 | 0.6635 | 0.0010 | 0.7259 | 0.0010 | 0.5416 | 0.0010 | 0.6003 | 0.0010 | 0.7407 | 0 |
| Brazil (50) v. Okinawa (9) | 44575.3713 | 0.0010 | 0.0207 | 0.0010 | -9.8829 | 1.0000 | 0.6634 | 0.0010 | 0.7060 | 0.0010 | 0.5961 | 0.0010 | 0.6501 | 0.0010 | 0.7808 | 0 |
| Gulf_of_Mexico (37) v. Reunion (28) | 56931.1754 | 0.0010 | 0.0205 | 0.0010 | -6.6372 | 1.0000 | 0.6632 | 0.0010 | 0.7172 | 0.0010 | 0.5320 | 0.0010 | 0.5909 | 0.0010 | 0.7330 | 0 |
| Darwin_Coastal (15) v. Gulf_of_Mexico (37) | 44211.4873 | 0.0010 | 0.0201 | 0.0010 | -7.8714 | 1.0000 | 0.6626 | 0.0010 | 0.7259 | 0.0010 | 0.5488 | 0.0010 | 0.6067 | 0.0010 | 0.7461 | 0 |
| Blue_mud_Bay (18) v. Costa_Rica (16) | 28324.6037 | 0.0010 | 0.0174 | 0.0010 | -12.6979 | 1.0000 | 0.6623 | 0.0010 | 0.7108 | 0.0010 | 0.6165 | 0.0010 | 0.6650 | 0.0010 | 0.7928 | 0 |
| Costa_Rica (16) v. South_Africa (22) | 31374.4245 | 0.0010 | 0.0172 | 0.0010 | -12.3324 | 1.0000 | 0.6623 | 0.0010 | 0.7094 | 0.0010 | 0.6108 | 0.0010 | 0.6594 | 0.0010 | 0.7885 | 0 |
| Brazil (50) v. Urauchi_River (35) | 74289.2054 | 0.0010 | 0.0211 | 0.0010 | -5.9170 | 1.0000 | 0.6621 | 0.0010 | 0.7099 | 0.0010 | 0.5202 | 0.0010 | 0.5801 | 0.0010 | 0.7238 | 0 |
| Brazil (50) v. Fitzroy_River (3) | 32393.5060 | 0.0010 | 0.0200 | 0.0010 | NA | NA | 0.6615 | 0.0036 | 0.7040 | 0.0036 | NA | NA | NA | NA | NA | |
| Brazil (50) v. Indonesia (4) | 34978.7881 | 0.0010 | 0.0201 | 0.0010 | NA | NA | 0.6610 | 0.0016 | 0.7023 | 0.0016 | NA | NA | NA | NA | NA | |
| Gulf_of_Mexico (37) v. Seychelles (36) | 64246.2659 | 0.0010 | 0.0204 | 0.0010 | -6.2762 | 1.0000 | 0.6609 | 0.0010 | 0.7120 | 0.0010 | 0.5254 | 0.0010 | 0.5846 | 0.0010 | 0.7277 | 0 |
| Costa_Rica (16) v. Wenlock_River (24) | 33052.2118 | 0.0010 | 0.0174 | 0.0010 | -11.5217 | 1.0000 | 0.6609 | 0.0010 | 0.7097 | 0.0010 | 0.6018 | 0.0010 | 0.6513 | 0.0010 | 0.7823 | 0 |
| Papua_New_Guinea (9) v. Western_North_Atlantic (8) | 15342.6408 | 0.0010 | 0.0198 | 0.0010 | -29.9304 | 1.0000 | 0.6609 | 0.0010 | 0.7091 | 0.0010 | 0.7937 | 0.0010 | 0.8255 | 0.0010 | 0.9027 | 0 |
| East_Alligator_River (47) v. Gulf_of_Mexico (37) | 73857.5801 | 0.0010 | 0.0201 | 0.0010 | -5.6121 | 1.0000 | 0.6602 | 0.0010 | 0.7153 | 0.0010 | 0.5204 | 0.0010 | 0.5796 | 0.0010 | 0.7235 | 0 |
| Caribbean_Sea (2) v. Urauchi_River (35) | 29663.0390 | 0.0020 | 0.0203 | 0.0020 | 14.4289 | 0.6792 | 0.6599 | 0.0080 | 0.7175 | 0.0174 | 1.6674 | 0.6792 | 1.5296 | 0.6792 | 1.1986 | 0 |
| Costa_Rica (16) v. Fiji (25) | 32838.2436 | 0.0010 | 0.0177 | 0.0010 | -12.8862 | 1.0000 | 0.6594 | 0.0010 | 0.7427 | 0.0010 | 0.6143 | 0.0010 | 0.6629 | 0.0010 | 0.7912 | 0 |
| Caribbean_Sea (2) v. Indonesia (4) | 5575.3583 | 0.0589 | 0.0193 | 0.0589 | NA | NA | 0.6592 | 0.0741 | 0.7108 | 0.1300 | NA | NA | NA | NA | NA | |

Table 19: Pairwise Fst for 3443 DArTcap loci between the all locations *(continued)*

| | Chi2 | Chi2.p.val | D | D.p.val | Fis | Fis.p.val | Fst | Fst.p.val | F’st | F’st.p.val | Gst | Gst.p.val | G’st | G’st.p.val | G”st | G”st |
|---|---|---|---|---|---|---|---|---|---|---|---|---|---|---|---|---|
| Brazil (50) v. Trinity_inlet (5) | 38191.2400 | 0.0010 | 0.0203 | 0.0010 | -12.3803 | 1.0000 | 0.6591 | 0.0012 | 0.7013 | 0.0012 | 0.6589 | 0.0013 | 0.7079 | 0.0013 | 0.8239 | 0 |
| Trinity_inlet (5) v. Western_North_Atlantic (8) | 11938.2254 | 0.0020 | 0.0205 | 0.0020 | -509.0860 | 1.0000 | 0.6586 | 0.0020 | 0.7105 | 0.0020 | 0.9854 | 0.0020 | 0.9880 | 0.0020 | 0.9939 | 0 |
| Thailand (5) v. Western_North_Atlantic (8) | 11762.3727 | 0.0010 | 0.0196 | 0.0010 | -1708.8139 | 1.0000 | 0.6580 | 0.0010 | 0.7092 | 0.0010 | 0.9956 | 0.0010 | 0.9963 | 0.0010 | 0.9982 | 0 |
| Costa_Rica (16) v. Reunion (28) | 35822.7035 | 0.0010 | 0.0173 | 0.0010 | -11.2181 | 1.0000 | 0.6579 | 0.0010 | 0.7153 | 0.0010 | 0.5972 | 0.0010 | 0.6469 | 0.0010 | 0.7790 | 0 |
| Brazil (50) v. Papua_New_Guinea (9) | 43914.5156 | 0.0010 | 0.0197 | 0.0010 | -8.8114 | 1.0000 | 0.6576 | 0.0010 | 0.7002 | 0.0010 | 0.5789 | 0.0010 | 0.6338 | 0.0010 | 0.7681 | 0 |
| Towns_River (10) v. Western_North_Atlantic (8) | 16205.3138 | 0.0010 | 0.0199 | 0.0010 | -25.5219 | 1.0000 | 0.6571 | 0.0010 | 0.7056 | 0.0010 | 0.7668 | 0.0010 | 0.8024 | 0.0010 | 0.8882 | 0 |
| Daly_River (49) v. Gulf_of_Mexico (37) | 75823.0284 | 0.0010 | 0.0203 | 0.0010 | -5.3530 | 1.0000 | 0.6571 | 0.0010 | 0.7122 | 0.0010 | 0.5163 | 0.0010 | 0.5760 | 0.0010 | 0.7204 | 0 |
| Brazil (50) v. Towns_River (10) | 45629.7634 | 0.0010 | 0.0198 | 0.0010 | -8.2103 | 1.0000 | 0.6568 | 0.0010 | 0.7019 | 0.0010 | 0.5654 | 0.0010 | 0.6217 | 0.0010 | 0.7584 | 0 |
| Brazil (50) v. Thailand (5) | 37344.9627 | 0.0010 | 0.0195 | 0.0010 | -12.8491 | 1.0000 | 0.6564 | 0.0012 | 0.6977 | 0.0012 | 0.6624 | 0.0013 | 0.7103 | 0.0013 | 0.8257 | 0 |
| Brazil (50) v. Roper_River (9) | 43908.8353 | 0.0010 | 0.0200 | 0.0010 | -8.6219 | 1.0000 | 0.6556 | 0.0010 | 0.7057 | 0.0010 | 0.5747 | 0.0010 | 0.6304 | 0.0010 | 0.7653 | 0 |
| Brazil (50) v. Unknown (10) | 45557.7229 | 0.0010 | 0.0198 | 0.0010 | -8.0175 | 1.0000 | 0.6555 | 0.0010 | 0.7003 | 0.0010 | 0.5615 | 0.0010 | 0.6181 | 0.0010 | 0.7554 | 0 |
| Brazil (50) v. Sri_Lanka (12) | 48090.5646 | 0.0010 | 0.0196 | 0.0010 | -7.4193 | 1.0000 | 0.6543 | 0.0010 | 0.6976 | 0.0010 | 0.5455 | 0.0010 | 0.6030 | 0.0010 | 0.7431 | 0 |
| Gulf_of_Mexico (37) v. Sydney_Harbour (65) | 89170.2545 | 0.0010 | 0.0203 | 0.0010 | -4.4543 | 1.0000 | 0.6543 | 0.0010 | 0.7080 | 0.0010 | 0.5102 | 0.0010 | 0.5703 | 0.0010 | 0.7154 | 0 |
| Caribbean_Sea (2) v. Fitzroy_River (3) | 4693.6426 | 0.1159 | 0.0192 | 0.1159 | NA | NA | 0.6543 | 0.2442 | 0.7213 | 1.0000 | NA | NA | NA | NA | NA | [illegible] |
| Unknown (10) v. Western_North_Atlantic (8) | 16181.0705 | 0.0010 | 0.0198 | 0.0010 | -24.0526 | 1.0000 | 0.6543 | 0.0010 | 0.7029 | 0.0010 | 0.7576 | 0.0010 | 0.7944 | 0.0010 | 0.8830 | 0 |
| Roper_River (9) v. Western_North_Atlantic (8) | 15145.0152 | 0.0010 | 0.0201 | 0.0010 | -28.9918 | 1.0000 | 0.6542 | 0.0010 | 0.7035 | 0.0010 | 0.7892 | 0.0010 | 0.8219 | 0.0010 | 0.9005 | 0 |
| Gulf_of_Mexico (37) v. South_Alligator_River (88) | 108650.5315 | 0.0010 | 0.0201 | 0.0010 | -3.7651 | 1.0000 | 0.6539 | 0.0010 | 0.7057 | 0.0010 | 0.5076 | 0.0010 | 0.5676 | 0.0010 | 0.7132 | 0 |
| Adelaide_River (18) v. Brazil (50) | 55498.2702 | 0.0010 | 0.0198 | 0.0010 | -6.1093 | 1.0000 | 0.6539 | 0.0010 | 0.7104 | 0.0010 | 0.5225 | 0.0010 | 0.5816 | 0.0010 | 0.7252 | 0 |
| Arabian_Sea (15) v. Brazil (50) | 52205.7665 | 0.0010 | 0.0198 | 0.0010 | -6.3824 | 1.0000 | 0.6538 | 0.0010 | 0.7111 | 0.0010 | 0.5278 | 0.0010 | 0.5868 | 0.0010 | 0.7296 | 0 |
| Costa_Rica (16) v. Seychelles (36) | 41929.4198 | 0.0010 | 0.0172 | 0.0010 | -10.2066 | 1.0000 | 0.6532 | 0.0010 | 0.7059 | 0.0010 | 0.5844 | 0.0010 | 0.6350 | 0.0010 | 0.7696 | 0 |
| Brazil (50) v. Mozambique (11) | 45890.2124 | 0.0010 | 0.0197 | 0.0010 | -8.0143 | 1.0000 | 0.6532 | 0.0010 | 0.7086 | 0.0010 | 0.5607 | 0.0010 | 0.6171 | 0.0010 | 0.7546 | 0 |
| Sri_Lanka (12) v. Western_North_Atlantic (8) | 17932.6616 | 0.0010 | 0.0197 | 0.0010 | -19.3031 | 1.0000 | 0.6531 | 0.0010 | 0.7009 | 0.0010 | 0.7186 | 0.0010 | 0.7601 | 0.0010 | 0.8604 | 0 |
| Brazil (50) v. Wenlock_River (24) | 62086.1831 | 0.0010 | 0.0200 | 0.0010 | -5.4436 | 1.0000 | 0.6525 | 0.0010 | 0.6994 | 0.0010 | 0.5114 | 0.0010 | 0.5713 | 0.0010 | 0.7163 | 0 |
| Brazil (50) v. South_Africa (22) | 59708.1807 | 0.0010 | 0.0198 | 0.0010 | -5.6168 | 1.0000 | 0.6522 | 0.0010 | 0.6981 | 0.0010 | 0.5134 | 0.0010 | 0.5730 | 0.0010 | 0.7178 | 0 |
| Brazil (50) v. Fiji (25) | 62085.9734 | 0.0010 | 0.0203 | 0.0010 | -6.1466 | 1.0000 | 0.6521 | 0.0010 | 0.7183 | 0.0010 | 0.5169 | 0.0010 | 0.5767 | 0.0010 | 0.7209 | 0 |
| Brazil (50) v. Reunion (28) | 65838.2138 | 0.0010 | 0.0199 | 0.0010 | -5.1468 | 1.0000 | 0.6518 | 0.0010 | 0.7038 | 0.0010 | 0.5076 | 0.0010 | 0.5676 | 0.0010 | 0.7132 | 0 |
| Adelaide_River (18) v. Western_North_Atlantic (8) | 23060.0428 | 0.0010 | 0.0198 | 0.0010 | -13.9178 | 1.0000 | 0.6515 | 0.0010 | 0.6982 | 0.0010 | 0.6626 | 0.0010 | 0.7104 | 0.0010 | 0.8258 | 0 |
| Costa_Rica (16) v. East_Alligator_River (47) | 50018.0707 | 0.0010 | 0.0171 | 0.0010 | -9.3118 | 1.0000 | 0.6514 | 0.0010 | 0.7000 | 0.0010 | 0.5760 | 0.0010 | 0.6269 | 0.0010 | 0.7633 | 0 |
| Blue_mud_Bay (18) v. Brazil (50) | 55679.1565 | 0.0010 | 0.0199 | 0.0010 | -5.7981 | 1.0000 | 0.6509 | 0.0010 | 0.7078 | 0.0010 | 0.5151 | 0.0010 | 0.5750 | 0.0010 | 0.7195 | 0 |
| Mozambique (11) v. Western_North_Atlantic (8) | 16446.4730 | 0.0010 | 0.0198 | 0.0010 | -23.8312 | 1.0000 | 0.6505 | 0.0010 | 0.6993 | 0.0010 | 0.7559 | 0.0010 | 0.7928 | 0.0010 | 0.8820 | 0 |
| Brazil (50) v. Seychelles (36) | 73511.3689 | 0.0010 | 0.0198 | 0.0010 | -4.9091 | 1.0000 | 0.6504 | 0.0010 | 0.7001 | 0.0010 | 0.5026 | 0.0010 | 0.5628 | 0.0010 | 0.7090 | 0 |
| Caribbean_Sea (2) v. Papua_New_Guinea (9) | 9592.1001 | 0.0280 | 0.0191 | 0.0280 | 8.7392 | 1.0000 | 0.6504 | 0.0426 | 0.7048 | 0.0592 | 3.1407 | 0.5225 | 2.6339 | 0.5225 | 1.3946 | 0 |
| Brazil (50) v. East_Alligator_River (47) | 83389.1059 | 0.0010 | 0.0196 | 0.0010 | -4.4497 | 1.0000 | 0.6503 | 0.0010 | 0.6977 | 0.0010 | 0.4993 | 0.0010 | 0.5593 | 0.0010 | 0.7061 | 0 |
| East_Alligator_River (47) v. Western_North_Atlantic (8) | 46048.2345 | 0.0010 | 0.0197 | 0.0010 | -8.9520 | 1.0000 | 0.6495 | 0.0010 | 0.6938 | 0.0010 | 0.5991 | 0.0010 | 0.6526 | 0.0010 | 0.7828 | 0 |
| Brazil (50) v. Darwin_Coastal (15) | 51918.8432 | 0.0010 | 0.0196 | 0.0010 | -6.1940 | 1.0000 | 0.6495 | 0.0010 | 0.6944 | 0.0010 | 0.5202 | 0.0010 | 0.5796 | 0.0010 | 0.7235 | 0 |
| Arabian_Sea (15) v. Western_North_Atlantic (8) | 20524.2457 | 0.0010 | 0.0199 | 0.0010 | -14.8171 | 1.0000 | 0.6495 | 0.0010 | 0.6971 | 0.0010 | 0.6748 | 0.0010 | 0.7215 | 0.0010 | 0.8337 | 0 |
| South_Africa (22) v. Western_North_Atlantic (8) | 26233.0639 | 0.0010 | 0.0199 | 0.0010 | -12.2867 | 1.0000 | 0.6488 | 0.0010 | 0.6951 | 0.0010 | 0.6416 | 0.0010 | 0.6916 | 0.0010 | 0.8121 | 0 |
| Wenlock_River (24) v. Western_North_Atlantic (8) | 27981.0891 | 0.0010 | 0.0201 | 0.0010 | -11.5652 | 1.0000 | 0.6487 | 0.0010 | 0.6949 | 0.0010 | 0.6333 | 0.0010 | 0.6842 | 0.0010 | 0.8066 | 0 |
| Reunion (28) v. Western_North_Atlantic (8) | 30966.6961 | 0.0010 | 0.0200 | 0.0010 | -11.2130 | 1.0000 | 0.6485 | 0.0010 | 0.6943 | 0.0010 | 0.6274 | 0.0010 | 0.6788 | 0.0010 | 0.8026 | 0 |
| Brazil (50) v. Daly_River (49) | 85531.1342 | 0.0010 | 0.0198 | 0.0010 | -4.1581 | 1.0000 | 0.6478 | 0.0010 | 0.6959 | 0.0010 | 0.4953 | 0.0010 | 0.5558 | 0.0010 | 0.7030 | 0 |
| Fiji (25) v. Western_North_Atlantic (8) | 27619.8196 | 0.0010 | 0.0203 | 0.0010 | -13.2118 | 1.0000 | 0.6477 | 0.0010 | 0.6944 | 0.0010 | 0.6488 | 0.0010 | 0.6984 | 0.0010 | 0.8171 | 0 |
| Seychelles (36) v. Western_North_Atlantic (8) | 37388.1295 | 0.0010 | 0.0199 | 0.0010 | -10.1738 | 1.0000 | 0.6475 | 0.0010 | 0.6927 | 0.0010 | 0.6128 | 0.0010 | 0.6654 | 0.0010 | 0.7925 | 0 |
| Costa_Rica (16) v. Daly_River (49) | 51564.8265 | 0.0010 | 0.0172 | 0.0010 | -8.6238 | 1.0000 | 0.6466 | 0.0010 | 0.6962 | 0.0010 | 0.5682 | 0.0010 | 0.6200 | 0.0010 | 0.7577 | 0 |
| Brazil (50) v. South_Alligator_River (88) | 118954.5484 | 0.0010 | 0.0196 | 0.0010 | -2.9357 | 1.0000 | 0.6460 | 0.0010 | 0.6968 | 0.0010 | 0.4895 | 0.0010 | 0.5500 | 0.0010 | 0.6979 | 0 |
| Brazil (50) v. Sydney_Harbour (65) | 99187.7704 | 0.0010 | 0.0198 | 0.0010 | -3.4957 | 1.0000 | 0.6458 | 0.0010 | 0.6981 | 0.0010 | 0.4910 | 0.0010 | 0.5517 | 0.0010 | 0.6993 | 0 |
| Clarence_River (83) v. Gulf_of_Mexico (37) | 104026.9077 | 0.0010 | 0.0203 | 0.0010 | -3.6088 | 1.0000 | 0.6458 | 0.0010 | 0.6984 | 0.0010 | 0.5003 | 0.0010 | 0.5612 | 0.0010 | 0.7075 | 0 |
| South_Alligator_River (88) v. Western_North_Atlantic (8) | 77018.9057 | 0.0010 | 0.0197 | 0.0010 | -5.6641 | 1.0000 | 0.6456 | 0.0010 | 0.6895 | 0.0010 | 0.5607 | 0.0010 | 0.6174 | 0.0010 | 0.7548 | 0 |
| Gulf_of_Mexico (37) v. Sierra_Leone (1) | 18859.1589 | 0.0170 | 0.0130 | 0.0170 | 3.5388 | 1.0000 | 0.6452 | 1.0000 | 0.6819 | 1.0000 | -4.0811 | 1.0000 | -2.4174 | 1.0000 | 2.1091 | 1 |
| Daly_River (49) v. Western_North_Atlantic (8) | 47643.4225 | 0.0010 | 0.0198 | 0.0010 | -8.2111 | 1.0000 | 0.6447 | 0.0010 | 0.6896 | 0.0010 | 0.5890 | 0.0010 | 0.6437 | 0.0010 | 0.7758 | 0 |
| Caribbean_Sea (2) v. East_Alligator_River (47) | 37280.7916 | 0.0020 | 0.0189 | 0.0020 | 23.9100 | 0.8542 | 0.6442 | 0.0042 | 0.6989 | 0.0076 | 1.2825 | 0.0167 | 1.2298 | 0.0208 | 1.1007 | 0 |
| Blue_mud_Bay (18) v. Western_North_Atlantic (8) | 22958.2348 | 0.0010 | 0.0200 | 0.0010 | -12.7907 | 1.0000 | 0.6441 | 0.0010 | 0.6917 | 0.0010 | 0.6485 | 0.0010 | 0.6981 | 0.0010 | 0.8169 | 0 |
| Caribbean_Sea (2) v. Towns_River (10) | 10301.4960 | 0.0210 | 0.0192 | 0.0210 | 9.2722 | 1.0000 | 0.6441 | 0.0358 | 0.7094 | 0.0488 | 2.8210 | 0.6443 | 2.3965 | 0.6443 | 1.3655 | 0 |
| Sydney_Harbour (65) v. Western_North_Atlantic (8) | 59441.3916 | 0.0010 | 0.0198 | 0.0010 | -6.8854 | 1.0000 | 0.6437 | 0.0010 | 0.6883 | 0.0010 | 0.5736 | 0.0010 | 0.6295 | 0.0010 | 0.7646 | 0 |

Table 19: Pairwise Fst for 3443 DArTcap loci between the all locations *(continued)*

| | Chi2 | Chi2.p.val | D | D.p.val | Fis | Fis.p.val | Fst | Fst.p.val | F'st | F'st.p.val | Gst | Gst.p.val | G'st | G'st.p.val | G"st | G"st |
|---|---|---|---|---|---|---|---|---|---|---|---|---|---|---|---|---|
| Costa_Rica (16) v. Sydney_Harbour (65) | 62651.9638 | 0.0010 | 0.0172 | 0.0010 | -7.4666 | 1.0000 | 0.6424 | 0.0010 | 0.6973 | 0.0010 | 0.5579 | 0.0010 | 0.6104 | 0.0010 | 0.7499 | 0 |
| Darwin_Coastal (15) v. Western_North_Atlantic (8) | 20353.8472 | 0.0010 | 0.0197 | 0.0010 | -14.4365 | 1.0000 | 0.6424 | 0.0010 | 0.6904 | 0.0010 | 0.6666 | 0.0010 | 0.7141 | 0.0010 | 0.8285 | 0 |
| Adelaide_River (18) v. Caribbean_Sea (2) | 16478.8830 | 0.0050 | 0.0191 | 0.0050 | 12.7618 | 0.6869 | 0.6417 | 0.0082 | 0.6854 | 0.0082 | 1.8326 | 0.5410 | 1.6578 | 0.6353 | 1.2322 | 0 |
| Caribbean_Sea (2) v. South_Alligator_River (88) | 65205.6279 | 0.0010 | 0.0190 | 0.0010 | -279.1614 | 1.0000 | 0.6413 | 0.0025 | 0.6978 | 0.0041 | 0.9847 | 0.0042 | 0.9873 | 0.0042 | 0.9936 | 0 |
| Caribbean_Sea (2) v. Unknown (10) | 10304.5492 | 0.0180 | 0.0191 | 0.0180 | 9.3650 | 1.0000 | 0.6410 | 0.0441 | 0.7051 | 0.0723 | 2.7377 | 0.6810 | 2.3348 | 0.6810 | 1.3571 | 0 |
| Caribbean_Sea (2) v. Seychelles (36) | 29492.5761 | 0.0050 | 0.0191 | 0.0050 | 18.4682 | 0.8259 | 0.6409 | 0.0107 | 0.7027 | 0.0204 | 1.4205 | 0.1473 | 1.3387 | 0.3661 | 1.1399 | 0 |
| Costa_Rica (16) v. South_Alligator_River (88) | 78976.1339 | 0.0010 | 0.0170 | 0.0010 | -6.1985 | 1.0000 | 0.6409 | 0.0010 | 0.6929 | 0.0010 | 0.5482 | 0.0010 | 0.6011 | 0.0010 | 0.7423 | 0 |
| Caribbean_Sea (2) v. Sri_Lanka (12) | 11881.2980 | 0.0090 | 0.0189 | 0.0090 | 10.4803 | 1.0000 | 0.6407 | 0.0120 | 0.6951 | 0.0142 | 2.3070 | 0.6416 | 2.0162 | 0.7832 | 1.3073 | 0 |
| Caribbean_Sea (2) v. Reunion (28) | 23726.1873 | 0.0030 | 0.0191 | 0.0030 | 16.1387 | 0.7800 | 0.6401 | 0.0080 | 0.7109 | 0.0145 | 1.5290 | 0.2750 | 1.4236 | 0.5850 | 1.1675 | 0 |
| Sierra_Leone (1) v. Western_North_Atlantic (8) | 6383.0393 | 0.1049 | 0.0128 | 0.1049 | NA | NA | 0.6399 | 1.0000 | 0.6928 | 1.0000 | NA | NA | NA | NA | NA | |
| Caribbean_Sea (2) v. Thailand (5) | 6365.8728 | 0.0659 | 0.0190 | 0.0659 | 6.5399 | 1.0000 | 0.6397 | 0.0876 | 0.6900 | 0.1204 | 7.7722 | 0.1447 | 5.9532 | 0.1447 | 1.5646 | 0 |
| Caribbean_Sea (2) v. Wenlock_River (24) | 20891.2023 | 0.0030 | 0.0192 | 0.0030 | 15.4454 | 0.8462 | 0.6397 | 0.0062 | 0.6990 | 0.0096 | 1.5702 | 0.1748 | 1.4554 | 0.4441 | 1.1772 | 0 |
| Caribbean_Sea (2) v. South_Africa (22) | 19348.6504 | 0.0060 | 0.0191 | 0.0060 | 14.6472 | 0.7519 | 0.6396 | 0.0123 | 0.6969 | 0.0189 | 1.6357 | 0.3722 | 1.5064 | 0.6692 | 1.1921 | 0 |
| Caribbean_Sea (2) v. Roper_River (9) | 9355.1430 | 0.0170 | 0.0194 | 0.0170 | 8.7860 | 1.0000 | 0.6390 | 0.0375 | 0.7344 | 0.0688 | 3.0794 | 0.4113 | 2.5844 | 0.5455 | 1.3884 | 0 |
| Caribbean_Sea (2) v. Fiji (25) | 20234.6055 | 0.0040 | 0.0196 | 0.0040 | 13.8263 | 0.4231 | 0.6389 | 0.0606 | 0.7519 | 0.1538 | 1.7211 | 0.4231 | 1.5712 | 0.4231 | 1.2099 | 0 |
| Brazil (50) v. Clarence_River (83) | 114423.6165 | 0.0010 | 0.0198 | 0.0010 | -2.8508 | 1.0000 | 0.6385 | 0.0010 | 0.6913 | 0.0010 | 0.4825 | 0.0010 | 0.5439 | 0.0010 | 0.6923 | 0 |
| Caribbean_Sea (2) v. Daly_River (49) | 38671.9822 | 0.0010 | 0.0191 | 0.0010 | 26.5220 | 0.6644 | 0.6385 | 0.0018 | 0.6942 | 0.0033 | 1.2399 | 0.0034 | 1.1954 | 0.0034 | 1.0873 | 0 |
| Caribbean_Sea (2) v. Sydney_Harbour (65) | 49538.2031 | 0.0010 | 0.0191 | 0.0010 | 49.8580 | 0.0079 | 0.6383 | 0.0024 | 0.6994 | 0.0039 | 1.1054 | 0.0039 | 1.0867 | 0.0039 | 1.0412 | 0 |
| Caribbean_Sea (2) v. Trinity_inlet (5) | 6416.0571 | 0.0569 | 0.0196 | 0.0569 | 6.5884 | 1.0000 | 0.6378 | 0.0945 | 0.6940 | 0.1652 | 7.0198 | 0.3540 | 5.3956 | 0.3540 | 1.5481 | 0 |
| Arabian_Sea (15) v. Caribbean_Sea (2) | 14193.4109 | 0.0080 | 0.0192 | 0.0080 | 12.1323 | 0.6689 | 0.6376 | 0.0150 | 0.6820 | 0.0150 | 1.9032 | 0.0912 | 1.7105 | 0.2736 | 1.2447 | 0 |
| Caribbean_Sea (2) v. Mozambique (11) | 10554.8552 | 0.0150 | 0.0190 | 0.0150 | 9.3888 | 1.0000 | 0.6367 | 0.0620 | 0.7509 | 0.3571 | 2.7335 | 1.0000 | 2.3336 | 1.0000 | 1.3572 | 1 |
| Caribbean_Sea (2) v. Sierra_Leone (1) | 2577.9083 | 0.3516 | 0.0123 | 0.3516 | NA | NA | 0.6360 | 0.3516 | 0.6736 | 0.3516 | NA | NA | NA | NA | NA | |
| Clarence_River (83) v. Western_North_Atlantic (8) | 72357.7473 | 0.0010 | 0.0198 | 0.0010 | -5.3436 | 1.0000 | 0.6348 | 0.0010 | 0.6800 | 0.0010 | 0.5509 | 0.0010 | 0.6087 | 0.0010 | 0.7477 | 0 |
| Blue_mud_Bay (18) v. Caribbean_Sea (2) | 16396.9017 | 0.0100 | 0.0192 | 0.0100 | 13.7335 | 0.5577 | 0.6321 | 0.0262 | 0.6769 | 0.0262 | 1.7098 | 0.0481 | 1.5626 | 0.0769 | 1.2076 | 0 |
| Clarence_River (83) v. Costa_Rica (16) | 74936.1797 | 0.0010 | 0.0172 | 0.0010 | -5.7062 | 1.0000 | 0.6312 | 0.0010 | 0.6761 | 0.0010 | 0.5381 | 0.0010 | 0.5921 | 0.0010 | 0.7348 | 0 |
| Caribbean_Sea (2) v. Clarence_River (83) | 60776.8282 | 0.0010 | 0.0191 | 0.0010 | -85.2037 | 1.0000 | 0.6295 | 0.0027 | 0.6892 | 0.0043 | 0.9522 | 0.0043 | 0.9602 | 0.0043 | 0.9796 | 0 |
| Caribbean_Sea (2) v. Darwin_Coastal (15) | 14031.3420 | 0.0060 | 0.0190 | 0.0060 | 12.4059 | 0.7853 | 0.6286 | 0.0136 | 0.6871 | 0.0251 | 1.8825 | 0.0368 | 1.6952 | 0.3804 | 1.2412 | 0 |
| Brazil (50) v. Sierra_Leone (1) | 19357.2075 | 0.0220 | 0.0128 | 0.0220 | 3.7736 | 1.0000 | 0.6231 | 1.0000 | 0.6609 | 1.0000 | -7.2591 | 1.0000 | -4.7985 | 1.0000 | 1.9264 | 1 |
| Caribbean_Sea (2) v. Costa_Rica (16) | 4585.3922 | 0.0140 | 0.0023 | 0.0140 | NA | NA | 0.4287 | 0.1186 | 0.4607 | 0.8235 | NA | NA | NA | NA | NA | |
| Costa_Rica (16) v. Western_North_Atlantic (8) | 6703.9713 | 0.0010 | 0.0025 | 0.0010 | -71.9795 | 1.0000 | 0.3964 | 0.0011 | 0.4187 | 0.0011 | 0.7688 | 0.0013 | 0.7844 | 0.0013 | 0.8781 | 0 |
| Costa_Rica (16) v. Gulf_of_Mexico (37) | 12724.5435 | 0.0010 | 0.0025 | 0.0010 | -17.2299 | 1.0000 | 0.3692 | 0.0010 | 0.4010 | 0.0010 | 0.3948 | 0.0010 | 0.4218 | 0.0010 | 0.5855 | 0 |
| Brazil (50) v. Costa_Rica (16) | 14591.9962 | 0.0010 | 0.0024 | 0.0010 | -12.5174 | 1.0000 | 0.3484 | 0.0010 | 0.3709 | 0.0010 | 0.3396 | 0.0010 | 0.3666 | 0.0010 | 0.5272 | 0 |
| Sierra_Leone (1) v. Urauchi_River (35) | 5368.2307 | 0.0280 | 0.0010 | 0.0280 | 3.5735 | 1.0000 | 0.2262 | 1.0000 | 0.2464 | 1.0000 | -1.8664 | 1.0000 | -1.0969 | 1.0000 | 3.4203 | 1 |
| Okinawa (9) v. Sierra_Leone (1) | 2105.6689 | 0.1139 | 0.0008 | 0.1139 | 2.9851 | 1.0000 | 0.2052 | 1.0000 | 0.2182 | 1.0000 | -1.4594 | 1.0000 | -0.6146 | 1.0000 | 4.5143 | 1 |
| Indonesia (4) v. Sierra_Leone (1) | 1287.3999 | 0.2338 | 0.0007 | 0.2338 | NA | NA | 0.1862 | 1.0000 | 0.1985 | 1.0000 | NA | NA | NA | NA | NA | |
| Papua_New_Guinea (9) v. Sierra_Leone (1) | 1927.2893 | 0.1149 | 0.0007 | 0.1149 | 3.0179 | 1.0000 | 0.1688 | 1.0000 | 0.1799 | 1.0000 | -1.4903 | 1.0000 | -0.6634 | 1.0000 | 4.3923 | 1 |
| East_Alligator_River (47) v. Sierra_Leone (1) | 5739.1351 | 0.0140 | 0.0007 | 0.0140 | 3.8286 | 1.0000 | 0.1680 | 1.0000 | 0.1790 | 1.0000 | -2.2140 | 1.0000 | -1.4735 | 1.0000 | 3.0375 | 1 |
| Sierra_Leone (1) v. South_Alligator_River (88) | 8806.2537 | 0.0170 | 0.0007 | 0.0170 | 4.3486 | 1.0000 | 0.1642 | 1.0000 | 0.1789 | 1.0000 | -3.6247 | 1.0000 | -2.7669 | 1.0000 | 2.4351 | 1 |
| Seychelles (36) v. Sierra_Leone (1) | 4675.7140 | 0.0260 | 0.0007 | 0.0260 | 3.7128 | 1.0000 | 0.1641 | 1.0000 | 0.1751 | 1.0000 | -2.0433 | 1.0000 | -1.2968 | 1.0000 | 3.2014 | 1 |
| Sierra_Leone (1) v. Trinity_inlet (5) | 1409.0407 | 0.1628 | 0.0009 | 0.1628 | 2.6294 | 1.0000 | 0.1619 | 1.0000 | 0.1777 | 1.0000 | -1.3772 | 1.0000 | -0.4524 | 1.0000 | 4.8499 | 1 |
| Fiji (25) v. Sierra_Leone (1) | 3717.0341 | 0.0360 | 0.0008 | 0.0779 | 3.5244 | 1.0000 | 0.1610 | 1.0000 | 0.1720 | 1.0000 | -1.8479 | 1.0000 | -1.0793 | 1.0000 | 3.4522 | 1 |
| Adelaide_River (18) v. Sierra_Leone (1) | 2963.9168 | 0.0410 | 0.0007 | 0.0410 | 3.4155 | 1.0000 | 0.1605 | 1.0000 | 0.1713 | 1.0000 | -1.7438 | 1.0000 | -0.9761 | 1.0000 | 3.6569 | 1 |
| Sierra_Leone (1) v. Wenlock_River (24) | 3561.8064 | 0.0470 | 0.0007 | 0.0470 | 3.5641 | 1.0000 | 0.1603 | 0.5465 | 0.1756 | 1.0000 | -1.8914 | 1.0000 | -1.1349 | 1.0000 | 3.3951 | 1 |
| Reunion (28) v. Sierra_Leone (1) | 3958.4759 | 0.0350 | 0.0007 | 0.0350 | 3.6090 | 1.0000 | 0.1589 | 1.0000 | 0.1697 | 1.0000 | -1.9249 | 1.0000 | -1.1728 | 1.0000 | 3.3492 | 1 |
| Arabian_Sea (15) v. Sierra_Leone (1) | 2671.9413 | 0.0729 | 0.0007 | 0.0729 | 3.3559 | 1.0000 | 0.1582 | 1.0000 | 0.1691 | 1.0000 | -1.7251 | 1.0000 | -0.9462 | 1.0000 | 3.6842 | 1 |
| Sierra_Leone (1) v. Sydney_Harbour (65) | 7160.6347 | 0.0200 | 0.0007 | 0.0200 | 4.0946 | 1.0000 | 0.1580 | 1.0000 | 0.1735 | 1.0000 | -2.7843 | 1.0000 | -2.0067 | 1.0000 | 2.6851 | 1 |
| Daly_River (49) v. Sierra_Leone (1) | 5861.0233 | 0.0190 | 0.0007 | 0.0190 | 3.8962 | 1.0000 | 0.1579 | 1.0000 | 0.1686 | 1.0000 | -2.3404 | 1.0000 | -1.5903 | 1.0000 | 2.9324 | 1 |
| Blue_mud_Bay (18) v. Sierra_Leone (1) | 3043.0477 | 0.0579 | 0.0008 | 0.0579 | 3.4601 | 1.0000 | 0.1578 | 1.0000 | 0.1690 | 1.0000 | -1.8161 | 1.0000 | -1.0423 | 1.0000 | 3.5024 | 1 |
| Sierra_Leone (1) v. Towns_River (10) | 2074.2417 | 0.0919 | 0.0007 | 0.0919 | 3.0823 | 1.0000 | 0.1578 | 1.0000 | 0.1750 | 1.0000 | -1.5258 | 1.0000 | -0.7056 | 1.0000 | 4.2441 | 1 |
| Sierra_Leone (1) v. Unknown (10) | 2031.8897 | 0.0899 | 0.0007 | 0.0899 | 3.0918 | 1.0000 | 0.1556 | 1.0000 | 0.1722 | 1.0000 | -1.5357 | 1.0000 | -0.7168 | 1.0000 | 4.2047 | 1 |
| Sierra_Leone (1) v. Sri_Lanka (12) | 2281.1615 | 0.0999 | 0.0007 | 0.0999 | 3.2091 | 1.0000 | 0.1553 | 1.0000 | 0.1691 | 1.0000 | -1.5995 | 1.0000 | -0.8048 | 1.0000 | 4.0102 | 1 |
| Fitzroy_River (3) v. Sierra_Leone (1) | 1083.3937 | 0.2557 | 0.0006 | 0.2557 | NA | NA | 0.1551 | 1.0000 | 0.1657 | 1.0000 | NA | NA | NA | NA | NA | |
| Sierra_Leone (1) v. Thailand (5) | 1375.9116 | 0.1518 | 0.0007 | 0.1518 | 2.6272 | 1.0000 | 0.1549 | 1.0000 | 0.1682 | 1.0000 | -1.3621 | 1.0000 | -0.4434 | 1.0000 | 4.9860 | 1 |

Table 19: Pairwise Fst for 3443 DArTcap loci between the all locations *(continued)*

| | Chi2 | Chi2.p.val | D | D.p.val | Fis | Fis.p.val | Fst | Fst.p.val | F'st | F'st.p.val | Gst | Gst.p.val | G'st | G'st.p.val | G"st | G"st |
|---|---|---|---|---|---|---|---|---|---|---|---|---|---|---|---|---|
| Clarence_River (83) v. Sierra_Leone (1) | 8224.2496 | 0.0130 | 0.0007 | 0.0130 | 4.4698 | 1.0000 | 0.1546 | 1.0000 | 0.1655 | 1.0000 | -4.1486 | 1.0000 | -3.2123 | 1.0000 | 2.3378 | 1 |
| Sierra_Leone (1) v. South_Africa (22) | 3261.7230 | 0.0490 | 0.0007 | 0.0490 | 3.5162 | 1.0000 | 0.1491 | 1.0000 | 0.1629 | 1.0000 | -1.8238 | 1.0000 | -1.0701 | 1.0000 | 3.5128 | 1 |
| Darwin_Coastal (15) v. Sierra_Leone (1) | 2529.3983 | 0.0739 | 0.0007 | 0.0739 | 3.3638 | 1.0000 | 0.1447 | 1.0000 | 0.1550 | 1.0000 | -1.7256 | 1.0000 | -0.9436 | 1.0000 | 3.6787 | 1 |
| Roper_River (9) v. Sierra_Leone (1) | 1871.8274 | 0.0989 | 0.0007 | 0.0989 | 3.0101 | 1.0000 | 0.1441 | 1.0000 | 0.1541 | 1.0000 | -1.4987 | 1.0000 | -0.6637 | 1.0000 | 4.3363 | 1 |
| Mozambique (11) v. Sierra_Leone (1) | 2129.2277 | 0.0939 | 0.0007 | 0.0939 | 3.0960 | 1.0000 | 0.1391 | 1.0000 | 0.1486 | 1.0000 | -1.5408 | 1.0000 | -0.7256 | 1.0000 | 4.1909 | 1 |
| Fiji (25) v. Urauchi_River (35) | 5569.2779 | 0.0010 | 0.0004 | 0.0010 | -9.0775 | 1.0000 | 0.0732 | 0.0010 | 0.0791 | 0.0010 | 0.1153 | 0.0010 | 0.1282 | 0.0010 | 0.2183 | 0 |
| Okinawa (9) v. Urauchi_River (35) | 3040.3882 | 0.0010 | 0.0003 | 0.0010 | -16.0695 | 0.9990 | 0.0568 | 0.0010 | 0.0616 | 0.0010 | 0.2555 | 0.0010 | 0.2754 | 0.0010 | 0.4229 | 0 |
| Fiji (25) v. Okinawa (9) | 2932.9282 | 0.0010 | 0.0003 | 0.0010 | -15.4252 | 0.9930 | 0.0555 | 0.0010 | 0.0596 | 0.0010 | 0.2679 | 0.0010 | 0.2889 | 0.0010 | 0.4392 | 0 |
| Blue_mud_Bay (18) v. Urauchi_River (35) | 4255.5358 | 0.0010 | 0.0003 | 0.0010 | -9.7147 | 0.9960 | 0.0532 | 0.0010 | 0.0576 | 0.0010 | 0.1226 | 0.0010 | 0.1360 | 0.0010 | 0.2303 | 0 |
| Towns_River (10) v. Urauchi_River (35) | 3360.0904 | 0.0010 | 0.0003 | 0.0010 | -13.7006 | 0.8601 | 0.0532 | 0.0010 | 0.0576 | 0.0010 | 0.2149 | 0.0010 | 0.2336 | 0.0010 | 0.3692 | 0 |
| Trinity_inlet (5) v. Urauchi_River (35) | 3094.1430 | 0.0010 | 0.0002 | 0.0030 | -24.2266 | 0.0161 | 0.0511 | 0.0010 | 0.0556 | 0.0010 | 0.4476 | 0.5246 | 0.4710 | 0.4543 | 0.6346 | 0 |
| Thailand (5) v. Urauchi_River (35) | 2925.5157 | 0.0010 | 0.0003 | 0.0030 | -24.7532 | 0.0473 | 0.0505 | 0.0020 | 0.0549 | 0.0020 | 0.4511 | 0.4728 | 0.4742 | 0.4215 | 0.6376 | 0 |
| Papua_New_Guinea (9) v. Urauchi_River (35) | 2922.8611 | 0.0010 | 0.0002 | 0.0010 | -15.0803 | 0.8791 | 0.0478 | 0.0010 | 0.0519 | 0.0010 | 0.2398 | 0.0020 | 0.2593 | 0.0020 | 0.4026 | 0 |
| Roper_River (9) v. Urauchi_River (35) | 3147.8105 | 0.0010 | 0.0002 | 0.0010 | -14.6169 | 0.6523 | 0.0478 | 0.0010 | 0.0518 | 0.0010 | 0.2402 | 0.0020 | 0.2601 | 0.0020 | 0.4034 | 0 |
| Reunion (28) v. Urauchi_River (35) | 4472.7684 | 0.0010 | 0.0002 | 0.0010 | -9.0169 | 1.0000 | 0.0470 | 0.0010 | 0.0508 | 0.0010 | 0.0941 | 0.0010 | 0.1048 | 0.0010 | 0.1818 | 0 |
| Mozambique (11) v. Urauchi_River (35) | 3233.5745 | 0.0010 | 0.0002 | 0.0010 | -13.4797 | 0.9970 | 0.0460 | 0.0010 | 0.0498 | 0.0010 | 0.2086 | 0.0010 | 0.2270 | 0.0010 | 0.3604 | 0 |
| Indonesia (4) v. Okinawa (9) | 1435.8764 | 0.0170 | 0.0002 | 0.0659 | NA | NA | 0.0448 | 0.0382 | 0.0484 | 0.0382 | NA | NA | NA | NA | NA | |
| South_Africa (22) v. Urauchi_River (35) | 3911.9240 | 0.0010 | 0.0002 | 0.0010 | -9.6010 | 1.0000 | 0.0446 | 0.0010 | 0.0482 | 0.0010 | 0.1068 | 0.0010 | 0.1185 | 0.0010 | 0.2036 | 0 |
| Sri_Lanka (12) v. Urauchi_River (35) | 3300.0097 | 0.0010 | 0.0002 | 0.0010 | -12.1349 | 0.7233 | 0.0445 | 0.0010 | 0.0482 | 0.0010 | 0.1722 | 0.0010 | 0.1886 | 0.0010 | 0.3078 | 0 |
| Darwin_Coastal (15) v. Urauchi_River (35) | 3612.6149 | 0.0010 | 0.0002 | 0.0010 | -10.4961 | 0.5185 | 0.0441 | 0.0010 | 0.0478 | 0.0010 | 0.1368 | 0.0010 | 0.1512 | 0.0010 | 0.2533 | 0 |
| Daly_River (49) v. Urauchi_River (35) | 5212.7936 | 0.0010 | 0.0002 | 0.0010 | -7.6065 | 1.0000 | 0.0436 | 0.0010 | 0.0469 | 0.0010 | 0.0682 | 0.0010 | 0.0763 | 0.0010 | 0.1353 | 0 |
| Unknown (10) v. Urauchi_River (35) | 3167.4032 | 0.0010 | 0.0002 | 0.0010 | -13.5824 | 0.5175 | 0.0430 | 0.0010 | 0.0467 | 0.0010 | 0.2082 | 0.0020 | 0.2266 | 0.0020 | 0.3599 | 0 |
| Adelaide_River (18) v. Urauchi_River (35) | 3676.8947 | 0.0010 | 0.0002 | 0.0010 | -10.3731 | 0.9980 | 0.0430 | 0.0010 | 0.0465 | 0.0010 | 0.1231 | 0.0010 | 0.1361 | 0.0010 | 0.2308 | 0 |
| Blue_mud_Bay (18) v. Fiji (25) | 3568.1928 | 0.0010 | 0.0002 | 0.0010 | -9.3354 | 1.0000 | 0.0424 | 0.0010 | 0.0482 | 0.0010 | 0.1297 | 0.0010 | 0.1439 | 0.0010 | 0.2422 | 0 |
| Sydney_Harbour (65) v. Urauchi_River (35) | 5618.9744 | 0.0010 | 0.0002 | 0.0010 | -6.8548 | 1.0000 | 0.0424 | 0.0010 | 0.0456 | 0.0010 | 0.0593 | 0.0010 | 0.0665 | 0.0010 | 0.1188 | 0 |
| Arabian_Sea (15) v. Urauchi_River (35) | 3558.2360 | 0.0010 | 0.0002 | 0.0010 | -10.6566 | 0.7892 | 0.0422 | 0.0010 | 0.0457 | 0.0010 | 0.1373 | 0.0010 | 0.1515 | 0.0010 | 0.2540 | 0 |
| East_Alligator_River (47) v. Urauchi_River (35) | 4841.2665 | 0.0010 | 0.0002 | 0.0010 | -7.9822 | 1.0000 | 0.0405 | 0.0010 | 0.0436 | 0.0010 | 0.0695 | 0.0010 | 0.0776 | 0.0010 | 0.1375 | 0 |
| Clarence_River (83) v. Urauchi_River (35) | 6104.6689 | 0.0010 | 0.0002 | 0.0010 | -5.7870 | 1.0000 | 0.0402 | 0.0010 | 0.0433 | 0.0010 | 0.0503 | 0.0010 | 0.0567 | 0.0010 | 0.1019 | 0 |
| Okinawa (9) v. Trinity_inlet (5) | 1588.4833 | 0.0050 | 0.0002 | 0.0190 | 432.3277 | 0.6034 | 0.0402 | 0.0170 | 0.0433 | 0.0170 | 1.0546 | 0.4296 | 1.0509 | 0.4296 | 1.0248 | 0 |
| Seychelles (36) v. Urauchi_River (35) | 4418.9082 | 0.0010 | 0.0002 | 0.0010 | -8.5682 | 1.0000 | 0.0402 | 0.0010 | 0.0433 | 0.0010 | 0.0793 | 0.0010 | 0.0884 | 0.0010 | 0.1554 | 0 |
| Urauchi_River (35) v. Wenlock_River (24) | 3973.5146 | 0.0010 | 0.0002 | 0.0010 | -9.2619 | 1.0000 | 0.0401 | 0.0010 | 0.0432 | 0.0010 | 0.0980 | 0.0010 | 0.1089 | 0.0010 | 0.1885 | 0 |
| South_Alligator_River (88) v. Urauchi_River (35) | 6136.5114 | 0.0010 | 0.0002 | 0.0010 | -6.0025 | 1.0000 | 0.0400 | 0.0010 | 0.0429 | 0.0010 | 0.0506 | 0.0010 | 0.0568 | 0.0010 | 0.1022 | 0 |
| Okinawa (9) v. Towns_River (10) | 1837.8829 | 0.0010 | 0.0002 | 0.0040 | -31.9259 | 0.9980 | 0.0395 | 0.0030 | 0.0429 | 0.0020 | 0.4923 | 0.0030 | 0.5141 | 0.0030 | 0.6744 | 0 |
| Fiji (25) v. Trinity_inlet (5) | 2589.6902 | 0.0010 | 0.0002 | 0.0010 | -23.3960 | 0.0232 | 0.0382 | 0.0010 | 0.0410 | 0.0010 | 0.4534 | 0.8894 | 0.4774 | 0.8805 | 0.6405 | 0 |
| Fiji (25) v. Towns_River (10) | 2733.0114 | 0.0010 | 0.0002 | 0.0010 | -13.2810 | 0.8402 | 0.0361 | 0.0010 | 0.0390 | 0.0010 | 0.2211 | 0.0110 | 0.2405 | 0.0100 | 0.3780 | 0 |
| Okinawa (9) v. Thailand (5) | 1494.7350 | 0.0180 | 0.0002 | 0.0380 | 269.9769 | 0.6104 | 0.0352 | 0.0360 | 0.0377 | 0.0360 | 1.0915 | 0.4336 | 1.0854 | 0.4346 | 1.0408 | 0 |
| Blue_mud_Bay (18) v. Okinawa (9) | 2145.4923 | 0.0040 | 0.0002 | 0.0030 | -16.6868 | 1.0000 | 0.0345 | 0.0010 | 0.0371 | 0.0010 | 0.2869 | 0.0010 | 0.3083 | 0.0010 | 0.4625 | 0 |
| Fitzroy_River (3) v. Okinawa (9) | 1304.4243 | 0.0340 | 0.0002 | 0.1339 | NA | NA | 0.0342 | 0.1151 | NA | NA | NA | NA | NA | NA | NA | |
| Okinawa (9) v. Papua_New_Guinea (9) | 1634.3227 | 0.0050 | 0.0002 | 0.0100 | -39.8064 | 0.9920 | 0.0337 | 0.0100 | 0.0363 | 0.0100 | 0.5557 | 0.0100 | 0.5759 | 0.0100 | 0.7274 | 0 |
| Mozambique (11) v. Okinawa (9) | 1820.9916 | 0.0020 | 0.0002 | 0.0020 | -30.2588 | 0.9970 | 0.0335 | 0.0020 | 0.0360 | 0.0020 | 0.4771 | 0.0040 | 0.4991 | 0.0040 | 0.6609 | 0 |
| Fiji (25) v. Wenlock_River (24) | 3435.2732 | 0.0010 | 0.0002 | 0.0010 | -8.9297 | 1.0000 | 0.0334 | 0.0010 | 0.0361 | 0.0010 | 0.1074 | 0.0010 | 0.1195 | 0.0010 | 0.2049 | 0 |
| Fiji (25) v. Mozambique (11) | 2767.8705 | 0.0010 | 0.0002 | 0.0010 | -13.0854 | 0.9810 | 0.0333 | 0.0010 | 0.0370 | 0.0010 | 0.2182 | 0.0010 | 0.2374 | 0.0010 | 0.3740 | 0 |
| Fiji (25) v. Reunion (28) | 3650.3859 | 0.0010 | 0.0002 | 0.0010 | -8.6350 | 1.0000 | 0.0331 | 0.0010 | 0.0363 | 0.0010 | 0.0993 | 0.0010 | 0.1106 | 0.0010 | 0.1909 | 0 |
| Fiji (25) v. South_Alligator_River (88) | 5517.0646 | 0.0010 | 0.0002 | 0.0010 | -5.5039 | 0.9780 | 0.0322 | 0.0010 | 0.0350 | 0.0010 | 0.0539 | 0.0010 | 0.0606 | 0.0010 | 0.1086 | 0 |
| Fiji (25) v. Sydney_Harbour (65) | 4910.5200 | 0.0010 | 0.0001 | 0.0010 | -6.2706 | 1.0000 | 0.0321 | 0.0010 | 0.0350 | 0.0010 | 0.0621 | 0.0010 | 0.0697 | 0.0010 | 0.1241 | 0 |
| Adelaide_River (18) v. Fiji (25) | 3161.2909 | 0.0010 | 0.0002 | 0.0010 | -9.8244 | 1.0000 | 0.0319 | 0.0010 | 0.0362 | 0.0010 | 0.1291 | 0.0010 | 0.1428 | 0.0010 | 0.2408 | 0 |
| Okinawa (9) v. Roper_River (9) | 1672.2631 | 0.0060 | 0.0002 | 0.0170 | -38.0541 | 0.9870 | 0.0318 | 0.0150 | 0.0354 | 0.0140 | 0.5499 | 0.0140 | 0.5707 | 0.0140 | 0.7230 | 0 |
| Fiji (25) v. Sri_Lanka (12) | 2847.4077 | 0.0010 | 0.0002 | 0.0010 | -11.4689 | 0.9241 | 0.0318 | 0.0010 | 0.0342 | 0.0010 | 0.1768 | 0.0010 | 0.1938 | 0.0010 | 0.3149 | 0 |
| Okinawa (9) v. Wenlock_River (24) | 2233.4560 | 0.0010 | 0.0002 | 0.0010 | -15.0648 | 1.0000 | 0.0318 | 0.0010 | 0.0345 | 0.0010 | 0.2499 | 0.0010 | 0.2697 | 0.0010 | 0.4158 | 0 |
| Fiji (25) v. Seychelles (36) | 3917.7856 | 0.0010 | 0.0002 | 0.0010 | -8.1055 | 1.0000 | 0.0318 | 0.0010 | 0.0346 | 0.0010 | 0.0861 | 0.0010 | 0.0962 | 0.0010 | 0.1678 | 0 |
| Daly_River (49) v. Fiji (25) | 4361.5812 | 0.0010 | 0.0002 | 0.0010 | -7.0577 | 1.0000 | 0.0317 | 0.0010 | 0.0351 | 0.0010 | 0.0714 | 0.0010 | 0.0800 | 0.0010 | 0.1413 | 0 |
| Darwin_Coastal (15) v. Fiji (25) | 3056.3905 | 0.0010 | 0.0002 | 0.0010 | -10.0134 | 0.9780 | 0.0315 | 0.0010 | 0.0360 | 0.0010 | 0.1424 | 0.0010 | 0.1575 | 0.0010 | 0.2626 | 0 |
| Fiji (25) v. Papua_New_Guinea (9) | 2504.3831 | 0.0010 | 0.0002 | 0.0010 | -14.3600 | 0.7732 | 0.0313 | 0.0010 | 0.0336 | 0.0010 | 0.2433 | 0.0729 | 0.2633 | 0.0709 | 0.4074 | 0 |

Table 19: Pairwise Fst for 3443 DArTcap loci between the all locations *(continued)*

| | Chi2 | Chi2.p.val | D | D.p.val | Fis | Fis.p.val | Fst | Fst.p.val | F'st | F'st.p.val | Gst | Gst.p.val | G'st | G'st.p.val | G"st | G"st |
|---|---|---|---|---|---|---|---|---|---|---|---|---|---|---|---|---|
| Fiji (25) v. South_Africa (22) | 3220.1518 | 0.0010 | 0.0002 | 0.0010 | -9.2524 | 1.0000 | 0.0304 | 0.0010 | 0.0328 | 0.0010 | 0.1125 | 0.0010 | 0.1250 | 0.0010 | 0.2135 | 0 |
| Okinawa (9) v. Reunion (28) | 2342.4528 | 0.0010 | 0.0002 | 0.0010 | -14.5997 | 0.9990 | 0.0302 | 0.0010 | 0.0333 | 0.0010 | 0.2369 | 0.0010 | 0.2562 | 0.0010 | 0.3987 | 0 |
| East_Alligator_River (47) v. Fiji (25) | 4262.6123 | 0.0010 | 0.0001 | 0.0010 | -7.4029 | 1.0000 | 0.0302 | 0.0010 | 0.0334 | 0.0010 | 0.0738 | 0.0010 | 0.0825 | 0.0010 | 0.1455 | 0 |
| Clarence_River (83) v. Fiji (25) | 5278.9751 | 0.0010 | 0.0002 | 0.0010 | -5.1588 | 1.0000 | 0.0296 | 0.0010 | 0.0325 | 0.0010 | 0.0509 | 0.0010 | 0.0575 | 0.0010 | 0.1031 | 0 |
| Okinawa (9) v. Unknown (10) | 1695.2462 | 0.0020 | 0.0002 | 0.0050 | -31.4185 | 0.9960 | 0.0293 | 0.0040 | 0.0318 | 0.0030 | 0.4842 | 0.0050 | 0.5060 | 0.0050 | 0.6672 | 0 |
| Arabian_Sea (15) v. Okinawa (9) | 1897.2786 | 0.0010 | 0.0002 | 0.0010 | -19.2703 | 1.0000 | 0.0290 | 0.0010 | 0.0312 | 0.0010 | 0.3270 | 0.0010 | 0.3489 | 0.0010 | 0.5093 | 0 |
| Fiji (25) v. Unknown (10) | 2586.6470 | 0.0010 | 0.0002 | 0.0010 | -13.0877 | 0.6224 | 0.0287 | 0.0010 | 0.0310 | 0.0010 | 0.2147 | 0.0350 | 0.2338 | 0.0320 | 0.3692 | 0 |
| Adelaide_River (18) v. Okinawa (9) | 1983.6289 | 0.0010 | 0.0001 | 0.0020 | -18.2479 | 0.9990 | 0.0287 | 0.0020 | 0.0308 | 0.0020 | 0.3016 | 0.0020 | 0.3228 | 0.0020 | 0.4797 | 0 |
| Fiji (25) v. Roper_River (9) | 2499.6078 | 0.0010 | 0.0002 | 0.0010 | -14.2578 | 0.5634 | 0.0284 | 0.0010 | 0.0310 | 0.0010 | 0.2464 | 0.1089 | 0.2669 | 0.0959 | 0.4119 | 0 |
| Clarence_River (83) v. Okinawa (9) | 3140.4000 | 0.0010 | 0.0001 | 0.0010 | -7.6006 | 1.0000 | 0.0278 | 0.0010 | 0.0298 | 0.0010 | 0.1247 | 0.0010 | 0.1379 | 0.0010 | 0.2335 | 0 |
| Okinawa (9) v. South_Alligator_River (88) | 3154.2653 | 0.0010 | 0.0001 | 0.0010 | -8.1770 | 1.0000 | 0.0277 | 0.0010 | 0.0301 | 0.0010 | 0.1312 | 0.0010 | 0.1445 | 0.0010 | 0.2437 | 0 |
| Okinawa (9) v. Sri_Lanka (12) | 1785.6135 | 0.0010 | 0.0002 | 0.0020 | -24.4796 | 0.9980 | 0.0277 | 0.0040 | 0.0299 | 0.0040 | 0.4009 | 0.0030 | 0.4232 | 0.0030 | 0.5882 | 0 |
| Darwin_Coastal (15) v. Okinawa (9) | 1908.6859 | 0.0010 | 0.0002 | 0.0010 | -18.8205 | 1.0000 | 0.0277 | 0.0020 | 0.0298 | 0.0020 | 0.3216 | 0.0020 | 0.3437 | 0.0020 | 0.5034 | 0 |
| Arabian_Sea (15) v. Fiji (25) | 2979.1597 | 0.0010 | 0.0001 | 0.0010 | -9.9954 | 0.9920 | 0.0273 | 0.0010 | 0.0311 | 0.0010 | 0.1401 | 0.0010 | 0.1548 | 0.0010 | 0.2587 | 0 |
| East_Alligator_River (47) v. Okinawa (9) | 2583.0886 | 0.0010 | 0.0001 | 0.0010 | -11.9573 | 1.0000 | 0.0270 | 0.0010 | 0.0289 | 0.0010 | 0.1865 | 0.0010 | 0.2032 | 0.0010 | 0.3284 | 0 |
| Daly_River (49) v. Okinawa (9) | 2684.5962 | 0.0010 | 0.0001 | 0.0010 | -11.1712 | 1.0000 | 0.0265 | 0.0010 | 0.0284 | 0.0010 | 0.1763 | 0.0010 | 0.1926 | 0.0010 | 0.3136 | 0 |
| Okinawa (9) v. Sydney_Harbour (65) | 2828.3738 | 0.0010 | 0.0002 | 0.0010 | -9.6236 | 1.0000 | 0.0263 | 0.0010 | 0.0287 | 0.0010 | 0.1523 | 0.0010 | 0.1671 | 0.0010 | 0.2772 | 0 |
| Okinawa (9) v. Seychelles (36) | 2346.3625 | 0.0010 | 0.0001 | 0.0010 | -13.3959 | 0.9990 | 0.0259 | 0.0010 | 0.0283 | 0.0010 | 0.2107 | 0.0010 | 0.2287 | 0.0010 | 0.3630 | 0 |
| Fiji (25) v. Thailand (5) | 2350.1840 | 0.0010 | 0.0002 | 0.0020 | -23.9823 | 0.0165 | 0.0255 | 0.0040 | 0.0273 | 0.0040 | 0.4525 | 0.9428 | 0.4759 | 0.9351 | 0.6392 | 0 |
| Okinawa (9) v. South_Africa (22) | 1993.2978 | 0.0010 | 0.0001 | 0.0020 | -16.1321 | 0.9980 | 0.0241 | 0.0010 | 0.0261 | 0.0010 | 0.2630 | 0.0020 | 0.2831 | 0.0020 | 0.4324 | 0 |
| Indonesia (4) v. Towns_River (10) | 1393.0214 | 0.0430 | 0.0002 | 0.0929 | NA | NA | 0.0233 | 0.0651 | NA | NA | NA | NA | NA | NA | NA | |
| Fitzroy_River (3) v. Indonesia (4) | 974.2003 | 0.3427 | 0.0002 | 0.4116 | NA | NA | 0.0225 | 0.3427 | 0.0243 | 0.3218 | NA | NA | NA | NA | NA | |
| Indonesia (4) v. Thailand (5) | 1117.0939 | 0.1449 | 0.0002 | 0.1968 | NA | NA | 0.0190 | 0.1918 | 0.0204 | 0.1918 | NA | NA | NA | NA | NA | |
| Blue_mud_Bay (18) v. Towns_River (10) | 2008.0221 | 0.0090 | 0.0001 | 0.0050 | -14.4111 | 0.9980 | 0.0187 | 0.0050 | 0.0202 | 0.0050 | 0.2402 | 0.0050 | 0.2604 | 0.0050 | 0.4036 | 0 |
| Fiji (25) v. Fitzroy_River (3) | 2109.6916 | 0.0090 | 0.0002 | 0.0200 | NA | NA | 0.0183 | 0.0248 | 0.0196 | 0.0248 | NA | NA | NA | NA | NA | |
| Papua_New_Guinea (9) v. Trinity_inlet (5) | 1396.6217 | 0.0020 | 0.0001 | 0.0769 | -448.5102 | 0.2630 | 0.0177 | 0.0020 | 0.0191 | 0.0020 | 0.9516 | 0.7090 | 0.9547 | 0.7070 | 0.9768 | 0 |
| Indonesia (4) v. Papua_New_Guinea (9) | 1247.3907 | 0.0909 | 0.0002 | 0.1099 | NA | NA | 0.0171 | 0.1049 | NA | NA | NA | NA | NA | NA | NA | |
| Indonesia (4) v. Trinity_inlet (5) | 1125.2542 | 0.1708 | 0.0002 | 0.1808 | NA | NA | 0.0170 | 0.2008 | NA | NA | NA | NA | NA | NA | NA | |
| Blue_mud_Bay (18) v. Trinity_inlet (5) | 1809.8972 | 0.0290 | 0.0001 | 0.1209 | -26.1771 | 0.3327 | 0.0165 | 0.0509 | 0.0178 | 0.0509 | 0.4839 | 0.4046 | 0.5074 | 0.3846 | 0.6680 | 0 |
| Reunion (28) v. Trinity_inlet (5) | 2038.0213 | 0.0020 | 0.0001 | 0.0539 | -21.6200 | 0.0200 | 0.0159 | 0.0060 | 0.0170 | 0.0060 | 0.4168 | 0.8457 | 0.4404 | 0.8246 | 0.6050 | 0 |
| Blue_mud_Bay (18) v. Reunion (28) | 2571.1409 | 0.0010 | 0.0001 | 0.0010 | -9.3099 | 0.9171 | 0.0151 | 0.0010 | 0.0165 | 0.0010 | 0.1083 | 0.0010 | 0.1204 | 0.0010 | 0.2063 | 0 |
| Indonesia (4) v. Seychelles (36) | 1963.5232 | 0.0120 | 0.0001 | 0.0320 | NA | NA | 0.0147 | 0.0150 | NA | NA | NA | NA | NA | NA | NA | |
| Reunion (28) v. Towns_River (10) | 2132.1053 | 0.0010 | 0.0001 | 0.0010 | -13.0433 | 0.8661 | 0.0140 | 0.0010 | 0.0150 | 0.0010 | 0.1990 | 0.0090 | 0.2167 | 0.0070 | 0.3467 | 0 |
| East_Alligator_River (47) v. Indonesia (4) | 2176.4787 | 0.0040 | 0.0001 | 0.0280 | NA | NA | 0.0135 | 0.0140 | 0.0144 | 0.0140 | NA | NA | NA | NA | NA | |
| Towns_River (10) v. Trinity_inlet (5) | 1426.5350 | 0.0160 | 0.0001 | 0.1688 | -135.4519 | 0.2408 | 0.0134 | 0.0709 | 0.0144 | 0.0709 | 0.8513 | 0.7213 | 0.8604 | 0.7173 | 0.9246 | 0 |
| Blue_mud_Bay (18) v. Darwin_Coastal (15) | 2097.6601 | 0.0020 | 0.0001 | 0.0010 | -10.8646 | 0.9940 | 0.0132 | 0.0010 | 0.0143 | 0.0010 | 0.1556 | 0.0010 | 0.1716 | 0.0010 | 0.2831 | 0 |
| Blue_mud_Bay (18) v. South_Africa (22) | 2309.9438 | 0.0010 | 0.0001 | 0.0020 | -9.8722 | 0.9960 | 0.0129 | 0.0010 | 0.0140 | 0.0010 | 0.1223 | 0.0010 | 0.1355 | 0.0010 | 0.2297 | 0 |
| Sri_Lanka (12) v. Trinity_inlet (5) | 1520.3449 | 0.0030 | 0.0001 | 0.0549 | -55.7394 | 0.2857 | 0.0129 | 0.0050 | 0.0139 | 0.0040 | 0.6880 | 0.6014 | 0.7052 | 0.5814 | 0.8253 | 0 |
| Indonesia (4) v. Wenlock_River (24) | 1690.0306 | 0.1089 | 0.0001 | 0.0779 | NA | NA | 0.0128 | 0.0251 | NA | NA | NA | NA | NA | NA | NA | |
| Indonesia (4) v. Reunion (28) | 1788.1551 | 0.0360 | 0.0001 | 0.0649 | NA | NA | 0.0125 | 0.0504 | NA | NA | NA | NA | NA | NA | NA | |
| Blue_mud_Bay (18) v. Fitzroy_River (3) | 1513.8502 | 0.3317 | 0.0002 | 0.1608 | NA | NA | 0.0124 | 0.1176 | 0.0134 | 0.1176 | NA | NA | NA | NA | NA | |
| Blue_mud_Bay (18) v. East_Alligator_River (47) | 2837.0933 | 0.0010 | 0.0001 | 0.0010 | -8.2119 | 0.0240 | 0.0124 | 0.0010 | 0.0134 | 0.0010 | 0.0822 | 0.0010 | 0.0917 | 0.0010 | 0.1607 | 0 |
| Arabian_Sea (15) v. Indonesia (4) | 1509.7166 | 0.1628 | 0.0001 | 0.0509 | NA | NA | 0.0124 | 0.0381 | 0.0133 | 0.0381 | NA | NA | NA | NA | NA | |
| Blue_mud_Bay (18) v. Seychelles (36) | 2612.4077 | 0.0010 | 0.0001 | 0.0010 | -8.8431 | 0.3766 | 0.0124 | 0.0010 | 0.0135 | 0.0010 | 0.0941 | 0.0010 | 0.1049 | 0.0010 | 0.1819 | 0 |
| Arabian_Sea (15) v. Blue_mud_Bay (18) | 2101.7710 | 0.0080 | 0.0001 | 0.0020 | -10.9137 | 0.9910 | 0.0122 | 0.0090 | 0.0133 | 0.0090 | 0.1557 | 0.0090 | 0.1714 | 0.0090 | 0.2831 | 0 |
| Indonesia (4) v. Unknown (10) | 1306.7498 | 0.0639 | 0.0001 | 0.1359 | NA | NA | 0.0121 | 0.0749 | NA | NA | NA | NA | NA | NA | NA | |
| Blue_mud_Bay (18) v. Mozambique (11) | 1857.3962 | 0.0360 | 0.0001 | 0.0170 | -14.2198 | 0.9990 | 0.0119 | 0.0190 | 0.0133 | 0.0170 | 0.2346 | 0.0020 | 0.2544 | 0.0020 | 0.3961 | 0 |
| Blue_mud_Bay (18) v. South_Alligator_River (88) | 3529.1418 | 0.0010 | 0.0001 | 0.0010 | -6.1041 | 0.0130 | 0.0118 | 0.0010 | 0.0128 | 0.0010 | 0.0563 | 0.0010 | 0.0633 | 0.0010 | 0.1132 | 0 |
| Blue_mud_Bay (18) v. Wenlock_River (24) | 2283.2736 | 0.0010 | 0.0001 | 0.0040 | -9.6120 | 0.9820 | 0.0117 | 0.0010 | 0.0127 | 0.0010 | 0.1152 | 0.0010 | 0.1278 | 0.0010 | 0.2180 | 0 |
| Blue_mud_Bay (18) v. Roper_River (9) | 1832.6707 | 0.0420 | 0.0001 | 0.0150 | -15.2951 | 0.9920 | 0.0116 | 0.0350 | 0.0128 | 0.0350 | 0.2653 | 0.0080 | 0.2864 | 0.0080 | 0.4360 | 0 |
| Blue_mud_Bay (18) v. Clarence_River (83) | 3400.7508 | 0.0010 | 0.0001 | 0.0010 | -5.6968 | 0.7393 | 0.0115 | 0.0010 | 0.0126 | 0.0010 | 0.0536 | 0.0010 | 0.0605 | 0.0010 | 0.1083 | 0 |
| Roper_River (9) v. Towns_River (10) | 1532.6049 | 0.0010 | 0.0001 | 0.0110 | -28.1377 | 0.9800 | 0.0115 | 0.0080 | 0.0125 | 0.0090 | 0.4569 | 0.0120 | 0.4794 | 0.0110 | 0.6427 | 0 |
| Indonesia (4) v. Mozambique (11) | 1304.4937 | 0.1818 | 0.0001 | 0.1069 | NA | NA | 0.0115 | 0.0898 | NA | NA | NA | NA | NA | NA | NA | |

Table 19: Pairwise Fst for 3443 DArTcap loci between the all locations *(continued)*

| | Chi2 | Chi2.p.val | D | D.p.val | Fis | Fis.p.val | Fst | Fst.p.val | F’st | F’st.p.val | Gst | Gst.p.val | G’st | G’st.p.val | G”st | G”st |
|---|---|---|---|---|---|---|---|---|---|---|---|---|---|---|---|---|
| South_Africa (22) v. Trinity_inlet (5) | 1810.5807 | 0.0020 | 0.0001 | 0.1209 | -25.1053 | 0.0280 | 0.0114 | 0.0040 | 0.0122 | 0.0040 | 0.4614 | 0.8450 | 0.4846 | 0.8270 | 0.6473 | 0 |
| Blue_mud_Bay (18) v. Daly_River (49) | 2894.5614 | 0.0010 | 0.0001 | 0.0010 | -7.7813 | 0.2418 | 0.0113 | 0.0010 | 0.0123 | 0.0010 | 0.0774 | 0.0010 | 0.0865 | 0.0010 | 0.1521 | 0 |
| Indonesia (4) v. Roper_River (9) | 1252.4752 | 0.1439 | 0.0002 | 0.1518 | NA | NA | 0.0112 | 0.1329 | 0.0127 | 0.1299 | NA | NA | NA | NA | NA | |
| Arabian_Sea (15) v. Trinity_inlet (5) | 1626.7233 | 0.0040 | 0.0001 | 0.1708 | -33.4915 | 0.2218 | 0.0111 | 0.0080 | 0.0120 | 0.0080 | 0.5542 | 0.5804 | 0.5759 | 0.5584 | 0.7271 | 0 |
| Mozambique (11) v. Papua_New_Guinea (9) | 1470.3666 | 0.0160 | 0.0001 | 0.1189 | -28.1255 | 0.8681 | 0.0111 | 0.0030 | 0.0120 | 0.0030 | 0.4506 | 0.0859 | 0.4728 | 0.0829 | 0.6365 | 0 |
| Blue_mud_Bay (18) v. Papua_New_Guinea (9) | 1730.8418 | 0.0639 | 0.0001 | 0.0400 | -15.8859 | 0.9990 | 0.0109 | 0.0370 | 0.0117 | 0.0370 | 0.2662 | 0.0120 | 0.2868 | 0.0130 | 0.4368 | 0 |
| Blue_mud_Bay (18) v. Thailand (5) | 1674.5510 | 0.0859 | 0.0001 | 0.0280 | -27.7177 | 0.4420 | 0.0109 | 0.1160 | 0.0117 | 0.1160 | 0.4946 | 0.4370 | 0.5175 | 0.4300 | 0.6772 | 0 |
| Indonesia (4) v. Sri_Lanka (12) | 1349.5519 | 0.1868 | 0.0001 | 0.0809 | NA | NA | 0.0108 | 0.0839 | NA | NA | NA | NA | NA | NA | NA | |
| Adelaide_River (18) v. Trinity_inlet (5) | 1700.6529 | 0.0020 | 0.0001 | 0.2308 | -30.6847 | 0.0380 | 0.0108 | 0.0100 | 0.0116 | 0.0100 | 0.5209 | 0.8681 | 0.5431 | 0.8601 | 0.6996 | 0 |
| Blue_mud_Bay (18) v. Sydney_Harbour (65) | 3170.9773 | 0.0010 | 0.0001 | 0.0010 | -6.8309 | 0.0869 | 0.0106 | 0.0010 | 0.0116 | 0.0010 | 0.0649 | 0.0010 | 0.0728 | 0.0010 | 0.1293 | 0 |
| Thailand (5) v. Towns_River (10) | 1415.4509 | 0.0160 | 0.0001 | 0.1099 | -145.7607 | 0.1968 | 0.0106 | 0.1059 | 0.0116 | 0.1059 | 0.8593 | 0.7762 | 0.8678 | 0.7742 | 0.9289 | 0 |
| Adelaide_River (18) v. Towns_River (10) | 1741.0276 | 0.0050 | 0.0001 | 0.0020 | -15.8939 | 0.9730 | 0.0103 | 0.0020 | 0.0111 | 0.0020 | 0.2537 | 0.0010 | 0.2737 | 0.0010 | 0.4206 | 0 |
| South_Alligator_River (88) v. Trinity_inlet (5) | 2945.0540 | 0.0010 | 0.0001 | 0.4036 | -9.6837 | 0.0030 | 0.0102 | 0.0090 | 0.0109 | 0.0090 | 0.2217 | 0.9060 | 0.2411 | 0.8610 | 0.3788 | 0 |
| Mozambique (11) v. Towns_River (10) | 1546.0188 | 0.0140 | 0.0001 | 0.0150 | -24.4358 | 0.9670 | 0.0100 | 0.0170 | 0.0109 | 0.0170 | 0.4042 | 0.0310 | 0.4268 | 0.0310 | 0.5918 | 0 |
| South_Alligator_River (88) v. Towns_River (10) | 2806.4763 | 0.0010 | 0.0001 | 0.0010 | -7.5273 | 0.4925 | 0.0099 | 0.0010 | 0.0106 | 0.0010 | 0.1054 | 0.0020 | 0.1168 | 0.0020 | 0.2010 | 0 |
| Blue_mud_Bay (18) v. Unknown (10) | 1823.9971 | 0.0310 | 0.0001 | 0.0120 | -14.2432 | 0.9840 | 0.0095 | 0.0400 | 0.0103 | 0.0400 | 0.2334 | 0.0250 | 0.2532 | 0.0260 | 0.3945 | 0 |
| Daly_River (49) v. Towns_River (10) | 2310.0903 | 0.0010 | 0.0001 | 0.0030 | -10.1573 | 0.8462 | 0.0095 | 0.0010 | 0.0102 | 0.0010 | 0.1450 | 0.0010 | 0.1595 | 0.0010 | 0.2659 | 0 |
| Papua_New_Guinea (9) v. Unknown (10) | 1404.1215 | 0.0120 | 0.0001 | 0.0080 | -28.7742 | 0.9940 | 0.0095 | 0.0030 | 0.0103 | 0.0020 | 0.4552 | 0.0070 | 0.4773 | 0.0070 | 0.6408 | 0 |
| Adelaide_River (18) v. Blue_mud_Bay (18) | 2068.4350 | 0.0080 | 0.0001 | 0.0190 | -10.7243 | 0.9890 | 0.0093 | 0.0090 | 0.0101 | 0.0090 | 0.1401 | 0.0090 | 0.1545 | 0.0090 | 0.2584 | 0 |
| Papua_New_Guinea (9) v. Towns_River (10) | 1433.7072 | 0.0440 | 0.0001 | 0.0929 | -29.2832 | 0.9540 | 0.0092 | 0.0529 | 0.0100 | 0.0529 | 0.4596 | 0.0519 | 0.4815 | 0.0529 | 0.6447 | 0 |
| Towns_River (10) v. Wenlock_River (24) | 1916.3447 | 0.0020 | 0.0001 | 0.1199 | -13.3737 | 0.9590 | 0.0091 | 0.0060 | 0.0099 | 0.0060 | 0.2069 | 0.0030 | 0.2250 | 0.0030 | 0.3579 | 0 |
| Mozambique (11) v. Wenlock_River (24) | 1929.1416 | 0.0010 | 0.0001 | 0.0020 | -13.1429 | 1.0000 | 0.0090 | 0.0010 | 0.0097 | 0.0010 | 0.2042 | 0.0010 | 0.2222 | 0.0010 | 0.3541 | 0 |
| Sydney_Harbour (65) v. Trinity_inlet (5) | 2520.8663 | 0.0010 | 0.0001 | 0.2008 | -11.9718 | 0.0030 | 0.0089 | 0.0060 | 0.0095 | 0.0060 | 0.2629 | 0.9370 | 0.2840 | 0.9150 | 0.4331 | 0 |
| East_Alligator_River (47) v. Towns_River (10) | 2244.5381 | 0.0010 | 0.0001 | 0.0100 | -10.8554 | 0.4186 | 0.0087 | 0.0010 | 0.0094 | 0.0010 | 0.1533 | 0.0040 | 0.1682 | 0.0020 | 0.2788 | 0 |
| Arabian_Sea (15) v. Towns_River (10) | 1732.2218 | 0.0050 | 0.0001 | 0.0270 | -16.0678 | 0.9910 | 0.0085 | 0.0060 | 0.0093 | 0.0050 | 0.2686 | 0.0020 | 0.2893 | 0.0020 | 0.4398 | 0 |
| Seychelles (36) v. Trinity_inlet (5) | 2158.9166 | 0.0010 | 0.0001 | 0.0849 | -18.3290 | 0.0040 | 0.0084 | 0.0180 | 0.0090 | 0.0180 | 0.3652 | 0.9720 | 0.3883 | 0.9650 | 0.5519 | 0 |
| Clarence_River (83) v. Towns_River (10) | 2567.2184 | 0.0060 | 0.0001 | 0.0020 | -7.1376 | 0.9940 | 0.0084 | 0.0010 | 0.0090 | 0.0010 | 0.1007 | 0.0010 | 0.1119 | 0.0010 | 0.1932 | 0 |
| Seychelles (36) v. Towns_River (10) | 2094.2492 | 0.0010 | 0.0001 | 0.0020 | -11.9077 | 0.7522 | 0.0082 | 0.0030 | 0.0088 | 0.0030 | 0.1729 | 0.0080 | 0.1891 | 0.0050 | 0.3086 | 0 |
| Blue_mud_Bay (18) v. Sri_Lanka (12) | 1869.4295 | 0.0470 | 0.0001 | 0.0190 | -12.6550 | 0.9720 | 0.0082 | 0.0470 | 0.0088 | 0.0470 | 0.1926 | 0.0460 | 0.2104 | 0.0470 | 0.3380 | 0 |
| South_Africa (22) v. Towns_River (10) | 1826.0931 | 0.0020 | 0.0001 | 0.0060 | -14.1714 | 0.9580 | 0.0081 | 0.0030 | 0.0087 | 0.0030 | 0.2203 | 0.0030 | 0.2390 | 0.0030 | 0.3764 | 0 |
| Fitzroy_River (3) v. Thailand (5) | 1023.9241 | 0.1439 | 0.0002 | 0.0380 | NA | NA | 0.0080 | 0.1648 | 0.0086 | 0.1709 | NA | NA | NA | NA | NA | |
| Sydney_Harbour (65) v. Towns_River (10) | 2386.4289 | 0.0010 | 0.0001 | 0.0020 | -8.8242 | 0.7662 | 0.0079 | 0.0010 | 0.0085 | 0.0010 | 0.1237 | 0.0040 | 0.1366 | 0.0030 | 0.2317 | 0 |
| Reunion (28) v. Roper_River (9) | 1942.5524 | 0.0010 | 0.0001 | 0.0010 | -13.6369 | 0.7123 | 0.0077 | 0.0110 | 0.0084 | 0.0100 | 0.2203 | 0.0579 | 0.2392 | 0.0519 | 0.3765 | 0 |
| Thailand (5) v. Trinity_inlet (5) | 1145.6720 | 0.0320 | 0.0001 | 0.1928 | 35.0389 | 0.9291 | 0.0076 | 0.1049 | 0.0083 | 0.1039 | 2.0068 | 0.0859 | 1.9180 | 0.0859 | 1.3053 | 0 |
| Darwin_Coastal (15) v. Indonesia (4) | 1422.5570 | 0.2637 | 0.0002 | 0.0300 | NA | NA | 0.0075 | 0.0969 | 0.0081 | 0.0969 | NA | NA | NA | NA | NA | |
| Darwin_Coastal (15) v. Reunion (28) | 2156.7119 | 0.0010 | 0.0001 | 0.0120 | -10.1157 | 0.0759 | 0.0075 | 0.0010 | 0.0083 | 0.0010 | 0.1239 | 0.0270 | 0.1372 | 0.0150 | 0.2324 | 0 |
| Mozambique (11) v. Trinity_inlet (5) | 1405.6092 | 0.0200 | 0.0001 | 0.3017 | -112.9994 | 0.1235 | 0.0075 | 0.0556 | 0.0081 | 0.0596 | 0.8264 | 0.9837 | 0.8369 | 0.9837 | 0.9107 | 0 |
| Sri_Lanka (12) v. Towns_River (10) | 1545.9168 | 0.0240 | 0.0001 | 0.0290 | -20.3100 | 0.9880 | 0.0075 | 0.0280 | 0.0081 | 0.0280 | 0.3372 | 0.0130 | 0.3592 | 0.0120 | 0.5208 | 0 |
| East_Alligator_River (47) v. Trinity_inlet (5) | 2350.5556 | 0.0010 | 0.0001 | 0.5954 | -15.8290 | 0.0010 | 0.0074 | 0.0220 | 0.0079 | 0.0220 | 0.3249 | 0.9800 | 0.3473 | 0.9780 | 0.5073 | 0 |
| Towns_River (10) v. Unknown (10) | 1479.3415 | 0.0180 | 0.0001 | 0.0110 | -24.5615 | 0.9660 | 0.0071 | 0.0380 | 0.0077 | 0.0380 | 0.4036 | 0.0360 | 0.4262 | 0.0370 | 0.5912 | 0 |
| Trinity_inlet (5) v. Wenlock_River (24) | 1866.8551 | 0.0040 | 0.0001 | 0.4885 | -22.7634 | 0.0180 | 0.0071 | 0.0370 | 0.0077 | 0.0370 | 0.4318 | 0.9131 | 0.4551 | 0.9021 | 0.6195 | 0 |
| Daly_River (49) v. Trinity_inlet (5) | 2304.8616 | 0.0010 | 0.0001 | 0.5225 | -14.6805 | 0.0080 | 0.0070 | 0.0200 | 0.0075 | 0.0200 | 0.3082 | 0.9261 | 0.3306 | 0.9061 | 0.4883 | 0 |
| Reunion (28) v. South_Alligator_River (88) | 2998.5133 | 0.0010 | 0.0000 | 0.0010 | -5.9769 | 0.4116 | 0.0069 | 0.0010 | 0.0075 | 0.0010 | 0.0390 | 0.0010 | 0.0439 | 0.0010 | 0.0798 | 0 |
| Arabian_Sea (15) v. Papua_New_Guinea (9) | 1609.9884 | 0.0430 | 0.0001 | 0.0310 | -17.7297 | 0.9960 | 0.0067 | 0.0070 | 0.0072 | 0.0070 | 0.2995 | 0.0040 | 0.3207 | 0.0040 | 0.4772 | 0 |
| Clarence_River (83) v. Trinity_inlet (5) | 2665.7839 | 0.0040 | 0.0001 | 0.4266 | -9.0782 | 0.1132 | 0.0065 | 0.0381 | 0.0070 | 0.0381 | 0.2099 | 0.5762 | 0.2291 | 0.5311 | 0.3628 | 0 |
| Trinity_inlet (5) v. Unknown (10) | 1318.5721 | 0.0140 | 0.0001 | 0.2118 | -123.0090 | 0.0410 | 0.0059 | 0.0749 | 0.0065 | 0.0750 | 0.8374 | 0.9480 | 0.8473 | 0.9440 | 0.9169 | 0 |
| Papua_New_Guinea (9) v. Reunion (28) | 1803.8110 | 0.0320 | 0.0001 | 0.0130 | -14.0484 | 0.8382 | 0.0058 | 0.0430 | 0.0064 | 0.0430 | 0.2185 | 0.1768 | 0.2368 | 0.1798 | 0.3736 | 0 |
| East_Alligator_River (47) v. Reunion (28) | 2508.8505 | 0.0010 | 0.0000 | 0.0010 | -7.8039 | 0.5375 | 0.0058 | 0.0010 | 0.0062 | 0.0010 | 0.0579 | 0.0010 | 0.0647 | 0.0010 | 0.1159 | 0 |
| Reunion (28) v. Sydney_Harbour (65) | 2703.5379 | 0.0010 | 0.0000 | 0.0010 | -6.6783 | 0.9201 | 0.0058 | 0.0010 | 0.0063 | 0.0010 | 0.0458 | 0.0010 | 0.0514 | 0.0010 | 0.0929 | 0 |
| Arabian_Sea (15) v. Darwin_Coastal (15) | 1855.7191 | 0.0010 | 0.0000 | 0.3477 | -11.8217 | 0.9980 | 0.0057 | 0.0020 | 0.0062 | 0.0020 | 0.1752 | 0.0020 | 0.1921 | 0.0020 | 0.3126 | 0 |
| Mozambique (11) v. South_Alligator_River (88) | 2543.3445 | 0.0080 | 0.0000 | 0.0220 | -7.4473 | 0.8442 | 0.0057 | 0.0010 | 0.0062 | 0.0010 | 0.1019 | 0.0010 | 0.1129 | 0.0010 | 0.1949 | 0 |
| Reunion (28) v. Unknown (10) | 1878.0032 | 0.0030 | 0.0001 | 0.0070 | -12.9993 | 0.4915 | 0.0057 | 0.0280 | 0.0061 | 0.0280 | 0.1940 | 0.1818 | 0.2115 | 0.1718 | 0.3396 | 0 |
| East_Alligator_River (47) v. Mozambique (11) | 2122.9034 | 0.0010 | 0.0000 | 0.0380 | -10.6939 | 0.9491 | 0.0055 | 0.0030 | 0.0060 | 0.0010 | 0.1497 | 0.0010 | 0.1644 | 0.0010 | 0.2732 | 0 |

Table 19: Pairwise Fst for 3443 DArTcap loci between the all locations *(continued)*

| | Chi2 | Chi2.p.val | D | D.p.val | Fis | Fis.p.val | Fst | Fst.p.val | F'st | F'st.p.val | Gst | Gst.p.val | G'st | G'st.p.val | G"st | G"st |
|---|---|---|---|---|---|---|---|---|---|---|---|---|---|---|---|---|
| Fitzroy_River (3) v. Trinity_inlet (5) | 979.7552 | 0.3616 | 0.0002 | 0.2987 | NA | NA | 0.0055 | 0.2348 | 0.0060 | 0.2524 | NA | NA | NA | NA | NA | |
| Clarence_River (83) v. Reunion (28) | 2845.1394 | 0.0010 | 0.0000 | 0.0010 | -5.6161 | 1.0000 | 0.0054 | 0.0010 | 0.0058 | 0.0010 | 0.0367 | 0.0010 | 0.0414 | 0.0010 | 0.0753 | [illegible] |
| Mozambique (11) v. Reunion (28) | 1869.1632 | 0.0150 | 0.0001 | 0.0470 | -12.9534 | 0.9810 | 0.0054 | 0.0250 | 0.0059 | 0.0280 | 0.1939 | 0.0020 | 0.2114 | 0.0020 | 0.3395 | [illegible] |
| Reunion (28) v. Wenlock_River (24) | 2157.5084 | 0.0010 | 0.0000 | 0.0010 | -9.1405 | 0.9860 | 0.0053 | 0.0010 | 0.0057 | 0.0010 | 0.0879 | 0.0030 | 0.0978 | 0.0030 | 0.1706 | [illegible] |
| Mozambique (11) v. Seychelles (36) | 1974.2569 | 0.0010 | 0.0001 | 0.0070 | -11.9120 | 0.9930 | 0.0051 | 0.0050 | 0.0056 | 0.0050 | 0.1714 | 0.0010 | 0.1876 | 0.0010 | 0.3065 | [illegible] |
| Darwin_Coastal (15) v. Mozambique (11) | 1637.7308 | 0.0440 | 0.0001 | 0.0110 | -15.8054 | 1.0000 | 0.0051 | 0.0180 | 0.0057 | 0.0170 | 0.2637 | 0.0010 | 0.2846 | 0.0010 | 0.4339 | [illegible] |
| Darwin_Coastal (15) v. Towns_River (10) | 1651.9702 | 0.0240 | 0.0001 | 0.2438 | -16.0045 | 0.9940 | 0.0050 | 0.0669 | 0.0054 | 0.0659 | 0.2663 | 0.0130 | 0.2872 | 0.0140 | 0.4371 | [illegible] |
| Darwin_Coastal (15) v. Trinity_inlet (5) | 1525.4953 | 0.0769 | 0.0001 | 0.1588 | -32.9632 | 0.3367 | 0.0050 | 0.1179 | 0.0054 | 0.1179 | 0.5483 | 0.5624 | 0.5704 | 0.5465 | 0.7225 | [illegible] |
| Brazil (50) v. Gulf_of_Mexico (37) | 2325.3566 | 0.0010 | 0.0000 | 0.0250 | -8.6882 | 1.0000 | 0.0049 | 0.0010 | 0.0053 | 0.0010 | 0.0536 | 0.0010 | 0.0593 | 0.0010 | 0.1071 | [illegible] |
| Reunion (28) v. Seychelles (36) | 2239.8359 | 0.0010 | 0.0000 | 0.0010 | -8.4873 | 0.9870 | 0.0048 | 0.0010 | 0.0052 | 0.0010 | 0.0685 | 0.0010 | 0.0766 | 0.0010 | 0.1358 | [illegible] |
| Fitzroy_River (3) v. Sri_Lanka (12) | 1298.4252 | 0.3377 | 0.0001 | 0.3936 | NA | NA | 0.0046 | 0.2299 | 0.0050 | 0.2397 | NA | NA | NA | NA | NA | |
| Seychelles (36) v. Thailand (5) | 1919.1018 | 0.0010 | 0.0001 | 0.0579 | -19.2828 | 0.0230 | 0.0046 | 0.1209 | 0.0049 | 0.1209 | 0.3739 | 0.9630 | 0.3967 | 0.9600 | 0.5609 | [illegible] |
| Daly_River (49) v. Mozambique (11) | 2181.4988 | 0.0030 | 0.0000 | 0.0549 | -10.0095 | 0.9910 | 0.0046 | 0.0050 | 0.0050 | 0.0030 | 0.1408 | 0.0010 | 0.1550 | 0.0010 | 0.2593 | [illegible] |
| Adelaide_River (18) v. Reunion (28) | 2014.6971 | 0.0010 | 0.0000 | 0.0050 | -10.1316 | 0.8472 | 0.0046 | 0.0070 | 0.0050 | 0.0070 | 0.1108 | 0.0200 | 0.1226 | 0.0200 | 0.2101 | [illegible] |
| Clarence_River (83) v. Roper_River (9) | 2381.9697 | 0.1499 | 0.0001 | 0.0030 | -7.3091 | 0.9540 | 0.0045 | 0.0170 | 0.0049 | 0.0170 | 0.1125 | 0.0010 | 0.1248 | 0.0010 | 0.2133 | [illegible] |
| Clarence_River (83) v. Mozambique (11) | 2435.0830 | 0.0669 | 0.0001 | 0.0040 | -6.9857 | 1.0000 | 0.0045 | 0.0070 | 0.0049 | 0.0070 | 0.0965 | 0.0010 | 0.1074 | 0.0010 | 0.1859 | [illegible] |
| Papua_New_Guinea (9) v. Thailand (5) | 1272.2324 | 0.0290 | 0.0001 | 0.2647 | -944.1888 | 0.3596 | 0.0044 | 0.1948 | 0.0047 | 0.1948 | 0.9760 | 0.9630 | 0.9775 | 0.9630 | 0.9886 | [illegible] |
| Papua_New_Guinea (9) v. Sri_Lanka (12) | 1411.8786 | 0.2338 | 0.0001 | 0.1928 | -22.9494 | 0.9371 | 0.0042 | 0.0999 | 0.0045 | 0.0989 | 0.3760 | 0.0529 | 0.3980 | 0.0519 | 0.5625 | [illegible] |
| Papua_New_Guinea (9) v. Seychelles (36) | 1790.9361 | 0.0689 | 0.0001 | 0.0160 | -13.0189 | 0.8691 | 0.0042 | 0.0360 | 0.0046 | 0.0360 | 0.1953 | 0.0829 | 0.2125 | 0.0799 | 0.3412 | [illegible] |
| Adelaide_River (18) v. Papua_New_Guinea (9) | 1536.5630 | 0.2018 | 0.0001 | 0.0100 | -17.4849 | 0.9441 | 0.0041 | 0.0589 | 0.0044 | 0.0589 | 0.2816 | 0.0230 | 0.3021 | 0.0260 | 0.4554 | [illegible] |
| Roper_River (9) v. South_Alligator_River (88) | 2529.3531 | 0.0120 | 0.0001 | 0.0040 | -7.7443 | 0.2088 | 0.0041 | 0.0310 | 0.0045 | 0.0310 | 0.1173 | 0.0150 | 0.1297 | 0.0120 | 0.2210 | [illegible] |
| Darwin_Coastal (15) v. Fitzroy_River (3) | 1353.1972 | 0.5704 | 0.0002 | 0.1429 | NA | NA | 0.0041 | 0.2203 | 0.0044 | 0.2203 | NA | NA | NA | NA | NA | |
| Papua_New_Guinea (9) v. Roper_River (9) | 1336.7463 | 0.1469 | 0.0001 | 0.2557 | -33.7010 | 0.8492 | 0.0041 | 0.1439 | 0.0045 | 0.1409 | 0.5104 | 0.1359 | 0.5318 | 0.1359 | 0.6900 | [illegible] |
| Arabian_Sea (15) v. Mozambique (11) | 1666.5157 | 0.0759 | 0.0001 | 0.2867 | -15.7147 | 1.0000 | 0.0041 | 0.0290 | 0.0045 | 0.0270 | 0.2620 | 0.0010 | 0.2825 | 0.0010 | 0.4315 | [illegible] |
| East_Alligator_River (47) v. Fitzroy_River (3) | 1932.3884 | 0.0559 | 0.0001 | 0.3397 | NA | NA | 0.0040 | 0.1998 | 0.0043 | 0.1998 | NA | NA | NA | NA | NA | |
| Adelaide_River (18) v. Darwin_Coastal (15) | 1799.5237 | 0.0110 | 0.0000 | 0.0999 | -11.7234 | 0.5345 | 0.0040 | 0.0120 | 0.0043 | 0.0120 | 0.1607 | 0.0250 | 0.1766 | 0.0220 | 0.2906 | [illegible] |
| Daly_River (49) v. Reunion (28) | 2408.6412 | 0.0010 | 0.0000 | 0.0010 | -7.5142 | 0.9081 | 0.0040 | 0.0010 | 0.0043 | 0.0010 | 0.0544 | 0.0010 | 0.0609 | 0.0010 | 0.1094 | [illegible] |
| Adelaide_River (18) v. Roper_River (9) | 1640.8032 | 0.0310 | 0.0001 | 0.0829 | -16.7916 | 0.8651 | 0.0039 | 0.0769 | 0.0043 | 0.0729 | 0.2794 | 0.0270 | 0.3003 | 0.0230 | 0.4531 | [illegible] |
| Sydney_Harbour (65) v. Wenlock_River (24) | 2459.1597 | 0.0010 | 0.0000 | 0.0010 | -6.8722 | 0.5624 | 0.0039 | 0.0010 | 0.0042 | 0.0010 | 0.0506 | 0.0010 | 0.0568 | 0.0010 | 0.1023 | [illegible] |
| Fitzroy_River (3) v. Papua_New_Guinea (9) | 1145.2011 | 0.2438 | 0.0001 | 0.5994 | NA | NA | 0.0039 | 0.3239 | NA | NA | NA | NA | NA | NA | NA | |
| Clarence_River (83) v. Papua_New_Guinea (9) | 2129.1521 | 0.7203 | 0.0001 | 0.0060 | -7.5970 | 0.9990 | 0.0038 | 0.0310 | 0.0041 | 0.0310 | 0.1121 | 0.0020 | 0.1241 | 0.0020 | 0.2124 | [illegible] |
| Darwin_Coastal (15) v. Thailand (5) | 1533.7705 | 0.0519 | 0.0001 | 0.0799 | -33.2654 | 0.4525 | 0.0038 | 0.1658 | 0.0041 | 0.1658 | 0.5486 | 0.5335 | 0.5704 | 0.5325 | 0.7226 | [illegible] |
| Thailand (5) v. Wenlock_River (24) | 1753.5950 | 0.0230 | 0.0001 | 0.2757 | -23.3907 | 0.0529 | 0.0038 | 0.1319 | 0.0041 | 0.1319 | 0.4350 | 0.9421 | 0.4579 | 0.9391 | 0.6222 | [illegible] |
| Brazil (50) v. Western_North_Atlantic (8) | 1764.9563 | 0.5994 | 0.0000 | 0.3846 | -14.8037 | 0.9937 | 0.0037 | 0.0943 | 0.0040 | 0.0943 | 0.2265 | 0.0053 | 0.2430 | 0.0063 | 0.3828 | [illegible] |
| Roper_River (9) v. Sri_Lanka (12) | 1491.0231 | 0.0380 | 0.0001 | 0.0460 | -22.0378 | 0.9461 | 0.0037 | 0.0689 | 0.0040 | 0.0729 | 0.3726 | 0.0200 | 0.3951 | 0.0180 | 0.5593 | [illegible] |
| East_Alligator_River (47) v. Thailand (5) | 2084.7688 | 0.0010 | 0.0001 | 0.2088 | -16.4398 | 0.0110 | 0.0036 | 0.1470 | 0.0039 | 0.1470 | 0.3295 | 0.9860 | 0.3515 | 0.9850 | 0.5123 | [illegible] |
| East_Alligator_River (47) v. Roper_River (9) | 2050.7647 | 0.0070 | 0.0001 | 0.0959 | -11.3029 | 0.2727 | 0.0036 | 0.0480 | 0.0039 | 0.0480 | 0.1720 | 0.0410 | 0.1881 | 0.0280 | 0.3073 | [illegible] |
| Darwin_Coastal (15) v. East_Alligator_River (47) | 2350.3973 | 0.0010 | 0.0000 | 0.0230 | -8.6418 | 0.0010 | 0.0036 | 0.0040 | 0.0039 | 0.0040 | 0.0920 | 0.4486 | 0.1024 | 0.3516 | 0.1780 | [illegible] |
| Arabian_Sea (15) v. South_Alligator_River (88) | 2963.9767 | 0.0010 | 0.0000 | 0.0140 | -6.3203 | 0.0340 | 0.0034 | 0.0100 | 0.0037 | 0.0100 | 0.0621 | 0.1149 | 0.0695 | 0.0919 | 0.1239 | [illegible] |
| Reunion (28) v. South_Africa (22) | 2046.1101 | 0.0040 | 0.0000 | 0.0020 | -9.3965 | 0.8302 | 0.0033 | 0.0110 | 0.0036 | 0.0110 | 0.0929 | 0.0220 | 0.1032 | 0.0230 | 0.1794 | [illegible] |
| Mozambique (11) v. Roper_River (9) | 1470.9006 | 0.0679 | 0.0001 | 0.0529 | -26.9897 | 0.4066 | 0.0033 | 0.1339 | 0.0037 | 0.1369 | 0.4442 | 0.4925 | 0.4667 | 0.4875 | 0.6308 | [illegible] |
| Mozambique (11) v. Sri_Lanka (12) | 1510.9759 | 0.1199 | 0.0001 | 0.1958 | -19.8424 | 0.9980 | 0.0033 | 0.0869 | 0.0036 | 0.0909 | 0.3304 | 0.0020 | 0.3523 | 0.0020 | 0.5132 | [illegible] |
| Mozambique (11) v. Sydney_Harbour (65) | 2275.9873 | 0.0120 | 0.0000 | 0.0330 | -8.6917 | 0.9810 | 0.0033 | 0.0200 | 0.0036 | 0.0210 | 0.1192 | 0.0010 | 0.1318 | 0.0010 | 0.2243 | [illegible] |
| Roper_River (9) v. Wenlock_River (24) | 1711.3405 | 0.1499 | 0.0001 | 0.1159 | -14.2935 | 0.8621 | 0.0033 | 0.0839 | 0.0036 | 0.0839 | 0.2327 | 0.0340 | 0.2521 | 0.0310 | 0.3933 | [illegible] |
| Reunion (28) v. Sri_Lanka (12) | 1944.4230 | 0.0050 | 0.0000 | 0.1419 | -11.5330 | 0.3806 | 0.0032 | 0.1039 | 0.0034 | 0.1059 | 0.1553 | 0.3856 | 0.1704 | 0.3756 | 0.2819 | [illegible] |
| Daly_River (49) v. Thailand (5) | 2220.9839 | 0.0020 | 0.0001 | 0.0490 | -14.8688 | 0.0470 | 0.0030 | 0.1788 | 0.0032 | 0.1788 | 0.3076 | 0.9409 | 0.3295 | 0.9369 | 0.4872 | [illegible] |
| Reunion (28) v. Thailand (5) | 1867.7590 | 0.0160 | 0.0001 | 0.0659 | -21.9852 | 0.0220 | 0.0030 | 0.2480 | 0.0032 | 0.2490 | 0.4139 | 0.9700 | 0.4368 | 0.9690 | 0.6017 | [illegible] |
| Daly_River (49) v. Unknown (10) | 2011.9401 | 0.0569 | 0.0001 | 0.0020 | -10.2255 | 0.5774 | 0.0029 | 0.0629 | 0.0032 | 0.0629 | 0.1423 | 0.0390 | 0.1566 | 0.0330 | 0.2616 | [illegible] |
| Unknown (10) v. Wenlock_River (24) | 1713.7439 | 0.1299 | 0.0001 | 0.0200 | -13.4525 | 0.7023 | 0.0029 | 0.0859 | 0.0032 | 0.0859 | 0.2045 | 0.0869 | 0.2225 | 0.0819 | 0.3545 | [illegible] |
| East_Alligator_River (47) v. Papua_New_Guinea (9) | 1885.6818 | 0.1578 | 0.0000 | 0.1209 | -11.6441 | 0.6424 | 0.0029 | 0.0909 | 0.0031 | 0.0909 | 0.1706 | 0.1309 | 0.1862 | 0.1249 | 0.3048 | [illegible] |
| Darwin_Coastal (15) v. Unknown (10) | 1578.8748 | 0.0839 | 0.0001 | 0.0290 | -15.8374 | 0.9940 | 0.0029 | 0.1029 | 0.0032 | 0.1019 | 0.2629 | 0.0130 | 0.2838 | 0.0130 | 0.4329 | [illegible] |
| Seychelles (36) v. Wenlock_River (24) | 2182.4773 | 0.0010 | 0.0000 | 0.0050 | -8.5257 | 0.6114 | 0.0029 | 0.0050 | 0.0031 | 0.0050 | 0.0733 | 0.0070 | 0.0818 | 0.0070 | 0.1445 | [illegible] |
| Seychelles (36) v. Unknown (10) | 1848.6789 | 0.0220 | 0.0001 | 0.0280 | -12.0359 | 0.3127 | 0.0029 | 0.0729 | 0.0031 | 0.0729 | 0.1714 | 0.2897 | 0.1875 | 0.2667 | 0.3064 | [illegible] |

Table 19: Pairwise Fst for 3443 DArTcap loci between the all locations *(continued)*

| | Chi2 | Chi2.p.val | D | D.p.val | Fis | Fis.p.val | Fst | Fst.p.val | F'st | F'st.p.val | Gst | Gst.p.val | G'st | G'st.p.val | G"st | G"st |
|---|---|---|---|---|---|---|---|---|---|---|---|---|---|---|---|---|
| Clarence_River (83) v. Seychelles (36) | 2706.1889 | 0.0010 | 0.0000 | 0.0020 | -5.3984 | 1.0000 | 0.0029 | 0.0010 | 0.0031 | 0.0010 | 0.0286 | 0.0010 | 0.0323 | 0.0010 | 0.0592 | 0 |
| Daly_River (49) v. Roper_River (9) | 2049.4392 | 0.0380 | 0.0001 | 0.0140 | -10.6572 | 0.6933 | 0.0028 | 0.0969 | 0.0030 | 0.0959 | 0.1627 | 0.0120 | 0.1784 | 0.0120 | 0.2934 | 0 |
| Darwin_Coastal (15) v. Papua_New_Guinea (9) | 1476.9374 | 0.4565 | 0.0001 | 0.4825 | -17.8863 | 0.9990 | 0.0028 | 0.1499 | 0.0030 | 0.1499 | 0.2995 | 0.0080 | 0.3210 | 0.0100 | 0.4775 | 0 |
| Papua_New_Guinea (9) v. Wenlock_River (24) | 1640.5390 | 0.3477 | 0.0001 | 0.2098 | -14.6334 | 0.9481 | 0.0027 | 0.1139 | 0.0030 | 0.1149 | 0.2307 | 0.0330 | 0.2495 | 0.0350 | 0.3902 | 0 |
| Mozambique (11) v. Unknown (10) | 1475.8046 | 0.0350 | 0.0001 | 0.0340 | -23.7327 | 0.8282 | 0.0027 | 0.1439 | 0.0030 | 0.1489 | 0.3939 | 0.1169 | 0.4164 | 0.1159 | 0.5813 | 0 |
| Daly_River (49) v. Darwin_Coastal (15) | 2314.4070 | 0.0010 | 0.0000 | 0.0739 | -8.3279 | 0.0080 | 0.0027 | 0.0250 | 0.0029 | 0.0250 | 0.0880 | 0.3297 | 0.0981 | 0.2667 | 0.1710 | 0 |
| Seychelles (36) v. Sydney_Harbour (65) | 2529.7314 | 0.0010 | 0.0000 | 0.0010 | -6.5067 | 0.9491 | 0.0027 | 0.0010 | 0.0030 | 0.0010 | 0.0367 | 0.0010 | 0.0412 | 0.0010 | 0.0751 | 0 |
| Thailand (5) v. Unknown (10) | 1296.8852 | 0.0280 | 0.0001 | 0.3027 | -134.9568 | 0.0521 | 0.0027 | 0.2520 | 0.0030 | 0.2490 | 0.8485 | 0.9409 | 0.8576 | 0.9409 | 0.9230 | 0 |
| Roper_River (9) v. Sydney_Harbour (65) | 2216.4061 | 0.0549 | 0.0001 | 0.0110 | -9.0859 | 0.4675 | 0.0027 | 0.0619 | 0.0030 | 0.0639 | 0.1378 | 0.0300 | 0.1519 | 0.0300 | 0.2546 | 0 |
| Arabian_Sea (15) v. Roper_River (9) | 1581.0131 | 0.1199 | 0.0001 | 0.1868 | -17.5233 | 0.9550 | 0.0026 | 0.1518 | 0.0029 | 0.1489 | 0.3012 | 0.0200 | 0.3229 | 0.0190 | 0.4796 | 0 |
| Darwin_Coastal (15) v. Seychelles (36) | 2141.2217 | 0.0010 | 0.0000 | 0.4835 | -9.3549 | 0.0020 | 0.0026 | 0.0400 | 0.0028 | 0.0400 | 0.1051 | 0.5654 | 0.1167 | 0.5165 | 0.2007 | 0 |
| Arabian_Sea (15) v. East_Alligator_River (47) | 2317.3558 | 0.0010 | 0.0000 | 0.4845 | -8.6769 | 0.0170 | 0.0026 | 0.0220 | 0.0028 | 0.0220 | 0.0918 | 0.4456 | 0.1020 | 0.3766 | 0.1774 | 0 |
| Adelaide_River (18) v. Wenlock_River (24) | 1885.7208 | 0.0250 | 0.0000 | 0.0200 | -10.3480 | 0.9051 | 0.0026 | 0.0350 | 0.0028 | 0.0350 | 0.1179 | 0.0390 | 0.1303 | 0.0380 | 0.2220 | 0 |
| Roper_River (9) v. Trinity_inlet (5) | 1319.8432 | 0.0380 | 0.0001 | 0.7762 | -352.3404 | 0.3508 | 0.0026 | 0.2857 | 0.0028 | 0.2897 | 0.9395 | 0.9678 | 0.9434 | 0.9678 | 0.9708 | 0 |
| Darwin_Coastal (15) v. Sydney_Harbour (65) | 2436.6114 | 0.0010 | 0.0000 | 0.2787 | -7.3777 | 0.0020 | 0.0026 | 0.0180 | 0.0028 | 0.0180 | 0.0746 | 0.5445 | 0.0835 | 0.4775 | 0.1471 | 0 |
| Roper_River (9) v. Seychelles (36) | 1888.0352 | 0.0140 | 0.0001 | 0.1119 | -12.5960 | 0.5574 | 0.0026 | 0.1449 | 0.0028 | 0.1469 | 0.1949 | 0.0769 | 0.2124 | 0.0739 | 0.3408 | 0 |
| Adelaide_River (18) v. Thailand (5) | 1531.3836 | 0.0839 | 0.0001 | 0.3177 | -32.3194 | 0.0661 | 0.0026 | 0.2408 | 0.0027 | 0.2448 | 0.5294 | 0.9299 | 0.5509 | 0.9299 | 0.7064 | 0 |
| Papua_New_Guinea (9) v. Sydney_Harbour (65) | 1927.3047 | 0.8122 | 0.0001 | 0.0160 | -9.5687 | 0.9481 | 0.0026 | 0.0849 | 0.0028 | 0.0849 | 0.1395 | 0.0919 | 0.1533 | 0.0969 | 0.2570 | 0 |
| South_Africa (22) v. Unknown (10) | 1621.6366 | 0.2817 | 0.0001 | 0.1409 | -14.1565 | 0.6803 | 0.0026 | 0.1049 | 0.0028 | 0.1049 | 0.2172 | 0.0879 | 0.2357 | 0.0809 | 0.3721 | 0 |
| South_Alligator_River (88) v. Thailand (5) | 2699.5695 | 0.0030 | 0.0001 | 0.1439 | -9.8722 | 0.0100 | 0.0025 | 0.2008 | 0.0026 | 0.2008 | 0.2195 | 0.9940 | 0.2384 | 0.9930 | 0.3755 | 0 |
| Darwin_Coastal (15) v. Sri_Lanka (12) | 1666.1141 | 0.0539 | 0.0000 | 0.3317 | -13.7594 | 0.9660 | 0.0025 | 0.1029 | 0.0027 | 0.1029 | 0.2160 | 0.0529 | 0.2350 | 0.0569 | 0.3709 | 0 |
| Roper_River (9) v. South_Africa (22) | 1695.4656 | 0.0450 | 0.0001 | 0.2008 | -15.0514 | 0.8751 | 0.0025 | 0.1429 | 0.0027 | 0.1459 | 0.2460 | 0.0300 | 0.2658 | 0.0270 | 0.4107 | 0 |
| Papua_New_Guinea (9) v. South_Alligator_River (88) | 2230.0794 | 0.3826 | 0.0000 | 0.0699 | -8.0400 | 0.8332 | 0.0023 | 0.0979 | 0.0025 | 0.0979 | 0.1164 | 0.0689 | 0.1284 | 0.0709 | 0.2193 | 0 |
| Roper_River (9) v. Unknown (10) | 1411.8743 | 0.0340 | 0.0001 | 0.0010 | -27.3583 | 0.8242 | 0.0023 | 0.2128 | 0.0025 | 0.2188 | 0.4461 | 0.1409 | 0.4686 | 0.1409 | 0.6325 | 0 |
| Fitzroy_River (3) v. Roper_River (9) | 1188.4235 | 0.2308 | 0.0002 | 0.3167 | NA | NA | 0.0022 | 0.3477 | 0.0026 | 0.3579 | NA | NA | NA | NA | NA | |
| Darwin_Coastal (15) v. South_Alligator_River (88) | 2811.3993 | 0.0010 | 0.0000 | 0.0440 | -6.3245 | 0.0010 | 0.0022 | 0.0420 | 0.0024 | 0.0420 | 0.0615 | 0.7173 | 0.0690 | 0.6134 | 0.1229 | 0 |
| Seychelles (36) v. South_Alligator_River (88) | 2659.6655 | 0.0010 | 0.0000 | 0.0010 | -5.7037 | 0.4446 | 0.0022 | 0.0010 | 0.0024 | 0.0010 | 0.0293 | 0.0010 | 0.0329 | 0.0010 | 0.0604 | 0 |
| Daly_River (49) v. Sri_Lanka (12) | 2096.2572 | 0.0250 | 0.0000 | 0.1279 | -9.3957 | 0.5435 | 0.0022 | 0.0809 | 0.0023 | 0.0809 | 0.1144 | 0.1698 | 0.1266 | 0.1548 | 0.2163 | 0 |
| Daly_River (49) v. Wenlock_River (24) | 2245.9005 | 0.0010 | 0.0000 | 0.0839 | -7.6746 | 0.5864 | 0.0021 | 0.0090 | 0.0023 | 0.0090 | 0.0595 | 0.0070 | 0.0666 | 0.0070 | 0.1190 | 0 |
| Adelaide_River (18) v. Seychelles (36) | 2019.8115 | 0.0010 | 0.0000 | 0.0260 | -9.5143 | 0.5165 | 0.0021 | 0.0490 | 0.0023 | 0.0490 | 0.0949 | 0.1269 | 0.1054 | 0.1219 | 0.1830 | 0 |
| Darwin_Coastal (15) v. Roper_River (9) | 1556.4537 | 0.1279 | 0.0001 | 0.0360 | -17.3274 | 0.9920 | 0.0021 | 0.1818 | 0.0023 | 0.1788 | 0.2989 | 0.0080 | 0.3209 | 0.0100 | 0.4771 | 0 |
| Adelaide_River (18) v. Unknown (10) | 1516.7211 | 0.4146 | 0.0001 | 0.1069 | -15.8870 | 0.6054 | 0.0021 | 0.1698 | 0.0023 | 0.1678 | 0.2495 | 0.1449 | 0.2693 | 0.1429 | 0.4152 | 0 |
| South_Alligator_River (88) v. Wenlock_River (24) | 2616.8520 | 0.0010 | 0.0000 | 0.0430 | -5.9995 | 0.1049 | 0.0021 | 0.0110 | 0.0022 | 0.0110 | 0.0408 | 0.0340 | 0.0458 | 0.0280 | 0.0832 | 0 |
| Arabian_Sea (15) v. Daly_River (49) | 2363.0448 | 0.0010 | 0.0000 | 0.1908 | -8.2842 | 0.2118 | 0.0021 | 0.0569 | 0.0022 | 0.0569 | 0.0872 | 0.1948 | 0.0971 | 0.1668 | 0.1695 | 0 |
| Clarence_River (83) v. South_Africa (22) | 2487.0747 | 0.0320 | 0.0000 | 0.0100 | -5.8620 | 0.9870 | 0.0020 | 0.0130 | 0.0022 | 0.0130 | 0.0430 | 0.0030 | 0.0484 | 0.0040 | 0.0876 | 0 |
| Papua_New_Guinea (9) v. South_Africa (22) | 1596.4856 | 0.3616 | 0.0001 | 0.0300 | -15.4042 | 0.9540 | 0.0020 | 0.1948 | 0.0022 | 0.1948 | 0.2440 | 0.0559 | 0.2633 | 0.0579 | 0.4078 | 0 |
| Arabian_Sea (15) v. South_Africa (22) | 1883.4848 | 0.0110 | 0.0000 | 0.1698 | -10.8150 | 0.3796 | 0.0020 | 0.0819 | 0.0022 | 0.0819 | 0.1385 | 0.1658 | 0.1526 | 0.1538 | 0.2557 | 0 |
| Arabian_Sea (15) v. Sydney_Harbour (65) | 2612.4950 | 0.0010 | 0.0000 | 0.0480 | -7.2564 | 0.1379 | 0.0019 | 0.0539 | 0.0021 | 0.0529 | 0.0732 | 0.3766 | 0.0818 | 0.3447 | 0.1444 | 0 |
| South_Africa (22) v. Wenlock_River (24) | 1930.3594 | 0.0150 | 0.0000 | 0.0030 | -9.6337 | 0.9151 | 0.0019 | 0.0529 | 0.0021 | 0.0529 | 0.1001 | 0.0529 | 0.1111 | 0.0529 | 0.1919 | 0 |
| Arabian_Sea (15) v. Reunion (28) | 2088.8182 | 0.0010 | 0.0000 | 0.0829 | -9.9971 | 0.1678 | 0.0019 | 0.1309 | 0.0021 | 0.1299 | 0.1199 | 0.4376 | 0.1326 | 0.4216 | 0.2255 | 0 |
| Adelaide_River (18) v. South_Africa (22) | 1788.5555 | 0.1339 | 0.0000 | 0.0330 | -10.8447 | 0.8671 | 0.0019 | 0.1089 | 0.0021 | 0.1089 | 0.1266 | 0.1049 | 0.1396 | 0.1059 | 0.2363 | 0 |
| Adelaide_River (18) v. Mozambique (11) | 1617.9908 | 0.2258 | 0.0001 | 0.3187 | -15.5226 | 0.9970 | 0.0019 | 0.2038 | 0.0021 | 0.1998 | 0.2453 | 0.0010 | 0.2649 | 0.0010 | 0.4097 | 0 |
| Sri_Lanka (12) v. Wenlock_River (24) | 1782.1350 | 0.1189 | 0.0000 | 0.4985 | -11.9012 | 0.5964 | 0.0019 | 0.1379 | 0.0020 | 0.1379 | 0.1650 | 0.2527 | 0.1808 | 0.2468 | 0.2968 | 0 |
| Clarence_River (83) v. Wenlock_River (24) | 2559.1166 | 0.0210 | 0.0000 | 0.0010 | -5.6874 | 0.9810 | 0.0018 | 0.0140 | 0.0020 | 0.0140 | 0.0392 | 0.0020 | 0.0441 | 0.0030 | 0.0802 | 0 |
| Arabian_Sea (15) v. Clarence_River (83) | 2778.3609 | 0.0010 | 0.0000 | 0.3666 | -6.0034 | 0.7872 | 0.0018 | 0.0669 | 0.0020 | 0.0669 | 0.0589 | 0.0619 | 0.0662 | 0.0659 | 0.1182 | 0 |
| South_Alligator_River (88) v. Sydney_Harbour (65) | 2824.9691 | 0.0010 | 0.0000 | 0.0010 | -4.8796 | 0.4725 | 0.0017 | 0.0010 | 0.0018 | 0.0010 | 0.0175 | 0.0010 | 0.0197 | 0.0010 | 0.0366 | 0 |
| Sri_Lanka (12) v. Sydney_Harbour (65) | 2223.8596 | 0.0729 | 0.0000 | 0.0579 | -8.1772 | 0.4206 | 0.0017 | 0.1109 | 0.0019 | 0.1109 | 0.0966 | 0.4046 | 0.1073 | 0.3876 | 0.1859 | 0 |
| East_Alligator_River (47) v. Wenlock_River (24) | 2175.7310 | 0.0040 | 0.0000 | 0.0699 | -8.0475 | 0.0529 | 0.0017 | 0.0350 | 0.0018 | 0.0350 | 0.0627 | 0.0959 | 0.0700 | 0.0819 | 0.1248 | 0 |
| Clarence_River (83) v. Sri_Lanka (12) | 2403.9630 | 0.1399 | 0.0000 | 0.1089 | -6.6491 | 0.9471 | 0.0016 | 0.1299 | 0.0018 | 0.1299 | 0.0775 | 0.0450 | 0.0866 | 0.0500 | 0.1522 | 0 |
| Clarence_River (83) v. Darwin_Coastal (15) | 2579.6377 | 0.0030 | 0.0000 | 0.1658 | -6.0741 | 0.1758 | 0.0016 | 0.0879 | 0.0018 | 0.0879 | 0.0594 | 0.2817 | 0.0668 | 0.2697 | 0.1192 | 0 |
| Adelaide_River (18) v. Arabian_Sea (15) | 1758.1951 | 0.1139 | 0.0000 | 0.5405 | -11.7334 | 0.4486 | 0.0016 | 0.1668 | 0.0017 | 0.1668 | 0.1596 | 0.2448 | 0.1751 | 0.2338 | 0.2887 | 0 |
| Brazil (50) v. Caribbean_Sea (2) | 1530.3572 | 0.8342 | 0.0002 | 0.3127 | 14.5573 | 0.3333 | 0.0016 | 0.8000 | 0.0017 | 0.8000 | 7.5662 | 1.0000 | 6.9471 | 1.0000 | 1.6942 | 1 |
| Daly_River (49) v. Seychelles (36) | 2284.1794 | 0.0020 | 0.0000 | 0.0010 | -7.2509 | 0.9351 | 0.0016 | 0.0210 | 0.0017 | 0.0210 | 0.0442 | 0.0170 | 0.0496 | 0.0170 | 0.0899 | 0 |

Table 19: Pairwise Fst for 3443 DArTcap loci between the all locations *(continued)*

| | Chi2 | Chi2.p.val | D | D.p.val | Fis | Fis.p.val | Fst | Fst.p.val | F'st | F'st.p.val | Gst | Gst.p.val | G'st | G'st.p.val | G"st | G"st |
|---|---|---|---|---|---|---|---|---|---|---|---|---|---|---|---|---|
| South_Alligator_River (88) v. Unknown (10) | 2280.8820 | 0.2557 | 0.0000 | 0.0509 | -7.6226 | 0.1648 | 0.0015 | 0.1838 | 0.0017 | 0.1838 | 0.1021 | 0.2837 | 0.1132 | 0.2557 | 0.1954 | 0 |
| Daly_River (49) v. Fitzroy_River (3) | 1956.9130 | 0.1688 | 0.0001 | 0.5514 | NA | NA | 0.0015 | 0.3833 | 0.0016 | 0.3833 | NA | NA | NA | NA | NA | |
| Adelaide_River (18) v. East_Alligator_River (47) | 2058.7551 | 0.0180 | 0.0000 | 0.1349 | -8.8501 | 0.1588 | 0.0015 | 0.0899 | 0.0016 | 0.0899 | 0.0820 | 0.2897 | 0.0912 | 0.2647 | 0.1600 | 0 |
| South_Africa (22) v. Sydney_Harbour (65) | 2300.0743 | 0.0160 | 0.0000 | 0.0150 | -7.0445 | 0.4625 | 0.0014 | 0.0430 | 0.0016 | 0.0430 | 0.0536 | 0.1059 | 0.0601 | 0.1039 | 0.1080 | 0 |
| Clarence_River (83) v. South_Alligator_River (88) | 3075.1132 | 0.0010 | 0.0000 | 0.0020 | -4.1410 | 0.7872 | 0.0014 | 0.0010 | 0.0015 | 0.0010 | 0.0131 | 0.0010 | 0.0148 | 0.0010 | 0.0275 | 0 |
| Arabian_Sea (15) v. Unknown (10) | 1582.6427 | 0.1898 | 0.0001 | 0.1329 | -15.9197 | 0.8631 | 0.0014 | 0.2468 | 0.0015 | 0.2448 | 0.2630 | 0.0989 | 0.2835 | 0.0989 | 0.4327 | 0 |
| Clarence_River (83) v. Thailand (5) | 2585.1670 | 0.0330 | 0.0001 | 0.4276 | -9.0719 | 0.2478 | 0.0014 | 0.2937 | 0.0015 | 0.2937 | 0.2057 | 0.8012 | 0.2244 | 0.7972 | 0.3568 | 0 |
| Daly_River (49) v. South_Alligator_River (88) | 2618.6747 | 0.0020 | 0.0000 | 0.0100 | -5.3747 | 0.1499 | 0.0013 | 0.0080 | 0.0014 | 0.0080 | 0.0222 | 0.0110 | 0.0251 | 0.0110 | 0.0462 | 0 |
| Fitzroy_River (3) v. South_Alligator_River (88) | 2115.2748 | 0.5614 | 0.0001 | 0.4705 | NA | NA | 0.0012 | 0.3760 | NA | NA | NA | NA | NA | NA | NA | |
| Mozambique (11) v. South_Africa (22) | 1659.7194 | 0.3926 | 0.0000 | 0.6713 | -13.9204 | 0.9970 | 0.0012 | 0.2418 | 0.0013 | 0.2458 | 0.2138 | 0.0010 | 0.2321 | 0.0010 | 0.3674 | 0 |
| Adelaide_River (18) v. Clarence_River (83) | 2285.3918 | 0.4885 | 0.0000 | 0.0070 | -6.2226 | 0.9940 | 0.0012 | 0.1159 | 0.0013 | 0.1159 | 0.0525 | 0.0170 | 0.0590 | 0.0210 | 0.1059 | 0 |
| Clarence_River (83) v. Unknown (10) | 2258.3642 | 0.3906 | 0.0000 | 0.1169 | -7.0606 | 0.9421 | 0.0012 | 0.2188 | 0.0012 | 0.2188 | 0.0958 | 0.0290 | 0.1066 | 0.0330 | 0.1847 | 0 |
| Arabian_Sea (15) v. Wenlock_River (24) | 1896.2451 | 0.0320 | 0.0000 | 0.3606 | -10.3971 | 0.3157 | 0.0011 | 0.2398 | 0.0012 | 0.2378 | 0.1295 | 0.4026 | 0.1430 | 0.3896 | 0.2413 | 0 |
| Adelaide_River (18) v. South_Alligator_River (88) | 2406.1195 | 0.0759 | 0.0000 | 0.3576 | -6.5125 | 0.2697 | 0.0011 | 0.1249 | 0.0012 | 0.1249 | 0.0542 | 0.2737 | 0.0607 | 0.2607 | 0.1090 | 0 |
| Arabian_Sea (15) v. Seychelles (36) | 2112.8080 | 0.0020 | 0.0000 | 0.2308 | -9.4415 | 0.0819 | 0.0011 | 0.2278 | 0.0012 | 0.2278 | 0.1051 | 0.6354 | 0.1166 | 0.6084 | 0.2006 | 0 |
| Clarence_River (83) v. Sydney_Harbour (65) | 2740.9833 | 0.0010 | 0.0000 | 0.0480 | -4.6643 | 1.0000 | 0.0011 | 0.0030 | 0.0012 | 0.0030 | 0.0169 | 0.0010 | 0.0191 | 0.0010 | 0.0354 | 0 |
| Sri_Lanka (12) v. Unknown (10) | 1483.9574 | 0.0979 | 0.0001 | 0.0290 | -19.6900 | 0.7862 | 0.0011 | 0.3207 | 0.0012 | 0.3187 | 0.3272 | 0.2028 | 0.3491 | 0.2048 | 0.5096 | 0 |
| Clarence_River (83) v. Daly_River (49) | 2573.0517 | 0.0270 | 0.0000 | 0.0090 | -5.0794 | 0.9990 | 0.0011 | 0.0140 | 0.0012 | 0.0150 | 0.0214 | 0.0070 | 0.0242 | 0.0070 | 0.0446 | 0 |
| Daly_River (49) v. Sydney_Harbour (65) | 2417.0567 | 0.0230 | 0.0000 | 0.0030 | -5.9562 | 0.7493 | 0.0011 | 0.0230 | 0.0012 | 0.0230 | 0.0274 | 0.0270 | 0.0309 | 0.0260 | 0.0568 | 0 |
| Darwin_Coastal (15) v. South_Africa (22) | 1818.1321 | 0.0509 | 0.0000 | 0.6623 | -10.7661 | 0.0490 | 0.0010 | 0.2458 | 0.0011 | 0.2468 | 0.1376 | 0.5215 | 0.1519 | 0.4805 | 0.2545 | 0 |
| East_Alligator_River (47) v. Unknown (10) | 1939.9793 | 0.0440 | 0.0000 | 0.2158 | -10.8538 | 0.0929 | 0.0010 | 0.2398 | 0.0011 | 0.2398 | 0.1494 | 0.4875 | 0.1640 | 0.4416 | 0.2726 | 0 |
| East_Alligator_River (47) v. South_Africa (22) | 2166.9004 | 0.0020 | 0.0000 | 0.1179 | -8.1874 | 0.0350 | 0.0010 | 0.1479 | 0.0011 | 0.1479 | 0.0668 | 0.3836 | 0.0745 | 0.3626 | 0.1325 | 0 |
| Adelaide_River (18) v. Sri_Lanka (12) | 1604.2655 | 0.3477 | 0.0000 | 0.4745 | -13.8605 | 0.4775 | 0.0010 | 0.3057 | 0.0011 | 0.3057 | 0.2032 | 0.3606 | 0.2210 | 0.3606 | 0.3526 | 0 |
| Clarence_River (83) v. East_Alligator_River (47) | 2548.0510 | 0.0290 | 0.0000 | 0.0020 | -5.2370 | 1.0000 | 0.0010 | 0.0180 | 0.0011 | 0.0180 | 0.0225 | 0.0030 | 0.0254 | 0.0040 | 0.0469 | 0 |
| Adelaide_River (18) v. Daly_River (49) | 2055.9520 | 0.1179 | 0.0000 | 0.0789 | -8.4300 | 0.6474 | 0.0009 | 0.2228 | 0.0009 | 0.2228 | 0.0775 | 0.2118 | 0.0863 | 0.2148 | 0.1520 | 0 |
| East_Alligator_River (47) v. Seychelles (36) | 2180.9182 | 0.0070 | 0.0000 | 0.1029 | -7.5575 | 0.2078 | 0.0008 | 0.1079 | 0.0009 | 0.1079 | 0.0463 | 0.1239 | 0.0519 | 0.1189 | 0.0939 | 0 |
| Darwin_Coastal (15) v. Wenlock_River (24) | 1847.5847 | 0.0789 | 0.0000 | 0.0539 | -10.3647 | 0.0410 | 0.0008 | 0.2807 | 0.0009 | 0.2807 | 0.1291 | 0.6503 | 0.1428 | 0.6194 | 0.2408 | 0 |
| South_Africa (22) v. South_Alligator_River (88) | 2453.2447 | 0.0380 | 0.0000 | 0.1209 | -6.1889 | 0.1149 | 0.0008 | 0.1668 | 0.0009 | 0.1668 | 0.0441 | 0.3666 | 0.0495 | 0.3506 | 0.0897 | 0 |
| Daly_River (49) v. Papua_New_Guinea (9) | 1879.0255 | 0.4426 | 0.0001 | 0.0150 | -10.9855 | 0.9421 | 0.0008 | 0.3267 | 0.0008 | 0.3267 | 0.1610 | 0.0579 | 0.1763 | 0.0639 | 0.2905 | 0 |
| Sydney_Harbour (65) v. Unknown (10) | 2020.8112 | 0.5445 | 0.0000 | 0.0470 | -8.9242 | 0.3716 | 0.0008 | 0.2967 | 0.0008 | 0.2967 | 0.1210 | 0.3347 | 0.1337 | 0.3157 | 0.2273 | 0 |
| Sydney_Harbour (65) v. Thailand (5) | 2291.4812 | 0.0100 | 0.0001 | 0.3696 | -12.1927 | 0.0210 | 0.0006 | 0.3956 | 0.0007 | 0.3956 | 0.2615 | 0.9930 | 0.2821 | 0.9920 | 0.4309 | 0 |
| South_Alligator_River (88) v. Sri_Lanka (12) | 2449.3820 | 0.0639 | 0.0000 | 0.2527 | -7.0459 | 0.1858 | 0.0006 | 0.3267 | 0.0007 | 0.3267 | 0.0806 | 0.6513 | 0.0898 | 0.6214 | 0.1577 | 0 |
| South_Africa (22) v. Sri_Lanka (12) | 1719.7177 | 0.1688 | 0.0000 | 0.4236 | -12.4584 | 0.5305 | 0.0005 | 0.3736 | 0.0006 | 0.3736 | 0.1750 | 0.4306 | 0.1913 | 0.4296 | 0.3118 | 0 |
| Seychelles (36) v. South_Africa (22) | 1996.9972 | 0.0420 | 0.0000 | 0.4086 | -8.8703 | 0.2827 | 0.0005 | 0.3387 | 0.0005 | 0.3387 | 0.0784 | 0.4625 | 0.0874 | 0.4565 | 0.1538 | 0 |
| East_Alligator_River (47) v. Sri_Lanka (12) | 2050.8942 | 0.0280 | 0.0000 | 0.5185 | -9.8506 | 0.1019 | 0.0004 | 0.4016 | 0.0005 | 0.4016 | 0.1190 | 0.8142 | 0.1313 | 0.8002 | 0.2237 | 0 |
| East_Alligator_River (47) v. Sydney_Harbour (65) | 2296.2695 | 0.3347 | 0.0000 | 0.3077 | -6.2028 | 0.9910 | 0.0003 | 0.3017 | 0.0003 | 0.3017 | 0.0287 | 0.2507 | 0.0324 | 0.2567 | 0.0594 | 0 |
| Adelaide_River (18) v. Sydney_Harbour (65) | 2180.1795 | 0.2088 | 0.0000 | 0.7872 | -7.4356 | 0.6014 | 0.0003 | 0.3686 | 0.0003 | 0.3686 | 0.0645 | 0.4785 | 0.0721 | 0.4815 | 0.1283 | 0 |
| Daly_River (49) v. East_Alligator_River (47) | 2217.7409 | 0.1718 | 0.0000 | 0.0789 | -6.7907 | 0.8801 | 0.0002 | 0.3467 | 0.0002 | 0.3467 | 0.0354 | 0.3337 | 0.0398 | 0.3357 | 0.0726 | 0 |
| East_Alligator_River (47) v. South_Alligator_River (88) | 2387.1555 | 0.4336 | 0.0000 | 0.3187 | -5.6194 | 0.8691 | 0.0002 | 0.3017 | 0.0002 | 0.3017 | 0.0232 | 0.2667 | 0.0262 | 0.2757 | 0.0483 | 0 |
| Seychelles (36) v. Sri_Lanka (12) | 1903.3093 | 0.0450 | 0.0000 | 0.1439 | -10.7907 | 0.2647 | 0.0002 | 0.4366 | 0.0002 | 0.4366 | 0.1362 | 0.7532 | 0.1499 | 0.7483 | 0.2518 | 0 |
| Fitzroy_River (3) v. Sydney_Harbour (65) | 2085.3225 | 0.2398 | 0.0001 | 0.5155 | NA | NA | 0.0002 | 0.4706 | NA | NA | NA | NA | NA | NA | NA | |
| Clarence_River (83) v. Fitzroy_River (3) | 2110.5316 | 0.6603 | 0.0001 | 0.1938 | NA | NA | 0.0001 | 0.5064 | 0.0001 | 0.5064 | NA | NA | NA | NA | NA | |
| Gulf_of_Mexico (37) v. Western_North_Atlantic (8) | 1571.2543 | 0.2028 | 0.0000 | 0.5015 | -21.7644 | 0.2311 | -0.0002 | 0.5258 | -0.0002 | 0.5258 | 0.3086 | 0.7866 | 0.3267 | 0.7877 | 0.4855 | 0 |
| Daly_River (49) v. South_Africa (22) | 2121.9984 | 0.0609 | 0.0000 | 0.5634 | -7.9159 | 0.4376 | -0.0003 | 0.6014 | -0.0003 | 0.6014 | 0.0635 | 0.5874 | 0.0710 | 0.5894 | 0.1264 | 0 |
| Roper_River (9) v. Thailand (5) | 1285.4966 | 0.0989 | 0.0001 | 0.1638 | -474.6881 | 0.3828 | -0.0003 | 0.5305 | -0.0003 | 0.5305 | 0.9540 | 0.9689 | 0.9569 | 0.9689 | 0.9780 | 0 |
| Sri_Lanka (12) v. Thailand (5) | 1360.2400 | 0.1798 | 0.0001 | 0.7243 | -60.7430 | 0.1210 | -0.0005 | 0.5774 | -0.0006 | 0.5774 | 0.7015 | 0.8930 | 0.7177 | 0.8930 | 0.8341 | 0 |
| Arabian_Sea (15) v. Fitzroy_River (3) | 1346.0295 | 0.7073 | 0.0001 | 0.3237 | NA | NA | -0.0007 | 0.5527 | -0.0008 | 0.5527 | NA | NA | NA | NA | NA | |
| Fitzroy_River (3) v. South_Africa (22) | 1516.0474 | 0.3716 | 0.0001 | 0.6913 | NA | NA | -0.0009 | 0.5625 | NA | NA | NA | NA | NA | NA | NA | |
| Arabian_Sea (15) v. Sri_Lanka (12) | 1614.4357 | 0.5485 | 0.0000 | 0.5265 | -13.9062 | 0.4665 | -0.0014 | 0.7892 | -0.0015 | 0.7892 | 0.2158 | 0.7333 | 0.2344 | 0.7383 | 0.3703 | 0 |
| South_Africa (22) v. Thailand (5) | 1618.4549 | 0.1039 | 0.0001 | 0.3107 | -25.7692 | 0.0260 | -0.0017 | 0.6883 | -0.0018 | 0.6873 | 0.4611 | 0.9830 | 0.4836 | 0.9820 | 0.6466 | 0 |
| Arabian_Sea (15) v. Thailand (5) | 1441.9783 | 0.4915 | 0.0001 | 0.5814 | -35.0742 | 0.1658 | -0.0019 | 0.7053 | -0.0020 | 0.7043 | 0.5595 | 0.8791 | 0.5805 | 0.8801 | 0.7310 | 0 |
| Caribbean_Sea (2) v. Gulf_of_Mexico (37) | 1323.0750 | 0.5325 | 0.0001 | 0.4585 | 11.3906 | 1.0000 | -0.0020 | 0.6667 | -0.0022 | 1.0000 | -9.8053 | 1.0000 | -8.6016 | 1.0000 | 2.0904 | 1 |
| Fitzroy_River (3) v. Reunion (28) | 1610.4646 | 0.3157 | 0.0001 | 0.7043 | NA | NA | -0.0022 | 0.5819 | NA | NA | NA | NA | NA | NA | NA | |

Table 19: Pairwise Fst for 3443 DArTcap loci between the all locations *(continued)*

| | Chi2 | Chi2.p.val | D | D.p.val | Fis | Fis.p.val | Fst | Fst.p.val | F’st | F’st.p.val | Gst | Gst.p.val | G’st | G’st.p.val | G”st | G”st[illegible] |
|---|---|---|---|---|---|---|---|---|---|---|---|---|---|---|---|---|
| Mozambique (11) v. Thailand (5) | 1270.4968 | 0.5125 | 0.0001 | 0.5764 | -134.4816 | 0.1924 | -0.0027 | 0.7163 | -0.0029 | 0.7133 | 0.8477 | 0.9946 | 0.8568 | 0.9946 | 0.9225 | 0[illegible] |
| Fitzroy_River (3) v. Wenlock_River (24) | 1443.6321 | 0.7393 | 0.0001 | 0.4186 | NA | NA | -0.0034 | 0.7015 | NA | NA | NA | NA | NA | NA | NA | [illegible] |
| Adelaide_River (18) v. Fitzroy_River (3) | 1339.0776 | 0.7542 | 0.0001 | 0.5734 | NA | NA | -0.0045 | 0.7262 | -0.0048 | 0.7262 | NA | NA | NA | NA | NA | [illegible] |
| Caribbean_Sea (2) v. Western_North_Atlantic (8) | 794.9991 | 0.6713 | 0.0001 | 0.4815 | NA | NA | -0.0077 | 1.0000 | -0.0083 | 1.0000 | NA | NA | NA | NA | NA | [illegible] |
| Fitzroy_River (3) v. Mozambique (11) | 1190.3368 | 0.7253 | 0.0001 | 0.9980 | NA | NA | -0.0093 | 0.8500 | NA | NA | NA | NA | NA | NA | NA | [illegible] |
| Adelaide_River (18) v. Indonesia (4) | 1529.8090 | 0.0579 | 0.0001 | 0.0200 | NA | NA | NA | NA | NA | NA | NA | NA | NA | NA | NA | [illegible] |
| Blue_mud_Bay (18) v. Indonesia (4) | 1605.8387 | 0.1548 | 0.0001 | 0.1039 | NA | NA | NA | NA | NA | NA | NA | NA | NA | NA | NA | [illegible] |
| Clarence_River (83) v. Indonesia (4) | 2448.1851 | 0.0969 | 0.0001 | 0.0100 | NA | NA | NA | NA | NA | NA | NA | NA | NA | NA | NA | [illegible] |
| Daly_River (49) v. Indonesia (4) | 2154.7460 | 0.0120 | 0.0001 | 0.0160 | NA | NA | NA | NA | NA | NA | NA | NA | NA | NA | NA | [illegible] |
| Fiji (25) v. Indonesia (4) | 2319.4034 | 0.0010 | 0.0002 | 0.0010 | NA | NA | NA | NA | NA | NA | NA | NA | NA | NA | NA | [illegible] |
| Fitzroy_River (3) v. Gulf_of_Mexico (37) | 28427.3648 | 0.0010 | 0.0204 | 0.0010 | NA | NA | NA | NA | NA | NA | NA | NA | NA | NA | NA | [illegible] |
| Fitzroy_River (3) v. Seychelles (36) | 1759.9167 | 0.1069 | 0.0001 | 0.3027 | NA | NA | NA | NA | NA | NA | NA | NA | NA | NA | NA | [illegible] |
| Fitzroy_River (3) v. Towns_River (10) | 1212.9971 | 0.1738 | 0.0001 | 0.3506 | NA | NA | NA | NA | NA | NA | NA | NA | NA | NA | NA | [illegible] |
| Fitzroy_River (3) v. Unknown (10) | 1163.6953 | 0.2967 | 0.0001 | 0.3666 | NA | NA | NA | NA | NA | NA | NA | NA | NA | NA | NA | [illegible] |
| Fitzroy_River (3) v. Urauchi_River (35) | 2762.4915 | 0.0010 | 0.0003 | 0.0110 | NA | NA | NA | NA | NA | NA | NA | NA | NA | NA | NA | [illegible] |
| Indonesia (4) v. South_Africa (22) | 1619.1846 | 0.0649 | 0.0001 | 0.0430 | NA | NA | NA | NA | NA | NA | NA | NA | NA | NA | NA | [illegible] |
| Indonesia (4) v. South_Alligator_River (88) | 2672.4750 | 0.0050 | 0.0001 | 0.0180 | NA | NA | NA | NA | NA | NA | NA | NA | NA | NA | NA | [illegible] |
| Indonesia (4) v. Sydney_Harbour (65) | 2370.0346 | 0.0040 | 0.0001 | 0.0050 | NA | NA | NA | NA | NA | NA | NA | NA | NA | NA | NA | [illegible] |
| Indonesia (4) v. Urauchi_River (35) | 2861.7966 | 0.0010 | 0.0003 | 0.0010 | NA | NA | NA | NA | NA | NA | NA | NA | NA | NA | NA | [illegible] |

### 8.7.2 Fst per Site

```
strata <- BS.dartcap.gt@schemes$Site
names(strata) <- BS.dartcap.gt@schemes$INDIVIDUALS
strataG::setStrata(BS.dartcap.gt) <- strata

Fst.gt <- strataG::popStructTest(
    BS.dartcap.gt,
    nrep = 1000,
    stats = "all",
    type = "pairwise",
    keep.null = FALSE,
    quietly = TRUE,
    nb.cores =  parallel::detectCores() - 1,
    write.output = FALSE
  )
save(Fst.gt, file = "DArTcap_ALL_FST_Sites.Rdata")
readr::write_tsv(Fst.gt$pairwise$result, path = "DArTcap_ALL_FST_Sites.txt")
```

```
load("DArTcap_ALL_FST_Sites.Rdata")
kableExtra::kbl(Fst.gt$pairwise$result[order(Fst.gt$pairwise$result$Fst,
                                       decreasing = TRUE),
                                  -c(1:4)], digits = 4,
  caption = "Pairwise Fst for 3443 DArTcap loci between the all sites",
  longtable = TRUE, booktabs = TRUE) %>%
  kableExtra::kable_styling(full_width = FALSE,font_size = 5,
                            latex_options = c("repeat_header", "hold_position")) %>%
  kableExtra::landscape()

pop.levels1 <- c("Costa_Rica" , "Brazil","Caribbean_Sea", "Gulf_of_Mexico",
                 "Western_North_Atlantic", "Sierra_Leone", "South_Africa",
                 "Mozambique", "Reunion" , "Seychelles", "Arabian_Sea",
                 "Sri_Lanka", "Thailand", "Indonesia", "Papua_New_Guinea" ,
                 "Australia", "Japan", "Fiji")
m2 <- Fst.gt$pairwise$result[order(match(Fst.gt$pairwise$result$strata.1,
                                         pop.levels1)),c(1,2,11)]
Gdf <- igraph::graph.data.frame(m2, directed = FALSE)
m2.1 <- igraph::as_adjacency_matrix(Gdf, names = TRUE, sparse = FALSE,
                                    attr = "Fst", type = 'lower')

m2 <- Fst.gt$pairwise$result[order(match(Fst.gt$pairwise$result$strata.1,
                                         pop.levels1)),c(1,2,12)]
Gdf <- igraph::graph.data.frame(m2, directed = FALSE)
m2.2 <- igraph::as_adjacency_matrix(Gdf, names = TRUE, sparse = FALSE,
                                    attr = "Fst.p.val", type = 'lower')
m2$Fst.p.adj <- p.adjust(m2$Fst.p.val, "bonferroni")
Gdf <- igraph::graph.data.frame(m2, directed = FALSE)
m2.3 <- igraph::as_adjacency_matrix(Gdf, names = TRUE, sparse = FALSE,
                                    attr = "Fst.p.adj", type = 'lower')

write.csv(m2.1, file = "DArTcap_ALL_FST_Sites.csv")
write.csv(m2.2, file = "DArTcap_ALL_FST_Pval_Sites.csv")
write.csv(m2.3, file = "DArTcap_ALL_FST_Pval_bonferroni_Sites.csv")
```

Table 20: Pairwise Fst for 3443 DArTcap loci between the all sites

| | Chi2 | Chi2.p.val | D | D.p.val | Fis | Fis.p.val | Fst | Fst.p.val | F'st | F'st.p.val | Gst | Gst.p.val | G'st | G'st.p.val | G"st | G"st.p.val |
|---|---|---|---|---|---|---|---|---|---|---|---|---|---|---|---|---|
| Costa Rica (16) v. Indonesia (4) | 16273.1465 | 0.0010 | 0.0174 | 0.0010 | NA | NA | 0.7066 | 0.0016 | 0.7426 | 0.0016 | NA | NA | NA | NA | NA | NA |
| Costa Rica (16) v. Sierra Leone (1) | 11158.8583 | 0.0649 | 0.0114 | 0.0649 | NA | NA | 0.7013 | 1.0000 | 0.7320 | 1.0000 | NA | NA | NA | NA | NA | NA |
| Costa Rica (16) v. Thailand (5) | 17395.3981 | 0.0010 | 0.0171 | 0.0010 | -128.4526 | 1.0000 | 0.6957 | 0.0012 | 0.7321 | 0.0012 | 0.9375 | 0.0013 | 0.9472 | 0.0013 | 0.9728 | 0.0013 |
| Costa Rica (16) v. Papua New Guinea (9) | 20872.6332 | 0.0010 | 0.0172 | 0.0010 | -25.9715 | 1.0000 | 0.6888 | 0.0010 | 0.7293 | 0.0010 | 0.7465 | 0.0010 | 0.7815 | 0.0010 | 0.8749 | 0.0010 |
| Gulf of Mexico (37) v. Indonesia (4) | 30354.5133 | 0.0010 | 0.0206 | 0.0010 | NA | NA | 0.6790 | 0.0018 | 0.7194 | 0.0018 | NA | NA | NA | NA | NA | NA |
| Costa Rica (16) v. Mozambique (11) | 21911.2511 | 0.0010 | 0.0171 | 0.0010 | -21.4465 | 1.0000 | 0.6766 | 0.0010 | 0.7455 | 0.0010 | 0.7102 | 0.0010 | 0.7492 | 0.0010 | 0.8533 | 0.0010 |
| Costa Rica (16) v. Sri Lanka (12) | 23381.6592 | 0.0010 | 0.0171 | 0.0010 | -18.2470 | 1.0000 | 0.6765 | 0.0010 | 0.7186 | 0.0010 | 0.6784 | 0.0010 | 0.7208 | 0.0010 | 0.8336 | 0.0010 |
| Gulf of Mexico (37) v. Papua New Guinea (9) | 37442.1062 | 0.0010 | 0.0202 | 0.0010 | -11.4281 | 1.0000 | 0.6735 | 0.0010 | 0.7158 | 0.0010 | 0.6173 | 0.0010 | 0.6692 | 0.0010 | 0.7955 | 0.0010 |
| Gulf of Mexico (37) v. Thailand (5) | 32159.8344 | 0.0010 | 0.0201 | 0.0010 | -19.0984 | 1.0000 | 0.6734 | 0.0013 | 0.7138 | 0.0013 | 0.7257 | 0.0014 | 0.7666 | 0.0014 | 0.8648 | 0.0014 |
| Arabian Sea (15) v. Costa Rica (16) | 25971.5113 | 0.0010 | 0.0172 | 0.0010 | -14.4992 | 1.0000 | 0.6703 | 0.0010 | 0.7190 | 0.0010 | 0.6412 | 0.0010 | 0.6874 | 0.0010 | 0.8095 | 0.0010 |
| Indonesia (4) v. Western North Atlantic (8) | 10796.1481 | 0.0050 | 0.0201 | 0.0050 | NA | NA | 0.6695 | 0.0057 | 0.7210 | 0.0057 | NA | NA | NA | NA | NA | NA |
| Gulf of Mexico (37) v. Sri Lanka (12) | 40959.6725 | 0.0010 | 0.0201 | 0.0010 | -9.3431 | 1.0000 | 0.6690 | 0.0010 | 0.7122 | 0.0010 | 0.5772 | 0.0010 | 0.6327 | 0.0010 | 0.7671 | 0.0010 |
| Gulf of Mexico (37) v. Japan (44) | 72645.9399 | 0.0010 | 0.0213 | 0.0010 | -6.5832 | 1.0000 | 0.6682 | 0.0010 | 0.7179 | 0.0010 | 0.5329 | 0.0010 | 0.5922 | 0.0010 | 0.7340 | 0.0010 |
| Gulf of Mexico (37) v. Mozambique (11) | 38936.2039 | 0.0010 | 0.0202 | 0.0010 | -10.5682 | 1.0000 | 0.6678 | 0.0010 | 0.7265 | 0.0010 | 0.5984 | 0.0010 | 0.6521 | 0.0010 | 0.7823 | 0.0010 |
| Arabian Sea (15) v. Gulf of Mexico (37) | 44493.1758 | 0.0010 | 0.0203 | 0.0010 | -7.9916 | 1.0000 | 0.6674 | 0.0010 | 0.7306 | 0.0010 | 0.5559 | 0.0010 | 0.6134 | 0.0010 | 0.7515 | 0.0010 |
| Gulf of Mexico (37) v. South Africa (22) | 51302.5655 | 0.0010 | 0.0203 | 0.0010 | -7.0620 | 1.0000 | 0.6645 | 0.0010 | 0.7110 | 0.0010 | 0.5385 | 0.0010 | 0.5969 | 0.0010 | 0.7380 | 0.0010 |
| Fiji (25) v. Gulf of Mexico (37) | 53347.0294 | 0.0010 | 0.0208 | 0.0010 | -7.6557 | 1.0000 | 0.6636 | 0.0010 | 0.7246 | 0.0010 | 0.5421 | 0.0010 | 0.6007 | 0.0010 | 0.7411 | 0.0010 |
| Gulf of Mexico (37) v. Reunion (28) | 56931.1754 | 0.0010 | 0.0205 | 0.0010 | -6.6372 | 1.0000 | 0.6632 | 0.0010 | 0.7172 | 0.0010 | 0.5320 | 0.0010 | 0.5909 | 0.0010 | 0.7330 | 0.0010 |
| Costa Rica (16) v. South Africa (22) | 31374.4245 | 0.0010 | 0.0172 | 0.0010 | -12.3324 | 1.0000 | 0.6623 | 0.0010 | 0.7094 | 0.0010 | 0.6108 | 0.0010 | 0.6594 | 0.0010 | 0.7885 | 0.0010 |
| Brazil (50) v. Indonesia (4) | 34978.7881 | 0.0010 | 0.0201 | 0.0010 | NA | NA | 0.6610 | 0.0016 | 0.7023 | 0.0016 | NA | NA | NA | NA | NA | NA |
| Costa Rica (16) v. Japan (44) | 48791.2085 | 0.0010 | 0.0182 | 0.0010 | -10.8638 | 1.0000 | 0.6609 | 0.0010 | 0.7115 | 0.0010 | 0.5930 | 0.0010 | 0.6435 | 0.0010 | 0.7762 | 0.0010 |
| Gulf of Mexico (37) v. Seychelles (36) | 64246.2659 | 0.0010 | 0.0204 | 0.0010 | -6.2762 | 1.0000 | 0.6609 | 0.0010 | 0.7120 | 0.0010 | 0.5254 | 0.0010 | 0.5846 | 0.0010 | 0.7277 | 0.0010 |
| Papua New Guinea (9) v. Western North Atlantic (8) | 15342.6408 | 0.0010 | 0.0198 | 0.0010 | -29.9304 | 1.0000 | 0.6609 | 0.0010 | 0.7091 | 0.0010 | 0.7937 | 0.0010 | 0.8255 | 0.0010 | 0.9027 | 0.0010 |
| Costa Rica (16) v. Fiji (25) | 32838.2436 | 0.0010 | 0.0177 | 0.0010 | -12.8862 | 1.0000 | 0.6594 | 0.0010 | 0.7427 | 0.0010 | 0.6143 | 0.0010 | 0.6629 | 0.0010 | 0.7912 | 0.0010 |
| Caribbean Sea (2) v. Indonesia (4) | 5575.3583 | 0.0619 | 0.0193 | 0.0619 | NA | NA | 0.6592 | 0.0772 | 0.7108 | 0.1330 | NA | NA | NA | NA | NA | NA |
| Japan (44) v. Western North Atlantic (8) | 44459.5258 | 0.0010 | 0.0209 | 0.0010 | -10.8838 | 1.0000 | 0.6581 | 0.0010 | 0.7026 | 0.0010 | 0.6233 | 0.0010 | 0.6755 | 0.0010 | 0.8001 | 0.0010 |
| Brazil (50) v. Japan (44) | 82414.0333 | 0.0010 | 0.0208 | 0.0010 | -5.2352 | 1.0000 | 0.6580 | 0.0010 | 0.7067 | 0.0010 | 0.5108 | 0.0010 | 0.5711 | 0.0010 | 0.7161 | 0.0010 |
| Thailand (5) v. Western North Atlantic (8) | 11762.3727 | 0.0030 | 0.0196 | 0.0030 | -1708.8139 | 1.0000 | 0.6580 | 0.0030 | 0.7092 | 0.0030 | 0.9956 | 0.0030 | 0.9963 | 0.0030 | 0.9982 | 0.0030 |
| Costa Rica (16) v. Reunion (28) | 35822.7035 | 0.0010 | 0.0173 | 0.0010 | -11.2181 | 1.0000 | 0.6579 | 0.0010 | 0.7153 | 0.0010 | 0.5972 | 0.0010 | 0.6469 | 0.0010 | 0.7790 | 0.0010 |
| Brazil (50) v. Papua New Guinea (9) | 43914.5156 | 0.0010 | 0.0197 | 0.0010 | -8.8114 | 1.0000 | 0.6576 | 0.0010 | 0.7002 | 0.0010 | 0.5789 | 0.0010 | 0.6338 | 0.0010 | 0.7681 | 0.0010 |
| Brazil (50) v. Thailand (5) | 37344.9627 | 0.0010 | 0.0195 | 0.0010 | -12.8491 | 1.0000 | 0.6564 | 0.0012 | 0.6977 | 0.0012 | 0.6624 | 0.0013 | 0.7103 | 0.0013 | 0.8257 | 0.0013 |
| Brazil (50) v. Sri Lanka (12) | 48090.5646 | 0.0010 | 0.0196 | 0.0010 | -7.4193 | 1.0000 | 0.6543 | 0.0010 | 0.6976 | 0.0010 | 0.5455 | 0.0010 | 0.6030 | 0.0010 | 0.7431 | 0.0010 |
| Arabian Sea (15) v. Brazil (50) | 52205.7665 | 0.0010 | 0.0198 | 0.0010 | -6.3824 | 1.0000 | 0.6538 | 0.0010 | 0.7111 | 0.0010 | 0.5278 | 0.0010 | 0.5868 | 0.0010 | 0.7296 | 0.0010 |
| Caribbean Sea (2) v. Japan (44) | 35935.8757 | 0.0030 | 0.0201 | 0.0030 | 17.3479 | 0.6953 | 0.6537 | 0.0110 | 0.7112 | 0.0204 | 1.4705 | 0.6562 | 1.3773 | 0.6953 | 1.1527 | 0.6953 |
| Costa Rica (16) v. Seychelles (36) | 41929.4198 | 0.0010 | 0.0172 | 0.0010 | -10.2066 | 1.0000 | 0.6532 | 0.0010 | 0.7059 | 0.0010 | 0.5844 | 0.0010 | 0.6350 | 0.0010 | 0.7696 | 0.0010 |
| Brazil (50) v. Mozambique (11) | 45890.2124 | 0.0010 | 0.0197 | 0.0010 | -8.0143 | 1.0000 | 0.6532 | 0.0010 | 0.7086 | 0.0010 | 0.5607 | 0.0010 | 0.6171 | 0.0010 | 0.7546 | 0.0010 |
| Sri Lanka (12) v. Western North Atlantic (8) | 17932.6616 | 0.0010 | 0.0197 | 0.0010 | -19.3031 | 1.0000 | 0.6531 | 0.0010 | 0.7009 | 0.0010 | 0.7186 | 0.0010 | 0.7601 | 0.0010 | 0.8604 | 0.0010 |
| Brazil (50) v. South Africa (22) | 59708.1807 | 0.0010 | 0.0198 | 0.0010 | -5.6168 | 1.0000 | 0.6522 | 0.0010 | 0.6981 | 0.0010 | 0.5134 | 0.0010 | 0.5730 | 0.0010 | 0.7178 | 0.0010 |
| Brazil (50) v. Fiji (25) | 62085.9734 | 0.0010 | 0.0203 | 0.0010 | -6.1466 | 1.0000 | 0.6521 | 0.0010 | 0.7183 | 0.0010 | 0.5169 | 0.0010 | 0.5767 | 0.0010 | 0.7209 | 0.0010 |
| Brazil (50) v. Reunion (28) | 65838.2138 | 0.0010 | 0.0199 | 0.0010 | -5.1468 | 1.0000 | 0.6518 | 0.0010 | 0.7038 | 0.0010 | 0.5076 | 0.0010 | 0.5676 | 0.0010 | 0.7132 | 0.0010 |
| Mozambique (11) v. Western North Atlantic (8) | 16446.4730 | 0.0010 | 0.0198 | 0.0010 | -23.8312 | 1.0000 | 0.6505 | 0.0010 | 0.6993 | 0.0010 | 0.7559 | 0.0010 | 0.7928 | 0.0010 | 0.8820 | 0.0010 |
| Brazil (50) v. Seychelles (36) | 73511.3689 | 0.0010 | 0.0198 | 0.0010 | -4.9091 | 1.0000 | 0.6504 | 0.0010 | 0.7001 | 0.0010 | 0.5026 | 0.0010 | 0.5628 | 0.0010 | 0.7090 | 0.0010 |
| Caribbean Sea (2) v. Papua New Guinea (9) | 9592.1001 | 0.0140 | 0.0191 | 0.0140 | 8.7392 | 1.0000 | 0.6504 | 0.0216 | 0.7048 | 0.0308 | 3.1407 | 0.4835 | 2.6339 | 0.4835 | 1.3946 | 0.4835 |
| Arabian Sea (15) v. Western North Atlantic (8) | 20524.2457 | 0.0010 | 0.0199 | 0.0010 | -14.8171 | 1.0000 | 0.6495 | 0.0010 | 0.6971 | 0.0010 | 0.6748 | 0.0010 | 0.7215 | 0.0010 | 0.8337 | 0.0010 |
| South Africa (22) v. Western North Atlantic (8) | 26233.0639 | 0.0010 | 0.0199 | 0.0010 | -12.2867 | 1.0000 | 0.6488 | 0.0010 | 0.6951 | 0.0010 | 0.6416 | 0.0010 | 0.6916 | 0.0010 | 0.8121 | 0.0010 |
| Reunion (28) v. Western North Atlantic (8) | 30966.6961 | 0.0010 | 0.0200 | 0.0010 | -11.2130 | 1.0000 | 0.6485 | 0.0010 | 0.6943 | 0.0010 | 0.6274 | 0.0010 | 0.6788 | 0.0010 | 0.8026 | 0.0010 |
| Fiji (25) v. Western North Atlantic (8) | 27619.8196 | 0.0010 | 0.0203 | 0.0010 | -13.2118 | 1.0000 | 0.6477 | 0.0010 | 0.6944 | 0.0010 | 0.6488 | 0.0010 | 0.6984 | 0.0010 | 0.8171 | 0.0010 |
| Seychelles (36) v. Western North Atlantic (8) | 37388.1295 | 0.0010 | 0.0199 | 0.0010 | -10.1738 | 1.0000 | 0.6475 | 0.0010 | 0.6927 | 0.0010 | 0.6128 | 0.0010 | 0.6654 | 0.0010 | 0.7925 | 0.0010 |
| Gulf of Mexico (37) v. Sierra Leone (1) | 18859.1589 | 0.0240 | 0.0130 | 0.0240 | 3.5388 | 1.0000 | 0.6452 | 1.0000 | 0.6819 | 1.0000 | -4.0811 | 1.0000 | -2.4174 | 1.0000 | 2.1091 | 1.0000 |
| Australia (444) v. Gulf of Mexico (37) | 374538.5986 | 0.0010 | 0.0203 | 0.0010 | -0.5217 | 1.0000 | 0.6416 | 0.0010 | 0.6878 | 0.0010 | 0.4921 | 0.0010 | 0.5531 | 0.0010 | 0.7005 | 0.0010 |
| Caribbean Sea (2) v. Seychelles (36) | 29492.5761 | 0.0020 | 0.0191 | 0.0020 | 18.4682 | 0.8525 | 0.6409 | 0.0046 | 0.7027 | 0.0087 | 1.4205 | 0.1982 | 1.3387 | 0.4240 | 1.1399 | 0.4608 |
| Caribbean Sea (2) v. Sri Lanka (12) | 11881.2980 | 0.0140 | 0.0189 | 0.0140 | 10.4803 | 1.0000 | 0.6407 | 0.0192 | 0.6951 | 0.0223 | 2.3070 | 0.6608 | 2.0162 | 0.7517 | 1.3073 | 0.7517 |
| Caribbean Sea (2) v. Reunion (28) | 23726.1873 | 0.0030 | 0.0191 | 0.0030 | 16.1387 | 0.8138 | 0.6401 | 0.0079 | 0.7109 | 0.0150 | 1.5290 | 0.2340 | 1.4236 | 0.5957 | 1.1675 | 0.7234 |

Table 20: Pairwise Fst for 3443 DArTcap loci between the all sites *(continued)*

| | Chi2 | Chi2.p.val | D | D.p.val | Fis | Fis.p.val | Fst | Fst.p.val | F'st | F'st.p.val | Gst | Gst.p.val | G'st | G'st.p.val | G"st | G"st.p.val |
|---|---|---|---|---|---|---|---|---|---|---|---|---|---|---|---|---|
| Sierra Leone (1) v. Western North Atlantic (8) | 6383.0393 | 0.1079 | 0.0128 | 0.1079 | NA | NA | 0.6399 | 1.0000 | 0.6928 | 1.0000 | NA | NA | NA | NA | NA | NA |
| Caribbean Sea (2) v. Thailand (5) | 6365.8728 | 0.0460 | 0.0190 | 0.0460 | 6.5399 | 1.0000 | 0.6397 | 0.0605 | 0.6900 | 0.0891 | 7.7722 | 0.1077 | 5.9532 | 0.1077 | 1.5646 | 0.4356 |
| Caribbean Sea (2) v. South Africa (22) | 19348.6504 | 0.0040 | 0.0191 | 0.0040 | 14.6472 | 0.8108 | 0.6396 | 0.0086 | 0.6969 | 0.0130 | 1.6357 | 0.3668 | 1.5064 | 0.7104 | 1.1921 | 0.7336 |
| Caribbean Sea (2) v. Fiji (25) | 20234.6055 | 0.0030 | 0.0196 | 0.0030 | 13.8263 | 0.5143 | 0.6389 | 0.0341 | 0.7519 | 0.0857 | 1.7211 | 0.5143 | 1.5712 | 0.5143 | 1.2099 | 0.5143 |
| Australia (444) v. Western North Atlantic (8) | 306261.9566 | 0.0010 | 0.0198 | 0.0010 | -0.8403 | 1.0000 | 0.6385 | 0.0010 | 0.6824 | 0.0010 | 0.5002 | 0.0010 | 0.5609 | 0.0010 | 0.7073 | 0.0010 |
| Arabian Sea (15) v. Caribbean Sea (2) | 14193.4109 | 0.0090 | 0.0192 | 0.0090 | 12.1323 | 0.6987 | 0.6376 | 0.0166 | 0.6820 | 0.0166 | 1.9032 | 0.1026 | 1.7105 | 0.3046 | 1.2447 | 0.4437 |
| Caribbean Sea (2) v. Mozambique (11) | 10554.8552 | 0.0110 | 0.0190 | 0.0110 | 9.3888 | 1.0000 | 0.6367 | 0.0445 | 0.7509 | 0.3056 | 2.7335 | 1.0000 | 2.3336 | 1.0000 | 1.3572 | 1.0000 |
| Caribbean Sea (2) v. Sierra Leone (1) | 2577.9083 | 0.3417 | 0.0123 | 0.3417 | NA | NA | 0.6360 | 0.3417 | 0.6736 | 0.3417 | NA | NA | NA | NA | NA | NA |
| Australia (444) v. Brazil (50) | 388501.9802 | 0.0010 | 0.0197 | 0.0010 | -0.3701 | 1.0000 | 0.6359 | 0.0010 | 0.6813 | 0.0010 | 0.4782 | 0.0010 | 0.5395 | 0.0010 | 0.6885 | 0.0010 |
| Australia (444) v. Caribbean Sea (2) | 253047.0846 | 0.0010 | 0.0191 | 0.0010 | -1.7172 | 1.0000 | 0.6353 | 0.0040 | 0.6787 | 0.0040 | 0.5730 | 0.0042 | 0.6287 | 0.0042 | 0.7639 | 0.0042 |
| Australia (444) v. Costa Rica (16) | 299205.7214 | 0.0010 | 0.0172 | 0.0010 | -1.1032 | 1.0000 | 0.6264 | 0.0010 | 0.6694 | 0.0010 | 0.5104 | 0.0010 | 0.5657 | 0.0010 | 0.7124 | 0.0010 |
| Brazil (50) v. Sierra Leone (1) | 19357.2075 | 0.0210 | 0.0128 | 0.0210 | 3.7736 | 1.0000 | 0.6231 | 1.0000 | 0.6609 | 1.0000 | -7.2591 | 1.0000 | -4.7985 | 1.0000 | 1.9264 | 1.0000 |
| Caribbean Sea (2) v. Costa Rica (16) | 4585.3922 | 0.0120 | 0.0023 | 0.0120 | NA | NA | 0.4287 | 0.0845 | 0.4607 | 0.7059 | NA | NA | NA | NA | NA | NA |
| Costa Rica (16) v. Western North Atlantic (8) | 6703.9713 | 0.0010 | 0.0025 | 0.0010 | -71.9795 | 1.0000 | 0.3964 | 0.0011 | 0.4187 | 0.0011 | 0.7688 | 0.0012 | 0.7844 | 0.0012 | 0.8781 | 0.0012 |
| Costa Rica (16) v. Gulf of Mexico (37) | 12724.5435 | 0.0010 | 0.0025 | 0.0010 | -17.2299 | 1.0000 | 0.3692 | 0.0010 | 0.4010 | 0.0010 | 0.3948 | 0.0010 | 0.4218 | 0.0010 | 0.5855 | 0.0010 |
| Brazil (50) v. Costa Rica (16) | 14591.9962 | 0.0010 | 0.0024 | 0.0010 | -12.5174 | 1.0000 | 0.3484 | 0.0010 | 0.3709 | 0.0010 | 0.3396 | 0.0010 | 0.3666 | 0.0010 | 0.5272 | 0.0010 |
| Japan (44) v. Sierra Leone (1) | 6184.4210 | 0.0190 | 0.0009 | 0.0190 | 3.6975 | 1.0000 | 0.2067 | 1.0000 | 0.2202 | 1.0000 | -2.0230 | 1.0000 | -1.2649 | 1.0000 | 3.2141 | 1.0000 |
| Indonesia (4) v. Sierra Leone (1) | 1287.3999 | 0.1898 | 0.0007 | 0.1898 | NA | NA | 0.1862 | 1.0000 | 0.1985 | 1.0000 | NA | NA | NA | NA | NA | NA |
| Papua New Guinea (9) v. Sierra Leone (1) | 1927.2893 | 0.1109 | 0.0007 | 0.1109 | 3.0179 | 1.0000 | 0.1688 | 1.0000 | 0.1799 | 1.0000 | -1.4903 | 1.0000 | -0.6634 | 1.0000 | 4.3923 | 1.0000 |
| Seychelles (36) v. Sierra Leone (1) | 4675.7140 | 0.0190 | 0.0007 | 0.0190 | 3.7128 | 1.0000 | 0.1641 | 1.0000 | 0.1751 | 1.0000 | -2.0433 | 1.0000 | -1.2968 | 1.0000 | 3.2014 | 1.0000 |
| Fiji (25) v. Sierra Leone (1) | 3717.0341 | 0.0400 | 0.0008 | 0.0789 | 3.5244 | 1.0000 | 0.1610 | 1.0000 | 0.1720 | 1.0000 | -1.8479 | 1.0000 | -1.0793 | 1.0000 | 3.4522 | 1.0000 |
| Reunion (28) v. Sierra Leone (1) | 3958.4759 | 0.0290 | 0.0007 | 0.0290 | 3.6090 | 1.0000 | 0.1589 | 1.0000 | 0.1697 | 1.0000 | -1.9249 | 1.0000 | -1.1728 | 1.0000 | 3.3492 | 1.0000 |
| Arabian Sea (15) v. Sierra Leone (1) | 2671.9413 | 0.0519 | 0.0007 | 0.0519 | 3.3559 | 1.0000 | 0.1582 | 1.0000 | 0.1691 | 1.0000 | -1.7251 | 1.0000 | -0.9462 | 1.0000 | 3.6842 | 1.0000 |
| Australia (444) v. Sierra Leone (1) | 27946.7116 | 0.0020 | 0.0007 | 0.0020 | -16.0712 | 1.0000 | 0.1572 | 1.0000 | 0.1679 | 1.0000 | 0.7680 | 1.0000 | 0.7836 | 1.0000 | 0.8776 | 1.0000 |
| Sierra Leone (1) v. Sri Lanka (12) | 2281.1615 | 0.0789 | 0.0007 | 0.0789 | 3.2091 | 1.0000 | 0.1553 | 1.0000 | 0.1691 | 1.0000 | -1.5995 | 1.0000 | -0.8048 | 1.0000 | 4.0102 | 1.0000 |
| Sierra Leone (1) v. Thailand (5) | 1375.9116 | 0.1708 | 0.0007 | 0.1708 | 2.6272 | 1.0000 | 0.1549 | 1.0000 | 0.1682 | 1.0000 | -1.3621 | 1.0000 | -0.4434 | 1.0000 | 4.9860 | 1.0000 |
| Sierra Leone (1) v. South Africa (22) | 3261.7230 | 0.0450 | 0.0007 | 0.0450 | 3.5162 | 1.0000 | 0.1491 | 1.0000 | 0.1629 | 1.0000 | -1.8238 | 1.0000 | -1.0701 | 1.0000 | 3.5128 | 1.0000 |
| Mozambique (11) v. Sierra Leone (1) | 2129.2277 | 0.0879 | 0.0007 | 0.0879 | 3.0960 | 1.0000 | 0.1391 | 1.0000 | 0.1486 | 1.0000 | -1.5408 | 1.0000 | -0.7256 | 1.0000 | 4.1909 | 1.0000 |
| Fiji (25) v. Japan (44) | 5695.6380 | 0.0010 | 0.0003 | 0.0010 | -8.2308 | 1.0000 | 0.0617 | 0.0010 | 0.0668 | 0.0010 | 0.0972 | 0.0010 | 0.1084 | 0.0010 | 0.1873 | 0.0010 |
| Japan (44) v. Thailand (5) | 2959.4339 | 0.0010 | 0.0002 | 0.0030 | -19.8728 | 0.0260 | 0.0369 | 0.0040 | 0.0394 | 0.0040 | 0.3877 | 0.4770 | 0.4109 | 0.4300 | 0.5755 | 0.4420 |
| Japan (44) v. Reunion (28) | 4298.1505 | 0.0010 | 0.0002 | 0.0010 | -8.2811 | 1.0000 | 0.0352 | 0.0010 | 0.0381 | 0.0010 | 0.0771 | 0.0010 | 0.0861 | 0.0010 | 0.1515 | 0.0010 |
| Japan (44) v. Papua New Guinea (9) | 2849.1844 | 0.0010 | 0.0002 | 0.0010 | -13.2332 | 0.8062 | 0.0350 | 0.0010 | 0.0375 | 0.0010 | 0.2059 | 0.0030 | 0.2239 | 0.0020 | 0.3564 | 0.0030 |
| Japan (44) v. Mozambique (11) | 3221.7881 | 0.0010 | 0.0002 | 0.0010 | -11.8248 | 0.9930 | 0.0345 | 0.0010 | 0.0376 | 0.0010 | 0.1777 | 0.0010 | 0.1944 | 0.0010 | 0.3160 | 0.0010 |
| Fiji (25) v. Mozambique (11) | 2767.8705 | 0.0010 | 0.0002 | 0.0010 | -13.0854 | 0.9810 | 0.0333 | 0.0010 | 0.0370 | 0.0010 | 0.2182 | 0.0020 | 0.2374 | 0.0020 | 0.3740 | 0.0020 |
| Fiji (25) v. Reunion (28) | 3650.3859 | 0.0010 | 0.0002 | 0.0010 | -8.6350 | 1.0000 | 0.0331 | 0.0010 | 0.0363 | 0.0010 | 0.0993 | 0.0010 | 0.1106 | 0.0010 | 0.1909 | 0.0010 |
| Fiji (25) v. Sri Lanka (12) | 2847.4077 | 0.0010 | 0.0002 | 0.0010 | -11.4689 | 0.9331 | 0.0318 | 0.0010 | 0.0342 | 0.0010 | 0.1768 | 0.0010 | 0.1938 | 0.0010 | 0.3149 | 0.0010 |
| Japan (44) v. South Africa (22) | 3710.1260 | 0.0010 | 0.0002 | 0.0010 | -8.7590 | 0.9970 | 0.0318 | 0.0010 | 0.0342 | 0.0010 | 0.0877 | 0.0010 | 0.0977 | 0.0010 | 0.1705 | 0.0010 |
| Fiji (25) v. Seychelles (36) | 3917.7856 | 0.0010 | 0.0002 | 0.0010 | -8.1055 | 1.0000 | 0.0318 | 0.0010 | 0.0346 | 0.0010 | 0.0861 | 0.0010 | 0.0962 | 0.0010 | 0.1678 | 0.0010 |
| Japan (44) v. Sri Lanka (12) | 3216.3450 | 0.0010 | 0.0002 | 0.0010 | -10.8310 | 0.4715 | 0.0318 | 0.0010 | 0.0340 | 0.0010 | 0.1458 | 0.0010 | 0.1605 | 0.0010 | 0.2674 | 0.0010 |
| Fiji (25) v. Papua New Guinea (9) | 2504.3831 | 0.0010 | 0.0002 | 0.0010 | -14.3600 | 0.7592 | 0.0313 | 0.0010 | 0.0336 | 0.0010 | 0.2433 | 0.0969 | 0.2633 | 0.0899 | 0.4074 | 0.0919 |
| Australia (444) v. Fiji (25) | 9451.6632 | 0.0010 | 0.0002 | 0.0010 | -1.4442 | 0.0410 | 0.0310 | 0.0010 | 0.0334 | 0.0010 | 0.0244 | 0.0010 | 0.0277 | 0.0010 | 0.0508 | 0.0010 |
| Arabian Sea (15) v. Japan (44) | 3464.5033 | 0.0010 | 0.0001 | 0.0010 | -9.6648 | 0.4216 | 0.0306 | 0.0010 | 0.0332 | 0.0010 | 0.1160 | 0.0010 | 0.1285 | 0.0010 | 0.2191 | 0.0010 |
| Fiji (25) v. South Africa (22) | 3220.1518 | 0.0010 | 0.0002 | 0.0010 | -9.2524 | 1.0000 | 0.0304 | 0.0010 | 0.0328 | 0.0010 | 0.1125 | 0.0010 | 0.1250 | 0.0010 | 0.2135 | 0.0010 |
| Australia (444) v. Japan (44) | 9334.2816 | 0.0010 | 0.0002 | 0.0010 | -1.7096 | 1.0000 | 0.0287 | 0.0010 | 0.0307 | 0.0010 | 0.0204 | 0.0010 | 0.0231 | 0.0010 | 0.0427 | 0.0010 |
| Japan (44) v. Seychelles (36) | 4153.7717 | 0.0010 | 0.0001 | 0.0010 | -7.9267 | 1.0000 | 0.0287 | 0.0010 | 0.0310 | 0.0010 | 0.0637 | 0.0010 | 0.0713 | 0.0010 | 0.1269 | 0.0010 |
| Arabian Sea (15) v. Fiji (25) | 2979.1597 | 0.0010 | 0.0001 | 0.0010 | -9.9954 | 0.9830 | 0.0273 | 0.0010 | 0.0311 | 0.0010 | 0.1401 | 0.0010 | 0.1548 | 0.0010 | 0.2587 | 0.0010 |
| Fiji (25) v. Thailand (5) | 2350.1840 | 0.0010 | 0.0002 | 0.0020 | -23.9823 | 0.0222 | 0.0255 | 0.0072 | 0.0273 | 0.0072 | 0.4525 | 0.9344 | 0.4759 | 0.9288 | 0.6392 | 0.9288 |
| Indonesia (4) v. Thailand (5) | 1117.0939 | 0.1229 | 0.0002 | 0.1958 | NA | NA | 0.0190 | 0.1798 | 0.0204 | 0.1798 | NA | NA | NA | NA | NA | NA |
| Indonesia (4) v. Papua New Guinea (9) | 1247.3907 | 0.0819 | 0.0002 | 0.1059 | NA | NA | 0.0171 | 0.1032 | NA | NA | NA | NA | NA | NA | NA | NA |
| Indonesia (4) v. Seychelles (36) | 1963.5232 | 0.0070 | 0.0001 | 0.0320 | NA | NA | 0.0147 | 0.0100 | NA | NA | NA | NA | NA | NA | NA | NA |
| Indonesia (4) v. Reunion (28) | 1788.1551 | 0.0270 | 0.0001 | 0.0759 | NA | NA | 0.0125 | 0.0556 | NA | NA | NA | NA | NA | NA | NA | NA |
| Arabian Sea (15) v. Indonesia (4) | 1509.7166 | 0.1508 | 0.0001 | 0.0320 | NA | NA | 0.0124 | 0.0241 | 0.0133 | 0.0241 | NA | NA | NA | NA | NA | NA |
| Indonesia (4) v. Mozambique (11) | 1304.4937 | 0.1918 | 0.0001 | 0.1499 | NA | NA | 0.0115 | 0.1110 | NA | NA | NA | NA | NA | NA | NA | NA |

Table 20: Pairwise Fst for 3443 DArTcap loci between the all sites *(continued)*

| | Chi2 | Chi2.p.val | D | D.p.val | Fis | Fis.p.val | Fst | Fst.p.val | F'st | F'st.p.val | Gst | Gst.p.val | G'st | G'st.p.val | G"st | G"st.p.val |
|---|---|---|---|---|---|---|---|---|---|---|---|---|---|---|---|---|
| Mozambique (11) v. Papua New Guinea (9) | 1470.3666 | 0.0100 | 0.0001 | 0.1289 | -28.1255 | 0.8661 | 0.0111 | 0.0030 | 0.0120 | 0.0030 | 0.4506 | 0.0849 | 0.4728 | 0.0819 | 0.6365 | 0.0819 |
| Indonesia (4) v. Sri Lanka (12) | 1349.5519 | 0.1608 | 0.0001 | 0.0909 | NA | NA | 0.0108 | 0.0829 | NA | NA | NA | NA | NA | NA | NA | NA |
| Arabian Sea (15) v. Papua New Guinea (9) | 1609.9884 | 0.0529 | 0.0001 | 0.0220 | -17.7297 | 0.9990 | 0.0067 | 0.0060 | 0.0072 | 0.0060 | 0.2995 | 0.0010 | 0.3207 | 0.0010 | 0.4772 | 0.0010 |
| Papua New Guinea (9) v. Reunion (28) | 1803.8110 | 0.0240 | 0.0001 | 0.0100 | -14.0484 | 0.8282 | 0.0058 | 0.0260 | 0.0064 | 0.0260 | 0.2185 | 0.1838 | 0.2368 | 0.1858 | 0.3736 | 0.1858 |
| Mozambique (11) v. Reunion (28) | 1869.1632 | 0.0150 | 0.0001 | 0.0360 | -12.9534 | 0.9880 | 0.0054 | 0.0280 | 0.0059 | 0.0290 | 0.1939 | 0.0040 | 0.2114 | 0.0040 | 0.3395 | 0.0040 |
| Australia (444) v. Reunion (28) | 4038.8765 | 0.0010 | 0.0000 | 0.0010 | -1.7006 | 0.4815 | 0.0052 | 0.0010 | 0.0056 | 0.0010 | 0.0112 | 0.0010 | 0.0127 | 0.0010 | 0.0236 | 0.0010 |
| Mozambique (11) v. Seychelles (36) | 1974.2569 | 0.0010 | 0.0001 | 0.0100 | -11.9120 | 0.9870 | 0.0051 | 0.0040 | 0.0056 | 0.0040 | 0.1714 | 0.0010 | 0.1876 | 0.0010 | 0.3065 | 0.0010 |
| Brazil (50) v. Gulf of Mexico (37) | 2325.3566 | 0.0010 | 0.0000 | 0.0190 | -8.6882 | 1.0000 | 0.0049 | 0.0010 | 0.0053 | 0.0010 | 0.0536 | 0.0010 | 0.0593 | 0.0010 | 0.1071 | 0.0010 |
| Reunion (28) v. Seychelles (36) | 2239.8359 | 0.0010 | 0.0000 | 0.0040 | -8.4873 | 0.9930 | 0.0048 | 0.0010 | 0.0052 | 0.0010 | 0.0685 | 0.0010 | 0.0766 | 0.0010 | 0.1358 | 0.0010 |
| Seychelles (36) v. Thailand (5) | 1919.1018 | 0.0030 | 0.0001 | 0.0749 | -19.2828 | 0.0160 | 0.0046 | 0.1049 | 0.0049 | 0.1059 | 0.3739 | 0.9790 | 0.3967 | 0.9790 | 0.5609 | 0.9790 |
| Australia (444) v. Mozambique (11) | 3639.2817 | 0.0579 | 0.0000 | 0.0210 | -1.8496 | 0.1159 | 0.0044 | 0.0010 | 0.0047 | 0.0010 | 0.0271 | 0.0010 | 0.0306 | 0.0010 | 0.0561 | 0.0010 |
| Papua New Guinea (9) v. Thailand (5) | 1272.2324 | 0.0410 | 0.0001 | 0.2567 | -944.1888 | 0.3776 | 0.0044 | 0.1998 | 0.0047 | 0.1998 | 0.9760 | 0.9560 | 0.9775 | 0.9560 | 0.9886 | 0.9560 |
| Papua New Guinea (9) v. Sri Lanka (12) | 1411.8786 | 0.2368 | 0.0001 | 0.1768 | -22.9494 | 0.9411 | 0.0042 | 0.0849 | 0.0045 | 0.0849 | 0.3760 | 0.0480 | 0.3980 | 0.0470 | 0.5625 | 0.0470 |
| Papua New Guinea (9) v. Seychelles (36) | 1790.9361 | 0.0749 | 0.0001 | 0.0150 | -13.0189 | 0.8761 | 0.0042 | 0.0340 | 0.0046 | 0.0340 | 0.1953 | 0.0649 | 0.2125 | 0.0699 | 0.3412 | 0.0679 |
| Arabian Sea (15) v. Mozambique (11) | 1666.5157 | 0.0699 | 0.0001 | 0.2727 | -15.7147 | 1.0000 | 0.0041 | 0.0380 | 0.0045 | 0.0360 | 0.2620 | 0.0010 | 0.2825 | 0.0010 | 0.4315 | 0.0010 |
| Brazil (50) v. Western North Atlantic (8) | 1764.9563 | 0.6284 | 0.0000 | 0.3706 | -14.8037 | 0.9958 | 0.0037 | 0.0884 | 0.0040 | 0.0884 | 0.2265 | 0.0063 | 0.2430 | 0.0073 | 0.3828 | 0.0063 |
| Reunion (28) v. South Africa (22) | 2046.1101 | 0.0040 | 0.0000 | 0.0020 | -9.3965 | 0.8252 | 0.0033 | 0.0230 | 0.0036 | 0.0230 | 0.0929 | 0.0420 | 0.1032 | 0.0400 | 0.1794 | 0.0420 |
| Mozambique (11) v. Sri Lanka (12) | 1510.9759 | 0.1429 | 0.0001 | 0.1918 | -19.8424 | 0.9980 | 0.0033 | 0.0889 | 0.0036 | 0.0919 | 0.3304 | 0.0020 | 0.3523 | 0.0020 | 0.5132 | 0.0020 |
| Reunion (28) v. Sri Lanka (12) | 1944.4230 | 0.0060 | 0.0000 | 0.1459 | -11.5330 | 0.3926 | 0.0032 | 0.0809 | 0.0034 | 0.0819 | 0.1553 | 0.3966 | 0.1704 | 0.3886 | 0.2819 | 0.3896 |
| Reunion (28) v. Thailand (5) | 1867.7590 | 0.0110 | 0.0001 | 0.0679 | -21.9852 | 0.0190 | 0.0030 | 0.2380 | 0.0032 | 0.2420 | 0.4139 | 0.9790 | 0.4368 | 0.9770 | 0.6017 | 0.9780 |
| Papua New Guinea (9) v. South Africa (22) | 1596.4856 | 0.3367 | 0.0001 | 0.0320 | -15.4042 | 0.9610 | 0.0020 | 0.2168 | 0.0022 | 0.2168 | 0.2440 | 0.0559 | 0.2633 | 0.0579 | 0.4078 | 0.0569 |
| Arabian Sea (15) v. South Africa (22) | 1883.4848 | 0.0050 | 0.0000 | 0.1908 | -10.8150 | 0.3726 | 0.0020 | 0.0909 | 0.0022 | 0.0909 | 0.1385 | 0.1888 | 0.1526 | 0.1708 | 0.2557 | 0.1788 |
| Arabian Sea (15) v. Reunion (28) | 2088.8182 | 0.0010 | 0.0000 | 0.0879 | -9.9971 | 0.1678 | 0.0019 | 0.1419 | 0.0021 | 0.1409 | 0.1199 | 0.4795 | 0.1326 | 0.4595 | 0.2255 | 0.4665 |
| Australia (444) v. Papua New Guinea (9) | 2714.5893 | 0.9471 | 0.0001 | 0.0200 | -1.9646 | 0.9720 | 0.0018 | 0.1179 | 0.0020 | 0.1179 | 0.0301 | 0.0340 | 0.0339 | 0.0360 | 0.0621 | 0.0350 |
| Arabian Sea (15) v. Australia (444) | 4448.0192 | 0.0010 | 0.0000 | 0.2068 | -1.7288 | 0.2877 | 0.0018 | 0.0220 | 0.0020 | 0.0220 | 0.0164 | 0.0679 | 0.0186 | 0.0629 | 0.0345 | 0.0659 |
| Brazil (50) v. Caribbean Sea (2) | 1530.3572 | 0.8222 | 0.0002 | 0.2847 | 14.5573 | 1.0000 | 0.0016 | 0.6250 | 0.0017 | 0.6250 | 7.5662 | 0.7500 | 6.9471 | 0.7500 | 1.6942 | 0.7500 |
| Australia (444) v. Seychelles (36) | 3423.0674 | 0.0629 | 0.0000 | 0.0030 | -1.6820 | 0.9201 | 0.0015 | 0.0010 | 0.0016 | 0.0010 | 0.0074 | 0.0010 | 0.0084 | 0.0010 | 0.0157 | 0.0010 |
| Australia (444) v. Thailand (5) | 3817.6280 | 0.0689 | 0.0001 | 0.2008 | -2.0420 | 0.2420 | 0.0014 | 0.3150 | 0.0015 | 0.3150 | 0.0537 | 0.6390 | 0.0603 | 0.6140 | 0.1082 | 0.6280 |
| Mozambique (11) v. South Africa (22) | 1659.7194 | 0.4166 | 0.0000 | 0.6663 | -13.9204 | 0.9950 | 0.0012 | 0.2757 | 0.0013 | 0.2817 | 0.2138 | 0.0010 | 0.2321 | 0.0010 | 0.3674 | 0.0010 |
| Arabian Sea (15) v. Seychelles (36) | 2112.8080 | 0.0010 | 0.0000 | 0.2068 | -9.4415 | 0.0899 | 0.0011 | 0.1848 | 0.0012 | 0.1848 | 0.1051 | 0.6304 | 0.1166 | 0.6114 | 0.2006 | 0.6184 |
| Australia (444) v. South Africa (22) | 3405.3277 | 0.1139 | 0.0000 | 0.1588 | -1.7372 | 0.4146 | 0.0008 | 0.1209 | 0.0008 | 0.1209 | 0.0112 | 0.1239 | 0.0127 | 0.1259 | 0.0237 | 0.1239 |
| Australia (444) v. Sri Lanka (12) | 3481.5843 | 0.1319 | 0.0000 | 0.3037 | -1.8199 | 0.7053 | 0.0005 | 0.3127 | 0.0006 | 0.3127 | 0.0206 | 0.2967 | 0.0232 | 0.2947 | 0.0429 | 0.2967 |
| South Africa (22) v. Sri Lanka (12) | 1719.7177 | 0.1728 | 0.0000 | 0.4416 | -12.4584 | 0.4905 | 0.0005 | 0.3646 | 0.0006 | 0.3656 | 0.1750 | 0.4675 | 0.1913 | 0.4615 | 0.3118 | 0.4625 |
| Seychelles (36) v. South Africa (22) | 1996.9972 | 0.0390 | 0.0000 | 0.3746 | -8.8703 | 0.2807 | 0.0005 | 0.3297 | 0.0005 | 0.3297 | 0.0784 | 0.4765 | 0.0874 | 0.4695 | 0.1538 | 0.4735 |
| Seychelles (36) v. Sri Lanka (12) | 1903.3093 | 0.0210 | 0.0000 | 0.1598 | -10.7907 | 0.2507 | 0.0002 | 0.4555 | 0.0002 | 0.4555 | 0.1362 | 0.7572 | 0.1499 | 0.7542 | 0.2518 | 0.7542 |
| Gulf of Mexico (37) v. Western North Atlantic (8) | 1571.2543 | 0.1748 | 0.0000 | 0.4486 | -21.7644 | 0.2218 | -0.0002 | 0.4883 | -0.0002 | 0.4883 | 0.3086 | 0.7946 | 0.3267 | 0.7923 | 0.4855 | 0.7934 |
| Sri Lanka (12) v. Thailand (5) | 1360.2400 | 0.1818 | 0.0001 | 0.7233 | -60.7430 | 0.1041 | -0.0005 | 0.5654 | -0.0006 | 0.5654 | 0.7015 | 0.9009 | 0.7177 | 0.9009 | 0.8341 | 0.9009 |
| Arabian Sea (15) v. Sri Lanka (12) | 1614.4357 | 0.5245 | 0.0000 | 0.4935 | -13.9062 | 0.4885 | -0.0014 | 0.7622 | -0.0015 | 0.7622 | 0.2158 | 0.7113 | 0.2344 | 0.7153 | 0.3703 | 0.7143 |
| South Africa (22) v. Thailand (5) | 1618.4549 | 0.0999 | 0.0001 | 0.3077 | -25.7692 | 0.0280 | -0.0017 | 0.6863 | -0.0018 | 0.6863 | 0.4611 | 0.9820 | 0.4836 | 0.9830 | 0.6466 | 0.9830 |
| Arabian Sea (15) v. Thailand (5) | 1441.9783 | 0.4725 | 0.0001 | 0.5614 | -35.0742 | 0.1528 | -0.0019 | 0.7233 | -0.0020 | 0.7233 | 0.5595 | 0.8841 | 0.5805 | 0.8851 | 0.7310 | 0.8841 |
| Caribbean Sea (2) v. Gulf of Mexico (37) | 1323.0750 | 0.5604 | 0.0001 | 0.4815 | 11.3906 | 1.0000 | -0.0020 | 0.5714 | -0.0022 | 1.0000 | -9.8053 | 1.0000 | -8.6016 | 1.0000 | 2.0904 | 1.0000 |
| Mozambique (11) v. Thailand (5) | 1270.4968 | 0.5355 | 0.0001 | 0.5844 | -134.4816 | 0.2073 | -0.0027 | 0.7239 | -0.0029 | 0.7159 | 0.8477 | 0.9925 | 0.8568 | 0.9925 | 0.9225 | 0.9925 |
| Caribbean Sea (2) v. Western North Atlantic (8) | 794.9991 | 0.6404 | 0.0001 | 0.4745 | NA | NA | -0.0077 | 1.0000 | -0.0083 | 1.0000 | NA | NA | NA | NA | NA | NA |
| Australia (444) v. Indonesia (4) | 4132.4024 | 0.0240 | 0.0001 | 0.0110 | NA | NA | NA | NA | NA | NA | NA | NA | NA | NA | NA | NA |
| Fiji (25) v. Indonesia (4) | 2319.4034 | 0.0010 | 0.0002 | 0.0020 | NA | NA | NA | NA | NA | NA | NA | NA | NA | NA | NA | NA |
| Indonesia (4) v. Japan (44) | 2889.8901 | 0.0010 | 0.0003 | 0.0020 | NA | NA | NA | NA | NA | NA | NA | NA | NA | NA | NA | NA |
| Indonesia (4) v. South Africa (22) | 1619.1846 | 0.0679 | 0.0001 | 0.0390 | NA | NA | NA | NA | NA | NA | NA | NA | NA | NA | NA | NA |

### 8.7.3 Fst per Region

```
strata <- BS.dartcap.gt@schemes$Region
strata[strata %in% c("E-IO","W-PAC")] <- "E-IO/W-PAC"
names(strata) <- BS.dartcap.gt@schemes$INDIVIDUALS
strataG::setStrata(BS.dartcap.gt) <- strata

Fst.gt <- strataG::popStructTest(
    BS.dartcap.gt,
    nrep = 1000,
    stats = "all",
    type = "pairwise",
    keep.null = FALSE,
    quietly = TRUE,
    nb.cores =  parallel::detectCores() - 1,
    write.output = FALSE
  )
save(Fst.gt, file = "DArTcap_ALL_FST_Regions.Rdata")
readr::write_tsv(Fst.gt$pairwise$result, path = "DArTcap_ALL_FST_Regions.txt")
```

```
load("DArTcap_ALL_FST_Regions.Rdata")
kableExtra::kbl(Fst.gt$pairwise$result[order(Fst.gt$pairwise$result$Fst,
                                       decreasing = TRUE),
                                  -c(1:4)], digits = 4,
  caption = "Pairwise Fst for 3443 DArTcap loci between the all regions",
  longtable = TRUE, booktabs = TRUE) %>%
  kableExtra::kable_styling(full_width = FALSE,font_size = 5,
                            latex_options = c("repeat_header", "hold_position")) %>%
  kableExtra::landscape()

pop.levels1 <- c("E-PAC", "W-ATL", "E-ATL", "W-IO", "N-IO", "E-IO/W-PAC",
                 "Japan" , "Fiji")

m2 <- Fst.gt$pairwise$result[order(match(Fst.gt$pairwise$result$strata.1,
                                         pop.levels1)),c(1,2,11)]
Gdf <- igraph::graph.data.frame(m2, directed = FALSE)
m2.1 <- igraph::as_adjacency_matrix(Gdf, names = TRUE, sparse = FALSE,
                                    attr = "Fst", type = 'lower')

m2 <- Fst.gt$pairwise$result[order(match(Fst.gt$pairwise$result$strata.1,
                                         pop.levels1)),c(1,2,12)]
Gdf <- igraph::graph.data.frame(m2, directed = FALSE)
m2.2 <- igraph::as_adjacency_matrix(Gdf, names = TRUE, sparse = FALSE,
                                    attr = "Fst.p.val", type = 'lower')
m2$Fst.p.adj <- p.adjust(m2$Fst.p.val, "bonferroni")
Gdf <- igraph::graph.data.frame(m2, directed = FALSE)
m2.3 <- igraph::as_adjacency_matrix(Gdf, names = TRUE, sparse = FALSE,
                                    attr = "Fst.p.adj", type = 'lower')

write.csv(m2.1, file = "DArTcap_ALL_FST_Regions.csv")
write.csv(m2.2, file = "DArTcap_ALL_FST_Pval_Regions.csv")
write.csv(m2.3, file = "DArTcap_ALL_FST_Pval_bonferroni_Regions.csv")
```

Table 21: Pairwise Fst for 3443 DArTcap loci between the all regions

| | Chi2 | Chi2.p.val | D | D.p.val | Fis | Fis.p.val | Fst | Fst.p.val | F'st | F'st.p.val | Gst | Gst.p.val | G'st | G'st.p.val | G"st | G"st.p.val |
|---|---|---|---|---|---|---|---|---|---|---|---|---|---|---|---|---|
| E-ATL (1) v. E-PAC (16) | 11158.858 | 0.0559 | 0.0114 | 0.0559 | NA | NA | 0.7013 | 1.000 | 0.7555 | 1.000 | NA | NA | NA | NA | NA | NA |
| Japan (44) v. W-ATL (97) | 120117.283 | 0.0010 | 0.0211 | 0.0010 | -4.0027 | 1.0000 | 0.6667 | 0.001 | 0.7245 | 0.001 | 0.5080 | 0.0010 | 0.5686 | 0.0010 | 0.7139 | 0.0010 |
| Fiji (25) v. W-ATL (97) | 95752.021 | 0.0010 | 0.0206 | 0.0010 | -4.6818 | 1.0000 | 0.6629 | 0.001 | 0.7235 | 0.001 | 0.5102 | 0.0010 | 0.5706 | 0.0010 | 0.7157 | 0.0010 |
| E-PAC (16) v. Japan (44) | 48791.209 | 0.0010 | 0.0182 | 0.0010 | -10.8638 | 1.0000 | 0.6609 | 0.001 | 0.7115 | 0.001 | 0.5930 | 0.0010 | 0.6435 | 0.0010 | 0.7762 | 0.0010 |
| N-IO (32) v. W-ATL (97) | 104843.972 | 0.0010 | 0.0200 | 0.0010 | -3.4887 | 1.0000 | 0.6595 | 0.001 | 0.7187 | 0.001 | 0.4966 | 0.0010 | 0.5574 | 0.0010 | 0.7042 | 0.0010 |
| E-PAC (16) v. Fiji (25) | 32838.244 | 0.0010 | 0.0177 | 0.0010 | -12.8862 | 1.0000 | 0.6594 | 0.001 | 0.7427 | 0.001 | 0.6143 | 0.0010 | 0.6629 | 0.0010 | 0.7912 | 0.0010 |
| W-ATL (97) v. W-IO (97) | 167080.101 | 0.0010 | 0.0201 | 0.0010 | -2.1320 | 1.0000 | 0.6526 | 0.001 | 0.7090 | 0.001 | 0.4891 | 0.0010 | 0.5500 | 0.0010 | 0.6978 | 0.0010 |
| E-PAC (16) v. N-IO (32) | 39020.274 | 0.0010 | 0.0172 | 0.0010 | -9.3166 | 1.0000 | 0.6516 | 0.001 | 0.6994 | 0.001 | 0.5776 | 0.0010 | 0.6289 | 0.0010 | 0.7648 | 0.0010 |
| E-IO/W-PAC (457) v. W-ATL (97) | 457028.313 | 0.0010 | 0.0200 | 0.0010 | -0.2794 | 1.0000 | 0.6413 | 0.001 | 0.6887 | 0.001 | 0.4836 | 0.0010 | 0.5448 | 0.0010 | 0.6932 | 0.0010 |
| E-PAC (16) v. W-IO (97) | 83951.675 | 0.0010 | 0.0172 | 0.0010 | -5.7967 | 1.0000 | 0.6372 | 0.001 | 0.7023 | 0.001 | 0.5446 | 0.0010 | 0.5979 | 0.0010 | 0.7397 | 0.0010 |
| E-ATL (1) v. W-ATL (97) | 27769.816 | 0.0090 | 0.0129 | 0.0090 | 4.1821 | 1.0000 | 0.6331 | 1.000 | 0.6949 | 1.000 | 22.5515 | 1.0000 | 16.9504 | 1.0000 | 1.6773 | 1.0000 |
| E-IO/W-PAC (457) v. E-PAC (16) | 306720.921 | 0.0010 | 0.0172 | 0.0010 | -1.0829 | 1.0000 | 0.6265 | 0.001 | 0.6694 | 0.001 | 0.5104 | 0.0010 | 0.5657 | 0.0010 | 0.7124 | 0.0010 |
| E-PAC (16) v. W-ATL (97) | 21245.739 | 0.0010 | 0.0025 | 0.0010 | -8.8181 | 1.0000 | 0.3490 | 0.001 | 0.3798 | 0.001 | 0.3044 | 0.0010 | 0.3305 | 0.0010 | 0.4868 | 0.0010 |
| E-ATL (1) v. Japan (44) | 6184.421 | 0.0210 | 0.0009 | 0.0210 | 3.6975 | 1.0000 | 0.2067 | 1.000 | 0.2253 | 1.000 | -2.0230 | 1.0000 | -1.2649 | 1.0000 | 3.2141 | 1.0000 |
| E-ATL (1) v. Fiji (25) | 3717.034 | 0.0370 | 0.0008 | 0.0719 | 3.5244 | 1.0000 | 0.1610 | 1.000 | 0.1913 | 1.000 | -1.8479 | 1.0000 | -1.0793 | 1.0000 | 3.4522 | 1.0000 |
| E-ATL (1) v. E-IO/W-PAC (457) | 28506.972 | 0.0040 | 0.0007 | 0.0040 | -14.8084 | 1.0000 | 0.1574 | 1.000 | 0.1724 | 1.000 | 0.7535 | 1.0000 | 0.7700 | 1.0000 | 0.8688 | 1.0000 |
| E-ATL (1) v. N-IO (32) | 4376.922 | 0.0360 | 0.0007 | 0.0360 | 3.7775 | 1.0000 | 0.1573 | 1.000 | 0.1714 | 1.000 | -2.1933 | 1.0000 | -1.4427 | 1.0000 | 3.0470 | 1.0000 |
| E-ATL (1) v. W-IO (97) | 9473.367 | 0.0130 | 0.0007 | 0.0130 | 4.4781 | 1.0000 | 0.1552 | 1.000 | 0.1731 | 1.000 | -4.1996 | 1.0000 | -3.2707 | 1.0000 | 2.3348 | 1.0000 |
| Fiji (25) v. Japan (44) | 5695.638 | 0.0010 | 0.0003 | 0.0010 | -8.2308 | 1.0000 | 0.0617 | 0.001 | 0.0668 | 0.001 | 0.0972 | 0.0010 | 0.1084 | 0.0010 | 0.1873 | 0.0010 |
| Fiji (25) v. W-IO (97) | 5603.824 | 0.0010 | 0.0002 | 0.0010 | -5.2621 | 0.9990 | 0.0313 | 0.001 | 0.0346 | 0.001 | 0.0517 | 0.0010 | 0.0581 | 0.0010 | 0.1044 | 0.0010 |
| E-IO/W-PAC (457) v. Fiji (25) | 9554.750 | 0.0010 | 0.0002 | 0.0010 | -1.4206 | 0.0460 | 0.0310 | 0.001 | 0.0334 | 0.001 | 0.0243 | 0.0010 | 0.0275 | 0.0010 | 0.0505 | 0.0010 |
| Japan (44) v. N-IO (32) | 4233.906 | 0.0010 | 0.0002 | 0.0010 | -7.4254 | 1.0000 | 0.0307 | 0.001 | 0.0331 | 0.001 | 0.0646 | 0.0010 | 0.0724 | 0.0010 | 0.1286 | 0.0010 |
| Fiji (25) v. N-IO (32) | 3818.756 | 0.0010 | 0.0002 | 0.0010 | -7.3585 | 1.0000 | 0.0301 | 0.001 | 0.0325 | 0.001 | 0.0821 | 0.0010 | 0.0918 | 0.0010 | 0.1607 | 0.0010 |
| Japan (44) v. W-IO (97) | 6003.192 | 0.0010 | 0.0002 | 0.0010 | -5.2873 | 1.0000 | 0.0295 | 0.001 | 0.0324 | 0.001 | 0.0373 | 0.0010 | 0.0420 | 0.0010 | 0.0764 | 0.0010 |
| E-IO/W-PAC (457) v. Japan (44) | 9393.609 | 0.0010 | 0.0002 | 0.0010 | -1.6860 | 1.0000 | 0.0287 | 0.001 | 0.0308 | 0.001 | 0.0204 | 0.0010 | 0.0230 | 0.0010 | 0.0425 | 0.0010 |
| E-IO/W-PAC (457) v. N-IO (32) | 4130.868 | 0.0010 | 0.0000 | 0.0100 | -1.5974 | 0.4575 | 0.0017 | 0.001 | 0.0019 | 0.001 | 0.0078 | 0.0010 | 0.0089 | 0.0010 | 0.0166 | 0.0010 |
| E-IO/W-PAC (457) v. W-IO (97) | 3975.773 | 0.0010 | 0.0000 | 0.0010 | -1.4588 | 0.2657 | 0.0014 | 0.001 | 0.0015 | 0.001 | 0.0031 | 0.0010 | 0.0036 | 0.0010 | 0.0067 | 0.0010 |
| N-IO (32) v. W-IO (97) | 2893.453 | 0.0010 | 0.0000 | 0.0080 | -5.0487 | 0.1868 | 0.0010 | 0.037 | 0.0011 | 0.036 | 0.0271 | 0.2897 | 0.0306 | 0.2747 | 0.0562 | 0.2817 |

## 8.8 PCA

```
pca1 <- adegenet::glPca(BS.dartcap.gl, nf = 5, parallel = TRUE,
                        n.cores = parallel::detectCores() - 1)
save(pca1, file = "DArTcap_ALL_PCA.Rdata")
```

```
load("DArTcap_ALL_PCA.Rdata")

BS.pca.scores <- as.data.frame(pca1$scores)
BS.pca.scores$pop <- pop(BS.dartcap.gl)
cols <- adegenet::funky(nPop(BS.dartcap.gl))

p <-
  ggplot2::ggplot(BS.pca.scores, ggplot2::aes(x = PC1, y = PC2, colour = pop)) +
  ggplot2::geom_point(size = 2) + ggplot2::scale_color_manual(values = cols) +
  ggplot2::geom_hline(yintercept = 0) + ggplot2::geom_vline(xintercept = 0) +
  ggplot2::theme_minimal() +
  ggplot2::theme(
    axis.text = ggplot2::element_text(size = 5),
    axis.title.x = ggplot2::element_text(size = 10),
    axis.title.y = ggplot2::element_text(size = 10),
    legend.text = ggplot2::element_text(size = 7)
  )
p
```

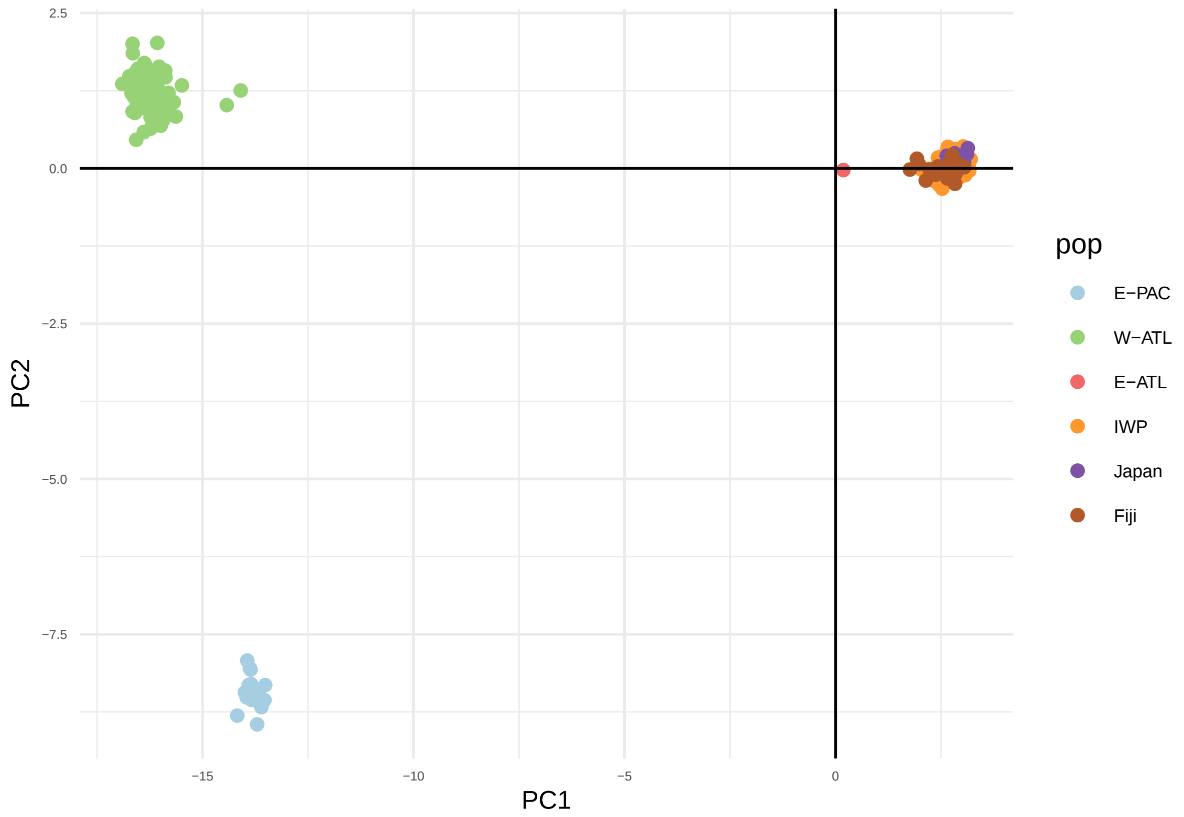


```
ggplot2::ggsave(p, filename = "DArTcap_ALL_PCA1.png", width = 30,
                height = 15,units = "cm")


p <-
  ggplot2::ggplot(BS.pca.scores, ggplot2::aes(x = PC1, y = PC3, colour = pop)) +
  ggplot2::geom_point(size = 2) + ggplot2::scale_color_manual(values = cols) +
```

```
  ggplot2::geom_hline(yintercept = 0) + ggplot2::geom_vline(xintercept = 0) +
  ggplot2::theme_minimal() +
  ggplot2::theme(
    axis.text = ggplot2::element_text(size = 5),
    axis.title.x = ggplot2::element_text(size = 10),
    axis.title.y = ggplot2::element_text(size = 10),
    legend.text = ggplot2::element_text(size = 7)
  )
p
```

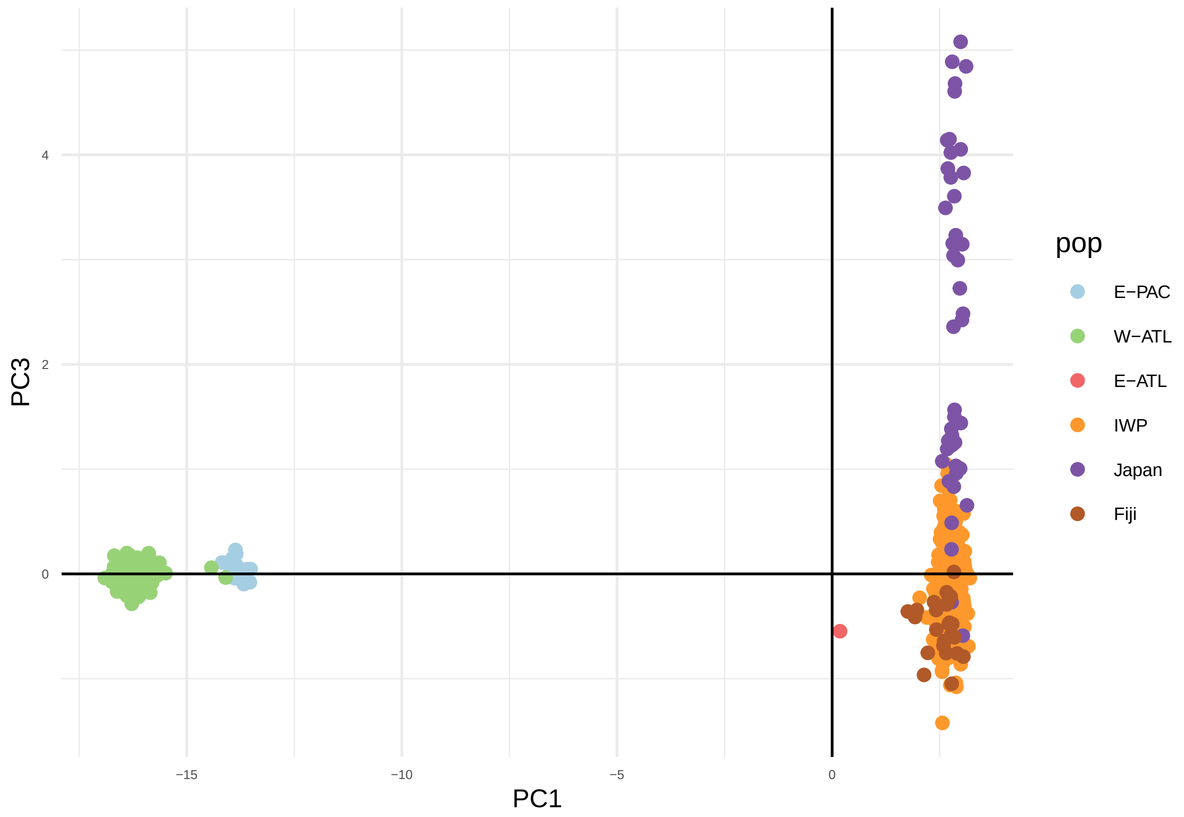


```
ggplot2::ggsave(p, filename = "DArTcap_ALL_PCA2.png", width = 30,
                height = 15,units = "cm")

p <-
  ggplot2::ggplot(BS.pca.scores, ggplot2::aes(x = PC2, y = PC3, colour = pop)) +
  ggplot2::geom_point(size = 2) + ggplot2::scale_color_manual(values = cols) +
  ggplot2::geom_hline(yintercept = 0) + ggplot2::geom_vline(xintercept = 0) +
  ggplot2::theme_minimal() +
  ggplot2::theme(
    axis.text = ggplot2::element_text(size = 5),
    axis.title.x = ggplot2::element_text(size = 10),
    axis.title.y = ggplot2::element_text(size = 10),
    legend.text = ggplot2::element_text(size = 7)
  )
p
```

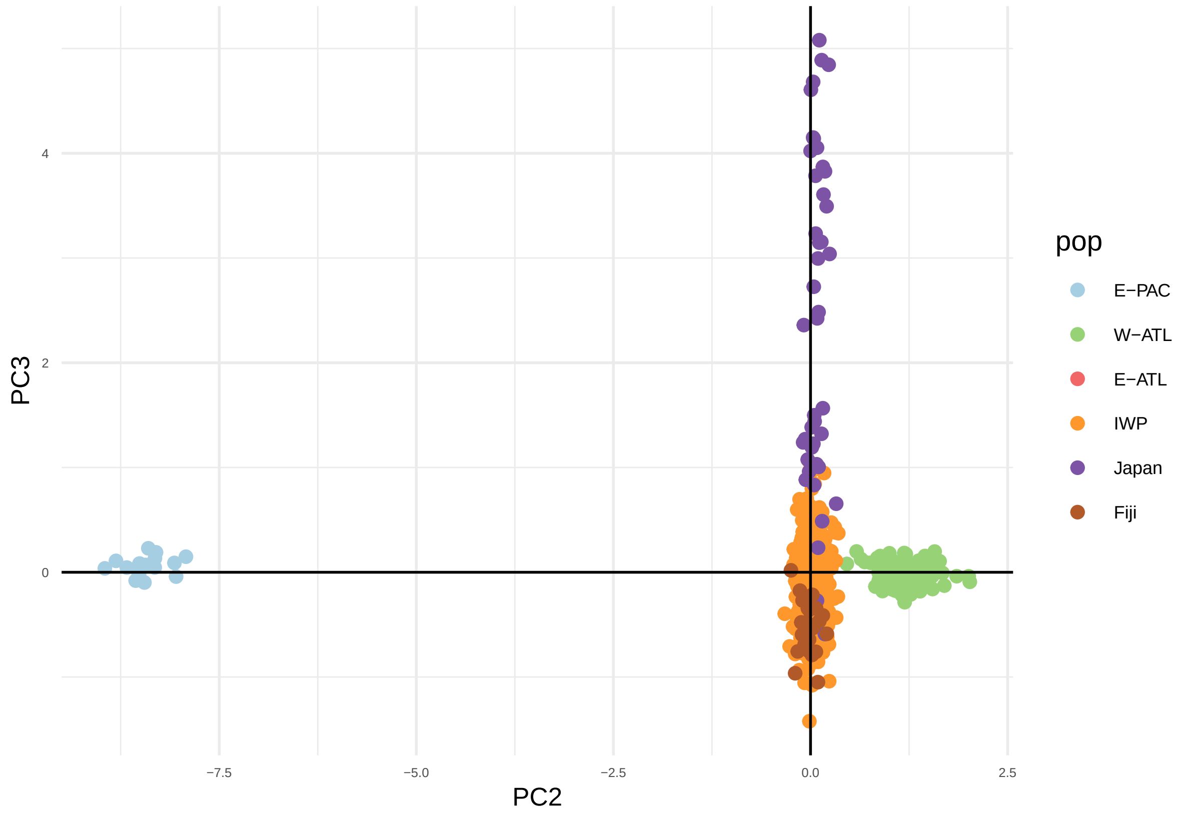


```
ggplot2::ggsave(p, filename = "DArTcap_ALL_PCA3.png", width = 30,
                height = 15,units = "cm")


p <-
  ggplot2::ggplot(BS.pca.scores, ggplot2::aes(x = PC3, y = PC4, colour = pop)) +
  ggplot2::geom_point(size = 2) + ggplot2::scale_color_manual(values = cols) +
  ggplot2::geom_hline(yintercept = 0) + ggplot2::geom_vline(xintercept = 0) +
  ggplot2::theme_minimal() +
  ggplot2::theme(
    axis.text = ggplot2::element_text(size = 5),
    axis.title.x = ggplot2::element_text(size = 10),
    axis.title.y = ggplot2::element_text(size = 10),
    legend.text = ggplot2::element_text(size = 7)
  )
p
```

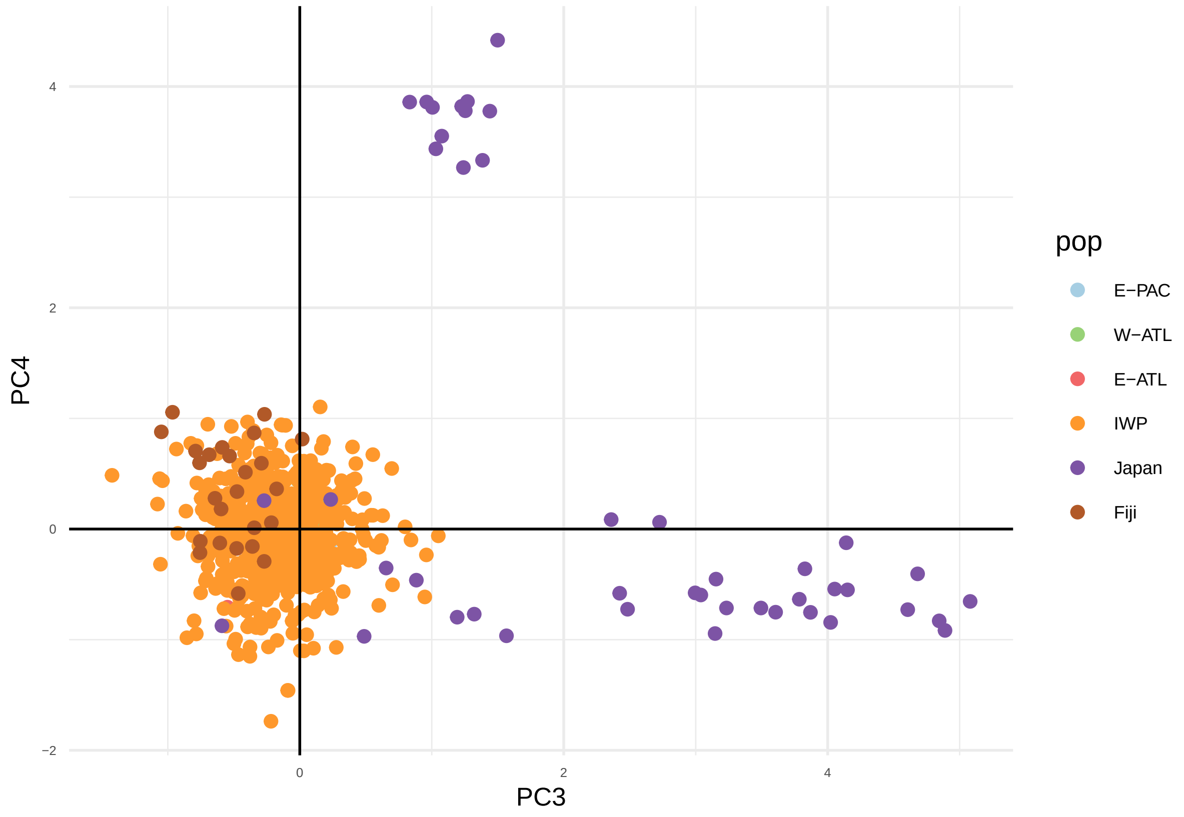


```
ggplot2::ggsave(p, filename = "DArTcap_ALL_PCA4.png", width = 30,
                height = 15,units = "cm")

p <-
  ggplot2::ggplot(BS.pca.scores, ggplot2::aes(x = PC3, y = PC5, colour = pop)) +
  ggplot2::geom_point(size = 2) + ggplot2::scale_color_manual(values = cols) +
  ggplot2::geom_hline(yintercept = 0) + ggplot2::geom_vline(xintercept = 0) +
  ggplot2::theme_minimal() +
  ggplot2::theme(
    axis.text = ggplot2::element_text(size = 5),
    axis.title.x = ggplot2::element_text(size = 10),
    axis.title.y = ggplot2::element_text(size = 10),
    legend.text = ggplot2::element_text(size = 7)
  )
p
```

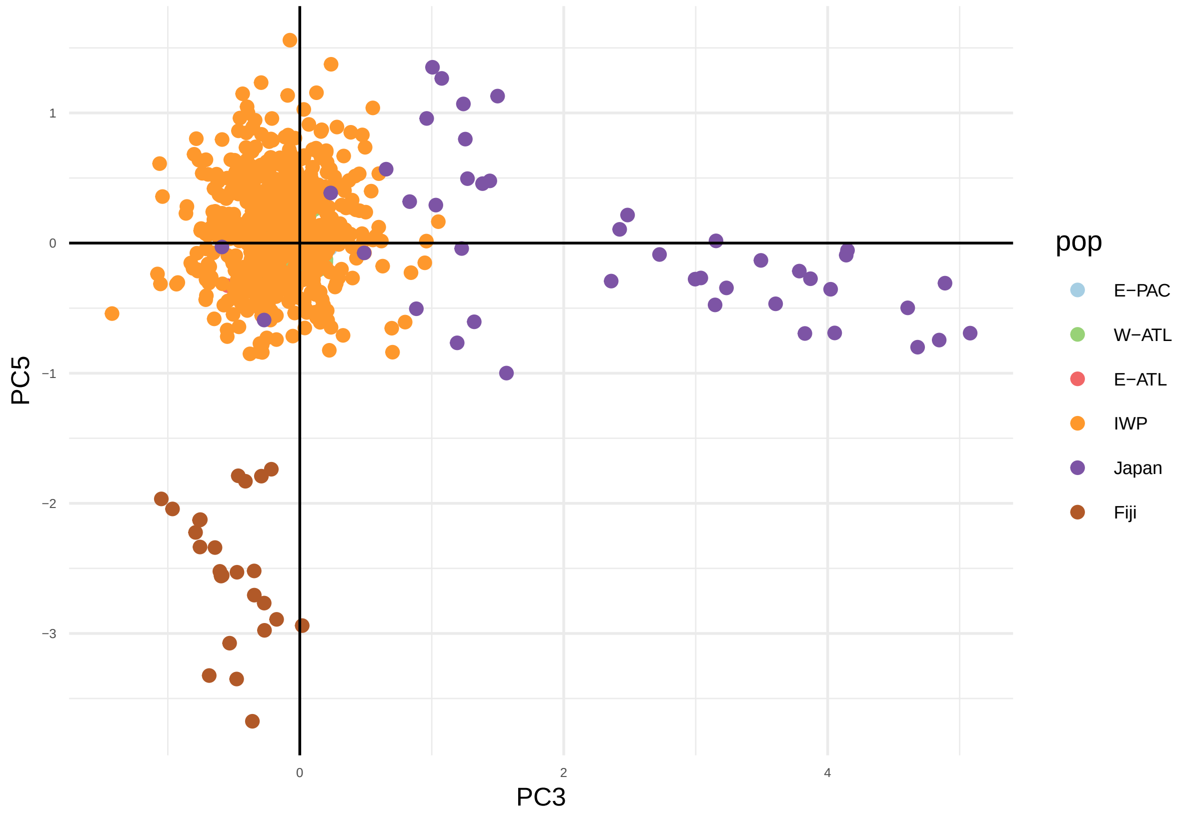


```
ggplot2::ggsave(p, filename = "DArTcap_ALL_PCA5.png", width = 30,
                height = 15,units = "cm")
```

## 8.9 DAPC

### 8.9.1 Optimal number of clusters with neutral markers

```
set.seed(124)
grp <-
  adegenet::find.clusters(
    BS.dartcap.gl,
    max.n.clust = 18,
    n.pca = adegenet::nInd(BS.dartcap.gl) / 3,
    stat = "BIC",
    parallel = TRUE,
    n.cores = parallel::detectCores() - 1
  )

save(grp, file = "DArTcap_ALL_grp_.Rdata")
```

```
load("DArTcap_ALL_grp_.Rdata")

y <- as.numeric(grp$Kstat)
x <- 1:18
data <- data.frame(x,y,stringsAsFactors = F)
plot <- ggplot2::ggplot(data, ggplot2::aes(x,y)) +
  ggplot2::geom_point(size = 5, shape = 1, color = "blue") +
  ggplot2::geom_line(size = 1,color = "blue") +
  ggplot2::scale_x_continuous(name = waiver(),
                              breaks = seq(from = 0,
                                           to = nrow(BS.dartcap.gl) - 1, by = 1)) +
```

```r
  ggplot2::labs(subtitle = "",
                y = "Bayesian Information Criterion",
                x = "Number of clusters",
                title = "",
                caption = "") +
  ggplot2::theme_classic() +
  ggplot2::theme(
    axis.text = ggplot2::element_text(size = 5),
    axis.title.x = ggplot2::element_text(size = 20,vjust = 0, hjust = 0.5),
    axis.title.y = ggplot2::element_text(size = 20,vjust = 2, hjust = 0.5))
print(plot)
```

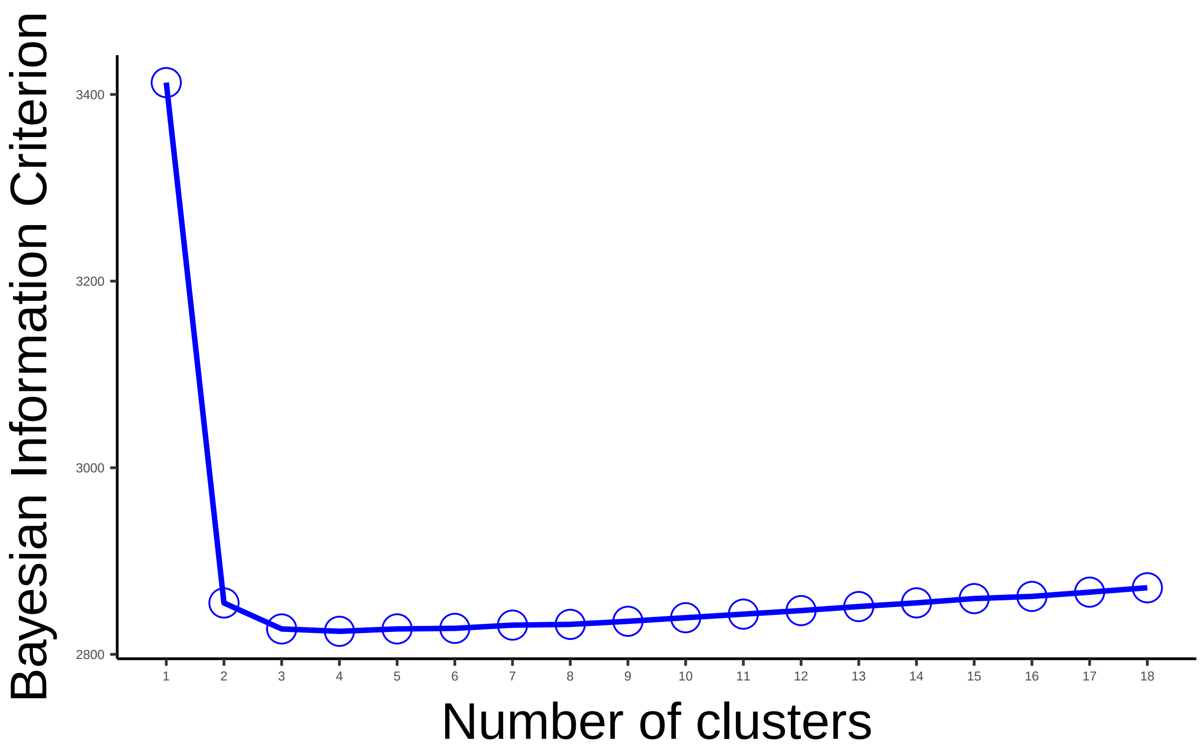


```r
ggplot2::ggsave(plot, filename = "DArTcap_ALL_grp.png", width = 30,
                height = 15,units = "cm")
```

### 8.9.2 Cross-validation to identify the optimal number of PCs

Uses a training - holdout set of individuals to check how reliable individuals can be assigned.

```r
set.seed(124)
x <- BS.dartcap.gl
x <- x[x$pop != "Sierra Leone",] #only1 sample

xval <-
  adegenet::xvalDapc(
  tab(x, NA.method = "mean"),
  adegenet::pop(x),
  n.pca = seq(1, adegenet::nInd(x), by = 50),
  training.set = 0.9,
  result = "groupMean",
  center = TRUE,
  scale = FALSE,
```

```r
  n.rep = 1000,
  parallel = "multicore",
  ncpus = 1,
  xval.plot = TRUE
  )
print(xval[2:6])
PC <- as.numeric(xval$`Number of PCs Achieving Lowest MSE`)

#refine the analysis
set.seed(124)
png(file="DArTcap_ALL_xval.png")
xval <-
  adegenet::xvalDapc(
  tab(x, NA.method = "mean"),
  adegenet::pop(x),
  n.pca = (PC - 15):(PC + 15),
  n.rep = 1000,
  parallel = "multicore",
  ncpus = 1,
  xval.plot = TRUE
  )
dev.off()
save(xval, file = "DArTcap_ALL_xval.rdata")
```

```r
load("DArTcap_ALL_xval.rdata")
print(xval[2:6])
PC <- as.numeric(xval$`Number of PCs Achieving Lowest MSE`)
```

```
## $`Median and Confidence Interval for Random Chance`
##       2.5%        50%      97.5%
## 0.02065971 0.03214135 0.04492563
##
## $`Mean Successful Assignment by Number of PCs of PCA`
##       186       187       188       189       190       191       192       193
## 0.2470927 0.2462834 0.2473624 0.2470312 0.2477719 0.2475748 0.2482662 0.2481249
##       194       195       196       197       198       199       200       201
## 0.2495385 0.2485589 0.2488955 0.2482662 0.2479600 0.2490014 0.2492077 0.2488088
##       202       203       204       205       206       207       208       209
## 0.2495266 0.2486349 0.2480373 0.2491746 0.2475283 0.2507537 0.2510989 0.2529229
##       210       211       212       213       214       215       216
## 0.2517774 0.2515806 0.2515262 0.2518021 0.2508993 0.2504337 0.2498401
##
## $`Number of PCs Achieving Highest Mean Success`
## [1] "209"
##
## $`Root Mean Squared Error by Number of PCs of PCA`
##       186       187       188       189       190       191       192       193
## 0.7530993 0.7539175 0.7528239 0.7531685 0.7524294 0.7526268 0.7519474 0.7520711
##       194       195       196       197       198       199       200       201
## 0.7506672 0.7516384 0.7513023 0.7519209 0.7522335 0.7512127 0.7509966 0.7513961
##       202       203       204       205       206       207       208       209
## 0.7506742 0.7515642 0.7521607 0.7510212 0.7526754 0.7494574 0.7491003 0.7472907
##       210       211       212       213       214       215       216
## 0.7484201 0.7486261 0.7486684 0.7483942 0.7493148 0.7497796 0.7503830
##
## $`Number of PCs Achieving Lowest MSE`
## [1] "209"
```

```r
dapc.all.object.names <- c()
for (K in 2:10) {
  set.seed(124)
  grp <- adegenet::find.clusters(BS.dartcap.gl, n.clust = K, n.pca = PC)
  set.seed(124)
  dapc.all.neutral <- adegenet::dapc(BS.dartcap.gl, grp$grp, n.da = K - 1,
                                      n.pca = PC)
  assign(paste0("dapc.all.", K),value = dapc.all.neutral)
  dapc.all.object.names <- c(dapc.all.object.names,
                                      paste0("dapc.all.", K))
}
save(list = dapc.all.object.names, dapc.all.object.names,
     file = "DArTcap_ALL_DAPC.Rdata")
```

### 8.9.3 DAPC barplot

Group individuals according to DAPC posterior membership.

```r
load("DArTcap_ALL_DAPC.Rdata")
for (K in 2:10) {
  colour <- colours.prior[1:K]
  dapc <- get(dapc.all.object.names[K - 1])
  post <- as.matrix(dapc$posterior)
  colnames(post) <- paste0("Group", 1:nlevels(dapc$grp))
  locations <- BS.dartcap.gl$pop

  plot.dapc.FDD(x = post,
                locations = locations,
                colour = colour,
                region.lwd = 1,
                plotTitle = paste0("DAPC: all Bull Sharks - subset Australia\n for K=",
                                   K, " & PC=", PC, sep = ""))
  dev.print(
    device = png,
    file = paste0("DArTcap_ALL_DAPC_barplot_K", K, ".png"),
    res = 300,
    width = 30,
    height = 15,
    units = "cm")
}
```

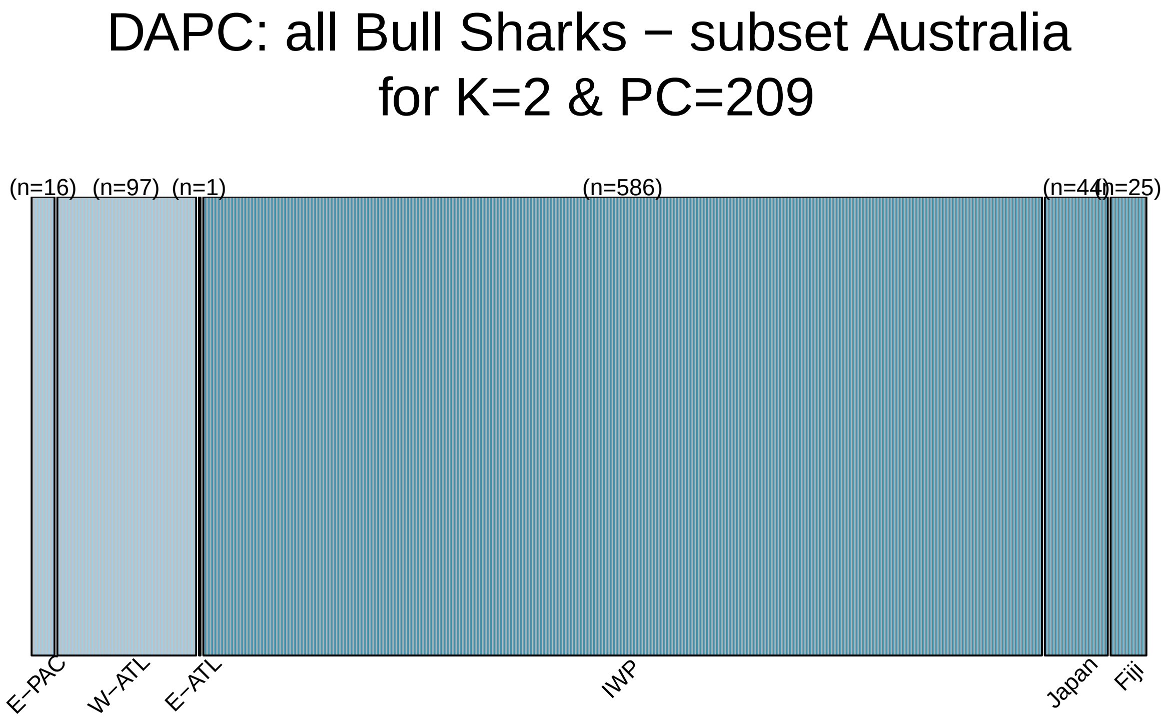
DAPC: all Bull Sharks − subset Australia
for K=2 & PC=209
(n=16)
(n=97)
(n=1)
(n=586)
(n=44)
(n=25)
E−PAC
W−ATL
E−ATL
IWP
Japan
Fiji

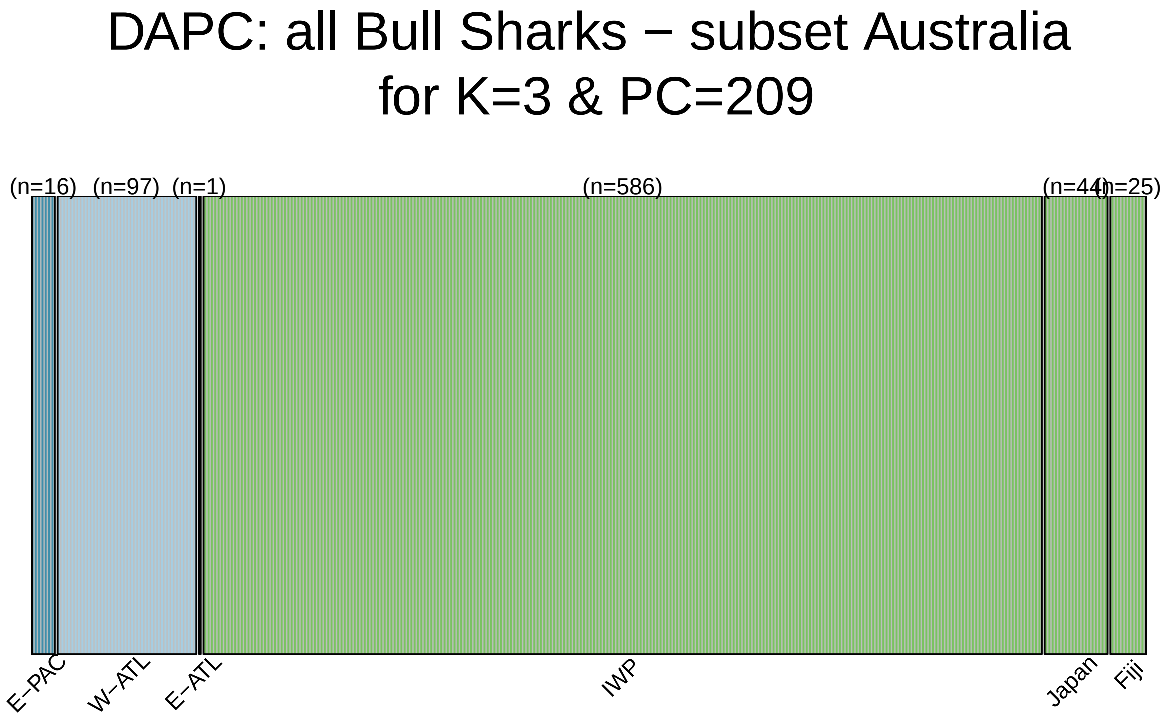
DAPC: all Bull Sharks − subset Australia
for K=3 & PC=209
(n=16)
(n=97)
(n=1)
(n=586)
(n=44)
(n=25)
E−PAC
W−ATL
E−ATL
IWP
Japan
Fiji

DAPC: all Bull Sharks − subset Australia
for K=4 & PC=209

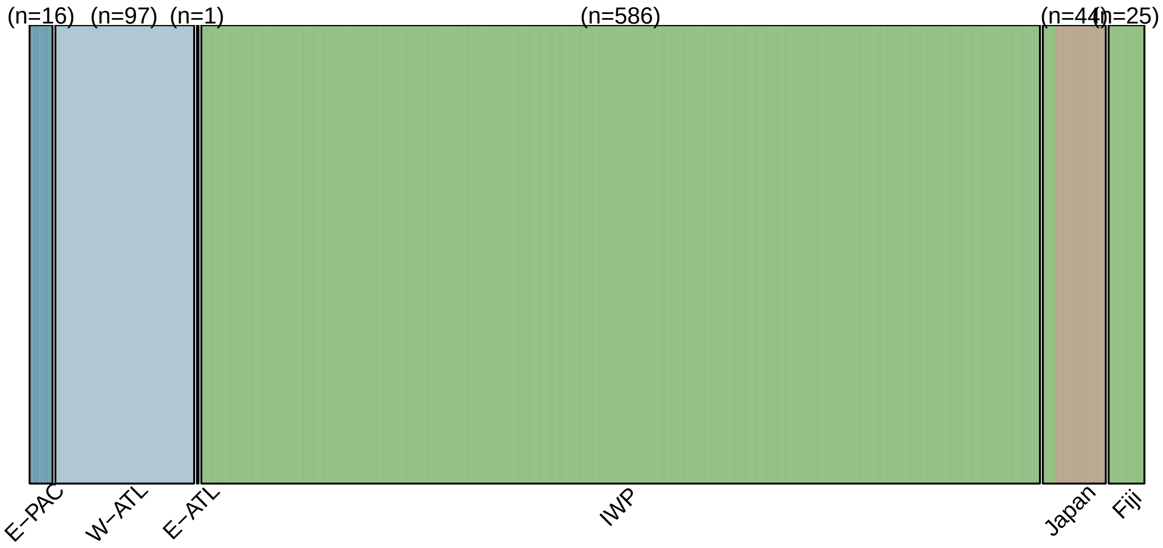


DAPC: all Bull Sharks − subset Australia
for K=5 & PC=209

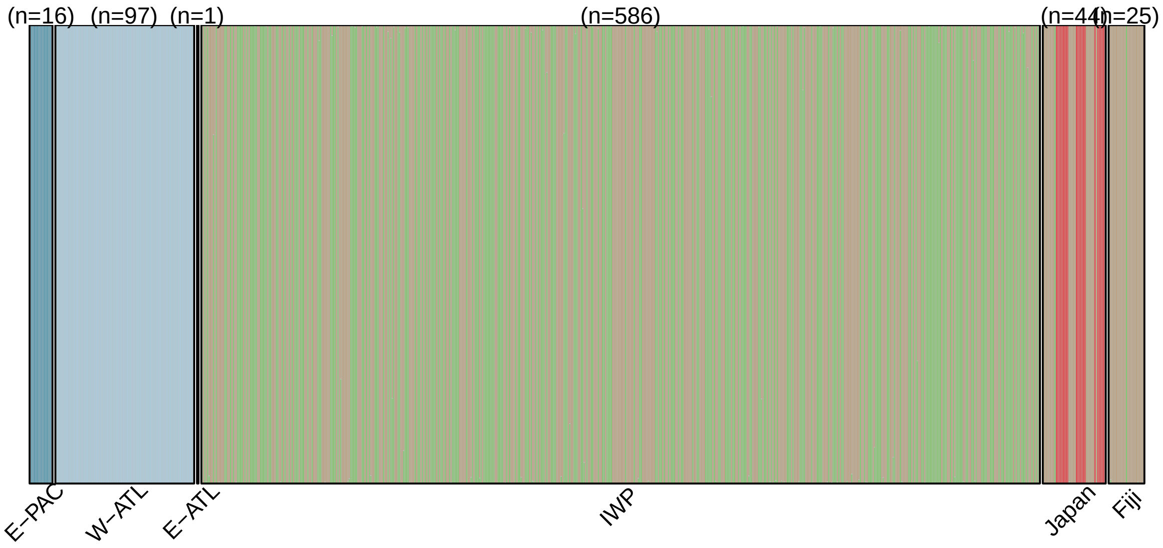

DAPC: all Bull Sharks − subset Australia
for K=6 & PC=209

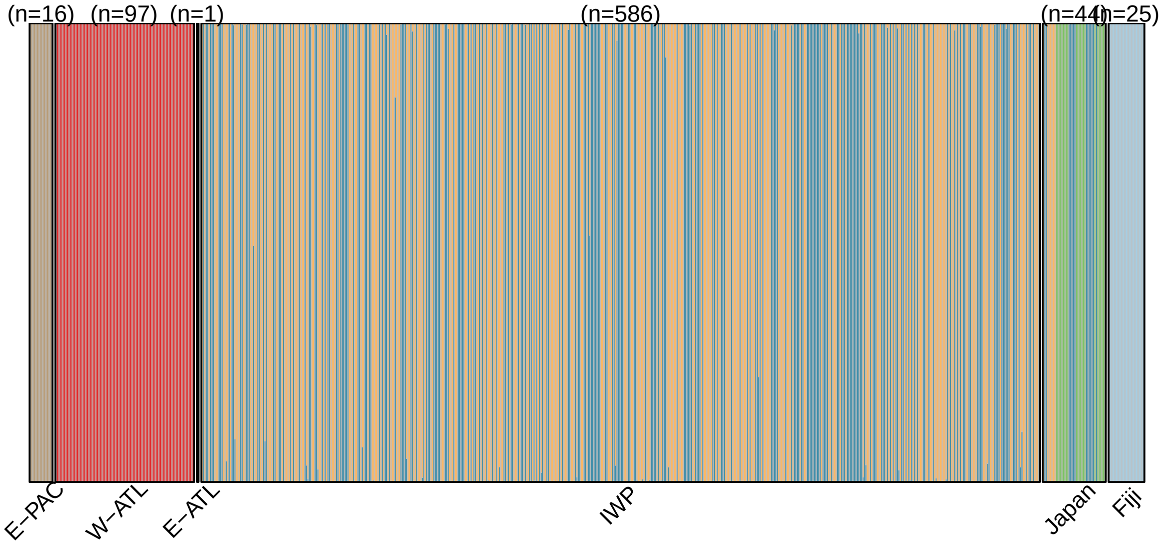


DAPC: all Bull Sharks − subset Australia
for K=7 & PC=209

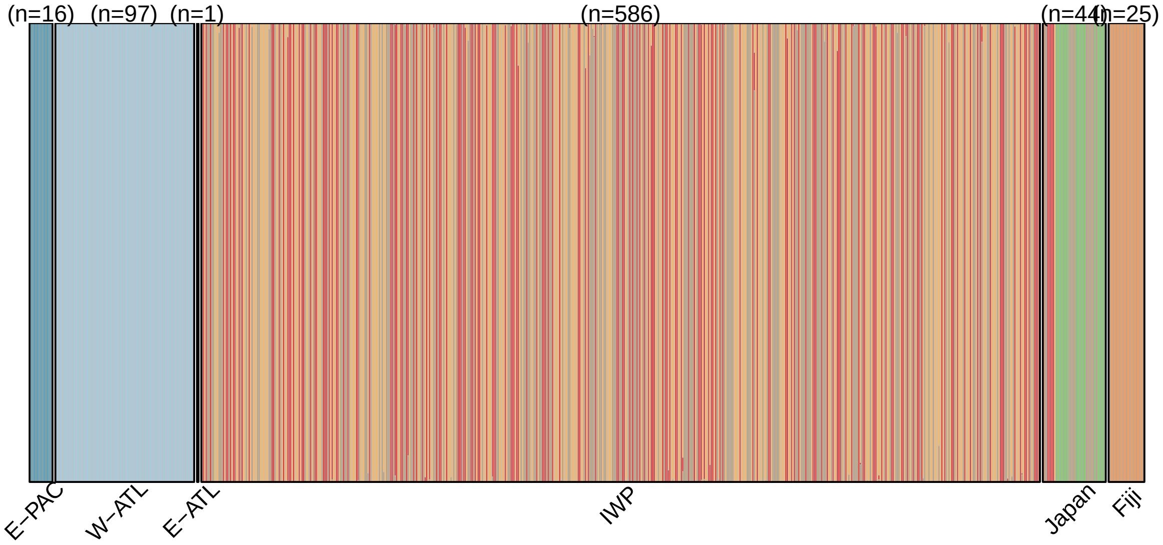

DAPC: all Bull Sharks − subset Australia
for K=8 & PC=209

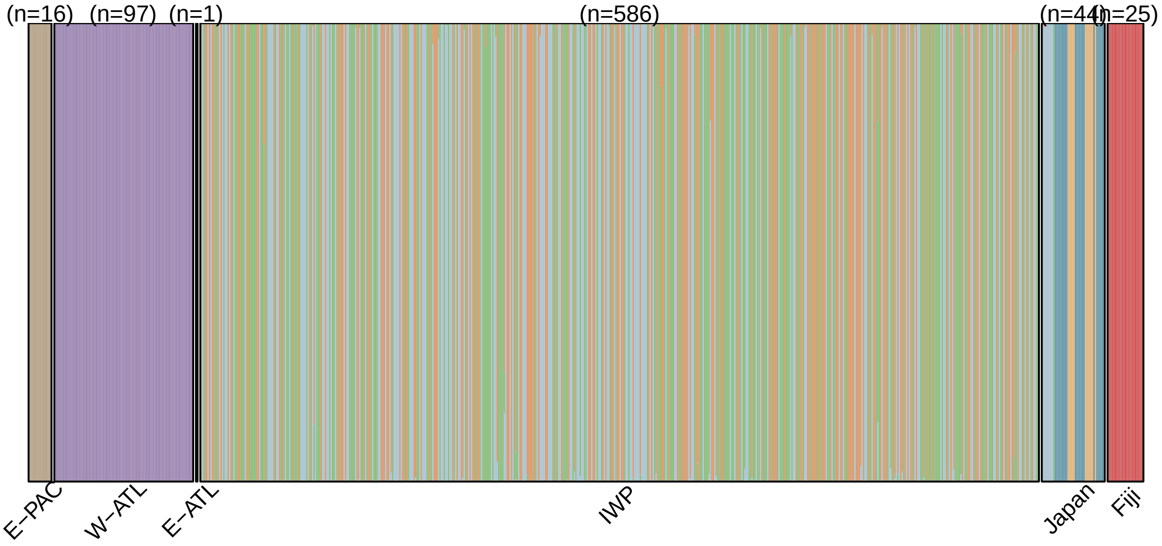


DAPC: all Bull Sharks − subset Australia
for K=9 & PC=209

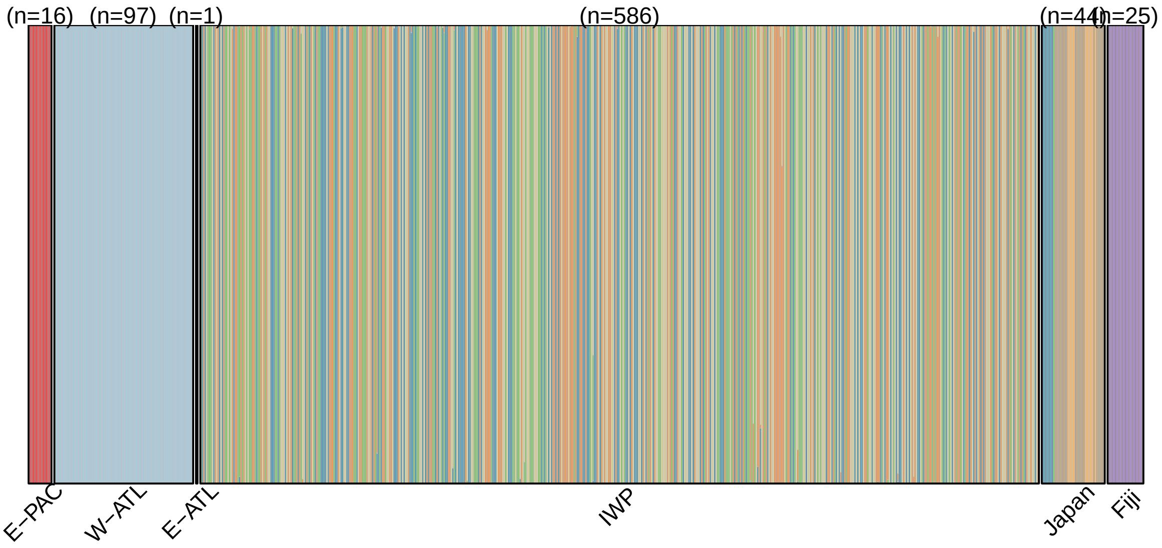

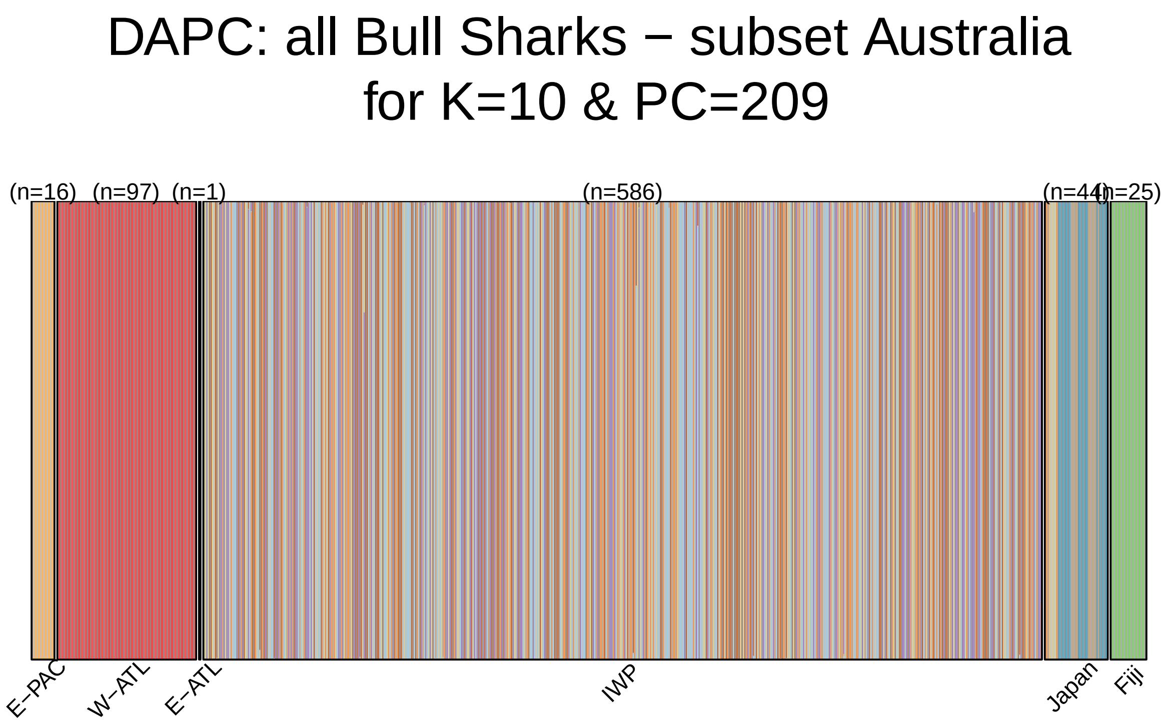


#### 8.9.4 DAPC scatterplot

```
load("DArTcap_ALL_DAPC.Rdata")

for (K in 3:10) {
  dapc <- get(dapc.all.object.names[K - 1])
  if (K == 2) {
    posi.leg <- "bottomright"
  } else {posi.leg <- "bottomleft"}
  dapc.plot <- ade4::scatter(dapc, grp = pop(BS.dartcap.gl), cex = 2, legend = TRUE,
                             col = colours.32,clabel = FALSE, posi.leg = posi.leg,
                             scree.pca = FALSE,posi.pca = "topleft", cleg = 0.5,
                             xax = 1, yax = 2, inset.solid = 0.3)
  dev.print(
    device = png,
    file = paste0("DArTcap_ALL_DAPC_scatterplot_K", K, ".png"),
    width = 30,
    height = 15,
    units = "cm",
    res = 300
  )
}
```

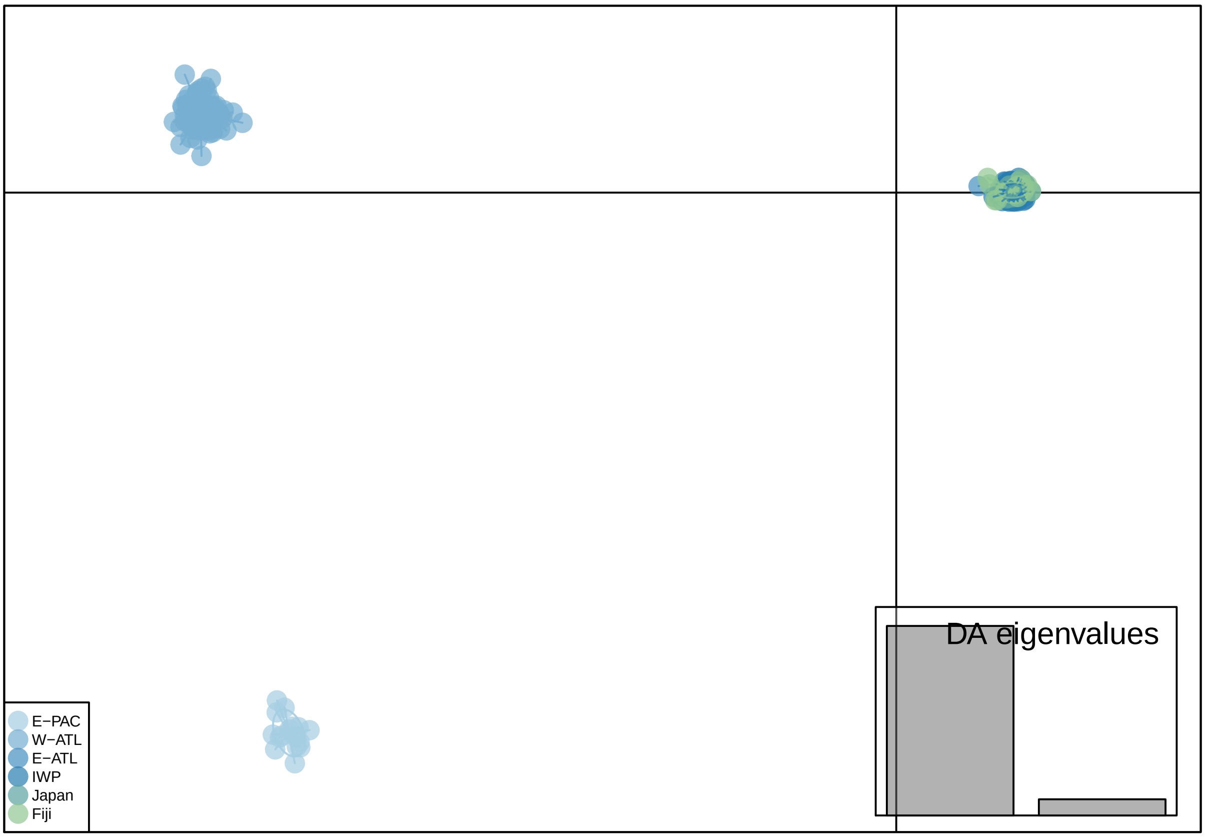
DA eigenvalues
E−PAC
W−ATL
E−ATL
IWP
Japan
Fiji

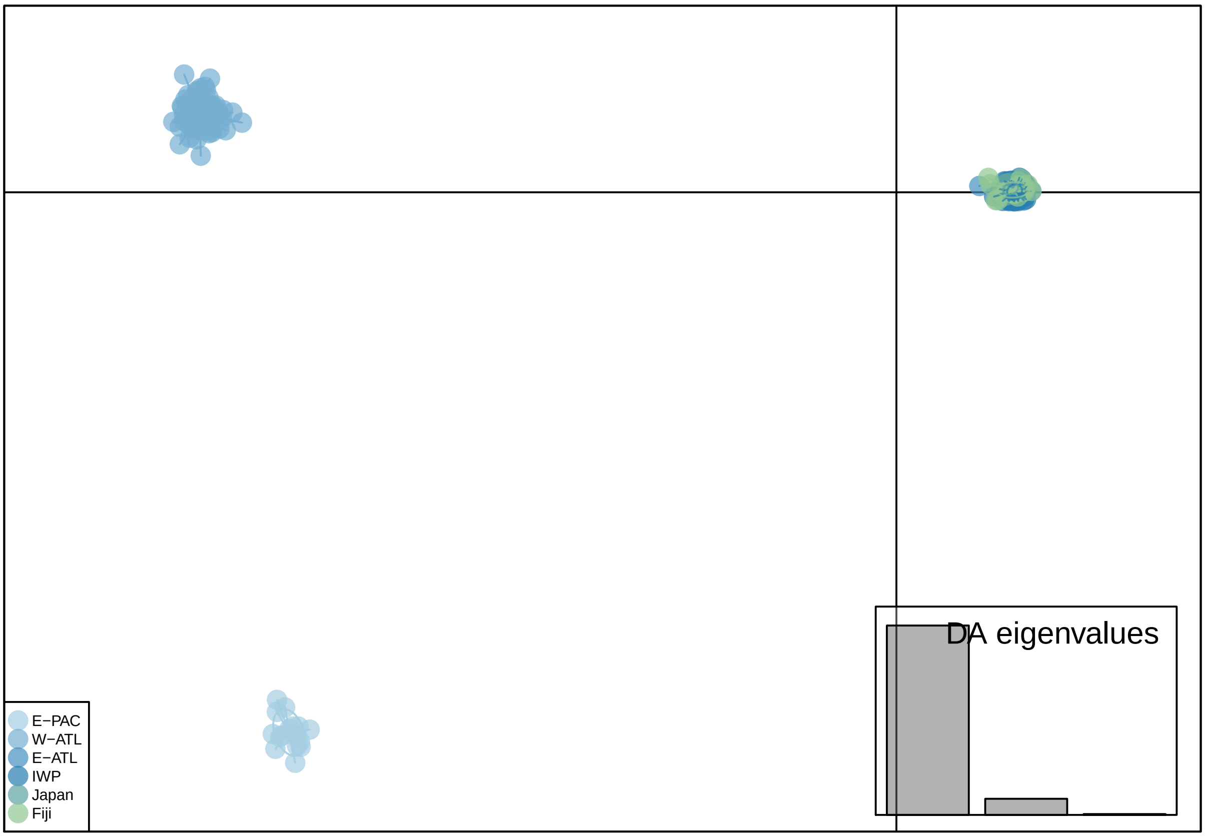
DA eigenvalues
E−PAC
W−ATL
E−ATL
IWP
Japan
Fiji

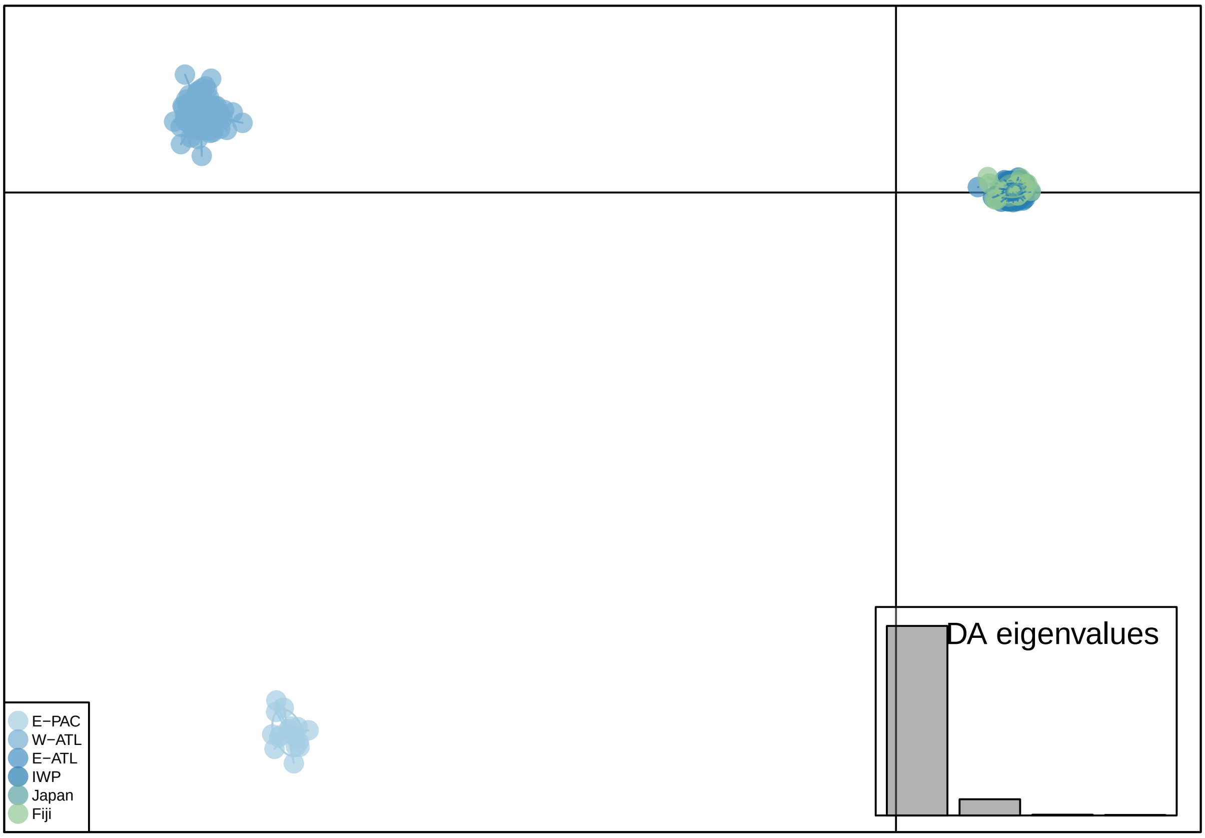
DA eigenvalues
E−PAC
W−ATL
E−ATL
IWP
Japan
Fiji

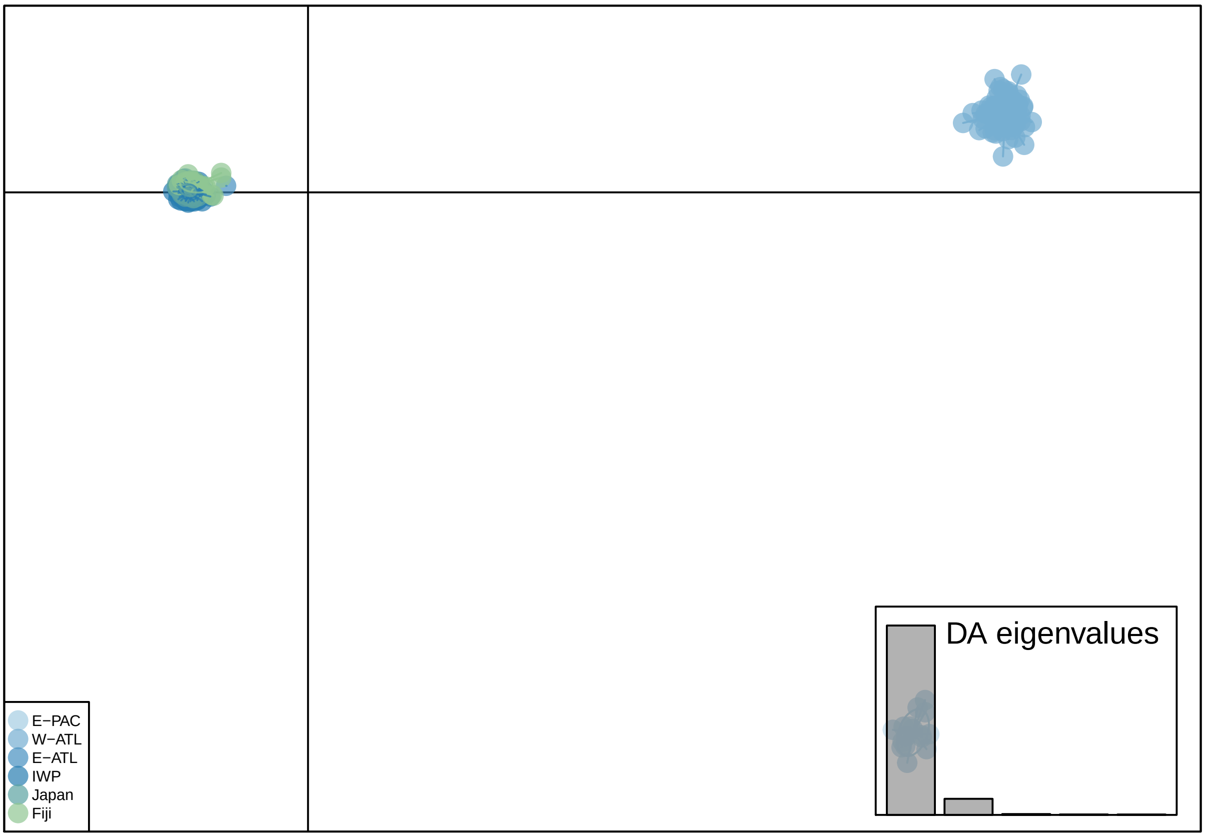
DA eigenvalues
E−PAC
W−ATL
E−ATL
IWP
Japan
Fiji

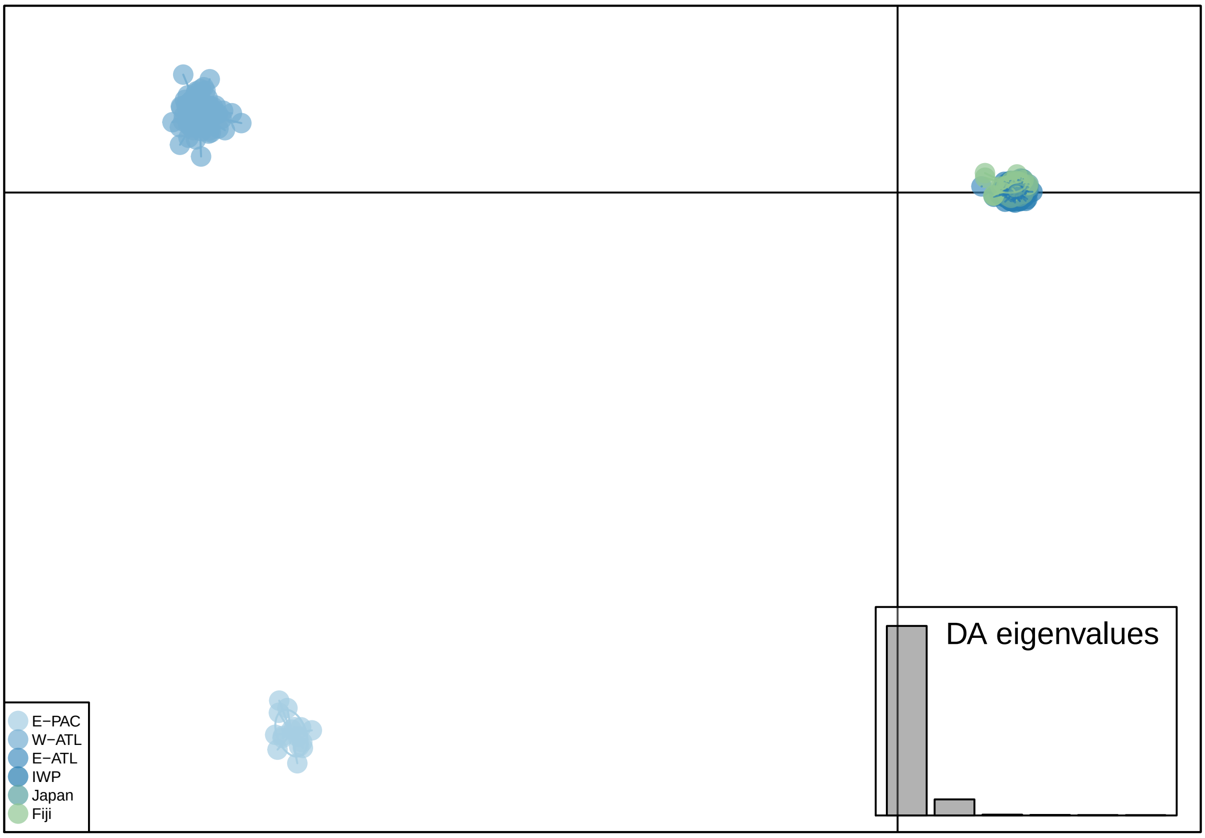
DA eigenvalues
E−PAC
W−ATL
E−ATL
IWP
Japan
Fiji

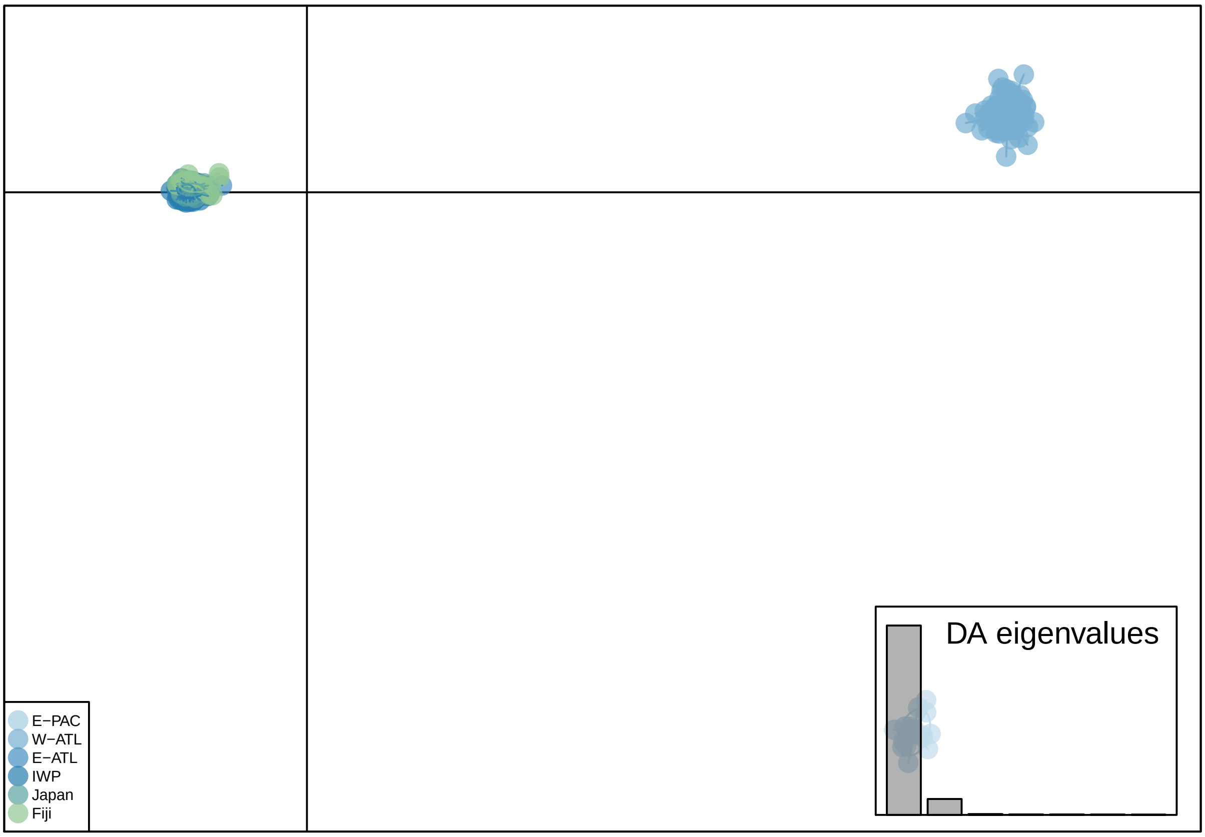
DA eigenvalues
E−PAC
W−ATL
E−ATL
IWP
Japan
Fiji

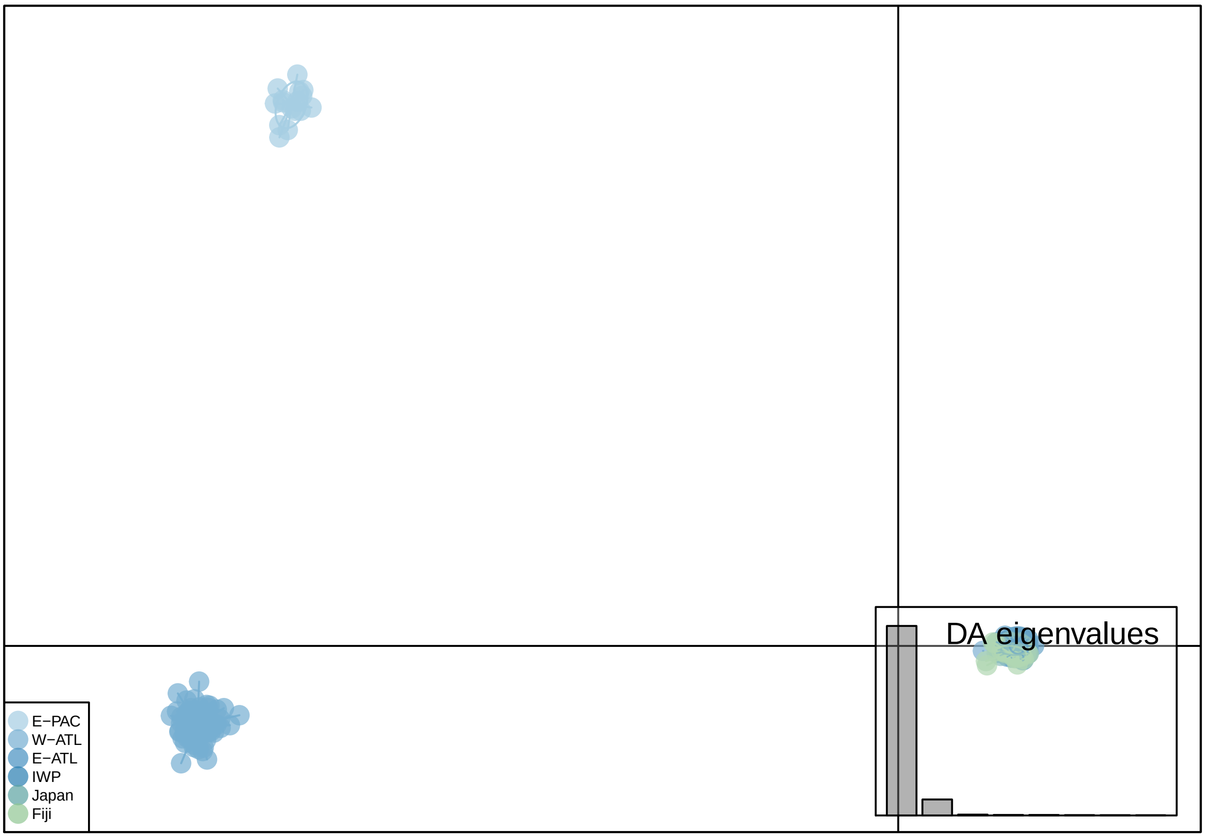


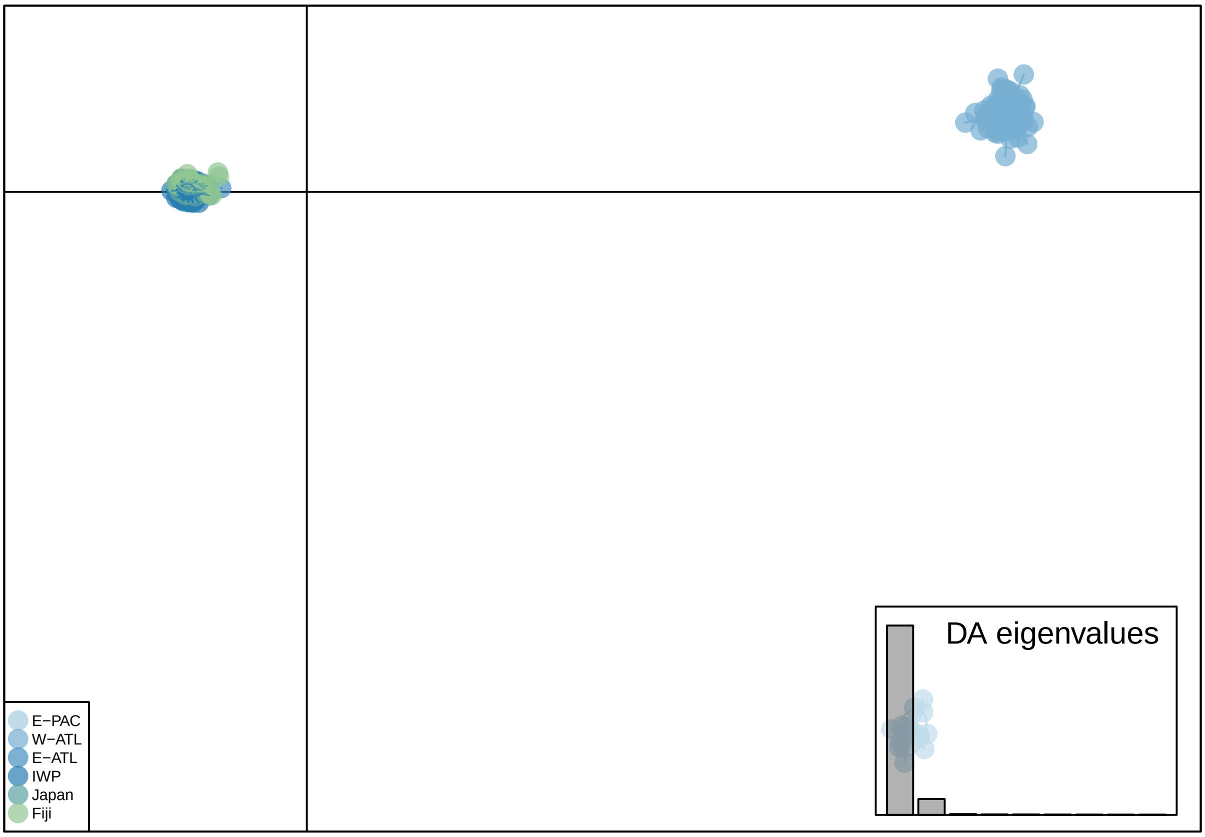


```
for (K in 2:10) {
  colour <- colours.prior[1:K]
  dapc <- get(dapc.all.object.names[K - 1])
  dapc.plot <- ade4::scatter(dapc, cex = 2, legend = TRUE, col = colour,
                             clabel = FALSE, posi.leg = "bottomleft",
```

```
                                    scree.pca = FALSE,  posi.pca = "topleft",
                                    cleg = 0.75, xax = 1, yax = 2, inset.solid = 0.3)
  dev.print(
    device = png,
    file = paste0("DArTcap_ALL_DAPC_scatterplot_pergroups_K",
                  K,".png"),
    width = 30,
    height = 15,
    units = "cm",
    res = 300
  )
}
```

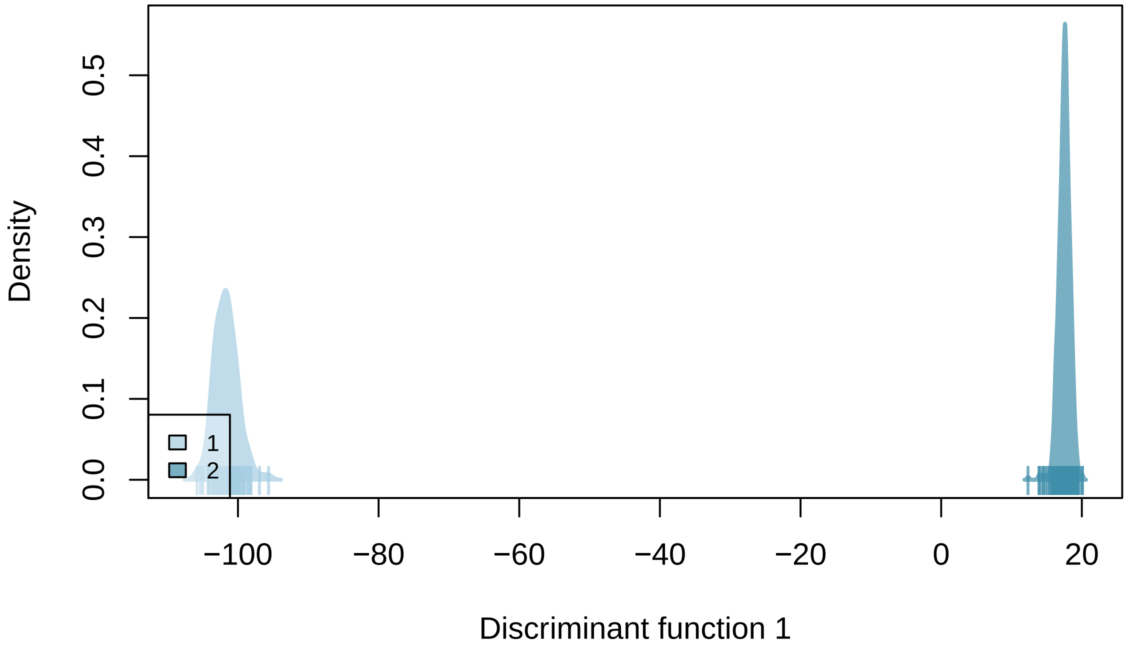

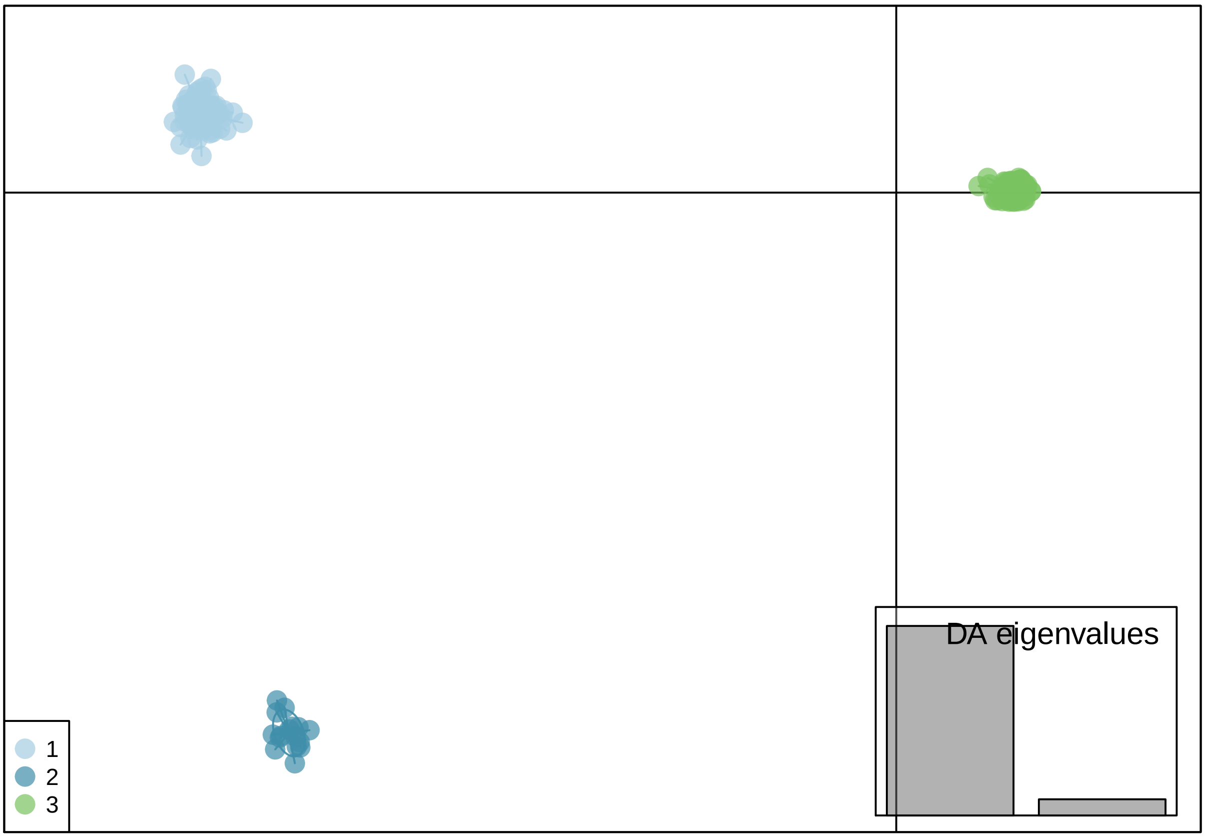
DA eigenvalues
1
2
3

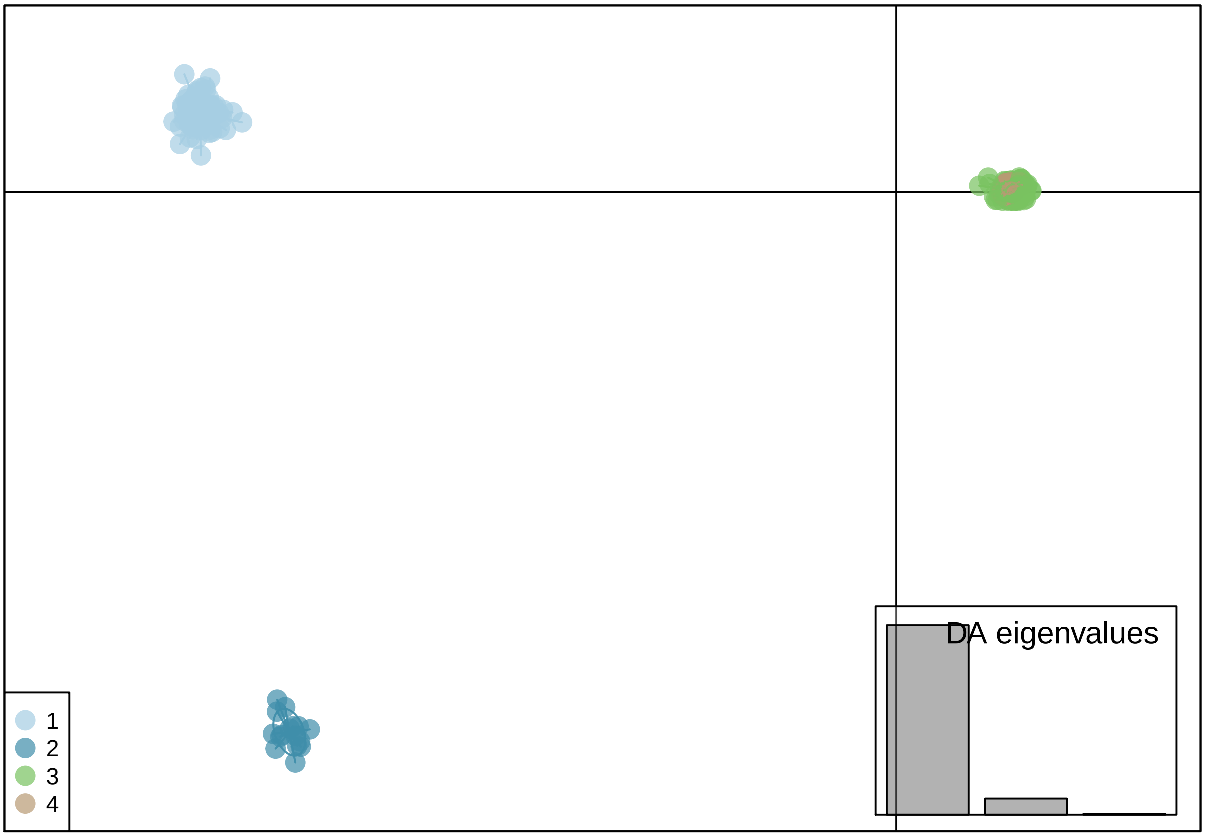
DA eigenvalues
1
2
3
4

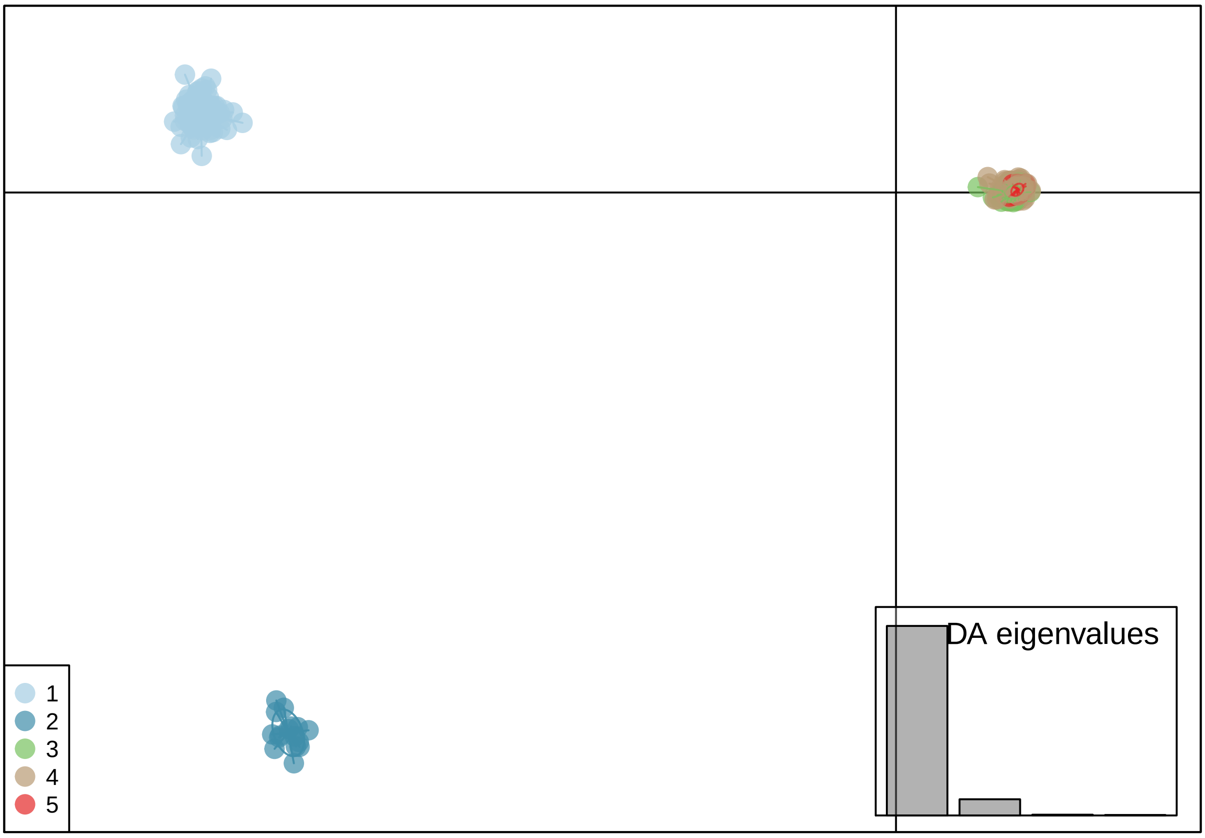
DA eigenvalues
1
2
3
4
5

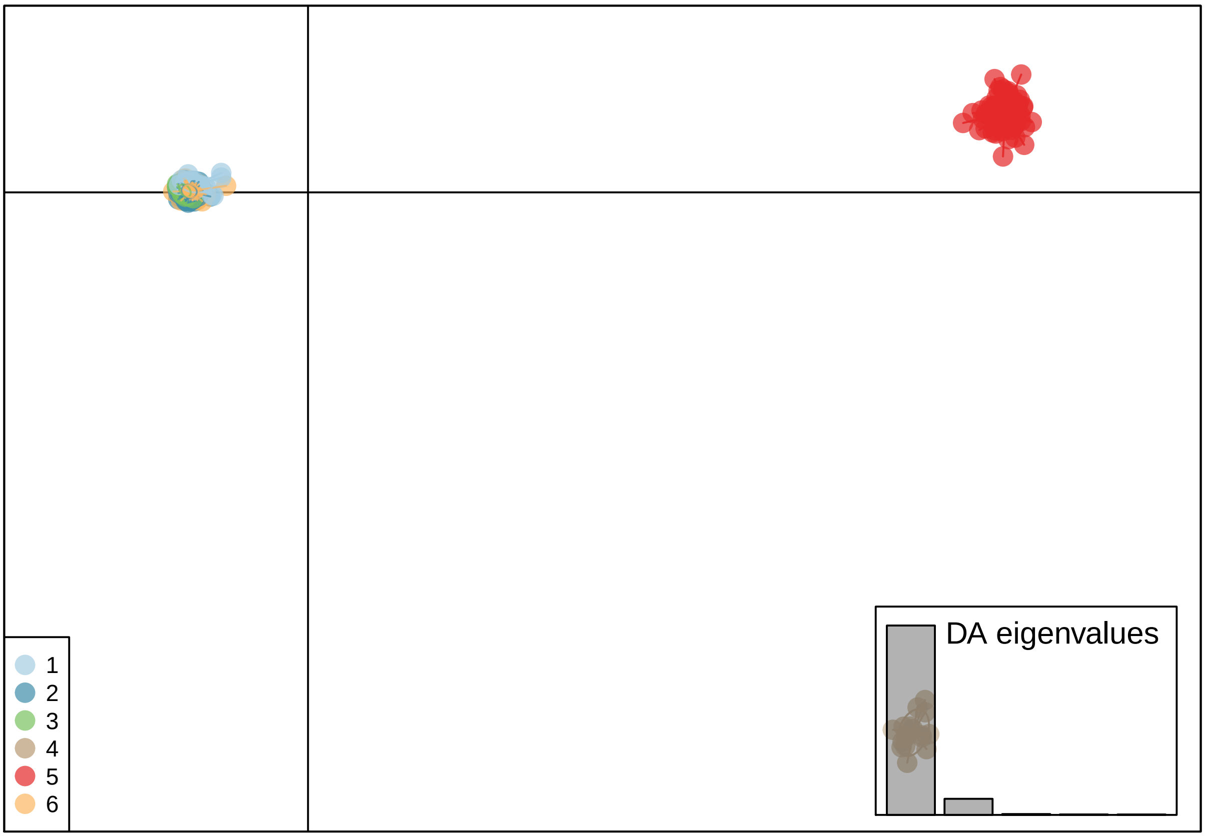
DA eigenvalues
1
2
3
4
5
6

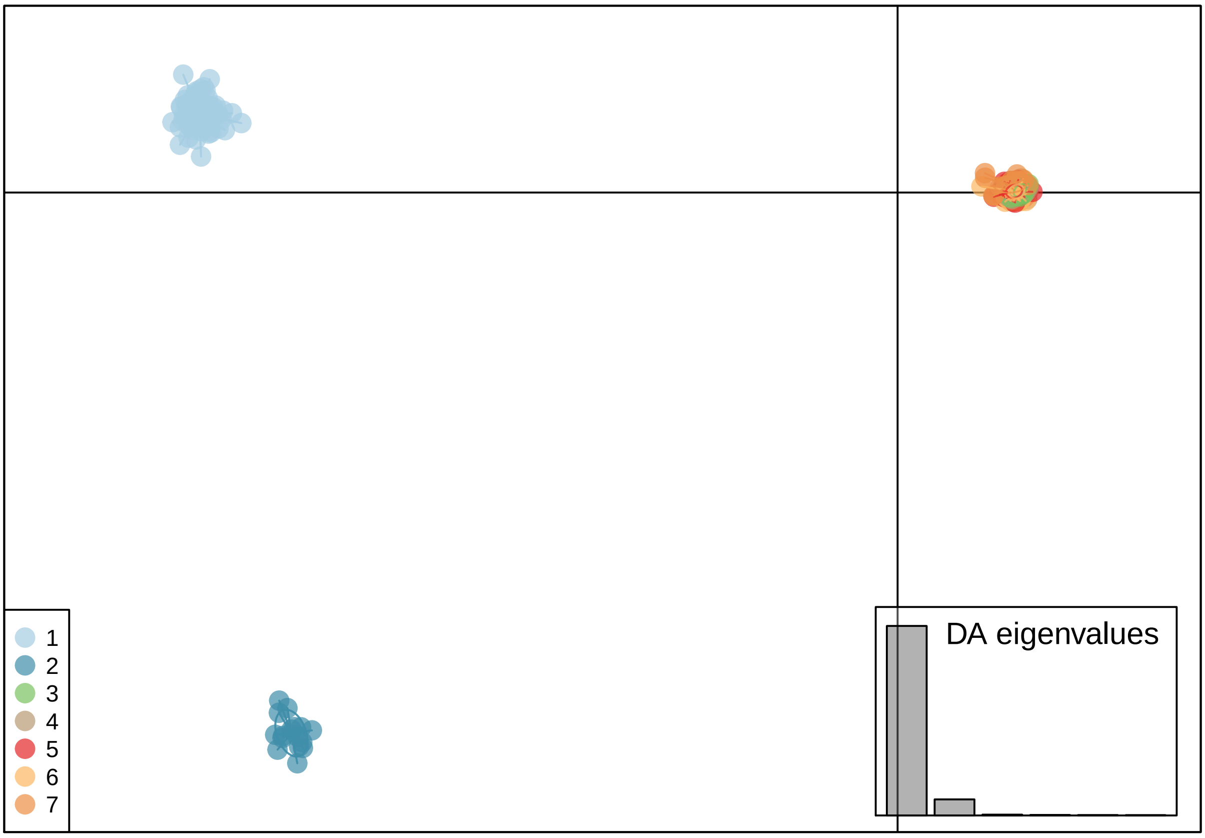
1
2
3
4
5
6
7
DA eigenvalues

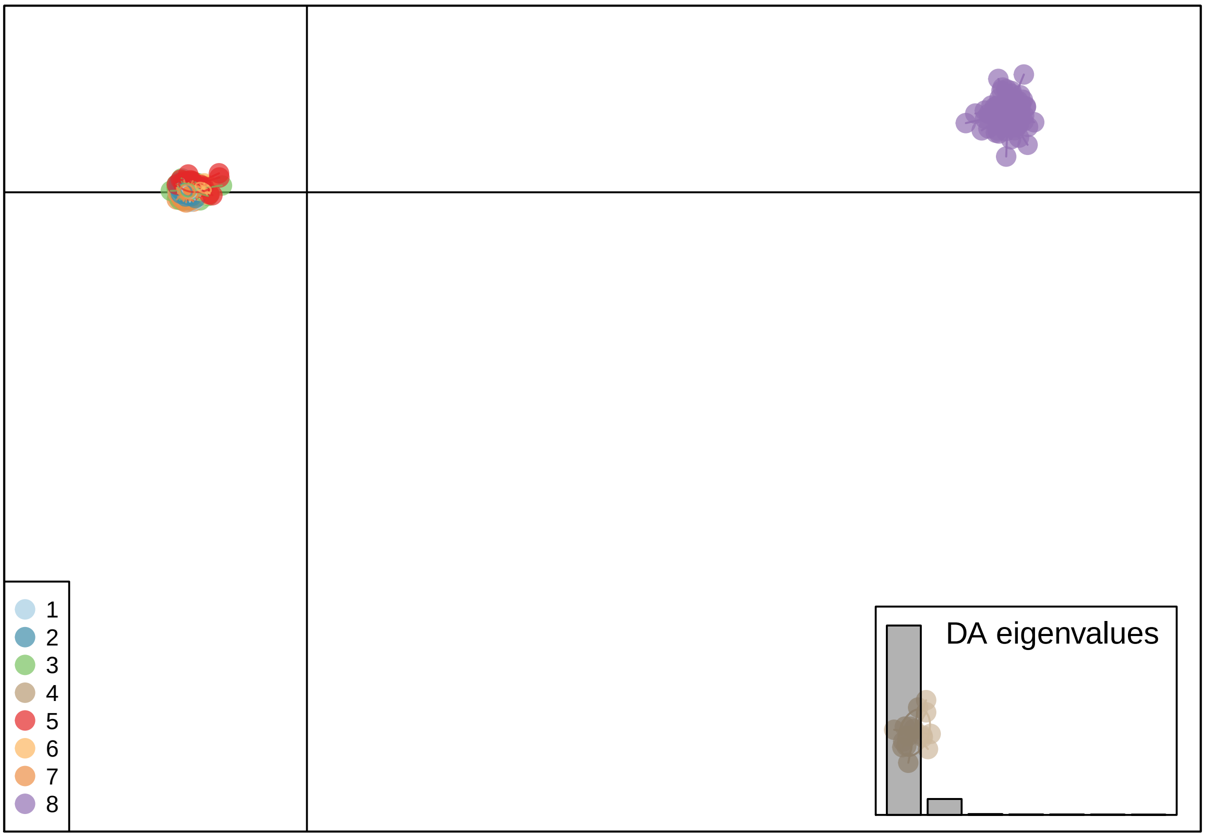
1
2
3
4
5
6
7
8
DA eigenvalues

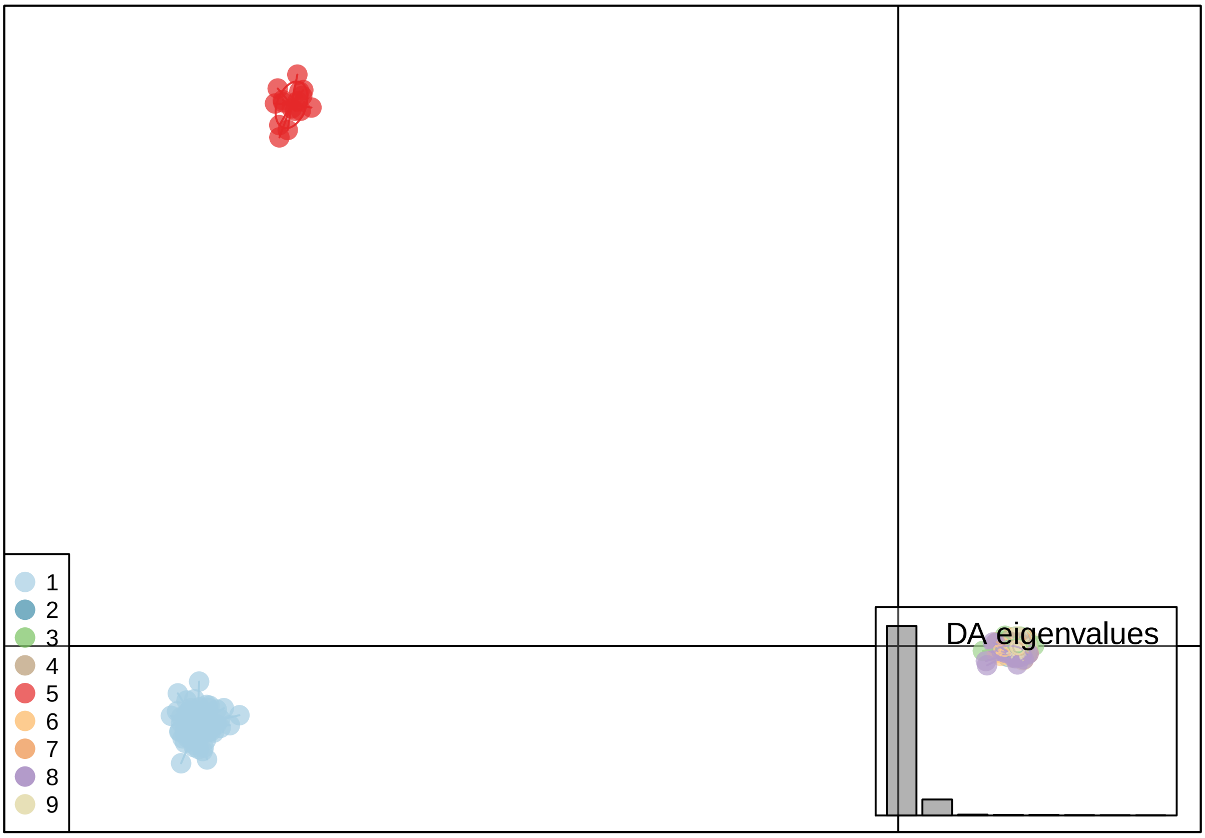


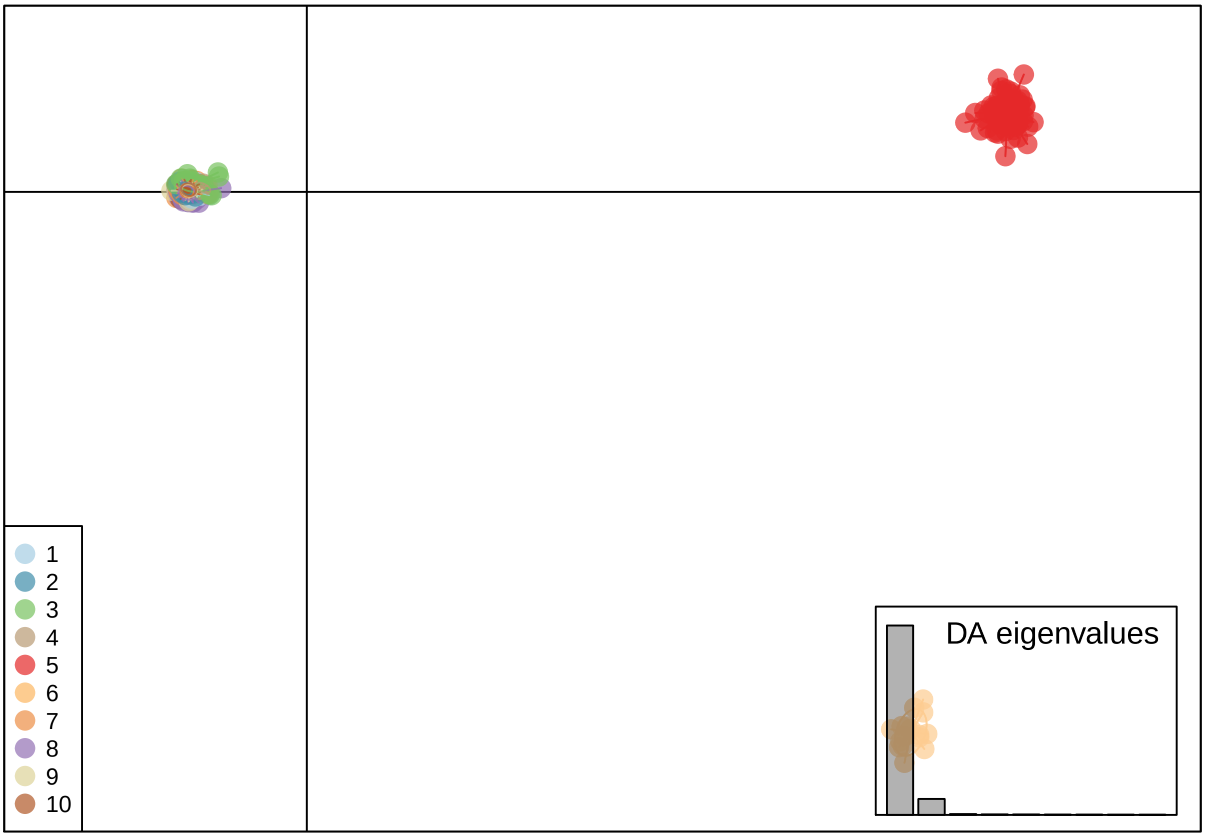


### 8.9.5 DAPC scatterplot - FLO

This code will *colour* individuals according to *sampling location* and **group** individuals according to DAPC **posterior membership**.

```r
load("DArTcap_ALL_DAPC.Rdata")

for (K in 3:10) {
  dapc <- get(dapc.all.object.names[K - 1])
  x <- dapc$ind.coord[,1]
  y <- dapc$ind.coord[,2]
  group <- paste("Group", as.numeric(dapc$assign))
  pop <- BS.dartcap.gl$pop
  df <- data.frame(x, y, group, pop)
  colnames(df) <- c("x","y","group","pop")
  gg <- merge(df,aggregate(cbind(mean.x = x,mean.y = y)~group, df, mean),
              by = "group")

  plot <- ggplot2::ggplot(gg, ggplot2::aes(x,y,color = pop)) +
    ggplot2::geom_point(size = 5) +
    ggplot2::geom_point(ggplot2::aes(x = mean.x, y = mean.y),size = 5,
                        shape = 17, color = "black") +
    ggplot2::geom_segment(ggplot2::aes(x = mean.x, y = mean.y, xend = x,
                                       yend = y)) +
    ggplot2::scale_colour_manual(values = colours.32) +
    ggplot2::scale_fill_manual(values = colours.32) +
    ggplot2::geom_hline(yintercept = 0) +
    ggplot2::geom_vline(xintercept = 0) +
    ggplot2::labs(subtitle = "",
        y = "PC2",
        x = "PC1",
        title = paste0("K=",K," & PC=",PC, sep = ""),
        caption = "") +
    ggplot2::theme_minimal() +
    ggplot2::theme(
      axis.text = ggplot2::element_text(size = 5),
      axis.title.x = ggplot2::element_text(size = 10),
      axis.title.y = ggplot2::element_text(size = 10),
      legend.text = ggplot2::element_text(size = 7))
  print(plot)
  ggplot2::ggsave(plot, filename = paste0("DArTcap_ALL_DAPC_scatterplot2_K", K, ".png"),
                  width = 30, height = 15,units = "cm")
}
```

K=3 & PC=209

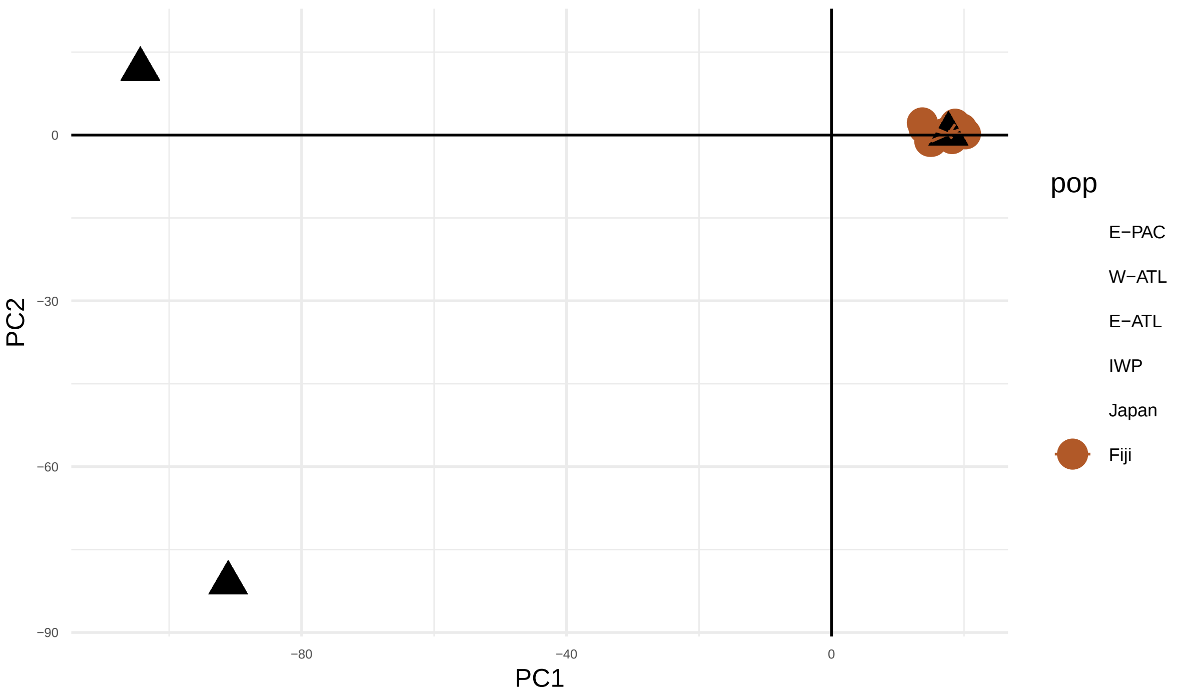


K=4 & PC=209

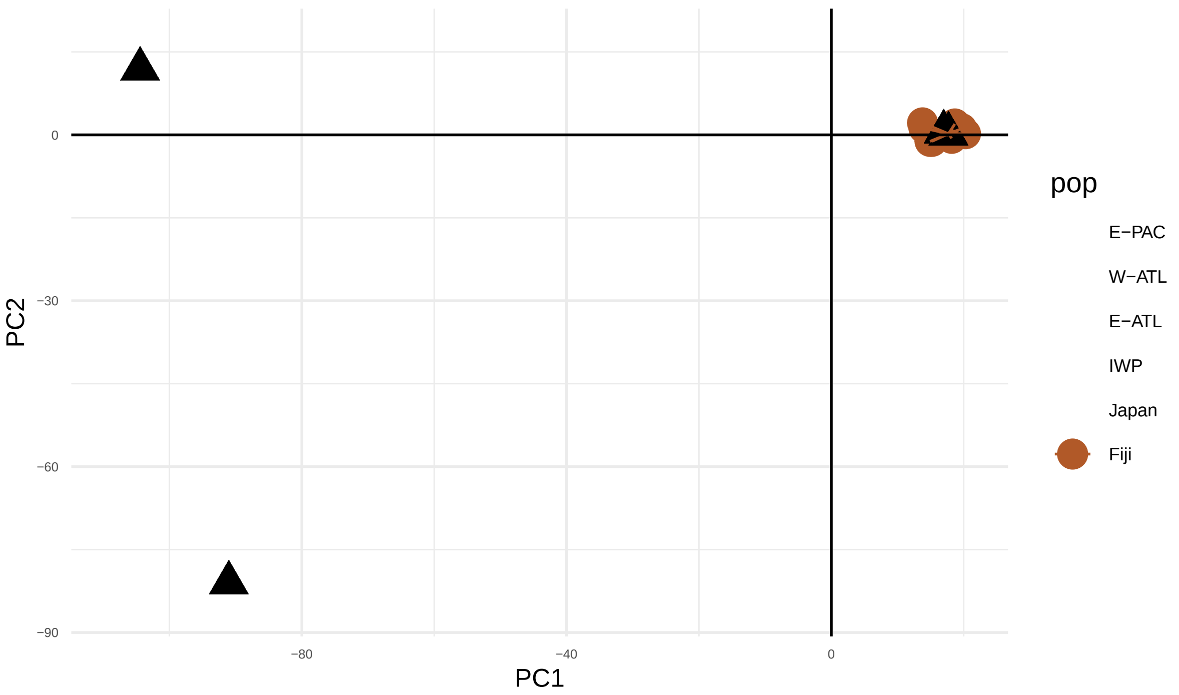

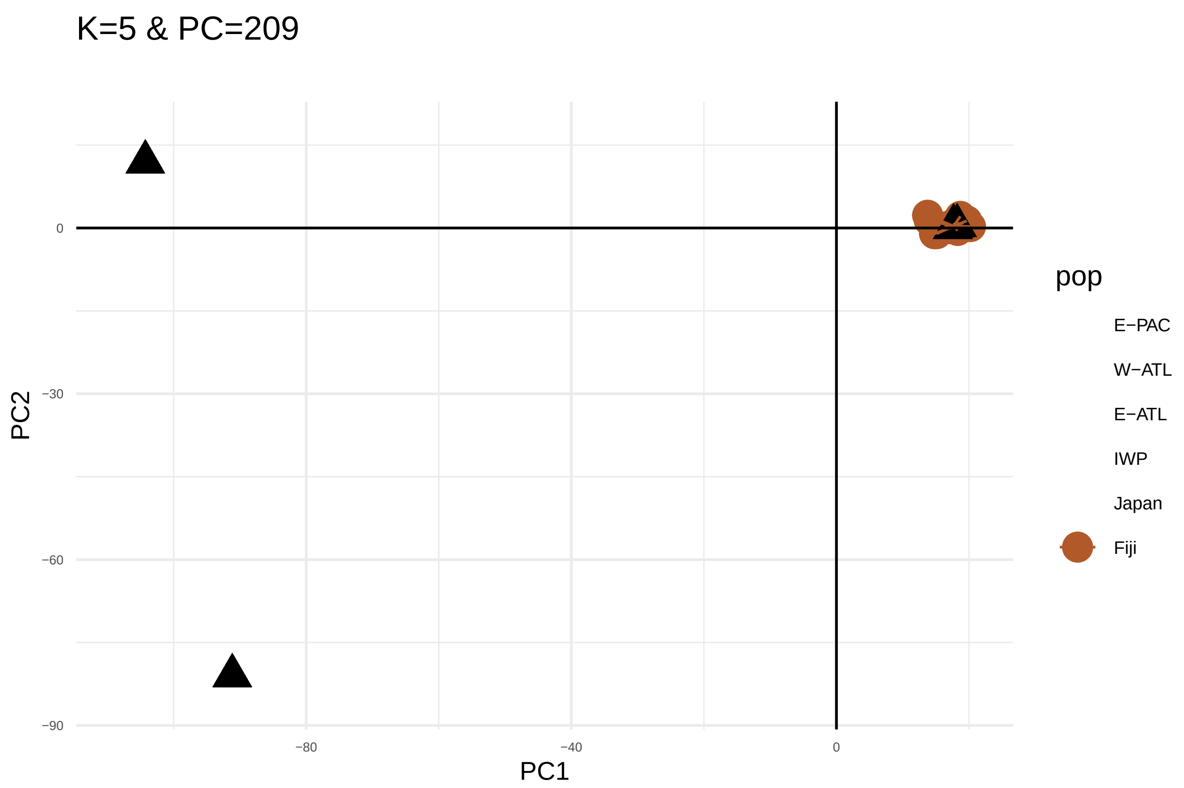
K=5 & PC=209
0
−30
−60
−90
PC2
−80
−40
0
PC1
pop
E−PAC
W−ATL
E−ATL
IWP
Japan
Fiji

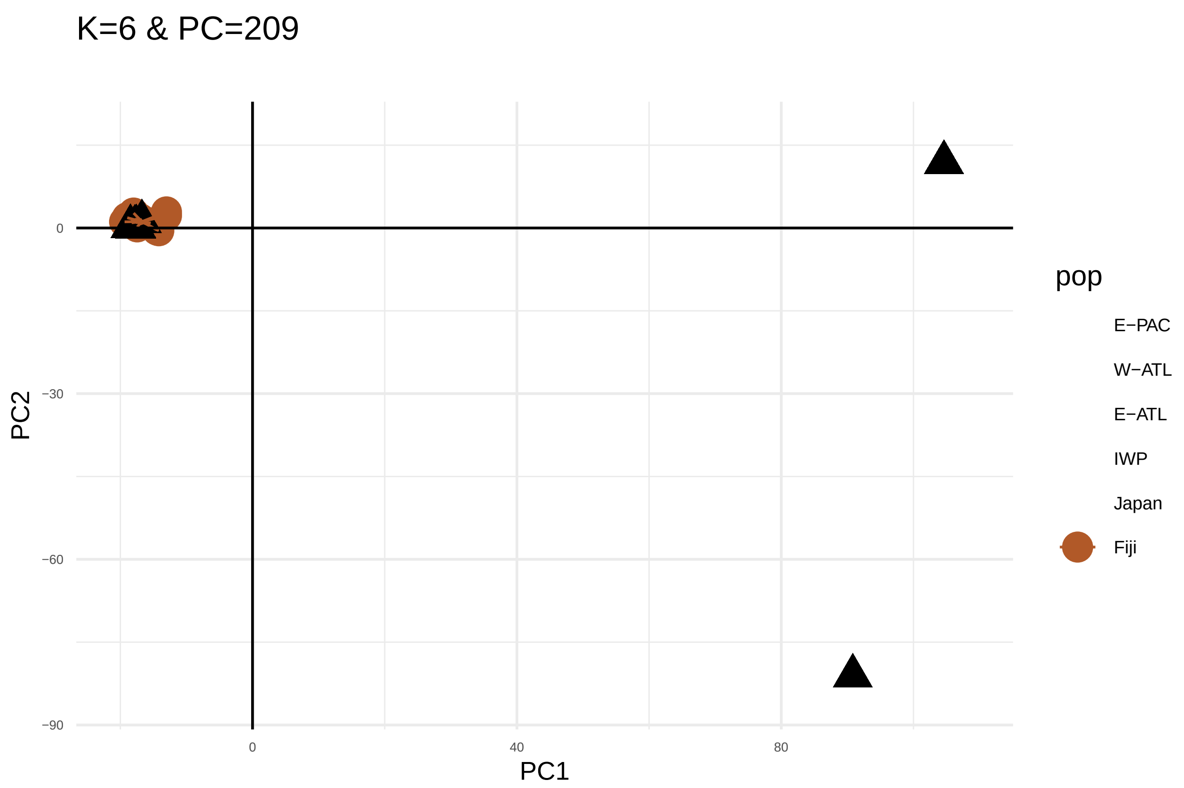
K=6 & PC=209
0
−30
−60
−90
PC2
0
40
80
PC1
pop
E−PAC
W−ATL
E−ATL
IWP
Japan
Fiji

K=7 & PC=209

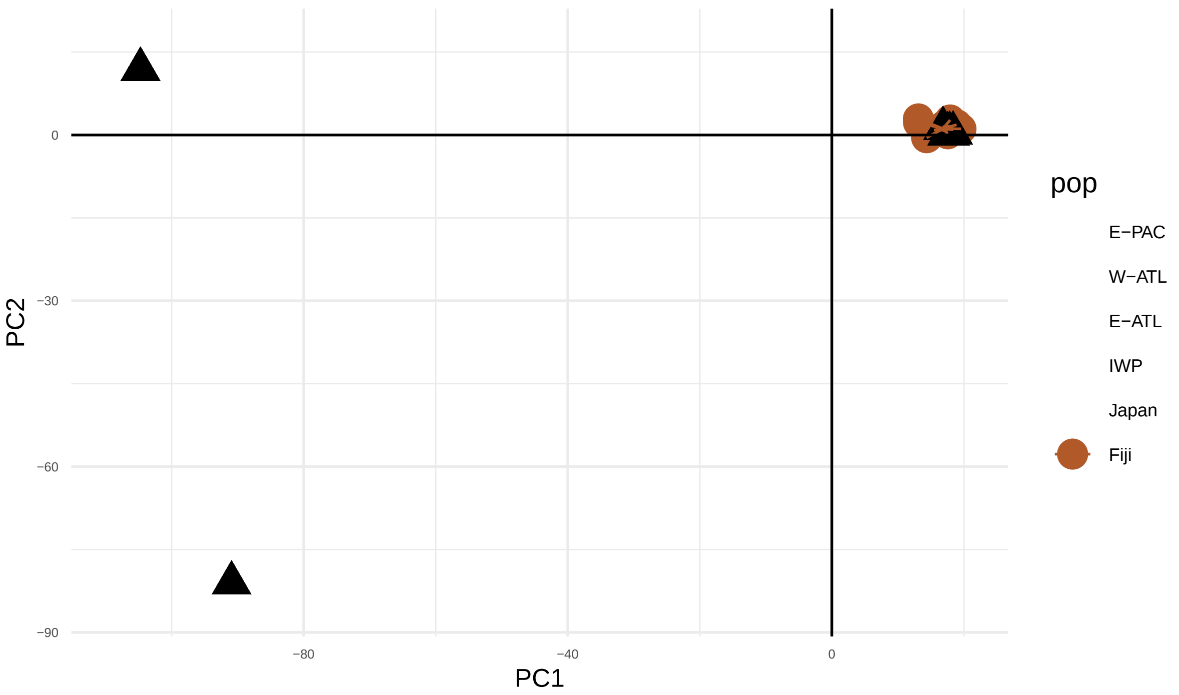


K=8 & PC=209

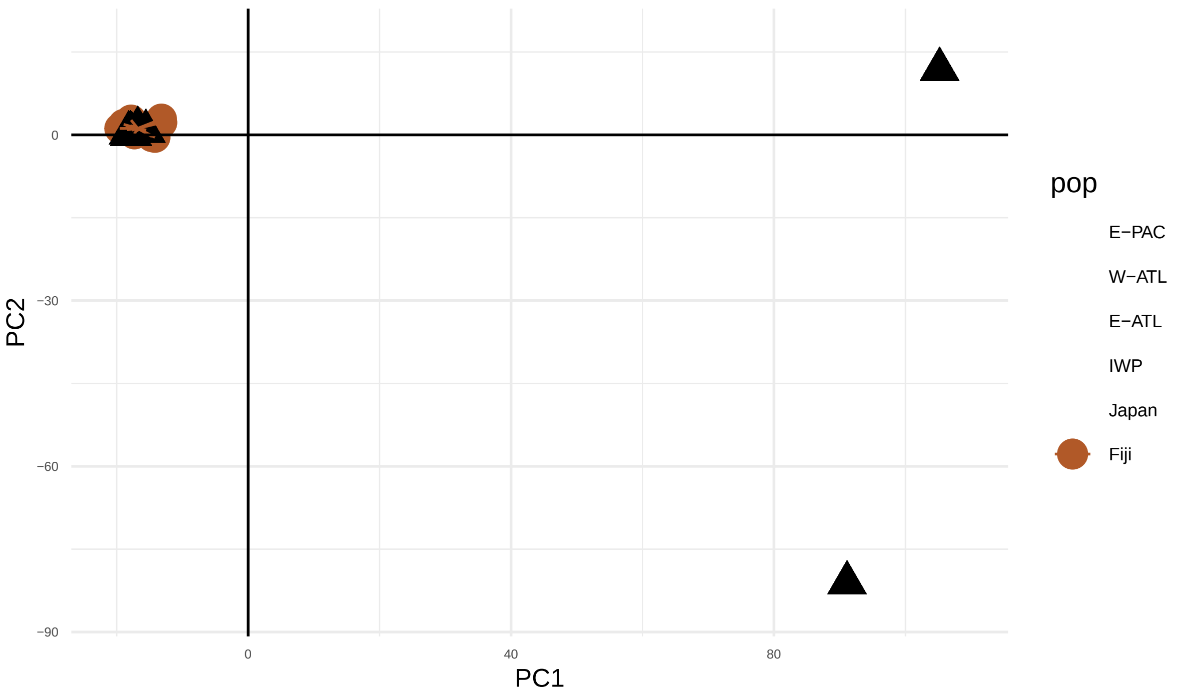

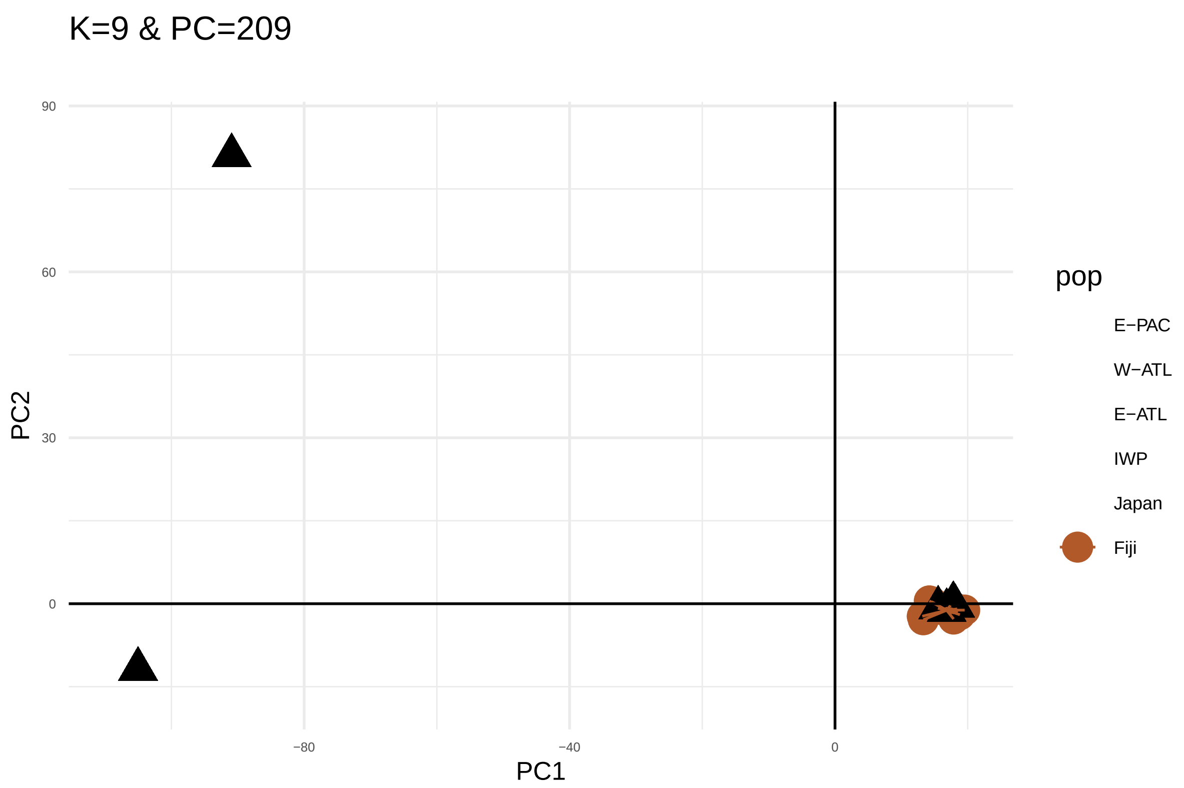


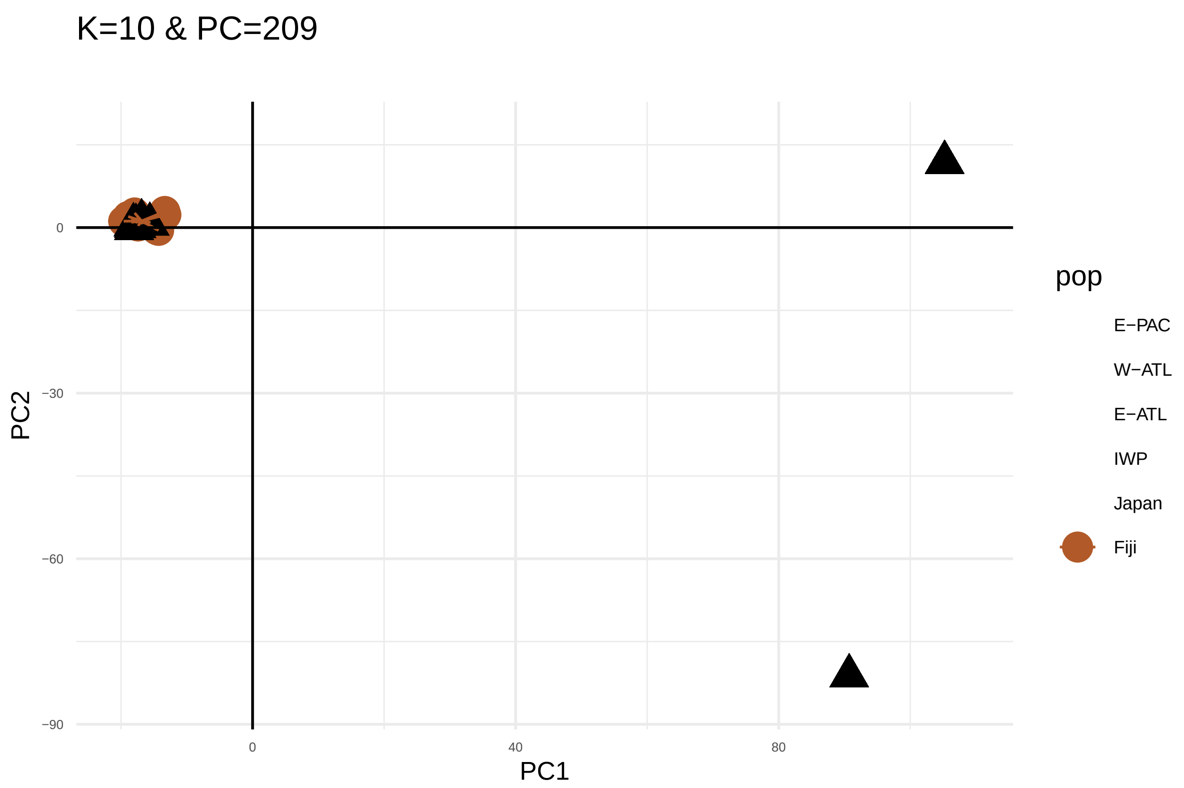


## 8.10 Provenance assignment

Using the package assignPOP to estimate the assignment success of the DArTcap data

### 8.10.1 Per Site

```r
pop.levels <- c("Costa Rica" , "Brazil", "Caribbean Sea", "Gulf of Mexico",
                "Western North Atlantic", "Sierra Leone", "South Africa",
                "Mozambique", "Reunion" , "Seychelles", "Arabian Sea",
                "Sri Lanka", "Thailand", "Indonesia", "Papua New Guinea" ,
                "Australia", "Japan", "Fiji")
BS.dartcap.gl$pop <- factor(BS.dartcap.gl$other$ind.metrics$Site,
                            levels = pop.levels)

gl2gpop(BS.dartcap.gl[!BS.dartcap.gl$pop %in% c("Caribbean Sea","Sierra Leone",
                                                "Indonesia", "Thailand"),],
        filename = "DArTcap_ALL_assign_Site_genepop.txt") #too few individuals

shortnames <- c("COR","BRZ", "GOM", "WNA", "SAF", "MOZ", "RUN" , "SEY",
                "ARS", "SRL", "PNG" , "AUS", "JAP", "FIJ")
gpop <- assignPOP::read.Genepop('DArTcap_ALL_assign_Site_genepop.txt',
  pop.names = shortnames, haploid = FALSE,  pos = 1)

assignPOP::assign.MC(
  x = gpop, train.inds = c(0.5, 0.9),train.loci = c(0.05, 0.1, 0.5, 1),
  loci.sample = 'fst',iterations = 1000, dir = 'AssignPOP_MC_Sites/', scaled = FALSE,
  pca.method = 'mixed', pca.PCs = 'kaiser-guttman',pca.loadings = F,
  model = 'svm', svm.kernel = 'linear',svm.cost = 1, ntree = 50,
  multiprocess = TRUE, processors = 3, skipQ = FALSE)

assignPOP::assign.kfold(
  x = gpop, k.fold = 8, train.loci = c(0.05, 0.1, 0.5, 1),loci.sample = 'fst',
  dir = 'AssignPOP_Kfold_Sites/', scaled = FALSE, pca.method = 'mixed',
  pca.PCs = 'kaiser-guttman', pca.loadings = F, model = 'svm',
  svm.kernel = 'linear',svm.cost = 1, ntree = 50, multiprocess = TRUE, processors = 3,
  skipQ =FALSE)
```

```r
shortnames <- c("COR","BRZ", "GOM", "WNA", "SAF", "MOZ", "RUN" , "SEY",
                "ARS", "SRL", "PNG" , "AUS", "JAP", "FIJ")
df1 <- assignPOP::accuracy.MC(dir = "AssignPOP_MC_Sites/")
MC.assign <- assignPOP::accuracy.plot(df1, pop = c("all",shortnames))
print(MC.assign)
```

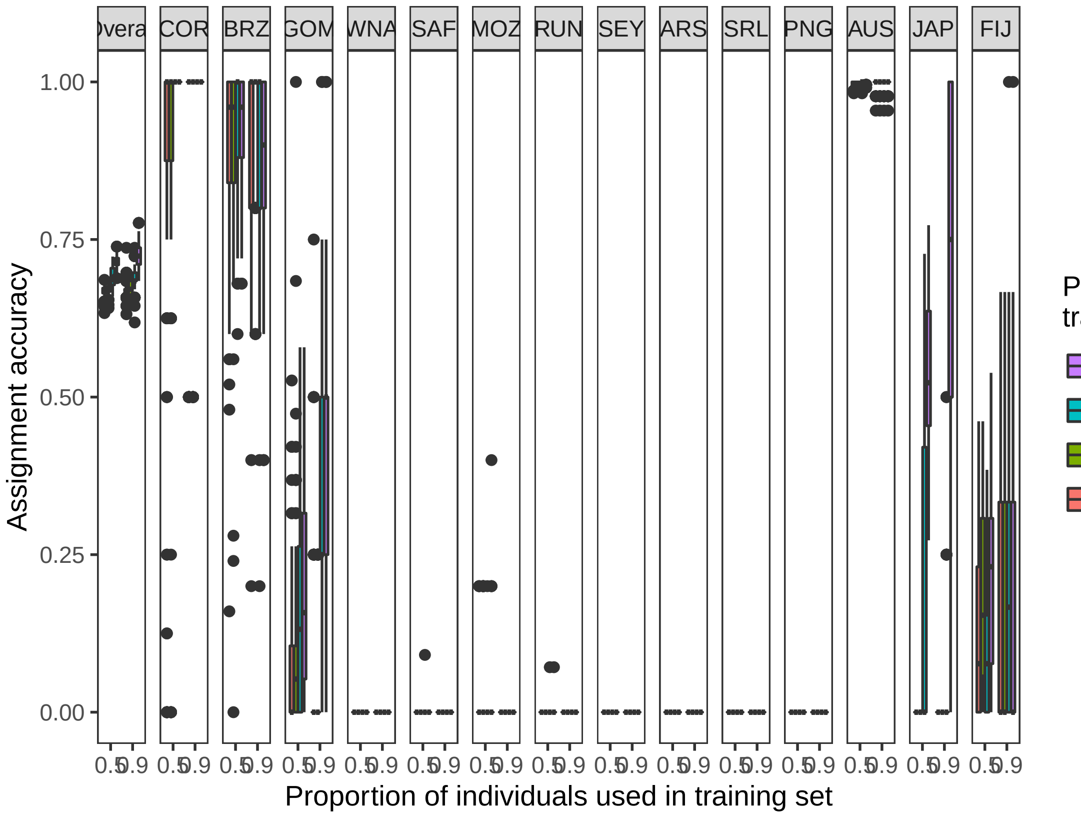


#### 8.10.1.1 AssignPOP

```
ggplot2::ggsave(MC.assign, filename = "DArTcap_ALL_assignPOP_MC_Sites.png",
                width = 30, height = 15,units = "cm")

df2 <- assignPOP::accuracy.kfold(dir = "AssignPOP_Kfold_Sites/")
kfold.assign <- assignPOP::accuracy.plot(df2, pop = c("all",shortnames))
print(kfold.assign)
```

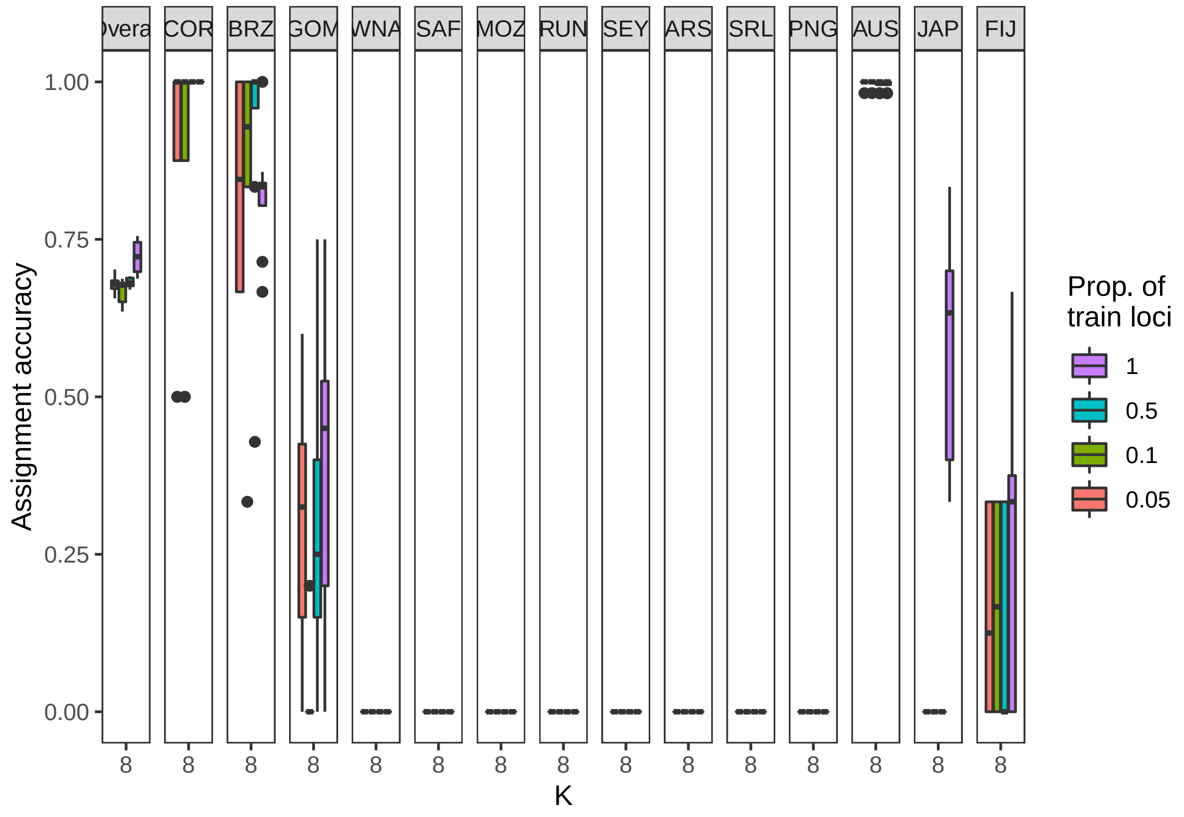


```
ggplot2::ggsave(kfold.assign, filename = "DArTcap_ALL_assignPOP_Kfold_Sites.png",
                width = 30, height = 15,units = "cm")


names(colours.14) <- shortnames
# memb.plot <- assignPOP::membership.plot(dir = "AssignPOP_Kfold_Sites/",
#                              style = 1,
#                              non.genetic = TRUE)
# #choose proportion training loci = 1
# save(memb.plot, file = "memb.plot1.Rdata")
load("memb.plot1.Rdata")
memb.plot$data$origin.pop <- factor(memb.plot$data$origin.pop,
                                    levels = shortnames)
memb.plot + ggplot2::scale_fill_manual(values = colours.14) +
  ggplot2::theme(axis.text.x = ggplot2::element_blank())
```

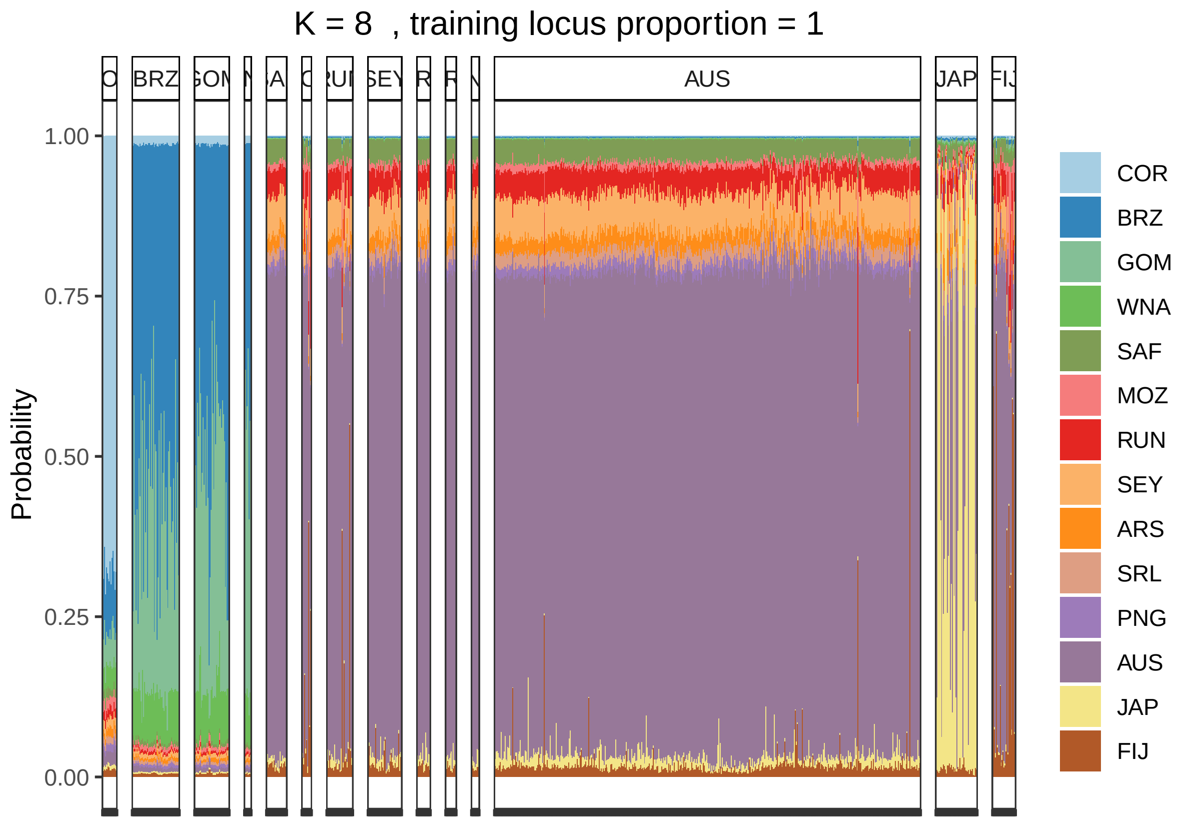


```r
ggplot2::ggsave(memb.plot,
                filename = "DArTcap_ALL_assignPOP_Kfold_membership_Sites.png",
                width = 30, height = 15,units = "cm")


df <- data.frame(
 ind = as.character(memb.plot$data$Ind.ID)[
    as.character(memb.plot$data$fold_n) == "fold_8"],
  pop = as.character(memb.plot$data$origin.pop)[
    as.character(memb.plot$data$fold_n) == "fold_8"],
  memb = as.character(memb.plot$data$pred.pop)[
    as.character(memb.plot$data$fold_n) == "fold_8"], stringsAsFactors = FALSE)

summary(df$pop[df$pop != df$memb]) # not a great assignment success
```

```
##
##   Correct assignment rates were estimated!!
##   A total of 800 assignment tests for 14 pops.
##   Results were also saved in a 'Rate_of_800_tests_14_pops.txt' file in the directory.
##   Correct assignment rates were estimated!!
##   A total of 32 assignment tests for 14 pops.
##   Results were also saved in a 'Rate_of_32_tests_14_pops.txt' file in the directory.   the 10 mos
## GOM RUN SEY SAF ARS FIJ JAP AUS BRZ MOZ
##  70  56  56  42  28  28  28  14  14  14
```

```r
pop.levels1 <- c("Costa Rica" , "Brazil","Caribbean Sea","Gulf of Mexico",
                 "Western North Atlantic", "Sierra Leone", "South Africa",
                 "Mozambique", "Reunion" , "Seychelles", "Arabian Sea",
                 "Sri Lanka", "Thailand", "Indonesia", "Papua New Guinea" ,
                 "Fitzroy River", "Daly River", "Adelaide River","Darwin Coastal",
```

```
                 "South Alligator River", "East Alligator River","Blue mud Bay",
                 "Roper River","Towns River","Unknown","Wenlock River",
                 "Trinity inlet","Clarence River", "Sydney Harbour", "Okinawa",
                 "Urauchi River", "Fiji")
pop.levels2 <- c("Costa Rica" , "Brazil","Caribbean Sea", "Gulf of Mexico",
                 "Western North Atlantic", "Sierra Leone", "South Africa",
                 "Mozambique", "Reunion" , "Seychelles", "Arabian Sea", "Sri Lanka",
                 "Thailand", "Indonesia", "Papua New Guinea" , "Australia",
                 "Japan", "Fiji")

BS.dartcap.rubias2 <- BS.dartcap.rubias
# BS.dartcap.rubias2$indiv == BS.dartcap.gt@schemes$INDIVIDUALS

BS.dartcap.rubias2$collection <- factor(BS.dartcap.gt@schemes$Site2,
                                        levels = pop.levels1)
BS.dartcap.rubias2$repunit <- factor(BS.dartcap.gt@schemes$Site,
                                     levels = pop.levels2)
BS.dartcap.rubias2 <- BS.dartcap.rubias2[order(BS.dartcap.rubias2$repunit,
                                               BS.dartcap.rubias2$collection),]


BS.dartcap.rubias2 %<>%
  dplyr::mutate_all(as.character)

BS_sims <- rubias::assess_reference_loo(reference = BS.dartcap.rubias2,
                    gen_start_col = 5,
                    reps = 5,
                    mixsize = 200,
                    return_indiv_posteriors = TRUE,
                    resampling_unit = "gene_copies")

repu_pofzs <- BS_sims$indiv_posteriors %>%
  dplyr::filter(repunit == simulated_repunit) %>%
  dplyr::group_by(iter, indiv, simulated_collection, repunit) %>%
  dplyr::summarise(repu_PofZ = sum(PofZ)) %>%
  dplyr::ungroup() %>%
  dplyr::arrange(repunit, simulated_collection) %>%
  dplyr::mutate(simulated_collection = factor(simulated_collection,
                                              levels = unique(simulated_collection)))

num_simmed <- BS_sims$indiv_posteriors %>%
  dplyr::group_by(iter, indiv) %>%
  dplyr::slice(1) %>%
  dplyr::ungroup() %>%
  dplyr::count(simulated_collection)

num_simmed <- num_simmed[order(factor(num_simmed$simulated_collection,
                                      levels = pop.levels1)),]
repu_pofzs$repunit <- factor(repu_pofzs$repunit, levels = pop.levels2)
repu_pofzs$simulated_collection <- factor(repu_pofzs$simulated_collection,
                                          levels = pop.levels1)
repu_pofzs <- repu_pofzs[order(repu_pofzs$repunit, repu_pofzs$simulated_collection),]

plot <- ggplot2::ggplot(repu_pofzs, ggplot2::aes(x = simulated_collection,
                                                 y = repu_PofZ)) +
  ggplot2::geom_boxplot(ggplot2::aes(colour = repunit)) +
  ggplot2::scale_color_manual(values = colours.18) +
  ggplot2::geom_text(data = num_simmed, mapping = aes(y = 1.025, label = n),
```

```r
                        angle = 90, hjust = 0, vjust = 0.5, size = 3) +
  ggplot2::labs(subtitle = "",
         y = "Assignment success",
         x = "Collection site") +
  ggplot2::theme(axis.text.x = ggplot2::element_text(angle = 90, hjust = 1,
                                                       size = 9, vjust = 0.5)) +
  ggplot2::ylim(c(NA, 1.05))
print(plot)
```

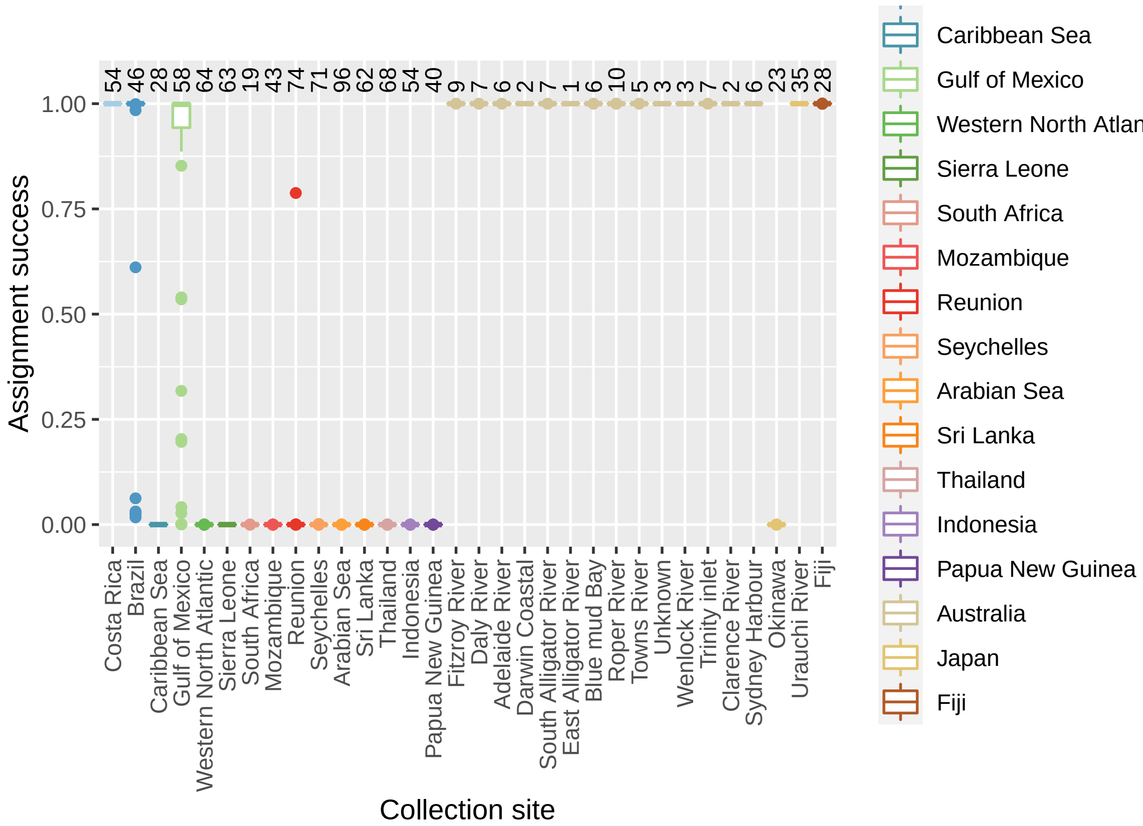


#### 8.10.1.2 Rubias

```r
ggplot2::ggsave(plot,
                filename = "DArTcap_ALL_Assign_Rubias_Sites_Assignment_success.png",
                width = 30, height = 15,units = "cm")

repunit_BS <- unique(BS.dartcap.rubias2$repunit)
BS_corr <- BS_sims$mixing_proportions %>%
  dplyr::mutate(repunit = ifelse(repunit %in% repunit_BS, repunit, "OTHER")) %>%
  dplyr::group_by(iter, repunit) %>%
  dplyr::summarise(true_repprop = sum(true_pi),
          reprop_posterior_mean = sum(post_mean_pi),
          repu_n = sum(n)) %>%
  dplyr::mutate(repu_n_prop = repu_n / sum(repu_n))

BS_corr$repunit <- factor(BS_corr$repunit, levels = pop.levels2)
plot <- ggplot2::ggplot(BS_corr, ggplot2::aes(x = repu_n_prop,
                                               y = reprop_posterior_mean,
                                               colour = repunit)) +
  ggplot2::geom_point(size = 5) +
  ggplot2::scale_color_manual(values = colours.18) +
  ggplot2::geom_abline(intercept = 0, slope = 1) +
  ggplot2::facet_wrap(~ repunit)
print(plot)
```

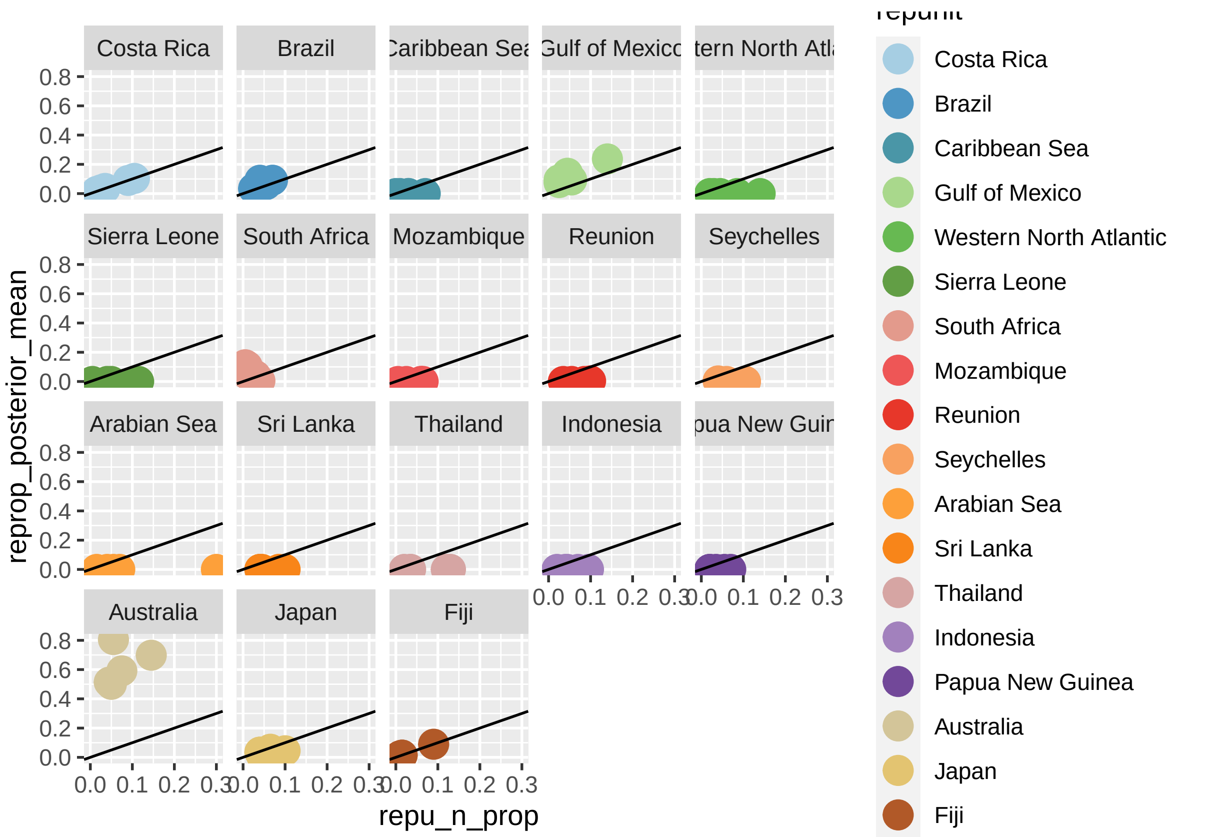


```
ggplot2::ggsave(plot, filename = "DArTcap_ALL_Assign_Rubias_Sites_correlation.png",
                width = 30, height = 15,units = "cm")
```

### 8.10.2 Per region

```
strata <- BS.dartcap.gl$other$ind.metrics$Region
strata[strata %in% c("W-IO","N-IO","E-IO","W-PAC","E-IO/W-PAC")] <- "IWP"
pop.levels <- c("E-PAC","W-ATL","E-ATL","IWP", "Japan","Fiji")
BS.dartcap.gl$pop <- factor(strata, levels = pop.levels)
BS.dartcap.gl <- BS.dartcap.gl[order(BS.dartcap.gl$pop, BS.dartcap.gl$ind.names)]

gl2gpop(BS.dartcap.gl[BS.dartcap.gl$pop != "E-ATL",],
        filename = "DArTcap_ALL_assign_Region_genepop.txt") #too few individuals
shortnames <- c("E-PAC","W-ATL","IWP", "Japan","Fiji")
gpop <- assignPOP::read.Genepop(
  'DArTcap_ALL_assign_Region_genepop.txt',
  pop.names = shortnames,
  haploid = FALSE,
  pos = 1
  )

assignPOP::assign.MC(
  x = gpop, train.inds = c(0.1, 0.5, 0.9),train.loci = c(0.05, 0.1, 0.5, 1),
  loci.sample = 'fst',iterations = 1000, dir = 'AssignPOP_MC_Regions/', scaled = FALSE,
  pca.method = 'mixed', pca.PCs = 'kaiser-guttman',pca.loadings = F,
  model = 'svm', svm.kernel = 'linear',svm.cost = 1, ntree = 50,
  multiprocess = TRUE, processors = 3, skipQ = FALSE)

assignPOP::assign.kfold(
  x = gpop, k.fold = 10, train.loci =  c(0.1, 0.5, 1), loci.sample = 'fst',
```

```r
  dir = 'AssignPOP_Kfold_Region/', scaled = FALSE, pca.method = 'mixed',
  pca.PCs = 'kaiser-guttman', pca.loadings = F, model = 'svm',
  svm.kernel = 'linear',svm.cost = 1, ntree = 50, multiprocess = TRUE, processors = 3,
  skipQ =FALSE)
```

```r
shortnames <- c("E-PAC","W-ATL","IWP", "Japan","Fiji")
df1 <- assignPOP::accuracy.MC(dir = "AssignPOP_MC_Regions/")
MC.assign <- assignPOP::accuracy.plot(df1, pop = c("all",shortnames))
print(MC.assign)
```

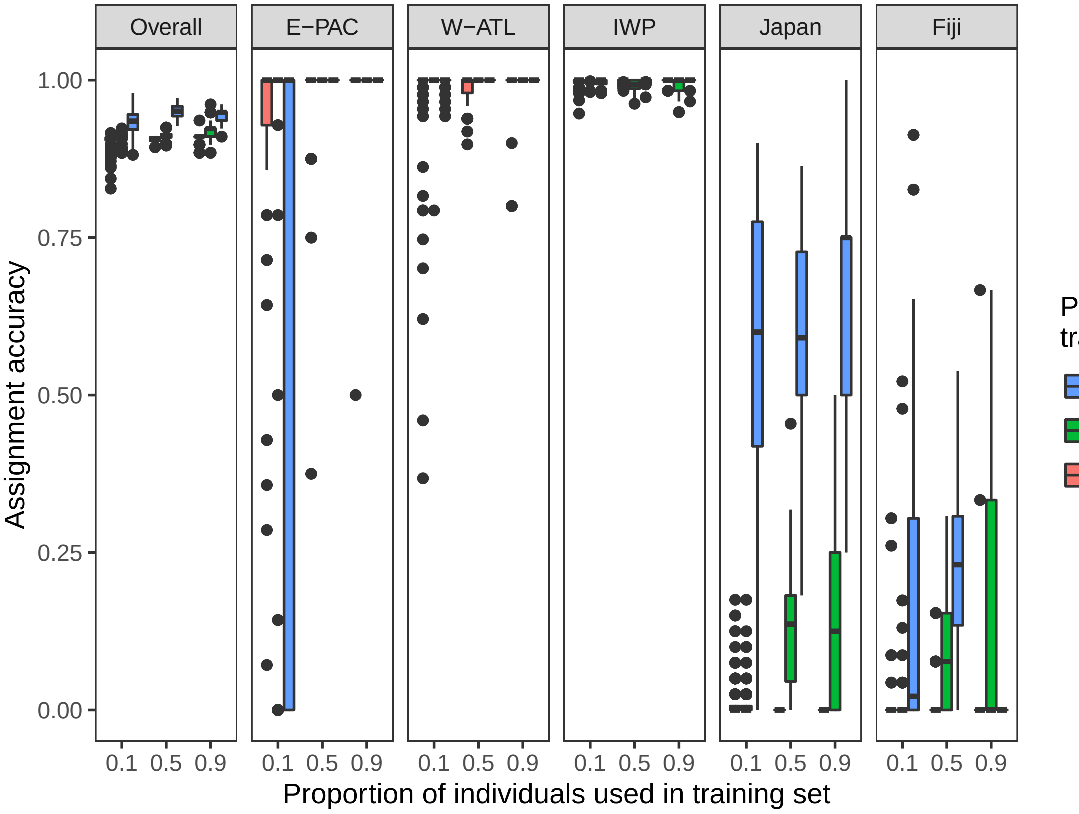


#### 8.10.2.1 AssignPOP

```r
ggplot2::ggsave(MC.assign, filename = "DArTcap_ALL_assignPOP_MC_Regions.png",
                width = 30, height = 15,units = "cm")

df2 <- assignPOP::accuracy.kfold(dir = "AssignPOP_Kfold_Region/")
kfold.assign <- assignPOP::accuracy.plot(df2, pop = c("all",shortnames))
print(kfold.assign)
```

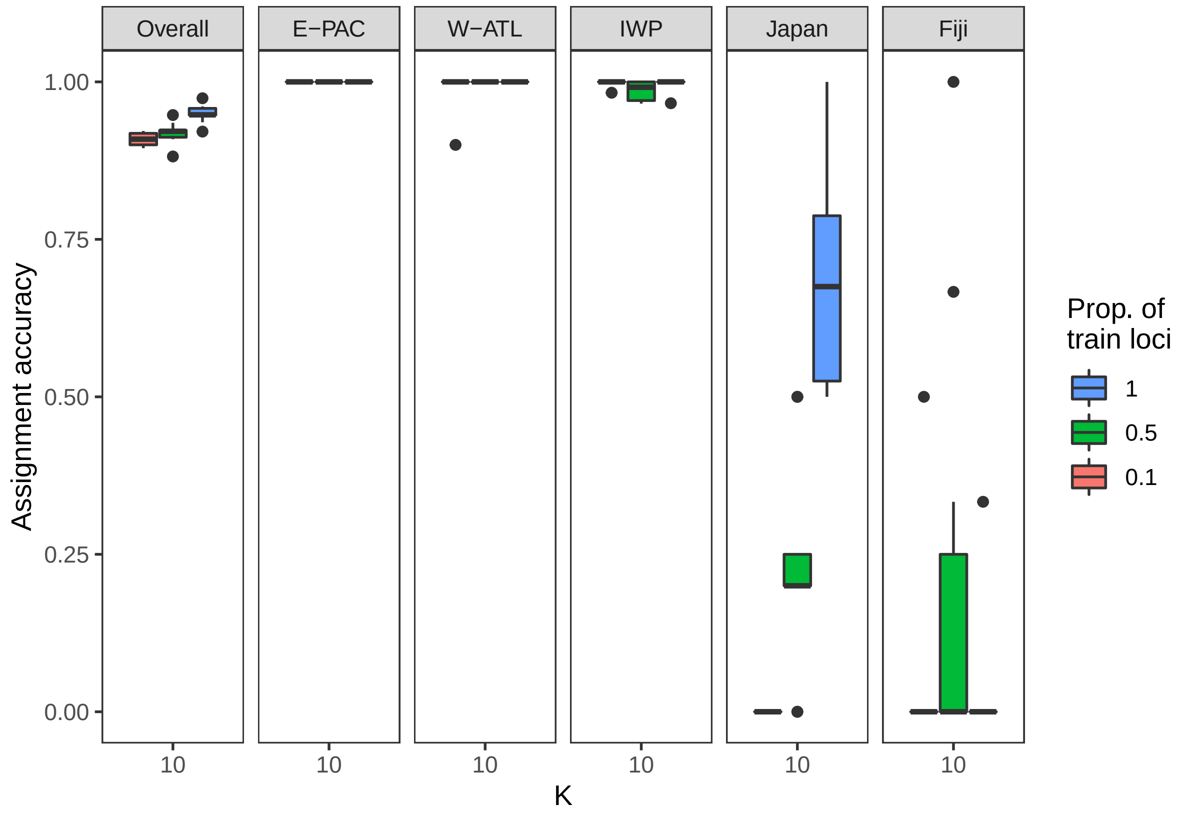


```
ggplot2::ggsave(kfold.assign, filename = "DArTcap_ALL_assignPOP_Kfold_Regions.png",
                width = 30, height = 15,units = "cm")

shortnames1 <- c("E.PAC","W.ATL","IWP", "Japan","Fiji")
colours.5 <- adegenet::funky(5)
names(colours.5) <- shortnames1
# memb.plot <- assignPOP::membership.plot(dir = "AssignPOP_Kfold_Region/",
#                              style = 1,
#                              non.genetic = TRUE)
# # choose proportion training loci = 1
# save(memb.plot, file = "memb.plot2.Rdata")
load("memb.plot2.Rdata")
memb.plot$data$origin.pop <- factor(memb.plot$data$origin.pop,
                                   levels = shortnames)
memb.plot + ggplot2::scale_fill_manual(values = colours.5) +
  ggplot2::theme(axis.text.x = ggplot2::element_blank())
```

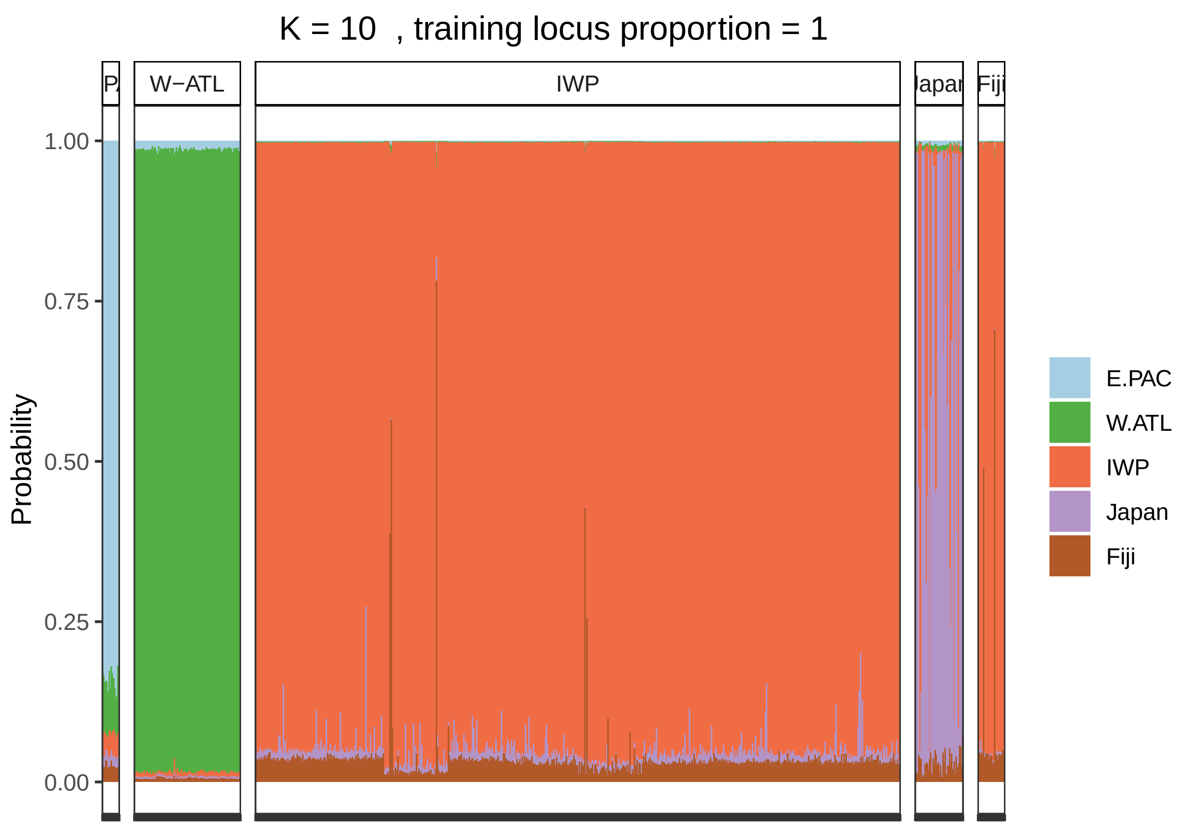


```
ggplot2::ggsave(memb.plot,
                filename = "DArTcap_ALL_assignPOP_Kfold_membership_Regions.png",
                width = 30, height = 15,units = "cm")


df <- data.frame(
  ind = as.character(memb.plot$data$Ind.ID)[
    as.character(memb.plot$data$fold_n) == "fold_10"],
  pop = as.character(memb.plot$data$origin.pop)[
    as.character(memb.plot$data$fold_n) == "fold_10"],
  memb = as.character(memb.plot$data$pred.pop)[
    as.character(memb.plot$data$fold_n) == "fold_10"], stringsAsFactors = FALSE)

summary(df$pop[df$pop != df$memb]) #still some difficulties to differentiate Japan and Fiji
```

```
##
##   Correct assignment rates were estimated!!
##   A total of 900 assignment tests for 5 pops.
##   Results were also saved in a 'Rate_of_900_tests_5_pops.txt' file in the directory.
##   Correct assignment rates were estimated!!
##   A total of 30 assignment tests for 5 pops.
##   Results were also saved in a 'Rate_of_30_tests_5_pops.txt' file in the directory. Fiji Japan
##    10     5
```

```
pop.levels1 <- c("Costa Rica" , "Brazil","Caribbean Sea", "Gulf of Mexico",
                 "Western North Atlantic", "Sierra Leone", "South Africa",
                 "Mozambique", "Reunion" , "Seychelles", "Arabian Sea", "Sri Lanka",
                 "Thailand", "Indonesia", "Papua New Guinea" , "Australia",
                 "Japan", "Fiji")
pop.levels2 <- c("E-PAC","W-ATL","E-ATL","IWP", "Japan","Fiji")
```

```r
BS.dartcap.rubias2 <- BS.dartcap.rubias
# BS.dartcap.rubias2$indiv == BS.dartcap.gt@schemes$INDIVIDUALS

strata <- BS.dartcap.gt@schemes$Region
strata[strata %in% c("W-IO","N-IO","E-IO","W-PAC","E-IO/W-PAC")] <- "IWP"
BS.dartcap.rubias2$collection <- factor(BS.dartcap.gt@schemes$Site,
                                        levels = pop.levels1)
BS.dartcap.rubias2$repunit <- factor(strata, levels = pop.levels2)
BS.dartcap.rubias2 <- BS.dartcap.rubias2[order(BS.dartcap.rubias2$repunit,
                                               BS.dartcap.rubias2$collection),]

BS.dartcap.rubias2 %<>%
  dplyr::mutate_all(as.character)

BS_sims <- rubias::assess_reference_loo(reference = BS.dartcap.rubias2,
                     gen_start_col = 5,
                     reps = 5,
                     mixsize = 200,
                     return_indiv_posteriors = TRUE,
                     resampling_unit = "gene_copies")

repu_pofzs <- BS_sims$indiv_posteriors %>%
  dplyr::filter(repunit == simulated_repunit) %>%
  dplyr::group_by(iter, indiv, simulated_collection, repunit) %>%
  dplyr::summarise(repu_PofZ = sum(PofZ)) %>%
  dplyr::ungroup() %>%
  dplyr::arrange(repunit, simulated_collection) %>%
  dplyr::mutate(simulated_collection = factor(simulated_collection,
                                              levels = unique(simulated_collection)))

num_simmed <- BS_sims$indiv_posteriors %>%
  dplyr::group_by(iter, indiv) %>%
  dplyr::slice(1) %>%
  dplyr::ungroup() %>%
  dplyr::count(simulated_collection)

num_simmed <- num_simmed[order(factor(num_simmed$simulated_collection,
                                      levels = pop.levels1)),]
repu_pofzs$repunit <- factor(repu_pofzs$repunit, levels = pop.levels2)
repu_pofzs$simulated_collection <- factor(repu_pofzs$simulated_collection,
                                          levels = pop.levels1)
repu_pofzs <- repu_pofzs[order(repu_pofzs$repunit, repu_pofzs$simulated_collection),]

plot <- ggplot2::ggplot(repu_pofzs, ggplot2::aes(x = simulated_collection,
                                                 y = repu_PofZ)) +
  ggplot2::geom_boxplot(ggplot2::aes(colour = repunit)) +
  ggplot2::scale_color_manual(values = colours.6) +
  ggplot2::geom_text(data = num_simmed, mapping = aes(y = 1.025, label = n),
                     angle = 90, hjust = 0, vjust = 0.5, size = 3) +
  ggplot2::labs(subtitle = "",
        y = "Assignment success",
        x = "Collection site") +
  ggplot2::theme(axis.text.x = ggplot2::element_text(angle = 90, hjust = 1,
                                                     size = 9, vjust = 0.5)) +
  ggplot2::ylim(c(NA, 1.05))
print(plot)
```

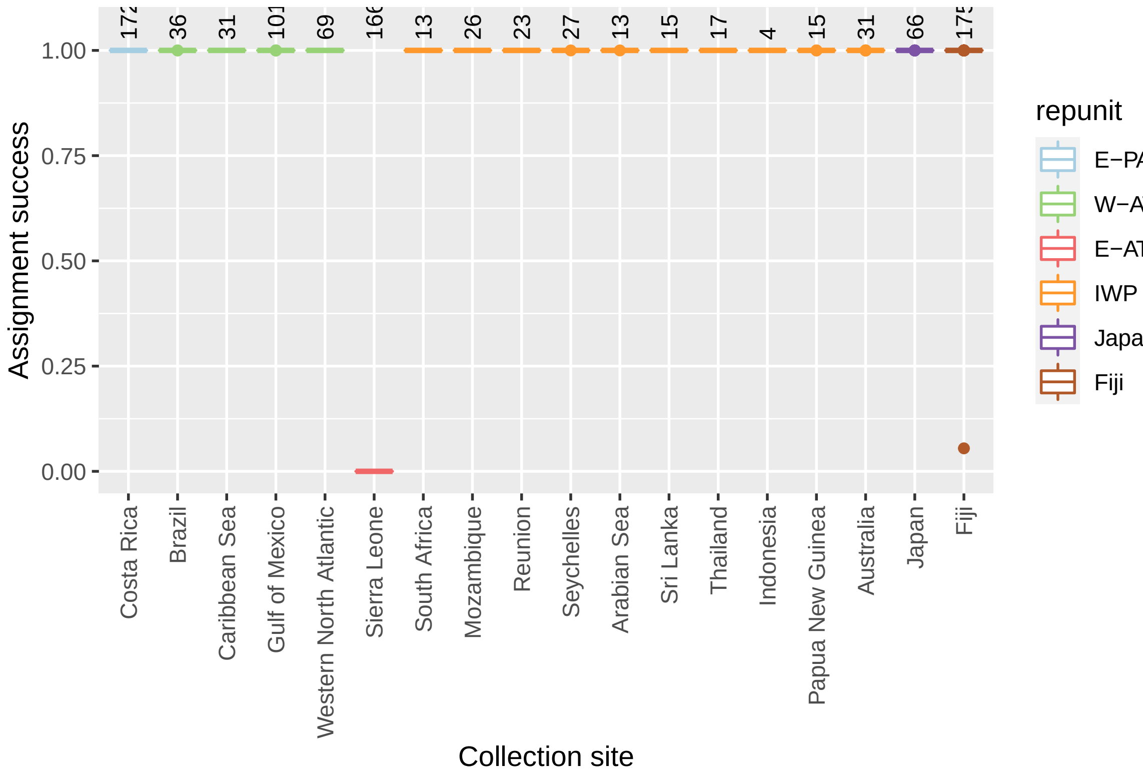


#### 8.10.2.2 Rubias

```
ggplot2::ggsave(plot,
                filename = "DArTcap_ALL_Assign_Rubias_Regions_Assignment_success.png",
                width = 30, height = 15,units = "cm")

repunit_BS <- unique(BS.dartcap.rubias2$repunit)
BS_corr <- BS_sims$mixing_proportions %>%
  dplyr::mutate(repunit = ifelse(repunit %in% repunit_BS, repunit, "OTHER")) %>%
  dplyr::group_by(iter, repunit) %>%
  dplyr::summarise(true_repprop = sum(true_pi),
            reprop_posterior_mean = sum(post_mean_pi),
            repu_n = sum(n)) %>%
  dplyr::mutate(repu_n_prop = repu_n / sum(repu_n))

BS_corr$repunit <- factor(BS_corr$repunit, levels = pop.levels2)
plot <- ggplot2::ggplot(BS_corr, ggplot2::aes(x = repu_n_prop,
                                              y = reprop_posterior_mean,
                                              colour = repunit)) +
  ggplot2::geom_point(size = 5) +
  ggplot2::scale_color_manual(values = colours.6) +
  ggplot2::geom_abline(intercept = 0, slope = 1) +
  ggplot2::facet_wrap(~ repunit)
print(plot)
```

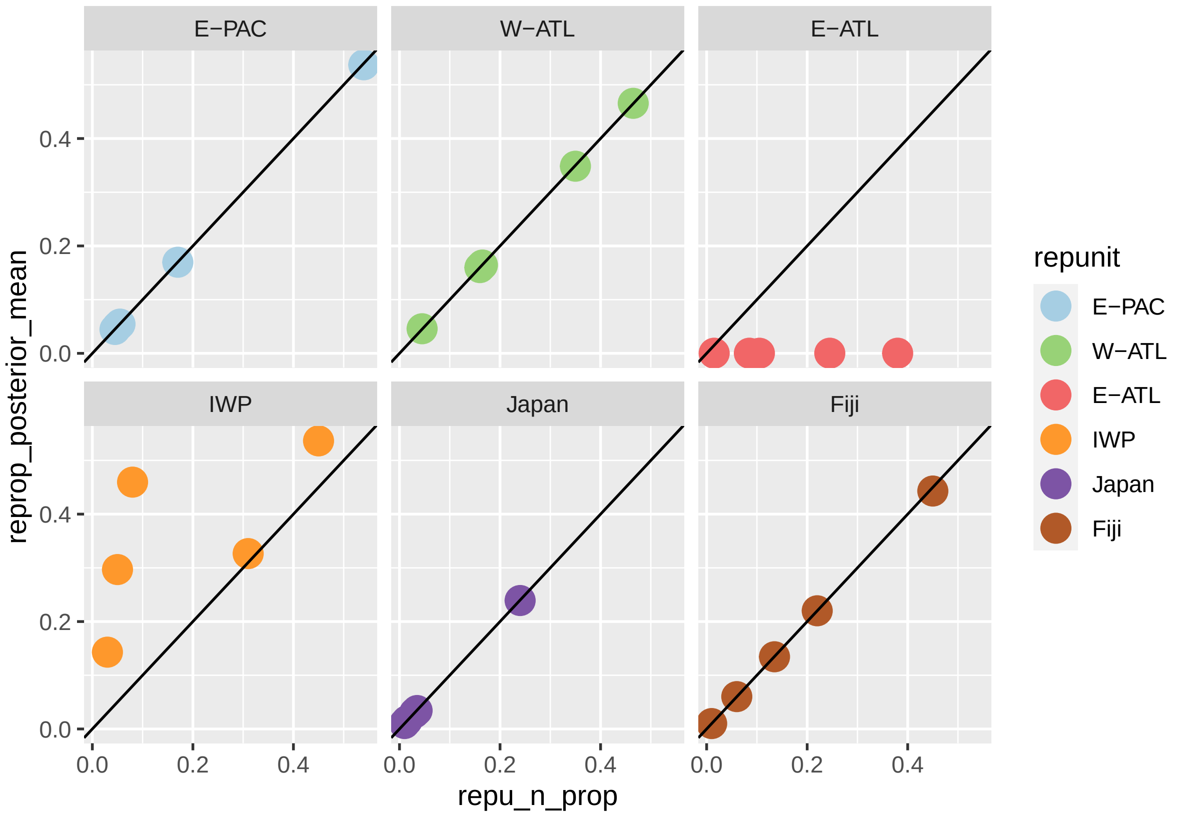


```
ggplot2::ggsave(plot, filename = "DArTcap_ALL_Assign_Rubias_Regions_correlation.png",
                width = 30, height = 15,units = "cm")
```

#### 8.10.3 Test number of informative markers chosen by DAPC contribution

Using a 50% training dataset and validate the assignment success on a 50% hold-out dataset.

```
region.levels <- c("E-PAC","W-ATL","E-ATL", "IWP", "Japan","Fiji")
BS.dartcap.gl1 <- BS.dartcap.gl
region <- BS.dartcap.gl$other$ind.metrics$Region
region[region %in% c("W-IO","N-IO", "E-IO", "E-IO/W-PAC", "W-PAC")] <- "IWP"
BS.dartcap.gl1$pop <- factor(region, levels = region.levels)

subset.names <- c()
for (i in levels(BS.dartcap.gl1$pop)) {
  gl <- BS.dartcap.gl1[BS.dartcap.gl1$pop == i,]
  subset.names <- c(subset.names,sample(gl$ind.names,
                                        size = ceiling(adegenet::nInd(gl)/2)))
}
BS.dartcap.gl2 <- BS.dartcap.gl1[BS.dartcap.gl1$ind.names %in% subset.names,]
BS.dartcap.gl3 <- BS.dartcap.gl1[!BS.dartcap.gl1$ind.names %in%
                                   subset.names[subset.names != "CL-SIL001-F"],] #only 1 sample


dapc1 <- adegenet::dapc(BS.dartcap.gl2, n.da = 5, n.pca = 76)
assign.success <- summary(dapc1)$assign.per.pop*100

plot.data <- data.frame(dapc1$ind.coord, pop = BS.dartcap.gl2$pop)
ggplot(plot.data, aes(x = LD1, y = LD2, col = pop)) + ggplot2::geom_point() +
  ggplot2::scale_color_manual(values = adegenet::funky(6))
ggplot(plot.data, aes(x = LD3, y = LD5, col = pop)) + ggplot2::geom_point() +
  ggplot2::scale_color_manual(values = adegenet::funky(6))
```

```r
ggplot(plot.data, aes(x = LD3, y = LD4, col = pop)) + ggplot2::geom_point() +
  ggplot2::scale_color_manual(values = adegenet::funky(6))
ggplot(plot.data, aes(x = LD4, y = LD5, col = pop)) + ggplot2::geom_point() +
  ggplot2::scale_color_manual(values = adegenet::funky(6))

marker.sample <- c(seq(5,100, by = 5), 125, 250, 375, 500)
loc.number <- list()
loc.names <- list()
assign.success <- data.frame(Pop =  levels(BS.dartcap.gl2$pop))
for (i in 1:length(marker.sample)) {
  loc.number[[i]] <- c(
    rownames(dapc1$var.contr %>%
        as.data.frame() %>%
        dplyr::slice_max(n = marker.sample[i] / 5, with_ties  = FALSE,
                         order_by = LD1)
    ),
    rownames(dapc1$var.contr %>%
        as.data.frame() %>%
        dplyr::slice_max(n = marker.sample[i] / 5, with_ties  = FALSE,
                         order_by = LD2)
    ),
    rownames(dapc1$var.contr %>%
        as.data.frame() %>%
        dplyr::slice_max(n = marker.sample[i] / 5, with_ties  = FALSE,
                         order_by = LD3)
    ),
    rownames(dapc1$var.contr %>%
        as.data.frame() %>%
        dplyr::slice_max(n = marker.sample[i] / 5, with_ties  = FALSE,
                         order_by = LD4)
    ),
    rownames(dapc1$var.contr %>%
        as.data.frame() %>%
        dplyr::slice_max(n = marker.sample[i] / 5, with_ties  = FALSE,
                         order_by = LD5)
    ))
  loc.names[[i]] <- BS.dartcap.gl2$loc.names[as.integer(loc.number[[i]])]
  BS.dartcap.gl4 <- BS.dartcap.gl3[, as.integer(loc.number[[i]])]
  dapc2 <- adegenet::dapc(BS.dartcap.gl4,n.da = 5, n.pca = 76) # DAPC on hold-out data
  assign.success[[i + 1]] <- as.double(summary(dapc2)$assign.per.pop*100)
}
colnames(assign.success) <- c("pop", as.character(marker.sample))
save(assign.success,marker.sample, loc.number,loc.names, subset.names,
     file = "DArTcap_ALL_Informative_DAPC_markers.Rdata")

loc.names2 <- unlist(loc.names)
provenance.markers <- BS.dartcap.gl$other$loc.metrics[
  BS.dartcap.gl$other$loc.metrics$LOCUS %in% loc.names2,]
readr::write_tsv(provenance.markers,
                 file = "500_DArTcap_markers_for_Bull_Shark_provenance.tsv")
```

```r
load("DArTcap_ALL_Informative_DAPC_markers.Rdata")
mean.assign.success <- colMeans(assign.success[,-1])

region.levels <- c("E-PAC", "W-ATL", "E-ATL", "IWP","Japan", "Fiji")

plot.data <- gather(assign.success, Markers, Assignment, 2:ncol(assign.success))
plot.data$Markers <- as.integer(plot.data$Markers)
```

```
plot.data$pop <- factor(plot.data$pop, levels = region.levels)
plot.data <- plot.data[order(as.integer(plot.data$Markers), plot.data$pop),]
assign.plot <- ggplot2::ggplot(plot.data,
                               aes(x = Markers,
                                   y = Assignment,
                                   col = pop,
                                   group = 1)) +
  ylab("Assignmenet success (%)") +
  xlab("Number of informative markers") +
  ggplot2::geom_point() +
  ggformula::geom_spline(aes(group = pop))

print(assign.plot)
```

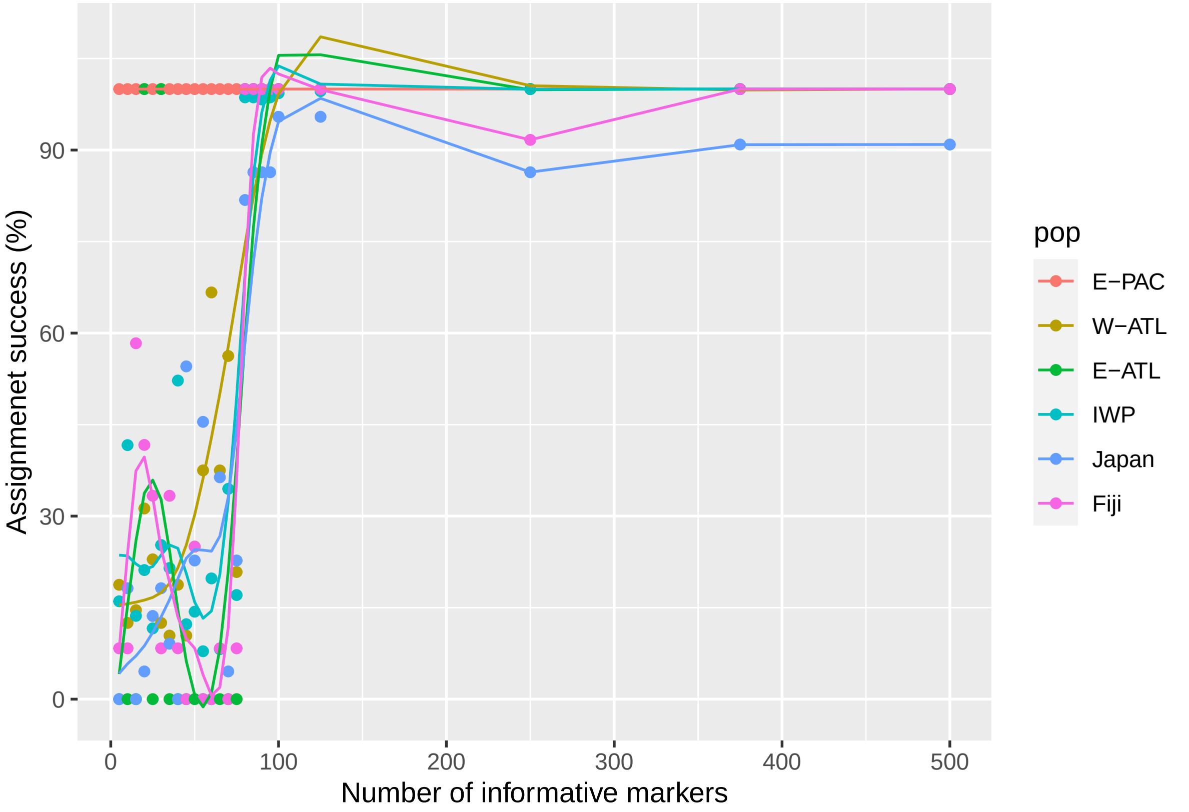


```
ggplot2::ggsave(assign.plot,
                filename = "DArTcap_ALL_Assign_DAPC.png",
                width = 30, height = 15, units = "cm")
```

Test rubias assignment success, based on 5-500 markers with highest loading contributions. Note that only samples from wild populations were use here (i.e. not the Okinawa aquarium, Japan).

```
load("DArTcap_ALL_Informative_DAPC_markers.Rdata")

pop.levels1 <- c("Costa Rica" , "Brazil","Caribbean Sea", "Gulf of Mexico",
                 "Western North Atlantic", "Sierra Leone", "South Africa",
                 "Mozambique", "Reunion" , "Seychelles", "Arabian Sea",
                 "Sri Lanka", "Thailand", "Indonesia", "Papua New Guinea" ,
                 "Australia", "Urauchi River, Japan", "Fiji")
pop.levels2 <- c("E-PAC","W-ATL","E-ATL","IWP", "Japan","Fiji")

colnames(BS.dartcap.rubias2)
BS.dartcap.rubias2 <- BS.dartcap.rubias
```

```r
# BS.dartcap.rubias2$indiv == BS.dartcap.gt@schemes$INDIVIDUALS

strata <- BS.dartcap.gt@schemes$Region
strata[strata %in% c("W-IO","N-IO","E-IO","W-PAC","E-IO/W-PAC")] <- "IWP"
BS.dartcap.rubias2$collection <- factor(BS.dartcap.gt@schemes$Site,
                                        levels = pop.levels1)
BS.dartcap.rubias2$repunit <- factor(strata, levels = pop.levels2)
BS.dartcap.rubias2 <- BS.dartcap.rubias2[order(BS.dartcap.rubias2$repunit,
                                               BS.dartcap.rubias2$collection),]

BS.dartcap.rubias2 %<>%
  dplyr::mutate_all(as.character)

res <- data.frame(iter = integer(), indiv = integer(),
                  simulated_collection = factor(),
                  repunit = factor(), repu_PofZ = double(), Nmarkers = double())
for (i in 1:length(marker.sample)) {
  locus.rubias <- stringr::str_match(pattern = "__\\s*(.*?)\\s*__",
                                     string =  colnames(BS.dartcap.rubias2))[,2]
  locus.rubias <- c("sample_type","repunit","collection","indiv",
                    locus.rubias[-c(1:4)])
  BS.dartcap.rubias3 <- BS.dartcap.rubias2[,locus.rubias %in%
                                             c("sample_type","repunit",
                                               "collection","indiv",
                                               loc.names[[i]])]
  BS_sims <- rubias::assess_reference_loo(reference = BS.dartcap.rubias3,
                       gen_start_col = 5,
                       reps = 5,
                       mixsize = 200,
                       return_indiv_posteriors = TRUE)
  repu_pofzs <- BS_sims$indiv_posteriors %>%
    dplyr::filter(repunit == simulated_repunit) %>%
    dplyr::group_by(iter, indiv, simulated_collection, repunit) %>%
    dplyr::summarise(repu_PofZ = sum(PofZ)) %>%
    dplyr::ungroup() %>%
    dplyr::arrange(repunit, simulated_collection) %>%
    dplyr::mutate(simulated_collection = factor(simulated_collection,
                                                levels = unique(simulated_collection)))

  num_simmed <- BS_sims$indiv_posteriors %>%
    dplyr::group_by(iter, indiv) %>%
    dplyr::slice(1) %>%
    dplyr::ungroup() %>%
    dplyr::count(simulated_collection)

  num_simmed <- num_simmed[order(factor(num_simmed$simulated_collection,
                                        levels = pop.levels1)),]
  repu_pofzs$repunit <- factor(repu_pofzs$repunit, levels = pop.levels2)
  repu_pofzs$simulated_collection <- factor(repu_pofzs$simulated_collection,
                                            levels = pop.levels1)
  repu_pofzs <- repu_pofzs[order(repu_pofzs$repunit,
                                 repu_pofzs$simulated_collection),]
  repu_pofzs$Nmarkers <- marker.sample[[i]]
  res <- rbind(res,repu_pofzs)
}
save(res, file = "DArTcap_ALL_Informative_DAPC_markers_rubias assignment2.Rdata")
```

```r
load("DArTcap_ALL_Informative_DAPC_markers_rubias_assignment2.Rdata")
plot <- ggplot2::ggplot(res, ggplot2::aes(x = as.factor(Nmarkers),
                                          y = repu_PofZ)) +
  ggplot2::geom_boxplot(ggplot2::aes(colour = repunit)) +
  ggplot2::scale_color_manual(values = colours.6) +
  ggplot2::facet_wrap(~simulated_collection) +
  ggplot2::labs(subtitle = "",
        y = "Assignment success",
        x = "Number of informative markers") +
  ggplot2::theme(axis.text.x = ggplot2::element_text(angle = 90,
                                                     hjust = 1, size = 9,
                                                     vjust = 0.5)) +
  ggplot2::ylim(c(NA, 1.05))
print(plot)
```

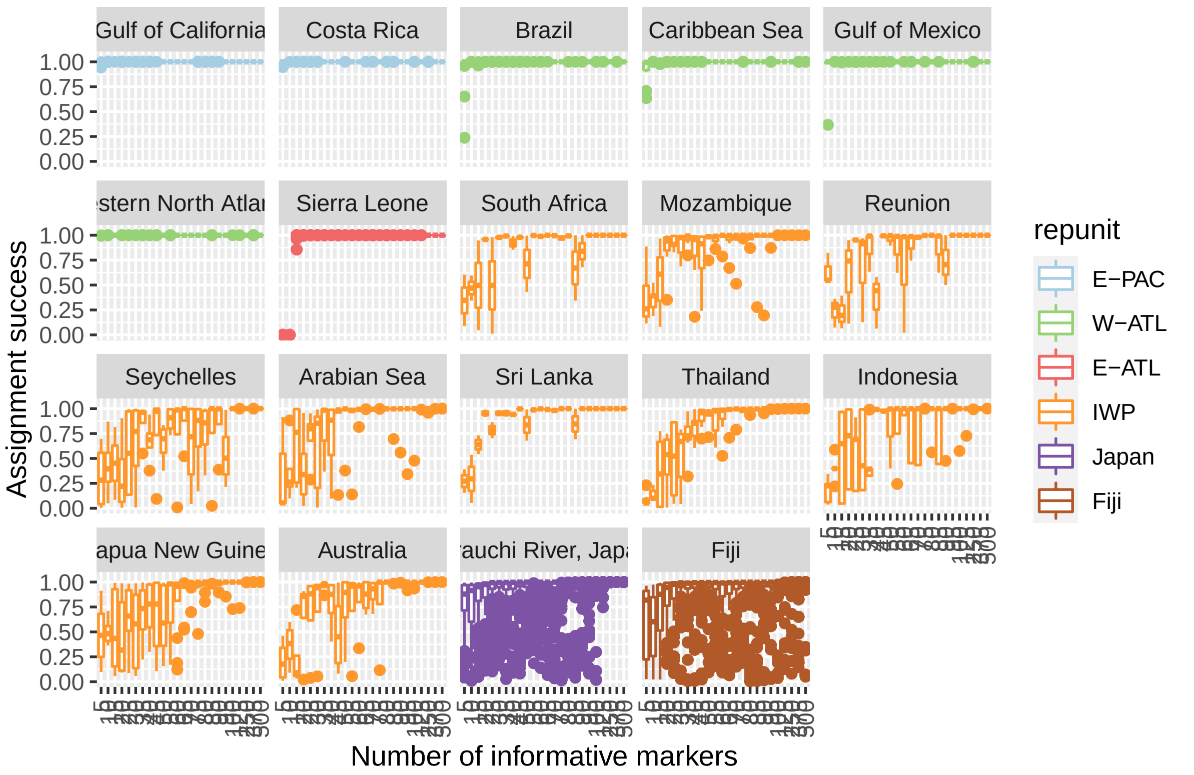


```r
ggplot2::ggsave(plot,
                filename = "DArTcap_ALL_Informative_DAPC_markers_rubias assignment2.png",
                width = 30, height = 15,units = "cm")
```

## 8.11 Sex-specific connectivity

### 8.11.1 Sex-linked markers

```r
load("DArTcap_Raw_tidy.Rdata")
BS.dartcap.raw.tidy <- BSsnp$tidy.data
length(unique(BS.dartcap.raw.tidy$INDIVIDUALS)) #942
length(unique(BS.dartcap.raw.tidy$MARKERS)) #19549

load("Sex-linked_marker_DArTcap.Rdata")
load("Sex_results.Rdata")
x.markers <- unique(c(bull_shark$sexy.summary$CLONE_ID[
```

```
  bull_shark$sexy.summary$MARKER_TYPE == "Homogametic_sex-marker"],
                    SLM$sexy.summary$CLONE_ID[
                      SLM$sexy.summary$MARKER_TYPE == "Homogametic_sex-marker"]))
y.markers <- unique(c(bull_shark$sexy.summary$CLONE_ID[
  bull_shark$sexy.summary$MARKER_TYPE == "Heterogametic_sex-marker"],
                    SLM$sexy.summary$CLONE_ID[
                      SLM$sexy.summary$MARKER_TYPE == "Heterogametic_sex-marker"]))

sum(unique(BS.dartcap.raw.tidy$LOCUS) %in% x.markers) ##121
BS.dartcap.x.tidy <- BS.dartcap.raw.tidy[BS.dartcap.raw.tidy$LOCUS %in% x.markers, ]

sum(unique(BS.dartcap.raw.tidy$LOCUS) %in% y.markers) ##0

Convert <- radiator::genomic_converter(BS.dartcap.x.tidy,
                                       output = c("genlight","gtypes"))
BS.dartcap.gl.x <- Convert$genlight
BS.dartcap.gt.x <- Convert$gtypes

pop.levels <- c("Costa Rica", "Brazil", "Caribbean Sea", "Gulf of Mexico",
                "Western North Atlantic", "Sierra Leone", "South Africa",
                "Mozambique", "Reunion" , "Seychelles", "Arabian Sea",
                "Sri Lanka", "Thailand", "Indonesia", "Papua New Guinea" ,
                "Australia", "Japan", "Fiji")
BS.dartcap.gl.x$pop <- factor(BS.dartcap.gl.x$other$ind.metrics$Site,
                              levels = pop.levels)
BS.dartcap.gl.x <- BS.dartcap.gl.x[order(BS.dartcap.gl.x$pop,
                                         BS.dartcap.gl.x$ind.names),]

pop.levels <- c("Costa_Rica", "Brazil", "Caribbean_Sea", "Gulf_of_Mexico",
                "Western_North_Atlantic", "Sierra_Leone", "South_Africa",
                "Mozambique", "Reunion" , "Seychelles", "Arabian_Sea", "Sri_Lanka",
                "Thailand", "Indonesia", "Papua_New_Guinea" , "Australia",
                "Japan", "Fiji")
BS.dartcap.gt.x@data$stratum <- factor(BS.dartcap.gt.x@data$stratum,
                                       levels = pop.levels)
BS.dartcap.gt.x <- BS.dartcap.gt.x[order(BS.dartcap.gt.x@data$id),]
BS.dartcap.gt.x@data <- BS.dartcap.gt.x@data[order(BS.dartcap.gt.x@data$id),]
BS.dartcap.gt.x@schemes <- BS.dartcap.gt.x@schemes[order(BS.dartcap.gt.x@schemes$id),]

save(BS.dartcap.x.tidy, BS.dartcap.gl.x,BS.dartcap.gt.x,
     file = "DArTcap_X_SLM.Rdata")
```

```
load("Sex-linked_marker_DArTcap.Rdata")
load("Sex_results.Rdata")
sex.markers <- unique(c(bull_shark$sexy.summary$CLONE_ID, SLM$sexy.summary$CLONE_ID))

load("DArTcap_X_SLM.Rdata")
sum(BS.dartcap.gl.x$other$loc.metrics$CloneID %in% sex.markers) #148


pop.levels <- c("Costa Rica" , "Brazil", "Caribbean Sea","Gulf of Mexico",
                "Western North Atlantic",   "Sierra Leone", "South Africa",
                "Mozambique", "Reunion" , "Seychelles", "Arabian Sea",
                "Sri Lanka", "Thailand", "Indonesia", "Papua New Guinea" ,
                "Australia", "Japan", "Fiji")
BS.dartcap.gl.x$pop <- factor(BS.dartcap.gl.x$other$ind.metrics$Site,
                              levels = pop.levels)
```

```r
gl.x.F.filtered <- BS.dartcap.gl.x[
  BS.dartcap.gl.x@other$ind.metrics$COMBINED_SEX == "F" &
    BS.dartcap.gl.x$ind.names %in% BS.dartcap.gl$ind.names,]
gl.x.M.filtered <- BS.dartcap.gl.x[
  BS.dartcap.gl.x@other$ind.metrics$COMBINED_SEX == "M" &
    BS.dartcap.gl.x$ind.names %in% BS.dartcap.gl$ind.names,]

gt.x.F.filtered <- BS.dartcap.gt.x[
  BS.dartcap.gt.x@schemes$COMBINED_SEX == "F" &
    getIndNames(BS.dartcap.gt.x) %in% getIndNames(BS.dartcap.gt),,drop = TRUE]
gt.x.M.filtered <- BS.dartcap.gt.x[
  BS.dartcap.gt.x@schemes$COMBINED_SEX == "M" &
    BS.dartcap.gt.x@schemes$id %in% BS.dartcap.gt@schemes$id,,drop = TRUE]
gt.x.F.filtered@schemes <- BS.dartcap.gt.x@schemes[
  BS.dartcap.gt.x@schemes$COMBINED_SEX == "F" &
    BS.dartcap.gt.x@schemes$id %in% BS.dartcap.gt@schemes$id,]
gt.x.M.filtered@schemes <- BS.dartcap.gt.x@schemes[
  BS.dartcap.gt.x@schemes$COMBINED_SEX == "M" &
    BS.dartcap.gt.x@schemes$id %in% BS.dartcap.gt@schemes$id,]

adegenet::nInd(gl.x.F.filtered) #353
summary(gl.x.F.filtered$pop)
adegenet::nInd(gl.x.M.filtered) #338
summary(gl.x.M.filtered$pop)
adegenet::nLoc(gl.x.F.filtered) #148
sum(as.data.frame(gl.x.M.filtered) == 1, na.rm = TRUE) #351
sum(as.data.frame(gl.x.F.filtered) == 1, na.rm = TRUE) #2156
```

```
## [1] 148
## [1] 353
##             Costa Rica          Gulf of Mexico Western North Atlantic
##                      9                      18                      6
##           Sierra Leone            South Africa             Mozambique
##                      1                       9                      5
##                Reunion              Seychelles            Arabian Sea
##                     13                      28                      9
##              Sri Lanka                Thailand              Indonesia
##                      7                       3                      1
##       Papua New Guinea               Australia                  Japan
##                      4                     193                     25
##                   Fiji
##                     22
## [1] 338
##             Costa Rica           Caribbean Sea         Gulf of Mexico
##                      7                       2                     19
## Western North Atlantic            South Africa             Mozambique
##                      2                      12                      6
##                Reunion              Seychelles            Arabian Sea
##                     15                       8                      6
##              Sri Lanka                Thailand              Indonesia
##                      5                       2                      3
##       Papua New Guinea               Australia                  Japan
##                      4                     225                     19
##                   Fiji
##                      3
## [1] 148
## [1] 351
```

```
## [1] 2156
```

```
pop.levels <- c("Costa Rica" , "Brazil", "Caribbean Sea", "Gulf of Mexico",
                "Western North Atlantic", "South Africa", "Mozambique",
                "Reunion" , "Seychelles", "Arabian Sea", "Sri Lanka", "Thailand",
                "Indonesia", "Papua New Guinea" , "Australia", "Japan", "Fiji")

gl <- BS.dartcap.gl
gl <- gl[gl$pop != "Sierra Leone"] #only 1 sample
gl$pop <- factor(gl$other$ind.metrics$Site, levels = pop.levels)
gl <- gl[order(gl$pop,gl$ind.names)]

gl2gpop(gl, filename = "BS_dartcap_all_Sites_genepop.txt")
bastat <-diveRsity::basicStats(infile = "BS_dartcap_all_Sites_genepop.txt",
                               outfile = "BS_dartcap_all_Sites_BasicStat_output.txt",
                               fis_ci = TRUE,ar_ci = TRUE, fis_boots = 1000,
                               ar_boots = 1000,mc_reps = 1000,
                               rarefaction = FALSE, ar_alpha = 0.05,
                               fis_alpha = 0.05)

length(bastat$main_tab)#17
gendiv <- data.frame(
  "Costa Rica" = bastat$main_tab[[1]]$overall,
  "Brazil" = bastat$main_tab[[2]]$overall,
  "Caribbean Sea" = bastat$main_tab[[3]]$overall,
  "Gulf of Mexico" = bastat$main_tab[[4]]$overall,
  "Western North Atlantic" = bastat$main_tab[[5]]$overall,
  "South Africa" = bastat$main_tab[[6]]$overall,
  "Mozambique" = bastat$main_tab[[7]]$overall,
  "Reunion" = bastat$main_tab[[8]]$overall,
  "Seychelles" = bastat$main_tab[[9]]$overall,
  "Arabian Sea" = bastat$main_tab[[10]]$overall,
  "Sri Lanka" = bastat$main_tab[[11]]$overall,
  "Thailand" = bastat$main_tab[[12]]$overall,
  "Indonesia" = bastat$main_tab[[13]]$overall,
  "Papua New Guinea" = bastat$main_tab[[14]]$overall,
  "Australia" = bastat$main_tab[[15]]$overall,
  "Japan" = bastat$main_tab[[16]]$overall,
  "Fiji" = bastat$main_tab[[17]]$overall,
  check.names = FALSE)
row.names(gendiv) <- rownames(bastat$main_tab[[1]])

df <- as.data.frame(gl)
df[df == "0"] <- "11"
df[df == "1"] <- "12"
df[df == "2"] <- "22"
gen.gi <- adegenet::df2genind(df, ploidy = 2,ncode = 2, pop = gl$pop)
ris_basic <- hierfstat::basic.stats(gen.gi)
fis.hierfstat <- apply(ris_basic$Fis, 2, mean, na.rm=T)
fis.CI <- hierfstat::boot.ppfis(gen.gi, nboot = 1000, quant = c(0.025,0.975),
                                diploid = TRUE,dig = 4)
df <- data.frame(fis.hierfstat,fis.CI.low = fis.CI$fis.ci[,1],
                 fis.CI.high = fis.CI$fis.ci[,2])
df <- as.data.frame(t(df))
gendiv <- rbind(gendiv, df)
readr::write_tsv(gendiv, path = "DArTcap_ALL_Sites_gendiv.txt")
save(gendiv, file = "DArTcap_ALL_Sites_gendiv.Rdata")
```

```
gl <- gl.x.F.filtered
gl <- gl[!gl$pop %in% c("Sierra Leone", "Indonesia")] #only 1 sample
gl <- gl[order(gl$pop,gl$ind.names)]

gl2gpop(gl, filename = "BS_dartcap_all_X_F_genepop.txt")
bastat <-diveRsity::basicStats(infile = "BS_dartcap_all_X_F_genepop.txt",
                               outfile = "BS_dartcap_all_X_F_BasicStat_output.txt",
                               fis_ci = TRUE,ar_ci = TRUE, fis_boots = 1000,
                               ar_boots = 1000,mc_reps = 1000,
                               rarefaction = FALSE, ar_alpha = 0.05,
                               fis_alpha = 0.05)

length(bastat$main_tab)#14
gendiv.x.F <- data.frame(
  "Costa Rica" = bastat$main_tab[[1]]$overall,
  "Gulf of Mexico" = bastat$main_tab[[2]]$overall,
  "Western North Atlantic" = bastat$main_tab[[3]]$overall,
  "South Africa" = bastat$main_tab[[4]]$overall,
  "Mozambique" = bastat$main_tab[[5]]$overall,
  "Reunion" = bastat$main_tab[[6]]$overall,
  "Seychelles" = bastat$main_tab[[7]]$overall,
  "Arabian Sea" = bastat$main_tab[[8]]$overall,
  "Sri Lanka" = bastat$main_tab[[9]]$overall,
  "Thailand" = bastat$main_tab[[10]]$overall,
  "Papua New Guinea" = bastat$main_tab[[11]]$overall,
  "Australia" = bastat$main_tab[[12]]$overall,
  "Japan" = bastat$main_tab[[13]]$overall,
  "Fiji" = bastat$main_tab[[14]]$overall,
  check.names = FALSE)
row.names(gendiv.x.F) <- rownames(bastat$main_tab[[1]])

df <- as.data.frame(gl)
df[df == "0"] <- "11"
df[df == "1"] <- "12"
df[df == "2"] <- "22"
gen.gi <- adegenet::df2genind(df, ploidy = 2,ncode = 2, pop = gl$pop)
ris_basic <- hierfstat::basic.stats(gen.gi)
fis.hierfstat <- apply(ris_basic$Fis, 2, mean, na.rm=T)
fis.CI <- hierfstat::boot.ppfis(gen.gi, nboot = 1000, quant = c(0.025,0.975),
                                diploid = TRUE,dig = 4)
df <- data.frame(fis.hierfstat,fis.CI.low = fis.CI$fis.ci[,1],
                 fis.CI.high = fis.CI$fis.ci[,2])
df <- as.data.frame(t(df))
gendiv.x.F <- rbind(gendiv.x.F, df)

readr::write_tsv(gendiv.x.F, path = "DArTcap_ALL_gendiv_X_F.txt")
save(gendiv.x.F, file = "DArTcap_ALL_gendiv_X_perSites.Rdata")


gl <- gl.x.M.filtered
gl <- gl[order(gl$pop,gl$ind.names)]

gl2gpop(gl, filename = "BS_dartcap_all_X_M_genepop.txt")
bastat <-diveRsity::basicStats(infile = "BS_dartcap_all_X_M_genepop.txt",
                               outfile = "BS_dartcap_all_X_M_BasicStat_output.txt",
                               fis_ci = TRUE,ar_ci = TRUE, fis_boots = 1000,
                               ar_boots = 1000,mc_reps = 1000,
```

```r
                                rarefaction = FALSE, ar_alpha = 0.05,
                                fis_alpha = 0.05)

length(bastat$main_tab)#16
gendiv.x.M <- data.frame(
  "Costa Rica" = bastat$main_tab[[1]]$overall,
  "Caribbean Sea" = bastat$main_tab[[2]]$overall,
  "Gulf of Mexico" = bastat$main_tab[[3]]$overall,
  "Western North Atlantic" = bastat$main_tab[[4]]$overall,
  "South Africa" = bastat$main_tab[[5]]$overall,
  "Mozambique" = bastat$main_tab[[6]]$overall,
  "Reunion" = bastat$main_tab[[7]]$overall,
  "Seychelles" = bastat$main_tab[[8]]$overall,
  "Arabian Sea" = bastat$main_tab[[9]]$overall,
  "Sri Lanka" = bastat$main_tab[[10]]$overall,
  "Thailand" = bastat$main_tab[[11]]$overall,
  "Indonesia" = bastat$main_tab[[12]]$overall,
  "Papua New Guinea" = bastat$main_tab[[13]]$overall,
  "Australia" = bastat$main_tab[[14]]$overall,
  "Japan" = bastat$main_tab[[15]]$overall,
  "Fiji" = bastat$main_tab[[16]]$overall,
  check.names = FALSE)
row.names(gendiv.x.M) <- rownames(bastat$main_tab[[1]])

df <- as.data.frame(gl)
df[df == "0"] <- "11"
df[df == "1"] <- "12"
df[df == "2"] <- "22"
gen.gi <- adegenet::df2genind(df, ploidy = 2,ncode = 2, pop = gl$pop)
ris_basic <- hierfstat::basic.stats(gen.gi)
fis.hierfstat <- apply(ris_basic$Fis, 2, mean, na.rm=T)
fis.CI <- hierfstat::boot.ppfis(gen.gi, nboot = 1000, quant = c(0.025,0.975),
                                 diploid = TRUE,dig = 4)
df <- data.frame(fis.hierfstat,fis.CI.low = fis.CI$fis.ci[,1],
                 fis.CI.high = fis.CI$fis.ci[,2])
df <- as.data.frame(t(df))
gendiv.x.M <- rbind(gendiv.x.M, df)

readr::write_tsv(gendiv.x.M, path = "DArTcap_ALL_gendiv_X_M.txt")
save(gendiv.x.F,gendiv.x.M,
     file = "DArTcap_ALL_gendiv_X_perSites.Rdata")
```

```r
load("DArTcap_ALL_Sites_gendiv.Rdata")
load("DArTcap_ALL_gendiv_X_perSites.Rdata")
knitr::kable(gendiv, digits = 4,
             caption = "Genetic diversity for all loci") %>%
  kableExtra::kable_styling(full_width = FALSE,font_size = 5,
                            latex_options = c( "hold_position")) %>%
  kableExtra::landscape()
knitr::kable(gendiv.x.F, digits = 4,
             caption = "Genetic diversity for all X-linked loci: Only females") %>%
  kableExtra::kable_styling(full_width = FALSE,font_size = 5,
                            latex_options = c( "hold_position")) %>%
  kableExtra::landscape()
knitr::kable(gendiv.x.M, digits = 4,
             caption = "Genetic diversity for all X-linked loci: Only males") %>%
  kableExtra::kable_styling(full_width = FALSE,font_size = 5,
```

```
                                latex_options = c( "hold_position")) %>%
  kableExtra::landscape()
```

#### 8.11.1.1 Genetic diversity

Table 22: Genetic diversity for all loci

| | Costa.Rica | Brazil | Caribbean.Sea | Gulf.of.Mexico | Western.North.Atlantic | South.Africa | Mozambique | Reunion | Seychelles | Arabian.Sea | Sri.Lanka | Thailand | Indonesia | Papua.New.Guinea | Australia |
|---|---|---|---|---|---|---|---|---|---|---|---|---|---|---|---|
| ar | 1.0610 | 1.0950 | 1.0640 | 1.0860 | 1.0850 | 1.1120 | 1.0870 | 1.1090 | 1.1120 | 1.1140 | 1.1110 | 1.1100 | 1.0960 | 1.1050 | 1.1160 |
| size | 15.6540 | 49.0870 | 1.9750 | 36.0400 | 7.8830 | 21.7470 | 10.2580 | 27.3810 | 35.4700 | 14.8430 | 11.8850 | 4.9650 | 3.9590 | 8.9060 | 438.3460 |
| obs__het | 0.0360 | 0.0520 | 0.0490 | 0.0480 | 0.0500 | 0.0600 | 0.0560 | 0.0590 | 0.0600 | 0.0620 | 0.0610 | 0.0660 | 0.0610 | 0.0590 | 0.0610 |
| exp__het | 0.0390 | 0.0560 | 0.0380 | 0.0530 | 0.0500 | 0.0620 | 0.0600 | 0.0620 | 0.0620 | 0.0630 | 0.0610 | 0.0590 | 0.0540 | 0.0580 | 0.0640 |
| uexp__het | 0.0410 | 0.0570 | 0.0510 | 0.0530 | 0.0540 | 0.0630 | 0.0640 | 0.0630 | 0.0630 | 0.0650 | 0.0640 | 0.0660 | 0.0610 | 0.0620 | 0.0640 |
| fis | 0.0450 | 0.0480 | -0.2930 | 0.0500 | -0.0250 | 0.0240 | 0.0520 | 0.0400 | 0.0260 | 0.0040 | -0.0050 | -0.1080 | -0.1360 | -0.0270 | 0.0430 |
| hwe__glb | 1.0000 | 1.0000 | 1.0000 | 1.0000 | 1.0000 | 1.0000 | 1.0000 | 1.0000 | 1.0000 | 1.0000 | 1.0000 | 1.0000 | 1.0000 | 1.0000 | 0.0000 |
| hwe__hom | 1.0000 | 1.0000 | 1.0000 | 1.0000 | 1.0000 | 1.0000 | 1.0000 | 1.0000 | 1.0000 | 1.0000 | 1.0000 | 1.0000 | 1.0000 | 1.0000 | 1.0000 |
| hwe__het | 1.0000 | 1.0000 | 1.0000 | 1.0000 | 1.0000 | 1.0000 | 1.0000 | 1.0000 | 1.0000 | 1.0000 | 1.0000 | 1.0000 | 1.0000 | 1.0000 | 0.0000 |
| fis__lo | -0.0090 | 0.0330 | -1.0000 | 0.0310 | -0.1450 | -0.0090 | -0.0350 | 0.0100 | 0.0020 | -0.0480 | -0.0770 | -0.3680 | -0.5470 | -0.1260 | 0.0370 |
| fis__hi | 0.0440 | 0.0520 | -0.2930 | 0.0530 | -0.0340 | 0.0180 | 0.0450 | 0.0370 | 0.0250 | -0.0030 | -0.0150 | -0.1080 | -0.1360 | -0.0370 | 0.0430 |
| ar__lo | 1.0130 | 1.0850 | 1.0330 | 1.0360 | 1.0290 | 1.0510 | 0.9460 | 1.0460 | 1.0600 | 1.0480 | 1.0490 | 1.0520 | 1.0470 | 1.0430 | 1.1050 |
| ar__hi | 1.0710 | 1.1070 | 1.0920 | 1.0990 | 1.1020 | 1.1220 | 1.1190 | 1.1210 | 1.1210 | 1.1280 | 1.1280 | 1.1290 | 1.1190 | 1.1180 | 1.1270 |
| fis.hierfstat | 0.0750 | 0.0576 | -0.0481 | 0.0638 | 0.0369 | 0.0465 | 0.0959 | 0.0572 | 0.0398 | 0.0370 | 0.0356 | -0.0069 | -0.0098 | 0.0285 | 0.0440 |
| fis.CI.low | 0.0785 | 0.0687 | -0.0302 | 0.0736 | 0.0321 | 0.0454 | 0.1015 | 0.0530 | 0.0328 | 0.0261 | 0.0130 | -0.0302 | -0.0282 | 0.0165 | 0.0387 |
| fis.CI.high | 0.1389 | 0.1058 | 0.1227 | 0.1164 | 0.0998 | 0.0787 | 0.1564 | 0.0888 | 0.0611 | 0.0675 | 0.0610 | 0.0374 | 0.0545 | 0.0702 | 0.0544 |

Table 23: Genetic diversity for all X-linked loci: Only females

| | Costa.Rica | Gulf.of.Mexico | Western.North.Atlantic | South.Africa | Mozambique | Reunion | Seychelles | Arabian.Sea | Sri.Lanka | Thailand | Papua.New.Guinea | Australia | Japan | Fiji |
|---|---|---|---|---|---|---|---|---|---|---|---|---|---|---|
| ar | 1.0190 | 1.0530 | 1.0470 | 1.0920 | 1.0920 | 1.0980 | 1.0980 | 1.0990 | 1.0910 | 1.0990 | 1.0780 | 1.1000 | 1.0920 | 1.0850 |
| size | 8.8450 | 17.7360 | 5.9120 | 8.9730 | 4.9590 | 12.8650 | 27.7160 | 8.8920 | 6.9320 | 2.9930 | 3.9860 | 191.2500 | 24.7090 | 21.3650 |
| obs_het | 0.0110 | 0.0220 | 0.0250 | 0.0410 | 0.0490 | 0.0470 | 0.0460 | 0.0420 | 0.0400 | 0.0560 | 0.0470 | 0.0440 | 0.0440 | 0.0420 |
| exp_het | 0.0180 | 0.0220 | 0.0210 | 0.0410 | 0.0390 | 0.0420 | 0.0450 | 0.0450 | 0.0410 | 0.0460 | 0.0340 | 0.0450 | 0.0420 | 0.0390 |
| uexp_het | 0.0120 | 0.0220 | 0.0230 | 0.0440 | 0.0440 | 0.0440 | 0.0460 | 0.0480 | 0.0450 | 0.0550 | 0.0390 | 0.0450 | 0.0430 | 0.0400 |
| fis | -0.0400 | -0.0280 | -0.1550 | -0.0050 | -0.2070 | -0.0870 | -0.0200 | -0.0120 | -0.0170 | -0.2090 | -0.3050 | 0.0110 | -0.0100 | -0.0470 |
| hwe_glb | 1.0000 | 1.0000 | 1.0000 | 1.0000 | 1.0000 | 1.0000 | 1.0000 | 1.0000 | 1.0000 | 1.0000 | 1.0000 | 1.0000 | 1.0000 | 1.0000 |
| hwe_hom | 1.0000 | 1.0000 | 1.0000 | 1.0000 | 1.0000 | 1.0000 | 1.0000 | 1.0000 | 1.0000 | 1.0000 | 1.0000 | 1.0000 | 1.0000 | 1.0000 |
| hwe_het | 1.0000 | 1.0000 | 1.0000 | 1.0000 | 1.0000 | 1.0000 | 1.0000 | 1.0000 | 1.0000 | 1.0000 | 1.0000 | 1.0000 | 1.0000 | 1.0000 |
| fis_lo | -0.3150 | -0.0970 | -0.5200 | -0.2080 | -0.5220 | -0.2140 | -0.0810 | -0.1710 | -0.3110 | -1.0000 | -0.7940 | -0.0030 | -0.0820 | -0.1150 |
| fis_hi | 0.1040 | -0.0040 | -0.0930 | 0.0640 | -0.1650 | -0.0430 | 0.0130 | 0.0650 | 0.1780 | -0.1810 | -0.2820 | 0.0250 | 0.0130 | -0.0200 |
| ar_lo | 1.0000 | 1.0200 | 1.0200 | 1.0470 | 1.0470 | 1.0610 | 1.0610 | 1.0470 | 1.0470 | 1.0340 | 1.0470 | 1.0680 | 1.0540 | 1.0410 |
| ar_hi | 1.0470 | 1.0880 | 1.0810 | 1.1350 | 1.1150 | 1.1280 | 1.1280 | 1.1420 | 1.1220 | 1.1220 | 1.1010 | 1.1420 | 1.1220 | 1.1280 |
| fis.hierfstat | 0.0188 | 0.0004 | -0.0666 | 0.0478 | -0.1052 | -0.0476 | -0.0022 | 0.0447 | 0.0573 | -0.0648 | -0.1848 | 0.0137 | 0.0087 | -0.0229 |
| fis.CI.low | -0.0323 | -0.1037 | -0.2234 | -0.1210 | -0.3056 | -0.1582 | -0.1452 | -0.0241 | -0.0408 | -0.3333 | -0.5000 | -0.0411 | -0.1585 | -0.1671 |
| fis.CI.high | 0.1157 | 0.1489 | 0.0141 | 0.2515 | 0.0690 | 0.0071 | 0.1465 | 0.2382 | 0.2372 | 0.2882 | -0.0485 | 0.1247 | 0.1417 | 0.0873 |

Table 24: Genetic diversity for all X-linked loci: Only males

| | Costa.Rica | Caribbean.Sea | Gulf.of.Mexico | Western.North.Atlantic | South.Africa | Mozambique | Reunion | Seychelles | Arabian.Sea | Sri.Lanka | Thailand | Indonesia | Papua.New.Guinea | Australia | Japan |
|---|---|---|---|---|---|---|---|---|---|---|---|---|---|---|---|
| ar | 0.994 | 0.999 | 1.0130 | 1.0440 | 1.0370 | 0.9950 | 1.0280 | 1.0360 | 1.0460 | 1.0130 | 1.002 | 1.0230 | 1.0330 | 1.0380 | 1.0180 |
| size | 6.696 | 1.932 | 18.3450 | 1.9530 | 11.6350 | 5.5340 | 14.5680 | 7.7500 | 5.8510 | 4.7970 | 1.946 | 2.9530 | 3.9460 | 218.2910 | 18.3110 |
| obs_het | 0.001 | 0.003 | 0.0070 | 0.0340 | 0.0100 | 0.0050 | 0.0030 | 0.0090 | 0.0190 | 0.0010 | 0.003 | 0.0050 | 0.0120 | 0.0080 | 0.0030 |
| exp_het | 0.009 | 0.013 | 0.0170 | 0.0490 | 0.0390 | 0.0450 | 0.0330 | 0.0400 | 0.0380 | 0.0290 | 0.023 | 0.0250 | 0.0280 | 0.0410 | 0.0270 |
| uexp_het | 0.010 | 0.017 | 0.0180 | 0.0470 | 0.0410 | 0.0420 | 0.0340 | 0.0430 | 0.0420 | 0.0320 | 0.021 | 0.0300 | 0.0320 | 0.0410 | 0.0280 |
| fis | 0.731 | 0.667 | 0.2970 | -0.0260 | 0.4100 | 0.7590 | 0.7650 | 0.6730 | 0.4300 | 0.9070 | 0.733 | 0.7330 | 0.4700 | 0.3660 | 0.8670 |
| hwe_glb | 1.000 | 1.000 | 1.0000 | 1.0000 | 1.0000 | 1.0000 | 1.0000 | 1.0000 | 1.0000 | 1.0000 | 1.000 | 1.0000 | 1.0000 | 0.0000 | 1.0000 |
| hwe_hom | 1.000 | 1.000 | 1.0000 | 1.0000 | 1.0000 | 1.0000 | 1.0000 | 1.0000 | 1.0000 | 1.0000 | 1.000 | 1.0000 | 1.0000 | 1.0000 | 1.0000 |
| hwe_het | 1.000 | 1.000 | 1.0000 | 1.0000 | 1.0000 | 1.0000 | 1.0000 | 1.0000 | 1.0000 | 1.0000 | 1.000 | 1.0000 | 1.0000 | 0.0000 | 1.0000 |
| fis_lo | 0.576 | -1.000 | 0.1470 | -1.0000 | 0.3640 | 0.5660 | 0.6990 | 0.5300 | -0.0180 | 0.7620 | -1.000 | -1.0000 | -0.4290 | 0.3680 | 0.6780 |
| fis_hi | 1.000 | 0.667 | 0.7460 | -0.0260 | 0.6110 | 1.0000 | 0.9090 | 0.8110 | 0.6300 | 1.0000 | 0.733 | 1.0000 | 1.0000 | 0.4670 | 0.9740 |
| ar_lo | 0.953 | 0.959 | 0.9530 | 0.9800 | 0.9660 | 0.9110 | 0.9730 | 0.9590 | 0.9800 | 0.9390 | 0.973 | 0.9860 | 0.9800 | 1.0070 | 0.9660 |
| ar_hi | 1.014 | 1.027 | 1.0680 | 1.0740 | 1.0680 | 1.0410 | 1.0540 | 1.0740 | 1.0880 | 1.0340 | 1.027 | 1.0540 | 1.0680 | 1.0740 | 1.0610 |
| fis.hierfstat | 0.750 | 0.750 | 0.3168 | 0.2308 | 0.4352 | 0.7804 | 0.7737 | 0.6971 | 0.4820 | 0.9167 | 0.800 | 0.7778 | 0.5403 | 0.3672 | 0.8728 |
| fis.CI.low | 0.000 | 0.000 | 0.3169 | 0.0000 | 0.5675 | 0.7849 | 0.8262 | 0.6609 | 0.3721 | 0.8571 | 0.500 | 0.6250 | 0.3334 | 0.7566 | 0.7840 |
| fis.CI.high | 1.000 | 1.000 | 0.7737 | 0.6667 | 0.8695 | 0.9802 | 0.9635 | 0.9264 | 0.7569 | 1.0000 | 1.000 | 1.0000 | 0.8800 | 0.8590 | 0.9707 |

```r
BS.dartcap.x.F.tidy <- BS.dartcap.x.tidy[BS.dartcap.x.tidy$INDIVIDUALS %in%
                                           gl.x.F.filtered$ind.names,]
BS.dartcap.x.F.tidy <- BS.dartcap.x.F.tidy[!BS.dartcap.x.F.tidy$POP_ID %in%
                                            c("Indonesia"), ]
BS.dartcap.x.F.tidy$POP_ID <- factor(BS.dartcap.x.F.tidy$POP_ID)
pi.sum.X.F <- radiator::pi(
  data = BS.dartcap.x.F.tidy,
  read.length = 70,
  parallel.core = parallel::detectCores() - 1,
  path.folder = NULL,
  verbose = TRUE
)

BS.dartcap.x.M.tidy <- BS.dartcap.x.tidy[BS.dartcap.x.tidy$INDIVIDUALS %in%
                                           gl.x.M.filtered$ind.names,]
BS.dartcap.x.M.tidy <- BS.dartcap.x.M.tidy[!BS.dartcap.x.M.tidy$POP_ID %in%
                                            c("Indonesia"), ]
BS.dartcap.x.M.tidy$POP_ID <- factor(BS.dartcap.x.M.tidy$POP_ID)

pi.sum.X.M <- radiator::pi(
  data = BS.dartcap.x.M.tidy,
  read.length = 70,
  parallel.core = parallel::detectCores() - 1,
  path.folder = NULL,
  verbose = TRUE
)

save(pi.sum.X.F, pi.sum.X.M, file = "DArTcap_ALL_pi_Sites_X_FM.Rdata")
```

```r
load("DArTcap_ALL_pi_FIS_Sites2.Rdata")
load("DArTcap_ALL_pi_Sites_X_FM.Rdata")
pi.sum.site$boxplot.pi
```

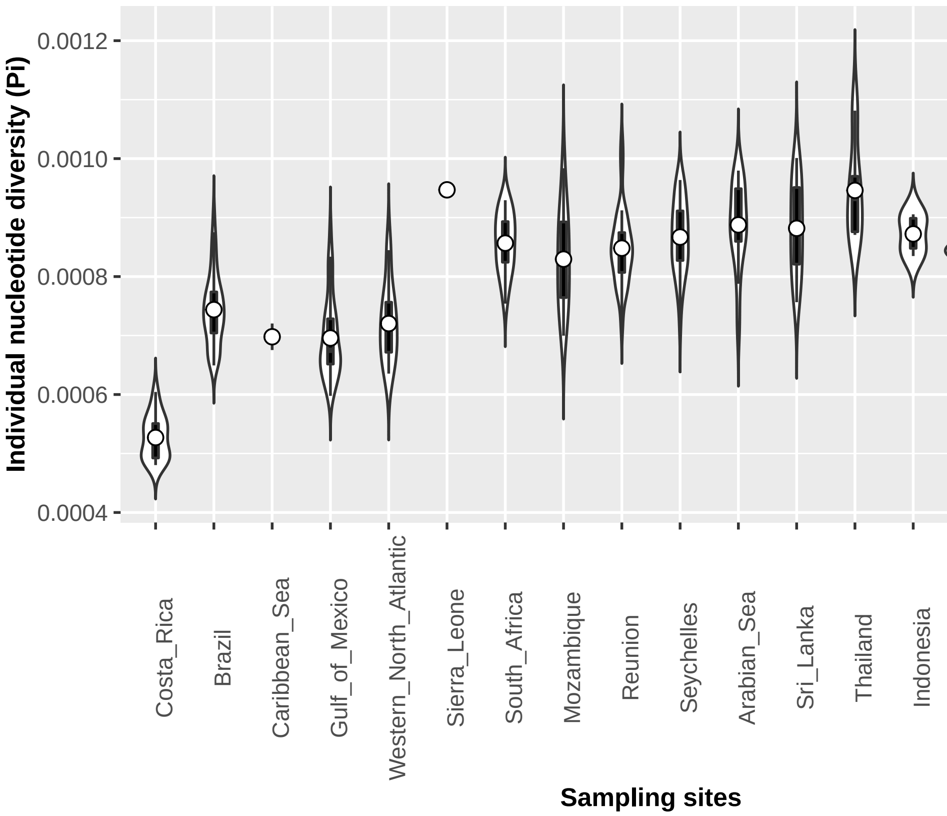


#### 8.11.1.1.1 Nucleotide diversity

```
pi.sum.X.F$boxplot.pi
```

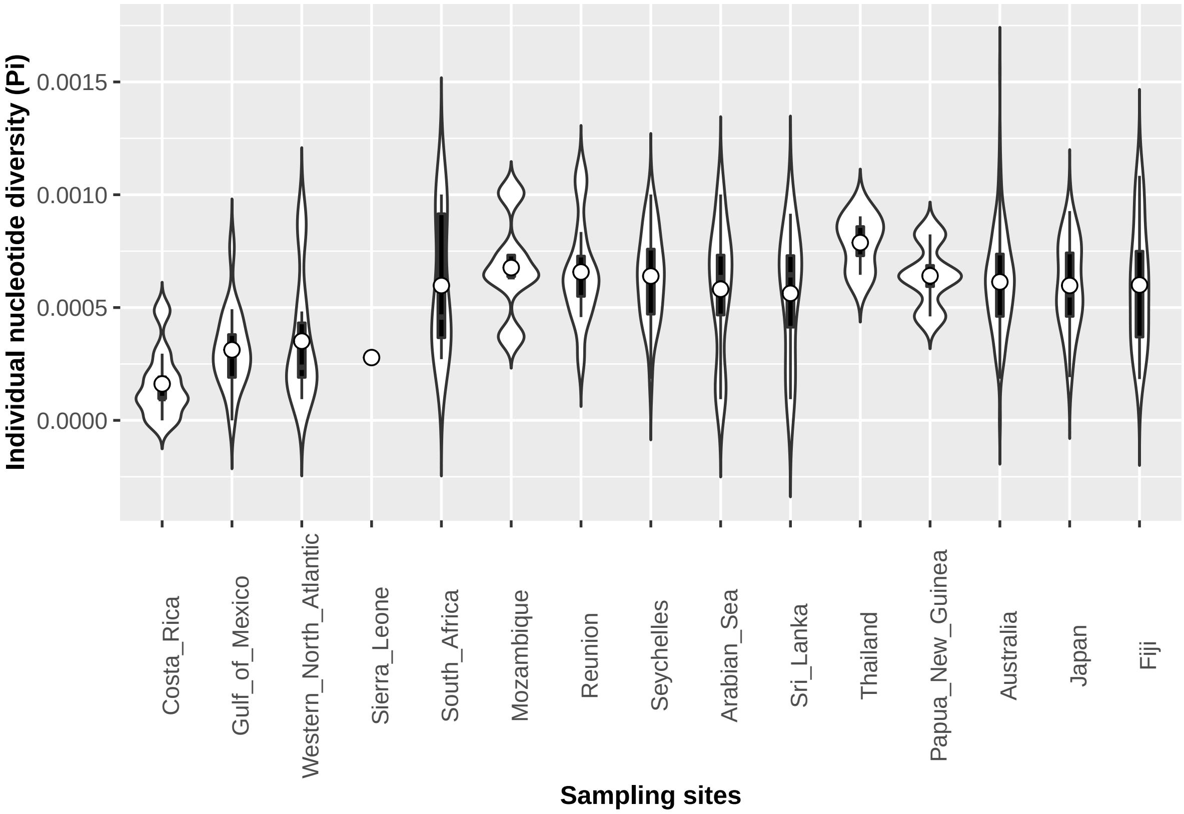


```
pi.sum.X.M$boxplot.pi
```

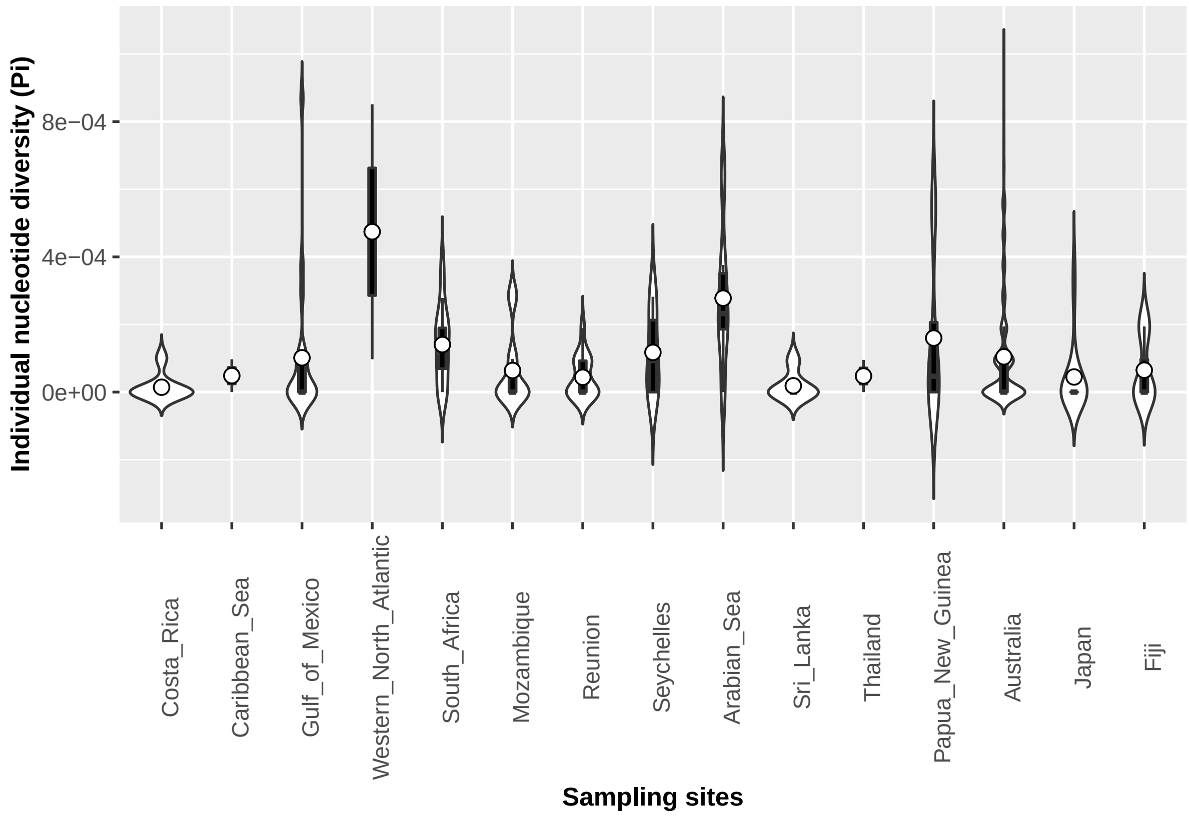


```
Fst.gt <-
  strataG::popStructTest(
    gt.all.neutral,
    nrep = 1000,
    stats = "all",
    type = "pairwise",
    keep.null = FALSE,
    quietly = TRUE,
    max.cores = parallel::detectCores() - 1,
    write.output = FALSE
  )

strata <- gt.x.F.filtered@schemes$Site
names(strata) <- gt.x.F.filtered@schemes$id
strataG::setStrata(gt.x.F.filtered) <- strata

Fst.x.F.filtered <-
  strataG::popStructTest(
    gt.x.F.filtered,
    nrep = 1000,
    stats = "all",
    type = "pairwise",
    keep.null = FALSE,
    quietly = TRUE,
    max.cores = parallel::detectCores() - 1,
    write.output = FALSE
  )

strata <- gt.x.M.filtered@schemes$Site
names(strata) <- gt.x.M.filtered@schemes$id
```

```r
strataG::setStrata(gt.x.M.filtered) <- strata

Fst.x.M.filtered <-
  strataG::popStructTest(
    gt.x.M.filtered,
    nrep = 1000,
    stats = "all",
    type = "pairwise",
    keep.null = FALSE,
    quietly = TRUE,
    max.cores = parallel::detectCores() - 1,
    write.output = FALSE
  )
save(Fst.x.F.filtered, Fst.x.M.filtered,
     file = "DArTcap_ALL_FST_X_Sites.Rdata")
```

```r
load("DArTcap_ALL_FST_Sites.Rdata")
load("DArTcap_ALL_FST_X_Sites.Rdata")

names.1 <- paste0(Fst.gt$pairwise$result$strata.1,"_",
                  Fst.gt$pairwise$result$strata.2)
names.2 <- paste0(Fst.x.F.filtered$pairwise$result$strata.1,"_",
                  Fst.x.F.filtered$pairwise$result$strata.2)
names.3 <- paste0(Fst.x.M.filtered$pairwise$result$strata.1,"_",
                  Fst.x.M.filtered$pairwise$result$strata.2)

result.1 <- Fst.gt$pairwise$result
result.1$names.comb <- names.1
result.2 <- Fst.x.F.filtered$pairwise$result
result.2$names.comb <- names.2
result.3 <- Fst.x.M.filtered$pairwise$result
result.3$names.comb <- names.3

data <- left_join(result.1,result.2, by = "names.comb" ) %>%
  left_join(., result.3, by = "names.comb")


pwFst <- data[,c(1:4,11:12, 24:25,32:33, 44:45,52:53)]
colnames(pwFst) <- c("strata.1", "strata.2", "n.auDNA.1", "n.auDNA.2",
                     "Fst.auDNA", "Fst.auDNA.Pval",
                     "n.xDNA.F.1", "n.xDNA.F.2", "Fst.xDNA.F", "Fst.xDNA.F.Pval",
                     "n.xDNA.M.1", "n.xDNA.M.2", "Fst.xDNA.M", "Fst.xDNA.M.Pval")

kableExtra::kbl(pwFst, digits = 4,
  caption = "Pairwise Fst for autosomal and X-linked markers in females and males",
  longtable = TRUE, booktabs = TRUE) %>%
  kableExtra::kable_styling(full_width = FALSE,font_size = 5,
                            latex_options = c("repeat_header", "hold_position")) %>%
  kableExtra::landscape()
```

**8.11.1.2 nuFst - xFst**

Table 25: Pairwise Fst for autosomal and X-linked markers in females and males

| strata.1 | strata.2 | n.auDNA.1 | n.auDNA.2 | Fst.auDNA | Fst.auDNA.Pval | n.xDNA.F.1 | n.xDNA.F.2 | Fst.xDNA.F | Fst.xDNA.F.Pval | n.xDNA.M.1 | n.xDNA.M.2 | Fst.xDNA.M | Fst.xDNA.M.Pval |
|---|---|---|---|---|---|---|---|---|---|---|---|---|---|
| Arabian Sea | Australia | 15 | 444 | 0.0018 | 0.0220 | 9 | 193 | -0.0076 | 0.7230 | 6 | 225 | 0.0028 | 0.4074 |
| Arabian Sea | Brazil | 15 | 50 | 0.6538 | 0.0010 | NA | NA | NA | NA | NA | NA | NA | NA |
| Arabian Sea | Caribbean Sea | 15 | 2 | 0.6376 | 0.0166 | NA | NA | NA | NA | 6 | 2 | 0.6899 | 0.0350 |
| Arabian Sea | Costa Rica | 15 | 16 | 0.6703 | 0.0010 | 9 | 9 | 0.7611 | 0.0010 | 6 | 7 | 0.7948 | 0.0020 |
| Arabian Sea | Fiji | 15 | 25 | 0.0273 | 0.0010 | 9 | 22 | 0.0588 | 0.0010 | 6 | 3 | NA | NA |
| Arabian Sea | Gulf of Mexico | 15 | 37 | 0.6674 | 0.0010 | 9 | 18 | 0.7427 | 0.0010 | 6 | 19 | 0.8008 | 0.0010 |
| Arabian Sea | Indonesia | 15 | 4 | 0.0124 | 0.0241 | 9 | 1 | -0.0347 | 0.6893 | 6 | 3 | 0.0257 | 0.3027 |
| Arabian Sea | Japan | 15 | 44 | 0.0306 | 0.0010 | 9 | 25 | 0.0545 | 0.0120 | 6 | 19 | 0.1678 | 0.0010 |
| Arabian Sea | Mozambique | 15 | 11 | 0.0041 | 0.0380 | 9 | 5 | 0.0132 | 0.2368 | 6 | 6 | -0.0377 | 0.7423 |
| Arabian Sea | Papua New Guinea | 15 | 9 | 0.0067 | 0.0060 | 9 | 4 | 0.0284 | 0.1588 | 6 | 4 | -0.0878 | 0.9590 |
| Arabian Sea | Reunion | 15 | 28 | 0.0019 | 0.1419 | 9 | 13 | -0.0053 | 0.6134 | 6 | 15 | 0.0362 | 0.1653 |
| Arabian Sea | Seychelles | 15 | 36 | 0.0011 | 0.1848 | 9 | 28 | 0.0026 | 0.3776 | 6 | 8 | -0.0040 | 0.4775 |
| Arabian Sea | Sierra Leone | 15 | 1 | 0.1582 | 1.0000 | 9 | 1 | 0.1749 | 0.2306 | NA | NA | NA | NA |
| Arabian Sea | South Africa | 15 | 22 | 0.0020 | 0.0909 | 9 | 9 | -0.0312 | 0.9760 | 6 | 12 | -0.0313 | 0.7960 |
| Arabian Sea | Sri Lanka | 15 | 12 | -0.0014 | 0.7622 | 9 | 7 | -0.0359 | 0.9610 | 6 | 5 | 0.0532 | 0.1291 |
| Arabian Sea | Thailand | 15 | 5 | -0.0019 | 0.7233 | 9 | 3 | -0.0366 | 0.8412 | 6 | 2 | NA | NA |
| Arabian Sea | Western North Atlantic | 15 | 8 | 0.6495 | 0.0010 | 9 | 6 | 0.7067 | 0.0010 | 6 | 2 | 0.6518 | 0.0390 |
| Australia | Brazil | 444 | 50 | 0.6359 | 0.0010 | NA | NA | NA | NA | NA | NA | NA | NA |
| Australia | Caribbean Sea | 444 | 2 | 0.6353 | 0.0040 | NA | NA | NA | NA | 225 | 2 | 0.6879 | 0.0021 |
| Australia | Costa Rica | 444 | 16 | 0.6264 | 0.0010 | 193 | 9 | 0.6928 | 0.0010 | 225 | 7 | 0.7135 | 0.0010 |
| Australia | Fiji | 444 | 25 | 0.0310 | 0.0010 | 193 | 22 | 0.0599 | 0.0010 | 225 | 3 | NA | NA |
| Australia | Gulf of Mexico | 444 | 37 | 0.6416 | 0.0010 | 193 | 18 | 0.6812 | 0.0010 | 225 | 19 | 0.7075 | 0.0010 |
| Australia | Indonesia | 444 | 4 | NA | NA | 193 | 1 | NA | NA | 225 | 3 | 0.0539 | 0.1994 |
| Australia | Japan | 444 | 44 | 0.0287 | 0.0010 | 193 | 25 | 0.0304 | 0.0010 | 225 | 19 | 0.1152 | 0.0010 |
| Australia | Mozambique | 444 | 11 | 0.0044 | 0.0010 | 193 | 5 | 0.0286 | 0.0682 | 225 | 6 | 0.0261 | 0.1758 |
| Australia | Papua New Guinea | 444 | 9 | 0.0018 | 0.1179 | 193 | 4 | 0.0298 | 0.1280 | 225 | 4 | -0.0225 | 0.6108 |
| Australia | Reunion | 444 | 28 | 0.0052 | 0.0010 | 193 | 13 | 0.0137 | 0.0440 | 225 | 15 | 0.0293 | 0.0280 |
| Australia | Seychelles | 444 | 36 | 0.0015 | 0.0010 | 193 | 28 | 0.0012 | 0.3197 | 225 | 8 | 0.0341 | 0.0785 |
| Australia | Sierra Leone | 444 | 1 | 0.1572 | 1.0000 | 193 | 1 | 0.1869 | 0.0107 | NA | NA | NA | NA |
| Australia | South Africa | 444 | 22 | 0.0008 | 0.1209 | 193 | 9 | -0.0152 | 0.9510 | 225 | 12 | 0.0176 | 0.1251 |
| Australia | Sri Lanka | 444 | 12 | 0.0005 | 0.3127 | 193 | 7 | -0.0118 | 0.7946 | 225 | 5 | 0.0363 | 0.1721 |
| Australia | Thailand | 444 | 5 | 0.0014 | 0.3150 | 193 | 3 | -0.0028 | 0.4341 | 225 | 2 | NA | NA |
| Australia | Western North Atlantic | 444 | 8 | 0.6385 | 0.0010 | 193 | 6 | 0.6809 | 0.0010 | 225 | 2 | 0.6803 | 0.0021 |
| Brazil | Caribbean Sea | 50 | 2 | 0.0016 | 0.6250 | NA | NA | NA | NA | NA | NA | NA | NA |
| Brazil | Costa Rica | 50 | 16 | 0.3484 | 0.0010 | NA | NA | NA | NA | NA | NA | NA | NA |
| Brazil | Fiji | 50 | 25 | 0.6521 | 0.0010 | NA | NA | NA | NA | NA | NA | NA | NA |
| Brazil | Gulf of Mexico | 50 | 37 | 0.0049 | 0.0010 | NA | NA | NA | NA | NA | NA | NA | NA |
| Brazil | Indonesia | 50 | 4 | 0.6610 | 0.0016 | NA | NA | NA | NA | NA | NA | NA | NA |
| Brazil | Japan | 50 | 44 | 0.6580 | 0.0010 | NA | NA | NA | NA | NA | NA | NA | NA |
| Brazil | Mozambique | 50 | 11 | 0.6532 | 0.0010 | NA | NA | NA | NA | NA | NA | NA | NA |
| Brazil | Papua New Guinea | 50 | 9 | 0.6576 | 0.0010 | NA | NA | NA | NA | NA | NA | NA | NA |
| Brazil | Reunion | 50 | 28 | 0.6518 | 0.0010 | NA | NA | NA | NA | NA | NA | NA | NA |
| Brazil | Seychelles | 50 | 36 | 0.6504 | 0.0010 | NA | NA | NA | NA | NA | NA | NA | NA |
| Brazil | Sierra Leone | 50 | 1 | 0.6231 | 1.0000 | NA | NA | NA | NA | NA | NA | NA | NA |
| Brazil | South Africa | 50 | 22 | 0.6522 | 0.0010 | NA | NA | NA | NA | NA | NA | NA | NA |
| Brazil | Sri Lanka | 50 | 12 | 0.6543 | 0.0010 | NA | NA | NA | NA | NA | NA | NA | NA |
| Brazil | Thailand | 50 | 5 | 0.6564 | 0.0012 | NA | NA | NA | NA | NA | NA | NA | NA |
| Brazil | Western North Atlantic | 50 | 8 | 0.0037 | 0.0884 | NA | NA | NA | NA | NA | NA | NA | NA |
| Caribbean Sea | Costa Rica | 2 | 16 | 0.4287 | 0.0845 | NA | NA | NA | NA | 2 | 7 | 0.6704 | 0.0380 |
| Caribbean Sea | Fiji | 2 | 25 | 0.6389 | 0.0341 | NA | NA | NA | NA | 2 | 3 | 0.7248 | 0.0939 |
| Caribbean Sea | Gulf of Mexico | 2 | 37 | -0.0020 | 0.5714 | NA | NA | NA | NA | 2 | 19 | -0.1106 | 0.7202 |
| Caribbean Sea | Indonesia | 2 | 4 | 0.6592 | 0.0772 | NA | NA | NA | NA | 2 | 3 | 0.7086 | 0.1089 |
| Caribbean Sea | Japan | 2 | 44 | 0.6537 | 0.0110 | NA | NA | NA | NA | 2 | 19 | 0.7804 | 0.0100 |

Table 25: Pairwise Fst for autosomal and X-linked markers in females and males *(continued)*

| strata.1 | strata.2 | n.auDNA.1 | n.auDNA.2 | Fst.auDNA | Fst.auDNA.Pval | n.xDNA.F.1 | n.xDNA.F.2 | Fst.xDNA.F | Fst.xDNA.F.Pval | n.xDNA.M.1 | n.xDNA.M.2 | Fst.xDNA.M | Fst.xDNA.M.Pval |
|---|---|---|---|---|---|---|---|---|---|---|---|---|---|
| Caribbean Sea | Mozambique | 2 | 11 | 0.6367 | 0.0445 | NA | NA | NA | NA | 2 | 6 | 0.6568 | 0.0815 |
| Caribbean Sea | Papua New Guinea | 2 | 9 | 0.6504 | 0.0216 | NA | NA | NA | NA | 2 | 4 | 0.7336 | 0.0679 |
| Caribbean Sea | Reunion | 2 | 28 | 0.6401 | 0.0079 | NA | NA | NA | NA | 2 | 15 | 0.7245 | 0.0061 |
| Caribbean Sea | Seychelles | 2 | 36 | 0.6409 | 0.0046 | NA | NA | NA | NA | 2 | 8 | 0.6816 | 0.0161 |
| Caribbean Sea | Sierra Leone | 2 | 1 | 0.6360 | 0.3417 | NA | NA | NA | NA | NA | NA | NA | NA |
| Caribbean Sea | South Africa | 2 | 22 | 0.6396 | 0.0086 | NA | NA | NA | NA | 2 | 12 | 0.6793 | 0.0061 |
| Caribbean Sea | Sri Lanka | 2 | 12 | 0.6407 | 0.0192 | NA | NA | NA | NA | 2 | 5 | 0.7267 | 0.0529 |
| Caribbean Sea | Thailand | 2 | 5 | 0.6397 | 0.0605 | NA | NA | NA | NA | 2 | 2 | 0.7647 | 0.3187 |
| Caribbean Sea | Western North Atlantic | 2 | 8 | -0.0077 | 1.0000 | NA | NA | NA | NA | 2 | 2 | -0.1220 | 1.0000 |
| Costa Rica | Fiji | 16 | 25 | 0.6594 | 0.0010 | 9 | 22 | 0.7623 | 0.0010 | 7 | 3 | 0.8562 | 0.0063 |
| Costa Rica | Gulf of Mexico | 16 | 37 | 0.3692 | 0.0010 | 9 | 18 | 0.5201 | 0.0010 | 7 | 19 | 0.6018 | 0.0010 |
| Costa Rica | Indonesia | 16 | 4 | 0.7066 | 0.0016 | 9 | 1 | 0.9021 | 0.1250 | 7 | 3 | 0.8386 | 0.0091 |
| Costa Rica | Japan | 16 | 44 | 0.6609 | 0.0010 | 9 | 25 | 0.7547 | 0.0010 | 7 | 19 | 0.8228 | 0.0010 |
| Costa Rica | Mozambique | 16 | 11 | 0.6766 | 0.0010 | 9 | 5 | 0.8143 | 0.0010 | 7 | 6 | 0.7916 | 0.0021 |
| Costa Rica | Papua New Guinea | 16 | 9 | 0.6888 | 0.0010 | 9 | 4 | 0.8405 | 0.0020 | 7 | 4 | 0.8378 | 0.0040 |
| Costa Rica | Reunion | 16 | 28 | 0.6579 | 0.0010 | 9 | 13 | NA | NA | 7 | 15 | 0.7800 | 0.0010 |
| Costa Rica | Seychelles | 16 | 36 | 0.6532 | 0.0010 | 9 | 28 | 0.7273 | 0.0010 | 7 | 8 | 0.7817 | 0.0010 |
| Costa Rica | Sierra Leone | 16 | 1 | 0.7013 | 1.0000 | 9 | 1 | 0.8934 | 0.1354 | NA | NA | NA | NA |
| Costa Rica | South Africa | 16 | 22 | 0.6623 | 0.0010 | 9 | 9 | 0.7768 | 0.0010 | 7 | 12 | 0.7505 | 0.0010 |
| Costa Rica | Sri Lanka | 16 | 12 | 0.6765 | 0.0010 | 9 | 7 | 0.7817 | 0.0010 | 7 | 5 | 0.8244 | 0.0010 |
| Costa Rica | Thailand | 16 | 5 | 0.6957 | 0.0012 | 9 | 3 | 0.8105 | 0.0060 | 7 | 2 | 0.8787 | 0.0326 |
| Costa Rica | Western North Atlantic | 16 | 8 | 0.3964 | 0.0011 | 9 | 6 | 0.5826 | 0.0020 | 7 | 2 | 0.6580 | 0.0320 |
| Fiji | Gulf of Mexico | 25 | 37 | 0.6636 | 0.0010 | 22 | 18 | 0.7498 | 0.0010 | 3 | 19 | 0.8276 | 0.0020 |
| Fiji | Indonesia | 25 | 4 | NA | NA | 22 | 1 | 0.0951 | 0.1054 | 3 | 3 | 0.0982 | 0.3397 |
| Fiji | Japan | 25 | 44 | 0.0617 | 0.0010 | 22 | 25 | 0.1148 | 0.0010 | 3 | 19 | NA | NA |
| Fiji | Mozambique | 25 | 11 | 0.0333 | 0.0010 | 22 | 5 | 0.1609 | 0.0010 | 3 | 6 | NA | NA |
| Fiji | Papua New Guinea | 25 | 9 | 0.0313 | 0.0010 | 22 | 4 | 0.0848 | 0.0051 | 3 | 4 | 0.0120 | 0.4436 |
| Fiji | Reunion | 25 | 28 | 0.0331 | 0.0010 | 22 | 13 | 0.0950 | 0.0010 | 3 | 15 | 0.0166 | 0.3347 |
| Fiji | Seychelles | 25 | 36 | 0.0318 | 0.0010 | 22 | 28 | 0.0723 | 0.0010 | 3 | 8 | NA | NA |
| Fiji | Sierra Leone | 25 | 1 | 0.1610 | 1.0000 | 22 | 1 | 0.3115 | 0.2784 | NA | NA | NA | NA |
| Fiji | South Africa | 25 | 22 | 0.0304 | 0.0010 | 22 | 9 | 0.0548 | 0.0040 | 3 | 12 | 0.0589 | 0.1608 |
| Fiji | Sri Lanka | 25 | 12 | 0.0318 | 0.0010 | 22 | 7 | 0.0654 | 0.0020 | 3 | 5 | 0.1228 | 0.0480 |
| Fiji | Thailand | 25 | 5 | 0.0255 | 0.0072 | 22 | 3 | 0.0657 | 0.0342 | 3 | 2 | -0.2218 | 0.9171 |
| Fiji | Western North Atlantic | 25 | 8 | 0.6477 | 0.0010 | 22 | 6 | 0.7318 | 0.0010 | 3 | 2 | 0.6637 | 0.1044 |
| Gulf of Mexico | Indonesia | 37 | 4 | 0.6790 | 0.0018 | 18 | 1 | 0.8293 | 0.0831 | 19 | 3 | 0.8060 | 0.0020 |
| Gulf of Mexico | Japan | 37 | 44 | 0.6682 | 0.0010 | 18 | 25 | 0.7451 | 0.0010 | 19 | 19 | 0.8170 | 0.0010 |
| Gulf of Mexico | Mozambique | 37 | 11 | 0.6678 | 0.0010 | 18 | 5 | 0.7747 | 0.0010 | 19 | 6 | 0.7948 | 0.0010 |
| Gulf of Mexico | Papua New Guinea | 37 | 9 | 0.6735 | 0.0010 | 18 | 4 | 0.7929 | 0.0010 | 19 | 4 | 0.8220 | 0.0010 |
| Gulf of Mexico | Reunion | 37 | 28 | 0.6632 | 0.0010 | 18 | 13 | 0.7422 | 0.0010 | 19 | 15 | 0.7851 | 0.0010 |
| Gulf of Mexico | Seychelles | 37 | 36 | 0.6609 | 0.0010 | 18 | 28 | 0.7200 | 0.0010 | 19 | 8 | 0.7963 | 0.0010 |
| Gulf of Mexico | Sierra Leone | 37 | 1 | 0.6452 | 1.0000 | 18 | 1 | 0.8057 | 0.0661 | NA | NA | NA | NA |
| Gulf of Mexico | South Africa | 37 | 22 | 0.6645 | 0.0010 | 18 | 9 | 0.7542 | 0.0010 | 19 | 12 | 0.7692 | 0.0010 |
| Gulf of Mexico | Sri Lanka | 37 | 12 | 0.6690 | 0.0010 | 18 | 7 | 0.7560 | 0.0010 | 19 | 5 | 0.8164 | 0.0010 |
| Gulf of Mexico | Thailand | 37 | 5 | 0.6734 | 0.0013 | 18 | 3 | 0.7681 | 0.0020 | 19 | 2 | 0.8304 | 0.0074 |
| Gulf of Mexico | Western North Atlantic | 37 | 8 | -0.0002 | 0.4883 | 18 | 6 | -0.0149 | 0.7213 | 19 | 2 | 0.3533 | 0.0604 |
| Indonesia | Japan | 4 | 44 | NA | NA | 1 | 25 | NA | NA | 3 | 19 | 0.3125 | 0.0032 |
| Indonesia | Mozambique | 4 | 11 | 0.0115 | 0.1110 | 1 | 5 | -0.0165 | 0.6753 | 3 | 6 | -0.0997 | 0.7803 |
| Indonesia | Papua New Guinea | 4 | 9 | 0.0171 | 0.1032 | 1 | 4 | 0.1621 | 0.1958 | 3 | 4 | 0.0199 | 0.4675 |
| Indonesia | Reunion | 4 | 28 | 0.0125 | 0.0556 | 1 | 13 | 0.0392 | 0.3337 | 3 | 15 | 0.0022 | 0.4426 |
| Indonesia | Seychelles | 4 | 36 | 0.0147 | 0.0100 | 1 | 28 | NA | NA | 3 | 8 | 0.0430 | 0.2531 |
| Indonesia | Sierra Leone | 4 | 1 | 0.1862 | 1.0000 | 1 | 1 | NA | NA | NA | NA | NA | NA |
| Indonesia | South Africa | 4 | 22 | NA | NA | 1 | 9 | NA | NA | 3 | 12 | 0.0597 | 0.1858 |
| Indonesia | Sri Lanka | 4 | 12 | 0.0108 | 0.0829 | 1 | 7 | -0.0706 | 0.4126 | 3 | 5 | 0.1017 | 0.1598 |

Table 25: Pairwise Fst for autosomal and X-linked markers in females and males *(continued)*

| strata.1 | strata.2 | n.auDNA.1 | n.auDNA.2 | Fst.auDNA | Fst.auDNA.Pval | n.xDNA.F.1 | n.xDNA.F.2 | Fst.xDNA.F | Fst.xDNA.F.Pval | n.xDNA.M.1 | n.xDNA.M.2 | Fst.xDNA.M | Fst.xDNA.M.Pval |
|---|---|---|---|---|---|---|---|---|---|---|---|---|---|
| Indonesia | Thailand | 4 | 5 | 0.0190 | 0.1798 | 1 | 3 | -0.0220 | 0.7762 | 3 | 2 | -0.0297 | 0.6044 |
| Indonesia | Western North Atlantic | 4 | 8 | 0.6695 | 0.0057 | 1 | 6 | 0.8338 | 0.1359 | 3 | 2 | 0.6238 | 0.0989 |
| Japan | Mozambique | 44 | 11 | 0.0345 | 0.0010 | 25 | 5 | 0.0767 | 0.0180 | 19 | 6 | 0.1620 | 0.0110 |
| Japan | Papua New Guinea | 44 | 9 | 0.0350 | 0.0010 | 25 | 4 | 0.0382 | 0.0769 | 19 | 4 | 0.1177 | 0.0548 |
| Japan | Reunion | 44 | 28 | 0.0352 | 0.0010 | 25 | 13 | 0.0865 | 0.0010 | 19 | 15 | 0.2451 | 0.0010 |
| Japan | Seychelles | 44 | 36 | 0.0287 | 0.0010 | 25 | 28 | 0.0256 | 0.0110 | 19 | 8 | 0.1521 | 0.0010 |
| Japan | Sierra Leone | 44 | 1 | 0.2067 | 1.0000 | 25 | 1 | 0.1477 | 0.0397 | NA | NA | NA | NA |
| Japan | South Africa | 44 | 22 | 0.0318 | 0.0010 | 25 | 9 | 0.0298 | 0.0310 | 19 | 12 | 0.1652 | 0.0010 |
| Japan | Sri Lanka | 44 | 12 | 0.0318 | 0.0010 | 25 | 7 | 0.0201 | 0.1009 | 19 | 5 | 0.3094 | 0.0020 |
| Japan | Thailand | 44 | 5 | 0.0369 | 0.0040 | 25 | 3 | 0.0730 | 0.0595 | 19 | 2 | NA | NA |
| Japan | Western North Atlantic | 44 | 8 | 0.6581 | 0.0010 | 25 | 6 | 0.7255 | 0.0010 | 19 | 2 | 0.7641 | 0.0036 |
| Mozambique | Papua New Guinea | 11 | 9 | 0.0111 | 0.0030 | 5 | 4 | 0.1287 | 0.0220 | 6 | 4 | -0.0214 | 0.5155 |
| Mozambique | Reunion | 11 | 28 | 0.0054 | 0.0280 | 5 | 13 | 0.0043 | 0.3566 | 6 | 15 | 0.0451 | 0.1484 |
| Mozambique | Seychelles | 11 | 36 | 0.0051 | 0.0040 | 5 | 28 | 0.0232 | 0.1053 | 6 | 8 | -0.0520 | 0.7932 |
| Mozambique | Sierra Leone | 11 | 1 | 0.1391 | 1.0000 | 5 | 1 | 0.3089 | 0.2481 | NA | NA | NA | NA |
| Mozambique | South Africa | 11 | 22 | 0.0012 | 0.2757 | 5 | 9 | 0.0099 | 0.3037 | 6 | 12 | 0.0265 | 0.2490 |
| Mozambique | Sri Lanka | 11 | 12 | 0.0033 | 0.0889 | 5 | 7 | 0.0267 | 0.1768 | 6 | 5 | 0.0429 | 0.2588 |
| Mozambique | Thailand | 11 | 5 | -0.0027 | 0.7239 | 5 | 3 | 0.0375 | 0.1508 | 6 | 2 | NA | NA |
| Mozambique | Western North Atlantic | 11 | 8 | 0.6505 | 0.0010 | 5 | 6 | 0.7504 | 0.0030 | 6 | 2 | 0.6036 | 0.0427 |
| Papua New Guinea | Reunion | 9 | 28 | 0.0058 | 0.0260 | 4 | 13 | 0.0691 | 0.0100 | 4 | 15 | 0.0128 | 0.3357 |
| Papua New Guinea | Seychelles | 9 | 36 | 0.0042 | 0.0340 | 4 | 28 | 0.0434 | 0.0779 | 4 | 8 | 0.0030 | 0.3912 |
| Papua New Guinea | Sierra Leone | 9 | 1 | 0.1688 | 1.0000 | 4 | 1 | 0.2146 | 0.2008 | NA | NA | NA | NA |
| Papua New Guinea | South Africa | 9 | 22 | 0.0020 | 0.2168 | 4 | 9 | 0.0154 | 0.2567 | 4 | 12 | 0.0024 | 0.4486 |
| Papua New Guinea | Sri Lanka | 9 | 12 | 0.0042 | 0.0849 | 4 | 7 | -0.0095 | 0.5654 | 4 | 5 | 0.1800 | 0.0350 |
| Papua New Guinea | Thailand | 9 | 5 | 0.0044 | 0.1998 | 4 | 3 | 0.0495 | 0.2018 | 4 | 2 | 0.0656 | 0.1948 |
| Papua New Guinea | Western North Atlantic | 9 | 8 | 0.6609 | 0.0010 | 4 | 6 | 0.7783 | 0.0060 | 4 | 2 | 0.6814 | 0.0659 |
| Reunion | Seychelles | 28 | 36 | 0.0048 | 0.0010 | 13 | 28 | 0.0176 | 0.0519 | 15 | 8 | 0.0763 | 0.0170 |
| Reunion | Sierra Leone | 28 | 1 | 0.1589 | 1.0000 | 13 | 1 | 0.2673 | 0.1113 | NA | NA | NA | NA |
| Reunion | South Africa | 28 | 22 | 0.0033 | 0.0230 | 13 | 9 | -0.0174 | 0.9251 | 15 | 12 | 0.0469 | 0.0440 |
| Reunion | Sri Lanka | 28 | 12 | 0.0032 | 0.0809 | 13 | 7 | 0.0037 | 0.3666 | 15 | 5 | 0.1078 | 0.0190 |
| Reunion | Thailand | 28 | 5 | 0.0030 | 0.2380 | 13 | 3 | 0.0052 | 0.3826 | 15 | 2 | -0.1061 | 0.8256 |
| Reunion | Western North Atlantic | 28 | 8 | 0.6485 | 0.0010 | 13 | 6 | 0.7150 | 0.0010 | 15 | 2 | 0.6998 | 0.0074 |
| Seychelles | Sierra Leone | 36 | 1 | 0.1641 | 1.0000 | 28 | 1 | 0.1876 | 0.0771 | NA | NA | NA | NA |
| Seychelles | South Africa | 36 | 22 | 0.0005 | 0.3297 | 28 | 9 | -0.0155 | 0.8911 | 8 | 12 | 0.0508 | 0.0589 |
| Seychelles | Sri Lanka | 36 | 12 | 0.0002 | 0.4555 | 28 | 7 | 0.0014 | 0.3610 | 8 | 5 | 0.0433 | 0.2150 |
| Seychelles | Thailand | 36 | 5 | 0.0046 | 0.1049 | 28 | 3 | 0.0140 | 0.2630 | 8 | 2 | NA | NA |
| Seychelles | Western North Atlantic | 36 | 8 | 0.6475 | 0.0010 | 28 | 6 | 0.6982 | 0.0010 | 8 | 2 | 0.6547 | 0.0334 |
| Sierra Leone | South Africa | 1 | 22 | 0.1491 | 1.0000 | 1 | 9 | 0.1700 | 0.1172 | NA | NA | NA | NA |
| Sierra Leone | Sri Lanka | 1 | 12 | 0.1553 | 1.0000 | 1 | 7 | 0.1404 | 0.3857 | NA | NA | NA | NA |
| Sierra Leone | Thailand | 1 | 5 | 0.1549 | 1.0000 | 1 | 3 | 0.1502 | 0.3541 | NA | NA | NA | NA |
| Sierra Leone | Western North Atlantic | 1 | 8 | 0.6399 | 1.0000 | 1 | 6 | 0.8145 | 0.1359 | NA | NA | NA | NA |
| South Africa | Sri Lanka | 22 | 12 | 0.0005 | 0.3646 | 9 | 7 | -0.0370 | 0.9770 | 12 | 5 | 0.0427 | 0.1808 |
| South Africa | Thailand | 22 | 5 | -0.0017 | 0.6863 | 9 | 3 | -0.0198 | 0.6372 | 12 | 2 | -0.0818 | 0.8021 |
| South Africa | Western North Atlantic | 22 | 8 | 0.6488 | 0.0010 | 9 | 6 | 0.7243 | 0.0020 | 12 | 2 | 0.6531 | 0.0196 |
| Sri Lanka | Thailand | 12 | 5 | -0.0005 | 0.5654 | 7 | 3 | -0.0284 | 0.7233 | 5 | 2 | -0.0471 | 0.6104 |
| Sri Lanka | Western North Atlantic | 12 | 8 | 0.6531 | 0.0010 | 7 | 6 | 0.7239 | 0.0020 | 5 | 2 | 0.6795 | 0.0525 |
| Thailand | Western North Atlantic | 5 | 8 | 0.6580 | 0.0030 | 3 | 6 | 0.7358 | 0.0080 | 2 | 2 | 0.6492 | 0.3257 |

```r
pwFis <- data[,c(1:4,11:12, 24:25,32:33, 44:45,52:53)]
colnames(pwFis) <- c("strata.1", "strata.2", "n.auDNA.1", "n.auDNA.2", "Fis.auDNA",
                     "Fis.auDNA.Pval",
                     "n.xDNA.F.1", "n.xDNA.F.2", "Fis.xDNA.F", "Fis.xDNA.F.Pval",
                     "n.xDNA.M.1", "n.xDNA.M.2", "Fis.xDNA.M", "Fis.xDNA.M.Pval")

kableExtra::kbl(pwFis, digits = 4,
  caption = "Pairwise Fis for autosomal and X-linked markers in females and males",
  longtable = TRUE, booktabs = TRUE) %>%
  kableExtra::kable_styling(full_width = FALSE,font_size = 5,
                            latex_options = c("repeat_header", "hold_position")) %>%
  kableExtra::landscape()
```

#### 8.11.1.3 nuFis - xFis

Table 26: Pairwise Fis for autosomal and X-linked markers in females and males

| strata.1 | strata.2 | n.auDNA.1 | n.auDNA.2 | Fis.auDNA | Fis.auDNA.Pval | n.xDNA.F.1 | n.xDNA.F.2 | Fis.xDNA.F | Fis.xDNA.F.Pval | n.xDNA.M.1 | n.xDNA.M.2 | Fis.xDNA.M | Fis.xDNA.M.Pval |
|---|---|---|---|---|---|---|---|---|---|---|---|---|---|
| Arabian Sea | Australia | 15 | 444 | 0.0018 | 0.0220 | 9 | 193 | -0.0076 | 0.7230 | 6 | 225 | 0.0028 | 0.4074 |
| Arabian Sea | Brazil | 15 | 50 | 0.6538 | 0.0010 | NA | NA | NA | NA | NA | NA | NA | NA |
| Arabian Sea | Caribbean Sea | 15 | 2 | 0.6376 | 0.0166 | NA | NA | NA | NA | 6 | 2 | 0.6899 | 0.0350 |
| Arabian Sea | Costa Rica | 15 | 16 | 0.6703 | 0.0010 | 9 | 9 | 0.7611 | 0.0010 | 6 | 7 | 0.7948 | 0.0020 |
| Arabian Sea | Fiji | 15 | 25 | 0.0273 | 0.0010 | 9 | 22 | 0.0588 | 0.0010 | 6 | 3 | NA | NA |
| Arabian Sea | Gulf of Mexico | 15 | 37 | 0.6674 | 0.0010 | 9 | 18 | 0.7427 | 0.0010 | 6 | 19 | 0.8008 | 0.0010 |
| Arabian Sea | Indonesia | 15 | 4 | 0.0124 | 0.0241 | 9 | 1 | -0.0347 | 0.6893 | 6 | 3 | 0.0257 | 0.3027 |
| Arabian Sea | Japan | 15 | 44 | 0.0306 | 0.0010 | 9 | 25 | 0.0545 | 0.0120 | 6 | 19 | 0.1678 | 0.0010 |
| Arabian Sea | Mozambique | 15 | 11 | 0.0041 | 0.0380 | 9 | 5 | 0.0132 | 0.2368 | 6 | 6 | -0.0377 | 0.7423 |
| Arabian Sea | Papua New Guinea | 15 | 9 | 0.0067 | 0.0060 | 9 | 4 | 0.0284 | 0.1588 | 6 | 4 | -0.0878 | 0.9590 |
| Arabian Sea | Reunion | 15 | 28 | 0.0019 | 0.1419 | 9 | 13 | -0.0053 | 0.6134 | 6 | 15 | 0.0362 | 0.1653 |
| Arabian Sea | Seychelles | 15 | 36 | 0.0011 | 0.1848 | 9 | 28 | 0.0026 | 0.3776 | 6 | 8 | -0.0040 | 0.4775 |
| Arabian Sea | Sierra Leone | 15 | 1 | 0.1582 | 1.0000 | 9 | 1 | 0.1749 | 0.2306 | NA | NA | NA | NA |
| Arabian Sea | South Africa | 15 | 22 | 0.0020 | 0.0909 | 9 | 9 | -0.0312 | 0.9760 | 6 | 12 | -0.0313 | 0.7960 |
| Arabian Sea | Sri Lanka | 15 | 12 | -0.0014 | 0.7622 | 9 | 7 | -0.0359 | 0.9610 | 6 | 5 | 0.0532 | 0.1291 |
| Arabian Sea | Thailand | 15 | 5 | -0.0019 | 0.7233 | 9 | 3 | -0.0366 | 0.8412 | 6 | 2 | NA | NA |
| Arabian Sea | Western North Atlantic | 15 | 8 | 0.6495 | 0.0010 | 9 | 6 | 0.7067 | 0.0010 | 6 | 2 | 0.6518 | 0.0390 |
| Australia | Brazil | 444 | 50 | 0.6359 | 0.0010 | NA | NA | NA | NA | NA | NA | NA | NA |
| Australia | Caribbean Sea | 444 | 2 | 0.6353 | 0.0040 | NA | NA | NA | NA | 225 | 2 | 0.6879 | 0.0021 |
| Australia | Costa Rica | 444 | 16 | 0.6264 | 0.0010 | 193 | 9 | 0.6928 | 0.0010 | 225 | 7 | 0.7135 | 0.0010 |
| Australia | Fiji | 444 | 25 | 0.0310 | 0.0010 | 193 | 22 | 0.0599 | 0.0010 | 225 | 3 | NA | NA |
| Australia | Gulf of Mexico | 444 | 37 | 0.6416 | 0.0010 | 193 | 18 | 0.6812 | 0.0010 | 225 | 19 | 0.7075 | 0.0010 |
| Australia | Indonesia | 444 | 4 | NA | NA | 193 | 1 | NA | NA | 225 | 3 | 0.0539 | 0.1994 |
| Australia | Japan | 444 | 44 | 0.0287 | 0.0010 | 193 | 25 | 0.0304 | 0.0010 | 225 | 19 | 0.1152 | 0.0010 |
| Australia | Mozambique | 444 | 11 | 0.0044 | 0.0010 | 193 | 5 | 0.0286 | 0.0682 | 225 | 6 | 0.0261 | 0.1758 |
| Australia | Papua New Guinea | 444 | 9 | 0.0018 | 0.1179 | 193 | 4 | 0.0298 | 0.1280 | 225 | 4 | -0.0225 | 0.6108 |
| Australia | Reunion | 444 | 28 | 0.0052 | 0.0010 | 193 | 13 | 0.0137 | 0.0440 | 225 | 15 | 0.0293 | 0.0280 |
| Australia | Seychelles | 444 | 36 | 0.0015 | 0.0010 | 193 | 28 | 0.0012 | 0.3197 | 225 | 8 | 0.0341 | 0.0785 |
| Australia | Sierra Leone | 444 | 1 | 0.1572 | 1.0000 | 193 | 1 | 0.1869 | 0.0107 | NA | NA | NA | NA |
| Australia | South Africa | 444 | 22 | 0.0008 | 0.1209 | 193 | 9 | -0.0152 | 0.9510 | 225 | 12 | 0.0176 | 0.1251 |
| Australia | Sri Lanka | 444 | 12 | 0.0005 | 0.3127 | 193 | 7 | -0.0118 | 0.7946 | 225 | 5 | 0.0363 | 0.1721 |
| Australia | Thailand | 444 | 5 | 0.0014 | 0.3150 | 193 | 3 | -0.0028 | 0.4341 | 225 | 2 | NA | NA |
| Australia | Western North Atlantic | 444 | 8 | 0.6385 | 0.0010 | 193 | 6 | 0.6809 | 0.0010 | 225 | 2 | 0.6803 | 0.0021 |
| Brazil | Caribbean Sea | 50 | 2 | 0.0016 | 0.6250 | NA | NA | NA | NA | NA | NA | NA | NA |
| Brazil | Costa Rica | 50 | 16 | 0.3484 | 0.0010 | NA | NA | NA | NA | NA | NA | NA | NA |
| Brazil | Fiji | 50 | 25 | 0.6521 | 0.0010 | NA | NA | NA | NA | NA | NA | NA | NA |
| Brazil | Gulf of Mexico | 50 | 37 | 0.0049 | 0.0010 | NA | NA | NA | NA | NA | NA | NA | NA |
| Brazil | Indonesia | 50 | 4 | 0.6610 | 0.0016 | NA | NA | NA | NA | NA | NA | NA | NA |
| Brazil | Japan | 50 | 44 | 0.6580 | 0.0010 | NA | NA | NA | NA | NA | NA | NA | NA |
| Brazil | Mozambique | 50 | 11 | 0.6532 | 0.0010 | NA | NA | NA | NA | NA | NA | NA | NA |
| Brazil | Papua New Guinea | 50 | 9 | 0.6576 | 0.0010 | NA | NA | NA | NA | NA | NA | NA | NA |
| Brazil | Reunion | 50 | 28 | 0.6518 | 0.0010 | NA | NA | NA | NA | NA | NA | NA | NA |
| Brazil | Seychelles | 50 | 36 | 0.6504 | 0.0010 | NA | NA | NA | NA | NA | NA | NA | NA |
| Brazil | Sierra Leone | 50 | 1 | 0.6231 | 1.0000 | NA | NA | NA | NA | NA | NA | NA | NA |
| Brazil | South Africa | 50 | 22 | 0.6522 | 0.0010 | NA | NA | NA | NA | NA | NA | NA | NA |
| Brazil | Sri Lanka | 50 | 12 | 0.6543 | 0.0010 | NA | NA | NA | NA | NA | NA | NA | NA |
| Brazil | Thailand | 50 | 5 | 0.6564 | 0.0012 | NA | NA | NA | NA | NA | NA | NA | NA |
| Brazil | Western North Atlantic | 50 | 8 | 0.0037 | 0.0884 | NA | NA | NA | NA | NA | NA | NA | NA |
| Caribbean Sea | Costa Rica | 2 | 16 | 0.4287 | 0.0845 | NA | NA | NA | NA | 2 | 7 | 0.6704 | 0.0380 |
| Caribbean Sea | Fiji | 2 | 25 | 0.6389 | 0.0341 | NA | NA | NA | NA | 2 | 3 | 0.7248 | 0.0939 |
| Caribbean Sea | Gulf of Mexico | 2 | 37 | -0.0020 | 0.5714 | NA | NA | NA | NA | 2 | 19 | -0.1106 | 0.7202 |
| Caribbean Sea | Indonesia | 2 | 4 | 0.6592 | 0.0772 | NA | NA | NA | NA | 2 | 3 | 0.7086 | 0.1089 |
| Caribbean Sea | Japan | 2 | 44 | 0.6537 | 0.0110 | NA | NA | NA | NA | 2 | 19 | 0.7804 | 0.0100 |

Table 26: Pairwise Fis for autosomal and X-linked markers in females and males *(continued)*

| strata.1 | strata.2 | n.auDNA.1 | n.auDNA.2 | Fis.auDNA | Fis.auDNA.Pval | n.xDNA.F.1 | n.xDNA.F.2 | Fis.xDNA.F | Fis.xDNA.F.Pval | n.xDNA.M.1 | n.xDNA.M.2 | Fis.xDNA.M | Fis.xDNA.M.Pval |
|---|---|---|---|---|---|---|---|---|---|---|---|---|---|
| Caribbean Sea | Mozambique | 2 | 11 | 0.6367 | 0.0445 | NA | NA | NA | NA | 2 | 6 | 0.6568 | 0.0815 |
| Caribbean Sea | Papua New Guinea | 2 | 9 | 0.6504 | 0.0216 | NA | NA | NA | NA | 2 | 4 | 0.7336 | 0.0679 |
| Caribbean Sea | Reunion | 2 | 28 | 0.6401 | 0.0079 | NA | NA | NA | NA | 2 | 15 | 0.7245 | 0.0061 |
| Caribbean Sea | Seychelles | 2 | 36 | 0.6409 | 0.0046 | NA | NA | NA | NA | 2 | 8 | 0.6816 | 0.0161 |
| Caribbean Sea | Sierra Leone | 2 | 1 | 0.6360 | 0.3417 | NA | NA | NA | NA | NA | NA | NA | NA |
| Caribbean Sea | South Africa | 2 | 22 | 0.6396 | 0.0086 | NA | NA | NA | NA | 2 | 12 | 0.6793 | 0.0061 |
| Caribbean Sea | Sri Lanka | 2 | 12 | 0.6407 | 0.0192 | NA | NA | NA | NA | 2 | 5 | 0.7267 | 0.0529 |
| Caribbean Sea | Thailand | 2 | 5 | 0.6397 | 0.0605 | NA | NA | NA | NA | 2 | 2 | 0.7647 | 0.3187 |
| Caribbean Sea | Western North Atlantic | 2 | 8 | -0.0077 | 1.0000 | NA | NA | NA | NA | 2 | 2 | -0.1220 | 1.0000 |
| Costa Rica | Fiji | 16 | 25 | 0.6594 | 0.0010 | 9 | 22 | 0.7623 | 0.0010 | 7 | 3 | 0.8562 | 0.0063 |
| Costa Rica | Gulf of Mexico | 16 | 37 | 0.3692 | 0.0010 | 9 | 18 | 0.5201 | 0.0010 | 7 | 19 | 0.6018 | 0.0010 |
| Costa Rica | Indonesia | 16 | 4 | 0.7066 | 0.0016 | 9 | 1 | 0.9021 | 0.1250 | 7 | 3 | 0.8386 | 0.0091 |
| Costa Rica | Japan | 16 | 44 | 0.6609 | 0.0010 | 9 | 25 | 0.7547 | 0.0010 | 7 | 19 | 0.8228 | 0.0010 |
| Costa Rica | Mozambique | 16 | 11 | 0.6766 | 0.0010 | 9 | 5 | 0.8143 | 0.0010 | 7 | 6 | 0.7916 | 0.0021 |
| Costa Rica | Papua New Guinea | 16 | 9 | 0.6888 | 0.0010 | 9 | 4 | 0.8405 | 0.0020 | 7 | 4 | 0.8378 | 0.0040 |
| Costa Rica | Reunion | 16 | 28 | 0.6579 | 0.0010 | 9 | 13 | NA | NA | 7 | 15 | 0.7800 | 0.0010 |
| Costa Rica | Seychelles | 16 | 36 | 0.6532 | 0.0010 | 9 | 28 | 0.7273 | 0.0010 | 7 | 8 | 0.7817 | 0.0010 |
| Costa Rica | Sierra Leone | 16 | 1 | 0.7013 | 1.0000 | 9 | 1 | 0.8934 | 0.1354 | NA | NA | NA | NA |
| Costa Rica | South Africa | 16 | 22 | 0.6623 | 0.0010 | 9 | 9 | 0.7768 | 0.0010 | 7 | 12 | 0.7505 | 0.0010 |
| Costa Rica | Sri Lanka | 16 | 12 | 0.6765 | 0.0010 | 9 | 7 | 0.7817 | 0.0010 | 7 | 5 | 0.8244 | 0.0010 |
| Costa Rica | Thailand | 16 | 5 | 0.6957 | 0.0012 | 9 | 3 | 0.8105 | 0.0060 | 7 | 2 | 0.8787 | 0.0326 |
| Costa Rica | Western North Atlantic | 16 | 8 | 0.3964 | 0.0011 | 9 | 6 | 0.5826 | 0.0020 | 7 | 2 | 0.6580 | 0.0320 |
| Fiji | Gulf of Mexico | 25 | 37 | 0.6636 | 0.0010 | 22 | 18 | 0.7498 | 0.0010 | 3 | 19 | 0.8276 | 0.0020 |
| Fiji | Indonesia | 25 | 4 | NA | NA | 22 | 1 | 0.0951 | 0.1054 | 3 | 3 | 0.0982 | 0.3397 |
| Fiji | Japan | 25 | 44 | 0.0617 | 0.0010 | 22 | 25 | 0.1148 | 0.0010 | 3 | 19 | NA | NA |
| Fiji | Mozambique | 25 | 11 | 0.0333 | 0.0010 | 22 | 5 | 0.1609 | 0.0010 | 3 | 6 | NA | NA |
| Fiji | Papua New Guinea | 25 | 9 | 0.0313 | 0.0010 | 22 | 4 | 0.0848 | 0.0051 | 3 | 4 | 0.0120 | 0.4436 |
| Fiji | Reunion | 25 | 28 | 0.0331 | 0.0010 | 22 | 13 | 0.0950 | 0.0010 | 3 | 15 | 0.0166 | 0.3347 |
| Fiji | Seychelles | 25 | 36 | 0.0318 | 0.0010 | 22 | 28 | 0.0723 | 0.0010 | 3 | 8 | NA | NA |
| Fiji | Sierra Leone | 25 | 1 | 0.1610 | 1.0000 | 22 | 1 | 0.3115 | 0.2784 | NA | NA | NA | NA |
| Fiji | South Africa | 25 | 22 | 0.0304 | 0.0010 | 22 | 9 | 0.0548 | 0.0040 | 3 | 12 | 0.0589 | 0.1608 |
| Fiji | Sri Lanka | 25 | 12 | 0.0318 | 0.0010 | 22 | 7 | 0.0654 | 0.0020 | 3 | 5 | 0.1228 | 0.0480 |
| Fiji | Thailand | 25 | 5 | 0.0255 | 0.0072 | 22 | 3 | 0.0657 | 0.0342 | 3 | 2 | -0.2218 | 0.9171 |
| Fiji | Western North Atlantic | 25 | 8 | 0.6477 | 0.0010 | 22 | 6 | 0.7318 | 0.0010 | 3 | 2 | 0.6637 | 0.1044 |
| Gulf of Mexico | Indonesia | 37 | 4 | 0.6790 | 0.0018 | 18 | 1 | 0.8293 | 0.0831 | 19 | 3 | 0.8060 | 0.0020 |
| Gulf of Mexico | Japan | 37 | 44 | 0.6682 | 0.0010 | 18 | 25 | 0.7451 | 0.0010 | 19 | 19 | 0.8170 | 0.0010 |
| Gulf of Mexico | Mozambique | 37 | 11 | 0.6678 | 0.0010 | 18 | 5 | 0.7747 | 0.0010 | 19 | 6 | 0.7948 | 0.0010 |
| Gulf of Mexico | Papua New Guinea | 37 | 9 | 0.6735 | 0.0010 | 18 | 4 | 0.7929 | 0.0010 | 19 | 4 | 0.8220 | 0.0010 |
| Gulf of Mexico | Reunion | 37 | 28 | 0.6632 | 0.0010 | 18 | 13 | 0.7422 | 0.0010 | 19 | 15 | 0.7851 | 0.0010 |
| Gulf of Mexico | Seychelles | 37 | 36 | 0.6609 | 0.0010 | 18 | 28 | 0.7200 | 0.0010 | 19 | 8 | 0.7963 | 0.0010 |
| Gulf of Mexico | Sierra Leone | 37 | 1 | 0.6452 | 1.0000 | 18 | 1 | 0.8057 | 0.0661 | NA | NA | NA | NA |
| Gulf of Mexico | South Africa | 37 | 22 | 0.6645 | 0.0010 | 18 | 9 | 0.7542 | 0.0010 | 19 | 12 | 0.7692 | 0.0010 |
| Gulf of Mexico | Sri Lanka | 37 | 12 | 0.6690 | 0.0010 | 18 | 7 | 0.7560 | 0.0010 | 19 | 5 | 0.8164 | 0.0010 |
| Gulf of Mexico | Thailand | 37 | 5 | 0.6734 | 0.0013 | 18 | 3 | 0.7681 | 0.0020 | 19 | 2 | 0.8304 | 0.0074 |
| Gulf of Mexico | Western North Atlantic | 37 | 8 | -0.0002 | 0.4883 | 18 | 6 | -0.0149 | 0.7213 | 19 | 2 | 0.3533 | 0.0604 |
| Indonesia | Japan | 4 | 44 | NA | NA | 1 | 25 | NA | NA | 3 | 19 | 0.3125 | 0.0032 |
| Indonesia | Mozambique | 4 | 11 | 0.0115 | 0.1110 | 1 | 5 | -0.0165 | 0.6753 | 3 | 6 | -0.0997 | 0.7803 |
| Indonesia | Papua New Guinea | 4 | 9 | 0.0171 | 0.1032 | 1 | 4 | 0.1621 | 0.1958 | 3 | 4 | 0.0199 | 0.4675 |
| Indonesia | Reunion | 4 | 28 | 0.0125 | 0.0556 | 1 | 13 | 0.0392 | 0.3337 | 3 | 15 | 0.0022 | 0.4426 |
| Indonesia | Seychelles | 4 | 36 | 0.0147 | 0.0100 | 1 | 28 | NA | NA | 3 | 8 | 0.0430 | 0.2531 |
| Indonesia | Sierra Leone | 4 | 1 | 0.1862 | 1.0000 | 1 | 1 | NA | NA | NA | NA | NA | NA |
| Indonesia | South Africa | 4 | 22 | NA | NA | 1 | 9 | NA | NA | 3 | 12 | 0.0597 | 0.1858 |
| Indonesia | Sri Lanka | 4 | 12 | 0.0108 | 0.0829 | 1 | 7 | -0.0706 | 0.4126 | 3 | 5 | 0.1017 | 0.1598 |

Table 26: Pairwise Fis for autosomal and X-linked markers in females and males *(continued)*

| strata.1 | strata.2 | n.auDNA.1 | n.auDNA.2 | Fis.auDNA | Fis.auDNA.Pval | n.xDNA.F.1 | n.xDNA.F.2 | Fis.xDNA.F | Fis.xDNA.F.Pval | n.xDNA.M.1 | n.xDNA.M.2 | Fis.xDNA.M | Fis.xDNA.M.Pval |
|---|---|---|---|---|---|---|---|---|---|---|---|---|---|
| Indonesia | Thailand | 4 | 5 | 0.0190 | 0.1798 | 1 | 3 | -0.0220 | 0.7762 | 3 | 2 | -0.0297 | 0.6044 |
| Indonesia | Western North Atlantic | 4 | 8 | 0.6695 | 0.0057 | 1 | 6 | 0.8338 | 0.1359 | 3 | 2 | 0.6238 | 0.0989 |
| Japan | Mozambique | 44 | 11 | 0.0345 | 0.0010 | 25 | 5 | 0.0767 | 0.0180 | 19 | 6 | 0.1620 | 0.0110 |
| Japan | Papua New Guinea | 44 | 9 | 0.0350 | 0.0010 | 25 | 4 | 0.0382 | 0.0769 | 19 | 4 | 0.1177 | 0.0548 |
| Japan | Reunion | 44 | 28 | 0.0352 | 0.0010 | 25 | 13 | 0.0865 | 0.0010 | 19 | 15 | 0.2451 | 0.0010 |
| Japan | Seychelles | 44 | 36 | 0.0287 | 0.0010 | 25 | 28 | 0.0256 | 0.0110 | 19 | 8 | 0.1521 | 0.0010 |
| Japan | Sierra Leone | 44 | 1 | 0.2067 | 1.0000 | 25 | 1 | 0.1477 | 0.0397 | NA | NA | NA | NA |
| Japan | South Africa | 44 | 22 | 0.0318 | 0.0010 | 25 | 9 | 0.0298 | 0.0310 | 19 | 12 | 0.1652 | 0.0010 |
| Japan | Sri Lanka | 44 | 12 | 0.0318 | 0.0010 | 25 | 7 | 0.0201 | 0.1009 | 19 | 5 | 0.3094 | 0.0020 |
| Japan | Thailand | 44 | 5 | 0.0369 | 0.0040 | 25 | 3 | 0.0730 | 0.0595 | 19 | 2 | NA | NA |
| Japan | Western North Atlantic | 44 | 8 | 0.6581 | 0.0010 | 25 | 6 | 0.7255 | 0.0010 | 19 | 2 | 0.7641 | 0.0036 |
| Mozambique | Papua New Guinea | 11 | 9 | 0.0111 | 0.0030 | 5 | 4 | 0.1287 | 0.0220 | 6 | 4 | -0.0214 | 0.5155 |
| Mozambique | Reunion | 11 | 28 | 0.0054 | 0.0280 | 5 | 13 | 0.0043 | 0.3566 | 6 | 15 | 0.0451 | 0.1484 |
| Mozambique | Seychelles | 11 | 36 | 0.0051 | 0.0040 | 5 | 28 | 0.0232 | 0.1053 | 6 | 8 | -0.0520 | 0.7932 |
| Mozambique | Sierra Leone | 11 | 1 | 0.1391 | 1.0000 | 5 | 1 | 0.3089 | 0.2481 | NA | NA | NA | NA |
| Mozambique | South Africa | 11 | 22 | 0.0012 | 0.2757 | 5 | 9 | 0.0099 | 0.3037 | 6 | 12 | 0.0265 | 0.2490 |
| Mozambique | Sri Lanka | 11 | 12 | 0.0033 | 0.0889 | 5 | 7 | 0.0267 | 0.1768 | 6 | 5 | 0.0429 | 0.2588 |
| Mozambique | Thailand | 11 | 5 | -0.0027 | 0.7239 | 5 | 3 | 0.0375 | 0.1508 | 6 | 2 | NA | NA |
| Mozambique | Western North Atlantic | 11 | 8 | 0.6505 | 0.0010 | 5 | 6 | 0.7504 | 0.0030 | 6 | 2 | 0.6036 | 0.0427 |
| Papua New Guinea | Reunion | 9 | 28 | 0.0058 | 0.0260 | 4 | 13 | 0.0691 | 0.0100 | 4 | 15 | 0.0128 | 0.3357 |
| Papua New Guinea | Seychelles | 9 | 36 | 0.0042 | 0.0340 | 4 | 28 | 0.0434 | 0.0779 | 4 | 8 | 0.0030 | 0.3912 |
| Papua New Guinea | Sierra Leone | 9 | 1 | 0.1688 | 1.0000 | 4 | 1 | 0.2146 | 0.2008 | NA | NA | NA | NA |
| Papua New Guinea | South Africa | 9 | 22 | 0.0020 | 0.2168 | 4 | 9 | 0.0154 | 0.2567 | 4 | 12 | 0.0024 | 0.4486 |
| Papua New Guinea | Sri Lanka | 9 | 12 | 0.0042 | 0.0849 | 4 | 7 | -0.0095 | 0.5654 | 4 | 5 | 0.1800 | 0.0350 |
| Papua New Guinea | Thailand | 9 | 5 | 0.0044 | 0.1998 | 4 | 3 | 0.0495 | 0.2018 | 4 | 2 | 0.0656 | 0.1948 |
| Papua New Guinea | Western North Atlantic | 9 | 8 | 0.6609 | 0.0010 | 4 | 6 | 0.7783 | 0.0060 | 4 | 2 | 0.6814 | 0.0659 |
| Reunion | Seychelles | 28 | 36 | 0.0048 | 0.0010 | 13 | 28 | 0.0176 | 0.0519 | 15 | 8 | 0.0763 | 0.0170 |
| Reunion | Sierra Leone | 28 | 1 | 0.1589 | 1.0000 | 13 | 1 | 0.2673 | 0.1113 | NA | NA | NA | NA |
| Reunion | South Africa | 28 | 22 | 0.0033 | 0.0230 | 13 | 9 | -0.0174 | 0.9251 | 15 | 12 | 0.0469 | 0.0440 |
| Reunion | Sri Lanka | 28 | 12 | 0.0032 | 0.0809 | 13 | 7 | 0.0037 | 0.3666 | 15 | 5 | 0.1078 | 0.0190 |
| Reunion | Thailand | 28 | 5 | 0.0030 | 0.2380 | 13 | 3 | 0.0052 | 0.3826 | 15 | 2 | -0.1061 | 0.8256 |
| Reunion | Western North Atlantic | 28 | 8 | 0.6485 | 0.0010 | 13 | 6 | 0.7150 | 0.0010 | 15 | 2 | 0.6998 | 0.0074 |
| Seychelles | Sierra Leone | 36 | 1 | 0.1641 | 1.0000 | 28 | 1 | 0.1876 | 0.0771 | NA | NA | NA | NA |
| Seychelles | South Africa | 36 | 22 | 0.0005 | 0.3297 | 28 | 9 | -0.0155 | 0.8911 | 8 | 12 | 0.0508 | 0.0589 |
| Seychelles | Sri Lanka | 36 | 12 | 0.0002 | 0.4555 | 28 | 7 | 0.0014 | 0.3610 | 8 | 5 | 0.0433 | 0.2150 |
| Seychelles | Thailand | 36 | 5 | 0.0046 | 0.1049 | 28 | 3 | 0.0140 | 0.2630 | 8 | 2 | NA | NA |
| Seychelles | Western North Atlantic | 36 | 8 | 0.6475 | 0.0010 | 28 | 6 | 0.6982 | 0.0010 | 8 | 2 | 0.6547 | 0.0334 |
| Sierra Leone | South Africa | 1 | 22 | 0.1491 | 1.0000 | 1 | 9 | 0.1700 | 0.1172 | NA | NA | NA | NA |
| Sierra Leone | Sri Lanka | 1 | 12 | 0.1553 | 1.0000 | 1 | 7 | 0.1404 | 0.3857 | NA | NA | NA | NA |
| Sierra Leone | Thailand | 1 | 5 | 0.1549 | 1.0000 | 1 | 3 | 0.1502 | 0.3541 | NA | NA | NA | NA |
| Sierra Leone | Western North Atlantic | 1 | 8 | 0.6399 | 1.0000 | 1 | 6 | 0.8145 | 0.1359 | NA | NA | NA | NA |
| South Africa | Sri Lanka | 22 | 12 | 0.0005 | 0.3646 | 9 | 7 | -0.0370 | 0.9770 | 12 | 5 | 0.0427 | 0.1808 |
| South Africa | Thailand | 22 | 5 | -0.0017 | 0.6863 | 9 | 3 | -0.0198 | 0.6372 | 12 | 2 | -0.0818 | 0.8021 |
| South Africa | Western North Atlantic | 22 | 8 | 0.6488 | 0.0010 | 9 | 6 | 0.7243 | 0.0020 | 12 | 2 | 0.6531 | 0.0196 |
| Sri Lanka | Thailand | 12 | 5 | -0.0005 | 0.5654 | 7 | 3 | -0.0284 | 0.7233 | 5 | 2 | -0.0471 | 0.6104 |
| Sri Lanka | Western North Atlantic | 12 | 8 | 0.6531 | 0.0010 | 7 | 6 | 0.7239 | 0.0020 | 5 | 2 | 0.6795 | 0.0525 |
| Thailand | Western North Atlantic | 5 | 8 | 0.6580 | 0.0030 | 3 | 6 | 0.7358 | 0.0080 | 2 | 2 | 0.6492 | 0.3257 |

### 8.11.2 Individual-based tests by Site

```
pop.levels <- c("Costa Rica" ,"Brazil", "Caribbean Sea",  "Gulf of Mexico",
                "Western North Atlantic",  "Sierra Leone", "South Africa",
                "Mozambique", "Reunion" , "Seychelles", "Arabian Sea", "Sri Lanka",
                "Thailand", "Indonesia", "Papua New Guinea" , "Australia",
                "Japan", "Fiji")

gl <- BS.dartcap.gl
gl$pop <- factor(gl$other$ind.metrics$Site, levels = pop.levels)
gl <- gl[order(gl$pop, gl$ind.names)]

gl$other$ind.metrics$`TL (mm)` <- as.integer(gl$other$ind.metrics$`TL (mm)`)
gl <- gl[(gl$other$ind.metrics$`TL (mm)` > 1500 &
            !is.na(gl$other$ind.metrics$`TL (mm)`)) | gl$pop == "Fiji",] #Because all Fiji samples w
gl <- gl[!gl$other$ind.metrics$Site %in% c("Caribbean Sea", "Sierra Leone",
                                           "Indonesia", "Japan"),] #need at least 2 samples per grou
Pop <- gl$pop

table( gl$other$ind.metrics$Site, gl$other$ind.metrics$COMBINED_SEX)

df <- as.data.frame(gl)
df[df == 0] <- 11
df[df == 1] <- 12
df[df == 2] <- 22
dat <- data.frame(as.character(Pop) , df)
sex <- gl$other$ind.metrics$Sex
dat <- dat[sex != "U",]
sex <- sex[sex != "U"]

FST <- hierfstat::sexbias.test(dat, sex, nperm = 100,
                               test = "FST",alternative = "two.sided")
#for mean you need at least 2 individuals per group
FIS <- hierfstat::sexbias.test(dat, sex, nperm = 100,
                               test = "FIS",alternative = "two.sided")
#for mean you need at least 2 individuals per group
mAIc <- hierfstat::sexbias.test(dat, sex, nperm = 100,
                                test = "mAIc",alternative = "two.sided")
#for mean you need at least 2 individuals per group
vAIc <- hierfstat::sexbias.test(dat, sex, nperm = 100,
                                test = "vAIc",alternative = "two.sided")
#for mean you need at least 2 individuals per group

aic <- hierfstat::AIc(dat)
plot.data <- data.frame(sex, aic)
rownames(plot.data) <- rownames(dat)

save(FST, FIS, mAIc, vAIc, aic, plot.data,
     file = "DArTcap_ALL_Aic_sex_Sites_OnlyAdults.Rdata")
```

```
load("DArTcap_ALL_Aic_sex_Sites_OnlyAdults.Rdata")

print(FST)
print(FIS)
print(mAIc)
print(vAIc)
```

```r
sex <- plot.data$sex
aic.F <- aic[sex == "F"]
aic.M <- aic[sex == "M"]

maic.F <- mean(aic.F, na.rm = TRUE)
maic.M <- mean(aic.M, na.rm = TRUE)
vaic.F <- var(aic.F, na.rm = TRUE)
vaic.M <- var(aic.M, na.rm = TRUE)


df <- data.frame(INDIVIDUALS = BS.dartcap.gl$ind.names,
                 pop = BS.dartcap.gl$other$ind.metrics$Site)
test.data <- data.frame(INDIVIDUALS = rownames(plot.data),plot.data,
                        stringsAsFactors = FALSE)

test.data %<>% dplyr::left_join(df, by = "INDIVIDUALS")
plot.data <- test.data
pop.levels <- c("Gulf of Mexico", "Western North Atlantic","Mozambique",
                "Reunion" , "Seychelles", "Arabian Sea", "Sri Lanka",
                "Australia", "Fiji")
plot.data$pop <- factor(plot.data$pop, levels = pop.levels)

aic.plot <- ggplot2::ggplot() +
  ggplot2::geom_histogram(data = subset(plot.data, sex == "F"),
                          ggplot2::aes(aic, fill = "F", y = ..count..),
                          binwidth = 5) +
  ggplot2::geom_histogram(data = subset(plot.data, sex == "M"),
                          ggplot2::aes(aic, fill = "M", y = -..count..),
                          binwidth = 5) +
  ggplot2::geom_hline(yintercept = 0) +
  ggplot2::scale_fill_manual(values = c("#E69F00", "#56B4E9"),
                             labels = c("Female", "Male")) +
  ggplot2::labs(x = "Corrected assignment index",
                y = "Number of individuals",
                fill = "") +
  ggplot2::facet_grid(pop~., scales = "free_y") +
  ggplot2::theme_minimal() +
  ggplot2::theme(
    axis.text.x = ggplot2::element_text(size = 10, angle = 0, face = "bold"),
    axis.text.y = ggplot2::element_text(size = 10, angle = 0, face = "bold"),
    axis.title = ggplot2::element_text(size = 15),
    legend.text = ggplot2::element_text(hjust = 0, size = 15),
    legend.title = ggplot2::element_text(hjust = 0, size = 15),
    strip.text.y = element_text(angle = 0,size = 10)
  )
print(aic.plot)
```

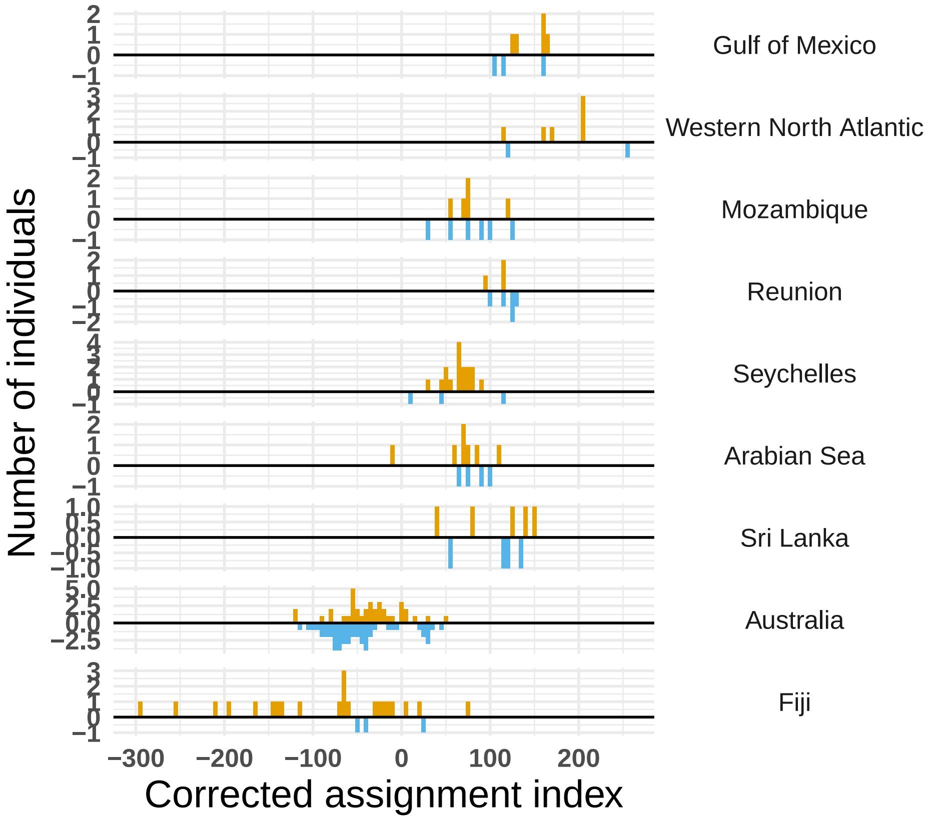


#### 8.11.2.1 Individual assignment

```
ggplot2::ggsave(plot = aic.plot,
                filename = "DArTcap_ALL_Aic_sex_Sites_OnlyAdults3.png",
       width = 30, height = 20, units = "cm")

res <- plot.data[(plot.data$pop == "Arabian Sea" & plot.data$aic < 0 &
                    !is.na(plot.data$aic)) |
                 (plot.data$pop == "Australia" & plot.data$aic < (-106) &
                    !is.na(plot.data$aic)) |
                 (plot.data$pop == "Fiji" & plot.data$aic < (-100) &
                    !is.na(plot.data$aic)), ]

knitr::kable(res, digits = 4,
             caption = "Individuals with low population assignment indices") %>%
  kableExtra::kable_styling(full_width = FALSE,font_size = 9,
                            latex_options = c( "hold_position"))
```

```
## $call
## hierfstat::sexbias.test(dat = dat, sex = sex, nperm = 100, test = "FST",
##     alternative = "two.sided")
##
## $statistic
## [1] 0.02967566
##
## $p.value
## [1] 1
##
## $call
## hierfstat::sexbias.test(dat = dat, sex = sex, nperm = 100, test = "FIS",
##     alternative = "two.sided")
##
## $statistic
```

```
## [1] 0.01131135
##
## $p.value
## [1] 0.63
##
## $call
## hierfstat::sexbias.test(dat = dat, sex = sex, nperm = 100, test = "mAIc",
##     alternative = "two.sided")
##
## $statistic
## [1] 0.5283601
##
## $p.value
## [1] 0.48
##
## $call
## hierfstat::sexbias.test(dat = dat, sex = sex, nperm = 100, test = "vAIc",
##     alternative = "two.sided")
##
## $statistic
## [1] 1.386015
##
## $p.value
## [1] 0.12
```

Table 27: Individuals with low population assignment indices

| | INDIVIDUALS | sex | aic | pop |
|---|---|---|---|---|
| 58 | CL-ARS012-F | F | -9.2111 | Arabian Sea |
| 147 | CLSH047-F | F | -118.9014 | Australia |
| 153 | CLSH055-M | M | -116.2028 | Australia |
| 157 | CLSH059-F | F | -119.5633 | Australia |
| 173 | CL-FIJ005-F.1 | F | -146.8808 | Fiji |
| 186 | CL-FIJ021-F | F | -253.5579 | Fiji |
| 187 | CL-FIJ022-F | F | -292.7706 | Fiji |
| 188 | CL-FIJ023-F | F | -138.5194 | Fiji |
| 189 | CL-FIJ024-F | F | -116.8141 | Fiji |
| 190 | CL-FIJ026-F | F | -162.5851 | Fiji |
| 191 | CL-FIJ027-F | F | -132.5392 | Fiji |
| 192 | CL-FIJ029-F | F | -195.0195 | Fiji |
| 193 | CL-FIJ031-F | F | -208.0313 | Fiji |

```r
strata <- BS.dartcap.gt@schemes$Site
names(strata) <- BS.dartcap.gt@schemes$INDIVIDUALS
strataG::setStrata(BS.dartcap.gt) <- strata

BS.dartcap.gt@schemes$`TL (mm)` <- as.integer(BS.dartcap.gt@schemes$`TL (mm)`)
gt.all.F <- BS.dartcap.gt[BS.dartcap.gt@schemes$COMBINED_SEX == "F" &
                                     (BS.dartcap.gt@schemes$`TL (mm)` > 1500 &
                                     !is.na(BS.dartcap.gt@schemes$`TL (mm)`)|
                                       BS.dartcap.gt@schemes$Site == "Fiji"),]
gt.all.M <- BS.dartcap.gt[BS.dartcap.gt@schemes$COMBINED_SEX == "M" &
                                     BS.dartcap.gt@schemes$ `TL (mm)` > 1500 &
                                     (!is.na(BS.dartcap.gt@schemes$`TL (mm)`)|
                                        BS.dartcap.gt@schemes$Site == "Fiji"),]

Fst.all.pop.F <-
```

```r
  strataG::popStructTest(
    gt.all.F,
    nrep = 1000,
    stats = c("fst", "fis"),
    type = "pairwise",
    keep.null = FALSE,
    quietly = TRUE,
    max.cores = parallel::detectCores() - 1,
    write.output = FALSE
  )
Fst.all.pop.M <-
  strataG::popStructTest(
    gt.all.M,
    nrep = 1000,
    stats = c("fst", "fis"),
    type = "pairwise",
    keep.null = FALSE,
    quietly = TRUE,
    max.cores = parallel::detectCores() - 1,
    write.output = FALSE
  )
save(Fst.all.pop.F, Fst.all.pop.M, file = "DArTcap_ALL_Fst_sex_Site2.Rdata")
```

```r
load("DArTcap_ALL_Fst_sex_Site2.Rdata")
dim(Fst.all.pop.F$pairwise$result) #91  8
dim(Fst.all.pop.M$pairwise$result) #66  8

names.1 <- paste0(Fst.all.pop.F$pairwise$result$strata.1,"_",
                  Fst.all.pop.F$pairwise$result$strata.2)
names.2 <- paste0(Fst.all.pop.M$pairwise$result$strata.1,"_",
                  Fst.all.pop.M$pairwise$result$strata.2)

result.1 <- Fst.all.pop.F$pairwise$result
result.1$names.comb <- names.1
result.2 <- Fst.all.pop.M$pairwise$result
result.2$names.comb <- names.2

data <- left_join(result.1,result.2, by = "names.comb" )

pwFst.FM <- data[,c(1:6, 12:15)]
colnames(pwFst.FM) <- c("strata.1", "strata.2", "n.auDNA.F.1", "n.auDNA.F.2",
                        "Fst.auDNA.F", "Fst.auDNA.F.Pval",
                     "n.auDNA.M.1", "n.auDNA.M.2", "Fst.auDNA.M",
                     "Fst.auDNA.M.Pval")
knitr::kable(pwFst.FM, digits = 4,
             caption = "Pairwise Fst between females and males for autosomal markers") %>%
  kableExtra::kable_styling(full_width = FALSE,font_size = 5,
                            latex_options = c( "hold_position")) %>%
  kableExtra::landscape()
```

#### 8.11.2.2 Fst test

```
## [1] 36  8
## [1] 21  8
```

## Table 28: Pairwise Fst between females and males for autosomal markers

| strata.1 | strata.2 | n.auDNA.F.1 | n.auDNA.F.2 | Fst.auDNA.F | Fst.auDNA.F.Pval | n.auDNA.M.1 | n.auDNA.M.2 | Fst.auDNA.M | Fst.auDNA.M.Pval |
|---|---|---|---|---|---|---|---|---|---|
| Arabian Sea | Australia | 12 | 48 | 0.0016 | 0.1199 | NA | NA | NA | NA |
| Arabian Sea | Brazil | 12 | 29 | 0.6526 | 0.0010 | NA | NA | NA | NA |
| Arabian Sea | Fiji | 12 | 5 | 0.0288 | 0.0010 | NA | NA | NA | NA |
| Arabian Sea | Japan | 12 | 2 | NA | NA | NA | NA | NA | NA |
| Arabian Sea | Mozambique | 12 | 3 | NA | NA | NA | NA | NA | NA |
| Arabian Sea | Reunion | 12 | 1 | NA | NA | NA | NA | NA | NA |
| Arabian Sea | Seychelles | 12 | 4 | -0.0050 | 0.8452 | NA | NA | NA | NA |
| Arabian Sea | South Africa | 12 | 11 | 0.0033 | 0.0699 | NA | NA | NA | NA |
| Australia | Brazil | 48 | 29 | 0.6449 | 0.0010 | 62 | 10 | 0.6375 | 0.0010 |
| Australia | Fiji | 48 | 5 | 0.0292 | 0.0010 | 62 | 4 | NA | NA |
| Australia | Japan | 48 | 2 | NA | NA | NA | NA | NA | NA |
| Australia | Mozambique | 48 | 3 | NA | NA | 62 | 1 | NA | NA |
| Australia | Reunion | 48 | 1 | NA | NA | NA | NA | NA | NA |
| Australia | Seychelles | 48 | 4 | -0.0020 | 0.6863 | 62 | 3 | 0.0085 | 0.0570 |
| Australia | South Africa | 48 | 11 | 0.0047 | 0.0020 | 62 | 2 | NA | NA |
| Brazil | Fiji | 29 | 5 | 0.6589 | 0.0011 | 10 | 4 | 0.6483 | 0.0034 |
| Brazil | Japan | 29 | 2 | 0.6688 | 0.0444 | NA | NA | NA | NA |
| Brazil | Mozambique | 29 | 3 | 0.6553 | 0.0055 | 10 | 1 | NA | NA |
| Brazil | Reunion | 29 | 1 | NA | NA | NA | NA | NA | NA |
| Brazil | Seychelles | 29 | 4 | 0.6572 | 0.0014 | 10 | 3 | 0.6580 | 0.0079 |
| Brazil | South Africa | 29 | 11 | 0.6546 | 0.0010 | 10 | 2 | 0.6600 | 0.0755 |
| Fiji | Japan | 5 | 2 | NA | NA | NA | NA | NA | NA |
| Fiji | Mozambique | 5 | 3 | 0.0214 | 0.0334 | 4 | 1 | NA | NA |
| Fiji | Reunion | 5 | 1 | NA | NA | NA | NA | NA | NA |
| Fiji | Seychelles | 5 | 4 | 0.0150 | 0.0300 | 4 | 3 | 0.0318 | 0.0545 |
| Fiji | South Africa | 5 | 11 | 0.0240 | 0.0030 | 4 | 2 | 0.0306 | 0.2331 |
| Japan | Mozambique | 2 | 3 | 0.0687 | 0.1755 | NA | NA | NA | NA |
| Japan | Reunion | 2 | 1 | 0.2182 | 0.3297 | NA | NA | NA | NA |
| Japan | Seychelles | 2 | 4 | 0.0917 | 0.0904 | NA | NA | NA | NA |
| Japan | South Africa | 2 | 11 | NA | NA | NA | NA | NA | NA |
| Mozambique | Reunion | 3 | 1 | NA | NA | NA | NA | NA | NA |
| Mozambique | Seychelles | 3 | 4 | NA | NA | 1 | 3 | NA | NA |
| Mozambique | South Africa | 3 | 11 | NA | NA | 1 | 2 | 0.0748 | 0.3377 |
| Reunion | Seychelles | 1 | 4 | NA | NA | NA | NA | NA | NA |
| Reunion | South Africa | 1 | 11 | NA | NA | NA | NA | NA | NA |
| Seychelles | South Africa | 4 | 11 | -0.0088 | 0.9590 | 3 | 2 | 0.0418 | 0.1094 |

```r
pwFis.FM <- data[,c(1:4,7:8, 12:13,16:17)]
colnames(pwFis.FM) <- c("strata.1", "strata.2", "n.auDNA.F.1", "n.auDNA.F.2",
                        "Fis.auDNA.F", "Fis.auDNA.F.Pval",
                     "n.auDNA.M.1", "n.auDNA.M.2", "Fis.auDNA.M",
                     "Fis.auDNA.M.Pval")
knitr::kable(pwFis.FM, digits = 4,
             caption = "Pairwise Fis between females and males for autosomal markers") %>%
  kableExtra::kable_styling(full_width = FALSE,font_size = 5,
                            latex_options = c( "hold_position")) %>%
  kableExtra::landscape()
```

#### 8.11.2.3 Fis test

Table 29: Pairwise Fis between females and males for autosomal markers

| strata.1 | strata.2 | n.auDNA.F.1 | n.auDNA.F.2 | Fis.auDNA.F | Fis.auDNA.F.Pval | n.auDNA.M.1 | n.auDNA.M.2 | Fis.auDNA.M | Fis.auDNA.M.Pval |
|---|---|---|---|---|---|---|---|---|---|
| Arabian Sea | Australia | 12 | 48 | -9.0058 | 0.1858 | NA | NA | NA | NA |
| Arabian Sea | Brazil | 12 | 29 | -9.0370 | 1.0000 | NA | NA | NA | NA |
| Arabian Sea | Fiji | 12 | 5 | -125.9573 | 1.0000 | NA | NA | NA | NA |
| Arabian Sea | Japan | 12 | 2 | NA | NA | NA | NA | NA | NA |
| Arabian Sea | Mozambique | 12 | 3 | NA | NA | NA | NA | NA | NA |
| Arabian Sea | Reunion | 12 | 1 | NA | NA | NA | NA | NA | NA |
| Arabian Sea | Seychelles | 12 | 4 | 367.8651 | 0.6845 | NA | NA | NA | NA |
| Arabian Sea | South Africa | 12 | 11 | -17.2164 | 0.9111 | NA | NA | NA | NA |
| Australia | Brazil | 48 | 29 | -5.1005 | 1.0000 | 62 | 10 | -6.2749 | 1.0000 |
| Australia | Fiji | 48 | 5 | -17.6149 | 1.0000 | 62 | 4 | NA | NA |
| Australia | Japan | 48 | 2 | NA | NA | NA | NA | NA | NA |
| Australia | Mozambique | 48 | 3 | NA | NA | 62 | 1 | NA | NA |
| Australia | Reunion | 48 | 1 | NA | NA | NA | NA | NA | NA |
| Australia | Seychelles | 48 | 4 | -22.5505 | 0.6372 | 62 | 3 | -43.3537 | 0.9922 |
| Australia | South Africa | 48 | 11 | -9.6001 | 0.3437 | 62 | 2 | NA | NA |
| Brazil | Fiji | 29 | 5 | -25.5253 | 1.0000 | 10 | 4 | NA | NA |
| Brazil | Japan | 29 | 2 | NA | NA | NA | NA | NA | NA |
| Brazil | Mozambique | 29 | 3 | NA | NA | 10 | 1 | NA | NA |
| Brazil | Reunion | 29 | 1 | NA | NA | NA | NA | NA | NA |
| Brazil | Seychelles | 29 | 4 | -36.1107 | 1.0000 | 10 | 3 | 20.0861 | 0.1136 |
| Brazil | South Africa | 29 | 11 | -9.8749 | 1.0000 | 10 | 2 | NA | NA |
| Fiji | Japan | 5 | 2 | NA | NA | NA | NA | NA | NA |
| Fiji | Mozambique | 5 | 3 | NA | NA | 4 | 1 | NA | NA |
| Fiji | Reunion | 5 | 1 | NA | NA | NA | NA | NA | NA |
| Fiji | Seychelles | 5 | 4 | 15.6026 | 0.4627 | 4 | 3 | NA | NA |
| Fiji | South Africa | 5 | 11 | -662.8514 | 0.9960 | 4 | 2 | NA | NA |
| Japan | Mozambique | 2 | 3 | NA | NA | NA | NA | NA | NA |
| Japan | Reunion | 2 | 1 | NA | NA | NA | NA | NA | NA |
| Japan | Seychelles | 2 | 4 | NA | NA | NA | NA | NA | NA |
| Japan | South Africa | 2 | 11 | NA | NA | NA | NA | NA | NA |
| Mozambique | Reunion | 3 | 1 | NA | NA | NA | NA | NA | NA |
| Mozambique | Seychelles | 3 | 4 | NA | NA | 1 | 3 | NA | NA |
| Mozambique | South Africa | 3 | 11 | NA | NA | 1 | 2 | NA | NA |
| Reunion | Seychelles | 1 | 4 | NA | NA | NA | NA | NA | NA |
| Reunion | South Africa | 1 | 11 | NA | NA | NA | NA | NA | NA |
| Seychelles | South Africa | 4 | 11 | 106.2843 | 0.6607 | 3 | 2 | NA | NA |

**8.11.2.4 Spatial correlation** Test the correlation between the genetic and geographic distance between the sampling sites. The genetic distance is calculated based on Fst and Euclidean distance.

```
# geographic file that contain coordinates of the 8 sampling locations
coord.sites <- readr::read_csv(file = "Least_cost_distance_Sites.csv")
coord.sites <- coord.sites[!coord.sites$Site %in% c("Costa.Rica", "Brazil",
                                                     "Caribbean.Sea", "Sierra.Leone",
                                                     "South.Africa", "Thailand",
                                                     "Indonesia", "Papua.New.Guinea",
                                                     "Japan"),]

world <- marmap::getNOAA.bathy(lon1 = -180, lon2 = 180, lat1 = -90, lat2 = 90,
                              resolution = 10) #Res 10 good for calculating a least cost path

dist.sites <- round(fossil::earth.dist(as.matrix(coord.sites[,-1])), 0)
trans1 <- marmap::trans.mat(world)
lc.dist.sites1 <- round(marmap::lc.dist(trans1, as.matrix(coord.sites[,-1]),
                                        res = "dist"), 0)
trans2 <- marmap::trans.mat(world,min.depth=-1, max.depth=-200)
lc.dist.sites2 <- round(marmap::lc.dist(trans2, as.matrix(coord.sites[,-1]),
                                        res = "dist"), 0)



pop.levels <- c("Costa Rica" , "Brazil","Caribbean Sea",  "Gulf of Mexico",
                "Western North Atlantic", "Sierra Leone", "South Africa",
                "Mozambique", "Reunion" , "Seychelles", "Arabian Sea", "Sri Lanka",
                "Thailand", "Indonesia", "Papua New Guinea" , "Australia",
                "Japan", "Fiji")
gl <- BS.dartcap.gl
gl$pop <- factor(gl$other$ind.metrics$Site, levels = pop.levels)
gl <- gl[order(gl$pop,gl$ind.names)]

gl$other$ind.metrics$`TL (mm)` <- as.integer(gl$other$ind.metrics$`TL (mm)`)
gl <- gl[(gl$other$ind.metrics$`TL (mm)` > 1500 &
            !is.na(gl$other$ind.metrics$`TL (mm)`)) | gl$pop == "Fiji",] #Because all Fiji samples w
gl <- gl[!gl$other$ind.metrics$Site %in% c("Caribbean Sea", "Sierra Leone",
                                          "Indonesia", "Japan"),] #need at least 2 samples per grou
table(gl$pop, gl$other$ind.metrics$COMBINED_SEX)
gl.F <- gl[gl@other$ind.metrics$COMBINED_SEX == "F",]
gl.M <- gl[gl@other$ind.metrics$COMBINED_SEX == "M",]

gi.F <- gl2gi_mvb(gl.F)
gi.M <- gl2gi_mvb(gl.M)
gpop <- adegenet::genind2genpop(gi.F)
Eucl.D.F <- adegenet::dist.genpop(gpop,method = 2) #method 2 Euclidean = Angular distance
gpop <- adegenet::genind2genpop(gi.M)
Eucl.D.M <- adegenet::dist.genpop(gpop,method = 2) #method 2 Euclidean = Angular distance

save(world, dist.sites, lc.dist.sites1, lc.dist.sites2, Eucl.D.F,
     Eucl.D.M, file = "DArTcap_ALL_autocorr_by_sex_Sites2.Rdata")
```

```
load("DArTcap_ALL_autocorr_by_sex_Sites2.Rdata")
load("DArTcap_ALL_autocorr_by_sex_Sites.Rdata")

Dgeo <- lc.dist.sites1
Dgen.F <- Eucl.D.F
Dgen.M <- Eucl.D.M
```

```
fst.test.F <- ade4::mantel.randtest(Dgen.F, Dgeo, nrepet = 10000)
plot(fst.test.F)
```

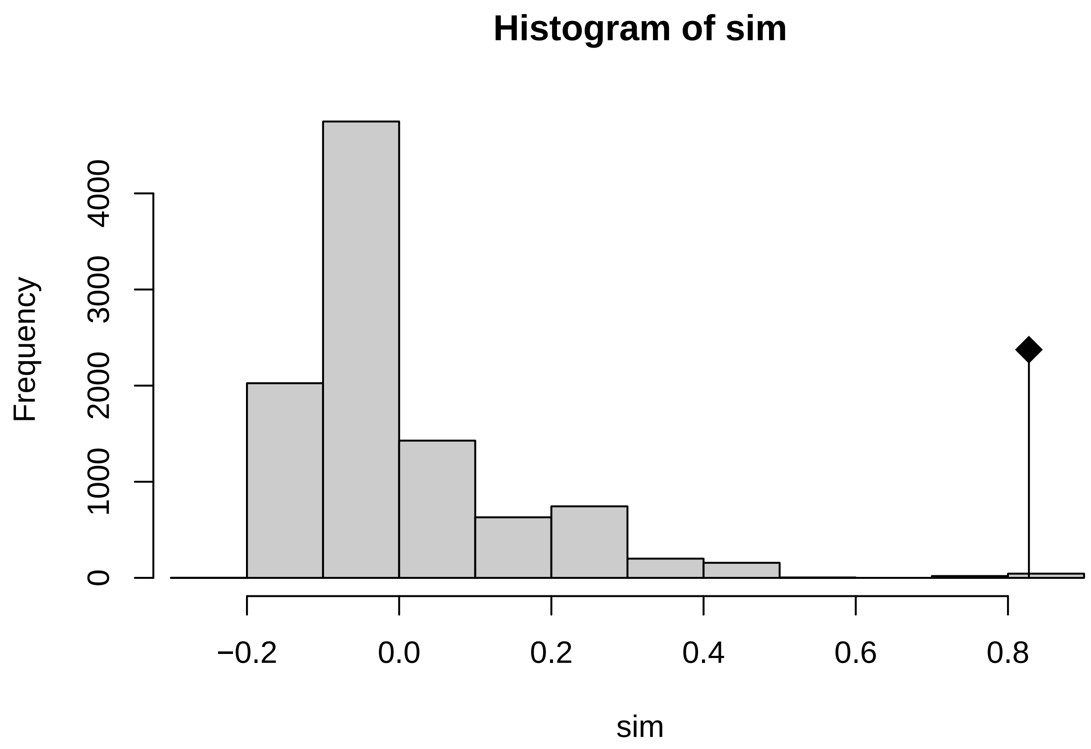


```
fst.test.F
fst.test.M <- ade4::mantel.randtest(Dgen.M, Dgeo, nrepet = 10000)
plot(fst.test.M)
```

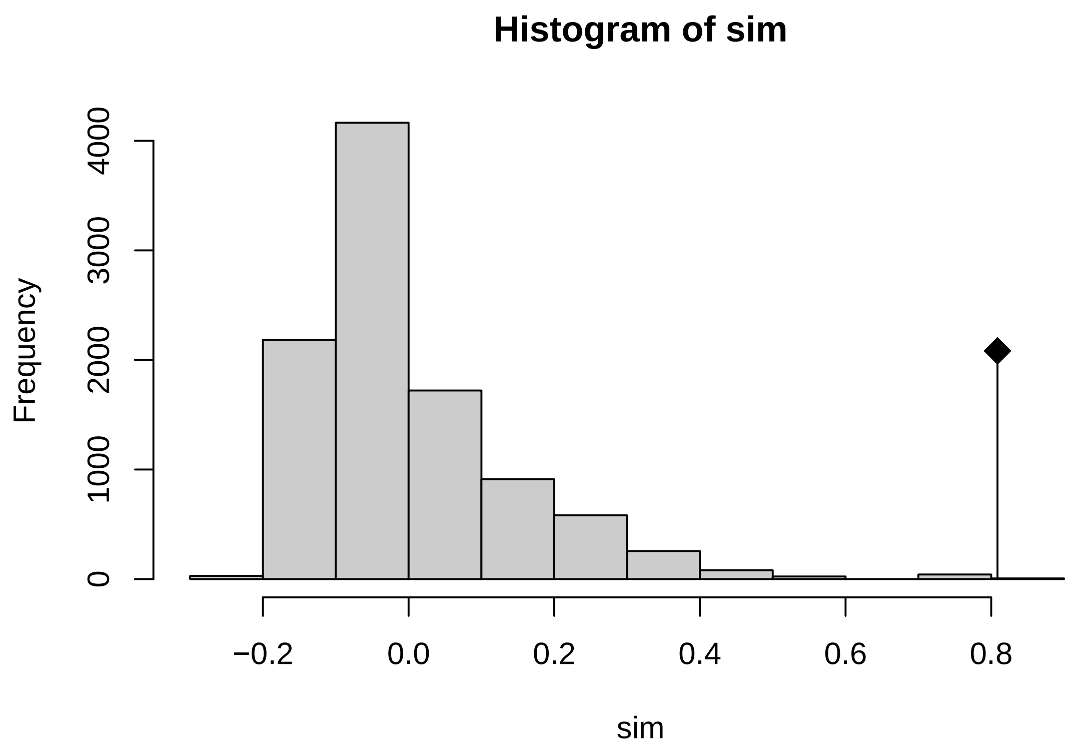


```
fst.test.M

corr.test.F <- vegan::mantel.correlog(
  Dgen.F, D.geo = Dgeo, XY = NULL, n.class = 11, break.pts = NULL,
  cutoff = FALSE, r.type = "pearson", nperm = 10000, mult = "holm",
  progressive = TRUE)
corr.test.F.data <- as.data.frame(corr.test.F$mantel.res)
corr.test.F.data$Sex <- "Female"

corr.test.M <- vegan::mantel.correlog(
  Dgen.M, D.geo = Dgeo, XY = NULL, n.class = 11, break.pts = NULL,
  cutoff = FALSE, r.type = "pearson", nperm = 10000, mult = "holm",
  progressive = TRUE)
corr.test.M.data <- as.data.frame(corr.test.M$mantel.res)
corr.test.M.data$Sex <- "Male"


plot.data <- rbind(corr.test.F.data, corr.test.M.data)

sig <- ifelse(plot.data$Pr.corrected. <= 0.05, "sig", "non-sig")
# sig <- ifelse(plot.data$Pr.Mantel. <= 0.05, "sig", "non-sig")
sig_cat <- sig
sig_cat[sig_cat == "sig" & plot.data$Mantel.cor < 0] <- "Lower"
sig_cat[sig_cat == "sig" & plot.data$Mantel.cor > 0] <- "Higher"
sig_cat[sig_cat == "non-sig"] <- "Neutral"
plot.data$sig <- factor(sig, levels = c("sig", "non-sig"))
plot.data$sig_cat <- factor(sig_cat, levels = c("Lower", "Neutral", "Higher"))


plot <- ggplot2::ggplot(plot.data, ggplot2::aes(x = class.index,
                                                y = Mantel.cor)) +
```

```r
  ggplot2::geom_hline(yintercept = 0,
             linetype = "dotted",
             size = 0.5) +
  ggplot2::geom_line(ggplot2::aes(group = Sex, color = Sex),
                     size = 1) +
  ggplot2::geom_point(ggplot2::aes(fill = sig_cat), size = 4,
                      shape = 21) +
  ggplot2::scale_fill_manual(values = c(alpha("darkblue", 0.6),
                                        gray.colors(12)[6], "red"),
                             labels = c("Signifcantly lower",
                                        "Non-significant",
                                        "Signifcantly higher")) +
  ggplot2::scale_color_manual(values = c("#E69F00", "#56B4E9")) +
  ggplot2::theme_bw() +
  ggplot2::theme(
    panel.background = ggplot2::element_rect(color = "white", fill = "white"),
    panel.grid.major.x = ggplot2::element_line(size = 0.1, color = "black"),
    axis.line = ggplot2::element_line(color = "black", size = 0.35),
    axis.text.x = ggplot2::element_text(size = 10, angle = 0, face = "bold"),
    axis.title = ggplot2::element_text(size = 15),
    axis.ticks = ggplot2::element_line(size = 0.15),
    legend.text = ggplot2::element_text(hjust = 0, size = 10),
    legend.title = ggplot2::element_text(hjust = 0, size = 10),
    panel.grid.major.y = ggplot2::element_blank(),
    panel.grid.minor.y = ggplot2::element_blank(),
    legend.position = "top",
    strip.text = ggplot2::element_text(size = 10)
  ) +
  # ggplot2::labs(title = "Euclidean distance") +
  ggplot2::xlab("Pairwise geographic distance (km)") +
  ylab("Mantel spatial correlation") +
  ggplot2::guides(
    fill = ggplot2::guide_legend(title = ""),
    color = ggplot2::guide_legend(title = "")
  )
print(plot)
```

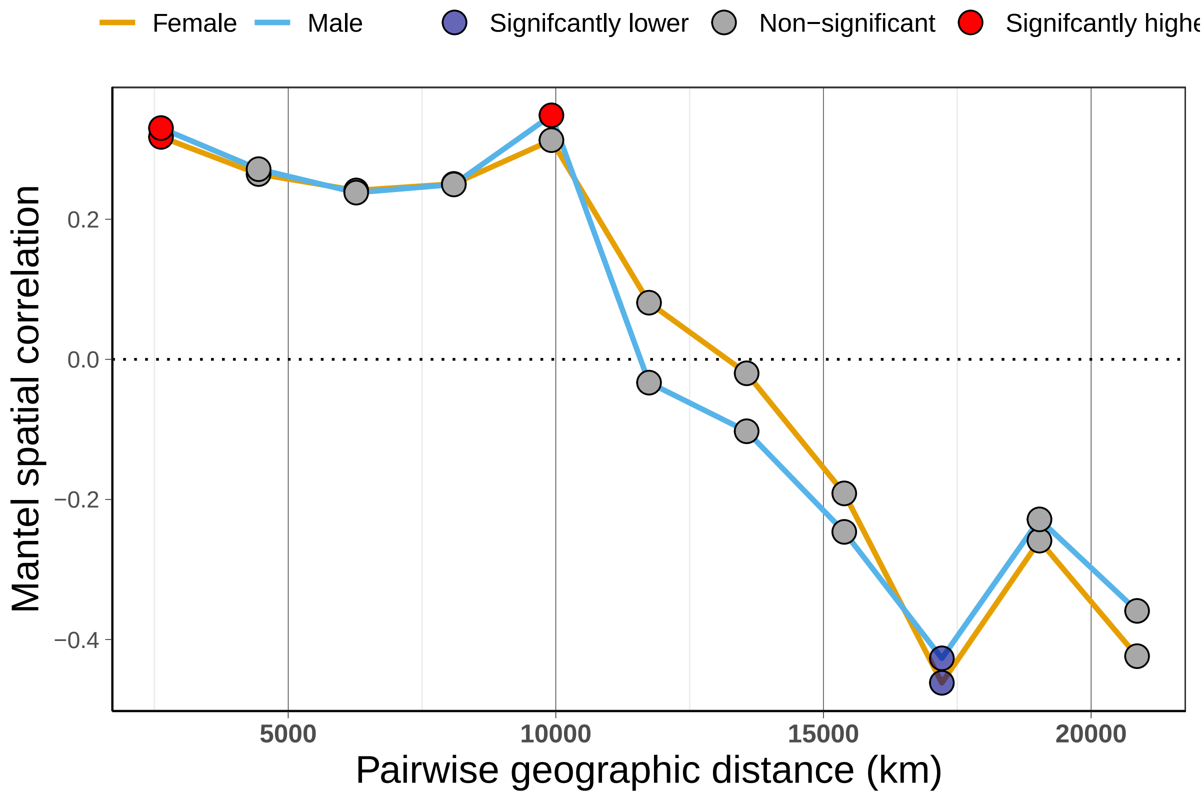


```r
ggplot2::ggsave(plot = plot,
                filename = "DArTcap_ALL_autocorr_by_sex_Sites_EUCL_FM3.png",
       width = 30, height = 15, units = "cm")
```

```
## Monte-Carlo test
## Call: ade4::mantel.randtest(m1 = Dgen.F, m2 = Dgeo, nrepet = 10000)
##
## Observation: 0.827511
##
## Based on 10000 replicates
## Simulated p-value: 0.00019998
## Alternative hypothesis: greater
##
##       Std.Obs   Expectation      Variance
##  5.700761e+00 -7.302451e-05  2.107456e-02
## Monte-Carlo test
## Call: ade4::mantel.randtest(m1 = Dgen.M, m2 = Dgeo, nrepet = 10000)
##
## Observation: 0.8086319
##
## Based on 10000 replicates
## Simulated p-value: 0.00039996
## Alternative hypothesis: greater
##
##       Std.Obs   Expectation      Variance
##  5.7551025251 -0.0002985323  0.0197567815
```

```r
gl <- BS.dartcap.gl
pop.levels <- c("Costa Rica" ,"Brazil", "Caribbean Sea",  "Gulf of Mexico",
                "Western North Atlantic",  "Sierra Leone", "South Africa",
```

```
                 "Mozambique", "Reunion" , "Seychelles", "Arabian Sea",
                 "Sri Lanka", "Thailand", "Indonesia", "Papua New Guinea" ,
                 "Australia", "Japan", "Fiji")
gl <- BS.dartcap.gl
gl$pop <- factor(gl$other$ind.metrics$Site, levels = pop.levels)
gl <- gl[order(gl$pop, gl$ind.names)]
gl$other$ind.metrics$`TL (mm)` <- as.integer(gl$other$ind.metrics$`TL (mm)`)
gl <- gl[(gl$other$ind.metrics$`TL (mm)` > 1500 &
            !is.na(gl$other$ind.metrics$`TL (mm)`)) | gl$pop == "Fiji",] #Because all Fiji samples w
gl <- gl[!gl$other$ind.metrics$Site %in% c("Caribbean Sea", "Sierra Leone", "Indonesia",
                                            "Japan"),] #need at least 2 samples per group
gl$other$loc.metrics.flags$monomorphs <- FALSE
gl$other$loc.metrics.flags$CallRate  <- FALSE


gl2rel <- function(gl, filename = "relate"){
  group.name <- paste(gl$other$ind.metrics$COMBINED_SEX,
                      gl$other$ind.metrics$COMBINED_SEX, "_",
                      gl$ind.names, sep = "")
  glmat <- as.matrix(gl)
  glmat[glmat==0] <- '2 2'
  glmat[glmat==1] <- '1 2'
  glmat[glmat==2] <- '1 1'
  glmat[is.na(glmat)] <- '0 0'
  df <- cbind(group.name, glmat)
  write.table(df, file = paste0(filename,".txt", sep = ""), quote = FALSE,
              sep = " ", row.names = FALSE, col.names = FALSE)
}


res.rel <- list()
summ.rel <- data.frame(sex.group = c("FF", "FM", "MM"))
plot.data <- data.frame(pair.no = NA,ind1.id = NA,ind2.id = NA, group = NA,
                        condition = NA, measurement = NA,Site = NA)
for (i in 1:nlevels(gl$pop)) {
  print(i)
  gl.tmp <- gl[gl$pop == levels(gl$pop)[i],]
  gl.tmp <- dartR::gl.filter.callrate(gl.tmp, method = 'loc', threshold = 1,
                                      mono.rm = TRUE, plot = FALSE, verbose = 0)

  gl2rel(gl.tmp, filename = "relate")
  data <- related::readgenotypedata("relate.txt")
  outfile <- related::coancestry (data$gdata , lynchli = 1, lynchrd = 1,
                                  quellergt = 1, wang = 1, ritland = 1)
  res <- outfile$relatedness[!outfile$relatedness$group %in%
                               c("FFUU","UUUU","MMUU","UUFF", "UUMM"),]
  res$group[res$group == "MMFF"] <- "FFMM"
  data_long <- gather(res, condition, measurement, 5:11, factor_key=TRUE)
  data_long$Site <- levels(gl$pop)[i]
  plot.data <- rbind(plot.data, data_long)
  summ <- dplyr::group_by(res, group) %>%
    dplyr::summarise(mean(ritland))
  colnames(summ) <- c("sex.group", levels(gl$pop)[i])
  summ.rel <- cbind(summ.rel, summ[,2])
  res.rel[[i]] <- res
}
plot.data <- plot.data[-1,]
save(res.rel,summ.rel,plot.data, file = "DArTcap_ALL_relatedness_sex_Site2.Rdata")
```

```r
load("DArTcap_ALL_relatedness_sex_Site2.Rdata")
pop.levels <- c("Gulf of Mexico", "Western North Atlantic",
                "Mozambique", "Reunion" , "Seychelles", "Arabian Sea", "Sri Lanka",
                "Australia", "Fiji")
plot.data$Site <- factor(plot.data$Site, levels = pop.levels)
plot.data <- plot.data[plot.data$condition == "ritland",]
plot <- ggplot2::ggplot(plot.data, ggplot2::aes(x = group,
                                                y = measurement,
                                                fill = group)) +
  ggplot2::geom_boxplot() +
  ggplot2::facet_wrap(Site~.) +
  ggplot2::labs(x = "Site", y = "Relatedness (Ritland 1996)")
print(plot)
```

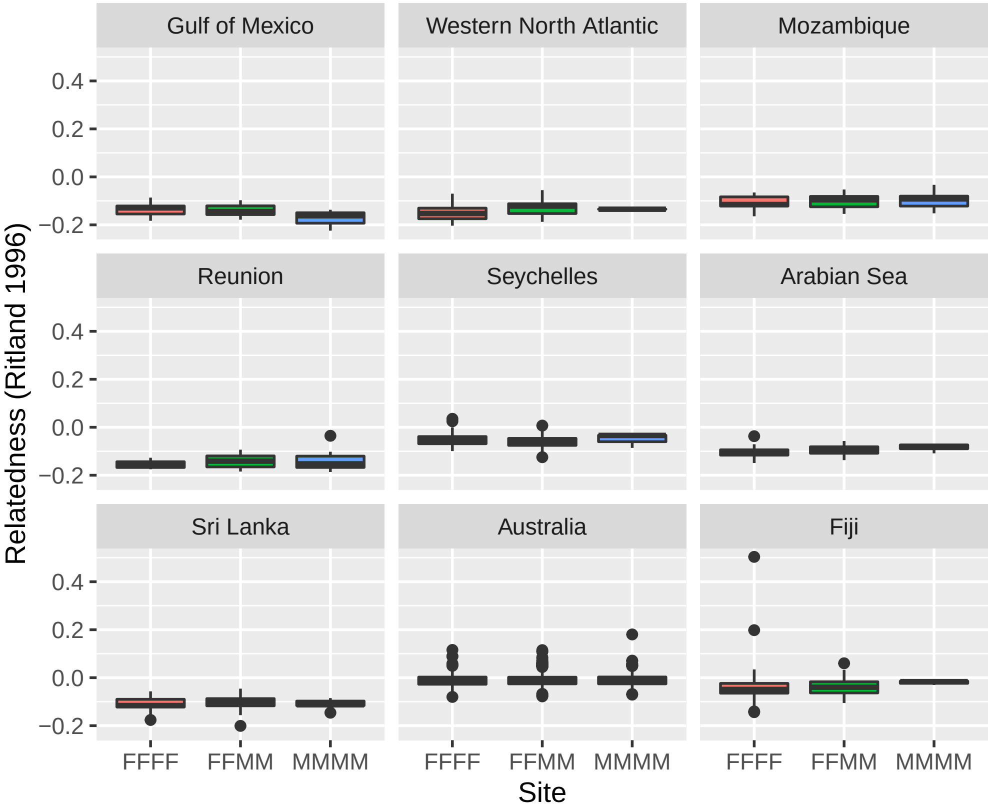


#### 8.11.2.5 Relatedness test

```r
knitr::kable(summ.rel, digits = 4,
             caption = "Relatedness between female and males according to Ritland (1996)") %>%
  kableExtra::kable_styling(full_width = FALSE,font_size = 5,
                            latex_options = c( "hold_position"))
```

Table 30: Relatedness between female and males according to Ritland (1996)

| sex.group | Gulf of Mexico | Western North Atlantic | Mozambique | Reunion | Seychelles | Arabian Sea | Sri Lanka | Australia | Fiji |
|---|---|---|---|---|---|---|---|---|---|
| FF | -0.1345 | -0.1476 | -0.1071 | -0.1541 | -0.0536 | -0.1026 | -0.1118 | -0.0115 | -0.0428 |
| FM | -0.1397 | -0.1266 | -0.1030 | -0.1414 | -0.0612 | -0.0968 | -0.1053 | -0.0108 | -0.0410 |
| MM | -0.1745 | -0.1349 | -0.0963 | -0.1383 | -0.0510 | -0.0838 | -0.1111 | -0.0107 | -0.0197 |

### 8.11.3 Individual-based tests by Region

```r
gl <- BS.dartcap.gl

pop.levels <- c("E-PAC","W-ATL","E-ATL","W-IO","N-IO","E-IO","W-PAC",
                "E-IO/W-PAC", "Japan","Fiji")
gl$pop <- factor(gl$other$ind.metrics$Region, levels = pop.levels)
gl$pop[gl$pop %in% c("E-IO","W-PAC")] <- "E-IO/W-PAC"
pop.levels <- c("E-PAC","W-ATL","E-ATL","W-IO","N-IO","E-IO/W-PAC",
                "Japan","Fiji")
gl$pop <- factor(gl$pop , levels = pop.levels)
gl <- gl[order(gl$pop, gl$ind.names),]

gl$other$ind.metrics$`TL (mm)` <- as.integer(gl$other$ind.metrics$`TL (mm)`)
gl <- gl[(gl$other$ind.metrics$`TL (mm)` > 1500 &
            !is.na(gl$other$ind.metrics$`TL (mm)`)) | gl$pop == "Fiji",] #Because all Fiji samples w
gl <- gl[!gl$other$ind.metrics$Site %in% c("E-ATL", "Japan"),]
Pop <- gl$pop

table(gl$pop, gl$other$ind.metrics$COMBINED_SEX)

df <- as.data.frame(gl)
df[df == 0] <- 11
df[df == 1] <- 12
df[df == 2] <- 22
dat <- data.frame(as.character(Pop) , df)
sex <- gl$other$ind.metrics$Sex
dat <- dat[sex != "U",]
sex <- sex[sex != "U"]
FST <- hierfstat::sexbias.test(dat, sex, nperm = 100,test = "FST",
                               alternative = "two.sided")
#for mean you need at least 2 individuals per group
FIS <- hierfstat::sexbias.test(dat, sex, nperm = 100,test = "FIS",
                               alternative = "two.sided")
#for mean you need at least 2 individuals per group
mAIc <- hierfstat::sexbias.test(dat, sex, nperm = 100,test = "mAIc",
                                alternative = "two.sided")
#for mean you need at least 2 individuals per group
vAIc <- hierfstat::sexbias.test(dat, sex, nperm = 100,test = "vAIc",
                                alternative = "two.sided")
#for mean you need at least 3 individuals per group

aic <- hierfstat::AIc(dat)
plot.data <- data.frame(sex, aic)
rownames(plot.data) <- rownames(dat)

save(FST, FIS, mAIc, vAIc, aic, plot.data,
     file = "DArTcap_ALL_Aic_sex_Regions_OnlyAdults.Rdata")
```

```r
load("DArTcap_ALL_Aic_sex_Regions_OnlyAdults.Rdata")

print(FST)
print(FIS)
print(mAIc)
print(vAIc)

sex <- plot.data$sex
aic.F <- aic[sex == "F"]
aic.M <- aic[sex == "M"]
```

```r
maic.F <- mean(aic.F, na.rm = TRUE)
maic.M <- mean(aic.M, na.rm = TRUE)
vaic.F <- var(aic.F, na.rm = TRUE)
vaic.M <- var(aic.M, na.rm = TRUE)

aic.plot <- ggplot2::ggplot() +
  ggplot2::geom_histogram(data = subset(plot.data, sex == "F"),
                          ggplot2::aes(aic, fill = "F", y =  ..count..)) +
  ggplot2::geom_histogram(data = subset(plot.data, sex == "M"),
                          ggplot2::aes(aic, fill = "M", y =  -..count..)) +
  ggplot2::geom_vline(ggplot2::aes(xintercept = maic.F), colour = "#E69F00") +
  ggplot2::geom_vline(ggplot2::aes(xintercept = maic.M), colour = "#56B4E9") +
  ggplot2::scale_fill_manual(values = c("#E69F00", "#56B4E9"),
                             labels = c("Female", "Male")) +
  ggplot2::labs(x = "Corrected assignment index",
                y = "Number of individuals",
                fill = "Sex") +
  ggplot2::theme_minimal()
print(aic.plot)
```

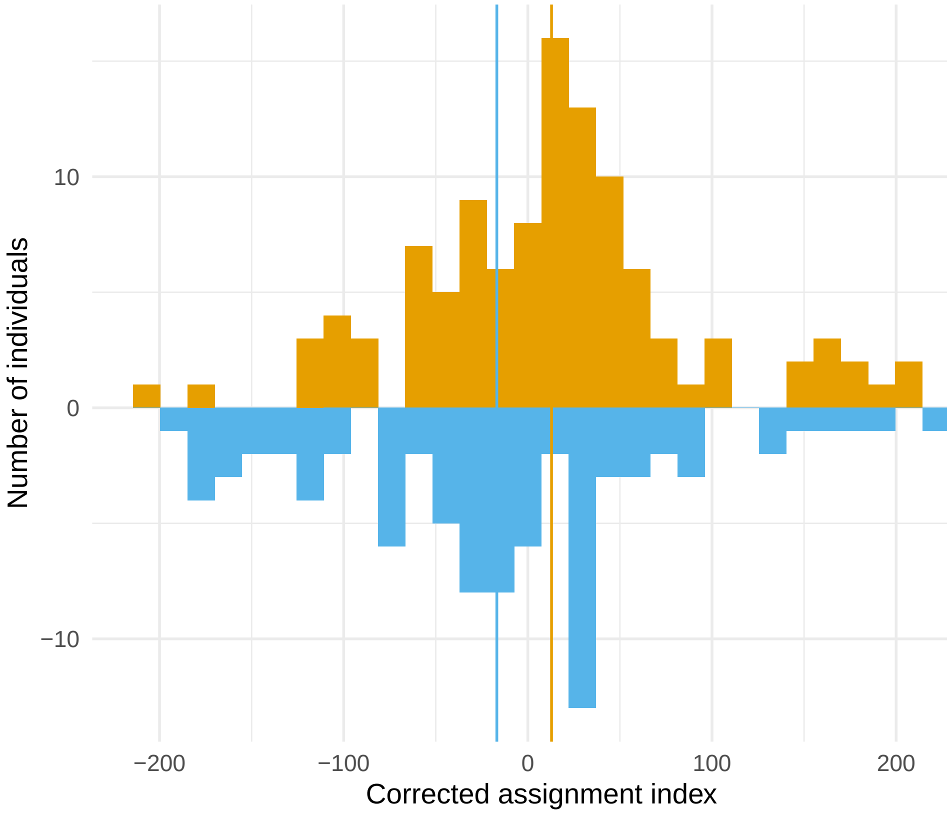


#### 8.11.3.1 Individual assignment

```r
region <- BS.dartcap.gl$other$ind.metrics$Region
region[region %in% c("E-IO","W-PAC")] <- "E-IO/W-PAC"
df <- data.frame(INDIVIDUALS = BS.dartcap.gl$ind.names, pop = region)
test.data <- data.frame(INDIVIDUALS = rownames(plot.data),plot.data,
                        stringsAsFactors = FALSE)
test.data %<>% dplyr::left_join(df, by = "INDIVIDUALS")
plot.data <- test.data
pop.levels <- c("W-ATL","W-IO","N-IO","E-IO/W-PAC","Fiji")
plot.data$pop <- factor(plot.data$pop, levels = pop.levels)

aic.plot <- ggplot2::ggplot() +
```

```
  ggplot2::geom_histogram(data = subset(plot.data, sex == "F"),
                          ggplot2::aes(aic, fill = "F", y = ..count..),
                          binwidth = 7) +
  ggplot2::geom_histogram(data = subset(plot.data, sex == "M"),
                          ggplot2::aes(aic, fill = "M", y = -..count..),
                          binwidth = 7) +
  ggplot2::geom_hline(yintercept = 0) +
  ggplot2::scale_fill_manual(values = c("#E69F00", "#56B4E9"),
                             labels = c("Female", "Male")) +
  ggplot2::labs(x = "Corrected assignment index",
                y = "Number of individuals",
                fill = "") +
  ggplot2::facet_grid(pop~., scales = "free_y") +
  ggplot2::theme_minimal() +
  ggplot2::theme(
    axis.text.x = ggplot2::element_text(size = 10, angle = 0, face = "bold"),
    axis.text.y = ggplot2::element_text(size = 10, angle = 0, face = "bold"),
    axis.title = ggplot2::element_text(size = 15),
    legend.text = ggplot2::element_text(hjust = 0, size = 15),
    legend.title = ggplot2::element_text(hjust = 0, size = 15),
    strip.text.y = element_text(angle = 0,size = 10)
  )
print(aic.plot)
```

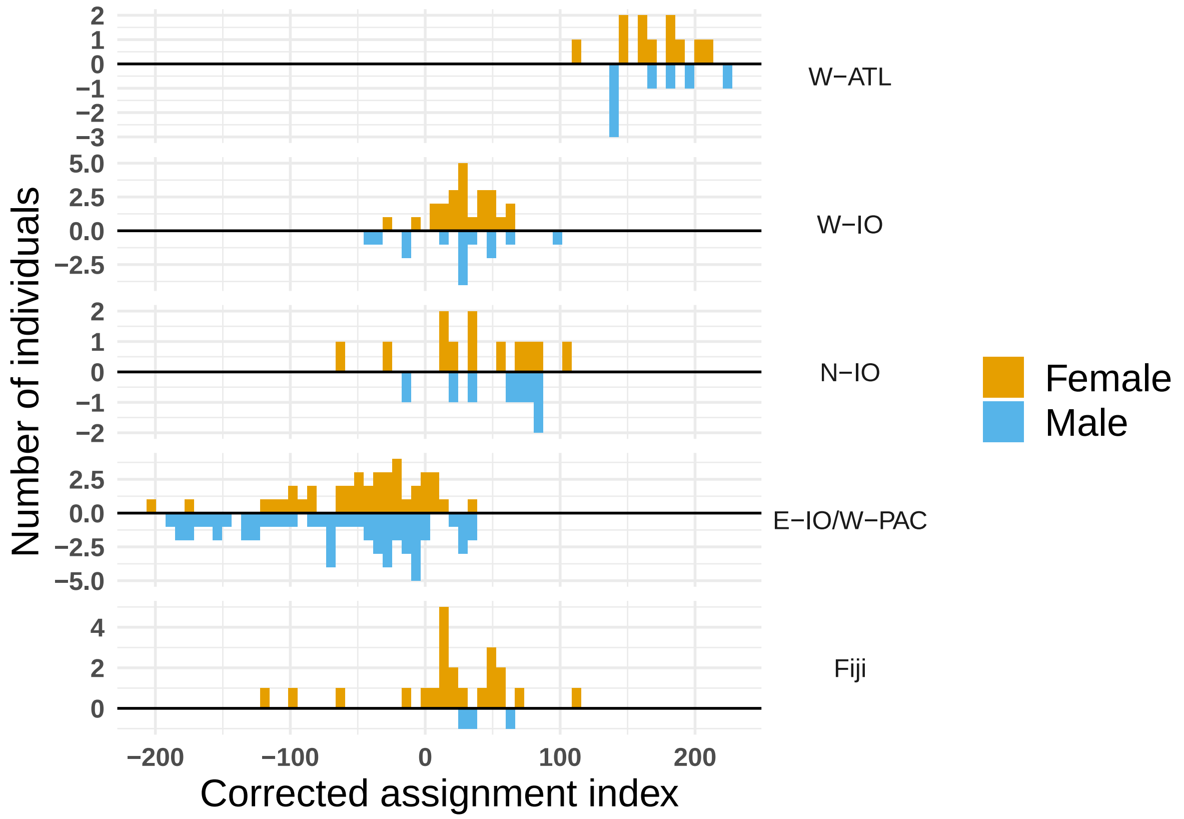


```
ggplot2::ggsave(plot = aic.plot, filename = "DArTcap_ALL_Aic_sex_Regions_OnlyAdults.png",
       width = 30, height = 15, units = "cm")

res <- plot.data[(plot.data$pop == "N-IO" & plot.data$aic < (-50) &
                    !is.na(plot.data$aic)) |
                 (plot.data$pop == "E-IO/W-PAC" & plot.data$aic < (-200) &
                    !is.na(plot.data$aic)) |
```

```
                 (plot.data$pop == "Fiji" & plot.data$aic < (-50) &
                    !is.na(plot.data$aic)), ]
knitr::kable(res, digits = 4,
             caption = "Individuals with low population assignment indices") %>%
  kableExtra::kable_styling(full_width = FALSE,font_size = 9,
                            latex_options = c( "hold_position")
)


dat <- plot.data$aic[plot.data$sex=="F" & plot.data$pop=="Fiji"]
quantile(dat, 0.025)
boxplot(dat,
  ylab = "Aic",
  main = "Boxplot of AIc"
)
```

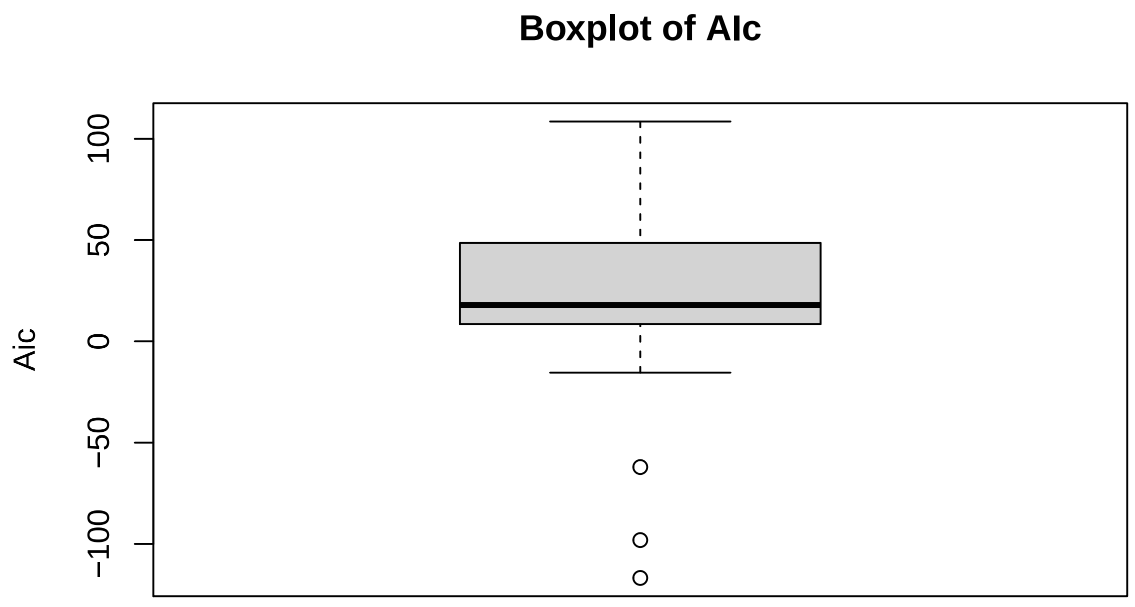


```
out <- boxplot.stats(dat)$out
```

```
## $call
## hierfstat::sexbias.test(dat = dat, sex = sex, nperm = 100, test = "FST",
##     alternative = "two.sided")
##
## $statistic
## [1] -0.02210322
##
## $p.value
## [1] 1
##
## $call
## hierfstat::sexbias.test(dat = dat, sex = sex, nperm = 100, test = "FIS",
##     alternative = "two.sided")
```

```
##
## $statistic
## [1] 0.01046105
##
## $p.value
## [1] 0.17
##
## $call
## hierfstat::sexbias.test(dat = dat, sex = sex, nperm = 100, test = "mAIc",
##     alternative = "two.sided")
##
## $statistic
## [1] 2.432244
##
## $p.value
## [1] 0.58
##
## $call
## hierfstat::sexbias.test(dat = dat, sex = sex, nperm = 100, test = "vAIc",
##     alternative = "two.sided")
##
## $statistic
## [1] 0.6944592
##
## $p.value
## [1] 0.05
```

Table 31: Individuals with low population assignment indices

| | INDIVIDUALS | sex | aic | pop |
|---|---|---|---|---|
| 60 | CL-ARS012-F | F | -64.7406 | N-IO |
| 149 | CLSH047-F | F | -204.4526 | E-IO/W-PAC |
| 175 | CL-FIJ005-F.1 | F | -116.7981 | Fiji |
| 188 | CL-FIJ021-F | F | -62.0468 | Fiji |
| 189 | CL-FIJ022-F | F | -98.1071 | Fiji |

```
##      2.5%
## -106.9853
```

```
strata <- BS.dartcap.gt@schemes$Region
strata[strata %in% c("E-IO","W-PAC")] <- "E-IO/W-PAC"
names(strata) <- BS.dartcap.gt@schemes$INDIVIDUALS
strataG::setStrata(BS.dartcap.gt) <- strata
BS.dartcap.gt@schemes$`TL (mm)` <- as.integer(BS.dartcap.gt@schemes$`TL (mm)`)
gt.all.F <- BS.dartcap.gt[BS.dartcap.gt@schemes$COMBINED_SEX == "F" &
                                     (BS.dartcap.gt@schemes$`TL (mm)` > 1500 &
                                     !is.na(BS.dartcap.gt@schemes$`TL (mm)`)|
                                       BS.dartcap.gt@schemes$Site == "Fiji"),]
gt.all.M <- BS.dartcap.gt[BS.dartcap.gt@schemes$COMBINED_SEX == "M" &
                                     BS.dartcap.gt@schemes$ `TL (mm)` > 1500 &
                                     (!is.na(BS.dartcap.gt@schemes$`TL (mm)`)|
                                        BS.dartcap.gt@schemes$Site == "Fiji"),]

Fst.all.region.F <-
  strataG::popStructTest(
    gt.all.F,
    nrep = 1000,
```

```
    stats = c("fst", "fis"),
    type = "pairwise",
    keep.null = FALSE,
    quietly = TRUE,
    max.cores = parallel::detectCores() - 1,
    write.output = FALSE
  )
save(Fst.all.region.F, file = "DArTcap_ALL_Fst_sex_Region.Rdata")
Fst.all.region.M <-
  strataG::popStructTest(
    gt.all.M,
    nrep = 1000,
    stats = c("fst", "fis"),
    type = "pairwise",
    keep.null = FALSE,
    quietly = TRUE,
    max.cores = parallel::detectCores() - 1,
    write.output = FALSE
  )
save(Fst.all.region.F, Fst.all.region.M,
     file = "DArTcap_ALL_Fst_sex_Region.Rdata")
```

```
load("DArTcap_ALL_Fst_sex_Region.Rdata")
dim(Fst.all.region.F$pairwise$result) #91  8
dim(Fst.all.region.M$pairwise$result) #66  8

names.1 <- paste0(Fst.all.region.F$pairwise$result$strata.1,"_",
                  Fst.all.region.F$pairwise$result$strata.2)
names.2 <- paste0(Fst.all.region.M$pairwise$result$strata.1,"_",
                  Fst.all.region.M$pairwise$result$strata.2)

result.1 <- Fst.all.region.F$pairwise$result
result.1$names.comb <- names.1
result.2 <- Fst.all.region.M$pairwise$result
result.2$names.comb <- names.2

data <- left_join(result.1,result.2, by = "names.comb" )

pwFst.FM <- data[,c(1:6, 12:15)]
colnames(pwFst.FM) <- c("strata.1", "strata.2", "n.auDNA.F.1", "n.auDNA.F.2",
                        "Fst.auDNA.F", "Fst.auDNA.F.Pval",
                     "n.auDNA.M.1", "n.auDNA.M.2", "Fst.auDNA.M", "Fst.auDNA.M.Pval")
knitr::kable(pwFst.FM, digits = 4,
             caption = "Pairwise Fst between females and males for autosomal markers") %>%
  kableExtra::kable_styling(full_width = FALSE,font_size = 5,
                            latex_options = c( "hold_position")) %>%
  kableExtra::landscape()
```

#### 8.11.3.2 Fst test

```
## [1] 21  8
## [1] 21  8
```

Table 32: Pairwise Fst between females and males for autosomal markers

| strata.1 | strata.2 | n.auDNA.F.1 | n.auDNA.F.2 | Fst.auDNA.F | Fst.auDNA.F.Pval | n.auDNA.M.1 | n.auDNA.M.2 | Fst.auDNA.M | Fst.auDNA.M.Pval |
|---|---|---|---|---|---|---|---|---|---|
| E-IO/W-PAC | E-PAC | 165 | 6 | NA | NA | 176 | 10 | NA | NA |
| E-IO/W-PAC | Fiji | 165 | 11 | 0.0288 | 0.0010 | 176 | 7 | 0.0306 | 0.0010 |
| E-IO/W-PAC | Japan | 165 | 2 | NA | NA | 176 | 2 | NA | NA |
| E-IO/W-PAC | N-IO | 165 | 19 | 0.0005 | 0.2517 | 176 | 8 | 0.0020 | 0.1279 |
| E-IO/W-PAC | W-ATL | 165 | 41 | 0.6392 | 0.0010 | 176 | 21 | 0.6365 | 0.0010 |
| E-IO/W-PAC | W-IO | 165 | 31 | 0.0018 | 0.0010 | 176 | 13 | 0.0006 | 0.2807 |
| E-PAC | Fiji | 6 | 11 | NA | NA | 10 | 7 | 0.6720 | 0.0010 |
| E-PAC | Japan | 6 | 2 | 0.7197 | 0.0796 | 10 | 2 | NA | NA |
| E-PAC | N-IO | 6 | 19 | NA | NA | 10 | 8 | NA | NA |
| E-PAC | W-ATL | 6 | 41 | NA | NA | 10 | 21 | NA | NA |
| E-PAC | W-IO | 6 | 31 | NA | NA | 10 | 13 | NA | NA |
| Fiji | Japan | 11 | 2 | NA | NA | 7 | 2 | NA | NA |
| Fiji | N-IO | 11 | 19 | 0.0267 | 0.0010 | 7 | 8 | 0.0269 | 0.0010 |
| Fiji | W-ATL | 11 | 41 | 0.6604 | 0.0010 | 7 | 21 | 0.6535 | 0.0010 |
| Fiji | W-IO | 11 | 31 | 0.0276 | 0.0010 | 7 | 13 | 0.0258 | 0.0010 |
| Japan | N-IO | 2 | 19 | NA | NA | 2 | 8 | 0.0447 | 0.0320 |
| Japan | W-ATL | 2 | 41 | 0.6704 | 0.0492 | 2 | 21 | NA | NA |
| Japan | W-IO | 2 | 31 | NA | NA | 2 | 13 | 0.0363 | 0.0177 |
| N-IO | W-ATL | 19 | 41 | 0.6538 | 0.0010 | 8 | 21 | 0.6540 | 0.0010 |
| N-IO | W-IO | 19 | 31 | 0.0007 | 0.2827 | 8 | 13 | 0.0011 | 0.3407 |
| W-ATL | W-IO | 41 | 31 | 0.6499 | 0.0010 | 21 | 13 | 0.6549 | 0.0010 |

```r
pwFis.FM <- data[,c(1:4,7:8, 12:13,16:17)]
colnames(pwFis.FM) <- c("strata.1", "strata.2", "n.auDNA.F.1", "n.auDNA.F.2",
                        "Fis.auDNA.F", "Fis.auDNA.F.Pval",
                     "n.auDNA.M.1", "n.auDNA.M.2", "Fis.auDNA.M", "Fis.auDNA.M.Pval")
knitr::kable(pwFis.FM, digits = 4,
             caption = "Pairwise Fis between females and males for autosomal markers") %>%
  kableExtra::kable_styling(full_width = FALSE,font_size = 5,
                            latex_options = c( "hold_position")) %>%
  kableExtra::landscape()
```

#### 8.11.3.3 Fis test

Table 33: Pairwise Fis between females and males for autosomal markers

| strata.1 | strata.2 | n.auDNA.F.1 | n.auDNA.F.2 | Fis.auDNA.F | Fis.auDNA.F.Pval | n.auDNA.M.1 | n.auDNA.M.2 | Fis.auDNA.M | Fis.auDNA.M.Pval |
|---|---|---|---|---|---|---|---|---|---|
| E-IO/W-PAC | E-PAC | 165 | 6 | NA | NA | 176 | 10 | NA | NA |
| E-IO/W-PAC | Fiji | 165 | 11 | -4.3618 | 0.9411 | 176 | 7 | -4.3394 | 0.5145 |
| E-IO/W-PAC | Japan | 165 | 2 | NA | NA | 176 | 2 | NA | NA |
| E-IO/W-PAC | N-IO | 165 | 19 | -3.9486 | 0.5804 | 176 | 8 | -4.4266 | 0.1439 |
| E-IO/W-PAC | W-ATL | 165 | 41 | -1.8571 | 1.0000 | 176 | 21 | -2.2058 | 1.0000 |
| E-IO/W-PAC | W-IO | 165 | 31 | -3.8246 | 0.4925 | 176 | 13 | -4.2056 | 0.8771 |
| E-PAC | Fiji | 6 | 11 | NA | NA | 10 | 7 | NA | NA |
| E-PAC | Japan | 6 | 2 | NA | NA | 10 | 2 | NA | NA |
| E-PAC | N-IO | 6 | 19 | NA | NA | 10 | 8 | NA | NA |
| E-PAC | W-ATL | 6 | 41 | NA | NA | 10 | 21 | NA | NA |
| E-PAC | W-IO | 6 | 31 | NA | NA | 10 | 13 | NA | NA |
| Fiji | Japan | 11 | 2 | NA | NA | 7 | 2 | NA | NA |
| Fiji | N-IO | 11 | 19 | -13.4680 | 1.0000 | 7 | 8 | -59.0989 | 1.0000 |
| Fiji | W-ATL | 11 | 41 | -9.5707 | 1.0000 | 7 | 21 | -18.4592 | 1.0000 |
| Fiji | W-IO | 11 | 31 | -11.5127 | 1.0000 | 7 | 13 | -30.2224 | 0.9910 |
| Japan | N-IO | 2 | 19 | NA | NA | 2 | 8 | NA | NA |
| Japan | W-ATL | 2 | 41 | NA | NA | 2 | 21 | NA | NA |
| Japan | W-IO | 2 | 31 | NA | NA | 2 | 13 | NA | NA |
| N-IO | W-ATL | 19 | 41 | -6.4767 | 1.0000 | 8 | 21 | -13.9027 | 1.0000 |
| N-IO | W-IO | 19 | 31 | -9.0011 | 0.4845 | 8 | 13 | -22.6698 | 0.0420 |
| W-ATL | W-IO | 41 | 31 | -5.5483 | 1.0000 | 21 | 13 | -10.8381 | 1.0000 |

```r
gl <- BS.dartcap.gl
pop.levels <- c("E-PAC","W-ATL","E-ATL","W-IO","N-IO","E-IO","W-PAC",
                "E-IO/W-PAC", "Japan","Fiji")
gl$pop <- factor(gl$other$ind.metrics$Region, levels = pop.levels)
gl$pop[gl$pop %in% c("E-IO","W-PAC")] <- "E-IO/W-PAC"
pop.levels <- c("E-PAC","W-ATL","E-ATL","W-IO","N-IO","E-IO/W-PAC",
                "Japan","Fiji")
gl$pop <- factor(gl$pop , levels = pop.levels)
gl <- gl[order(gl$pop, gl$ind.names),]

gl$other$ind.metrics$`TL (mm)` <- as.integer(gl$other$ind.metrics$`TL (mm)`)
gl <- gl[(gl$other$ind.metrics$`TL (mm)` > 1500 &
            !is.na(gl$other$ind.metrics$`TL (mm)`)) | gl$pop == "Fiji",] #Because all Fiji samples w
gl <- gl[!gl$other$ind.metrics$Site == "E-ATL",] #only 1 sample
gl$other$loc.metrics.flags$monomorphs <- FALSE
gl$other$loc.metrics.flags$CallRate  <- FALSE


gl2rel <- function(gl, filename = "relate"){
  group.name <- paste(gl$other$ind.metrics$COMBINED_SEX,
                      gl$other$ind.metrics$COMBINED_SEX, "_",
                      gl$ind.names, sep = "")
  glmat <- as.matrix(gl)
  glmat[glmat==0] <- '2 2'
  glmat[glmat==1] <- '1 2'
  glmat[glmat==2] <- '1 1'
  glmat[is.na(glmat)] <- '0 0'
  df <- cbind(group.name, glmat)
  write.table(df, file = paste0(filename,".txt", sep = ""), quote = FALSE,
              sep = " ", row.names = FALSE, col.names = FALSE)
}


res.rel <- list()
summ.rel <- data.frame(sex.group = c("FF", "FM", "MM"))
plot.data <- data.frame(pair.no = NA,ind1.id = NA,ind2.id = NA, group = NA,
                        condition = NA, measurement = NA,Site = NA)
for (i in 1:nlevels(gl$pop)) {
  print(i)
  gl.tmp <- gl[gl$pop == levels(gl$pop)[i],]
  gl.tmp <- dartR::gl.filter.callrate(gl.tmp, method = 'loc', threshold = 1,
                                      mono.rm = TRUE, plot = FALSE, verbose = 0)

  gl2rel(gl.tmp, filename = "relate")
  data <- related::readgenotypedata("relate.txt")
  outfile <- related::coancestry (data$gdata , lynchli = 1, lynchrd = 1,
                                  quellergt = 1, wang = 1, ritland = 1)
  res <- outfile$relatedness[!outfile$relatedness$group %in%
                               c("FFUU","UUUU","MMUU","UUFF", "UUMM"),]
  res$group[res$group == "MMFF"] <- "FFMM"
  data_long <- gather(res, condition, measurement, 5:11, factor_key=TRUE)
  data_long$Site <- levels(gl$pop)[i]
  plot.data <- rbind(plot.data, data_long)
  summ <- dplyr::group_by(res, group) %>%
    dplyr::summarise(mean(ritland))
  colnames(summ) <- c("sex.group", levels(gl$pop)[i])
  summ.rel <- cbind(summ.rel, summ[,2])
```

```
  res.rel[[i]] <- res
}
plot.data <- plot.data[-1,]
save(res.rel,summ.rel,plot.data,
     file = "DArTcap_ALL_relatedness_sex_Region.Rdata")
```

```
load("DArTcap_ALL_relatedness_sex_Region.Rdata")
pop.levels <- c("E-PAC","W-ATL","W-IO","N-IO","E-IO/W-PAC", "Japan","Fiji")
plot.data$Site <- factor(plot.data$Site, levels = pop.levels)
plot.data <- plot.data[plot.data$condition == "ritland",]
plot <- ggplot2::ggplot(plot.data, ggplot2::aes(x = group, y = measurement,
                                                 fill = group)) +
  ggplot2::geom_boxplot() +
  ggplot2::facet_wrap(Site~.) +
  ggplot2::labs(x = "Site", y = "Relatedness (Ritland 1996)")
print(plot)
```

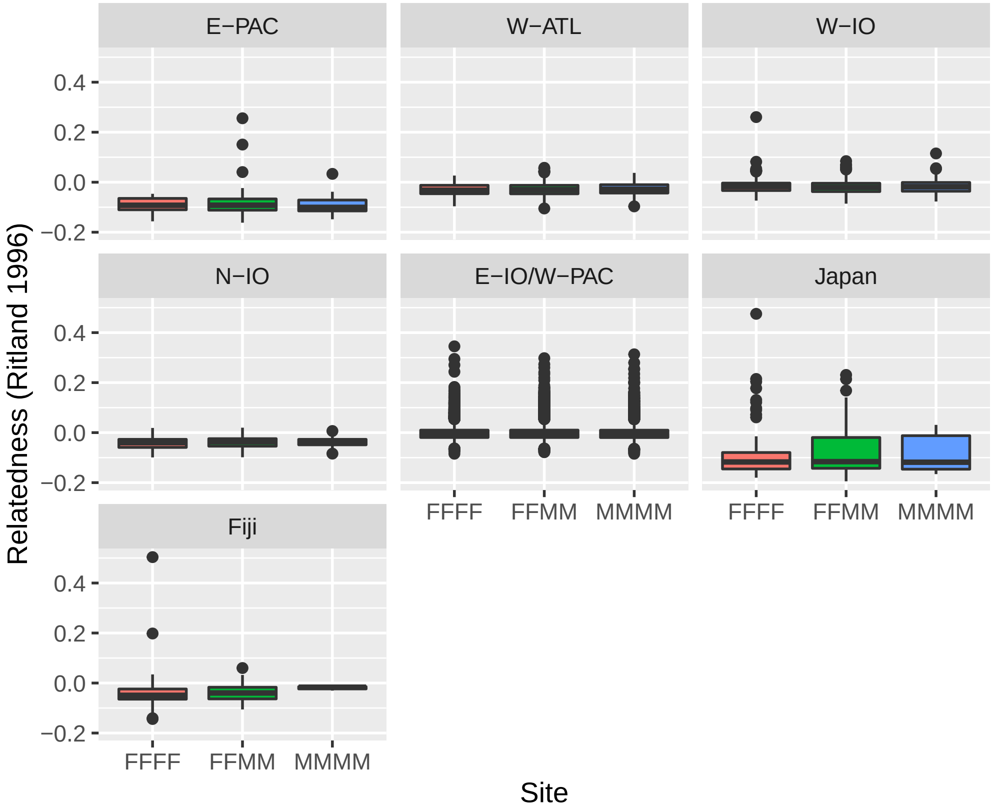


#### 8.11.3.4 Relatedness test

```
knitr::kable(summ.rel, digits = 2,
             caption = "Relatedness between female and males according to Ritland (1996)") %>%
  kableExtra::kable_styling(full_width = FALSE,font_size = 5,
                            latex_options = c( "hold_position"))
```

Table 34: Relatedness between female and males according to Ritland (1996)

| sex.group | E-PAC | W-ATL | W-IO | N-IO | E-IO/W-PAC | Japan | Fiji |
|---|---|---|---|---|---|---|---|
| FF | -0.09 | -0.03 | -0.02 | -0.04 | 0 | -0.07 | -0.04 |
| FM | -0.08 | -0.03 | -0.02 | -0.04 | 0 | -0.06 | -0.04 |
| MM | -0.09 | -0.03 | -0.02 | -0.04 | 0 | -0.08 | -0.02 |

# 9 DATA3: DArTcap - All data, subset Australia

## 9.1 Set strata

```
strata <- radiator::read_strata("Bull_shark_DArTcap_strata.tsv",
                                keep.two = FALSE)$strata %>%
  dplyr:: filter(SpeciesID_genetic == "Carcharhinus leucas")
id.sub <- c(strata$INDIVIDUALS[strata$Site != "Australia"],
            sample(strata$INDIVIDUALS[strata$Site == "Australia"], size = 60))
strata <- strata[strata$INDIVIDUALS %in% id.sub,]
readr::write_tsv(strata, path = "Bull_shark_DArTcap_strata3_AUS_subset.tsv")
```

## 9.2 Filter data

1. Marker Reproducibility: 0.98
2. Individual missingness: 0.35
3. Individual Het - low: 0
4. Individual Het - high: 0.08
5. Individual total coverage - low: 10,000
6. Individual total coverage - high: 1,000,000
7. Marker MAC - low: 4
8. Marker coverage - low: 5
9. Marker coverage - high:90
10. Marker maximum missing proportion: 0.1
11. Marker snp position on locus: n
12. Max 3 SNPs per locus
13. 1 SNP per locus according to mac
14. no LD filter
15. Individual Het - low: 0.037
16. Individual Het - high: 0.1165
17. Duplicated individuals: 0.1
18. Filter on HWE: 2 pops, 0.001

```
dartcap.data <- "SEQ_SNPs_counts_0_Target_merge.csv"
strata <- "Bull_shark_DArTcap_strata3_AUS_subset.tsv"
BSsnp <- radiator::filter_rad(dartcap.data, strata,
                              parallel.core = 1)
BS.dartcap.sub.gds <- BSsnp$gds
BS.dartcap.sub.tidy <- BSsnp$output$tidy.data
print(BS.dartcap.sub.gds, expand = TRUE, all = TRUE, attribute = TRUE,
      attribute.trim = FALSE)

save(BS.dartcap.sub.tidy, file = "DArTcap_ALL_AUS_subset.Rdata")
```

Table 35:

| FILTERS | VALUES | BEFORE | BLACKLIST |
|---|---|---|---|
| Filter DArT reproducibility | 0.98 | 465 / 30 / 1 / 26335 / 37537 | 0 / 0 / 0 / 1043 / 2250 |
| Filter monomorphic markers | NA | 465 / 30 / 1 / 25292 / 35287 | 0 / 0 / 0 / 12443 / 20404 |
| Filter markers in common | NA | 465 / 30 / 1 / 12849 / 14883 | 0 / 0 / 0 / 5477 / 6377 |
| Filter individuals based on missingness | 0.35 | 465 / 30 / 1 / 7372 / 8506 | 10 / 0 / 0 / 0 / 0 |
| Filter individuals based on heterozygosity | 0 0.05 | 455 / 30 / 1 / 7372 / 8506 | 14 / 0 / 0 / 0 / 0 |
| Filter individuals based on total coverage | 10000 1e+06 | 441 / 30 / 1 / 7372 / 8506 | 2 / 0 / 0 / 0 / 0 |
| Filter monomorphic markers | NA | 439 / 30 / 1 / 7372 / 8506 | 0 / 0 / 0 / 1571 / 2036 |
| Filter MAC | 4 | 439 / 30 / 1 / 5801 / 6470 | 0 / 0 / 0 / 3211 / 3699 |
| Filter coverage min / max | 5-90 | 439 / 30 / 1 / 2590 / 2771 | 0 / 0 / 0 / 611 / 633 |
| Filter genotyping | 0.1 | 439 / 30 / 1 / 1979 / 2138 | 0 / 0 / 0 / 94 / 107 |
| Filter SNPs position on the read | all | 439 / 30 / 1 / 1885 / 2031 | 0 / 0 / 0 / 0 / 0 |
| Filter markers snp number | 3 | 439 / 30 / 1 / 1885 / 2031 | 0 / 0 / 0 / 0 / 0 |
| Filter short ld | mac | 439 / 30 / 1 / 1885 / 2031 | 0 / 0 / 0 / 0 / 146 |
| detect mixed genomes | 0.037 0.1165 | 439 / 30 / 1 / 1885 / 1885 | 11 / 0 / 0 / 0 / 0 |
| Filter monomorphic markers | NA | 428 / 30 / 1 / 1885 / 1885 | 0 / 0 / 0 / 0 / 0 |
| detect duplicate genomes | 0.1 | 428 / 30 / 1 / 1885 / 1885 | 40 / 0 / 0 / 0 / 0 |
| Filter monomorphic markers | NA | 388 / 30 / 1 / 1885 / 1885 | 0 / 0 / 0 / 0 / 0 |
| Filter HWE | 20.001 | 388 / 30 / 1 / 1885 / 1885 | 0 / 0 / 0 / 7 / 7 |

### 9.2.1 Remove extra low and high heterozygous individuals

Because the average heterozygosity per sampling location is not even, certain individuals cannot be excluded without removing 'good' individuals.

```
rm <-  c("CL-ARS021-F","CL-GOM025-F.1","CL-GOM012-F","CL-SAF014-M","CL-SAF002-F",
         "CL-BRZ038-U","CLF003-F","CL-GOM011-F","CL-SAF015-F","CL-SAF003-M",
         "CL-PNG005-U","CLCR056-M","CLCR047.1","CLCR055-M","CLCR066-M")

BS.dartcap.sub.tidy <- BS.dartcap.sub.tidy[!BS.dartcap.sub.tidy$INDIVIDUALS %in% rm,]
length(unique(BS.dartcap.sub.tidy$INDIVIDUALS)) # 382
```

‘

## 9.3 Save data

```
Convert <- radiator::genomic_converter(BS.dartcap.sub.tidy,
                                       output = c("genlight", "stockr",
                                                  "pcadapt", "gtypes", "rubias"))
BS.dartcap.sub.gl <- Convert$genlight
BS.dartcap.sub.stkr <- Convert$stockr
BS.dartcap.sub.pc <- Convert$pcadapt
BS.dartcap.sub.gt <- Convert$gtypes
BS.dartcap.sub.rubias <- Convert$rubias

markers.meta <- radiator::extract_markers_metadata(BS.dartcap.sub.gds,
                                                   whitelist = TRUE)
individuals.meta <- radiator::extract_individuals_metadata(BS.dartcap.sub.gds,
                                                           whitelist = TRUE)
individuals.meta <- individuals.meta[individuals.meta$INDIVIDUALS %in%
                                       BS.dartcap.sub.gl$ind.names,]
BS.dartcap.sub.gl$other$ind.metrics <- individuals.meta
BS.dartcap.sub.gl$other$loc.metrics <- markers.meta
BS.dartcap.sub.gt@schemes <- individuals.meta

pop.levels <- c("Costa Rica" , "Brazil","Caribbean Sea","Gulf of Mexico",
                "Western North Atlantic", "Sierra Leone", "South Africa",
                "Mozambique", "Reunion" , "Seychelles", "Arabian Sea",
                "Sri Lanka", "Thailand", "Indonesia", "Papua New Guinea" ,
                "Fitzroy River", "Daly River", "Adelaide River","Darwin Coastal",
                "South Alligator River", "East Alligator River","Blue mud Bay",
                "Roper River","Towns River","Unknown","Wenlock River","Trinity inlet",
                "Clarence River", "Sydney Harbour", "Okinawa", "Urauchi River",
                "Fiji")
BS.dartcap.sub.gl$pop <- factor(BS.dartcap.sub.gl$other$ind.metrics$Site2,
                                levels = pop.levels)
BS.dartcap.sub.gl <- BS.dartcap.sub.gl[order(BS.dartcap.sub.gl$pop,
                                             BS.dartcap.sub.gl$ind.names),]
BS.dartcap.sub.gl <- BS.dartcap.sub.gl[order(BS.dartcap.sub.gl$pop,
                                             BS.dartcap.gl$ind.names)]
BS.dartcap.sub.rubias$repunit <- factor(BS.dartcap.sub.rubias$repunit,
                                        levels = pop.levels)

save(BS.dartcap.sub.tidy, BS.dartcap.sub.gl, BS.dartcap.sub.stkr, BS.dartcap.sub.pc,
     BS.dartcap.sub.gt,BS.dartcap.sub.rubias,
     markers.meta, individuals.meta, file = "DArTcap_ALL_AUS_subset.Rdata")
```

## 9.4 Load data

```r
load("DArTcap_ALL_AUS_subset.Rdata")

pop.levels <- c("Costa Rica" , "Brazil","Caribbean Sea", "Gulf of Mexico",
                "Western North Atlantic", "Sierra Leone", "South Africa",
                "Mozambique", "Reunion" , "Seychelles", "Arabian Sea", "Sri Lanka",
                "Thailand", "Indonesia", "Papua New Guinea" , "Australia",
                "Japan", "Fiji")

BS.dartcap.sub.gl$pop <- factor(BS.dartcap.sub.gl$other$ind.metrics$Site,
                                levels = pop.levels)
BS.dartcap.sub.gl <- BS.dartcap.sub.gl[order(BS.dartcap.sub.gl$pop,
                                             BS.dartcap.sub.gl$ind.names)]

unique(BS.dartcap.sub.tidy$POP_ID)
AUS <- c( "Daly_River","Wenlock_River","Sydney_Harbour","Clarence_River",
          "Adelaide_River","South_Alligator_River","Trinity_inlet",
          "East_Alligator_River","Towns_River","Darwin_Coastal","Unknown")
JAP <- c( "Urauchi_River", "Okinawa")
POP_ID <- as.character(BS.dartcap.sub.tidy$POP_ID)
POP_ID[POP_ID %in% AUS] <- "Australia"
POP_ID[POP_ID %in% JAP] <- "Japan"
pop.levels1 <- stringr::str_replace_all(pop.levels, " ", "_")
BS.dartcap.sub.tidy$POP_ID <- factor(POP_ID, levels = pop.levels1)
BS.dartcap.sub.tidy <- BS.dartcap.sub.tidy[order(BS.dartcap.sub.tidy$POP_ID,
                                                 BS.dartcap.sub.tidy$INDIVIDUALS),]

adegenet::nInd(BS.dartcap.sub.gl) # 382
adegenet::nLoc(BS.dartcap.sub.gl) # 1878
summary(BS.dartcap.sub.gl$pop)
sum(duplicated(BS.dartcap.sub.gl$other$ind.metrics$`Genetic code`))# 0 replicate left
sum(baits$CloneID %in% BS.dartcap.sub.gl$other$loc.metrics$LOCUS) #88
```

```
##  [1] Fiji                   Arabian_Sea           Sri_Lanka
##  [4] Western_North_Atlantic Costa_Rica            Thailand
##  [7] Mozambique             Gulf_of_Mexico        Reunion
## [10] Urauchi_River          South_Africa          Papua_New_Guinea
## [13] Sierra_Leone           Okinawa               Brazil
## [16] Seychelles             Caribbean_Sea         Indonesia
## [19] Daly_River             Wenlock_River         Sydney_Harbour
## [22] Clarence_River         Adelaide_River        South_Alligator_River
## [25] Trinity_inlet          East_Alligator_River  Towns_River
## [28] Darwin_Coastal         Unknown
## 30 Levels: Fiji Arabian_Sea Sri_Lanka Western_North_Atlantic ... Unknown
## [1] 382
## [1] 1878
##             Costa Rica                 Brazil          Caribbean Sea
##                     15                     52                      2
##         Gulf of Mexico Western North Atlantic           Sierra Leone
##                     40                      8                      1
##           South Africa             Mozambique                Reunion
##                     23                     11                     28
##             Seychelles            Arabian Sea              Sri Lanka
##                     36                     15                     12
##               Thailand              Indonesia       Papua New Guinea
##                      5                      4                      8
##              Australia                  Japan                   Fiji
```

```
##                       54                      44                      24
## [1] 0
## [1] 88
```

## 9.5 Remove sex-linked markers

```
load("Sex-linked_marker_DArTcap.Rdata")
load("Sex_results.Rdata")
sum(bull_shark$sexy.summary$CLONE_ID %in% SLM$sexy.summary$CLONE_ID) #33
sum(SLM$sexy.summary$CLONE_ID %in% bull_shark$sexy.summary$CLONE_ID) #23
sex.markers <- unique(c(bull_shark$sexy.summary$CLONE_ID,
                        SLM$sexy.summary$CLONE_ID))

sum(BS.dartcap.sub.gl$loc.names %in% sex.markers) #29
locnames <- BS.dartcap.sub.gl$loc.names
BS.dartcap.sub.gl <- BS.dartcap.sub.gl[,!locnames %in% sex.markers]
BS.dartcap.sub.gl$other$loc.metrics <- BS.dartcap.sub.gl$other$loc.metrics[
  !locnames %in% sex.markers,]

sum(sex.markers %in% unique(BS.dartcap.sub.tidy$LOCUS)) # 29
BS.dartcap.sub.tidy <- BS.dartcap.sub.tidy[!BS.dartcap.sub.tidy$LOCUS %in%
                                             sex.markers,]

locus.gt <- stringr::str_match(pattern = "__\\s*(.*?)\\s*__",
                               string =  BS.dartcap.sub.gt@data$locus)[,2]
BS.dartcap.sub.gt <- BS.dartcap.sub.gt[,!locus.gt %in% sex.markers, ,
                                       drop = TRUE]

locus.rubias <- stringr::str_match(pattern = "__\\s*(.*?)\\s*__",
                                   string =  colnames(BS.dartcap.sub.rubias))[,2]
BS.dartcap.sub.rubias <- BS.dartcap.sub.rubias[,!locus.rubias %in% sex.markers]
```

```
## [1] 33
## [1] 23
## [1] 29
## [1] 29
```

## 9.6 Genetic diversity

```
set.seed(124)
pop.levels <- c("Costa Rica" , "Brazil","Caribbean Sea","Gulf of Mexico",
                "Western North Atlantic", "South Africa", "Mozambique", "Reunion" ,
                "Seychelles", "Arabian Sea", "Sri Lanka", "Thailand", "Indonesia",
                "Papua New Guinea" , "Australia", "Japan", "Fiji")

gl <- BS.dartcap.sub.gl
gl <- gl[gl$pop != "Sierra Leone"] #only 1 sample
gl$pop <- factor(gl$other$ind.metrics$Site, levels = pop.levels)
gl <- gl[order(gl$pop,gl$ind.names)]

gl2gpop(gl, filename = "BS_dartcap_aus_subset_genepop.txt")
bastat <-diveRsity::basicStats(infile = "BS_dartcap_aus_subset_genepop.txt",
                               outfile = "BS_dartcap_aus_subset_BasicStat_output.txt",
                               fis_ci = TRUE,ar_ci = TRUE, fis_boots = 1000,
                               ar_boots = 1000,mc_reps = 1000,
                               rarefaction = FALSE, ar_alpha = 0.05,
                               fis_alpha = 0.05)
length(bastat$main_tab) #17
```

```r
gendiv <- data.frame(
  "Costa Rica" = bastat$main_tab[[1]]$overall,
  "Brazil" = bastat$main_tab[[2]]$overall,
  "Caribbean Sea" = bastat$main_tab[[3]]$overall,
  "Gulf of Mexico" = bastat$main_tab[[4]]$overall,
  "Western North Atlantic" = bastat$main_tab[[5]]$overall,
  "South Africa" = bastat$main_tab[[6]]$overall,
  "Mozambique" = bastat$main_tab[[7]]$overall,
  "Reunion" = bastat$main_tab[[8]]$overall,
  "Seychelles" = bastat$main_tab[[9]]$overall,
  "Arabian Sea" = bastat$main_tab[[10]]$overall,
  "Sri Lanka" = bastat$main_tab[[11]]$overall,
  "Thailand" = bastat$main_tab[[12]]$overall,
  "Indonesia" = bastat$main_tab[[13]]$overall,
  "Papua New Guinea" = bastat$main_tab[[14]]$overall,
  "Australia" = bastat$main_tab[[15]]$overall,
  "Japan" = bastat$main_tab[[16]]$overall,
  "Fiji" = bastat$main_tab[[17]]$overall,
  check.names = FALSE)
row.names(gendiv) <- rownames(bastat$main_tab[[1]])

df <- as.data.frame(gl)
df[df == "0"] <- "11"
df[df == "1"] <- "12"
df[df == "2"] <- "22"
gen.gi <- adegenet::df2genind(df, ploidy = 2,ncode = 2, pop = gl$pop)
ris_basic <- hierfstat::basic.stats(gen.gi)
fis.hierfstat <- apply(ris_basic$Fis, 2, mean, na.rm=T)
fis.CI <- hierfstat::boot.ppfis(gen.gi, nboot = 1000, quant = c(0.025,0.975),
                                diploid = TRUE,dig = 4)
df <- data.frame(fis.hierfstat,fis.CI.low = fis.CI$fis.ci[,1],
                 fis.CI.high = fis.CI$fis.ci[,2])
df <- as.data.frame(t(df))
gendiv <- rbind(gendiv, df)

readr::write_tsv(gendiv, path = "DArTcap_aus_subset_gendiv.txt")
save(gendiv, file = "DArTcap_aus_subset_gendiv.Rdata")
```

```r
load("DArTcap_aus_subset_gendiv.Rdata")
shortnames <- c("COR","BRZ", "CAR", "GOM", "WNA", "SAF", "MOZ", "RUN" ,
                "SEY", "ARS", "SRL", "TAI", "IND", "PNG" , "AUS", "JAP", "FIJ")
knitr::kable(gendiv, col.names = shortnames, digits = 4,caption = "") %>%
  kableExtra::kable_styling(full_width = FALSE,font_size = 5,
                            latex_options = c( "hold_position")) %>%
  kableExtra::landscape()
```

Table 36:

| | COR | BRZ | CAR | GOM | WNA | SAF | MOZ | RUN | SEY | ARS | SRL | TAI | IND | PNG | AUS | JAP | FIJ |
|---|---|---|---|---|---|---|---|---|---|---|---|---|---|---|---|---|---|
| ar | 1.0770 | 1.1230 | 1.0880 | 1.1140 | 1.1120 | 1.1640 | 1.1570 | 1.1670 | 1.1710 | 1.1690 | 1.1680 | 1.1690 | 1.1440 | 1.1620 | 1.1720 | 1.1700 | 1.1600 |
| size | 14.8250 | 51.5410 | 1.9920 | 39.1040 | 7.9670 | 22.6290 | 10.7600 | 27.7700 | 35.7970 | 14.9280 | 11.9440 | 4.9830 | 3.9750 | 7.9570 | 53.7240 | 43.7640 | 23.4910 |
| obs_het | 0.0440 | 0.0670 | 0.0630 | 0.0640 | 0.0640 | 0.0890 | 0.0870 | 0.0900 | 0.0920 | 0.0920 | 0.0950 | 0.1010 | 0.0890 | 0.0910 | 0.0910 | 0.0950 | 0.0870 |
| exp_het | 0.0450 | 0.0680 | 0.0470 | 0.0650 | 0.0610 | 0.0930 | 0.0910 | 0.0930 | 0.0930 | 0.0920 | 0.0910 | 0.0890 | 0.0790 | 0.0880 | 0.0950 | 0.0930 | 0.0910 |
| uexp_het | 0.0470 | 0.0690 | 0.0630 | 0.0660 | 0.0650 | 0.0950 | 0.0960 | 0.0940 | 0.0950 | 0.0960 | 0.0950 | 0.0990 | 0.0890 | 0.0940 | 0.0960 | 0.0940 | 0.0940 |
| fis | 0.0090 | 0.0250 | -0.3240 | 0.0220 | -0.0520 | 0.0240 | 0.0240 | 0.0200 | 0.0150 | -0.0030 | -0.0270 | -0.1230 | -0.1440 | -0.0420 | 0.0250 | -0.0030 | 0.0290 |
| hwe_glb | 1.0000 | 1.0000 | 1.0000 | 1.0000 | 1.0000 | 1.0000 | 1.0000 | 1.0000 | 1.0000 | 1.0000 | 1.0000 | 1.0000 | 1.0000 | 1.0000 | 1.0000 | 1.0000 | 1.0000 |
| hwe_hom | 1.0000 | 1.0000 | 1.0000 | 1.0000 | 1.0000 | 1.0000 | 1.0000 | 1.0000 | 1.0000 | 1.0000 | 1.0000 | 1.0000 | 1.0000 | 1.0000 | 1.0000 | 1.0000 | 1.0000 |
| hwe_het | 1.0000 | 1.0000 | 1.0000 | 1.0000 | 1.0000 | 1.0000 | 1.0000 | 1.0000 | 1.0000 | 1.0000 | 1.0000 | 1.0000 | 1.0000 | 1.0000 | 1.0000 | 1.0000 | 1.0000 |
| fis_lo | -0.0470 | 0.0040 | -1.0000 | -0.0050 | -0.1620 | -0.0100 | -0.0590 | -0.0110 | -0.0110 | -0.0580 | -0.0980 | -0.3820 | -0.5790 | -0.1600 | 0.0080 | -0.0270 | -0.0060 |
| fis_hi | 0.0050 | 0.0260 | -0.3240 | 0.0250 | -0.0600 | 0.0210 | 0.0160 | 0.0190 | 0.0140 | -0.0090 | -0.0370 | -0.1230 | -0.1440 | -0.0540 | 0.0280 | -0.0030 | 0.0300 |
| ar_lo | 1.0330 | 1.1090 | 1.0580 | 1.0880 | 1.0570 | 1.0820 | 1.0490 | 1.0870 | 1.1570 | 1.0840 | 1.0840 | 1.0900 | 1.0730 | 1.0740 | 1.1530 | 1.1250 | 1.0800 |
| ar_hi | 1.0890 | 1.1360 | 1.1160 | 1.1330 | 1.1270 | 1.1810 | 1.1830 | 1.1880 | 1.1880 | 1.1940 | 1.1980 | 1.1960 | 1.1800 | 1.1880 | 1.1920 | 1.1890 | 1.1840 |
| fis.hierfstat | 0.0418 | 0.0343 | -0.0841 | 0.0340 | 0.0101 | 0.0450 | 0.0681 | 0.0378 | 0.0282 | 0.0293 | 0.0139 | -0.0229 | -0.0180 | 0.0194 | 0.0344 | 0.0086 | 0.0500 |
| fis.CI.low | 0.0200 | 0.0188 | -0.0760 | 0.0135 | -0.0139 | 0.0430 | 0.0631 | 0.0277 | 0.0175 | 0.0141 | -0.0234 | -0.0551 | -0.0484 | 0.0013 | 0.0304 | -0.0282 | 0.0526 |
| fis.CI.high | 0.0898 | 0.0506 | 0.1036 | 0.0565 | 0.0637 | 0.0815 | 0.1264 | 0.0643 | 0.0486 | 0.0604 | 0.0306 | 0.0183 | 0.0466 | 0.0645 | 0.0588 | 0.0074 | 0.0929 |

### 9.6.1 Number of monomorphic markers per population

```r
pop.levels <- c("Costa Rica" , "Brazil","Caribbean Sea","Gulf of Mexico",
                "Western North Atlantic","Sierra Leone", "South Africa",
                "Mozambique", "Reunion" , "Seychelles", "Arabian Sea",
                "Sri Lanka", "Thailand", "Indonesia", "Papua New Guinea" ,
                "Australia", "Japan", "Fiji")

monomorphics <- c()
for (i in 1:length(pop.levels)) {
  x <- BS.dartcap.sub.gl[BS.dartcap.sub.gl$pop == pop.levels[i]]
  x2 <- dartR::gl.filter.monomorphs(x, verbose = 0)
  locs <-  adegenet::nLoc(x) - adegenet::nLoc(x2)
  monomorphics[i] <- locs
}
names(monomorphics) <- pop.levels
shortnames <- c("COR","BRZ", "CAR", "GOM", "WNA","SIL", "SAF", "MOZ", "RUN" ,
                "SEY", "ARS", "SRL", "TAI", "IND", "PNG" , "AUS", "JAP", "FIJ")
knitr::kable(t(as.table(monomorphics)), col.names = shortnames, digits = 4,
             caption = "") %>%
  kableExtra::kable_styling(full_width = FALSE,font_size = 5,
                            latex_options = c( "hold_position"))
```

Table 37:

| COR | BRZ | CAR | GOM | WNA | SIL | SAF | MOZ | RUN | SEY | ARS | SRL | TAI | IND | PNG | AUS | JAP | FIJ |
|---|---|---|---|---|---|---|---|---|---|---|---|---|---|---|---|---|---|
| 1335 | 812 | 1476 | 973 | 1270 | 1791 | 845 | 1082 | 813 | 741 | 969 | 1040 | 1294 | 1398 | 1175 | 627 | 831 | 929 |

### 9.6.2 Nucleotide diversity and inbreeding

```r
pi.sum <- radiator::pi(
  data = BS.dartcap.sub.tidy,
  read.length = 70,
  parallel.core = parallel::detectCores() - 1,
  path.folder = NULL,
  verbose = TRUE
)

FIS.sum<- radiator::ibdg_fh(data = BS.dartcap.sub.tidy,
        path.folder = NULL, verbose = TRUE)

save(pi.sum, FIS.sum, file = "DArTcap_aus_subset_pi_FIS_Sites.Rdata")
```

```r
load("DArTcap_aus_subset_pi_FIS_Sites.Rdata")
pi.sum$boxplot.pi
```

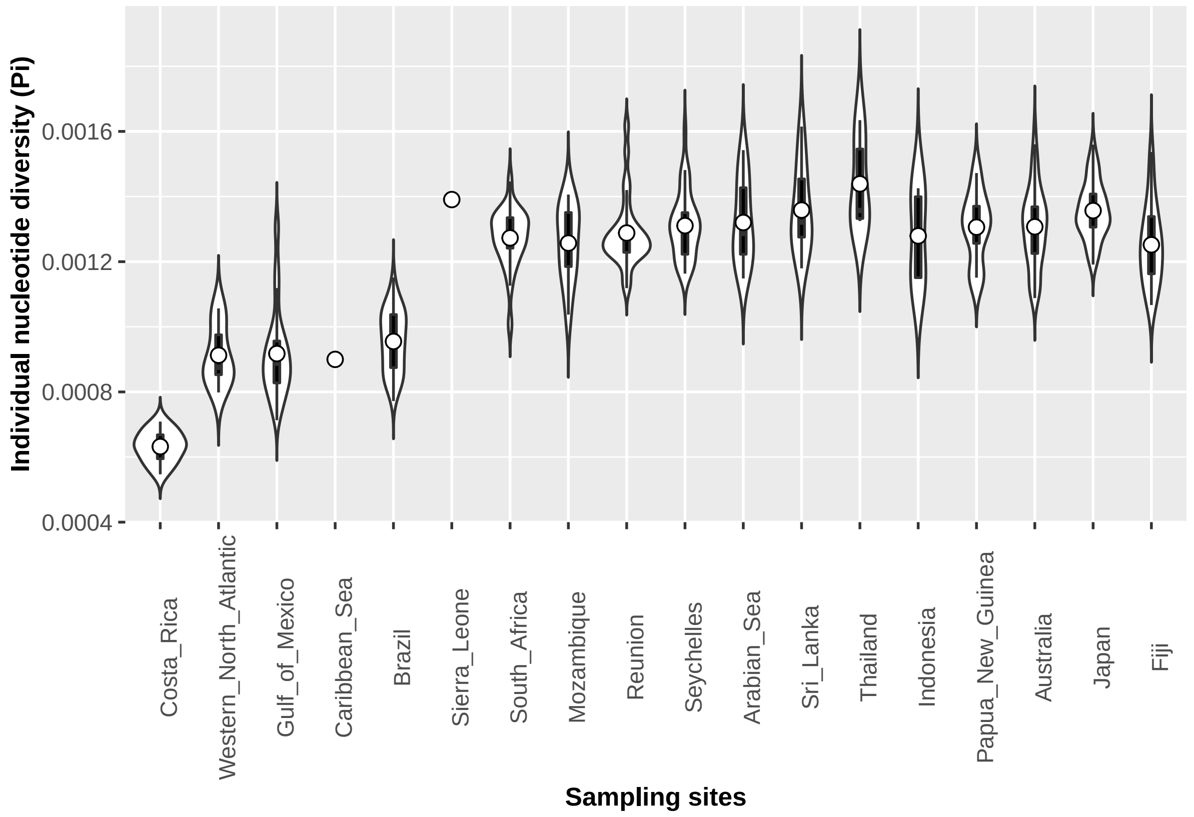


```
FIS.sum$fh.box.plot
```

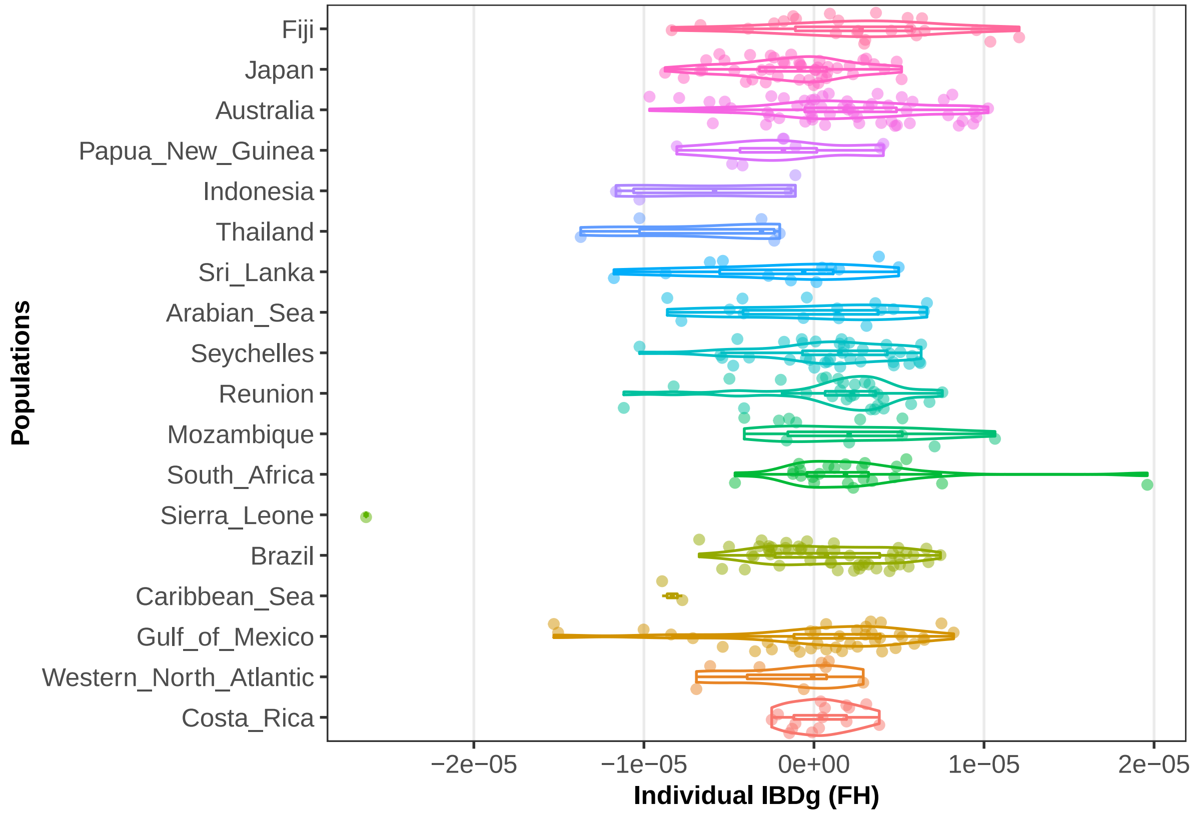

## 9.7 Genetic differentiation

```
Fst.gt <- strataG::popStructTest(
    BS.dartcap.sub.gt,
    nrep = 1000,
    stats = "all",
    type = "pairwise",
    keep.null = FALSE,
    quietly = TRUE,
    nb.cores =  parallel::detectCores() - 1,
    write.output = FALSE
  )
save(Fst.gt, file = "DArTcap_aus_subset_FST.Rdata")
readr::write_tsv(Fst.gt$pairwise$result, path = "DArTcap_aus_subset_FST.txt")
```

```
load("DArTcap_aus_subset_FST.Rdata")

kableExtra::kbl(Fst.gt$pairwise$result[order(Fst.gt$pairwise$result$Fst,
                                        decreasing = TRUE),
                                  -c(1:4)], digits = 4,
  caption = "Pairwise Fst for 1849 DArTcap loci between the all sites, but with a subset of Australi
  longtable = TRUE, booktabs = TRUE) %>%
  kableExtra::kable_styling(full_width = FALSE,font_size = 5,
                            latex_options = c("repeat_header", "hold_position")) %>%
  kableExtra::landscape()
```

Table 38: Pairwise Fst for 1849 DArTcap loci between the all sites, but with a subset of Australia

| | Chi2 | Chi2.p.val | D | D.p.val | Fis | Fis.p.val | Fst | Fst.p.val | F’st | F’st.p.val | Gst | Gst.p.val | G’st | G’st.p.val | G”st | G”st |
|---|---|---|---|---|---|---|---|---|---|---|---|---|---|---|---|---|
| Costa_Rica (15) v. Trinity_inlet (1) | 8283.4461 | 0.0619 | 0.0204 | 0.0619 | 3.2680 | 1.0000 | 0.7414 | 1.0000 | 0.7793 | 1.0000 | -3.9814 | 1.0000 | -2.0069 | 1.0000 | 2.0086 | 1 |
| Costa_Rica (15) v. Towns_River (2) | 9222.5207 | 0.0060 | 0.0252 | 0.0060 | 11.5056 | 1.0000 | 0.7261 | 0.0240 | 0.7667 | 0.0240 | 2.0202 | 0.0423 | 1.7680 | 0.0423 | 1.2543 | 0 |
| Costa_Rica (15) v. Sierra_Leone (1) | 7661.4419 | 0.0579 | 0.0166 | 0.0579 | 3.2672 | 1.0000 | 0.7221 | 1.0000 | 0.7592 | 1.0000 | -3.4660 | 1.0000 | -1.7230 | 1.0000 | 2.1042 | 1 |
| Costa_Rica (15) v. Darwin_Coastal (2) | 9257.6375 | 0.0050 | 0.0249 | 0.0050 | 12.5054 | 1.0000 | 0.7219 | 0.0133 | 0.7619 | 0.0133 | 1.8793 | 0.0219 | 1.6654 | 0.0219 | 1.2311 | 0 |
| Costa_Rica (15) v. Unknown (2) | 9189.7795 | 0.0090 | 0.0247 | 0.0090 | 12.3599 | 1.0000 | 0.7207 | 0.0256 | 0.7606 | 0.0256 | 1.9014 | 0.0435 | 1.6832 | 0.0435 | 1.2355 | 0 |
| Adelaide_River (4) v. Costa_Rica (15) | 10908.0732 | 0.0010 | 0.0266 | 0.0010 | -44.1032 | 1.0000 | 0.7088 | 0.0011 | 0.7613 | 0.0013 | 0.8711 | 0.0013 | 0.8955 | 0.0013 | 0.9441 | 0 |
| Costa_Rica (15) v. Daly_River (3) | 10225.9312 | 0.0020 | 0.0261 | 0.0020 | 381.6588 | 0.0040 | 0.7084 | 0.0028 | 0.7505 | 0.0028 | 1.0169 | 0.0040 | 1.0135 | 0.0040 | 1.0067 | 0 |
| Costa_Rica (15) v. Indonesia (4) | 10792.2218 | 0.0010 | 0.0264 | 0.0010 | NA | NA | 0.7064 | 0.0011 | 0.7496 | 0.0011 | NA | NA | NA | NA | NA | |
| Costa_Rica (15) v. Wenlock_River (4) | 11032.6671 | 0.0010 | 0.0266 | 0.0010 | -27.0705 | 1.0000 | 0.6990 | 0.0011 | 0.7429 | 0.0011 | 0.8096 | 0.0013 | 0.8450 | 0.0013 | 0.9143 | 0 |
| Costa_Rica (15) v. Thailand (5) | 11727.2773 | 0.0010 | 0.0272 | 0.0010 | -17.0550 | 1.0000 | 0.6913 | 0.0010 | 0.7365 | 0.0010 | 0.7263 | 0.0011 | 0.7748 | 0.0011 | 0.8696 | 0 |
| Costa_Rica (15) v. East_Alligator_River (7) | 12626.4320 | 0.0010 | 0.0259 | 0.0010 | -12.3826 | 1.0000 | 0.6815 | 0.0010 | 0.7280 | 0.0010 | 0.6599 | 0.0010 | 0.7160 | 0.0010 | 0.8289 | 0 |
| Costa_Rica (15) v. Okinawa (9) | 13962.7470 | 0.0010 | 0.0280 | 0.0010 | -11.8202 | 1.0000 | 0.6776 | 0.0010 | 0.7260 | 0.0010 | 0.6408 | 0.0010 | 0.6999 | 0.0010 | 0.8171 | 0 |
| Gulf_of_Mexico (40) v. Trinity_inlet (1) | 16001.1132 | 0.0250 | 0.0239 | 0.0250 | 4.0949 | 1.0000 | 0.6769 | 1.0000 | 0.7254 | 1.0000 | 13.4579 | 1.0000 | 9.2853 | 1.0000 | 1.5731 | 1 |
| Costa_Rica (15) v. Papua_New_Guinea (8) | 13298.5700 | 0.0010 | 0.0267 | 0.0010 | -10.6383 | 1.0000 | 0.6758 | 0.0010 | 0.7241 | 0.0010 | 0.6319 | 0.0010 | 0.6918 | 0.0010 | 0.8112 | 0 |
| Costa_Rica (15) v. Sydney_Harbour (8) | 13439.5029 | 0.0010 | 0.0274 | 0.0010 | -10.4232 | 1.0000 | 0.6744 | 0.0010 | 0.7239 | 0.0010 | 0.6278 | 0.0010 | 0.6889 | 0.0010 | 0.8089 | 0 |
| Trinity_inlet (1) v. Western_North_Atlantic (8) | 5008.5600 | 0.1189 | 0.0234 | 0.1189 | 3.1212 | 1.0000 | 0.6741 | 0.5409 | 0.7283 | 1.0000 | -4.3408 | 1.0000 | -2.1394 | 1.0000 | 1.9397 | 1 |
| Gulf_of_Mexico (40) v. Towns_River (2) | 18358.0969 | 0.0030 | 0.0286 | 0.0030 | 58.2252 | 0.2747 | 0.6713 | 0.0067 | 0.7206 | 0.0067 | 1.0919 | 0.7963 | 1.0718 | 0.7963 | 1.0343 | 0 |
| Caribbean_Sea (2) v. Trinity_inlet (1) | 1969.5667 | 0.3467 | 0.0234 | 0.3467 | NA | NA | 0.6711 | 0.3467 | 0.7214 | 0.3467 | NA | NA | NA | NA | NA | |
| Adelaide_River (4) v. Gulf_of_Mexico (40) | 22033.4331 | 0.0010 | 0.0306 | 0.0010 | -9.6500 | 0.9812 | 0.6703 | 0.0010 | 0.7479 | 0.0010 | 0.6557 | 0.0010 | 0.7170 | 0.0010 | 0.8291 | 0 |
| Darwin_Coastal (2) v. Gulf_of_Mexico (40) | 18729.0359 | 0.0010 | 0.0286 | 0.0010 | 137.1104 | 0.0030 | 0.6698 | 0.0023 | 0.7484 | 0.0030 | 1.0368 | 0.7508 | 1.0289 | 0.7508 | 1.0142 | 0 |
| Gulf_of_Mexico (40) v. Unknown (2) | 18394.7831 | 0.0010 | 0.0289 | 0.0010 | 102.7843 | 0.0032 | 0.6693 | 0.0024 | 0.7182 | 0.0024 | 1.0502 | 0.7331 | 1.0393 | 0.7331 | 1.0192 | 0 |
| Gulf_of_Mexico (40) v. Indonesia (4) | 21967.7890 | 0.0010 | 0.0305 | 0.0010 | NA | NA | 0.6687 | 0.0010 | 0.7187 | 0.0010 | NA | NA | NA | NA | NA | |
| Daly_River (3) v. Gulf_of_Mexico (40) | 20742.6390 | 0.0010 | 0.0303 | 0.0010 | -12.9215 | 0.2460 | 0.6678 | 0.0012 | 0.7462 | 0.0012 | 0.7187 | 0.0012 | 0.7716 | 0.0012 | 0.8671 | 0 |
| Costa_Rica (15) v. South_Alligator_River (10) | 14341.6767 | 0.0010 | 0.0265 | 0.0010 | -9.4239 | 1.0000 | 0.6671 | 0.0010 | 0.7161 | 0.0010 | 0.6054 | 0.0010 | 0.6679 | 0.0010 | 0.7931 | 0 |
| Gulf_of_Mexico (40) v. Wenlock_River (4) | 22399.0465 | 0.0010 | 0.0303 | 0.0010 | -7.9557 | 0.4767 | 0.6655 | 0.0010 | 0.7159 | 0.0010 | 0.6189 | 0.0010 | 0.6859 | 0.0010 | 0.8060 | 0 |
| Gulf_of_Mexico (40) v. Thailand (5) | 23758.0033 | 0.0010 | 0.0308 | 0.0010 | -6.5729 | 0.9990 | 0.6639 | 0.0010 | 0.7148 | 0.0010 | 0.5763 | 0.0010 | 0.6484 | 0.0010 | 0.7769 | 0 |
| Gulf_of_Mexico (40) v. Okinawa (9) | 27391.1075 | 0.0010 | 0.0320 | 0.0010 | -5.5230 | 1.0000 | 0.6622 | 0.0010 | 0.7142 | 0.0010 | 0.5356 | 0.0010 | 0.6107 | 0.0010 | 0.7465 | 0 |
| Towns_River (2) v. Western_North_Atlantic (8) | 5814.0950 | 0.0290 | 0.0280 | 0.0290 | 11.2684 | 0.7018 | 0.6622 | 0.0420 | 0.7164 | 0.0588 | 1.8961 | 0.4077 | 1.6634 | 0.4077 | 1.2291 | 0 |
| Brazil (52) v. Trinity_inlet (1) | 16194.6308 | 0.0180 | 0.0234 | 0.0180 | 4.6021 | 1.0000 | 0.6606 | 0.4615 | 0.7103 | 0.4615 | 4.5555 | 1.0000 | 3.4695 | 1.0000 | 1.4445 | 1 |
| East_Alligator_River (7) v. Gulf_of_Mexico (40) | 25167.0866 | 0.0010 | 0.0300 | 0.0010 | -5.5072 | 1.0000 | 0.6604 | 0.0010 | 0.7363 | 0.0010 | 0.5436 | 0.0010 | 0.6171 | 0.0010 | 0.7520 | 0 |
| Gulf_of_Mexico (40) v. Sydney_Harbour (8) | 26569.0852 | 0.0010 | 0.0312 | 0.0010 | -4.9426 | 1.0000 | 0.6593 | 0.0010 | 0.7118 | 0.0010 | 0.5261 | 0.0010 | 0.6025 | 0.0010 | 0.7395 | 0 |
| Gulf_of_Mexico (40) v. Papua_New_Guinea (8) | 26294.0411 | 0.0010 | 0.0305 | 0.0010 | -4.7993 | 1.0000 | 0.6589 | 0.0010 | 0.7107 | 0.0010 | 0.5264 | 0.0010 | 0.6018 | 0.0010 | 0.7391 | 0 |
| Darwin_Coastal (2) v. Western_North_Atlantic (8) | 5822.3231 | 0.0310 | 0.0278 | 0.0310 | 12.2432 | 0.3403 | 0.6580 | 0.0471 | 0.7125 | 0.0541 | 1.7761 | 0.3403 | 1.5768 | 0.3403 | 1.2078 | 0 |
| Unknown (2) v. Western_North_Atlantic (8) | 5787.2291 | 0.0170 | 0.0280 | 0.0170 | 11.9544 | 0.4007 | 0.6578 | 0.0231 | 0.7122 | 0.0304 | 1.8144 | 0.3596 | 1.6053 | 0.3596 | 1.2151 | 0 |
| Costa_Rica (15) v. Mozambique (11) | 14811.2507 | 0.0010 | 0.0265 | 0.0010 | -8.0933 | 1.0000 | 0.6574 | 0.0010 | 0.7168 | 0.0010 | 0.5824 | 0.0010 | 0.6474 | 0.0010 | 0.7772 | 0 |
| Adelaide_River (4) v. Brazil (52) | 24872.8512 | 0.0010 | 0.0298 | 0.0010 | -6.8222 | 0.9886 | 0.6572 | 0.0010 | 0.7183 | 0.0010 | 0.6052 | 0.0010 | 0.6723 | 0.0010 | 0.7958 | 0 |
| Brazil (52) v. Towns_River (2) | 19897.1581 | 0.0010 | 0.0279 | 0.0010 | -54.6719 | 0.3446 | 0.6568 | 0.0020 | 0.7070 | 0.0020 | 0.9245 | 0.0026 | 0.9399 | 0.0026 | 0.9688 | 0 |
| Gulf_of_Mexico (40) v. South_Alligator_River (10) | 27892.8176 | 0.0010 | 0.0306 | 0.0010 | -4.5377 | 1.0000 | 0.6560 | 0.0010 | 0.7080 | 0.0010 | 0.5142 | 0.0010 | 0.5904 | 0.0010 | 0.7295 | 0 |
| Brazil (52) v. Indonesia (4) | 24789.4679 | 0.0010 | 0.0297 | 0.0010 | NA | NA | 0.6554 | 0.0010 | 0.7061 | 0.0010 | NA | NA | NA | NA | NA | |
| Clarence_River (11) v. Costa_Rica (15) | 15107.8708 | 0.0010 | 0.0272 | 0.0010 | -7.4903 | 1.0000 | 0.6553 | 0.0010 | 0.7111 | 0.0010 | 0.5719 | 0.0010 | 0.6390 | 0.0010 | 0.7703 | 0 |
| Costa_Rica (15) v. Sri_Lanka (12) | 15422.5073 | 0.0010 | 0.0268 | 0.0010 | -7.7350 | 1.0000 | 0.6551 | 0.0010 | 0.7061 | 0.0010 | 0.5741 | 0.0010 | 0.6400 | 0.0010 | 0.7713 | 0 |
| Brazil (52) v. Darwin_Coastal (2) | 20142.8088 | 0.0020 | 0.0278 | 0.0020 | -35.8236 | 0.1843 | 0.6549 | 0.0040 | 0.7049 | 0.0040 | 0.8898 | 0.0051 | 0.9120 | 0.0051 | 0.9534 | 0 |
| Brazil (52) v. Unknown (2) | 19725.1591 | 0.0020 | 0.0281 | 0.0020 | -44.9478 | 0.1772 | 0.6545 | 0.0041 | 0.7045 | 0.0041 | 0.9093 | 0.0079 | 0.9277 | 0.0079 | 0.9621 | 0 |
| Brazil (52) v. Daly_River (3) | 22999.2758 | 0.0010 | 0.0295 | 0.0010 | -8.4252 | 0.1928 | 0.6544 | 0.0011 | 0.7050 | 0.0011 | 0.6515 | 0.0012 | 0.7136 | 0.0012 | 0.8266 | 0 |
| Brazil (52) v. Wenlock_River (4) | 25237.7951 | 0.0010 | 0.0295 | 0.0010 | -5.8267 | 0.2828 | 0.6529 | 0.0010 | 0.7039 | 0.0010 | 0.5741 | 0.0010 | 0.6456 | 0.0010 | 0.7749 | 0 |
| Brazil (52) v. Thailand (5) | 27112.0193 | 0.0010 | 0.0301 | 0.0010 | -4.9947 | 0.9910 | 0.6522 | 0.0010 | 0.7037 | 0.0010 | 0.5410 | 0.0010 | 0.6159 | 0.0010 | 0.7508 | 0 |
| Brazil (52) v. Okinawa (9) | 31644.5308 | 0.0010 | 0.0312 | 0.0010 | -4.4824 | 1.0000 | 0.6517 | 0.0010 | 0.7039 | 0.0010 | 0.5122 | 0.0010 | 0.5886 | 0.0010 | 0.7279 | 0 |
| Adelaide_River (4) v. Western_North_Atlantic (8) | 7323.2616 | 0.0050 | 0.0299 | 0.0050 | -35.6066 | 1.0000 | 0.6517 | 0.0050 | 0.7071 | 0.0050 | 0.8587 | 0.0050 | 0.8872 | 0.0050 | 0.9393 | 0 |
| Gulf_of_Mexico (40) v. Mozambique (11) | 28536.3039 | 0.0010 | 0.0305 | 0.0010 | -4.1008 | 1.0000 | 0.6515 | 0.0010 | 0.7092 | 0.0010 | 0.5020 | 0.0010 | 0.5793 | 0.0010 | 0.7199 | 0 |
| Clarence_River (11) v. Gulf_of_Mexico (40) | 29041.3696 | 0.0010 | 0.0310 | 0.0010 | -3.7833 | 1.0000 | 0.6506 | 0.0010 | 0.7260 | 0.0010 | 0.4939 | 0.0010 | 0.5729 | 0.0010 | 0.7141 | 0 |
| Gulf_of_Mexico (40) v. Sri_Lanka (12) | 29374.9490 | 0.0010 | 0.0305 | 0.0010 | -3.8221 | 1.0000 | 0.6502 | 0.0010 | 0.7032 | 0.0010 | 0.4957 | 0.0010 | 0.5734 | 0.0010 | 0.7148 | 0 |
| Indonesia (4) v. Western_North_Atlantic (8) | 7265.3114 | 0.0040 | 0.0296 | 0.0040 | NA | NA | 0.6500 | 0.0040 | 0.7052 | 0.0040 | NA | NA | NA | NA | NA | |
| Brazil (52) v. East_Alligator_River (7) | 28932.2700 | 0.0010 | 0.0292 | 0.0010 | -4.3123 | 1.0000 | 0.6491 | 0.0010 | 0.7005 | 0.0010 | 0.5162 | 0.0010 | 0.5915 | 0.0010 | 0.7306 | 0 |

Table 38: Pairwise Fst for 1849 DArTcap loci between the all sites, but with a subset of Australia
*(continued)*

| | Chi2 | Chi2.p.val | D | D.p.val | Fis | Fis.p.val | Fst | Fst.p.val | F'st | F'st.p.val | Gst | Gst.p.val | G'st | G'st.p.val | G”st | G”st |
|---|---|---|---|---|---|---|---|---|---|---|---|---|---|---|---|---|
| Brazil (52) v. Sydney_Harbour (8) | 30704.2647 | 0.0010 | 0.0304 | 0.0010 | -3.8210 | 1.0000 | 0.6489 | 0.0010 | 0.7016 | 0.0010 | 0.5006 | 0.0010 | 0.5783 | 0.0010 | 0.7190 | 0 |
| Brazil (52) v. Papua_New_Guinea (8) | 30294.1036 | 0.0010 | 0.0297 | 0.0010 | -3.8839 | 1.0000 | 0.6482 | 0.0010 | 0.7003 | 0.0010 | 0.5033 | 0.0010 | 0.5800 | 0.0010 | 0.7206 | 0 |
| Gulf_of_Mexico (40) v. Sierra_Leone (1) | 14355.5370 | 0.0200 | 0.0186 | 0.0200 | 4.0852 | 1.0000 | 0.6474 | 1.0000 | 0.6939 | 1.0000 | 42.8681 | 1.0000 | 29.3232 | 1.0000 | 1.6456 | 1 |
| Arabian_Sea (15) v. Gulf_of_Mexico (40) | 31767.5391 | 0.0010 | 0.0307 | 0.0010 | -3.2755 | 1.0000 | 0.6469 | 0.0010 | 0.7208 | 0.0010 | 0.4856 | 0.0010 | 0.5644 | 0.0010 | 0.7068 | 0 |
| Daly_River (3) v. Western_North_Atlantic (8) | 6679.0770 | 0.0050 | 0.0295 | 0.0050 | -685.1037 | 1.0000 | 0.6468 | 0.0051 | 0.7028 | 0.0053 | 0.9917 | 0.0053 | 0.9935 | 0.0053 | 0.9967 | 0 |
| Brazil (52) v. South_Alligator_River (10) | 32317.7661 | 0.0010 | 0.0297 | 0.0010 | -3.5437 | 1.0000 | 0.6461 | 0.0010 | 0.6983 | 0.0010 | 0.4919 | 0.0010 | 0.5691 | 0.0010 | 0.7112 | 0 |
| Gulf_of_Mexico (40) v. Urauchi_River (35) | 45733.6541 | 0.0010 | 0.0335 | 0.0010 | -3.2182 | 1.0000 | 0.6458 | 0.0010 | 0.7037 | 0.0010 | 0.4911 | 0.0010 | 0.5709 | 0.0010 | 0.7122 | 0 |
| Arabian_Sea (15) v. Costa_Rica (15) | 17106.4638 | 0.0010 | 0.0269 | 0.0010 | -6.3976 | 1.0000 | 0.6456 | 0.0010 | 0.7014 | 0.0010 | 0.5518 | 0.0010 | 0.6201 | 0.0010 | 0.7552 | 0 |
| Fiji (24) v. Gulf_of_Mexico (40) | 37844.7634 | 0.0010 | 0.0320 | 0.0010 | -3.4626 | 1.0000 | 0.6451 | 0.0010 | 0.7173 | 0.0010 | 0.4884 | 0.0010 | 0.5674 | 0.0010 | 0.7094 | 0 |
| Sierra_Leone (1) v. Western_North_Atlantic (8) | 4475.4535 | 0.1109 | 0.0179 | 0.1109 | 3.1210 | 1.0000 | 0.6427 | 0.5337 | 0.6945 | 1.0000 | -3.6139 | 1.0000 | -1.7595 | 1.0000 | 2.0557 | 1 |
| Brazil (52) v. Mozambique (11) | 33120.9672 | 0.0010 | 0.0297 | 0.0010 | -3.2204 | 1.0000 | 0.6425 | 0.0010 | 0.6992 | 0.0010 | 0.4814 | 0.0010 | 0.5596 | 0.0010 | 0.7027 | 0 |
| Brazil (52) v. Clarence_River (11) | 33703.3157 | 0.0010 | 0.0301 | 0.0010 | -2.9623 | 1.0000 | 0.6418 | 0.0010 | 0.6951 | 0.0010 | 0.4739 | 0.0010 | 0.5535 | 0.0010 | 0.6971 | 0 |
| Brazil (52) v. Sri_Lanka (12) | 33981.3894 | 0.0010 | 0.0297 | 0.0010 | -3.1417 | 1.0000 | 0.6412 | 0.0010 | 0.6942 | 0.0010 | 0.4774 | 0.0010 | 0.5557 | 0.0010 | 0.6993 | 0 |
| Brazil (52) v. Urauchi_River (35) | 52029.7382 | 0.0010 | 0.0326 | 0.0010 | -2.5235 | 1.0000 | 0.6405 | 0.0010 | 0.6977 | 0.0010 | 0.4762 | 0.0010 | 0.5564 | 0.0010 | 0.6995 | 0 |
| Caribbean_Sea (2) v. Sierra_Leone (1) | 1784.8167 | 0.3397 | 0.0178 | 0.3397 | NA | NA | 0.6403 | 0.3397 | 0.6886 | 0.3397 | NA | NA | NA | NA | NA | |
| Caribbean_Sea (2) v. Towns_River (2) | 2563.0048 | 0.3457 | 0.0281 | 0.3457 | NA | NA | 0.6394 | 0.3457 | 0.7010 | 0.1698 | NA | NA | NA | NA | NA | |
| Arabian_Sea (15) v. Brazil (52) | 36760.3836 | 0.0010 | 0.0299 | 0.0010 | -2.6437 | 1.0000 | 0.6391 | 0.0010 | 0.7002 | 0.0010 | 0.4686 | 0.0010 | 0.5479 | 0.0010 | 0.6921 | 0 |
| Wenlock_River (4) v. Western_North_Atlantic (8) | 7366.7088 | 0.0040 | 0.0296 | 0.0040 | -22.6825 | 1.0000 | 0.6389 | 0.0040 | 0.6957 | 0.0040 | 0.7985 | 0.0040 | 0.8384 | 0.0040 | 0.9101 | 0 |
| Brazil (52) v. Fiji (24) | 43520.2780 | 0.0010 | 0.0312 | 0.0010 | -2.7987 | 1.0000 | 0.6386 | 0.0010 | 0.7006 | 0.0010 | 0.4726 | 0.0010 | 0.5520 | 0.0010 | 0.6958 | 0 |
| Gulf_of_Mexico (40) v. South_Africa (23) | 36690.5573 | 0.0010 | 0.0306 | 0.0010 | -2.5036 | 1.0000 | 0.6384 | 0.0010 | 0.7000 | 0.0010 | 0.4741 | 0.0010 | 0.5531 | 0.0010 | 0.6968 | 0 |
| Costa_Rica (15) v. Fiji (24) | 21613.7417 | 0.0010 | 0.0280 | 0.0010 | -6.1858 | 1.0000 | 0.6369 | 0.0010 | 0.7048 | 0.0010 | 0.5462 | 0.0010 | 0.6151 | 0.0010 | 0.7511 | 0 |
| Gulf_of_Mexico (40) v. Reunion (28) | 40078.5966 | 0.0010 | 0.0308 | 0.0010 | -2.4030 | 1.0000 | 0.6365 | 0.0010 | 0.6949 | 0.0010 | 0.4737 | 0.0010 | 0.5530 | 0.0010 | 0.6967 | 0 |
| Thailand (5) v. Western_North_Atlantic (8) | 8033.0419 | 0.0030 | 0.0301 | 0.0030 | -14.7546 | 1.0000 | 0.6351 | 0.0030 | 0.6924 | 0.0030 | 0.7200 | 0.0030 | 0.7731 | 0.0030 | 0.8681 | 0 |
| Costa_Rica (15) v. Urauchi_River (35) | 27662.1809 | 0.0010 | 0.0291 | 0.0010 | -6.0830 | 1.0000 | 0.6343 | 0.0010 | 0.6924 | 0.0010 | 0.5466 | 0.0010 | 0.6163 | 0.0010 | 0.7519 | 0 |
| Okinawa (9) v. Western_North_Atlantic (8) | 10363.4737 | 0.0010 | 0.0312 | 0.0010 | -10.3435 | 1.0000 | 0.6338 | 0.0010 | 0.6907 | 0.0010 | 0.6395 | 0.0010 | 0.7033 | 0.0010 | 0.8190 | 0 |
| East_Alligator_River (7) v. Western_North_Atlantic (8) | 9094.2573 | 0.0010 | 0.0293 | 0.0010 | -10.6806 | 1.0000 | 0.6329 | 0.0010 | 0.6893 | 0.0010 | 0.6553 | 0.0010 | 0.7164 | 0.0010 | 0.8287 | 0 |
| Brazil (52) v. South_Africa (23) | 42160.0679 | 0.0010 | 0.0297 | 0.0010 | -2.0854 | 1.0000 | 0.6321 | 0.0010 | 0.6922 | 0.0010 | 0.4600 | 0.0010 | 0.5392 | 0.0010 | 0.6844 | 0 |
| Brazil (52) v. Reunion (28) | 45869.0575 | 0.0010 | 0.0300 | 0.0010 | -1.8295 | 1.0000 | 0.6308 | 0.0010 | 0.6883 | 0.0010 | 0.4592 | 0.0010 | 0.5386 | 0.0010 | 0.6838 | 0 |
| Brazil (52) v. Sierra_Leone (1) | 14333.3910 | 0.0180 | 0.0181 | 0.0180 | 4.5739 | 1.0000 | 0.6300 | 1.0000 | 0.6775 | 1.0000 | 6.0027 | 1.0000 | 4.5323 | 1.0000 | 1.5044 | 1 |
| Gulf_of_Mexico (40) v. Seychelles (36) | 44912.0898 | 0.0010 | 0.0309 | 0.0010 | -2.1106 | 1.0000 | 0.6300 | 0.0010 | 0.6884 | 0.0010 | 0.4691 | 0.0010 | 0.5488 | 0.0010 | 0.6928 | 0 |
| Caribbean_Sea (2) v. Unknown (2) | 2542.7000 | 0.3526 | 0.0277 | 0.3526 | 3.7075 | 1.0000 | 0.6299 | 0.3526 | 0.6878 | 0.1858 | -7.0754 | 0.3526 | -3.9645 | 0.3526 | 1.8171 | 0 |
| Costa_Rica (15) v. South_Africa (23) | 20816.9559 | 0.0010 | 0.0266 | 0.0010 | -5.1465 | 1.0000 | 0.6295 | 0.0010 | 0.6943 | 0.0010 | 0.5297 | 0.0010 | 0.5993 | 0.0010 | 0.7381 | 0 |
| Papua_New_Guinea (8) v. Western_North_Atlantic (8) | 9706.4549 | 0.0010 | 0.0298 | 0.0010 | -9.0767 | 1.0000 | 0.6284 | 0.0010 | 0.6854 | 0.0010 | 0.6257 | 0.0010 | 0.6910 | 0.0010 | 0.8099 | 0 |
| Caribbean_Sea (2) v. Darwin_Coastal (2) | 2559.0238 | 0.3157 | 0.0278 | 0.3157 | 3.7461 | 1.0000 | 0.6267 | 0.3157 | 0.6847 | 0.1499 | -7.7041 | 1.0000 | -4.3619 | 1.0000 | 1.7998 | 0 |
| Sydney_Harbour (8) v. Western_North_Atlantic (8) | 9780.1098 | 0.0010 | 0.0305 | 0.0010 | -9.0582 | 1.0000 | 0.6264 | 0.0010 | 0.6842 | 0.0010 | 0.6239 | 0.0010 | 0.6901 | 0.0010 | 0.8092 | 0 |
| Brazil (52) v. Seychelles (36) | 50969.9405 | 0.0010 | 0.0300 | 0.0010 | -1.5817 | 1.0000 | 0.6254 | 0.0010 | 0.6828 | 0.0010 | 0.4554 | 0.0010 | 0.5352 | 0.0010 | 0.6806 | 0 |
| Costa_Rica (15) v. Reunion (28) | 23454.2807 | 0.0010 | 0.0269 | 0.0010 | -4.7007 | 1.0000 | 0.6251 | 0.0010 | 0.6845 | 0.0010 | 0.5245 | 0.0010 | 0.5947 | 0.0010 | 0.7341 | 0 |
| South_Alligator_River (10) v. Western_North_Atlantic (8) | 10838.2134 | 0.0010 | 0.0298 | 0.0010 | -8.1197 | 1.0000 | 0.6247 | 0.0010 | 0.6819 | 0.0010 | 0.6019 | 0.0010 | 0.6698 | 0.0010 | 0.7939 | 0 |
| Caribbean_Sea (2) v. Indonesia (4) | 3700.8168 | 0.0699 | 0.0295 | 0.0699 | NA | NA | 0.6214 | 0.0745 | 0.6826 | 0.1163 | NA | NA | NA | NA | NA | |
| Urauchi_River (35) v. Western_North_Atlantic (8) | 24604.6728 | 0.0010 | 0.0327 | 0.0010 | -5.1041 | 1.0000 | 0.6198 | 0.0010 | 0.6790 | 0.0010 | 0.5422 | 0.0010 | 0.6179 | 0.0010 | 0.7522 | 0 |
| Adelaide_River (4) v. Caribbean_Sea (2) | 3718.3094 | 0.0749 | 0.0296 | 0.0749 | 7.2692 | 1.0000 | 0.6189 | 0.0851 | 0.6763 | 0.0851 | 3.0806 | 0.0995 | 2.4719 | 0.0995 | 1.3607 | 0 |
| Mozambique (11) v. Western_North_Atlantic (8) | 11263.4552 | 0.0010 | 0.0297 | 0.0010 | -6.9854 | 1.0000 | 0.6164 | 0.0010 | 0.6744 | 0.0010 | 0.5790 | 0.0010 | 0.6495 | 0.0010 | 0.7780 | 0 |
| Sri_Lanka (12) v. Western_North_Atlantic (8) | 11868.0788 | 0.0010 | 0.0298 | 0.0010 | -6.5049 | 1.0000 | 0.6160 | 0.0010 | 0.6740 | 0.0010 | 0.5679 | 0.0010 | 0.6395 | 0.0010 | 0.7701 | 0 |
| Costa_Rica (15) v. Seychelles (36) | 27353.9216 | 0.0010 | 0.0269 | 0.0010 | -4.0224 | 1.0000 | 0.6158 | 0.0010 | 0.6746 | 0.0010 | 0.5142 | 0.0010 | 0.5852 | 0.0010 | 0.7261 | 0 |
| Fiji (24) v. Western_North_Atlantic (8) | 18191.8114 | 0.0010 | 0.0311 | 0.0010 | -5.3489 | 1.0000 | 0.6135 | 0.0010 | 0.6724 | 0.0010 | 0.5429 | 0.0010 | 0.6176 | 0.0010 | 0.7521 | 0 |
| Clarence_River (11) v. Western_North_Atlantic (8) | 11446.3472 | 0.0010 | 0.0302 | 0.0010 | -6.4380 | 1.0000 | 0.6127 | 0.0010 | 0.6717 | 0.0010 | 0.5666 | 0.0010 | 0.6393 | 0.0010 | 0.7698 | 0 |
| Arabian_Sea (15) v. Western_North_Atlantic (8) | 13534.4065 | 0.0010 | 0.0299 | 0.0010 | -5.4304 | 1.0000 | 0.6107 | 0.0010 | 0.6694 | 0.0010 | 0.5454 | 0.0010 | 0.6195 | 0.0010 | 0.7538 | 0 |
| Caribbean_Sea (2) v. Okinawa (9) | 6348.1732 | 0.0180 | 0.0310 | 0.0180 | 15.4804 | 0.0216 | 0.6097 | 0.0212 | 0.6726 | 0.0212 | 1.4948 | 0.0216 | 1.3717 | 0.0216 | 1.1490 | 0 |
| Caribbean_Sea (2) v. Urauchi_River (35) | 19161.2817 | 0.0010 | 0.0324 | 0.0010 | -55.0393 | 1.0000 | 0.6090 | 0.0014 | 0.6750 | 0.0018 | 0.9271 | 0.0019 | 0.9427 | 0.0019 | 0.9703 | 0 |
| South_Africa (23) v. Western_North_Atlantic (8) | 17466.8199 | 0.0010 | 0.0298 | 0.0010 | -4.1921 | 1.0000 | 0.6047 | 0.0010 | 0.6636 | 0.0010 | 0.5205 | 0.0010 | 0.5964 | 0.0010 | 0.7345 | 0 |
| Reunion (28) v. Western_North_Atlantic (8) | 20237.7524 | 0.0010 | 0.0301 | 0.0010 | -3.8775 | 1.0000 | 0.6046 | 0.0010 | 0.6637 | 0.0010 | 0.5155 | 0.0010 | 0.5919 | 0.0010 | 0.7308 | 0 |
| Caribbean_Sea (2) v. East_Alligator_River (7) | 5259.9809 | 0.0260 | 0.0293 | 0.0260 | 13.8073 | 0.0327 | 0.6029 | 0.0285 | 0.6651 | 0.0327 | 1.5621 | 0.0327 | 1.4218 | 0.0327 | 1.1646 | 0 |

Table 38: Pairwise Fst for 1849 DArTcap loci between the all sites, but with a subset of Australia
*(continued)*

| | Chi2 | Chi2.p.val | D | D.p.val | Fis | Fis.p.val | Fst | Fst.p.val | F'st | F'st.p.val | Gst | Gst.p.val | G'st | G'st.p.val | G"st | G"st |
|---|---|---|---|---|---|---|---|---|---|---|---|---|---|---|---|---|
| Caribbean_Sea (2) v. Daly_River (3) | 3149.1187 | 0.1209 | 0.0294 | 0.1209 | 5.8210 | 1.0000 | 0.6020 | 0.1209 | 0.6626 | 0.1209 | 5.2198 | 0.1315 | 3.8800 | 0.1315 | 1.4630 | 0 |
| Seychelles (36) v. Western_North_Atlantic (8) | 24232.9745 | 0.0010 | 0.0301 | 0.0010 | -3.3147 | 1.0000 | 0.5999 | 0.0010 | 0.6595 | 0.0010 | 0.5041 | 0.0010 | 0.5815 | 0.0010 | 0.7218 | 0 |
| Caribbean_Sea (2) v. Papua_New_Guinea (8) | 5760.8634 | 0.0260 | 0.0296 | 0.0260 | 17.1768 | 0.0308 | 0.5982 | 0.0295 | 0.6625 | 0.0295 | 1.4017 | 0.0308 | 1.3042 | 0.0308 | 1.1266 | 0 |
| Caribbean_Sea (2) v. South_Alligator_River (10) | 6781.6840 | 0.0160 | 0.0297 | 0.0160 | 22.4176 | 0.0210 | 0.5979 | 0.0183 | 0.6609 | 0.0194 | 1.2800 | 0.0210 | 1.2140 | 0.0210 | 1.0939 | 0 |
| Caribbean_Sea (2) v. Fiji (24) | 13143.0515 | 0.0050 | 0.0311 | 0.0050 | -91.8125 | 1.0000 | 0.5970 | 0.0100 | 0.6805 | 0.0133 | 0.9541 | 0.0135 | 0.9639 | 0.0135 | 0.9815 | 0 |
| Caribbean_Sea (2) v. Thailand (5) | 4251.8777 | 0.0529 | 0.0302 | 0.0529 | 10.0058 | 0.5177 | 0.5966 | 0.0555 | 0.6593 | 0.0555 | 1.9743 | 0.0646 | 1.7099 | 0.0646 | 1.2387 | 0 |
| Caribbean_Sea (2) v. Wenlock_River (4) | 3714.4194 | 0.0679 | 0.0295 | 0.0679 | 7.9460 | 0.3622 | 0.5952 | 0.0726 | 0.6574 | 0.0726 | 2.5621 | 0.0784 | 2.1141 | 0.0784 | 1.3128 | 0 |
| Caribbean_Sea (2) v. Sydney_Harbour (8) | 5786.2777 | 0.0220 | 0.0302 | 0.0220 | 17.8923 | 0.0285 | 0.5929 | 0.0269 | 0.6595 | 0.0269 | 1.3780 | 0.0285 | 1.2858 | 0.0285 | 1.1202 | 0 |
| Caribbean_Sea (2) v. Sri_Lanka (12) | 7665.6193 | 0.0200 | 0.0294 | 0.0200 | 53.9606 | 0.0251 | 0.5901 | 0.0234 | 0.6545 | 0.0242 | 1.0951 | 0.0251 | 1.0739 | 0.0251 | 1.0353 | 0 |
| Caribbean_Sea (2) v. Reunion (28) | 15203.0711 | 0.0020 | 0.0297 | 0.0020 | -17.6781 | 1.0000 | 0.5883 | 0.0030 | 0.6575 | 0.0031 | 0.8191 | 0.0032 | 0.8550 | 0.0032 | 0.9203 | 0 |
| Arabian_Sea (15) v. Caribbean_Sea (2) | 9164.2691 | 0.0070 | 0.0298 | 0.0070 | -220.7442 | 1.0000 | 0.5868 | 0.0080 | 0.6471 | 0.0080 | 0.9803 | 0.0085 | 0.9846 | 0.0085 | 0.9922 | 0 |
| Caribbean_Sea (2) v. Mozambique (11) | 7144.3222 | 0.0120 | 0.0295 | 0.0120 | 35.0583 | 0.0319 | 0.5863 | 0.0244 | 0.6684 | 0.0284 | 1.1574 | 0.0319 | 1.1215 | 0.0319 | 1.0563 | 0 |
| Caribbean_Sea (2) v. Seychelles (36) | 18801.7891 | 0.0030 | 0.0299 | 0.0030 | -11.6595 | 1.0000 | 0.5863 | 0.0040 | 0.6530 | 0.0043 | 0.7643 | 0.0044 | 0.8099 | 0.0044 | 0.8923 | 0 |
| Caribbean_Sea (2) v. South_Africa (23) | 12645.9916 | 0.0040 | 0.0296 | 0.0040 | -23.8798 | 1.0000 | 0.5858 | 0.0054 | 0.6649 | 0.0059 | 0.8548 | 0.0060 | 0.8841 | 0.0060 | 0.9375 | 0 |
| Caribbean_Sea (2) v. Clarence_River (11) | 7257.9437 | 0.0090 | 0.0300 | 0.0090 | 47.1972 | 0.0120 | 0.5831 | 0.0110 | 0.6491 | 0.0110 | 1.1102 | 0.0120 | 1.0851 | 0.0120 | 1.0403 | 0 |
| Caribbean_Sea (2) v. Costa_Rica (15) | 2893.6819 | 0.0060 | 0.0032 | 0.0060 | 9.3903 | 1.0000 | 0.4308 | 0.0192 | 0.4601 | 0.0341 | -52.5077 | 1.0000 | -43.5363 | 1.0000 | 1.8647 | 1 |
| Costa_Rica (15) v. Western_North_Atlantic (8) | 4105.2733 | 0.0010 | 0.0032 | 0.0010 | -30.8133 | 1.0000 | 0.3832 | 0.0010 | 0.4057 | 0.0010 | 0.6068 | 0.0010 | 0.6338 | 0.0010 | 0.7721 | 0 |
| Costa_Rica (15) v. Gulf_of_Mexico (40) | 8142.6036 | 0.0010 | 0.0034 | 0.0010 | -11.1297 | 1.0000 | 0.3526 | 0.0010 | 0.3869 | 0.0010 | 0.3368 | 0.0010 | 0.3682 | 0.0010 | 0.5274 | 0 |
| Brazil (52) v. Costa_Rica (15) | 9169.3636 | 0.0010 | 0.0032 | 0.0010 | -8.1577 | 1.0000 | 0.3323 | 0.0010 | 0.3565 | 0.0010 | 0.2953 | 0.0010 | 0.3258 | 0.0010 | 0.4795 | 0 |
| Sierra_Leone (1) v. Urauchi_River (35) | 3537.3346 | 0.0270 | 0.0019 | 0.0270 | 4.5406 | 1.0000 | 0.1875 | 1.0000 | 0.2084 | 1.0000 | -5.5054 | 1.0000 | -3.9614 | 1.0000 | 2.1012 | 1 |
| Okinawa (9) v. Sierra_Leone (1) | 1449.3241 | 0.1129 | 0.0016 | 0.1129 | 3.3895 | 1.0000 | 0.1572 | 1.0000 | 0.1730 | 1.0000 | -2.1137 | 1.0000 | -1.1208 | 1.0000 | 2.9042 | 1 |
| Indonesia (4) v. Sierra_Leone (1) | 935.4608 | 0.2348 | 0.0013 | 0.2348 | NA | NA | 0.1556 | 0.5622 | 0.1711 | 0.5622 | NA | NA | NA | NA | NA | |
| Fiji (24) v. Sierra_Leone (1) | 2413.7037 | 0.0430 | 0.0016 | 0.0430 | 4.4327 | 1.0000 | 0.1373 | 1.0000 | 0.1514 | 1.0000 | -4.8777 | 1.0000 | -3.4768 | 1.0000 | 2.1545 | 1 |
| Adelaide_River (4) v. Sierra_Leone (1) | 910.9826 | 0.2028 | 0.0014 | 0.2028 | 2.5969 | 1.0000 | 0.1325 | 1.0000 | 0.1460 | 1.0000 | -1.5533 | 1.0000 | -0.5169 | 1.0000 | 3.7415 | 1 |
| Sierra_Leone (1) v. Towns_River (2) | 662.4667 | 0.3536 | 0.0012 | 0.3536 | NA | NA | 0.1288 | 0.3536 | 0.1453 | 0.3536 | NA | NA | NA | NA | NA | |
| East_Alligator_River (7) v. Sierra_Leone (1) | 1156.7100 | 0.1359 | 0.0014 | 0.1359 | 3.2295 | 1.0000 | 0.1265 | 1.0000 | 0.1396 | 1.0000 | -2.0160 | 1.0000 | -1.0346 | 1.0000 | 3.0026 | 1 |
| Reunion (28) v. Sierra_Leone (1) | 2602.7451 | 0.0360 | 0.0014 | 0.0360 | 4.9788 | 1.0000 | 0.1248 | 0.5538 | 0.1379 | 0.5538 | -12.5774 | 1.0000 | -9.7044 | 1.0000 | 1.9246 | 1 |
| Arabian_Sea (15) v. Sierra_Leone (1) | 1781.1143 | 0.0639 | 0.0014 | 0.0639 | 4.1711 | 1.0000 | 0.1233 | 1.0000 | 0.1365 | 1.0000 | -3.9437 | 1.0000 | -2.7023 | 1.0000 | 2.2577 | 1 |
| Seychelles (36) v. Sierra_Leone (1) | 3018.7283 | 0.0270 | 0.0014 | 0.0270 | 5.5605 | 1.0000 | 0.1219 | 1.0000 | 0.1347 | 1.0000 | 36.3786 | 1.0000 | 29.5263 | 1.0000 | 1.7632 | 1 |
| Sierra_Leone (1) v. Sydney_Harbour (8) | 1283.9870 | 0.1209 | 0.0015 | 0.1209 | 3.4030 | 1.0000 | 0.1211 | 1.0000 | 0.1359 | 1.0000 | -2.2485 | 1.0000 | -1.2355 | 1.0000 | 2.7906 | 1 |
| Papua_New_Guinea (8) v. Sierra_Leone (1) | 1248.3091 | 0.1189 | 0.0013 | 0.1189 | 3.3834 | 1.0000 | 0.1199 | 0.5219 | 0.1325 | 0.5219 | -2.1846 | 1.0000 | -1.1957 | 1.0000 | 2.8535 | 1 |
| Sierra_Leone (1) v. Sri_Lanka (12) | 1556.1057 | 0.0719 | 0.0013 | 0.0719 | 3.8713 | 1.0000 | 0.1178 | 0.4444 | 0.1314 | 1.0000 | -2.9790 | 1.0000 | -1.9075 | 1.0000 | 2.4692 | 1 |
| Sierra_Leone (1) v. Thailand (5) | 978.5387 | 0.1698 | 0.0015 | 0.1698 | 2.9111 | 1.0000 | 0.1168 | 1.0000 | 0.1305 | 1.0000 | -1.7844 | 1.0000 | -0.7697 | 1.0000 | 3.2560 | 1 |
| Clarence_River (11) v. Sierra_Leone (1) | 1453.4856 | 0.0879 | 0.0015 | 0.0879 | 3.8156 | 1.0000 | 0.1139 | 0.5146 | 0.1265 | 0.5146 | -2.9214 | 1.0000 | -1.8332 | 1.0000 | 2.4746 | 1 |
| Sierra_Leone (1) v. South_Alligator_River (10) | 1400.4489 | 0.0839 | 0.0013 | 0.0839 | 3.5773 | 1.0000 | 0.1138 | 0.3294 | 0.1266 | 0.5091 | -2.3774 | 1.0000 | -1.3837 | 1.0000 | 2.7306 | 1 |
| Darwin_Coastal (2) v. Sierra_Leone (1) | 659.5417 | 0.3267 | 0.0011 | 0.3267 | NA | NA | 0.1104 | 0.3267 | 0.1220 | 0.3267 | NA | NA | NA | NA | NA | |
| Sierra_Leone (1) v. South_Africa (23) | 2193.7608 | 0.0430 | 0.0013 | 0.0799 | 4.7410 | 1.0000 | 0.1085 | 1.0000 | 0.1239 | 1.0000 | -7.5959 | 1.0000 | -5.7079 | 1.0000 | 2.0170 | 1 |
| Mozambique (11) v. Sierra_Leone (1) | 1497.0249 | 0.0939 | 0.0014 | 0.0939 | 3.7329 | 1.0000 | 0.1052 | 1.0000 | 0.1165 | 1.0000 | -2.7257 | 1.0000 | -1.6785 | 1.0000 | 2.5521 | 1 |
| Sierra_Leone (1) v. Wenlock_River (4) | 897.8710 | 0.2128 | 0.0016 | 0.2128 | 2.6582 | 1.0000 | 0.1021 | 0.5285 | 0.1144 | 1.0000 | -1.6367 | 1.0000 | -0.5907 | 1.0000 | 3.4985 | 1 |
| Sierra_Leone (1) v. Unknown (2) | 634.6667 | 0.3347 | 0.0010 | 0.3347 | NA | NA | 0.0965 | 0.3347 | 0.1079 | 0.3347 | NA | NA | NA | NA | NA | |
| Daly_River (3) v. Sierra_Leone (1) | 777.5623 | 0.2657 | 0.0014 | 0.2657 | 2.3085 | 1.0000 | 0.0859 | 1.0000 | 0.0956 | 1.0000 | -1.4345 | 1.0000 | -0.3314 | 1.0000 | 4.0639 | 1 |
| Fiji (24) v. Urauchi_River (35) | 4024.2388 | 0.0010 | 0.0009 | 0.0010 | -4.3109 | 1.0000 | 0.0717 | 0.0010 | 0.0794 | 0.0010 | 0.0750 | 0.0010 | 0.0889 | 0.0010 | 0.1525 | 0 |
| Trinity_inlet (1) v. Urauchi_River (35) | 1666.6853 | 0.0260 | 0.0011 | 0.0260 | 4.4311 | 1.0000 | 0.0649 | 0.5000 | 0.0722 | 1.0000 | -3.5925 | 1.0000 | -2.5347 | 1.0000 | 2.3634 | 1 |
| Okinawa (9) v. Trinity_inlet (1) | 950.1565 | 0.0839 | 0.0011 | 0.2008 | 3.3500 | 1.0000 | 0.0639 | 1.0000 | 0.0703 | 1.0000 | -1.8885 | 1.0000 | -0.9747 | 1.0000 | 3.2226 | 1 |
| Okinawa (9) v. Urauchi_River (35) | 2432.4996 | 0.0010 | 0.0007 | 0.0010 | -6.4012 | 0.9920 | 0.0633 | 0.0010 | 0.0702 | 0.0010 | 0.1362 | 0.0010 | 0.1579 | 0.0010 | 0.2588 | 0 |
| Darwin_Coastal (2) v. Urauchi_River (35) | 1785.5963 | 0.0030 | 0.0007 | 0.0320 | -148.9762 | 0.4298 | 0.0568 | 0.0115 | 0.0631 | 0.0129 | 0.9236 | 0.6125 | 0.9308 | 0.6080 | 0.9640 | 0 |
| Fiji (24) v. Okinawa (9) | 2148.5829 | 0.0010 | 0.0006 | 0.0010 | -6.0998 | 1.0000 | 0.0550 | 0.0010 | 0.0607 | 0.0010 | 0.1382 | 0.0010 | 0.1602 | 0.0010 | 0.2622 | 0 |
| Indonesia (4) v. Okinawa (9) | 1136.2430 | 0.0150 | 0.0006 | 0.0370 | NA | NA | 0.0508 | 0.0210 | 0.0562 | 0.0230 | NA | NA | NA | NA | NA | |
| Daly_River (3) v. Urauchi_River (35) | 1910.5521 | 0.0010 | 0.0006 | 0.0050 | -12.7465 | 0.0509 | 0.0500 | 0.0022 | 0.0555 | 0.0022 | 0.4084 | 0.3905 | 0.4439 | 0.3319 | 0.6051 | 0 |
| Adelaide_River (4) v. Urauchi_River (35) | 1905.4973 | 0.0010 | 0.0006 | 0.0040 | -9.6565 | 0.7655 | 0.0498 | 0.0041 | 0.0553 | 0.0041 | 0.2903 | 0.0194 | 0.3223 | 0.0194 | 0.4748 | 0 |
| Darwin_Coastal (2) v. Okinawa (9) | 1025.9434 | 0.0400 | 0.0007 | 0.1648 | 15.7435 | 0.8638 | 0.0493 | 0.1257 | 0.0547 | 0.0805 | 3.0311 | 0.4817 | 2.7481 | 0.4817 | 1.4336 | 0 |
| Sydney_Harbour (8) v. Urauchi_River (35) | 2307.4937 | 0.0010 | 0.0006 | 0.0010 | -6.0692 | 0.3447 | 0.0487 | 0.0010 | 0.0541 | 0.0010 | 0.1324 | 0.0020 | 0.1540 | 0.0010 | 0.2529 | 0 |

Table 38: Pairwise Fst for 1849 DArTcap loci between the all sites, but with a subset of Australia
*(continued)*

| | Chi2 | Chi2.p.val | D | D.p.val | Fis | Fis.p.val | Fst | Fst.p.val | F’st | F’st.p.val | Gst | Gst.p.val | G’st | G’st.p.val | G”st | G”st |
|---|---|---|---|---|---|---|---|---|---|---|---|---|---|---|---|---|
| Thailand (5) v. Urauchi_River (35) | 2122.4778 | 0.0010 | 0.0006 | 0.0020 | -7.4291 | 0.0821 | 0.0470 | 0.0010 | 0.0522 | 0.0010 | 0.2054 | 0.0320 | 0.2342 | 0.0280 | 0.3647 | 0 |
| Urauchi_River (35) v. Wenlock_River (4) | 2035.7053 | 0.0010 | 0.0005 | 0.0110 | -8.6894 | 0.0416 | 0.0469 | 0.0101 | 0.0517 | 0.0101 | 0.2666 | 0.1653 | 0.2991 | 0.1278 | 0.4466 | 0 |
| East_Alligator_River (7) v. Urauchi_River (35) | 2125.4060 | 0.0010 | 0.0005 | 0.0010 | -6.5674 | 0.7413 | 0.0469 | 0.0010 | 0.0521 | 0.0010 | 0.1517 | 0.0010 | 0.1748 | 0.0010 | 0.2835 | 0 |
| Reunion (28) v. Urauchi_River (35) | 3314.2143 | 0.0010 | 0.0005 | 0.0010 | -3.9530 | 1.0000 | 0.0468 | 0.0010 | 0.0519 | 0.0010 | 0.0554 | 0.0010 | 0.0660 | 0.0010 | 0.1151 | 0 |
| Towns_River (2) v. Urauchi_River (35) | 1574.6857 | 0.0030 | 0.0008 | 0.0230 | 321.1123 | 0.2485 | 0.0468 | 0.0112 | 0.0520 | 0.0134 | 1.0394 | 0.0676 | 1.0357 | 0.0676 | 1.0175 | 0 |
| South_Alligator_River (10) v. Urauchi_River (35) | 2278.7697 | 0.0010 | 0.0004 | 0.0010 | -5.7953 | 0.8681 | 0.0459 | 0.0010 | 0.0509 | 0.0010 | 0.1110 | 0.0010 | 0.1296 | 0.0010 | 0.2166 | 0 |
| Mozambique (11) v. Urauchi_River (35) | 2368.2467 | 0.0010 | 0.0004 | 0.0010 | -5.4065 | 0.6653 | 0.0444 | 0.0010 | 0.0493 | 0.0010 | 0.1008 | 0.0010 | 0.1183 | 0.0010 | 0.1990 | 0 |
| South_Africa (23) v. Urauchi_River (35) | 2934.1943 | 0.0010 | 0.0005 | 0.0010 | -4.1179 | 1.0000 | 0.0440 | 0.0010 | 0.0488 | 0.0010 | 0.0593 | 0.0010 | 0.0705 | 0.0010 | 0.1226 | 0 |
| Sri_Lanka (12) v. Urauchi_River (35) | 2425.6881 | 0.0010 | 0.0005 | 0.0010 | -5.1672 | 0.8611 | 0.0439 | 0.0010 | 0.0488 | 0.0010 | 0.0912 | 0.0010 | 0.1073 | 0.0010 | 0.1819 | 0 |
| Papua_New_Guinea (8) v. Urauchi_River (35) | 2065.3361 | 0.0010 | 0.0005 | 0.0010 | -6.2555 | 0.6294 | 0.0431 | 0.0010 | 0.0478 | 0.0010 | 0.1327 | 0.0020 | 0.1539 | 0.0020 | 0.2531 | 0 |
| Clarence_River (11) v. Urauchi_River (35) | 2409.3563 | 0.0010 | 0.0004 | 0.0010 | -5.2721 | 0.2388 | 0.0428 | 0.0010 | 0.0476 | 0.0010 | 0.0960 | 0.0010 | 0.1131 | 0.0010 | 0.1908 | 0 |
| Arabian_Sea (15) v. Urauchi_River (35) | 2605.1108 | 0.0010 | 0.0005 | 0.0010 | -4.7753 | 0.9401 | 0.0425 | 0.0010 | 0.0472 | 0.0010 | 0.0765 | 0.0010 | 0.0905 | 0.0010 | 0.1551 | 0 |
| Daly_River (3) v. Fiji (24) | 1731.2166 | 0.0010 | 0.0006 | 0.0010 | -12.0407 | 0.0737 | 0.0402 | 0.0011 | 0.0459 | 0.0012 | 0.4043 | 0.7450 | 0.4401 | 0.7181 | 0.6013 | 0 |
| Okinawa (9) v. Thailand (5) | 1196.3141 | 0.0070 | 0.0005 | 0.0320 | -15.0077 | 0.7992 | 0.0401 | 0.0240 | 0.0444 | 0.0240 | 0.4052 | 0.0609 | 0.4407 | 0.0599 | 0.6020 | 0 |
| Unknown (2) v. Urauchi_River (35) | 1644.1489 | 0.0070 | 0.0006 | 0.0549 | -270.8899 | 0.6652 | 0.0396 | 0.0137 | 0.0439 | 0.0084 | 0.9559 | 0.4700 | 0.9600 | 0.4700 | 0.9796 | 0 |
| Mozambique (11) v. Okinawa (9) | 1445.0771 | 0.0010 | 0.0004 | 0.0020 | -8.1618 | 1.0000 | 0.0392 | 0.0010 | 0.0434 | 0.0010 | 0.2018 | 0.0010 | 0.2298 | 0.0010 | 0.3591 | 0 |
| Okinawa (9) v. Towns_River (2) | 910.1759 | 0.0759 | 0.0007 | 0.0999 | 13.5855 | 0.9769 | 0.0382 | 0.1535 | 0.0421 | 0.1535 | 4.2907 | 0.0373 | 3.8174 | 0.0373 | 1.5325 | 0 |
| Seychelles (36) v. Urauchi_River (35) | 3210.9643 | 0.0010 | 0.0004 | 0.0010 | -3.6732 | 1.0000 | 0.0380 | 0.0010 | 0.0421 | 0.0010 | 0.0451 | 0.0010 | 0.0538 | 0.0010 | 0.0947 | 0 |
| Fiji (24) v. Mozambique (11) | 2051.6416 | 0.0010 | 0.0003 | 0.0010 | -5.1843 | 0.9930 | 0.0374 | 0.0010 | 0.0418 | 0.0010 | 0.1043 | 0.0010 | 0.1224 | 0.0010 | 0.2053 | 0 |
| East_Alligator_River (7) v. Okinawa (9) | 1188.0786 | 0.0110 | 0.0004 | 0.0240 | -11.7379 | 0.9840 | 0.0367 | 0.0130 | 0.0407 | 0.0130 | 0.3034 | 0.0130 | 0.3360 | 0.0130 | 0.4906 | 0 |
| Okinawa (9) v. South_Alligator_River (10) | 1350.8773 | 0.0010 | 0.0005 | 0.0010 | -9.2828 | 1.0000 | 0.0364 | 0.0010 | 0.0403 | 0.0010 | 0.2249 | 0.0010 | 0.2539 | 0.0010 | 0.3909 | 0 |
| Okinawa (9) v. Reunion (28) | 1846.5327 | 0.0010 | 0.0004 | 0.0010 | -5.3551 | 1.0000 | 0.0363 | 0.0010 | 0.0405 | 0.0010 | 0.1125 | 0.0010 | 0.1313 | 0.0010 | 0.2192 | 0 |
| Fiji (24) v. Sri_Lanka (12) | 2069.2960 | 0.0010 | 0.0004 | 0.0010 | -4.8808 | 0.9990 | 0.0361 | 0.0010 | 0.0400 | 0.0010 | 0.0928 | 0.0010 | 0.1092 | 0.0010 | 0.1848 | 0 |
| Arabian_Sea (15) v. Okinawa (9) | 1483.1431 | 0.0010 | 0.0004 | 0.0010 | -6.9062 | 1.0000 | 0.0355 | 0.0010 | 0.0394 | 0.0010 | 0.1577 | 0.0010 | 0.1817 | 0.0010 | 0.2932 | 0 |
| Okinawa (9) v. Sydney_Harbour (8) | 1279.3208 | 0.0020 | 0.0005 | 0.0030 | -10.0700 | 0.9900 | 0.0351 | 0.0060 | 0.0390 | 0.0060 | 0.2587 | 0.0080 | 0.2902 | 0.0080 | 0.4361 | 0 |
| Adelaide_River (4) v. Okinawa (9) | 1057.1778 | 0.0300 | 0.0004 | 0.0709 | -28.1316 | 0.9276 | 0.0350 | 0.0509 | 0.0387 | 0.0493 | 0.5974 | 0.0674 | 0.6264 | 0.0674 | 0.7661 | 0 |
| Fiji (24) v. Wenlock_River (4) | 1686.2553 | 0.0010 | 0.0005 | 0.0010 | -8.5160 | 0.0590 | 0.0348 | 0.0010 | 0.0385 | 0.0010 | 0.2708 | 0.4669 | 0.3037 | 0.4059 | 0.4521 | 0 |
| Adelaide_River (4) v. Fiji (24) | 1520.2940 | 0.0010 | 0.0005 | 0.0010 | -9.6552 | 0.6875 | 0.0347 | 0.0010 | 0.0396 | 0.0010 | 0.2972 | 0.1076 | 0.3295 | 0.0984 | 0.4832 | 0 |
| Fiji (24) v. Reunion (28) | 2589.1311 | 0.0010 | 0.0003 | 0.0010 | -3.7079 | 1.0000 | 0.0345 | 0.0010 | 0.0384 | 0.0010 | 0.0542 | 0.0010 | 0.0645 | 0.0010 | 0.1126 | 0 |
| Okinawa (9) v. Sri_Lanka (12) | 1415.0744 | 0.0010 | 0.0004 | 0.0010 | -7.7167 | 1.0000 | 0.0344 | 0.0010 | 0.0382 | 0.0010 | 0.1828 | 0.0010 | 0.2090 | 0.0010 | 0.3312 | 0 |
| Okinawa (9) v. Wenlock_River (4) | 1075.3271 | 0.0150 | 0.0004 | 0.1019 | -22.5017 | 0.5000 | 0.0344 | 0.0549 | 0.0381 | 0.0549 | 0.5419 | 0.2798 | 0.5746 | 0.2525 | 0.7241 | 0 |
| Clarence_River (11) v. Okinawa (9) | 1373.5403 | 0.0020 | 0.0004 | 0.0020 | -7.8838 | 0.9980 | 0.0344 | 0.0030 | 0.0382 | 0.0030 | 0.1914 | 0.0030 | 0.2190 | 0.0030 | 0.3445 | 0 |
| Fiji (24) v. South_Alligator_River (10) | 1944.1067 | 0.0010 | 0.0003 | 0.0010 | -5.5174 | 0.9960 | 0.0341 | 0.0010 | 0.0377 | 0.0010 | 0.1115 | 0.0010 | 0.1301 | 0.0010 | 0.2174 | 0 |
| Daly_River (3) v. Okinawa (9) | 1055.9473 | 0.0380 | 0.0005 | 0.1888 | -101.0515 | 0.4168 | 0.0332 | 0.0960 | 0.0369 | 0.0960 | 0.8683 | 0.5081 | 0.8803 | 0.5081 | 0.9360 | 0 |
| Fiji (24) v. Seychelles (36) | 2711.5330 | 0.0010 | 0.0003 | 0.0010 | -3.4246 | 1.0000 | 0.0327 | 0.0010 | 0.0364 | 0.0010 | 0.0471 | 0.0010 | 0.0562 | 0.0010 | 0.0987 | 0 |
| Fiji (24) v. Sydney_Harbour (8) | 1799.8069 | 0.0010 | 0.0004 | 0.0010 | -5.8538 | 0.6304 | 0.0326 | 0.0010 | 0.0362 | 0.0010 | 0.1317 | 0.0010 | 0.1531 | 0.0010 | 0.2516 | 0 |
| Fiji (24) v. South_Africa (23) | 2316.0134 | 0.0010 | 0.0004 | 0.0010 | -3.9623 | 1.0000 | 0.0324 | 0.0010 | 0.0364 | 0.0010 | 0.0595 | 0.0010 | 0.0708 | 0.0010 | 0.1230 | 0 |
| Okinawa (9) v. Unknown (2) | 938.6960 | 0.0689 | 0.0006 | 0.2288 | 15.0048 | 0.9129 | 0.0321 | 0.1044 | 0.0354 | 0.1044 | 3.4339 | 0.3076 | 3.0945 | 0.3076 | 1.4724 | 0 |
| Okinawa (9) v. Papua_New_Guinea (8) | 1208.1026 | 0.0070 | 0.0004 | 0.0120 | -10.3885 | 0.9840 | 0.0318 | 0.0140 | 0.0352 | 0.0140 | 0.2627 | 0.0160 | 0.2938 | 0.0150 | 0.4407 | 0 |
| Clarence_River (11) v. Fiji (24) | 1992.4897 | 0.0010 | 0.0003 | 0.0010 | -4.9915 | 0.8262 | 0.0316 | 0.0010 | 0.0358 | 0.0010 | 0.0962 | 0.0010 | 0.1134 | 0.0010 | 0.1912 | 0 |
| Okinawa (9) v. Seychelles (36) | 1808.3408 | 0.0010 | 0.0004 | 0.0010 | -4.9176 | 1.0000 | 0.0308 | 0.0010 | 0.0343 | 0.0010 | 0.0983 | 0.0010 | 0.1151 | 0.0010 | 0.1943 | 0 |
| Indonesia (4) v. Trinity_inlet (1) | 667.8216 | 0.1808 | 0.0006 | 0.3876 | NA | NA | 0.0304 | 0.6424 | 0.0334 | 0.6424 | NA | NA | NA | NA | NA | |
| East_Alligator_River (7) v. Fiji (24) | 1671.9338 | 0.0010 | 0.0004 | 0.0010 | -6.4000 | 0.8521 | 0.0303 | 0.0010 | 0.0344 | 0.0010 | 0.1519 | 0.0010 | 0.1749 | 0.0010 | 0.2837 | 0 |
| Darwin_Coastal (2) v. Indonesia (4) | 702.5932 | 0.3277 | 0.0006 | 0.2028 | NA | NA | 0.0301 | 0.3501 | 0.0334 | 0.4926 | NA | NA | NA | NA | NA | |
| Fiji (24) v. Thailand (5) | 1694.0464 | 0.0010 | 0.0004 | 0.0010 | -7.3051 | 0.1212 | 0.0300 | 0.0010 | 0.0332 | 0.0010 | 0.2070 | 0.2094 | 0.2359 | 0.1633 | 0.3670 | 0 |
| Fiji (24) v. Papua_New_Guinea (8) | 1699.2647 | 0.0010 | 0.0004 | 0.0010 | -6.0110 | 0.8472 | 0.0289 | 0.0010 | 0.0320 | 0.0010 | 0.1326 | 0.0010 | 0.1537 | 0.0010 | 0.2528 | 0 |
| Darwin_Coastal (2) v. Fiji (24) | 1471.1495 | 0.0090 | 0.0006 | 0.0250 | -141.3704 | 0.5160 | 0.0289 | 0.0085 | 0.0331 | 0.0115 | 0.9211 | 0.8484 | 0.9285 | 0.8484 | 0.9628 | 0 |
| Arabian_Sea (15) v. Fiji (24) | 2078.4294 | 0.0010 | 0.0003 | 0.0010 | -4.4720 | 0.9990 | 0.0288 | 0.0010 | 0.0325 | 0.0010 | 0.0747 | 0.0010 | 0.0884 | 0.0010 | 0.1518 | 0 |
| Okinawa (9) v. South_Africa (23) | 1575.4970 | 0.0010 | 0.0003 | 0.0010 | -5.7127 | 1.0000 | 0.0285 | 0.0010 | 0.0322 | 0.0010 | 0.1195 | 0.0010 | 0.1391 | 0.0010 | 0.2310 | 0 |
| Indonesia (4) v. Sydney_Harbour (8) | 971.3347 | 0.0529 | 0.0005 | 0.0190 | NA | NA | 0.0230 | 0.0320 | 0.0257 | 0.0320 | NA | NA | NA | NA | NA | |
| Clarence_River (11) v. Indonesia (4) | 1039.7297 | 0.0719 | 0.0004 | 0.0270 | NA | NA | 0.0226 | 0.0270 | 0.0251 | 0.0270 | NA | NA | NA | NA | NA | |
| East_Alligator_River (7) v. Indonesia (4) | 902.0496 | 0.0709 | 0.0004 | 0.1009 | NA | NA | 0.0222 | 0.0430 | 0.0246 | 0.0430 | NA | NA | NA | NA | NA | |

Table 38: Pairwise Fst for 1849 DArTcap loci between the all sites, but with a subset of Australia
*(continued)*

| | Chi2 | Chi2.p.val | D | D.p.val | Fis | Fis.p.val | Fst | Fst.p.val | F'st | F'st.p.val | Gst | Gst.p.val | G'st | G'st.p.val | G"st | G"st |
|---|---|---|---|---|---|---|---|---|---|---|---|---|---|---|---|---|
| Indonesia (4) v. South_Alligator_River (10) | 1014.3479 | 0.0290 | 0.0004 | 0.0629 | NA | NA | 0.0217 | 0.0250 | NA | NA | NA | NA | NA | NA | NA | |
| Towns_River (2) v. Trinity_inlet (1) | 514.2333 | 0.3367 | 0.0005 | 0.6633 | NA | NA | 0.0208 | 0.3367 | 0.0229 | 0.3367 | NA | NA | NA | NA | NA | |
| Fiji (24) v. Trinity_inlet (1) | 1235.3158 | 0.1319 | 0.0009 | 0.6324 | 4.3398 | 1.0000 | 0.0188 | 1.0000 | 0.0207 | 1.0000 | -3.4436 | 1.0000 | -2.4129 | 1.0000 | 2.3967 | 1 |
| Daly_River (3) v. East_Alligator_River (7) | 892.7082 | 0.0320 | 0.0004 | 0.0370 | -516.5499 | 0.5186 | 0.0185 | 0.0292 | 0.0206 | 0.0292 | 0.9730 | 0.7704 | 0.9756 | 0.7674 | 0.9876 | 0 |
| Indonesia (4) v. Towns_River (2) | 677.8618 | 0.3946 | 0.0006 | 0.2687 | NA | NA | 0.0183 | 0.3357 | 0.0202 | 0.3357 | NA | NA | NA | NA | NA | |
| Darwin_Coastal (2) v. Thailand (5) | 769.5439 | 0.0589 | 0.0005 | 0.1578 | 10.2804 | 1.0000 | 0.0181 | 0.0615 | 0.0202 | 0.0615 | 9.2098 | 0.0723 | 7.8492 | 0.0723 | 1.6708 | 0 |
| Adelaide_River (4) v. Indonesia (4) | 779.0209 | 0.3726 | 0.0004 | 0.3686 | NA | NA | 0.0176 | 0.3726 | 0.0195 | 0.3626 | NA | NA | NA | NA | NA | |
| Indonesia (4) v. Thailand (5) | 843.8398 | 0.1399 | 0.0004 | 0.2048 | NA | NA | 0.0174 | 0.2008 | 0.0193 | 0.2008 | NA | NA | NA | NA | NA | |
| Fiji (24) v. Towns_River (2) | 1248.1029 | 0.0599 | 0.0006 | 0.0070 | 174.3806 | 0.4225 | 0.0173 | 0.0365 | 0.0191 | 0.0365 | 1.0735 | 0.3526 | 1.0664 | 0.3526 | 1.0320 | 0 |
| Indonesia (4) v. Mozambique (11) | 1008.3876 | 0.0889 | 0.0004 | 0.0729 | NA | NA | 0.0173 | 0.0412 | NA | NA | NA | NA | NA | NA | NA | |
| Indonesia (4) v. Reunion (28) | 1297.9224 | 0.0320 | 0.0003 | 0.0340 | NA | NA | 0.0170 | 0.0171 | NA | NA | NA | NA | NA | NA | NA | |
| Indonesia (4) v. Seychelles (36) | 1356.4138 | 0.0160 | 0.0003 | 0.0210 | NA | NA | 0.0157 | 0.0070 | NA | NA | NA | NA | NA | NA | NA | |
| Indonesia (4) v. Papua_New_Guinea (8) | 917.6883 | 0.1169 | 0.0003 | 0.1638 | NA | NA | 0.0157 | 0.1139 | NA | NA | NA | NA | NA | NA | NA | |
| Indonesia (4) v. Wenlock_River (4) | 811.3902 | 0.3497 | 0.0004 | 0.3227 | NA | NA | 0.0154 | 0.3497 | 0.0170 | 0.3497 | NA | NA | NA | NA | NA | |
| Fiji (24) v. Unknown (2) | 1339.3128 | 0.0220 | 0.0006 | 0.0370 | -617.4173 | 0.8660 | 0.0147 | 0.0660 | 0.0163 | 0.0698 | 0.9807 | 0.6340 | 0.9825 | 0.6340 | 0.9912 | 0 |
| Indonesia (4) v. Sri_Lanka (12) | 982.6080 | 0.2048 | 0.0003 | 0.0480 | NA | NA | 0.0139 | 0.0430 | NA | NA | NA | NA | NA | NA | NA | |
| Papua_New_Guinea (8) v. Trinity_inlet (1) | 825.1671 | 0.2278 | 0.0007 | 0.6613 | 3.3463 | 1.0000 | 0.0133 | 0.5312 | 0.0147 | 0.5312 | -1.9354 | 1.0000 | -1.0364 | 1.0000 | 3.1770 | 1 |
| Arabian_Sea (15) v. Indonesia (4) | 1094.2521 | 0.1029 | 0.0003 | 0.0350 | NA | NA | 0.0132 | 0.0370 | 0.0146 | 0.0370 | NA | NA | NA | NA | NA | |
| East_Alligator_River (7) v. Wenlock_River (4) | 922.8167 | 0.0240 | 0.0003 | 0.1538 | -25.4102 | 0.2238 | 0.0111 | 0.0470 | 0.0123 | 0.0470 | 0.5873 | 0.6334 | 0.6178 | 0.6104 | 0.7592 | 0 |
| Adelaide_River (4) v. South_Alligator_River (10) | 941.5904 | 0.0380 | 0.0003 | 0.3856 | -20.2887 | 0.9076 | 0.0099 | 0.0300 | 0.0109 | 0.0301 | 0.5020 | 0.0502 | 0.5339 | 0.0502 | 0.6897 | 0 |
| Adelaide_River (4) v. Daly_River (3) | 770.7919 | 0.0899 | 0.0004 | 0.5734 | 18.8076 | 0.4316 | 0.0096 | 0.2048 | 0.0106 | 0.2048 | 2.5450 | 0.8242 | 2.3400 | 0.8242 | 1.3780 | 0 |
| Sydney_Harbour (8) v. Wenlock_River (4) | 962.0491 | 0.0220 | 0.0003 | 0.0819 | -18.8519 | 0.3387 | 0.0094 | 0.0390 | 0.0104 | 0.0390 | 0.5013 | 0.4864 | 0.5355 | 0.4734 | 0.6906 | 0 |
| Reunion (28) v. South_Alligator_River (10) | 1364.2555 | 0.0040 | 0.0002 | 0.0070 | -5.1789 | 0.9091 | 0.0094 | 0.0040 | 0.0104 | 0.0040 | 0.0895 | 0.0060 | 0.1049 | 0.0060 | 0.1785 | 0 |
| Adelaide_River (4) v. Reunion (28) | 1222.1808 | 0.1469 | 0.0003 | 0.1459 | -7.8471 | 0.8586 | 0.0089 | 0.0803 | 0.0100 | 0.0808 | 0.2403 | 0.0939 | 0.2695 | 0.0960 | 0.4110 | 0 |
| Clarence_River (11) v. Reunion (28) | 1454.0720 | 0.0020 | 0.0001 | 0.0300 | -4.6313 | 0.1269 | 0.0087 | 0.0060 | 0.0097 | 0.0060 | 0.0756 | 0.0300 | 0.0895 | 0.0240 | 0.1535 | 0 |
| East_Alligator_River (7) v. Thailand (5) | 902.3264 | 0.0270 | 0.0003 | 0.0500 | -17.0205 | 0.3666 | 0.0086 | 0.0490 | 0.0095 | 0.0490 | 0.4489 | 0.2987 | 0.4833 | 0.2667 | 0.6434 | 0 |
| Adelaide_River (4) v. Sri_Lanka (12) | 988.2344 | 0.2757 | 0.0003 | 0.0879 | -13.8484 | 0.9530 | 0.0085 | 0.0629 | 0.0095 | 0.0629 | 0.3944 | 0.0090 | 0.4279 | 0.0080 | 0.5897 | 0 |
| Daly_River (3) v. Papua_New_Guinea (8) | 874.8755 | 0.1678 | 0.0003 | 0.5355 | -75.9501 | 0.1439 | 0.0085 | 0.1738 | 0.0095 | 0.1718 | 0.8341 | 0.8611 | 0.8490 | 0.8611 | 0.9177 | 0 |
| East_Alligator_River (7) v. Reunion (28) | 1286.8956 | 0.0689 | 0.0002 | 0.0230 | -5.6705 | 0.8591 | 0.0084 | 0.0330 | 0.0094 | 0.0330 | 0.1232 | 0.0370 | 0.1428 | 0.0350 | 0.2368 | 0 |
| Sri_Lanka (12) v. Sydney_Harbour (8) | 1099.1365 | 0.0140 | 0.0001 | 0.3746 | -7.5142 | 0.7592 | 0.0080 | 0.0070 | 0.0089 | 0.0070 | 0.1768 | 0.0090 | 0.2025 | 0.0090 | 0.3223 | 0 |
| Daly_River (3) v. Seychelles (36) | 1335.6156 | 0.0440 | 0.0003 | 0.2308 | -7.8035 | 0.0631 | 0.0079 | 0.1247 | 0.0088 | 0.1250 | 0.2857 | 0.7872 | 0.3186 | 0.7597 | 0.4700 | 0 |
| East_Alligator_River (7) v. Towns_River (2) | 760.0716 | 0.4406 | 0.0005 | 0.0669 | 12.0980 | 0.9713 | 0.0079 | 0.1856 | 0.0088 | 0.1856 | 5.6343 | 0.0276 | 4.9526 | 0.0276 | 1.5958 | 0 |
| Indonesia (4) v. Unknown (2) | 667.3588 | 0.4715 | 0.0004 | 0.3916 | NA | NA | 0.0075 | 0.4715 | 0.0082 | 0.4715 | NA | NA | NA | NA | NA | |
| Adelaide_River (4) v. East_Alligator_River (7) | 856.8397 | 0.1708 | 0.0003 | 0.1449 | -35.7227 | 0.8791 | 0.0074 | 0.1239 | 0.0082 | 0.1279 | 0.6665 | 0.0929 | 0.6916 | 0.0929 | 0.8149 | 0 |
| Mozambique (11) v. Papua_New_Guinea (8) | 1043.5265 | 0.1369 | 0.0001 | 0.7403 | -8.2953 | 0.8332 | 0.0073 | 0.0330 | 0.0081 | 0.0340 | 0.2000 | 0.0709 | 0.2273 | 0.0679 | 0.3561 | 0 |
| Arabian_Sea (15) v. East_Alligator_River (7) | 1104.7522 | 0.1259 | 0.0002 | 0.0440 | -7.2099 | 0.9500 | 0.0073 | 0.0090 | 0.0081 | 0.0090 | 0.1720 | 0.0020 | 0.1968 | 0.0020 | 0.3147 | 0 |
| Seychelles (36) v. Sydney_Harbour (8) | 1400.0457 | 0.0020 | 0.0002 | 0.0140 | -4.7432 | 0.2977 | 0.0071 | 0.0110 | 0.0079 | 0.0110 | 0.0909 | 0.0350 | 0.1068 | 0.0280 | 0.1812 | 0 |
| Reunion (28) v. Thailand (5) | 1346.7053 | 0.0150 | 0.0002 | 0.1489 | -6.2375 | 0.0980 | 0.0065 | 0.1089 | 0.0072 | 0.1099 | 0.1680 | 0.5850 | 0.1931 | 0.5440 | 0.3092 | 0 |
| Reunion (28) v. Wenlock_River (4) | 1346.2016 | 0.0140 | 0.0002 | 0.4995 | -7.0671 | 0.0424 | 0.0065 | 0.1512 | 0.0072 | 0.1512 | 0.2193 | 0.8184 | 0.2486 | 0.7871 | 0.3837 | 0 |
| South_Alligator_River (10) v. Sydney_Harbour (8) | 1018.1389 | 0.0360 | 0.0001 | 0.6803 | -8.9427 | 0.7003 | 0.0063 | 0.0330 | 0.0070 | 0.0330 | 0.2163 | 0.0689 | 0.2447 | 0.0619 | 0.3790 | 0 |
| Arabian_Sea (15) v. Clarence_River (11) | 1207.4258 | 0.0200 | 0.0001 | 0.4695 | -5.5931 | 0.7213 | 0.0063 | 0.0130 | 0.0070 | 0.0130 | 0.1071 | 0.0230 | 0.1256 | 0.0220 | 0.2102 | 0 |
| Clarence_River (11) v. Sri_Lanka (12) | 1163.7924 | 0.0220 | 0.0002 | 0.0280 | -6.2141 | 0.9191 | 0.0063 | 0.0420 | 0.0070 | 0.0420 | 0.1280 | 0.0470 | 0.1490 | 0.0460 | 0.2456 | 0 |
| East_Alligator_River (7) v. South_Alligator_River (10) | 975.5935 | 0.0999 | 0.0002 | 0.3696 | -10.1834 | 0.8392 | 0.0062 | 0.0629 | 0.0069 | 0.0629 | 0.2532 | 0.0759 | 0.2831 | 0.0759 | 0.4280 | 0 |
| Sri_Lanka (12) v. Thailand (5) | 1037.7147 | 0.0609 | 0.0002 | 0.4406 | -9.7728 | 0.2797 | 0.0060 | 0.0709 | 0.0066 | 0.0709 | 0.2751 | 0.4695 | 0.3076 | 0.4246 | 0.4569 | 0 |
| Mozambique (11) v. Wenlock_River (4) | 1031.0080 | 0.1069 | 0.0003 | 0.3397 | -13.2967 | 0.0834 | 0.0060 | 0.1541 | 0.0066 | 0.1572 | 0.3917 | 0.8642 | 0.4271 | 0.8588 | 0.5883 | 0 |
| Sri_Lanka (12) v. Towns_River (2) | 859.1948 | 0.6703 | 0.0005 | 0.2198 | 26.6100 | 0.9777 | 0.0059 | 0.2900 | 0.0065 | 0.2900 | 1.7219 | 0.0432 | 1.6385 | 0.0432 | 1.2346 | 0 |
| East_Alligator_River (7) v. Sydney_Harbour (8) | 932.6645 | 0.1908 | 0.0002 | 0.3337 | -11.1195 | 0.9461 | 0.0057 | 0.0609 | 0.0064 | 0.0609 | 0.2909 | 0.0589 | 0.3231 | 0.0599 | 0.4757 | 0 |
| Arabian_Sea (15) v. Mozambique (11) | 1207.9446 | 0.0180 | 0.0001 | 0.1768 | -5.8082 | 0.9371 | 0.0057 | 0.0110 | 0.0064 | 0.0110 | 0.1127 | 0.0050 | 0.1315 | 0.0060 | 0.2195 | 0 |
| South_Africa (23) v. Towns_River (2) | 1027.9503 | 0.7682 | 0.0005 | 0.1199 | -118.5844 | 0.9803 | 0.0055 | 0.1743 | 0.0061 | 0.1743 | 0.9082 | 0.0319 | 0.9165 | 0.0319 | 0.9562 | 0 |
| Mozambique (11) v. Reunion (28) | 1367.2751 | 0.0080 | 0.0001 | 0.2677 | -4.8361 | 0.4216 | 0.0054 | 0.0320 | 0.0061 | 0.0330 | 0.0789 | 0.0210 | 0.0929 | 0.0180 | 0.1592 | 0 |
| Caribbean_Sea (2) v. Gulf_of_Mexico (40) | 911.5208 | 0.3417 | 0.0002 | 0.4266 | 19.0179 | 0.4043 | 0.0053 | 0.2949 | 0.0059 | 0.2943 | 3.1415 | 0.6170 | 2.9350 | 0.6099 | 1.4672 | 0 |
| Reunion (28) v. Sydney_Harbour (8) | 1294.5729 | 0.0430 | 0.0001 | 0.1399 | -5.3241 | 0.2318 | 0.0053 | 0.0609 | 0.0059 | 0.0609 | 0.1046 | 0.1968 | 0.1222 | 0.1798 | 0.2053 | 0 |

Table 38: Pairwise Fst for 1849 DArTcap loci between the all sites, but with a subset of Australia
*(continued)*

| | Chi2 | Chi2.p.val | D | D.p.val | Fis | Fis.p.val | Fst | Fst.p.val | F’st | F’st.p.val | Gst | Gst.p.val | G’st | G’st.p.val | G”st | G”st |
|---|---|---|---|---|---|---|---|---|---|---|---|---|---|---|---|---|
| Adelaide_River (4) v. Clarence_River (11) | 941.4020 | 0.4066 | 0.0003 | 0.0939 | -14.6119 | 0.9790 | 0.0052 | 0.2657 | 0.0058 | 0.2657 | 0.4116 | 0.0310 | 0.4458 | 0.0320 | 0.6074 | 0 |
| Brazil (52) v. Western_North_Atlantic (8) | 1078.0912 | 0.6563 | 0.0000 | 0.5255 | -8.6921 | 0.9870 | 0.0052 | 0.0410 | 0.0056 | 0.0410 | 0.1440 | 0.0190 | 0.1594 | 0.0190 | 0.2652 | 0 |
| East_Alligator_River (7) v. Mozambique (11) | 1030.0325 | 0.1499 | 0.0002 | 0.2228 | -8.9136 | 0.7942 | 0.0052 | 0.0649 | 0.0058 | 0.0639 | 0.2245 | 0.1758 | 0.2532 | 0.1798 | 0.3901 | 0 |
| Reunion (28) v. Sri_Lanka (12) | 1390.3773 | 0.0070 | 0.0001 | 0.0939 | -4.5725 | 0.6923 | 0.0051 | 0.0170 | 0.0057 | 0.0170 | 0.0691 | 0.0340 | 0.0817 | 0.0320 | 0.1410 | 0 |
| Reunion (28) v. Seychelles (36) | 1571.8855 | 0.0010 | 0.0001 | 0.0050 | -3.5600 | 0.9990 | 0.0051 | 0.0010 | 0.0056 | 0.0010 | 0.0310 | 0.0010 | 0.0371 | 0.0010 | 0.0661 | 0 |
| Clarence_River (11) v. East_Alligator_River (7) | 996.9789 | 0.2727 | 0.0002 | 0.2438 | -8.5864 | 0.9820 | 0.0050 | 0.1778 | 0.0055 | 0.1788 | 0.2148 | 0.0639 | 0.2434 | 0.0729 | 0.3771 | 0 |
| Clarence_River (11) v. South_Africa (23) | 1250.3632 | 0.0400 | 0.0001 | 0.1469 | -4.9951 | 0.1229 | 0.0048 | 0.0270 | 0.0055 | 0.0270 | 0.0838 | 0.1728 | 0.0989 | 0.1518 | 0.1686 | 0 |
| South_Alligator_River (10) v. Wenlock_River (4) | 969.7762 | 0.0599 | 0.0002 | 0.8402 | -16.3397 | 0.0671 | 0.0048 | 0.1852 | 0.0053 | 0.1852 | 0.4447 | 0.8519 | 0.4792 | 0.8448 | 0.6395 | 0 |
| South_Africa (23) v. South_Alligator_River (10) | 1209.3466 | 0.1548 | 0.0001 | 0.2957 | -5.4308 | 0.9191 | 0.0047 | 0.0410 | 0.0052 | 0.0420 | 0.0962 | 0.0959 | 0.1125 | 0.0959 | 0.1904 | 0 |
| Daly_River (3) v. Sri_Lanka (12) | 961.0287 | 0.3117 | 0.0003 | 0.5015 | -21.5636 | 0.1465 | 0.0045 | 0.2503 | 0.0050 | 0.2503 | 0.5599 | 0.8535 | 0.5916 | 0.8555 | 0.7382 | 0 |
| Papua_New_Guinea (8) v. Reunion (28) | 1210.6043 | 0.2228 | 0.0001 | 0.2677 | -5.5034 | 0.6444 | 0.0045 | 0.1039 | 0.0050 | 0.1039 | 0.1073 | 0.1359 | 0.1250 | 0.1319 | 0.2098 | 0 |
| Darwin_Coastal (2) v. Reunion (28) | 1230.3032 | 0.1578 | 0.0004 | 0.7063 | -33.5158 | 0.3099 | 0.0044 | 0.2821 | 0.0050 | 0.2559 | 0.7277 | 0.6901 | 0.7499 | 0.6901 | 0.8553 | 0 |
| Mozambique (11) v. Towns_River (2) | 895.5756 | 0.5105 | 0.0006 | 0.0959 | 22.5004 | 0.6591 | 0.0044 | 0.2655 | 0.0049 | 0.2655 | 1.9386 | 0.3788 | 1.8244 | 0.3788 | 1.2805 | 0 |
| Reunion (28) v. Towns_River (2) | 1183.6801 | 0.2657 | 0.0005 | 0.1289 | -54.0274 | 0.9062 | 0.0044 | 0.3009 | 0.0049 | 0.3009 | 0.8160 | 0.0973 | 0.8315 | 0.0973 | 0.9072 | 0 |
| Adelaide_River (4) v. Seychelles (36) | 1216.3055 | 0.3546 | 0.0002 | 0.3706 | -6.6652 | 0.9221 | 0.0043 | 0.1878 | 0.0048 | 0.1878 | 0.2046 | 0.0699 | 0.2315 | 0.0769 | 0.3621 | 0 |
| Reunion (28) v. South_Africa (23) | 1472.1707 | 0.0050 | 0.0001 | 0.0020 | -3.8544 | 0.9640 | 0.0043 | 0.0080 | 0.0049 | 0.0080 | 0.0408 | 0.0080 | 0.0486 | 0.0080 | 0.0859 | 0 |
| Clarence_River (11) v. Daly_River (3) | 930.0515 | 0.3766 | 0.0003 | 0.4905 | -22.8094 | 0.4525 | 0.0043 | 0.3463 | 0.0048 | 0.3463 | 0.5795 | 0.5325 | 0.6112 | 0.5325 | 0.7538 | 0 |
| Arabian_Sea (15) v. Papua_New_Guinea (8) | 1089.1414 | 0.1978 | 0.0001 | 0.4316 | -6.8242 | 0.8721 | 0.0042 | 0.0589 | 0.0047 | 0.0589 | 0.1507 | 0.0360 | 0.1736 | 0.0350 | 0.2819 | 0 |
| South_Africa (23) v. Sydney_Harbour (8) | 1180.4263 | 0.2168 | 0.0002 | 0.0629 | -5.7041 | 0.2408 | 0.0042 | 0.0639 | 0.0047 | 0.0639 | 0.1160 | 0.3387 | 0.1351 | 0.3197 | 0.2250 | 0 |
| Mozambique (11) v. Sri_Lanka (12) | 1130.5400 | 0.0819 | 0.0002 | 0.0869 | -6.5048 | 0.8641 | 0.0041 | 0.0809 | 0.0046 | 0.0829 | 0.1345 | 0.0719 | 0.1558 | 0.0699 | 0.2559 | 0 |
| Mozambique (11) v. Seychelles (36) | 1390.0386 | 0.0140 | 0.0001 | 0.1888 | -4.3710 | 0.3556 | 0.0041 | 0.0300 | 0.0046 | 0.0300 | 0.0671 | 0.0150 | 0.0794 | 0.0120 | 0.1372 | 0 |
| Seychelles (36) v. South_Alligator_River (10) | 1305.5406 | 0.0609 | 0.0001 | 0.0949 | -4.6829 | 0.8661 | 0.0041 | 0.0340 | 0.0045 | 0.0340 | 0.0749 | 0.0260 | 0.0881 | 0.0260 | 0.1517 | 0 |
| South_Alligator_River (10) v. Thailand (5) | 983.2481 | 0.0150 | 0.0003 | 0.0919 | -12.3873 | 0.0929 | 0.0040 | 0.1738 | 0.0045 | 0.1748 | 0.3381 | 0.6873 | 0.3721 | 0.6573 | 0.5308 | 0 |
| Daly_River (3) v. Thailand (5) | 763.8674 | 0.2288 | 0.0003 | 0.5375 | 46.6455 | 0.5788 | 0.0039 | 0.3835 | 0.0043 | 0.3835 | 1.3796 | 0.4751 | 1.3362 | 0.4629 | 1.1413 | 0 |
| Clarence_River (11) v. Seychelles (36) | 1411.9116 | 0.0040 | 0.0001 | 0.1169 | -4.2002 | 0.0639 | 0.0038 | 0.0480 | 0.0043 | 0.0480 | 0.0629 | 0.1948 | 0.0747 | 0.1578 | 0.1295 | 0 |
| Clarence_River (11) v. Mozambique (11) | 1124.7493 | 0.1159 | 0.0001 | 0.4705 | -6.5442 | 0.8362 | 0.0038 | 0.1479 | 0.0043 | 0.1469 | 0.1404 | 0.1329 | 0.1628 | 0.1329 | 0.2659 | 0 |
| Brazil (52) v. Gulf_of_Mexico (40) | 1437.4854 | 0.0010 | 0.0000 | 0.1738 | -5.5718 | 1.0000 | 0.0038 | 0.0010 | 0.0041 | 0.0010 | 0.0328 | 0.0010 | 0.0373 | 0.0010 | 0.0679 | 0 |
| Adelaide_River (4) v. Papua_New_Guinea (8) | 850.1131 | 0.4146 | 0.0003 | 0.1578 | -26.3131 | 0.8939 | 0.0037 | 0.2727 | 0.0041 | 0.2727 | 0.5825 | 0.1231 | 0.6117 | 0.1221 | 0.7546 | 0 |
| Mozambique (11) v. South_Africa (23) | 1238.8978 | 0.1728 | 0.0001 | 0.5335 | -5.0399 | 0.4366 | 0.0037 | 0.0569 | 0.0041 | 0.0589 | 0.0860 | 0.0699 | 0.1011 | 0.0669 | 0.1722 | 0 |
| Seychelles (36) v. Thailand (5) | 1283.7125 | 0.0829 | 0.0002 | 0.0679 | -5.5690 | 0.0919 | 0.0036 | 0.1808 | 0.0040 | 0.1818 | 0.1468 | 0.6863 | 0.1698 | 0.6284 | 0.2761 | 0 |
| Arabian_Sea (15) v. Sydney_Harbour (8) | 1135.0700 | 0.0909 | 0.0001 | 0.5415 | -6.5343 | 0.5744 | 0.0036 | 0.0939 | 0.0040 | 0.0939 | 0.1451 | 0.1239 | 0.1677 | 0.1139 | 0.2732 | 0 |
| Arabian_Sea (15) v. South_Africa (23) | 1320.0624 | 0.0220 | 0.0001 | 0.2258 | -4.4496 | 0.6823 | 0.0035 | 0.0230 | 0.0039 | 0.0230 | 0.0624 | 0.0480 | 0.0740 | 0.0490 | 0.1284 | 0 |
| Arabian_Sea (15) v. South_Alligator_River (10) | 1115.0126 | 0.1938 | 0.0001 | 0.0989 | -6.3538 | 0.9441 | 0.0034 | 0.0909 | 0.0038 | 0.0909 | 0.1248 | 0.0569 | 0.1447 | 0.0589 | 0.2396 | 0 |
| Adelaide_River (4) v. Arabian_Sea (15) | 993.5807 | 0.6264 | 0.0002 | 0.6264 | -11.2483 | 0.9449 | 0.0034 | 0.2593 | 0.0038 | 0.2595 | 0.3324 | 0.0541 | 0.3652 | 0.0571 | 0.5236 | 0 |
| Adelaide_River (4) v. Thailand (5) | 781.4286 | 0.2188 | 0.0004 | 0.0220 | -195.7076 | 0.9401 | 0.0033 | 0.3377 | 0.0037 | 0.3377 | 0.9266 | 0.0719 | 0.9334 | 0.0719 | 0.9654 | 0 |
| East_Alligator_River (7) v. Seychelles (36) | 1195.8916 | 0.5554 | 0.0001 | 0.3237 | -5.1411 | 0.8711 | 0.0032 | 0.1279 | 0.0035 | 0.1279 | 0.1061 | 0.0669 | 0.1236 | 0.0689 | 0.2077 | 0 |
| South_Africa (23) v. Sri_Lanka (12) | 1252.2227 | 0.0989 | 0.0001 | 0.1938 | -4.8724 | 0.7183 | 0.0032 | 0.0589 | 0.0035 | 0.0599 | 0.0770 | 0.1229 | 0.0908 | 0.1179 | 0.1558 | 0 |
| Clarence_River (11) v. Sydney_Harbour (8) | 1006.3243 | 0.3387 | 0.0002 | 0.0400 | -7.7431 | 0.8202 | 0.0031 | 0.2587 | 0.0035 | 0.2587 | 0.1845 | 0.1978 | 0.2113 | 0.2028 | 0.3341 | 0 |
| Adelaide_River (4) v. Mozambique (11) | 946.6996 | 0.4605 | 0.0003 | 0.1898 | -16.2080 | 0.5687 | 0.0030 | 0.3086 | 0.0035 | 0.3001 | 0.4403 | 0.5100 | 0.4737 | 0.5268 | 0.6346 | 0 |
| Seychelles (36) v. Wenlock_River (4) | 1327.9458 | 0.0400 | 0.0002 | 0.8771 | -6.1447 | 0.0170 | 0.0030 | 0.2657 | 0.0033 | 0.2657 | 0.1896 | 0.9221 | 0.2166 | 0.9031 | 0.3414 | 0 |
| Arabian_Sea (15) v. Reunion (28) | 1444.5863 | 0.0050 | 0.0001 | 0.1469 | -4.1460 | 0.3976 | 0.0030 | 0.0799 | 0.0033 | 0.0799 | 0.0541 | 0.1309 | 0.0642 | 0.1249 | 0.1122 | 0 |
| Papua_New_Guinea (8) v. Thailand (5) | 910.1297 | 0.1439 | 0.0002 | 0.7672 | -14.2907 | 0.1918 | 0.0029 | 0.3267 | 0.0032 | 0.3277 | 0.3904 | 0.6573 | 0.4252 | 0.6364 | 0.5866 | 0 |
| Sydney_Harbour (8) v. Thailand (5) | 925.8270 | 0.0949 | 0.0002 | 0.4525 | -13.6220 | 0.3506 | 0.0027 | 0.2807 | 0.0030 | 0.2807 | 0.3795 | 0.5225 | 0.4147 | 0.5065 | 0.5758 | 0 |
| Arabian_Sea (15) v. Darwin_Coastal (2) | 1005.1362 | 0.3586 | 0.0004 | 0.5405 | 111.3996 | 0.6475 | 0.0026 | 0.3474 | 0.0029 | 0.3474 | 1.1179 | 0.3391 | 1.1060 | 0.3391 | 1.0501 | 0 |
| East_Alligator_River (7) v. Trinity_inlet (1) | 720.2502 | 0.2248 | 0.0007 | 0.6064 | 3.1924 | 1.0000 | 0.0026 | 1.0000 | 0.0029 | 1.0000 | -1.7843 | 1.0000 | -0.8893 | 1.0000 | 3.4089 | 1 |
| Adelaide_River (4) v. Trinity_inlet (1) | 666.6240 | 0.1878 | 0.0006 | 1.0000 | 2.5850 | 1.0000 | 0.0026 | 0.5040 | 0.0028 | 0.5040 | -1.4523 | 1.0000 | -0.4728 | 1.0000 | 4.2563 | 1 |
| Clarence_River (11) v. South_Alligator_River (10) | 1061.2904 | 0.1259 | 0.0001 | 0.4026 | -7.2648 | 0.8482 | 0.0025 | 0.2378 | 0.0028 | 0.2378 | 0.1570 | 0.2118 | 0.1807 | 0.2158 | 0.2919 | 0 |
| Mozambique (11) v. South_Alligator_River (10) | 1064.2663 | 0.2298 | 0.0001 | 0.4675 | -7.4770 | 0.7512 | 0.0024 | 0.2148 | 0.0027 | 0.2168 | 0.1636 | 0.2248 | 0.1875 | 0.2248 | 0.3017 | 0 |
| South_Africa (23) v. Thailand (5) | 1203.2235 | 0.0519 | 0.0002 | 0.7353 | -6.7062 | 0.0669 | 0.0024 | 0.2478 | 0.0027 | 0.2478 | 0.1820 | 0.8142 | 0.2083 | 0.7652 | 0.3302 | 0 |
| Daly_River (3) v. Mozambique (11) | 949.4926 | 0.3996 | 0.0003 | 0.7712 | -26.0216 | 0.0914 | 0.0023 | 0.3981 | 0.0026 | 0.4020 | 0.6137 | 0.9173 | 0.6433 | 0.9173 | 0.7789 | 0 |
| Darwin_Coastal (2) v. Towns_River (2) | 576.4500 | 0.6364 | 0.0004 | 0.6364 | NA | NA | 0.0022 | 0.6364 | 0.0024 | 0.4835 | NA | NA | NA | NA | NA | |
| Sri_Lanka (12) v. Trinity_inlet (1) | 913.8882 | 0.3706 | 0.0007 | 0.5095 | 3.8034 | 1.0000 | 0.0020 | 0.6017 | 0.0022 | 0.6017 | -2.4250 | 1.0000 | -1.5183 | 1.0000 | 2.7672 | 1 |

Table 38: Pairwise Fst for 1849 DArTcap loci between the all sites, but with a subset of Australia
*(continued)*

| | Chi2 | Chi2.p.val | D | D.p.val | Fis | Fis.p.val | Fst | Fst.p.val | F’st | F’st.p.val | Gst | Gst.p.val | G’st | G’st.p.val | G”st | G”st |
|---|---|---|---|---|---|---|---|---|---|---|---|---|---|---|---|---|
| East_Alligator_River (7) v. Sri_Lanka (12) | 976.7977 | 0.5315 | 0.0002 | 0.2258 | -8.4117 | 0.7602 | 0.0019 | 0.2897 | 0.0021 | 0.2897 | 0.2040 | 0.2008 | 0.2311 | 0.2008 | 0.3614 | 0 |
| Seychelles (36) v. Sri_Lanka (12) | 1345.7969 | 0.0480 | 0.0001 | 0.0559 | -4.2228 | 0.6104 | 0.0019 | 0.1508 | 0.0021 | 0.1508 | 0.0587 | 0.1558 | 0.0695 | 0.1489 | 0.1211 | 0 |
| Darwin_Coastal (2) v. South_Alligator_River (10) | 878.4396 | 0.1818 | 0.0004 | 0.6284 | 20.5747 | 0.5574 | 0.0018 | 0.4458 | 0.0020 | 0.4639 | 2.1530 | 0.4962 | 2.0093 | 0.5100 | 1.3201 | 0 |
| Papua_New_Guinea (8) v. Sydney_Harbour (8) | 931.5514 | 0.4366 | 0.0002 | 0.3616 | -9.9910 | 0.6553 | 0.0018 | 0.3097 | 0.0020 | 0.3087 | 0.2538 | 0.3237 | 0.2844 | 0.3227 | 0.4292 | 0 |
| Papua_New_Guinea (8) v. Sri_Lanka (12) | 989.6342 | 0.6074 | 0.0001 | 0.7303 | -7.7814 | 0.6913 | 0.0017 | 0.2957 | 0.0019 | 0.2957 | 0.1787 | 0.2677 | 0.2040 | 0.2717 | 0.3247 | 0 |
| Papua_New_Guinea (8) v. Seychelles (36) | 1161.5383 | 0.7343 | 0.0001 | 0.5824 | -4.9730 | 0.7103 | 0.0017 | 0.2577 | 0.0019 | 0.2577 | 0.0921 | 0.2268 | 0.1079 | 0.2218 | 0.1832 | 0 |
| Mozambique (11) v. Sydney_Harbour (8) | 1026.9265 | 0.3387 | 0.0002 | 0.5954 | -8.0200 | 0.3706 | 0.0015 | 0.3407 | 0.0016 | 0.3437 | 0.1920 | 0.4975 | 0.2189 | 0.4935 | 0.3448 | 0 |
| Daly_River (3) v. Sydney_Harbour (8) | 864.6303 | 0.2947 | 0.0003 | 0.7153 | -62.0937 | 0.1833 | 0.0014 | 0.4328 | 0.0015 | 0.4257 | 0.8033 | 0.8452 | 0.8208 | 0.8452 | 0.9006 | 0 |
| Darwin_Coastal (2) v. Seychelles (36) | 1268.7705 | 0.1409 | 0.0003 | 0.9061 | -19.4939 | 0.5145 | 0.0014 | 0.4030 | 0.0015 | 0.4075 | 0.6036 | 0.4882 | 0.6327 | 0.4910 | 0.7710 | 0 |
| Gulf_of_Mexico (40) v. Western_North_Atlantic (8) | 1031.9298 | 0.1209 | 0.0000 | 0.4056 | -11.8541 | 0.3487 | 0.0013 | 0.3317 | 0.0014 | 0.3357 | 0.1902 | 0.7433 | 0.2081 | 0.7433 | 0.3347 | 0 |
| Daly_River (3) v. South_Africa (23) | 1101.0772 | 0.5564 | 0.0002 | 0.9381 | -10.7395 | 0.0394 | 0.0012 | 0.3963 | 0.0014 | 0.3977 | 0.3634 | 0.9565 | 0.3980 | 0.9534 | 0.5585 | 0 |
| Arabian_Sea (15) v. Seychelles (36) | 1397.9962 | 0.0220 | 0.0001 | 0.1948 | -3.9018 | 0.3017 | 0.0012 | 0.2338 | 0.0013 | 0.2338 | 0.0463 | 0.2897 | 0.0552 | 0.2807 | 0.0970 | 0 |
| Daly_River (3) v. Reunion (28) | 1221.9850 | 0.1578 | 0.0002 | 0.8132 | -9.4452 | 0.0557 | 0.0012 | 0.3774 | 0.0013 | 0.3715 | 0.3277 | 0.9474 | 0.3615 | 0.9443 | 0.5191 | 0 |
| South_Alligator_River (10) v. Sri_Lanka (12) | 1038.7596 | 0.3407 | 0.0001 | 0.2278 | -7.1773 | 0.7373 | 0.0012 | 0.3287 | 0.0013 | 0.3287 | 0.1492 | 0.2927 | 0.1716 | 0.3017 | 0.2791 | 0 |
| Sydney_Harbour (8) v. Towns_River (2) | 805.0978 | 0.5055 | 0.0005 | 0.2977 | 14.8429 | 0.9618 | 0.0011 | 0.4925 | 0.0013 | 0.4925 | 3.2821 | 0.0688 | 2.9674 | 0.1116 | 1.4594 | 0 |
| Clarence_River (11) v. Thailand (5) | 986.7409 | 0.2378 | 0.0002 | 0.3736 | -9.8864 | 0.5285 | 0.0010 | 0.4545 | 0.0011 | 0.4545 | 0.2813 | 0.4745 | 0.3147 | 0.4665 | 0.4651 | 0 |
| Arabian_Sea (15) v. Daly_River (3) | 1027.3070 | 0.3107 | 0.0003 | 0.7193 | -15.4736 | 0.1748 | 0.0009 | 0.4188 | 0.0010 | 0.4188 | 0.4663 | 0.8425 | 0.5007 | 0.8445 | 0.6595 | 0 |
| Arabian_Sea (15) v. Wenlock_River (4) | 1051.2558 | 0.3437 | 0.0002 | 0.7922 | -9.8805 | 0.1189 | 0.0009 | 0.4316 | 0.0010 | 0.4316 | 0.3017 | 0.8511 | 0.3352 | 0.8452 | 0.4893 | 0 |
| Brazil (52) v. Caribbean_Sea (2) | 1012.4079 | 0.6693 | 0.0002 | 0.6314 | 43.6705 | 0.8585 | 0.0008 | 0.3664 | 0.0009 | 0.3664 | 1.4301 | 0.1538 | 1.3964 | 0.1538 | 1.1631 | 0 |
| Clarence_River (11) v. Papua_New_Guinea (8) | 974.7991 | 0.4995 | 0.0002 | 0.3876 | -7.9186 | 0.8941 | 0.0008 | 0.4016 | 0.0009 | 0.4016 | 0.1865 | 0.2697 | 0.2131 | 0.2857 | 0.3368 | 0 |
| Seychelles (36) v. South_Africa (23) | 1344.5068 | 0.3107 | 0.0001 | 0.4236 | -3.5881 | 0.4885 | 0.0005 | 0.3117 | 0.0006 | 0.3107 | 0.0327 | 0.2697 | 0.0391 | 0.2707 | 0.0695 | 0 |
| South_Africa (23) v. Wenlock_River (4) | 1186.3131 | 0.1848 | 0.0002 | 0.7163 | -7.5866 | 0.0190 | 0.0005 | 0.4314 | 0.0005 | 0.4314 | 0.2349 | 0.9520 | 0.2651 | 0.9439 | 0.4049 | 0 |
| Darwin_Coastal (2) v. Sydney_Harbour (8) | 827.2518 | 0.3566 | 0.0004 | 0.9550 | 16.9535 | 0.5166 | 0.0005 | 0.4439 | 0.0005 | 0.3963 | 2.6332 | 0.4983 | 2.4143 | 0.4983 | 1.3893 | 0 |
| East_Alligator_River (7) v. South_Africa (23) | 1086.5319 | 0.8432 | 0.0001 | 0.6893 | -6.0880 | 0.7692 | 0.0003 | 0.4246 | 0.0004 | 0.4236 | 0.1323 | 0.4176 | 0.1529 | 0.4256 | 0.2519 | 0 |
| Clarence_River (11) v. Towns_River (2) | 826.5694 | 0.6853 | 0.0005 | 0.2717 | 24.3432 | 1.0000 | 0.0003 | 0.4728 | 0.0003 | 0.4728 | 1.8187 | 0.0108 | 1.7205 | 0.0108 | 1.2556 | 0 |
| Papua_New_Guinea (8) v. South_Alligator_River (10) | 943.7299 | 0.4645 | 0.0002 | 0.5524 | -9.3035 | 0.3846 | 0.0002 | 0.4625 | 0.0002 | 0.4625 | 0.2199 | 0.5105 | 0.2479 | 0.5045 | 0.3835 | 0 |
| Papua_New_Guinea (8) v. South_Africa (23) | 1107.5972 | 0.7433 | 0.0001 | 0.3776 | -5.8132 | 0.5964 | 0.0002 | 0.4525 | 0.0002 | 0.4525 | 0.1159 | 0.5025 | 0.1347 | 0.5105 | 0.2246 | 0 |
| Seychelles (36) v. Towns_River (2) | 1124.2415 | 0.6953 | 0.0005 | 0.1658 | -24.8041 | 0.9673 | 0.0000 | 0.4602 | 0.0000 | 0.4602 | 0.6643 | 0.0327 | 0.6898 | 0.0299 | 0.8136 | 0 |
| Clarence_River (11) v. Darwin_Coastal (2) | 880.5217 | 0.4296 | 0.0004 | 0.5175 | 33.2906 | 0.9103 | 0.0000 | 0.4833 | 0.0000 | 0.4833 | 1.5119 | 0.1539 | 1.4529 | 0.1539 | 1.1803 | 0 |
| Arabian_Sea (15) v. Thailand (5) | 1028.5982 | 0.4076 | 0.0002 | 0.4545 | -8.3948 | 0.1960 | 0.0000 | 0.4860 | 0.0000 | 0.4860 | 0.2323 | 0.7830 | 0.2625 | 0.7750 | 0.4015 | 0 |
| Clarence_River (11) v. Wenlock_River (4) | 977.8491 | 0.3636 | 0.0002 | 0.8032 | -12.0893 | 0.3556 | 0.0000 | 0.4935 | -0.0001 | 0.4935 | 0.3630 | 0.6204 | 0.3985 | 0.6214 | 0.5587 | 0 |
| Adelaide_River (4) v. South_Africa (23) | 1029.3770 | 0.9431 | 0.0002 | 0.6813 | -8.6341 | 0.8164 | -0.0003 | 0.5045 | -0.0003 | 0.5045 | 0.2601 | 0.2247 | 0.2901 | 0.2317 | 0.4367 | 0 |
| Darwin_Coastal (2) v. Papua_New_Guinea (8) | 789.9184 | 0.4545 | 0.0003 | 0.8871 | 16.0588 | 0.5132 | -0.0003 | 0.4844 | -0.0003 | 0.4844 | 2.9094 | 0.5921 | 2.6527 | 0.5921 | 1.4227 | 0 |
| Thailand (5) v. Wenlock_River (4) | 790.7350 | 0.4256 | 0.0004 | 0.1079 | -63.2605 | 0.5215 | -0.0003 | 0.4895 | -0.0003 | 0.4895 | 0.8020 | 0.4915 | 0.8198 | 0.4915 | 0.9000 | 0 |
| Mozambique (11) v. Thailand (5) | 954.9798 | 0.4545 | 0.0002 | 0.7103 | -10.8081 | 0.0796 | -0.0003 | 0.5030 | -0.0004 | 0.5020 | 0.3024 | 0.9113 | 0.3359 | 0.9083 | 0.4901 | 0 |
| Arabian_Sea (15) v. Sri_Lanka (12) | 1114.7194 | 0.6723 | 0.0001 | 0.3257 | -5.5370 | 0.5694 | -0.0003 | 0.5654 | -0.0004 | 0.5654 | 0.0983 | 0.5395 | 0.1151 | 0.5365 | 0.1943 | 0 |
| Thailand (5) v. Towns_River (2) | 699.8974 | 0.5435 | 0.0005 | 0.1499 | 9.4260 | 1.0000 | -0.0005 | 0.5367 | -0.0005 | 0.5367 | 29.2654 | 0.0672 | 24.4901 | 0.0672 | 1.7761 | 0 |
| Sri_Lanka (12) v. Wenlock_River (4) | 949.1637 | 0.5405 | 0.0002 | 0.6673 | -12.2126 | 0.1040 | -0.0011 | 0.5460 | -0.0012 | 0.5460 | 0.3586 | 0.9060 | 0.3932 | 0.9090 | 0.5534 | 0 |
| Daly_River (3) v. Towns_River (2) | 635.9042 | 0.4865 | 0.0006 | 0.3966 | 5.6817 | 0.8871 | -0.0015 | 0.4865 | -0.0017 | 0.4865 | -3.5528 | 0.1009 | -2.5127 | 0.1009 | 2.3760 | 0 |
| East_Alligator_River (7) v. Papua_New_Guinea (8) | 891.1094 | 0.7732 | 0.0001 | 0.9291 | -11.1798 | 0.3916 | -0.0017 | 0.6653 | -0.0018 | 0.6653 | 0.2883 | 0.6454 | 0.3197 | 0.6454 | 0.4719 | 0 |
| Reunion (28) v. Trinity_inlet (1) | 1235.6088 | 0.1958 | 0.0007 | 0.7193 | 4.8126 | 1.0000 | -0.0018 | 0.5333 | -0.0020 | 0.5333 | -5.4582 | 1.0000 | -4.1318 | 1.0000 | 2.1511 | 1 |
| South_Alligator_River (10) v. Trinity_inlet (1) | 827.4026 | 0.2567 | 0.0007 | 0.7193 | 3.5281 | 1.0000 | -0.0019 | 1.0000 | -0.0021 | 1.0000 | -2.0482 | 1.0000 | -1.1634 | 1.0000 | 3.0639 | 1 |
| Adelaide_River (4) v. Wenlock_River (4) | 756.8356 | 0.5884 | 0.0003 | 0.8541 | 60.9724 | 0.3724 | -0.0023 | 0.6134 | -0.0026 | 0.6004 | 1.2906 | 0.6556 | 1.2598 | 0.6556 | 1.1134 | 0 |
| South_Alligator_River (10) v. Towns_River (2) | 823.2817 | 0.4036 | 0.0004 | 0.4276 | 17.2796 | 0.8650 | -0.0026 | 0.5903 | -0.0028 | 0.5903 | 2.6859 | 0.0802 | 2.4692 | 0.0802 | 1.3986 | 0 |
| Darwin_Coastal (2) v. South_Africa (23) | 1088.5364 | 0.5954 | 0.0003 | 0.9730 | -47.5421 | 0.3403 | -0.0030 | 0.5766 | -0.0034 | 0.5846 | 0.7928 | 0.6758 | 0.8104 | 0.6758 | 0.8943 | 0 |
| Adelaide_River (4) v. Sydney_Harbour (8) | 834.4309 | 0.7363 | 0.0002 | 0.8082 | -24.8980 | 0.8938 | -0.0031 | 0.6613 | -0.0034 | 0.6572 | 0.5674 | 0.1800 | 0.5975 | 0.1891 | 0.7432 | 0 |
| Papua_New_Guinea (8) v. Wenlock_River (4) | 857.8924 | 0.6174 | 0.0002 | 0.6943 | -20.1621 | 0.0290 | -0.0032 | 0.6803 | -0.0036 | 0.6803 | 0.5130 | 0.9590 | 0.5459 | 0.9580 | 0.6999 | 0 |
| Papua_New_Guinea (8) v. Towns_River (2) | 750.3967 | 0.7173 | 0.0004 | 0.4026 | 14.0403 | 0.9477 | -0.0033 | 0.5678 | -0.0036 | 0.5678 | 3.7850 | 0.0753 | 3.3999 | 0.0753 | 1.5016 | 0 |
| Seychelles (36) v. Trinity_inlet (1) | 1222.1414 | 0.2717 | 0.0007 | 0.6384 | 5.2967 | 1.0000 | -0.0034 | 1.0000 | -0.0037 | 1.0000 | -11.2934 | 1.0000 | -8.9809 | 1.0000 | 1.9696 | 1 |
| Adelaide_River (4) v. Unknown (2) | 680.5192 | 0.3257 | 0.0004 | 0.8032 | 7.4697 | 0.3084 | -0.0034 | 0.5786 | -0.0038 | 0.5786 | -7.6348 | 0.8000 | -6.0014 | 0.8000 | 2.0553 | 0 |
| Arabian_Sea (15) v. Towns_River (2) | 935.7404 | 0.6953 | 0.0005 | 0.1998 | 53.6252 | 0.9933 | -0.0036 | 0.5948 | -0.0039 | 0.5948 | 1.2749 | 0.0189 | 1.2464 | 0.0189 | 1.1083 | 0 |
| Darwin_Coastal (2) v. Sri_Lanka (12) | 879.6630 | 0.5954 | 0.0003 | 0.7383 | 37.5049 | 0.3973 | -0.0037 | 0.6048 | -0.0042 | 0.6048 | 1.4461 | 0.5728 | 1.3969 | 0.5605 | 1.1623 | 0 |

Table 38: Pairwise Fst for 1849 DArTcap loci between the all sites, but with a subset of Australia
*(continued)*

| | Chi2 | Chi2.p.val | D | D.p.val | Fis | Fis.p.val | Fst | Fst.p.val | F'st | F'st.p.val | Gst | Gst.p.val | G'st | G'st.p.val | G"st | G"st |
|---|---|---|---|---|---|---|---|---|---|---|---|---|---|---|---|---|
| Adelaide_River (4) v. Towns_River (2) | 628.5349 | 0.7313 | 0.0004 | 0.7483 | 6.9651 | 1.0000 | -0.0044 | 0.5927 | -0.0048 | 0.5927 | -5.2701 | 0.1431 | -4.0376 | 0.1431 | 2.1798 | 0 |
| Darwin_Coastal (2) v. East_Alligator_River (7) | 750.0513 | 0.5764 | 0.0003 | 0.7832 | 13.5712 | 0.5058 | -0.0046 | 0.6767 | -0.0051 | 0.6923 | 4.0794 | 0.6773 | 3.6425 | 0.6773 | 1.5202 | 0 |
| Adelaide_River (4) v. Darwin_Coastal (2) | 673.5342 | 0.2727 | 0.0003 | 1.0000 | 7.5064 | 0.2357 | -0.0047 | 0.4874 | -0.0052 | 0.4874 | -7.8941 | 0.8750 | -6.2094 | 0.8750 | 2.0457 | 1 |
| Caribbean_Sea (2) v. Western_North_Atlantic (8) | 508.1961 | 0.7443 | 0.0002 | 0.7502 | 8.5480 | 0.7754 | -0.0053 | 0.7561 | -0.0058 | 0.6254 | -4.1316 | 0.3832 | -3.3410 | 0.3832 | 2.3862 | 0 |
| Darwin_Coastal (2) v. Trinity_inlet (1) | 521.3750 | 0.6643 | 0.0005 | 0.6643 | NA | NA | -0.0058 | 1.0000 | -0.0064 | 0.6643 | NA | NA | NA | NA | NA | |
| Daly_River (3) v. South_Alligator_River (10) | 874.9653 | 0.3796 | 0.0002 | 0.9760 | -40.4013 | 0.0626 | -0.0059 | 0.7916 | -0.0066 | 0.7916 | 0.7121 | 0.9507 | 0.7354 | 0.9507 | 0.8455 | 0 |
| Daly_River (3) v. Wenlock_River (4) | 740.3896 | 0.7672 | 0.0004 | 0.7213 | 23.1691 | 0.2727 | -0.0065 | 0.7672 | -0.0072 | 0.7672 | 1.9954 | 0.5854 | 1.8670 | 0.5854 | 1.2894 | 0 |
| Darwin_Coastal (2) v. Unknown (2) | 559.5262 | 1.0000 | 0.0004 | 1.0000 | 3.8406 | 0.3287 | -0.0065 | 1.0000 | -0.0072 | 1.0000 | -1.9410 | 0.3287 | -1.0623 | 0.3287 | 3.1916 | 0 |
| Arabian_Sea (15) v. Trinity_inlet (1) | 914.4754 | 0.6993 | 0.0007 | 0.4915 | 4.0808 | 1.0000 | -0.0066 | 1.0000 | -0.0073 | 1.0000 | -2.9064 | 1.0000 | -1.9490 | 1.0000 | 2.5469 | 1 |
| Towns_River (2) v. Unknown (2) | 544.1417 | 0.6583 | 0.0005 | 0.3127 | NA | NA | -0.0067 | 1.0000 | -0.0074 | 0.8292 | NA | NA | NA | NA | NA | |
| Darwin_Coastal (2) v. Mozambique (11) | 858.6603 | 0.7612 | 0.0004 | 0.8432 | 27.7003 | 0.3692 | -0.0079 | 0.6713 | -0.0090 | 0.6307 | 1.6825 | 0.6774 | 1.6025 | 0.6774 | 1.2246 | 0 |
| Daly_River (3) v. Darwin_Coastal (2) | 645.8198 | 0.6154 | 0.0005 | 0.6154 | 6.0191 | 0.6508 | -0.0080 | 0.7133 | -0.0089 | 0.7133 | -4.2394 | 0.5359 | -3.0819 | 0.4420 | 2.2601 | 0 |
| Clarence_River (11) v. Trinity_inlet (1) | 840.8295 | 0.3896 | 0.0008 | 0.4855 | 3.7561 | 1.0000 | -0.0086 | 0.4824 | -0.0095 | 0.4824 | -2.3866 | 1.0000 | -1.4671 | 1.0000 | 2.7792 | 1 |
| Thailand (5) v. Unknown (2) | 670.2214 | 0.7942 | 0.0005 | 0.1928 | 9.9531 | 1.0000 | -0.0094 | 0.8402 | -0.0104 | 0.8402 | 14.8594 | 0.0499 | 12.5654 | 0.0499 | 1.7292 | 0 |
| East_Alligator_River (7) v. Unknown (2) | 735.2443 | 0.6653 | 0.0003 | 0.8791 | 13.2841 | 0.5807 | -0.0095 | 0.7922 | -0.0105 | 0.7922 | 4.3775 | 0.5143 | 3.8981 | 0.5143 | 1.5389 | 0 |
| South_Africa (23) v. Trinity_inlet (1) | 1010.1417 | 0.7982 | 0.0007 | 0.8332 | 4.5868 | 1.0000 | -0.0096 | 1.0000 | -0.0107 | 1.0000 | -4.3532 | 1.0000 | -3.2004 | 1.0000 | 2.2527 | 1 |
| Clarence_River (11) v. Unknown (2) | 806.1261 | 0.8252 | 0.0004 | 0.4955 | 29.8404 | 0.9452 | -0.0102 | 0.7260 | -0.0113 | 0.7260 | 1.6118 | 0.0635 | 1.5409 | 0.0635 | 1.2071 | 0 |
| Papua_New_Guinea (8) v. Unknown (2) | 769.2311 | 0.7203 | 0.0003 | 0.8791 | 15.8789 | 0.4786 | -0.0103 | 0.8567 | -0.0114 | 0.8567 | 3.0248 | 0.5577 | 2.7542 | 0.5577 | 1.4359 | 0 |
| Darwin_Coastal (2) v. Wenlock_River (4) | 667.6609 | 1.0000 | 0.0004 | 0.8751 | 8.0633 | 0.7143 | -0.0114 | 0.9259 | -0.0127 | 0.9259 | -19.5609 | 0.1925 | -15.7431 | 0.1925 | 1.9021 | 0 |
| Reunion (28) v. Unknown (2) | 1051.9413 | 0.7143 | 0.0003 | 0.8791 | -37.6713 | 0.4035 | -0.0115 | 0.8006 | -0.0127 | 0.8006 | 0.7465 | 0.6316 | 0.7670 | 0.6368 | 0.8666 | 0 |
| Sri_Lanka (12) v. Unknown (2) | 887.2710 | 0.5205 | 0.0003 | 0.7552 | 34.4005 | 0.6362 | -0.0118 | 0.8682 | -0.0130 | 0.8682 | 1.5064 | 0.3441 | 1.4506 | 0.3441 | 1.1798 | 0 |
| Thailand (5) v. Trinity_inlet (1) | 642.0085 | 0.6623 | 0.0007 | 0.8362 | 2.8892 | 1.0000 | -0.0124 | 1.0000 | -0.0137 | 1.0000 | -1.6216 | 1.0000 | -0.6815 | 1.0000 | 3.7051 | 1 |
| Sydney_Harbour (8) v. Trinity_inlet (1) | 757.5779 | 0.5604 | 0.0007 | 0.8941 | 3.3631 | 1.0000 | -0.0135 | 1.0000 | -0.0150 | 1.0000 | -1.9376 | 1.0000 | -1.0397 | 1.0000 | 3.1755 | 1 |
| Seychelles (36) v. Unknown (2) | 1050.5379 | 0.9121 | 0.0003 | 0.9431 | -21.9235 | 0.6319 | -0.0137 | 0.9383 | -0.0152 | 0.9383 | 0.6280 | 0.4087 | 0.6553 | 0.4174 | 0.7883 | 0 |
| Towns_River (2) v. Wenlock_River (4) | 658.9220 | 0.9411 | 0.0005 | 0.3586 | 7.6012 | 0.8929 | -0.0140 | 0.8537 | -0.0157 | 0.8308 | -10.1449 | 0.2273 | -8.0333 | 0.2273 | 1.9878 | 0 |
| Arabian_Sea (15) v. Unknown (2) | 890.8158 | 0.8871 | 0.0003 | 0.9860 | 81.3743 | 0.6275 | -0.0148 | 0.9607 | -0.0164 | 0.9607 | 1.1720 | 0.3686 | 1.1546 | 0.3686 | 1.0712 | 0 |
| Sydney_Harbour (8) v. Unknown (2) | 761.5227 | 0.7922 | 0.0004 | 0.8521 | 16.1916 | 0.5161 | -0.0151 | 0.9387 | -0.0168 | 0.9387 | 2.8872 | 0.3986 | 2.6344 | 0.3986 | 1.4205 | 0 |
| South_Africa (23) v. Unknown (2) | 1001.7286 | 0.8781 | 0.0003 | 0.9790 | -58.1887 | 0.4757 | -0.0153 | 0.9590 | -0.0169 | 0.9590 | 0.8227 | 0.5604 | 0.8378 | 0.5604 | 0.9110 | 0 |
| Unknown (2) v. Wenlock_River (4) | 644.6439 | 1.0000 | 0.0004 | 1.0000 | 8.0260 | 0.5532 | -0.0187 | 1.0000 | -0.0209 | 1.0000 | -16.1912 | 0.1790 | -13.0059 | 0.1790 | 1.9220 | 0 |
| South_Alligator_River (10) v. Unknown (2) | 761.8605 | 0.9600 | 0.0003 | 0.9750 | 19.7495 | 0.5007 | -0.0207 | 1.0000 | -0.0229 | 1.0000 | 2.3162 | 0.3737 | 2.1534 | 0.3737 | 1.3478 | 0 |
| Trinity_inlet (1) v. Wenlock_River (4) | 631.1450 | 1.0000 | 0.0008 | 1.0000 | 2.6428 | 1.0000 | -0.0262 | 1.0000 | -0.0294 | 1.0000 | -1.5163 | 1.0000 | -0.5327 | 1.0000 | 3.9687 | 1 |
| Daly_River (3) v. Unknown (2) | 597.6625 | 1.0000 | 0.0004 | 1.0000 | 5.9453 | 0.5563 | -0.0278 | 1.0000 | -0.0309 | 1.0000 | -3.8841 | 0.1192 | -2.8042 | 0.2373 | 2.3190 | 0 |
| Mozambique (11) v. Trinity_inlet (1) | 817.3900 | 0.7413 | 0.0007 | 0.8132 | 3.6731 | 1.0000 | -0.0288 | 1.0000 | -0.0319 | 1.0000 | -2.2404 | 1.0000 | -1.3475 | 1.0000 | 2.8926 | 1 |
| Mozambique (11) v. Unknown (2) | 815.7891 | 0.9011 | 0.0003 | 1.0000 | 26.7065 | 0.3398 | -0.0297 | 0.9757 | -0.0329 | 0.9757 | 1.7470 | 0.6988 | 1.6607 | 0.6988 | 1.2405 | 0 |
| Trinity_inlet (1) v. Unknown (2) | 467.2917 | 1.0000 | 0.0004 | 1.0000 | NA | NA | -0.0360 | 1.0000 | -0.0402 | 1.0000 | NA | NA | NA | NA | NA | |
| Daly_River (3) v. Trinity_inlet (1) | 566.7849 | 1.0000 | 0.0007 | 1.0000 | 2.2976 | 1.0000 | -0.0465 | 1.0000 | -0.0517 | 1.0000 | -1.3569 | 1.0000 | -0.2993 | 1.0000 | 4.6405 | 1 |
| Daly_River (3) v. Indonesia (4) | 788.6998 | 0.2787 | 0.0005 | 0.3636 | NA | NA | NA | NA | NA | NA | NA | NA | NA | NA | NA | |
| Fiji (24) v. Indonesia (4) | 1624.5788 | 0.0010 | 0.0005 | 0.0010 | NA | NA | NA | NA | NA | NA | NA | NA | NA | NA | NA | |
| Indonesia (4) v. South_Africa (23) | 1195.6827 | 0.0829 | 0.0003 | 0.0559 | NA | NA | NA | NA | NA | NA | NA | NA | NA | NA | NA | |
| Indonesia (4) v. Urauchi_River (35) | 2021.5069 | 0.0010 | 0.0008 | 0.0010 | NA | NA | NA | NA | NA | NA | NA | NA | NA | NA | NA | |
| Sierra_Leone (1) v. Trinity_inlet (1) | 566.6667 | 1.0000 | 0.0009 | 1.0000 | NA | NA | NA | NA | NA | NA | NA | NA | NA | NA | NA | |

## 9.8 PCA

```
pca1 <- adegenet::glPca(BS.dartcap.sub.gl, nf = 5, parallel = TRUE,
                        n.cores = parallel::detectCores() - 1)
save(pca1, file = "DArTcap_AUS_subset_PCA.Rdata")
```

```
load("DArTcap_AUS_subset_PCA.Rdata")

BS.pca.scores <- as.data.frame(pca1$scores)
BS.pca.scores$pop <- pop(BS.dartcap.sub.gl)
cols <- adegenet::funky(nPop(BS.dartcap.sub.gl))


p <-
  ggplot2::ggplot(BS.pca.scores, ggplot2::aes(x = PC1, y = PC2, colour = pop)) +
  ggplot2::geom_point(size = 2) + ggplot2::scale_color_manual(values = cols) +
  ggplot2::geom_hline(yintercept = 0) + ggplot2::geom_vline(xintercept = 0) +
  ggplot2::theme_minimal() +
  ggplot2::theme(
    axis.text = ggplot2::element_text(size = 5),
    axis.title.x = ggplot2::element_text(size = 10),
    axis.title.y = ggplot2::element_text(size = 10),
    legend.text = ggplot2::element_text(size = 7)
  )
p
```

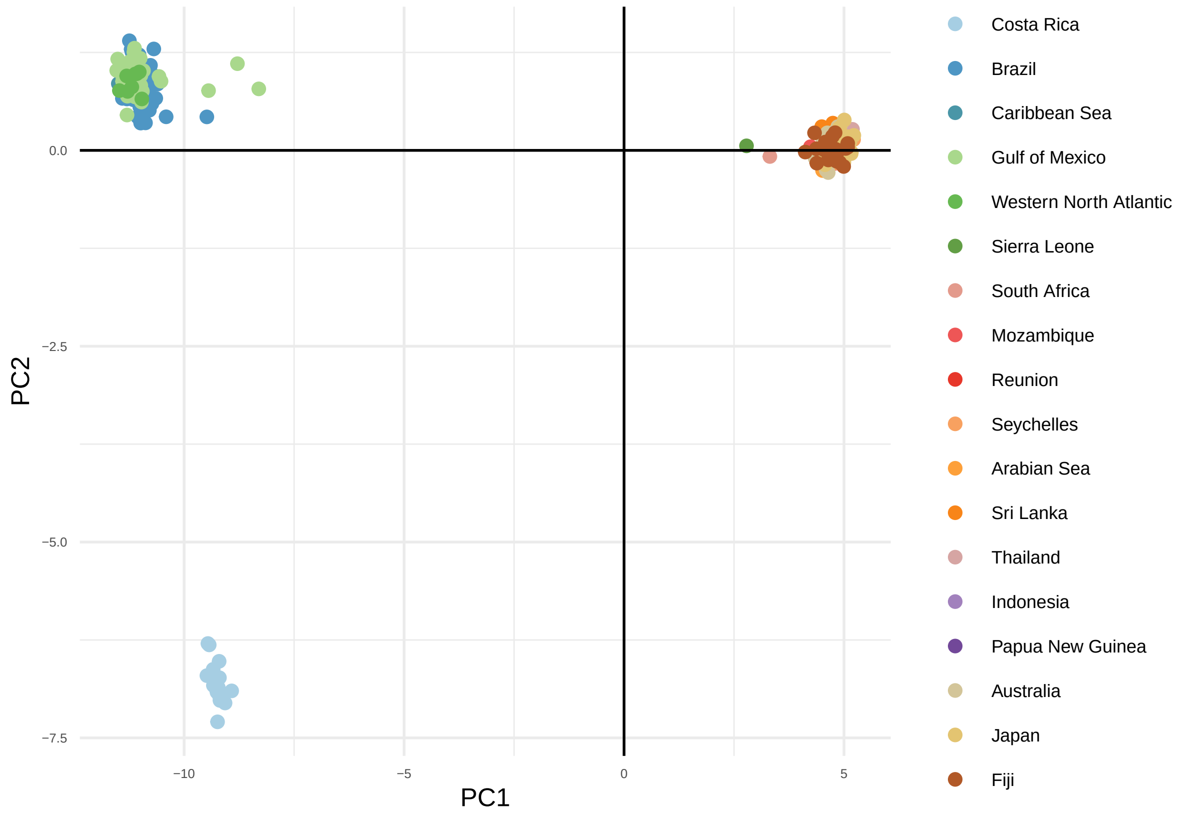


```
ggplot2::ggsave(p, filename = "DArTcap_AUS_subset_PCA1.png", width = 30,
                height = 15,units = "cm")


p <-
  ggplot2::ggplot(BS.pca.scores, ggplot2::aes(x = PC1, y = PC3, colour = pop)) +
```

```r
  ggplot2::geom_point(size = 2) + ggplot2::scale_color_manual(values = cols) +
  ggplot2::geom_hline(yintercept = 0) + ggplot2::geom_vline(xintercept = 0) +
  ggplot2::theme_minimal() +
  ggplot2::theme(
    axis.text = ggplot2::element_text(size = 5),
    axis.title.x = ggplot2::element_text(size = 10),
    axis.title.y = ggplot2::element_text(size = 10),
    legend.text = ggplot2::element_text(size = 7)
  )
p
```

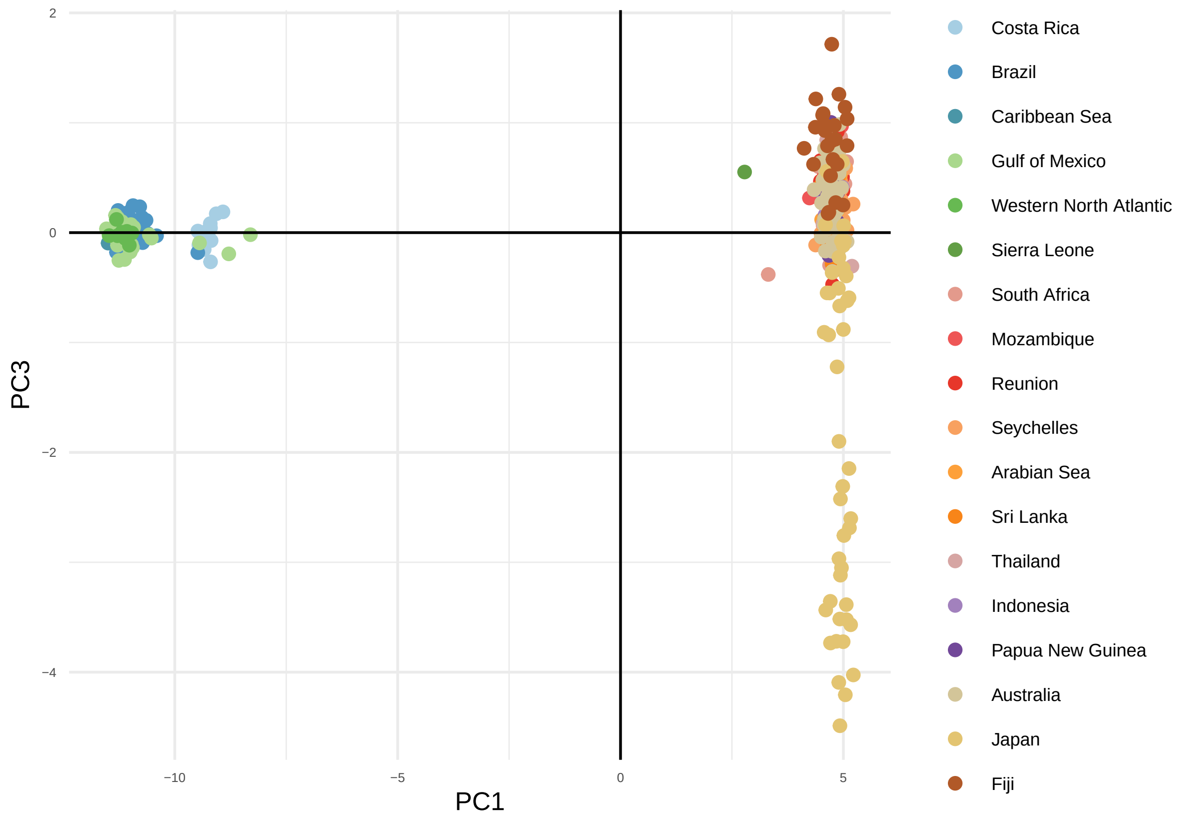


```r
ggplot2::ggsave(p, filename = "DArTcap_AUS_subset_PCA2.png", width = 30,
                height = 15,units = "cm")


p <-
  ggplot2::ggplot(BS.pca.scores, ggplot2::aes(x = PC2, y = PC3, colour = pop)) +
  ggplot2::geom_point(size = 2) + ggplot2::scale_color_manual(values = cols) +
  ggplot2::geom_hline(yintercept = 0) + ggplot2::geom_vline(xintercept = 0) +
  ggplot2::theme_minimal() +
  ggplot2::theme(
    axis.text = ggplot2::element_text(size = 5),
    axis.title.x = ggplot2::element_text(size = 10),
    axis.title.y = ggplot2::element_text(size = 10),
    legend.text = ggplot2::element_text(size = 7)
  )
p
```

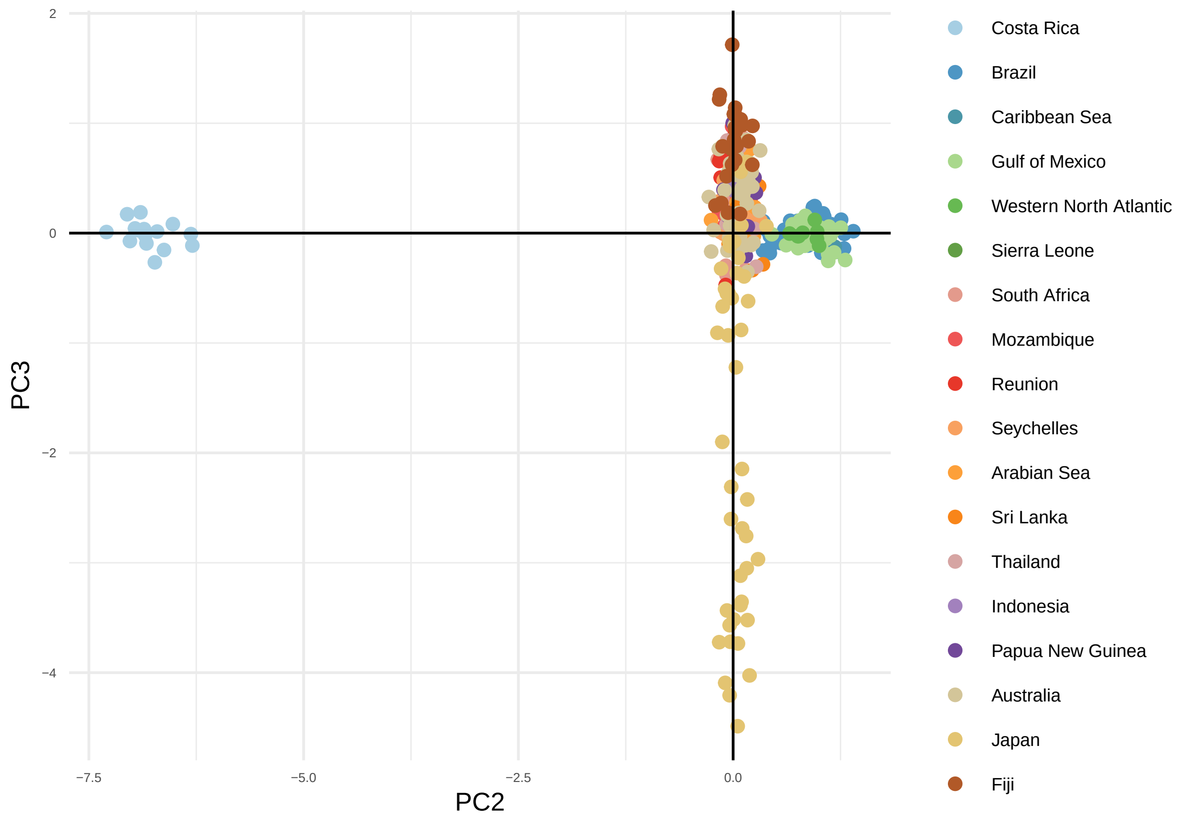


```
ggplot2::ggsave(p, filename = "DArTcap_AUS_subset_PCA3.png", width = 30,
                height = 15,units = "cm")


p <-
  ggplot2::ggplot(BS.pca.scores, ggplot2::aes(x = PC3, y = PC4, colour = pop)) +
  ggplot2::geom_point(size = 2) + ggplot2::scale_color_manual(values = cols) +
  ggplot2::geom_hline(yintercept = 0) + ggplot2::geom_vline(xintercept = 0) +
  ggplot2::theme_minimal() +
  ggplot2::theme(
    axis.text = ggplot2::element_text(size = 5),
    axis.title.x = ggplot2::element_text(size = 10),
    axis.title.y = ggplot2::element_text(size = 10),
    legend.text = ggplot2::element_text(size = 7)
  )
p
```

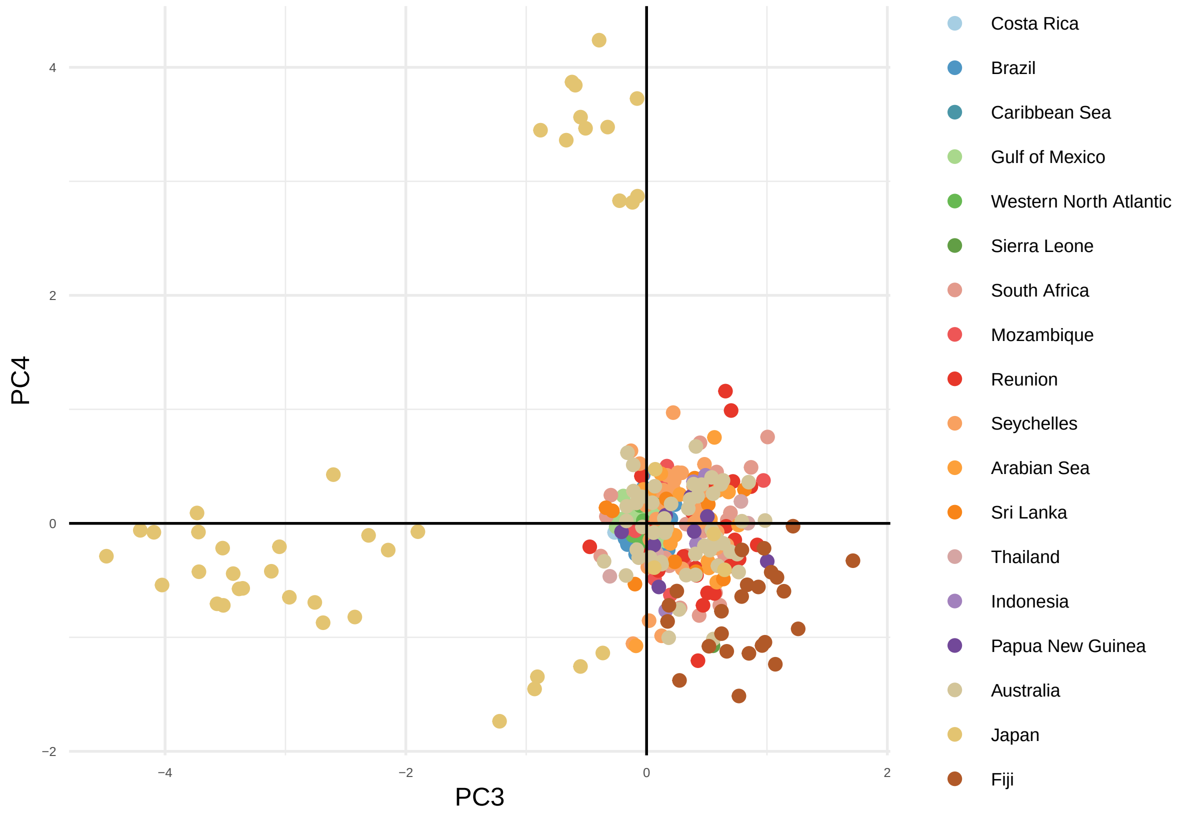


```r
ggplot2::ggsave(p, filename = "DArTcap_AUS_subset_PCA4.png", width = 30,
                height = 15,units = "cm")

p <-
  ggplot2::ggplot(BS.pca.scores, ggplot2::aes(x = PC3, y = PC5, colour = pop)) +
  ggplot2::geom_point(size = 2) + ggplot2::scale_color_manual(values = cols) +
  ggplot2::geom_hline(yintercept = 0) + ggplot2::geom_vline(xintercept = 0) +
  ggplot2::theme_minimal() +
  ggplot2::theme(
    axis.text = ggplot2::element_text(size = 5),
    axis.title.x = ggplot2::element_text(size = 10),
    axis.title.y = ggplot2::element_text(size = 10),
    legend.text = ggplot2::element_text(size = 7)
  )
p
```

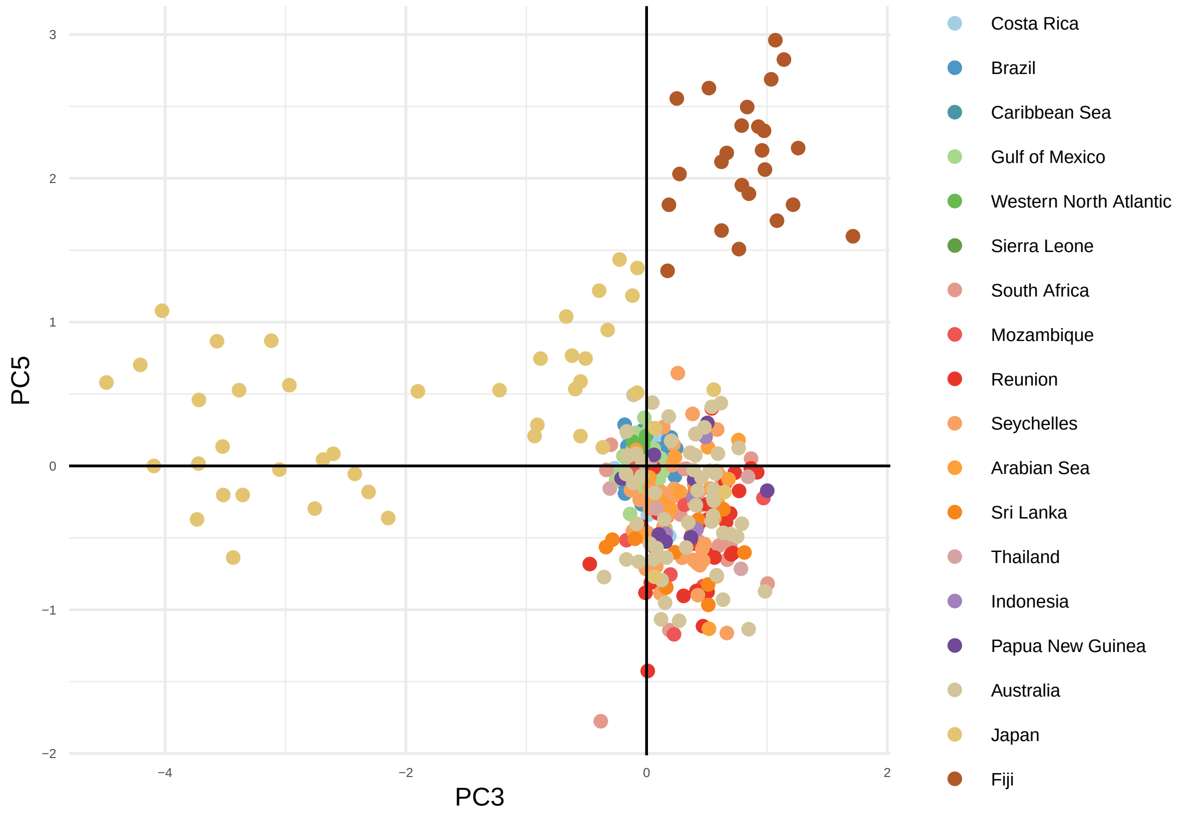


```
ggplot2::ggsave(p, filename = "DArTcap_AUS_subset_PCA5.png", width = 30,
                height = 15,units = "cm")

p <-
  ggplot2::ggplot(BS.pca.scores, ggplot2::aes(x = PC4, y = PC5, colour = pop)) +
  ggplot2::geom_point(size = 2) + ggplot2::scale_color_manual(values = cols) +
  ggplot2::geom_hline(yintercept = 0) + ggplot2::geom_vline(xintercept = 0) +
  ggplot2::theme_minimal() +
  ggplot2::theme(
    axis.text = ggplot2::element_text(size = 5),
    axis.title.x = ggplot2::element_text(size = 10),
    axis.title.y = ggplot2::element_text(size = 10),
    legend.text = ggplot2::element_text(size = 7)
  )
p
```

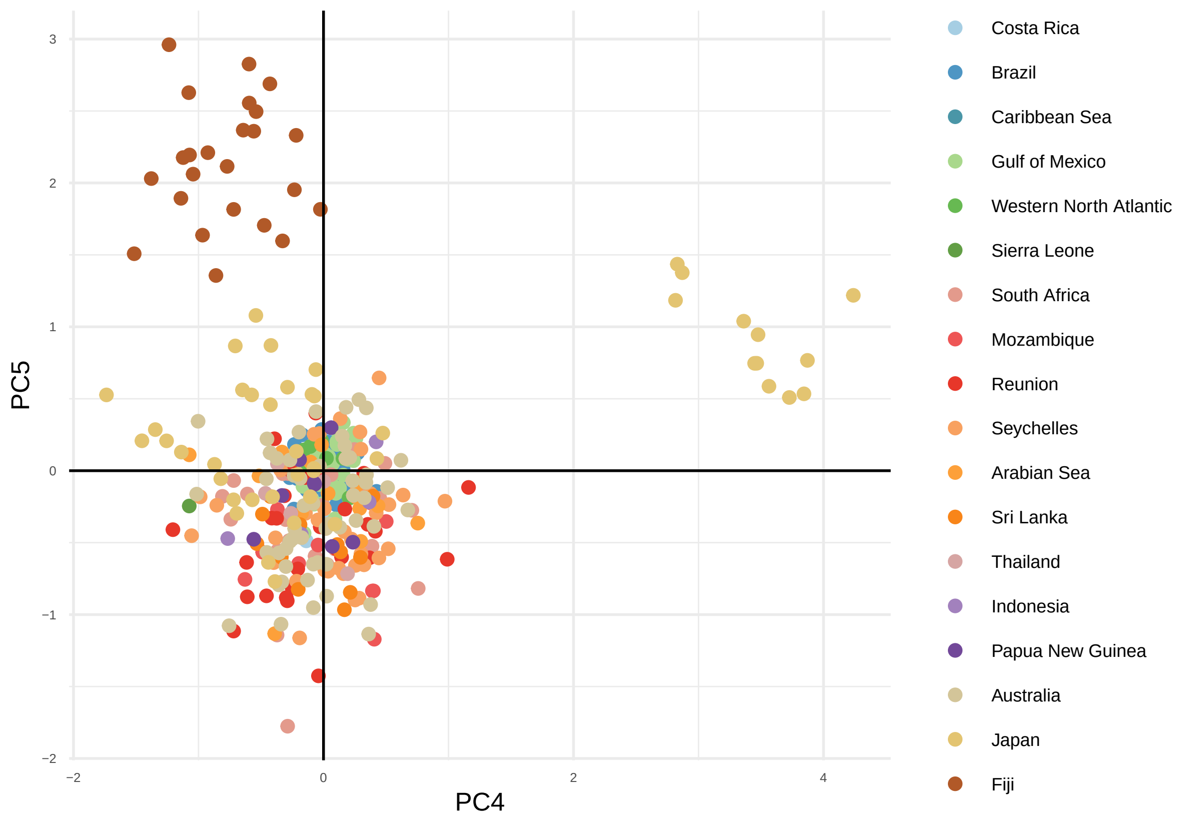


```
ggplot2::ggsave(p, filename = "DArTcap_AUS_subset_PCA6.png", width = 30,
                height = 15,units = "cm")
```

## 9.9 DAPC

### 9.9.1 Optimal number of clusters with neutral markers

```
set.seed(124)
grp <-
  adegenet::find.clusters(
    BS.dartcap.sub.gl,
    max.n.clust = 18,
    n.pca = adegenet::nInd(BS.dartcap.sub.gl) / 3,
    stat = "BIC",
    parallel = TRUE,
    n.cores = parallel::detectCores() - 1
  )

save(grp, file = "DArTcap_AUS_subset_grp_.Rdata")
```

```
load("DArTcap_AUS_subset_grp_.Rdata")

y <- as.numeric(grp$Kstat)
x <- 1:18
data <- data.frame(x,y,stringsAsFactors = F)
plot <- ggplot2::ggplot(data, ggplot2::aes(x,y)) +
  ggplot2::geom_point(size = 5, shape = 1, color = "blue") +
  ggplot2::geom_line(size = 1,color = "blue") +
  ggplot2::scale_x_continuous(name = waiver(),
                              breaks = seq(from = 0,
                                          to = nrow(BS.dartcap.sub.gl) - 1,
```

```
                                              by = 1)) +
  ggplot2::labs(subtitle = "",
                y = "Bayesian Information Criterion",
                x = "Number of clusters",
                title = "",
                caption = "") +
  ggplot2::theme_classic() +
  ggplot2::theme(
    axis.text = ggplot2::element_text(size = 5),
    axis.title.x = ggplot2::element_text(size = 20,vjust = 0, hjust = 0.5),
    axis.title.y = ggplot2::element_text(size = 20,vjust = 2, hjust = 0.5))
print(plot)
```

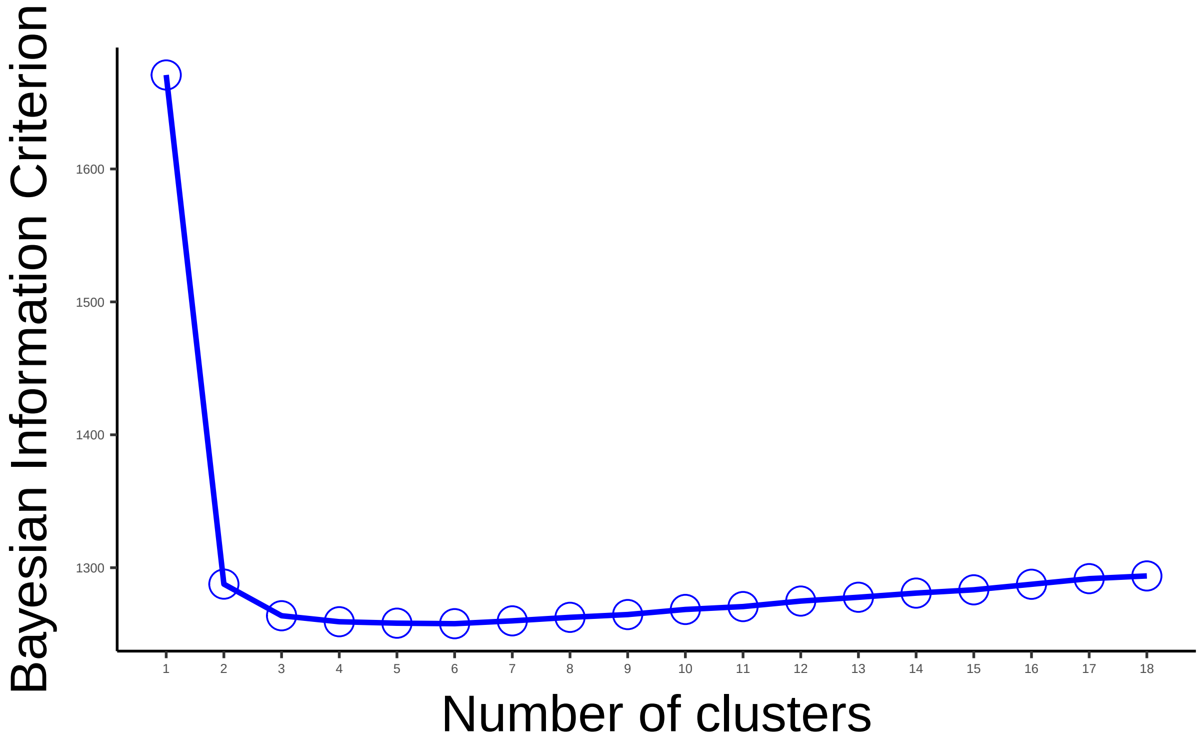


```
ggplot2::ggsave(plot, filename = "DArTcap_AUS_subset_grp.png", width = 30,
                height = 15,units = "cm")
```

### 9.9.2 Cross-validation to identify the optimal number of PCs

Uses a training - holdout set of individuals to check how reliable individuals can be assigned.

```
set.seed(124)
x <- BS.dartcap.sub.gl
x <- x[x$pop != "Sierra Leone",]

xval <-
  adegenet::xvalDapc(
  tab(x, NA.method = "mean"),
  adegenet::pop(x),
  n.pca = seq(1, adegenet::nInd(x), by = 50),
  training.set = 0.9,
  result = "groupMean",
  center = TRUE,
```

```
  scale = FALSE,
  n.rep = 1000,
  parallel = "multicore",
  ncpus = 1,
  xval.plot = TRUE
  )
print(xval[2:6])
PC <- as.numeric(xval$`Number of PCs Achieving Lowest MSE`)

#refine the analysis
set.seed(124)
png(file="DArTcap_AUS_subset_xval.png")
xval <-
  adegenet::xvalDapc(
  tab(x, NA.method = "mean"),
  adegenet::pop(x),
  n.pca = (PC - 15):(PC + 15),
  n.rep = 1000,
  parallel = "multicore",
  ncpus = 1,
  xval.plot = TRUE
  )
dev.off()
save(xval, file = "DArTcap_AUS_subset_xval.rdata")
```

```
load("DArTcap_AUS_subset_xval.rdata")
print(xval[2:6])
PC <- as.numeric(xval$`Number of PCs Achieving Lowest MSE`)
```

```
## $`Median and Confidence Interval for Random Chance`
##       2.5%        50%      97.5%
## 0.03861550 0.05695908 0.08868781
##
## $`Mean Successful Assignment by Number of PCs of PCA`
##        36        37        38        39        40        41        42        43
## 0.3338533 0.3343388 0.3376489 0.3371772 0.3356450 0.3439839 0.3454886 0.3482663
##        44        45        46        47        48        49        50        51
## 0.3494908 0.3464274 0.3467327 0.3502776 0.3493193 0.3541947 0.3556213 0.3585058
##        52        53        54        55        56        57        58        59
## 0.3548909 0.3550931 0.3545634 0.3529064 0.3560120 0.3526906 0.3537629 0.3516880
##        60        61        62        63        64        65        66
## 0.3503346 0.3497025 0.3500120 0.3511077 0.3515862 0.3513141 0.3521010
##
## $`Number of PCs Achieving Highest Mean Success`
## [1] "51"
##
## $`Root Mean Squared Error by Number of PCs of PCA`
##        36        37        38        39        40        41        42        43
## 0.6665824 0.6660893 0.6627735 0.6632440 0.6647774 0.6564674 0.6550179 0.6522013
##        44        45        46        47        48        49        50        51
## 0.6509811 0.6540736 0.6537285 0.6502198 0.6511483 0.6462868 0.6448894 0.6419722
##        52        53        54        55        56        57        58        59
## 0.6456393 0.6453911 0.6459184 0.6475422 0.6444630 0.6478024 0.6467376 0.6487830
##        60        61        62        63        64        65        66
## 0.6501345 0.6507866 0.6504542 0.6494049 0.6489529 0.6491762 0.6484199
##
## $`Number of PCs Achieving Lowest MSE`
## [1] "51"
```

```r
dapc.all.object.names <- c()
for (K in 2:10) {
  set.seed(124)
  grp <- adegenet::find.clusters(BS.dartcap.sub.gl, n.clust = K, n.pca = PC)
  set.seed(124)
  dapc.all.neutral <- adegenet::dapc(BS.dartcap.sub.gl, grp$grp, n.da = K - 1,
                                     n.pca = PC)
  assign(paste0("dapc.all.", K),value = dapc.all.neutral)
  dapc.all.object.names <- c(dapc.all.object.names,
                                     paste0("dapc.all.", K))
}
save(list = dapc.all.object.names, dapc.all.object.names,
     file = "DArTcap_AUS_subset_DAPC.Rdata")
```

### 9.9.3 DAPC barplot

Group individuals according to DAPC posterior membership.

```r
load("DArTcap_AUS_subset_DAPC.Rdata")
for (K in 2:10) {
  colour <- colours.prior[1:K]
  dapc <- get(dapc.all.object.names[K - 1])
  post <- as.matrix(dapc$posterior)
  colnames(post) <- paste0("Group", 1:nlevels(dapc$grp))
  locations <- BS.dartcap.sub.gl$pop

  plot.dapc.FDD(x = post,
                locations = locations,
                colour = colour,
                region.lwd = 1,
                plotTitle = paste0("DAPC: all Bull Sharks - subset Australia\n for K=",
                                   K, " & PC=", PC, sep = ""))
  dev.print(
    device = png,
    file = paste0("DArTcap_AUS_subset_DAPC_barplot_K", K, ".png"),
    res = 300,
    width = 30,
    height = 15,
    units = "cm")
}
```

# DAPC: all Bull Sharks − subset Australia for K=2 & PC=51

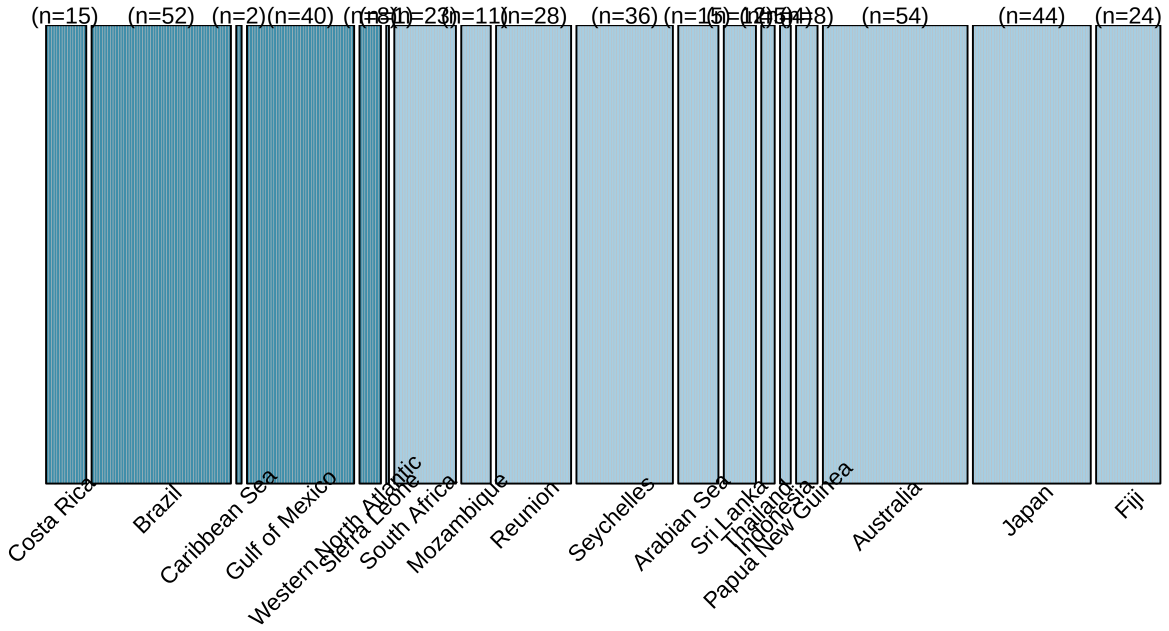


# DAPC: all Bull Sharks − subset Australia for K=3 & PC=51

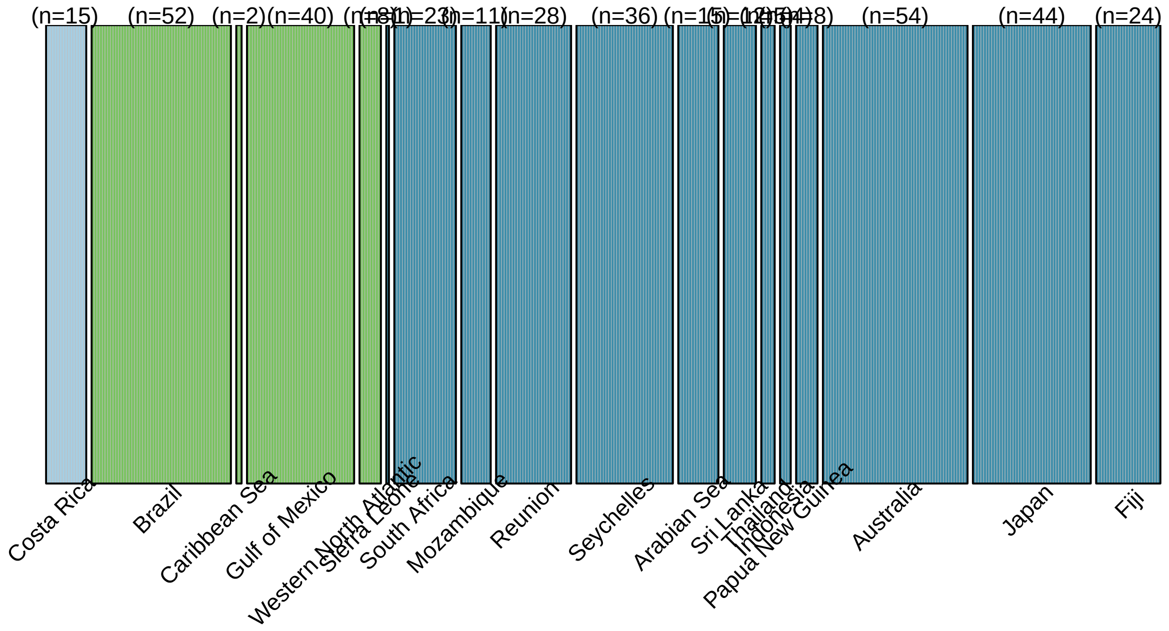

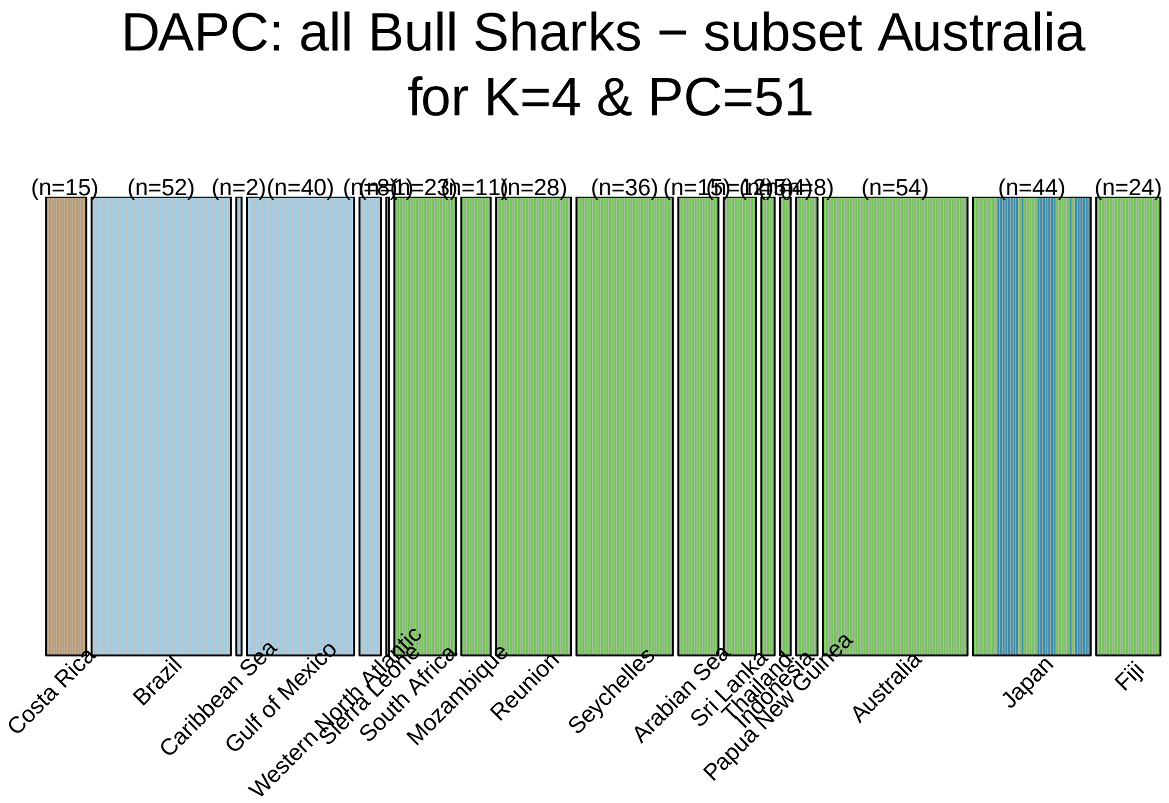
DAPC: all Bull Sharks − subset Australia
for K=4 & PC=51
(n=15)
(n=52)
(n=2)
(n=40)
(n=23)
(n=11)
(n=28)
(n=36)
(n=54)
(n=44)
(n=24)
Costa Rica
Brazil
Caribbean Sea
Gulf of Mexico
Western North Atlantic
Sierra Leone
South Africa
Mozambique
Reunion
Seychelles
Arabian Sea
Sri Lanka
Thailand
Indonesia
Papua New Guinea
Australia
Japan
Fiji

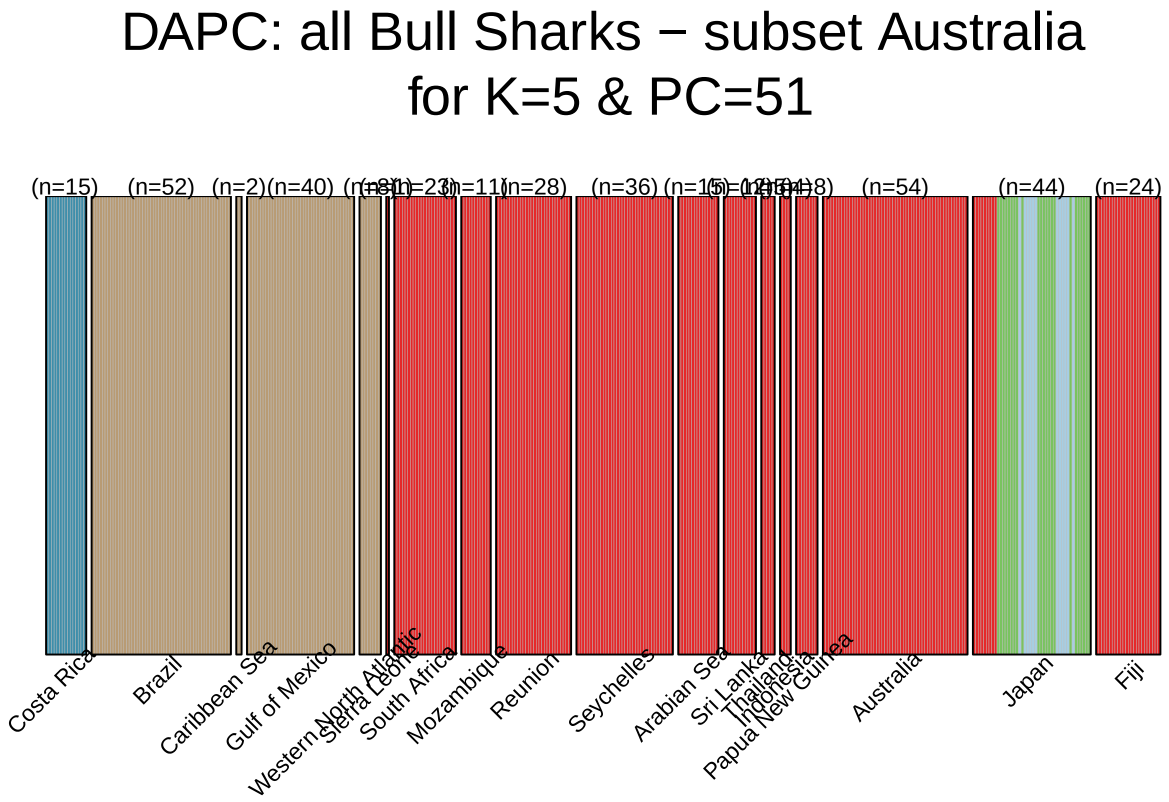
DAPC: all Bull Sharks − subset Australia
for K=5 & PC=51
(n=15)
(n=52)
(n=2)
(n=40)
(n=23)
(n=11)
(n=28)
(n=36)
(n=54)
(n=44)
(n=24)
Costa Rica
Brazil
Caribbean Sea
Gulf of Mexico
Western North Atlantic
Sierra Leone
South Africa
Mozambique
Reunion
Seychelles
Arabian Sea
Sri Lanka
Thailand
Indonesia
Papua New Guinea
Australia
Japan
Fiji

# DAPC: all Bull Sharks − subset Australia for K=6 & PC=51

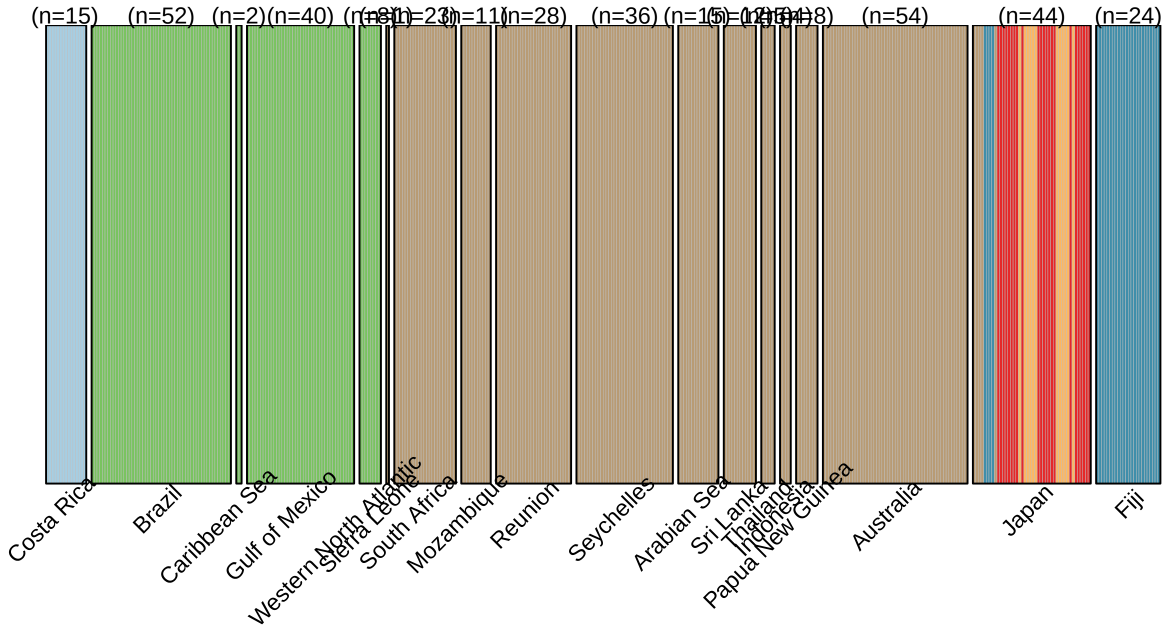


# DAPC: all Bull Sharks − subset Australia for K=7 & PC=51

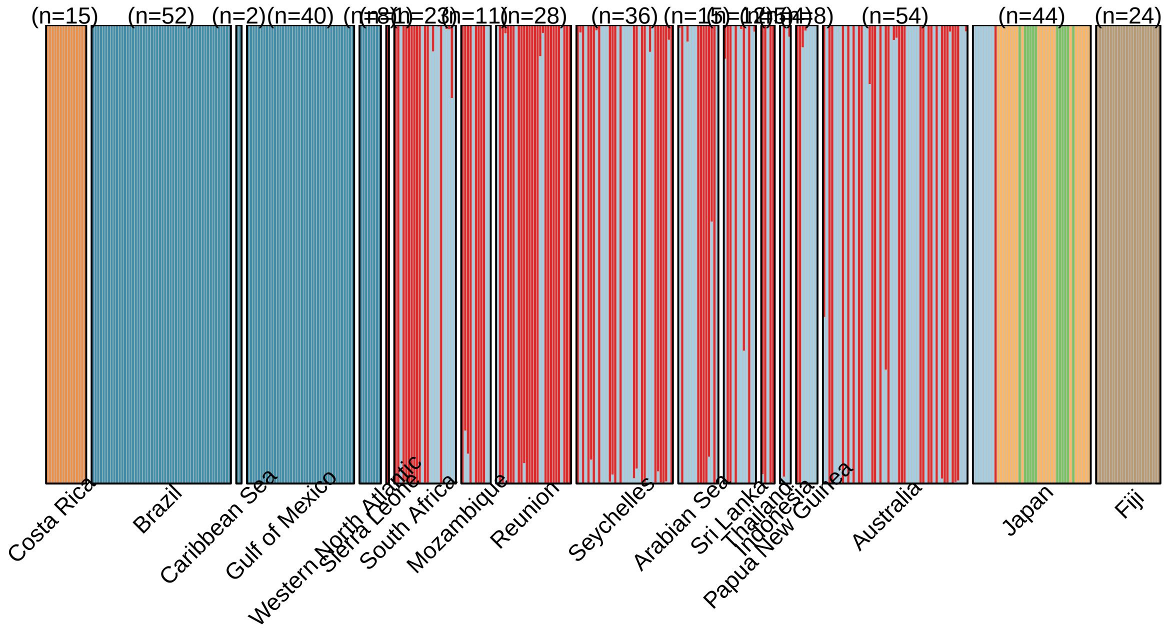

DAPC: all Bull Sharks − subset Australia
for K=8 & PC=51

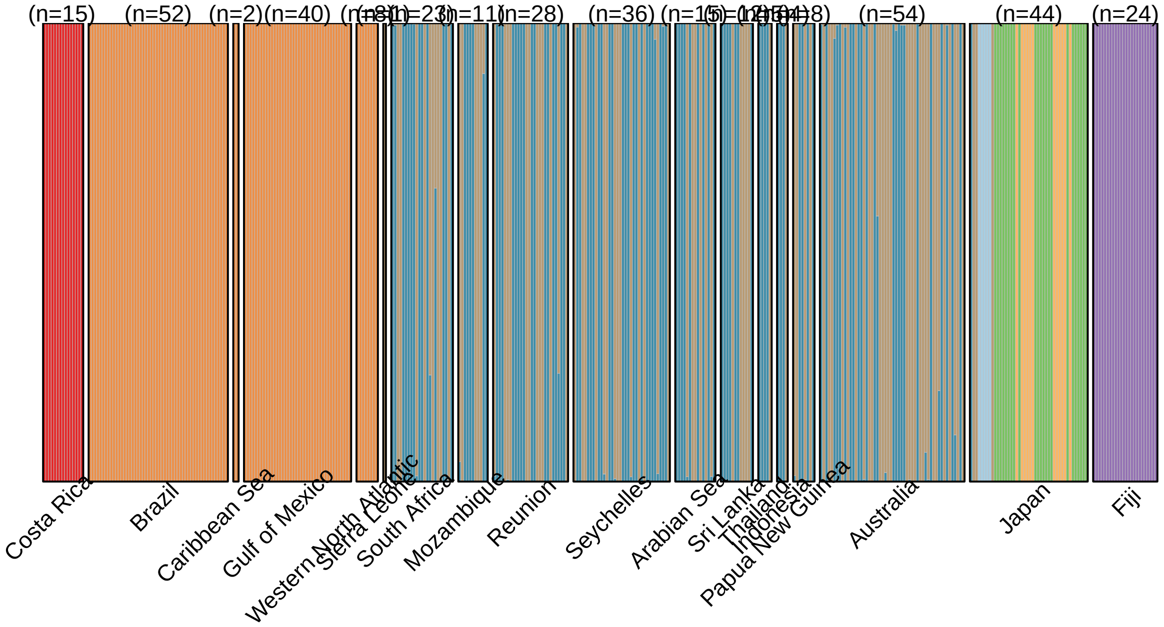


DAPC: all Bull Sharks − subset Australia
for K=9 & PC=51

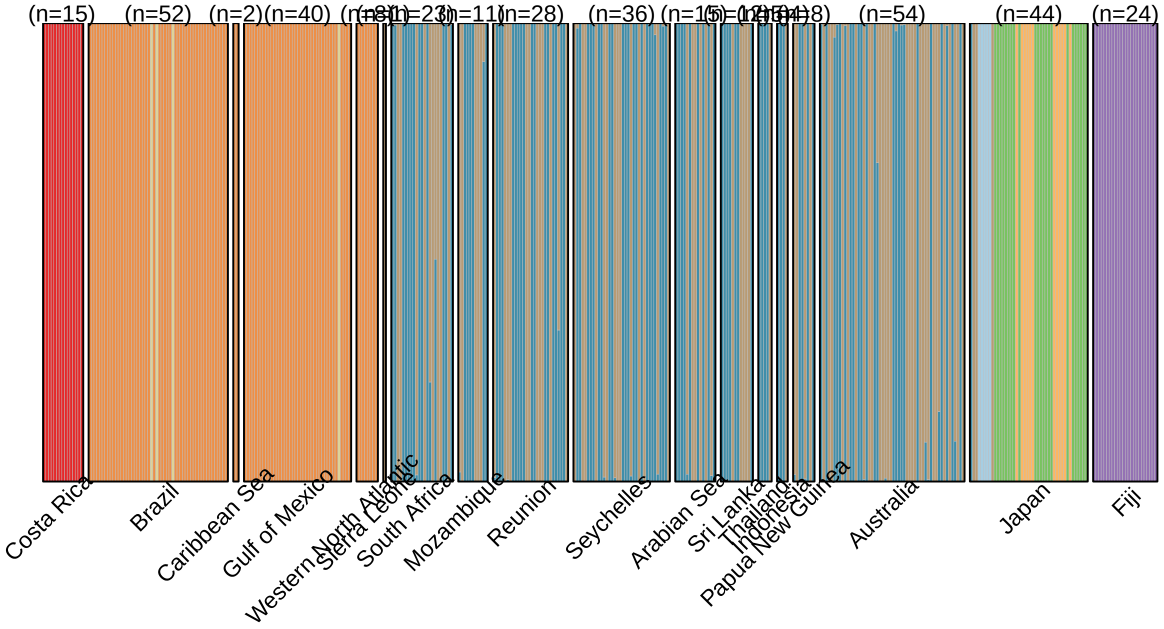

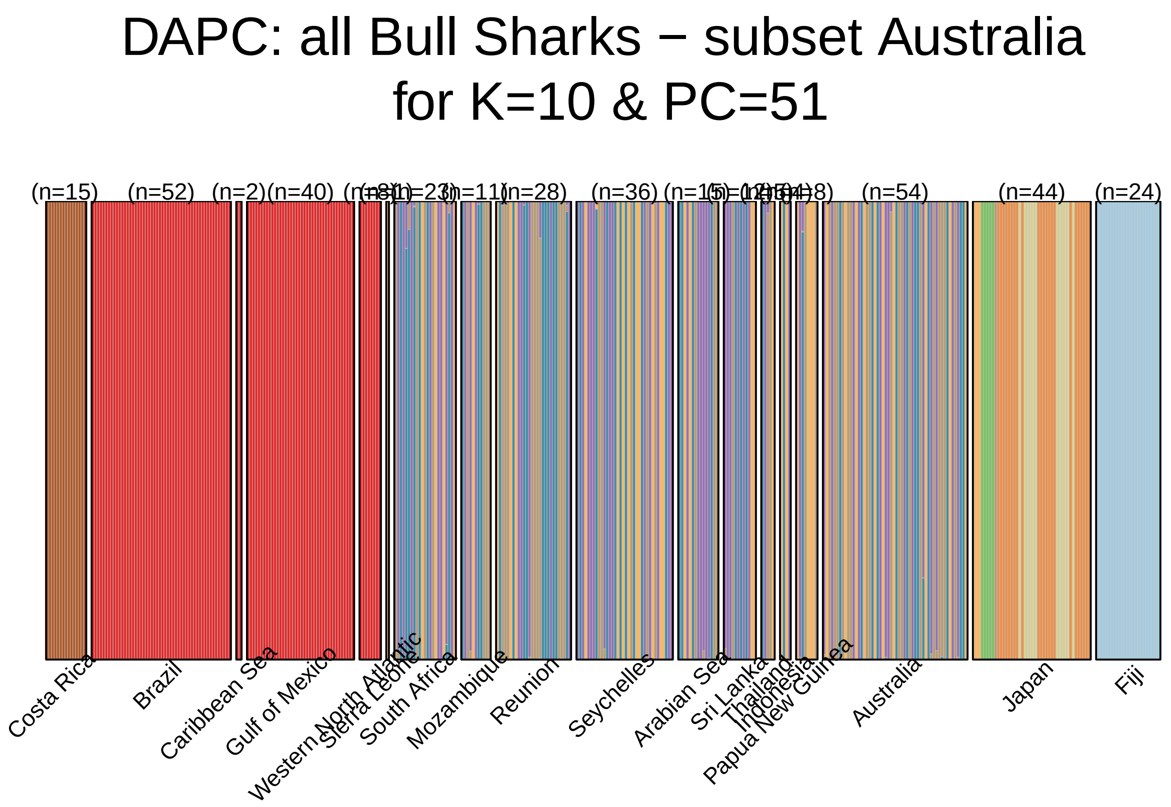


### 9.9.4 DAPC scatterplot

```
load("DArTcap_AUS_subset_DAPC.Rdata")

for (K in 3:10) {
  dapc <- get(dapc.all.object.names[K - 1])
  if (K == 2) {
    posi.leg <- "bottomright"
  } else {posi.leg <- "bottomleft"}
  dapc.plot <- ade4::scatter(dapc, grp = pop(BS.dartcap.sub.gl), cex = 2,
                             legend = TRUE, col = colours.14,clabel = FALSE,
                             posi.leg = posi.leg, scree.pca = FALSE,
                             posi.pca = "topleft", cleg = 0.75, xax = 1,
                             yax = 2, inset.solid = 0.3)
  dev.print(
    device = png,
    file = paste0("DArTcap_AUS_subset_DAPC_scatterplot_K", K, ".png"),
    width = 30,
    height = 15,
    units = "cm",
    res = 300
  )
}
```

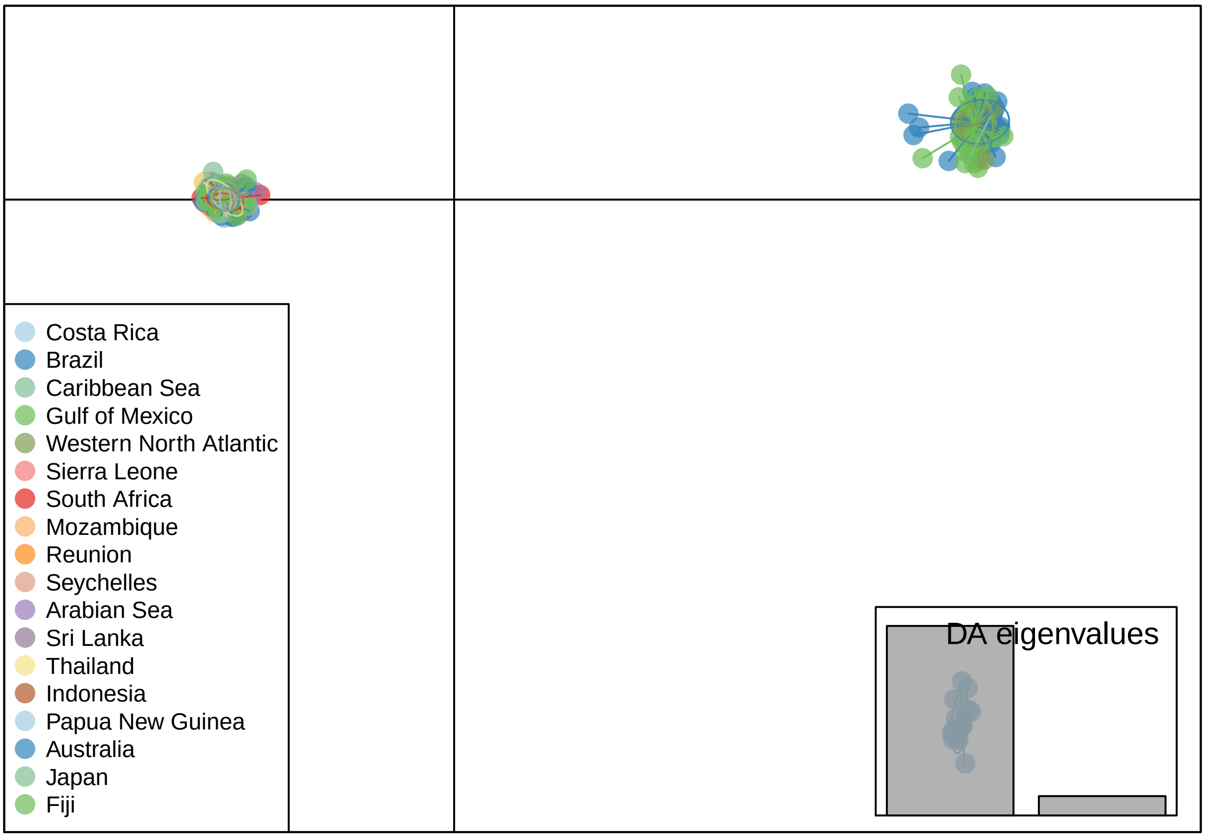
Costa Rica
Brazil
Caribbean Sea
Gulf of Mexico
Western North Atlantic
Sierra Leone
South Africa
Mozambique
Reunion
Seychelles
Arabian Sea
Sri Lanka
Thailand
Indonesia
Papua New Guinea
Australia
Japan
Fiji
DA eigenvalues

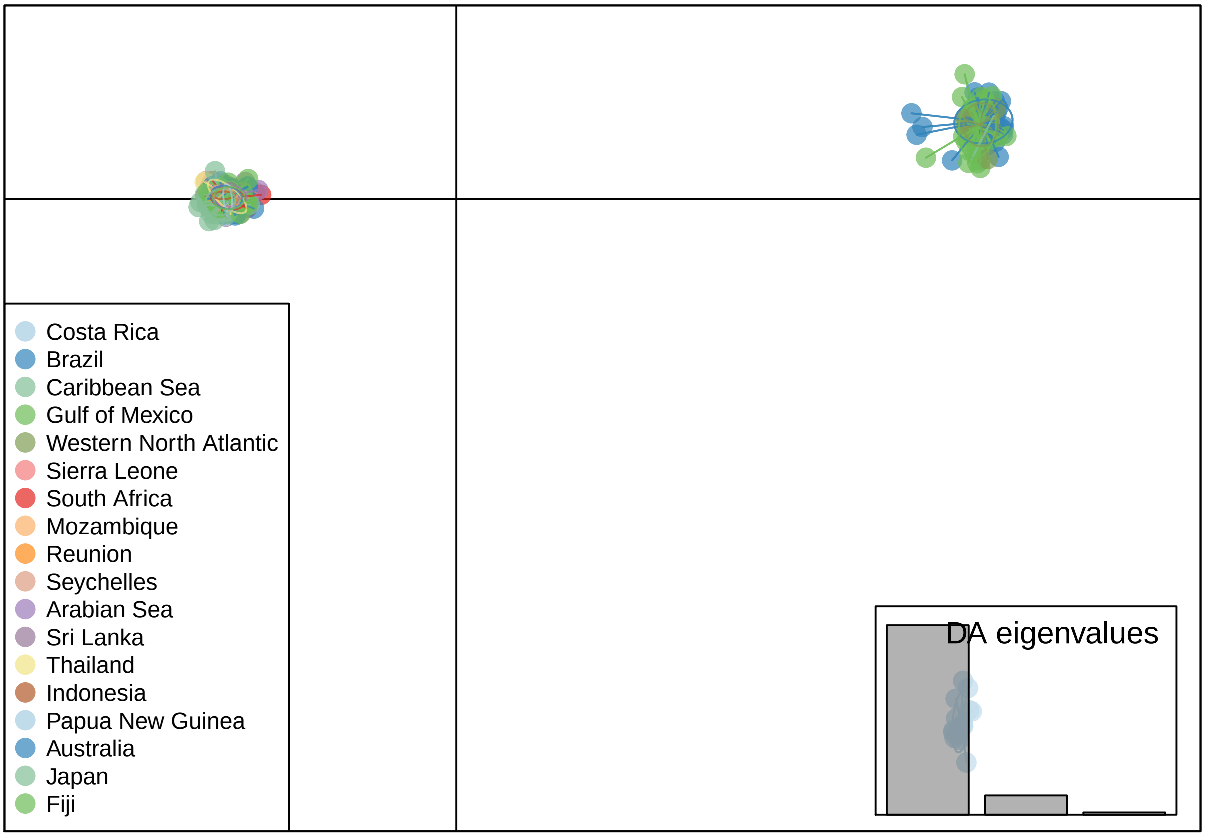
Costa Rica
Brazil
Caribbean Sea
Gulf of Mexico
Western North Atlantic
Sierra Leone
South Africa
Mozambique
Reunion
Seychelles
Arabian Sea
Sri Lanka
Thailand
Indonesia
Papua New Guinea
Australia
Japan
Fiji
DA eigenvalues

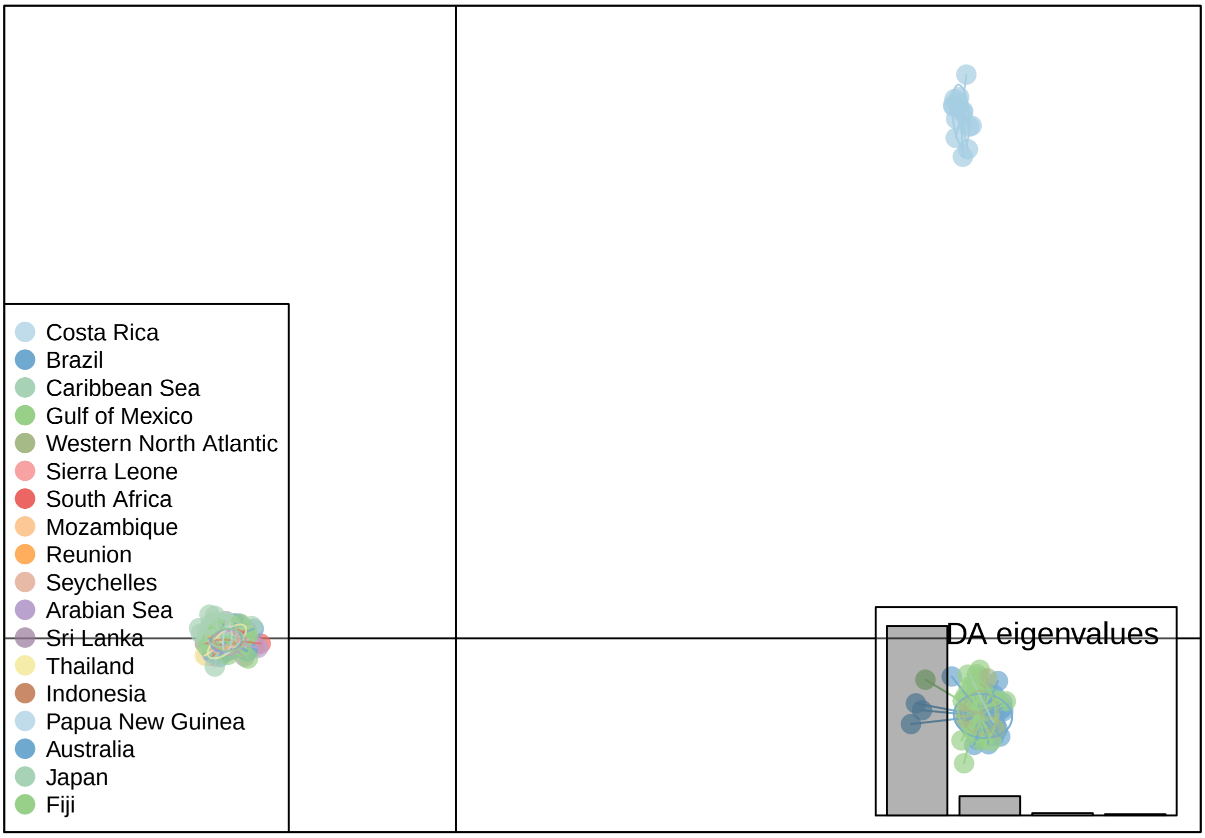
Costa Rica
Brazil
Caribbean Sea
Gulf of Mexico
Western North Atlantic
Sierra Leone
South Africa
Mozambique
Reunion
Seychelles
Arabian Sea
Sri Lanka
Thailand
Indonesia
Papua New Guinea
Australia
Japan
Fiji
DA eigenvalues

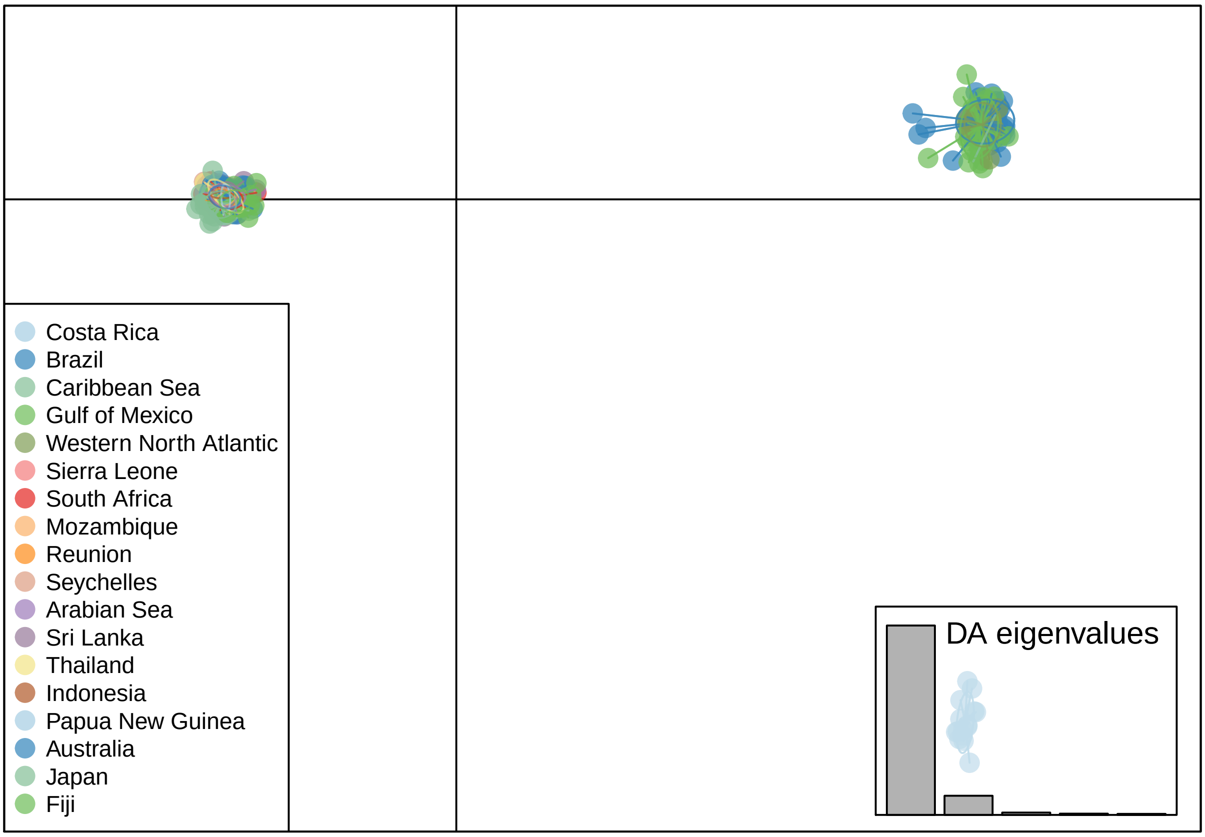
Costa Rica
Brazil
Caribbean Sea
Gulf of Mexico
Western North Atlantic
Sierra Leone
South Africa
Mozambique
Reunion
Seychelles
Arabian Sea
Sri Lanka
Thailand
Indonesia
Papua New Guinea
Australia
Japan
Fiji
DA eigenvalues

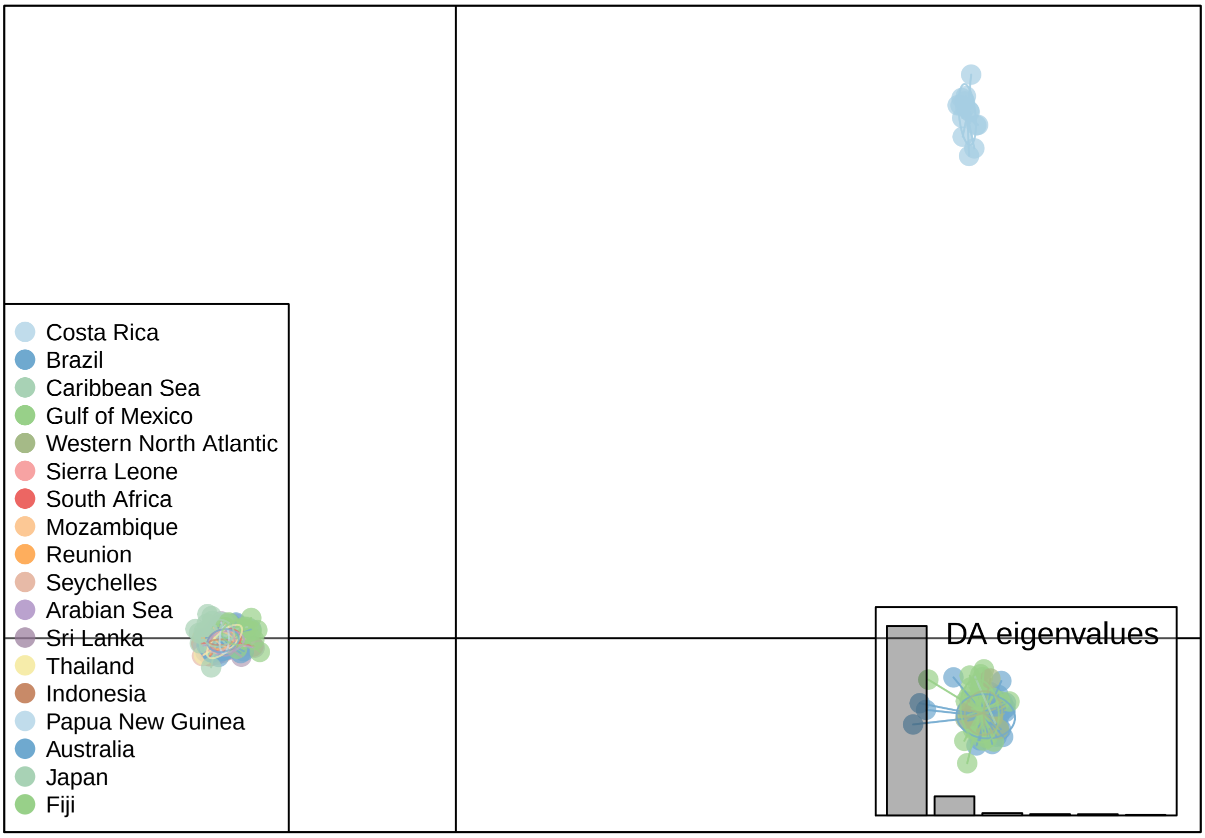
Costa Rica
Brazil
Caribbean Sea
Gulf of Mexico
Western North Atlantic
Sierra Leone
South Africa
Mozambique
Reunion
Seychelles
Arabian Sea
Sri Lanka
Thailand
Indonesia
Papua New Guinea
Australia
Japan
Fiji
DA eigenvalues

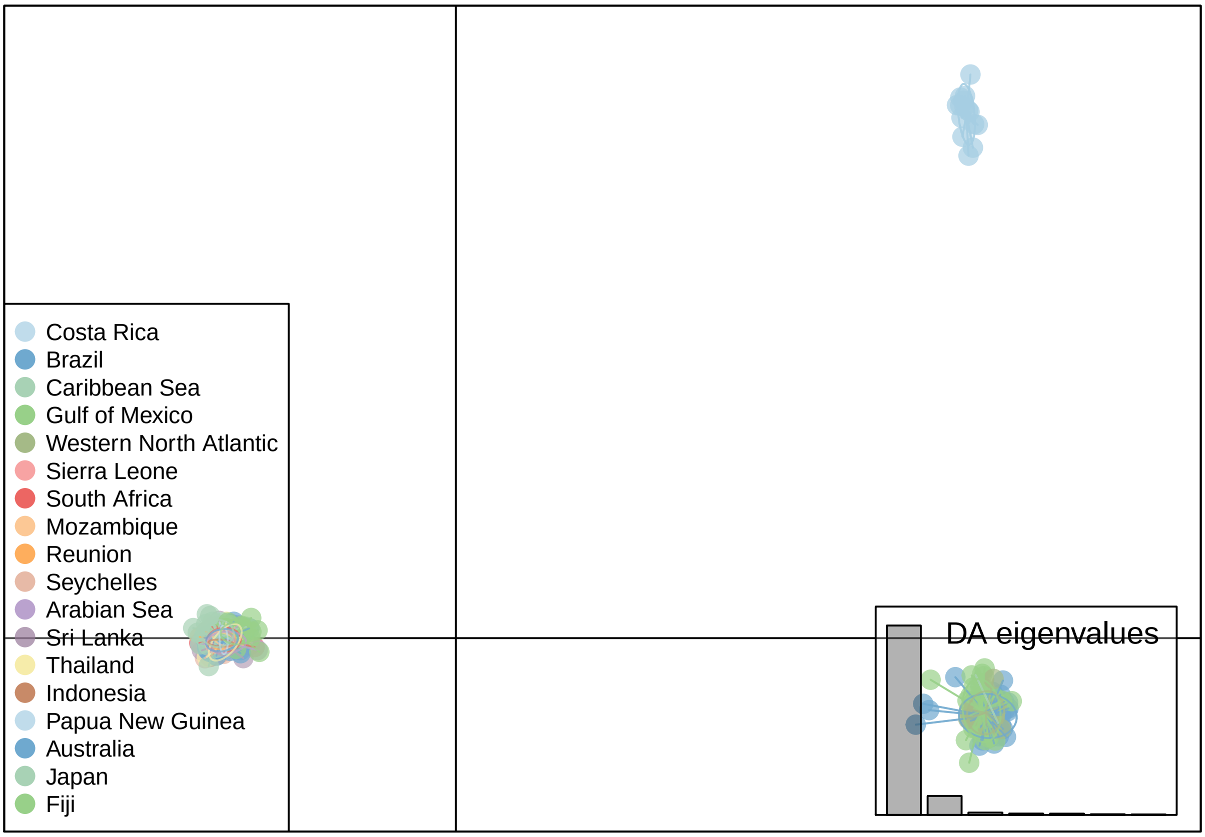
Costa Rica
Brazil
Caribbean Sea
Gulf of Mexico
Western North Atlantic
Sierra Leone
South Africa
Mozambique
Reunion
Seychelles
Arabian Sea
Sri Lanka
Thailand
Indonesia
Papua New Guinea
Australia
Japan
Fiji
DA eigenvalues

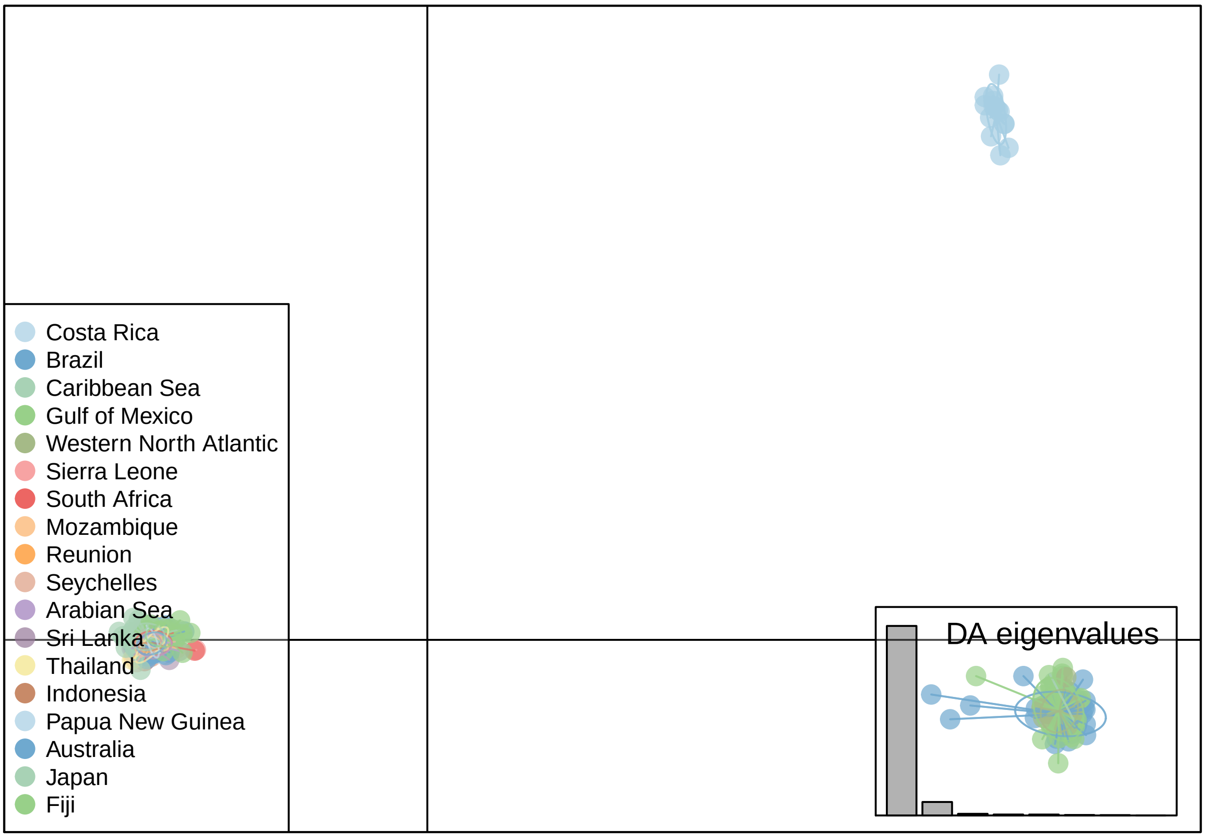


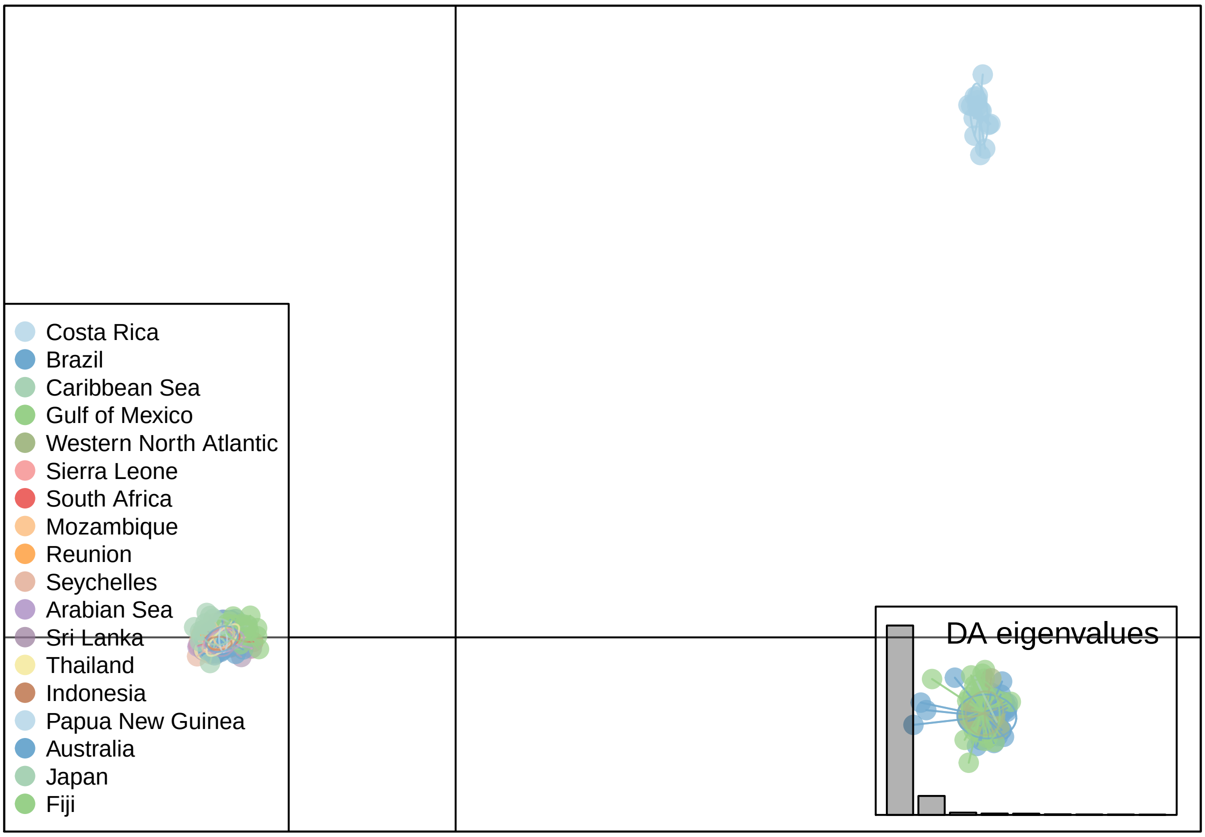


```
for (K in 2:10) {
  colour <- colours.prior[1:K]
  dapc <- get(dapc.all.object.names[K - 1])
  dapc.plot <- ade4::scatter(dapc, cex = 2, legend = TRUE, col = colour,
                             clabel = FALSE, posi.leg = "bottomleft",
```

```
                              scree.pca = FALSE,
                                      posi.pca = "topleft", cleg = 0.75,
                              xax = 1, yax = 2, inset.solid = 0.3)
  dev.print(
    device = png,
    file = paste0("DArTcap_AUS_subset_DAPC_scatterplot_pergroups_K",
                  K,".png"),
    width = 30,
    height = 15,
    units = "cm",
    res = 300
  )
}
```

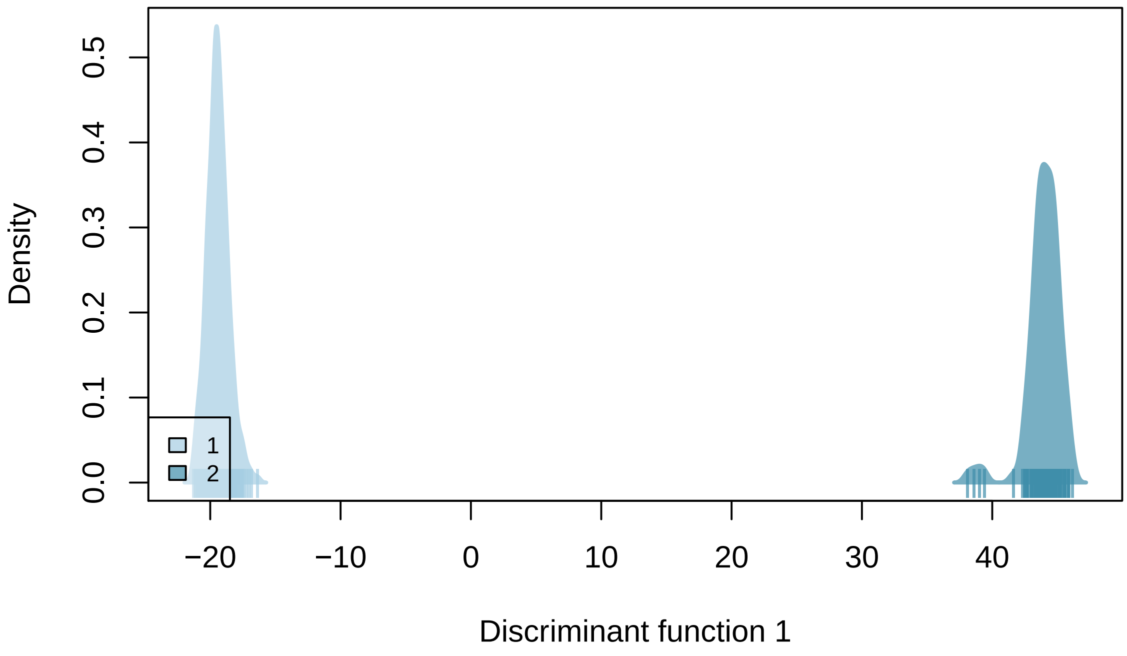

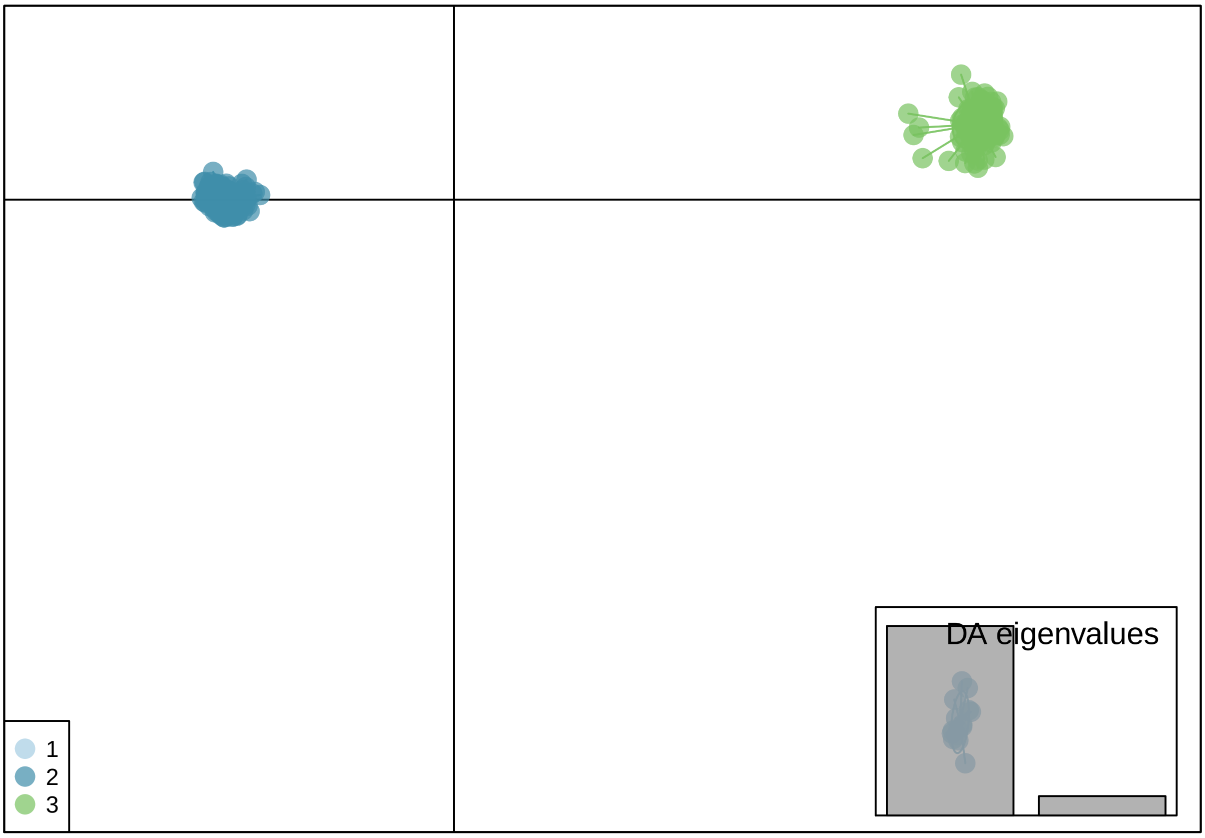
DA eigenvalues
1
2
3

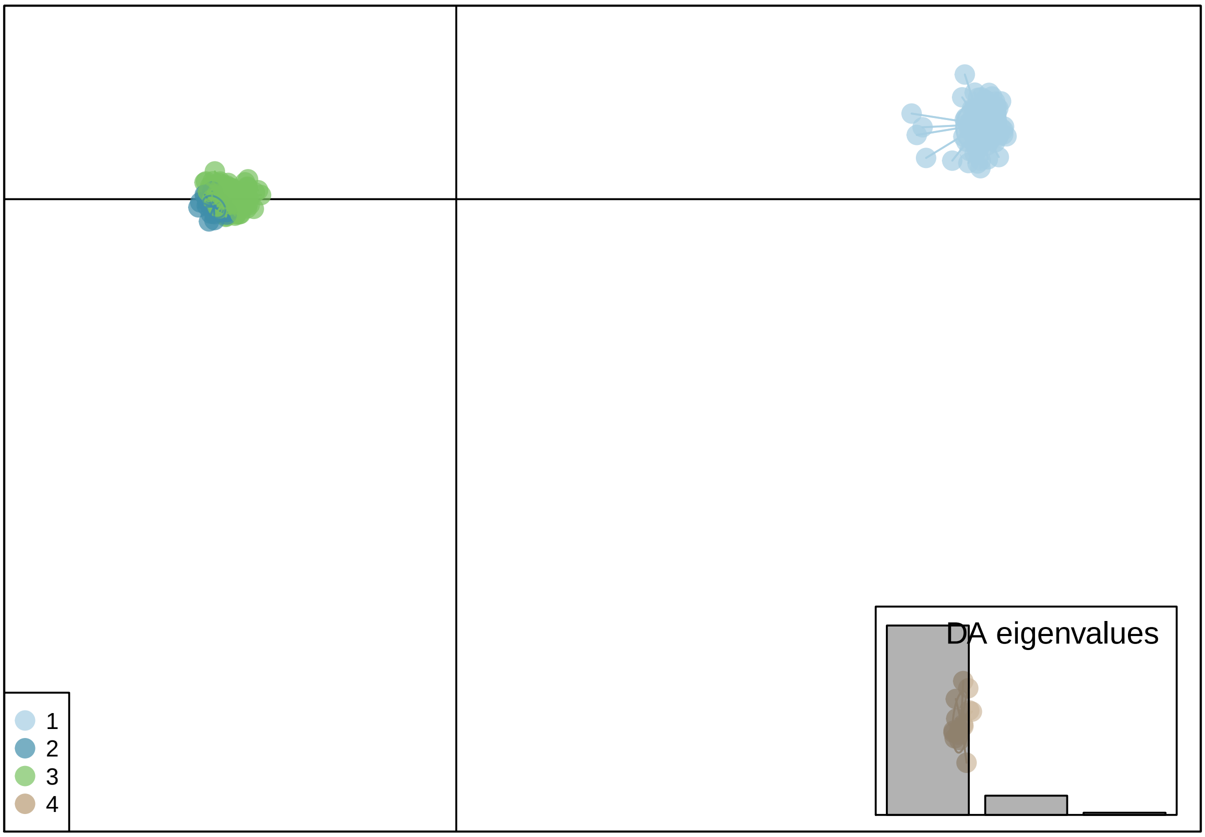
DA eigenvalues
1
2
3
4

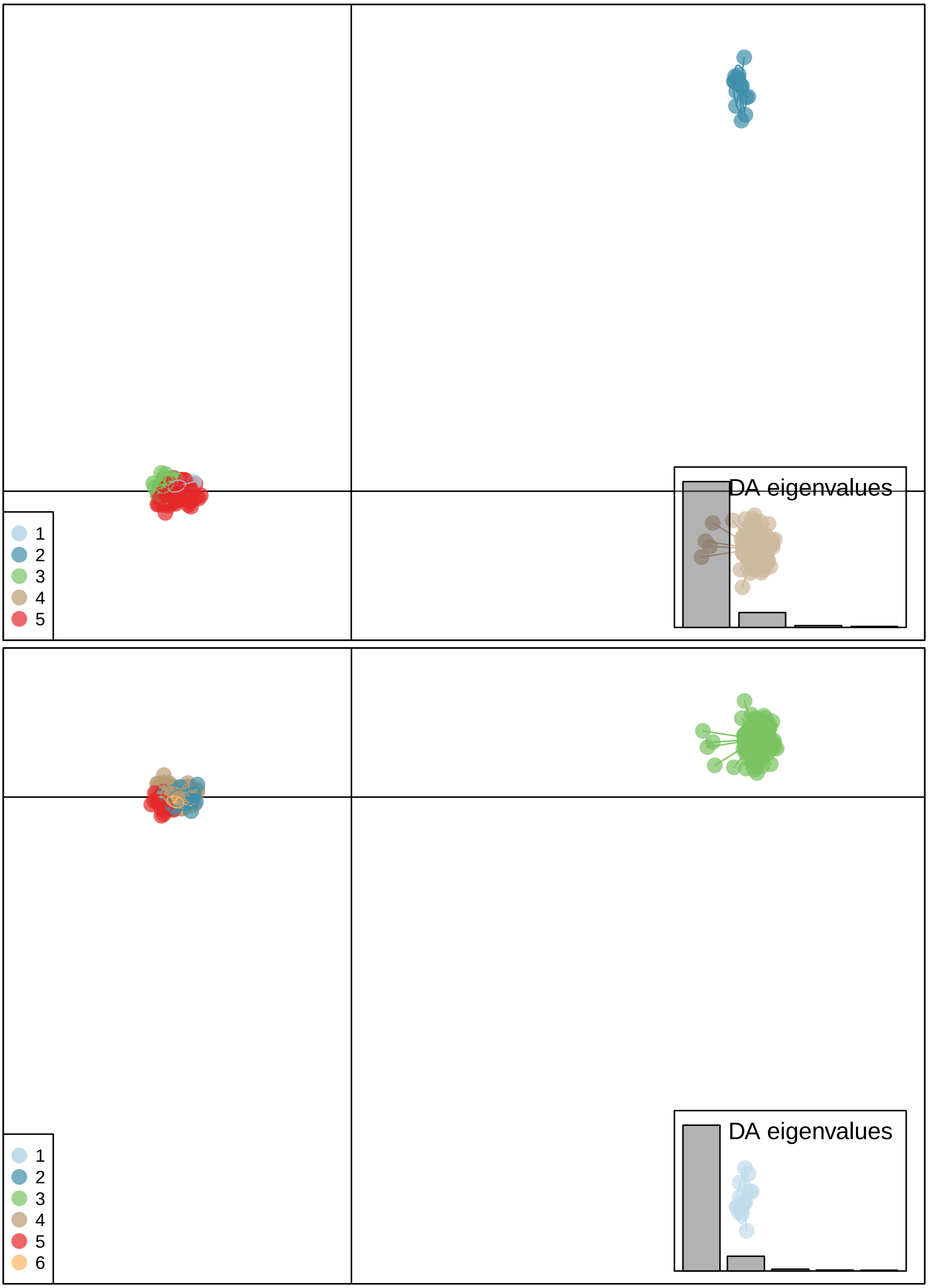
DA eigenvalues
1
2
3
4
5
DA eigenvalues
1
2
3
4
5
6

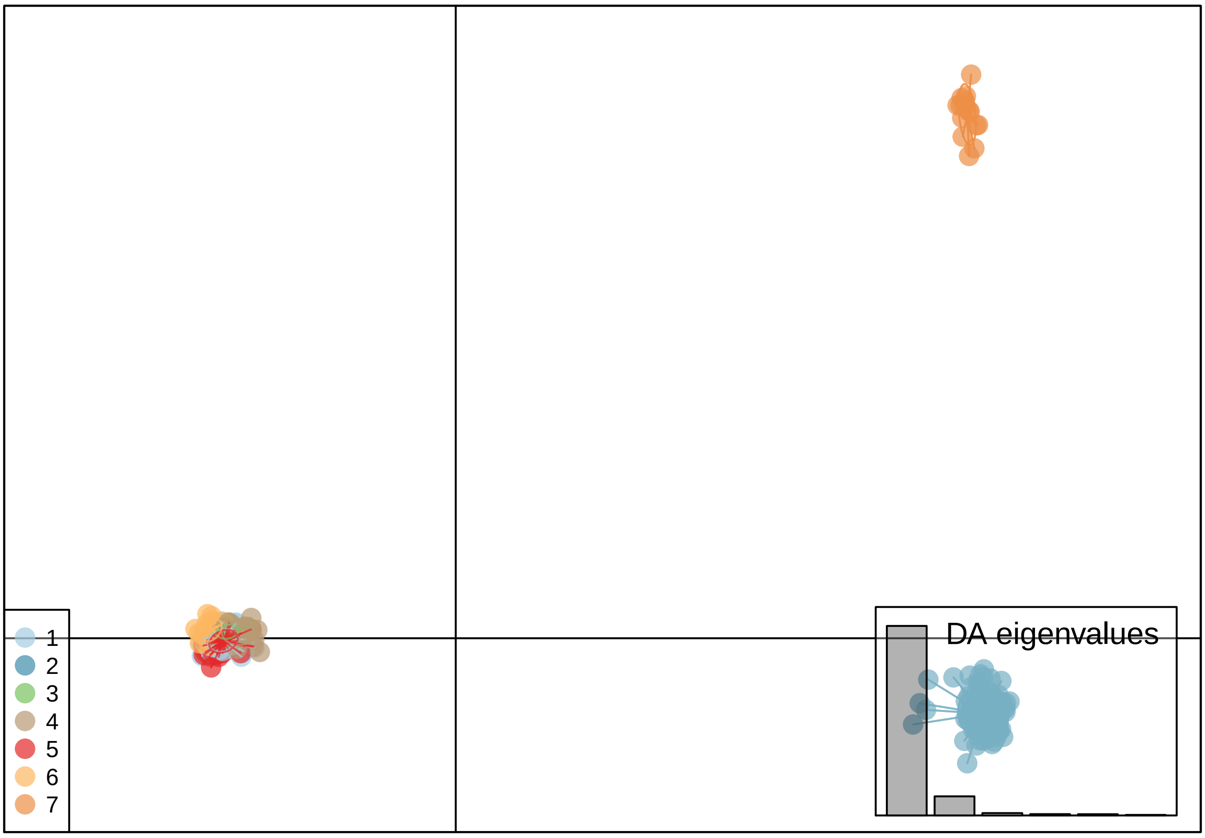
1
2
3
4
5
6
7
DA eigenvalues

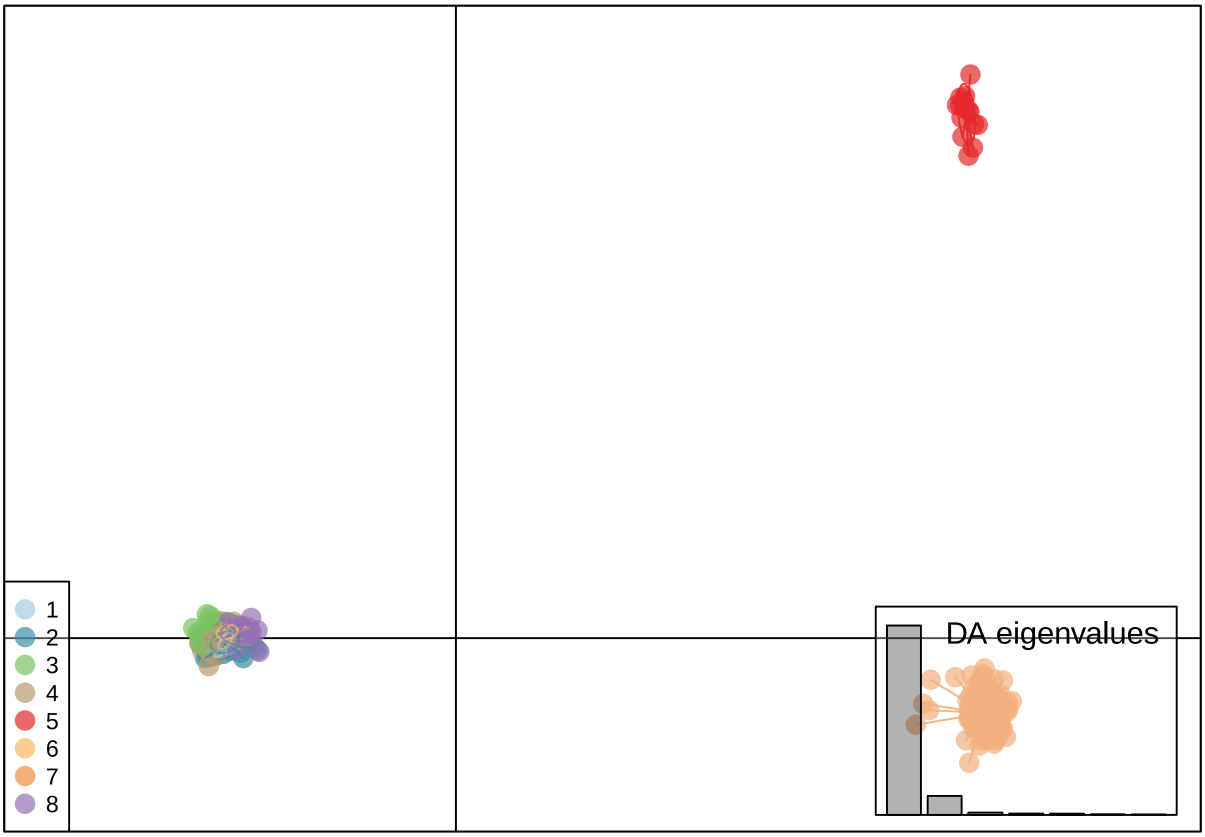
1
2
3
4
5
6
7
8
DA eigenvalues

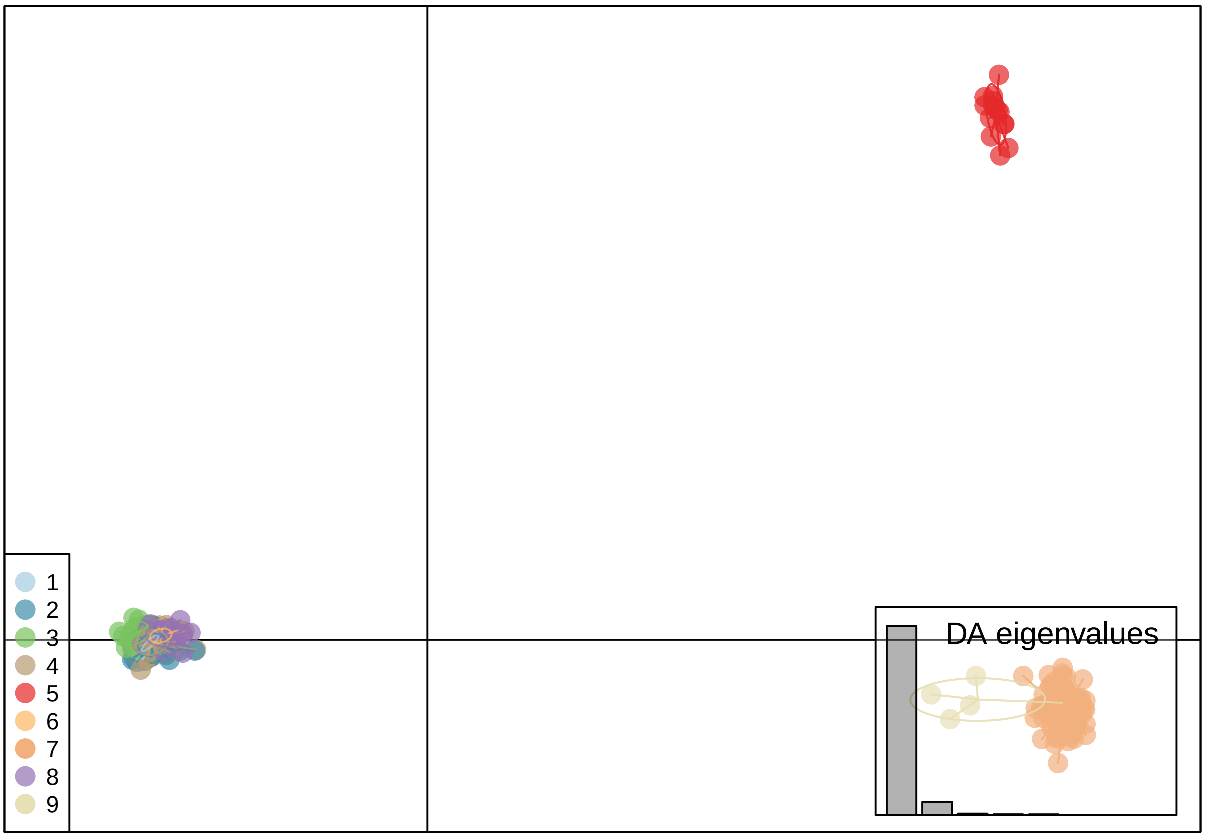


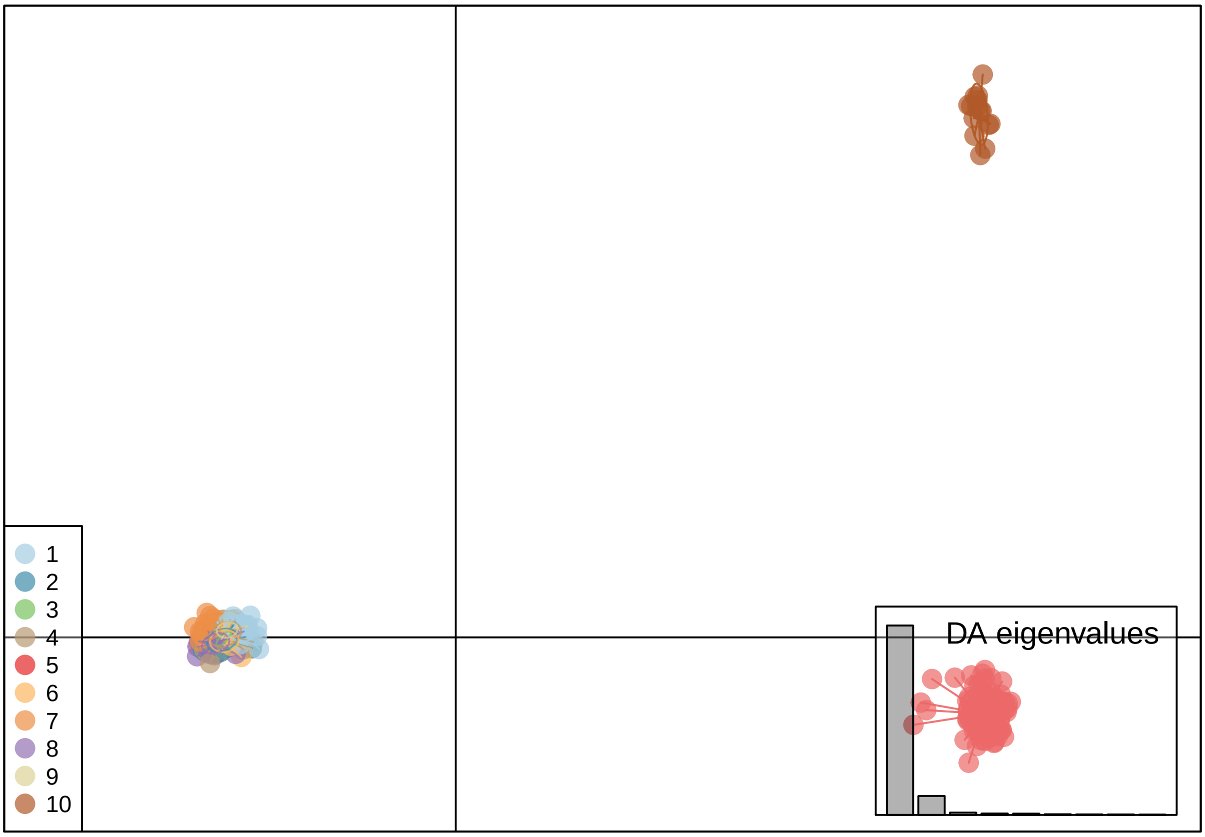


### 9.9.5 DAPC scatterplot - FLO

This code will *colour* individuals according to *sampling location* and **group** individuals according to DAPC **posterior membership**.

```r
for (K in 3:10) {
  dapc <- get(dapc.all.object.names[K - 1])
  x <- dapc$ind.coord[,1]
  y <- dapc$ind.coord[,2]
  group <- paste("Group", as.numeric(dapc$assign))
  pop <- BS.dartcap.sub.gl$pop
  df <- data.frame(x, y, group, pop)
  colnames(df) <- c("x","y","group","pop")
  gg <- merge(df,aggregate(cbind(mean.x = x, mean.y = y)~group, df, mean),
              by = "group")

  plot <- ggplot2::ggplot(gg, ggplot2::aes(x,y,color = pop)) +
    ggplot2::geom_point(size = 5) +
    ggplot2::geom_point(ggplot2::aes(x = mean.x, y = mean.y), size = 5,
                        shape = 17, color = "black") +
    ggplot2::geom_segment(ggplot2::aes(x = mean.x, y = mean.y, xend = x,
                                       yend = y)) +
    ggplot2::scale_colour_manual(values = colours.18) +
    ggplot2::scale_fill_manual(values = colours.18) +
    ggplot2::geom_hline(yintercept = 0) +
    ggplot2::geom_vline(xintercept = 0) +
    ggplot2::labs(subtitle = "",
         y = "PC2",
         x = "PC1",
         title = paste0("K=",K," & PC=",PC, sep = ""),
         caption = "") +
    ggplot2::theme_minimal() +
    ggplot2::theme(
      axis.text = ggplot2::element_text(size = 5),
      axis.title.x = ggplot2::element_text(size = 10),
      axis.title.y = ggplot2::element_text(size = 10),
      legend.text = ggplot2::element_text(size = 7))
  print(plot)
  ggplot2::ggsave(plot,
                  filename = paste0("DArTcap_AUS_subset_DAPC_scatterplot2_K", K, ".png"),
                  width = 30, height = 15,units = "cm")
}
```

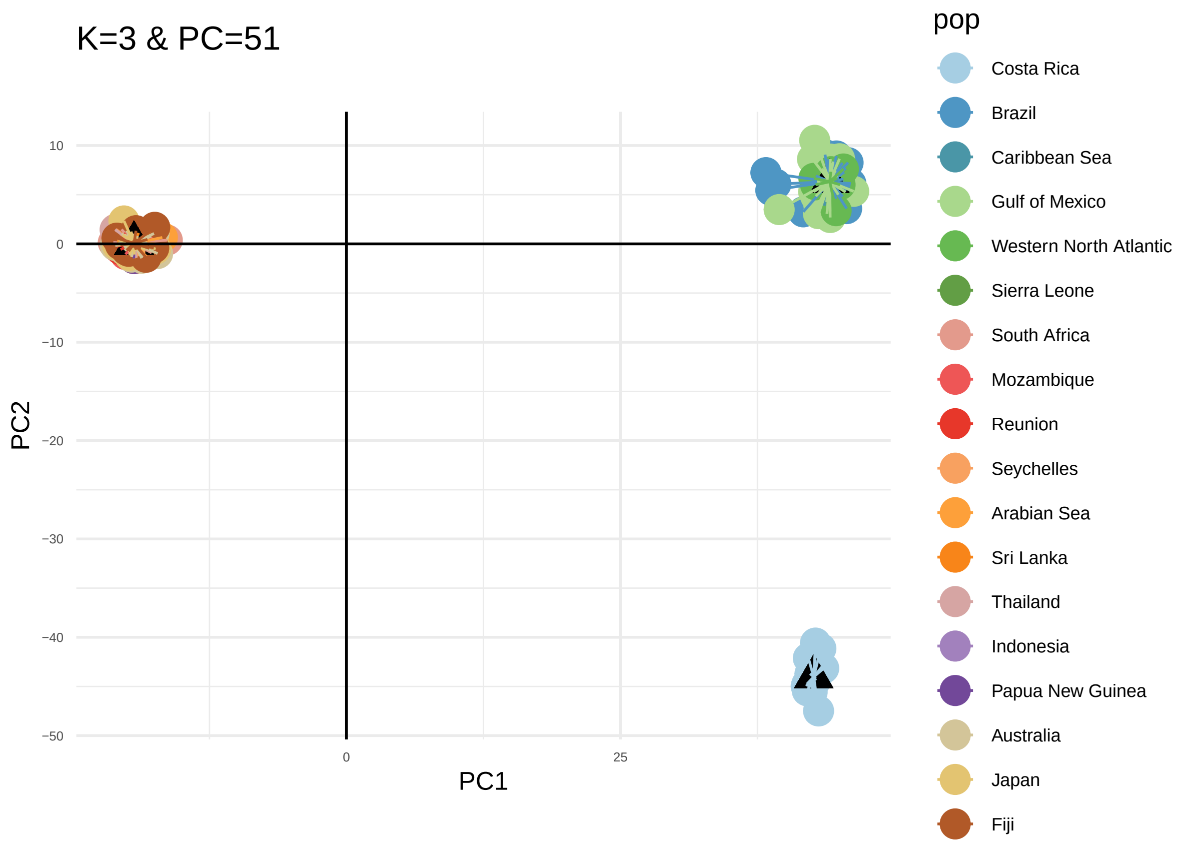
K=3 & PC=51
PC2
PC1
10
0
−10
−20
−30
−40
−50
0
25
pop
Costa Rica
Brazil
Caribbean Sea
Gulf of Mexico
Western North Atlantic
Sierra Leone
South Africa
Mozambique
Reunion
Seychelles
Arabian Sea
Sri Lanka
Thailand
Indonesia
Papua New Guinea
Australia
Japan
Fiji

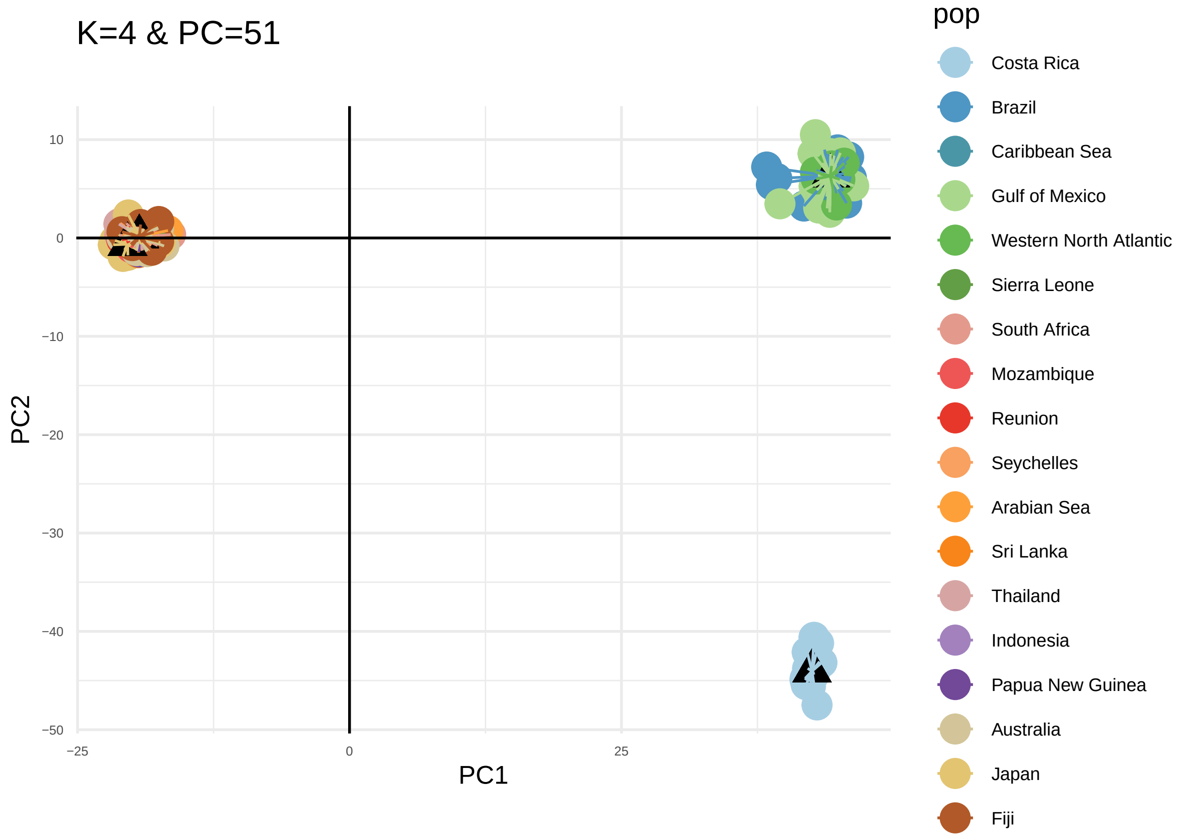
K=4 & PC=51
PC2
PC1
10
0
−10
−20
−30
−40
−50
−25
0
25
pop
Costa Rica
Brazil
Caribbean Sea
Gulf of Mexico
Western North Atlantic
Sierra Leone
South Africa
Mozambique
Reunion
Seychelles
Arabian Sea
Sri Lanka
Thailand
Indonesia
Papua New Guinea
Australia
Japan
Fiji

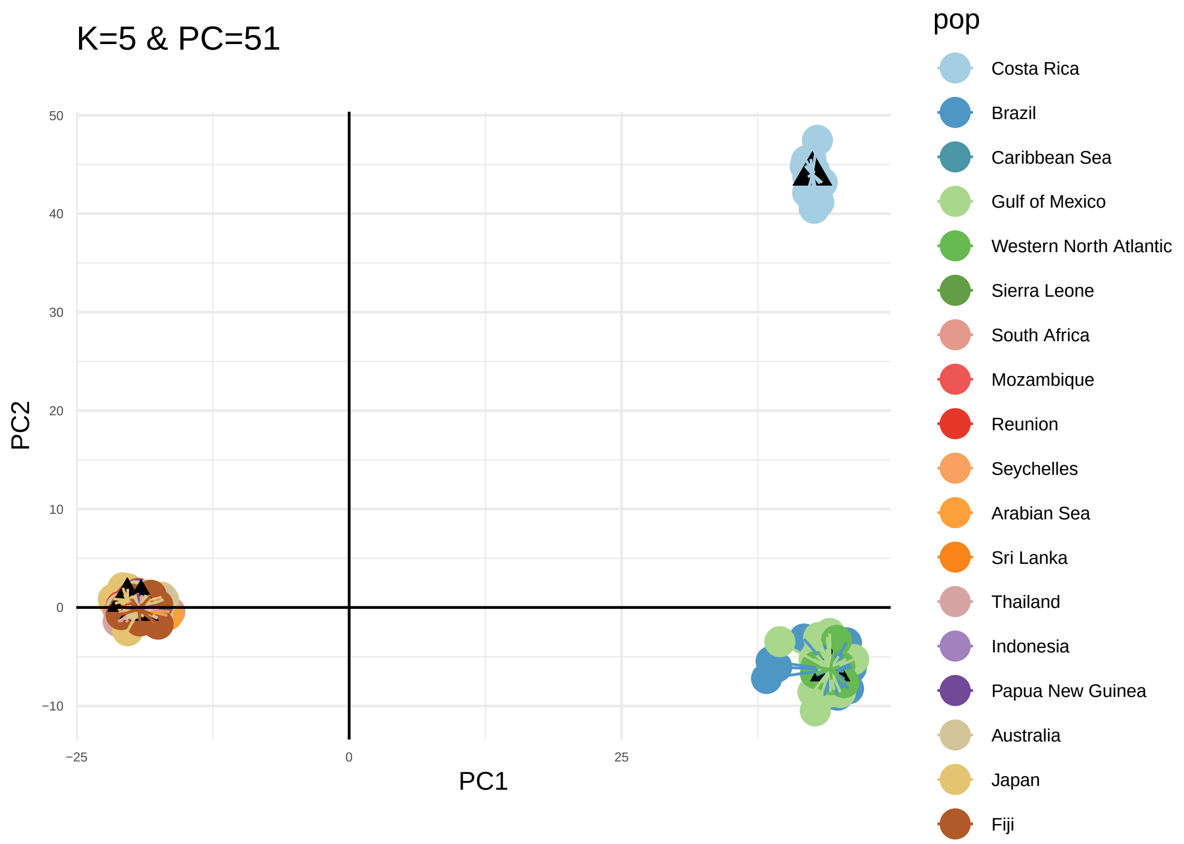
K=5 & PC=51
PC2
50
40
30
20
10
0
−10
−25
0
25
PC1
pop
Costa Rica
Brazil
Caribbean Sea
Gulf of Mexico
Western North Atlantic
Sierra Leone
South Africa
Mozambique
Reunion
Seychelles
Arabian Sea
Sri Lanka
Thailand
Indonesia
Papua New Guinea
Australia
Japan
Fiji

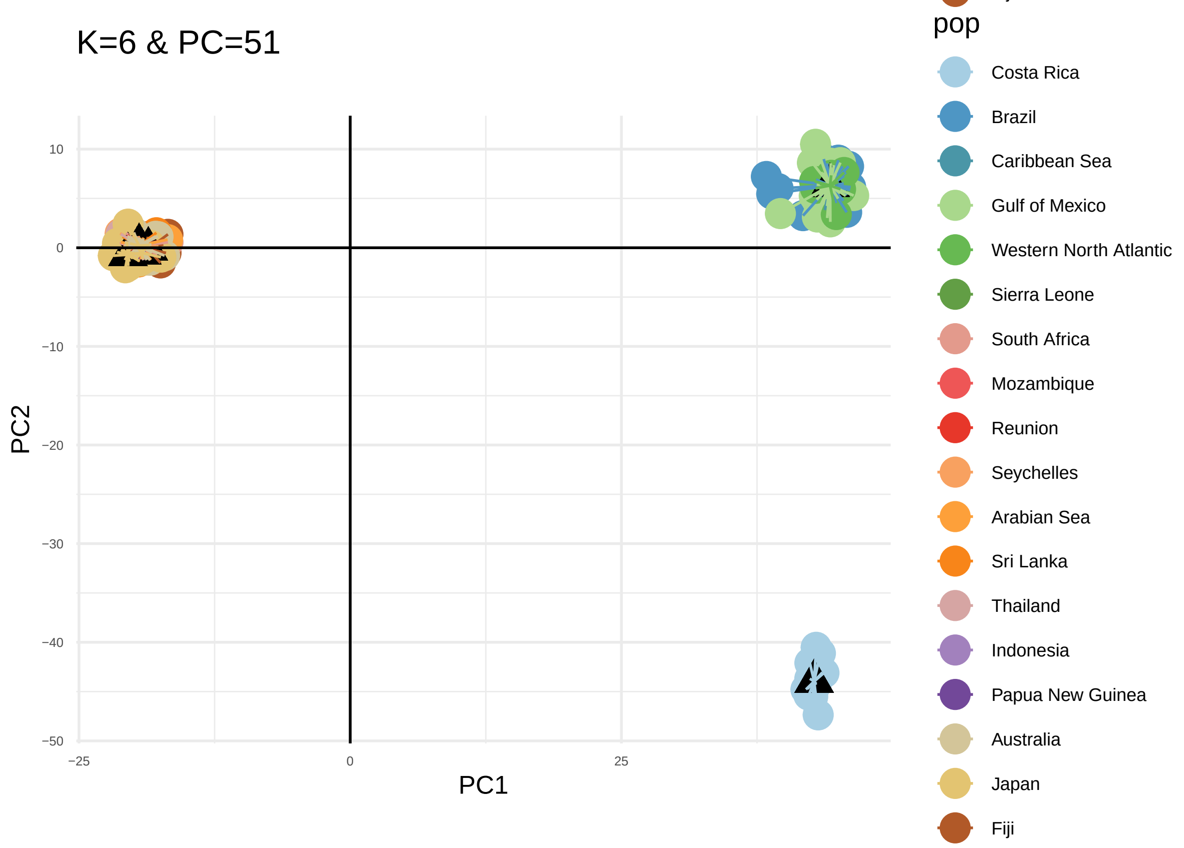
K=6 & PC=51
PC2
10
0
−10
−20
−30
−40
−50
−25
0
25
PC1
pop
Costa Rica
Brazil
Caribbean Sea
Gulf of Mexico
Western North Atlantic
Sierra Leone
South Africa
Mozambique
Reunion
Seychelles
Arabian Sea
Sri Lanka
Thailand
Indonesia
Papua New Guinea
Australia
Japan
Fiji

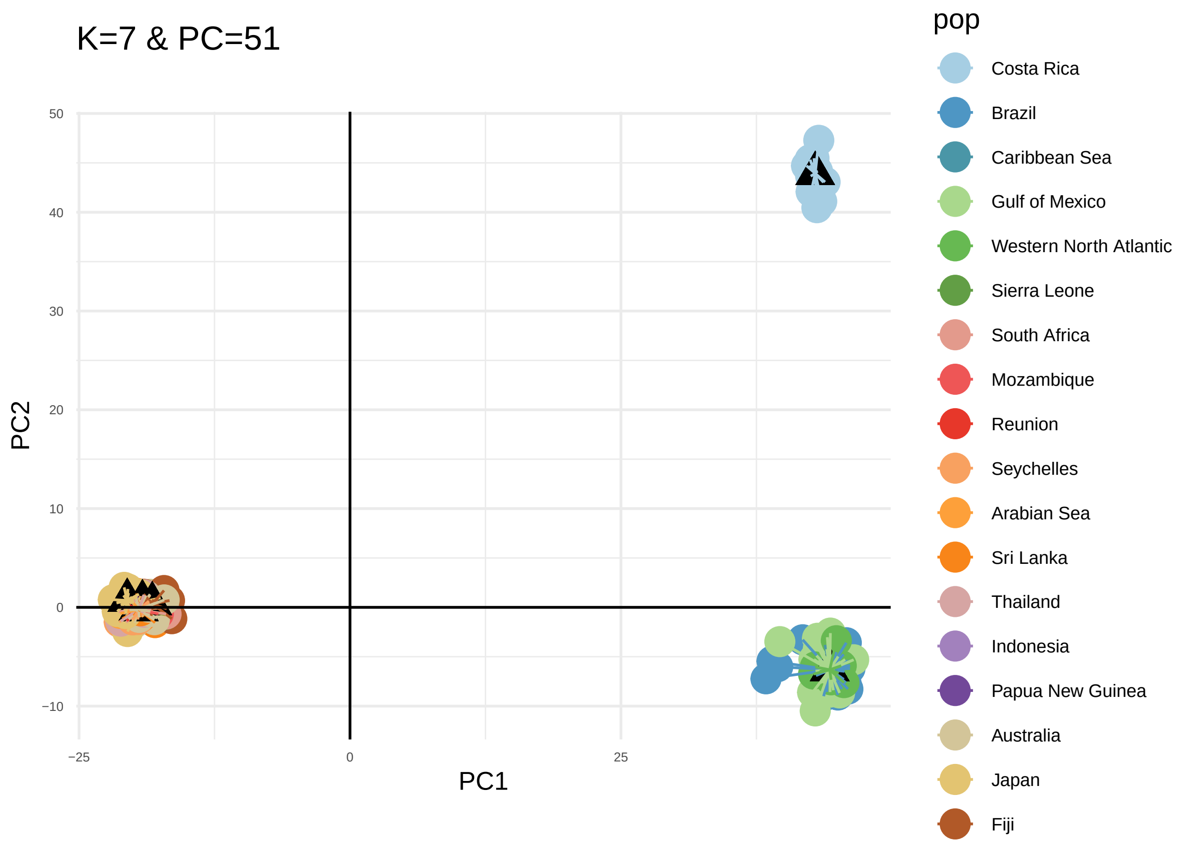
K=7 & PC=51
PC2
PC1
50
40
30
20
10
0
−10
−25
0
25
pop
Costa Rica
Brazil
Caribbean Sea
Gulf of Mexico
Western North Atlantic
Sierra Leone
South Africa
Mozambique
Reunion
Seychelles
Arabian Sea
Sri Lanka
Thailand
Indonesia
Papua New Guinea
Australia
Japan
Fiji

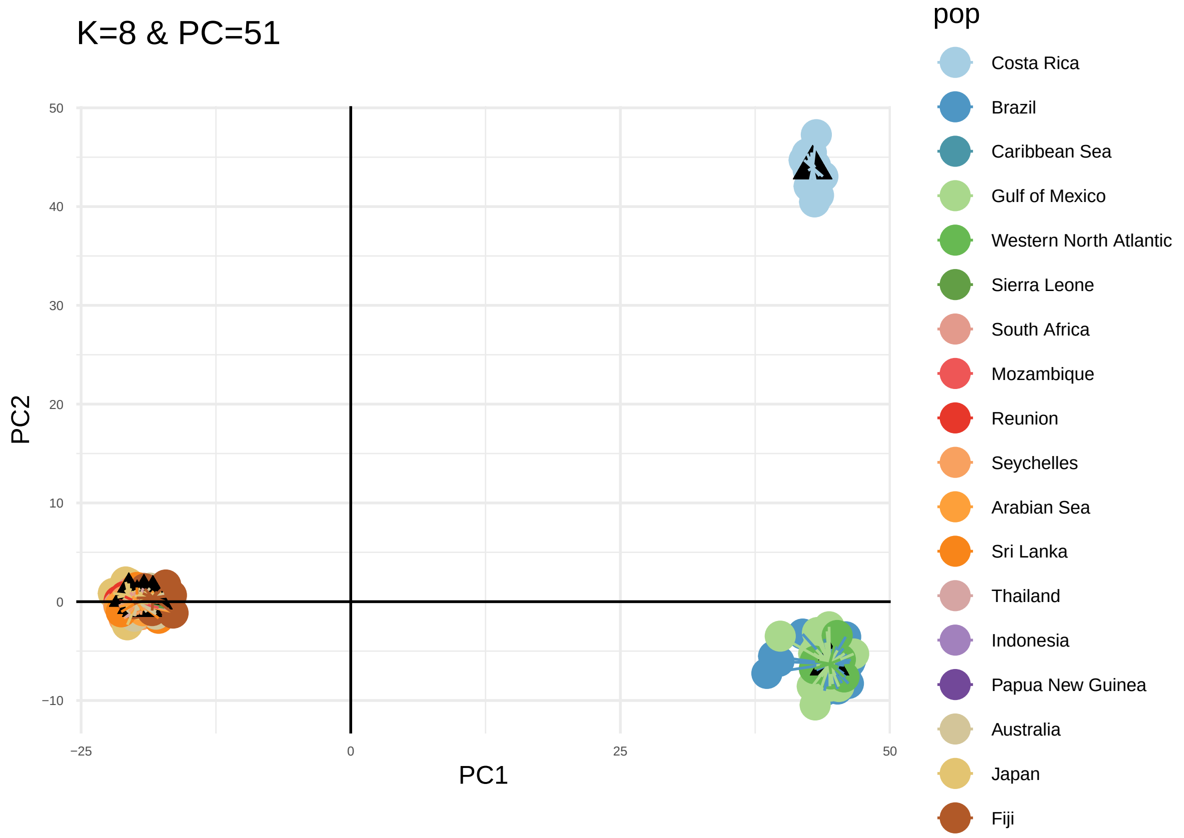
K=8 & PC=51
PC2
PC1
50
40
30
20
10
0
−10
−25
0
25
50
pop
Costa Rica
Brazil
Caribbean Sea
Gulf of Mexico
Western North Atlantic
Sierra Leone
South Africa
Mozambique
Reunion
Seychelles
Arabian Sea
Sri Lanka
Thailand
Indonesia
Papua New Guinea
Australia
Japan
Fiji

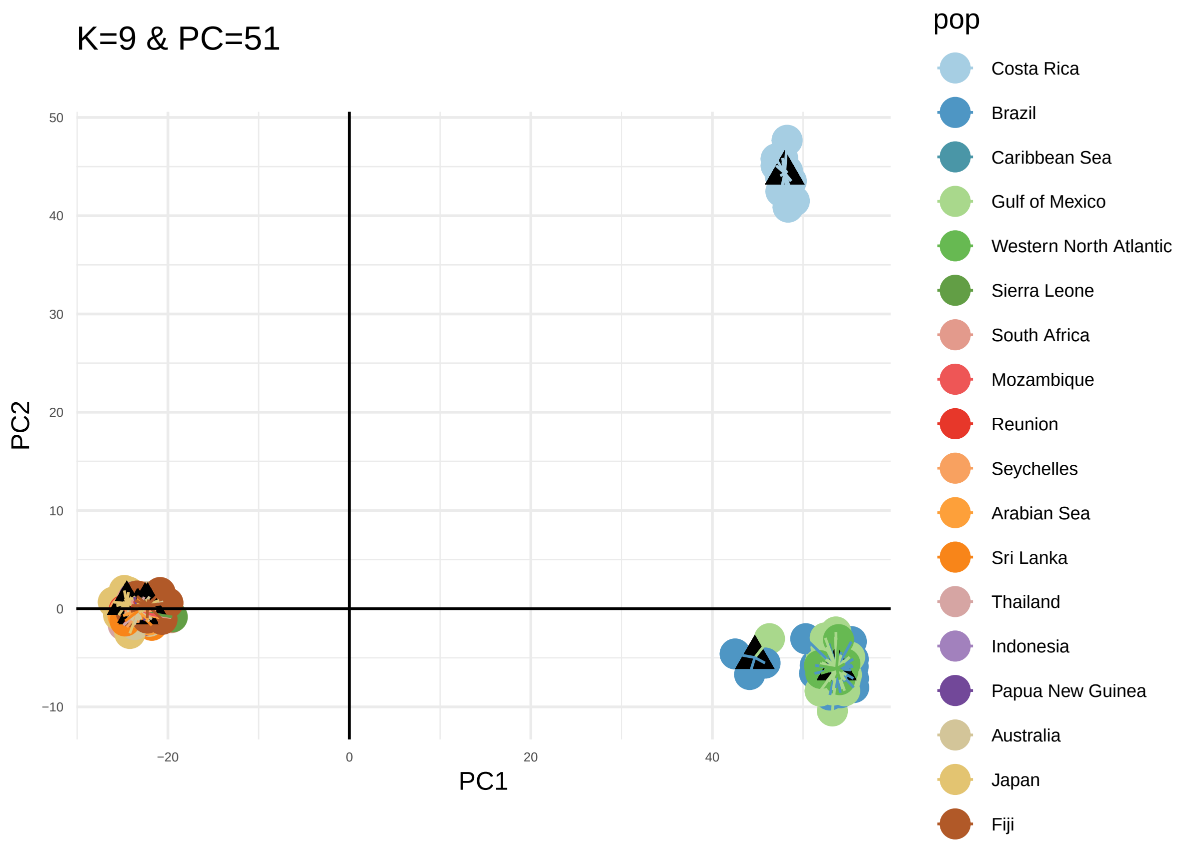
K=9 & PC=51
PC2
PC1
50
40
30
20
10
0
−10
−20
0
20
40
pop
Costa Rica
Brazil
Caribbean Sea
Gulf of Mexico
Western North Atlantic
Sierra Leone
South Africa
Mozambique
Reunion
Seychelles
Arabian Sea
Sri Lanka
Thailand
Indonesia
Papua New Guinea
Australia
Japan
Fiji

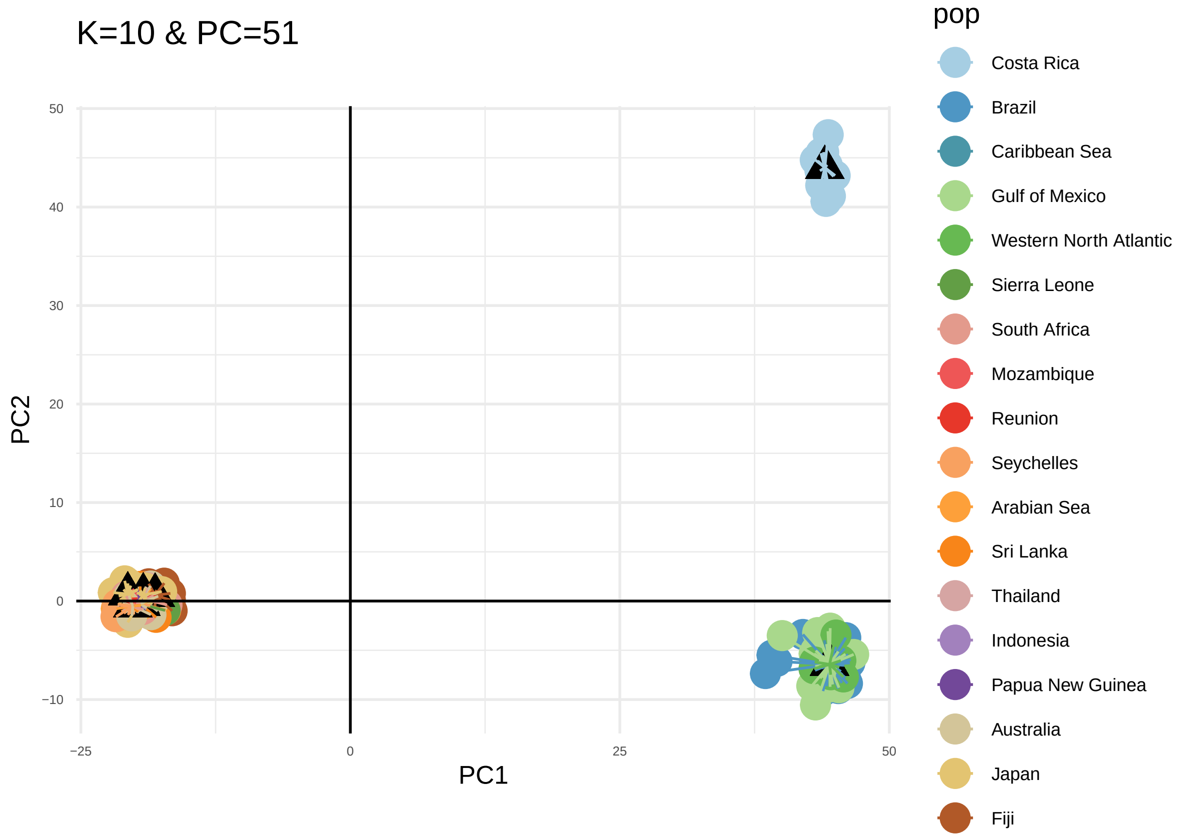
K=10 & PC=51
PC2
PC1
50
40
30
20
10
0
−10
−25
0
25
50
pop
Costa Rica
Brazil
Caribbean Sea
Gulf of Mexico
Western North Atlantic
Sierra Leone
South Africa
Mozambique
Reunion
Seychelles
Arabian Sea
Sri Lanka
Thailand
Indonesia
Papua New Guinea
Australia
Japan
Fiji

# 10 DATA4: DArTcap - West-Atlantic

## 10.1 Set strata

```
strata <- radiator::read_strata("Bull_shark_DArTcap_strata.tsv",
                                keep.two = FALSE)$strata %>%
  dplyr:: filter(SpeciesID_genetic == "Carcharhinus leucas") %>%
  dplyr:: filter(Region == "W-ATL")
readr::write_tsv(strata, path = "Bull_shark_DArTcap_strata4_WATL.tsv")
```

## 10.2 Filter data

1. Marker Reproducibility: 0.98
2. Individual missingness: 0.35
3. Individual Het - low: 0
4. Individual Het - high: 0.07
5. Individual total coverage - low: 10,000
6. Individual total coverage - high: 1,000,000
7. Marker MAC - low: 3
8. Marker coverage - low: 5
9. Marker coverage - high:65
10. Marker maximum missing proportion: 0.1
11. Marker snp position on locus: n
12. Max 2 SNPs per locus
13. 1 SNP per locus according to mac
14. no LD filter
15. Individual Het - low: 0.141
16. Individual Het - high: 0.200
17. Duplicated individuals: 0.35
18. No filter on HWE

```
dartcap.data <- "SEQ_SNPs_counts_0_Target_merge.csv"
strata <- "Bull_shark_DArTcap_strata4_WATL.tsv"
BSsnp <- radiator::filter_rad(dartcap.data, strata,
                              parallel.core = 1)
BS.dartcap.WATL.gds <- BSsnp$gds
BS.dartcap.WATL.tidy <- BSsnp$output$tidy.data
print(BS.dartcap.WATL.gds, expand = TRUE, all = TRUE, attribute = TRUE,
      attribute.trim = FALSE)

save(BS.dartcap.WATL.tidy, file = "DArTcap_WATL.Rdata")
```

Table 39:

| FILTERS | VALUES | BEFORE | BLACKLIST |
|---|---|---|---|
| Filter DArT reproducibility | 0.98 | 125 / 4 / 1 / 26335 / 37537 | 0 / 0 / 0 / 1043 / 2250 |
| Filter monomorphic markers | NA | 125 / 4 / 1 / 25292 / 35287 | 0 / 0 / 0 / 19147 / 28615 |
| Filter markers in common | NA | 125 / 4 / 1 / 6145 / 6672 | 0 / 0 / 0 / 384 / 506 |
| Filter individuals based on missingness | 0.35 | 125 / 4 / 1 / 5761 / 6166 | 12 / 0 / 0 / 0 / 0 |
| Filter individuals based on heterozygosity | 0 0.08 | 113 / 4 / 1 / 5761 / 6166 | 0 / 0 / 0 / 0 / 0 |
| Filter individuals based on total coverage | 10000 1e+06 | 113 / 4 / 1 / 5761 / 6166 | 3 / 0 / 0 / 0 / 0 |
| Filter monomorphic markers | NA | 110 / 4 / 1 / 5761 / 6166 | 0 / 0 / 0 / 1074 / 1201 |
| Filter MAC | 3 | 110 / 4 / 1 / 4687 / 4965 | 0 / 0 / 0 / 3182 / 3402 |
| Filter coverage min / max | 5-65 | 110 / 4 / 1 / 1505 / 1563 | 0 / 0 / 0 / 486 / 507 |
| Filter genotyping | 0.1 | 110 / 4 / 1 / 1019 / 1056 | 0 / 0 / 0 / 74 / 100 |
| Filter SNPs position on the read | all | 110 / 4 / 1 / 945 / 956 | 0 / 0 / 0 / 0 / 0 |
| Filter markers snp number | 2 | 110 / 4 / 1 / 945 / 956 | 0 / 0 / 0 / 0 / 0 |
| Filter short ld | mac | 110 / 4 / 1 / 945 / 956 | 0 / 0 / 0 / 0 / 11 |
| detect mixed genomes | 0.141 0.2 | 110 / 4 / 1 / 945 / 945 | 8 / 0 / 0 / 0 / 0 |
| Filter monomorphic markers | NA | 102 / 4 / 1 / 945 / 945 | 0 / 0 / 0 / 0 / 0 |
| detect duplicate genomes | 0.35 | 102 / 4 / 1 / 945 / 945 | 11 / 0 / 0 / 0 / 0 |
| Filter monomorphic markers | NA | 91 / 4 / 1 / 945 / 945 | 0 / 0 / 0 / 3 / 3 |
| Filter HWE | NA | 91 / 4 / 1 / 942 / 942 | 0 / 0 / 0 / 0 / 0 |

### 10.2.1 Remove extra low and high heterozygous individuals

Because the average heterozygosity per sampling location is not even, certain individuals cannot be excluded without removing 'good' individuals.

```
rm <-  c("CL-ARS021-F","CL-GOM025-F.1","CL-GOM012-F","CL-SAF014-M","CL-SAF002-F",
         "CL-BRZ038-U","CLF003-F","CL-GOM011-F","CL-SAF015-F","CL-SAF003-M",
         "CL-PNG005-U","CLCR056-M","CLCR047.1","CLCR055-M","CLCR066-M")

BS.dartcap.WATL.tidy <- BS.dartcap.WATL.tidy[
  !BS.dartcap.WATL.tidy$INDIVIDUALS %in% rm,]
length(unique(BS.dartcap.WATL.tidy$INDIVIDUALS)) # 91
```

'

## 10.3 Save data

```
Convert <- radiator::genomic_converter(BS.dartcap.WATL.tidy,
                                       output = c("genlight", "stockr",
                                                  "pcadapt", "gtypes", "rubias"))
BS.dartcap.WATL.gl <- Convert$genlight
BS.dartcap.WATL.stkr <- Convert$stockr
BS.dartcap.WATL.pc <- Convert$pcadapt
BS.dartcap.WATL.gt <- Convert$gtypes
BS.dartcap.WATL.rubias <- Convert$rubias


markers.meta <- radiator::extract_markers_metadata(BS.dartcap.WATL.gds,
                                                   whitelist = TRUE)
individuals.meta <- radiator::extract_individuals_metadata(BS.dartcap.WATL.gds,
                                                           whitelist = TRUE)
individuals.meta <- individuals.meta[individuals.meta$INDIVIDUALS %in%
                                       BS.dartcap.WATL.gl$ind.names,]
BS.dartcap.WATL.gl$other$ind.metrics <- individuals.meta
BS.dartcap.WATL.gl$other$loc.metrics <- markers.meta
BS.dartcap.WATL.gt@schemes <- individuals.meta

pop.levels <- c("Brazil","Caribbean Sea","Gulf of Mexico",  "Western North Atlantic")
BS.dartcap.WATL.gl$pop <- factor(BS.dartcap.WATL.gl$other$ind.metrics$Site2,
                                 levels = pop.levels)
BS.dartcap.WATL.gl <- BS.dartcap.WATL.gl[order(BS.dartcap.WATL.gl$pop,
                                               BS.dartcap.WATL.gl$ind.names),]
BS.dartcap.WATL.gl <- BS.dartcap.WATL.gl[order(BS.dartcap.WATL.gl$pop,
                                               BS.dartcap.WATL.gl$ind.names)]
BS.dartcap.WATL.rubias$repunit <- factor(BS.dartcap.WATL.rubias$repunit,
                                         levels = pop.levels)

save(BS.dartcap.WATL.tidy, BS.dartcap.WATL.gl, BS.dartcap.WATL.stkr,
     BS.dartcap.WATL.pc,BS.dartcap.WATL.gt,BS.dartcap.WATL.rubias,
     markers.meta, individuals.meta, file = "DArTcap_WATL.Rdata")
```

## 10.4 Load data

```
load("DArTcap_WATL.Rdata")
pop.levels <- c("Brazil","Caribbean Sea","Gulf of Mexico",  "Western North Atlantic")

BS.dartcap.WATL.gl$pop <- factor(BS.dartcap.WATL.gl$other$ind.metrics$Site,
                                 levels = pop.levels)
```

```r
BS.dartcap.WATL.gl <- BS.dartcap.WATL.gl[order(BS.dartcap.WATL.gl$pop,
                                               BS.dartcap.WATL.gl$ind.names)]

pop.levels1 <- stringr::str_replace_all(pop.levels, " ", "_")
BS.dartcap.WATL.tidy$POP_ID <- factor(BS.dartcap.WATL.tidy$POP_ID,
                                      levels = pop.levels1)
BS.dartcap.WATL.tidy <- BS.dartcap.WATL.tidy[order(BS.dartcap.WATL.tidy$POP_ID,
                                                   BS.dartcap.WATL.tidy$INDIVIDUALS),]

adegenet::nInd(BS.dartcap.WATL.gl) # 91
adegenet::nLoc(BS.dartcap.WATL.gl) # 942
summary(BS.dartcap.WATL.gl$pop)
sum(duplicated(BS.dartcap.WATL.gl$other$ind.metrics$`Genetic code`))# 0 replicate left
sum(baits$CloneID %in% BS.dartcap.WATL.gl$other$loc.metrics$LOCUS) #24
```

```
## [1] 91
## [1] 942
##                  Brazil          Caribbean Sea         Gulf of Mexico
##                      47                      2                     34
## Western North Atlantic
##                      8
## [1] 0
## [1] 24
```

### 10.5 Remove sex-linked markers

```r
load("Sex-linked_marker_DArTcap.Rdata")
load("Sex_results.Rdata")
sum(bull_shark$sexy.summary$CLONE_ID %in% SLM$sexy.summary$CLONE_ID) #33
sum(SLM$sexy.summary$CLONE_ID %in% bull_shark$sexy.summary$CLONE_ID) #23
sex.markers <- unique(c(bull_shark$sexy.summary$CLONE_ID, SLM$sexy.summary$CLONE_ID))

sum(BS.dartcap.WATL.gl$loc.names %in% sex.markers) #11
locnames <- BS.dartcap.WATL.gl$loc.names
BS.dartcap.WATL.gl <- BS.dartcap.WATL.gl[,!locnames %in% sex.markers]
BS.dartcap.WATL.gl$other$loc.metrics <- BS.dartcap.WATL.gl$other$loc.metrics[
  !locnames %in% sex.markers,]

sum(sex.markers %in% unique(BS.dartcap.WATL.tidy$LOCUS)) # 11
BS.dartcap.WATL.tidy <- BS.dartcap.WATL.tidy[!BS.dartcap.WATL.tidy$LOCUS %in%
                                               sex.markers,]

locus.gt <- stringr::str_match(pattern = "__\\s*(.*?)\\s*__",
                               string =  BS.dartcap.WATL.gt@data$locus)[,2]
BS.dartcap.WATL.gt <- BS.dartcap.WATL.gt[,!locus.gt %in% sex.markers, ,
                                         drop = TRUE]

locus.rubias <- stringr::str_match(pattern = "__\\s*(.*?)\\s*__",
                                   string =  colnames(BS.dartcap.WATL.rubias))[,2]
BS.dartcap.WATL.rubias <- BS.dartcap.WATL.rubias[,!locus.rubias %in% sex.markers]
```

```
## [1] 33
## [1] 23
## [1] 11
## [1] 11
```

## 10.6 Genetic diversity

```
set.seed(124)
pop.levels <- c("Brazil","Caribbean Sea","Gulf of Mexico", "Western North Atlantic")

gl <- BS.dartcap.WATL.gl
gl$pop <- factor(gl$other$ind.metrics$Site, levels = pop.levels)
gl <- gl[order(gl$pop,gl$ind.names)]


gl2gpop(gl, filename = "BS_dartcap_WATL_genepop.txt")
bastat <-diveRsity::basicStats(infile = "BS_dartcap_WATL_genepop.txt",
                               outfile = "BS_dartcap_WATL_BasicStat_output.txt",
                               fis_ci = TRUE,ar_ci = TRUE, fis_boots = 1000,
                               ar_boots = 1000,mc_reps = 1000,
                               rarefaction = FALSE, ar_alpha = 0.05,
                               fis_alpha = 0.05)

length(bastat$main_tab)#4
gendiv <- data.frame(
  "Brazil" = bastat$main_tab[[1]]$overall,
  "Caribbean Sea" = bastat$main_tab[[2]]$overall,
  "Gulf of Mexico" = bastat$main_tab[[3]]$overall,
  "Western North Atlantic" = bastat$main_tab[[4]]$overall,
  check.names = FALSE)
row.names(gendiv) <- rownames(bastat$main_tab[[1]])

df <- as.data.frame(gl)
df[df == "0"] <- "11"
df[df == "1"] <- "12"
df[df == "2"] <- "22"
gen.gi <- adegenet::df2genind(df, ploidy = 2,ncode = 2, pop = gl$pop)
ris_basic <- hierfstat::basic.stats(gen.gi)
fis.hierfstat <- apply(ris_basic$Fis, 2, mean, na.rm=T)
fis.CI <- hierfstat::boot.ppfis(gen.gi, nboot = 1000, quant = c(0.025,0.975),
                                diploid = TRUE,dig = 4)
df <- data.frame(fis.hierfstat,fis.CI.low = fis.CI$fis.ci[,1],
                 fis.CI.high = fis.CI$fis.ci[,2])
df <- as.data.frame(t(df))
gendiv <- rbind(gendiv, df)

readr::write_tsv(gendiv, path = "DArTcap_WATL_gendiv.txt")
save(gendiv, file = "DArTcap_WATL_gendiv.Rdata")
```

```
load("DArTcap_WATL_gendiv.Rdata")
shortnames <- c("BRZ", "CAR", "GOM", "WNA")
knitr::kable(gendiv, col.names = shortnames, digits = 4,caption = "") %>%
  kableExtra::kable_styling(full_width = FALSE,font_size = 5,
                            latex_options = c( "hold_position"))
```

### 10.6.1 Number of monomorphic markers per population

```
pop.levels <- c("Brazil","Caribbean Sea","Gulf of Mexico", "Western North Atlantic")

monomorphics <- c()
for (i in 1:length(pop.levels)) {
  x <- BS.dartcap.WATL.gl[BS.dartcap.WATL.gl$pop == pop.levels[i]]
  x2 <- dartR::gl.filter.monomorphs(x, verbose = 0)
```

Table 40:

| | BRZ | CAR | GOM | WNA |
|---|---|---|---|---|
| ar | 1.3270 | 1.2470 | 1.3070 | 1.2960 |
| size | 46.7390 | 1.9940 | 33.7090 | 7.9720 |
| obs_het | 0.1770 | 0.1780 | 0.1680 | 0.1700 |
| exp_het | 0.1820 | 0.1340 | 0.1720 | 0.1640 |
| uexp_het | 0.1840 | 0.1790 | 0.1750 | 0.1750 |
| fis | 0.0300 | -0.3290 | 0.0290 | -0.0390 |
| hwe_glb | 1.0000 | 1.0000 | 1.0000 | 1.0000 |
| hwe_hom | 1.0000 | 1.0000 | 1.0000 | 1.0000 |
| hwe_het | 1.0000 | 1.0000 | 1.0000 | 1.0000 |
| fis_lo | 0.0080 | -1.0000 | 0.0000 | -0.1530 |
| fis_hi | 0.0300 | -0.3290 | 0.0300 | -0.0490 |
| ar_lo | 1.3010 | 1.1680 | 1.1880 | 1.1570 |
| ar_hi | 1.3500 | 1.3230 | 1.3320 | 1.3400 |
| fis.hierfstat | 0.0401 | -0.0997 | 0.0430 | 0.0214 |
| fis.CI.low | 0.0192 | -0.0784 | 0.0208 | -0.0031 |
| fis.CI.high | 0.0538 | 0.0811 | 0.0658 | 0.0719 |

```
  locs <-  adegenet::nLoc(x) - adegenet::nLoc(x2)
  monomorphics[i] <- locs
}
names(monomorphics) <- pop.levels
shortnames <- c("BRZ", "CAR", "GOM", "WNA")
knitr::kable(t(as.table(monomorphics)), col.names = shortnames, digits = 4,
             caption = "") %>%
  kableExtra::kable_styling(full_width = FALSE,font_size = 5,
                            latex_options = c( "hold_position"))
```

Table 41:

| BRZ | CAR | GOM | WNA |
|---|---|---|---|
| 36 | 682 | 144 | 385 |

### 10.6.2 Nucleotide diversity and inbreeding

```
pi.sum <- radiator::pi(
  data = BS.dartcap.WATL.tidy,
  read.length = 70,
  parallel.core = parallel::detectCores() - 1,
  path.folder = NULL,
  verbose = TRUE
)

FIS.sum<- radiator::ibdg_fh(data = BS.dartcap.WATL.tidy,
        path.folder = NULL, verbose = TRUE)

save(pi.sum, FIS.sum, file = "DArTcap_WATL_pi_FIS_Sites.Rdata")
```

```
load("DArTcap_WATL_pi_FIS_Sites.Rdata")
pi.sum$boxplot.pi
```

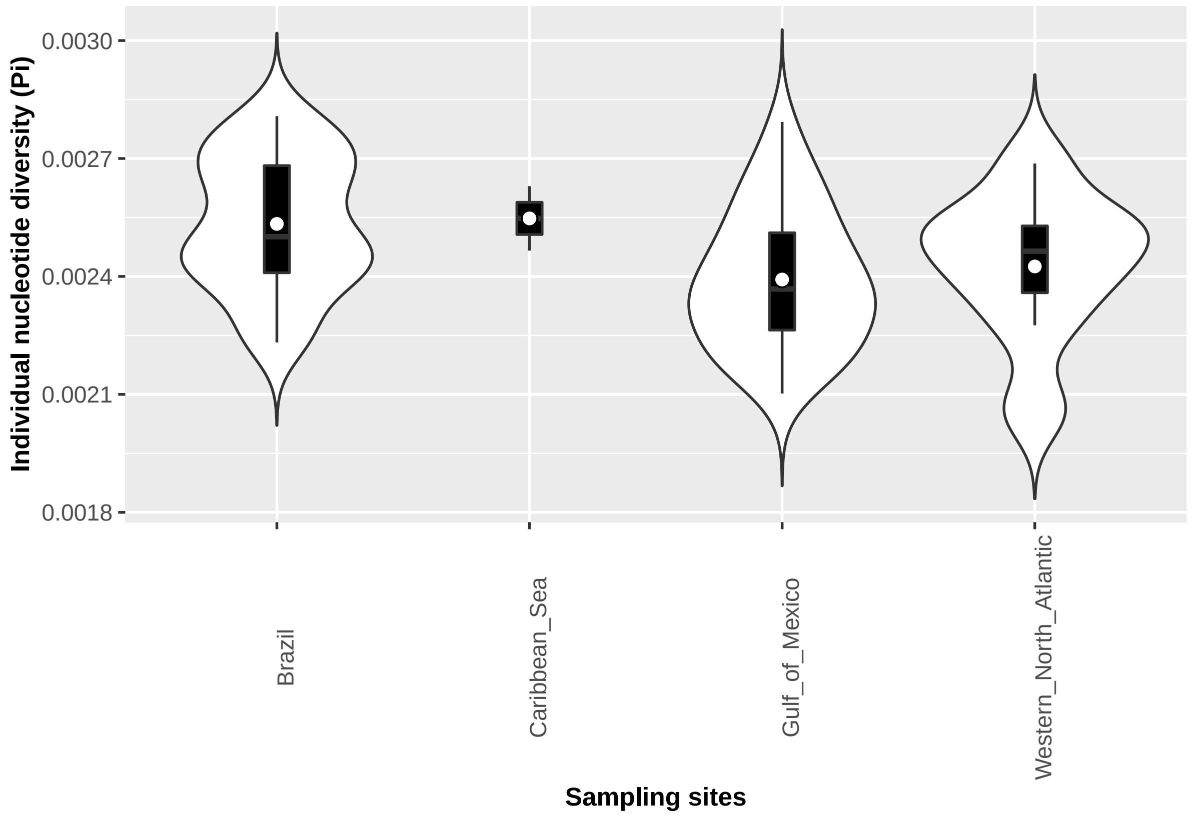


```
FIS.sum$fh.box.plot
```

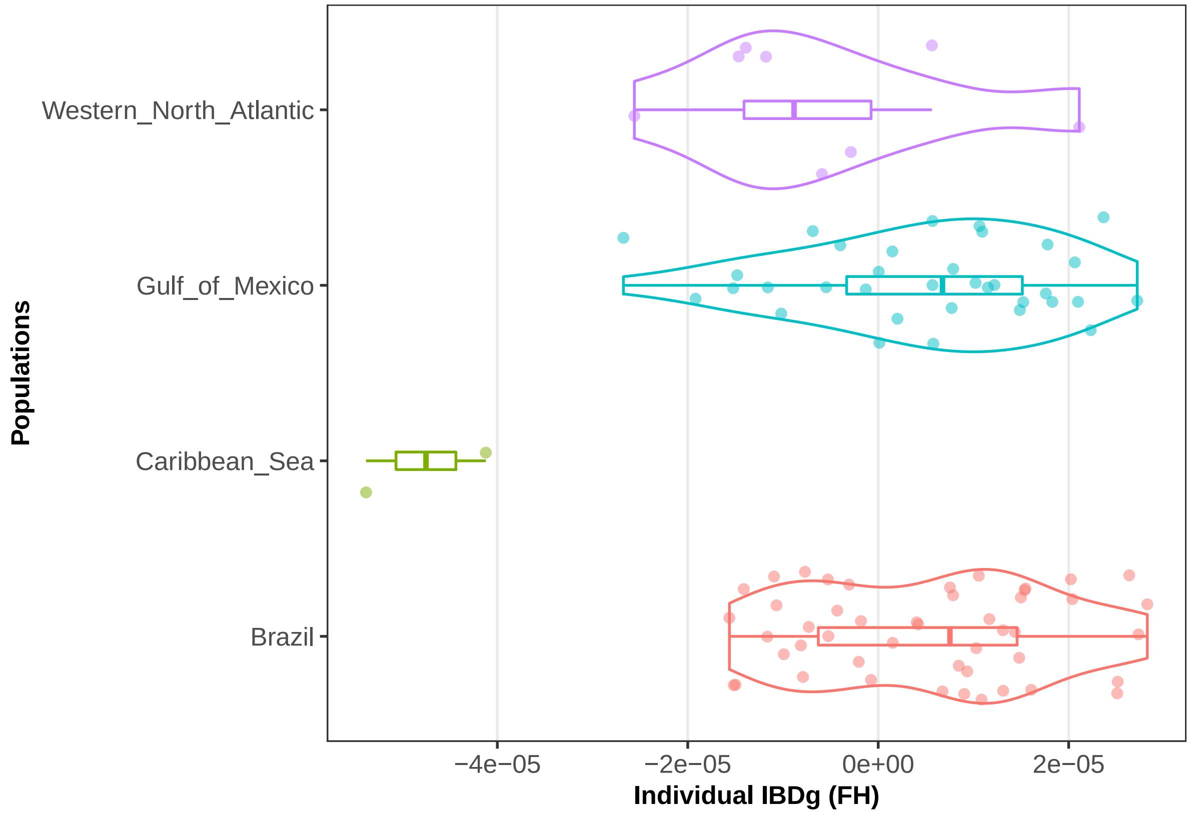

## 10.7 Genetic differentiation

```
Fst.gt <-
  strataG::popStructTest(
    BS.dartcap.WATL.gt,
    nrep = 1000,
    stats = "all",
    type = "pairwise",
    keep.null = FALSE,
    quietly = TRUE,
    nb.cores =  parallel::detectCores() - 1,
    write.output = FALSE
  )
save(Fst.gt, file = "DArTcap_WATL_FST.Rdata")
readr::write_tsv(Fst.gt$pairwise$result, path = "DArTcap_WATL_FST.txt")
```

```
load("DArTcap_WATL_FST.Rdata")
knitr::kable(
  Fst.gt$pairwise$result[order(Fst.gt$pairwise$result$Fst,
                                        decreasing = TRUE),
                                  -c(1:4)], digits = 4,
  caption = "Pairwise Fst for 931 loci between the West Atlantic sites") %>%
  kableExtra::kable_styling(full_width = FALSE,font_size = 5,
                            latex_options = c( "hold_position")) %>%
  kableExtra::landscape()
```

Table 42: Pairwise Fst for 931 loci between the West Atlantic sites

| | Chi2 | Chi2.p.val | D | D.p.val | Fis | Fis.p.val | Fst | Fst.p.val | F'st | F'st.p.val | Gst | Gst.p.val | G'st | G'st.p.val | G"st | G"st.p.val |
|---|---|---|---|---|---|---|---|---|---|---|---|---|---|---|---|---|
| Brazil (47) v. Western_North_Atlantic (8) | 976.2829 | 0.3686 | 0.0005 | 0.2098 | -0.1240 | 0.8032 | 0.0067 | 0.0160 | 0.0082 | 0.0160 | 0.0069 | 0.0120 | 0.0099 | 0.0120 | 0.0167 | 0.0120 |
| Brazil (47) v. Gulf_of_Mexico (34) | 1267.0889 | 0.0010 | 0.0002 | 0.0220 | 0.0271 | 0.9920 | 0.0061 | 0.0010 | 0.0075 | 0.0010 | 0.0032 | 0.0010 | 0.0046 | 0.0010 | 0.0079 | 0.0010 |
| Gulf_of_Mexico (34) v. Western_North_Atlantic (8) | 948.0094 | 0.0739 | 0.0004 | 0.2627 | -0.6087 | 0.7113 | 0.0027 | 0.1479 | 0.0033 | 0.1479 | 0.0144 | 0.1558 | 0.0203 | 0.1518 | 0.0341 | 0.1538 |
| Brazil (47) v. Caribbean_Sea (2) | 997.6150 | 0.3347 | 0.0016 | 0.6563 | -0.1850 | 0.6014 | 0.0012 | 0.4338 | 0.0015 | 0.4338 | 0.0177 | 0.4113 | 0.0253 | 0.4113 | 0.0423 | 0.4113 |
| Caribbean_Sea (2) v. Gulf_of_Mexico (34) | 982.2654 | 0.0599 | 0.0014 | 0.7093 | -1.0572 | 0.3402 | -0.0008 | 0.5051 | -0.0010 | 0.5051 | 0.0777 | 0.6655 | 0.1054 | 0.6199 | 0.1698 | 0.6324 |
| Caribbean_Sea (2) v. Western_North_Atlantic (8) | 657.8576 | 0.4326 | 0.0015 | 0.4366 | -4.6634 | 0.3099 | -0.0023 | 0.5425 | -0.0028 | 0.5425 | 0.3066 | 0.6501 | 0.3701 | 0.6706 | 0.5180 | 0.6706 |

## 10.8 PCA

```
pca1 <- adegenet::glPca(BS.dartcap.WATL.gl, nf = 5, parallel = TRUE,
                         n.cores = parallel::detectCores() - 1)
save(pca1, file = "DArTcap_WATL_PCA.Rdata")
```

```
load("DArTcap_WATL_PCA.Rdata")

BS.pca.scores <- as.data.frame(pca1$scores)
BS.pca.scores$pop <- pop(BS.dartcap.WATL.gl)
cols <- adegenet::funky(nPop(BS.dartcap.WATL.gl))


p <-
  ggplot2::ggplot(BS.pca.scores, ggplot2::aes(x = PC1, y = PC2, colour = pop)) +
  ggplot2::geom_point(size = 2) + ggplot2::scale_color_manual(values = cols) +
  ggplot2::geom_hline(yintercept = 0) + ggplot2::geom_vline(xintercept = 0) +
  ggplot2::theme_minimal() +
  ggplot2::theme(
    axis.text = ggplot2::element_text(size = 5),
    axis.title.x = ggplot2::element_text(size = 10),
    axis.title.y = ggplot2::element_text(size = 10),
    legend.text = ggplot2::element_text(size = 7)
  )
p
```

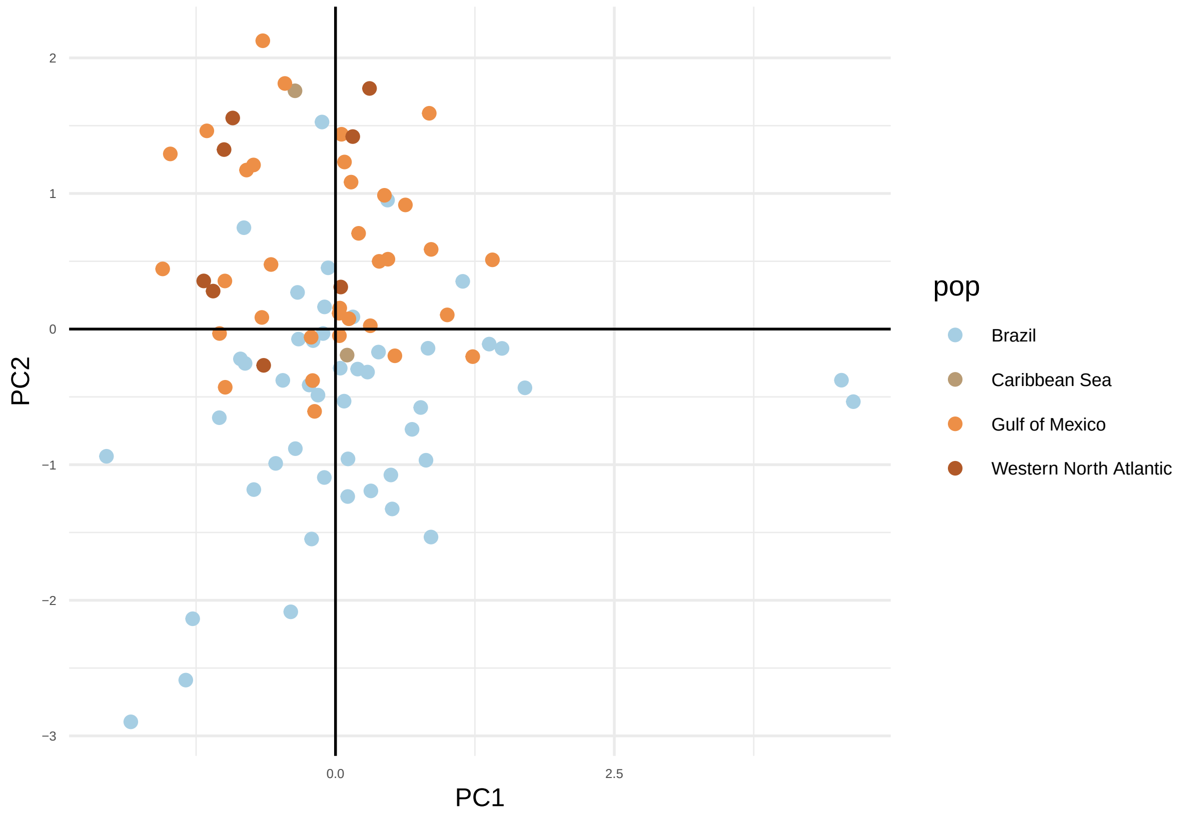


```
ggplot2::ggsave(p, filename = "DArTcap_WATL_PCA1.png", width = 30,
                height = 15,units = "cm")


p <-
  ggplot2::ggplot(BS.pca.scores, ggplot2::aes(x = PC1, y = PC3, colour = pop)) +
```

```
  ggplot2::geom_point(size = 2) + ggplot2::scale_color_manual(values = cols) +
  ggplot2::geom_hline(yintercept = 0) + ggplot2::geom_vline(xintercept = 0) +
  ggplot2::theme_minimal() +
  ggplot2::theme(
    axis.text = ggplot2::element_text(size = 5),
    axis.title.x = ggplot2::element_text(size = 10),
    axis.title.y = ggplot2::element_text(size = 10),
    legend.text = ggplot2::element_text(size = 7)
  )
p
```

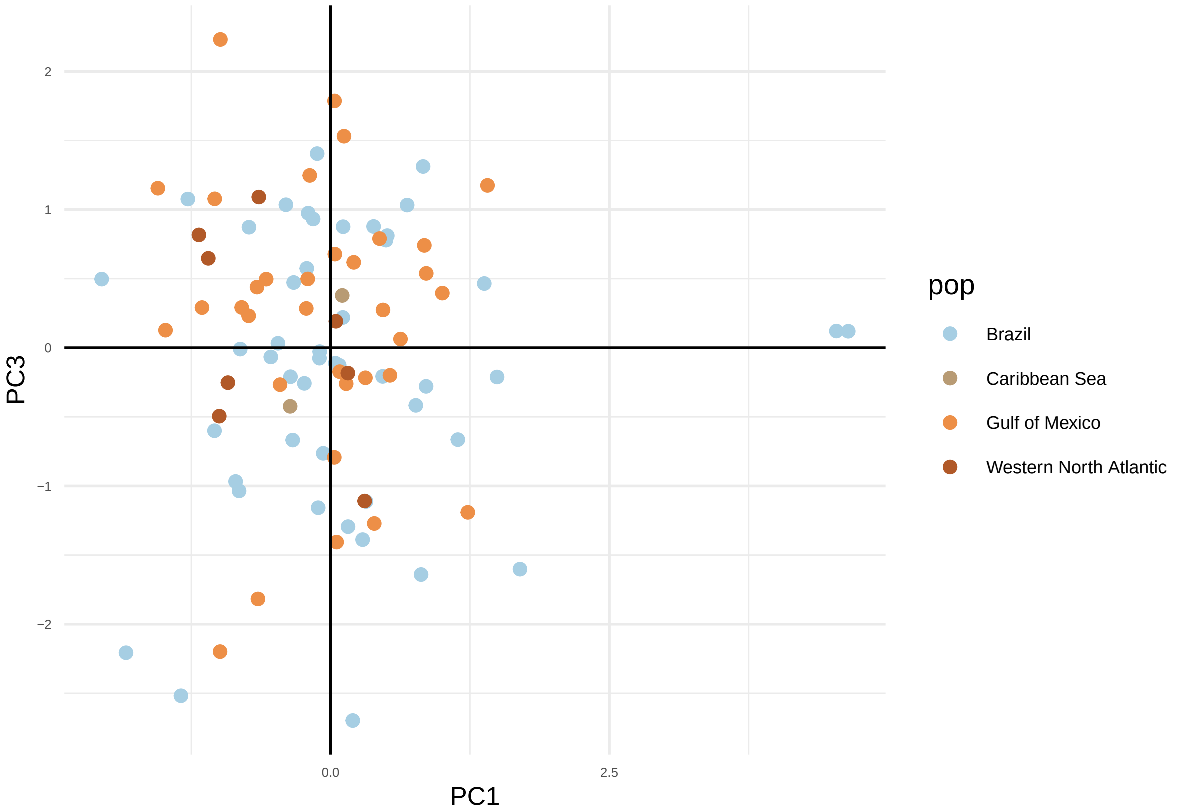


```
ggplot2::ggsave(p, filename = "DArTcap_WATL_PCA2.png", width = 30,
                height = 15,units = "cm")

p <-
  ggplot2::ggplot(BS.pca.scores, ggplot2::aes(x = PC2, y = PC3, colour = pop)) +
  ggplot2::geom_point(size = 2) + ggplot2::scale_color_manual(values = cols) +
  ggplot2::geom_hline(yintercept = 0) + ggplot2::geom_vline(xintercept = 0) +
  ggplot2::theme_minimal() +
  ggplot2::theme(
    axis.text = ggplot2::element_text(size = 5),
    axis.title.x = ggplot2::element_text(size = 10),
    axis.title.y = ggplot2::element_text(size = 10),
    legend.text = ggplot2::element_text(size = 7)
  )
p
```

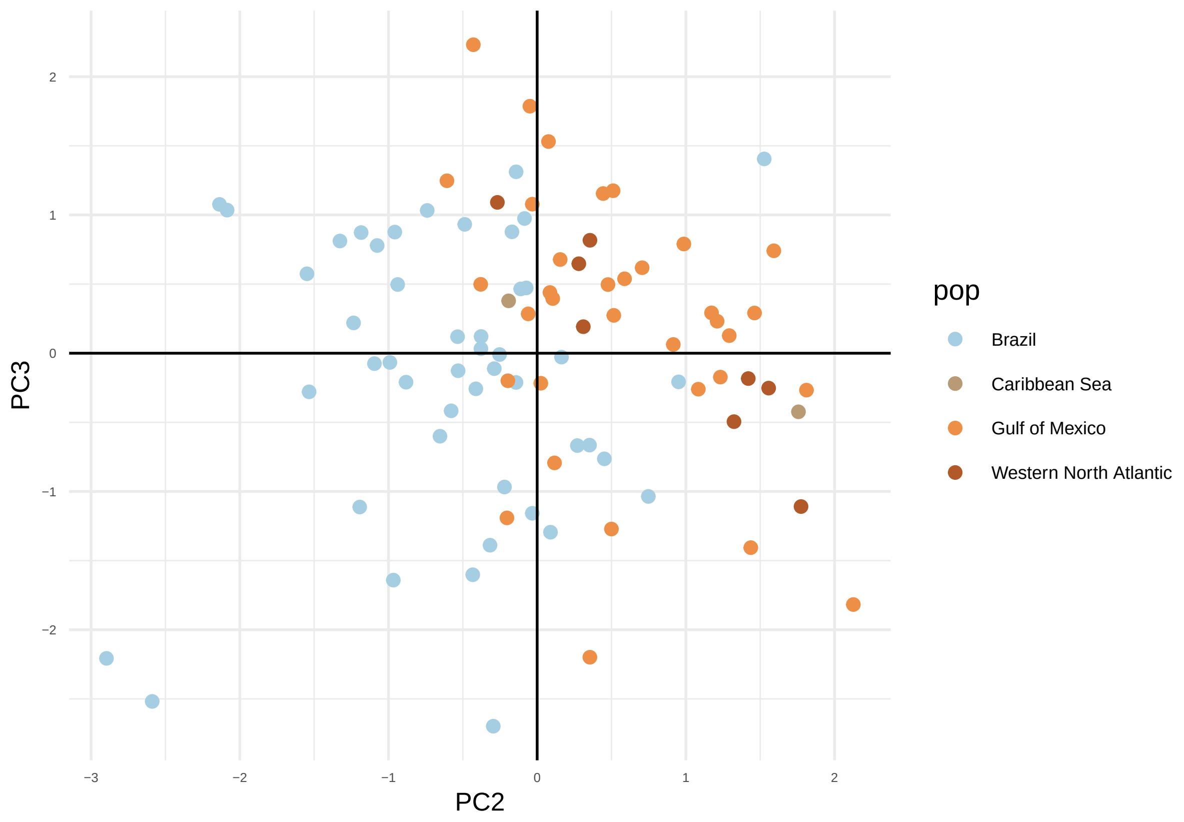


```r
ggplot2::ggsave(p, filename = "DArTcap_WATL_PCA3.png", width = 30,
                height = 15,units = "cm")


p <-
  ggplot2::ggplot(BS.pca.scores, ggplot2::aes(x = PC3, y = PC4, colour = pop)) +
  ggplot2::geom_point(size = 2) + ggplot2::scale_color_manual(values = cols) +
  ggplot2::geom_hline(yintercept = 0) + ggplot2::geom_vline(xintercept = 0) +
  ggplot2::theme_minimal() +
  ggplot2::theme(
    axis.text = ggplot2::element_text(size = 5),
    axis.title.x = ggplot2::element_text(size = 10),
    axis.title.y = ggplot2::element_text(size = 10),
    legend.text = ggplot2::element_text(size = 7)
  )
p
```

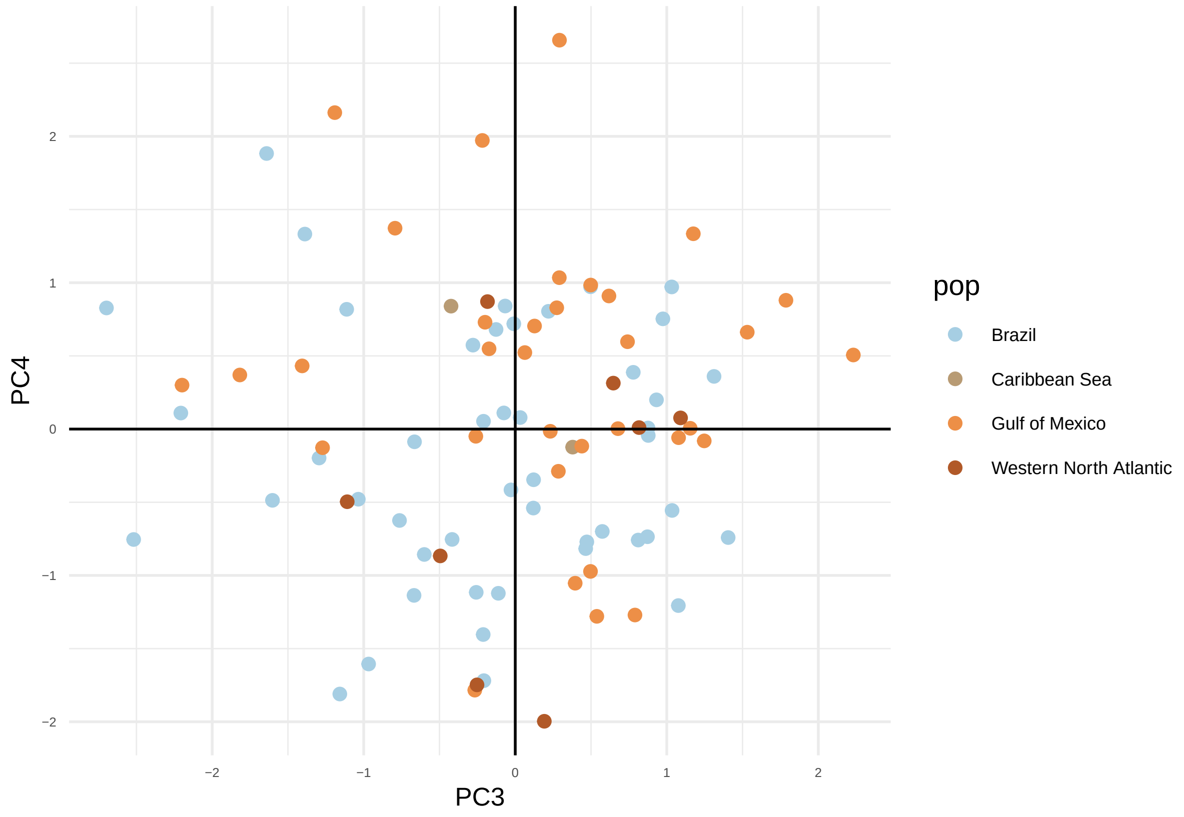


```
ggplot2::ggsave(p, filename = "DArTcap_WATL_PCA4.png", width = 30,
                height = 15,units = "cm")

p <-
  ggplot2::ggplot(BS.pca.scores, ggplot2::aes(x = PC3, y = PC5, colour = pop)) +
  ggplot2::geom_point(size = 2) + ggplot2::scale_color_manual(values = cols) +
  ggplot2::geom_hline(yintercept = 0) + ggplot2::geom_vline(xintercept = 0) +
  ggplot2::theme_minimal() +
  ggplot2::theme(
    axis.text = ggplot2::element_text(size = 5),
    axis.title.x = ggplot2::element_text(size = 10),
    axis.title.y = ggplot2::element_text(size = 10),
    legend.text = ggplot2::element_text(size = 7)
  )
p
```

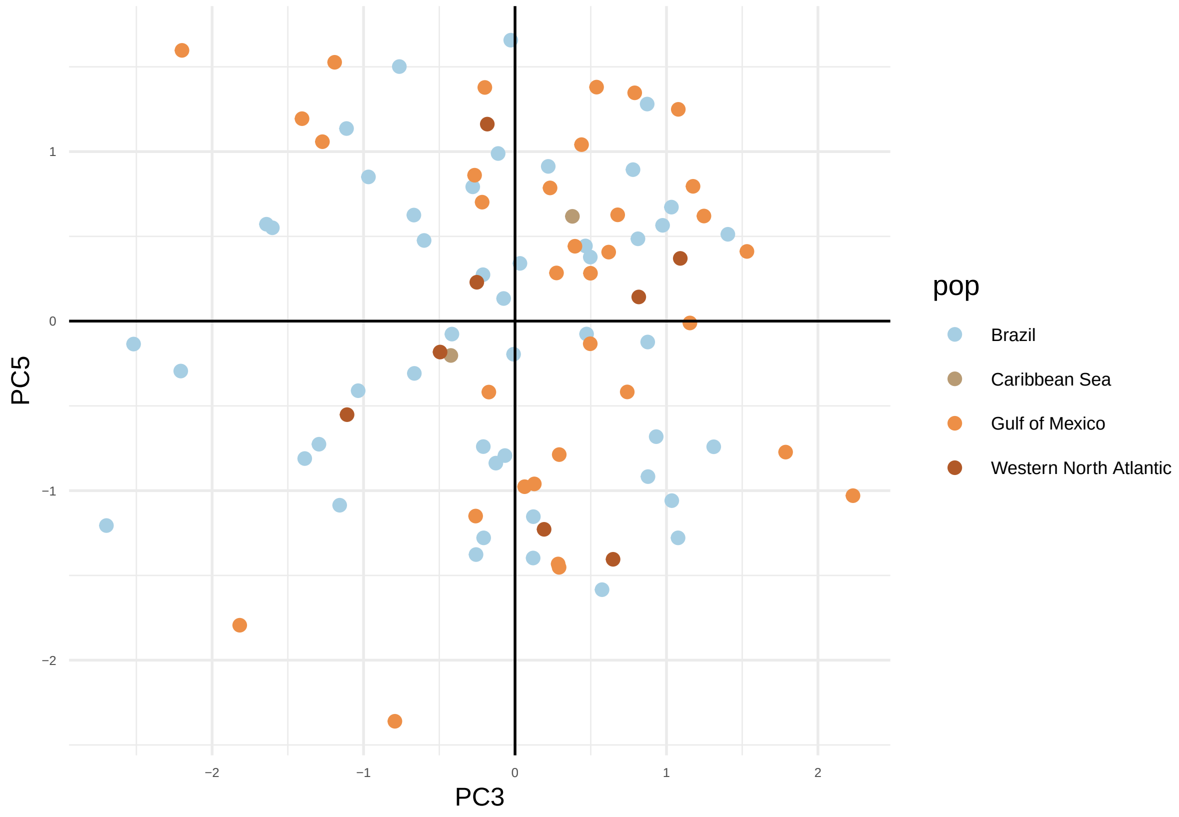


```
ggplot2::ggsave(p, filename = "DArTcap_WATL_PCA5.png", width = 30,
                height = 15,units = "cm")
```

## 10.9 DAPC

### 10.9.1 Optimal number of clusters with neutral markers

```
set.seed(124)
grp <-
  adegenet::find.clusters(
    BS.dartcap.WATL.gl,
    max.n.clust = 10,
    n.pca = adegenet::nInd(BS.dartcap.WATL.gl) / 3,
    stat = "BIC",
    parallel = TRUE,
    n.cores = parallel::detectCores() - 1
  )

save(grp, file = "DArTcap_WATL_grp.Rdata")
```

```
load("DArTcap_WATL_grp.Rdata")

y <- as.numeric(grp$Kstat)
x <- 1:10
data <- data.frame(x,y,stringsAsFactors = F)
plot <- ggplot2::ggplot(data, ggplot2::aes(x,y)) +
  ggplot2::geom_point(size = 5, shape = 1, color = "blue") +
  ggplot2::geom_line(size = 1,color = "blue") +
  ggplot2::scale_x_continuous(name = waiver(),
                              breaks = seq(from = 0,
                                           to = nrow(BS.dartcap.WATL.gl) - 1,
```

```
                                          by = 1)) +
  ggplot2::labs(subtitle = "",
                y = "Bayesian Information Criterion",
                x = "Number of clusters",
                title = "",
                caption = "") +
  ggplot2::theme_classic() +
  ggplot2::theme(
    axis.text = ggplot2::element_text(size = 5),
    axis.title.x = ggplot2::element_text(size = 20,vjust = 0, hjust = 0.5),
    axis.title.y = ggplot2::element_text(size = 20,vjust = 2, hjust = 0.5))
print(plot)
```

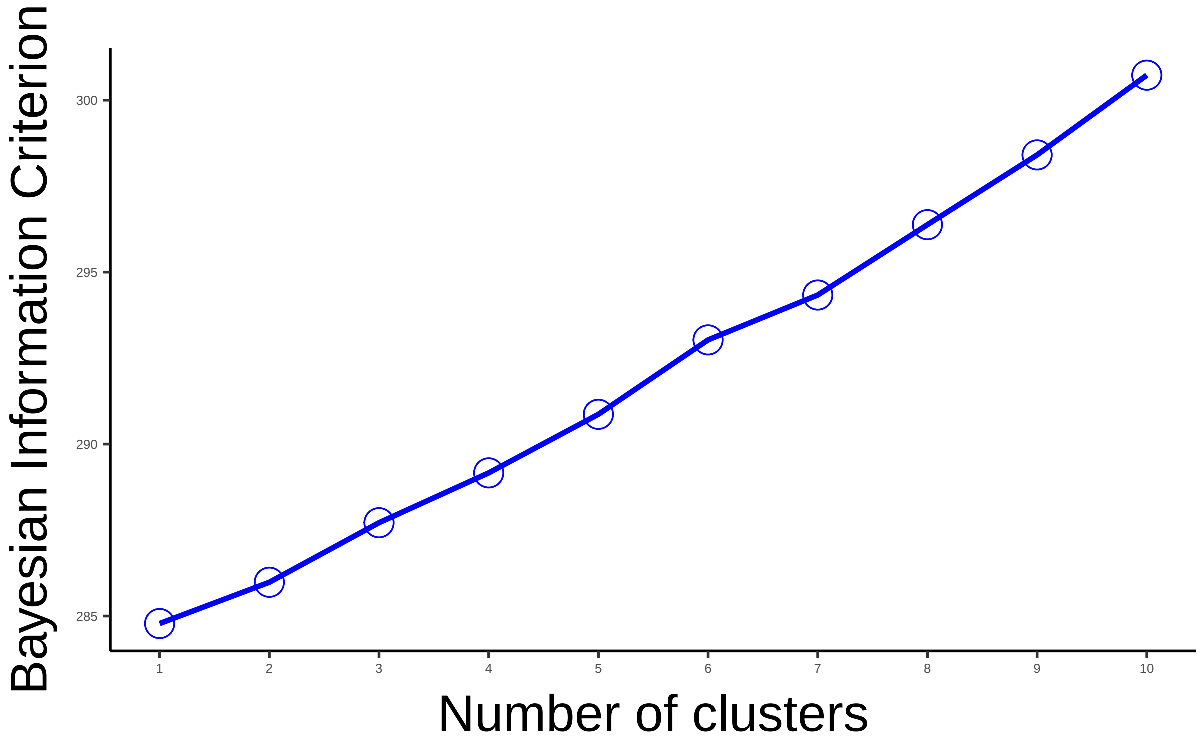


```
ggplot2::ggsave(plot, filename = "DArTcap_WATL_grp.png", width = 30,
                height = 15,units = "cm")
```

### 10.9.2 Cross-validation to identify the optimal number of PCs

Uses a training - holdout set of individuals to check how reliable individuals can be assigned.

```
set.seed(124)
x <- BS.dartcap.WATL.gl

xval <-
  adegenet::xvalDapc(
  tab(x, NA.method = "mean"),
  adegenet::pop(x),
  n.pca = seq(1, adegenet::nInd(x), by = 10),
  training.set = 0.9,
  result = "groupMean",
  center = TRUE,
  scale = FALSE,
```

```r
  n.rep = 1000,
  parallel = "multicore",
  ncpus = 1,
  xval.plot = TRUE
  )
print(xval[2:6])
PC <- as.numeric(xval$`Number of PCs Achieving Lowest MSE`)

#refine the analysis
set.seed(124)
png(file="DArTcap_WATL_xval.png")
xval <-
  adegenet::xvalDapc(
  tab(x, NA.method = "mean"),
  adegenet::pop(x),
  n.pca = (PC - 15):(PC + 15),
  n.rep = 1000,
  parallel = "multicore",
  ncpus = 1,
  xval.plot = TRUE
  )
dev.off()
save(xval, file = "DArTcap_WATL_xval.rdata")
```

```r
load("DArTcap_WATL_xval.rdata")
print(xval[2:6])
PC <- as.numeric(xval$`Number of PCs Achieving Lowest MSE`)
```

```
## $`Median and Confidence Interval for Random Chance`
##      2.5%       50%     97.5%
## 0.1799124 0.2468515 0.3532883
##
## $`Mean Successful Assignment by Number of PCs of PCA`
##         6         7         8         9        10        11        12        13
## 0.4011797 0.3993416 0.3917924 0.3895222 0.3781002 0.3818176 0.3772300 0.3763163
##        14        15        16        17        18        19        20        21
## 0.3724941 0.3787820 0.3765043 0.3744480 0.3960404 0.3886894 0.4241026 0.4147128
##        22        23        24        25        26        27        28        29
## 0.4074313 0.4047105 0.3907887 0.3772498 0.3712890 0.3764682 0.3709447 0.3634170
##        30        31        32        33        34        35        36
## 0.3573215 0.3559965 0.3442917 0.3289377 0.3282196 0.3141950 0.3014086
##
## $`Number of PCs Achieving Highest Mean Success`
## [1] "20"
##
## $`Root Mean Squared Error by Number of PCs of PCA`
##         6         7         8         9        10        11        12        13
## 0.6005609 0.6023505 0.6100747 0.6124445 0.6237366 0.6201820 0.6245583 0.6256715
##        14        15        16        17        18        19        20        21
## 0.6291852 0.6230769 0.6254401 0.6279443 0.6068985 0.6146509 0.5811306 0.5905123
##        22        23        24        25        26        27        28        29
## 0.5966892 0.5991244 0.6128676 0.6264447 0.6323786 0.6272664 0.6330625 0.6403656
##        30        31        32        33        34        35        36
## 0.6464854 0.6481012 0.6597074 0.6747974 0.6761027 0.6901762 0.7027381
##
## $`Number of PCs Achieving Lowest MSE`
## [1] "20"
```

```
dapc.all.object.names <- c()
for (K in 2:10) {
  set.seed(124)
  grp <- adegenet::find.clusters(BS.dartcap.WATL.gl, n.clust = K, n.pca = PC)
  set.seed(124)
  dapc.all.neutral <- adegenet::dapc(BS.dartcap.WATL.gl, grp$grp, n.da = K - 1,
                                     n.pca = PC)
  assign(paste0("dapc.all.", K),value = dapc.all.neutral)
  dapc.all.object.names <- c(dapc.all.object.names,
                                     paste0("dapc.all.", K))
}
save(list = dapc.all.object.names, dapc.all.object.names,
     file = "DArTcap_WATL_DAPC.Rdata")
```

#### 10.9.3 DAPC barplot

Group individuals according to DAPC posterior membership.

```
load("DArTcap_WATL_DAPC.Rdata")
for (K in 2:10) {
  colour <- colours.prior[1:K]
  dapc <- get(dapc.all.object.names[K - 1])
  post <- as.matrix(dapc$posterior)
  colnames(post) <- paste0("Group", 1:nlevels(dapc$grp))
  locations <- BS.dartcap.WATL.gl$pop

  plot.dapc.FDD(x = post,
                locations = locations,
                colour = colour,
                region.lwd = 1,
                plotTitle = paste0("DAPC: all Bull Sharks - subset Australia\n for K=",
                                   K, " & PC=", PC, sep = ""))
  dev.print(
    device = png,
    file = paste0("DArTcap_WATL_DAPC_barplot_K", K, ".png"),
    res = 300,
    width = 30,
    height = 15,
    units = "cm")
}
```

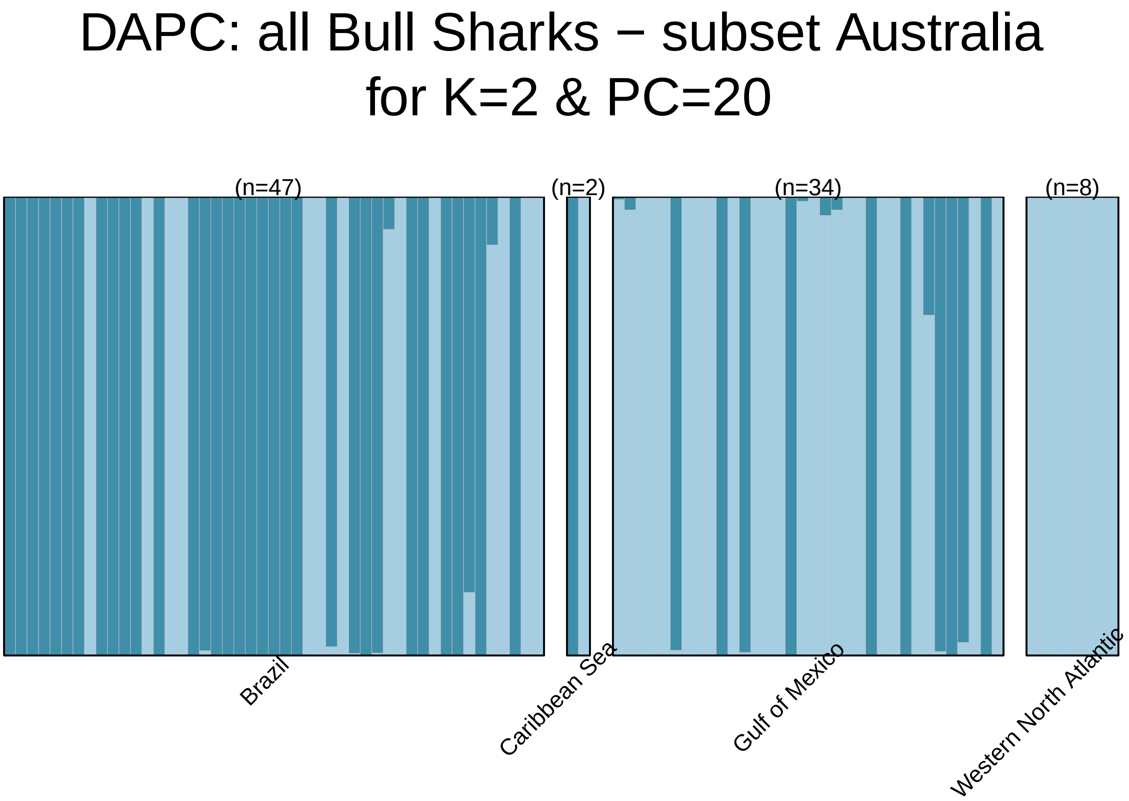
DAPC: all Bull Sharks − subset Australia
for K=2 & PC=20
(n=47)
(n=2)
(n=34)
(n=8)
Brazil
Caribbean Sea
Gulf of Mexico
Western North Atlantic

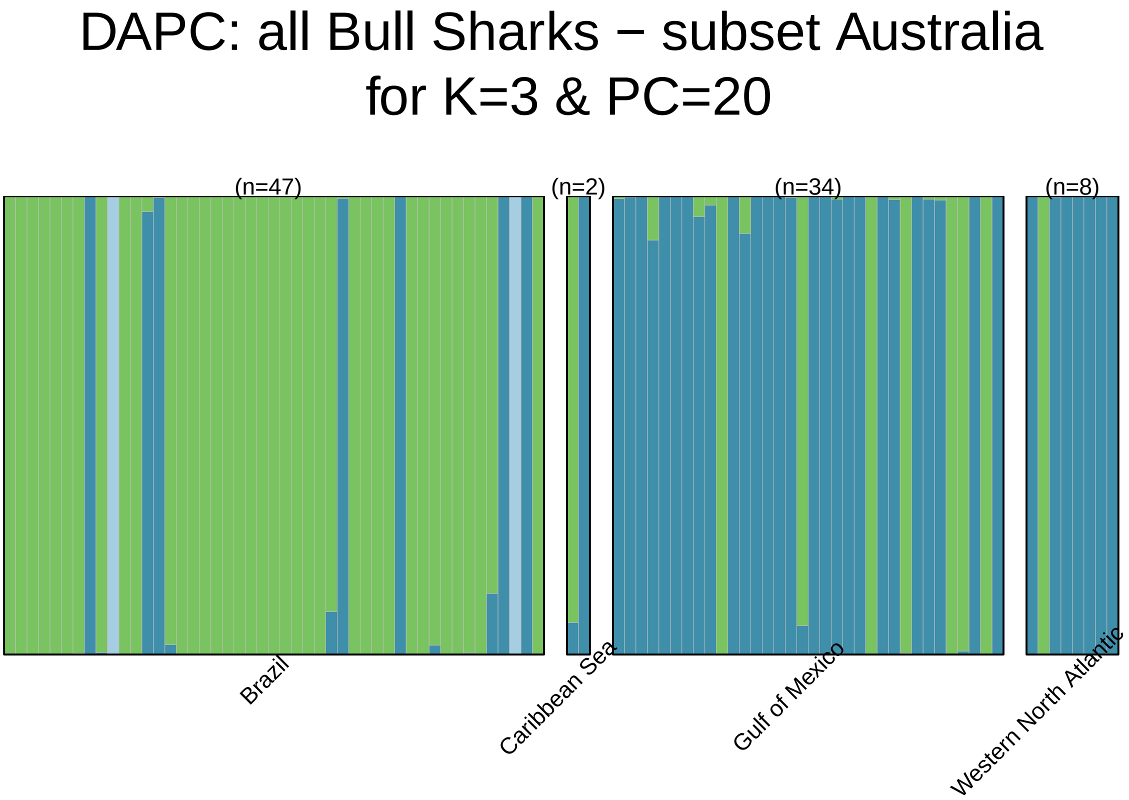
DAPC: all Bull Sharks − subset Australia
for K=3 & PC=20
(n=47)
(n=2)
(n=34)
(n=8)
Brazil
Caribbean Sea
Gulf of Mexico
Western North Atlantic

# DAPC: all Bull Sharks − subset Australia for K=4 & PC=20

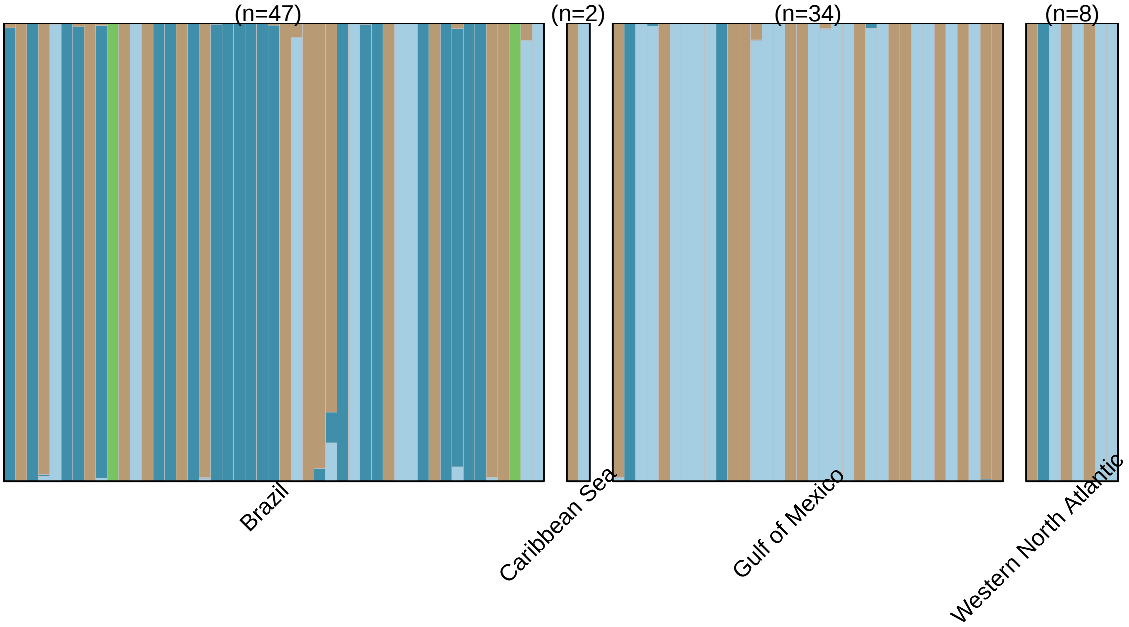


# DAPC: all Bull Sharks − subset Australia for K=5 & PC=20

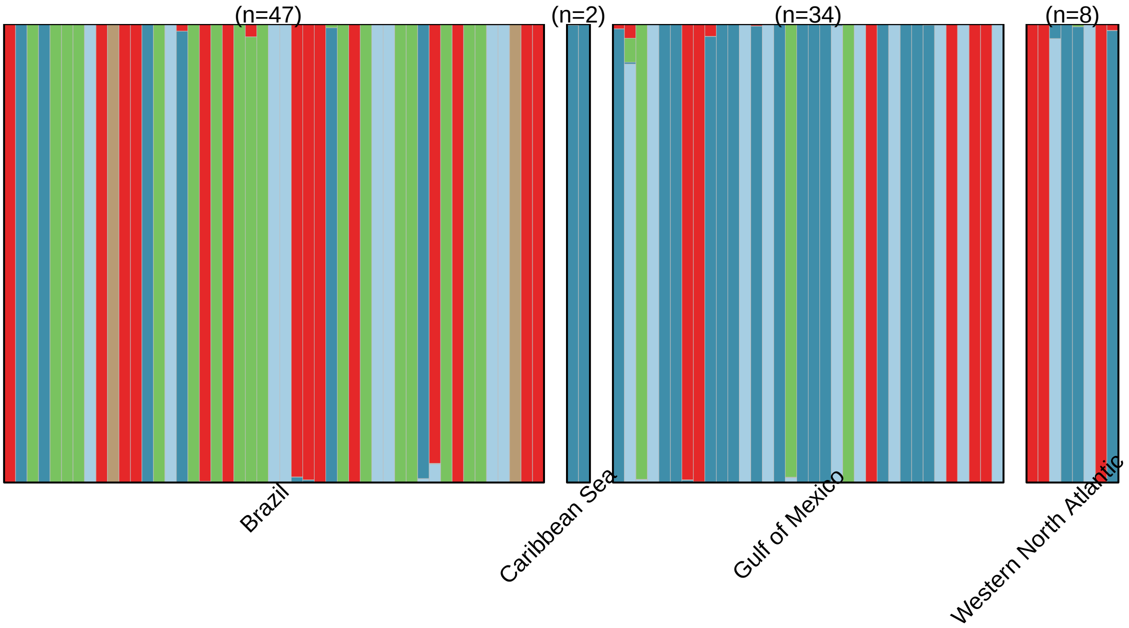

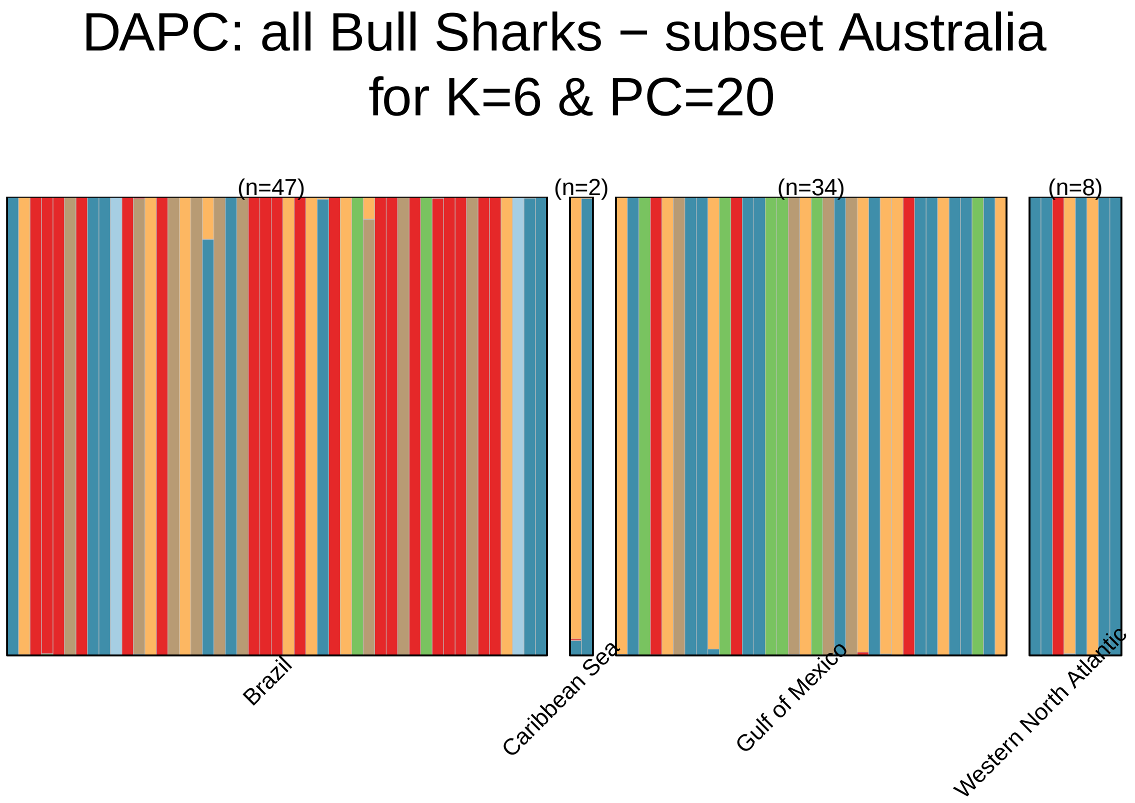
DAPC: all Bull Sharks − subset Australia
for K=6 & PC=20
(n=47)
(n=2)
(n=34)
(n=8)
Brazil
Caribbean Sea
Gulf of Mexico
Western North Atlantic

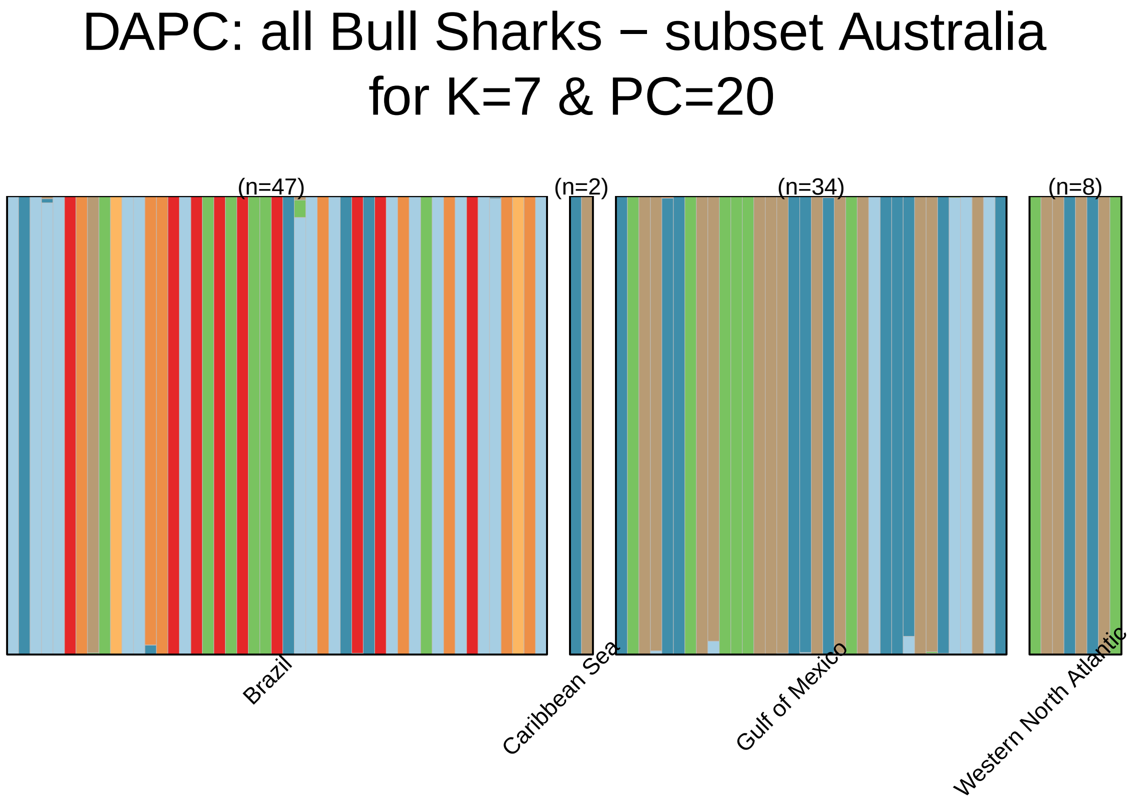
DAPC: all Bull Sharks − subset Australia
for K=7 & PC=20
(n=47)
(n=2)
(n=34)
(n=8)
Brazil
Caribbean Sea
Gulf of Mexico
Western North Atlantic

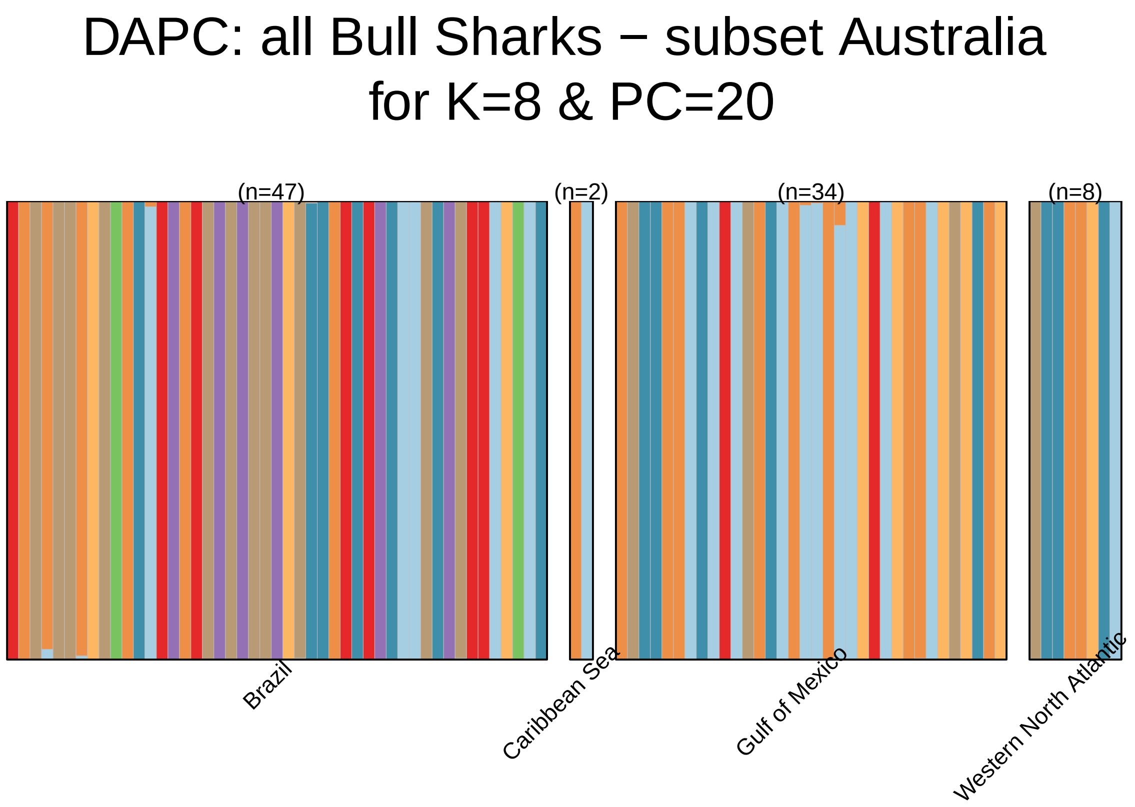
DAPC: all Bull Sharks − subset Australia
for K=8 & PC=20
(n=47)
(n=2)
(n=34)
(n=8)
Brazil
Caribbean Sea
Gulf of Mexico
Western North Atlantic

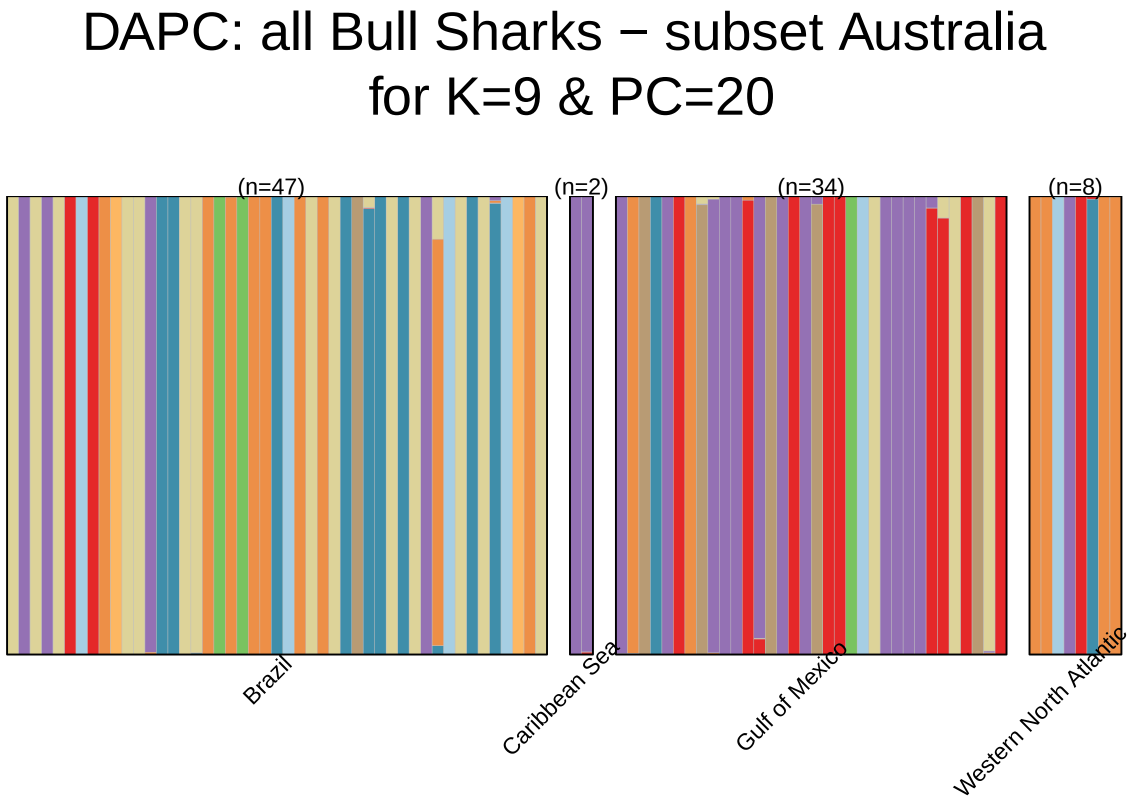
DAPC: all Bull Sharks − subset Australia
for K=9 & PC=20
(n=47)
(n=2)
(n=34)
(n=8)
Brazil
Caribbean Sea
Gulf of Mexico
Western North Atlantic

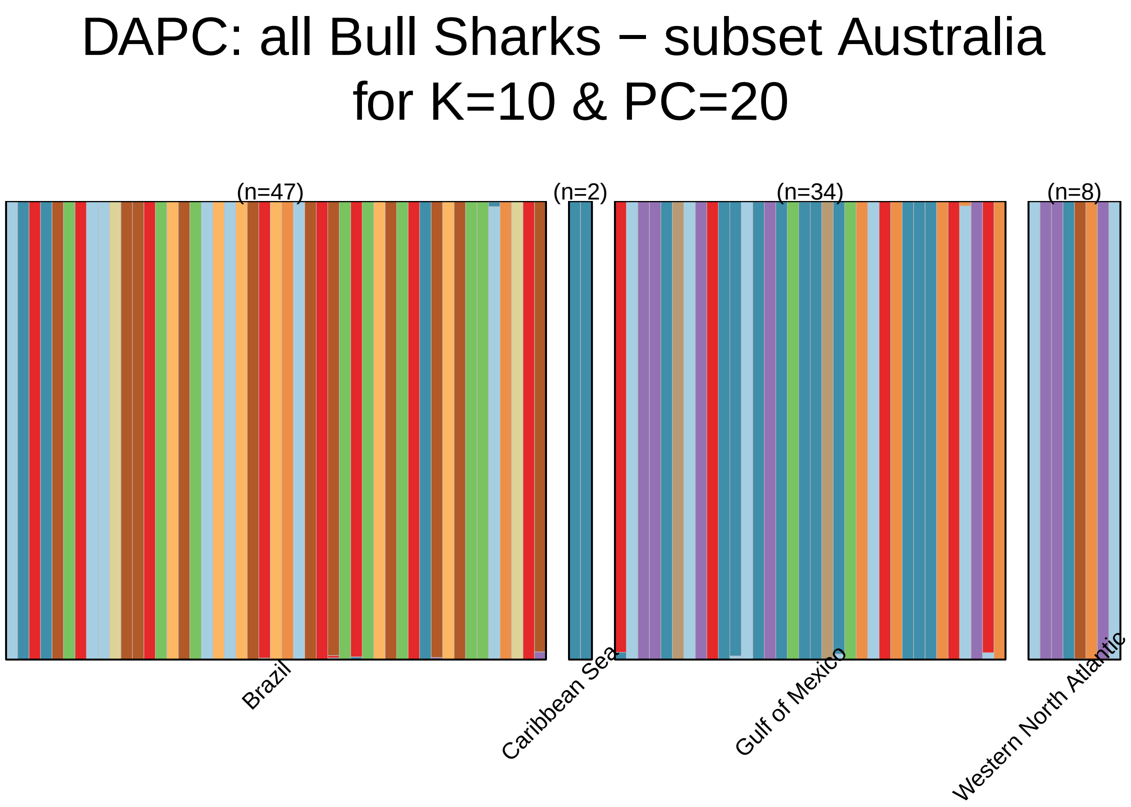


### 10.9.4 DAPC scatterplot

```
load("DArTcap_WATL_DAPC.Rdata")

for (K in 3:10) {
  dapc <- get(dapc.all.object.names[K - 1])
  if (K == 2) {
    posi.leg <- "bottomright"
  } else {posi.leg <- "bottomleft"}
  dapc.plot <- ade4::scatter(dapc, grp = pop(BS.dartcap.WATL.gl), cex = 2,
                             legend = TRUE, col = colours.4,clabel = FALSE,
                             posi.leg = posi.leg, scree.pca = FALSE,
                             posi.pca = "topleft", cleg = 0.75, xax = 1,
                             yax = 2, inset.solid = 0.3)
  dev.print(
    device = png,
    file = paste0("DArTcap_WATL_DAPC_scatterplot_K", K, ".png"),
    width = 30,
    height = 15,
    units = "cm",
    res = 300
  )
}
```

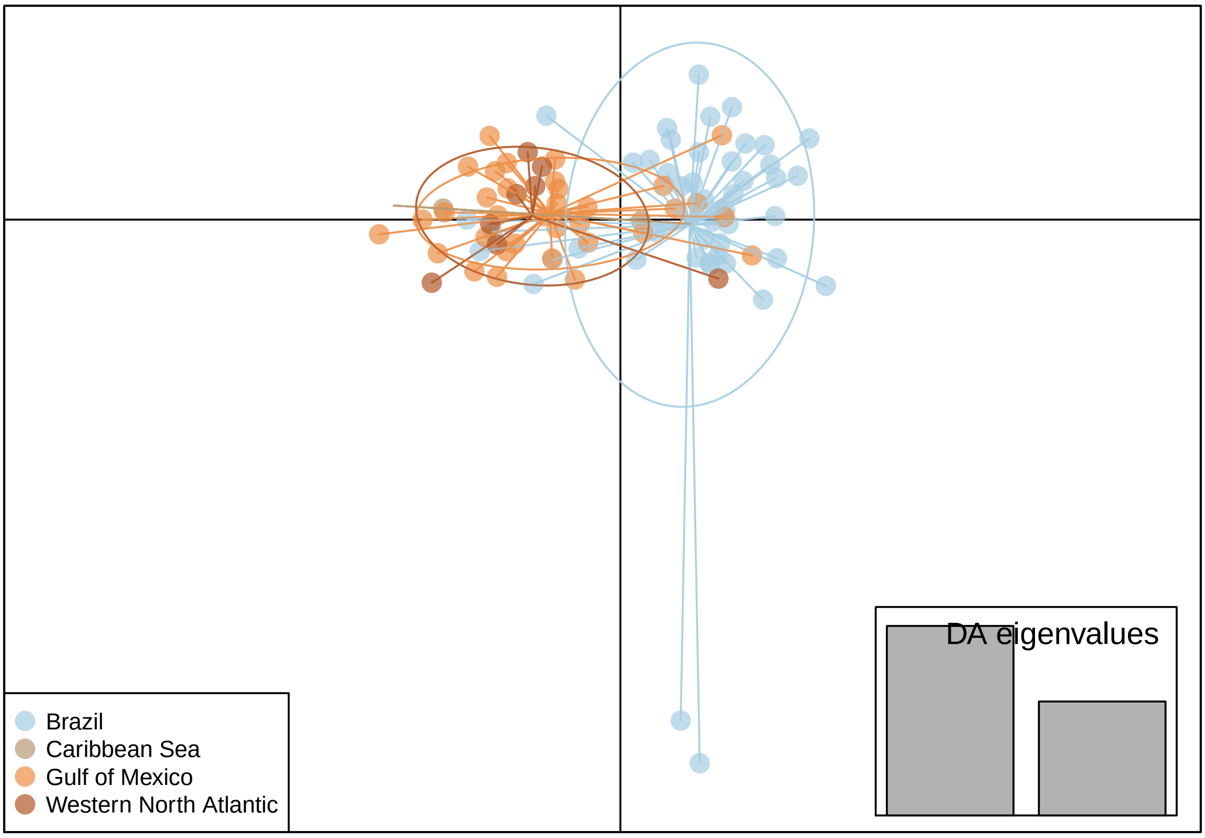
DA eigenvalues
Brazil
Caribbean Sea
Gulf of Mexico
Western North Atlantic

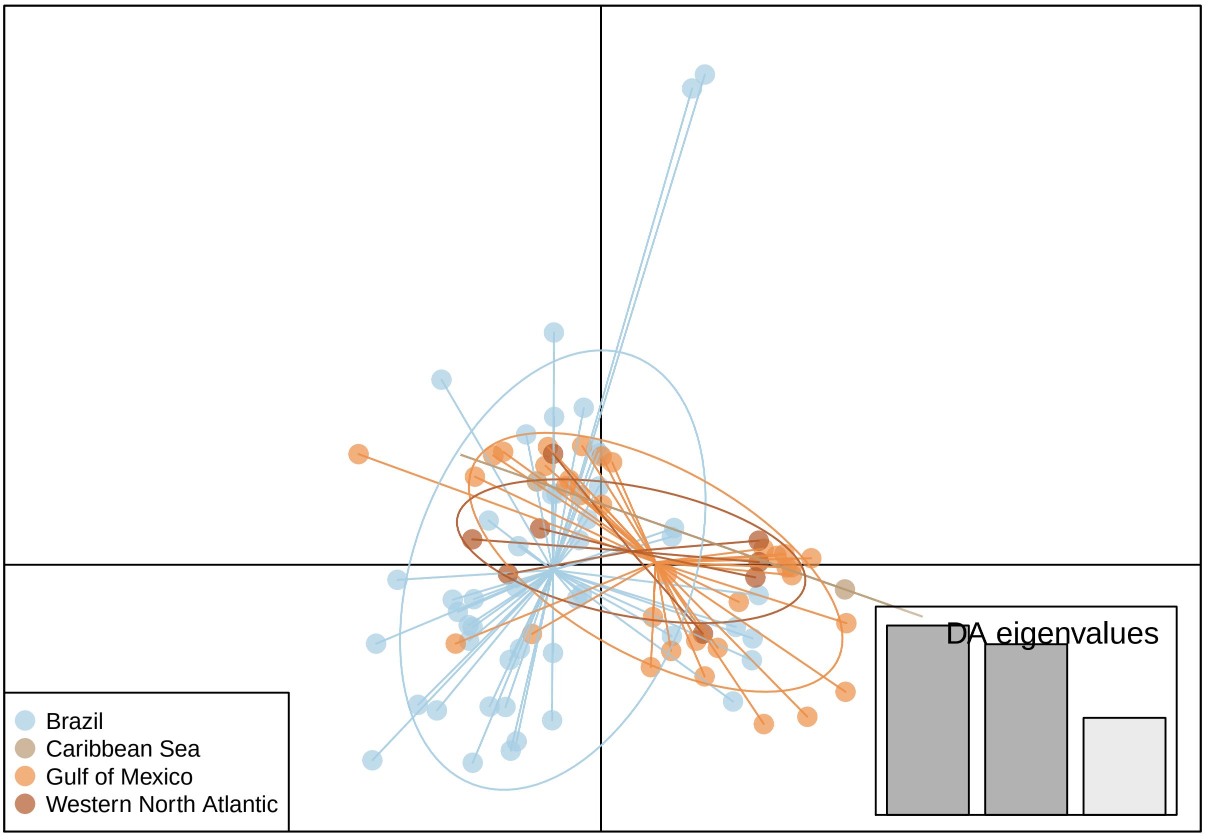
DA eigenvalues
Brazil
Caribbean Sea
Gulf of Mexico
Western North Atlantic

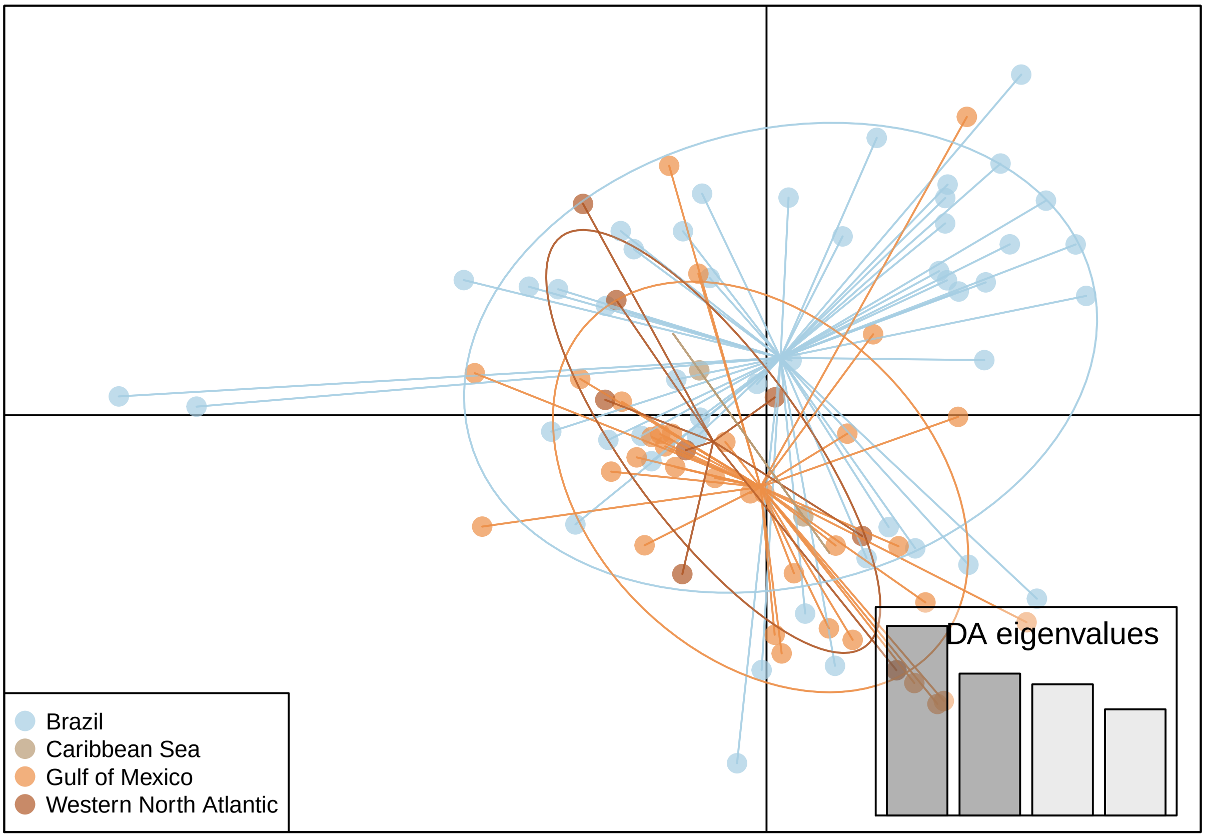
DA eigenvalues
Brazil
Caribbean Sea
Gulf of Mexico
Western North Atlantic

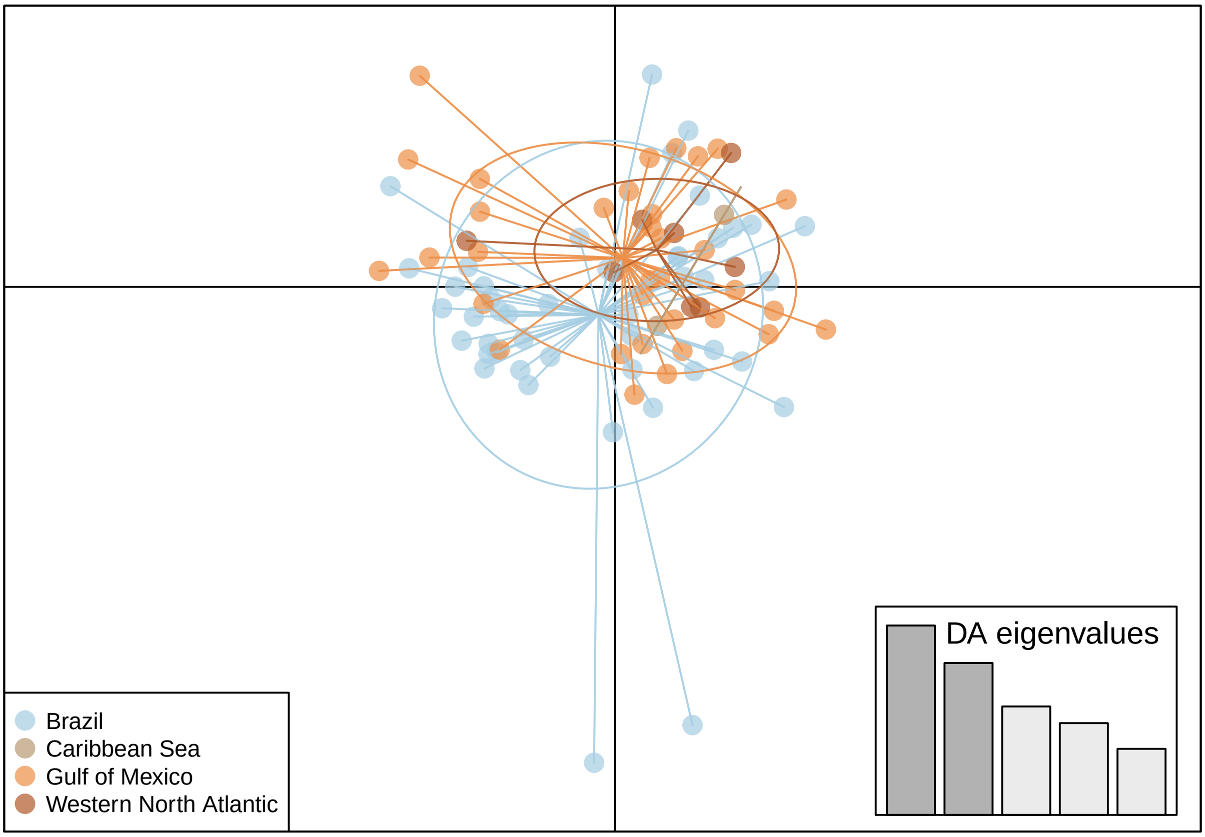
DA eigenvalues
Brazil
Caribbean Sea
Gulf of Mexico
Western North Atlantic

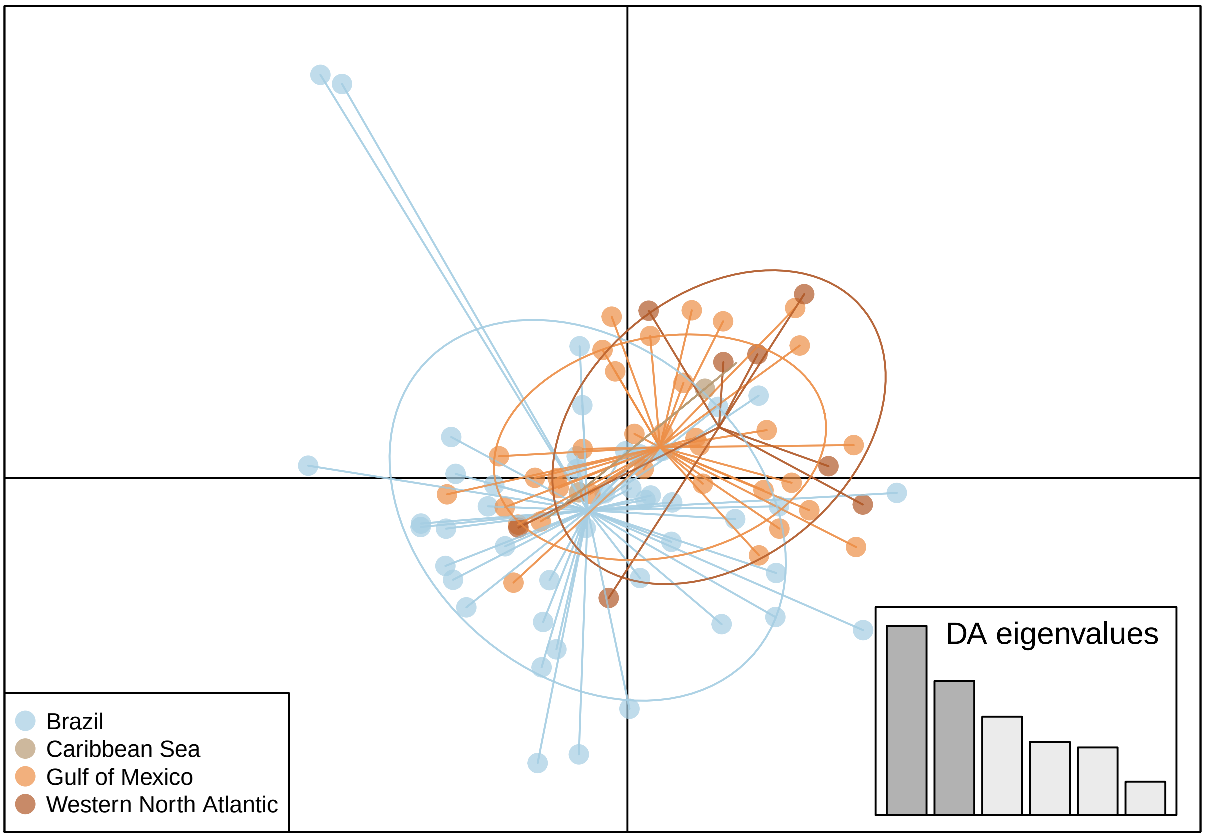
DA eigenvalues
Brazil
Caribbean Sea
Gulf of Mexico
Western North Atlantic

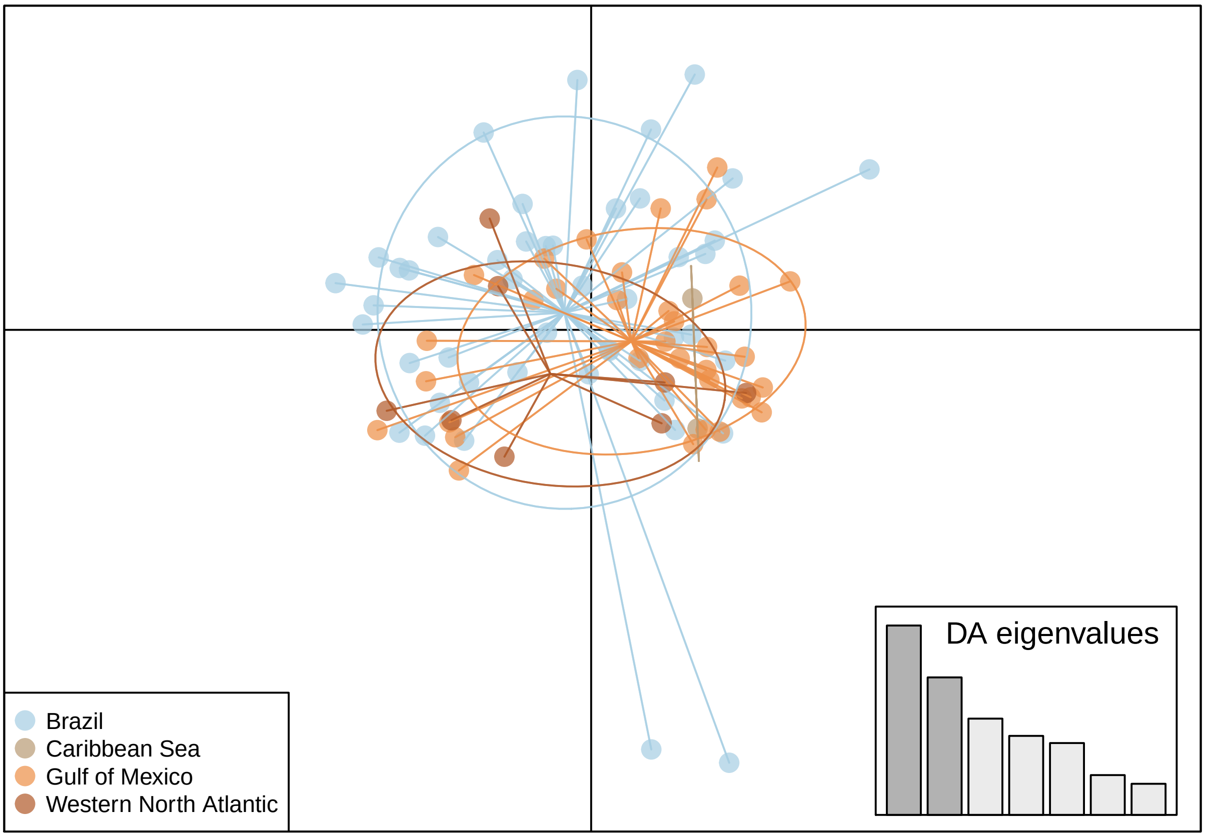
DA eigenvalues
Brazil
Caribbean Sea
Gulf of Mexico
Western North Atlantic

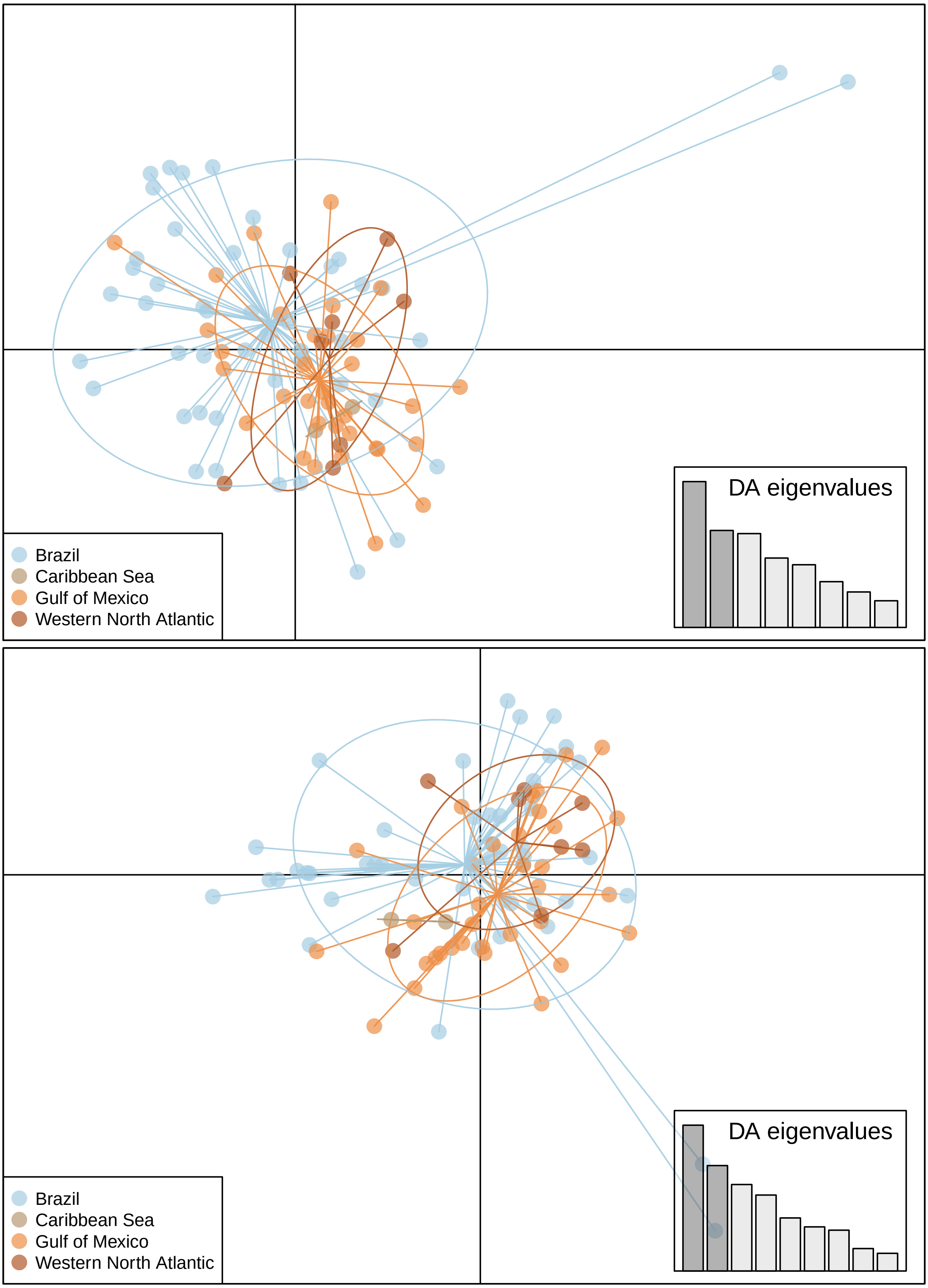


```
for (K in 2:10) {
  colour <- colours.prior[1:K]
  dapc <- get(dapc.all.object.names[K - 1])
  dapc.plot <- ade4::scatter(dapc, cex = 2, legend = TRUE, col = colour,
                             clabel = FALSE, posi.leg = "bottomleft",
```

```
                              scree.pca = FALSE,  posi.pca = "topleft",
                              cleg = 0.75, xax = 1, yax = 2,
                              inset.solid = 0.3)
  dev.print(
    device = png,
    file = paste0("DArTcap_WATL_DAPC_scatterplot_pergroups_K",
                  K,".png"),
    width = 30,
    height = 15,
    units = "cm",
    res = 300
  )
}
```

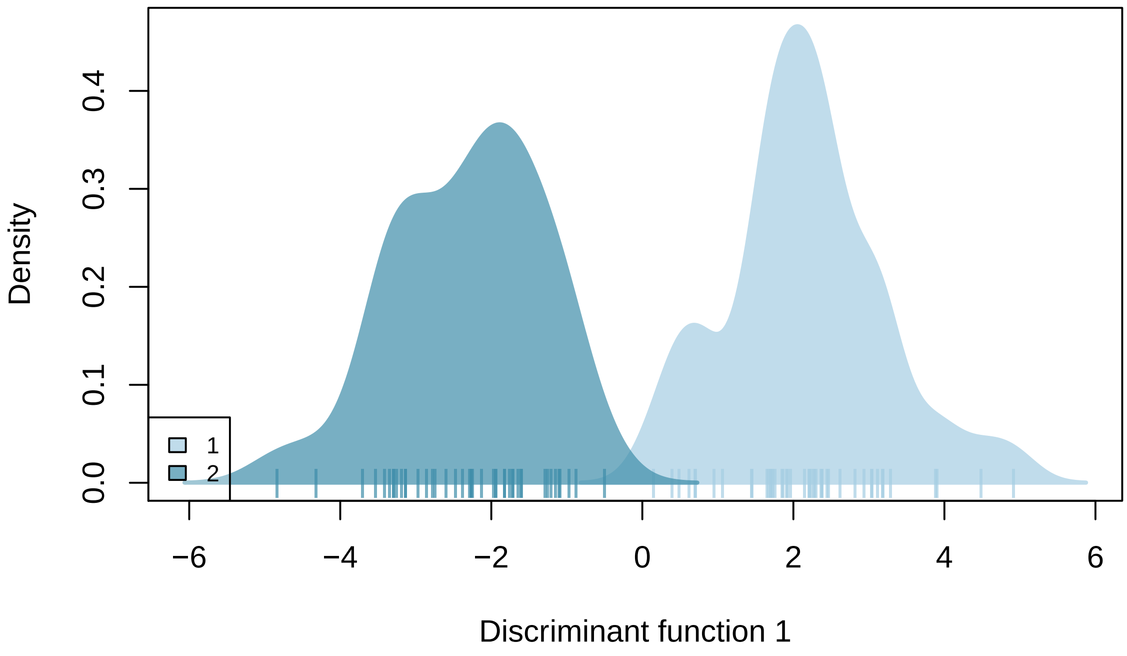

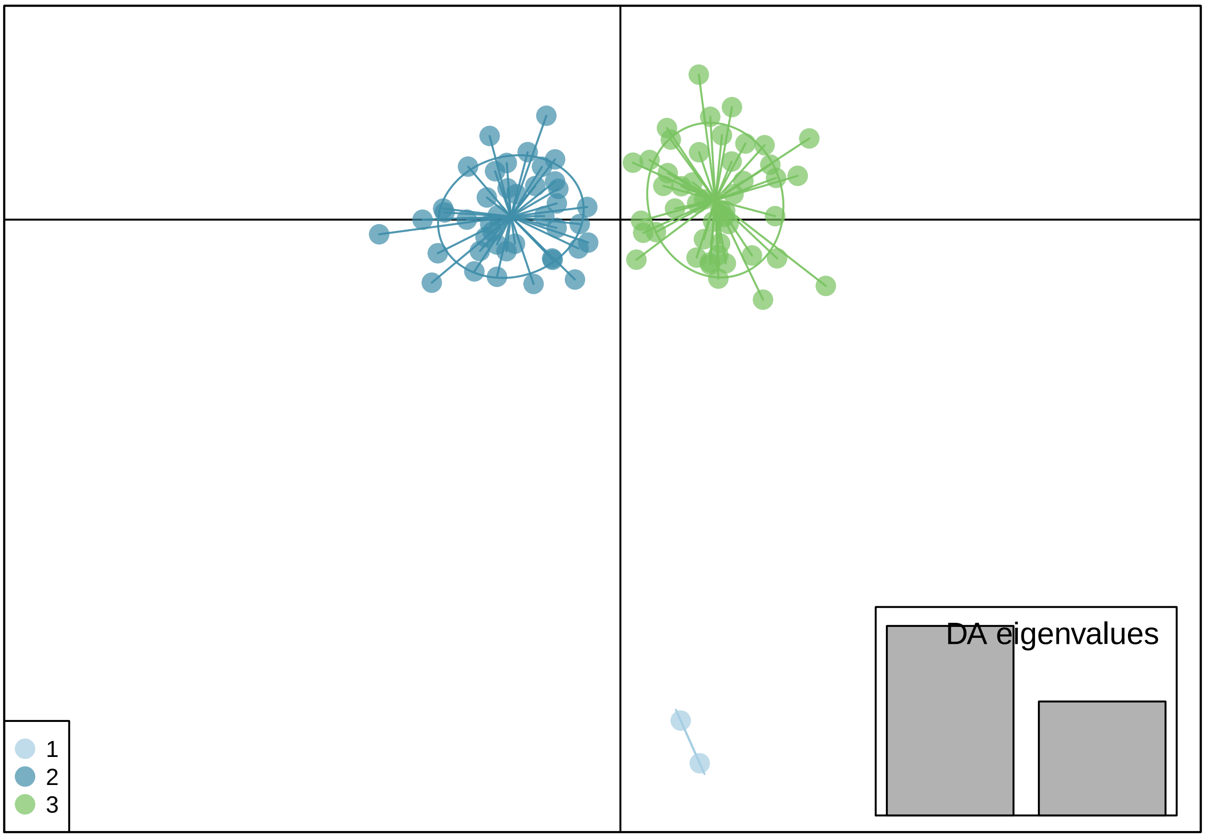
DA eigenvalues
1
2
3

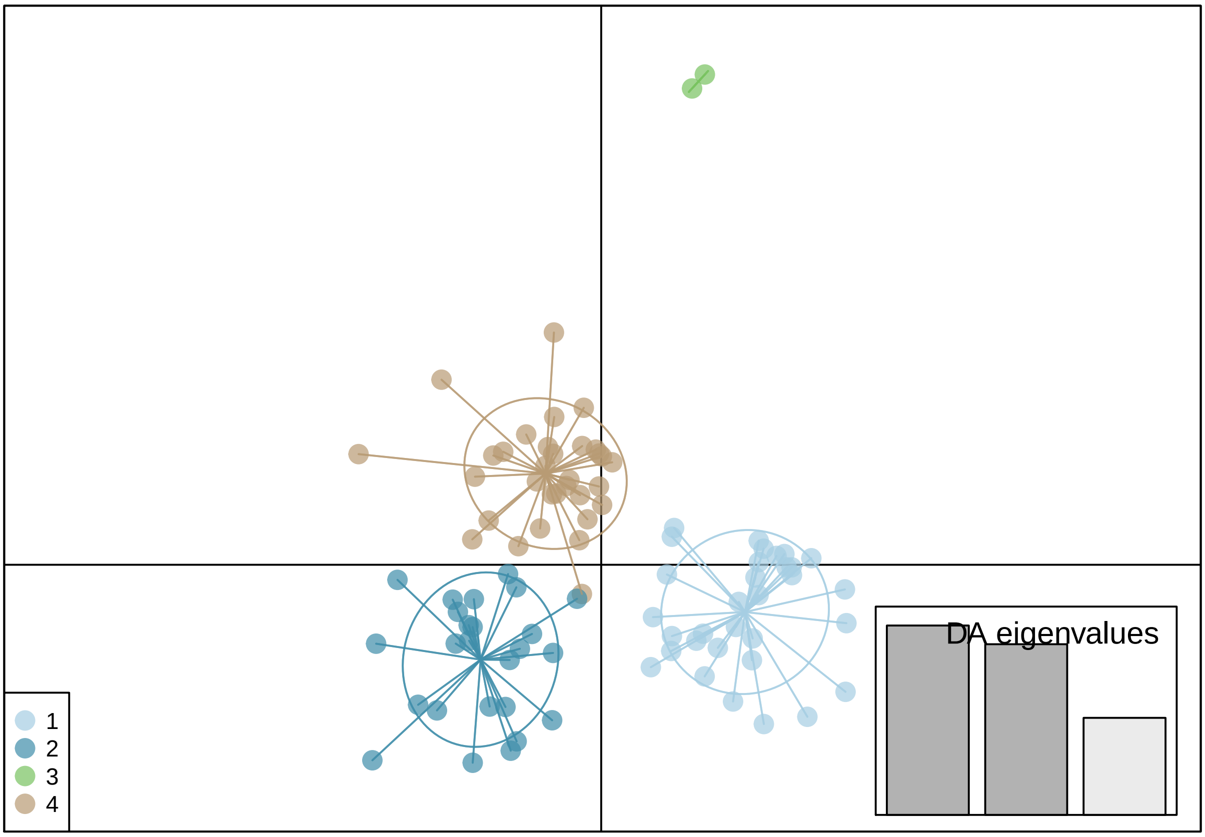
DA eigenvalues
1
2
3
4

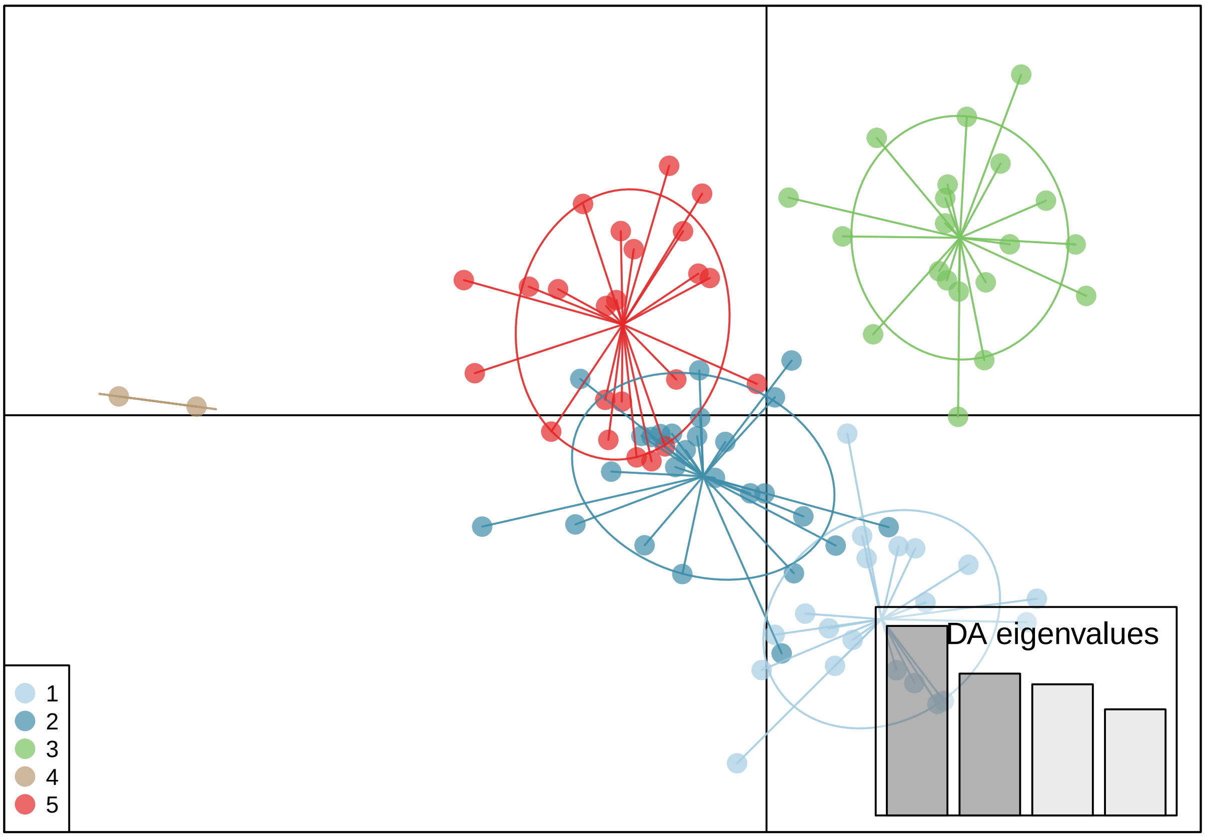
DA eigenvalues
1
2
3
4
5

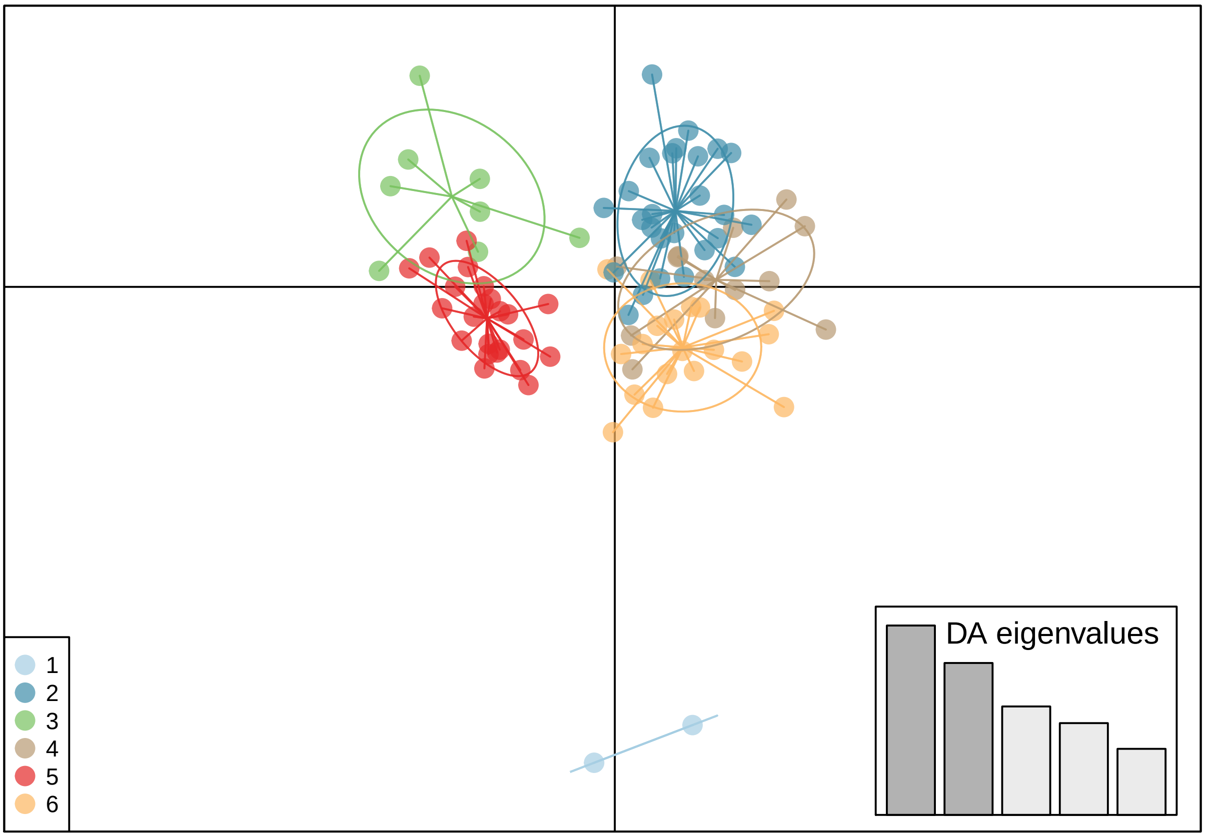
DA eigenvalues
1
2
3
4
5
6

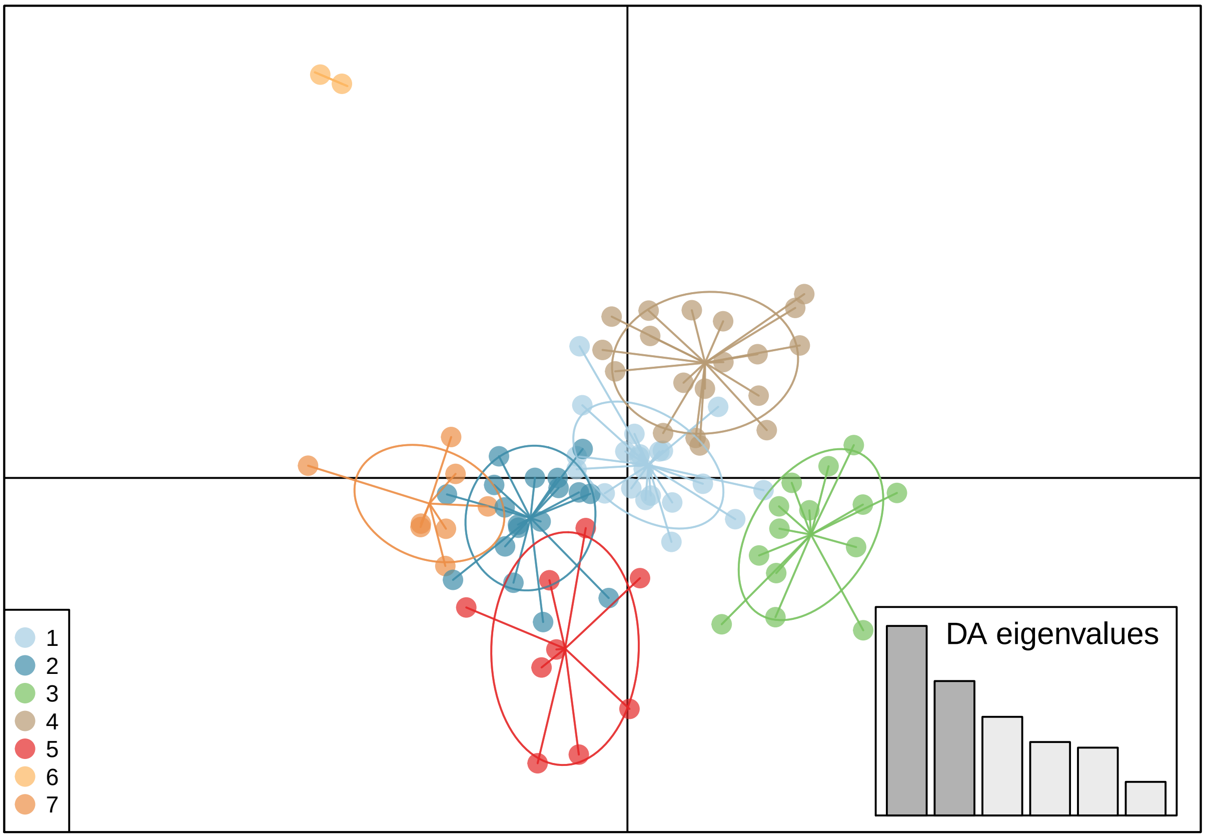
1
2
3
4
5
6
7
DA eigenvalues

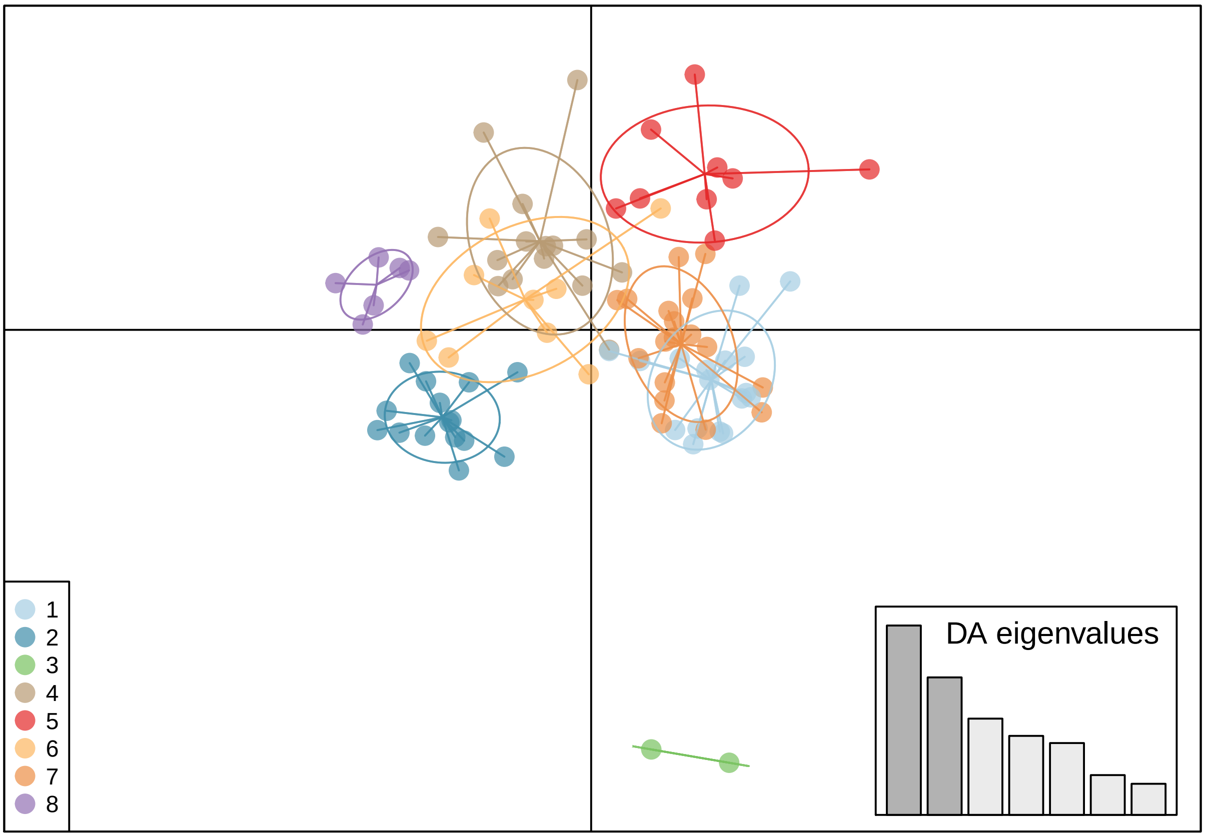
1
2
3
4
5
6
7
8
DA eigenvalues

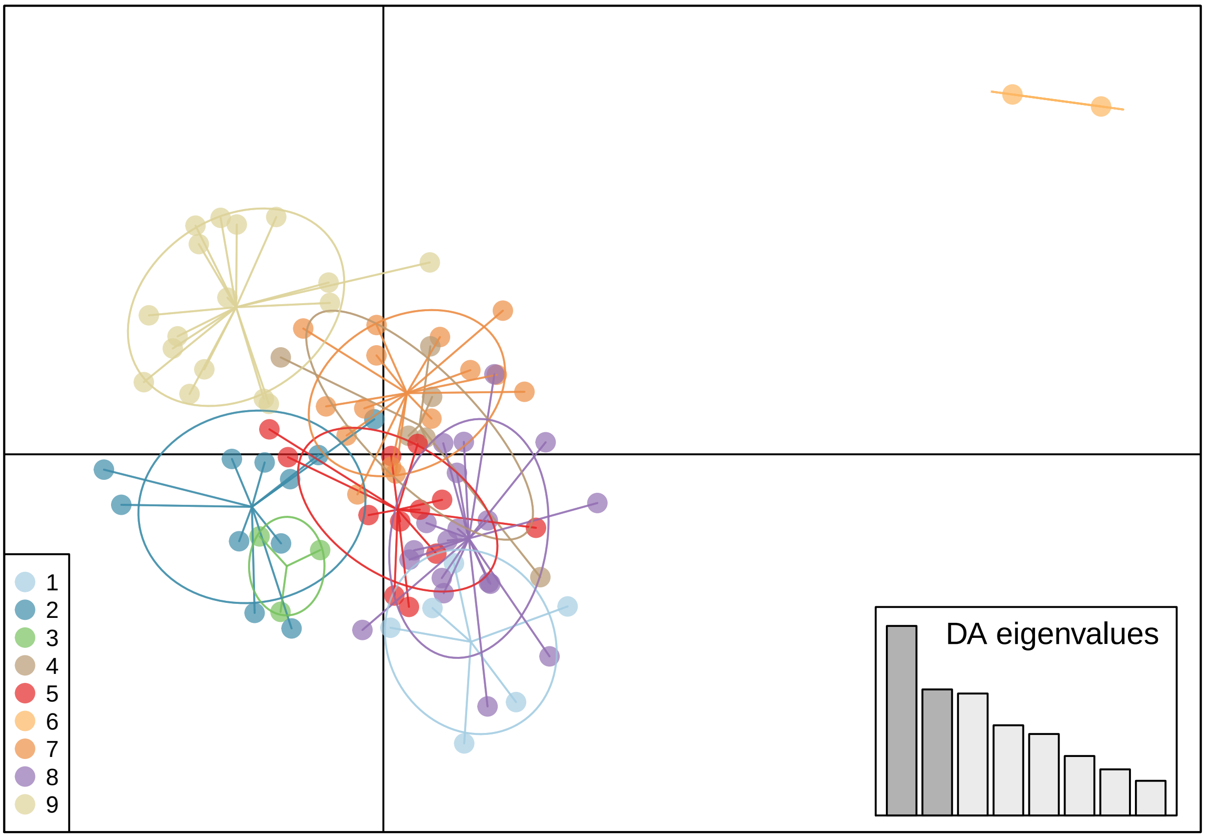


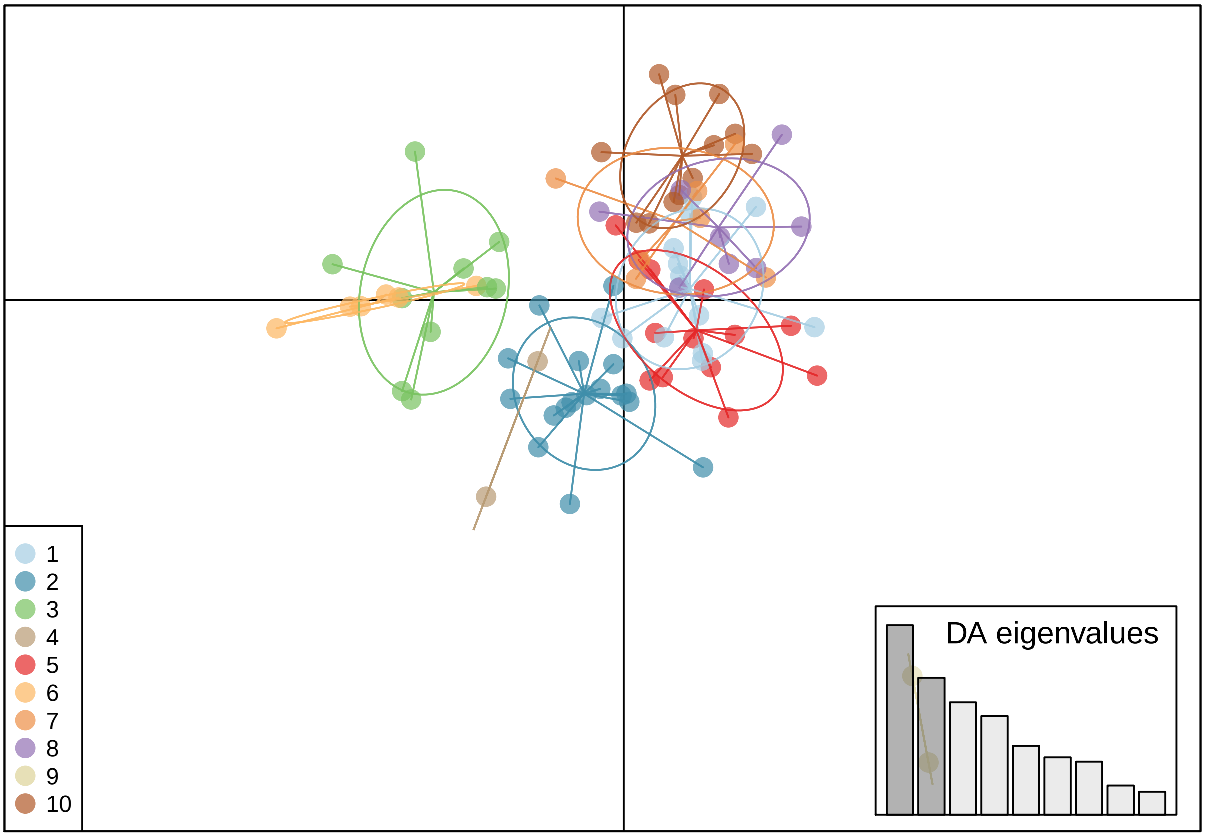


### 10.9.5 DAPC scatterplot - FLO

This code will *colour* individuals according to *sampling location* and **group** individuals according to DAPC **posterior membership**.

```r
for (K in 3:10) {
  dapc <- get(dapc.all.object.names[K - 1])
  x <- dapc$ind.coord[,1]
  y <- dapc$ind.coord[,2]
  group <- paste("Group", as.numeric(dapc$assign))
  pop <- BS.dartcap.WATL.gl$pop
  df <- data.frame(x, y, group, pop)
  colnames(df) <- c("x","y","group","pop")
  gg <- merge(df,aggregate(cbind(mean.x = x,mean.y = y)~group,df,mean),
              by  =  "group")

  plot <- ggplot2::ggplot(gg, ggplot2::aes(x,y,color = pop)) +
    ggplot2::geom_point(size = 5) +
    ggplot2::geom_point(ggplot2::aes(x = mean.x,y = mean.y),size = 5, shape = 17,
                        color = "black") +
    ggplot2::geom_segment(ggplot2::aes(x = mean.x, y = mean.y, xend = x,
                                       yend = y)) +
    ggplot2::scale_colour_manual(values = colours.4) +
    ggplot2::scale_fill_manual(values = colours.4) +
    ggplot2::geom_hline(yintercept = 0) +
    ggplot2::geom_vline(xintercept = 0) +
    ggplot2::labs(subtitle = "",
         y = "PC2",
         x = "PC1",
         title = paste0("K=",K," & PC=",PC, sep = ""),
         caption = "") +
    ggplot2::theme_minimal() +
    ggplot2::theme(
      axis.text = ggplot2::element_text(size = 5),
      axis.title.x = ggplot2::element_text(size = 10),
      axis.title.y = ggplot2::element_text(size = 10),
      legend.text = ggplot2::element_text(size = 7))
  print(plot)
  ggplot2::ggsave(plot, filename = paste0("DArTcap_WATL_DAPC_scatterplot2_K",
                                          K, ".png"),
                  width = 30, height = 15,units = "cm")
}
```

## K=3 & PC=20

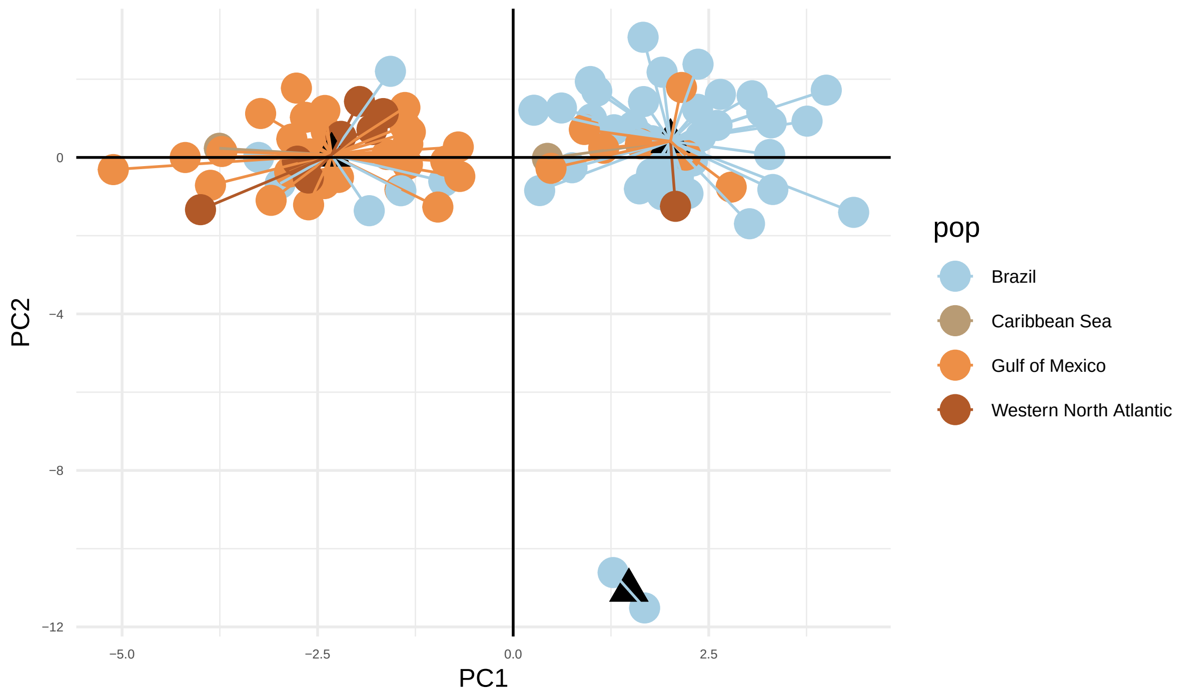


## K=4 & PC=20

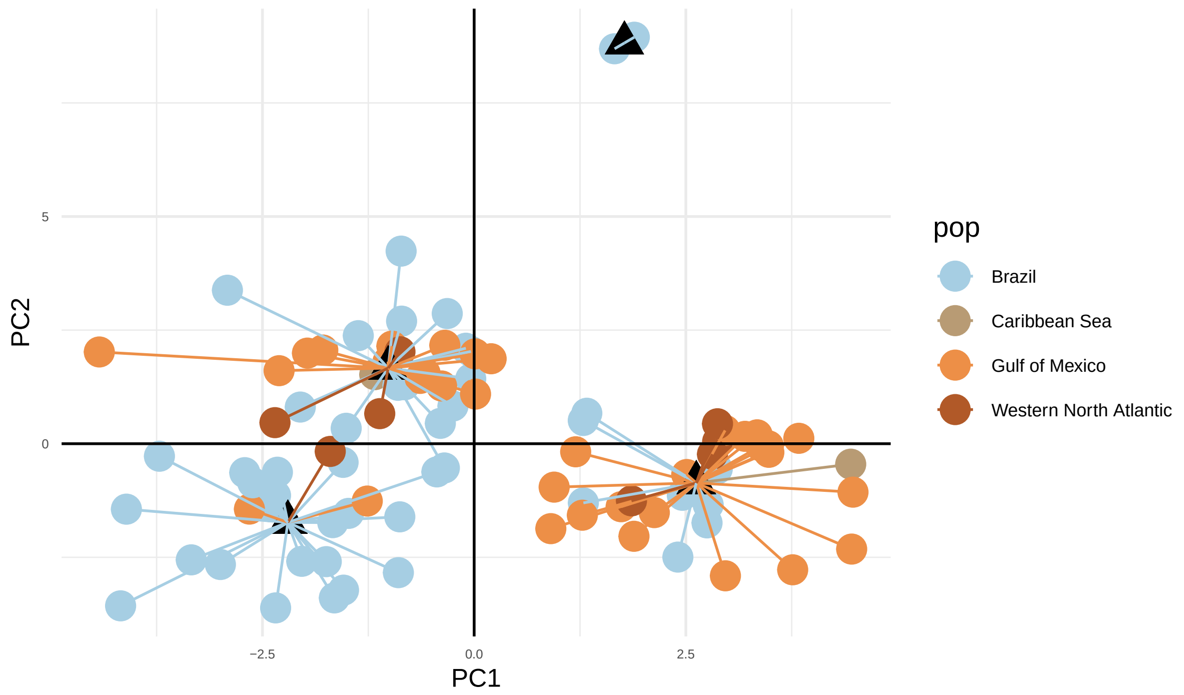

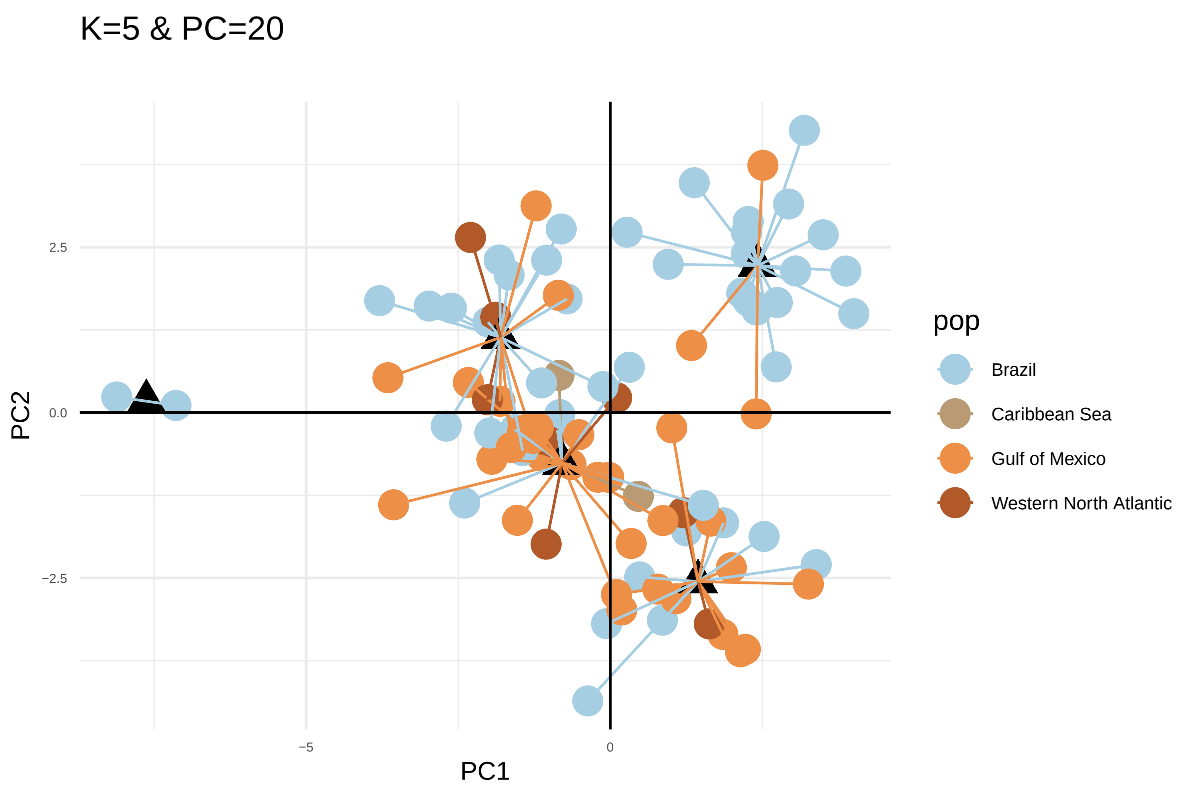
K=5 & PC=20
PC2
PC1
2.5
0.0
−2.5
−5
0
pop
Brazil
Caribbean Sea
Gulf of Mexico
Western North Atlantic

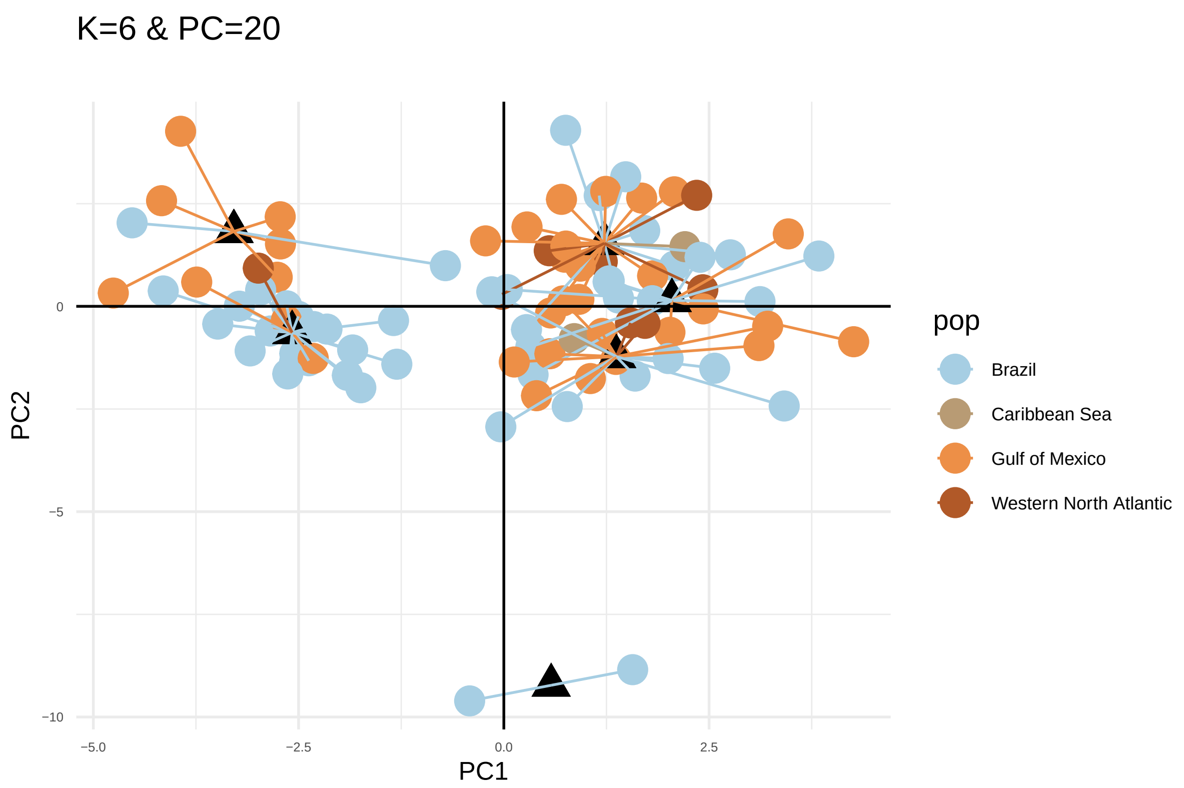
K=6 & PC=20
PC2
PC1
0
−5
−10
−5.0
−2.5
0.0
2.5
pop
Brazil
Caribbean Sea
Gulf of Mexico
Western North Atlantic

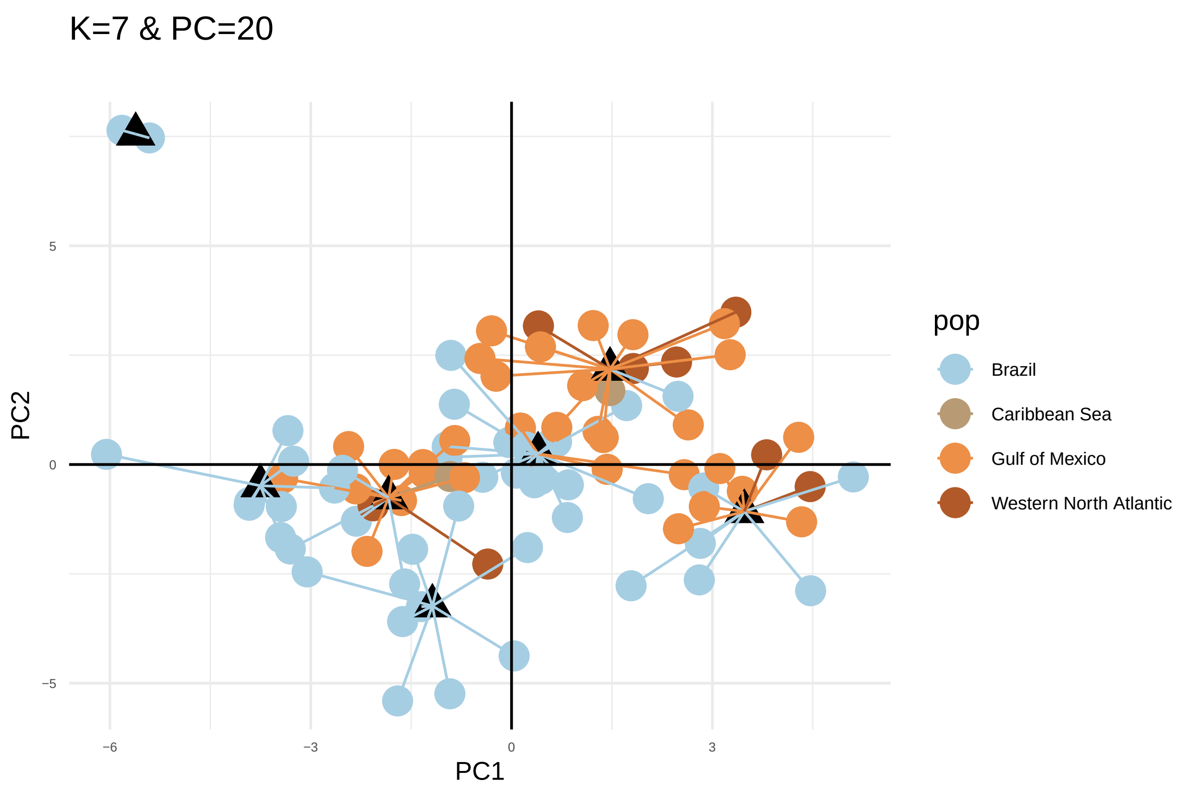
K=7 & PC=20
5
0
−5
−6
−3
0
3
PC1
PC2
pop
Brazil
Caribbean Sea
Gulf of Mexico
Western North Atlantic

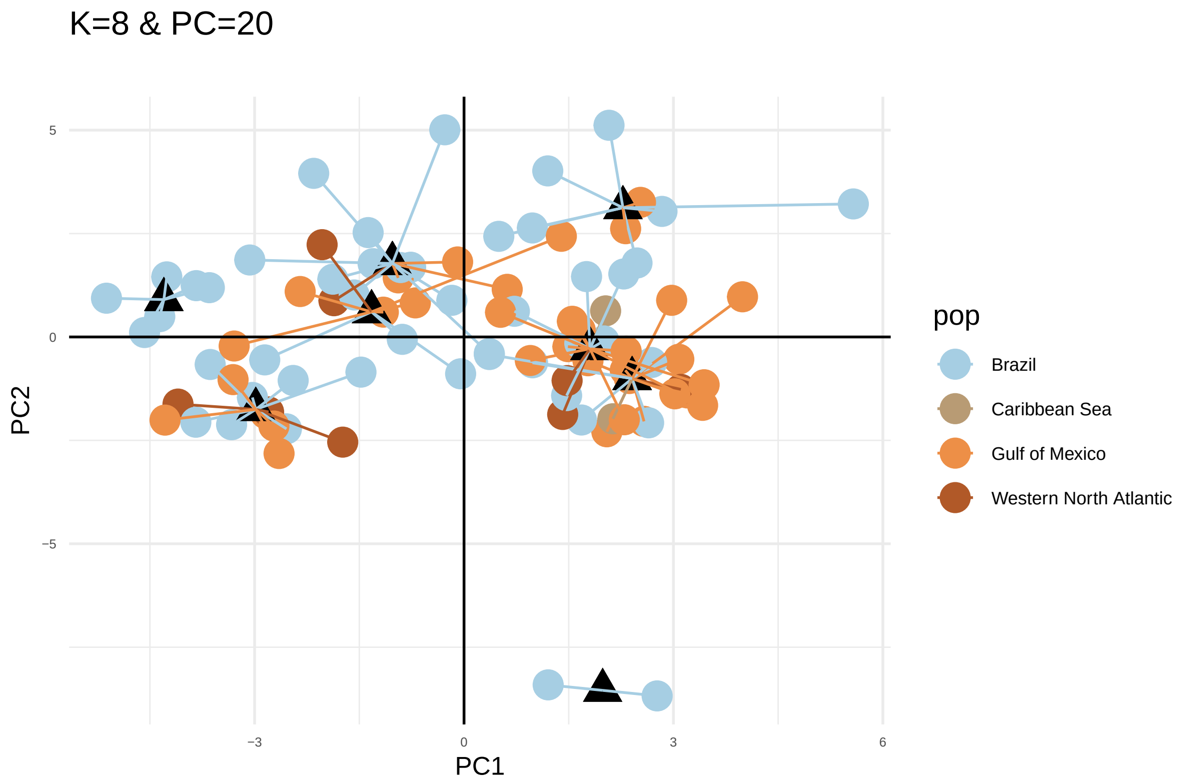
K=8 & PC=20
5
0
−5
−3
0
3
6
PC1
PC2
pop
Brazil
Caribbean Sea
Gulf of Mexico
Western North Atlantic

K=9 & PC=20

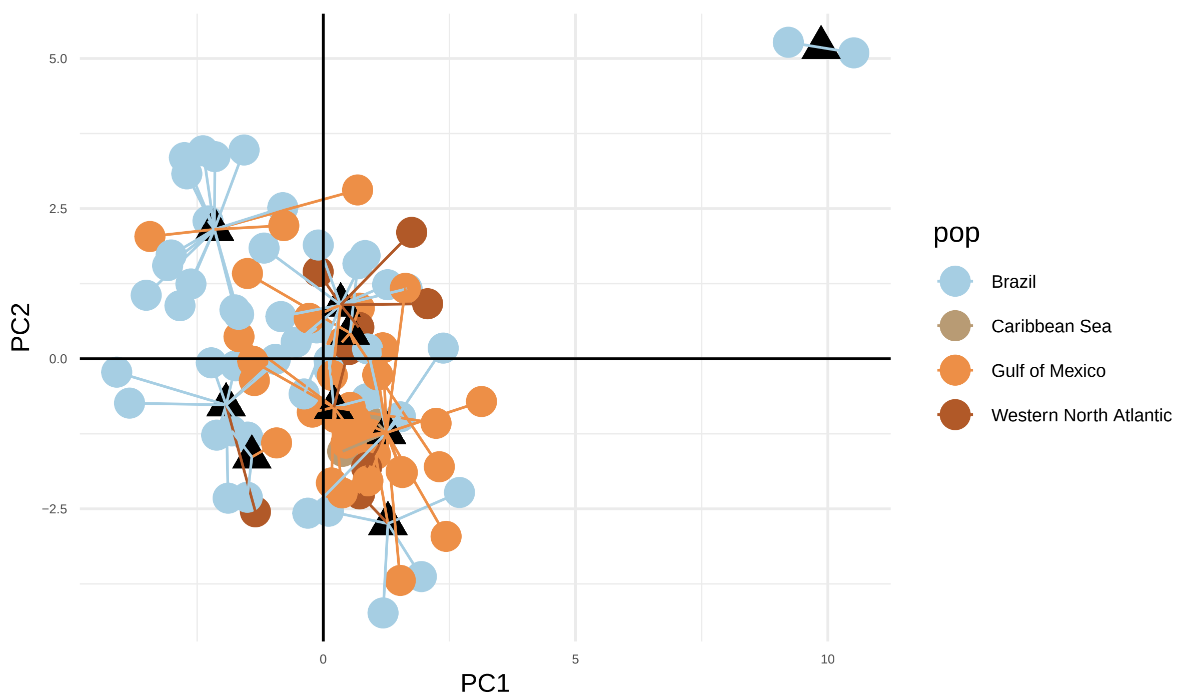


K=10 & PC=20

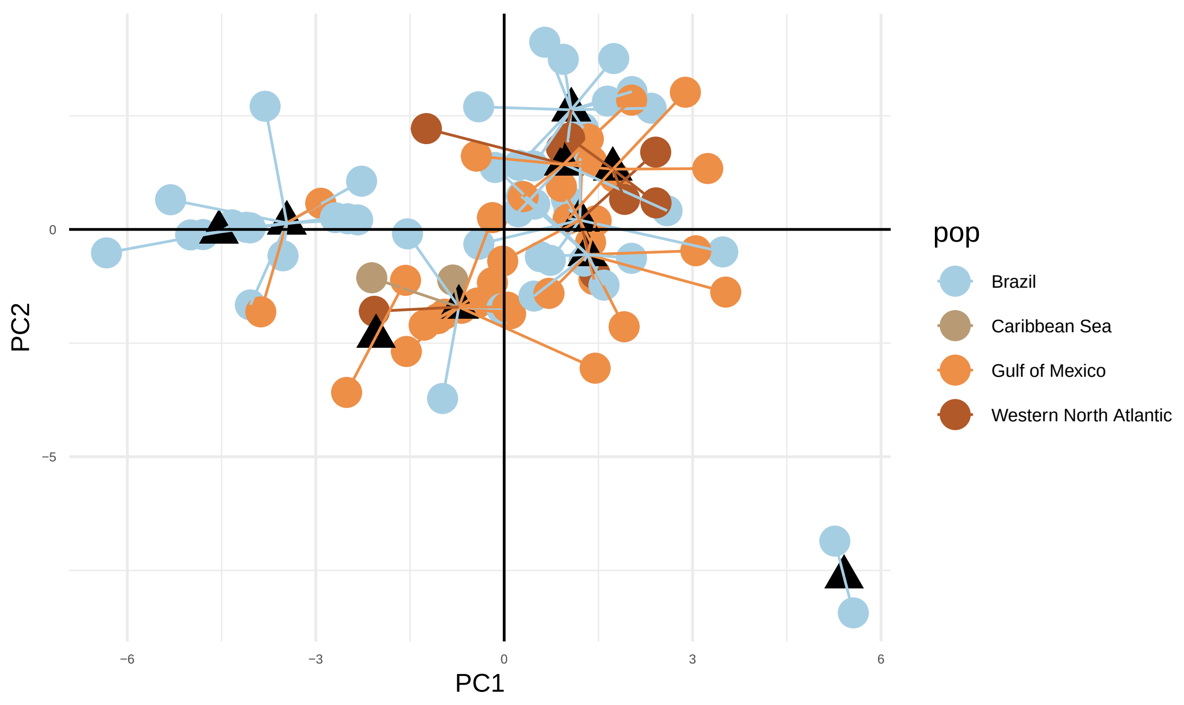

# 11 DATA5: DArTcap - Indo - West Pacific

## 11.1 Set strata

```
strata <- radiator::read_strata("Bull_shark_DArTcap_strata.tsv",
                                keep.two = FALSE)$strata %>%
  dplyr:: filter(SpeciesID_genetic == "Carcharhinus leucas") %>%
  dplyr:: filter(Region %in% c("W-IO","N-IO", "E-IO","E-IO/W-PAC","W-PAC",
                               "Japan", "Fiji"))

readr::write_tsv(strata, path = "Bull_shark_DArTcap_strata5_IWP.tsv")
```

## 11.2 Filter data

1. Marker Reproducibility: 0.98
2. Individual missingness: 0.25
3. Individual Het - low: 0
4. Individual Het - high: 0.07
5. Individual total coverage - low: 10,000
6. Individual total coverage - high: 1,000,000
7. Marker MAC - low: 3
8. Marker coverage - low: 4
9. Marker coverage - high:60
10. Marker maximum missing proportion: 0.1
11. Marker snp position on locus: n
12. Max 2 SNPs per locus
13. 1 SNP per locus according to mac
14. no LD filter
15. Individual Het - low: 0.051
16. Individual Het - high: 0.076
17. Duplicated individuals: 0.2
18. Filter on HWE: 4 pops, 0.001

```
dartcap.data <- "SEQ_SNPs_counts_0_Target_merge.csv"
strata <- "Bull_shark_DArTcap_strata5_IWP.tsv"
BSsnp <- radiator::filter_rad(dartcap.data, strata,
                              parallel.core = 1)
BS.dartcap.IWP.gds <- BSsnp$gds
BS.dartcap.IWP.tidy <- BSsnp$output$tidy.data
print(BS.dartcap.IWP.gds, expand = TRUE, all = TRUE, attribute = TRUE,
      attribute.trim = FALSE)

save(BS.dartcap.IWP.tidy, file = "DArTcap_IWP.Rdata")
```

Table 43:

| FILTERS | VALUES | BEFORE | BLACKLIST |
|---|---|---|---|
| Filter DArT reproducibility | 0.98 | 798 / 26 / 1 / 26335 / 37537 | 0 / 0 / 0 / 1043 / 2250 |
| Filter monomorphic markers | NA | 798 / 26 / 1 / 25292 / 35287 | 0 / 0 / 0 / 11939 / 19900 |
| Filter markers in common | NA | 798 / 26 / 1 / 13353 / 15387 | 0 / 0 / 0 / 187 / 224 |
| Filter individuals based on missingness | 0.25 | 798 / 26 / 1 / 13166 / 15163 | 12 / 0 / 0 / 0 / 0 |
| Filter individuals based on heterozygosity | 0 0.07 | 786 / 26 / 1 / 13166 / 15163 | 9 / 0 / 0 / 0 / 0 |
| Filter individuals based on total coverage | 10000 1e+07 | 777 / 26 / 1 / 13166 / 15163 | 2 / 0 / 0 / 0 / 0 |
| Filter monomorphic markers | NA | 775 / 26 / 1 / 13166 / 15163 | 0 / 0 / 0 / 2030 / 2633 |
| Filter MAC | 3 | 775 / 26 / 1 / 11136 / 12530 | 0 / 0 / 0 / 5456 / 6385 |
| Filter coverage min / max | 4-60 | 775 / 26 / 1 / 5680 / 6145 | 0 / 0 / 0 / 1612 / 1695 |
| Filter genotyping | 0.1 | 775 / 26 / 1 / 4068 / 4450 | 0 / 0 / 0 / 591 / 640 |
| Filter SNPs position on the read | all | 775 / 26 / 1 / 3477 / 3810 | 0 / 0 / 0 / 0 / 0 |
| Filter markers snp number | 3 | 775 / 26 / 1 / 3477 / 3810 | 0 / 0 / 0 / 5 / 21 |
| Filter short ld | mac | 775 / 26 / 1 / 3472 / 3789 | 0 / 0 / 0 / 0 / 317 |
| detect mixed genomes | 0.051 0.076 | 775 / 26 / 1 / 3472 / 3472 | 71 / 0 / 0 / 0 / 0 |
| Filter monomorphic markers | NA | 704 / 26 / 1 / 3472 / 3472 | 0 / 0 / 0 / 9 / 9 |
| detect duplicate genomes | 0.2 | 704 / 26 / 1 / 3463 / 3463 | 68 / 0 / 0 / 0 / 0 |
| Filter monomorphic markers | NA | 636 / 26 / 1 / 3463 / 3463 | 0 / 0 / 0 / 3 / 3 |
| Filter HWE | 40.001 | 636 / 26 / 1 / 3460 / 3460 | 0 / 0 / 0 / 9 / 9 |

### 11.2.1 Remove extra low and high heterozygous individuals

Because the average heterozygosity per sampling location is not even, certain individuals cannot be excluded without removing 'good' individuals.

```
rm <-  c("CL-ARS021-F","CL-GOM025-F.1","CL-GOM012-F","CL-SAF014-M","CL-SAF002-F",
         "CL-BRZ038-U","CLF003-F","CL-GOM011-F","CL-SAF015-F","CL-SAF003-M",
         "CL-PNG005-U","CLCR056-M","CLCR047.1","CLCR055-M","CLCR066-M")

BS.dartcap.IWP.tidy <- BS.dartcap.IWP.tidy[!BS.dartcap.IWP.tidy$INDIVIDUALS %in% rm,]
length(unique(BS.dartcap.IWP.tidy$INDIVIDUALS)) # 635
```

'

## 11.3 Save data

```
Convert <- radiator::genomic_converter(BS.dartcap.IWP.tidy,
                                       output = c("genlight", "stockr",
                                                  "pcadapt", "gtypes", "rubias"))
BS.dartcap.IWP.gl <- Convert$genlight
BS.dartcap.IWP.stkr <- Convert$stockr
BS.dartcap.IWP.pc <- Convert$pcadapt
BS.dartcap.IWP.gt <- Convert$gtypes
BS.dartcap.IWP.rubias <- Convert$rubias


markers.meta <- radiator::extract_markers_metadata(BS.dartcap.IWP.gds,
                                                   whitelist = TRUE)
individuals.meta <- radiator::extract_individuals_metadata(BS.dartcap.IWP.gds,
                                                           whitelist = TRUE)
individuals.meta <- individuals.meta[individuals.meta$INDIVIDUALS %in%
                                       BS.dartcap.IWP.gl$ind.names,]
BS.dartcap.IWP.gl$other$ind.metrics <- individuals.meta
BS.dartcap.IWP.gl$other$loc.metrics <- markers.meta
BS.dartcap.IWP.gt@schemes <- individuals.meta

pop.levels <- c("South Africa", "Mozambique", "Reunion" , "Seychelles",
                "Arabian Sea", "Sri Lanka", "Thailand", "Indonesia",
                "Papua New Guinea" , "Fitzroy River", "Daly River",
                "Adelaide River","Darwin Coastal",  "South Alligator River",
                "East Alligator River","Blue mud Bay", "Roper River",
                "Towns River","Unknown","Wenlock River","Trinity inlet",
                "Clarence River", "Sydney Harbour", "Okinawa", "Urauchi River",
                "Fiji")
BS.dartcap.IWP.gl$pop <- factor(BS.dartcap.IWP.gl$other$ind.metrics$Site2,
                                levels = pop.levels)
BS.dartcap.IWP.gl <- BS.dartcap.IWP.gl[order(BS.dartcap.IWP.gl$pop,
                                             BS.dartcap.IWP.gl$ind.names),]
BS.dartcap.IWP.gl <- BS.dartcap.IWP.gl[order(BS.dartcap.IWP.gl$pop,
                                             BS.dartcap.IWP.gl$ind.names)]
BS.dartcap.IWP.rubias$repunit <- factor(BS.dartcap.IWP.rubias$repunit,
                                        levels = pop.levels)

save(BS.dartcap.IWP.tidy, BS.dartcap.IWP.gl, BS.dartcap.IWP.stkr,
     BS.dartcap.IWP.pc,BS.dartcap.IWP.gt,BS.dartcap.IWP.rubias,
     markers.meta, individuals.meta, file = "DArTcap_IWP.Rdata")
```

## 11.4 Load data

```
load("DArTcap_IWP.Rdata")
pop.levels <- c("South Africa", "Mozambique", "Reunion" , "Seychelles",
                "Arabian Sea", "Sri Lanka", "Thailand", "Indonesia",
                "Papua New Guinea" , "Fitzroy River", "Daly River",
                "Adelaide River","Darwin Coastal",  "South Alligator River",
                "East Alligator River","Blue mud Bay", "Roper River",
                "Towns River","Unknown","Wenlock River","Trinity inlet",
                "Clarence River", "Sydney Harbour","Okinawa", "Urauchi River",
                "Fiji")
BS.dartcap.IWP.gl$pop <- factor(BS.dartcap.IWP.gl$pop, levels = pop.levels)
BS.dartcap.IWP.gl <- BS.dartcap.IWP.gl[order(BS.dartcap.IWP.gl$pop,
                                             BS.dartcap.IWP.gl$ind.names),]

pop.levels1 <- stringr::str_replace_all(pop.levels, " ", "_")
BS.dartcap.IWP.tidy$POP_ID <- factor(BS.dartcap.IWP.tidy$POP_ID,
                                     levels = pop.levels1)
BS.dartcap.IWP.tidy <- BS.dartcap.IWP.tidy[order(BS.dartcap.IWP.tidy$POP_ID,
                                                 BS.dartcap.IWP.tidy$INDIVIDUALS),]

adegenet::nInd(BS.dartcap.IWP.gl) # 635
adegenet::nLoc(BS.dartcap.IWP.gl) # 3451
summary(BS.dartcap.IWP.gl$pop)
sum(duplicated(BS.dartcap.IWP.gl$other$ind.metrics$`Genetic code`))# 0 replicate left
sum(baits$CloneID %in% BS.dartcap.IWP.gl$other$loc.metrics$LOCUS) #74
```

```
## [1] 635
## [1] 3451
##         South Africa            Mozambique               Reunion
##                   21                    11                    28
##           Seychelles           Arabian Sea             Sri Lanka
##                   36                    15                    12
##             Thailand             Indonesia      Papua New Guinea
##                    4                     4                     9
##        Fitzroy River            Daly River        Adelaide River
##                    3                    49                    18
##       Darwin Coastal South Alligator River  East Alligator River
##                   13                    88                    47
##         Blue mud Bay           Roper River           Towns River
##                   17                     9                    10
##              Unknown         Wenlock River         Trinity inlet
##                   10                    24                     4
##       Clarence River        Sydney Harbour               Okinawa
##                   74                    65                     9
##        Urauchi River                  Fiji
##                   35                    20
## [1] 0
## [1] 74
```

## 11.5 Remove sex-linked markers

```
load("Sex-linked_marker_DArTcap.Rdata")
load("Sex_results.Rdata")
sum(bull_shark$sexy.summary$CLONE_ID %in% SLM$sexy.summary$CLONE_ID) #33
sum(SLM$sexy.summary$CLONE_ID %in% bull_shark$sexy.summary$CLONE_ID) #23
sex.markers <- unique(c(bull_shark$sexy.summary$CLONE_ID,
                        SLM$sexy.summary$CLONE_ID))
```

```r
sum(BS.dartcap.IWP.gl$loc.names %in% sex.markers) #35
locnames <- BS.dartcap.IWP.gl$loc.names
BS.dartcap.IWP.gl <- BS.dartcap.IWP.gl[,!locnames %in% sex.markers]
BS.dartcap.IWP.gl$other$loc.metrics <- BS.dartcap.IWP.gl$other$loc.metrics[
  !locnames %in% sex.markers,]

sum(sex.markers %in% unique(BS.dartcap.IWP.tidy$LOCUS)) # 35
BS.dartcap.IWP.tidy <- BS.dartcap.IWP.tidy[!BS.dartcap.IWP.tidy$LOCUS %in%
                                             sex.markers,]

locus.gt <- stringr::str_match(pattern = "__\\s*(.*?)\\s*__",
                               string =  BS.dartcap.IWP.gt@data$locus)[,2]
BS.dartcap.IWP.gt <- BS.dartcap.IWP.gt[,!locus.gt %in% sex.markers, ,
                                       drop = TRUE]

locus.rubias <- stringr::str_match(pattern = "__\\s*(.*?)\\s*__",
                                   string =  colnames(BS.dartcap.IWP.rubias))[,2]
BS.dartcap.IWP.rubias <- BS.dartcap.IWP.rubias[,!locus.rubias %in% sex.markers]
```

```
## [1] 33
## [1] 23
## [1] 35
## [1] 35
```

## 11.6 Genetic diversity

```r
set.seed(124)
pop.levels <- c( "South Africa", "Mozambique", "Reunion", "Seychelles",
                 "Arabian Sea", "Sri Lanka", "Thailand", "Indonesia",
                 "Papua New Guinea" , "Fitzroy River", "Daly River", "Adelaide River",
                 "Darwin Coastal",  "South Alligator River", "East Alligator River",
                 "Blue mud Bay", "Roper River", "Towns River", "Unknown",
                 "Wenlock River", "Trinity inlet", "Clarence River", "Sydney Harbour",
                 "Okinawa", "Urauchi River", "Fiji")

gl <- BS.dartcap.IWP.gl
gl$pop <- factor(gl$other$ind.metrics$Site2, levels = pop.levels)
gl <- gl[order(gl$pop,gl$ind.names)]


gl2gpop(gl, filename = "BS_dartcap_IWP_genepop.txt")
bastat <-diveRsity::basicStats(infile = "BS_dartcap_IWP_genepop.txt",
                               outfile = "BS_dartcap_IWP_BasicStat_output.txt",
                               fis_ci = TRUE,ar_ci = TRUE, fis_boots = 1000,
                               ar_boots = 1000,mc_reps = 1000,
                               rarefaction = FALSE, ar_alpha = 0.05,
                               fis_alpha = 0.05)


length(bastat$main_tab) #26
gendiv <- data.frame(
  "South Africa" = bastat$main_tab[[1]]$overall,
  "Mozambique" = bastat$main_tab[[2]]$overall,
  "Reunion" = bastat$main_tab[[3]]$overall,
  "Seychelles" = bastat$main_tab[[4]]$overall,
  "Arabian Sea" = bastat$main_tab[[5]]$overall,
  "Sri Lanka" = bastat$main_tab[[6]]$overall,
```

```r
  "Thailand" = bastat$main_tab[[7]]$overall,
  "Indonesia" = bastat$main_tab[[8]]$overall,
  "Papua New Guinea" = bastat$main_tab[[9]]$overall,
  "Fitzroy River" = bastat$main_tab[[10]]$overall,
  "Daly River" = bastat$main_tab[[11]]$overall,
  "Adelaide River" = bastat$main_tab[[12]]$overall,
  "Darwin Coastal" = bastat$main_tab[[13]]$overall,
  "South Alligator River" = bastat$main_tab[[14]]$overall,
  "East Alligator River" = bastat$main_tab[[15]]$overall,
  "Blue mud Bay" = bastat$main_tab[[16]]$overall,
  "Roper River" = bastat$main_tab[[17]]$overall,
  "Towns River" = bastat$main_tab[[18]]$overall,
  "Unknown" = bastat$main_tab[[19]]$overall,
  "Wenlock River" = bastat$main_tab[[20]]$overall,
  "Trinity inlet" = bastat$main_tab[[21]]$overall,
  "Clarence River" = bastat$main_tab[[22]]$overall,
  "Sydney Harbour" = bastat$main_tab[[23]]$overall,
  "Urauchi River" = bastat$main_tab[[24]]$overall,
  "Okinawa" = bastat$main_tab[[25]]$overall,
  "Fiji" = bastat$main_tab[[26]]$overall,
  check.names = FALSE)
row.names(gendiv) <- rownames(bastat$main_tab[[1]])

df <- as.data.frame(gl)
df[df == "0"] <- "11"
df[df == "1"] <- "12"
df[df == "2"] <- "22"
gen.gi <- adegenet::df2genind(df, ploidy = 2,ncode = 2, pop = gl$pop)
ris_basic <- hierfstat::basic.stats(gen.gi)
fis.hierfstat <- apply(ris_basic$Fis, 2, mean, na.rm=T)
fis.CI <- hierfstat::boot.ppfis(gen.gi, nboot = 1000, quant = c(0.025,0.975),
                                diploid = TRUE,dig = 4)
df <- data.frame(fis.hierfstat,fis.CI.low = fis.CI$fis.ci[,1],
                 fis.CI.high = fis.CI$fis.ci[,2])
df <- as.data.frame(t(df))
gendiv <- rbind(gendiv, df)

readr::write_tsv(gendiv, path = "DArTcap_IWP_gendiv.txt")
save(gendiv, file = "DArTcap_IWP_gendiv.Rdata")
```

```r
load("DArTcap_IWP_gendiv.Rdata")
shortnames <- c("SAF", "MOZ", "RUN" , "SEY", "ARS", "SRL", "TAI", "IND", "PNG" ,
                "FZR", "DAR", "ADR","DWC",  "SAR", "EAR","BMB", "ROR","TOR","UNK",
                "WER","TRI","CLR", "SYH", "URR","OKI", "FIJ")
knitr::kable(gendiv, col.names = shortnames, digits = 4,caption = "") %>%
  kableExtra::kable_styling(full_width = FALSE,font_size = 5,
                            latex_options = c( "hold_position")) %>%
  kableExtra::landscape()
```

Table 44:

| | SAF | MOZ | RUN | SEY | ARS | SRL | TAI | IND | PNG | FZR | DAR | ADR | DWC | SAR | EAR | BMB | ROR | TOR | UNK | WER | TRI |
|---|---|---|---|---|---|---|---|---|---|---|---|---|---|---|---|---|---|---|---|---|---|
| ar | 1.1500 | 1.1290 | 1.1480 | 1.1500 | 1.1540 | 1.1480 | 1.1340 | 1.1230 | 1.1420 | 1.1120 | 1.1540 | 1.1490 | 1.1530 | 1.1530 | 1.1500 | 1.1550 | 1.1390 | 1.1410 | 1.1450 | 1.1530 | 1.1360 |
| size | 20.7310 | 10.2200 | 27.3360 | 35.4140 | 14.8220 | 11.8720 | 3.9710 | 3.9530 | 8.8880 | 2.9070 | 48.4430 | 17.7910 | 12.8110 | 86.9180 | 46.4840 | 16.7170 | 8.5940 | 9.8000 | 9.7930 | 23.6650 | 3.9430 |
| obs__het | 0.0610 | 0.0570 | 0.0600 | 0.0610 | 0.0640 | 0.0630 | 0.0660 | 0.0620 | 0.0610 | 0.0610 | 0.0610 | 0.0620 | 0.0640 | 0.0610 | 0.0610 | 0.0660 | 0.0600 | 0.0600 | 0.0630 | 0.0620 | 0.0680 |
| exp__het | 0.0640 | 0.0620 | 0.0640 | 0.0640 | 0.0650 | 0.0630 | 0.0590 | 0.0550 | 0.0600 | 0.0530 | 0.0650 | 0.0630 | 0.0650 | 0.0640 | 0.0630 | 0.0660 | 0.0630 | 0.0610 | 0.0630 | 0.0650 | 0.0610 |
| uexp__het | 0.0660 | 0.0660 | 0.0650 | 0.0650 | 0.0670 | 0.0660 | 0.0670 | 0.0630 | 0.0640 | 0.0630 | 0.0650 | 0.0650 | 0.0680 | 0.0650 | 0.0640 | 0.0680 | 0.0670 | 0.0640 | 0.0660 | 0.0660 | 0.0700 |
| fis | 0.0320 | 0.0540 | 0.0470 | 0.0300 | 0.0100 | -0.0020 | -0.1320 | -0.1330 | -0.0200 | -0.1720 | 0.0390 | 0.0120 | -0.0010 | 0.0360 | 0.0320 | -0.0010 | 0.0180 | -0.0020 | -0.0160 | 0.0290 | -0.1280 |
| hwe__glb | 1.0000 | 1.0000 | 1.0000 | 1.0000 | 1.0000 | 1.0000 | 1.0000 | 1.0000 | 1.0000 | 1.0000 | 1.0000 | 1.0000 | 1.0000 | 1.0000 | 1.0000 | 1.0000 | 1.0000 | 1.0000 | 1.0000 | 1.0000 | 1.0000 |
| hwe__hom | 1.0000 | 1.0000 | 1.0000 | 1.0000 | 1.0000 | 1.0000 | 1.0000 | 1.0000 | 1.0000 | 1.0000 | 1.0000 | 1.0000 | 1.0000 | 1.0000 | 1.0000 | 1.0000 | 1.0000 | 1.0000 | 1.0000 | 1.0000 | 1.0000 |
| hwe__het | 1.0000 | 1.0000 | 1.0000 | 1.0000 | 1.0000 | 1.0000 | 1.0000 | 1.0000 | 1.0000 | 1.0000 | 1.0000 | 1.0000 | 1.0000 | 1.0000 | 1.0000 | 1.0000 | 1.0000 | 1.0000 | 1.0000 | 1.0000 | 1.0000 |
| fis__lo | -0.0010 | -0.0350 | 0.0140 | 0.0070 | -0.0430 | -0.0750 | -0.3760 | -0.5440 | -0.1190 | -1.0000 | 0.0220 | -0.0290 | -0.0590 | 0.0270 | 0.0140 | -0.0450 | -0.0940 | -0.0840 | -0.0970 | -0.0060 | -0.3810 |
| fis__hi | 0.0270 | 0.0470 | 0.0440 | 0.0290 | 0.0020 | -0.0120 | -0.1320 | -0.1330 | -0.0290 | -0.1720 | 0.0380 | 0.0060 | -0.0100 | 0.0370 | 0.0300 | -0.0080 | 0.0110 | -0.0100 | -0.0230 | 0.0260 | -0.1280 |
| ar__lo | 1.1130 | 1.0440 | 1.1110 | 1.1140 | 1.1150 | 1.1120 | 1.0590 | 1.0470 | 1.1120 | 1.0040 | 1.1190 | 1.1120 | 1.1150 | 1.1220 | 1.1140 | 1.1180 | 1.0920 | 1.1040 | 1.1100 | 1.1150 | 1.0520 |
| ar__hi | 1.1630 | 1.1580 | 1.1600 | 1.1620 | 1.1730 | 1.1720 | 1.1630 | 1.1590 | 1.1590 | 1.1500 | 1.1650 | 1.1640 | 1.1730 | 1.1640 | 1.1620 | 1.1760 | 1.1620 | 1.1610 | 1.1630 | 1.1680 | 1.1680 |
| fis.hierfstat | 0.0554 | 0.0983 | 0.0641 | 0.0433 | 0.0429 | 0.0388 | -0.0044 | -0.0062 | 0.0349 | 0.0007 | 0.0485 | 0.0394 | 0.0368 | 0.0418 | 0.0423 | 0.0283 | 0.0727 | 0.0475 | 0.0338 | 0.0490 | -0.0008 |
| fis.CI.low | 0.0563 | 0.1030 | 0.0615 | 0.0396 | 0.0329 | 0.0192 | -0.0185 | -0.0232 | 0.0306 | -0.0037 | 0.0487 | 0.0359 | 0.0324 | 0.0410 | 0.0394 | 0.0184 | 0.0741 | 0.0480 | 0.0311 | 0.0496 | -0.0147 |
| fis.CI.high | 0.0928 | 0.1607 | 0.0973 | 0.0672 | 0.0731 | 0.0668 | 0.0560 | 0.0587 | 0.0789 | 0.0915 | 0.0735 | 0.0718 | 0.0753 | 0.0626 | 0.0669 | 0.0581 | 0.1286 | 0.0990 | 0.0783 | 0.0796 | 0.0589 |

### 11.6.1 Number of monomorphic markers per population

```
pop.levels <- c( "South Africa", "Mozambique", "Reunion", "Seychelles",
                 "Arabian Sea", "Sri Lanka", "Thailand", "Indonesia",
                 "Papua New Guinea" , "Fitzroy River", "Daly River", "Adelaide River",
                 "Darwin Coastal",  "South Alligator River", "East Alligator River",
                 "Blue mud Bay", "Roper River", "Towns River", "Unknown",
                 "Wenlock River", "Trinity inlet", "Clarence River", "Sydney Harbour",
                 "Urauchi River", "Okinawa", "Fiji")
monomorphics <- c()
for (i in 1:length(pop.levels)) {
  x <- BS.dartcap.IWP.gl[BS.dartcap.IWP.gl$pop == pop.levels[i]]
  x2 <- dartR::gl.filter.monomorphs(x, verbose = 0)
  locs <-  adegenet::nLoc(x) - adegenet::nLoc(x2)
  monomorphics[i] <- locs
}
names(monomorphics) <- pop.levels
shortnames <- c("SAF", "MOZ", "RUN" , "SEY", "ARS", "SRL", "TAI", "IND", "PNG" ,
                "FZR", "DAR", "ADR","DWC",  "SAR", "EAR","BMB", "ROR","TOR","UNK",
                "WER","TRI","CLR", "SYH", "URR","OKI", "FIJ")
knitr::kable(t(as.table(monomorphics)), col.names = shortnames, digits = 4,
             caption = "") %>%
  kableExtra::kable_styling(full_width = FALSE,font_size = 5,
                            latex_options = c( "hold_position"))
```

Table 45:

| SAF | MOZ | RUN | SEY | ARS | SRL | TAI | IND | PNG | FZR | DAR | ADR | DWC | SAR | EAR | BMB | ROR | TOR | UNK | WE[illegible] |
|---|---|---|---|---|---|---|---|---|---|---|---|---|---|---|---|---|---|---|---|
| 2050 | 2406 | 1947 | 1837 | 2119 | 2306 | 2773 | 2825 | 2469 | 2919 | 1624 | 2134 | 2231 | 1235 | 1690 | 2087 | 2489 | 2452 | 2415 | 195[illegible] |

### 11.6.2 Nucleotide diversity and inbreeding

```
pi.sum <- radiator::pi(
  data = BS.dartcap.IWP.tidy,
  read.length = 70,
  parallel.core = parallel::detectCores() - 1,
  path.folder = NULL,
  verbose = TRUE
)

FIS.sum<- radiator::ibdg_fh(data = BS.dartcap.IWP.tidy,
        path.folder = NULL, verbose = TRUE)

save(pi.sum, FIS.sum, file = "DArTcap_IWP_pi_FIS_Sites2.Rdata")
```

```
load("DArTcap_IWP_pi_FIS_Sites2.Rdata")
pi.sum$boxplot.pi
```

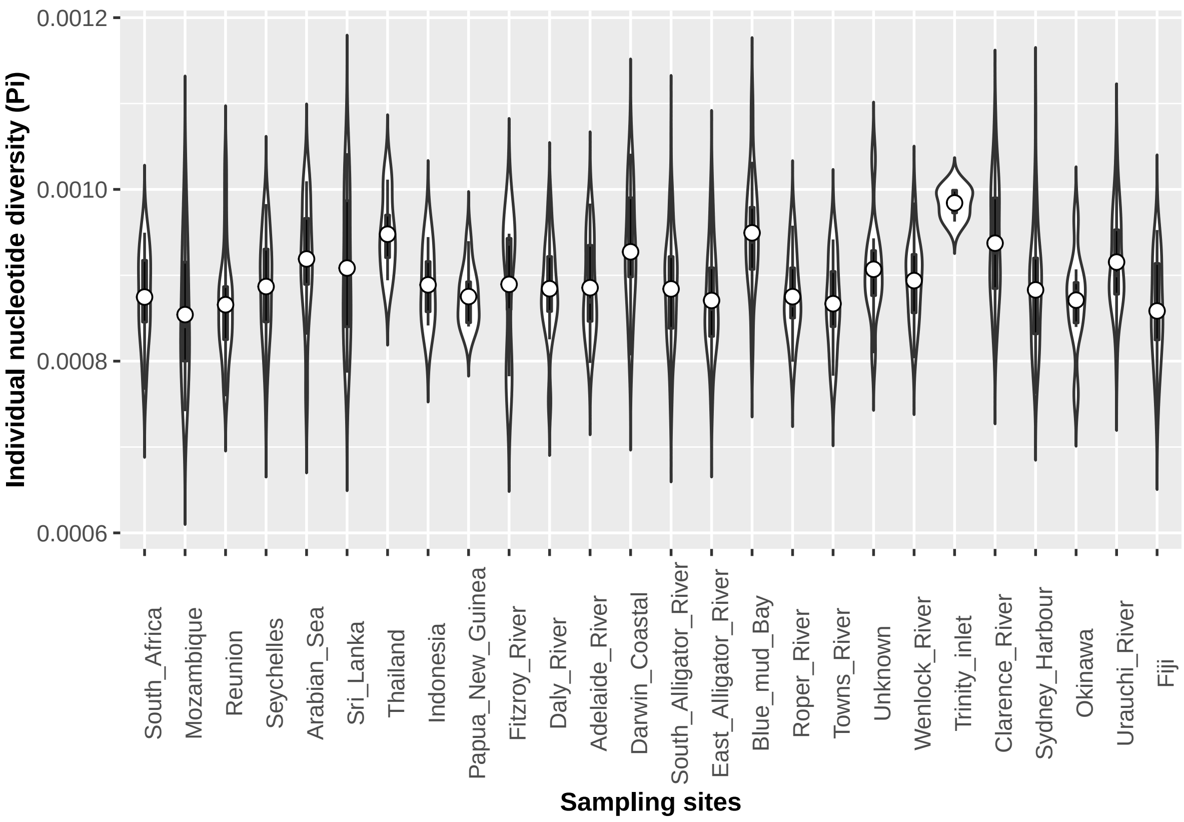


```
FIS.sum$fh.box.plot
```

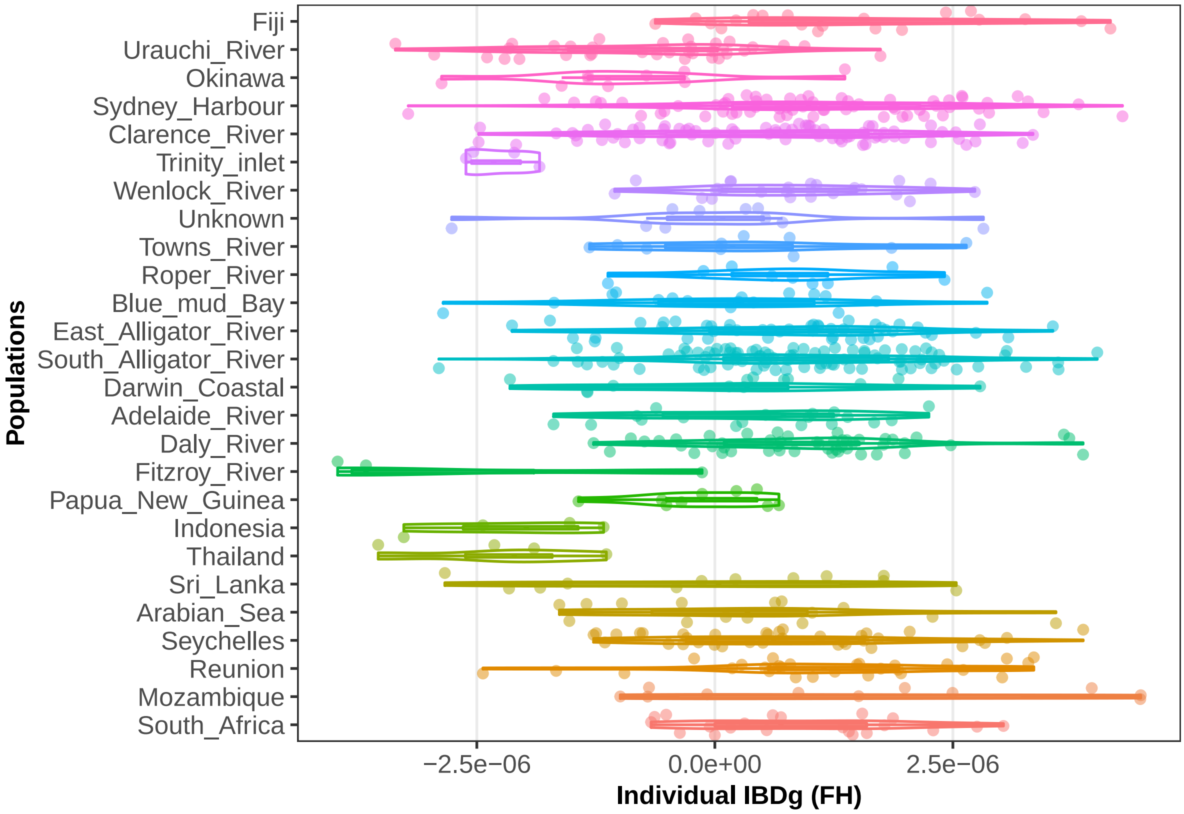

## 11.7 Genetic differentiation

```
Fst.gt <- strataG::popStructTest(
    BS.dartcap.IWP.gt,
    nrep = 1000,
    stats = "all",
    type = "pairwise",
    keep.null = FALSE,
    quietly = TRUE,
    nb.cores =  parallel::detectCores() - 1,
    write.output = FALSE
  )
save(Fst.gt, file = "DArTcap_IWP_FST.Rdata")
readr::write_tsv(Fst.gt$pairwise$result, path = "DArTcap_IWP_FST.txt")
```

```
load("DArTcap_IWP_FST.Rdata")

kableExtra::kbl(Fst.gt$pairwise$result[order(Fst.gt$pairwise$result$Fst,
                                        decreasing = TRUE),
                                  -c(1:4)], digits = 4,
  caption = "Pairwise Fst for 3416 DArTcap loci between the IWP sites",
  longtable = TRUE, booktabs = TRUE) %>%
  kableExtra::kable_styling(full_width = FALSE,font_size = 5,
                          latex_options = c("repeat_header", "hold_position")) %>%
  kableExtra::landscape()
```

Table 46: Pairwise Fst for 3416 DArTcap loci between the IWP sites

| | Chi2 | Chi2.p.val | D | D.p.val | Fis | Fis.p.val | Fst | Fst.p.val | F’st | F’st.p.val | Gst | Gst.p.val | G’st | G’st.p.val | G”st | G”st.p.val |
|---|---|---|---|---|---|---|---|---|---|---|---|---|---|---|---|---|
| Fiji (20) v. Urauchi_River (35) | 5388.4074 | 0.0010 | 4e-04 | 0.0010 | -9.0446 | 1.0000 | 0.0760 | 0.0010 | 0.0825 | 0.0010 | 0.1261 | 0.0010 | 0.1402 | 0.0010 | 0.2364 | 0.0010 |
| Indonesia (4) v. Urauchi_River (35) | 2993.2512 | 0.0010 | 4e-04 | 0.0010 | -41.5699 | 0.5787 | 0.0637 | 0.0020 | 0.0695 | 0.0021 | 0.6333 | 0.1502 | 0.6525 | 0.1439 | 0.7872 | 0.1439 |
| Fiji (20) v. Okinawa (9) | 2794.0827 | 0.0010 | 3e-04 | 0.0010 | -16.3836 | 0.9990 | 0.0586 | 0.0010 | 0.0631 | 0.0010 | 0.2921 | 0.0010 | 0.3143 | 0.0010 | 0.4694 | 0.0010 |
| Okinawa (9) v. Urauchi_River (35) | 3217.6843 | 0.0010 | 3e-04 | 0.0010 | -14.4384 | 0.9920 | 0.0586 | 0.0010 | 0.0638 | 0.0010 | 0.2391 | 0.0010 | 0.2590 | 0.0010 | 0.4020 | 0.0010 |
| Towns_River (10) v. Urauchi_River (35) | 3526.7775 | 0.0010 | 3e-04 | 0.0010 | -12.3740 | 0.9271 | 0.0543 | 0.0010 | 0.0591 | 0.0010 | 0.2009 | 0.0010 | 0.2195 | 0.0010 | 0.3501 | 0.0010 |
| Trinity_inlet (4) v. Urauchi_River (35) | 3102.3571 | 0.0010 | 3e-04 | 0.0060 | -34.4901 | 0.0312 | 0.0535 | 0.0041 | 0.0585 | 0.0042 | 0.5876 | 0.7031 | 0.6090 | 0.6562 | 0.7537 | 0.6667 |
| Blue_mud_Bay (17) v. Urauchi_River (35) | 4357.8966 | 0.0010 | 3e-04 | 0.0010 | -8.8342 | 0.9530 | 0.0520 | 0.0010 | 0.0566 | 0.0010 | 0.1175 | 0.0010 | 0.1309 | 0.0010 | 0.2223 | 0.0010 |
| Thailand (4) v. Urauchi_River (35) | 2896.9249 | 0.0010 | 3e-04 | 0.0030 | -34.7092 | 0.0402 | 0.0510 | 0.0020 | 0.0557 | 0.0011 | 0.5847 | 0.7799 | 0.6057 | 0.7619 | 0.7512 | 0.7672 |
| Roper_River (9) v. Urauchi_River (35) | 3338.2073 | 0.0010 | 3e-04 | 0.0010 | -13.0815 | 0.6304 | 0.0496 | 0.0010 | 0.0541 | 0.0010 | 0.2235 | 0.0010 | 0.2434 | 0.0010 | 0.3816 | 0.0010 |
| Reunion (28) v. Urauchi_River (35) | 4762.2518 | 0.0010 | 3e-04 | 0.0010 | -8.1018 | 1.0000 | 0.0494 | 0.0010 | 0.0536 | 0.0010 | 0.0890 | 0.0010 | 0.0995 | 0.0010 | 0.1731 | 0.0010 |
| Papua_New_Guinea (9) v. Urauchi_River (35) | 3112.8556 | 0.0010 | 2e-04 | 0.0010 | -13.3160 | 0.8492 | 0.0490 | 0.0010 | 0.0534 | 0.0010 | 0.2208 | 0.0010 | 0.2402 | 0.0010 | 0.3776 | 0.0010 |
| Mozambique (11) v. Urauchi_River (35) | 3474.1127 | 0.0010 | 2e-04 | 0.0010 | -12.0236 | 0.9980 | 0.0490 | 0.0010 | 0.0533 | 0.0010 | 0.1941 | 0.0010 | 0.2124 | 0.0010 | 0.3404 | 0.0010 |
| Darwin_Coastal (13) v. Urauchi_River (35) | 3713.8639 | 0.0010 | 2e-04 | 0.0010 | -10.0498 | 0.3896 | 0.0467 | 0.0010 | 0.0508 | 0.0010 | 0.1448 | 0.0010 | 0.1603 | 0.0010 | 0.2666 | 0.0010 |
| South_Africa (21) v. Urauchi_River (35) | 4127.6671 | 0.0010 | 3e-04 | 0.0010 | -8.7052 | 1.0000 | 0.0463 | 0.0010 | 0.0502 | 0.0010 | 0.1027 | 0.0010 | 0.1145 | 0.0010 | 0.1970 | 0.0010 |
| Sri_Lanka (12) v. Urauchi_River (35) | 3514.0811 | 0.0010 | 2e-04 | 0.0010 | -10.8402 | 0.6384 | 0.0456 | 0.0010 | 0.0496 | 0.0010 | 0.1592 | 0.0020 | 0.1754 | 0.0010 | 0.2887 | 0.0010 |
| Adelaide_River (18) v. Urauchi_River (35) | 3899.2966 | 0.0010 | 2e-04 | 0.0010 | -9.3785 | 1.0000 | 0.0449 | 0.0010 | 0.0488 | 0.0010 | 0.1156 | 0.0010 | 0.1285 | 0.0010 | 0.2188 | 0.0010 |
| Unknown (10) v. Urauchi_River (35) | 3357.2812 | 0.0010 | 2e-04 | 0.0010 | -12.1235 | 0.5524 | 0.0442 | 0.0010 | 0.0481 | 0.0010 | 0.1929 | 0.0020 | 0.2111 | 0.0020 | 0.3387 | 0.0020 |
| Daly_River (49) v. Urauchi_River (35) | 5465.3608 | 0.0010 | 2e-04 | 0.0010 | -6.6411 | 1.0000 | 0.0440 | 0.0010 | 0.0476 | 0.0010 | 0.0631 | 0.0010 | 0.0710 | 0.0010 | 0.1261 | 0.0010 |
| Sydney_Harbour (65) v. Urauchi_River (35) | 5957.1169 | 0.0010 | 2e-04 | 0.0010 | -5.9518 | 1.0000 | 0.0433 | 0.0010 | 0.0467 | 0.0010 | 0.0552 | 0.0010 | 0.0622 | 0.0010 | 0.1112 | 0.0010 |
| Arabian_Sea (15) v. Urauchi_River (35) | 3856.4156 | 0.0010 | 2e-04 | 0.0010 | -9.3106 | 0.6993 | 0.0431 | 0.0010 | 0.0469 | 0.0010 | 0.1249 | 0.0010 | 0.1387 | 0.0010 | 0.2344 | 0.0010 |
| Indonesia (4) v. Okinawa (9) | 1478.2974 | 0.0120 | 2e-04 | 0.0639 | 43.3210 | 0.7992 | 0.0431 | 0.0291 | 0.0467 | 0.0293 | 1.7858 | 0.3486 | 1.7220 | 0.3649 | 1.2592 | 0.3670 |
| Blue_mud_Bay (17) v. Fiji (20) | 3330.6963 | 0.0010 | 2e-04 | 0.0010 | -9.5476 | 1.0000 | 0.0426 | 0.0010 | 0.0477 | 0.0010 | 0.1449 | 0.0010 | 0.1608 | 0.0010 | 0.2670 | 0.0010 |
| Clarence_River (74) v. Urauchi_River (35) | 6255.5369 | 0.0010 | 2e-04 | 0.0010 | -4.9884 | 1.0000 | 0.0418 | 0.0010 | 0.0452 | 0.0010 | 0.0482 | 0.0010 | 0.0545 | 0.0010 | 0.0980 | 0.0010 |
| Seychelles (36) v. Urauchi_River (35) | 4708.7080 | 0.0010 | 2e-04 | 0.0010 | -7.6307 | 1.0000 | 0.0417 | 0.0010 | 0.0452 | 0.0010 | 0.0742 | 0.0010 | 0.0832 | 0.0010 | 0.1465 | 0.0010 |
| East_Alligator_River (47) v. Urauchi_River (35) | 5161.3271 | 0.0010 | 2e-04 | 0.0010 | -6.9864 | 1.0000 | 0.0416 | 0.0010 | 0.0450 | 0.0010 | 0.0644 | 0.0010 | 0.0723 | 0.0010 | 0.1284 | 0.0010 |
| Urauchi_River (35) v. Wenlock_River (24) | 4244.2007 | 0.0010 | 2e-04 | 0.0010 | -8.1753 | 1.0000 | 0.0410 | 0.0010 | 0.0443 | 0.0010 | 0.0902 | 0.0010 | 0.1008 | 0.0010 | 0.1753 | 0.0010 |
| South_Alligator_River (88) v. Urauchi_River (35) | 6536.0386 | 0.0010 | 2e-04 | 0.0010 | -4.9996 | 1.0000 | 0.0408 | 0.0010 | 0.0439 | 0.0010 | 0.0463 | 0.0010 | 0.0522 | 0.0010 | 0.0942 | 0.0010 |
| Okinawa (9) v. Towns_River (10) | 1928.3144 | 0.0010 | 2e-04 | 0.0020 | -28.1073 | 1.0000 | 0.0407 | 0.0010 | 0.0443 | 0.0010 | 0.4626 | 0.0010 | 0.4853 | 0.0010 | 0.6481 | 0.0010 |
| Okinawa (9) v. Trinity_inlet (4) | 1562.9446 | 0.0130 | 2e-04 | 0.0470 | 54.7396 | 0.5153 | 0.0394 | 0.0291 | 0.0424 | 0.0291 | 1.5314 | 0.7249 | 1.4886 | 0.7372 | 1.1930 | 0.7382 |
| Fiji (20) v. Trinity_inlet (4) | 2323.4592 | 0.0010 | 3e-04 | 0.0050 | -52.4566 | 0.0322 | 0.0384 | 0.0073 | 0.0412 | 0.0073 | 0.7000 | 0.9236 | 0.7174 | 0.9236 | 0.8338 | 0.9236 |
| Okinawa (9) v. Thailand (4) | 1509.6999 | 0.0180 | 2e-04 | 0.0639 | 53.8887 | 0.4297 | 0.0381 | 0.0330 | 0.0408 | 0.0330 | 1.5562 | 0.7745 | 1.5120 | 0.7867 | 1.2003 | 0.7887 |
| Fiji (20) v. Towns_River (10) | 2620.1691 | 0.0010 | 2e-04 | 0.0010 | -13.9361 | 0.9830 | 0.0374 | 0.0010 | 0.0406 | 0.0010 | 0.2406 | 0.0010 | 0.2612 | 0.0010 | 0.4045 | 0.0010 |
| Fiji (20) v. Indonesia (4) | 2161.3683 | 0.0010 | 2e-04 | 0.0080 | -71.6720 | 0.2780 | 0.0374 | 0.0042 | 0.0401 | 0.0052 | 0.7598 | 0.6916 | 0.7737 | 0.6857 | 0.8714 | 0.6857 |
| Fitzroy_River (3) v. Okinawa (9) | 1350.3746 | 0.0440 | 2e-04 | 0.0879 | NA | NA | 0.0367 | 0.0832 | NA | NA | NA | NA | NA | NA | NA | NA |
| Fiji (20) v. Papua_New_Guinea (9) | 2482.7329 | 0.0010 | 2e-04 | 0.0010 | -14.8453 | 0.9580 | 0.0360 | 0.0010 | 0.0387 | 0.0010 | 0.2628 | 0.0020 | 0.2841 | 0.0020 | 0.4331 | 0.0020 |
| Fiji (20) v. Mozambique (11) | 2692.3627 | 0.0010 | 2e-04 | 0.0010 | -13.5223 | 0.9980 | 0.0357 | 0.0010 | 0.0399 | 0.0010 | 0.2359 | 0.0010 | 0.2565 | 0.0010 | 0.3984 | 0.0010 |
| Okinawa (9) v. Papua_New_Guinea (9) | 1733.6778 | 0.0050 | 2e-04 | 0.0110 | -33.1632 | 0.9940 | 0.0352 | 0.0090 | 0.0380 | 0.0090 | 0.5126 | 0.0090 | 0.5344 | 0.0090 | 0.6922 | 0.0090 |
| Fiji (20) v. Wenlock_River (24) | 3382.0143 | 0.0010 | 2e-04 | 0.0010 | -8.7077 | 1.0000 | 0.0352 | 0.0010 | 0.0383 | 0.0010 | 0.1156 | 0.0010 | 0.1287 | 0.0010 | 0.2190 | 0.0010 |
| Fiji (20) v. Reunion (28) | 3540.6320 | 0.0010 | 2e-04 | 0.0010 | -8.6755 | 1.0000 | 0.0350 | 0.0010 | 0.0385 | 0.0010 | 0.1099 | 0.0010 | 0.1224 | 0.0010 | 0.2093 | 0.0010 |
| Fiji (20) v. Sri_Lanka (12) | 2768.2168 | 0.0010 | 2e-04 | 0.0010 | -11.6935 | 0.9970 | 0.0340 | 0.0010 | 0.0367 | 0.0010 | 0.1909 | 0.0010 | 0.2092 | 0.0010 | 0.3360 | 0.0010 |
| Blue_mud_Bay (17) v. Okinawa (9) | 2200.2988 | 0.0010 | 2e-04 | 0.0010 | -15.2999 | 1.0000 | 0.0339 | 0.0010 | 0.0365 | 0.0010 | 0.2746 | 0.0010 | 0.2964 | 0.0010 | 0.4480 | 0.0010 |
| Mozambique (11) v. Okinawa (9) | 1914.0960 | 0.0020 | 2e-04 | 0.0020 | -26.0982 | 0.9960 | 0.0336 | 0.0020 | 0.0363 | 0.0020 | 0.4425 | 0.0030 | 0.4655 | 0.0030 | 0.6294 | 0.0030 |
| Fiji (20) v. South_Alligator_River (88) | 5324.9691 | 0.0010 | 2e-04 | 0.0010 | -4.9823 | 0.9980 | 0.0336 | 0.0010 | 0.0366 | 0.0010 | 0.0572 | 0.0010 | 0.0644 | 0.0010 | 0.1150 | 0.0010 |
| Adelaide_River (18) v. Fiji (20) | 3045.6429 | 0.0010 | 2e-04 | 0.0010 | -9.9690 | 1.0000 | 0.0333 | 0.0010 | 0.0372 | 0.0010 | 0.1412 | 0.0010 | 0.1562 | 0.0010 | 0.2606 | 0.0010 |
| Fiji (20) v. Unknown (10) | 2556.4398 | 0.0010 | 2e-04 | 0.0010 | -13.5310 | 0.9291 | 0.0329 | 0.0010 | 0.0357 | 0.0010 | 0.2335 | 0.0010 | 0.2539 | 0.0010 | 0.3952 | 0.0010 |
| Daly_River (49) v. Fiji (20) | 4192.4120 | 0.0010 | 2e-04 | 0.0010 | -6.7568 | 1.0000 | 0.0328 | 0.0010 | 0.0359 | 0.0010 | 0.0775 | 0.0010 | 0.0869 | 0.0010 | 0.1526 | 0.0010 |
| Fiji (20) v. South_Africa (21) | 3137.9922 | 0.0010 | 2e-04 | 0.0010 | -9.3698 | 1.0000 | 0.0328 | 0.0010 | 0.0355 | 0.0010 | 0.1267 | 0.0010 | 0.1407 | 0.0010 | 0.2373 | 0.0010 |
| Fiji (20) v. Sydney_Harbour (65) | 4716.2588 | 0.0010 | 1e-04 | 0.0010 | -5.9569 | 1.0000 | 0.0327 | 0.0010 | 0.0358 | 0.0010 | 0.0672 | 0.0010 | 0.0755 | 0.0010 | 0.1337 | 0.0010 |
| Fiji (20) v. Seychelles (36) | 3775.7483 | 0.0010 | 2e-04 | 0.0010 | -7.9274 | 1.0000 | 0.0327 | 0.0010 | 0.0357 | 0.0010 | 0.0939 | 0.0010 | 0.1050 | 0.0010 | 0.1818 | 0.0010 |
| Darwin_Coastal (13) v. Fiji (20) | 2818.4246 | 0.0010 | 2e-04 | 0.0010 | -10.9110 | 0.9920 | 0.0324 | 0.0010 | 0.0365 | 0.0010 | 0.1742 | 0.0010 | 0.1918 | 0.0010 | 0.3117 | 0.0010 |
| East_Alligator_River (47) v. Fiji (20) | 4146.6801 | 0.0010 | 2e-04 | 0.0010 | -7.1477 | 1.0000 | 0.0317 | 0.0010 | 0.0347 | 0.0010 | 0.0806 | 0.0010 | 0.0902 | 0.0010 | 0.1581 | 0.0010 |
| Okinawa (9) v. Wenlock_River (24) | 2354.3461 | 0.0010 | 2e-04 | 0.0010 | -13.1658 | 1.0000 | 0.0311 | 0.0010 | 0.0340 | 0.0010 | 0.2274 | 0.0010 | 0.2470 | 0.0010 | 0.3865 | 0.0010 |
| Clarence_River (74) v. Fiji (20) | 4865.4235 | 0.0010 | 2e-04 | 0.0010 | -4.8476 | 1.0000 | 0.0303 | 0.0010 | 0.0331 | 0.0010 | 0.0560 | 0.0010 | 0.0633 | 0.0010 | 0.1130 | 0.0010 |

Table 46: Pairwise Fst for 3416 DArTcap loci between the IWP sites *(continued)*

| | Chi2 | Chi2.p.val | D | D.p.val | Fis | Fis.p.val | Fst | Fst.p.val | F'st | F'st.p.val | Gst | Gst.p.val | G'st | G'st.p.val | G"st | G"st.p.val |
|---|---|---|---|---|---|---|---|---|---|---|---|---|---|---|---|---|
| Okinawa (9) v. Reunion (28) | 2426.4054 | 0.0010 | 2e-04 | 0.0010 | -13.1817 | 0.9990 | 0.0301 | 0.0010 | 0.0333 | 0.0010 | 0.2207 | 0.0010 | 0.2398 | 0.0010 | 0.3772 | 0.0010 |
| Okinawa (9) v. Roper_River (9) | 1739.1946 | 0.0050 | 2e-04 | 0.0110 | -32.2964 | 0.9940 | 0.0301 | 0.0090 | 0.0337 | 0.0070 | 0.5102 | 0.0060 | 0.5324 | 0.0070 | 0.6904 | 0.0070 |
| Fiji (20) v. Roper_River (9) | 2421.4187 | 0.0010 | 2e-04 | 0.0010 | -14.8553 | 0.7992 | 0.0296 | 0.0010 | 0.0326 | 0.0010 | 0.2658 | 0.0100 | 0.2875 | 0.0080 | 0.4371 | 0.0090 |
| Okinawa (9) v. Unknown (10) | 1764.3354 | 0.0010 | 2e-04 | 0.0040 | -27.2677 | 0.9970 | 0.0290 | 0.0040 | 0.0316 | 0.0040 | 0.4507 | 0.0040 | 0.4735 | 0.0040 | 0.6371 | 0.0040 |
| Adelaide_River (18) v. Okinawa (9) | 2070.0534 | 0.0010 | 1e-04 | 0.0010 | -16.4720 | 0.9990 | 0.0290 | 0.0010 | 0.0312 | 0.0010 | 0.2821 | 0.0010 | 0.3034 | 0.0010 | 0.4567 | 0.0010 |
| Clarence_River (74) v. Okinawa (9) | 3208.9096 | 0.0010 | 2e-04 | 0.0010 | -6.6401 | 1.0000 | 0.0280 | 0.0010 | 0.0301 | 0.0010 | 0.1141 | 0.0010 | 0.1268 | 0.0010 | 0.2162 | 0.0010 |
| Arabian_Sea (15) v. Fiji (20) | 2920.1431 | 0.0010 | 2e-04 | 0.0010 | -9.7938 | 1.0000 | 0.0280 | 0.0010 | 0.0314 | 0.0010 | 0.1479 | 0.0010 | 0.1636 | 0.0010 | 0.2713 | 0.0010 |
| Arabian_Sea (15) v. Okinawa (9) | 1982.5267 | 0.0010 | 2e-04 | 0.0010 | -16.6438 | 1.0000 | 0.0278 | 0.0010 | 0.0300 | 0.0010 | 0.2970 | 0.0010 | 0.3191 | 0.0010 | 0.4750 | 0.0010 |
| Okinawa (9) v. Sri_Lanka (12) | 1865.0523 | 0.0020 | 2e-04 | 0.0050 | -21.3791 | 0.9990 | 0.0278 | 0.0040 | 0.0300 | 0.0040 | 0.3704 | 0.0030 | 0.3933 | 0.0030 | 0.5573 | 0.0030 |
| Okinawa (9) v. South_Alligator_River (88) | 3295.2727 | 0.0010 | 2e-04 | 0.0010 | -6.8669 | 1.0000 | 0.0272 | 0.0010 | 0.0297 | 0.0010 | 0.1149 | 0.0010 | 0.1274 | 0.0010 | 0.2173 | 0.0010 |
| East_Alligator_River (47) v. Okinawa (9) | 2729.5616 | 0.0010 | 1e-04 | 0.0010 | -10.4814 | 1.0000 | 0.0271 | 0.0010 | 0.0290 | 0.0010 | 0.1693 | 0.0010 | 0.1855 | 0.0010 | 0.3034 | 0.0010 |
| Daly_River (49) v. Okinawa (9) | 2787.1429 | 0.0010 | 1e-04 | 0.0010 | -9.7344 | 1.0000 | 0.0263 | 0.0010 | 0.0283 | 0.0010 | 0.1591 | 0.0010 | 0.1749 | 0.0010 | 0.2881 | 0.0010 |
| Okinawa (9) v. Sydney_Harbour (65) | 2961.6580 | 0.0010 | 2e-04 | 0.0010 | -8.4520 | 1.0000 | 0.0261 | 0.0010 | 0.0287 | 0.0010 | 0.1383 | 0.0010 | 0.1526 | 0.0010 | 0.2555 | 0.0010 |
| Okinawa (9) v. Seychelles (36) | 2430.2545 | 0.0010 | 2e-04 | 0.0010 | -11.9599 | 1.0000 | 0.0257 | 0.0010 | 0.0281 | 0.0010 | 0.1942 | 0.0010 | 0.2119 | 0.0010 | 0.3400 | 0.0010 |
| Darwin_Coastal (13) v. Okinawa (9) | 1888.3229 | 0.0020 | 2e-04 | 0.0040 | -18.9395 | 1.0000 | 0.0255 | 0.0030 | 0.0276 | 0.0030 | 0.3355 | 0.0010 | 0.3584 | 0.0010 | 0.5196 | 0.0010 |
| Fiji (20) v. Thailand (4) | 2116.3784 | 0.0010 | 2e-04 | 0.0130 | -52.7453 | 0.0182 | 0.0243 | 0.0187 | 0.0261 | 0.0187 | 0.6943 | 0.9720 | 0.7114 | 0.9708 | 0.8297 | 0.9708 |
| Fitzroy_River (3) v. Indonesia (4) | 1013.2851 | 0.2817 | 2e-04 | 0.3157 | NA | NA | 0.0242 | 0.2817 | 0.0262 | 0.3047 | NA | NA | NA | NA | NA | NA |
| Okinawa (9) v. South_Africa (21) | 2060.9975 | 0.0010 | 1e-04 | 0.0010 | -14.8221 | 1.0000 | 0.0240 | 0.0010 | 0.0261 | 0.0010 | 0.2509 | 0.0010 | 0.2712 | 0.0010 | 0.4173 | 0.0010 |
| Fiji (20) v. Fitzroy_River (3) | 1990.0145 | 0.0060 | 2e-04 | 0.0160 | NA | NA | 0.0235 | 0.0134 | 0.0253 | 0.0134 | NA | NA | NA | NA | NA | NA |
| Indonesia (4) v. Towns_River (10) | 1433.2349 | 0.0549 | 2e-04 | 0.1019 | 78.7206 | 0.6112 | 0.0219 | 0.0680 | 0.0241 | 0.0680 | 1.3369 | 0.4028 | 1.3120 | 0.4028 | 1.1335 | 0.4028 |
| Papua_New_Guinea (9) v. Trinity_inlet (4) | 1435.1476 | 0.0020 | 1e-04 | 0.0509 | 81.0946 | 0.2884 | 0.0189 | 0.0040 | 0.0204 | 0.0040 | 1.3103 | 0.8281 | 1.2867 | 0.8342 | 1.1241 | 0.8342 |
| Blue_mud_Bay (17) v. Towns_River (10) | 1991.2789 | 0.0120 | 1e-04 | 0.0110 | -13.2927 | 0.9990 | 0.0151 | 0.0090 | 0.0165 | 0.0090 | 0.2290 | 0.0020 | 0.2492 | 0.0020 | 0.3891 | 0.0020 |
| Blue_mud_Bay (17) v. Indonesia (4) | 1621.5721 | 0.2178 | 1e-04 | 0.0899 | -53.8364 | 0.9329 | 0.0149 | 0.0661 | 0.0161 | 0.0661 | 0.7026 | 0.0855 | 0.7192 | 0.0865 | 0.8351 | 0.0865 |
| Indonesia (4) v. Papua_New_Guinea (9) | 1301.8746 | 0.1199 | 2e-04 | 0.1149 | 56.8305 | 0.8128 | 0.0146 | 0.1269 | 0.0159 | 0.1269 | 1.5187 | 0.2212 | 1.4790 | 0.2232 | 1.1902 | 0.2232 |
| Adelaide_River (18) v. Indonesia (4) | 1593.1398 | 0.0619 | 1e-04 | 0.0300 | -73.8098 | 0.8877 | 0.0146 | 0.0300 | 0.0157 | 0.0300 | 0.7615 | 0.0772 | 0.7750 | 0.0752 | 0.8723 | 0.0752 |
| Indonesia (4) v. Thailand (4) | 1073.1255 | 0.3027 | 2e-04 | 0.3347 | 14.2870 | 0.6334 | 0.0136 | 0.3926 | 0.0147 | 0.3926 | 21.5582 | 0.3387 | 19.1145 | 0.3387 | 1.8030 | 0.3786 |
| Mozambique (11) v. Papua_New_Guinea (9) | 1581.5293 | 0.0040 | 1e-04 | 0.1429 | -23.7994 | 0.9151 | 0.0135 | 0.0010 | 0.0146 | 0.0010 | 0.4123 | 0.0509 | 0.4355 | 0.0480 | 0.6003 | 0.0470 |
| Blue_mud_Bay (17) v. Reunion (28) | 2620.0231 | 0.0010 | 1e-04 | 0.0010 | -8.5001 | 0.5764 | 0.0132 | 0.0010 | 0.0146 | 0.0010 | 0.1029 | 0.0010 | 0.1149 | 0.0010 | 0.1975 | 0.0010 |
| Reunion (28) v. Towns_River (10) | 2203.4878 | 0.0010 | 1e-04 | 0.0010 | -11.8453 | 0.9011 | 0.0129 | 0.0010 | 0.0139 | 0.0010 | 0.1845 | 0.0070 | 0.2019 | 0.0060 | 0.3262 | 0.0060 |
| Reunion (28) v. Trinity_inlet (4) | 2044.6432 | 0.0040 | 1e-04 | 0.0390 | -29.1164 | 0.0226 | 0.0121 | 0.0462 | 0.0130 | 0.0462 | 0.5424 | 0.9126 | 0.5647 | 0.9074 | 0.7178 | 0.9074 |
| Indonesia (4) v. Seychelles (36) | 2027.1014 | 0.0180 | 1e-04 | 0.0559 | -26.0019 | 0.7127 | 0.0119 | 0.0250 | 0.0131 | 0.0252 | 0.5045 | 0.2550 | 0.5265 | 0.2540 | 0.6853 | 0.2540 |
| Indonesia (4) v. South_Alligator_River (88) | 2787.3675 | 0.0190 | 1e-04 | 0.0340 | -10.2014 | 0.7748 | 0.0119 | 0.0142 | 0.0130 | 0.0143 | 0.2747 | 0.0931 | 0.2957 | 0.0839 | 0.4474 | 0.0870 |
| Indonesia (4) v. Trinity_inlet (4) | 1088.9811 | 0.3497 | 2e-04 | 0.3457 | 14.3096 | 0.6786 | 0.0112 | 0.4336 | 0.0122 | 0.4116 | 17.6351 | 0.2784 | 15.6467 | 0.2784 | 1.7860 | 0.2559 |
| Blue_mud_Bay (17) v. Trinity_inlet (4) | 1739.6500 | 0.0659 | 1e-04 | 0.1359 | -41.9806 | 0.4728 | 0.0112 | 0.0782 | 0.0121 | 0.0782 | 0.6485 | 0.4441 | 0.6681 | 0.4338 | 0.7987 | 0.4379 |
| Indonesia (4) v. Mozambique (11) | 1359.9089 | 0.2288 | 2e-04 | 0.1798 | 94.6150 | 0.1131 | 0.0110 | 0.1058 | 0.0128 | 0.0996 | 1.2671 | 0.8907 | 1.2477 | 0.8907 | 1.1093 | 0.8907 |
| Towns_River (10) v. Trinity_inlet (4) | 1434.7344 | 0.0290 | 1e-04 | 0.2717 | 123.4753 | 0.1840 | 0.0109 | 0.1280 | 0.0118 | 0.1280 | 1.1923 | 0.8643 | 1.1783 | 0.8684 | 1.0813 | 0.8695 |
| Indonesia (4) v. Unknown (10) | 1361.0802 | 0.0679 | 1e-04 | 0.1339 | 94.2569 | 0.6197 | 0.0108 | 0.0759 | 0.0119 | 0.0760 | 1.2697 | 0.3823 | 1.2502 | 0.3793 | 1.1102 | 0.3793 |
| Indonesia (4) v. Sydney_Harbour (65) | 2470.6889 | 0.0050 | 1e-04 | 0.0160 | -13.9163 | 0.7865 | 0.0107 | 0.0070 | 0.0118 | 0.0070 | 0.3433 | 0.1813 | 0.3659 | 0.1843 | 0.5280 | 0.1823 |
| Blue_mud_Bay (17) v. South_Africa (21) | 2344.0332 | 0.0010 | 1e-04 | 0.0060 | -9.0482 | 0.9880 | 0.0107 | 0.0010 | 0.0117 | 0.0010 | 0.1183 | 0.0010 | 0.1317 | 0.0010 | 0.2235 | 0.0010 |
| Daly_River (49) v. Indonesia (4) | 2246.8295 | 0.0110 | 1e-04 | 0.0210 | -17.8710 | 0.8645 | 0.0107 | 0.0240 | 0.0114 | 0.0240 | 0.4048 | 0.0954 | 0.4277 | 0.0894 | 0.5926 | 0.0934 |
| Papua_New_Guinea (9) v. Towns_River (10) | 1505.8548 | 0.0350 | 1e-04 | 0.1039 | -25.4511 | 0.9730 | 0.0106 | 0.0190 | 0.0116 | 0.0190 | 0.4267 | 0.0270 | 0.4495 | 0.0270 | 0.6142 | 0.0270 |
| Blue_mud_Bay (17) v. Seychelles (36) | 2642.0241 | 0.0010 | 1e-04 | 0.0010 | -7.9749 | 0.1808 | 0.0105 | 0.0010 | 0.0114 | 0.0010 | 0.0885 | 0.0020 | 0.0991 | 0.0020 | 0.1723 | 0.0020 |
| Indonesia (4) v. Wenlock_River (24) | 1748.9899 | 0.2168 | 1e-04 | 0.1329 | -35.4065 | 0.9284 | 0.0103 | 0.0300 | 0.0113 | 0.0302 | 0.5933 | 0.0736 | 0.6135 | 0.0736 | 0.7574 | 0.0736 |
| Blue_mud_Bay (17) v. Mozambique (11) | 1905.0446 | 0.0629 | 1e-04 | 0.0270 | -12.8792 | 1.0000 | 0.0101 | 0.0320 | 0.0114 | 0.0320 | 0.2216 | 0.0010 | 0.2416 | 0.0010 | 0.3792 | 0.0010 |
| Roper_River (9) v. Towns_River (10) | 1592.7288 | 0.0020 | 1e-04 | 0.0090 | -24.7724 | 0.9371 | 0.0101 | 0.0190 | 0.0111 | 0.0220 | 0.4263 | 0.0270 | 0.4496 | 0.0250 | 0.6141 | 0.0250 |
| Papua_New_Guinea (9) v. Unknown (10) | 1495.8544 | 0.0160 | 1e-04 | 0.0100 | -24.3306 | 0.9920 | 0.0101 | 0.0050 | 0.0110 | 0.0050 | 0.4159 | 0.0070 | 0.4390 | 0.0070 | 0.6038 | 0.0070 |
| Arabian_Sea (15) v. Blue_mud_Bay (17) | 2173.1892 | 0.0080 | 1e-04 | 0.0010 | -9.6558 | 0.9930 | 0.0100 | 0.0100 | 0.0109 | 0.0100 | 0.1434 | 0.0090 | 0.1590 | 0.0090 | 0.2644 | 0.0090 |
| East_Alligator_River (47) v. Indonesia (4) | 2259.8999 | 0.0060 | 1e-04 | 0.0589 | -20.2203 | 0.7319 | 0.0100 | 0.0231 | 0.0107 | 0.0231 | 0.4354 | 0.1472 | 0.4579 | 0.1381 | 0.6223 | 0.1421 |
| Blue_mud_Bay (17) v. East_Alligator_River (47) | 2861.5864 | 0.0010 | 1e-04 | 0.0010 | -7.3078 | 0.0100 | 0.0099 | 0.0010 | 0.0108 | 0.0010 | 0.0762 | 0.0010 | 0.0854 | 0.0010 | 0.1502 | 0.0010 |
| Blue_mud_Bay (17) v. Roper_River (9) | 1851.5513 | 0.0529 | 1e-04 | 0.0130 | -14.0082 | 0.9910 | 0.0096 | 0.0300 | 0.0107 | 0.0290 | 0.2526 | 0.0100 | 0.2741 | 0.0100 | 0.4205 | 0.0090 |
| Adelaide_River (18) v. Trinity_inlet (4) | 1751.2904 | 0.0040 | 1e-04 | 0.2178 | -51.7056 | 0.1121 | 0.0096 | 0.0440 | 0.0103 | 0.0440 | 0.6912 | 0.8218 | 0.7085 | 0.8178 | 0.8276 | 0.8178 |
| Indonesia (4) v. Reunion (28) | 1850.3730 | 0.0500 | 1e-04 | 0.1768 | -34.5664 | 0.5329 | 0.0095 | 0.0675 | 0.0105 | 0.0688 | 0.5819 | 0.4579 | 0.6022 | 0.4559 | 0.7485 | 0.4579 |
| Blue_mud_Bay (17) v. South_Alligator_River (88) | 3600.8806 | 0.0010 | 1e-04 | 0.0010 | -5.0876 | 0.0070 | 0.0094 | 0.0010 | 0.0102 | 0.0010 | 0.0493 | 0.0010 | 0.0557 | 0.0010 | 0.1001 | 0.0010 |

Table 46: Pairwise Fst for 3416 DArTcap loci between the IWP sites *(continued)*

| | Chi2 | Chi2.p.val | D | D.p.val | Fis | Fis.p.val | Fst | Fst.p.val | F'st | F'st.p.val | Gst | Gst.p.val | G'st | G'st.p.val | G"st | G"st.p.val |
|---|---|---|---|---|---|---|---|---|---|---|---|---|---|---|---|---|
| Adelaide_River (18) v. Towns_River (10) | 1812.9247 | 0.0020 | 1e-04 | 0.0010 | -14.4419 | 0.9780 | 0.0094 | 0.0030 | 0.0102 | 0.0030 | 0.2364 | 0.0020 | 0.2562 | 0.0020 | 0.3984 | 0.0020 |
| Blue_mud_Bay (17) v. Clarence_River (74) | 3350.6386 | 0.0010 | 1e-04 | 0.0010 | -4.8805 | 0.4286 | 0.0093 | 0.0010 | 0.0102 | 0.0010 | 0.0491 | 0.0010 | 0.0556 | 0.0010 | 0.0998 | 0.0010 |
| Blue_mud_Bay (17) v. Fitzroy_River (3) | 1504.2709 | 0.5175 | 2e-04 | 0.1578 | NA | NA | 0.0093 | 0.1775 | 0.0100 | 0.1775 | NA | NA | NA | NA | NA | NA |
| Blue_mud_Bay (17) v. Darwin_Coastal (13) | 2023.6360 | 0.0210 | 1e-04 | 0.0030 | -10.6158 | 0.9620 | 0.0092 | 0.0170 | 0.0100 | 0.0160 | 0.1655 | 0.0190 | 0.1827 | 0.0190 | 0.2987 | 0.0190 |
| Indonesia (4) v. Roper_River (9) | 1302.7152 | 0.1858 | 2e-04 | 0.2138 | 57.8007 | 0.4702 | 0.0091 | 0.1888 | 0.0104 | 0.1798 | 1.4946 | 0.5348 | 1.4562 | 0.5318 | 1.1829 | 0.5318 |
| Clarence_River (74) v. Indonesia (4) | 2487.5808 | 0.1479 | 1e-04 | 0.0180 | -9.9007 | 0.9869 | 0.0090 | 0.0291 | 0.0097 | 0.0291 | 0.2703 | 0.0172 | 0.2916 | 0.0182 | 0.4423 | 0.0172 |
| Arabian_Sea (15) v. Indonesia (4) | 1560.3991 | 0.3427 | 1e-04 | 0.1019 | -71.6146 | 0.9710 | 0.0090 | 0.0689 | 0.0097 | 0.0689 | 0.7619 | 0.0270 | 0.7756 | 0.0270 | 0.8726 | 0.0270 |
| Blue_mud_Bay (17) v. Daly_River (49) | 2911.0649 | 0.0010 | 1e-04 | 0.0010 | -6.8504 | 0.1069 | 0.0090 | 0.0010 | 0.0098 | 0.0010 | 0.0711 | 0.0010 | 0.0799 | 0.0010 | 0.1410 | 0.0010 |
| Indonesia (4) v. Sri_Lanka (12) | 1402.8136 | 0.2088 | 1e-04 | 0.0749 | 1084.9211 | 0.1946 | 0.0089 | 0.0649 | 0.0097 | 0.0651 | 1.0200 | 0.0802 | 1.0187 | 0.0802 | 1.0092 | 0.0802 |
| Blue_mud_Bay (17) v. Sydney_Harbour (65) | 3254.5310 | 0.0010 | 1e-04 | 0.0010 | -5.9744 | 0.0370 | 0.0089 | 0.0010 | 0.0097 | 0.0010 | 0.0596 | 0.0010 | 0.0671 | 0.0010 | 0.1196 | 0.0010 |
| Mozambique (11) v. Wenlock_River (24) | 2038.9295 | 0.0010 | 1e-04 | 0.0020 | -11.4864 | 1.0000 | 0.0088 | 0.0010 | 0.0096 | 0.0010 | 0.1847 | 0.0010 | 0.2024 | 0.0010 | 0.3267 | 0.0010 |
| Blue_mud_Bay (17) v. Wenlock_River (24) | 2318.9034 | 0.0020 | 1e-04 | 0.0030 | -8.5499 | 0.9041 | 0.0088 | 0.0020 | 0.0096 | 0.0020 | 0.1064 | 0.0030 | 0.1187 | 0.0030 | 0.2035 | 0.0030 |
| Blue_mud_Bay (17) v. Papua_New_Guinea (9) | 1764.8423 | 0.1229 | 1e-04 | 0.0290 | -14.2905 | 0.9980 | 0.0088 | 0.0529 | 0.0095 | 0.0529 | 0.2500 | 0.0110 | 0.2709 | 0.0130 | 0.4167 | 0.0130 |
| South_Alligator_River (88) v. Towns_River (10) | 2897.7702 | 0.0010 | 1e-04 | 0.0020 | -6.3324 | 0.6973 | 0.0086 | 0.0020 | 0.0092 | 0.0020 | 0.0906 | 0.0030 | 0.1010 | 0.0030 | 0.1758 | 0.0030 |
| Daly_River (49) v. Towns_River (10) | 2404.8295 | 0.0010 | 1e-04 | 0.0060 | -8.9135 | 0.9510 | 0.0086 | 0.0010 | 0.0092 | 0.0010 | 0.1301 | 0.0010 | 0.1439 | 0.0010 | 0.2424 | 0.0010 |
| South_Alligator_River (88) v. Trinity_inlet (4) | 2956.3054 | 0.0100 | 1e-04 | 0.1369 | -9.5168 | 0.0429 | 0.0085 | 0.0182 | 0.0091 | 0.0182 | 0.2619 | 0.7357 | 0.2834 | 0.6765 | 0.4321 | 0.6980 |
| Mozambique (11) v. Towns_River (10) | 1597.6281 | 0.0230 | 1e-04 | 0.0370 | -21.6602 | 0.9560 | 0.0083 | 0.0240 | 0.0091 | 0.0250 | 0.3762 | 0.0360 | 0.3993 | 0.0360 | 0.5635 | 0.0360 |
| Towns_River (10) v. Wenlock_River (24) | 2004.3581 | 0.0040 | 1e-04 | 0.1269 | -11.8097 | 0.9920 | 0.0082 | 0.0060 | 0.0089 | 0.0060 | 0.1877 | 0.0030 | 0.2054 | 0.0030 | 0.3310 | 0.0030 |
| South_Africa (21) v. Trinity_inlet (4) | 1798.3932 | 0.0050 | 1e-04 | 0.1029 | -38.5483 | 0.0937 | 0.0081 | 0.0391 | 0.0087 | 0.0391 | 0.6192 | 0.8056 | 0.6393 | 0.7946 | 0.7772 | 0.7946 |
| Arabian_Sea (15) v. Towns_River (10) | 1835.2962 | 0.0070 | 1e-04 | 0.0380 | -14.0075 | 1.0000 | 0.0080 | 0.0040 | 0.0088 | 0.0040 | 0.2433 | 0.0010 | 0.2638 | 0.0010 | 0.4079 | 0.0010 |
| Adelaide_River (18) v. Blue_mud_Bay (17) | 2114.9291 | 0.0090 | 1e-04 | 0.0110 | -9.8243 | 0.9780 | 0.0077 | 0.0160 | 0.0084 | 0.0160 | 0.1332 | 0.0160 | 0.1476 | 0.0160 | 0.2478 | 0.0160 |
| South_Africa (21) v. Towns_River (10) | 1892.1145 | 0.0010 | 1e-04 | 0.0050 | -12.9725 | 0.9950 | 0.0076 | 0.0020 | 0.0082 | 0.0020 | 0.2085 | 0.0010 | 0.2272 | 0.0010 | 0.3605 | 0.0010 |
| Sydney_Harbour (65) v. Trinity_inlet (4) | 2594.0254 | 0.0010 | 1e-04 | 0.0559 | -12.7534 | 0.0130 | 0.0075 | 0.0330 | 0.0080 | 0.0330 | 0.3228 | 0.8967 | 0.3460 | 0.8746 | 0.5056 | 0.8816 |
| Fitzroy_River (3) v. Thailand (4) | 989.0411 | 0.1029 | 2e-04 | 0.1109 | NA | NA | 0.0074 | 0.1219 | 0.0080 | 0.1219 | NA | NA | NA | NA | NA | NA |
| Fitzroy_River (3) v. Papua_New_Guinea (9) | 1197.1304 | 0.2627 | 1e-04 | 0.3566 | NA | NA | 0.0074 | 0.1320 | NA | NA | NA | NA | NA | NA | NA | NA |
| Clarence_River (74) v. Towns_River (10) | 2623.8601 | 0.0220 | 1e-04 | 0.0020 | -6.2361 | 1.0000 | 0.0074 | 0.0030 | 0.0080 | 0.0030 | 0.0906 | 0.0010 | 0.1012 | 0.0010 | 0.1759 | 0.0010 |
| Papua_New_Guinea (9) v. Reunion (28) | 1927.2220 | 0.0240 | 1e-04 | 0.0200 | -12.5260 | 0.7762 | 0.0073 | 0.0210 | 0.0080 | 0.0190 | 0.2013 | 0.1499 | 0.2194 | 0.1419 | 0.3502 | 0.1429 |
| Sri_Lanka (12) v. Trinity_inlet (4) | 1503.1102 | 0.0330 | 1e-04 | 0.0569 | -227.3737 | 0.4076 | 0.0072 | 0.0589 | 0.0078 | 0.0589 | 0.9149 | 0.5793 | 0.9205 | 0.5783 | 0.9585 | 0.5783 |
| Darwin_Coastal (13) v. Reunion (28) | 2217.5237 | 0.0010 | 1e-04 | 0.0160 | -9.6755 | 0.0689 | 0.0072 | 0.0040 | 0.0080 | 0.0040 | 0.1301 | 0.0779 | 0.1443 | 0.0579 | 0.2428 | 0.0619 |
| East_Alligator_River (47) v. Towns_River (10) | 2304.7931 | 0.0010 | 1e-04 | 0.0230 | -9.6023 | 0.5854 | 0.0071 | 0.0020 | 0.0077 | 0.0020 | 0.1381 | 0.0020 | 0.1523 | 0.0020 | 0.2551 | 0.0020 |
| Blue_mud_Bay (17) v. Unknown (10) | 1823.4100 | 0.0709 | 1e-04 | 0.0180 | -13.0600 | 0.9890 | 0.0071 | 0.0599 | 0.0077 | 0.0599 | 0.2220 | 0.0200 | 0.2420 | 0.0200 | 0.3797 | 0.0200 |
| Indonesia (4) v. South_Africa (21) | 1655.1729 | 0.1439 | 1e-04 | 0.0789 | -47.8486 | 0.9358 | 0.0070 | 0.0671 | 0.0076 | 0.0672 | 0.6669 | 0.0602 | 0.6844 | 0.0602 | 0.8107 | 0.0602 |
| Reunion (28) v. Roper_River (9) | 2017.2914 | 0.0090 | 1e-04 | 0.0040 | -12.2881 | 0.5055 | 0.0069 | 0.0210 | 0.0076 | 0.0210 | 0.2037 | 0.0779 | 0.2224 | 0.0739 | 0.3540 | 0.0759 |
| Reunion (28) v. South_Alligator_River (88) | 3161.5912 | 0.0010 | 0e+00 | 0.0010 | -5.0515 | 0.6953 | 0.0069 | 0.0010 | 0.0075 | 0.0010 | 0.0339 | 0.0010 | 0.0383 | 0.0010 | 0.0699 | 0.0010 |
| Darwin_Coastal (13) v. Indonesia (4) | 1425.2385 | 0.3616 | 2e-04 | 0.0310 | -193.2944 | 0.9609 | 0.0069 | 0.1139 | 0.0075 | 0.1149 | 0.8993 | 0.0431 | 0.9056 | 0.0431 | 0.9503 | 0.0431 |
| Sydney_Harbour (65) v. Towns_River (10) | 2467.2546 | 0.0010 | 1e-04 | 0.0010 | -7.8019 | 0.8841 | 0.0069 | 0.0010 | 0.0074 | 0.0010 | 0.1114 | 0.0020 | 0.1237 | 0.0020 | 0.2115 | 0.0020 |
| Seychelles (36) v. Towns_River (10) | 2141.3092 | 0.0010 | 1e-04 | 0.0030 | -10.6753 | 0.8951 | 0.0068 | 0.0040 | 0.0073 | 0.0040 | 0.1580 | 0.0070 | 0.1737 | 0.0070 | 0.2864 | 0.0070 |
| East_Alligator_River (47) v. Trinity_inlet (4) | 2454.4429 | 0.0010 | 1e-04 | 0.2258 | -18.1408 | 0.0161 | 0.0067 | 0.0440 | 0.0072 | 0.0440 | 0.4090 | 0.9335 | 0.4326 | 0.9215 | 0.5973 | 0.9245 |
| Arabian_Sea (15) v. Papua_New_Guinea (9) | 1716.2144 | 0.0999 | 1e-04 | 0.0739 | -15.0745 | 1.0000 | 0.0066 | 0.0080 | 0.0072 | 0.0080 | 0.2678 | 0.0030 | 0.2890 | 0.0030 | 0.4392 | 0.0030 |
| Daly_River (49) v. Trinity_inlet (4) | 2351.4533 | 0.0020 | 1e-04 | 0.0829 | -16.3546 | 0.0817 | 0.0066 | 0.0480 | 0.0070 | 0.0480 | 0.3846 | 0.7399 | 0.4085 | 0.7177 | 0.5728 | 0.7228 |
| Mozambique (11) v. Reunion (28) | 2001.5428 | 0.0100 | 1e-04 | 0.0330 | -11.6557 | 0.9820 | 0.0063 | 0.0140 | 0.0069 | 0.0140 | 0.1798 | 0.0020 | 0.1970 | 0.0020 | 0.3194 | 0.0020 |
| East_Alligator_River (47) v. Mozambique (11) | 2275.8266 | 0.0020 | 1e-04 | 0.0200 | -9.3675 | 0.8931 | 0.0062 | 0.0040 | 0.0068 | 0.0040 | 0.1348 | 0.0010 | 0.1489 | 0.0010 | 0.2500 | 0.0010 |
| Arabian_Sea (15) v. Trinity_inlet (4) | 1632.4740 | 0.0859 | 1e-04 | 0.3227 | -53.2932 | 0.4764 | 0.0061 | 0.0739 | 0.0066 | 0.0739 | 0.7051 | 0.4462 | 0.7221 | 0.4352 | 0.8370 | 0.4372 |
| Reunion (28) v. Sydney_Harbour (65) | 2891.3000 | 0.0010 | 0e+00 | 0.0010 | -5.8632 | 0.9770 | 0.0060 | 0.0010 | 0.0066 | 0.0010 | 0.0411 | 0.0010 | 0.0464 | 0.0010 | 0.0840 | 0.0010 |
| Sri_Lanka (12) v. Towns_River (10) | 1608.1513 | 0.0539 | 1e-04 | 0.0669 | -17.9931 | 0.9940 | 0.0060 | 0.0519 | 0.0066 | 0.0519 | 0.3108 | 0.0140 | 0.3330 | 0.0150 | 0.4911 | 0.0140 |
| Mozambique (11) v. South_Alligator_River (88) | 2752.7983 | 0.0040 | 1e-04 | 0.0170 | -6.2009 | 0.7053 | 0.0060 | 0.0020 | 0.0066 | 0.0020 | 0.0874 | 0.0010 | 0.0976 | 0.0010 | 0.1702 | 0.0010 |
| Seychelles (36) v. Trinity_inlet (4) | 2212.2961 | 0.0020 | 1e-04 | 0.1578 | -22.2859 | 0.0101 | 0.0060 | 0.0991 | 0.0064 | 0.0991 | 0.4651 | 0.9576 | 0.4886 | 0.9485 | 0.6510 | 0.9505 |
| East_Alligator_River (47) v. Reunion (28) | 2652.4144 | 0.0010 | 0e+00 | 0.0010 | -6.8905 | 0.7512 | 0.0058 | 0.0010 | 0.0063 | 0.0010 | 0.0520 | 0.0010 | 0.0584 | 0.0010 | 0.1050 | 0.0010 |
| Clarence_River (74) v. Reunion (28) | 2970.7179 | 0.0010 | 0e+00 | 0.0010 | -4.8433 | 1.0000 | 0.0058 | 0.0010 | 0.0063 | 0.0010 | 0.0335 | 0.0010 | 0.0379 | 0.0010 | 0.0690 | 0.0010 |
| Blue_mud_Bay (17) v. Sri_Lanka (12) | 1915.8700 | 0.0649 | 1e-04 | 0.0150 | -11.3891 | 0.9590 | 0.0057 | 0.0729 | 0.0062 | 0.0749 | 0.1798 | 0.0629 | 0.1975 | 0.0649 | 0.3198 | 0.0639 |
| Towns_River (10) v. Unknown (10) | 1526.0653 | 0.0480 | 1e-04 | 0.0190 | -21.7538 | 0.9261 | 0.0056 | 0.0989 | 0.0061 | 0.0969 | 0.3753 | 0.0939 | 0.3984 | 0.0959 | 0.5625 | 0.0959 |
| Trinity_inlet (4) v. Wenlock_River (24) | 1904.5321 | 0.0280 | 1e-04 | 0.3397 | -29.6457 | 0.1071 | 0.0055 | 0.1031 | 0.0060 | 0.1048 | 0.5499 | 0.7970 | 0.5722 | 0.7788 | 0.7240 | 0.7848 |
| Papua_New_Guinea (9) v. Sri_Lanka (12) | 1525.0491 | 0.1588 | 1e-04 | 0.2418 | -19.5709 | 0.9830 | 0.0055 | 0.0340 | 0.0059 | 0.0340 | 0.3410 | 0.0120 | 0.3636 | 0.0120 | 0.5255 | 0.0120 |

Table 46: Pairwise Fst for 3416 DArTcap loci between the IWP sites *(continued)*

| | Chi2 | Chi2.p.val | D | D.p.val | Fis | Fis.p.val | Fst | Fst.p.val | F'st | F'st.p.val | Gst | Gst.p.val | G'st | G'st.p.val | G"st | G"st.p.val |
|---|---|---|---|---|---|---|---|---|---|---|---|---|---|---|---|---|
| East_Alligator_River (47) v. Fitzroy_River (3) | 1989.2696 | 0.1598 | 1e-04 | 0.1119 | NA | NA | 0.0054 | 0.1721 | 0.0058 | 0.1711 | NA | NA | NA | NA | NA | NA |
| Reunion (28) v. Unknown (10) | 1995.5362 | 0.0070 | 1e-04 | 0.0110 | -11.6580 | 0.3177 | 0.0054 | 0.0350 | 0.0059 | 0.0370 | 0.1785 | 0.2308 | 0.1956 | 0.2098 | 0.3175 | 0.2158 |
| Reunion (28) v. Wenlock_River (24) | 2317.4735 | 0.0010 | 1e-04 | 0.0010 | -8.0692 | 0.9760 | 0.0053 | 0.0010 | 0.0058 | 0.0010 | 0.0790 | 0.0010 | 0.0884 | 0.0010 | 0.1552 | 0.0010 |
| Papua_New_Guinea (9) v. Seychelles (36) | 1930.8100 | 0.0450 | 1e-04 | 0.1219 | -11.4056 | 0.8921 | 0.0053 | 0.0140 | 0.0059 | 0.0140 | 0.1769 | 0.0310 | 0.1937 | 0.0320 | 0.3149 | 0.0320 |
| Clarence_River (74) v. Papua_New_Guinea (9) | 2326.9965 | 0.5385 | 1e-04 | 0.0170 | -6.5637 | 0.9980 | 0.0052 | 0.0110 | 0.0056 | 0.0110 | 0.1010 | 0.0010 | 0.1125 | 0.0010 | 0.1940 | 0.0010 |
| Thailand (4) v. Towns_River (10) | 1371.3016 | 0.0480 | 1e-04 | 0.3187 | 123.5569 | 0.0511 | 0.0052 | 0.2523 | 0.0058 | 0.2523 | 1.1964 | 0.9559 | 1.1823 | 0.9559 | 1.0830 | 0.9559 |
| Clarence_River (74) v. Mozambique (11) | 2553.2382 | 0.0629 | 1e-04 | 0.0090 | -6.0722 | 1.0000 | 0.0052 | 0.0040 | 0.0057 | 0.0040 | 0.0872 | 0.0010 | 0.0975 | 0.0010 | 0.1699 | 0.0010 |
| Arabian_Sea (15) v. Darwin_Coastal (13) | 1910.4886 | 0.0050 | 0e+00 | 0.5145 | -11.1126 | 0.9780 | 0.0052 | 0.0050 | 0.0056 | 0.0050 | 0.1778 | 0.0060 | 0.1955 | 0.0050 | 0.3169 | 0.0050 |
| Mozambique (11) v. Seychelles (36) | 2100.4591 | 0.0010 | 1e-04 | 0.0010 | -10.5278 | 0.9920 | 0.0051 | 0.0030 | 0.0056 | 0.0030 | 0.1556 | 0.0010 | 0.1713 | 0.0010 | 0.2829 | 0.0010 |
| Adelaide_River (18) v. Papua_New_Guinea (9) | 1633.3815 | 0.1299 | 1e-04 | 0.0210 | -15.5144 | 0.9151 | 0.0051 | 0.0320 | 0.0055 | 0.0320 | 0.2595 | 0.0290 | 0.2800 | 0.0280 | 0.4283 | 0.0280 |
| Darwin_Coastal (13) v. Mozambique (11) | 1670.2078 | 0.0739 | 1e-04 | 0.0330 | -15.5169 | 0.9990 | 0.0050 | 0.0320 | 0.0057 | 0.0300 | 0.2737 | 0.0010 | 0.2955 | 0.0010 | 0.4469 | 0.0010 |
| Daly_River (49) v. Mozambique (11) | 2340.9063 | 0.0010 | 1e-04 | 0.0300 | -8.6633 | 0.9960 | 0.0049 | 0.0010 | 0.0054 | 0.0010 | 0.1254 | 0.0010 | 0.1389 | 0.0010 | 0.2348 | 0.0010 |
| Adelaide_River (18) v. Reunion (28) | 2121.8280 | 0.0020 | 0e+00 | 0.0080 | -9.2177 | 0.7982 | 0.0049 | 0.0040 | 0.0054 | 0.0040 | 0.1025 | 0.0220 | 0.1140 | 0.0200 | 0.1964 | 0.0200 |
| Darwin_Coastal (13) v. Fitzroy_River (3) | 1348.0815 | 0.6484 | 2e-04 | 0.1099 | NA | NA | 0.0047 | 0.2063 | 0.0051 | 0.2063 | NA | NA | NA | NA | NA | NA |
| Trinity_inlet (4) v. Unknown (10) | 1339.6359 | 0.0210 | 1e-04 | 0.3457 | 161.3117 | 0.0524 | 0.0045 | 0.1497 | 0.0049 | 0.1503 | 1.1420 | 0.9568 | 1.1318 | 0.9568 | 1.0615 | 0.9568 |
| Reunion (28) v. Seychelles (36) | 2332.0104 | 0.0010 | 0e+00 | 0.0080 | -7.5897 | 0.9970 | 0.0045 | 0.0010 | 0.0049 | 0.0010 | 0.0621 | 0.0010 | 0.0697 | 0.0010 | 0.1240 | 0.0010 |
| Fitzroy_River (3) v. Sri_Lanka (12) | 1355.5475 | 0.3746 | 1e-04 | 0.2877 | NA | NA | 0.0044 | 0.2205 | 0.0048 | 0.2247 | NA | NA | NA | NA | NA | NA |
| Clarence_River (74) v. Trinity_inlet (4) | 2669.1115 | 0.0260 | 1e-04 | 0.2018 | -9.2359 | 0.2214 | 0.0043 | 0.1263 | 0.0046 | 0.1273 | 0.2559 | 0.4541 | 0.2776 | 0.4255 | 0.4248 | 0.4378 |
| Clarence_River (74) v. Roper_River (9) | 2508.0552 | 0.1179 | 1e-04 | 0.0050 | -6.3572 | 0.8791 | 0.0042 | 0.0320 | 0.0045 | 0.0320 | 0.1012 | 0.0020 | 0.1130 | 0.0020 | 0.1945 | 0.0020 |
| Arabian_Sea (15) v. Mozambique (11) | 1795.4018 | 0.0869 | 1e-04 | 0.2717 | -13.4631 | 1.0000 | 0.0041 | 0.0290 | 0.0047 | 0.0250 | 0.2345 | 0.0010 | 0.2548 | 0.0010 | 0.3964 | 0.0010 |
| Daly_River (49) v. Reunion (28) | 2558.8318 | 0.0010 | 0e+00 | 0.0010 | -6.5805 | 0.9890 | 0.0040 | 0.0010 | 0.0044 | 0.0010 | 0.0485 | 0.0010 | 0.0546 | 0.0010 | 0.0983 | 0.0010 |
| Adelaide_River (18) v. Darwin_Coastal (13) | 1862.0462 | 0.0120 | 1e-04 | 0.1179 | -11.3164 | 0.3497 | 0.0040 | 0.0240 | 0.0044 | 0.0240 | 0.1680 | 0.0659 | 0.1847 | 0.0619 | 0.3020 | 0.0639 |
| Darwin_Coastal (13) v. Papua_New_Guinea (9) | 1550.2356 | 0.3237 | 1e-04 | 0.5195 | -17.3639 | 0.9980 | 0.0040 | 0.0729 | 0.0044 | 0.0729 | 0.3074 | 0.0100 | 0.3297 | 0.0120 | 0.4873 | 0.0110 |
| Roper_River (9) v. South_Alligator_River (88) | 2717.7623 | 0.0140 | 1e-04 | 0.0040 | -6.4670 | 0.1588 | 0.0039 | 0.0400 | 0.0043 | 0.0400 | 0.1007 | 0.0120 | 0.1122 | 0.0090 | 0.1934 | 0.0100 |
| Sydney_Harbour (65) v. Wenlock_River (24) | 2707.1511 | 0.0010 | 0e+00 | 0.0010 | -5.8688 | 0.3287 | 0.0039 | 0.0010 | 0.0042 | 0.0010 | 0.0440 | 0.0010 | 0.0497 | 0.0010 | 0.0898 | 0.0010 |
| Blue_mud_Bay (17) v. Thailand (4) | 1612.2558 | 0.2567 | 1e-04 | 0.1259 | -43.0209 | 0.3513 | 0.0037 | 0.3029 | 0.0039 | 0.3039 | 0.6487 | 0.6487 | 0.6678 | 0.6487 | 0.7985 | 0.6477 |
| Arabian_Sea (15) v. South_Alligator_River (88) | 3346.1117 | 0.0010 | 0e+00 | 0.0110 | -5.1203 | 0.0120 | 0.0037 | 0.0020 | 0.0040 | 0.0020 | 0.0516 | 0.0779 | 0.0581 | 0.0579 | 0.1043 | 0.0679 |
| Adelaide_River (18) v. Roper_River (9) | 1736.7142 | 0.0190 | 1e-04 | 0.1049 | -15.0451 | 0.7932 | 0.0037 | 0.0779 | 0.0040 | 0.0769 | 0.2589 | 0.0330 | 0.2798 | 0.0290 | 0.4279 | 0.0290 |
| Reunion (28) v. Sri_Lanka (12) | 2078.6819 | 0.0060 | 1e-04 | 0.1169 | -10.3263 | 0.1908 | 0.0036 | 0.0729 | 0.0039 | 0.0729 | 0.1420 | 0.4575 | 0.1568 | 0.4416 | 0.2617 | 0.4525 |
| Reunion (28) v. South_Africa (21) | 2165.2760 | 0.0050 | 0e+00 | 0.0030 | -8.5336 | 0.7033 | 0.0036 | 0.0170 | 0.0039 | 0.0180 | 0.0878 | 0.0310 | 0.0980 | 0.0280 | 0.1708 | 0.0300 |
| East_Alligator_River (47) v. Papua_New_Guinea (9) | 2053.2167 | 0.0889 | 1e-04 | 0.0719 | -10.1188 | 0.5854 | 0.0036 | 0.0480 | 0.0039 | 0.0480 | 0.1528 | 0.0839 | 0.1679 | 0.0779 | 0.2782 | 0.0809 |
| Darwin_Coastal (13) v. East_Alligator_River (47) | 2477.1204 | 0.0010 | 0e+00 | 0.0799 | -7.9754 | 0.0020 | 0.0036 | 0.0080 | 0.0039 | 0.0080 | 0.0952 | 0.4356 | 0.1062 | 0.3427 | 0.1838 | 0.3846 |
| Papua_New_Guinea (9) v. Sydney_Harbour (65) | 2113.4285 | 0.5914 | 1e-04 | 0.0579 | -8.3339 | 0.9191 | 0.0036 | 0.0280 | 0.0039 | 0.0290 | 0.1251 | 0.0330 | 0.1383 | 0.0330 | 0.2341 | 0.0330 |
| Mozambique (11) v. Sydney_Harbour (65) | 2433.8819 | 0.0030 | 1e-04 | 0.0260 | -7.5988 | 0.9481 | 0.0035 | 0.0150 | 0.0038 | 0.0150 | 0.1068 | 0.0010 | 0.1187 | 0.0010 | 0.2037 | 0.0010 |
| Mozambique (11) v. Roper_River (9) | 1555.3710 | 0.0460 | 1e-04 | 0.0490 | -23.2409 | 0.4456 | 0.0035 | 0.1279 | 0.0039 | 0.1309 | 0.4092 | 0.4595 | 0.4327 | 0.4486 | 0.5974 | 0.4515 |
| Darwin_Coastal (13) v. Towns_River (10) | 1645.6191 | 0.0939 | 1e-04 | 0.3626 | -15.9843 | 0.9850 | 0.0035 | 0.1658 | 0.0038 | 0.1658 | 0.2784 | 0.0410 | 0.3001 | 0.0450 | 0.4525 | 0.0430 |
| East_Alligator_River (47) v. Roper_River (9) | 2200.2523 | 0.0040 | 1e-04 | 0.0739 | -9.9052 | 0.1708 | 0.0034 | 0.0539 | 0.0037 | 0.0519 | 0.1549 | 0.0430 | 0.1705 | 0.0340 | 0.2817 | 0.0380 |
| Daly_River (49) v. Unknown (10) | 2182.7564 | 0.0220 | 1e-04 | 0.0020 | -8.8620 | 0.5774 | 0.0033 | 0.0270 | 0.0036 | 0.0270 | 0.1269 | 0.0160 | 0.1405 | 0.0150 | 0.2373 | 0.0160 |
| Mozambique (11) v. Sri_Lanka (12) | 1608.2682 | 0.1489 | 1e-04 | 0.1938 | -17.2515 | 0.9970 | 0.0033 | 0.1189 | 0.0036 | 0.1219 | 0.3013 | 0.0030 | 0.3235 | 0.0030 | 0.4802 | 0.0030 |
| Mozambique (11) v. Unknown (10) | 1573.3394 | 0.0240 | 1e-04 | 0.0160 | -20.5176 | 0.8462 | 0.0033 | 0.1129 | 0.0036 | 0.1179 | 0.3614 | 0.0909 | 0.3846 | 0.0889 | 0.5479 | 0.0899 |
| Fitzroy_River (3) v. Trinity_inlet (4) | 957.9348 | 0.5135 | 2e-04 | 0.1219 | NA | NA | 0.0031 | 0.4070 | 0.0034 | 0.4070 | NA | NA | NA | NA | NA | NA |
| Darwin_Coastal (13) v. Unknown (10) | 1631.5012 | 0.0659 | 1e-04 | 0.0280 | -15.4535 | 0.9820 | 0.0030 | 0.0879 | 0.0033 | 0.0859 | 0.2715 | 0.0190 | 0.2932 | 0.0200 | 0.4442 | 0.0190 |
| Mozambique (11) v. Trinity_inlet (4) | 1410.4190 | 0.0619 | 2e-04 | 0.1439 | 174.0446 | 0.0260 | 0.0030 | 0.2226 | 0.0032 | 0.2270 | 1.1295 | 0.9812 | 1.1202 | 0.9812 | 1.0564 | 0.9812 |
| Papua_New_Guinea (9) v. South_Alligator_River (88) | 2486.1042 | 0.1788 | 0e+00 | 0.1938 | -6.6420 | 0.8042 | 0.0030 | 0.0759 | 0.0032 | 0.0759 | 0.0993 | 0.0589 | 0.1103 | 0.0569 | 0.1906 | 0.0569 |
| Seychelles (36) v. Unknown (10) | 1986.4551 | 0.0110 | 1e-04 | 0.0340 | -10.6108 | 0.3626 | 0.0029 | 0.0659 | 0.0032 | 0.0659 | 0.1552 | 0.1548 | 0.1709 | 0.1479 | 0.2823 | 0.1508 |
| Roper_River (9) v. Wenlock_River (24) | 1824.1022 | 0.1688 | 1e-04 | 0.1548 | -12.4579 | 0.8861 | 0.0029 | 0.1069 | 0.0031 | 0.1089 | 0.2101 | 0.0270 | 0.2292 | 0.0270 | 0.3630 | 0.0260 |
| Clarence_River (74) v. Seychelles (36) | 2842.1582 | 0.0010 | 0e+00 | 0.0010 | -4.5702 | 1.0000 | 0.0028 | 0.0010 | 0.0031 | 0.0010 | 0.0255 | 0.0010 | 0.0290 | 0.0010 | 0.0532 | 0.0010 |
| Daly_River (49) v. Roper_River (9) | 2196.8381 | 0.0170 | 1e-04 | 0.0140 | -9.2527 | 0.5425 | 0.0028 | 0.0739 | 0.0030 | 0.0729 | 0.1453 | 0.0250 | 0.1604 | 0.0250 | 0.2669 | 0.0240 |
| Darwin_Coastal (13) v. Trinity_inlet (4) | 1482.6685 | 0.1758 | 1e-04 | 0.2757 | -97.0409 | 0.3746 | 0.0027 | 0.2527 | 0.0029 | 0.2527 | 0.8177 | 0.6042 | 0.8291 | 0.6012 | 0.9060 | 0.6012 |
| Roper_River (9) v. Unknown (10) | 1497.2012 | 0.0210 | 1e-04 | 0.0030 | -23.5918 | 0.7962 | 0.0027 | 0.1618 | 0.0029 | 0.1638 | 0.4115 | 0.1628 | 0.4350 | 0.1618 | 0.5997 | 0.1618 |
| Roper_River (9) v. Sri_Lanka (12) | 1577.5046 | 0.0509 | 1e-04 | 0.0899 | -19.0935 | 0.9311 | 0.0027 | 0.1439 | 0.0029 | 0.1499 | 0.3405 | 0.0330 | 0.3635 | 0.0330 | 0.5252 | 0.0330 |
| Papua_New_Guinea (9) v. Wenlock_River (24) | 1756.5815 | 0.4076 | 1e-04 | 0.1928 | -12.6433 | 0.9850 | 0.0026 | 0.1479 | 0.0029 | 0.1479 | 0.2068 | 0.0200 | 0.2253 | 0.0220 | 0.3580 | 0.0200 |
| Papua_New_Guinea (9) v. Roper_River (9) | 1390.0644 | 0.2827 | 1e-04 | 0.4116 | -28.3085 | 0.7512 | 0.0026 | 0.2358 | 0.0029 | 0.2308 | 0.4677 | 0.2198 | 0.4904 | 0.2178 | 0.6528 | 0.2178 |

Table 46: Pairwise Fst for 3416 DArTcap loci between the IWP sites *(continued)*

| | Chi2 | Chi2.p.val | D | D.p.val | Fis | Fis.p.val | Fst | Fst.p.val | F'st | F'st.p.val | Gst | Gst.p.val | G'st | G'st.p.val | G"st | G"st.p.val |
|---|---|---|---|---|---|---|---|---|---|---|---|---|---|---|---|---|
| Seychelles (36) v. Wenlock_River (24) | 2314.3078 | 0.0010 | 0e+00 | 0.0140 | -7.4752 | 0.3876 | 0.0026 | 0.0080 | 0.0028 | 0.0080 | 0.0651 | 0.0210 | 0.0731 | 0.0200 | 0.1298 | 0.0200 |
| Unknown (10) v. Wenlock_River (24) | 1812.7937 | 0.1528 | 1e-04 | 0.0270 | -11.7854 | 0.8362 | 0.0026 | 0.0779 | 0.0028 | 0.0799 | 0.1848 | 0.0280 | 0.2024 | 0.0280 | 0.3268 | 0.0290 |
| Seychelles (36) v. Sydney_Harbour (65) | 2677.3422 | 0.0010 | 0e+00 | 0.0020 | -5.6200 | 0.9151 | 0.0026 | 0.0010 | 0.0028 | 0.0010 | 0.0321 | 0.0010 | 0.0363 | 0.0010 | 0.0662 | 0.0010 |
| Arabian_Sea (15) v. East_Alligator_River (47) | 2544.4612 | 0.0010 | 0e+00 | 0.4655 | -7.4604 | 0.0070 | 0.0025 | 0.0240 | 0.0027 | 0.0240 | 0.0803 | 0.4735 | 0.0898 | 0.3936 | 0.1575 | 0.4276 |
| South_Africa (21) v. Unknown (10) | 1709.9569 | 0.2907 | 1e-04 | 0.2188 | -12.8478 | 0.7812 | 0.0024 | 0.1079 | 0.0026 | 0.1079 | 0.2046 | 0.0509 | 0.2232 | 0.0480 | 0.3551 | 0.0500 |
| Thailand (4) v. Trinity_inlet (4) | 1056.3377 | 0.2228 | 1e-04 | 0.4955 | 15.2219 | 0.7152 | 0.0024 | 0.3327 | 0.0026 | 0.3157 | 9.5347 | 0.2690 | 8.5308 | 0.2690 | 1.7149 | 0.2384 |
| Darwin_Coastal (13) v. Sydney_Harbour (65) | 2601.2776 | 0.0010 | 0e+00 | 0.3257 | -6.6880 | 0.0050 | 0.0023 | 0.0350 | 0.0025 | 0.0350 | 0.0765 | 0.5445 | 0.0858 | 0.4835 | 0.1508 | 0.5005 |
| Darwin_Coastal (13) v. Sri_Lanka (12) | 1708.2605 | 0.1049 | 1e-04 | 0.1808 | -13.3115 | 0.8821 | 0.0023 | 0.1578 | 0.0025 | 0.1578 | 0.2228 | 0.1499 | 0.2427 | 0.1499 | 0.3807 | 0.1499 |
| Adelaide_River (18) v. Unknown (10) | 1608.8782 | 0.3317 | 1e-04 | 0.0500 | -14.2537 | 0.6294 | 0.0023 | 0.1399 | 0.0025 | 0.1389 | 0.2308 | 0.1019 | 0.2506 | 0.0909 | 0.3911 | 0.0929 |
| Daly_River (49) v. Darwin_Coastal (13) | 2432.8262 | 0.0010 | 0e+00 | 0.3187 | -7.5514 | 0.0400 | 0.0023 | 0.0509 | 0.0024 | 0.0509 | 0.0895 | 0.3886 | 0.1001 | 0.3447 | 0.1741 | 0.3636 |
| Darwin_Coastal (13) v. South_Alligator_River (88) | 3020.1834 | 0.0010 | 0e+00 | 0.0689 | -5.4505 | 0.0070 | 0.0022 | 0.0460 | 0.0024 | 0.0460 | 0.0608 | 0.5495 | 0.0683 | 0.4705 | 0.1217 | 0.5005 |
| Darwin_Coastal (13) v. Seychelles (36) | 2232.0110 | 0.0010 | 0e+00 | 0.1778 | -8.7589 | 0.0220 | 0.0022 | 0.0719 | 0.0024 | 0.0709 | 0.1092 | 0.6114 | 0.1215 | 0.5495 | 0.2080 | 0.5774 |
| Papua_New_Guinea (9) v. South_Africa (21) | 1692.4121 | 0.3646 | 1e-04 | 0.0959 | -13.8424 | 0.9720 | 0.0022 | 0.1778 | 0.0024 | 0.1778 | 0.2283 | 0.0410 | 0.2477 | 0.0400 | 0.3876 | 0.0400 |
| Clarence_River (74) v. South_Africa (21) | 2637.0514 | 0.0330 | 0e+00 | 0.0140 | -5.0636 | 0.9491 | 0.0022 | 0.0110 | 0.0024 | 0.0110 | 0.0401 | 0.0050 | 0.0453 | 0.0050 | 0.0821 | 0.0050 |
| Roper_River (9) v. Sydney_Harbour (65) | 2381.0609 | 0.0120 | 1e-04 | 0.0270 | -7.9318 | 0.3576 | 0.0021 | 0.1239 | 0.0023 | 0.1259 | 0.1231 | 0.0420 | 0.1365 | 0.0370 | 0.2312 | 0.0380 |
| Clarence_River (74) v. Sri_Lanka (12) | 2584.2490 | 0.0599 | 0e+00 | 0.0509 | -5.7213 | 0.8621 | 0.0021 | 0.0769 | 0.0023 | 0.0769 | 0.0690 | 0.0759 | 0.0776 | 0.0819 | 0.1372 | 0.0799 |
| Arabian_Sea (15) v. Sydney_Harbour (65) | 2905.2078 | 0.0010 | 0e+00 | 0.0320 | -6.1908 | 0.0470 | 0.0021 | 0.0350 | 0.0023 | 0.0350 | 0.0636 | 0.3806 | 0.0715 | 0.3407 | 0.1271 | 0.3616 |
| Adelaide_River (18) v. Wenlock_River (24) | 1976.3190 | 0.0609 | 0e+00 | 0.0370 | -9.2034 | 0.9471 | 0.0021 | 0.0689 | 0.0023 | 0.0689 | 0.1065 | 0.0480 | 0.1184 | 0.0480 | 0.2033 | 0.0490 |
| Fitzroy_River (3) v. South_Alligator_River (88) | 2156.0229 | 0.7113 | 1e-04 | 0.2857 | NA | NA | 0.0021 | 0.3323 | NA | NA | NA | NA | NA | NA | NA | NA |
| Daly_River (49) v. Sri_Lanka (12) | 2290.0326 | 0.0070 | 0e+00 | 0.1129 | -8.0934 | 0.4046 | 0.0021 | 0.0879 | 0.0022 | 0.0879 | 0.1006 | 0.2408 | 0.1120 | 0.2218 | 0.1931 | 0.2288 |
| Arabian_Sea (15) v. Reunion (28) | 2269.4690 | 0.0010 | 0e+00 | 0.0789 | -8.7636 | 0.0380 | 0.0020 | 0.1409 | 0.0022 | 0.1389 | 0.1073 | 0.5714 | 0.1194 | 0.5365 | 0.2047 | 0.5524 |
| Arabian_Sea (15) v. Clarence_River (74) | 3038.3218 | 0.0010 | 0e+00 | 0.4965 | -5.0190 | 0.4326 | 0.0020 | 0.0569 | 0.0022 | 0.0569 | 0.0511 | 0.1099 | 0.0578 | 0.1059 | 0.1036 | 0.1079 |
| Seychelles (36) v. South_Alligator_River (88) | 2819.2585 | 0.0010 | 0e+00 | 0.0010 | -4.7139 | 0.4575 | 0.0020 | 0.0010 | 0.0022 | 0.0010 | 0.0245 | 0.0010 | 0.0278 | 0.0010 | 0.0511 | 0.0010 |
| Roper_River (9) v. South_Africa (21) | 1789.5620 | 0.0400 | 1e-04 | 0.2418 | -13.6047 | 0.8551 | 0.0019 | 0.1958 | 0.0021 | 0.1998 | 0.2309 | 0.0490 | 0.2508 | 0.0480 | 0.3913 | 0.0480 |
| South_Africa (21) v. Wenlock_River (24) | 2050.3229 | 0.0330 | 0e+00 | 0.0370 | -8.5770 | 0.9201 | 0.0019 | 0.0679 | 0.0021 | 0.0679 | 0.0926 | 0.0659 | 0.1033 | 0.0659 | 0.1793 | 0.0659 |
| Arabian_Sea (15) v. Daly_River (49) | 2622.2411 | 0.0010 | 0e+00 | 0.2547 | -6.9893 | 0.0769 | 0.0019 | 0.0699 | 0.0020 | 0.0699 | 0.0749 | 0.3407 | 0.0840 | 0.2857 | 0.1478 | 0.3027 |
| Adelaide_River (18) v. Mozambique (11) | 1707.2964 | 0.1618 | 1e-04 | 0.3506 | -13.9099 | 0.9970 | 0.0018 | 0.2008 | 0.0021 | 0.1948 | 0.2267 | 0.0010 | 0.2462 | 0.0010 | 0.3855 | 0.0010 |
| Roper_River (9) v. Seychelles (36) | 1987.1160 | 0.0260 | 1e-04 | 0.2118 | -11.1310 | 0.4176 | 0.0018 | 0.2278 | 0.0020 | 0.2298 | 0.1767 | 0.1189 | 0.1938 | 0.1149 | 0.3149 | 0.1159 |
| Clarence_River (74) v. Wenlock_River (24) | 2748.2030 | 0.0090 | 0e+00 | 0.0060 | -4.7666 | 0.8991 | 0.0018 | 0.0160 | 0.0020 | 0.0160 | 0.0342 | 0.0080 | 0.0388 | 0.0080 | 0.0706 | 0.0080 |
| Daly_River (49) v. Fitzroy_River (3) | 2019.8560 | 0.2847 | 1e-04 | 0.3556 | NA | NA | 0.0018 | 0.3371 | 0.0019 | 0.3371 | NA | NA | NA | NA | NA | NA |
| Daly_River (49) v. Wenlock_River (24) | 2405.4276 | 0.0030 | 0e+00 | 0.1479 | -6.5842 | 0.3816 | 0.0018 | 0.0230 | 0.0020 | 0.0230 | 0.0517 | 0.0300 | 0.0582 | 0.0300 | 0.1045 | 0.0290 |
| Adelaide_River (18) v. Seychelles (36) | 2109.7935 | 0.0110 | 0e+00 | 0.0440 | -8.5416 | 0.5664 | 0.0018 | 0.0679 | 0.0020 | 0.0679 | 0.0864 | 0.1379 | 0.0964 | 0.1359 | 0.1682 | 0.1389 |
| South_Alligator_River (88) v. Wenlock_River (24) | 2854.0892 | 0.0010 | 0e+00 | 0.0559 | -4.9225 | 0.0430 | 0.0018 | 0.0190 | 0.0019 | 0.0190 | 0.0339 | 0.0649 | 0.0384 | 0.0539 | 0.0699 | 0.0589 |
| Arabian_Sea (15) v. Unknown (10) | 1714.4087 | 0.1798 | 1e-04 | 0.0659 | -13.6519 | 0.9341 | 0.0018 | 0.1808 | 0.0019 | 0.1808 | 0.2356 | 0.0450 | 0.2559 | 0.0450 | 0.3978 | 0.0450 |
| Adelaide_River (18) v. Arabian_Sea (15) | 1898.0038 | 0.0500 | 0e+00 | 0.4765 | -10.3121 | 0.3177 | 0.0017 | 0.1489 | 0.0019 | 0.1479 | 0.1437 | 0.2378 | 0.1586 | 0.2238 | 0.2643 | 0.2328 |
| Sri_Lanka (12) v. Sydney_Harbour (65) | 2404.9826 | 0.0320 | 0e+00 | 0.0679 | -7.1263 | 0.2767 | 0.0017 | 0.1159 | 0.0019 | 0.1159 | 0.0856 | 0.5035 | 0.0957 | 0.4865 | 0.1670 | 0.4975 |
| Arabian_Sea (15) v. Roper_River (9) | 1686.4100 | 0.2348 | 1e-04 | 0.2547 | -14.9567 | 0.9491 | 0.0017 | 0.2398 | 0.0019 | 0.2368 | 0.2697 | 0.0330 | 0.2914 | 0.0300 | 0.4419 | 0.0310 |
| South_Alligator_River (88) v. Sydney_Harbour (65) | 3078.4199 | 0.0010 | 0e+00 | 0.0020 | -3.8867 | 0.6064 | 0.0017 | 0.0010 | 0.0018 | 0.0010 | 0.0142 | 0.0010 | 0.0162 | 0.0010 | 0.0300 | 0.0010 |
| Clarence_River (74) v. Darwin_Coastal (13) | 2647.7639 | 0.0190 | 0e+00 | 0.1628 | -5.4426 | 0.2697 | 0.0017 | 0.1129 | 0.0018 | 0.1129 | 0.0618 | 0.2298 | 0.0697 | 0.2188 | 0.1238 | 0.2228 |
| East_Alligator_River (47) v. Wenlock_River (24) | 2346.9028 | 0.0020 | 0e+00 | 0.0879 | -6.9942 | 0.0120 | 0.0017 | 0.0260 | 0.0018 | 0.0260 | 0.0552 | 0.1109 | 0.0620 | 0.0979 | 0.1111 | 0.1069 |
| Daly_River (49) v. Papua_New_Guinea (9) | 2036.7218 | 0.3197 | 1e-04 | 0.1908 | -9.4690 | 0.9291 | 0.0016 | 0.2008 | 0.0017 | 0.1998 | 0.1432 | 0.0549 | 0.1578 | 0.0559 | 0.2633 | 0.0559 |
| Adelaide_River (18) v. South_Africa (21) | 1879.9233 | 0.1029 | 0e+00 | 0.1249 | -9.9455 | 0.8831 | 0.0016 | 0.1359 | 0.0017 | 0.1359 | 0.1199 | 0.1239 | 0.1329 | 0.1229 | 0.2257 | 0.1229 |
| Clarence_River (74) v. South_Alligator_River (88) | 3314.7268 | 0.0010 | 0e+00 | 0.0010 | -3.1910 | 0.0969 | 0.0016 | 0.0010 | 0.0017 | 0.0010 | 0.0110 | 0.0010 | 0.0125 | 0.0010 | 0.0233 | 0.0010 |
| Sri_Lanka (12) v. Wenlock_River (24) | 1903.0051 | 0.1628 | 0e+00 | 0.5215 | -10.3649 | 0.6304 | 0.0015 | 0.1858 | 0.0017 | 0.1858 | 0.1472 | 0.2777 | 0.1624 | 0.2717 | 0.2699 | 0.2727 |
| South_Africa (21) v. Sydney_Harbour (65) | 2507.1685 | 0.0040 | 0e+00 | 0.0699 | -6.1389 | 0.3097 | 0.0015 | 0.0519 | 0.0016 | 0.0519 | 0.0490 | 0.1399 | 0.0552 | 0.1349 | 0.0994 | 0.1369 |
| Sri_Lanka (12) v. Unknown (10) | 1585.8206 | 0.0509 | 1e-04 | 0.0260 | -17.1253 | 0.9001 | 0.0015 | 0.2248 | 0.0016 | 0.2238 | 0.2984 | 0.0959 | 0.3205 | 0.0969 | 0.4767 | 0.0959 |
| Mozambique (11) v. South_Africa (21) | 1765.9109 | 0.2607 | 0e+00 | 0.6713 | -12.6116 | 0.9980 | 0.0015 | 0.1968 | 0.0016 | 0.2028 | 0.2012 | 0.0010 | 0.2197 | 0.0010 | 0.3504 | 0.0010 |
| South_Alligator_River (88) v. Unknown (10) | 2469.8689 | 0.1908 | 1e-04 | 0.0190 | -6.3938 | 0.2088 | 0.0014 | 0.1758 | 0.0015 | 0.1758 | 0.0879 | 0.1908 | 0.0981 | 0.1708 | 0.1710 | 0.1798 |
| Arabian_Sea (15) v. South_Africa (21) | 2004.8732 | 0.0100 | 0e+00 | 0.3047 | -9.5733 | 0.2667 | 0.0013 | 0.1838 | 0.0014 | 0.1828 | 0.1269 | 0.3077 | 0.1407 | 0.2937 | 0.2375 | 0.2987 |
| Daly_River (49) v. Seychelles (36) | 2423.7529 | 0.0030 | 0e+00 | 0.0010 | -6.2612 | 0.9261 | 0.0013 | 0.0300 | 0.0014 | 0.0300 | 0.0386 | 0.0210 | 0.0435 | 0.0220 | 0.0791 | 0.0220 |
| Darwin_Coastal (13) v. Wenlock_River (24) | 1943.7442 | 0.1039 | 1e-04 | 0.0380 | -9.6767 | 0.1798 | 0.0013 | 0.1978 | 0.0014 | 0.1978 | 0.1328 | 0.4356 | 0.1472 | 0.4166 | 0.2472 | 0.4236 |
| Clarence_River (74) v. Unknown (10) | 2399.8405 | 0.3956 | 0e+00 | 0.1109 | -6.1173 | 0.9371 | 0.0013 | 0.2008 | 0.0014 | 0.2008 | 0.0860 | 0.0280 | 0.0962 | 0.0280 | 0.1678 | 0.0280 |
| Clarence_River (74) v. Daly_River (49) | 2752.4200 | 0.0260 | 0e+00 | 0.0150 | -4.1806 | 1.0000 | 0.0012 | 0.0130 | 0.0012 | 0.0130 | 0.0187 | 0.0090 | 0.0213 | 0.0090 | 0.0392 | 0.0090 |

Table 46: Pairwise Fst for 3416 DArTcap loci between the IWP sites *(continued)*

| | Chi2 | Chi2.p.val | D | D.p.val | Fis | Fis.p.val | Fst | Fst.p.val | F'st | F'st.p.val | Gst | Gst.p.val | G'st | G'st.p.val | G"st | G"st.p.val |
|---|---|---|---|---|---|---|---|---|---|---|---|---|---|---|---|---|
| Daly_River (49) v. South_Alligator_River (88) | 2810.8610 | 0.0030 | 0e+00 | 0.0150 | -4.3444 | 0.1948 | 0.0011 | 0.0110 | 0.0012 | 0.0110 | 0.0182 | 0.0160 | 0.0206 | 0.0140 | 0.0382 | 0.0140 |
| Clarence_River (74) v. Sydney_Harbour (65) | 2878.2925 | 0.0060 | 0e+00 | 0.3536 | -3.8645 | 1.0000 | 0.0011 | 0.0080 | 0.0012 | 0.0080 | 0.0149 | 0.0070 | 0.0170 | 0.0070 | 0.0315 | 0.0070 |
| East_Alligator_River (47) v. Seychelles (36) | 2325.5770 | 0.0100 | 0e+00 | 0.1009 | -6.6050 | 0.2208 | 0.0011 | 0.0529 | 0.0012 | 0.0529 | 0.0411 | 0.0569 | 0.0463 | 0.0549 | 0.0839 | 0.0559 |
| East_Alligator_River (47) v. South_Africa (21) | 2308.1099 | 0.0040 | 0e+00 | 0.3487 | -7.2438 | 0.0230 | 0.0011 | 0.1039 | 0.0012 | 0.1039 | 0.0617 | 0.3816 | 0.0692 | 0.3467 | 0.1233 | 0.3596 |
| Adelaide_River (18) v. Clarence_River (74) | 2394.5540 | 0.5914 | 0e+00 | 0.0160 | -5.3691 | 0.9880 | 0.0011 | 0.1379 | 0.0012 | 0.1379 | 0.0469 | 0.0430 | 0.0529 | 0.0509 | 0.0953 | 0.0460 |
| Adelaide_River (18) v. East_Alligator_River (47) | 2166.7993 | 0.0450 | 0e+00 | 0.1788 | -7.8315 | 0.1748 | 0.0011 | 0.1558 | 0.0012 | 0.1558 | 0.0734 | 0.3257 | 0.0821 | 0.2977 | 0.1449 | 0.3107 |
| Adelaide_River (18) v. South_Alligator_River (88) | 2539.1492 | 0.1459 | 0e+00 | 0.3786 | -5.5005 | 0.3846 | 0.0010 | 0.1718 | 0.0011 | 0.1718 | 0.0465 | 0.2607 | 0.0523 | 0.2498 | 0.0945 | 0.2527 |
| Clarence_River (74) v. East_Alligator_River (47) | 2713.2285 | 0.0330 | 0e+00 | 0.0010 | -4.3753 | 1.0000 | 0.0010 | 0.0310 | 0.0010 | 0.0310 | 0.0199 | 0.0150 | 0.0226 | 0.0150 | 0.0416 | 0.0150 |
| Daly_River (49) v. Sydney_Harbour (65) | 2589.7394 | 0.0160 | 0e+00 | 0.0050 | -5.0120 | 0.7043 | 0.0010 | 0.0240 | 0.0010 | 0.0230 | 0.0234 | 0.0320 | 0.0265 | 0.0320 | 0.0487 | 0.0320 |
| Fitzroy_River (3) v. Reunion (28) | 1669.1039 | 0.4336 | 1e-04 | 0.4366 | NA | NA | 0.0008 | 0.4078 | NA | NA | NA | NA | NA | NA | NA | NA |
| Seychelles (36) v. Thailand (4) | 1904.6806 | 0.0559 | 1e-04 | 0.1548 | -23.3446 | 0.0151 | 0.0008 | 0.4270 | 0.0009 | 0.4270 | 0.4708 | 0.9899 | 0.4938 | 0.9899 | 0.6558 | 0.9899 |
| South_Alligator_River (88) v. Sri_Lanka (12) | 2671.5402 | 0.0400 | 0e+00 | 0.2158 | -5.8582 | 0.1209 | 0.0008 | 0.2877 | 0.0008 | 0.2877 | 0.0684 | 0.6833 | 0.0766 | 0.6553 | 0.1357 | 0.6713 |
| Arabian_Sea (15) v. Seychelles (36) | 2295.8547 | 0.0010 | 0e+00 | 0.2717 | -8.1978 | 0.0330 | 0.0008 | 0.2747 | 0.0008 | 0.2747 | 0.0928 | 0.7572 | 0.1036 | 0.7303 | 0.1797 | 0.7393 |
| Fitzroy_River (3) v. Sydney_Harbour (65) | 2180.2845 | 0.2917 | 1e-04 | 0.2547 | NA | NA | 0.0007 | 0.4063 | NA | NA | NA | NA | NA | NA | NA | NA |
| Adelaide_River (18) v. Sri_Lanka (12) | 1703.5383 | 0.2807 | 0e+00 | 0.5115 | -12.3870 | 0.2967 | 0.0007 | 0.3576 | 0.0008 | 0.3576 | 0.1860 | 0.5215 | 0.2035 | 0.5065 | 0.3284 | 0.5115 |
| South_Africa (21) v. South_Alligator_River (88) | 2690.1292 | 0.0190 | 0e+00 | 0.3367 | -5.1108 | 0.0839 | 0.0007 | 0.2008 | 0.0008 | 0.2008 | 0.0383 | 0.4106 | 0.0432 | 0.3956 | 0.0785 | 0.4016 |
| Sydney_Harbour (65) v. Unknown (10) | 2210.5736 | 0.2937 | 1e-04 | 0.0370 | -7.7849 | 0.4006 | 0.0007 | 0.3347 | 0.0008 | 0.3347 | 0.1081 | 0.2498 | 0.1202 | 0.2328 | 0.2061 | 0.2388 |
| Adelaide_River (18) v. Daly_River (49) | 2167.7013 | 0.2138 | 0e+00 | 0.0709 | -7.3999 | 0.6933 | 0.0006 | 0.2767 | 0.0007 | 0.2767 | 0.0689 | 0.2448 | 0.0772 | 0.2488 | 0.1367 | 0.2448 |
| East_Alligator_River (47) v. Sri_Lanka (12) | 2203.9336 | 0.0120 | 0e+00 | 0.5085 | -8.6225 | 0.0440 | 0.0006 | 0.3556 | 0.0006 | 0.3556 | 0.1061 | 0.8601 | 0.1179 | 0.8422 | 0.2025 | 0.8511 |
| Arabian_Sea (15) v. Wenlock_River (24) | 2056.0306 | 0.0190 | 0e+00 | 0.2647 | -8.9214 | 0.2517 | 0.0005 | 0.3576 | 0.0005 | 0.3576 | 0.1134 | 0.5295 | 0.1261 | 0.5185 | 0.2151 | 0.5235 |
| Seychelles (36) v. South_Africa (21) | 2113.0760 | 0.0360 | 0e+00 | 0.5465 | -7.9629 | 0.2048 | 0.0004 | 0.3117 | 0.0004 | 0.3117 | 0.0733 | 0.5175 | 0.0820 | 0.5085 | 0.1447 | 0.5125 |
| Darwin_Coastal (13) v. South_Africa (21) | 1878.9222 | 0.0829 | 0e+00 | 0.8452 | -10.3985 | 0.0739 | 0.0004 | 0.4026 | 0.0004 | 0.4026 | 0.1469 | 0.6294 | 0.1623 | 0.6024 | 0.2696 | 0.6144 |
| East_Alligator_River (47) v. Unknown (10) | 2062.6184 | 0.0599 | 0e+00 | 0.3197 | -9.5133 | 0.1039 | 0.0003 | 0.4056 | 0.0003 | 0.4046 | 0.1337 | 0.4905 | 0.1477 | 0.4525 | 0.2482 | 0.4705 |
| East_Alligator_River (47) v. Sydney_Harbour (65) | 2454.4485 | 0.2647 | 0e+00 | 0.3347 | -5.3022 | 0.9960 | 0.0001 | 0.3616 | 0.0002 | 0.3616 | 0.0248 | 0.3117 | 0.0280 | 0.3177 | 0.0515 | 0.3157 |
| Papua_New_Guinea (9) v. Thailand (4) | 1281.3563 | 0.1409 | 1e-04 | 0.2687 | 80.2821 | 0.0180 | 0.0001 | 0.4555 | 0.0001 | 0.4555 | 1.3270 | 0.9860 | 1.3028 | 0.9860 | 1.1301 | 0.9860 |
| Thailand (4) v. Unknown (10) | 1305.2689 | 0.1069 | 1e-04 | 0.2857 | 160.1180 | 0.0415 | 0.0000 | 0.4577 | 0.0000 | 0.4577 | 1.1458 | 0.9615 | 1.1355 | 0.9615 | 1.0631 | 0.9615 |
| Seychelles (36) v. Sri_Lanka (12) | 2037.9513 | 0.0330 | 0e+00 | 0.1768 | -9.4906 | 0.1908 | 0.0000 | 0.5165 | 0.0000 | 0.5165 | 0.1221 | 0.8322 | 0.1352 | 0.8222 | 0.2293 | 0.8282 |
| Daly_River (49) v. East_Alligator_River (47) | 2356.7982 | 0.2807 | 0e+00 | 0.1838 | -5.8189 | 0.8212 | 0.0000 | 0.4835 | 0.0000 | 0.4835 | 0.0305 | 0.4685 | 0.0345 | 0.4705 | 0.0631 | 0.4695 |
| Adelaide_River (18) v. Sydney_Harbour (65) | 2302.3892 | 0.2438 | 0e+00 | 0.8442 | -6.5338 | 0.6104 | 0.0000 | 0.4945 | 0.0000 | 0.4945 | 0.0573 | 0.5644 | 0.0644 | 0.5704 | 0.1151 | 0.5694 |
| Darwin_Coastal (13) v. Roper_River (9) | 1574.5441 | 0.2408 | 1e-04 | 0.1938 | -17.1266 | 0.9411 | 0.0000 | 0.5075 | 0.0000 | 0.5075 | 0.3088 | 0.0819 | 0.3316 | 0.0829 | 0.4893 | 0.0829 |
| East_Alligator_River (47) v. South_Alligator_River (88) | 2557.4033 | 0.4945 | 0e+00 | 0.4216 | -4.5998 | 0.8761 | -0.0001 | 0.5544 | -0.0001 | 0.5544 | 0.0191 | 0.5105 | 0.0216 | 0.5245 | 0.0399 | 0.5155 |
| Clarence_River (74) v. Fitzroy_River (3) | 2109.1963 | 0.8302 | 2e-04 | 0.0809 | NA | NA | -0.0001 | 0.4757 | -0.0001 | 0.4757 | NA | NA | NA | NA | NA | NA |
| Fitzroy_River (3) v. Roper_River (9) | 1221.3031 | 0.3287 | 2e-04 | 0.4076 | NA | NA | -0.0002 | 0.4633 | -0.0002 | 0.4501 | NA | NA | NA | NA | NA | NA |
| Darwin_Coastal (13) v. Thailand (4) | 1455.0609 | 0.3127 | 1e-04 | 0.1688 | -93.7242 | 0.2302 | -0.0002 | 0.4900 | -0.0002 | 0.4900 | 0.8097 | 0.8070 | 0.8213 | 0.8080 | 0.9013 | 0.8080 |
| Daly_River (49) v. South_Africa (21) | 2295.0260 | 0.0380 | 0e+00 | 0.5534 | -6.8820 | 0.3167 | -0.0002 | 0.5684 | -0.0002 | 0.5684 | 0.0577 | 0.6154 | 0.0648 | 0.6104 | 0.1158 | 0.6134 |
| South_Africa (21) v. Sri_Lanka (12) | 1795.5717 | 0.2657 | 0e+00 | 0.5445 | -11.2577 | 0.4076 | -0.0003 | 0.5365 | -0.0003 | 0.5365 | 0.1632 | 0.6114 | 0.1795 | 0.6054 | 0.2946 | 0.6064 |
| East_Alligator_River (47) v. Thailand (4) | 2065.5141 | 0.0679 | 1e-04 | 0.5784 | -18.6980 | 0.0181 | -0.0007 | 0.5707 | -0.0007 | 0.5707 | 0.4088 | 0.9879 | 0.4319 | 0.9879 | 0.5967 | 0.9879 |
| Fitzroy_River (3) v. South_Africa (21) | 1557.7934 | 0.4975 | 1e-04 | 0.5664 | NA | NA | -0.0016 | 0.6127 | NA | NA | NA | NA | NA | NA | NA | NA |
| Arabian_Sea (15) v. Sri_Lanka (12) | 1725.5932 | 0.6693 | 0e+00 | 0.5984 | -12.0014 | 0.4735 | -0.0017 | 0.8382 | -0.0019 | 0.8382 | 0.1923 | 0.7862 | 0.2104 | 0.7922 | 0.3378 | 0.7902 |
| Adelaide_River (18) v. Thailand (4) | 1511.7822 | 0.3177 | 1e-04 | 0.7493 | -54.5834 | 0.0431 | -0.0018 | 0.6240 | -0.0019 | 0.6240 | 0.6965 | 0.9709 | 0.7131 | 0.9709 | 0.8309 | 0.9709 |
| Arabian_Sea (15) v. Fitzroy_River (3) | 1390.8797 | 0.8531 | 1e-04 | 0.2388 | NA | NA | -0.0021 | 0.6133 | -0.0023 | 0.6133 | NA | NA | NA | NA | NA | NA |
| South_Alligator_River (88) v. Thailand (4) | 2650.8199 | 0.0519 | 1e-04 | 0.7502 | -9.5226 | 0.0245 | -0.0023 | 0.6957 | -0.0024 | 0.6957 | 0.2525 | 0.9949 | 0.2732 | 0.9949 | 0.4197 | 0.9949 |
| Roper_River (9) v. Trinity_inlet (4) | 1320.9973 | 0.1029 | 1e-04 | 0.8222 | 80.2456 | 0.0721 | -0.0023 | 0.6373 | -0.0025 | 0.6333 | 1.3145 | 0.9540 | 1.2904 | 0.9540 | 1.1255 | 0.9540 |
| Clarence_River (74) v. Thailand (4) | 2461.8605 | 0.1998 | 1e-04 | 0.6374 | -9.2000 | 0.2889 | -0.0023 | 0.6767 | -0.0025 | 0.6767 | 0.2478 | 0.9030 | 0.2688 | 0.9111 | 0.4140 | 0.9061 |
| Daly_River (49) v. Thailand (4) | 2171.0507 | 0.0320 | 1e-04 | 0.5914 | -16.2078 | 0.0374 | -0.0023 | 0.7180 | -0.0025 | 0.7180 | 0.3737 | 0.9838 | 0.3970 | 0.9848 | 0.5610 | 0.9838 |
| Thailand (4) v. Wenlock_River (24) | 1746.8600 | 0.2418 | 1e-04 | 0.4366 | -29.7036 | 0.0594 | -0.0024 | 0.6727 | -0.0026 | 0.6754 | 0.5430 | 0.9638 | 0.5648 | 0.9658 | 0.7180 | 0.9658 |
| Fitzroy_River (3) v. Wenlock_River (24) | 1498.1683 | 0.8242 | 1e-04 | 0.2368 | NA | NA | -0.0031 | 0.6519 | NA | NA | NA | NA | NA | NA | NA | NA |
| Reunion (28) v. Thailand (4) | 1844.1871 | 0.0529 | 1e-04 | 0.4715 | -28.9871 | 0.0062 | -0.0032 | 0.6958 | -0.0034 | 0.6948 | 0.5317 | 0.9969 | 0.5535 | 0.9969 | 0.7085 | 0.9969 |
| Sydney_Harbour (65) v. Thailand (4) | 2327.3946 | 0.0579 | 1e-04 | 0.8611 | -12.8716 | 0.0262 | -0.0041 | 0.8739 | -0.0044 | 0.8739 | 0.3168 | 0.9939 | 0.3392 | 0.9960 | 0.4982 | 0.9960 |
| Adelaide_River (18) v. Fitzroy_River (3) | 1369.5109 | 0.8232 | 1e-04 | 0.5584 | NA | NA | -0.0047 | 0.7308 | -0.0051 | 0.7318 | NA | NA | NA | NA | NA | NA |
| Sri_Lanka (12) v. Thailand (4) | 1356.9701 | 0.5075 | 1e-04 | 0.8801 | -256.1097 | 0.1510 | -0.0050 | 0.8621 | -0.0054 | 0.8621 | 0.9218 | 0.8620 | 0.9268 | 0.8630 | 0.9619 | 0.8630 |
| South_Africa (21) v. Thailand (4) | 1572.0260 | 0.5405 | 1e-04 | 0.5285 | -38.5533 | 0.0212 | -0.0072 | 0.9679 | -0.0078 | 0.9679 | 0.6106 | 0.9889 | 0.6304 | 0.9909 | 0.7705 | 0.9889 |
| Roper_River (9) v. Thailand (4) | 1261.5201 | 0.4156 | 1e-04 | 0.7303 | 81.4655 | 0.0153 | -0.0074 | 0.8831 | -0.0080 | 0.8831 | 1.3158 | 0.9878 | 1.2921 | 0.9878 | 1.1261 | 0.9878 |

Table 46: Pairwise Fst for 3416 DArTcap loci between the IWP sites *(continued)*

| | Chi2 | Chi2.p.val | D | D.p.val | Fis | Fis.p.val | Fst | Fst.p.val | F’st | F’st.p.val | Gst | Gst.p.val | G’st | G’st.p.val | G”st | G”st.p.val |
|---|---|---|---|---|---|---|---|---|---|---|---|---|---|---|---|---|
| Arabian_Sea (15) v. Thailand (4) | 1433.1283 | 0.9071 | 1e-04 | 0.7912 | -54.8151 | 0.1910 | -0.0079 | 0.9750 | -0.0085 | 0.9750 | 0.7041 | 0.8640 | 0.7207 | 0.8670 | 0.8361 | 0.8660 |
| Mozambique (11) v. Thailand (4) | 1247.1550 | 0.8891 | 1e-04 | 0.9630 | 157.8144 | 0.0075 | -0.0084 | 0.9346 | -0.0091 | 0.9281 | 1.1490 | 0.9938 | 1.1385 | 0.9938 | 1.0645 | 0.9938 |
| Fitzroy_River (3) v. Mozambique (11) | 1230.1591 | 0.7792 | 1e-04 | 0.9800 | NA | NA | -0.0097 | 0.8419 | NA | NA | NA | NA | NA | NA | NA | NA |
| Fitzroy_River (3) v. Seychelles (36) | 1808.8073 | 0.1868 | 1e-04 | 0.1908 | NA | NA | NA | NA | NA | NA | NA | NA | NA | NA | NA | NA |
| Fitzroy_River (3) v. Towns_River (10) | 1249.3455 | 0.2168 | 2e-04 | 0.2737 | NA | NA | NA | NA | NA | NA | NA | NA | NA | NA | NA | NA |
| Fitzroy_River (3) v. Unknown (10) | 1211.0390 | 0.4166 | 2e-04 | 0.2687 | NA | NA | NA | NA | NA | NA | NA | NA | NA | NA | NA | NA |
| Fitzroy_River (3) v. Urauchi_River (35) | 2817.7795 | 0.0010 | 3e-04 | 0.0060 | NA | NA | NA | NA | NA | NA | NA | NA | NA | NA | NA | NA |

## 11.8 PCA

```
pca1 <- adegenet::glPca(BS.dartcap.IWP.gl, nf = 5, parallel = TRUE,
                        n.cores = parallel::detectCores() - 1)
save(pca1, file = "DArTcap_IWP_PCA.Rdata")
```

```
load("DArTcap_IWP_PCA.Rdata")

BS.pca.scores <- as.data.frame(pca1$scores)
BS.pca.scores$pop <- pop(BS.dartcap.IWP.gl)
cols <- adegenet::funky(nPop(BS.dartcap.IWP.gl))


p <-
  ggplot2::ggplot(BS.pca.scores, ggplot2::aes(x = PC1, y = PC2, colour = pop)) +
  ggplot2::geom_point(size = 2) + ggplot2::scale_color_manual(values = cols) +
  ggplot2::geom_hline(yintercept = 0) + ggplot2::geom_vline(xintercept = 0) +
  ggplot2::theme_minimal() +
  ggplot2::theme(
    axis.text = ggplot2::element_text(size = 5),
    axis.title.x = ggplot2::element_text(size = 10),
    axis.title.y = ggplot2::element_text(size = 10),
    legend.text = ggplot2::element_text(size = 7)
  )
p
```

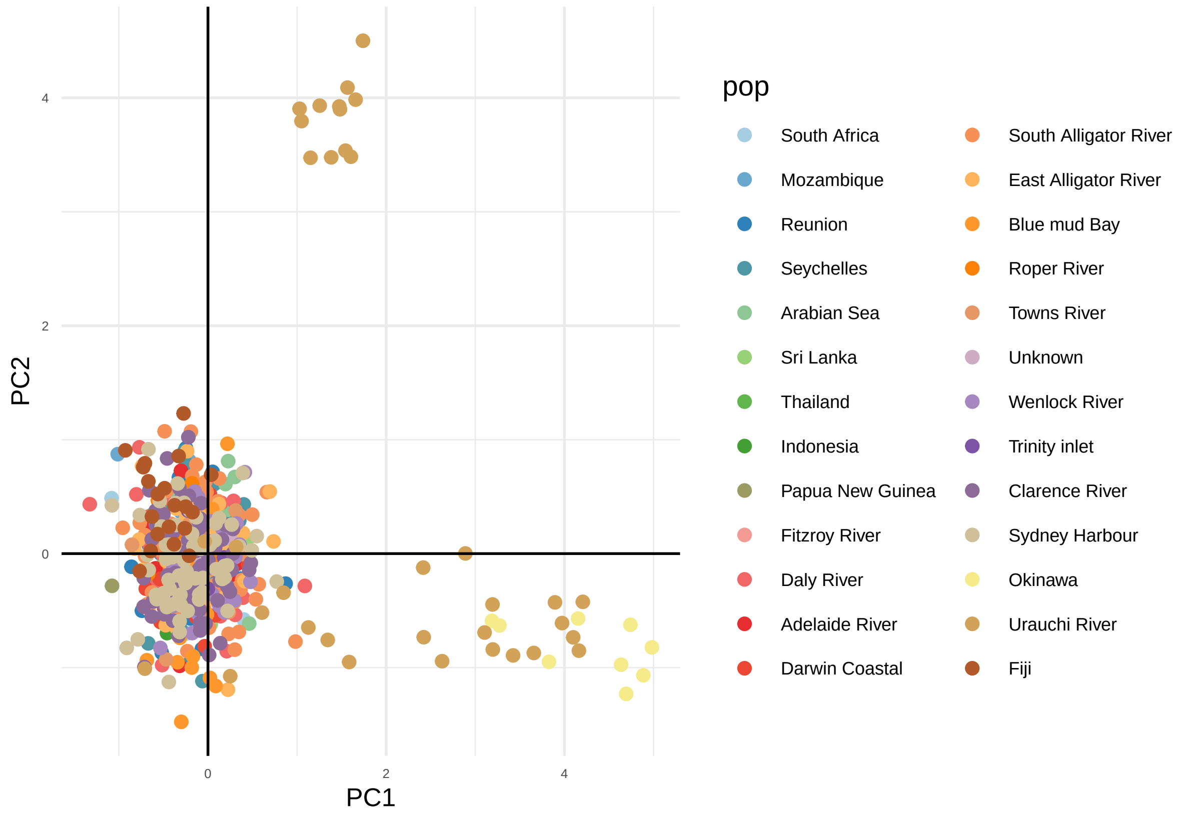


```
ggplot2::ggsave(p, filename = "DArTcap_IWP_PCA1.png", width = 30,
                height = 15,units = "cm")


p <-
  ggplot2::ggplot(BS.pca.scores, ggplot2::aes(x = PC1, y = PC3, colour = pop)) +
```

```
  ggplot2::geom_point(size = 2) + ggplot2::scale_color_manual(values = cols) +
  ggplot2::geom_hline(yintercept = 0) + ggplot2::geom_vline(xintercept = 0) +
  ggplot2::theme_minimal() +
  ggplot2::theme(
    axis.text = ggplot2::element_text(size = 5),
    axis.title.x = ggplot2::element_text(size = 10),
    axis.title.y = ggplot2::element_text(size = 10),
    legend.text = ggplot2::element_text(size = 7)
  )
p
```

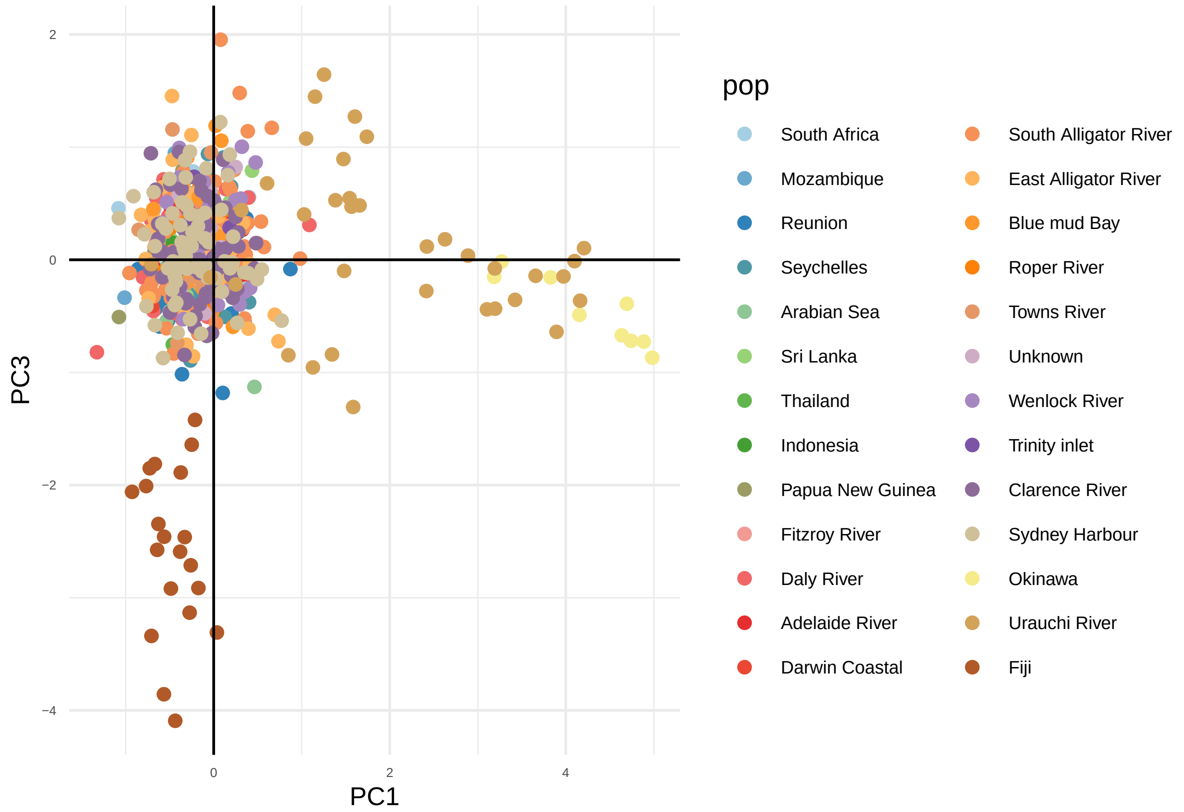


```
ggplot2::ggsave(p, filename = "DArTcap_IWP_PCA2.png", width = 30,
                height = 15,units = "cm")

p <-
  ggplot2::ggplot(BS.pca.scores, ggplot2::aes(x = PC2, y = PC3, colour = pop)) +
  ggplot2::geom_point(size = 2) + ggplot2::scale_color_manual(values = cols) +
  ggplot2::geom_hline(yintercept = 0) + ggplot2::geom_vline(xintercept = 0) +
  ggplot2::theme_minimal() +
  ggplot2::theme(
    axis.text = ggplot2::element_text(size = 5),
    axis.title.x = ggplot2::element_text(size = 10),
    axis.title.y = ggplot2::element_text(size = 10),
    legend.text = ggplot2::element_text(size = 7)
  )
p
```

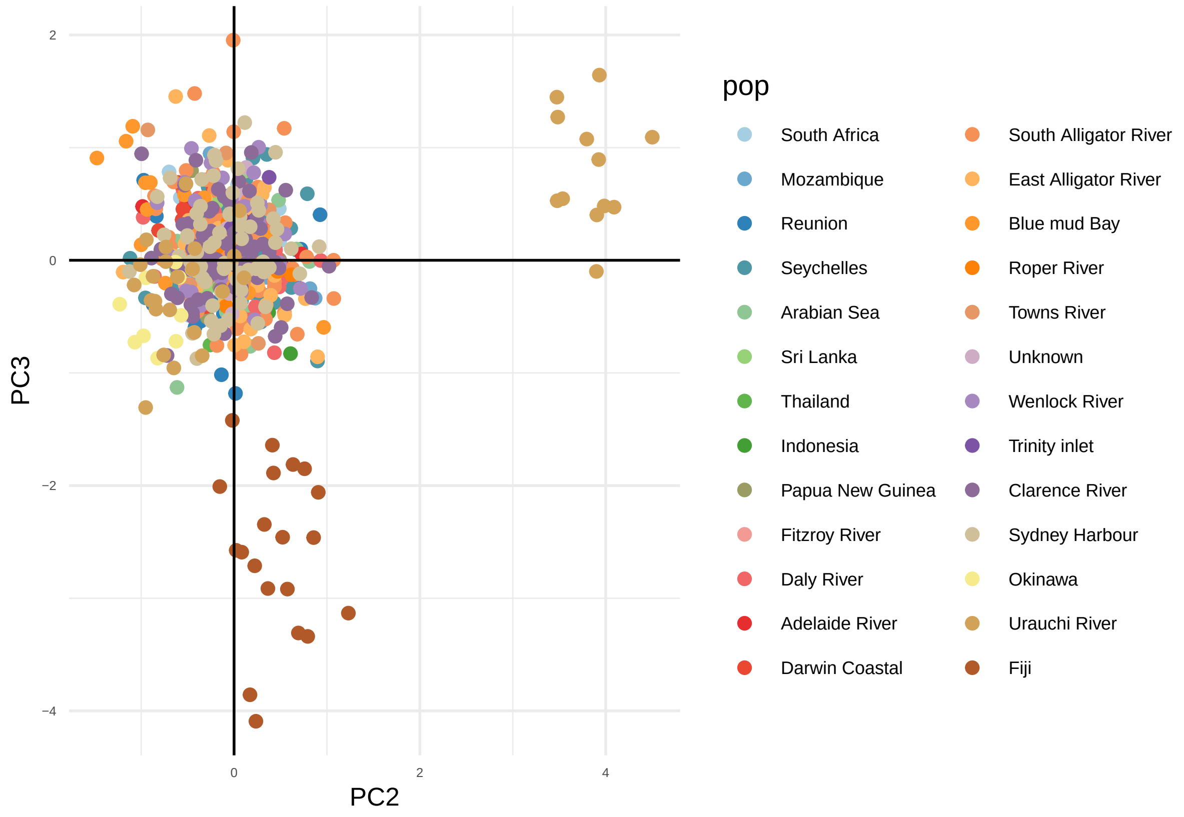


```
ggplot2::ggsave(p, filename = "DArTcap_IWP_PCA3.png", width = 30,
                height = 15,units = "cm")


p <-
  ggplot2::ggplot(BS.pca.scores, ggplot2::aes(x = PC3, y = PC4, colour = pop)) +
  ggplot2::geom_point(size = 2) + ggplot2::scale_color_manual(values = cols) +
  ggplot2::geom_hline(yintercept = 0) + ggplot2::geom_vline(xintercept = 0) +
  ggplot2::theme_minimal() +
  ggplot2::theme(
    axis.text = ggplot2::element_text(size = 5),
    axis.title.x = ggplot2::element_text(size = 10),
    axis.title.y = ggplot2::element_text(size = 10),
    legend.text = ggplot2::element_text(size = 7)
  )
p
```

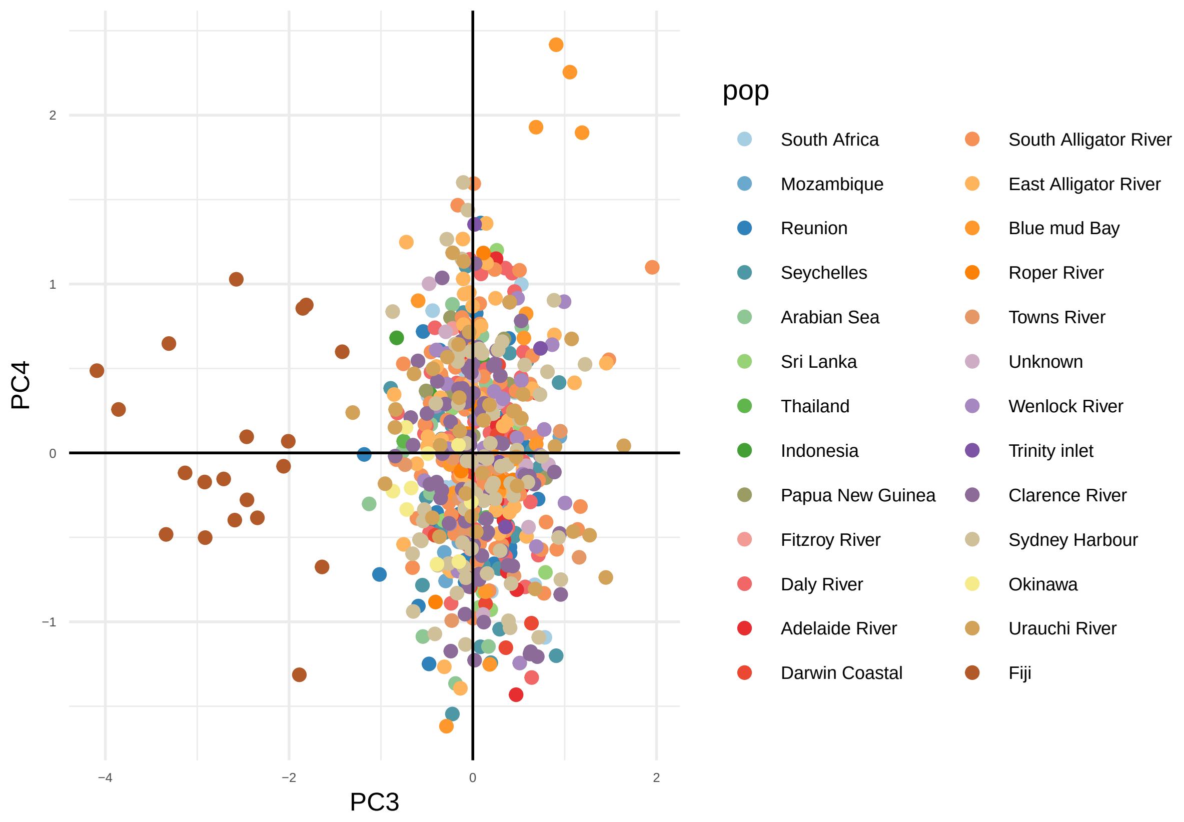


```
ggplot2::ggsave(p, filename = "DArTcap_IWP_PCA4.png", width = 30,
                height = 15,units = "cm")

p <-
  ggplot2::ggplot(BS.pca.scores, ggplot2::aes(x = PC3, y = PC5, colour = pop)) +
  ggplot2::geom_point(size = 2) + ggplot2::scale_color_manual(values = cols) +
  ggplot2::geom_hline(yintercept = 0) + ggplot2::geom_vline(xintercept = 0) +
  ggplot2::theme_minimal() +
  ggplot2::theme(
    axis.text = ggplot2::element_text(size = 5),
    axis.title.x = ggplot2::element_text(size = 10),
    axis.title.y = ggplot2::element_text(size = 10),
    legend.text = ggplot2::element_text(size = 7)
  )
p
```

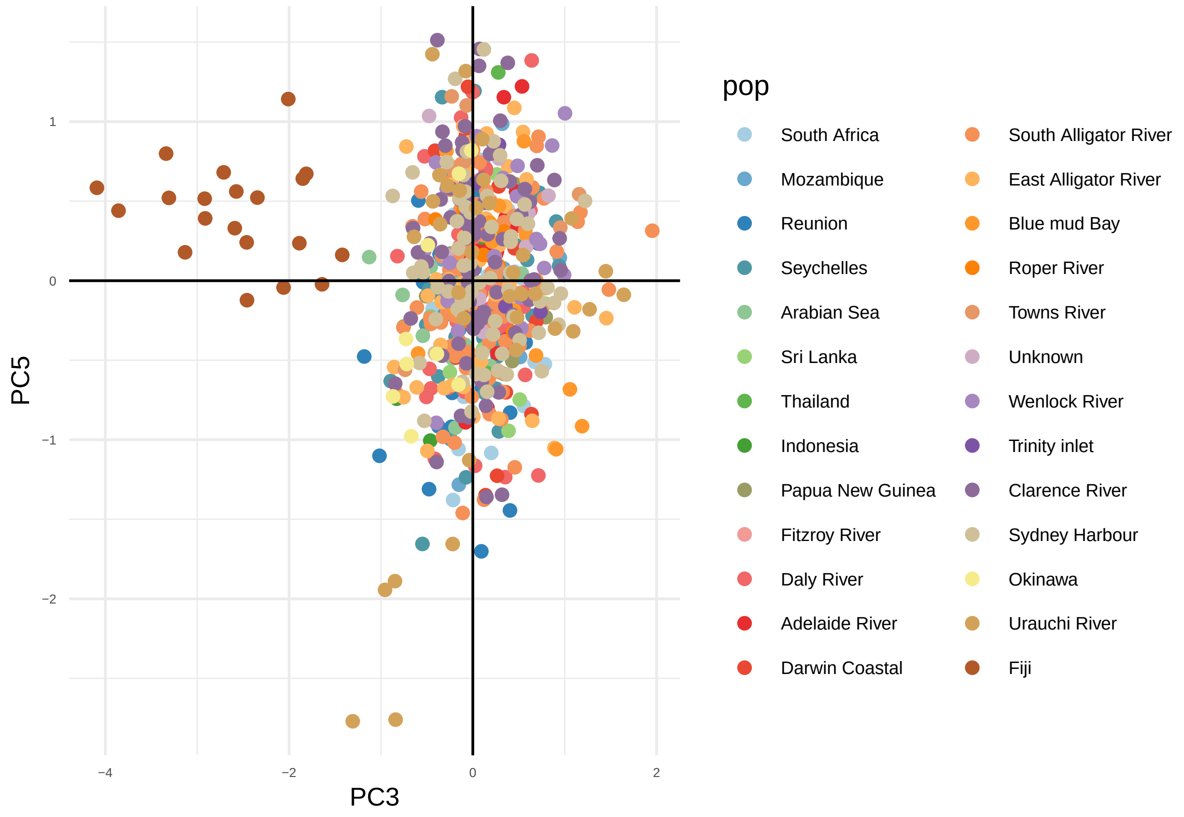


```
ggplot2::ggsave(p, filename = "DArTcap_IWP_PCA5.png", width = 30,
                height = 15,units = "cm")
```

## 11.9 DAPC

### 11.9.1 Optimal number of clusters with neutral markers

```
set.seed(124)
grp <-
  adegenet::find.clusters(
    BS.dartcap.IWP.gl,
    max.n.clust = 26,
    n.pca = adegenet::nInd(BS.dartcap.IWP.gl) / 3,
    stat = "BIC",
    parallel = TRUE,
    n.cores = parallel::detectCores() - 1
  )

save(grp, file = "DArTcap_IWP_grp.Rdata")
```

```
load("DArTcap_IWP_grp.Rdata")

y <- as.numeric(grp$Kstat)
x <- 1:26
data <- data.frame(x,y,stringsAsFactors = F)
plot <- ggplot2::ggplot(data, ggplot2::aes(x,y)) +
  ggplot2::geom_point(size = 5, shape = 1, color = "blue") +
  ggplot2::geom_line(size = 1,color = "blue") +
  ggplot2::scale_x_continuous(name = waiver(),
                              breaks = seq(from = 0,
                                           to = nrow(BS.dartcap.IWP.gl) - 1,
```

```
                                                        by = 1)) +
  ggplot2::labs(subtitle = "",
                y = "Bayesian Information Criterion",
                x = "Number of clusters",
                title = "",
                caption = "") +
  ggplot2::theme_classic() +
  ggplot2::theme(
    axis.text = ggplot2::element_text(size = 5),
    axis.title.x = ggplot2::element_text(size = 20,vjust = 0, hjust = 0.5),
    axis.title.y = ggplot2::element_text(size = 20,vjust = 2, hjust = 0.5))
print(plot)
```

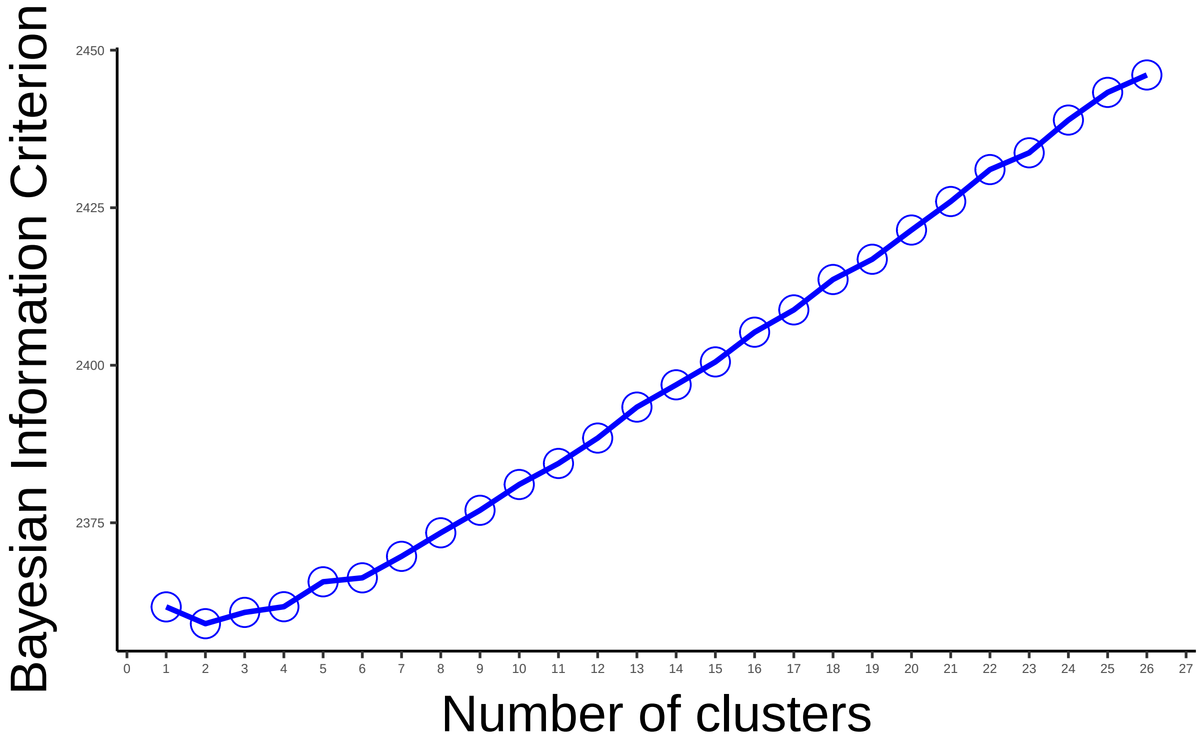


```
ggplot2::ggsave(plot, filename = "DArTcap_IWP_grp.png", width = 30,
                height = 15,units = "cm")
```

### 11.9.2 Cross-validation to identify the optimal number of PCs

Uses a training - holdout set of individuals to check how reliable individuals can be assigned.

```
set.seed(124)
x <- BS.dartcap.IWP.gl

xval <-
  adegenet::xvalDapc(
  tab(x, NA.method = "mean"),
  adegenet::pop(x),
  n.pca = seq(1, adegenet::nInd(x), by = 50),
  training.set = 0.9,
  result = "groupMean",
  center = TRUE,
  scale = FALSE,
```

```
  n.rep = 1000,
  parallel = "multicore",
  ncpus = 1,
  xval.plot = TRUE
  )
print(xval[2:6])
PC <- as.numeric(xval$`Number of PCs Achieving Lowest MSE`)

#refine the analysis
set.seed(124)
png(file="DArTcap_IWP_xval.png")
xval <-
  adegenet::xvalDapc(
  tab(x, NA.method = "mean"),
  adegenet::pop(x),
  n.pca = (PC - 15):(PC + 15),
  n.rep = 1000,
  parallel = "multicore",
  ncpus = 1,
  xval.plot = TRUE
  )
dev.off()
save(xval, file = "DArTcap_IWP_xval.rdata")
```

```
load("DArTcap_IWP_xval.rdata")
print(xval[2:6])
PC <- as.numeric(xval$`Number of PCs Achieving Lowest MSE`)
```

```
## $`Median and Confidence Interval for Random Chance`
##       2.5%        50%      97.5%
## 0.02438735 0.03734027 0.05508623
##
## $`Mean Successful Assignment by Number of PCs of PCA`
##       186       187       188       189       190       191       192       193
## 0.2324804 0.2306034 0.2321176 0.2329844 0.2300221 0.2284677 0.2318179 0.2328035
##       194       195       196       197       198       199       200       201
## 0.2288689 0.2310550 0.2324531 0.2295151 0.2295611 0.2286921 0.2269646 0.2322773
##       202       203       204       205       206       207       208       209
## 0.2295792 0.2298527 0.2290045 0.2298239 0.2280632 0.2260452 0.2277085 0.2286509
##       210       211       212       213       214       215       216
## 0.2277546 0.2264222 0.2281876 0.2280022 0.2292683 0.2289469 0.2295428
##
## $`Number of PCs Achieving Highest Mean Success`
## [1] "189"
##
## $`Root Mean Squared Error by Number of PCs of PCA`
##       186       187       188       189       190       191       192       193
## 0.7680637 0.7699528 0.7684520 0.7675715 0.7705111 0.7721376 0.7687126 0.7677602
##       194       195       196       197       198       199       200       201
## 0.7716703 0.7695454 0.7680836 0.7710186 0.7710227 0.7718648 0.7735771 0.7682334
##       202       203       204       205       206       207       208       209
## 0.7709948 0.7707494 0.7715608 0.7706960 0.7724861 0.7745113 0.7728612 0.7719175
##       210       211       212       213       214       215       216
## 0.7728060 0.7741488 0.7723893 0.7725922 0.7712791 0.7716179 0.7710302
##
## $`Number of PCs Achieving Lowest MSE`
## [1] "189"
```

```r
dapc.all.object.names <- c()
for (K in 2:10) {
  set.seed(124)
  grp <- adegenet::find.clusters(BS.dartcap.IWP.gl, n.clust = K, n.pca = PC)
  set.seed(124)
  dapc.all.neutral <- adegenet::dapc(BS.dartcap.IWP.gl, grp$grp, n.da = K - 1,
                                     n.pca = PC)
  assign(paste0("dapc.all.", K),value = dapc.all.neutral)
  dapc.all.object.names <- c(dapc.all.object.names,
                                     paste0("dapc.all.", K))
}
save(list = dapc.all.object.names, dapc.all.object.names,
     file = "DArTcap_IWP_DAPC.Rdata")
```

### 11.9.3 DAPC barplot

Group individuals according to DAPC posterior membership.

```r
load("DArTcap_IWP_DAPC.Rdata")
for (K in 2:10) {
  colour <- colours.prior[1:K]
  dapc <- get(dapc.all.object.names[K - 1])
  post <- as.matrix(dapc$posterior)
  colnames(post) <- paste0("Group", 1:nlevels(dapc$grp))
  locations <- BS.dartcap.IWP.gl$pop

  plot.dapc.FDD(x = post,
                locations = locations,
                colour = colour,
                region.lwd = 1,
                plotTitle = paste0("DAPC: all Bull Sharks - subset Australia\n for K=",
                                   K, " & PC=", PC, sep = ""))
  dev.print(
    device = png,
    file = paste0("DArTcap_IWP_DAPC_barplot_K", K, ".png"),
    res = 300,
    width = 30,
    height = 15,
    units = "cm")
}
```

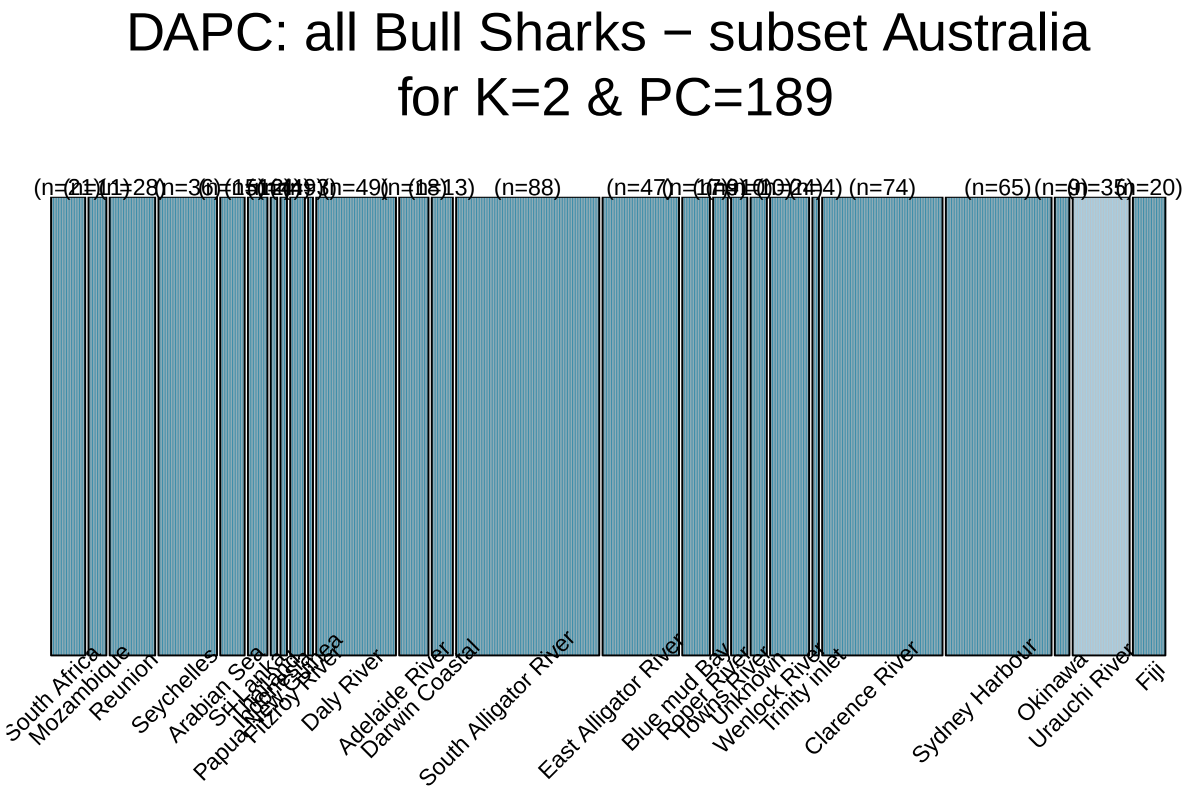
DAPC: all Bull Sharks − subset Australia
for K=2 & PC=189
(n=88)
(n=74)
(n=65)
(n=20)
South Africa
Mozambique
Reunion
Seychelles
Arabian Sea
Sri Lanka
Papua New Guinea
Fitzroy River
Daly River
Adelaide River
Darwin Coastal
South Alligator River
East Alligator River
Blue mud Bay
Roper River
Towns River
Unknown
Wenlock River
Trinity Inlet
Clarence River
Sydney Harbour
Okinawa
Urauchi River
Fiji

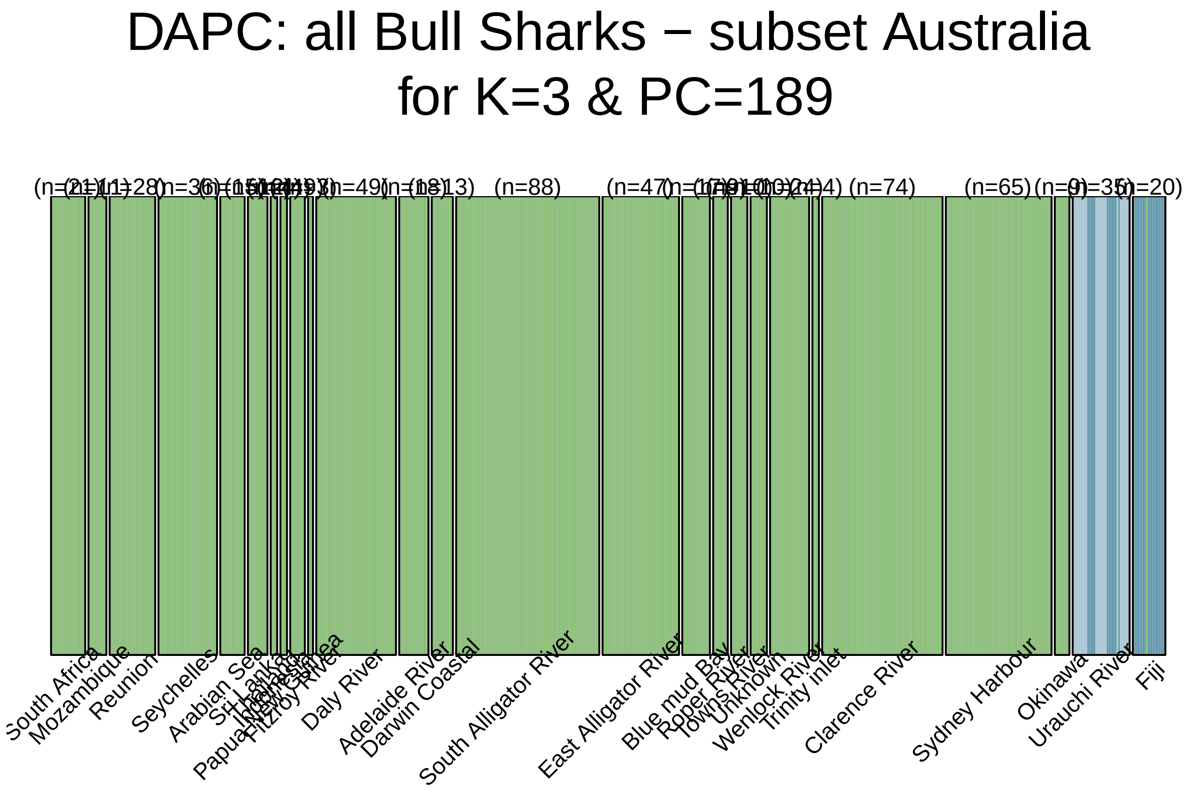
DAPC: all Bull Sharks − subset Australia
for K=3 & PC=189
(n=88)
(n=74)
(n=65)
(n=20)
South Africa
Mozambique
Reunion
Seychelles
Arabian Sea
Sri Lanka
Papua New Guinea
Fitzroy River
Daly River
Adelaide River
Darwin Coastal
South Alligator River
East Alligator River
Blue mud Bay
Roper River
Towns River
Unknown
Wenlock River
Trinity Inlet
Clarence River
Sydney Harbour
Okinawa
Urauchi River
Fiji

DAPC: all Bull Sharks − subset Australia
for K=4 & PC=189

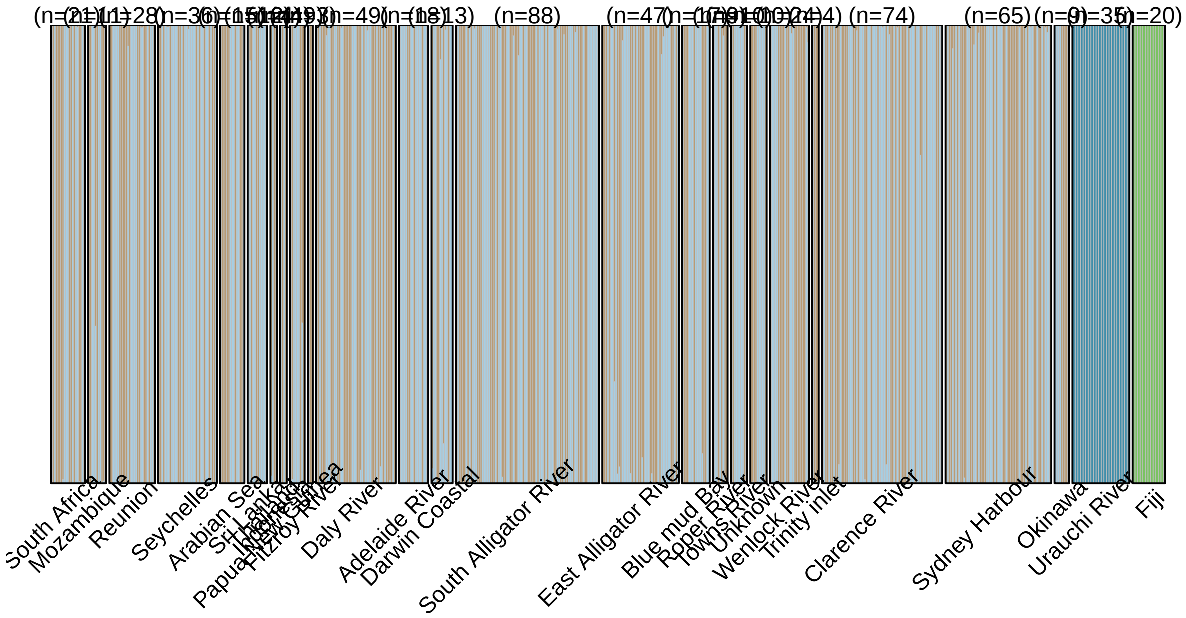


DAPC: all Bull Sharks − subset Australia
for K=5 & PC=189

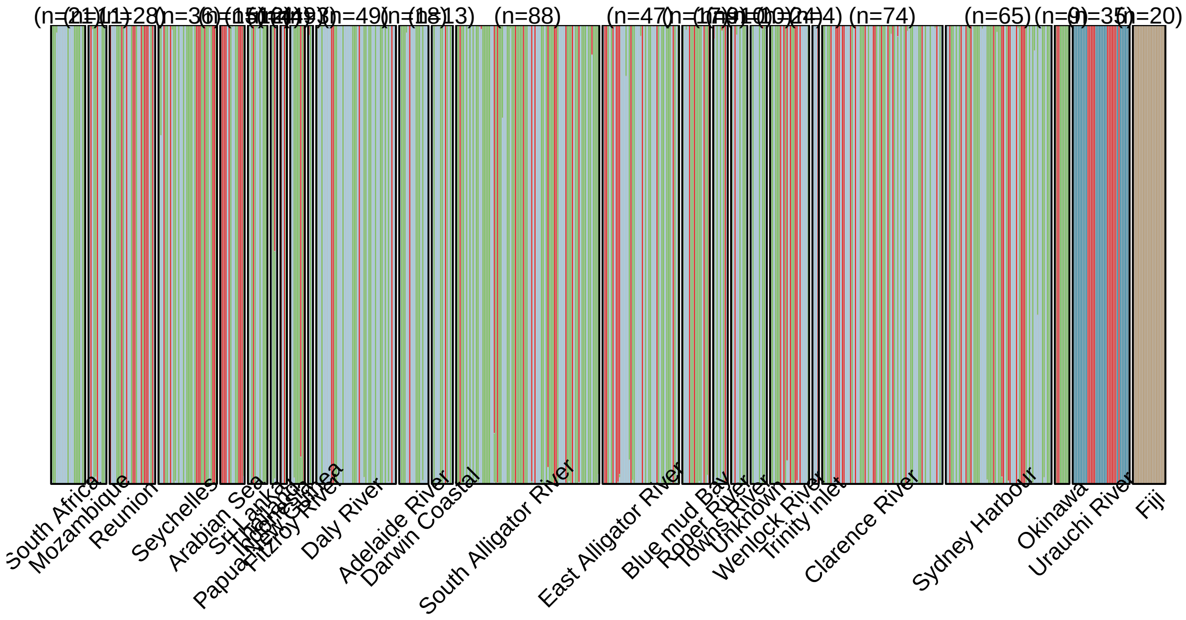

# DAPC: all Bull Sharks − subset Australia for K=6 & PC=189

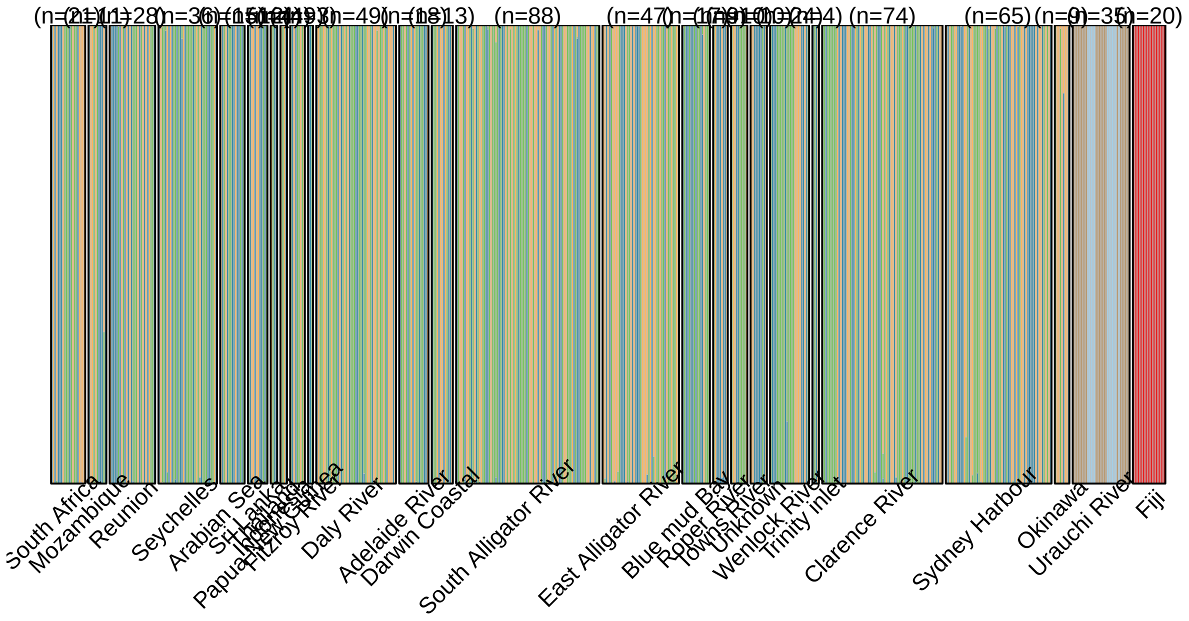


# DAPC: all Bull Sharks − subset Australia for K=7 & PC=189

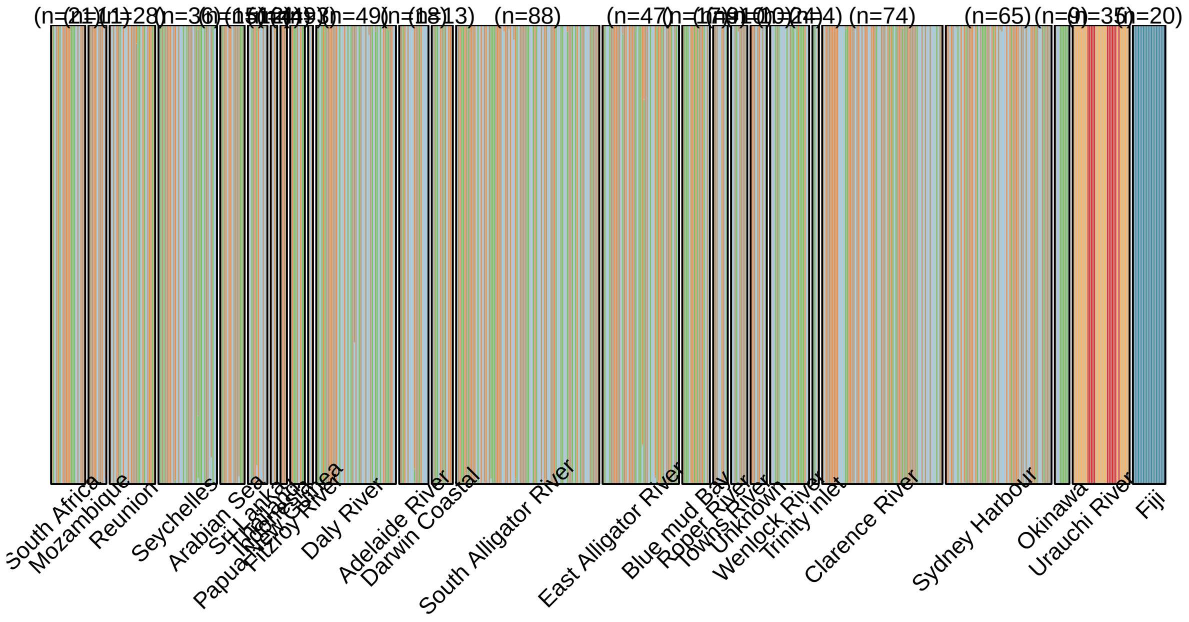

DAPC: all Bull Sharks − subset Australia
for K=8 & PC=189

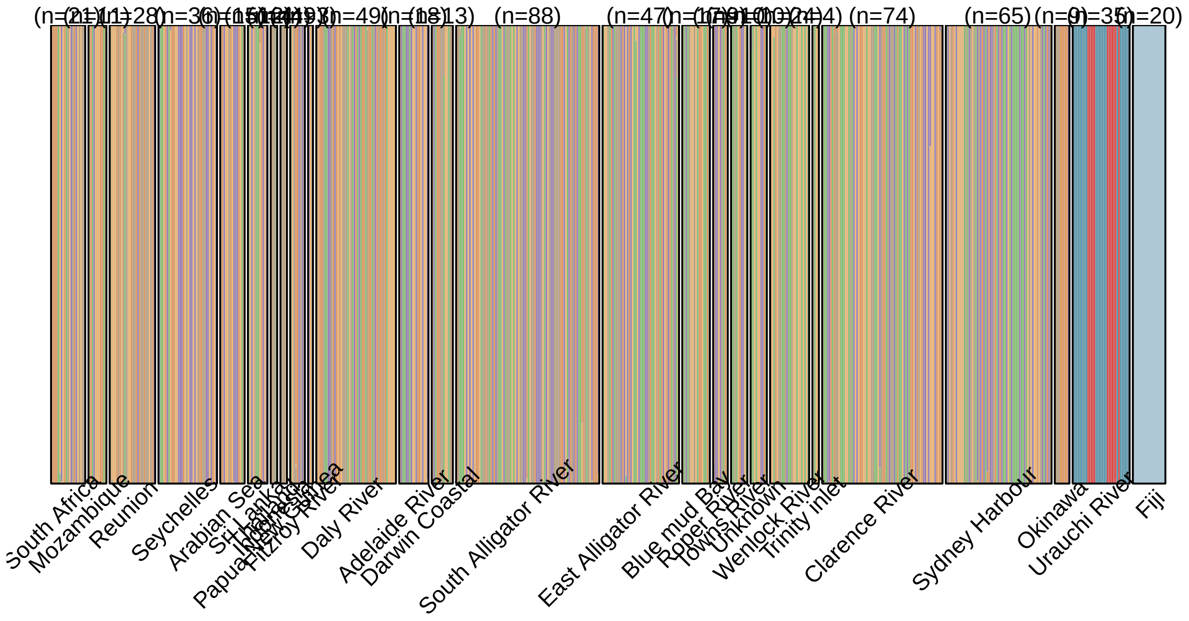


DAPC: all Bull Sharks − subset Australia
for K=9 & PC=189

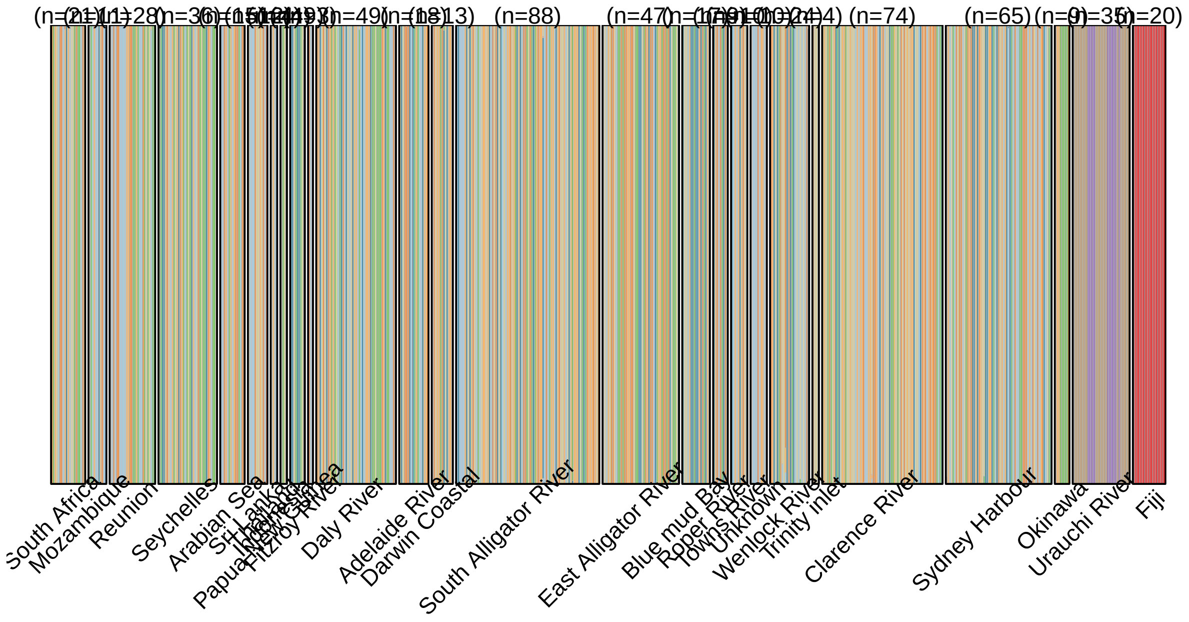

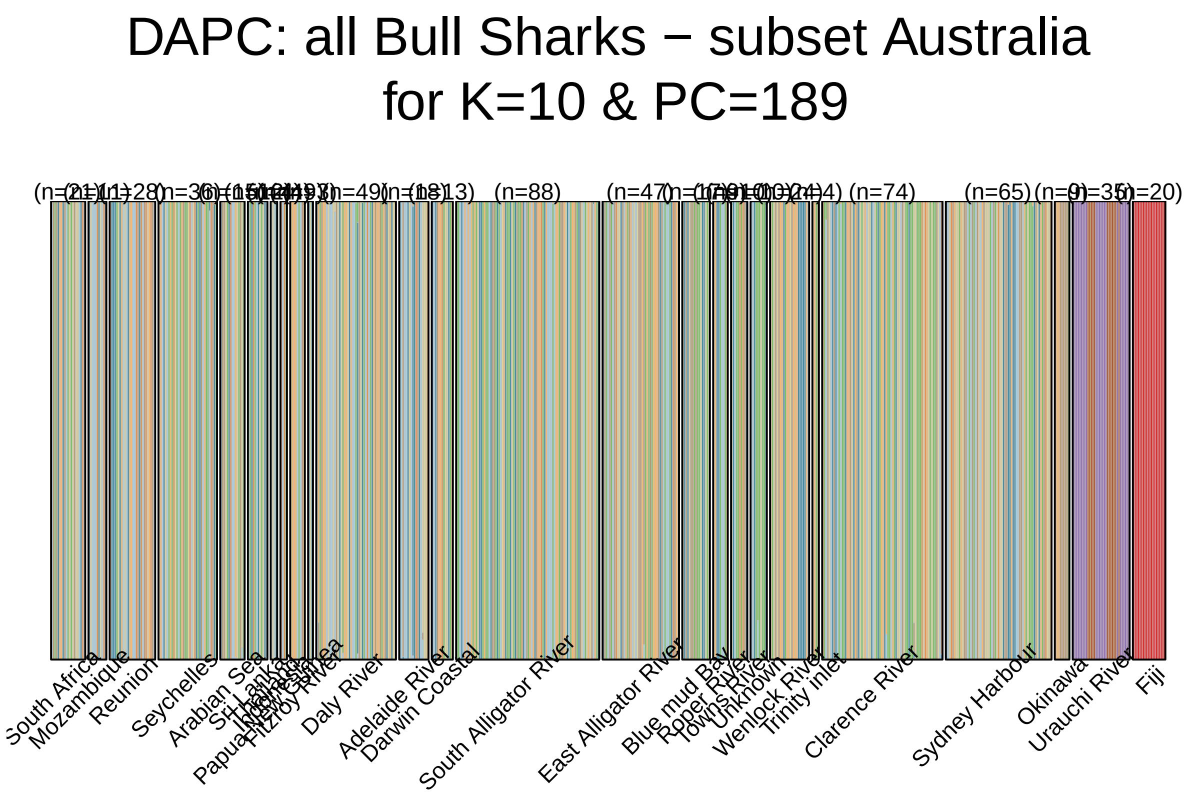


### 11.9.4 DAPC scatterplot

```
load("DArTcap_IWP_DAPC.Rdata")

for (K in 3:10) {
  dapc <- get(dapc.all.object.names[K - 1])
  if (K == 2) {
    posi.leg <- "bottomright"
  } else {posi.leg <- "bottomleft"}
  dapc.plot <- ade4::scatter(dapc, grp = pop(BS.dartcap.IWP.gl), cex = 2,
                             legend = TRUE, col = colours.26,clabel = FALSE,
                             posi.leg = posi.leg, scree.pca = FALSE,
                             posi.pca = "topleft", cleg = 0.5, xax = 1,
                             yax = 2, inset.solid = 0.3)
  dev.print(
    device = png,
    file = paste0("DArTcap_IWP_DAPC_scatterplot_K", K, ".png"),
    width = 30,
    height = 15,
    units = "cm",
    res = 300
  )
}
```

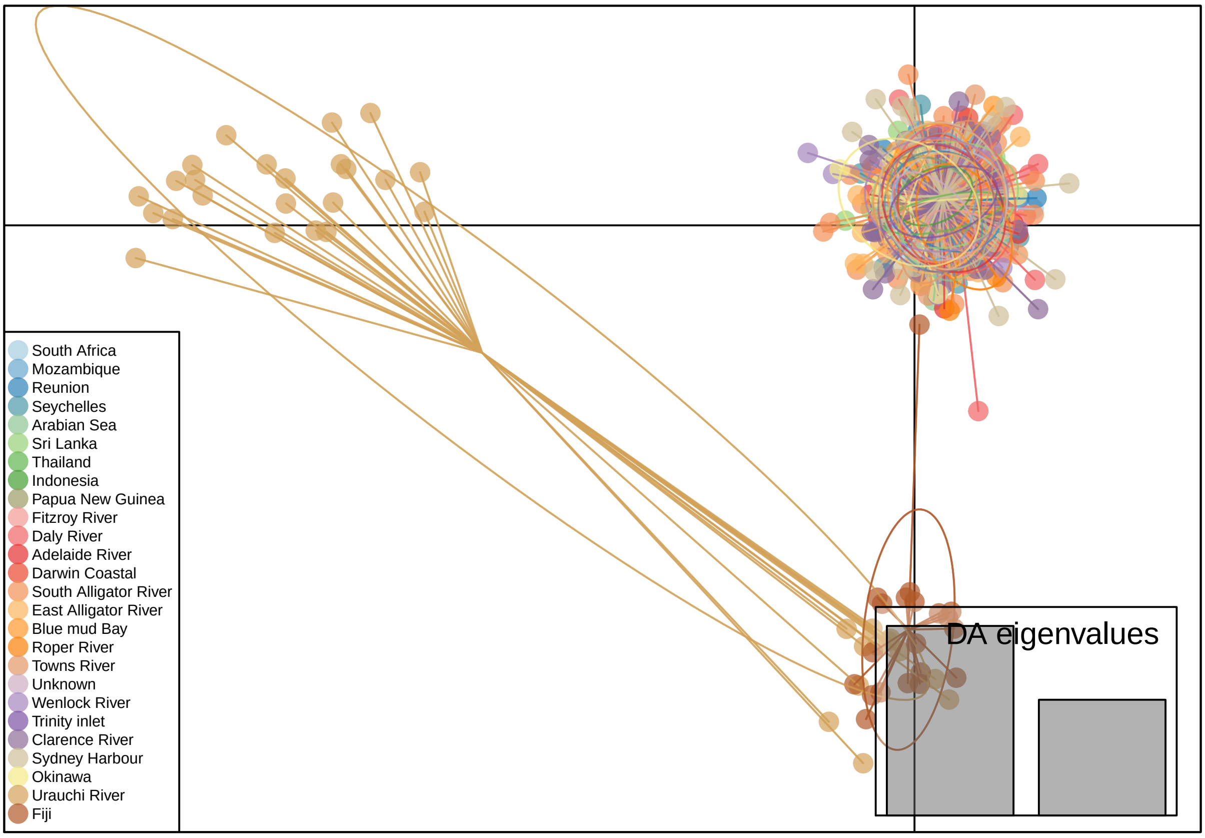
DA eigenvalues
South Africa
Mozambique
Reunion
Seychelles
Arabian Sea
Sri Lanka
Thailand
Indonesia
Papua New Guinea
Fitzroy River
Daly River
Adelaide River
Darwin Coastal
South Alligator River
East Alligator River
Blue mud Bay
Roper River
Towns River
Unknown
Wenlock River
Trinity inlet
Clarence River
Sydney Harbour
Okinawa
Urauchi River
Fiji

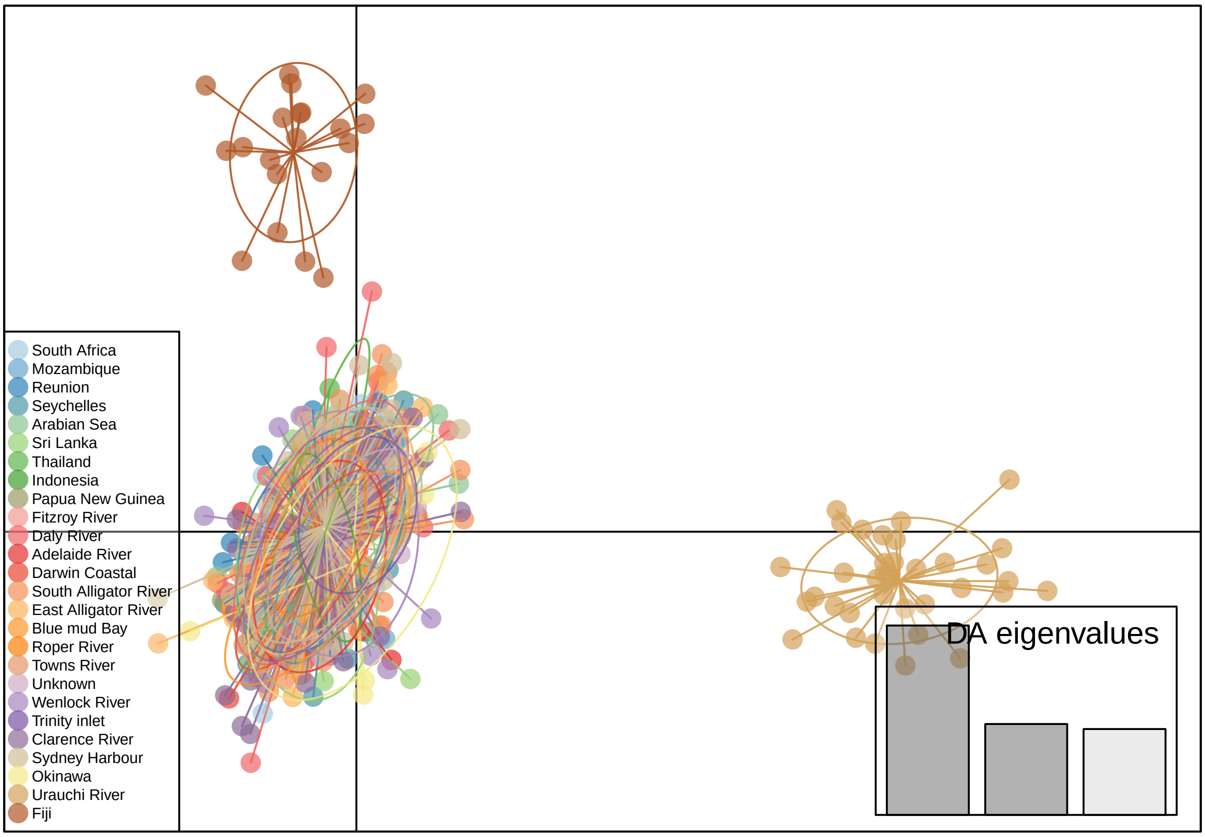
DA eigenvalues
South Africa
Mozambique
Reunion
Seychelles
Arabian Sea
Sri Lanka
Thailand
Indonesia
Papua New Guinea
Fitzroy River
Daly River
Adelaide River
Darwin Coastal
South Alligator River
East Alligator River
Blue mud Bay
Roper River
Towns River
Unknown
Wenlock River
Trinity inlet
Clarence River
Sydney Harbour
Okinawa
Urauchi River
Fiji

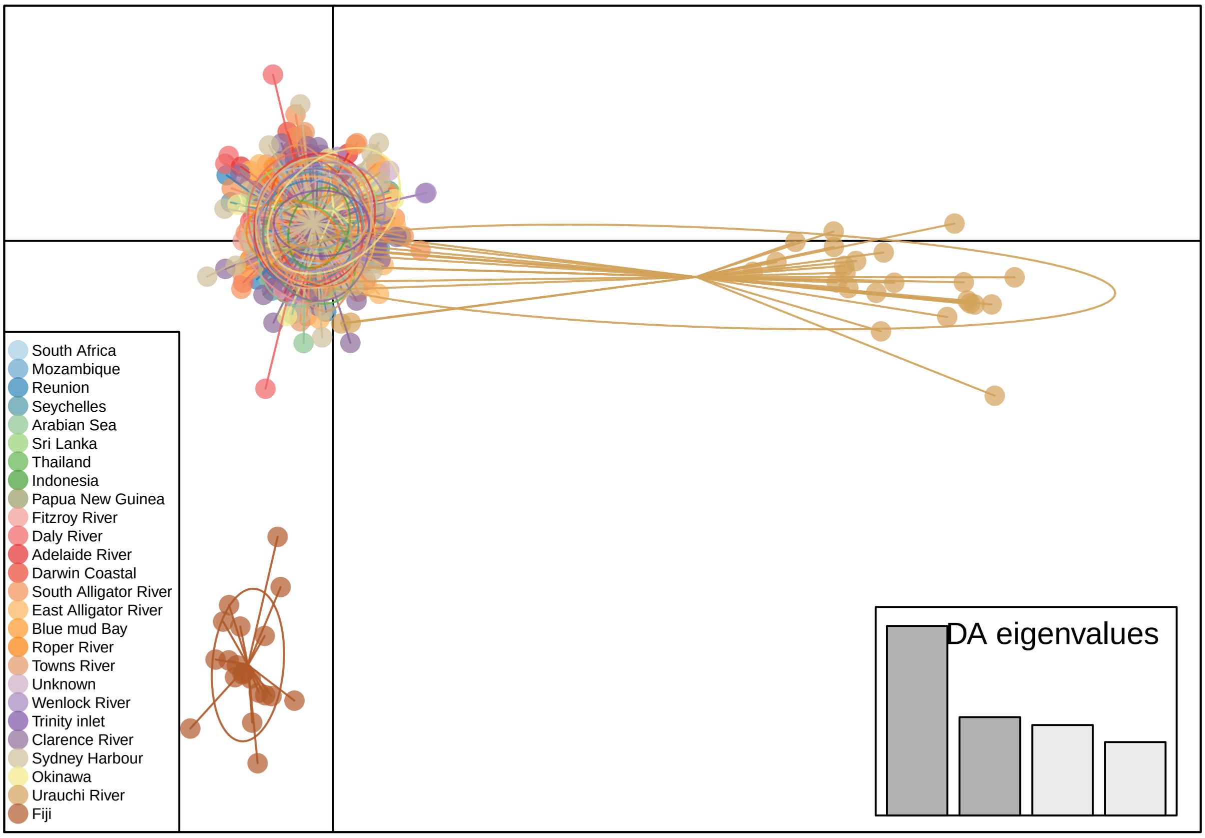
South Africa
Mozambique
Reunion
Seychelles
Arabian Sea
Sri Lanka
Thailand
Indonesia
Papua New Guinea
Fitzroy River
Daly River
Adelaide River
Darwin Coastal
South Alligator River
East Alligator River
Blue mud Bay
Roper River
Towns River
Unknown
Wenlock River
Trinity inlet
Clarence River
Sydney Harbour
Okinawa
Urauchi River
Fiji
DA eigenvalues

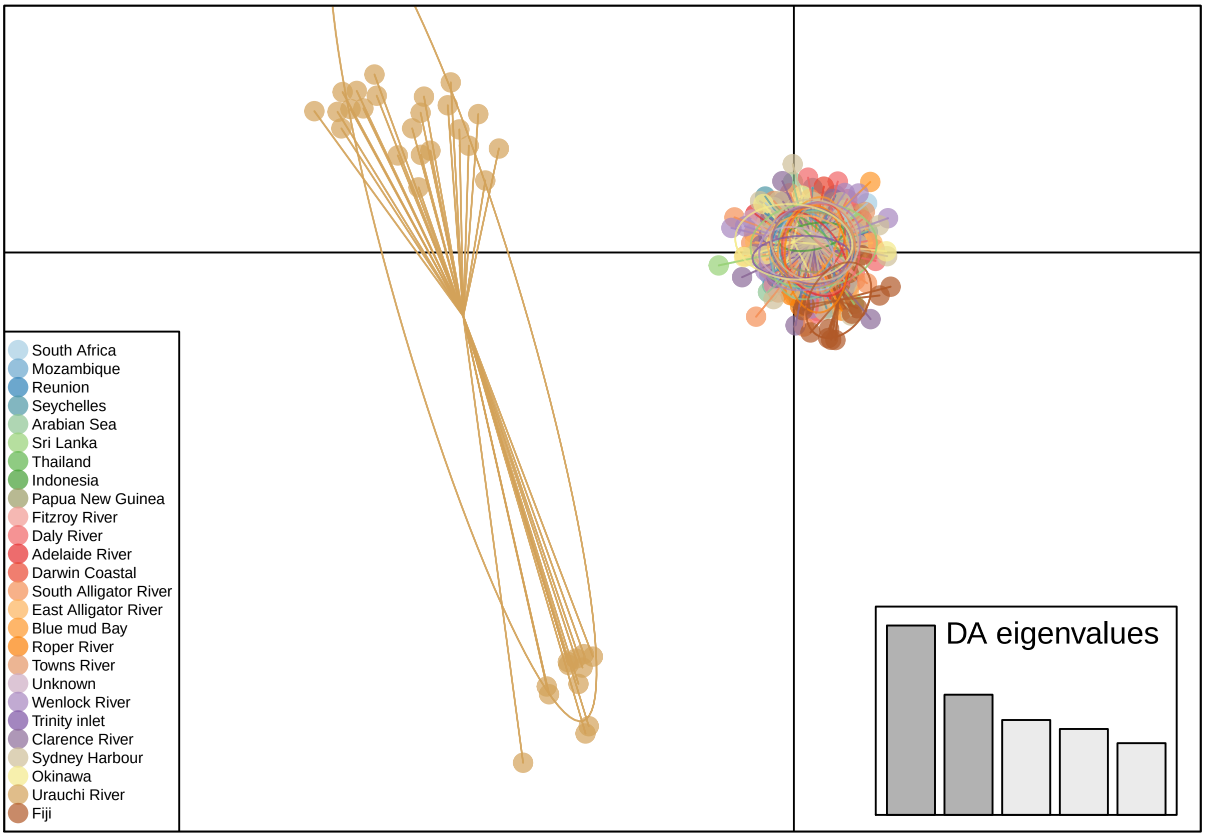
South Africa
Mozambique
Reunion
Seychelles
Arabian Sea
Sri Lanka
Thailand
Indonesia
Papua New Guinea
Fitzroy River
Daly River
Adelaide River
Darwin Coastal
South Alligator River
East Alligator River
Blue mud Bay
Roper River
Towns River
Unknown
Wenlock River
Trinity inlet
Clarence River
Sydney Harbour
Okinawa
Urauchi River
Fiji
DA eigenvalues

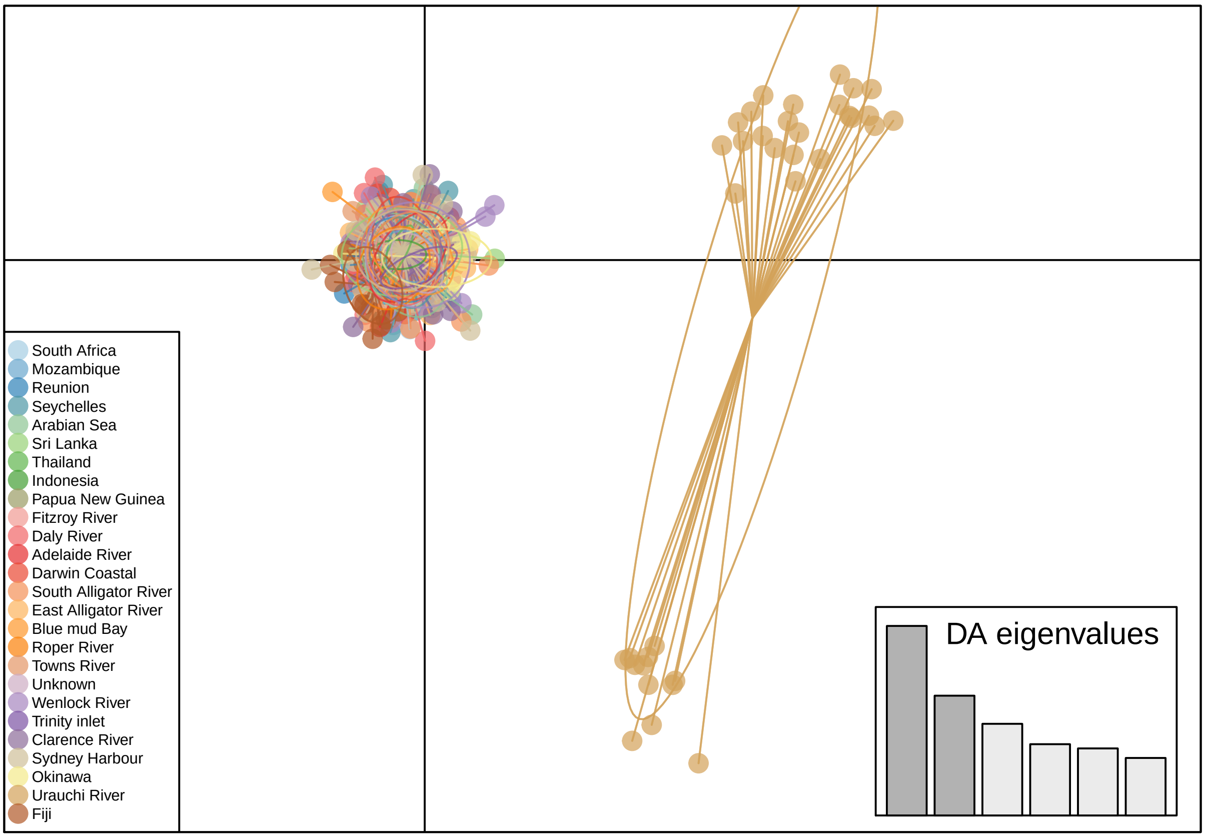
South Africa
Mozambique
Reunion
Seychelles
Arabian Sea
Sri Lanka
Thailand
Indonesia
Papua New Guinea
Fitzroy River
Daly River
Adelaide River
Darwin Coastal
South Alligator River
East Alligator River
Blue mud Bay
Roper River
Towns River
Unknown
Wenlock River
Trinity inlet
Clarence River
Sydney Harbour
Okinawa
Urauchi River
Fiji
DA eigenvalues

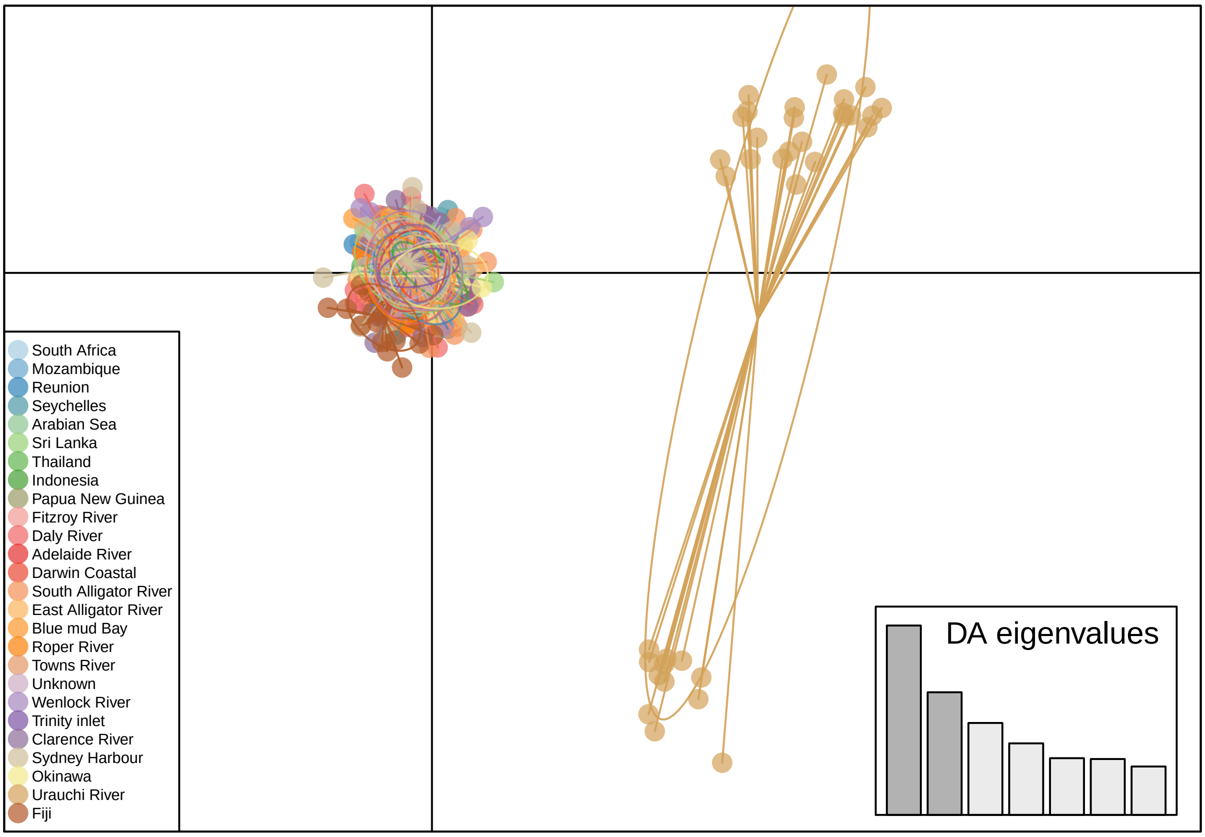
South Africa
Mozambique
Reunion
Seychelles
Arabian Sea
Sri Lanka
Thailand
Indonesia
Papua New Guinea
Fitzroy River
Daly River
Adelaide River
Darwin Coastal
South Alligator River
East Alligator River
Blue mud Bay
Roper River
Towns River
Unknown
Wenlock River
Trinity inlet
Clarence River
Sydney Harbour
Okinawa
Urauchi River
Fiji
DA eigenvalues

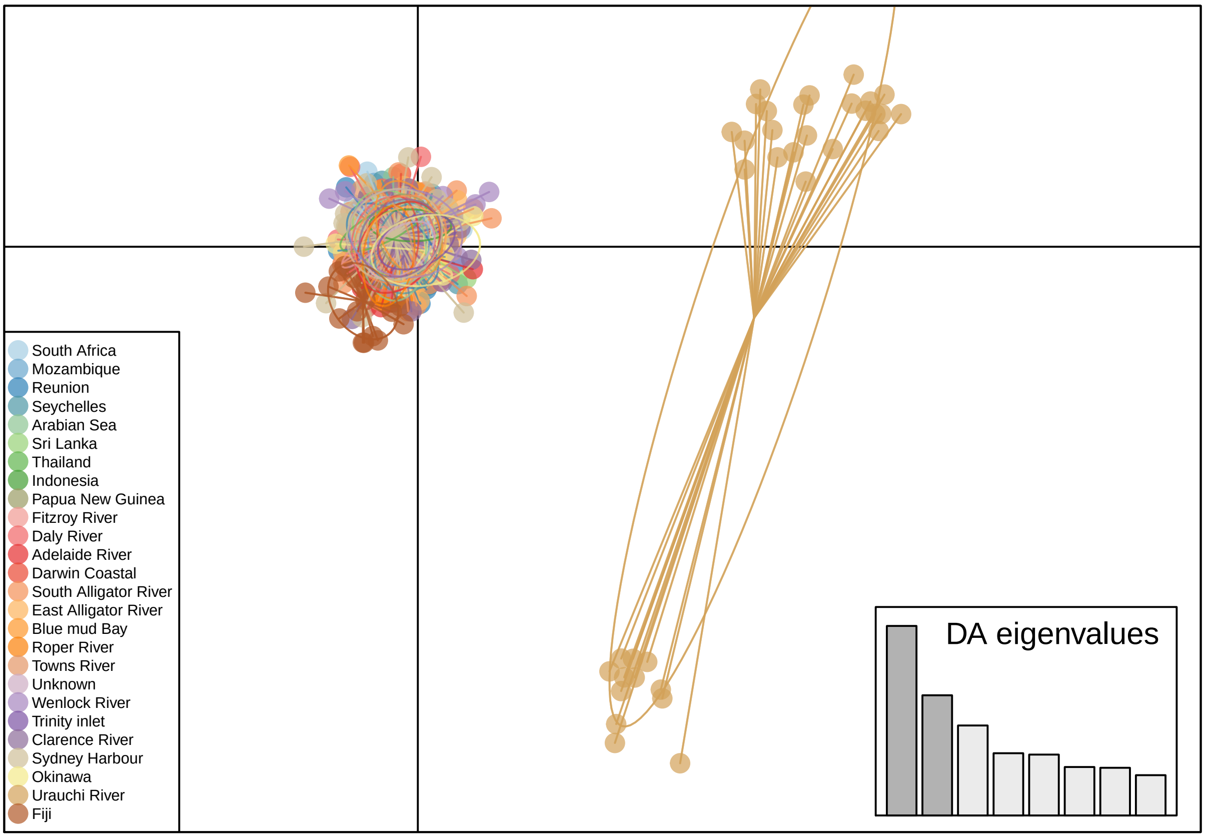


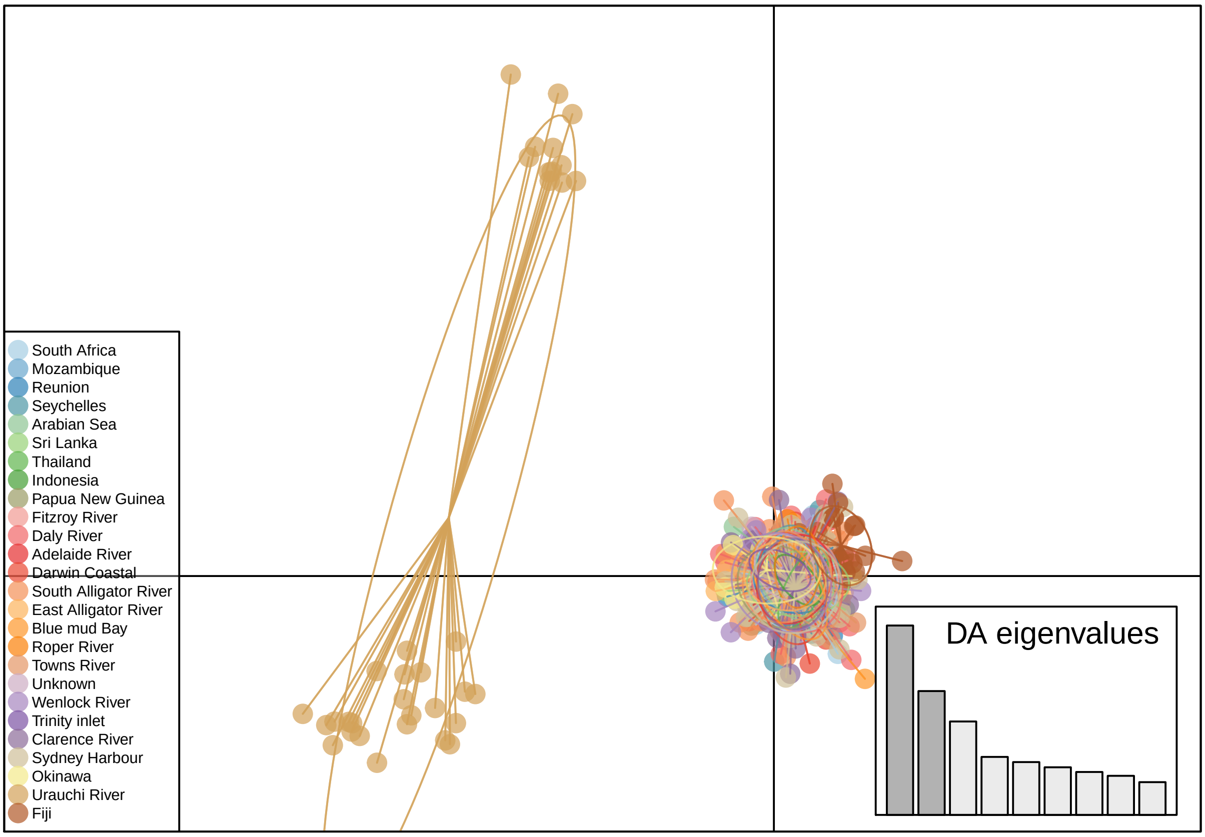


```
for (K in 2:10) {
  colour <- colours.prior[1:K]
  dapc <- get(dapc.all.object.names[K - 1])
  dapc.plot <- ade4::scatter(dapc, cex = 2, legend = TRUE, col = colour,
                              clabel = FALSE, posi.leg = "bottomleft",
```

```
                                scree.pca = FALSE, posi.pca = "topleft",
                                cleg = 0.75, xax = 1, yax = 2, inset.solid = 0.3)
  dev.print(
    device = png,
    file = paste0("DArTcap_IWP_DAPC_scatterplot_pergroups_K",
                  K,".png"),
    width = 30,
    height = 15,
    units = "cm",
    res = 300
  )
}
```

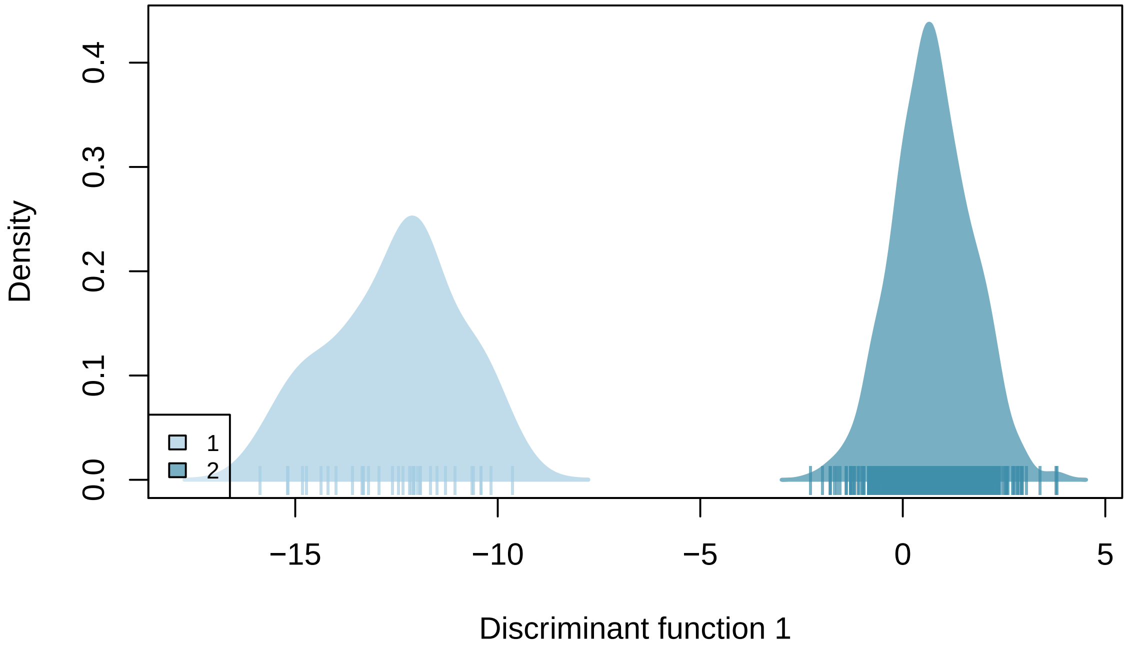

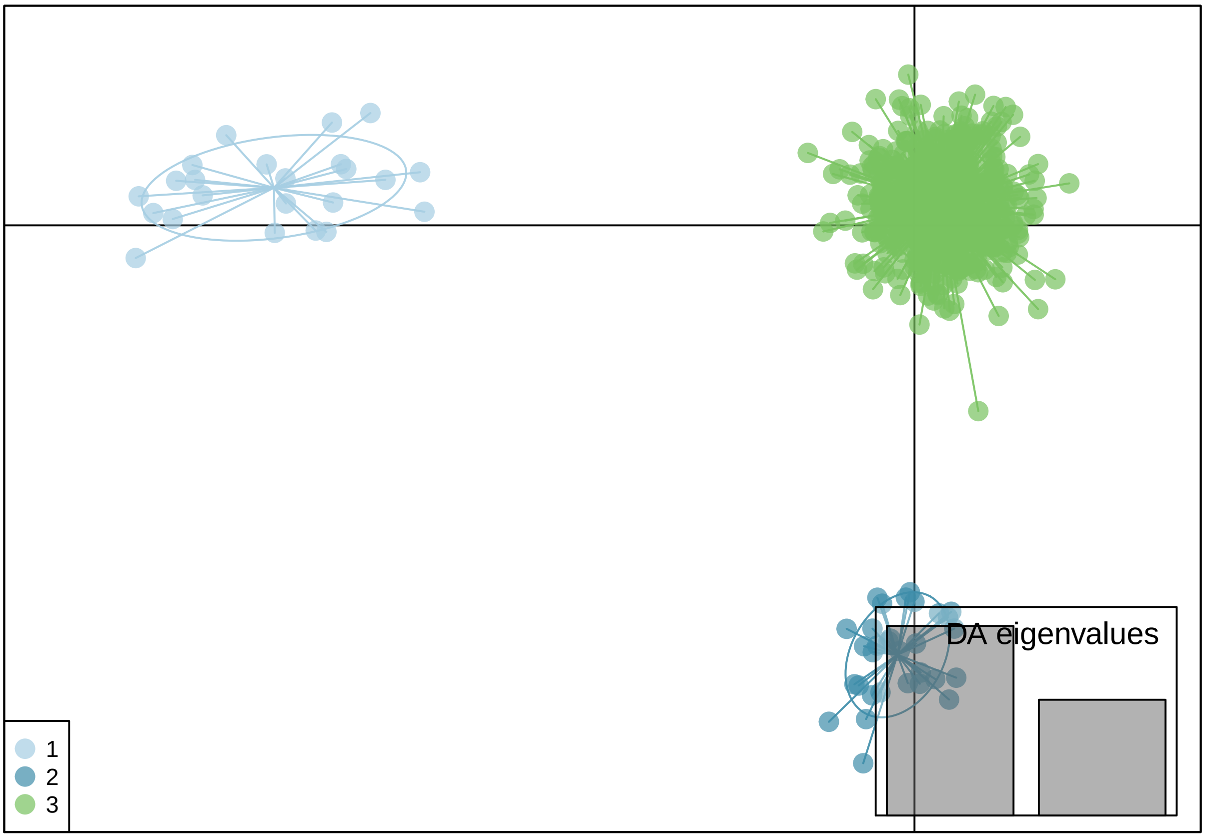
DA eigenvalues
1
2
3

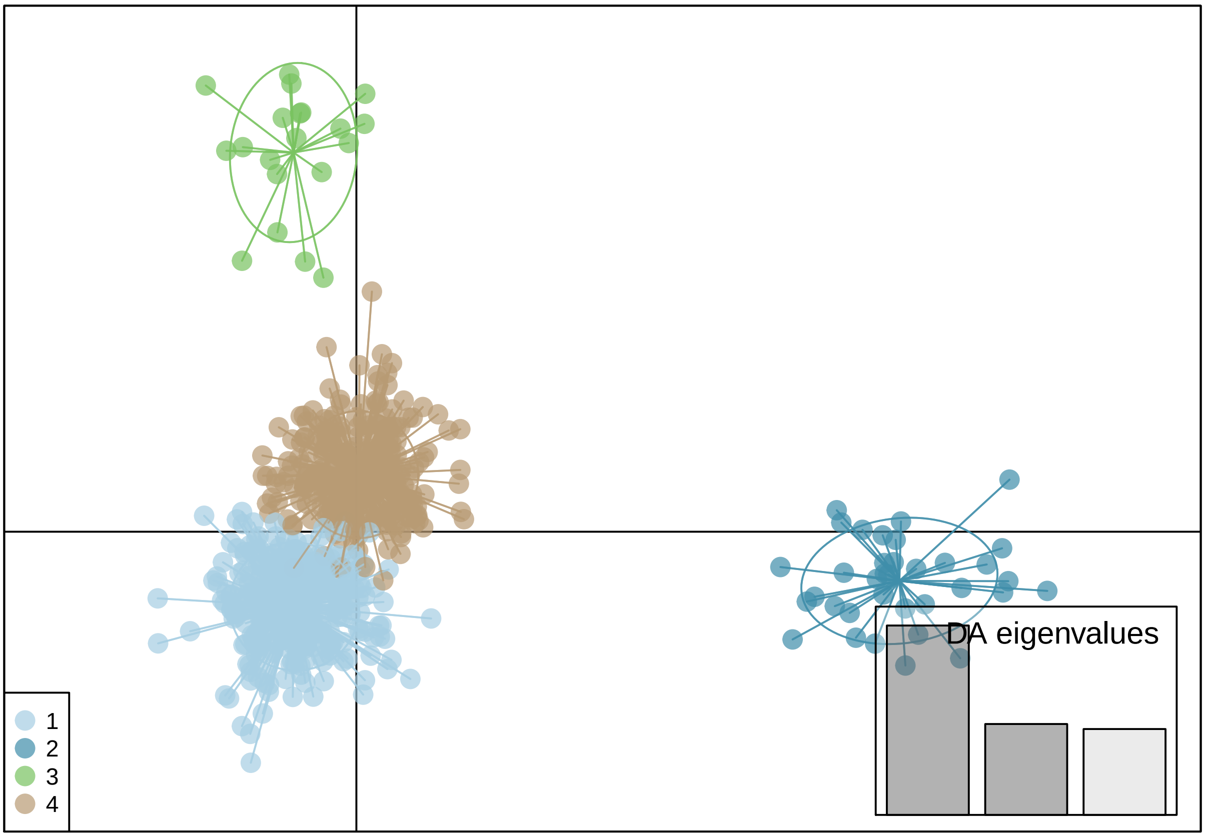
DA eigenvalues
1
2
3
4

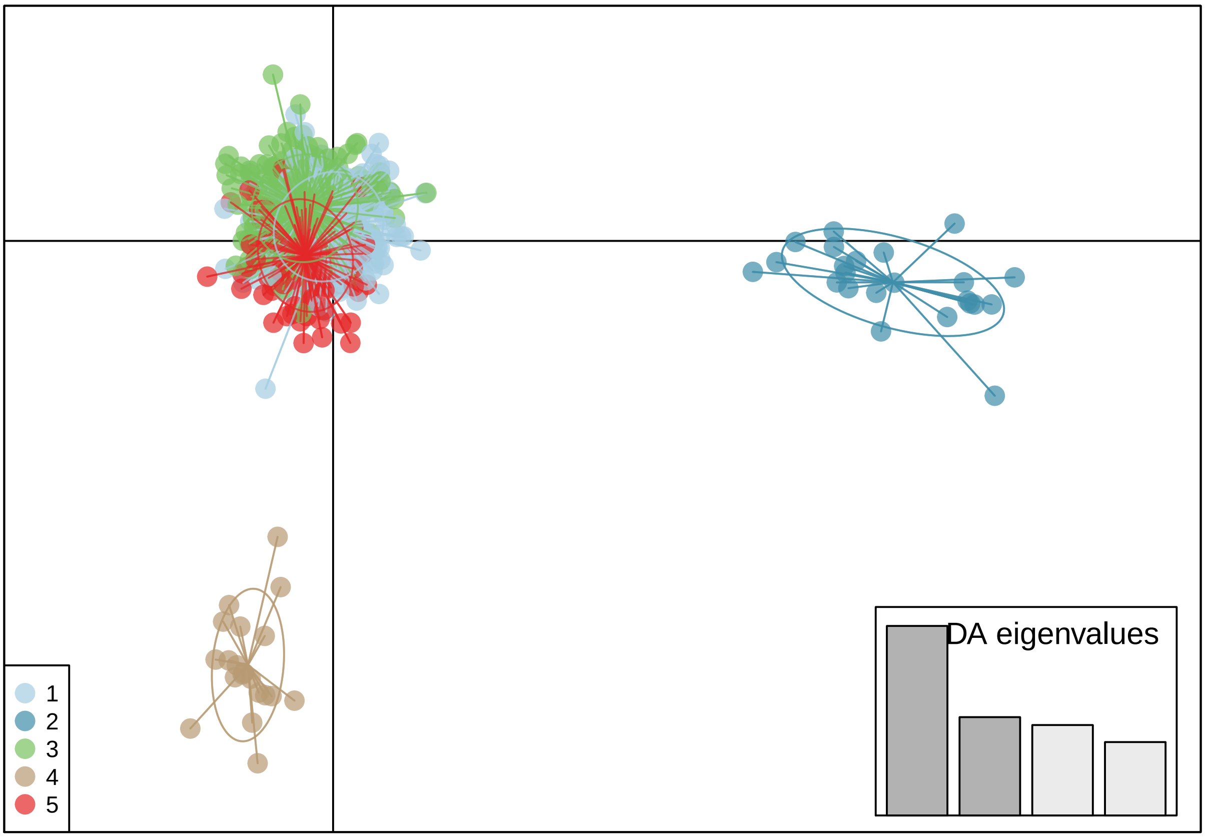
DA eigenvalues
1
2
3
4
5

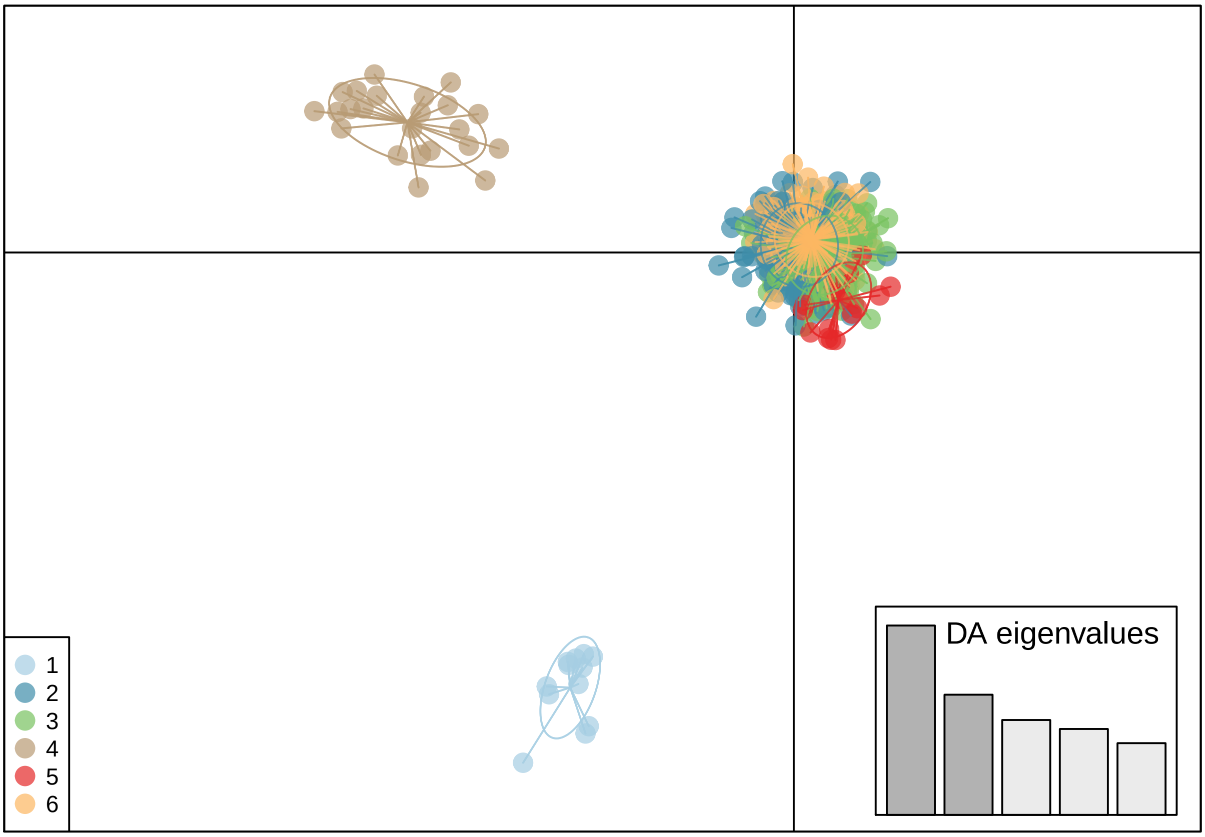
DA eigenvalues
1
2
3
4
5
6

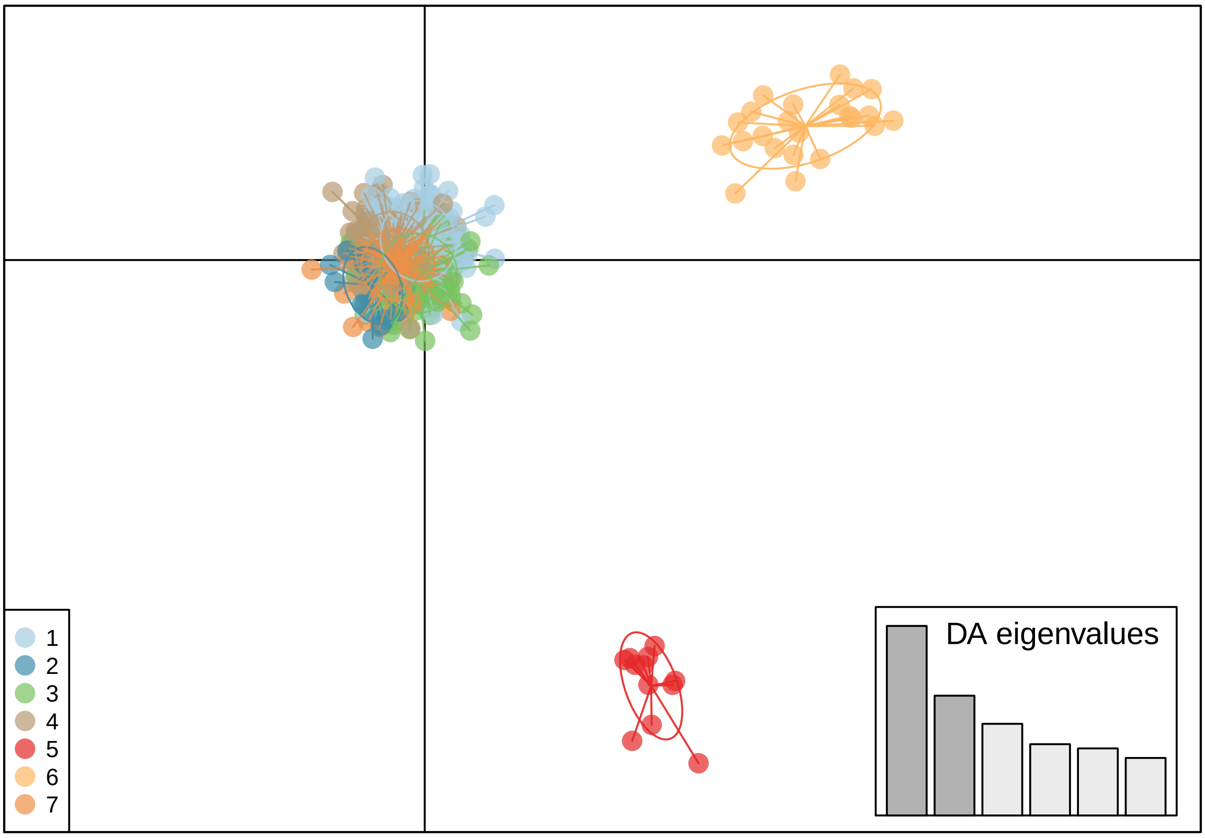
1
2
3
4
5
6
7
DA eigenvalues

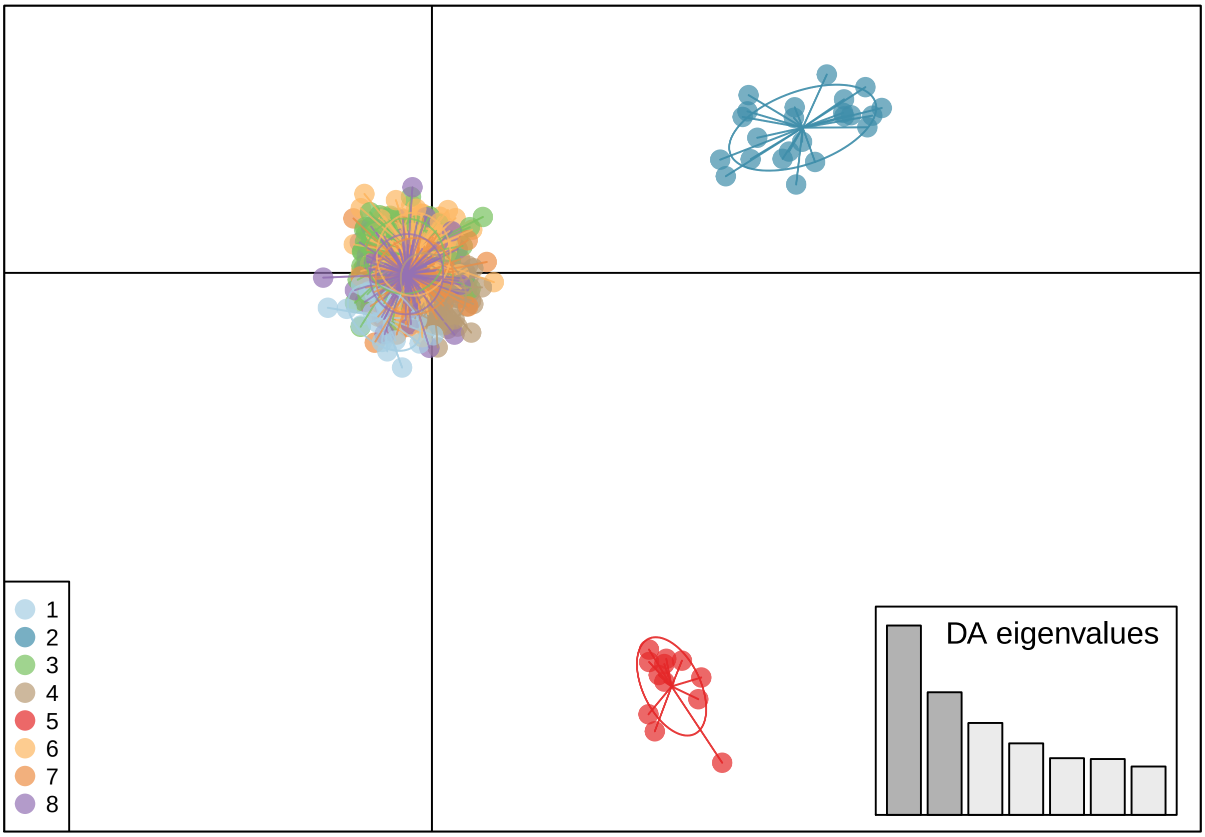
1
2
3
4
5
6
7
8
DA eigenvalues

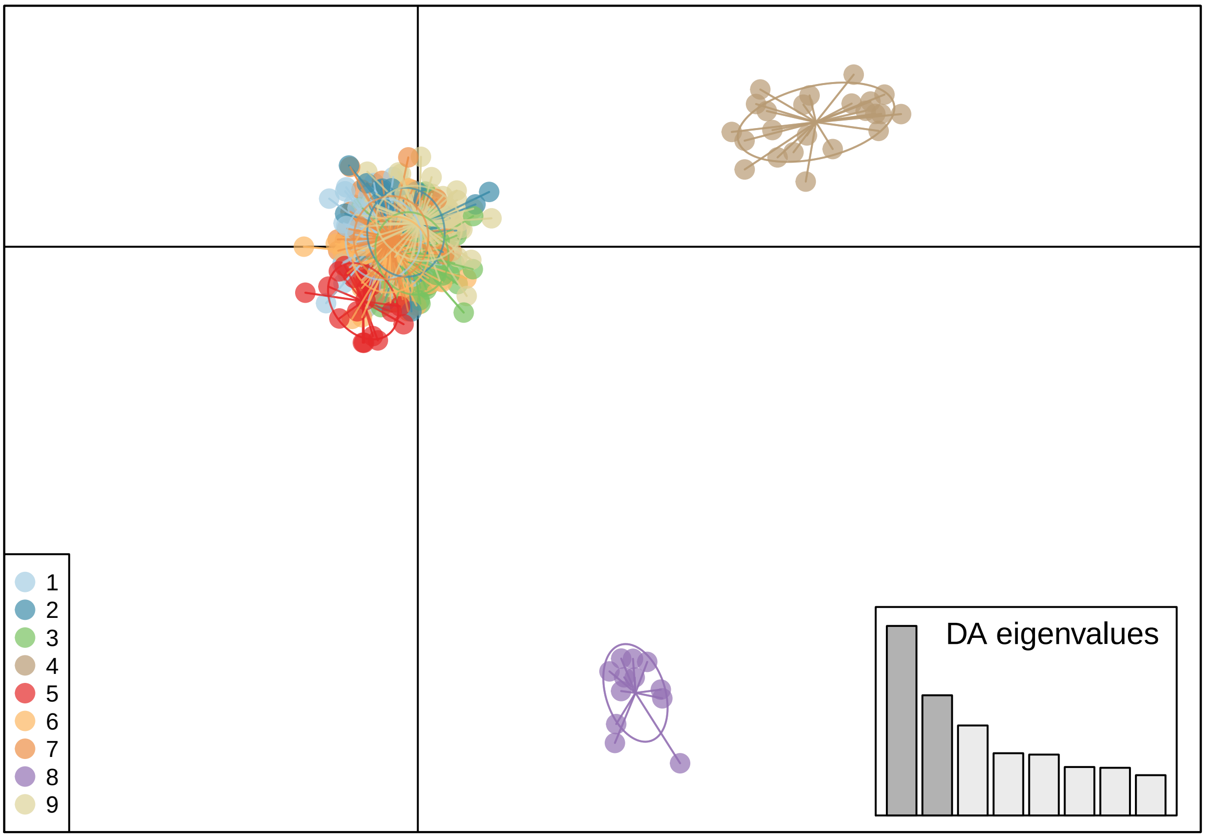


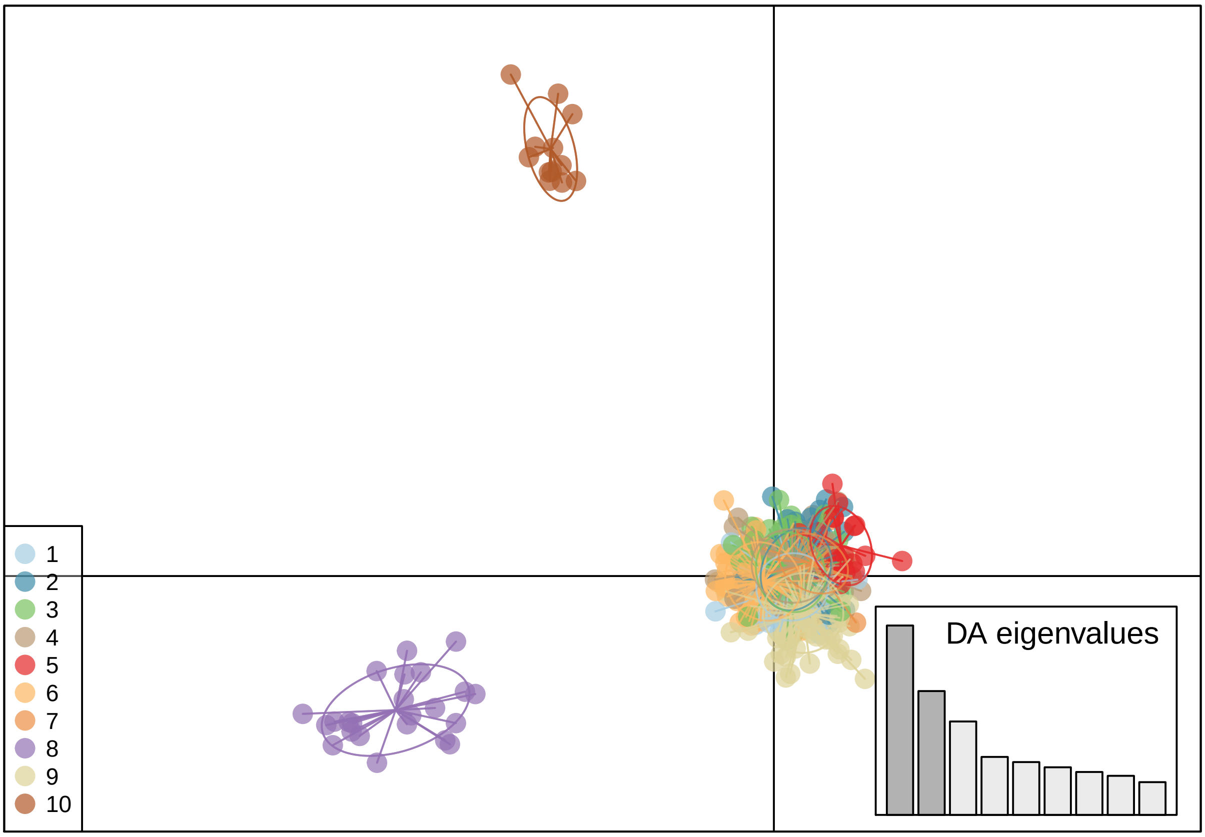


### 11.9.5 DAPC scatterplot - FLO

This code will *colour* individuals according to *sampling location* and **group** individuals according to DAPC **posterior membership**.

```r
for (K in 3:10) {
  dapc <- get(dapc.all.object.names[K - 1])
  x <- dapc$ind.coord[,1]
  y <- dapc$ind.coord[,2]
  group <- paste("Group", as.numeric(dapc$assign))
  pop <- BS.dartcap.IWP.gl$pop
  df <- data.frame(x, y, group, pop)
  colnames(df) <- c("x","y","group","pop")
  gg <- merge(df,aggregate(cbind(mean.x = x,mean.y = y)~group,df,mean),
              by = "group")

  plot <- ggplot2::ggplot(gg, ggplot2::aes(x,y,color = pop)) +
    ggplot2::geom_point(size = 5) +
    ggplot2::geom_point(ggplot2::aes(x = mean.x,y = mean.y),size = 5,
                        shape = 17, color = "black") +
    ggplot2::geom_segment(ggplot2::aes(x = mean.x, y = mean.y, xend = x,
                                       yend = y)) +
    ggplot2::scale_colour_manual(values = colours.26) +
    ggplot2::scale_fill_manual(values = colours.26) +
    ggplot2::geom_hline(yintercept = 0) +
    ggplot2::geom_vline(xintercept = 0) +
    ggplot2::labs(subtitle = "",
         y = "PC2",
         x = "PC1",
         title = paste0("K=",K," & PC=",PC, sep = ""),
         caption = "") +
    ggplot2::theme_minimal() +
    ggplot2::theme(
      axis.text = ggplot2::element_text(size = 5),
      axis.title.x = ggplot2::element_text(size = 10),
      axis.title.y = ggplot2::element_text(size = 10),
      legend.text = ggplot2::element_text(size = 7))
  print(plot)
  ggplot2::ggsave(plot, filename = paste0("DArTcap_IWP_DAPC_scatterplot2_K",
                                          K, ".png"),
                  width = 30, height = 15,units = "cm")
}
```

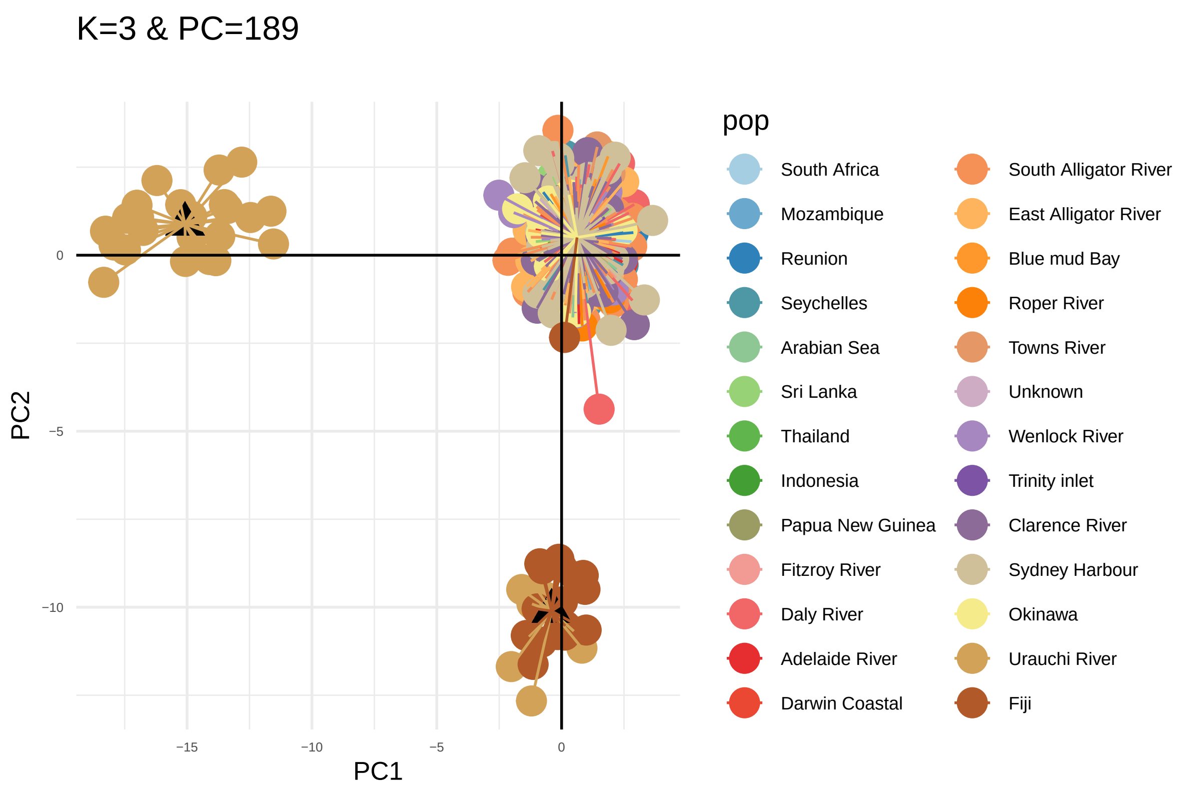
K=3 & PC=189
PC2
PC1
0
−5
−10
−15
−10
−5
0
pop
South Africa
Mozambique
Reunion
Seychelles
Arabian Sea
Sri Lanka
Thailand
Indonesia
Papua New Guinea
Fitzroy River
Daly River
Adelaide River
Darwin Coastal
South Alligator River
East Alligator River
Blue mud Bay
Roper River
Towns River
Unknown
Wenlock River
Trinity inlet
Clarence River
Sydney Harbour
Okinawa
Urauchi River
Fiji

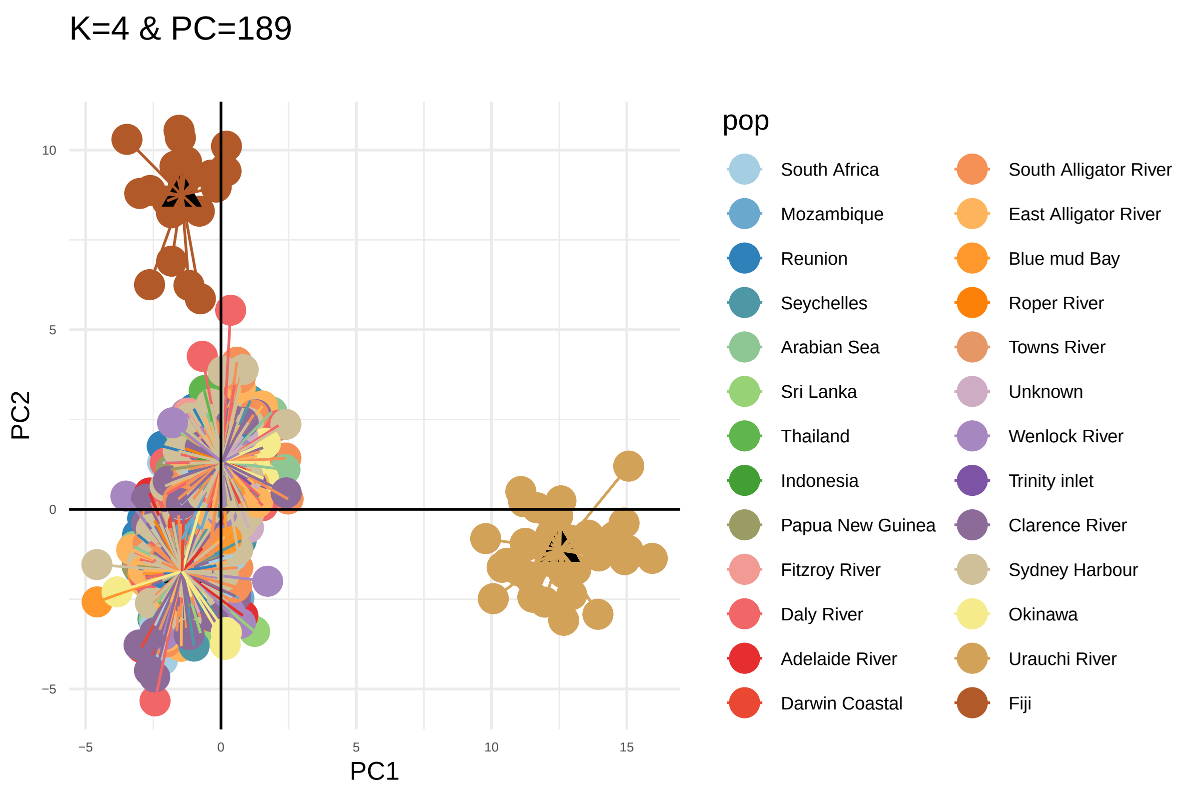
K=4 & PC=189
PC2
PC1
10
5
0
−5
−5
0
5
10
15
pop
South Africa
Mozambique
Reunion
Seychelles
Arabian Sea
Sri Lanka
Thailand
Indonesia
Papua New Guinea
Fitzroy River
Daly River
Adelaide River
Darwin Coastal
South Alligator River
East Alligator River
Blue mud Bay
Roper River
Towns River
Unknown
Wenlock River
Trinity inlet
Clarence River
Sydney Harbour
Okinawa
Urauchi River
Fiji

## K=5 & PC=189

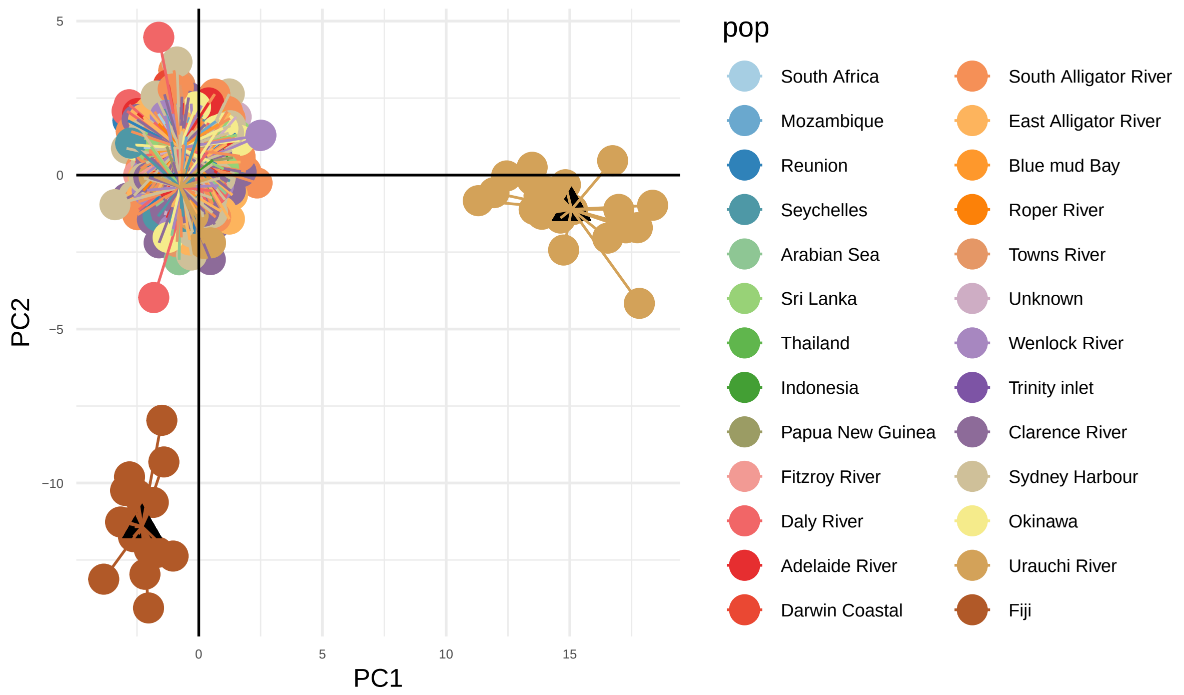


## K=6 & PC=189

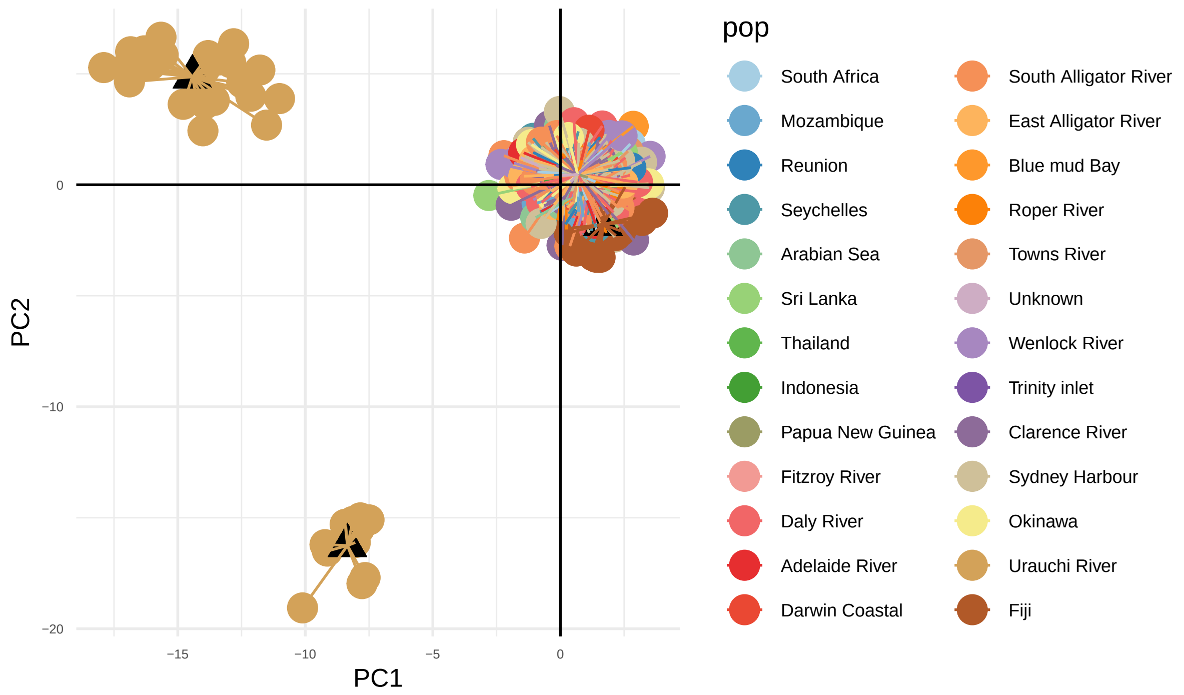

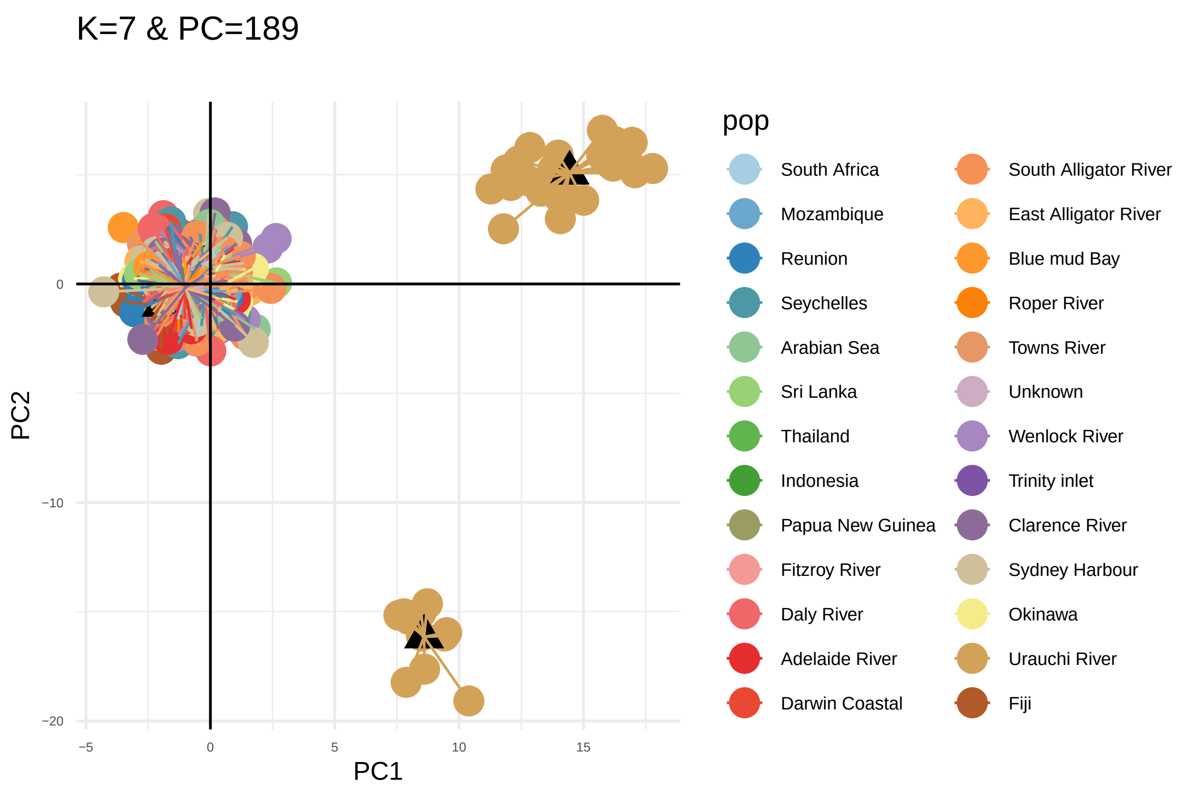
K=7 & PC=189
pop
South Africa
Mozambique
Reunion
Seychelles
Arabian Sea
Sri Lanka
Thailand
Indonesia
Papua New Guinea
Fitzroy River
Daly River
Adelaide River
Darwin Coastal
South Alligator River
East Alligator River
Blue mud Bay
Roper River
Towns River
Unknown
Wenlock River
Trinity inlet
Clarence River
Sydney Harbour
Okinawa
Urauchi River
Fiji
PC2
0
−10
−20
−5
0
5
10
15
PC1

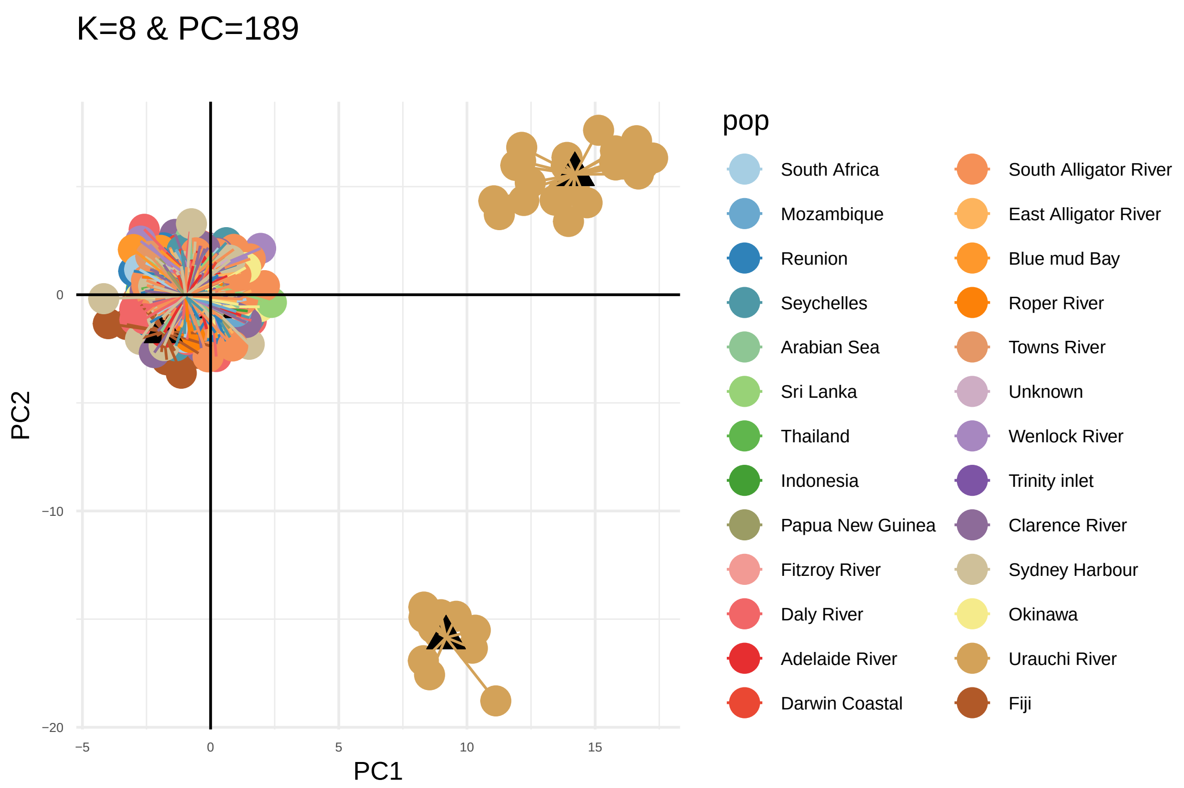
K=8 & PC=189
pop
South Africa
Mozambique
Reunion
Seychelles
Arabian Sea
Sri Lanka
Thailand
Indonesia
Papua New Guinea
Fitzroy River
Daly River
Adelaide River
Darwin Coastal
South Alligator River
East Alligator River
Blue mud Bay
Roper River
Towns River
Unknown
Wenlock River
Trinity inlet
Clarence River
Sydney Harbour
Okinawa
Urauchi River
Fiji
PC2
0
−10
−20
−5
0
5
10
15
PC1

K=9 & PC=189

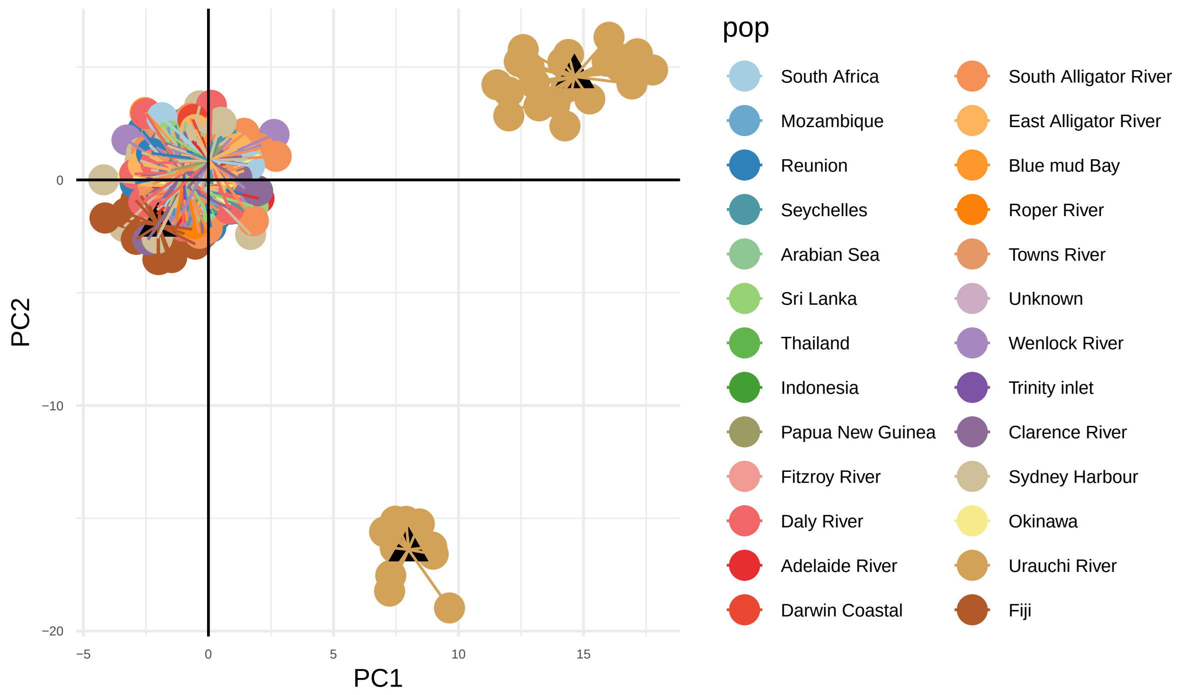


K=10 & PC=189

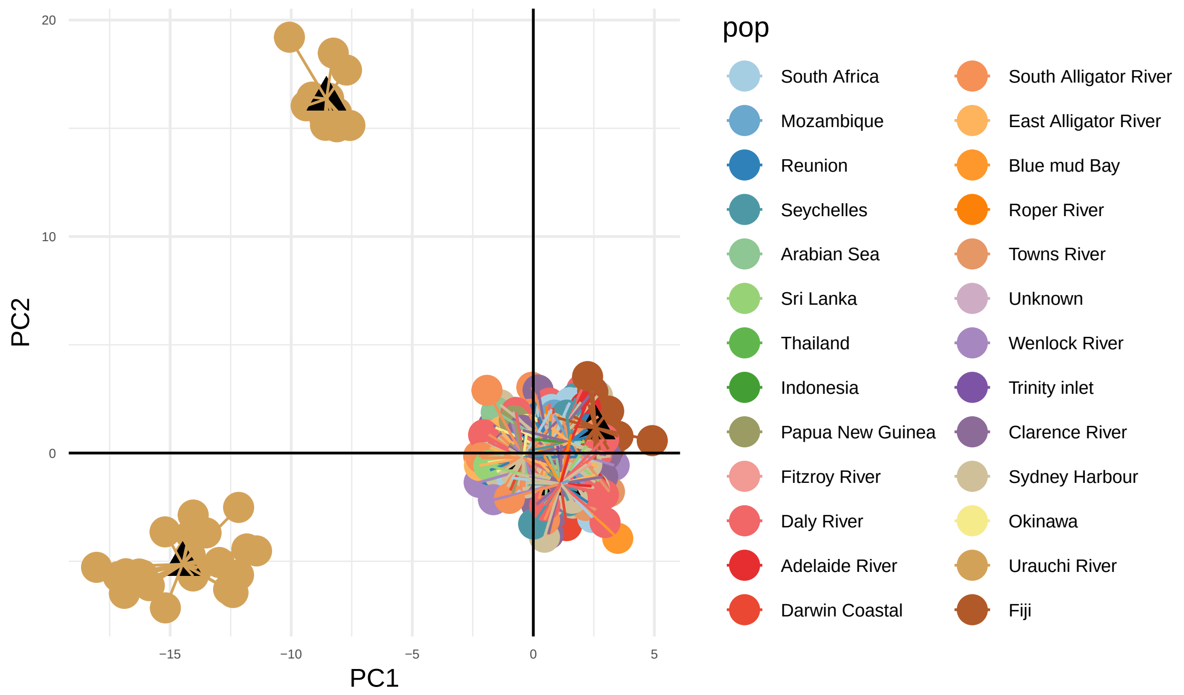

# 12 DATA6: DArTcap - Indo - West Pacific: no Japan or Fiji and subset from Australia

## 12.1 Set strata

```
strata <- radiator::read_strata("Bull_shark_DArTcap_strata5_IWP.tsv",
                                keep.two = FALSE)$strata %>%
  dplyr:: filter(Region %in% c("W-IO","N-IO", "E-IO","E-IO/W-PAC","W-PAC"))
id.sub <- c(strata$INDIVIDUALS[strata$Site != "Australia"],
            sample(strata$INDIVIDUALS[strata$Site == "Australia"], size = 60))
strata <- strata[strata$INDIVIDUALS %in% id.sub,]
readr::write_tsv(strata, path = "Bull_shark_DArTcap_strata6_IWP2.tsv")
```

## 12.2 Filter data

1. Marker Reproducibility: 0.98
2. Individual missingness: 0.25
3. Individual Het - low: 0
4. Individual Het - high: 0.07
5. Individual total coverage - low: 10,000
6. Individual total coverage - high: 1,000,000
7. Marker MAC - low: 3
8. Marker coverage - low: 5
9. Marker coverage - high:80
10. Marker maximum missing proportion: 0.1
11. Marker snp position on locus: n
12. Max 2 SNPs per locus
13. 1 SNP per locus according to mac
14. no LD filter
15. Individual Het - low: 0.100
16. Individual Het - high: 0.1337
17. Duplicated individuals: 0.2
18. Filter on HWE: 2 pops, 0.001

```
dartcap.data <- "SEQ_SNPs_counts_0_Target_merge.csv"
strata <- "Bull_shark_DArTcap_strata6_IWP2.tsv"
BSsnp <- radiator::filter_rad(dartcap.data, strata,
                              parallel.core = 1)
BS.dartcap.IWP2.gds <- BSsnp$gds
BS.dartcap.IWP2.tidy <- BSsnp$output$tidy.data
print(BS.dartcap.IWP2.gds, expand = TRUE, all = TRUE, attribute = TRUE,
      attribute.trim = FALSE)

save(BS.dartcap.IWP2.tidy, file = "DArTcap_IWP2.Rdata")
```

Table 47:

| FILTERS | VALUES | BEFORE | BLACKLIST |
|---|---|---|---|
| Filter DArT reproducibility | 0.98 | 241 / 21 / 1 / 26335 / 37537 | 0 / 0 / 0 / 1043 / 2250 |
| Filter monomorphic markers | NA | 241 / 21 / 1 / 25292 / 35287 | 0 / 0 / 0 / 15102 / 23847 |
| Filter markers in common | NA | 241 / 21 / 1 / 10190 / 11440 | 0 / 0 / 0 / 2105 / 2291 |
| Filter individuals based on missingness | 0.25 | 241 / 21 / 1 / 8085 / 9149 | 4 / 0 / 0 / 0 / 0 |
| Filter individuals based on heterozygosity | 0 0.07 | 237 / 21 / 1 / 8085 / 9149 | 10 / 0 / 0 / 0 / 0 |
| Filter individuals based on total coverage | 10000 1e+06 | 227 / 21 / 1 / 8085 / 9149 | 1 / 0 / 0 / 0 / 0 |
| Filter monomorphic markers | NA | 226 / 21 / 1 / 8085 / 9149 | 0 / 0 / 0 / 2298 / 2825 |
| Filter MAC | 3 | 226 / 21 / 1 / 5787 / 6324 | 0 / 0 / 0 / 3358 / 3750 |
| Filter coverage min / max | 5-80 | 226 / 21 / 1 / 2429 / 2574 | 0 / 0 / 0 / 526 / 537 |
| Filter genotyping | 0.1 | 226 / 21 / 1 / 1903 / 2037 | 0 / 0 / 0 / 91 / 97 |
| Filter SNPs position on the read | all | 226 / 21 / 1 / 1812 / 1940 | 0 / 0 / 0 / 0 / 0 |
| Filter markers snp number | 3 | 226 / 21 / 1 / 1812 / 1940 | 0 / 0 / 0 / 0 / 0 |
| detect mixed genomes | 0.1 0.1337 | 226 / 21 / 1 / 1812 / 1940 | 20 / 0 / 0 / 0 / 0 |
| Filter monomorphic markers | NA | 206 / 21 / 1 / 1812 / 1940 | 0 / 0 / 0 / 1 / 1 |
| detect duplicate genomes | 0.2 | 206 / 21 / 1 / 1811 / 1939 | 17 / 0 / 0 / 0 / 0 |
| Filter monomorphic markers | NA | 189 / 21 / 1 / 1811 / 1939 | 0 / 0 / 0 / 1 / 1 |
| Filter HWE | 20.001 | 189 / 21 / 1 / 1810 / 1938 | 0 / 0 / 0 / 2 / 2 |

### 12.2.1 Remove extra low and high heterozygous individuals

Because the average heterozygosity per sampling location is not even, certain individuals cannot be excluded without removing 'good' individuals.

```
rm <-  c("CL-ARS021-F","CL-GOM025-F.1","CL-GOM012-F","CL-SAF014-M","CL-SAF002-F",
         "CL-BRZ038-U","CLF003-F","CL-GOM011-F","CL-SAF015-F","CL-SAF003-M",
         "CL-PNG005-U","CLCR056-M","CLCR047.1","CLCR055-M","CLCR066-M")

BS.dartcap.IWP2.tidy <- BS.dartcap.IWP2.tidy[
  !BS.dartcap.IWP2.tidy$INDIVIDUALS %in% rm,]
length(unique(BS.dartcap.IWP2.tidy$INDIVIDUALS)) # 189
```

## 12.3 Save data

```
Convert <- radiator::genomic_converter(BS.dartcap.IWP2.tidy,
                                       output = c("genlight", "stockr",
                                                  "pcadapt", "gtypes", "rubias"))
BS.dartcap.IWP2.gl <- Convert$genlight
BS.dartcap.IWP2.stkr <- Convert$stockr
BS.dartcap.IWP2.pc <- Convert$pcadapt
BS.dartcap.IWP2.gt <- Convert$gtypes
BS.dartcap.IWP2.rubias <- Convert$rubias

markers.meta <- radiator::extract_markers_metadata(BS.dartcap.IWP2.gds,
                                                   whitelist = TRUE)
individuals.meta <- radiator::extract_individuals_metadata(BS.dartcap.IWP2.gds,
                                                           whitelist = TRUE)
individuals.meta <- individuals.meta[individuals.meta$INDIVIDUALS %in%
                                       BS.dartcap.IWP2.gl$ind.names,]
BS.dartcap.IWP2.gl$other$ind.metrics <- individuals.meta
BS.dartcap.IWP2.gl$other$loc.metrics <- markers.meta
BS.dartcap.IWP2.gt@schemes <- individuals.meta

pop.levels <- c("South Africa", "Mozambique", "Reunion" , "Seychelles",
                "Arabian Sea", "Sri Lanka", "Thailand", "Indonesia",
                "Papua New Guinea" , "Fitzroy River", "Daly River",
                "Adelaide River","Darwin Coastal",  "South Alligator River",
                "East Alligator River","Blue mud Bay", "Roper River",
                "Towns River","Wenlock River", "Trinity inlet","Clarence River",
                "Sydney Harbour")
BS.dartcap.IWP2.gl$pop <- factor(BS.dartcap.IWP2.gl$other$ind.metrics$Site2,
                                 levels = pop.levels)
BS.dartcap.IWP2.gl <- BS.dartcap.IWP2.gl[order(BS.dartcap.IWP2.gl$pop,
                                               BS.dartcap.IWP2.gl$ind.names),]
BS.dartcap.IWP2.gl <- BS.dartcap.IWP2.gl[order(BS.dartcap.IWP2.gl$pop,
                                               BS.dartcap.IWP2.gl$ind.names)]
BS.dartcap.IWP2.rubias$repunit <- factor(BS.dartcap.IWP2.rubias$repunit,
                                         levels = pop.levels)

save(BS.dartcap.IWP2.tidy, BS.dartcap.IWP2.gl, BS.dartcap.IWP2.stkr,
     BS.dartcap.IWP2.pc,BS.dartcap.IWP2.gt,BS.dartcap.IWP2.rubias,
     markers.meta, individuals.meta, file = "DArTcap_IWP2.Rdata")
```

## 12.4 Load data

```
load("DArTcap_IWP2.Rdata")
```

```r
pop.levels <- c("South Africa", "Mozambique", "Reunion" , "Seychelles",
                "Arabian Sea", "Sri Lanka", "Thailand", "Indonesia",
                "Papua New Guinea" , "Australia")
BS.dartcap.IWP2.gl$pop <- factor(BS.dartcap.IWP2.gl$other$ind.metrics$Site,
                                 levels = pop.levels)
BS.dartcap.IWP2.gl <- BS.dartcap.IWP2.gl[order(BS.dartcap.IWP2.gl$pop,
                                               BS.dartcap.IWP2.gl$ind.names),]


unique(BS.dartcap.IWP2.tidy$POP_ID)
AUS <- c( "Daly_River", "Wenlock_River", "Sydney_Harbour","Clarence_River",
          "Fitzroy_River","Adelaide_River","South_Alligator_River","Trinity_inlet",
          "Blue_mud_Bay","East_Alligator_River","Towns_River","Darwin_Coastal")
POP_ID <- as.character(BS.dartcap.IWP2.tidy$POP_ID)
POP_ID[POP_ID %in% AUS] <- "Australia"
pop.levels1 <- stringr::str_replace_all(pop.levels, " ", "_")
BS.dartcap.IWP2.tidy$POP_ID <- factor(POP_ID, levels = pop.levels1)
BS.dartcap.IWP2.tidy <- BS.dartcap.IWP2.tidy[order(BS.dartcap.IWP2.tidy$POP_ID,
                                                   BS.dartcap.IWP2.tidy$INDIVIDUALS),]

adegenet::nInd(BS.dartcap.IWP2.gl) # 189
adegenet::nLoc(BS.dartcap.IWP2.gl) # 1808
summary(BS.dartcap.IWP2.gl$pop)
sum(duplicated(BS.dartcap.IWP2.gl$other$ind.metrics$`Genetic code`))# 0 replicate left
sum(baits$CloneID %in% BS.dartcap.IWP2.gl$other$loc.metrics$LOCUS) #72
```

```
##  [1] Arabian_Sea           Sri_Lanka             Thailand
##  [4] Mozambique            Reunion               South_Africa
##  [7] Papua_New_Guinea      Seychelles            Indonesia
## [10] Daly_River            Wenlock_River         Sydney_Harbour
## [13] Clarence_River        Fitzroy_River         Adelaide_River
## [16] South_Alligator_River Trinity_inlet         Blue_mud_Bay
## [19] East_Alligator_River  Towns_River           Darwin_Coastal
## 21 Levels: Arabian_Sea Sri_Lanka Thailand Mozambique Reunion ... Darwin_Coastal
## [1] 189
## [1] 1936
##     South Africa       Mozambique          Reunion       Seychelles
##               22                9               26               35
##      Arabian Sea        Sri Lanka         Thailand        Indonesia
##               15               11                4                4
## Papua New Guinea        Australia
##                9               54
## [1] 0
## [1] 72
```

### 12.5 Remove sex-linked markers

```r
load("Sex-linked_marker_DArTcap.Rdata")
load("Sex_results.Rdata")
sum(bull_shark$sexy.summary$CLONE_ID %in% SLM$sexy.summary$CLONE_ID) #33
sum(SLM$sexy.summary$CLONE_ID %in% bull_shark$sexy.summary$CLONE_ID) #23
sex.markers <- unique(c(bull_shark$sexy.summary$CLONE_ID,
                        SLM$sexy.summary$CLONE_ID))

sum(BS.dartcap.IWP2.gl$loc.names %in% sex.markers) #23
locnames <- BS.dartcap.IWP2.gl$loc.names
BS.dartcap.IWP2.gl <- BS.dartcap.IWP2.gl[,!locnames %in% sex.markers]
```

```r
BS.dartcap.IWP2.gl$other$loc.metrics <- BS.dartcap.IWP2.gl$other$loc.metrics[
  !locnames %in% sex.markers,]

sum(sex.markers %in% unique(BS.dartcap.IWP2.tidy$LOCUS)) # 22
BS.dartcap.IWP2.tidy <- BS.dartcap.IWP2.tidy[!BS.dartcap.IWP2.tidy$LOCUS %in%
                                                sex.markers,]

locus.gt <- stringr::str_match(pattern = "__\\s*(.*?)\\s*__",
                               string =  BS.dartcap.IWP2.gt@data$locus)[,2]
BS.dartcap.IWP2.gt <- BS.dartcap.IWP2.gt[,!locus.gt %in% sex.markers, ,
                                          drop = TRUE]

locus.rubias <- stringr::str_match(pattern = "__\\s*(.*?)\\s*__",
                                   string =  colnames(BS.dartcap.IWP2.rubias))[,2]
BS.dartcap.IWP2.rubias <- BS.dartcap.IWP2.rubias[,!locus.rubias %in%
                                                    sex.markers]
```

```
## [1] 33
## [1] 23
## [1] 23
## [1] 22
```

## 12.6 Genetic diversity

```r
set.seed(124)

pop.levels <- c("South Africa", "Mozambique", "Reunion" , "Seychelles",
                "Arabian Sea", "Sri Lanka", "Thailand", "Indonesia",
                "Papua New Guinea" , "Australia")

gl <- BS.dartcap.IWP2.gl
gl$pop <- factor(gl$other$ind.metrics$Site, levels = pop.levels)
gl <- gl[order(gl$pop,gl$ind.names)]

gl2gpop(gl, filename = "BS_dartcap_IWP2_genepop.txt")
bastat <-diveRsity::basicStats(infile = "BS_dartcap_IWP2_genepop.txt",
                               outfile = "BS_dartcap_IWP2_BasicStat_output.txt",
                               fis_ci = TRUE,ar_ci = TRUE, fis_boots = 1000,
                               ar_boots = 1000,mc_reps = 1000,
                               rarefaction = FALSE, ar_alpha = 0.05,
                               fis_alpha = 0.05)

length(bastat$main_tab)#10
gendiv <- data.frame(
  "South Africa" = bastat$main_tab[[1]]$overall,
  "Mozambique" = bastat$main_tab[[2]]$overall,
  "Reunion" = bastat$main_tab[[3]]$overall,
  "Seychelles" = bastat$main_tab[[4]]$overall,
  "Arabian Sea" = bastat$main_tab[[5]]$overall,
  "Sri Lanka" = bastat$main_tab[[6]]$overall,
  "Thailand" = bastat$main_tab[[7]]$overall,
  "Indonesia" = bastat$main_tab[[8]]$overall,
  "Papua New Guinea" = bastat$main_tab[[9]]$overall,
  "Australia" = bastat$main_tab[[10]]$overall,
  check.names = FALSE)
row.names(gendiv) <- rownames(bastat$main_tab[[1]])

df <- as.data.frame(gl)
```

```
df[df == "0"] <- "11"
df[df == "1"] <- "12"
df[df == "2"] <- "22"
gen.gi <- adegenet::df2genind(df, ploidy = 2,ncode = 2, pop = gl$pop)
ris_basic <- hierfstat::basic.stats(gen.gi)
fis.hierfstat <- apply(ris_basic$Fis, 2, mean, na.rm=T)
fis.CI <- hierfstat::boot.ppfis(gen.gi, nboot = 1000, quant = c(0.025,0.975),
                                diploid = TRUE,dig = 4)
df <- data.frame(fis.hierfstat,fis.CI.low = fis.CI$fis.ci[,1],
                 fis.CI.high = fis.CI$fis.ci[,2])
df <- as.data.frame(t(df))
gendiv <- rbind(gendiv, df)

readr::write_tsv(gendiv, path = "DArTcap_IWP2_gendiv.txt")
save(gendiv, file = "DArTcap_IWP2_gendiv.Rdata")
```

```
load("DArTcap_IWP2_gendiv.Rdata")
shortnames <- c("SAF", "MOZ", "RUN" , "SEY", "ARS", "SRL", "TAI", "IND",
                "PNG" , "AUS")
knitr::kable(gendiv, col.names = shortnames, digits = 4,caption = "") %>%
  kableExtra::kable_styling(full_width = FALSE,font_size = 5,
                            latex_options = c( "hold_position"))
```

Table 48:

| | SAF | MOZ | RUN | SEY | ARS | SRL | TAI | IND | PNG | AUS |
|---|---|---|---|---|---|---|---|---|---|---|
| ar | 1.3280 | 1.3010 | 1.3220 | 1.3300 | 1.3210 | 1.3080 | 1.2800 | 1.2580 | 1.3050 | 1.3340 |
| size | 21.8700 | 8.7070 | 25.6740 | 34.7520 | 14.9200 | 10.9490 | 3.9810 | 3.9740 | 8.9420 | 53.6900 |
| obs_het | 0.1150 | 0.1140 | 0.1130 | 0.1170 | 0.1160 | 0.1160 | 0.1240 | 0.1150 | 0.1160 | 0.1150 |
| exp_het | 0.1200 | 0.1180 | 0.1190 | 0.1200 | 0.1180 | 0.1150 | 0.1090 | 0.1020 | 0.1140 | 0.1210 |
| uexp_het | 0.1230 | 0.1250 | 0.1220 | 0.1220 | 0.1220 | 0.1210 | 0.1250 | 0.1170 | 0.1210 | 0.1230 |
| fis | 0.0350 | 0.0210 | 0.0430 | 0.0270 | 0.0030 | -0.0120 | -0.1360 | -0.1300 | -0.0260 | 0.0430 |
| hwe_glb | 1.0000 | 1.0000 | 1.0000 | 1.0000 | 1.0000 | 1.0000 | 1.0000 | 1.0000 | 1.0000 | 1.0000 |
| hwe_hom | 1.0000 | 1.0000 | 1.0000 | 1.0000 | 1.0000 | 1.0000 | 1.0000 | 1.0000 | 1.0000 | 1.0000 |
| hwe_het | 1.0000 | 1.0000 | 1.0000 | 1.0000 | 1.0000 | 1.0000 | 1.0000 | 1.0000 | 1.0000 | 1.0000 |
| fis_lo | 0.0000 | -0.0830 | 0.0080 | 0.0020 | -0.0530 | -0.0930 | -0.3910 | -0.5630 | -0.1220 | 0.0270 |
| fis_hi | 0.0280 | 0.0080 | 0.0390 | 0.0250 | -0.0040 | -0.0210 | -0.1360 | -0.1300 | -0.0360 | 0.0410 |
| ar_lo | 1.2780 | 1.2020 | 1.2710 | 1.2800 | 1.2290 | 1.2150 | 1.2240 | 1.1690 | 1.2160 | 1.2830 |
| ar_hi | 1.3570 | 1.3530 | 1.3470 | 1.3520 | 1.3550 | 1.3570 | 1.3500 | 1.3200 | 1.3500 | 1.3590 |
| fis.hierfstat | 0.0570 | 0.0750 | 0.0613 | 0.0409 | 0.0353 | 0.0326 | -0.0083 | -0.0052 | 0.0286 | 0.0518 |
| fis.CI.low | 0.0515 | 0.0693 | 0.0585 | 0.0292 | 0.0255 | 0.0109 | -0.0232 | -0.0178 | 0.0215 | 0.0506 |
| fis.CI.high | 0.0855 | 0.1314 | 0.0937 | 0.0579 | 0.0669 | 0.0606 | 0.0511 | 0.0617 | 0.0716 | 0.0762 |

### 12.6.1 Number of monomorphic markers per population

```
pop.levels <- c("South Africa", "Mozambique", "Reunion" , "Seychelles",
                "Arabian Sea", "Sri Lanka", "Thailand", "Indonesia",
                "Papua New Guinea" , "Australia")

monomorphics <- c()
for (i in 1:length(pop.levels)) {
  x <- BS.dartcap.IWP2.gl[BS.dartcap.IWP2.gl$pop == pop.levels[i]]
  x2 <- dartR::gl.filter.monomorphs(x, verbose = 0)
  locs <-  adegenet::nLoc(x) - adegenet::nLoc(x2)
  monomorphics[i] <- locs
}
names(monomorphics) <- pop.levels
shortnames <- c("SAF", "MOZ", "RUN" , "SEY", "ARS", "SRL", "TAI", "IND",
                "PNG" , "AUS")
knitr::kable(t(as.table(monomorphics)), col.names = shortnames, digits = 4,
             caption = "") %>%
  kableExtra::kable_styling(full_width = FALSE,font_size = 5,
                            latex_options = c( "hold_position"))
```

Table 49:

| SAF | MOZ | RUN | SEY | ARS | SRL | TAI | IND | PNG | AUS |
|---|---|---|---|---|---|---|---|---|---|
| 625 | 1012 | 627 | 491 | 794 | 928 | 1299 | 1356 | 1010 | 276 |

### 12.6.2 Nucleotide diversity and inbreeding

```
pi.sum <- radiator::pi(
  data = BS.dartcap.IWP2.tidy,
  read.length = 70,
  parallel.core = parallel::detectCores() - 1,
  path.folder = NULL,
  verbose = TRUE
)

FIS.sum<- radiator::ibdg_fh(data = BS.dartcap.IWP2.tidy,
        path.folder = NULL, verbose = TRUE)

save(pi.sum, FIS.sum, file = "DArTcap_IWP2_pi_FIS_Sites.Rdata")
```

```
load("DArTcap_IWP2_pi_FIS_Sites.Rdata")
pi.sum$boxplot.pi
```

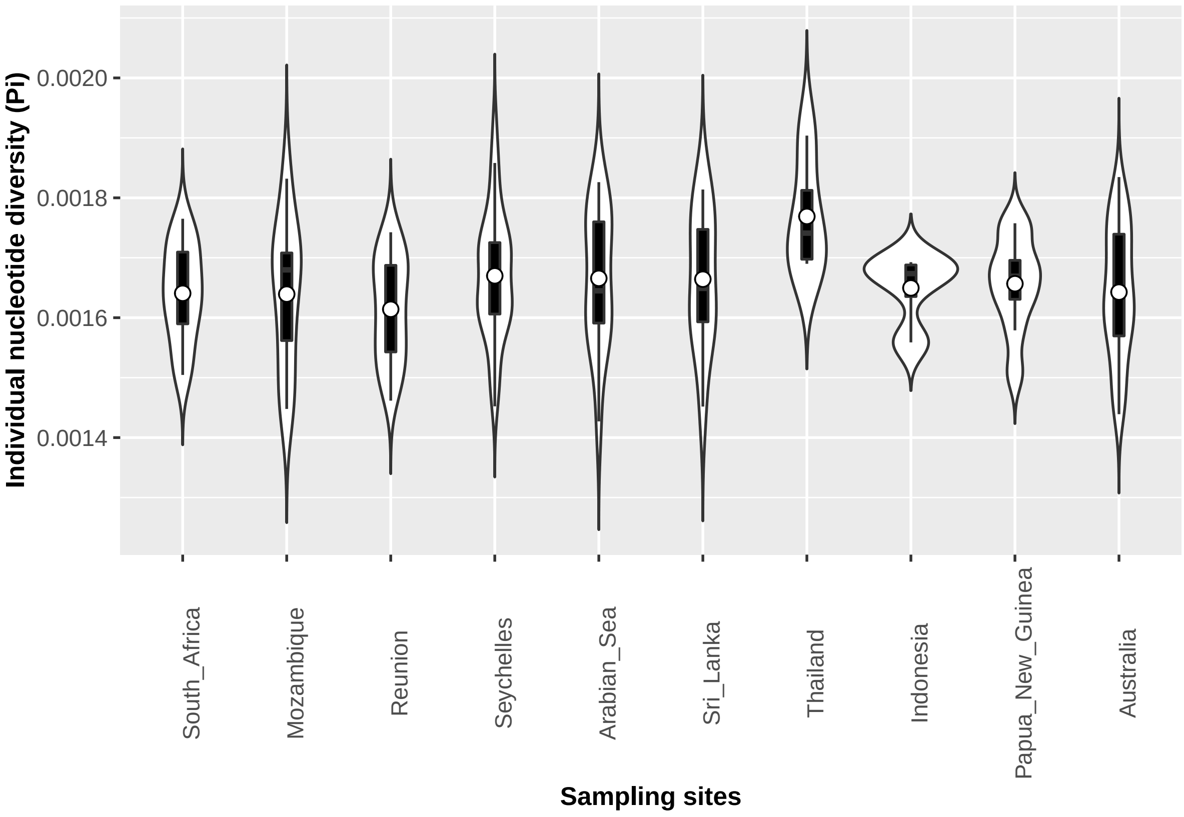


```
FIS.sum$fh.box.plot
```

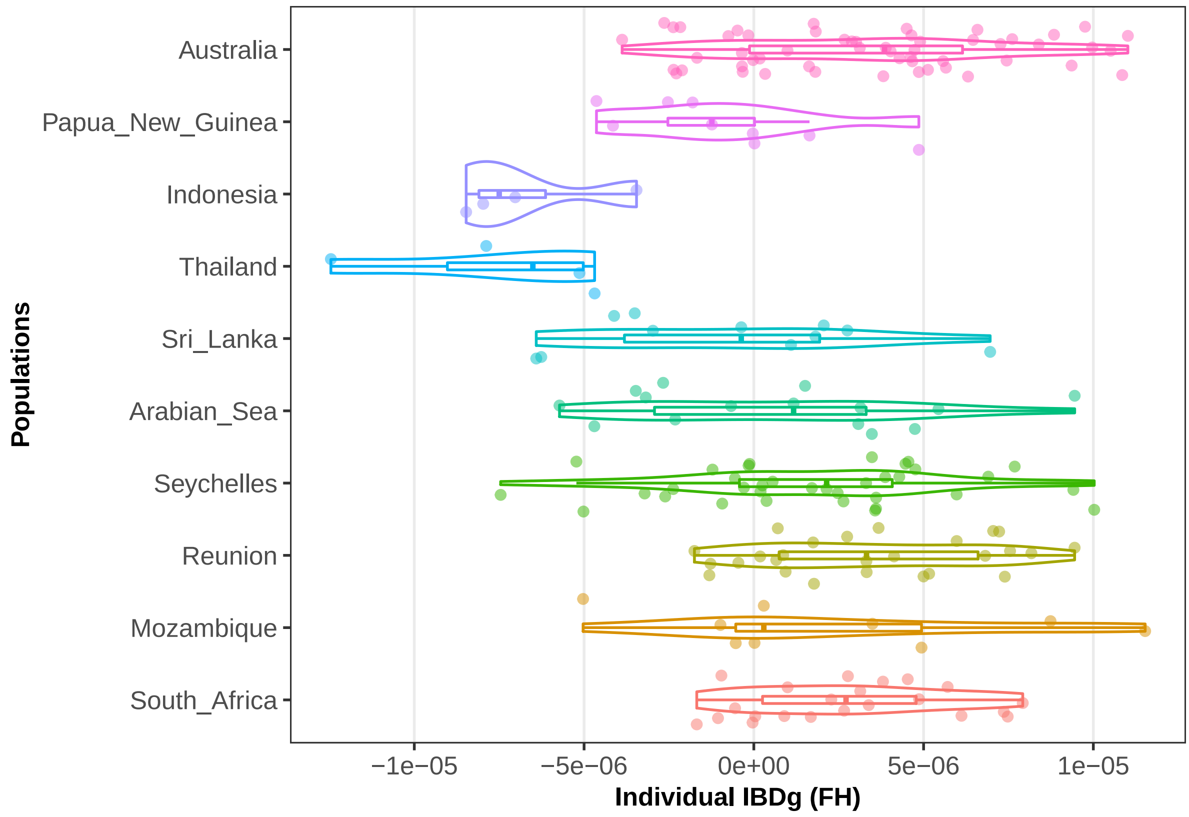


## 12.7 Genetic differentiation

```
strata <- BS.dartcap.IWP2.gt@schemes$Site
names(strata) <- BS.dartcap.IWP2.gt@schemes$INDIVIDUALS
strataG::setStrata(BS.dartcap.IWP2.gt) <- strata

Fst.gt <- strataG::popStructTest(
    BS.dartcap.IWP2.gt,
    nrep = 1000,
    stats = "all",
    type = "pairwise",
    keep.null = FALSE,
    quietly = TRUE,
    nb.cores =  parallel::detectCores() - 1,
    write.output = FALSE
  )
save(Fst.gt, file = "DArTcap_IWP2_FST.Rdata")
readr::write_tsv(Fst.gt$pairwise$result, path = "DArTcap_IWP2_FST.txt")
```

```
load("DArTcap_IWP2_FST.Rdata")

kableExtra::kbl(Fst.gt$pairwise$result[order(Fst.gt$pairwise$result$Fst,
                                              decreasing = TRUE),
                                        -c(1:4)], digits = 4,
  caption = "Pairwise Fst for 1924 DArTcap loci between the IWP sites (no JAP/FIJ and with subset of
  longtable = TRUE, booktabs = TRUE) %>%
  kableExtra::kable_styling(full_width = FALSE,font_size = 5,
                            latex_options = c("repeat_header", "hold_position")) %>%
  kableExtra::landscape()
```

Table 50: Pairwise Fst for 1924 DArTcap loci between the IWP sites (no JAP/FIJ and with subset of AUS)

| | Chi2 | Chi2.p.val | D | D.p.val | Fis | Fis.p.val | Fst | Fst.p.val | F'st | F'st.p.val | Gst | Gst.p.val | G'st | G'st.p.val | G"st | G"st.p.val |
|---|---|---|---|---|---|---|---|---|---|---|---|---|---|---|---|---|
| Indonesia (4) v. Papua New Guinea (9) | 1334.839 | 0.0589 | 7e-04 | 0.0370 | -6.7164 | 0.9730 | 0.0218 | 0.0430 | 0.0250 | 0.0430 | 0.2650 | 0.0310 | 0.3054 | 0.0340 | 0.4510 | 0.0330 |
| Indonesia (4) v. Seychelles (35) | 1830.180 | 0.0190 | 5e-04 | 0.0220 | -2.2181 | 0.9770 | 0.0165 | 0.0120 | 0.0190 | 0.0120 | 0.0884 | 0.0100 | 0.1085 | 0.0110 | 0.1809 | 0.0100 |
| Indonesia (4) v. Thailand (4) | 1079.938 | 0.3516 | 7e-04 | 0.3237 | -18.9626 | 0.6533 | 0.0161 | 0.3776 | 0.0184 | 0.3776 | 0.5845 | 0.3776 | 0.6236 | 0.3776 | 0.7625 | 0.3776 |
| Arabian Sea (15) v. Indonesia (4) | 1528.321 | 0.0330 | 6e-04 | 0.0290 | -4.0712 | 0.9749 | 0.0159 | 0.0291 | 0.0182 | 0.0291 | 0.1610 | 0.0291 | 0.1921 | 0.0301 | 0.3041 | 0.0301 |
| Indonesia (4) v. Reunion (26) | 1717.534 | 0.0390 | 6e-04 | 0.0480 | -3.0000 | 0.9538 | 0.0149 | 0.0392 | 0.0173 | 0.0392 | 0.1165 | 0.0452 | 0.1414 | 0.0472 | 0.2310 | 0.0472 |
| Indonesia (4) v. Mozambique (9) | 1307.929 | 0.1868 | 6e-04 | 0.0939 | -6.3804 | 0.8108 | 0.0142 | 0.0750 | 0.0169 | 0.0742 | 0.2569 | 0.2358 | 0.2976 | 0.2461 | 0.4411 | 0.2430 |
| Australia (54) v. Indonesia (4) | 1973.169 | 0.0410 | 6e-04 | 0.0020 | -1.1134 | 0.9980 | 0.0142 | 0.0030 | 0.0162 | 0.0030 | 0.0495 | 0.0030 | 0.0619 | 0.0030 | 0.1062 | 0.0030 |
| Indonesia (4) v. Sri Lanka (11) | 1355.726 | 0.1229 | 6e-04 | 0.0559 | -5.5080 | 0.9570 | 0.0140 | 0.0639 | 0.0160 | 0.0639 | 0.2153 | 0.0589 | 0.2518 | 0.0589 | 0.3844 | 0.0589 |
| Mozambique (9) v. Papua New Guinea (9) | 1508.837 | 0.0030 | 3e-04 | 0.1219 | -3.6037 | 0.9990 | 0.0132 | 0.0020 | 0.0152 | 0.0020 | 0.1074 | 0.0020 | 0.1316 | 0.0020 | 0.2159 | 0.0020 |
| Indonesia (4) v. South Africa (22) | 1583.104 | 0.1738 | 5e-04 | 0.0220 | -3.0250 | 0.9930 | 0.0109 | 0.0210 | 0.0125 | 0.0210 | 0.1180 | 0.0120 | 0.1432 | 0.0120 | 0.2337 | 0.0120 |
| Papua New Guinea (9) v. Reunion (26) | 1804.936 | 0.0130 | 3e-04 | 0.0390 | -2.0813 | 0.9381 | 0.0076 | 0.0210 | 0.0088 | 0.0200 | 0.0446 | 0.0200 | 0.0559 | 0.0200 | 0.0961 | 0.0200 |
| Arabian Sea (15) v. Papua New Guinea (9) | 1633.386 | 0.0010 | 3e-04 | 0.0270 | -2.6123 | 0.9560 | 0.0071 | 0.0040 | 0.0081 | 0.0040 | 0.0626 | 0.0040 | 0.0778 | 0.0040 | 0.1321 | 0.0040 |
| Mozambique (9) v. Reunion (26) | 1891.018 | 0.0020 | 3e-04 | 0.0420 | -1.9940 | 0.2917 | 0.0069 | 0.0170 | 0.0080 | 0.0170 | 0.0443 | 0.0120 | 0.0557 | 0.0100 | 0.0957 | 0.0120 |
| Papua New Guinea (9) v. Sri Lanka (11) | 1432.583 | 0.1319 | 3e-04 | 0.2837 | -3.3196 | 0.9500 | 0.0060 | 0.0250 | 0.0069 | 0.0250 | 0.0860 | 0.0240 | 0.1057 | 0.0240 | 0.1765 | 0.0240 |
| Australia (54) v. Reunion (26) | 2304.753 | 0.0010 | 2e-04 | 0.0010 | -0.5943 | 0.8412 | 0.0056 | 0.0010 | 0.0065 | 0.0010 | 0.0077 | 0.0010 | 0.0098 | 0.0010 | 0.0174 | 0.0010 |
| Mozambique (9) v. Seychelles (35) | 1914.374 | 0.0020 | 2e-04 | 0.1319 | -1.5864 | 0.0350 | 0.0056 | 0.0130 | 0.0064 | 0.0140 | 0.0330 | 0.0120 | 0.0418 | 0.0110 | 0.0724 | 0.0110 |
| Reunion (26) v. Seychelles (35) | 2114.155 | 0.0010 | 1e-04 | 0.0020 | -1.0778 | 0.9451 | 0.0053 | 0.0010 | 0.0061 | 0.0010 | 0.0124 | 0.0010 | 0.0158 | 0.0010 | 0.0279 | 0.0010 |
| Arabian Sea (15) v. Mozambique (9) | 1641.319 | 0.0080 | 3e-04 | 0.0709 | -2.4872 | 0.3127 | 0.0049 | 0.0260 | 0.0057 | 0.0230 | 0.0605 | 0.0160 | 0.0756 | 0.0150 | 0.1283 | 0.0150 |
| Papua New Guinea (9) v. Seychelles (35) | 1774.081 | 0.0230 | 3e-04 | 0.0100 | -1.7114 | 0.7393 | 0.0048 | 0.0210 | 0.0055 | 0.0210 | 0.0337 | 0.0190 | 0.0424 | 0.0200 | 0.0736 | 0.0190 |
| Reunion (26) v. South Africa (22) | 1983.382 | 0.0010 | 2e-04 | 0.0010 | -1.2610 | 0.9870 | 0.0044 | 0.0050 | 0.0050 | 0.0050 | 0.0165 | 0.0050 | 0.0210 | 0.0050 | 0.0369 | 0.0050 |
| Reunion (26) v. Sri Lanka (11) | 1814.081 | 0.0120 | 2e-04 | 0.2887 | -1.8398 | 0.9500 | 0.0044 | 0.0430 | 0.0050 | 0.0430 | 0.0335 | 0.0470 | 0.0421 | 0.0460 | 0.0732 | 0.0470 |
| Arabian Sea (15) v. Reunion (26) | 1947.800 | 0.0010 | 2e-04 | 0.0589 | -1.5135 | 0.9421 | 0.0033 | 0.0589 | 0.0038 | 0.0589 | 0.0228 | 0.0619 | 0.0289 | 0.0619 | 0.0505 | 0.0619 |
| Australia (54) v. Mozambique (9) | 1997.119 | 0.0280 | 2e-04 | 0.3057 | -0.8019 | 0.0150 | 0.0032 | 0.0400 | 0.0037 | 0.0400 | 0.0165 | 0.0430 | 0.0211 | 0.0380 | 0.0370 | 0.0410 |
| Mozambique (9) v. Sri Lanka (11) | 1449.012 | 0.2138 | 3e-04 | 0.1209 | -3.2057 | 0.5754 | 0.0026 | 0.1628 | 0.0030 | 0.1728 | 0.0842 | 0.1159 | 0.1039 | 0.1129 | 0.1735 | 0.1139 |
| Australia (54) v. Papua New Guinea (9) | 1827.823 | 0.3746 | 2e-04 | 0.0160 | -0.9249 | 0.9231 | 0.0020 | 0.1399 | 0.0023 | 0.1399 | 0.0176 | 0.0789 | 0.0224 | 0.0849 | 0.0393 | 0.0809 |
| Papua New Guinea (9) v. South Africa (22) | 1614.736 | 0.2517 | 3e-04 | 0.0430 | -2.0687 | 0.9461 | 0.0019 | 0.1878 | 0.0022 | 0.1878 | 0.0432 | 0.1179 | 0.0541 | 0.1209 | 0.0933 | 0.1209 |
| Mozambique (9) v. South Africa (22) | 1631.674 | 0.3137 | 2e-04 | 0.5315 | -2.0358 | 0.2198 | 0.0017 | 0.2258 | 0.0019 | 0.2278 | 0.0438 | 0.1309 | 0.0552 | 0.1259 | 0.0949 | 0.1269 |
| Arabian Sea (15) v. South Africa (22) | 1771.898 | 0.0190 | 2e-04 | 0.2907 | -1.5184 | 0.9520 | 0.0016 | 0.1289 | 0.0018 | 0.1289 | 0.0233 | 0.1089 | 0.0295 | 0.1119 | 0.0516 | 0.1099 |
| Arabian Sea (15) v. Australia (54) | 2068.432 | 0.0010 | 1e-04 | 0.2168 | -0.5996 | 0.9411 | 0.0010 | 0.1718 | 0.0012 | 0.1718 | 0.0078 | 0.1409 | 0.0099 | 0.1419 | 0.0176 | 0.1419 |
| Arabian Sea (15) v. Seychelles (35) | 1886.888 | 0.0080 | 1e-04 | 0.2697 | -1.2555 | 0.5355 | 0.0010 | 0.2378 | 0.0012 | 0.2378 | 0.0163 | 0.2567 | 0.0207 | 0.2577 | 0.0363 | 0.2577 |
| Australia (54) v. South Africa (22) | 1970.053 | 0.0859 | 1e-04 | 0.0549 | -0.5473 | 0.3107 | 0.0005 | 0.2587 | 0.0006 | 0.2587 | 0.0053 | 0.2797 | 0.0067 | 0.2787 | 0.0120 | 0.2797 |
| Seychelles (35) v. Thailand (4) | 1705.603 | 0.0979 | 4e-04 | 0.1618 | -2.0796 | 0.2700 | 0.0005 | 0.4290 | 0.0005 | 0.4290 | 0.0742 | 0.6640 | 0.0920 | 0.6410 | 0.1547 | 0.6540 |
| Seychelles (35) v. South Africa (22) | 1865.330 | 0.0849 | 1e-04 | 0.3716 | -1.0063 | 0.0649 | 0.0004 | 0.3417 | 0.0005 | 0.3417 | 0.0102 | 0.3666 | 0.0130 | 0.3616 | 0.0230 | 0.3656 |
| Australia (54) v. Sri Lanka (11) | 1880.911 | 0.2897 | 1e-04 | 0.4825 | -0.7601 | 0.9740 | 0.0003 | 0.4016 | 0.0003 | 0.4016 | 0.0117 | 0.3017 | 0.0149 | 0.3097 | 0.0263 | 0.3047 |
| Australia (54) v. Seychelles (35) | 2054.899 | 0.0110 | 1e-04 | 0.0300 | -0.4074 | 0.9950 | 0.0002 | 0.3856 | 0.0002 | 0.3856 | 0.0029 | 0.3656 | 0.0037 | 0.3666 | 0.0066 | 0.3656 |
| South Africa (22) v. Sri Lanka (11) | 1612.444 | 0.5534 | 2e-04 | 0.3057 | -1.8525 | 0.9181 | -0.0001 | 0.5205 | -0.0001 | 0.5205 | 0.0330 | 0.4336 | 0.0416 | 0.4466 | 0.0722 | 0.4426 |
| Seychelles (35) v. Sri Lanka (11) | 1717.271 | 0.2478 | 1e-04 | 0.5295 | -1.5159 | 0.6593 | -0.0002 | 0.5305 | -0.0003 | 0.5305 | 0.0236 | 0.5375 | 0.0298 | 0.5375 | 0.0522 | 0.5385 |
| Papua New Guinea (9) v. Thailand (4) | 1254.400 | 0.2298 | 4e-04 | 0.4356 | -5.7035 | 0.1330 | -0.0005 | 0.4760 | -0.0006 | 0.4760 | 0.2233 | 0.7660 | 0.2613 | 0.7370 | 0.3961 | 0.7440 |
| Arabian Sea (15) v. Sri Lanka (11) | 1503.034 | 0.6863 | 2e-04 | 0.8232 | -2.3378 | 0.3007 | -0.0021 | 0.8771 | -0.0024 | 0.8771 | 0.0460 | 0.8761 | 0.0575 | 0.8731 | 0.0989 | 0.8721 |
| Sri Lanka (11) v. Thailand (4) | 1276.722 | 0.4625 | 3e-04 | 0.8172 | -4.9286 | 0.0999 | -0.0032 | 0.7582 | -0.0036 | 0.7582 | 0.1875 | 0.9051 | 0.2220 | 0.8971 | 0.3448 | 0.9021 |
| Australia (54) v. Thailand (4) | 1796.699 | 0.4376 | 3e-04 | 0.6623 | -1.0756 | 0.6853 | -0.0033 | 0.7922 | -0.0038 | 0.7922 | 0.0378 | 0.8242 | 0.0477 | 0.8092 | 0.0824 | 0.8172 |
| Reunion (26) v. Thailand (4) | 1670.672 | 0.0689 | 4e-04 | 0.4106 | -2.6801 | 0.2305 | -0.0033 | 0.6854 | -0.0038 | 0.6854 | 0.0957 | 0.8457 | 0.1175 | 0.8307 | 0.1946 | 0.8357 |
| Arabian Sea (15) v. Thailand (4) | 1371.048 | 0.4426 | 3e-04 | 0.6813 | -3.7791 | 0.1171 | -0.0052 | 0.8989 | -0.0059 | 0.8989 | 0.1395 | 0.9690 | 0.1682 | 0.9620 | 0.2701 | 0.9640 |
| South Africa (22) v. Thailand (4) | 1491.926 | 0.5934 | 4e-04 | 0.4416 | -2.7460 | 0.3227 | -0.0063 | 0.9451 | -0.0072 | 0.9451 | 0.0985 | 0.9650 | 0.1208 | 0.9650 | 0.1996 | 0.9660 |
| Mozambique (9) v. Thailand (4) | 1174.486 | 0.8771 | 4e-04 | 0.8801 | -5.8031 | 0.0738 | -0.0078 | 0.9108 | -0.0090 | 0.9108 | 0.2277 | 0.9713 | 0.2668 | 0.9682 | 0.4028 | 0.9713 |

## 12.8 PCA

```r
pca1 <- adegenet::glPca(BS.dartcap.IWP2.gl, nf = 5, parallel = TRUE,
                        n.cores = parallel::detectCores() - 1)
save(pca1, file = "DArTcap_IWP2_PCA.Rdata")
```

```r
load("DArTcap_IWP2_PCA.Rdata")

BS.pca.scores <- as.data.frame(pca1$scores)
BS.pca.scores$pop <- pop(BS.dartcap.IWP2.gl)
cols <- adegenet::funky(nPop(BS.dartcap.IWP2.gl))


p <-
  ggplot2::ggplot(BS.pca.scores, ggplot2::aes(x = PC1, y = PC2, colour = pop)) +
  ggplot2::geom_point(size = 2) + ggplot2::scale_color_manual(values = cols) +
  ggplot2::geom_hline(yintercept = 0) + ggplot2::geom_vline(xintercept = 0) +
  ggplot2::theme_minimal() +
  ggplot2::theme(
    axis.text = ggplot2::element_text(size = 5),
    axis.title.x = ggplot2::element_text(size = 10),
    axis.title.y = ggplot2::element_text(size = 10),
    legend.text = ggplot2::element_text(size = 7)
  )
p
```

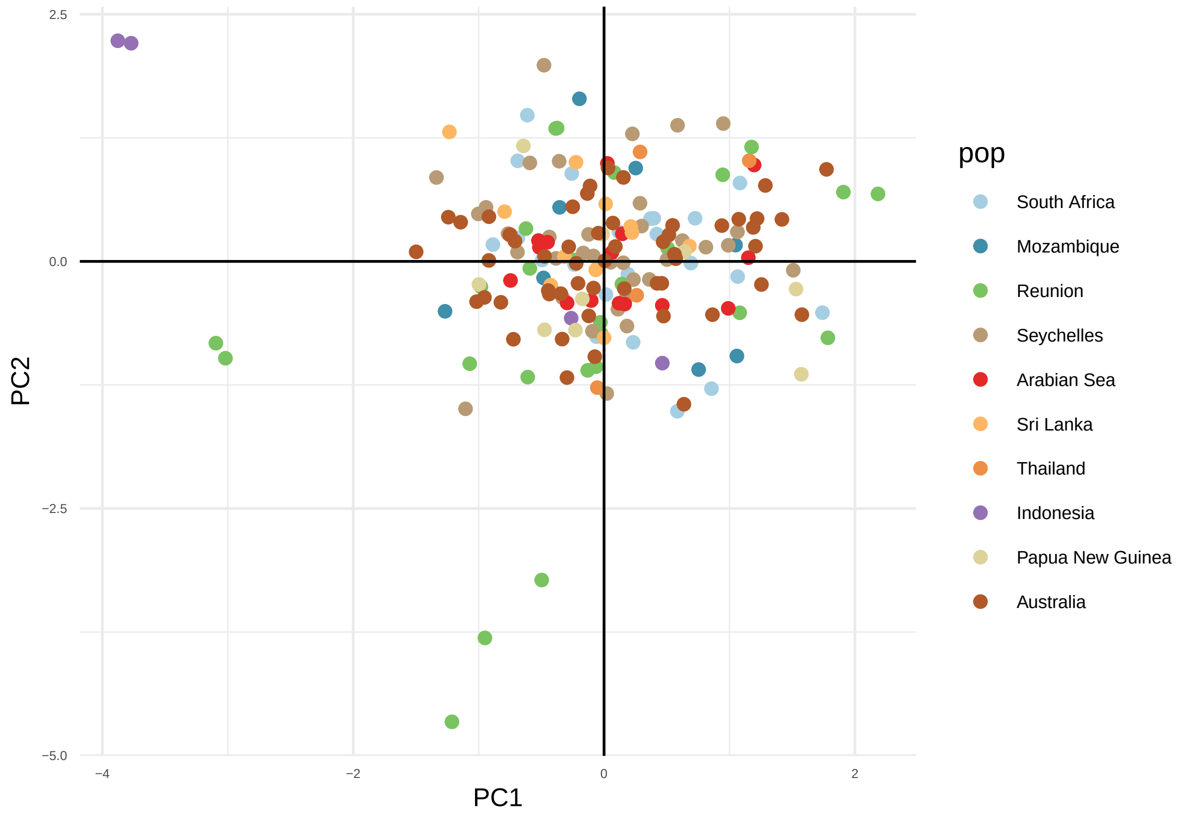


```r
ggplot2::ggsave(p, filename = "DArTcap_IWP2_PCA1.png", width = 30,
                height = 15,units = "cm")


p <-
  ggplot2::ggplot(BS.pca.scores, ggplot2::aes(x = PC1, y = PC3, colour = pop)) +
```

```
  ggplot2::geom_point(size = 2) + ggplot2::scale_color_manual(values = cols) +
  ggplot2::geom_hline(yintercept = 0) + ggplot2::geom_vline(xintercept = 0) +
  ggplot2::theme_minimal() +
  ggplot2::theme(
    axis.text = ggplot2::element_text(size = 5),
    axis.title.x = ggplot2::element_text(size = 10),
    axis.title.y = ggplot2::element_text(size = 10),
    legend.text = ggplot2::element_text(size = 7)
  )
p
```

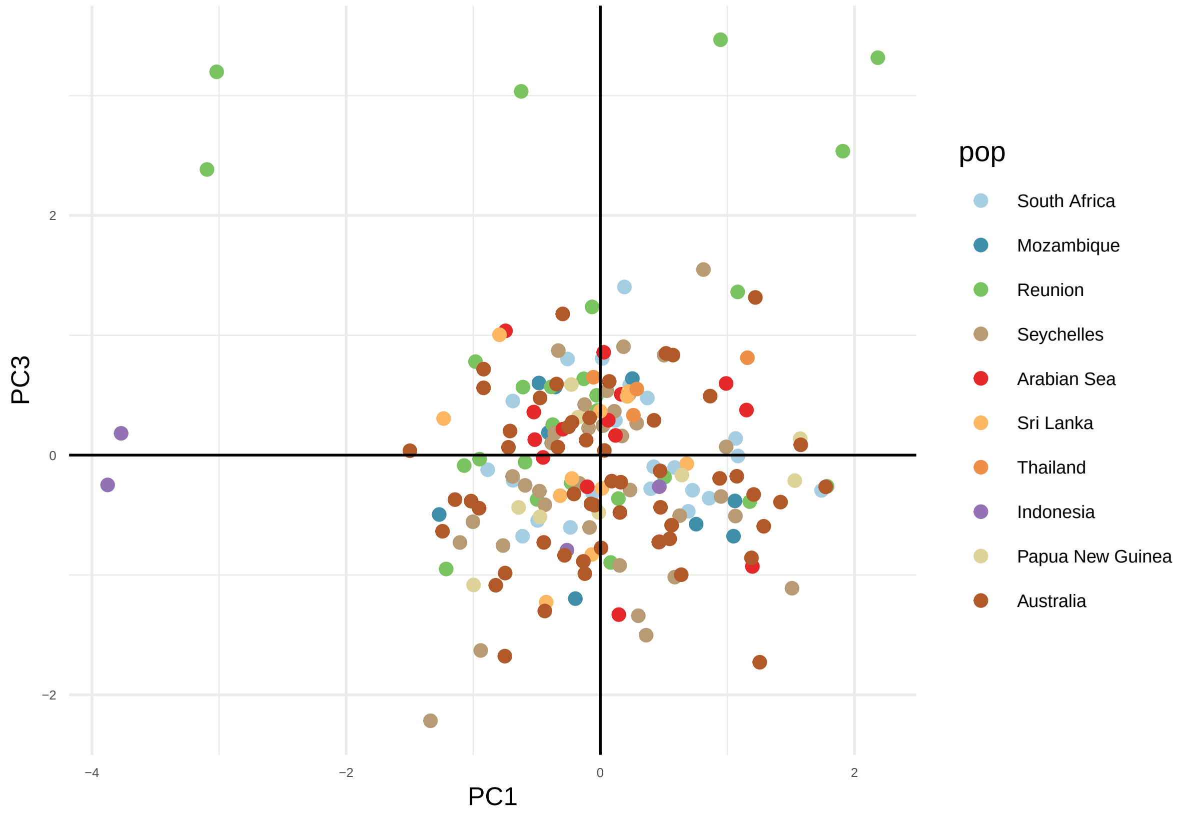


```
ggplot2::ggsave(p, filename = "DArTcap_IWP2_PCA2.png", width = 30,
                height = 15,units = "cm")

p <-
  ggplot2::ggplot(BS.pca.scores, ggplot2::aes(x = PC2, y = PC3, colour = pop)) +
  ggplot2::geom_point(size = 2) + ggplot2::scale_color_manual(values = cols) +
  ggplot2::geom_hline(yintercept = 0) + ggplot2::geom_vline(xintercept = 0) +
  ggplot2::theme_minimal() +
  ggplot2::theme(
    axis.text = ggplot2::element_text(size = 5),
    axis.title.x = ggplot2::element_text(size = 10),
    axis.title.y = ggplot2::element_text(size = 10),
    legend.text = ggplot2::element_text(size = 7)
  )
p
```

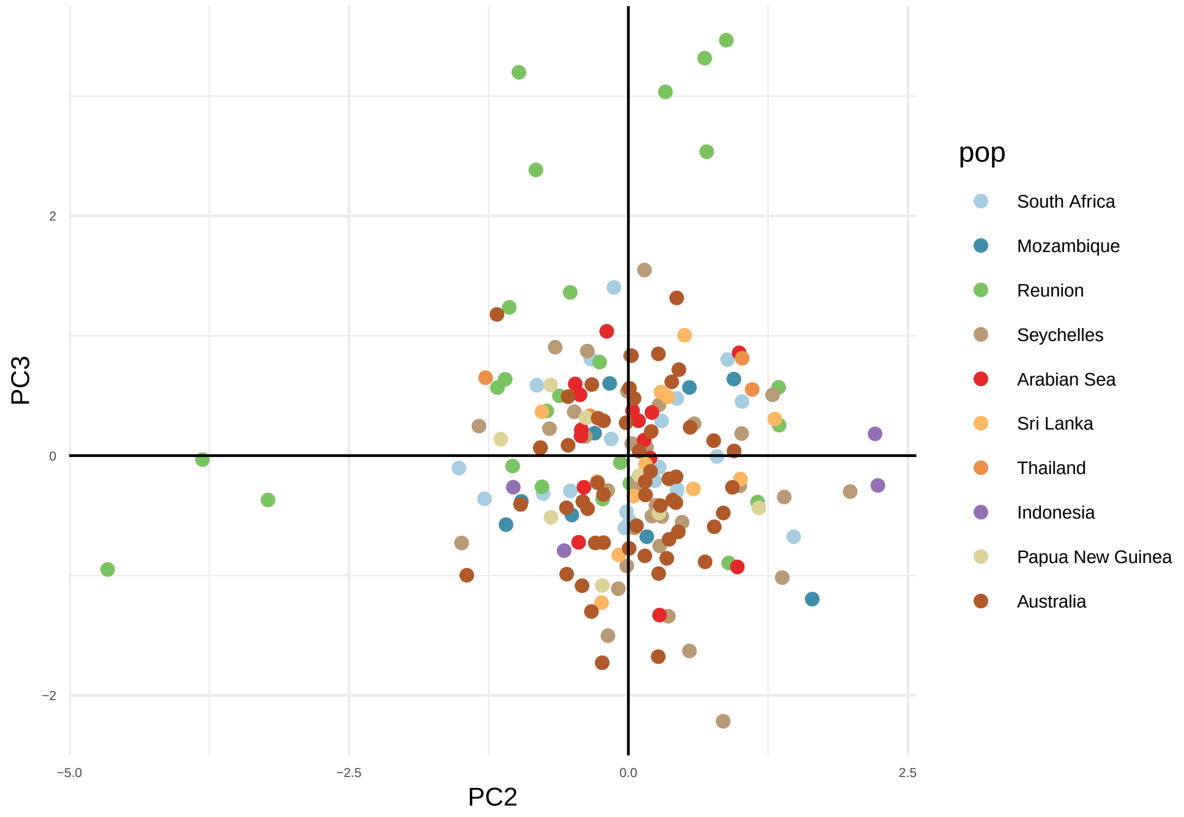


```
ggplot2::ggsave(p, filename = "DArTcap_IWP2_PCA3.png", width = 30,
                height = 15,units = "cm")


p <-
  ggplot2::ggplot(BS.pca.scores, ggplot2::aes(x = PC3, y = PC4, colour = pop)) +
  ggplot2::geom_point(size = 2) + ggplot2::scale_color_manual(values = cols) +
  ggplot2::geom_hline(yintercept = 0) + ggplot2::geom_vline(xintercept = 0) +
  ggplot2::theme_minimal() +
  ggplot2::theme(
    axis.text = ggplot2::element_text(size = 5),
    axis.title.x = ggplot2::element_text(size = 10),
    axis.title.y = ggplot2::element_text(size = 10),
    legend.text = ggplot2::element_text(size = 7)
  )
p
```

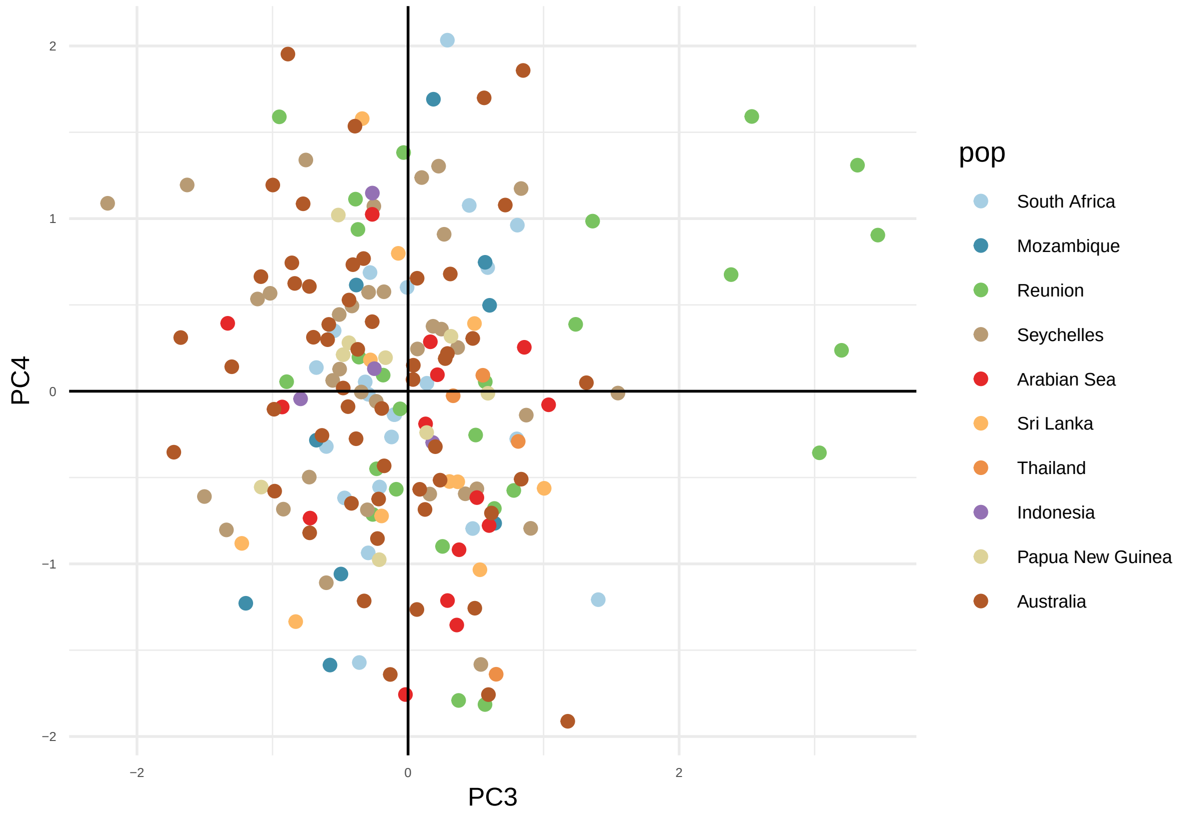


```r
ggplot2::ggsave(p, filename = "DArTcap_IWP2_PCA4.png", width = 30,
                height = 15,units = "cm")

p <-
  ggplot2::ggplot(BS.pca.scores, ggplot2::aes(x = PC3, y = PC5, colour = pop)) +
  ggplot2::geom_point(size = 2) + ggplot2::scale_color_manual(values = cols) +
  ggplot2::geom_hline(yintercept = 0) + ggplot2::geom_vline(xintercept = 0) +
  ggplot2::theme_minimal() +
  ggplot2::theme(
    axis.text = ggplot2::element_text(size = 5),
    axis.title.x = ggplot2::element_text(size = 10),
    axis.title.y = ggplot2::element_text(size = 10),
    legend.text = ggplot2::element_text(size = 7)
  )
p
```

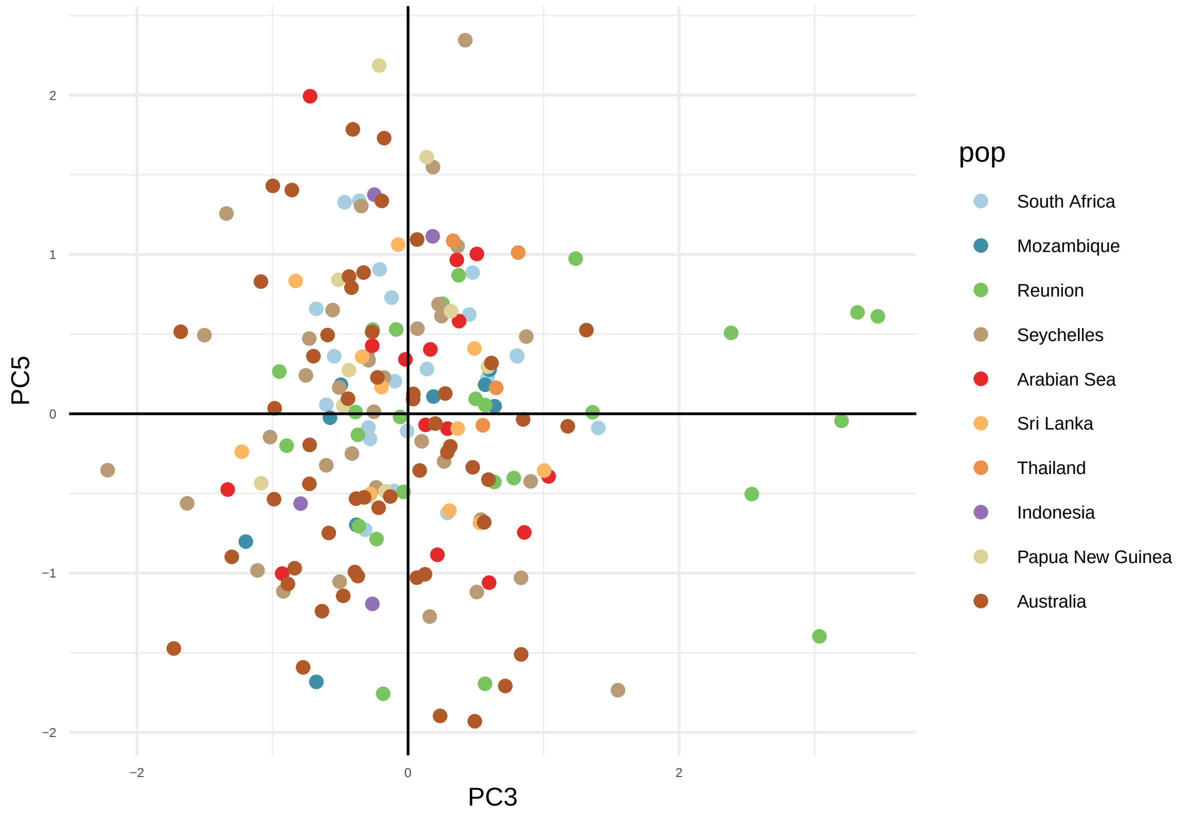


```
ggplot2::ggsave(p, filename = "DArTcap_IWP2_PCA5.png", width = 30,
                height = 15,units = "cm")
```

## 12.9 DAPC

### 12.9.1 Optimal number of clusters with neutral markers

```
set.seed(124)
grp <-
  adegenet::find.clusters(
    BS.dartcap.IWP2.gl,
    max.n.clust = 10,
    n.pca = adegenet::nInd(BS.dartcap.IWP2.gl) / 3,
    stat = "BIC",
    parallel = TRUE,
    n.cores = parallel::detectCores() - 1
  )

save(grp, file = "DArTcap_IWP2_grp.Rdata")
```

```
load("DArTcap_IWP2_grp.Rdata")

y <- as.numeric(grp$Kstat)
x <- 1:10
data <- data.frame(x,y,stringsAsFactors = F)
plot <- ggplot2::ggplot(data, ggplot2::aes(x,y)) +
  ggplot2::geom_point(size = 5, shape = 1, color = "blue") +
  ggplot2::geom_line(size = 1,color = "blue") +
  ggplot2::scale_x_continuous(name = waiver(),
                              breaks = seq(from = 0,
                                           to = nrow(BS.dartcap.IWP2.gl) - 1,
```

```
                                               by = 1)) +
  ggplot2::labs(subtitle = "",
                y = "Bayesian Information Criterion",
                x = "Number of clusters",
                title = "",
                caption = "") +
  ggplot2::theme_classic() +
  ggplot2::theme(
    axis.text = ggplot2::element_text(size = 5),
    axis.title.x = ggplot2::element_text(size = 20,vjust = 0, hjust = 0.5),
    axis.title.y = ggplot2::element_text(size = 20,vjust = 2, hjust = 0.5))
print(plot)
```

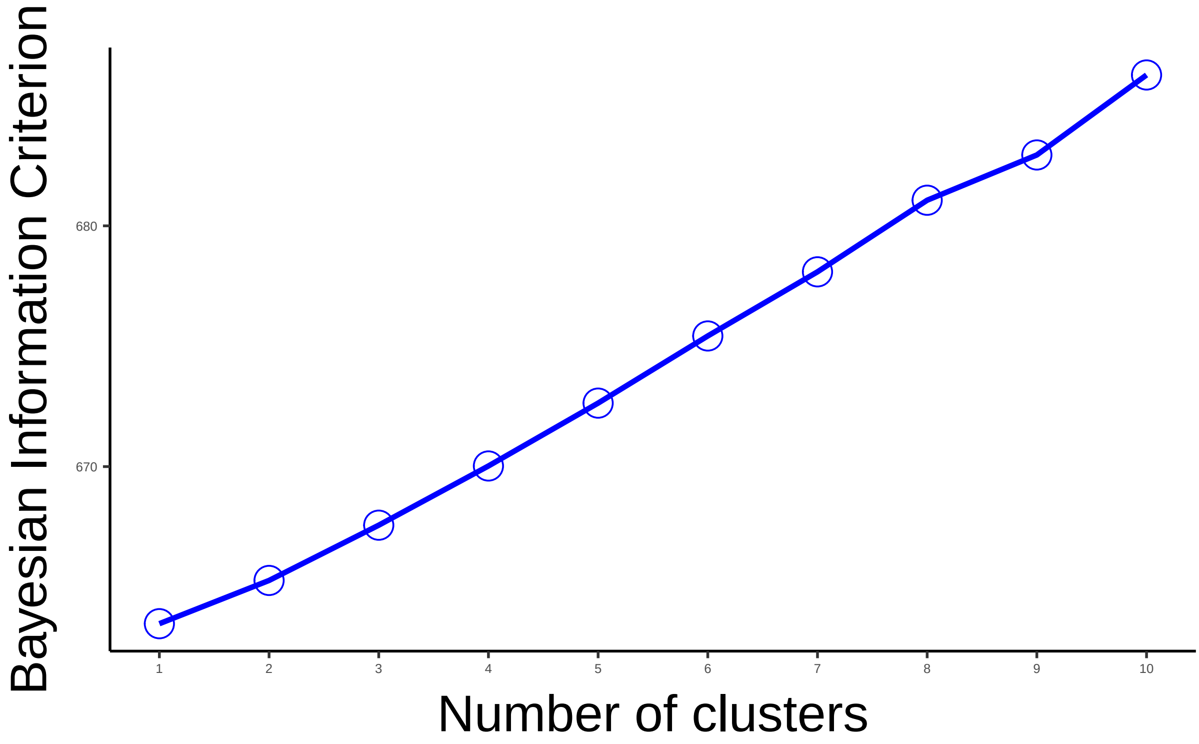


```
ggplot2::ggsave(plot, filename = "DArTcap_IWP2_grp.png", width = 30,
                height = 15,units = "cm")
```

### 12.9.2 Cross-validation to identify the optimal number of PCs

Uses a training - holdout set of individuals to check how reliable individuals can be assigned.

```
set.seed(124)
x <- BS.dartcap.IWP2.gl
x$loc.names <- x$other$loc.metrics$MARKERS

xval <-
  adegenet::xvalDapc(
  tab(x, NA.method = "mean"),
  adegenet::pop(x),
  n.pca = seq(1, adegenet::nInd(x), by = 50),
  training.set = 0.9,
  result = "groupMean",
  center = TRUE,
```

```r
  scale = FALSE,
  n.rep = 1000,
  parallel = "multicore",
  ncpus = 1,
  xval.plot = TRUE
  )
print(xval[2:6])
PC <- as.numeric(xval$`Number of PCs Achieving Lowest MSE`)

#refine the analysis
set.seed(124)
png(file="DArTcap_IWP2_xval.png")
xval <-
  adegenet::xvalDapc(
  tab(x, NA.method = "mean"),
  adegenet::pop(x),
  n.pca = (PC - 15):(PC + 15),
  n.rep = 1000,
  parallel = "multicore",
  ncpus = 1,
  xval.plot = TRUE
  )
dev.off()
save(xval, file = "DArTcap_IWP2_xval.rdata")
```

```r
load("DArTcap_IWP2_xval.rdata")
print(xval[2:6])
PC <- as.numeric(xval$`Number of PCs Achieving Lowest MSE`)
```

```
## $`Median and Confidence Interval for Random Chance`
##       2.5%        50%      97.5%
## 0.06180334 0.09925630 0.13873901
##
## $`Mean Successful Assignment by Number of PCs of PCA`
##        36        37        38        39        40        41        42        43
## 0.1930960 0.2027357 0.1990421 0.2038611 0.2115881 0.2072631 0.2076766 0.2035103
##        44        45        46        47        48        49        50        51
## 0.2019516 0.2055075 0.2049619 0.2057060 0.2084857 0.2061317 0.2061722 0.2070675
##        52        53        54        55        56        57        58        59
## 0.2073901 0.2032528 0.2099532 0.2065258 0.2039921 0.2058381 0.2079246 0.2048988
##        60        61        62        63        64        65        66
## 0.2099571 0.2044837 0.2062091 0.2048567 0.2004135 0.1984770 0.2010897
##
## $`Number of PCs Achieving Highest Mean Success`
## [1] "40"
##
## $`Root Mean Squared Error by Number of PCs of PCA`
##        36        37        38        39        40        41        42        43
## 0.8095453 0.8002350 0.8037620 0.7990092 0.7914135 0.7955968 0.7953047 0.7994862
##        44        45        46        47        48        49        50        51
## 0.8008700 0.7974081 0.7978982 0.7972406 0.7942725 0.7966445 0.7966600 0.7956200
##        52        53        54        55        56        57        58        59
## 0.7955904 0.7998644 0.7930704 0.7964852 0.7991029 0.7970035 0.7948072 0.7979593
##        60        61        62        63        64        65        66
## 0.7929512 0.7983287 0.7964667 0.7979294 0.8022374 0.8045400 0.8018440
##
## $`Number of PCs Achieving Lowest MSE`
## [1] "40"
```

```r
dapc.all.object.names <- c()
for (K in 2:10) {
  set.seed(124)
  grp <- adegenet::find.clusters(BS.dartcap.IWP2.gl, n.clust = K, n.pca = PC)
  set.seed(124)
  dapc.all.neutral <- adegenet::dapc(BS.dartcap.IWP2.gl, grp$grp, n.da = K - 1,
                                   n.pca = PC)
  assign(paste0("dapc.all.", K),value = dapc.all.neutral)
  dapc.all.object.names <- c(dapc.all.object.names,
                                   paste0("dapc.all.", K))
}
save(list = dapc.all.object.names, dapc.all.object.names,
     file = "DArTcap_IWP2_DAPC.Rdata")
```

### 12.9.3 DAPC barplot

Group individuals according to DAPC posterior membership.

```r
load("DArTcap_IWP2_DAPC.Rdata")
for (K in 2:10) {
  colour <- colours.prior[1:K]
  dapc <- get(dapc.all.object.names[K - 1])
  post <- as.matrix(dapc$posterior)
  colnames(post) <- paste0("Group", 1:nlevels(dapc$grp))
  locations <- BS.dartcap.IWP2.gl$pop

  plot.dapc.FDD(x = post,
              locations = locations,
              colour = colour,
              region.lwd = 1,
              plotTitle = paste0("DAPC: all Bull Sharks - subset Australia\n for K=",
                                K, " & PC=", PC, sep = ""))
  dev.print(
    device = png,
    file = paste0("DArTcap_IWP2_DAPC_barplot_K", K, ".png"),
    res = 300,
    width = 30,
    height = 15,
    units = "cm")
}
```

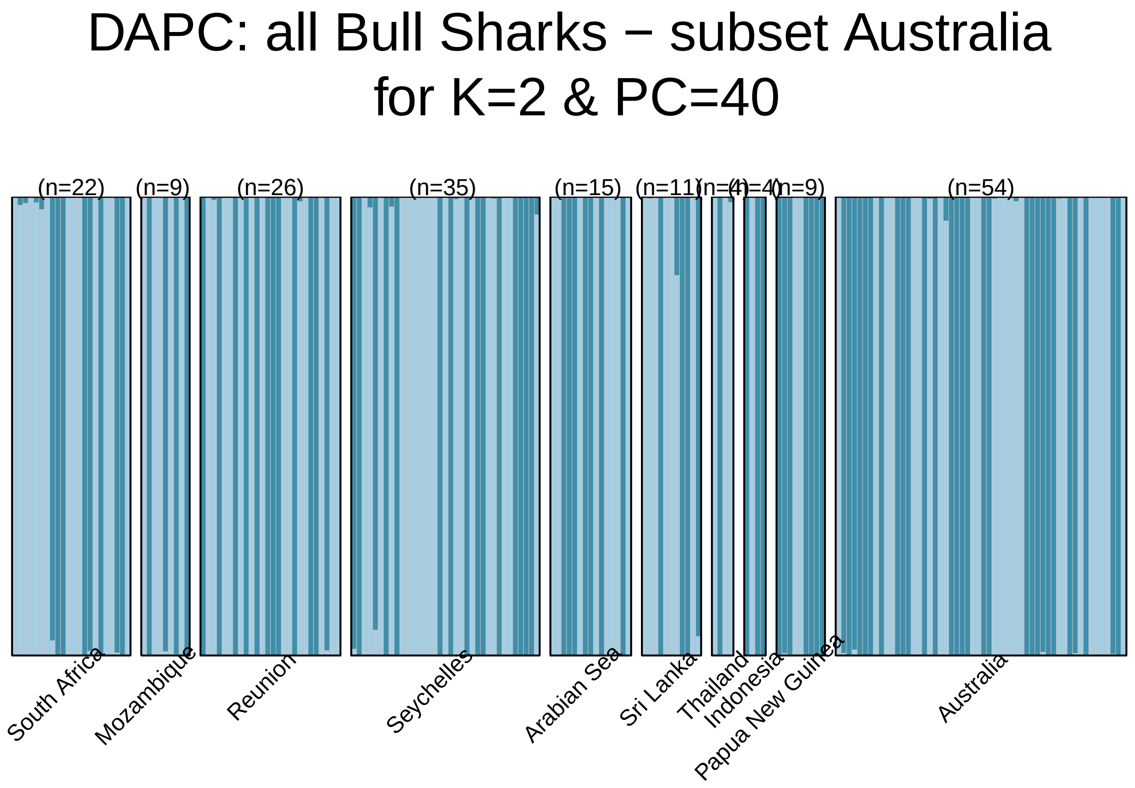
DAPC: all Bull Sharks − subset Australia
for K=2 & PC=40
(n=22)
(n=9)
(n=26)
(n=35)
(n=15)
(n=11)
(n=4)
(n=4)
(n=9)
(n=54)
South Africa
Mozambique
Reunion
Seychelles
Arabian Sea
Sri Lanka
Thailand
Indonesia
Papua New Guinea
Australia

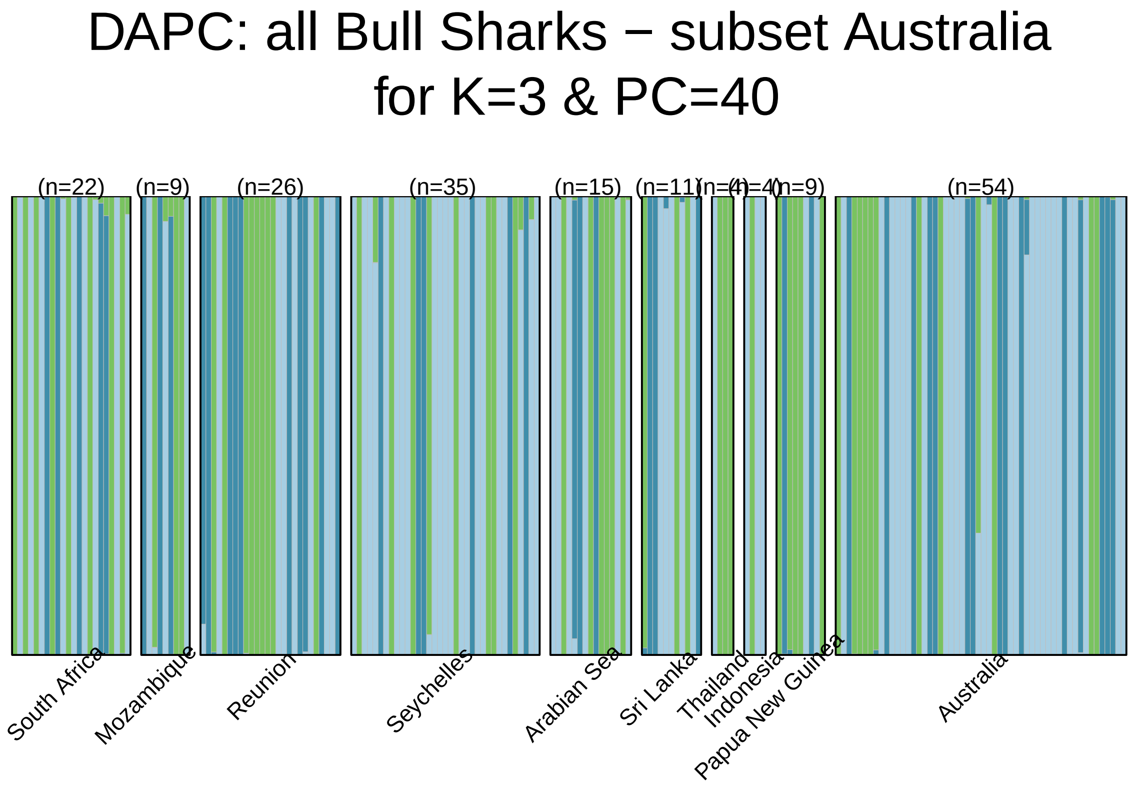
DAPC: all Bull Sharks − subset Australia
for K=3 & PC=40
(n=22)
(n=9)
(n=26)
(n=35)
(n=15)
(n=11)
(n=4)
(n=4)
(n=9)
(n=54)
South Africa
Mozambique
Reunion
Seychelles
Arabian Sea
Sri Lanka
Thailand
Indonesia
Papua New Guinea
Australia

## DAPC: all Bull Sharks − subset Australia for K=4 & PC=40

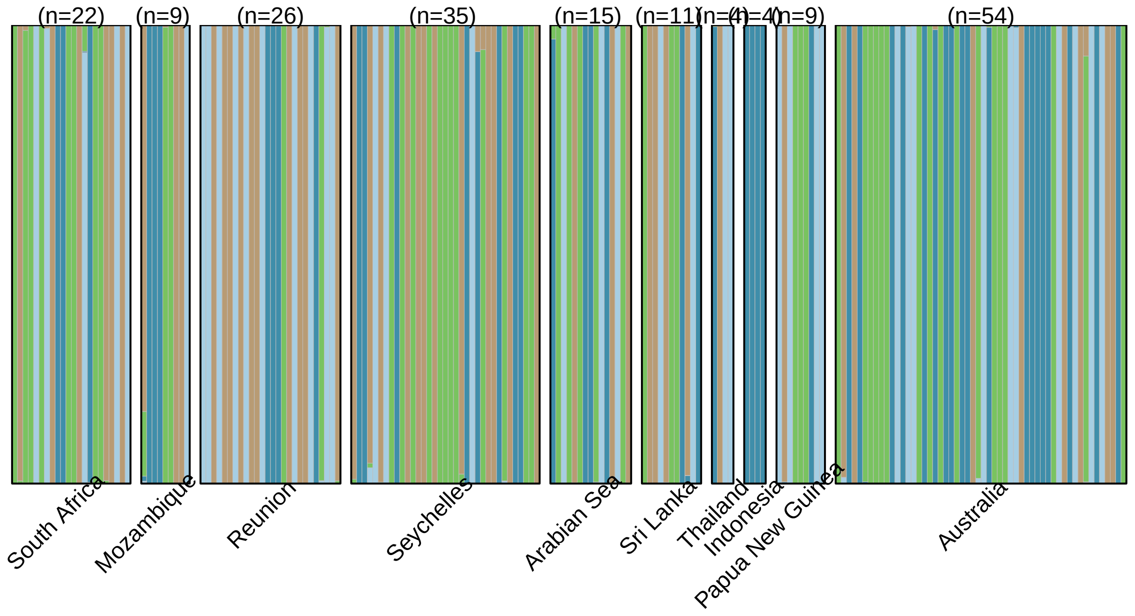


## DAPC: all Bull Sharks − subset Australia for K=5 & PC=40

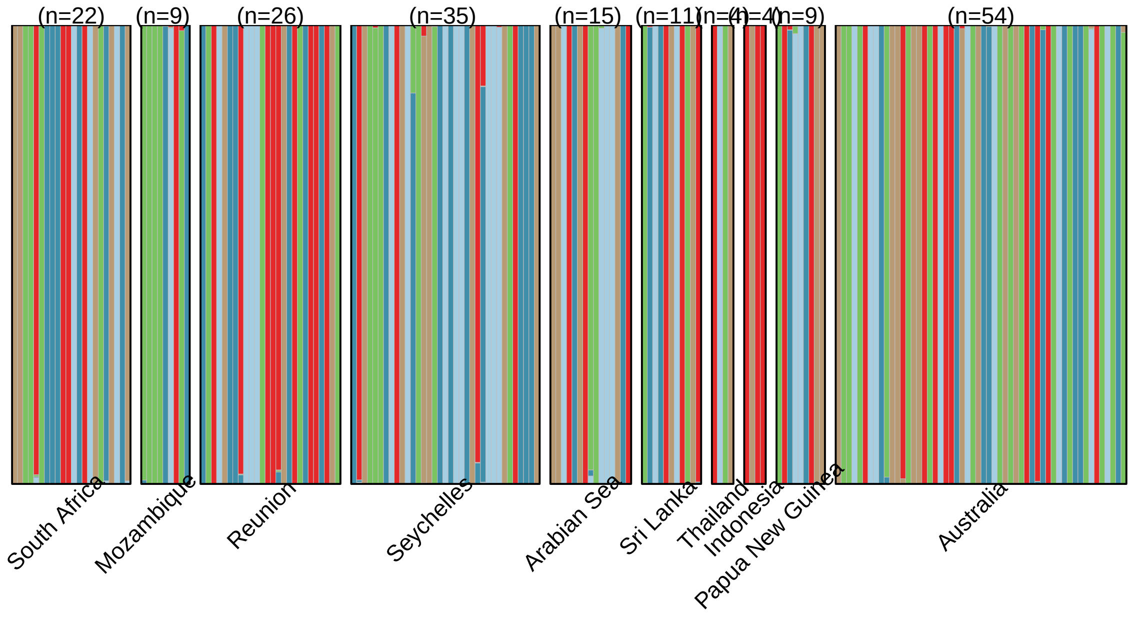

# DAPC: all Bull Sharks − subset Australia for K=6 & PC=40

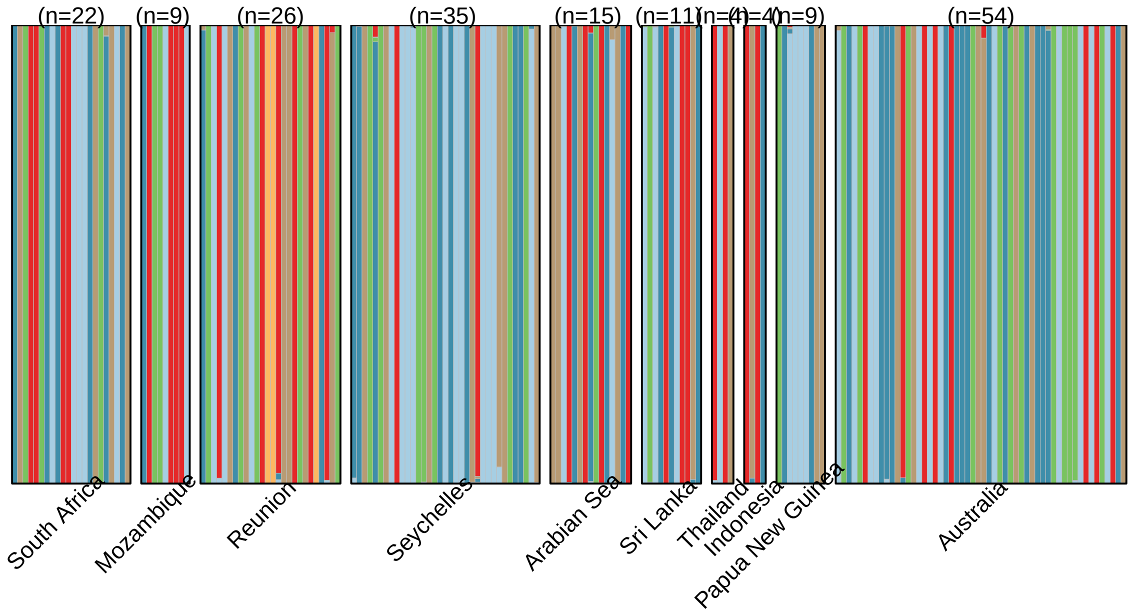


# DAPC: all Bull Sharks − subset Australia for K=7 & PC=40

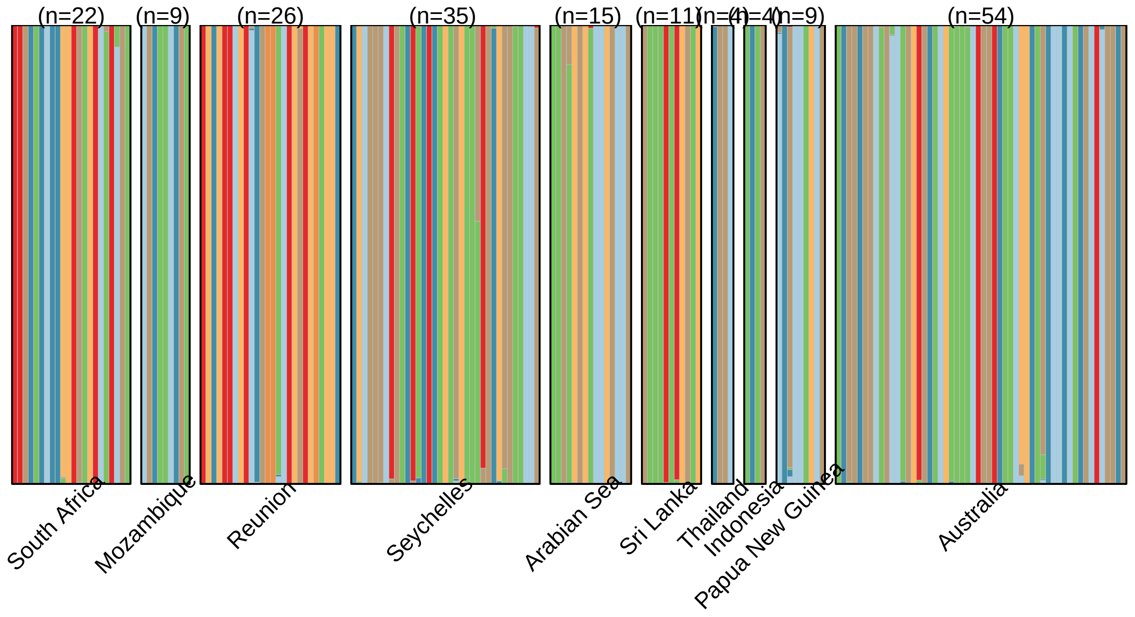

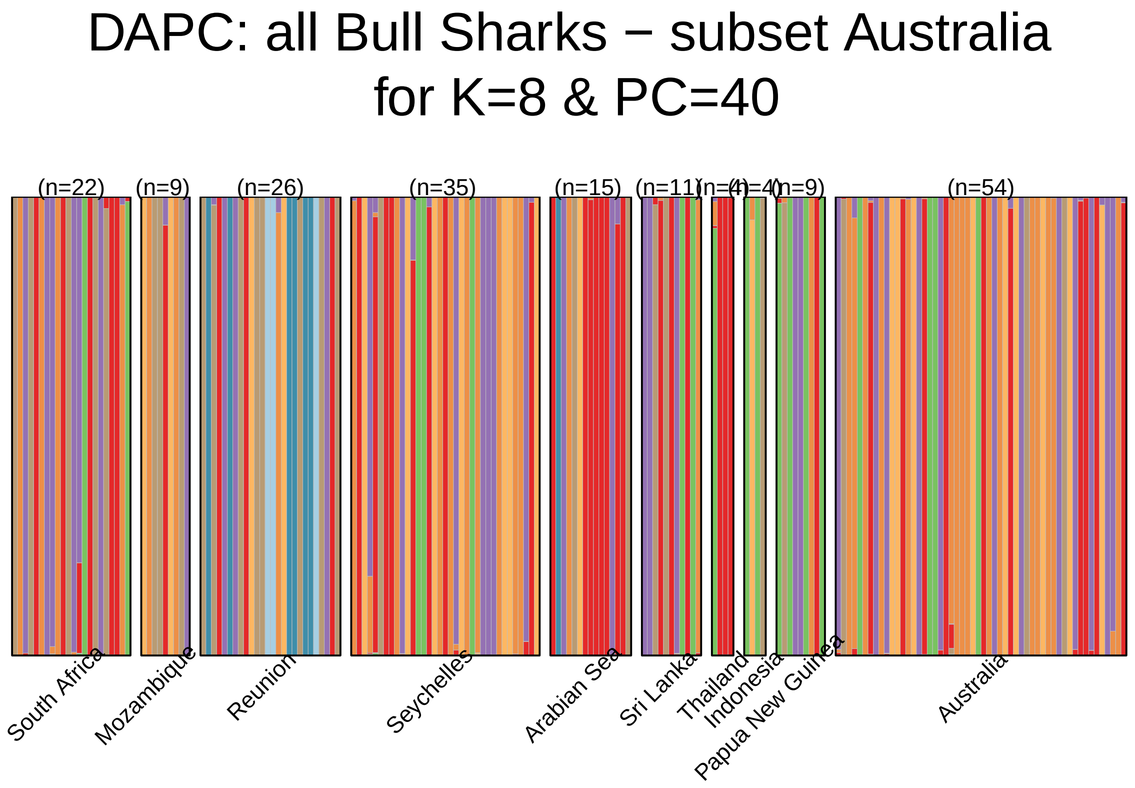
DAPC: all Bull Sharks − subset Australia
for K=8 & PC=40
(n=22)
(n=9)
(n=26)
(n=35)
(n=15)
(n=11)
(n=4)
(n=4)
(n=9)
(n=54)
South Africa
Mozambique
Reunion
Seychelles
Arabian Sea
Sri Lanka
Thailand
Indonesia
Papua New Guinea
Australia

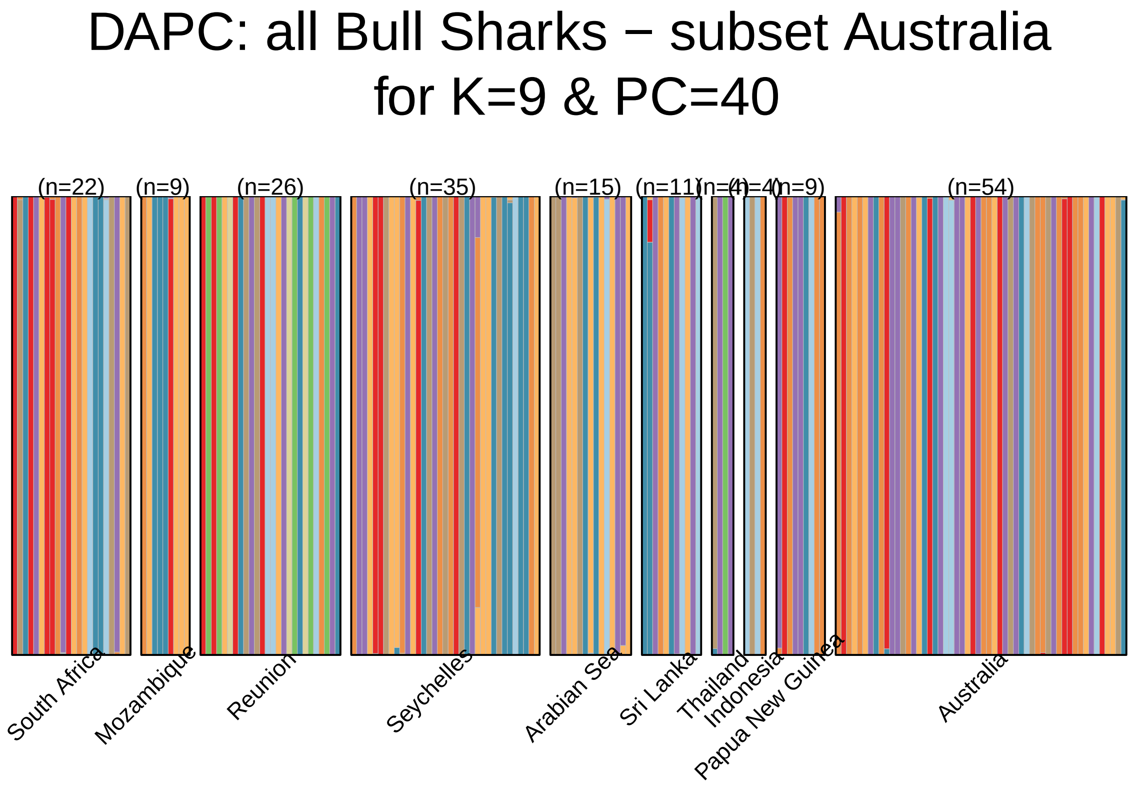
DAPC: all Bull Sharks − subset Australia
for K=9 & PC=40
(n=22)
(n=9)
(n=26)
(n=35)
(n=15)
(n=11)
(n=4)
(n=4)
(n=9)
(n=54)
South Africa
Mozambique
Reunion
Seychelles
Arabian Sea
Sri Lanka
Thailand
Indonesia
Papua New Guinea
Australia

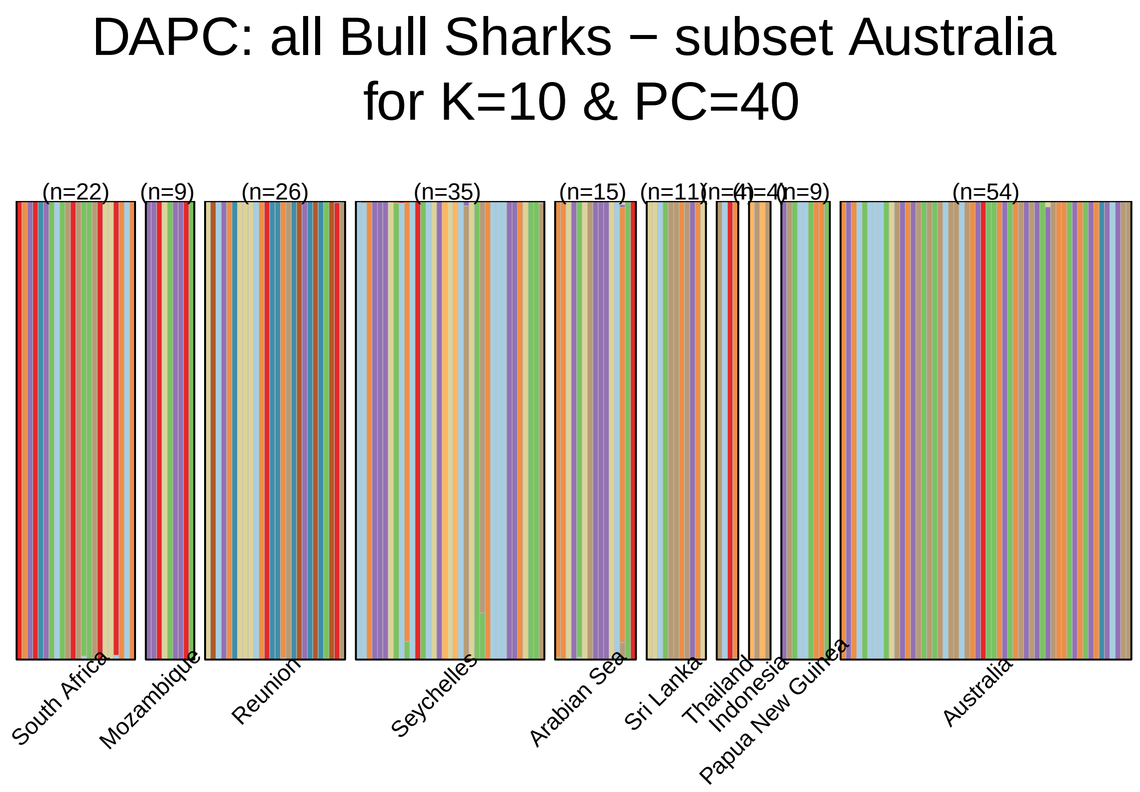


### 12.9.4 DAPC scatterplot

```
load("DArTcap_IWP2_DAPC.Rdata")

for (K in 3:10) {
  dapc <- get(dapc.all.object.names[K - 1])
  if (K == 2) {
    posi.leg <- "bottomright"
  } else {posi.leg <- "bottomleft"}
  dapc.plot <- ade4::scatter(dapc, grp = pop(BS.dartcap.IWP2.gl), cex = 2,
                             legend = TRUE, col = colours.10, clabel = FALSE,
                             posi.leg = posi.leg, scree.pca = FALSE,
                             posi.pca = "topleft", cleg = 0.75, xax = 1,
                             yax = 2, inset.solid = 0.3)
  dev.print(
    device = png,
    file = paste0("DArTcap_IWP2_DAPC_scatterplot_K", K, ".png"),
    width = 30,
    height = 15,
    units = "cm",
    res = 300
  )
}
```

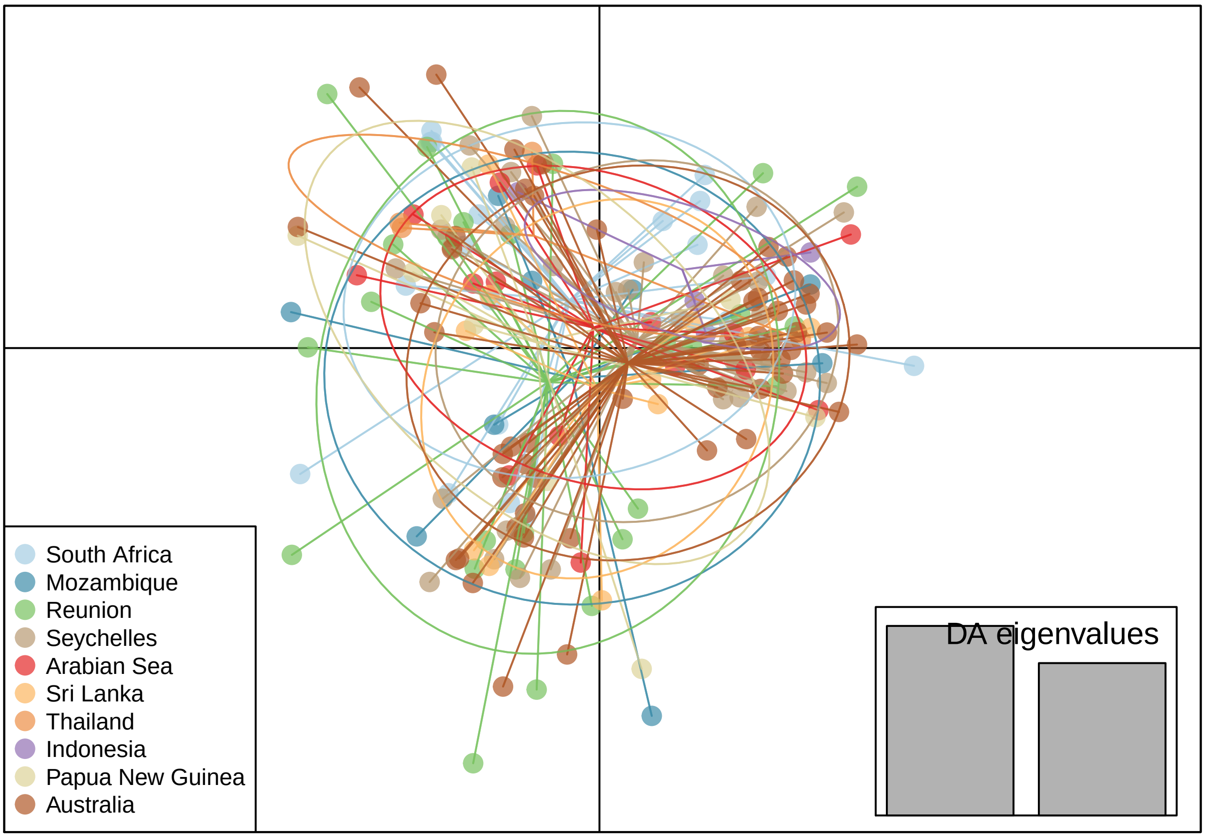
South Africa
Mozambique
Reunion
Seychelles
Arabian Sea
Sri Lanka
Thailand
Indonesia
Papua New Guinea
Australia
DA eigenvalues

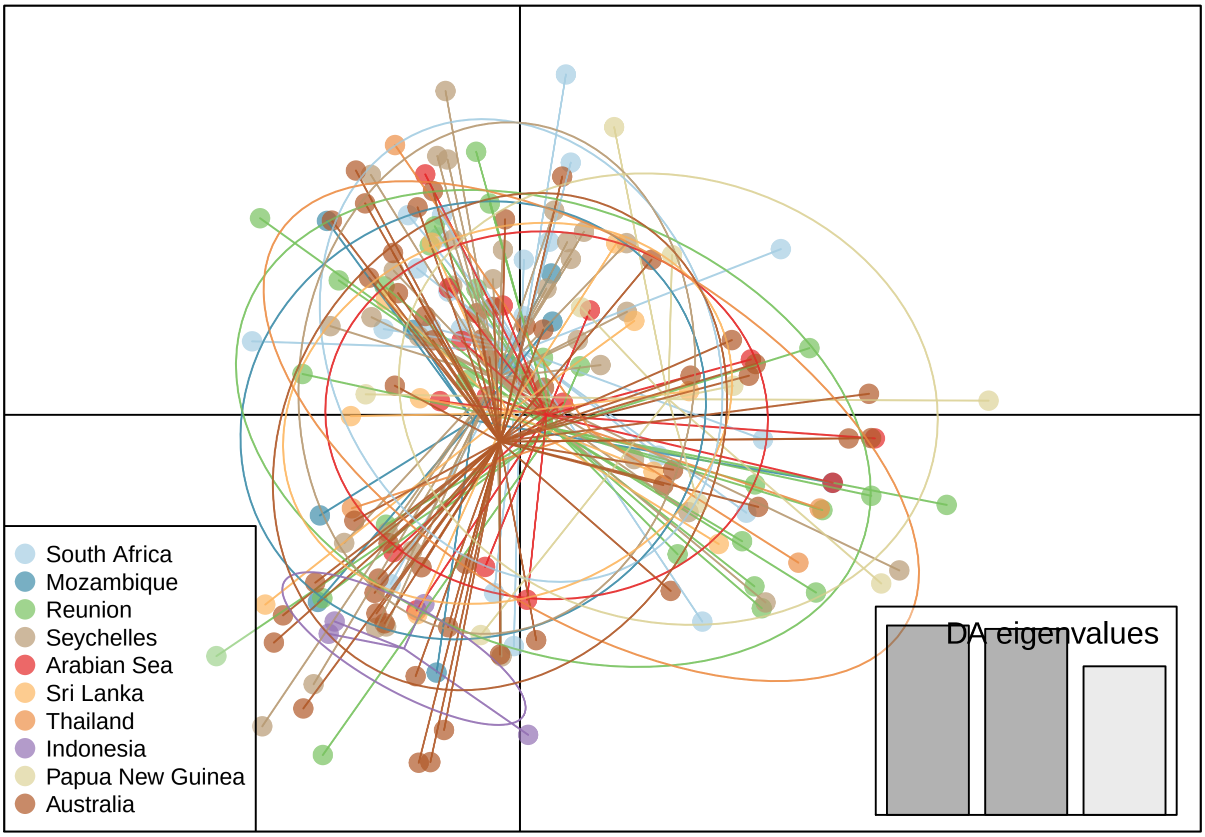
South Africa
Mozambique
Reunion
Seychelles
Arabian Sea
Sri Lanka
Thailand
Indonesia
Papua New Guinea
Australia
DA eigenvalues

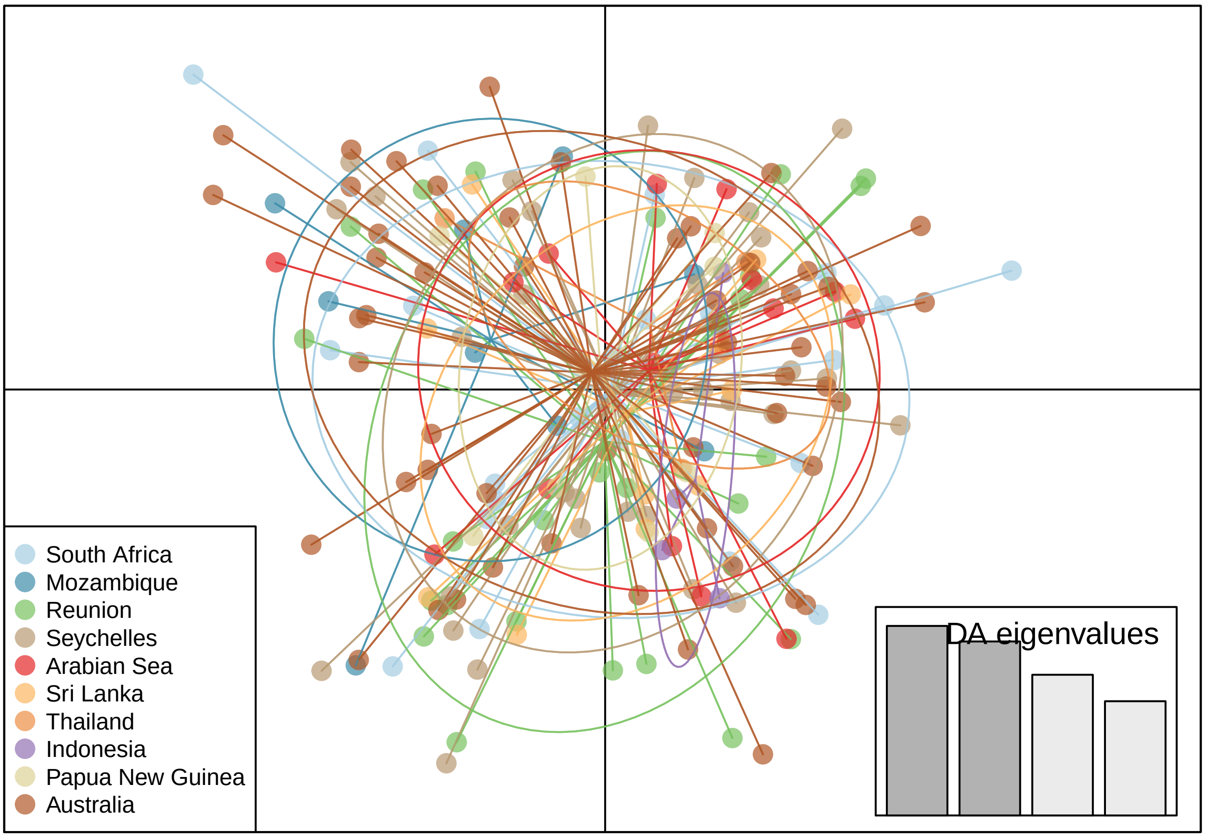
South Africa
Mozambique
Reunion
Seychelles
Arabian Sea
Sri Lanka
Thailand
Indonesia
Papua New Guinea
Australia
DA eigenvalues

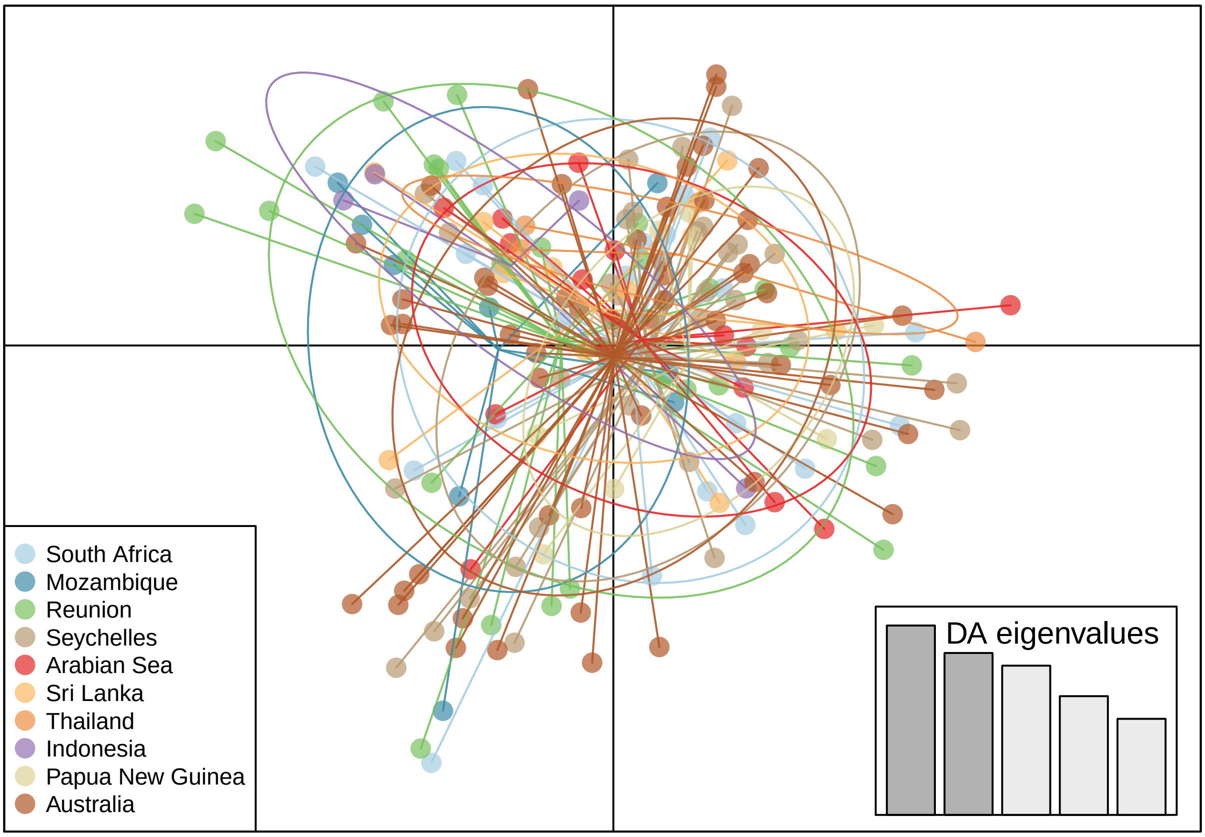
South Africa
Mozambique
Reunion
Seychelles
Arabian Sea
Sri Lanka
Thailand
Indonesia
Papua New Guinea
Australia
DA eigenvalues

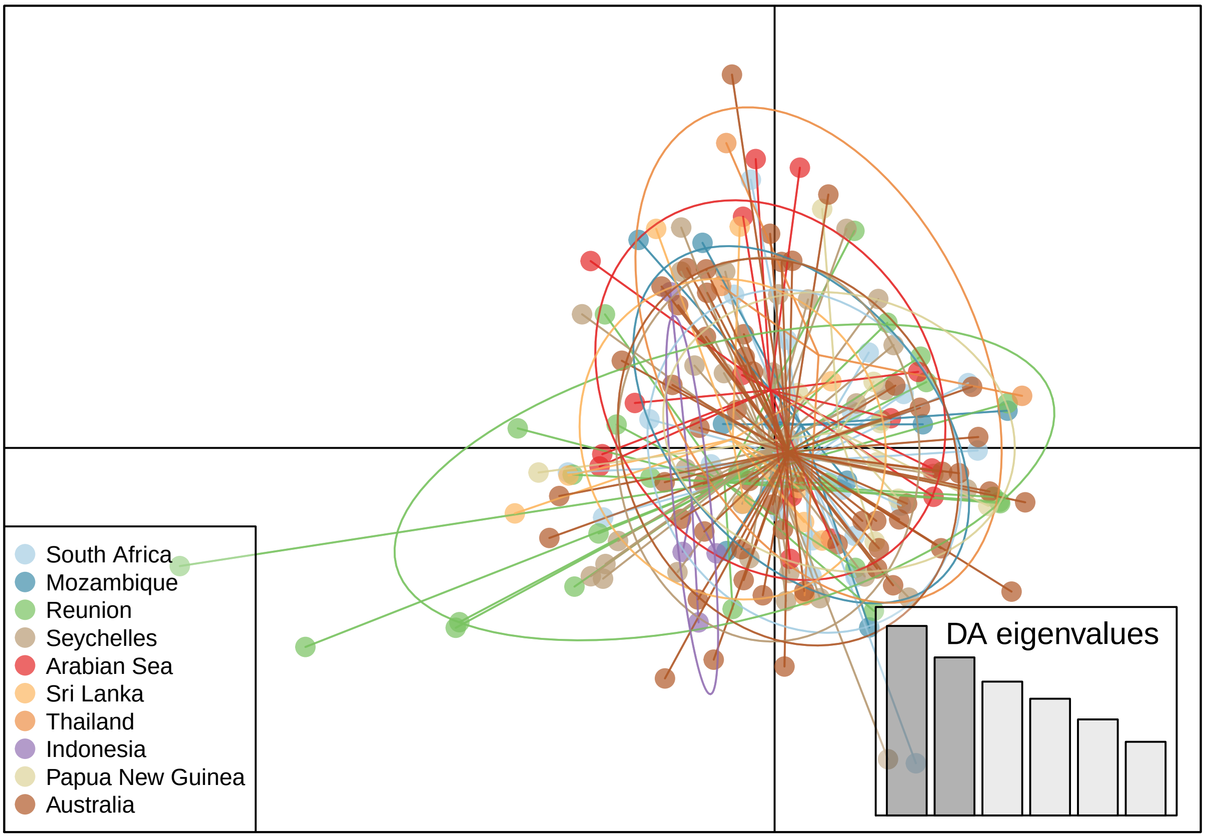

South Africa
Mozambique
Reunion
Seychelles
Arabian Sea
Sri Lanka
Thailand
Indonesia
Papua New Guinea
Australia
DA eigenvalues


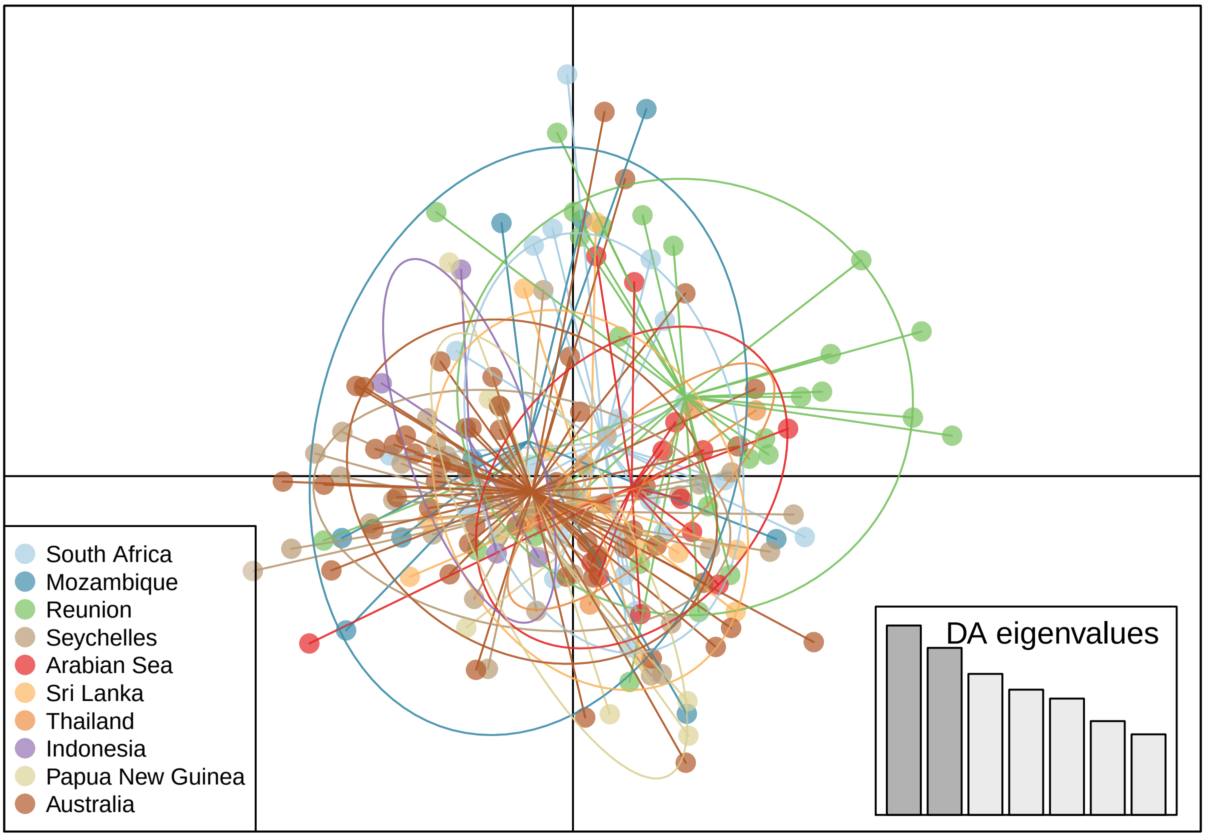

South Africa
Mozambique
Reunion
Seychelles
Arabian Sea
Sri Lanka
Thailand
Indonesia
Papua New Guinea
Australia
DA eigenvalues

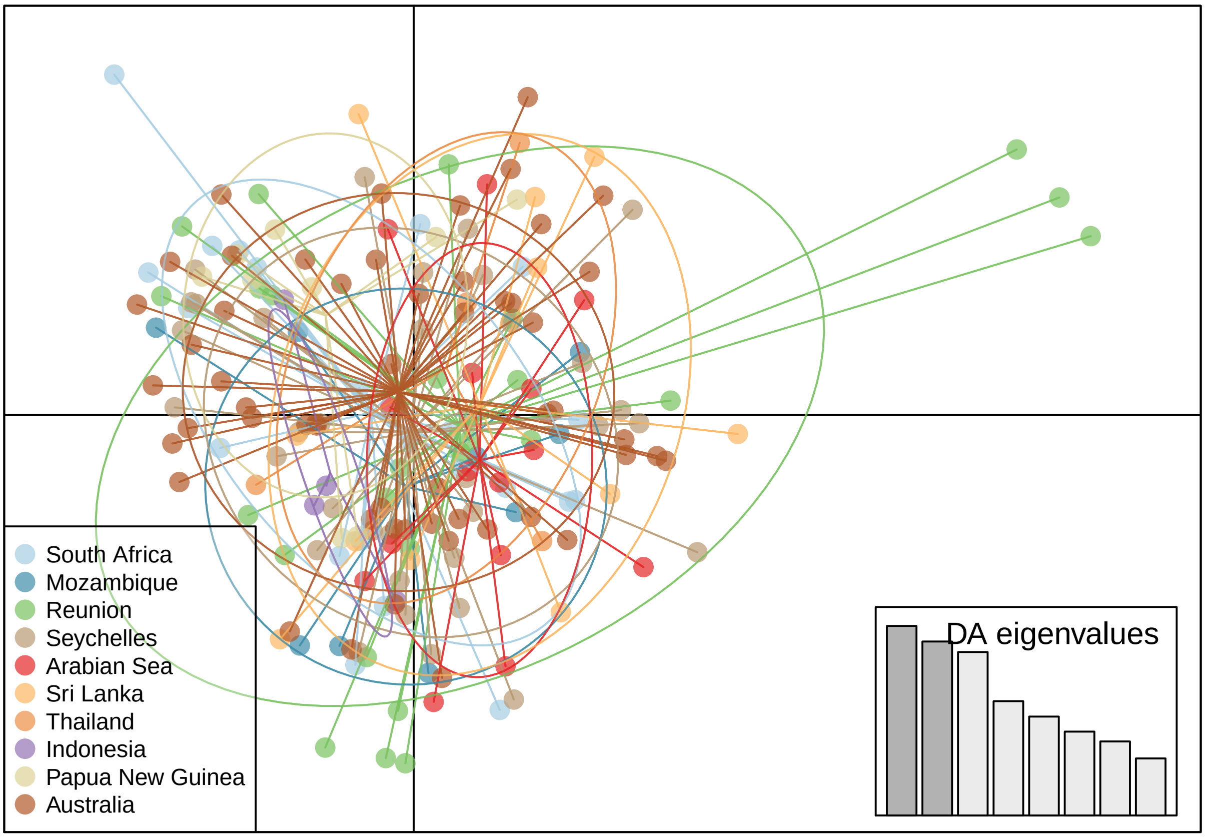


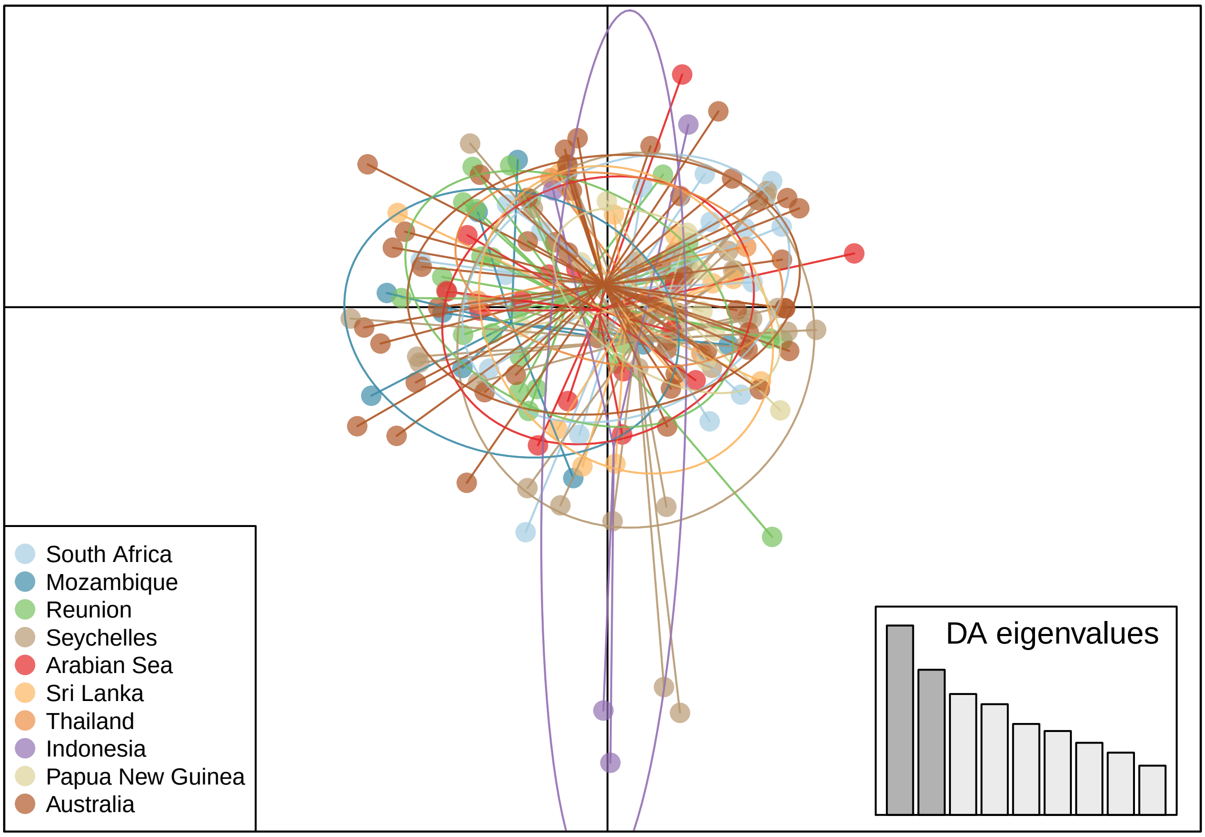


```
for (K in 2:10) {
  colour <- colours.prior[1:K]
  dapc <- get(dapc.all.object.names[K - 1])
  dapc.plot <- ade4::scatter(dapc, cex = 2, legend = TRUE, col = colour,
                             clabel = FALSE, posi.leg = "bottomleft",
```

```
                              scree.pca = FALSE,  posi.pca = "topleft",
                              cleg = 0.75, xax = 1, yax = 2, inset.solid = 0.3)
  dev.print(
    device = png,
    file = paste0("DArTcap_IWP2_DAPC_scatterplot_pergroups_K",
                  K,".png"),
    width = 30,
    height = 15,
    units = "cm",
    res = 300
  )
}
```

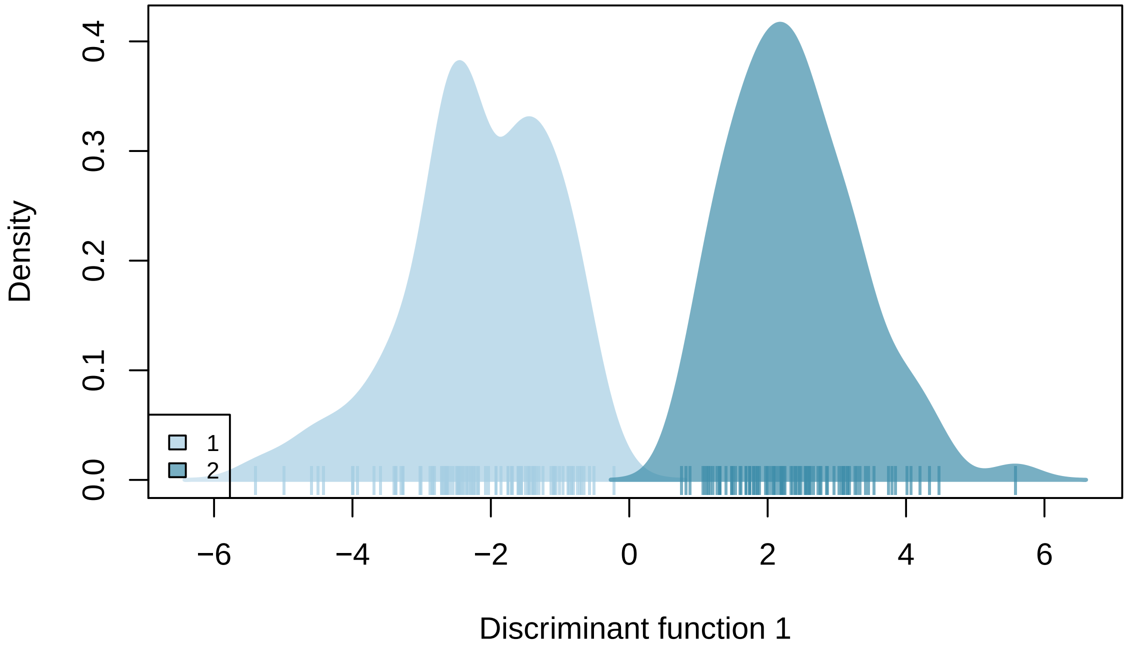

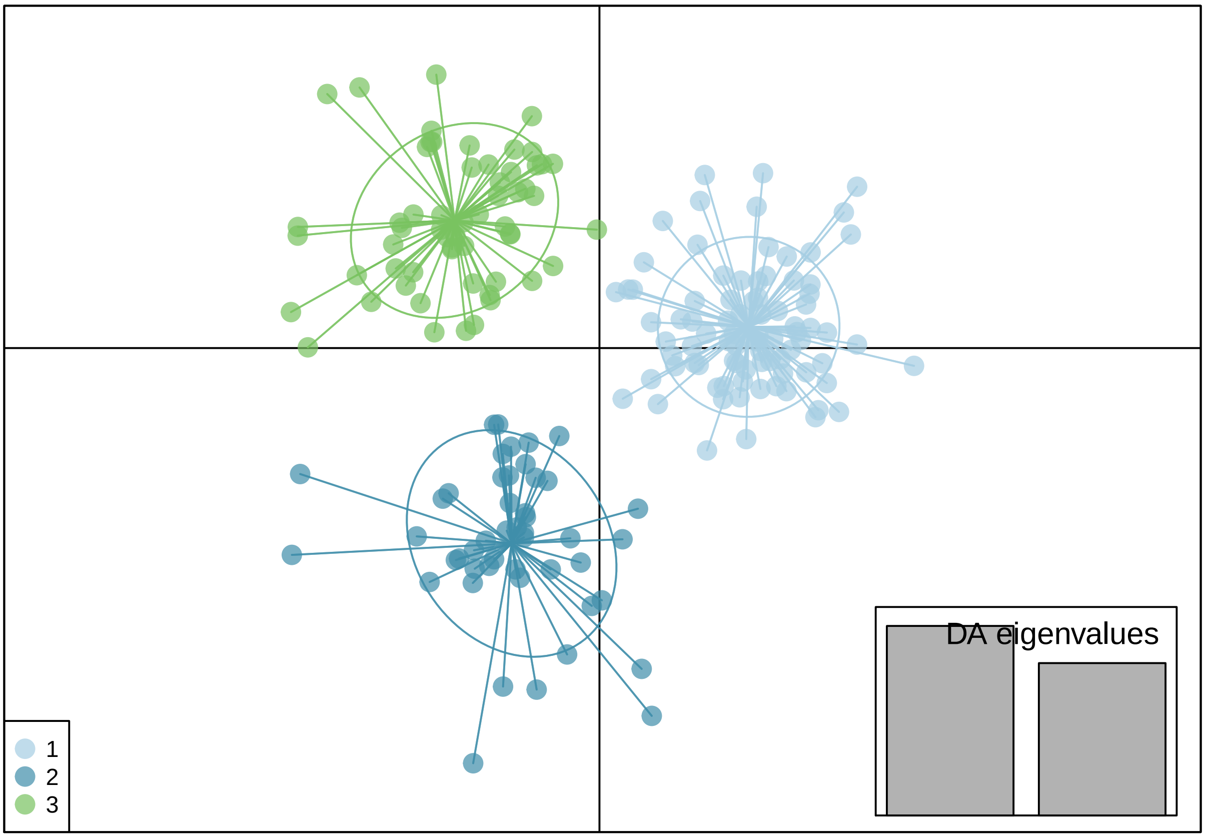
DA eigenvalues
1
2
3

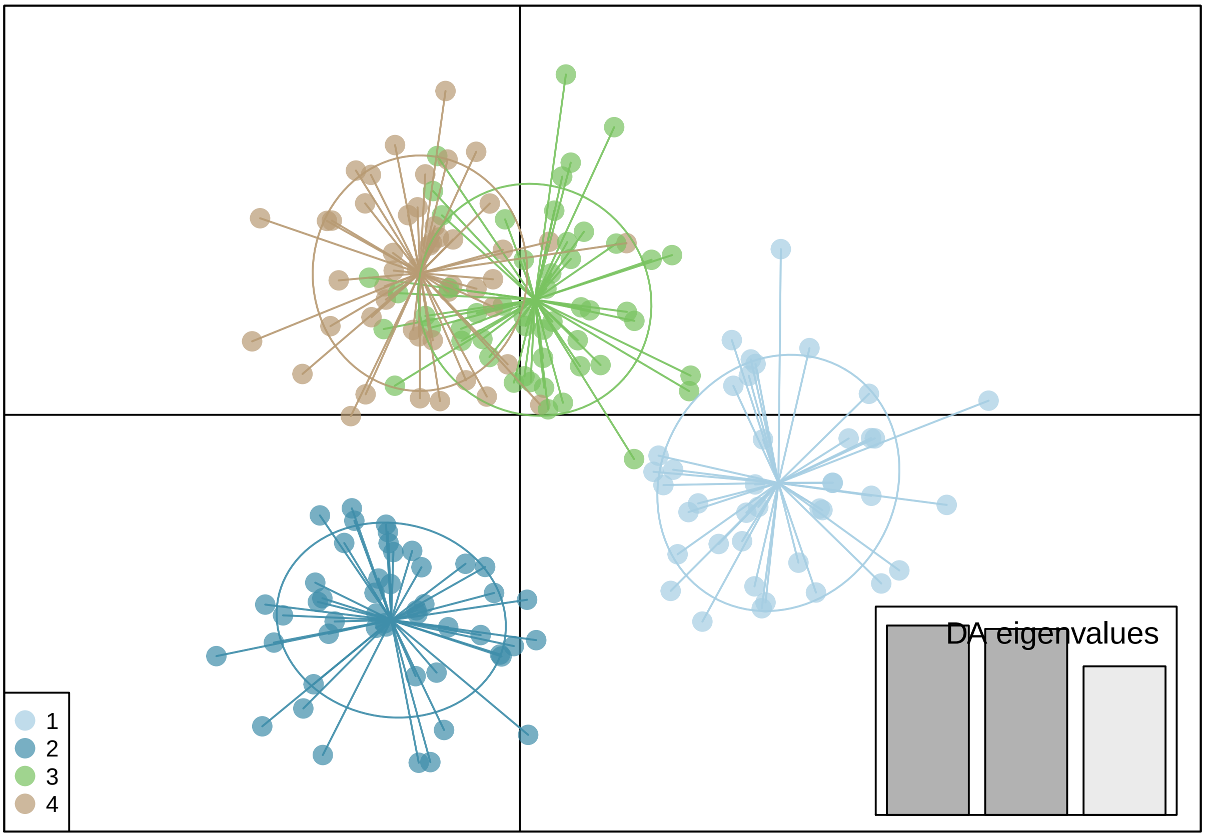
DA eigenvalues
1
2
3
4

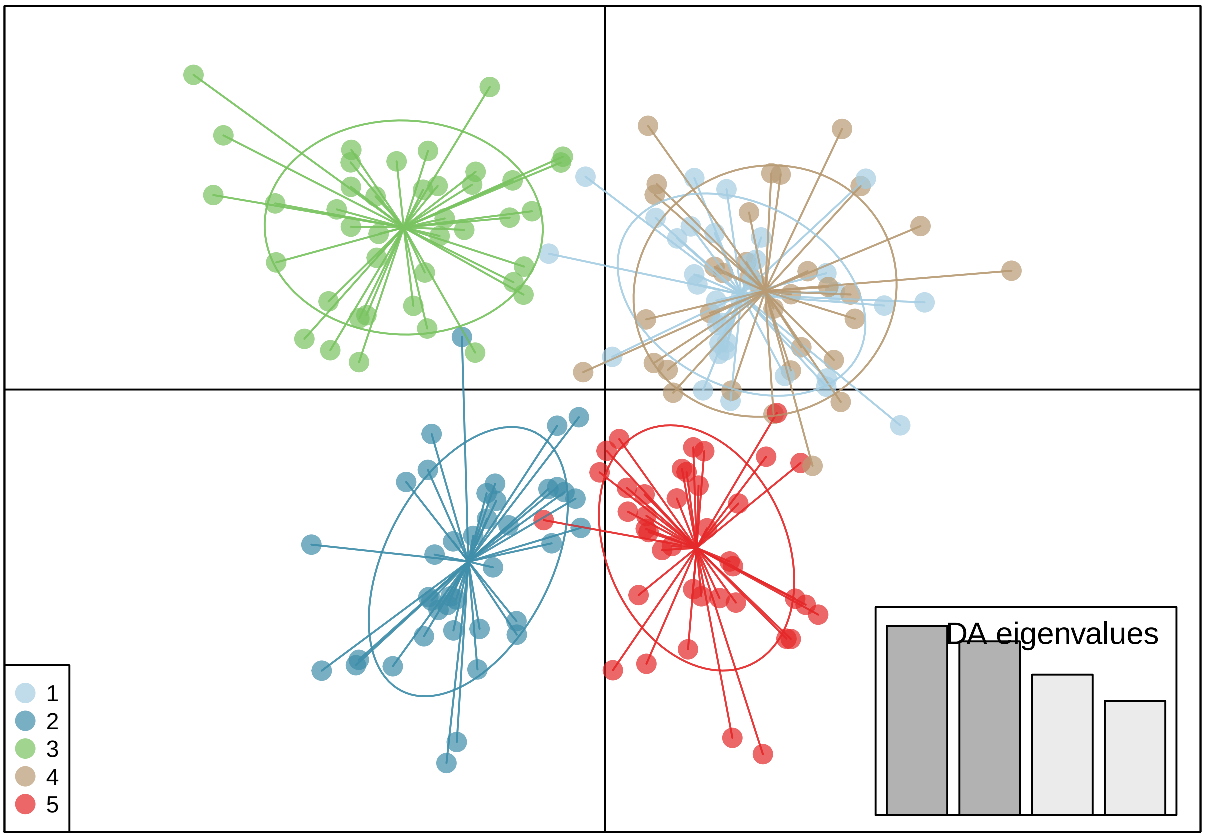

1
2
3
4
5
DA eigenvalues


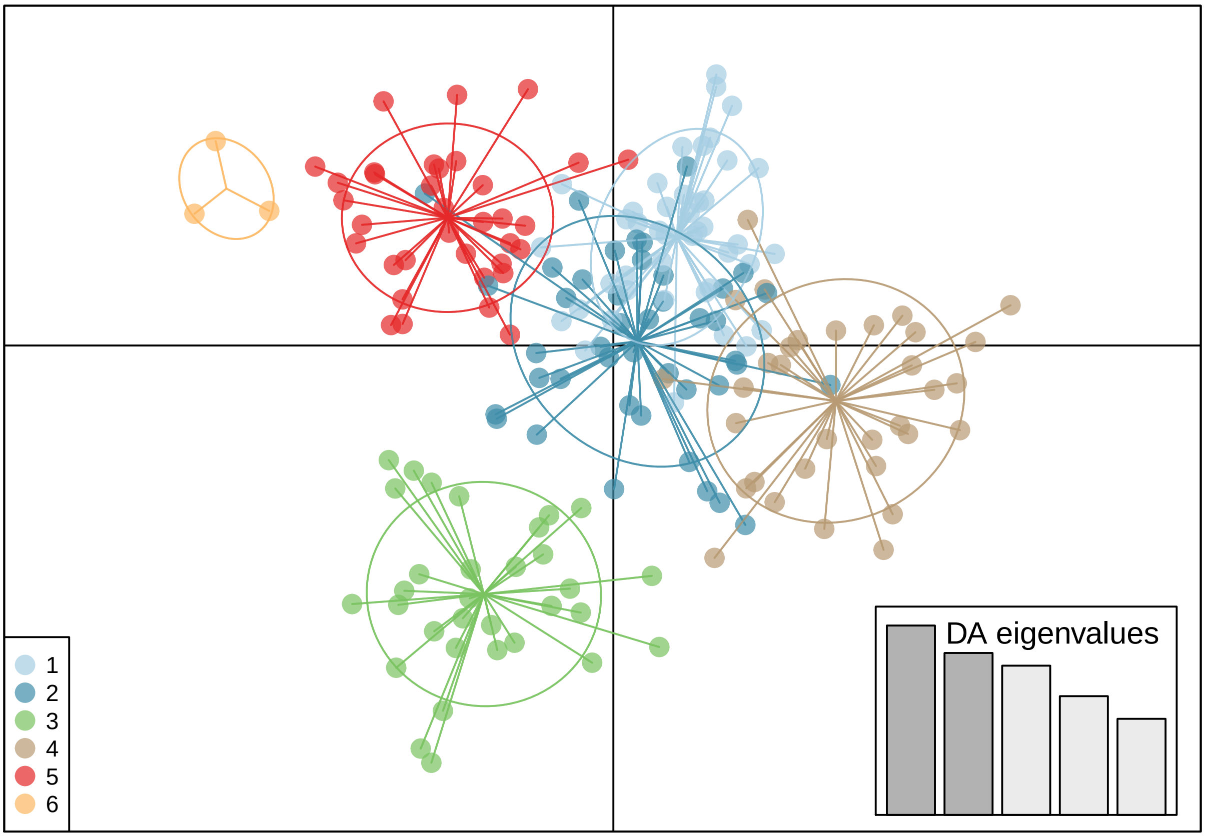

1
2
3
4
5
6
DA eigenvalues

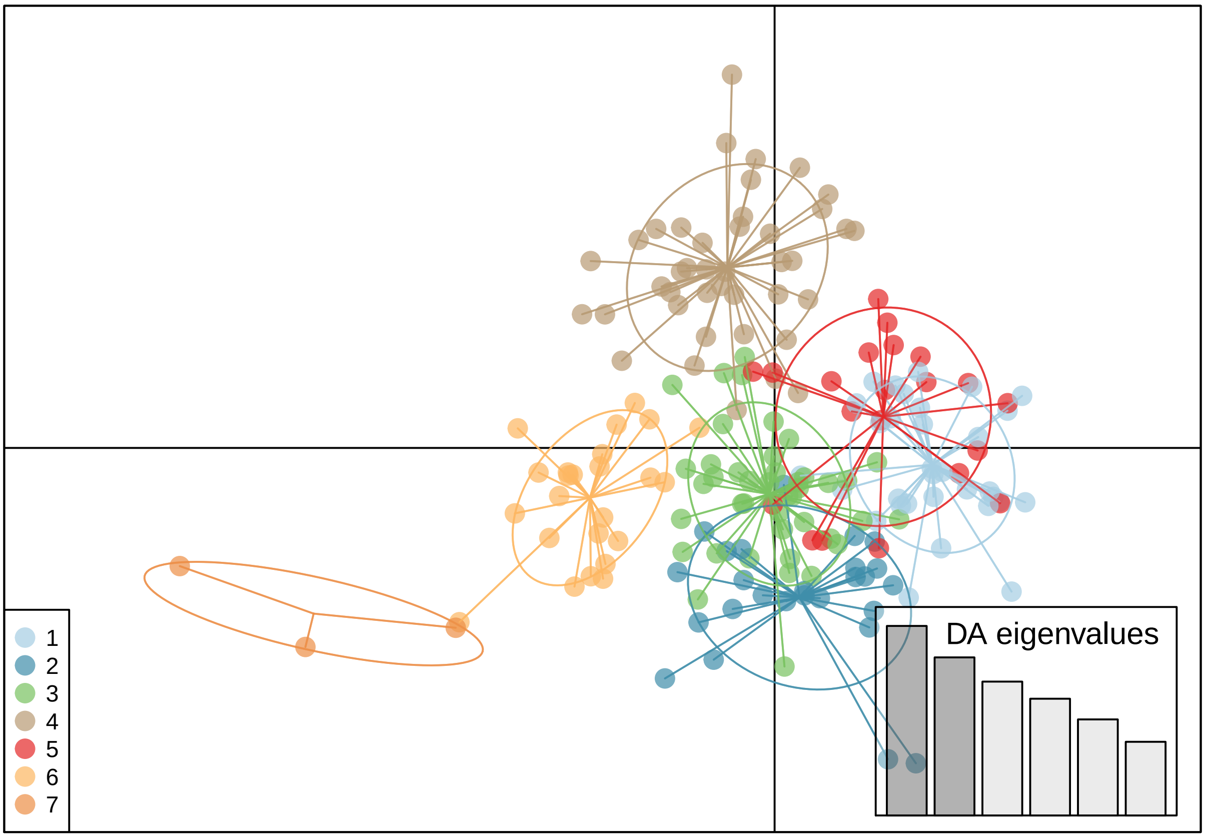

DA eigenvalues
1
2
3
4
5
6
7


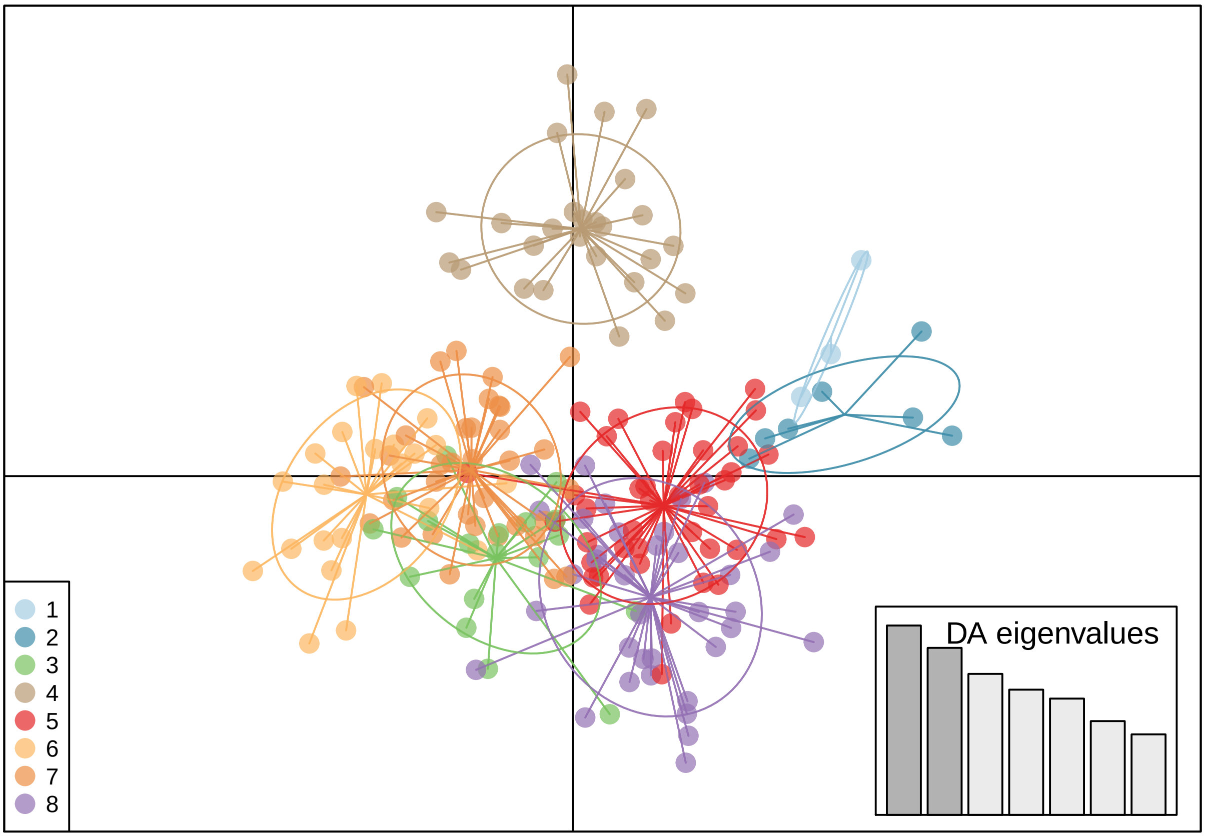

DA eigenvalues
1
2
3
4
5
6
7
8

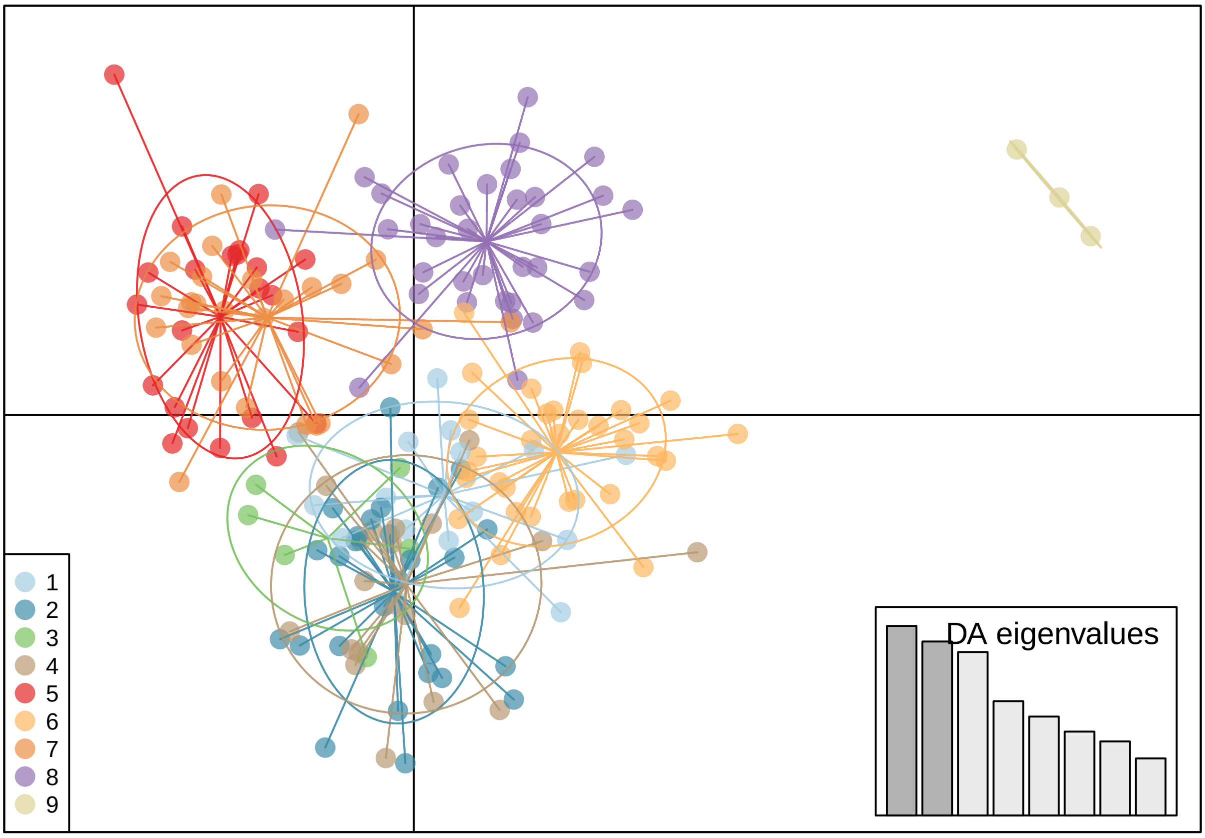


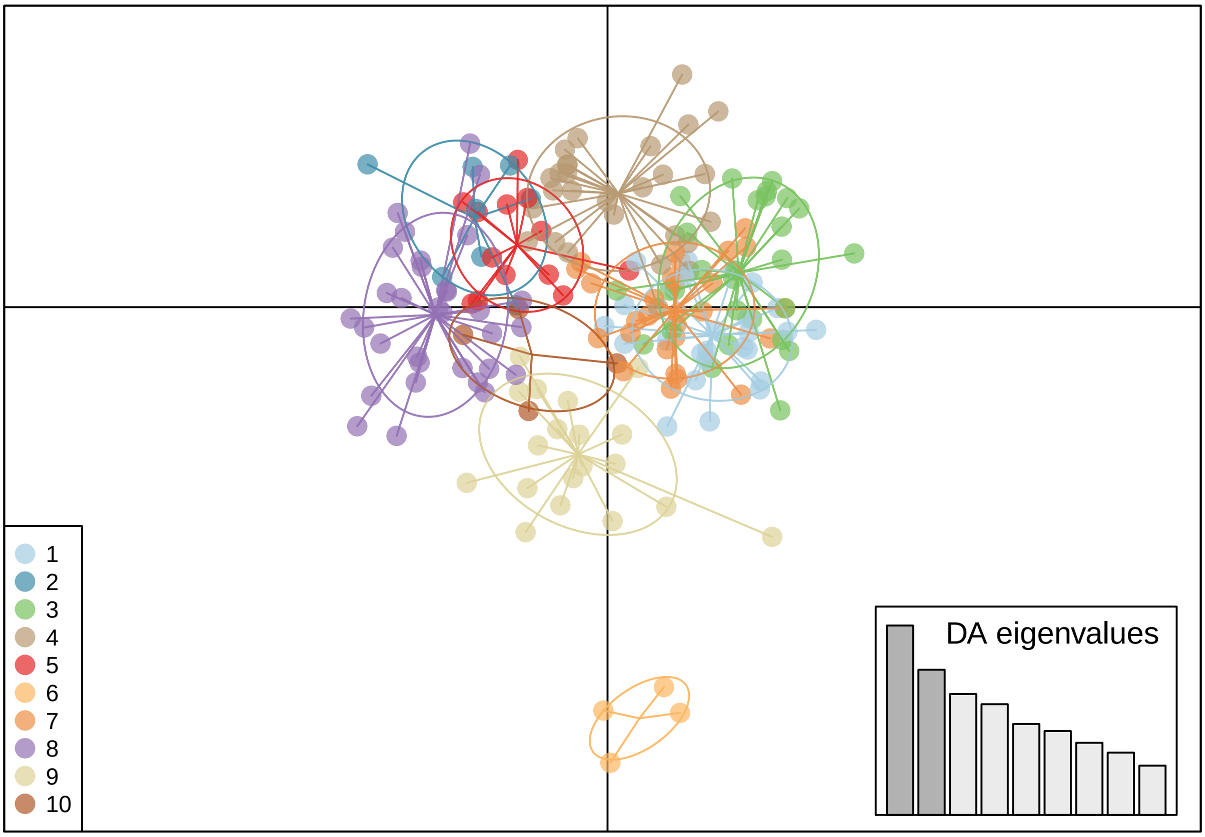


### 12.9.5 DAPC scatterplot - FLO

This code will *colour* individuals according to *sampling location* and **group** individuals according to DAPC **posterior membership**.

```r
for (K in 3:10) {
  dapc <- get(dapc.all.object.names[K - 1])
  x <- dapc$ind.coord[,1]
  y <- dapc$ind.coord[,2]
  group <- paste("Group", as.numeric(dapc$assign))
  pop <- BS.dartcap.IWP2.gl$pop
  df <- data.frame(x, y, group, pop)
  colnames(df) <- c("x","y","group","pop")
  gg <- merge(df,aggregate(cbind(mean.x = x,mean.y = y)~group,df,mean),
              by = "group")

  plot <- ggplot2::ggplot(gg, ggplot2::aes(x,y,color = pop)) +
    ggplot2::geom_point(size = 5) +
    ggplot2::geom_point(ggplot2::aes(x = mean.x,y = mean.y),size = 5,
                        shape = 17, color = "black") +
    ggplot2::geom_segment(ggplot2::aes(x = mean.x, y = mean.y,
                                       xend = x, yend = y)) +
    ggplot2::scale_colour_manual(values = colours.10) +
    ggplot2::scale_fill_manual(values = colours.10) +
    ggplot2::geom_hline(yintercept = 0) +
    ggplot2::geom_vline(xintercept = 0) +
    ggplot2::labs(subtitle = "",
         y = "PC2",
         x = "PC1",
         title = paste0("K=",K," & PC=",PC, sep = ""),
         caption = "") +
    ggplot2::theme_minimal() +
    ggplot2::theme(
      axis.text = ggplot2::element_text(size = 5),
      axis.title.x = ggplot2::element_text(size = 10),
      axis.title.y = ggplot2::element_text(size = 10),
      legend.text = ggplot2::element_text(size = 7))
  print(plot)
  ggplot2::ggsave(plot,
                  filename = paste0("DArTcap_IWP2_DAPC_scatterplot2_K", K, ".png"),
                  width = 30, height = 15,units = "cm")
}
```

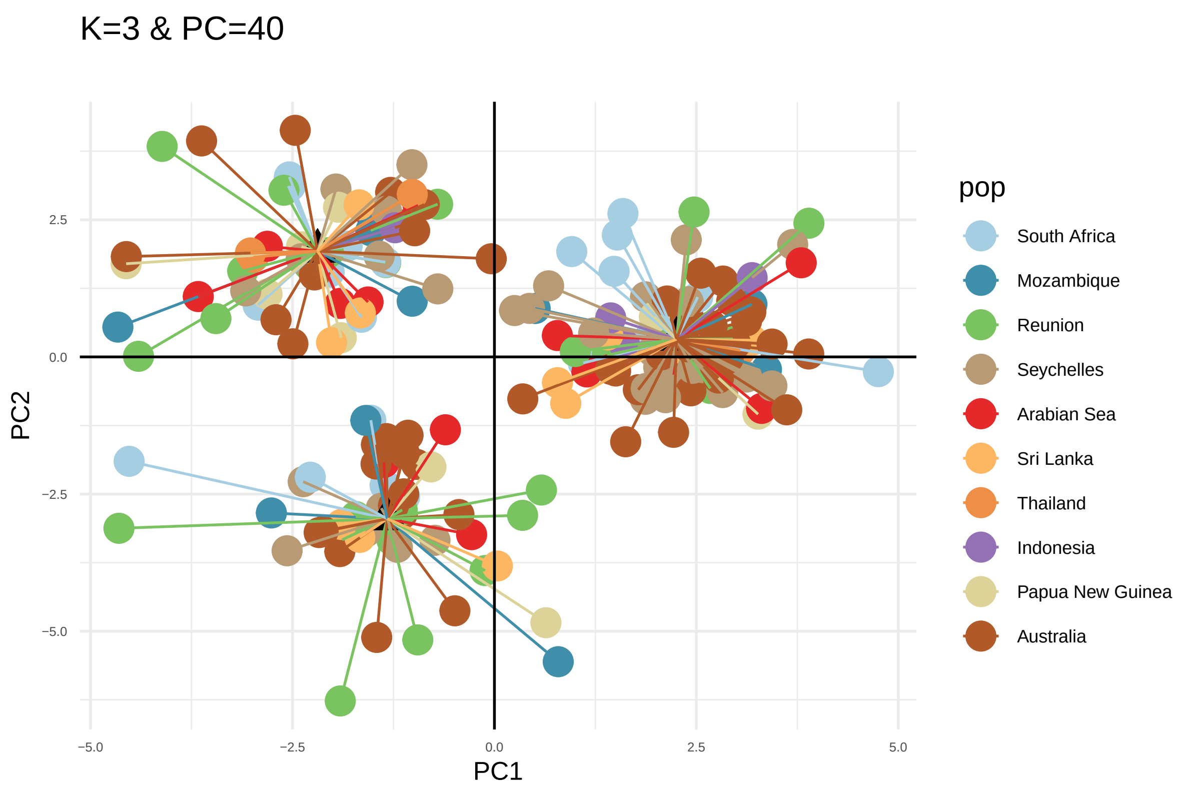
K=3 & PC=40
PC2
PC1
2.5
0.0
−2.5
−5.0
−5.0
−2.5
0.0
2.5
5.0
pop
South Africa
Mozambique
Reunion
Seychelles
Arabian Sea
Sri Lanka
Thailand
Indonesia
Papua New Guinea
Australia

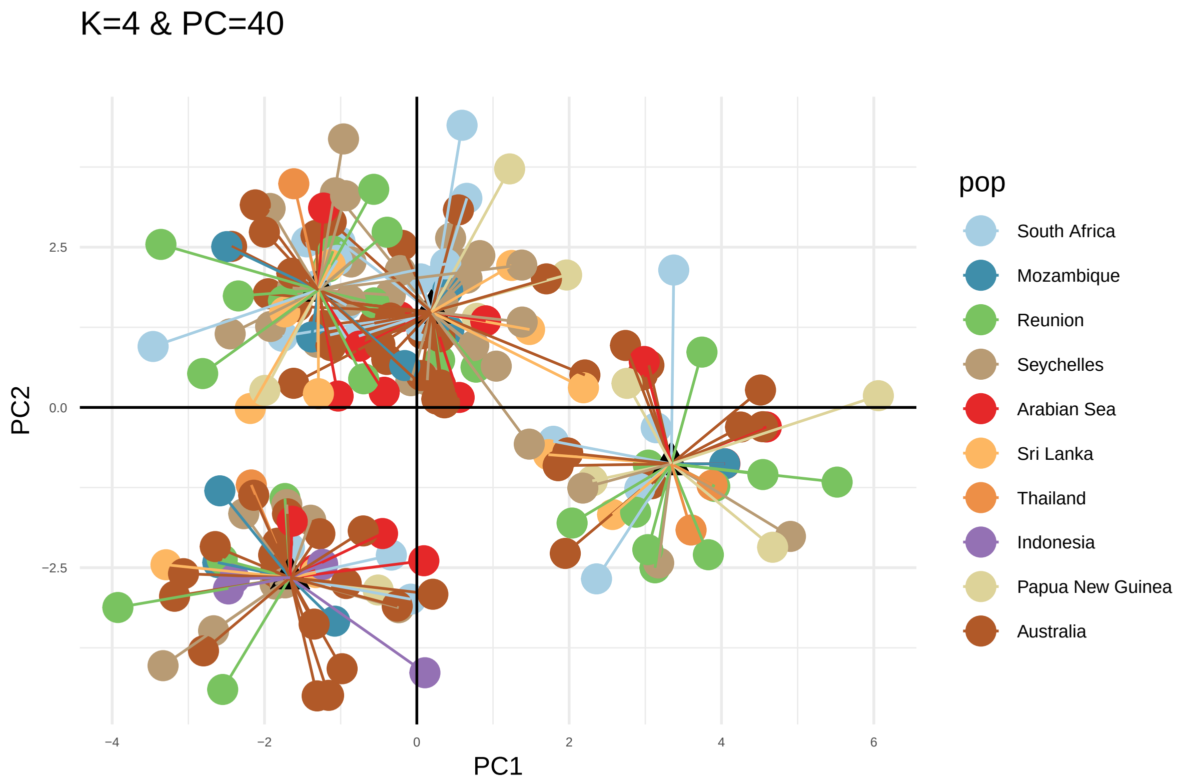
K=4 & PC=40
PC2
PC1
2.5
0.0
−2.5
−4
−2
0
2
4
6
pop
South Africa
Mozambique
Reunion
Seychelles
Arabian Sea
Sri Lanka
Thailand
Indonesia
Papua New Guinea
Australia

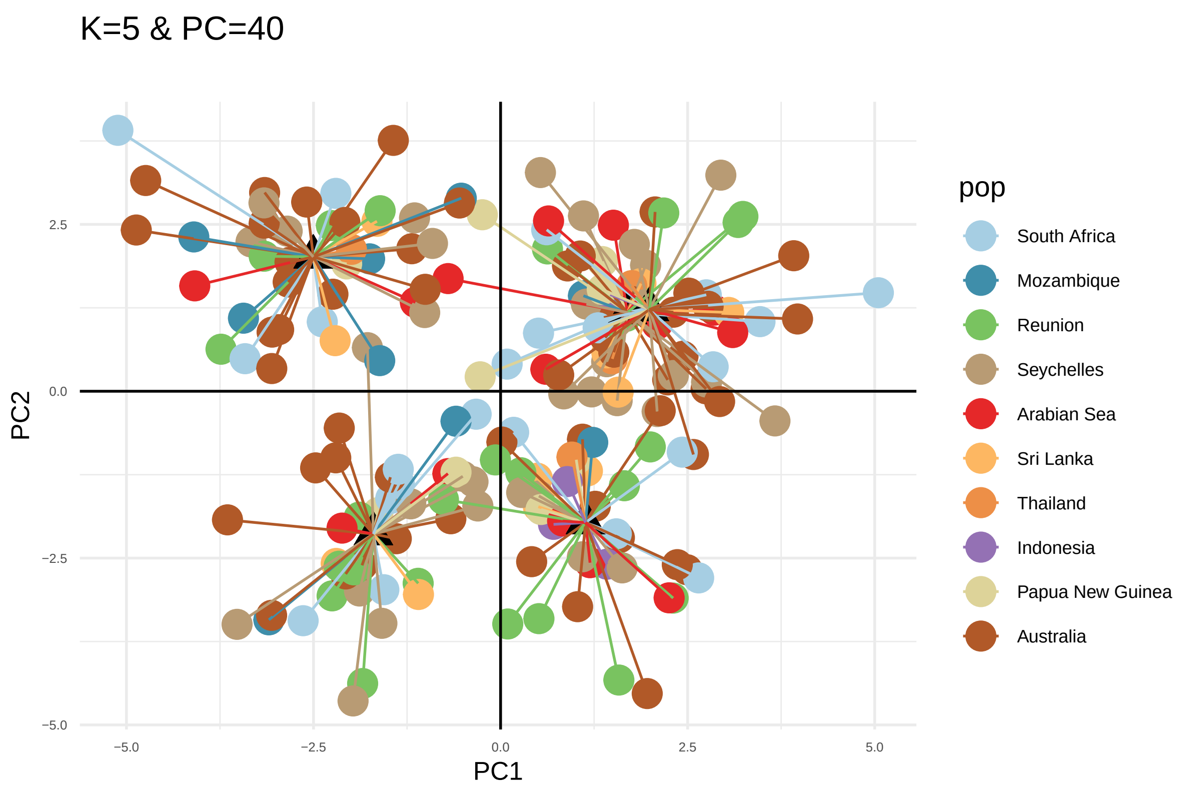
K=5 & PC=40
PC2
PC1
2.5
0.0
−2.5
−5.0
−5.0
−2.5
0.0
2.5
5.0
pop
South Africa
Mozambique
Reunion
Seychelles
Arabian Sea
Sri Lanka
Thailand
Indonesia
Papua New Guinea
Australia

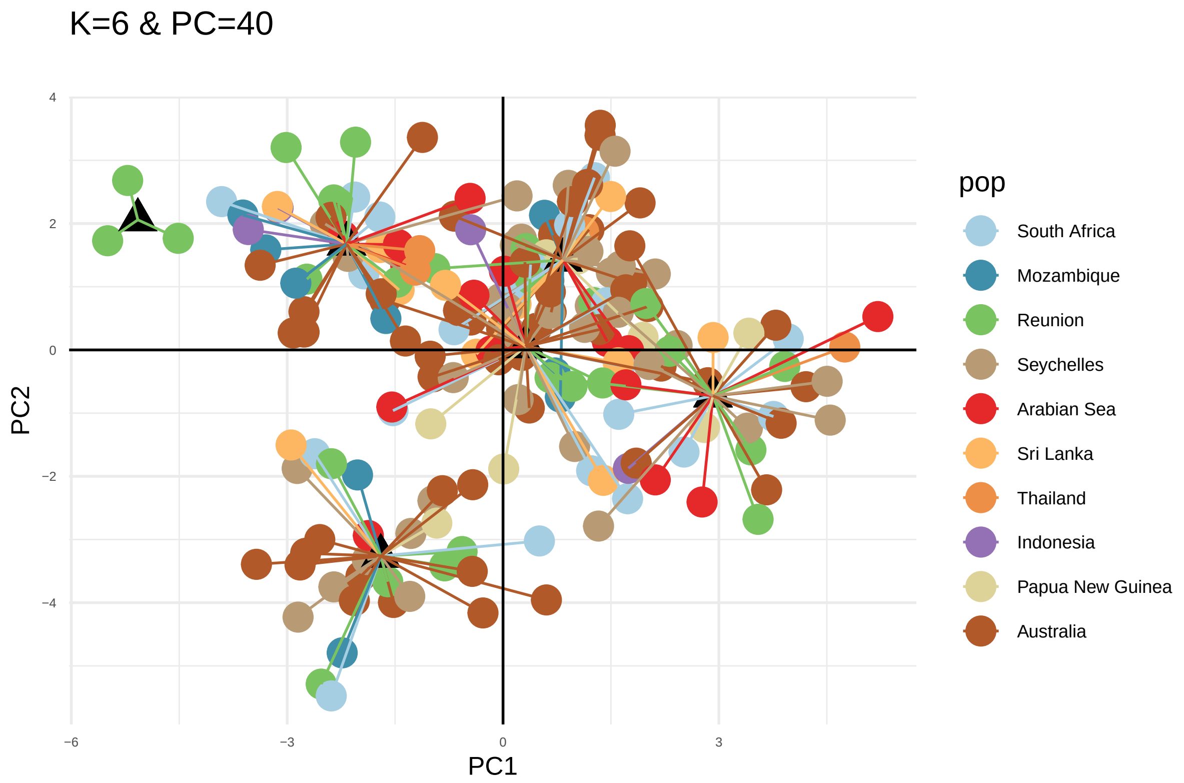
K=6 & PC=40
PC2
PC1
4
2
0
−2
−4
−6
−3
0
3
pop
South Africa
Mozambique
Reunion
Seychelles
Arabian Sea
Sri Lanka
Thailand
Indonesia
Papua New Guinea
Australia

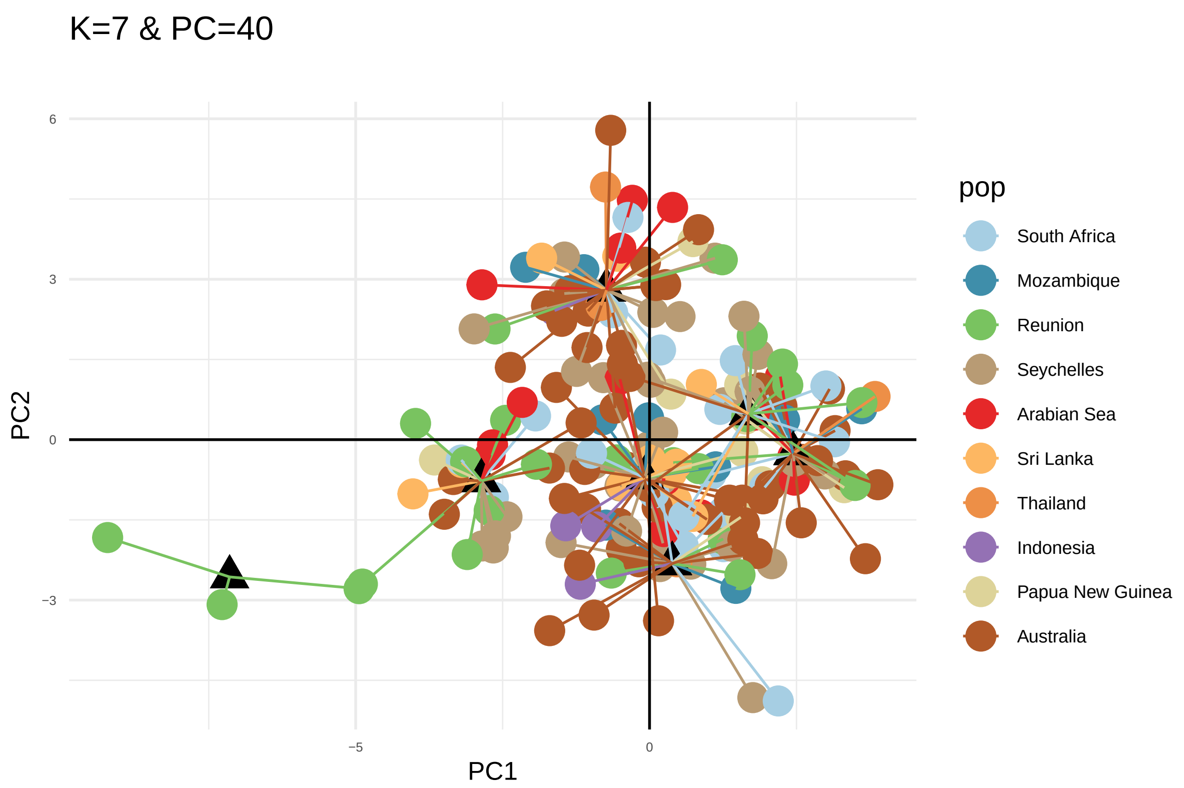
K=7 & PC=40
PC2
PC1
6
3
0
−3
−5
0
pop
South Africa
Mozambique
Reunion
Seychelles
Arabian Sea
Sri Lanka
Thailand
Indonesia
Papua New Guinea
Australia

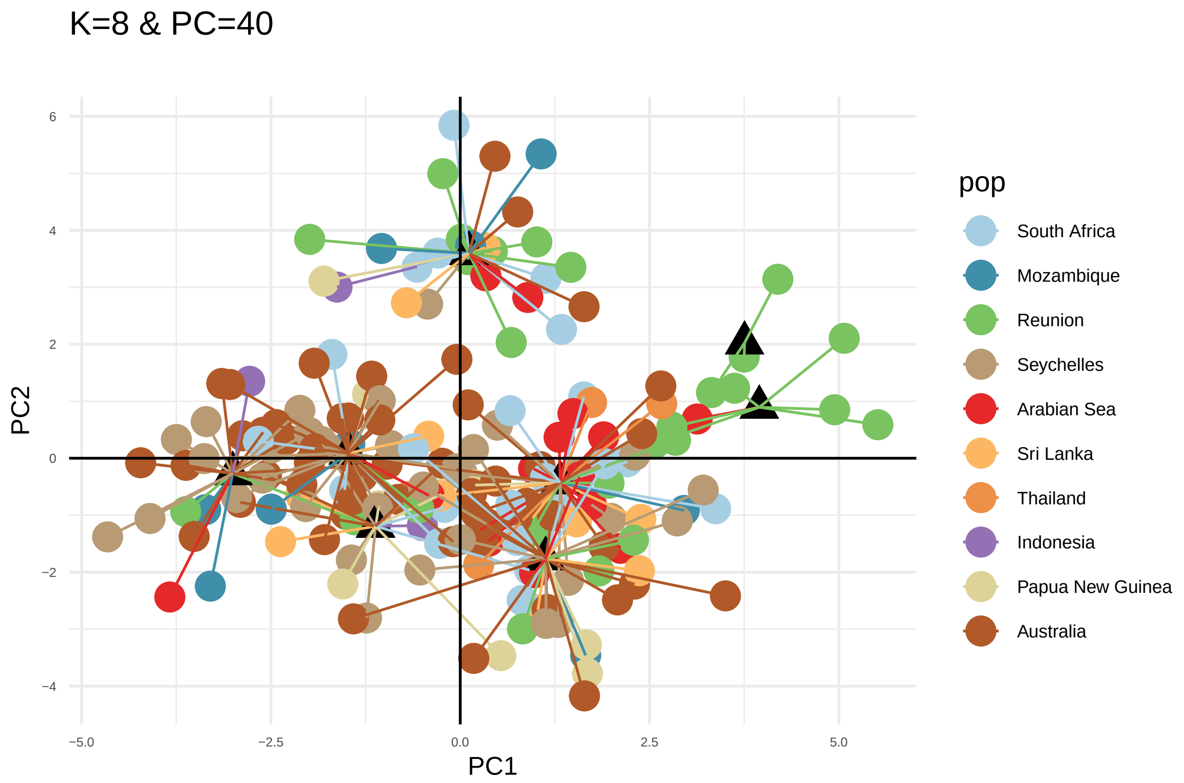
K=8 & PC=40
PC2
PC1
6
4
2
0
−2
−4
−5.0
−2.5
0.0
2.5
5.0
pop
South Africa
Mozambique
Reunion
Seychelles
Arabian Sea
Sri Lanka
Thailand
Indonesia
Papua New Guinea
Australia

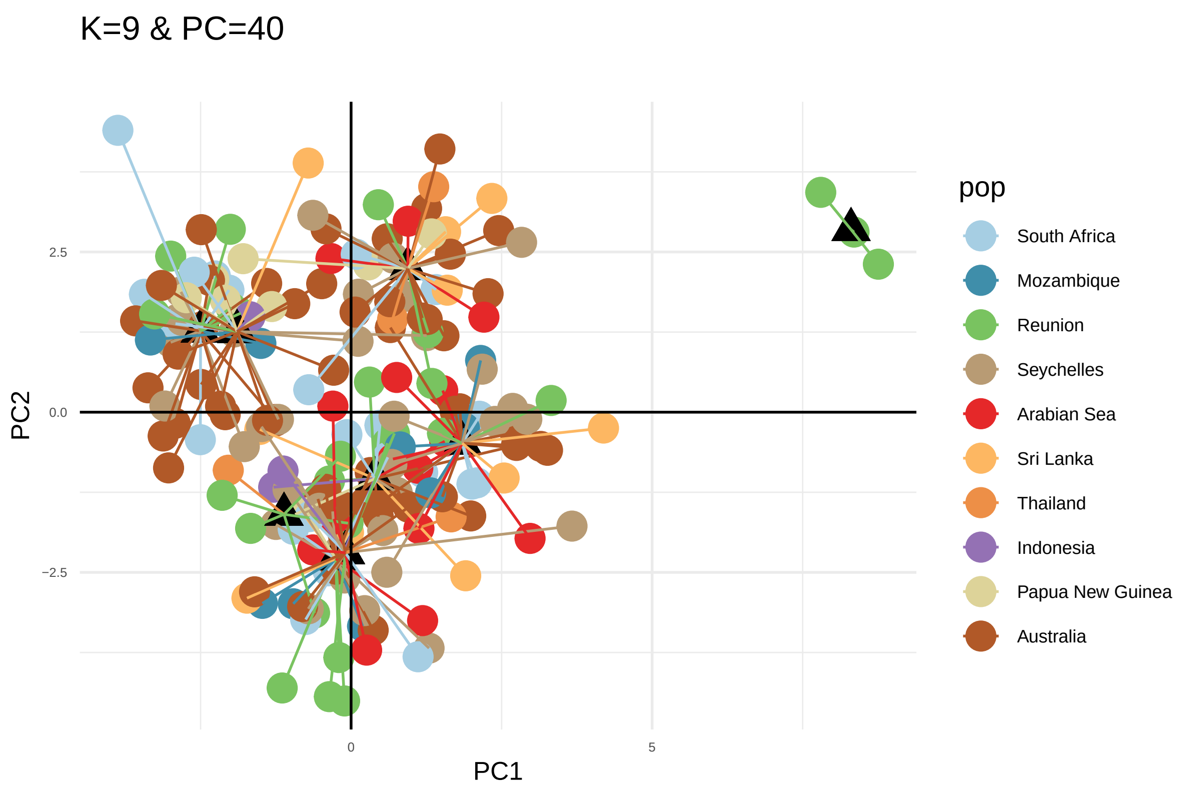
K=9 & PC=40
2.5
0.0
−2.5
0
5
PC1
PC2
pop
South Africa
Mozambique
Reunion
Seychelles
Arabian Sea
Sri Lanka
Thailand
Indonesia
Papua New Guinea
Australia

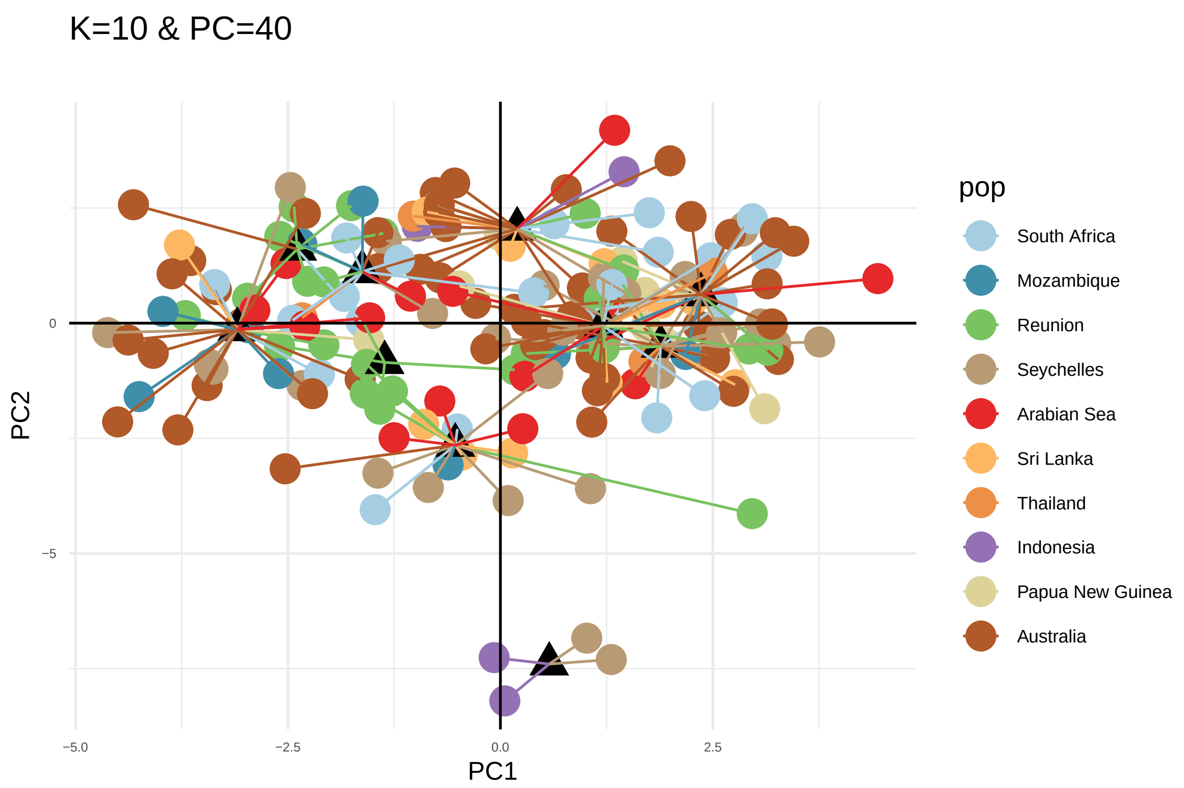
K=10 & PC=40
0
−5
−5.0
−2.5
0.0
2.5
PC1
PC2
pop
South Africa
Mozambique
Reunion
Seychelles
Arabian Sea
Sri Lanka
Thailand
Indonesia
Papua New Guinea
Australia

# 13 Mitogenome analyses

## 13.1 Sequencing primers

Primers were designed to amplify the mitochondrial genome of *C. leucas*. The mitochondrial genomes were amplified with two primer pairs.

Table 51: Primer sequences used for amplifying the two halves of the mitochondrial genome

| Mitogenome fragment | Primer name | Primer sequence | Amplicon length (bp) | Ref |
|---|---|---|---|---|
| A | BSh(1975L_for | 5'-AACACAAACTCCGCCTGTTTACCAAAAACATC | 9039 | Devloo-Delva et al. in prep |
| A | BSh(10999H_rev | 5'-CAACCGGCAATTGGAGCTTCAACGTGGG | 9039 | Devloo-Delva et al. in prep |
| B | BSh(9198L_for | 5'-GAGCCCATCATAGCTTAATAGAAGGTAAC | 9710 | Devloo-Delva et al. in prep |
| B | 16s(747-H_rev | 5'-GGTGTCTAAAGCTCCATAGGGTCTTCTCGTCT | 9710 | Devloo-Delva et al. in prep |

## 13.2 Preparing the mitogenome data files

### 13.2.1 MultiDNA format

```
f.in <- "BS_mitogenome_361_no ambiguous.fasta"
metafile <- "1.BullShark_AllSamples_metadata.csv"
BS.ml <- apex::read.multiFASTA(f.in)
(apex::setLocusNames(BS.ml) <- "Mitogenome")
```

### 13.2.2 Genind format

```
BS.gi <- apex::multidna2genind(BS.ml, mlst = TRUE)
```

### 13.2.3 Add metadata

```
meta <- read.csv(metafile)
meta <- meta[meta$mtDNA_ID %in% indNames(BS.gi),]
meta <- meta[order(match(meta$mtDNA_ID, indNames(BS.gi))),]

unique(meta$Location)

pop.levels0 <- c("Gulf of California","Costa Rica","Sierra Leone","South Africa",
                 "Reunion","Seychelles","Abu Dhabi","Dubai","Sri Lanka","Thailand",
                 "South-Indonesia","Jakarta","Omati River","Kikori Delta",
                 "Turama River","Sepik River","Fitzroy River", "Victoria River",
                 "Daly River","Adelaide River",
                 "South Alligator River","East Alligator River","Blue mud Bay",
                 "Roper River",
                 "Towns River","Cape York, QLD","Trinity inlet","Clarence River",
                 "Sydney Harbour",
                 "Okinawa ","Iriomote Island","Fiji")
meta$Location <- factor(x = meta$Location, levels = pop.levels0)

pop.levels1 <- c("Gulf of California","Costa Rica","Sierra Leone", "South Africa",
                 "Reunion","Seychelles","Arabian Sea","Sri Lanka","Thailand",
                 "Indonesia","Papua New Guinea","Australia","Japan","Fiji")
meta$Site <- factor(x = meta$Site, levels = pop.levels1)

pop.levels2 <- c("E-PAC","E-ATL","W-IO","N-IO","E-IO","W-PAC","Japan","Fiji")
meta$Region <- factor(x = meta$Region, levels = pop.levels2)

BS.gi$other$ind.meta <- meta
my_strata <- data.frame(Location = meta$Location,
                        Site = meta$Site ,
```

```r
                        regions = meta$Region)
strata(BS.gi) <- my_strata
BS.gi$pop <- as.factor(meta$Site)

BS.gi <- BS.gi[order(BS.gi$other$ind.meta$Region, BS.gi$other$ind.meta$Site,
                     BS.gi$other$ind.meta$Location, indNames(BS.gi)),]
```

```
##  [1] "Kikori Delta"          "Okinawa "              "Iriomote Island"
##  [4] "South Africa"          "Clarence River"        "Sydney Harbour"
##  [7] "Adelaide River"        "Daly River"            "Victoria River"
## [10] "Turama River"          "Omati River"           "Sepik River"
## [13] "Towns River"           "East Alligator River"  "South Alligator River"
## [16] "Cape York, QLD"        "Blue mud Bay"          "Fitzroy River"
## [19] "Roper River"           "Abu Dhabi"             "Thailand"
## [22] "Dubai"                 "South-Indonesia"       "Sri Lanka"
## [25] "Jakarta"               "Fiji"                  "Trinity inlet"
## [28] "Reunion"               "Seychelles"            "Sierra Leone"
## [31] "Costa Rica"            "Gulf of California"
```

### 13.2.4 Gtypes format

```r
Location <-  BS.gi$other$ind.meta$Location[order(match(indNames(BS.gi),
                                                  BS.ml@labels))]
Site <- BS.gi$other$ind.meta$Site[order(match(indNames(BS.gi),
                                         BS.ml@labels))]
Region <- BS.gi$other$ind.meta$Region[order(match(indNames(BS.gi),
                                             BS.ml@labels))]

strata.schemes <- data.frame(Region, Site, Location)
row.names(strata.schemes) <- BS.ml@labels

BS.gt <- strataG::sequence2gtypes(BS.ml, strata = Site, seq.names = NULL,
                                  schemes = strata.schemes,
                                  description = NULL, other = NULL)
```

### 13.2.5 Haplotype format

#### 13.2.5.1 Haplotypes package

This package includes indels: 26 HTs

```r
BS.dna <- haplotypes::read.fas(f.in)
BS.hap <- haplotypes::haplotype(BS.dna,indels = "sic")

Location <- BS.gi$other$ind.meta$Location[order(match(indNames(BS.gi),
                                                 BS.dna@seqnames))]
Site <- BS.gi$other$ind.meta$Site[order(match(indNames(BS.gi),
                                         BS.dna@seqnames))]
Region <- BS.gi$other$ind.meta$Region[order(match(indNames(BS.gi),
                                             BS.dna@seqnames))]

BS.hapgroup <- haplotypes::grouping(BS.hap,Site)
```

#### 13.2.5.2 Pegas package

This package does not include indels: 165Hts

```r
BS.bin <- read.FASTA(f.in, type = "DNA")
HTs <- paste0("HT", sprintf('%0.3d', 1:165))
BS.h <- pegas::haplotype(BS.bin, HTs)
```

### 13.2.6 Save data

```
save(BS.ml,BS.bin, BS.dna, BS.gi, BS.gt, BS.hap, BS.h,
     file = "6.BS.Rdata")
```

### 13.2.7 Load data

```
load("6.BS.Rdata")
adegenet::nInd(BS.gi)
summary(BS.gi$pop)
summary(BS.gi$other$ind.meta$Location)
```

```
## [1] 359
## Gulf of California         Costa Rica       Sierra Leone       South Africa
##                  1                 15                  1                  7
##            Reunion         Seychelles        Arabian Sea          Sri Lanka
##                 16                 18                 19                 12
##           Thailand          Indonesia   Papua New Guinea          Australia
##                  6                  6                 15                203
##              Japan               Fiji
##                 31                  9
##    Gulf of California            Costa Rica          Sierra Leone
##                     1                    15                     1
##          South Africa               Reunion            Seychelles
##                     7                    16                    18
##           Arabian Sea             Sri Lanka              Thailand
##                    19                    12                     6
##             Indonesia      Papua New Guinea         Fitzroy River
##                     6                    15                     5
##        Victoria River            Daly River        Adelaide River
##                    21                    25                    18
## South Alligator River  East Alligator River          Blue mud Bay
##                    38                    17                    13
##           Roper River           Towns River         Wenlock River
##                     4                     5                    19
##         Trinity inlet        Clarence River        Sydney Harbour
##                     4                    21                    13
##               Okinawa         Urauchi River                  Fiji
##                    10                    21                     9
```

```
strata <- as.character(BS.gt@schemes$Site)
names(strata) <- as.character(BS.gt@schemes$id)
strataG::setStrata(BS.gt) <- strata
BS.gt.AUS <- BS.gt[,,"Australia", drop = TRUE]

schemes.AUS <- BS.gt.AUS@schemes[BS.gt.AUS@schemes$Site == "Australia",]
strata <- as.character(schemes.AUS$Location)
names(strata) <- as.character(schemes.AUS$id)
strataG::setStrata(BS.gt.AUS) <- strata
BS.gt.AUS@schemes <- schemes.AUS

ind.names <- names(BS.dna) %in% BS.gt.AUS@data$id
BS.dna.AUS <- as.dna(BS.dna[ind.names, , drop = TRUE])
BS.bin.AUS <- subset(BS.bin, names(BS.bin) %in% BS.gt.AUS@data$id)
BS.gi.aus <- BS.gi[adegenet::indNames(BS.gi) %in% BS.gt.AUS@data$id]
BS.hap.aus <- haplotypes::haplotype(BS.dna.AUS,indels = "sic")
BS.h.aus <- strataG::labelHaplotypes(BS.bin.AUS, prefix = "HT",
```

```
use.indels = TRUE)
```

##### 13.2.7.1 Subset Australia

## 13.3 Genetic diversity

### 13.3.1 Overall diversity

```
seqLL <- strataG::sequenceLikelihoods(BS.bin, model = "N",
                                      pairwise.deletion = FALSE, n = NULL,
                                      plot = TRUE, simplify = TRUE)
```

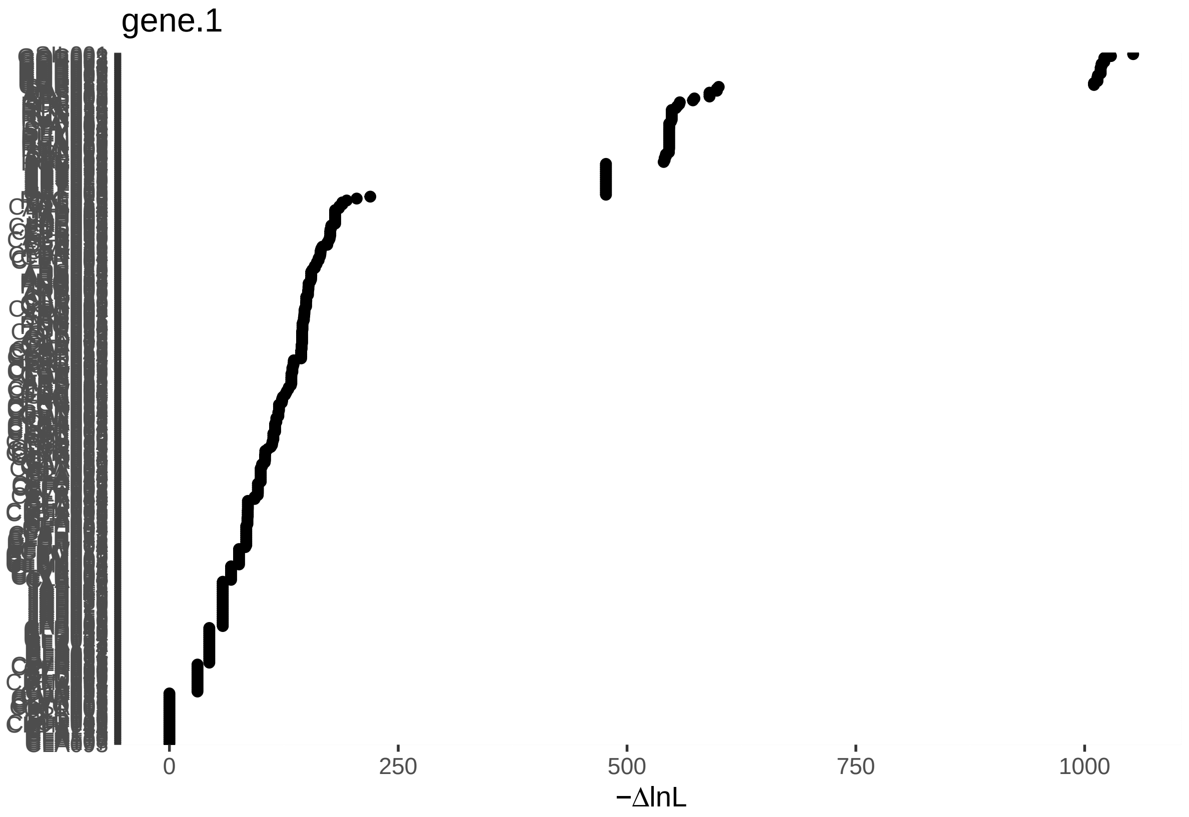


```
{bases <- haplotypes::basecomp(BS.dna)
print(paste0("Mitogenome base composition:"))
print(colMeans(bases))}

{pi.gt <- strataG::nucleotideDiversity(BS.gt, bases = c("a", "c", "g", "t"),
                                       simplify = TRUE)
plot(names(pi.gt[pi.gt != 0]),pi.gt[pi.gt != 0],
     xlab = "Base number in mitogenome",
     ylab = "Nucleotide diversity at each base",
     main = "Overall nucleotide diversity")}
```

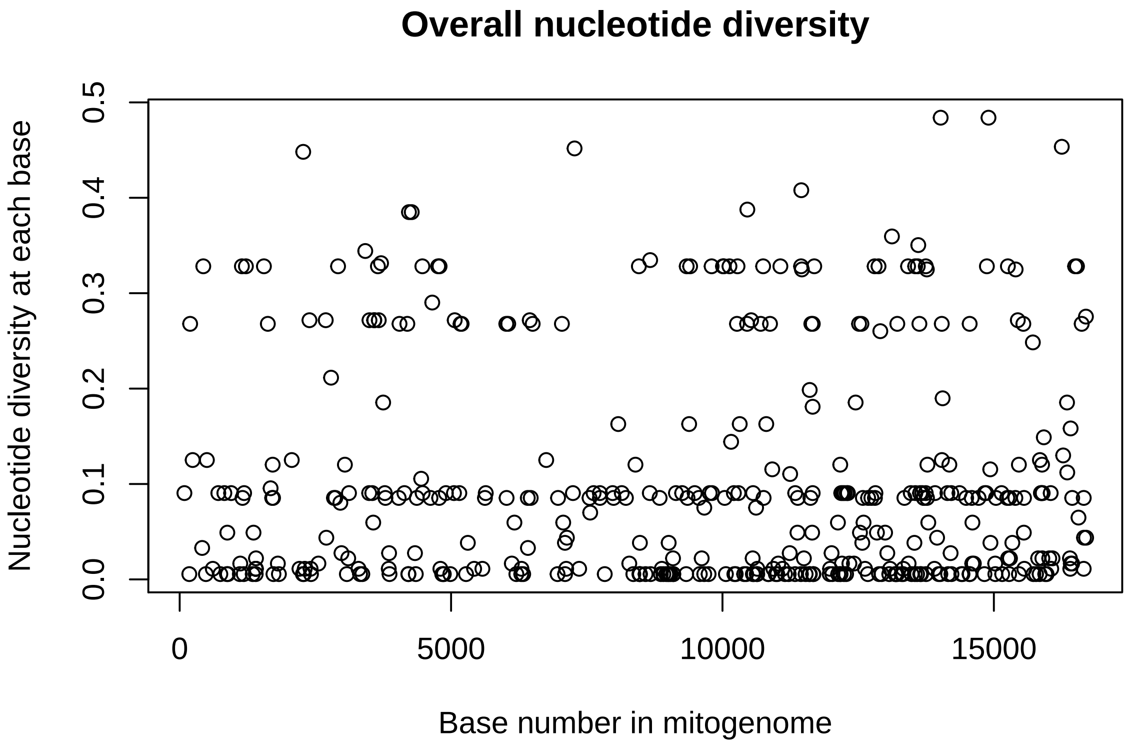


```
print(paste0("Nucleotide diversity according to strataG = ",mean(pi.gt)))
{print(paste0("Nucleotide diversity and variance according to pegas:"))
pegas::nuc.div(BS.bin, variance = TRUE, pairwise.deletion = FALSE)}
{print(paste0("Haplotype diversity and variance according to pegas:"))
pegas::hap.div(BS.bin, variance = TRUE, method = "Nei")}

varsites <- strataG::variableSites(BS.gt, bases = c("a", "c", "g", "t", "-"),
                                   simplify = TRUE)

kableExtra::kbl(t(varsites$site.freqs),
  caption = "Variable sites in mitogenome for all individuals",
  longtable = TRUE, booktabs = TRUE) %>%
  kableExtra::kable_styling(full_width = FALSE,font_size = 5,
                            latex_options = c("repeat_header", "hold_position"))
```

```
## [1] "Mitogenome base composition:"
##         A         T         G         C
## 0.3146193 0.2430317 0.1311460 0.3112030
## [1] "Nucleotide diversity according to strataG = 0.00263330094093478"
## [1] "Nucleotide diversity and variance according to pegas:"
## [1] 2.634878e-03 1.604305e-06
## [1] "Haplotype diversity and variance according to pegas:"
## [1] 9.886556e-01 2.119587e-06
```

Table 52: Variable sites in mitogenome for all individuals

| | a | c | g | t | - |
|---|---|---|---|---|---|
| 55 | 0 | 0 | 0 | 295 | 64 |
| 87 | 342 | 0 | 17 | 0 | 0 |
| 177 | 0 | 358 | 0 | 1 | 0 |
| 193 | 57 | 0 | 302 | 0 | 0 |
| 239 | 0 | 335 | 0 | 24 | 0 |
| 414 | 353 | 0 | 6 | 0 | 0 |
| 435 | 0 | 285 | 0 | 74 | 0 |
| 483 | 1 | 0 | 358 | 0 | 0 |
| 501 | 0 | 335 | 0 | 24 | 0 |

Table 52: Variable sites in mitogenome for all individuals *(continued)*

| | a | c | g | t | - |
|---|---|---|---|---|---|
| 610 | 0 | 357 | 0 | 2 | 0 |
| 712 | 0 | 342 | 0 | 17 | 0 |
| 749 | 358 | 0 | 0 | 1 | 0 |
| 806 | 0 | 0 | 0 | 4 | 355 |
| 823 | 0 | 17 | 0 | 342 | 0 |
| 870 | 1 | 0 | 358 | 0 | 0 |
| 878 | 0 | 350 | 0 | 9 | 0 |
| 889 | 0 | 1 | 0 | 358 | 0 |
| 948 | 0 | 17 | 0 | 342 | 0 |
| 1108 | 1 | 0 | 358 | 0 | 0 |
| 1116 | 356 | 0 | 3 | 0 | 0 |
| 1146 | 0 | 285 | 0 | 74 | 0 |
| 1158 | 0 | 343 | 0 | 16 | 0 |
| 1180 | 0 | 358 | 0 | 1 | 0 |
| 1187 | 0 | 17 | 0 | 342 | 0 |
| 1220 | 74 | 0 | 285 | 0 | 0 |
| 1346 | 0 | 358 | 0 | 1 | 0 |
| 1360 | 0 | 350 | 0 | 9 | 0 |
| 1399 | 2 | 0 | 357 | 0 | 0 |
| 1401 | 0 | 358 | 0 | 1 | 0 |
| 1402 | 0 | 2 | 0 | 357 | 0 |
| 1403 | 357 | 0 | 0 | 2 | 0 |
| 1404 | 357 | 0 | 0 | 2 | 0 |
| 1406 | 0 | 355 | 0 | 4 | 0 |
| 1552 | 0 | 285 | 0 | 74 | 0 |
| 1624 | 0 | 57 | 0 | 302 | 0 |
| 1675 | 18 | 0 | 341 | 0 | 0 |
| 1704 | 0 | 16 | 0 | 343 | 0 |
| 1713 | 0 | 336 | 0 | 23 | 0 |
| 1720 | 0 | 16 | 0 | 343 | 0 |
| 1728 | 0 | 1 | 0 | 358 | 0 |
| 1809 | 0 | 3 | 0 | 356 | 0 |
| 1828 | 1 | 0 | 358 | 0 | 0 |
| 2065 | 0 | 335 | 0 | 24 | 0 |
| 2207 | 0 | 2 | 0 | 357 | 0 |
| 2270 | 0 | 358 | 0 | 1 | 0 |
| 2276 | 0 | 121 | 0 | 238 | 0 |
| 2288 | 0 | 358 | 0 | 1 | 0 |
| 2304 | 0 | 2 | 0 | 357 | 0 |
| 2391 | 0 | 58 | 0 | 301 | 0 |
| 2412 | 0 | 2 | 0 | 357 | 0 |
| 2419 | 0 | 358 | 0 | 1 | 0 |
| 2556 | 0 | 356 | 0 | 3 | 0 |
| 2691 | 0 | 301 | 0 | 58 | 0 |
| 2702 | 0 | 8 | 0 | 351 | 0 |
| 2789 | 0 | 316 | 0 | 43 | 0 |
| 2842 | 0 | 343 | 0 | 16 | 0 |
| 2868 | 0 | 343 | 0 | 16 | 0 |
| 2918 | 0 | 285 | 0 | 74 | 0 |
| 2960 | 0 | 344 | 0 | 15 | 0 |
| 2981 | 0 | 5 | 0 | 354 | 0 |
| 3044 | 0 | 336 | 0 | 23 | 0 |
| 3080 | 0 | 358 | 0 | 1 | 0 |
| 3105 | 4 | 0 | 355 | 0 | 0 |
| 3119 | 17 | 0 | 342 | 0 | 0 |
| 3295 | 2 | 0 | 357 | 0 | 0 |
| 3323 | 0 | 358 | 0 | 1 | 0 |
| 3365 | 0 | 358 | 0 | 1 | 0 |
| 3419 | 0 | 280 | 0 | 79 | 0 |
| 3491 | 17 | 0 | 342 | 0 | 0 |
| 3497 | 0 | 58 | 0 | 301 | 0 |
| 3548 | 0 | 342 | 0 | 17 | 0 |
| 3566 | 0 | 11 | 0 | 348 | 0 |
| 3582 | 0 | 58 | 0 | 301 | 0 |
| 3587 | 0 | 301 | 0 | 58 | 0 |
| 3652 | 0 | 74 | 0 | 285 | 0 |
| 3668 | 0 | 58 | 0 | 301 | 0 |
| 3710 | 0 | 75 | 0 | 284 | 0 |
| 3749 | 0 | 322 | 0 | 37 | 0 |
| 3779 | 0 | 17 | 0 | 342 | 0 |
| 3791 | 0 | 16 | 0 | 343 | 0 |
| 3852 | 357 | 0 | 2 | 0 | 0 |
| 3858 | 5 | 0 | 354 | 0 | 0 |
| 3866 | 1 | 0 | 358 | 0 | 0 |
| 4035 | 0 | 343 | 0 | 16 | 0 |
| 4050 | 0 | 302 | 0 | 57 | 0 |
| 4140 | 0 | 17 | 0 | 342 | 0 |
| 4194 | 0 | 57 | 0 | 302 | 0 |
| 4212 | 0 | 358 | 0 | 1 | 0 |
| 4215 | 0 | 1 | 0 | 358 | 0 |
| 4224 | 0 | 93 | 0 | 266 | 0 |
| 4275 | 0 | 93 | 0 | 266 | 0 |
| 4335 | 0 | 354 | 0 | 5 | 0 |
| 4350 | 0 | 358 | 0 | 1 | 0 |

Table 52: Variable sites in mitogenome for all individuals *(continued)*

| | a | c | g | t | - |
|---|---|---|---|---|---|
| 4368 | 0 | 343 | 0 | 16 | 0 |
| 4449 | 0 | 339 | 0 | 20 | 0 |
| 4471 | 0 | 285 | 0 | 74 | 0 |
| 4476 | 0 | 17 | 0 | 342 | 0 |
| 4620 | 0 | 343 | 0 | 16 | 0 |
| 4653 | 0 | 296 | 0 | 63 | 0 |
| 4761 | 0 | 285 | 0 | 74 | 0 |
| 4780 | 0 | 16 | 0 | 343 | 0 |
| 4788 | 74 | 0 | 285 | 0 | 0 |
| 4803 | 0 | 357 | 0 | 2 | 0 |
| 4837 | 0 | 1 | 0 | 358 | 0 |
| 4857 | 0 | 358 | 0 | 1 | 0 |
| 4866 | 0 | 1 | 0 | 358 | 0 |
| 4905 | 0 | 342 | 0 | 17 | 0 |
| 4986 | 0 | 358 | 0 | 1 | 0 |
| 5053 | 342 | 0 | 0 | 17 | 0 |
| 5067 | 0 | 58 | 0 | 301 | 0 |
| 5152 | 0 | 17 | 0 | 342 | 0 |
| 5175 | 0 | 57 | 0 | 302 | 0 |
| 5194 | 57 | 0 | 302 | 0 | 0 |
| 5276 | 1 | 0 | 358 | 0 | 0 |
| 5309 | 7 | 0 | 352 | 0 | 0 |
| 5425 | 2 | 0 | 357 | 0 | 0 |
| 5577 | 2 | 0 | 357 | 0 | 0 |
| 5624 | 0 | 16 | 0 | 343 | 0 |
| 5639 | 0 | 17 | 0 | 342 | 0 |
| 6017 | 0 | 57 | 0 | 302 | 0 |
| 6023 | 0 | 343 | 0 | 16 | 0 |
| 6053 | 0 | 302 | 0 | 57 | 0 |
| 6119 | 0 | 356 | 0 | 3 | 0 |
| 6167 | 0 | 11 | 0 | 348 | 0 |
| 6203 | 0 | 358 | 0 | 1 | 0 |
| 6293 | 1 | 0 | 358 | 0 | 0 |
| 6297 | 2 | 0 | 357 | 0 | 0 |
| 6329 | 0 | 1 | 0 | 358 | 0 |
| 6413 | 0 | 16 | 0 | 343 | 0 |
| 6416 | 0 | 353 | 0 | 6 | 0 |
| 6449 | 301 | 0 | 58 | 0 | 0 |
| 6467 | 0 | 16 | 0 | 343 | 0 |
| 6503 | 0 | 57 | 0 | 302 | 0 |
| 6753 | 0 | 335 | 0 | 24 | 0 |
| 6962 | 0 | 358 | 0 | 1 | 0 |
| 6971 | 0 | 343 | 0 | 16 | 0 |
| 7042 | 0 | 302 | 0 | 57 | 0 |
| 7066 | 0 | 348 | 0 | 11 | 0 |
| 7098 | 0 | 7 | 0 | 352 | 0 |
| 7100 | 0 | 358 | 0 | 1 | 0 |
| 7121 | 0 | 357 | 0 | 2 | 0 |
| 7134 | 8 | 0 | 351 | 0 | 0 |
| 7245 | 0 | 17 | 0 | 342 | 0 |
| 7275 | 0 | 236 | 0 | 123 | 0 |
| 7360 | 2 | 0 | 357 | 0 | 0 |
| 7551 | 0 | 343 | 0 | 16 | 0 |
| 7563 | 0 | 346 | 0 | 13 | 0 |
| 7626 | 0 | 342 | 0 | 17 | 0 |
| 7741 | 342 | 0 | 17 | 0 | 0 |
| 7743 | 0 | 343 | 0 | 16 | 0 |
| 7831 | 1 | 0 | 358 | 0 | 0 |
| 7976 | 0 | 17 | 0 | 342 | 0 |
| 7993 | 0 | 343 | 0 | 16 | 0 |
| 8080 | 0 | 327 | 0 | 32 | 0 |
| 8145 | 0 | 17 | 0 | 342 | 0 |
| 8221 | 0 | 343 | 0 | 16 | 0 |
| 8283 | 0 | 3 | 0 | 356 | 0 |
| 8358 | 0 | 1 | 0 | 358 | 0 |
| 8397 | 336 | 0 | 0 | 23 | 0 |
| 8460 | 0 | 285 | 0 | 74 | 0 |
| 8470 | 0 | 358 | 0 | 1 | 0 |
| 8475 | 0 | 358 | 0 | 1 | 0 |
| 8479 | 0 | 352 | 0 | 7 | 0 |
| 8481 | 0 | 358 | 0 | 1 | 0 |
| 8583 | 0 | 1 | 0 | 358 | 0 |
| 8661 | 17 | 0 | 342 | 0 | 0 |
| 8668 | 0 | 76 | 0 | 283 | 0 |
| 8676 | 0 | 1 | 0 | 358 | 0 |
| 8842 | 0 | 343 | 0 | 16 | 0 |
| 8864 | 1 | 0 | 358 | 0 | 0 |
| 8882 | 0 | 357 | 0 | 2 | 0 |
| 8911 | 0 | 1 | 0 | 358 | 0 |
| 8963 | 0 | 1 | 0 | 358 | 0 |
| 8995 | 0 | 358 | 0 | 1 | 0 |
| 9009 | 7 | 0 | 352 | 0 | 0 |
| 9017 | 0 | 1 | 0 | 358 | 0 |
| 9048 | 1 | 0 | 358 | 0 | 0 |

Table 52: Variable sites in mitogenome for all individuals *(continued)*

| | a | c | g | t | - |
|---|---|---|---|---|---|
| 9089 | 0 | 355 | 0 | 4 | 0 |
| 9092 | 0 | 1 | 0 | 358 | 0 |
| 9095 | 0 | 358 | 0 | 1 | 0 |
| 9143 | 0 | 342 | 0 | 17 | 0 |
| 9257 | 0 | 17 | 0 | 342 | 0 |
| 9332 | 1 | 0 | 358 | 0 | 0 |
| 9341 | 0 | 285 | 0 | 74 | 0 |
| 9356 | 0 | 343 | 0 | 16 | 0 |
| 9386 | 0 | 327 | 0 | 32 | 0 |
| 9404 | 0 | 285 | 0 | 74 | 0 |
| 9488 | 0 | 17 | 0 | 342 | 0 |
| 9566 | 16 | 0 | 343 | 0 | 0 |
| 9582 | 0 | 1 | 0 | 358 | 0 |
| 9617 | 0 | 4 | 0 | 355 | 0 |
| 9664 | 0 | 358 | 0 | 1 | 0 |
| 9665 | 0 | 345 | 0 | 14 | 0 |
| 9744 | 0 | 358 | 0 | 1 | 0 |
| 9764 | 0 | 342 | 0 | 17 | 0 |
| 9800 | 0 | 74 | 0 | 285 | 0 |
| 9807 | 0 | 342 | 0 | 17 | 0 |
| 10009 | 285 | 0 | 74 | 0 | 0 |
| 10025 | 0 | 74 | 0 | 285 | 0 |
| 10040 | 16 | 0 | 343 | 0 | 0 |
| 10063 | 0 | 1 | 0 | 358 | 0 |
| 10129 | 0 | 285 | 0 | 74 | 0 |
| 10159 | 0 | 28 | 0 | 331 | 0 |
| 10210 | 0 | 17 | 0 | 342 | 0 |
| 10219 | 0 | 358 | 0 | 1 | 0 |
| 10244 | 0 | 358 | 0 | 1 | 0 |
| 10267 | 0 | 302 | 0 | 57 | 0 |
| 10277 | 0 | 74 | 0 | 285 | 0 |
| 10294 | 0 | 17 | 0 | 342 | 0 |
| 10318 | 0 | 327 | 0 | 32 | 0 |
| 10399 | 0 | 358 | 0 | 1 | 0 |
| 10437 | 0 | 358 | 0 | 1 | 0 |
| 10454 | 0 | 57 | 0 | 302 | 0 |
| 10458 | 0 | 94 | 0 | 265 | 0 |
| 10528 | 0 | 301 | 0 | 58 | 0 |
| 10556 | 4 | 0 | 355 | 0 | 0 |
| 10560 | 358 | 0 | 1 | 0 | 0 |
| 10564 | 0 | 342 | 0 | 17 | 0 |
| 10578 | 0 | 1 | 0 | 358 | 0 |
| 10619 | 14 | 0 | 345 | 0 | 0 |
| 10632 | 0 | 1 | 0 | 358 | 0 |
| 10641 | 0 | 1 | 0 | 358 | 0 |
| 10642 | 0 | 357 | 0 | 2 | 0 |
| 10704 | 0 | 57 | 0 | 302 | 0 |
| 10749 | 74 | 0 | 285 | 0 | 0 |
| 10758 | 0 | 16 | 0 | 343 | 0 |
| 10807 | 0 | 327 | 0 | 32 | 0 |
| 10840 | 0 | 358 | 0 | 1 | 0 |
| 10851 | 358 | 0 | 1 | 0 | 0 |
| 10876 | 0 | 57 | 0 | 302 | 0 |
| 10917 | 337 | 0 | 22 | 0 | 0 |
| 10939 | 2 | 0 | 357 | 0 | 0 |
| 10983 | 0 | 1 | 0 | 358 | 0 |
| 11010 | 0 | 358 | 0 | 1 | 0 |
| 11028 | 0 | 3 | 0 | 356 | 0 |
| 11067 | 0 | 285 | 0 | 74 | 0 |
| 11105 | 0 | 2 | 0 | 357 | 0 |
| 11151 | 0 | 0 | 1 | 358 | 0 |
| 11220 | 1 | 358 | 0 | 0 | 0 |
| 11239 | 5 | 0 | 354 | 0 | 0 |
| 11247 | 0 | 21 | 0 | 338 | 0 |
| 11337 | 0 | 358 | 0 | 1 | 0 |
| 11340 | 0 | 342 | 0 | 17 | 0 |
| 11382 | 0 | 350 | 0 | 9 | 0 |
| 11388 | 16 | 0 | 343 | 0 | 0 |
| 11445 | 0 | 358 | 0 | 1 | 0 |
| 11448 | 0 | 74 | 0 | 285 | 0 |
| 11452 | 0 | 102 | 0 | 257 | 0 |
| 11463 | 0 | 73 | 0 | 286 | 0 |
| 11500 | 0 | 355 | 0 | 4 | 0 |
| 11532 | 0 | 358 | 0 | 1 | 0 |
| 11599 | 358 | 0 | 1 | 0 | 0 |
| 11607 | 0 | 40 | 0 | 319 | 0 |
| 11610 | 0 | 358 | 0 | 1 | 0 |
| 11621 | 0 | 343 | 0 | 16 | 0 |
| 11637 | 0 | 57 | 0 | 302 | 0 |
| 11652 | 0 | 350 | 0 | 9 | 0 |
| 11661 | 0 | 323 | 0 | 36 | 0 |
| 11662 | 0 | 342 | 0 | 17 | 0 |
| 11664 | 0 | 302 | 0 | 57 | 0 |
| 11670 | 0 | 358 | 0 | 1 | 0 |

Table 52: Variable sites in mitogenome for all individuals *(continued)*

| | a | c | g | t | - |
|---|---|---|---|---|---|
| 11676 | 0 | 358 | 0 | 1 | 0 |
| 11691 | 0 | 74 | 0 | 285 | 0 |
| 11976 | 358 | 0 | 1 | 0 | 0 |
| 11982 | 2 | 0 | 357 | 0 | 0 |
| 12010 | 0 | 354 | 0 | 5 | 0 |
| 12023 | 1 | 0 | 358 | 0 | 0 |
| 12127 | 0 | 348 | 0 | 11 | 0 |
| 12131 | 0 | 358 | 0 | 1 | 0 |
| 12167 | 0 | 358 | 0 | 1 | 0 |
| 12170 | 0 | 336 | 0 | 23 | 0 |
| 12176 | 0 | 358 | 0 | 1 | 0 |
| 12188 | 0 | 342 | 0 | 17 | 0 |
| 12202 | 0 | 3 | 0 | 356 | 0 |
| 12219 | 0 | 342 | 0 | 17 | 0 |
| 12239 | 0 | 1 | 0 | 358 | 0 |
| 12251 | 0 | 342 | 0 | 17 | 0 |
| 12278 | 0 | 358 | 0 | 1 | 0 |
| 12290 | 0 | 17 | 0 | 342 | 0 |
| 12311 | 0 | 17 | 0 | 342 | 0 |
| 12338 | 0 | 356 | 0 | 3 | 0 |
| 12423 | 3 | 0 | 356 | 0 | 0 |
| 12453 | 37 | 0 | 322 | 0 | 0 |
| 12516 | 0 | 302 | 0 | 57 | 0 |
| 12533 | 0 | 350 | 0 | 9 | 0 |
| 12560 | 0 | 302 | 0 | 57 | 0 |
| 12576 | 0 | 352 | 0 | 7 | 0 |
| 12587 | 0 | 16 | 0 | 343 | 0 |
| 12599 | 0 | 348 | 0 | 11 | 0 |
| 12626 | 0 | 357 | 0 | 2 | 0 |
| 12678 | 0 | 1 | 0 | 358 | 0 |
| 12683 | 0 | 343 | 0 | 16 | 0 |
| 12743 | 16 | 0 | 0 | 343 | 0 |
| 12803 | 0 | 74 | 0 | 285 | 0 |
| 12809 | 0 | 343 | 0 | 16 | 0 |
| 12818 | 0 | 342 | 0 | 17 | 0 |
| 12846 | 9 | 0 | 350 | 0 | 0 |
| 12875 | 74 | 0 | 285 | 0 | 0 |
| 12885 | 1 | 0 | 358 | 0 | 0 |
| 12908 | 0 | 304 | 0 | 55 | 0 |
| 12932 | 0 | 358 | 0 | 1 | 0 |
| 12996 | 9 | 0 | 350 | 0 | 0 |
| 13037 | 5 | 0 | 0 | 354 | 0 |
| 13073 | 0 | 1 | 0 | 358 | 0 |
| 13088 | 0 | 2 | 0 | 357 | 0 |
| 13122 | 84 | 0 | 275 | 0 | 0 |
| 13183 | 0 | 1 | 0 | 358 | 0 |
| 13190 | 358 | 0 | 0 | 1 | 0 |
| 13200 | 1 | 0 | 358 | 0 | 0 |
| 13217 | 0 | 1 | 0 | 358 | 0 |
| 13221 | 57 | 0 | 302 | 0 | 0 |
| 13301 | 0 | 358 | 0 | 1 | 0 |
| 13328 | 0 | 358 | 0 | 1 | 0 |
| 13332 | 2 | 0 | 357 | 0 | 0 |
| 13352 | 0 | 343 | 0 | 16 | 0 |
| 13419 | 285 | 0 | 74 | 0 | 0 |
| 13429 | 0 | 356 | 0 | 3 | 0 |
| 13469 | 0 | 342 | 0 | 17 | 0 |
| 13484 | 0 | 1 | 0 | 358 | 0 |
| 13536 | 0 | 7 | 0 | 352 | 0 |
| 13544 | 0 | 1 | 0 | 358 | 0 |
| 13550 | 285 | 0 | 74 | 0 | 0 |
| 13554 | 0 | 17 | 0 | 342 | 0 |
| 13586 | 0 | 1 | 0 | 358 | 0 |
| 13598 | 0 | 285 | 0 | 74 | 0 |
| 13607 | 0 | 81 | 0 | 278 | 0 |
| 13628 | 0 | 57 | 0 | 302 | 0 |
| 13640 | 0 | 17 | 0 | 342 | 0 |
| 13643 | 0 | 358 | 0 | 1 | 0 |
| 13659 | 0 | 358 | 0 | 1 | 0 |
| 13679 | 0 | 342 | 0 | 17 | 0 |
| 13709 | 0 | 16 | 0 | 343 | 0 |
| 13710 | 0 | 343 | 0 | 16 | 0 |
| 13745 | 0 | 17 | 0 | 342 | 0 |
| 13746 | 0 | 74 | 0 | 285 | 0 |
| 13750 | 0 | 1 | 0 | 358 | 0 |
| 13765 | 0 | 286 | 0 | 73 | 0 |
| 13768 | 0 | 16 | 0 | 343 | 0 |
| 13776 | 0 | 336 | 0 | 23 | 0 |
| 13792 | 0 | 348 | 0 | 11 | 0 |
| 13905 | 0 | 357 | 0 | 2 | 0 |
| 13916 | 0 | 342 | 0 | 17 | 0 |
| 13954 | 0 | 8 | 0 | 351 | 0 |
| 13983 | 0 | 1 | 0 | 358 | 0 |
| 14014 | 0 | 358 | 0 | 1 | 0 |

Table 52: Variable sites in mitogenome for all individuals *(continued)*

| | a | c | g | t | - |
|---|---|---|---|---|---|
| 14020 | 0 | 146 | 0 | 213 | 0 |
| 14040 | 0 | 302 | 0 | 57 | 0 |
| 14043 | 0 | 24 | 0 | 335 | 0 |
| 14056 | 0 | 321 | 0 | 38 | 0 |
| 14145 | 17 | 0 | 342 | 0 | 0 |
| 14158 | 358 | 0 | 1 | 0 | 0 |
| 14180 | 23 | 0 | 336 | 0 | 0 |
| 14203 | 5 | 0 | 354 | 0 | 0 |
| 14215 | 17 | 0 | 342 | 0 | 0 |
| 14219 | 1 | 0 | 358 | 0 | 0 |
| 14367 | 0 | 342 | 0 | 17 | 0 |
| 14400 | 0 | 358 | 0 | 1 | 0 |
| 14422 | 1 | 0 | 358 | 0 | 0 |
| 14490 | 0 | 16 | 0 | 343 | 0 |
| 14551 | 1 | 0 | 358 | 0 | 0 |
| 14554 | 57 | 0 | 302 | 0 | 0 |
| 14557 | 0 | 358 | 0 | 1 | 0 |
| 14592 | 0 | 16 | 0 | 343 | 0 |
| 14601 | 0 | 356 | 0 | 3 | 0 |
| 14605 | 11 | 0 | 348 | 0 | 0 |
| 14631 | 0 | 356 | 0 | 3 | 0 |
| 14718 | 0 | 343 | 0 | 16 | 0 |
| 14826 | 1 | 0 | 358 | 0 | 0 |
| 14832 | 342 | 0 | 17 | 0 | 0 |
| 14859 | 0 | 342 | 0 | 17 | 0 |
| 14871 | 0 | 74 | 0 | 285 | 0 |
| 14901 | 0 | 213 | 0 | 146 | 0 |
| 14933 | 0 | 337 | 0 | 22 | 0 |
| 14937 | 0 | 352 | 0 | 7 | 0 |
| 15024 | 0 | 356 | 0 | 3 | 0 |
| 15027 | 0 | 358 | 0 | 1 | 0 |
| 15039 | 0 | 343 | 0 | 16 | 0 |
| 15141 | 0 | 17 | 0 | 342 | 0 |
| 15153 | 0 | 1 | 0 | 358 | 0 |
| 15246 | 0 | 343 | 0 | 16 | 0 |
| 15258 | 0 | 285 | 0 | 74 | 0 |
| 15262 | 0 | 4 | 0 | 355 | 0 |
| 15281 | 0 | 358 | 0 | 1 | 0 |
| 15285 | 0 | 343 | 0 | 16 | 0 |
| 15297 | 0 | 4 | 0 | 355 | 0 |
| 15340 | 7 | 0 | 352 | 0 | 0 |
| 15396 | 0 | 343 | 0 | 16 | 0 |
| 15401 | 0 | 286 | 0 | 73 | 0 |
| 15441 | 0 | 58 | 0 | 301 | 0 |
| 15461 | 0 | 336 | 0 | 23 | 0 |
| 15462 | 0 | 358 | 0 | 1 | 0 |
| 15543 | 0 | 57 | 0 | 302 | 0 |
| 15552 | 0 | 350 | 0 | 9 | 0 |
| 15555 | 16 | 0 | 343 | 0 | 0 |
| 15561 | 0 | 2 | 0 | 357 | 0 |
| 15671 | 40 | 0 | 0 | 0 | 319 |
| 15718 | 0 | 52 | 0 | 307 | 0 |
| 15728 | 0 | 1 | 0 | 358 | 0 |
| 15777 | 358 | 0 | 1 | 0 | 0 |
| 15805 | 358 | 0 | 0 | 0 | 1 |
| 15816 | 4 | 0 | 355 | 0 | 0 |
| 15843 | 0 | 358 | 0 | 1 | 0 |
| 15848 | 0 | 24 | 0 | 335 | 0 |
| 15867 | 0 | 342 | 0 | 17 | 0 |
| 15869 | 17 | 0 | 342 | 0 | 0 |
| 15888 | 0 | 23 | 0 | 336 | 0 |
| 15891 | 342 | 0 | 17 | 0 | 0 |
| 15893 | 0 | 4 | 0 | 355 | 0 |
| 15899 | 342 | 0 | 17 | 0 | 0 |
| 15920 | 29 | 0 | 330 | 0 | 0 |
| 15946 | 0 | 358 | 0 | 1 | 0 |
| 15971 | 0 | 1 | 0 | 358 | 0 |
| 16020 | 0 | 0 | 4 | 355 | 0 |
| 16047 | 17 | 0 | 0 | 342 | 0 |
| 16057 | 0 | 357 | 0 | 2 | 0 |
| 16080 | 355 | 0 | 4 | 0 | 0 |
| 16084 | 0 | 0 | 0 | 355 | 4 |
| 16251 | 235 | 0 | 124 | 0 | 0 |
| 16277 | 0 | 25 | 0 | 334 | 0 |
| 16348 | 322 | 0 | 37 | 0 | 0 |
| 16352 | 338 | 13 | 0 | 8 | 0 |
| 16402 | 0 | 4 | 0 | 355 | 0 |
| 16408 | 2 | 0 | 357 | 0 | 0 |
| 16414 | 328 | 0 | 31 | 0 | 0 |
| 16417 | 0 | 3 | 0 | 356 | 0 |
| 16442 | 356 | 0 | 3 | 0 | 0 |
| 16443 | 0 | 343 | 0 | 16 | 0 |
| 16496 | 0 | 285 | 0 | 74 | 0 |
| 16531 | 74 | 285 | 0 | 0 | 0 |

Table 52: Variable sites in mitogenome for all individuals *(continued)*

| | a | c | g | t | - |
|---|---|---|---|---|---|
| 16559 | 0 | 347 | 0 | 12 | 0 |
| 16562 | 137 | 0 | 0 | 0 | 222 |
| 16618 | 57 | 0 | 302 | 0 | 0 |
| 16655 | 0 | 2 | 0 | 357 | 0 |
| 16656 | 0 | 16 | 0 | 343 | 0 |
| 16660 | 0 | 351 | 0 | 8 | 0 |
| 16686 | 0 | 0 | 0 | 56 | 303 |
| 16687 | 56 | 0 | 0 | 0 | 303 |
| 16696 | 300 | 0 | 59 | 0 | 0 |
| 16700 | 351 | 0 | 8 | 0 | 0 |
| 16705 | 0 | 0 | 0 | 358 | 1 |
| 16706 | 0 | 0 | 358 | 0 | 1 |

### 13.3.2 Diversity per location

```r
strata <- as.character(BS.gt@schemes$Location)
names(strata) <- as.character(BS.gt@schemes$id)
strataG::setStrata(BS.gt) <- strata

divers.location <- data.frame(Location = character(), pi.strataG = numeric(),
                           pi.pegas = numeric(), pi.pegas.var = numeric(),
                           HT.richness.strataG = numeric(), h.pegas = numeric(),
                           h.pegas.var = numeric())
for (Location in levels(BS.gt@schemes$Location)) {
  print(Location)
  BS.gt.sub <- BS.gt[,,Location, drop = TRUE]
  BS.bin.sub <- subset(BS.bin, names(BS.bin) %in% BS.gt.sub@data$id)
  BS.h.sub <- strataG::labelHaplotypes(BS.bin.sub, prefix = "HT",
                                        use.indels = TRUE)
  BS.dna.sub <- as.dna(BS.dna[names(BS.dna) %in% BS.gt.sub@data$id, ,
                              drop = TRUE])
  bases <- haplotypes::basecomp(BS.dna.sub)
  print(paste0("Mitogenome base composition:"))
  print(colMeans(bases))

  pi.gt <-
    strataG::nucleotideDiversity(BS.gt.sub,bases = c("a", "c", "g", "t"),
    simplify = TRUE)
  pi.peg <- pegas::nuc.div(BS.bin.sub, variance = TRUE,
                           pairwise.deletion = FALSE)
  h.peg <- pegas::hap.div(BS.bin.sub, variance = TRUE, method = "Nei")
  res <- data.frame(Location, mean(pi.gt), pi.peg[1], pi.peg[2],
                    length(unique(BS.h.sub$haps)),
                    h.peg[1], h.peg[2])
  colnames(res) <- c("Location", "pi.strataG", "pi.pegas", "pi.pegas.var",
                     "HT.richness.strataG", "h.pegas", "h.pegas.var")
  divers.location <- rbind(divers.location, res)
}

knitr::kable(
  divers.location,
  caption = "Nucleotide and haplotype diversity per Location",
  # format = "html",
  digits = 6
  ) %>%
  kableExtra::kable_styling(full_width = FALSE,font_size = 5,
                            latex_options = c( "hold_position"))
```

```r
dA.gt <- strataG::nucleotideDivergence(BS.gt,
                                       probs = c(0, 0.025, 0.5, 0.975, 1),
                                       model = "RAW")
knitr::kable(
  dA.gt$within[order(dA.gt$within$mean, decreasing = TRUE), -1],
  # format = "html",
  caption = "Nucleotide divergence within each Location",
  digits = 6
  ) %>%
  kableExtra::kable_styling(full_width = FALSE,font_size = 5,
                            latex_options = c( "hold_position"))

kableExtra::kbl(dA.gt$between[order(dA.gt$between$mean, decreasing = TRUE), -1],
  caption = "Nucleotide divergence between each Location",
  longtable = TRUE, booktabs = TRUE) %>%
  kableExtra::kable_styling(full_width = FALSE,font_size = 5,
                            latex_options = c("repeat_header", "hold_position"))

hapcount <- pegas::haploFreq(BS.dna@sequence, fac = BS.gt@schemes$Location,
                             split = NULL, what = 1, haplo = NULL)
row.names(hapcount) <- paste0("HT", sprintf('%0.2d', 1:BS.hap@nhap))
shortnames <- c("GOC", "COR","SIL", "SAF", "RUN", "SEY", "ARS", "SRL", "TAI",
                "IND", "PNG", "FZR", "VIR", "DAR", "ADR","SAR", "EAR","BMB",
                "ROR","TOR","WER","TRI","CLR", "SYH", "URR","OKI", "FIJ")

kableExtra::kbl(hapcount, col.names = shortnames,
  caption = "Haplotype counts per Location according to pegas",
  longtable = TRUE, booktabs = TRUE) %>%
  kableExtra::kable_styling(full_width = FALSE,font_size = 5,
                            latex_options = c("repeat_header", "hold_position")) %>%
  kableExtra::landscape()

# group.levels <- levels(BS.gi$other$ind.meta$Location)
group.levels <- c("Gulf of California","Costa Rica","Sierra Leone","South Africa",
                  "Reunion","Seychelles","Arabian Sea","Sri Lanka", "Thailand",
                  "Indonesia","Papua New Guinea","Fitzroy River","Victoria River",
                  "Daly River","Adelaide River","South Alligator River",
                  "East Alligator River","Blue mud Bay","Roper River","Towns River",
                  "Wenlock River","Trinity inlet","Clarence River","Sydney Harbour",
                  "Okinawa","Urauchi River","Fiji")

group.names <- BS.gi$other$ind.meta$Location

HTs <- paste0("HT", sprintf('%0.3d', 1:BS.hap@nhap))
df <- data.frame(group = NULL,nind = NULL, hap = NULL, nhap = NULL,
                 hapfreq = NULL, stringsAsFactors = FALSE)
for (k in 1:length(group.levels)) {
  group.inds <- adegenet::indNames(BS.gi)[group.names == group.levels[k]]
  for (i in 1:BS.hap@nhap) {
    group <- group.levels[k]
    nind <- length(group.inds)
    hap <- HTs[i]
    nhap <- sum(group.inds %in% BS.hap@haplist[[i]])
    hapfreq <- nhap/nind
    df <- plyr::rbind.fill(df, data.frame(group = group, nind = nind, hap = hap,
                                          nhap = nhap, hapfreq = hapfreq,
                                          stringsAsFactors = FALSE))
```

```
  }
}

df$group <- factor(df$group, levels = group.levels)

haplotype.labels <- unique(df$hap)
haplotype.labels2 <- haplotype.labels[seq(1, length(haplotype.labels), by = 5)]

HT.plot <- ggplot2::ggplot(data = df) +
  ggplot2::geom_bar(ggplot2::aes(x = hap, y = nhap, fill = group),
                    stat = "identity",position = "stack") +
  ggplot2::labs(x = "Haplotypes", y = "Number of individuals") +
  ggplot2::scale_fill_manual(values = colours.27) +
  ggplot2::scale_x_discrete(breaks = haplotype.labels2) +
  ggplot2::theme(
      axis.text = ggplot2::element_text(angle = 45, hjust = 1, size = 5),
      axis.title.x = ggplot2::element_text(size = 10),
      axis.title.y = ggplot2::element_text(size = 10),
      legend.text = ggplot2::element_text(size = 7))
print(HT.plot)
```

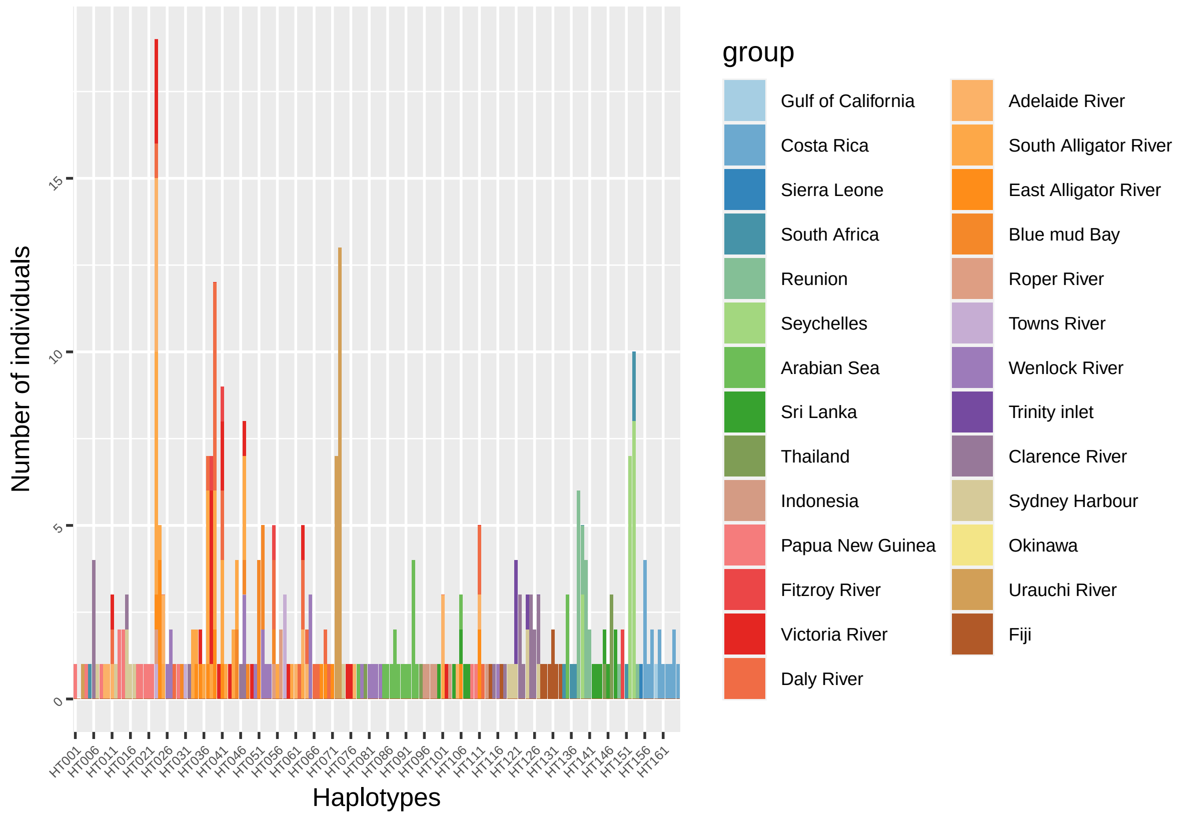


```
ggplot2::ggsave(HT.plot, filename = "HT_barplot_per_Location.png", width = 30,
                height = 15, units = "cm")
```

```
## [1] "Gulf of California"
## [1] "Mitogenome base composition:"
##         A         T         G         C
## 0.3151972 0.2423535 0.1307236 0.3117256
## [1] "Costa Rica"
## [1] "Mitogenome base composition:"
##         A         T         G         C
```

```
## 0.3151516 0.2424022 0.1307582 0.3116879
## [1] "Sierra Leone"
## [1] "Mitogenome base composition:"
##         A         T         G         C
## 0.3145576 0.2423680 0.1312702 0.3118041
## [1] "South Africa"
## [1] "Mitogenome base composition:"
##         A         T         G         C
## 0.3148254 0.2429858 0.1309720 0.3112168
## [1] "Reunion"
## [1] "Mitogenome base composition:"
##         A         T         G         C
## 0.3148585 0.2431229 0.1309273 0.3110912
## [1] "Seychelles"
## [1] "Mitogenome base composition:"
##         A         T         G         C
## 0.3149296 0.2427989 0.1309067 0.3113648
## [1] "Arabian Sea"
## [1] "Mitogenome base composition:"
##         A         T         G         C
## 0.3146861 0.2433287 0.1310578 0.3109275
## [1] "Sri Lanka"
## [1] "Mitogenome base composition:"
##         A         T         G         C
## 0.3147039 0.2430626 0.1310614 0.3111722
## [1] "Thailand"
## [1] "Mitogenome base composition:"
##         A         T         G         C
## 0.3147902 0.2430688 0.1310021 0.3111388
## [1] "Indonesia"
## [1] "Mitogenome base composition:"
##         A         T         G         C
## 0.3145539 0.2432308 0.1311531 0.3110621
## [1] "Papua New Guinea"
## [1] "Mitogenome base composition:"
##         A         T         G         C
## 0.3145063 0.2429284 0.1312395 0.3113257
## [1] "Fitzroy River"
## [1] "Mitogenome base composition:"
##         A         T         G         C
## 0.3146811 0.2429575 0.1311042 0.3112572
## [1] "Victoria River"
## [1] "Mitogenome base composition:"
##         A         T         G         C
## 0.3144800 0.2431203 0.1312718 0.3111278
## [1] "Daly River"
## [1] "Mitogenome base composition:"
##         A         T         G         C
## 0.3145202 0.2431297 0.1312253 0.3111248
## [1] "Adelaide River"
## [1] "Mitogenome base composition:"
##         A         T         G         C
## 0.3144881 0.2430869 0.1312493 0.3111758
## [1] "South Alligator River"
## [1] "Mitogenome base composition:"
##         A         T         G         C
## 0.3144745 0.2430951 0.1312734 0.3111570
## [1] "East Alligator River"
```

```
## [1] "Mitogenome base composition:"
##         A         T         G         C
## 0.3145003 0.2431766 0.1312349 0.3110882
## [1] "Blue mud Bay"
## [1] "Mitogenome base composition:"
##         A         T         G         C
## 0.3144696 0.2430240 0.1312830 0.3112234
## [1] "Roper River"
## [1] "Mitogenome base composition:"
##         A         T         G         C
## 0.3144773 0.2430786 0.1313041 0.3111400
## [1] "Towns River"
## [1] "Mitogenome base composition:"
##         A         T         G         C
## 0.3144650 0.2430620 0.1313124 0.3111606
## [1] "Wenlock River"
## [1] "Mitogenome base composition:"
##         A         T         G         C
## 0.3145160 0.2430987 0.1312248 0.3111606
## [1] "Trinity inlet"
## [1] "Mitogenome base composition:"
##         A         T         G         C
## 0.3146261 0.2429249 0.1311603 0.3112887
## [1] "Clarence River"
## [1] "Mitogenome base composition:"
##         A         T         G         C
## 0.3145786 0.2429470 0.1311568 0.3113176
## [1] "Sydney Harbour"
## [1] "Mitogenome base composition:"
##         A         T         G         C
## 0.3145503 0.2429270 0.1312004 0.3113223
## [1] "Okinawa "
## [1] "Mitogenome base composition:"
##         A         T         G         C
## 0.3147088 0.2429724 0.1310459 0.3112729
## [1] "Urauchi River"
## [1] "Mitogenome base composition:"
##         A         T         G         C
## 0.3146495 0.2431236 0.1311374 0.3110895
## [1] "Fiji"
## [1] "Mitogenome base composition:"
##         A         T         G         C
## 0.3146038 0.2429049 0.1311531 0.3113381
```

Table 55: Nucleotide divergence between each Location

| | strata.1 | strata.2 | dA | mean | q.0 | q.0.025 | q.0.5 | q.0.975 | q.1 |
|---|---|---|---|---|---|---|---|---|---|
| 297 | Sierra Leone | South Africa | 0.0081950 | 0.0091702 | 0.0089820 | 0.0089820 | 0.0092814 | 0.0092814 | 0.0092814 |
| 206 | Gulf of California | South Africa | 0.0081779 | 0.0091531 | 0.0089820 | 0.0089820 | 0.0091617 | 0.0093832 | 0.0094012 |
| 200 | Gulf of California | Okinawa | 0.0088263 | 0.0091138 | 0.0090419 | 0.0090419 | 0.0091617 | 0.0091617 | 0.0091617 |
| 202 | Gulf of California | Reunion | 0.0089646 | 0.0091093 | 0.0090419 | 0.0090419 | 0.0091617 | 0.0091617 | 0.0091617 |
| 263 | Reunion | Sierra Leone | 0.0089646 | 0.0091093 | 0.0090419 | 0.0090419 | 0.0091617 | 0.0091617 | 0.0091617 |
| 111 | Costa Rica | South Africa | 0.0079994 | 0.0091092 | 0.0088623 | 0.0088623 | 0.0091018 | 0.0094012 | 0.0094012 |
| 34 | Arabian Sea | Gulf of California | 0.0082085 | 0.0091049 | 0.0089222 | 0.0089760 | 0.0091018 | 0.0092545 | 0.0092814 |
| 199 | Gulf of California | Indonesia | 0.0088583 | 0.0090818 | 0.0089820 | 0.0089970 | 0.0091018 | 0.0091018 | 0.0091018 |
| 105 | Costa Rica | Okinawa | 0.0086478 | 0.0090699 | 0.0089222 | 0.0089222 | 0.0090419 | 0.0091617 | 0.0091617 |
| 201 | Gulf of California | Papua New Guinea | 0.0087505 | 0.0090659 | 0.0089222 | 0.0089222 | 0.0091018 | 0.0092006 | 0.0092216 |
| 107 | Costa Rica | Reunion | 0.0087861 | 0.0090654 | 0.0089222 | 0.0089222 | 0.0090419 | 0.0091617 | 0.0091617 |
| 29 | Arabian Sea | Costa Rica | 0.0080300 | 0.0090610 | 0.0088024 | 0.0088683 | 0.0090419 | 0.0092216 | 0.0092814 |
| 213 | Gulf of California | Urauchi River | 0.0089963 | 0.0090476 | 0.0090419 | 0.0090419 | 0.0090419 | 0.0091018 | 0.0091617 |
| 104 | Costa Rica | Indonesia | 0.0086798 | 0.0090379 | 0.0088623 | 0.0088757 | 0.0090419 | 0.0091018 | 0.0091018 |
| 210 | Gulf of California | Thailand | 0.0075689 | 0.0090319 | 0.0089222 | 0.0089296 | 0.0090419 | 0.0091467 | 0.0091617 |
| 41 | Arabian Sea | Sierra Leone | 0.0081266 | 0.0090230 | 0.0088024 | 0.0088563 | 0.0089820 | 0.0092814 | 0.0092814 |
| 9 | Adelaide River | Gulf of California | 0.0086730 | 0.0090220 | 0.0087425 | 0.0088189 | 0.0089820 | 0.0092216 | 0.0092216 |
| 106 | Costa Rica | Papua New Guinea | 0.0085720 | 0.0090220 | 0.0088024 | 0.0088383 | 0.0090419 | 0.0092216 | 0.0092216 |
| 163 | Fiji | Gulf of California | 0.0083350 | 0.0090220 | 0.0089820 | 0.0089820 | 0.0090419 | 0.0090419 | 0.0090419 |

Table 55: Nucleotide divergence between each Location *(continued)*

| | strata.1 | strata.2 | dA | mean | q.0 | q.0.025 | q.0.5 | q.0.975 | q.1 |
|---|---|---|---|---|---|---|---|---|---|
| 204 | Gulf of California | Seychelles | 0.0088623 | 0.0090120 | 0.0089820 | 0.0089820 | 0.0089820 | 0.0091617 | 0.0091617 |
| 208 | Gulf of California | Sri Lanka | 0.0074932 | 0.0090120 | 0.0089222 | 0.0089222 | 0.0089820 | 0.0091452 | 0.0091617 |
| 286 | Seychelles | Sierra Leone | 0.0088623 | 0.0090120 | 0.0089820 | 0.0089820 | 0.0089820 | 0.0091617 | 0.0091617 |
| 124 | Daly River | Gulf of California | 0.0087076 | 0.0090084 | 0.0087425 | 0.0087425 | 0.0089820 | 0.0092216 | 0.0092216 |
| 215 | Gulf of California | Wenlock River | 0.0086977 | 0.0090041 | 0.0088623 | 0.0088892 | 0.0089820 | 0.0091018 | 0.0091018 |
| 118 | Costa Rica | Urauchi River | 0.0088178 | 0.0090037 | 0.0089222 | 0.0089222 | 0.0089820 | 0.0091018 | 0.0091617 |
| 209 | Gulf of California | Sydney Harbour | 0.0087049 | 0.0089959 | 0.0087425 | 0.0087605 | 0.0090419 | 0.0092635 | 0.0092814 |
| 236 | Okinawa | Sierra Leone | 0.0087066 | 0.0089940 | 0.0089222 | 0.0089222 | 0.0090419 | 0.0090419 | 0.0090419 |
| 301 | Sierra Leone | Thailand | 0.0075289 | 0.0089920 | 0.0088024 | 0.0088249 | 0.0090419 | 0.0090419 | 0.0090419 |
| 115 | Costa Rica | Thailand | 0.0073904 | 0.0089880 | 0.0088024 | 0.0088159 | 0.0089820 | 0.0091617 | 0.0091617 |
| 144 | East Alligator River | Gulf of California | 0.0087240 | 0.0089785 | 0.0087425 | 0.0087425 | 0.0089820 | 0.0091617 | 0.0091617 |
| 4 | Adelaide River | Costa Rica | 0.0084945 | 0.0089780 | 0.0086228 | 0.0086826 | 0.0089222 | 0.0092216 | 0.0092216 |
| 101 | Costa Rica | Fiji | 0.0081565 | 0.0089780 | 0.0088623 | 0.0088623 | 0.0089820 | 0.0090419 | 0.0090419 |
| 214 | Gulf of California | Victoria River | 0.0087542 | 0.0089763 | 0.0088623 | 0.0088623 | 0.0089222 | 0.0091617 | 0.0091617 |
| 211 | Gulf of California | Towns River | 0.0089042 | 0.0089701 | 0.0089222 | 0.0089281 | 0.0089820 | 0.0089820 | 0.0089820 |
| 109 | Costa Rica | Seychelles | 0.0086838 | 0.0089681 | 0.0088623 | 0.0088623 | 0.0089820 | 0.0091617 | 0.0091617 |
| 113 | Costa Rica | Sri Lanka | 0.0073147 | 0.0089681 | 0.0088024 | 0.0088024 | 0.0089820 | 0.0091617 | 0.0091617 |
| 99 | Costa Rica | Daly River | 0.0085291 | 0.0089645 | 0.0086228 | 0.0086826 | 0.0089820 | 0.0092216 | 0.0092216 |
| 221 | Indonesia | Sierra Leone | 0.0087385 | 0.0089621 | 0.0088623 | 0.0088772 | 0.0089820 | 0.0089820 | 0.0089820 |
| 299 | Sierra Leone | Sri Lanka | 0.0074433 | 0.0089621 | 0.0088024 | 0.0088024 | 0.0089820 | 0.0091452 | 0.0091617 |
| 120 | Costa Rica | Wenlock River | 0.0085192 | 0.0089602 | 0.0087425 | 0.0088024 | 0.0089820 | 0.0091018 | 0.0091018 |
| 58 | Blue mud Bay | Gulf of California | 0.0089068 | 0.0089590 | 0.0088623 | 0.0088802 | 0.0089820 | 0.0089820 | 0.0089820 |
| 114 | Costa Rica | Sydney Harbour | 0.0085264 | 0.0089519 | 0.0086228 | 0.0086826 | 0.0089820 | 0.0092814 | 0.0092814 |
| 207 | Gulf of California | South Alligator River | 0.0088432 | 0.0089474 | 0.0088623 | 0.0088623 | 0.0089222 | 0.0090509 | 0.0091617 |
| 181 | Fitzroy River | Gulf of California | 0.0073713 | 0.0089461 | 0.0088623 | 0.0088623 | 0.0089222 | 0.0090419 | 0.0090419 |
| 250 | Papua New Guinea | Sierra Leone | 0.0086307 | 0.0089461 | 0.0088024 | 0.0088024 | 0.0089820 | 0.0090808 | 0.0091018 |
| 203 | Gulf of California | Roper River | 0.0088822 | 0.0089371 | 0.0088623 | 0.0088668 | 0.0089521 | 0.0089820 | 0.0089820 |
| 100 | Costa Rica | East Alligator River | 0.0085455 | 0.0089346 | 0.0086228 | 0.0086826 | 0.0089222 | 0.0091617 | 0.0091617 |
| 119 | Costa Rica | Victoria River | 0.0085746 | 0.0089313 | 0.0087425 | 0.0087425 | 0.0089222 | 0.0091617 | 0.0091617 |
| 304 | Sierra Leone | Urauchi River | 0.0088765 | 0.0089279 | 0.0089222 | 0.0089222 | 0.0089222 | 0.0089820 | 0.0090419 |
| 116 | Costa Rica | Towns River | 0.0087257 | 0.0089261 | 0.0088024 | 0.0088024 | 0.0089222 | 0.0089820 | 0.0089820 |
| 53 | Blue mud Bay | Costa Rica | 0.0087283 | 0.0089151 | 0.0087425 | 0.0088024 | 0.0089222 | 0.0089820 | 0.0089820 |
| 112 | Costa Rica | South Alligator River | 0.0086647 | 0.0089035 | 0.0087425 | 0.0088024 | 0.0089222 | 0.0090419 | 0.0091617 |
| 102 | Costa Rica | Fitzroy River | 0.0071928 | 0.0089022 | 0.0087425 | 0.0087425 | 0.0088623 | 0.0090419 | 0.0090419 |
| 170 | Fiji | Sierra Leone | 0.0082152 | 0.0089022 | 0.0088623 | 0.0088623 | 0.0089222 | 0.0089222 | 0.0089222 |
| 108 | Costa Rica | Roper River | 0.0087037 | 0.0088932 | 0.0087425 | 0.0087425 | 0.0089222 | 0.0089820 | 0.0089820 |
| 16 | Adelaide River | Sierra Leone | 0.0085400 | 0.0088889 | 0.0086228 | 0.0086991 | 0.0088623 | 0.0091018 | 0.0091018 |
| 81 | Clarence River | Gulf of California | 0.0087257 | 0.0088765 | 0.0087425 | 0.0087725 | 0.0088623 | 0.0091018 | 0.0091018 |
| 300 | Sierra Leone | Sydney Harbour | 0.0085851 | 0.0088761 | 0.0086228 | 0.0086407 | 0.0089222 | 0.0091437 | 0.0091617 |
| 188 | Fitzroy River | Sierra Leone | 0.0072994 | 0.0088743 | 0.0087425 | 0.0087425 | 0.0088024 | 0.0090419 | 0.0090419 |
| 306 | Sierra Leone | Wenlock River | 0.0085653 | 0.0088717 | 0.0088024 | 0.0088024 | 0.0088623 | 0.0089222 | 0.0089222 |
| 131 | Daly River | Sierra Leone | 0.0085687 | 0.0088695 | 0.0086228 | 0.0086228 | 0.0088623 | 0.0091018 | 0.0091018 |
| 151 | East Alligator River | Sierra Leone | 0.0086043 | 0.0088588 | 0.0086228 | 0.0086228 | 0.0088623 | 0.0090419 | 0.0090419 |
| 305 | Sierra Leone | Victoria River | 0.0086287 | 0.0088509 | 0.0087425 | 0.0087425 | 0.0088024 | 0.0090419 | 0.0090419 |
| 302 | Sierra Leone | Towns River | 0.0087844 | 0.0088503 | 0.0088024 | 0.0088084 | 0.0088623 | 0.0088623 | 0.0088623 |
| 65 | Blue mud Bay | Sierra Leone | 0.0087870 | 0.0088392 | 0.0087425 | 0.0087605 | 0.0088623 | 0.0088623 | 0.0088623 |
| 76 | Clarence River | Costa Rica | 0.0085472 | 0.0088326 | 0.0086228 | 0.0086826 | 0.0088024 | 0.0091018 | 0.0091018 |
| 298 | Sierra Leone | South Alligator River | 0.0087203 | 0.0088245 | 0.0087425 | 0.0087425 | 0.0088024 | 0.0089222 | 0.0089222 |
| 275 | Roper River | Sierra Leone | 0.0087625 | 0.0088174 | 0.0087425 | 0.0087470 | 0.0088323 | 0.0088623 | 0.0088623 |
| 212 | Gulf of California | Trinity inlet | 0.0088024 | 0.0088024 | 0.0088024 | 0.0088024 | 0.0088024 | 0.0088024 | 0.0088024 |
| 88 | Clarence River | Sierra Leone | 0.0086116 | 0.0087625 | 0.0086826 | 0.0086826 | 0.0087425 | 0.0089820 | 0.0089820 |
| 117 | Costa Rica | Trinity inlet | 0.0086239 | 0.0087585 | 0.0086826 | 0.0086826 | 0.0087425 | 0.0088024 | 0.0088024 |
| 303 | Sierra Leone | Trinity inlet | 0.0086826 | 0.0086826 | 0.0086826 | 0.0086826 | 0.0086826 | 0.0086826 | 0.0086826 |
| 205 | Gulf of California | Sierra Leone | 0.0063473 | 0.0063473 | 0.0063473 | 0.0063473 | 0.0063473 | 0.0063473 | 0.0063473 |
| 110 | Costa Rica | Sierra Leone | 0.0061688 | 0.0063034 | 0.0062275 | 0.0062275 | 0.0062874 | 0.0063473 | 0.0063473 |
| 233 | Okinawa | Reunion | 0.0053298 | 0.0057620 | 0.0052695 | 0.0053862 | 0.0058683 | 0.0059880 | 0.0059880 |
| 273 | Reunion | Wenlock River | 0.0051835 | 0.0056347 | 0.0052695 | 0.0053293 | 0.0056287 | 0.0058084 | 0.0058084 |
| 13 | Adelaide River | Reunion | 0.0051326 | 0.0056262 | 0.0050898 | 0.0052695 | 0.0056287 | 0.0059177 | 0.0059281 |
| 128 | Daly River | Reunion | 0.0051775 | 0.0056231 | 0.0050898 | 0.0052695 | 0.0056287 | 0.0059281 | 0.0059281 |
| 148 | East Alligator River | Reunion | 0.0052036 | 0.0056028 | 0.0050898 | 0.0052695 | 0.0056287 | 0.0058683 | 0.0058683 |
| 247 | Papua New Guinea | Reunion | 0.0051292 | 0.0055893 | 0.0052096 | 0.0053278 | 0.0055689 | 0.0059880 | 0.0059880 |
| 272 | Reunion | Victoria River | 0.0052170 | 0.0055838 | 0.0052096 | 0.0052695 | 0.0056287 | 0.0058683 | 0.0058683 |
| 62 | Blue mud Bay | Reunion | 0.0053863 | 0.0055833 | 0.0052096 | 0.0053293 | 0.0055689 | 0.0056886 | 0.0056886 |
| 267 | Reunion | Sydney Harbour | 0.0051384 | 0.0055740 | 0.0050898 | 0.0052201 | 0.0055689 | 0.0059281 | 0.0059281 |
| 265 | Reunion | South Alligator River | 0.0053227 | 0.0055716 | 0.0052096 | 0.0052695 | 0.0055689 | 0.0057485 | 0.0058683 |
| 218 | Indonesia | Reunion | 0.0051981 | 0.0055664 | 0.0052096 | 0.0052545 | 0.0055689 | 0.0058084 | 0.0058084 |
| 271 | Reunion | Urauchi River | 0.0053675 | 0.0055635 | 0.0052695 | 0.0052695 | 0.0056287 | 0.0058683 | 0.0059880 |
| 269 | Reunion | Towns River | 0.0053164 | 0.0055269 | 0.0052695 | 0.0053293 | 0.0055689 | 0.0056886 | 0.0056886 |
| 261 | Reunion | Roper River | 0.0053056 | 0.0055052 | 0.0052096 | 0.0053039 | 0.0055389 | 0.0056287 | 0.0056287 |
| 85 | Clarence River | Reunion | 0.0052052 | 0.0055008 | 0.0051497 | 0.0052096 | 0.0055090 | 0.0056886 | 0.0058084 |
| 235 | Okinawa | Seychelles | 0.0050579 | 0.0054950 | 0.0052096 | 0.0052096 | 0.0055689 | 0.0059880 | 0.0059880 |
| 270 | Reunion | Trinity inlet | 0.0052819 | 0.0054266 | 0.0051497 | 0.0051497 | 0.0054491 | 0.0055090 | 0.0055090 |
| 296 | Seychelles | Wenlock River | 0.0049116 | 0.0053677 | 0.0052096 | 0.0052096 | 0.0053293 | 0.0057769 | 0.0058084 |
| 15 | Adelaide River | Seychelles | 0.0048607 | 0.0053593 | 0.0050299 | 0.0050299 | 0.0053293 | 0.0058638 | 0.0059281 |
| 130 | Daly River | Seychelles | 0.0049056 | 0.0053561 | 0.0050299 | 0.0050299 | 0.0052994 | 0.0058683 | 0.0059281 |
| 249 | Papua New Guinea | Seychelles | 0.0048743 | 0.0053393 | 0.0051497 | 0.0051497 | 0.0052695 | 0.0056886 | 0.0059880 |
| 150 | East Alligator River | Seychelles | 0.0049316 | 0.0053358 | 0.0050299 | 0.0050299 | 0.0053293 | 0.0058084 | 0.0058683 |
| 295 | Seychelles | Victoria River | 0.0049494 | 0.0053213 | 0.0051497 | 0.0051497 | 0.0052096 | 0.0057485 | 0.0058683 |
| 64 | Blue mud Bay | Seychelles | 0.0051144 | 0.0053163 | 0.0051497 | 0.0051497 | 0.0052695 | 0.0056886 | 0.0056886 |
| 292 | Seychelles | Towns River | 0.0050998 | 0.0053154 | 0.0052096 | 0.0052096 | 0.0052695 | 0.0056751 | 0.0056886 |
| 290 | Seychelles | Sydney Harbour | 0.0048664 | 0.0053071 | 0.0050299 | 0.0050299 | 0.0052695 | 0.0057695 | 0.0059281 |
| 288 | Seychelles | South Alligator River | 0.0050508 | 0.0053047 | 0.0051497 | 0.0051497 | 0.0052096 | 0.0056886 | 0.0058683 |
| 220 | Indonesia | Seychelles | 0.0049261 | 0.0052994 | 0.0051497 | 0.0051497 | 0.0052695 | 0.0057275 | 0.0058084 |
| 294 | Seychelles | Urauchi River | 0.0050955 | 0.0052965 | 0.0052096 | 0.0052096 | 0.0052096 | 0.0056287 | 0.0059880 |

Table 55: Nucleotide divergence between each Location *(continued)*

| | strata.1 | strata.2 | dA | mean | q.0 | q.0.025 | q.0.5 | q.0.975 | q.1 |
|---|---|---|---|---|---|---|---|---|---|
| 274 | Roper River | Seychelles | 0.0050798 | 0.0052844 | 0.0051497 | 0.0051497 | 0.0052695 | 0.0056287 | 0.0056287 |
| 87 | Clarence River | Seychelles | 0.0049333 | 0.0052338 | 0.0050898 | 0.0050898 | 0.0051497 | 0.0056287 | 0.0058084 |
| 293 | Seychelles | Trinity inlet | 0.0050100 | 0.0051597 | 0.0050898 | 0.0050898 | 0.0050898 | 0.0055090 | 0.0055090 |
| 237 | Okinawa | South Africa | 0.0038785 | 0.0051411 | 0.0015569 | 0.0015569 | 0.0056886 | 0.0061078 | 0.0061078 |
| 167 | Fiji | Reunion | 0.0041974 | 0.0050291 | 0.0003593 | 0.0009581 | 0.0055090 | 0.0056287 | 0.0056287 |
| 315 | South Africa | Wenlock River | 0.0037074 | 0.0049890 | 0.0011976 | 0.0012575 | 0.0053892 | 0.0059281 | 0.0059281 |
| 132 | Daly River | South Africa | 0.0036989 | 0.0049749 | 0.0009581 | 0.0011587 | 0.0055090 | 0.0060479 | 0.0060479 |
| 17 | Adelaide River | South Africa | 0.0036450 | 0.0049691 | 0.0009581 | 0.0011976 | 0.0054491 | 0.0059880 | 0.0060479 |
| 222 | Indonesia | South Africa | 0.0037599 | 0.0049587 | 0.0014970 | 0.0016168 | 0.0053892 | 0.0059281 | 0.0059281 |
| 152 | East Alligator River | South Africa | 0.0037233 | 0.0049530 | 0.0011377 | 0.0012545 | 0.0054491 | 0.0059880 | 0.0059880 |
| 313 | South Africa | Urauchi River | 0.0039256 | 0.0049521 | 0.0015569 | 0.0015569 | 0.0053293 | 0.0057485 | 0.0061078 |
| 314 | South Africa | Victoria River | 0.0037316 | 0.0049289 | 0.0009581 | 0.0011976 | 0.0053892 | 0.0059880 | 0.0059880 |
| 66 | Blue mud Bay | South Africa | 0.0038940 | 0.0049214 | 0.0011377 | 0.0011976 | 0.0053892 | 0.0058084 | 0.0058084 |
| 307 | South Africa | South Alligator River | 0.0038322 | 0.0049115 | 0.0011377 | 0.0011976 | 0.0053293 | 0.0058683 | 0.0059880 |
| 251 | Papua New Guinea | South Africa | 0.0036145 | 0.0049050 | 0.0006587 | 0.0007784 | 0.0053892 | 0.0060000 | 0.0061078 |
| 309 | South Africa | Sydney Harbour | 0.0036342 | 0.0049003 | 0.0007784 | 0.0007934 | 0.0054491 | 0.0060329 | 0.0060479 |
| 311 | South Africa | Towns River | 0.0038503 | 0.0048914 | 0.0011976 | 0.0012485 | 0.0052695 | 0.0058084 | 0.0058084 |
| 38 | Arabian Sea | Reunion | 0.0038407 | 0.0048818 | 0.0002395 | 0.0002395 | 0.0056886 | 0.0059536 | 0.0059880 |
| 276 | Roper River | South Africa | 0.0038352 | 0.0048653 | 0.0011377 | 0.0011781 | 0.0052695 | 0.0057485 | 0.0057485 |
| 89 | Clarence River | South Africa | 0.0037373 | 0.0048633 | 0.0007784 | 0.0012754 | 0.0052695 | 0.0058084 | 0.0059281 |
| 169 | Fiji | Seychelles | 0.0039726 | 0.0048093 | 0.0002994 | 0.0002994 | 0.0053293 | 0.0056287 | 0.0056287 |
| 312 | South Africa | Trinity inlet | 0.0038238 | 0.0047990 | 0.0013174 | 0.0013174 | 0.0052096 | 0.0056287 | 0.0056287 |
| 40 | Arabian Sea | Seychelles | 0.0037038 | 0.0047500 | 0.0003593 | 0.0007680 | 0.0053892 | 0.0058368 | 0.0059880 |
| 171 | Fiji | South Africa | 0.0029002 | 0.0045623 | 0.0002994 | 0.0003653 | 0.0053892 | 0.0057485 | 0.0057485 |
| 42 | Arabian Sea | South Africa | 0.0026248 | 0.0044964 | 0.0001198 | 0.0001198 | 0.0054491 | 0.0060479 | 0.0061078 |
| 241 | Okinawa | Thailand | 0.0022315 | 0.0039820 | 0.0003593 | 0.0003593 | 0.0052695 | 0.0057485 | 0.0057485 |
| 255 | Papua New Guinea | Thailand | 0.0021450 | 0.0039235 | 0.0002395 | 0.0003593 | 0.0053293 | 0.0057350 | 0.0057485 |
| 337 | Thailand | Towns River | 0.0023812 | 0.0039102 | 0.0007186 | 0.0007620 | 0.0053892 | 0.0054491 | 0.0054491 |
| 70 | Blue mud Bay | Thailand | 0.0023838 | 0.0038991 | 0.0006587 | 0.0007186 | 0.0053892 | 0.0054491 | 0.0054491 |
| 331 | Sydney Harbour | Thailand | 0.0021267 | 0.0038807 | 0.0005389 | 0.0005988 | 0.0052695 | 0.0056287 | 0.0056287 |
| 280 | Roper River | Thailand | 0.0023593 | 0.0038772 | 0.0006587 | 0.0006931 | 0.0053293 | 0.0054491 | 0.0054491 |
| 318 | South Alligator River | Thailand | 0.0023087 | 0.0038759 | 0.0002395 | 0.0006587 | 0.0053293 | 0.0055090 | 0.0055689 |
| 21 | Adelaide River | Thailand | 0.0020592 | 0.0038711 | 0.0001796 | 0.0002799 | 0.0053293 | 0.0055689 | 0.0056287 |
| 340 | Thailand | Victoria River | 0.0021818 | 0.0038670 | 0.0003593 | 0.0004940 | 0.0053293 | 0.0055090 | 0.0055689 |
| 136 | Daly River | Thailand | 0.0020864 | 0.0038503 | 0.0001796 | 0.0002994 | 0.0053293 | 0.0055689 | 0.0056287 |
| 341 | Thailand | Wenlock River | 0.0020802 | 0.0038497 | 0.0001796 | 0.0002395 | 0.0053892 | 0.0055090 | 0.0055090 |
| 156 | East Alligator River | Thailand | 0.0021271 | 0.0038447 | 0.0001796 | 0.0002710 | 0.0053293 | 0.0055090 | 0.0055689 |
| 93 | Clarence River | Thailand | 0.0022027 | 0.0038167 | 0.0005988 | 0.0005988 | 0.0052695 | 0.0055015 | 0.0055689 |
| 226 | Indonesia | Thailand | 0.0020592 | 0.0037458 | 0.0002994 | 0.0004042 | 0.0052096 | 0.0055689 | 0.0055689 |
| 338 | Thailand | Trinity inlet | 0.0022794 | 0.0037425 | 0.0005988 | 0.0005988 | 0.0052395 | 0.0052695 | 0.0052695 |
| 175 | Fiji | Thailand | 0.0015703 | 0.0037203 | 0.0000599 | 0.0003593 | 0.0054192 | 0.0055090 | 0.0055090 |
| 185 | Fitzroy River | Reunion | 0.0020005 | 0.0037201 | 0.0002395 | 0.0003563 | 0.0054491 | 0.0056287 | 0.0056287 |
| 339 | Thailand | Urauchi River | 0.0021920 | 0.0037064 | 0.0003593 | 0.0003593 | 0.0052695 | 0.0056437 | 0.0057485 |
| 46 | Arabian Sea | Thailand | 0.0012181 | 0.0035776 | 0.0001198 | 0.0001198 | 0.0053892 | 0.0057695 | 0.0058683 |
| 239 | Okinawa | Sri Lanka | 0.0017706 | 0.0035768 | 0.0003593 | 0.0003593 | 0.0052695 | 0.0058683 | 0.0058683 |
| 253 | Papua New Guinea | Sri Lanka | 0.0016908 | 0.0035250 | 0.0002395 | 0.0002994 | 0.0053293 | 0.0056886 | 0.0058683 |
| 326 | Sri Lanka | Towns River | 0.0019163 | 0.0035010 | 0.0007186 | 0.0007470 | 0.0053892 | 0.0055090 | 0.0055090 |
| 68 | Blue mud Bay | Sri Lanka | 0.0019174 | 0.0034884 | 0.0006587 | 0.0007186 | 0.0053293 | 0.0055090 | 0.0055090 |
| 324 | Sri Lanka | Sydney Harbour | 0.0016760 | 0.0034857 | 0.0003593 | 0.0004716 | 0.0052096 | 0.0056886 | 0.0058084 |
| 278 | Roper River | Sri Lanka | 0.0018944 | 0.0034681 | 0.0006587 | 0.0006692 | 0.0053293 | 0.0054985 | 0.0055090 |
| 316 | South Alligator River | Sri Lanka | 0.0018413 | 0.0034643 | 0.0000599 | 0.0006587 | 0.0053293 | 0.0055090 | 0.0057485 |
| 329 | Sri Lanka | Victoria River | 0.0017100 | 0.0034510 | 0.0001796 | 0.0002994 | 0.0053293 | 0.0056287 | 0.0057485 |
| 19 | Adelaide River | Sri Lanka | 0.0015829 | 0.0034506 | 0.0000599 | 0.0000823 | 0.0053293 | 0.0056886 | 0.0058084 |
| 134 | Daly River | Sri Lanka | 0.0016279 | 0.0034475 | 0.0001796 | 0.0002395 | 0.0052695 | 0.0056886 | 0.0058084 |
| 330 | Sri Lanka | Wenlock River | 0.0016216 | 0.0034468 | 0.0002395 | 0.0002994 | 0.0053293 | 0.0056287 | 0.0056886 |
| 189 | Fitzroy River | South Africa | 0.0008871 | 0.0034371 | 0.0001796 | 0.0001796 | 0.0051497 | 0.0057485 | 0.0057485 |
| 154 | East Alligator River | Sri Lanka | 0.0016604 | 0.0034337 | 0.0000000 | 0.0001796 | 0.0052695 | 0.0056287 | 0.0056886 |
| 91 | Clarence River | Sri Lanka | 0.0017402 | 0.0034098 | 0.0005988 | 0.0005988 | 0.0052096 | 0.0055090 | 0.0056886 |
| 173 | Fiji | Sri Lanka | 0.0011952 | 0.0034010 | 0.0000599 | 0.0003398 | 0.0052695 | 0.0055090 | 0.0055090 |
| 327 | Sri Lanka | Trinity inlet | 0.0018146 | 0.0033333 | 0.0005988 | 0.0005988 | 0.0051796 | 0.0053892 | 0.0053892 |
| 44 | Arabian Sea | Sri Lanka | 0.0009149 | 0.0033302 | 0.0000000 | 0.0003593 | 0.0053293 | 0.0057485 | 0.0058084 |
| 328 | Sri Lanka | Urauchi River | 0.0017366 | 0.0033067 | 0.0003593 | 0.0003593 | 0.0052096 | 0.0056392 | 0.0058683 |
| 224 | Indonesia | Sri Lanka | 0.0015436 | 0.0032859 | 0.0001198 | 0.0001662 | 0.0051796 | 0.0055823 | 0.0056886 |
| 187 | Fitzroy River | Seychelles | 0.0015589 | 0.0032834 | 0.0001796 | 0.0001796 | 0.0051497 | 0.0056153 | 0.0056287 |
| 193 | Fitzroy River | Thailand | 0.0000898 | 0.0031277 | 0.0004192 | 0.0004192 | 0.0052695 | 0.0053892 | 0.0053892 |
| 191 | Fitzroy River | Sri Lanka | -0.0000158 | 0.0030778 | 0.0004192 | 0.0004192 | 0.0052695 | 0.0053892 | 0.0054491 |
| 33 | Arabian Sea | Fitzroy River | 0.0004156 | 0.0028869 | 0.0006587 | 0.0007395 | 0.0010778 | 0.0057275 | 0.0057485 |
| 308 | South Africa | Sri Lanka | 0.0003717 | 0.0028657 | 0.0002395 | 0.0002395 | 0.0013174 | 0.0058638 | 0.0058683 |
| 266 | Reunion | Sri Lanka | 0.0011883 | 0.0028518 | 0.0002994 | 0.0004057 | 0.0010180 | 0.0057485 | 0.0057485 |
| 183 | Fitzroy River | Okinawa | 0.0008970 | 0.0027593 | 0.0007784 | 0.0007784 | 0.0010778 | 0.0056287 | 0.0056287 |
| 325 | Sri Lanka | Thailand | -0.0002457 | 0.0027362 | 0.0000000 | 0.0000599 | 0.0009581 | 0.0055090 | 0.0055689 |
| 162 | Fiji | Fitzroy River | 0.0004381 | 0.0026999 | 0.0004790 | 0.0005030 | 0.0008383 | 0.0053892 | 0.0053892 |
| 182 | Fitzroy River | Indonesia | 0.0008204 | 0.0026188 | 0.0007186 | 0.0007186 | 0.0008982 | 0.0054491 | 0.0054491 |
| 196 | Fitzroy River | Urauchi River | 0.0009743 | 0.0026005 | 0.0007784 | 0.0007784 | 0.0008383 | 0.0052695 | 0.0056287 |
| 310 | South Africa | Thailand | 0.0001095 | 0.0025478 | 0.0002395 | 0.0002410 | 0.0011976 | 0.0058683 | 0.0058683 |
| 289 | Seychelles | Sri Lanka | 0.0008598 | 0.0025283 | 0.0002395 | 0.0002395 | 0.0009281 | 0.0056886 | 0.0057485 |
| 184 | Fitzroy River | Papua New Guinea | 0.0006311 | 0.0025214 | 0.0001796 | 0.0002305 | 0.0007186 | 0.0055778 | 0.0056287 |
| 192 | Fitzroy River | Sydney Harbour | 0.0006436 | 0.0025094 | 0.0005389 | 0.0005389 | 0.0007186 | 0.0055329 | 0.0055689 |
| 268 | Reunion | Thailand | 0.0008847 | 0.0024925 | 0.0002994 | 0.0003593 | 0.0010778 | 0.0057485 | 0.0057485 |
| 8 | Adelaide River | Fitzroy River | 0.0005114 | 0.0024351 | 0.0000599 | 0.0000599 | 0.0008982 | 0.0055090 | 0.0055689 |
| 80 | Clarence River | Fitzroy River | 0.0006958 | 0.0024214 | 0.0000599 | 0.0000599 | 0.0006587 | 0.0053293 | 0.0054491 |
| 198 | Fitzroy River | Wenlock River | 0.0005329 | 0.0024141 | 0.0000599 | 0.0000599 | 0.0008383 | 0.0054491 | 0.0054491 |
| 123 | Daly River | Fitzroy River | 0.0005244 | 0.0024000 | 0.0000000 | 0.0000000 | 0.0008982 | 0.0055689 | 0.0055689 |
| 195 | Fitzroy River | Trinity inlet | 0.0008204 | 0.0023952 | 0.0005389 | 0.0005389 | 0.0005988 | 0.0051497 | 0.0051497 |

Table 55: Nucleotide divergence between each Location *(continued)*

| | strata.1 | strata.2 | dA | mean | q.0 | q.0.025 | q.0.5 | q.0.975 | q.1 |
|---|---|---|---|---|---|---|---|---|---|
| 143 | East Alligator River | Fitzroy River | 0.0005053 | 0.0023346 | 0.0000000 | 0.0000599 | 0.0008383 | 0.0055090 | 0.0055090 |
| 197 | Fitzroy River | Victoria River | 0.0004836 | 0.0022806 | 0.0000000 | 0.0000000 | 0.0008383 | 0.0055090 | 0.0055090 |
| 194 | Fitzroy River | Towns River | 0.0005629 | 0.0022036 | 0.0000599 | 0.0000599 | 0.0001796 | 0.0053293 | 0.0053293 |
| 190 | Fitzroy River | South Alligator River | 0.0005145 | 0.0021935 | 0.0000000 | 0.0000000 | 0.0001198 | 0.0053892 | 0.0055090 |
| 57 | Blue mud Bay | Fitzroy River | 0.0005655 | 0.0021925 | 0.0000000 | 0.0000359 | 0.0001796 | 0.0053293 | 0.0053293 |
| 186 | Fitzroy River | Roper River | 0.0005409 | 0.0021707 | 0.0000000 | 0.0000000 | 0.0001497 | 0.0053293 | 0.0053293 |
| 32 | Arabian Sea | Fiji | 0.0004830 | 0.0020664 | 0.0007186 | 0.0008383 | 0.0010180 | 0.0057485 | 0.0057485 |
| 291 | Seychelles | Thailand | 0.0004431 | 0.0020559 | 0.0002395 | 0.0002395 | 0.0002994 | 0.0056677 | 0.0057485 |
| 37 | Arabian Sea | Papua New Guinea | 0.0005237 | 0.0017355 | 0.0002395 | 0.0003593 | 0.0010778 | 0.0058084 | 0.0061078 |
| 47 | Arabian Sea | Towns River | 0.0007320 | 0.0016943 | 0.0007186 | 0.0007784 | 0.0009581 | 0.0057874 | 0.0058084 |
| 27 | Arabian Sea | Blue mud Bay | 0.0007450 | 0.0016936 | 0.0006587 | 0.0007784 | 0.0009581 | 0.0058084 | 0.0058084 |
| 45 | Arabian Sea | Sydney Harbour | 0.0005045 | 0.0016919 | 0.0003593 | 0.0006587 | 0.0010180 | 0.0058683 | 0.0059880 |
| 36 | Arabian Sea | Okinawa | 0.0005079 | 0.0016918 | 0.0003593 | 0.0004790 | 0.0010778 | 0.0061078 | 0.0061078 |
| 39 | Arabian Sea | Roper River | 0.0007119 | 0.0016633 | 0.0006587 | 0.0007710 | 0.0008982 | 0.0057485 | 0.0057485 |
| 43 | Arabian Sea | South Alligator River | 0.0006600 | 0.0016606 | 0.0000599 | 0.0005988 | 0.0008982 | 0.0058084 | 0.0059880 |
| 50 | Arabian Sea | Victoria River | 0.0005133 | 0.0016319 | 0.0001796 | 0.0005389 | 0.0008982 | 0.0058683 | 0.0059880 |
| 28 | Arabian Sea | Clarence River | 0.0005690 | 0.0016163 | 0.0005988 | 0.0006587 | 0.0008383 | 0.0057485 | 0.0059281 |
| 1 | Adelaide River | Arabian Sea | 0.0003674 | 0.0016127 | 0.0000599 | 0.0003308 | 0.0008982 | 0.0059281 | 0.0059880 |
| 165 | Fiji | Okinawa | 0.0006025 | 0.0015768 | 0.0008383 | 0.0008383 | 0.0011377 | 0.0057485 | 0.0057485 |
| 30 | Arabian Sea | Daly River | 0.0003677 | 0.0015650 | 0.0001796 | 0.0002994 | 0.0008383 | 0.0059880 | 0.0060479 |
| 31 | Arabian Sea | East Alligator River | 0.0004134 | 0.0015643 | 0.0000000 | 0.0002994 | 0.0008982 | 0.0059251 | 0.0059880 |
| 51 | Arabian Sea | Wenlock River | 0.0003530 | 0.0015559 | 0.0002395 | 0.0002994 | 0.0008982 | 0.0058683 | 0.0059281 |
| 48 | Arabian Sea | Trinity inlet | 0.0006415 | 0.0015380 | 0.0005988 | 0.0005988 | 0.0007784 | 0.0056287 | 0.0056287 |
| 166 | Fiji | Papua New Guinea | 0.0004459 | 0.0014482 | 0.0005988 | 0.0006587 | 0.0009581 | 0.0054491 | 0.0057485 |
| 35 | Arabian Sea | Indonesia | 0.0003155 | 0.0014355 | 0.0001198 | 0.0002395 | 0.0005988 | 0.0057305 | 0.0059281 |
| 7 | Adelaide River | Fiji | 0.0003953 | 0.0014312 | 0.0005988 | 0.0006587 | 0.0008982 | 0.0055674 | 0.0056886 |
| 178 | Fiji | Urauchi River | 0.0006916 | 0.0014298 | 0.0008383 | 0.0008383 | 0.0009581 | 0.0053892 | 0.0057485 |
| 164 | Fiji | Indonesia | 0.0005111 | 0.0014216 | 0.0007186 | 0.0007186 | 0.0009581 | 0.0054491 | 0.0055689 |
| 122 | Daly River | Fiji | 0.0004161 | 0.0014039 | 0.0005988 | 0.0006347 | 0.0008982 | 0.0055928 | 0.0056886 |
| 180 | Fiji | Wenlock River | 0.0003909 | 0.0013842 | 0.0004192 | 0.0004790 | 0.0008982 | 0.0055090 | 0.0055689 |
| 49 | Arabian Sea | Urauchi River | 0.0004341 | 0.0013819 | 0.0003593 | 0.0003593 | 0.0005389 | 0.0057485 | 0.0061078 |
| 142 | East Alligator River | Fiji | 0.0004319 | 0.0013733 | 0.0005988 | 0.0005988 | 0.0008982 | 0.0055090 | 0.0056287 |
| 179 | Fiji | Victoria River | 0.0004640 | 0.0013731 | 0.0007186 | 0.0007186 | 0.0008383 | 0.0054491 | 0.0056287 |
| 176 | Fiji | Towns River | 0.0006191 | 0.0013719 | 0.0007784 | 0.0007784 | 0.0008982 | 0.0054491 | 0.0054491 |
| 56 | Blue mud Bay | Fiji | 0.0006217 | 0.0013609 | 0.0007186 | 0.0007725 | 0.0008383 | 0.0054491 | 0.0054491 |
| 172 | Fiji | South Alligator River | 0.0005574 | 0.0013485 | 0.0007186 | 0.0007186 | 0.0008383 | 0.0054491 | 0.0056287 |
| 168 | Fiji | Roper River | 0.0005971 | 0.0013390 | 0.0007186 | 0.0007186 | 0.0008383 | 0.0054491 | 0.0054491 |
| 264 | Reunion | South Africa | 0.0001344 | 0.0012543 | 0.0000599 | 0.0001527 | 0.0008982 | 0.0050299 | 0.0050299 |
| 287 | Seychelles | South Africa | 0.0000627 | 0.0011876 | 0.0000000 | 0.0000000 | 0.0010778 | 0.0046108 | 0.0050299 |
| 174 | Fiji | Sydney Harbour | 0.0001813 | 0.0011592 | 0.0001796 | 0.0001796 | 0.0008383 | 0.0055090 | 0.0056886 |
| 240 | Okinawa | Sydney Harbour | 0.0004663 | 0.0010447 | 0.0006587 | 0.0006722 | 0.0010180 | 0.0014371 | 0.0014371 |
| 242 | Okinawa | Towns River | 0.0006766 | 0.0010299 | 0.0008383 | 0.0008383 | 0.0010778 | 0.0011377 | 0.0011377 |
| 232 | Okinawa | Papua New Guinea | 0.0004255 | 0.0010283 | 0.0003593 | 0.0003593 | 0.0010180 | 0.0013772 | 0.0013772 |
| 60 | Blue mud Bay | Okinawa | 0.0006793 | 0.0010189 | 0.0007784 | 0.0007919 | 0.0010778 | 0.0011377 | 0.0011377 |
| 11 | Adelaide River | Okinawa | 0.0003657 | 0.0010020 | 0.0002994 | 0.0003593 | 0.0010778 | 0.0014970 | 0.0014970 |
| 234 | Okinawa | Roper River | 0.0006547 | 0.0009970 | 0.0007784 | 0.0007784 | 0.0010180 | 0.0011377 | 0.0011377 |
| 238 | Okinawa | South Alligator River | 0.0006018 | 0.0009934 | 0.0003593 | 0.0007784 | 0.0010778 | 0.0011976 | 0.0011976 |
| 245 | Okinawa | Victoria River | 0.0004822 | 0.0009917 | 0.0004790 | 0.0005988 | 0.0010778 | 0.0013832 | 0.0014371 |
| 217 | Indonesia | Papua New Guinea | 0.0004378 | 0.0009767 | 0.0002994 | 0.0004192 | 0.0010778 | 0.0011976 | 0.0012575 |
| 126 | Daly River | Okinawa | 0.0003747 | 0.0009629 | 0.0002994 | 0.0002994 | 0.0010180 | 0.0013174 | 0.0014371 |
| 246 | Okinawa | Wenlock River | 0.0003655 | 0.0009593 | 0.0003593 | 0.0004192 | 0.0010778 | 0.0011976 | 0.0011976 |
| 146 | East Alligator River | Okinawa | 0.0004091 | 0.0009510 | 0.0002994 | 0.0002994 | 0.0009581 | 0.0011976 | 0.0011976 |
| 83 | Clarence River | Okinawa | 0.0005038 | 0.0009421 | 0.0007186 | 0.0007186 | 0.0009581 | 0.0012575 | 0.0012575 |
| 79 | Clarence River | Fiji | 0.0000972 | 0.0009350 | 0.0001796 | 0.0001796 | 0.0002994 | 0.0053473 | 0.0055689 |
| 258 | Papua New Guinea | Urauchi River | 0.0005602 | 0.0009269 | 0.0003593 | 0.0003593 | 0.0010180 | 0.0012066 | 0.0013772 |
| 227 | Indonesia | Towns River | 0.0006367 | 0.0009261 | 0.0007784 | 0.0008219 | 0.0009581 | 0.0009581 | 0.0009581 |
| 59 | Blue mud Bay | Indonesia | 0.0006393 | 0.0009151 | 0.0007186 | 0.0007784 | 0.0009581 | 0.0009581 | 0.0009581 |
| 216 | Indonesia | Okinawa | 0.0003952 | 0.0009062 | 0.0004192 | 0.0004192 | 0.0010778 | 0.0012575 | 0.0012575 |
| 343 | Towns River | Urauchi River | 0.0007804 | 0.0008976 | 0.0008383 | 0.0008383 | 0.0008982 | 0.0011377 | 0.0011377 |
| 334 | Sydney Harbour | Urauchi River | 0.0005548 | 0.0008971 | 0.0006587 | 0.0006587 | 0.0008383 | 0.0011976 | 0.0014371 |
| 225 | Indonesia | Sydney Harbour | 0.0003822 | 0.0008967 | 0.0003593 | 0.0004790 | 0.0008982 | 0.0012575 | 0.0012575 |
| 219 | Indonesia | Roper River | 0.0006148 | 0.0008932 | 0.0007186 | 0.0007530 | 0.0008982 | 0.0009581 | 0.0009581 |
| 73 | Blue mud Bay | Urauchi River | 0.0007830 | 0.0008866 | 0.0007784 | 0.0007784 | 0.0008982 | 0.0011377 | 0.0011377 |
| 223 | Indonesia | South Alligator River | 0.0005495 | 0.0008772 | 0.0000599 | 0.0006198 | 0.0008982 | 0.0010180 | 0.0011377 |
| 283 | Roper River | Urauchi River | 0.0007585 | 0.0008647 | 0.0007784 | 0.0007784 | 0.0008982 | 0.0010734 | 0.0011377 |
| 243 | Okinawa | Trinity inlet | 0.0005749 | 0.0008623 | 0.0007186 | 0.0007186 | 0.0009581 | 0.0009581 | 0.0009581 |
| 321 | South Alligator River | Urauchi River | 0.0006951 | 0.0008506 | 0.0003593 | 0.0005808 | 0.0008383 | 0.0010778 | 0.0011976 |
| 260 | Papua New Guinea | Wenlock River | 0.0002140 | 0.0008358 | 0.0002395 | 0.0002994 | 0.0008383 | 0.0011377 | 0.0011976 |
| 230 | Indonesia | Victoria River | 0.0003831 | 0.0008288 | 0.0000599 | 0.0001796 | 0.0008982 | 0.0012575 | 0.0012575 |
| 349 | Urauchi River | Victoria River | 0.0005482 | 0.0008216 | 0.0004790 | 0.0004790 | 0.0008383 | 0.0011976 | 0.0014371 |
| 127 | Daly River | Papua New Guinea | 0.0001985 | 0.0008147 | 0.0001796 | 0.0002395 | 0.0007186 | 0.0012575 | 0.0014371 |
| 96 | Clarence River | Urauchi River | 0.0006076 | 0.0008098 | 0.0007186 | 0.0007186 | 0.0007784 | 0.0010180 | 0.0012575 |
| 10 | Adelaide River | Indonesia | 0.0002343 | 0.0008067 | 0.0000599 | 0.0001796 | 0.0008982 | 0.0013174 | 0.0013174 |
| 82 | Clarence River | Indonesia | 0.0004316 | 0.0008060 | 0.0005389 | 0.0005464 | 0.0008383 | 0.0010778 | 0.0010778 |
| 125 | Daly River | Indonesia | 0.0002812 | 0.0008056 | 0.0002395 | 0.0003593 | 0.0008383 | 0.0012575 | 0.0012575 |
| 336 | Sydney Harbour | Wenlock River | 0.0002061 | 0.0008034 | 0.0002395 | 0.0005479 | 0.0007784 | 0.0011377 | 0.0012575 |
| 135 | Daly River | Sydney Harbour | 0.0002082 | 0.0008000 | 0.0004192 | 0.0004251 | 0.0007784 | 0.0012575 | 0.0013772 |
| 145 | East Alligator River | Indonesia | 0.0003186 | 0.0007966 | 0.0001198 | 0.0002395 | 0.0008982 | 0.0010464 | 0.0011377 |
| 231 | Indonesia | Wenlock River | 0.0002663 | 0.0007963 | 0.0002994 | 0.0004087 | 0.0008982 | 0.0010284 | 0.0010778 |
| 24 | Adelaide River | Urauchi River | 0.0003959 | 0.0007962 | 0.0002994 | 0.0002994 | 0.0008383 | 0.0012575 | 0.0014970 |
| 84 | Clarence River | Papua New Guinea | 0.0003227 | 0.0007889 | 0.0001198 | 0.0002395 | 0.0008383 | 0.0010778 | 0.0012575 |
| 20 | Adelaide River | Sydney Harbour | 0.0001480 | 0.0007879 | 0.0001198 | 0.0004192 | 0.0007186 | 0.0012081 | 0.0014371 |
| 147 | East Alligator River | Papua New Guinea | 0.0002163 | 0.0007862 | 0.0001796 | 0.0002395 | 0.0007784 | 0.0011976 | 0.0012575 |
| 12 | Adelaide River | Papua New Guinea | 0.0001208 | 0.0007851 | 0.0001796 | 0.0002395 | 0.0007784 | 0.0012740 | 0.0014970 |

Table 55: Nucleotide divergence between each Location *(continued)*

| | strata.1 | strata.2 | dA | mean | q.0 | q.0.025 | q.0.5 | q.0.975 | q.1 |
|---|---|---|---|---|---|---|---|---|---|
| 177 | Fiji | Trinity inlet | 0.0000915 | 0.0007784 | 0.0001796 | 0.0001796 | 0.0002395 | 0.0052695 | 0.0052695 |
| 155 | East Alligator River | Sydney Harbour | 0.0002243 | 0.0007698 | 0.0003593 | 0.0004192 | 0.0007186 | 0.0011377 | 0.0013174 |
| 257 | Papua New Guinea | Trinity inlet | 0.0004511 | 0.0007665 | 0.0005988 | 0.0005988 | 0.0007784 | 0.0009581 | 0.0009581 |
| 159 | East Alligator River | Urauchi River | 0.0004507 | 0.0007565 | 0.0002994 | 0.0002994 | 0.0008383 | 0.0011377 | 0.0011976 |
| 139 | Daly River | Urauchi River | 0.0003991 | 0.0007512 | 0.0002994 | 0.0002994 | 0.0007784 | 0.0011976 | 0.0014371 |
| 259 | Papua New Guinea | Victoria River | 0.0002109 | 0.0007484 | 0.0001796 | 0.0002395 | 0.0006587 | 0.0011467 | 0.0014371 |
| 262 | Reunion | Seychelles | 0.0004528 | 0.0007473 | 0.0000000 | 0.0000000 | 0.0008982 | 0.0010180 | 0.0010778 |
| 3 | Adelaide River | Clarence River | 0.0002448 | 0.0007445 | 0.0000000 | 0.0001198 | 0.0007186 | 0.0011377 | 0.0013174 |
| 350 | Urauchi River | Wenlock River | 0.0003857 | 0.0007435 | 0.0003593 | 0.0003593 | 0.0008383 | 0.0011377 | 0.0011976 |
| 335 | Sydney Harbour | Victoria River | 0.0002281 | 0.0007412 | 0.0000599 | 0.0005269 | 0.0006587 | 0.0011377 | 0.0013772 |
| 332 | Sydney Harbour | Towns River | 0.0003802 | 0.0007370 | 0.0005988 | 0.0005988 | 0.0007186 | 0.0010778 | 0.0010778 |
| 256 | Papua New Guinea | Towns River | 0.0003533 | 0.0007345 | 0.0002395 | 0.0002994 | 0.0007186 | 0.0011377 | 0.0011377 |
| 346 | Trinity inlet | Urauchi River | 0.0006786 | 0.0007300 | 0.0007186 | 0.0007186 | 0.0007186 | 0.0009581 | 0.0009581 |
| 69 | Blue mud Bay | Sydney Harbour | 0.0003846 | 0.0007278 | 0.0005389 | 0.0005988 | 0.0007186 | 0.0010778 | 0.0010778 |
| 77 | Clarence River | Daly River | 0.0002697 | 0.0007213 | 0.0000000 | 0.0001796 | 0.0007186 | 0.0010778 | 0.0012575 |
| 61 | Blue mud Bay | Papua New Guinea | 0.0003534 | 0.0007210 | 0.0001796 | 0.0002994 | 0.0007186 | 0.0011377 | 0.0011377 |
| 317 | South Alligator River | Sydney Harbour | 0.0003244 | 0.0007195 | 0.0002994 | 0.0005389 | 0.0006587 | 0.0010778 | 0.0013174 |
| 23 | Adelaide River | Trinity inlet | 0.0003697 | 0.0007186 | 0.0004192 | 0.0004192 | 0.0006587 | 0.0010180 | 0.0010180 |
| 228 | Indonesia | Trinity inlet | 0.0004950 | 0.0007186 | 0.0005389 | 0.0005389 | 0.0007784 | 0.0007784 | 0.0007784 |
| 252 | Papua New Guinea | South Alligator River | 0.0002978 | 0.0007173 | 0.0001796 | 0.0002395 | 0.0007186 | 0.0011377 | 0.0012575 |
| 279 | Roper River | Sydney Harbour | 0.0003600 | 0.0007059 | 0.0005389 | 0.0005389 | 0.0006587 | 0.0010614 | 0.0010778 |
| 248 | Papua New Guinea | Roper River | 0.0003313 | 0.0007016 | 0.0001796 | 0.0002680 | 0.0007186 | 0.0011093 | 0.0011377 |
| 98 | Clarence River | Wenlock River | 0.0002388 | 0.0006961 | 0.0000000 | 0.0001198 | 0.0007186 | 0.0010180 | 0.0010778 |
| 138 | Daly River | Trinity inlet | 0.0003890 | 0.0006898 | 0.0004192 | 0.0004192 | 0.0006587 | 0.0009581 | 0.0009581 |
| 254 | Papua New Guinea | Sydney Harbour | 0.0000803 | 0.0006866 | 0.0001198 | 0.0001198 | 0.0007784 | 0.0012575 | 0.0014371 |
| 78 | Clarence River | East Alligator River | 0.0002733 | 0.0006786 | 0.0000000 | 0.0001796 | 0.0007186 | 0.0010180 | 0.0011377 |
| 26 | Adelaide River | Wenlock River | 0.0000118 | 0.0006671 | 0.0000000 | 0.0000000 | 0.0007784 | 0.0012575 | 0.0013174 |
| 97 | Clarence River | Victoria River | 0.0002936 | 0.0006666 | 0.0000000 | 0.0001198 | 0.0006587 | 0.0010180 | 0.0012575 |
| 5 | Adelaide River | Daly River | 0.0000156 | 0.0006653 | 0.0000000 | 0.0000000 | 0.0007186 | 0.0013772 | 0.0014371 |
| 348 | Trinity inlet | Wenlock River | 0.0003554 | 0.0006618 | 0.0002395 | 0.0002395 | 0.0006587 | 0.0007784 | 0.0007784 |
| 347 | Trinity inlet | Victoria River | 0.0004366 | 0.0006587 | 0.0005389 | 0.0005389 | 0.0005988 | 0.0009581 | 0.0009581 |
| 158 | East Alligator River | Trinity inlet | 0.0004007 | 0.0006552 | 0.0004192 | 0.0004192 | 0.0006587 | 0.0008383 | 0.0008383 |
| 342 | Towns River | Trinity inlet | 0.0005808 | 0.0006467 | 0.0005988 | 0.0005988 | 0.0006587 | 0.0006587 | 0.0006587 |
| 244 | Okinawa | Urauchi River | 0.0002977 | 0.0006364 | 0.0000000 | 0.0000000 | 0.0010778 | 0.0010778 | 0.0010778 |
| 72 | Blue mud Bay | Trinity inlet | 0.0005834 | 0.0006357 | 0.0005389 | 0.0005389 | 0.0006587 | 0.0006587 | 0.0006587 |
| 94 | Clarence River | Towns River | 0.0004186 | 0.0006353 | 0.0000000 | 0.0001198 | 0.0007186 | 0.0009222 | 0.0009581 |
| 141 | Daly River | Wenlock River | 0.0000181 | 0.0006253 | 0.0000000 | 0.0000599 | 0.0006587 | 0.0011377 | 0.0012575 |
| 320 | South Alligator River | Trinity inlet | 0.0005198 | 0.0006240 | 0.0005389 | 0.0005389 | 0.0005988 | 0.0007455 | 0.0008383 |
| 52 | Blue mud Bay | Clarence River | 0.0004190 | 0.0006221 | 0.0000000 | 0.0000599 | 0.0006587 | 0.0009581 | 0.0009581 |
| 6 | Adelaide River | East Alligator River | 0.0000179 | 0.0006213 | 0.0000000 | 0.0000000 | 0.0006587 | 0.0012799 | 0.0013772 |
| 90 | Clarence River | South Alligator River | 0.0003648 | 0.0006198 | 0.0000000 | 0.0001198 | 0.0006587 | 0.0008982 | 0.0011377 |
| 282 | Roper River | Trinity inlet | 0.0005589 | 0.0006138 | 0.0005389 | 0.0005389 | 0.0006287 | 0.0006587 | 0.0006587 |
| 86 | Clarence River | Roper River | 0.0003995 | 0.0006052 | 0.0000000 | 0.0000644 | 0.0006587 | 0.0008937 | 0.0009581 |
| 25 | Adelaide River | Victoria River | 0.0000216 | 0.0005926 | 0.0000000 | 0.0000000 | 0.0007186 | 0.0013174 | 0.0013772 |
| 161 | East Alligator River | Wenlock River | 0.0000260 | 0.0005869 | 0.0000000 | 0.0000629 | 0.0005988 | 0.0010180 | 0.0010778 |
| 229 | Indonesia | Urauchi River | 0.0003116 | 0.0005864 | 0.0004192 | 0.0004192 | 0.0005389 | 0.0011377 | 0.0012575 |
| 351 | Victoria River | Wenlock River | 0.0000386 | 0.0005671 | 0.0000000 | 0.0000599 | 0.0007186 | 0.0010778 | 0.0012575 |
| 22 | Adelaide River | Towns River | 0.0001521 | 0.0005669 | 0.0000000 | 0.0000000 | 0.0006587 | 0.0010778 | 0.0010778 |
| 140 | Daly River | Victoria River | 0.0000365 | 0.0005595 | 0.0000000 | 0.0000000 | 0.0005988 | 0.0011976 | 0.0013772 |
| 121 | Daly River | East Alligator River | 0.0000035 | 0.0005588 | 0.0000000 | 0.0000000 | 0.0005389 | 0.0011377 | 0.0013174 |
| 18 | Adelaide River | South Alligator River | 0.0000964 | 0.0005495 | 0.0000000 | 0.0000000 | 0.0006587 | 0.0010778 | 0.0013772 |
| 2 | Adelaide River | Blue mud Bay | 0.0001450 | 0.0005461 | 0.0000000 | 0.0000599 | 0.0006287 | 0.0010778 | 0.0010778 |
| 14 | Adelaide River | Roper River | 0.0001351 | 0.0005389 | 0.0000000 | 0.0000000 | 0.0006287 | 0.0010314 | 0.0010778 |
| 137 | Daly River | Towns River | 0.0001679 | 0.0005346 | 0.0000000 | 0.0000599 | 0.0004192 | 0.0010778 | 0.0010778 |
| 92 | Clarence River | Sydney Harbour | 0.0000899 | 0.0005317 | 0.0000000 | 0.0000000 | 0.0006587 | 0.0010778 | 0.0011976 |
| 54 | Blue mud Bay | Daly River | 0.0001692 | 0.0005222 | 0.0000000 | 0.0000599 | 0.0004192 | 0.0010778 | 0.0010778 |
| 345 | Towns River | Wenlock River | 0.0001484 | 0.0005206 | 0.0000000 | 0.0000599 | 0.0002994 | 0.0009581 | 0.0009581 |
| 323 | South Alligator River | Wenlock River | 0.0000982 | 0.0005087 | 0.0000000 | 0.0000000 | 0.0003593 | 0.0009581 | 0.0010778 |
| 129 | Daly River | Roper River | 0.0001467 | 0.0005024 | 0.0000000 | 0.0000000 | 0.0003892 | 0.0010778 | 0.0010778 |
| 133 | Daly River | South Alligator River | 0.0000915 | 0.0004964 | 0.0000000 | 0.0000000 | 0.0004192 | 0.0010778 | 0.0013174 |
| 285 | Roper River | Wenlock River | 0.0001343 | 0.0004956 | 0.0000000 | 0.0000524 | 0.0002994 | 0.0009581 | 0.0009581 |
| 75 | Blue mud Bay | Wenlock River | 0.0001326 | 0.0004912 | 0.0000000 | 0.0000000 | 0.0002994 | 0.0009581 | 0.0009581 |
| 160 | East Alligator River | Victoria River | 0.0000142 | 0.0004908 | 0.0000000 | 0.0000599 | 0.0002994 | 0.0010778 | 0.0013174 |
| 333 | Sydney Harbour | Trinity inlet | 0.0001604 | 0.0004514 | 0.0000000 | 0.0000000 | 0.0005988 | 0.0009581 | 0.0009581 |
| 157 | East Alligator River | Towns River | 0.0001122 | 0.0004325 | 0.0000000 | 0.0000659 | 0.0002994 | 0.0010180 | 0.0010180 |
| 55 | Blue mud Bay | East Alligator River | 0.0001127 | 0.0004194 | 0.0000000 | 0.0000599 | 0.0002994 | 0.0010180 | 0.0010180 |
| 153 | East Alligator River | South Alligator River | 0.0000469 | 0.0004055 | 0.0000000 | 0.0000000 | 0.0002395 | 0.0010180 | 0.0010778 |
| 149 | East Alligator River | Roper River | 0.0000913 | 0.0004007 | 0.0000000 | 0.0000404 | 0.0002395 | 0.0010180 | 0.0010180 |
| 344 | Towns River | Victoria River | 0.0000559 | 0.0003439 | 0.0000000 | 0.0000000 | 0.0001796 | 0.0010180 | 0.0010180 |
| 322 | South Alligator River | Victoria River | 0.0000103 | 0.0003366 | 0.0000000 | 0.0000000 | 0.0001198 | 0.0010180 | 0.0013174 |
| 74 | Blue mud Bay | Victoria River | 0.0000536 | 0.0003279 | 0.0000000 | 0.0000599 | 0.0001796 | 0.0010180 | 0.0010180 |
| 284 | Roper River | Victoria River | 0.0000388 | 0.0003158 | 0.0000000 | 0.0000000 | 0.0001796 | 0.0010180 | 0.0010180 |
| 103 | Costa Rica | Gulf of California | 0.0001728 | 0.0003074 | 0.0002395 | 0.0002395 | 0.0002994 | 0.0004192 | 0.0004192 |
| 319 | South Alligator River | Towns River | 0.0000417 | 0.0002118 | 0.0000000 | 0.0000000 | 0.0001796 | 0.0008278 | 0.0010180 |
| 67 | Blue mud Bay | South Alligator River | 0.0000342 | 0.0001906 | 0.0000000 | 0.0000000 | 0.0001796 | 0.0008593 | 0.0010180 |
| 277 | Roper River | South Alligator River | 0.0000245 | 0.0001836 | 0.0000000 | 0.0000000 | 0.0001796 | 0.0008054 | 0.0010180 |
| 71 | Blue mud Bay | Towns River | 0.0000533 | 0.0001713 | 0.0000000 | 0.0000599 | 0.0001796 | 0.0002395 | 0.0002395 |
| 95 | Clarence River | Trinity inlet | 0.0000202 | 0.0001711 | 0.0000000 | 0.0000000 | 0.0000599 | 0.0007784 | 0.0007784 |
| 63 | Blue mud Bay | Roper River | 0.0000438 | 0.0001509 | 0.0000000 | 0.0000165 | 0.0001198 | 0.0002395 | 0.0002395 |
| 281 | Roper River | Towns River | -0.0000220 | 0.0000988 | 0.0000000 | 0.0000000 | 0.0001198 | 0.0002395 | 0.0002395 |

Table 53: Nucleotide and haplotype diversity per Location

| | Location | pi.strataG | pi.pegas | pi.pegas.var | HT.richness.strataG | h.pegas | h.pegas.var |
|---|---|---|---|---|---|---|---|
| 1 | Gulf of California | NaN | NaN | NaN | 1 | NaN | NaN |
| 11 | Costa Rica | 0.000269 | 0.000269 | 0e+00 | 9 | 0.914286 | 0.002208 |
| 12 | Sierra Leone | NaN | NaN | NaN | 1 | NaN | NaN |
| 13 | South Africa | NaN | 0.001950 | 1e-06 | 6 | 0.952381 | 0.005950 |
| 14 | Reunion | 0.000289 | 0.000289 | 0e+00 | 6 | 0.808333 | 0.004289 |
| 15 | Seychelles | 0.000299 | 0.000299 | 0e+00 | 3 | 0.660131 | 0.002867 |
| 16 | Arabian Sea | 0.001792 | 0.001792 | 1e-06 | 13 | 0.941520 | 0.001170 |
| 17 | Sri Lanka | 0.003036 | 0.003037 | 3e-06 | 11 | 0.984848 | 0.000986 |
| 18 | Thailand | NaN | 0.002925 | 3e-06 | 4 | 0.800000 | 0.025926 |
| 19 | Indonesia | 0.000447 | 0.000447 | 0e+00 | 6 | 1.000000 | 0.004630 |
| 110 | Papua New Guinea | 0.000630 | 0.000631 | 0e+00 | 13 | 0.980952 | 0.000600 |
| 111 | Fitzroy River | 0.003148 | 0.003149 | 4e-06 | 4 | 0.900000 | 0.017760 |
| 112 | Victoria River | 0.000444 | 0.000444 | 0e+00 | 14 | 0.933333 | 0.001401 |
| 113 | Daly River | 0.000601 | 0.000601 | 0e+00 | 16 | 0.936667 | 0.001178 |
| 114 | Adelaide River | 0.000697 | 0.000698 | 0e+00 | 12 | 0.921569 | 0.002350 |
| 115 | South Alligator River | 0.000208 | 0.000208 | 0e+00 | 18 | 0.934566 | 0.000394 |
| 116 | East Alligator River | 0.000509 | 0.000509 | 0e+00 | 12 | 0.941176 | 0.001566 |
| 117 | Blue mud Bay | 0.000104 | 0.000104 | 0e+00 | 7 | 0.871795 | 0.003701 |
| 118 | Roper River | 0.000110 | 0.000110 | 0e+00 | 3 | 0.833333 | 0.035156 |
| 119 | Towns River | NaN | 0.000132 | 0e+00 | 3 | 0.700000 | 0.043040 |
| 120 | Wenlock River | 0.000612 | 0.000613 | 0e+00 | 14 | 0.964912 | 0.000549 |
| 121 | Trinity inlet | 0.000000 | 0.000000 | 0e+00 | 2 | 0.500000 | 0.066406 |
| 122 | Clarence River | 0.000302 | 0.000302 | 0e+00 | 12 | 0.933333 | 0.000784 |
| 123 | Sydney Harbour | 0.000582 | 0.000582 | 0e+00 | 11 | 0.974359 | 0.000968 |
| 124 | Okinawa | 0.000575 | 0.000575 | 0e+00 | 3 | 0.711111 | 0.005120 |
| 125 | Urauchi River | 0.000103 | 0.000103 | 0e+00 | 3 | 0.528571 | 0.005884 |
| 126 | Fiji | NaN | 0.001374 | 1e-06 | 8 | 0.972222 | 0.002578 |

Table 54: Nucleotide divergence within each Location

| | stratum | mean | q.0 | q.0.025 | q.0.5 | q.0.975 | q.1 |
|---|---|---|---|---|---|---|---|
| 9 | Fitzroy River | 0.003150 | 0 | 0.0e+00 | 0.005210 | 0.005269 | 0.005269 |
| 20 | Sri Lanka | 0.003038 | 0 | 3.7e-05 | 0.005210 | 0.005569 | 0.005629 |
| 22 | Thailand | 0.002926 | 0 | 0.0e+00 | 0.005269 | 0.005428 | 0.005449 |
| 18 | South Africa | 0.001950 | 0 | 6.0e-05 | 0.001138 | 0.005150 | 0.005150 |
| 2 | Arabian Sea | 0.001793 | 0 | 0.0e+00 | 0.000240 | 0.006048 | 0.006108 |
| 8 | Fiji | 0.001374 | 0 | 0.0e+00 | 0.000240 | 0.005509 | 0.005509 |
| 1 | Adelaide River | 0.000698 | 0 | 0.0e+00 | 0.000778 | 0.001377 | 0.001437 |
| 13 | Papua New Guinea | 0.000631 | 0 | 0.0e+00 | 0.000778 | 0.001257 | 0.001377 |
| 27 | Wenlock River | 0.000613 | 0 | 0.0e+00 | 0.000838 | 0.001018 | 0.001018 |
| 6 | Daly River | 0.000602 | 0 | 0.0e+00 | 0.000599 | 0.001257 | 0.001377 |
| 21 | Sydney Harbour | 0.000582 | 0 | 0.0e+00 | 0.000778 | 0.001082 | 0.001257 |
| 12 | Okinawa | 0.000575 | 0 | 0.0e+00 | 0.001078 | 0.001078 | 0.001078 |
| 7 | East Alligator River | 0.000509 | 0 | 0.0e+00 | 0.000449 | 0.001078 | 0.001078 |
| 11 | Indonesia | 0.000447 | 0 | 0.0e+00 | 0.000240 | 0.001078 | 0.001078 |
| 26 | Victoria River | 0.000444 | 0 | 0.0e+00 | 0.000180 | 0.001018 | 0.001317 |
| 4 | Clarence River | 0.000302 | 0 | 0.0e+00 | 0.000120 | 0.000838 | 0.001078 |
| 16 | Seychelles | 0.000299 | 0 | 0.0e+00 | 0.000000 | 0.001018 | 0.001018 |
| 14 | Reunion | 0.000289 | 0 | 0.0e+00 | 0.000120 | 0.001078 | 0.001078 |
| 5 | Costa Rica | 0.000269 | 0 | 0.0e+00 | 0.000299 | 0.000419 | 0.000419 |
| 19 | South Alligator River | 0.000208 | 0 | 0.0e+00 | 0.000180 | 0.000958 | 0.001078 |
| 23 | Towns River | 0.000132 | 0 | 0.0e+00 | 0.000180 | 0.000240 | 0.000240 |
| 15 | Roper River | 0.000110 | 0 | 7.0e-06 | 0.000120 | 0.000180 | 0.000180 |
| 3 | Blue mud Bay | 0.000104 | 0 | 0.0e+00 | 0.000120 | 0.000240 | 0.000240 |
| 25 | Urauchi River | 0.000103 | 0 | 0.0e+00 | 0.000000 | 0.001078 | 0.001078 |
| 24 | Trinity inlet | 0.000000 | 0 | 0.0e+00 | 0.000000 | 0.000000 | 0.000000 |
| 10 | Gulf of California | NA | NA | NA | NA | NA | NA |
| 17 | Sierra Leone | NA | NA | NA | NA | NA | NA |

Table 56: Haplotype counts per Location according to pegas

| | GOC | COR | SIL | SAF | RUN | SEY | ARS | SRL | TAI | IND | PNG | FZR | VIR | DAR | ADR | SAR | EAR | BMB | ROR | TOR | WER | TRI | CLR | SYH | URR | OKI | FIJ |
|---|---|---|---|---|---|---|---|---|---|---|---|---|---|---|---|---|---|---|---|---|---|---|---|---|---|---|---|
| HT01 | 0 | 0 | 0 | 1 | 0 | 0 | 0 | 0 | 0 | 0 | 0 | 0 | 0 | 0 | 0 | 0 | 0 | 0 | 0 | 0 | 0 | 0 | 0 | 0 | 0 | 0 | 0 |
| HT02 | 0 | 0 | 0 | 1 | 0 | 0 | 0 | 0 | 0 | 0 | 0 | 0 | 0 | 0 | 0 | 0 | 0 | 0 | 0 | 0 | 0 | 0 | 0 | 0 | 0 | 0 | 0 |
| HT03 | 0 | 0 | 0 | 1 | 0 | 0 | 0 | 0 | 0 | 0 | 0 | 0 | 0 | 0 | 0 | 0 | 0 | 0 | 0 | 0 | 0 | 0 | 0 | 0 | 0 | 0 | 0 |
| HT04 | 0 | 0 | 0 | 0 | 1 | 0 | 0 | 0 | 0 | 0 | 0 | 0 | 0 | 0 | 0 | 0 | 0 | 0 | 0 | 0 | 0 | 0 | 0 | 0 | 0 | 0 | 0 |
| HT05 | 0 | 0 | 0 | 0 | 0 | 0 | 0 | 0 | 3 | 0 | 0 | 0 | 0 | 0 | 0 | 0 | 0 | 0 | 0 | 0 | 0 | 0 | 0 | 0 | 0 | 0 | 0 |
| HT06 | 0 | 0 | 0 | 0 | 0 | 0 | 0 | 2 | 0 | 0 | 0 | 0 | 0 | 0 | 0 | 0 | 0 | 0 | 0 | 0 | 0 | 0 | 0 | 0 | 0 | 0 | 0 |
| HT07 | 0 | 0 | 0 | 0 | 0 | 0 | 0 | 1 | 0 | 0 | 0 | 0 | 0 | 0 | 0 | 0 | 0 | 0 | 0 | 0 | 0 | 0 | 0 | 0 | 0 | 0 | 0 |
| HT08 | 0 | 0 | 0 | 0 | 1 | 0 | 0 | 0 | 0 | 0 | 0 | 0 | 0 | 0 | 0 | 0 | 0 | 0 | 0 | 0 | 0 | 0 | 0 | 0 | 0 | 0 | 0 |
| HT09 | 0 | 0 | 0 | 1 | 0 | 0 | 0 | 0 | 0 | 0 | 0 | 0 | 0 | 0 | 0 | 0 | 0 | 0 | 0 | 0 | 0 | 0 | 0 | 0 | 0 | 0 | 0 |
| HT10 | 0 | 0 | 0 | 2 | 0 | 8 | 0 | 0 | 0 | 0 | 0 | 0 | 0 | 0 | 0 | 0 | 0 | 0 | 0 | 0 | 0 | 0 | 0 | 0 | 0 | 0 | 0 |
| HT11 | 0 | 0 | 0 | 0 | 0 | 7 | 0 | 0 | 0 | 0 | 0 | 0 | 0 | 0 | 0 | 0 | 0 | 0 | 0 | 0 | 0 | 0 | 0 | 0 | 0 | 0 | 0 |
| HT12 | 0 | 0 | 0 | 0 | 0 | 0 | 0 | 0 | 0 | 0 | 0 | 2 | 0 | 0 | 0 | 0 | 0 | 0 | 0 | 0 | 0 | 0 | 0 | 0 | 0 | 0 | 0 |
| HT13 | 0 | 0 | 0 | 0 | 0 | 0 | 0 | 1 | 0 | 0 | 0 | 0 | 0 | 0 | 0 | 0 | 0 | 0 | 0 | 0 | 0 | 0 | 0 | 0 | 0 | 0 | 0 |
| HT14 | 0 | 0 | 0 | 0 | 0 | 0 | 0 | 1 | 0 | 0 | 0 | 0 | 0 | 0 | 0 | 0 | 0 | 0 | 0 | 0 | 0 | 0 | 0 | 0 | 0 | 0 | 0 |
| HT15 | 0 | 0 | 0 | 0 | 6 | 0 | 0 | 0 | 0 | 0 | 0 | 0 | 0 | 0 | 0 | 0 | 0 | 0 | 0 | 0 | 0 | 0 | 0 | 0 | 0 | 0 | 0 |
| HT16 | 0 | 0 | 0 | 0 | 2 | 3 | 0 | 0 | 0 | 0 | 0 | 0 | 0 | 0 | 0 | 0 | 0 | 0 | 0 | 0 | 0 | 0 | 0 | 0 | 0 | 0 | 0 |
| HT17 | 0 | 0 | 0 | 0 | 2 | 0 | 0 | 0 | 0 | 0 | 0 | 0 | 0 | 0 | 0 | 0 | 0 | 0 | 0 | 0 | 0 | 0 | 0 | 0 | 0 | 0 | 0 |
| HT18 | 0 | 0 | 0 | 0 | 4 | 0 | 0 | 0 | 0 | 0 | 0 | 0 | 0 | 0 | 0 | 0 | 0 | 0 | 0 | 0 | 0 | 0 | 0 | 0 | 0 | 0 | 0 |
| HT19 | 0 | 0 | 0 | 0 | 0 | 0 | 0 | 0 | 0 | 0 | 0 | 0 | 0 | 0 | 0 | 0 | 0 | 0 | 0 | 0 | 0 | 0 | 0 | 0 | 0 | 0 | 1 |
| HT20 | 0 | 0 | 0 | 0 | 0 | 0 | 0 | 1 | 0 | 0 | 0 | 0 | 0 | 0 | 0 | 0 | 0 | 0 | 0 | 0 | 0 | 0 | 0 | 0 | 0 | 0 | 0 |
| HT21 | 0 | 0 | 0 | 0 | 0 | 0 | 0 | 1 | 1 | 0 | 0 | 0 | 0 | 0 | 0 | 0 | 0 | 0 | 0 | 0 | 0 | 0 | 0 | 0 | 0 | 0 | 0 |
| HT22 | 0 | 0 | 0 | 0 | 0 | 0 | 3 | 0 | 0 | 0 | 0 | 0 | 0 | 0 | 0 | 0 | 0 | 0 | 0 | 0 | 0 | 0 | 0 | 0 | 0 | 0 | 0 |
| HT23 | 0 | 0 | 0 | 0 | 0 | 0 | 0 | 0 | 0 | 0 | 0 | 0 | 0 | 0 | 0 | 0 | 0 | 0 | 0 | 0 | 0 | 1 | 0 | 2 | 0 | 0 | 0 |
| HT24 | 0 | 0 | 0 | 0 | 0 | 0 | 0 | 0 | 0 | 0 | 0 | 0 | 0 | 0 | 0 | 0 | 0 | 0 | 0 | 0 | 0 | 3 | 0 | 1 | 0 | 0 | 0 |
| HT25 | 0 | 0 | 0 | 0 | 0 | 0 | 0 | 0 | 0 | 0 | 0 | 0 | 0 | 1 | 0 | 0 | 0 | 0 | 0 | 0 | 0 | 0 | 0 | 0 | 0 | 0 | 0 |
| HT26 | 0 | 0 | 0 | 0 | 0 | 0 | 0 | 0 | 0 | 0 | 0 | 0 | 0 | 0 | 0 | 0 | 0 | 0 | 0 | 0 | 0 | 0 | 0 | 1 | 0 | 0 | 0 |
| HT27 | 0 | 0 | 0 | 0 | 0 | 0 | 0 | 0 | 0 | 0 | 0 | 0 | 0 | 0 | 0 | 0 | 0 | 0 | 0 | 0 | 1 | 0 | 0 | 0 | 0 | 0 | 0 |
| HT28 | 0 | 0 | 0 | 0 | 0 | 0 | 0 | 0 | 0 | 1 | 0 | 0 | 0 | 0 | 0 | 0 | 0 | 0 | 0 | 0 | 0 | 0 | 0 | 0 | 0 | 0 | 0 |
| HT29 | 0 | 0 | 0 | 0 | 0 | 0 | 0 | 0 | 0 | 0 | 0 | 0 | 0 | 0 | 0 | 2 | 0 | 0 | 0 | 0 | 0 | 0 | 0 | 0 | 0 | 0 | 0 |
| HT30 | 0 | 0 | 0 | 0 | 0 | 0 | 0 | 0 | 0 | 0 | 0 | 0 | 0 | 0 | 0 | 1 | 4 | 0 | 0 | 0 | 0 | 0 | 0 | 0 | 0 | 0 | 0 |
| HT31 | 0 | 0 | 0 | 0 | 0 | 0 | 0 | 0 | 0 | 0 | 0 | 0 | 0 | 0 | 2 | 1 | 0 | 0 | 0 | 0 | 0 | 0 | 0 | 0 | 0 | 0 | 0 |
| HT32 | 0 | 0 | 0 | 0 | 0 | 0 | 0 | 0 | 0 | 0 | 1 | 0 | 0 | 0 | 0 | 0 | 0 | 0 | 0 | 0 | 0 | 0 | 0 | 0 | 0 | 0 | 0 |
| HT33 | 0 | 0 | 0 | 0 | 0 | 0 | 0 | 0 | 0 | 0 | 0 | 0 | 1 | 0 | 0 | 0 | 0 | 0 | 0 | 0 | 0 | 0 | 0 | 0 | 0 | 0 | 0 |
| HT34 | 0 | 0 | 0 | 0 | 0 | 0 | 0 | 0 | 0 | 0 | 0 | 1 | 5 | 0 | 0 | 1 | 0 | 0 | 0 | 0 | 0 | 0 | 0 | 0 | 0 | 0 | 0 |
| HT35 | 0 | 0 | 0 | 0 | 0 | 0 | 0 | 0 | 0 | 0 | 0 | 0 | 1 | 0 | 0 | 0 | 0 | 0 | 0 | 0 | 0 | 0 | 0 | 0 | 0 | 0 | 0 |
| HT36 | 0 | 0 | 0 | 0 | 0 | 0 | 0 | 0 | 0 | 0 | 0 | 0 | 0 | 0 | 0 | 0 | 0 | 0 | 0 | 3 | 0 | 0 | 0 | 0 | 0 | 0 | 0 |
| HT37 | 0 | 0 | 0 | 0 | 0 | 0 | 0 | 0 | 0 | 0 | 0 | 0 | 0 | 0 | 0 | 0 | 0 | 0 | 2 | 0 | 0 | 0 | 0 | 0 | 0 | 0 | 0 |
| HT38 | 0 | 0 | 0 | 0 | 0 | 0 | 0 | 0 | 0 | 0 | 0 | 0 | 0 | 0 | 0 | 1 | 1 | 0 | 0 | 0 | 0 | 0 | 0 | 0 | 0 | 0 | 0 |
| HT39 | 0 | 0 | 0 | 0 | 0 | 0 | 0 | 0 | 0 | 0 | 0 | 0 | 0 | 0 | 0 | 2 | 0 | 0 | 0 | 0 | 0 | 0 | 0 | 0 | 0 | 0 | 0 |
| HT40 | 0 | 0 | 0 | 0 | 0 | 0 | 0 | 0 | 0 | 0 | 0 | 0 | 1 | 0 | 0 | 0 | 1 | 0 | 0 | 0 | 0 | 0 | 0 | 0 | 0 | 0 | 0 |
| HT41 | 0 | 0 | 0 | 0 | 0 | 0 | 0 | 0 | 0 | 0 | 0 | 0 | 0 | 0 | 0 | 1 | 0 | 0 | 0 | 0 | 0 | 0 | 0 | 0 | 0 | 0 | 0 |
| HT42 | 0 | 0 | 0 | 0 | 0 | 0 | 0 | 0 | 0 | 0 | 0 | 0 | 0 | 0 | 0 | 1 | 0 | 0 | 0 | 0 | 0 | 0 | 0 | 0 | 0 | 0 | 0 |
| HT43 | 0 | 0 | 0 | 0 | 0 | 0 | 0 | 0 | 0 | 0 | 0 | 0 | 0 | 0 | 0 | 1 | 0 | 0 | 0 | 0 | 0 | 0 | 0 | 0 | 0 | 0 | 0 |
| HT44 | 0 | 0 | 0 | 0 | 0 | 0 | 0 | 0 | 0 | 0 | 0 | 1 | 0 | 2 | 0 | 0 | 0 | 1 | 1 | 0 | 0 | 0 | 0 | 0 | 0 | 0 | 0 |
| HT45 | 0 | 0 | 0 | 0 | 0 | 0 | 0 | 0 | 0 | 0 | 0 | 0 | 0 | 0 | 0 | 2 | 0 | 2 | 0 | 0 | 0 | 0 | 0 | 0 | 0 | 0 | 0 |
| HT46 | 0 | 0 | 0 | 0 | 0 | 0 | 0 | 0 | 0 | 0 | 0 | 1 | 2 | 2 | 0 | 3 | 1 | 0 | 0 | 0 | 0 | 0 | 0 | 0 | 0 | 0 | 0 |
| HT47 | 0 | 0 | 0 | 0 | 0 | 0 | 0 | 0 | 0 | 0 | 0 | 0 | 1 | 0 | 0 | 0 | 0 | 0 | 0 | 0 | 0 | 0 | 0 | 0 | 0 | 0 | 0 |
| HT48 | 0 | 0 | 0 | 0 | 0 | 0 | 0 | 0 | 0 | 0 | 0 | 0 | 0 | 0 | 1 | 0 | 0 | 0 | 0 | 0 | 0 | 0 | 0 | 0 | 0 | 0 | 0 |
| HT49 | 0 | 0 | 0 | 0 | 0 | 0 | 0 | 0 | 0 | 0 | 0 | 0 | 0 | 0 | 0 | 1 | 0 | 0 | 0 | 0 | 0 | 0 | 0 | 0 | 0 | 0 | 0 |
| HT50 | 0 | 0 | 0 | 0 | 0 | 0 | 0 | 0 | 0 | 0 | 0 | 0 | 0 | 6 | 0 | 4 | 2 | 0 | 0 | 0 | 0 | 0 | 0 | 0 | 0 | 0 | 0 |
| HT51 | 0 | 0 | 0 | 0 | 0 | 0 | 0 | 0 | 0 | 0 | 0 | 0 | 0 | 1 | 0 | 5 | 1 | 0 | 0 | 0 | 0 | 0 | 0 | 0 | 0 | 0 | 0 |
| HT52 | 0 | 0 | 0 | 0 | 0 | 0 | 0 | 0 | 0 | 0 | 0 | 0 | 0 | 1 | 0 | 0 | 0 | 0 | 0 | 0 | 0 | 0 | 0 | 0 | 0 | 0 | 0 |
| HT53 | 0 | 0 | 0 | 0 | 0 | 0 | 0 | 0 | 0 | 0 | 0 | 0 | 0 | 0 | 0 | 0 | 0 | 1 | 0 | 0 | 0 | 0 | 0 | 0 | 0 | 0 | 0 |

Table 56: Haplotype counts per Location according to pegas *(continued)*

| | GOC | COR | SIL | SAF | RUN | SEY | ARS | SRL | TAI | IND | PNG | FZR | VIR | DAR | ADR | SAR | EAR | BMB | ROR | TOR | WER | TRI | CLR | SYH | URR | OKI | FIJ |
|---|---|---|---|---|---|---|---|---|---|---|---|---|---|---|---|---|---|---|---|---|---|---|---|---|---|---|---|
| HT54 | 0 | 0 | 0 | 0 | 0 | 0 | 0 | 0 | 0 | 0 | 0 | 0 | 0 | 0 | 0 | 0 | 0 | 0 | 0 | 0 | 1 | 0 | 0 | 0 | 0 | 0 | 0 |
| HT55 | 0 | 0 | 0 | 0 | 0 | 0 | 0 | 0 | 0 | 0 | 0 | 0 | 3 | 1 | 5 | 7 | 1 | 0 | 1 | 1 | 0 | 0 | 0 | 0 | 0 | 0 | 0 |
| HT56 | 0 | 0 | 0 | 0 | 0 | 0 | 0 | 0 | 0 | 0 | 0 | 0 | 1 | 0 | 0 | 3 | 0 | 1 | 0 | 0 | 2 | 0 | 1 | 0 | 0 | 0 | 0 |
| HT57 | 0 | 0 | 0 | 0 | 0 | 0 | 0 | 0 | 0 | 0 | 0 | 0 | 0 | 0 | 0 | 0 | 0 | 0 | 0 | 0 | 1 | 0 | 0 | 0 | 0 | 0 | 0 |
| HT58 | 0 | 0 | 0 | 0 | 0 | 0 | 0 | 0 | 0 | 0 | 0 | 0 | 0 | 0 | 0 | 0 | 0 | 0 | 0 | 0 | 1 | 0 | 0 | 0 | 0 | 0 | 0 |
| HT59 | 0 | 0 | 0 | 0 | 0 | 0 | 0 | 0 | 0 | 0 | 0 | 0 | 1 | 0 | 0 | 0 | 0 | 0 | 0 | 0 | 0 | 0 | 0 | 0 | 0 | 0 | 0 |
| HT60 | 0 | 0 | 0 | 0 | 0 | 0 | 0 | 0 | 0 | 0 | 0 | 0 | 0 | 0 | 0 | 0 | 0 | 0 | 0 | 0 | 2 | 0 | 0 | 0 | 0 | 0 | 0 |
| HT61 | 0 | 0 | 0 | 0 | 0 | 0 | 0 | 0 | 0 | 0 | 0 | 0 | 0 | 0 | 0 | 0 | 0 | 0 | 0 | 0 | 1 | 0 | 0 | 0 | 0 | 0 | 0 |
| HT62 | 0 | 0 | 0 | 0 | 0 | 0 | 0 | 0 | 0 | 0 | 0 | 0 | 0 | 0 | 0 | 0 | 0 | 0 | 0 | 0 | 1 | 0 | 0 | 0 | 0 | 0 | 0 |
| HT63 | 0 | 0 | 0 | 0 | 0 | 0 | 0 | 0 | 0 | 0 | 0 | 0 | 0 | 0 | 0 | 0 | 0 | 0 | 0 | 0 | 1 | 0 | 0 | 0 | 0 | 0 | 0 |
| HT64 | 0 | 0 | 0 | 0 | 0 | 0 | 1 | 0 | 0 | 0 | 0 | 0 | 0 | 0 | 0 | 0 | 0 | 0 | 0 | 0 | 0 | 0 | 0 | 0 | 0 | 0 | 0 |
| HT65 | 0 | 0 | 0 | 0 | 0 | 0 | 1 | 0 | 0 | 0 | 0 | 0 | 0 | 0 | 0 | 0 | 0 | 0 | 0 | 0 | 0 | 0 | 0 | 0 | 0 | 0 | 0 |
| HT66 | 0 | 0 | 0 | 0 | 0 | 0 | 0 | 0 | 0 | 0 | 0 | 0 | 0 | 0 | 0 | 0 | 0 | 0 | 0 | 0 | 1 | 0 | 0 | 0 | 0 | 0 | 0 |
| HT67 | 0 | 0 | 0 | 0 | 0 | 0 | 1 | 0 | 0 | 0 | 0 | 0 | 0 | 0 | 0 | 0 | 0 | 0 | 0 | 0 | 0 | 0 | 0 | 0 | 0 | 0 | 0 |
| HT68 | 0 | 0 | 0 | 0 | 0 | 0 | 0 | 0 | 0 | 0 | 0 | 0 | 0 | 0 | 0 | 0 | 0 | 0 | 0 | 0 | 3 | 0 | 0 | 0 | 0 | 0 | 0 |
| HT69 | 0 | 0 | 0 | 0 | 0 | 0 | 2 | 0 | 0 | 0 | 0 | 0 | 0 | 0 | 0 | 0 | 0 | 0 | 0 | 0 | 0 | 0 | 0 | 0 | 0 | 0 | 0 |
| HT70 | 0 | 0 | 0 | 0 | 0 | 0 | 1 | 0 | 0 | 0 | 0 | 0 | 0 | 0 | 0 | 0 | 0 | 0 | 0 | 0 | 0 | 0 | 0 | 0 | 0 | 0 | 0 |
| HT71 | 0 | 0 | 0 | 0 | 0 | 0 | 1 | 0 | 0 | 0 | 0 | 0 | 0 | 0 | 0 | 0 | 0 | 0 | 0 | 0 | 0 | 0 | 0 | 0 | 0 | 0 | 0 |
| HT72 | 0 | 0 | 0 | 0 | 0 | 0 | 1 | 0 | 0 | 0 | 0 | 0 | 0 | 0 | 0 | 0 | 0 | 0 | 0 | 0 | 0 | 0 | 0 | 0 | 0 | 0 | 0 |
| HT73 | 0 | 0 | 0 | 0 | 0 | 0 | 0 | 0 | 1 | 0 | 0 | 0 | 0 | 0 | 0 | 0 | 0 | 0 | 0 | 0 | 0 | 0 | 0 | 0 | 0 | 0 | 0 |
| HT74 | 0 | 0 | 0 | 0 | 0 | 0 | 0 | 0 | 0 | 0 | 1 | 0 | 0 | 0 | 0 | 0 | 0 | 0 | 0 | 0 | 0 | 0 | 0 | 0 | 0 | 0 | 0 |
| HT75 | 0 | 0 | 0 | 0 | 0 | 0 | 0 | 0 | 0 | 0 | 1 | 0 | 0 | 0 | 0 | 0 | 0 | 0 | 0 | 0 | 0 | 0 | 0 | 0 | 0 | 0 | 0 |
| HT76 | 0 | 0 | 0 | 0 | 0 | 0 | 0 | 0 | 0 | 0 | 0 | 0 | 0 | 1 | 0 | 0 | 0 | 0 | 0 | 0 | 0 | 0 | 0 | 0 | 0 | 0 | 0 |
| HT77 | 0 | 0 | 0 | 0 | 0 | 0 | 0 | 0 | 0 | 0 | 0 | 0 | 0 | 2 | 1 | 0 | 2 | 0 | 0 | 0 | 0 | 0 | 0 | 0 | 0 | 0 | 0 |
| HT78 | 0 | 0 | 0 | 0 | 0 | 0 | 1 | 0 | 0 | 0 | 0 | 0 | 0 | 0 | 0 | 0 | 0 | 0 | 0 | 0 | 0 | 0 | 0 | 0 | 0 | 0 | 0 |
| HT79 | 0 | 0 | 0 | 0 | 0 | 0 | 4 | 0 | 0 | 0 | 0 | 0 | 0 | 0 | 0 | 0 | 0 | 0 | 0 | 0 | 0 | 0 | 0 | 0 | 0 | 0 | 0 |
| HT80 | 0 | 0 | 0 | 0 | 0 | 0 | 1 | 0 | 0 | 0 | 0 | 0 | 0 | 0 | 0 | 0 | 0 | 0 | 0 | 0 | 0 | 0 | 0 | 0 | 0 | 0 | 0 |
| HT81 | 0 | 0 | 0 | 0 | 0 | 0 | 0 | 0 | 0 | 0 | 0 | 0 | 0 | 0 | 0 | 0 | 0 | 0 | 0 | 0 | 1 | 0 | 0 | 0 | 0 | 0 | 0 |
| HT82 | 0 | 0 | 0 | 0 | 0 | 0 | 0 | 0 | 0 | 0 | 0 | 0 | 0 | 0 | 0 | 0 | 0 | 0 | 0 | 0 | 1 | 0 | 0 | 0 | 0 | 0 | 0 |
| HT83 | 0 | 0 | 0 | 0 | 0 | 0 | 1 | 0 | 0 | 0 | 0 | 0 | 0 | 0 | 0 | 0 | 0 | 0 | 0 | 0 | 0 | 0 | 0 | 0 | 0 | 0 | 0 |
| HT84 | 0 | 0 | 0 | 0 | 0 | 0 | 0 | 0 | 0 | 0 | 0 | 0 | 0 | 0 | 0 | 0 | 0 | 0 | 0 | 0 | 0 | 0 | 1 | 0 | 0 | 0 | 0 |
| HT85 | 0 | 0 | 0 | 0 | 0 | 0 | 0 | 0 | 0 | 0 | 0 | 0 | 0 | 0 | 0 | 0 | 0 | 0 | 0 | 0 | 0 | 0 | 2 | 1 | 0 | 0 | 0 |
| HT86 | 0 | 0 | 0 | 0 | 0 | 0 | 0 | 0 | 0 | 0 | 0 | 0 | 0 | 0 | 0 | 0 | 0 | 0 | 0 | 0 | 0 | 0 | 3 | 0 | 0 | 0 | 0 |
| HT87 | 0 | 0 | 0 | 0 | 0 | 0 | 0 | 0 | 0 | 0 | 0 | 0 | 0 | 0 | 0 | 0 | 0 | 0 | 0 | 0 | 0 | 0 | 2 | 0 | 0 | 0 | 0 |
| HT88 | 0 | 0 | 0 | 0 | 0 | 0 | 0 | 0 | 0 | 0 | 0 | 0 | 0 | 0 | 0 | 0 | 0 | 0 | 0 | 0 | 0 | 0 | 0 | 1 | 0 | 0 | 0 |
| HT89 | 0 | 0 | 0 | 0 | 0 | 0 | 0 | 0 | 0 | 0 | 0 | 0 | 0 | 0 | 0 | 0 | 0 | 0 | 0 | 0 | 0 | 0 | 0 | 0 | 0 | 0 | 1 |
| HT90 | 0 | 0 | 0 | 0 | 0 | 0 | 0 | 0 | 0 | 0 | 0 | 0 | 0 | 0 | 0 | 0 | 0 | 0 | 0 | 0 | 0 | 0 | 0 | 0 | 0 | 0 | 1 |
| HT91 | 0 | 0 | 0 | 0 | 0 | 0 | 0 | 0 | 0 | 0 | 0 | 0 | 0 | 0 | 0 | 0 | 0 | 0 | 0 | 0 | 0 | 0 | 0 | 0 | 0 | 0 | 1 |
| HT92 | 0 | 0 | 0 | 0 | 0 | 0 | 0 | 0 | 0 | 0 | 0 | 0 | 0 | 0 | 0 | 0 | 0 | 0 | 0 | 0 | 0 | 0 | 1 | 0 | 0 | 0 | 0 |
| HT93 | 0 | 0 | 0 | 0 | 0 | 0 | 0 | 0 | 0 | 0 | 0 | 0 | 0 | 0 | 0 | 0 | 0 | 0 | 0 | 0 | 0 | 0 | 4 | 0 | 0 | 0 | 0 |
| HT94 | 0 | 0 | 0 | 0 | 0 | 0 | 0 | 0 | 0 | 0 | 0 | 0 | 0 | 0 | 0 | 0 | 0 | 0 | 0 | 0 | 0 | 0 | 3 | 0 | 0 | 0 | 0 |
| HT95 | 0 | 0 | 0 | 0 | 0 | 0 | 0 | 0 | 0 | 0 | 0 | 0 | 0 | 0 | 0 | 0 | 0 | 0 | 0 | 0 | 0 | 0 | 1 | 0 | 0 | 0 | 0 |
| HT96 | 0 | 0 | 0 | 0 | 0 | 0 | 0 | 0 | 0 | 0 | 0 | 0 | 0 | 0 | 0 | 0 | 0 | 0 | 0 | 0 | 0 | 0 | 0 | 1 | 0 | 0 | 0 |
| HT97 | 0 | 0 | 0 | 0 | 0 | 0 | 0 | 0 | 0 | 0 | 0 | 0 | 0 | 0 | 0 | 0 | 0 | 0 | 0 | 0 | 0 | 0 | 0 | 0 | 0 | 0 | 1 |
| HT98 | 0 | 0 | 0 | 0 | 0 | 0 | 0 | 0 | 0 | 0 | 0 | 0 | 0 | 0 | 0 | 0 | 0 | 0 | 0 | 0 | 0 | 0 | 0 | 0 | 0 | 0 | 2 |
| HT99 | 0 | 0 | 0 | 0 | 0 | 0 | 0 | 0 | 0 | 0 | 0 | 0 | 0 | 0 | 0 | 0 | 0 | 0 | 0 | 0 | 0 | 0 | 0 | 0 | 0 | 0 | 1 |
| HT100 | 0 | 0 | 0 | 0 | 0 | 0 | 0 | 0 | 0 | 0 | 0 | 0 | 0 | 0 | 0 | 0 | 0 | 0 | 0 | 0 | 0 | 0 | 0 | 0 | 0 | 0 | 1 |
| HT101 | 0 | 0 | 0 | 0 | 0 | 0 | 0 | 0 | 0 | 0 | 0 | 0 | 0 | 0 | 0 | 0 | 1 | 0 | 0 | 0 | 0 | 0 | 0 | 0 | 0 | 0 | 0 |
| HT102 | 0 | 0 | 0 | 0 | 0 | 0 | 0 | 0 | 0 | 0 | 0 | 0 | 0 | 1 | 0 | 0 | 1 | 0 | 0 | 0 | 0 | 0 | 0 | 0 | 0 | 0 | 0 |
| HT103 | 0 | 0 | 0 | 0 | 0 | 0 | 0 | 0 | 0 | 0 | 0 | 0 | 0 | 1 | 0 | 0 | 0 | 0 | 0 | 0 | 0 | 0 | 0 | 0 | 0 | 0 | 0 |
| HT104 | 0 | 0 | 0 | 0 | 0 | 0 | 0 | 0 | 0 | 0 | 0 | 0 | 0 | 0 | 0 | 0 | 1 | 0 | 0 | 0 | 0 | 0 | 0 | 0 | 0 | 0 | 0 |
| HT105 | 0 | 0 | 0 | 0 | 0 | 0 | 0 | 0 | 0 | 0 | 0 | 0 | 1 | 2 | 1 | 1 | 0 | 0 | 0 | 0 | 0 | 0 | 0 | 0 | 0 | 0 | 0 |
| HT106 | 0 | 0 | 0 | 0 | 0 | 0 | 0 | 0 | 0 | 0 | 0 | 0 | 0 | 1 | 1 | 0 | 0 | 0 | 0 | 0 | 0 | 0 | 0 | 0 | 0 | 0 | 0 |

Table 56: Haplotype counts per Location according to pegas *(continued)*

| | GOC | COR | SIL | SAF | RUN | SEY | ARS | SRL | TAI | IND | PNG | FZR | VIR | DAR | ADR | SAR | EAR | BMB | ROR | TOR | WER | TRI | CLR | SYH | URR | OKI | FIJ |
|---|---|---|---|---|---|---|---|---|---|---|---|---|---|---|---|---|---|---|---|---|---|---|---|---|---|---|---|
| HT107 | 0 | 0 | 0 | 0 | 0 | 0 | 0 | 0 | 0 | 0 | 0 | 0 | 0 | 0 | 0 | 0 | 0 | 0 | 0 | 0 | 0 | 0 | 1 | 0 | 0 | 0 | 0 |
| HT108 | 0 | 0 | 0 | 1 | 0 | 0 | 0 | 0 | 0 | 0 | 0 | 0 | 0 | 0 | 0 | 0 | 0 | 0 | 0 | 0 | 0 | 0 | 0 | 0 | 0 | 0 | 0 |
| HT109 | 0 | 0 | 0 | 0 | 0 | 0 | 0 | 0 | 0 | 0 | 0 | 0 | 0 | 0 | 0 | 0 | 0 | 0 | 0 | 0 | 0 | 0 | 0 | 1 | 0 | 0 | 0 |
| HT110 | 0 | 0 | 0 | 0 | 0 | 0 | 0 | 0 | 0 | 0 | 1 | 0 | 0 | 0 | 0 | 0 | 0 | 0 | 0 | 0 | 0 | 0 | 0 | 0 | 0 | 0 | 0 |
| HT111 | 0 | 0 | 0 | 0 | 0 | 0 | 0 | 0 | 0 | 0 | 1 | 0 | 0 | 0 | 0 | 0 | 0 | 0 | 0 | 0 | 0 | 0 | 0 | 0 | 0 | 0 | 0 |
| HT112 | 0 | 0 | 0 | 0 | 0 | 0 | 0 | 0 | 0 | 0 | 0 | 0 | 0 | 0 | 0 | 0 | 0 | 0 | 0 | 0 | 0 | 0 | 0 | 1 | 0 | 0 | 0 |
| HT113 | 0 | 0 | 0 | 0 | 0 | 0 | 0 | 0 | 0 | 0 | 1 | 0 | 0 | 0 | 0 | 0 | 0 | 0 | 0 | 0 | 0 | 0 | 0 | 0 | 0 | 0 | 0 |
| HT114 | 0 | 0 | 0 | 0 | 0 | 0 | 0 | 0 | 0 | 0 | 1 | 0 | 0 | 0 | 0 | 0 | 0 | 0 | 0 | 0 | 0 | 0 | 0 | 0 | 0 | 0 | 0 |
| HT115 | 0 | 0 | 0 | 0 | 0 | 0 | 0 | 0 | 0 | 0 | 2 | 0 | 0 | 0 | 0 | 0 | 0 | 0 | 0 | 0 | 0 | 0 | 0 | 0 | 0 | 0 | 0 |
| HT116 | 0 | 0 | 0 | 0 | 0 | 0 | 0 | 0 | 0 | 0 | 2 | 0 | 0 | 0 | 0 | 0 | 0 | 0 | 0 | 0 | 0 | 0 | 0 | 0 | 0 | 0 | 0 |
| HT117 | 0 | 0 | 0 | 0 | 0 | 0 | 0 | 0 | 0 | 0 | 0 | 0 | 0 | 0 | 0 | 0 | 0 | 0 | 0 | 0 | 0 | 0 | 1 | 2 | 0 | 0 | 0 |
| HT118 | 0 | 0 | 0 | 0 | 0 | 0 | 0 | 0 | 0 | 0 | 0 | 0 | 0 | 0 | 0 | 0 | 0 | 0 | 0 | 0 | 0 | 0 | 0 | 1 | 0 | 0 | 0 |
| HT119 | 0 | 0 | 0 | 0 | 0 | 0 | 0 | 0 | 0 | 0 | 1 | 0 | 0 | 0 | 0 | 0 | 0 | 0 | 0 | 0 | 0 | 0 | 0 | 0 | 0 | 0 | 0 |
| HT120 | 0 | 0 | 0 | 0 | 0 | 0 | 0 | 0 | 0 | 0 | 0 | 0 | 0 | 0 | 0 | 0 | 0 | 0 | 0 | 1 | 0 | 0 | 0 | 0 | 0 | 0 | 0 |
| HT121 | 0 | 0 | 0 | 0 | 0 | 0 | 0 | 0 | 0 | 0 | 0 | 0 | 0 | 0 | 0 | 0 | 0 | 0 | 0 | 0 | 0 | 0 | 1 | 0 | 0 | 0 | 0 |
| HT122 | 0 | 0 | 0 | 0 | 0 | 0 | 0 | 0 | 0 | 0 | 1 | 0 | 0 | 0 | 0 | 0 | 0 | 0 | 0 | 0 | 0 | 0 | 0 | 0 | 0 | 0 | 0 |
| HT123 | 0 | 0 | 0 | 0 | 0 | 0 | 0 | 0 | 0 | 0 | 1 | 0 | 0 | 0 | 0 | 0 | 0 | 0 | 0 | 0 | 0 | 0 | 0 | 0 | 0 | 0 | 0 |
| HT124 | 0 | 0 | 0 | 0 | 0 | 0 | 0 | 0 | 0 | 0 | 0 | 0 | 0 | 0 | 0 | 0 | 0 | 0 | 0 | 0 | 0 | 0 | 0 | 0 | 4 | 1 | 0 |
| HT125 | 0 | 0 | 0 | 0 | 0 | 0 | 0 | 0 | 0 | 0 | 0 | 0 | 0 | 0 | 0 | 0 | 0 | 0 | 0 | 0 | 0 | 0 | 0 | 0 | 2 | 0 | 0 |
| HT126 | 0 | 0 | 0 | 0 | 0 | 0 | 0 | 0 | 0 | 0 | 0 | 0 | 0 | 0 | 0 | 0 | 0 | 1 | 0 | 0 | 0 | 0 | 0 | 0 | 0 | 0 | 0 |
| HT127 | 0 | 0 | 0 | 0 | 0 | 0 | 0 | 0 | 0 | 0 | 0 | 0 | 0 | 0 | 0 | 0 | 0 | 3 | 0 | 0 | 2 | 0 | 0 | 0 | 0 | 0 | 0 |
| HT128 | 0 | 0 | 0 | 0 | 0 | 0 | 0 | 0 | 0 | 0 | 0 | 0 | 0 | 0 | 0 | 0 | 0 | 4 | 0 | 0 | 0 | 0 | 0 | 0 | 0 | 0 | 0 |
| HT129 | 0 | 0 | 0 | 0 | 0 | 0 | 0 | 0 | 0 | 0 | 0 | 0 | 0 | 1 | 0 | 0 | 0 | 0 | 0 | 0 | 0 | 0 | 0 | 0 | 0 | 0 | 0 |
| HT130 | 0 | 0 | 0 | 0 | 0 | 0 | 0 | 0 | 0 | 0 | 0 | 0 | 0 | 1 | 0 | 0 | 0 | 0 | 0 | 0 | 0 | 0 | 0 | 0 | 0 | 0 | 0 |
| HT131 | 0 | 0 | 0 | 0 | 0 | 0 | 0 | 0 | 0 | 1 | 0 | 0 | 0 | 0 | 0 | 0 | 0 | 0 | 0 | 0 | 0 | 0 | 0 | 0 | 0 | 0 | 0 |
| HT132 | 0 | 0 | 0 | 0 | 0 | 0 | 0 | 0 | 0 | 1 | 0 | 0 | 0 | 0 | 0 | 0 | 0 | 0 | 0 | 0 | 0 | 0 | 0 | 0 | 0 | 0 | 0 |
| HT133 | 0 | 0 | 0 | 0 | 0 | 0 | 0 | 1 | 0 | 0 | 0 | 0 | 0 | 0 | 0 | 0 | 0 | 0 | 0 | 0 | 0 | 0 | 0 | 0 | 0 | 0 | 0 |
| HT134 | 0 | 0 | 0 | 0 | 0 | 0 | 0 | 0 | 0 | 0 | 0 | 0 | 1 | 0 | 0 | 0 | 0 | 0 | 0 | 0 | 0 | 0 | 0 | 0 | 0 | 0 | 0 |
| HT135 | 0 | 0 | 0 | 0 | 0 | 0 | 0 | 0 | 0 | 0 | 0 | 0 | 1 | 0 | 0 | 0 | 0 | 0 | 0 | 0 | 0 | 0 | 0 | 0 | 0 | 0 | 0 |
| HT136 | 0 | 0 | 0 | 0 | 0 | 0 | 0 | 0 | 0 | 0 | 0 | 0 | 0 | 0 | 2 | 1 | 0 | 0 | 0 | 0 | 0 | 0 | 0 | 0 | 0 | 0 | 0 |
| HT137 | 0 | 0 | 0 | 0 | 0 | 0 | 1 | 1 | 0 | 0 | 0 | 0 | 0 | 0 | 0 | 0 | 1 | 0 | 0 | 0 | 0 | 0 | 0 | 0 | 0 | 0 | 0 |
| HT138 | 0 | 0 | 0 | 0 | 0 | 0 | 0 | 1 | 0 | 0 | 0 | 0 | 0 | 0 | 0 | 0 | 0 | 0 | 0 | 0 | 0 | 0 | 0 | 0 | 0 | 0 | 0 |
| HT139 | 0 | 0 | 0 | 0 | 0 | 0 | 0 | 0 | 0 | 0 | 0 | 0 | 0 | 0 | 1 | 0 | 0 | 0 | 0 | 0 | 0 | 0 | 0 | 0 | 0 | 0 | 0 |
| HT140 | 0 | 0 | 0 | 0 | 0 | 0 | 0 | 1 | 0 | 0 | 0 | 0 | 0 | 0 | 0 | 0 | 0 | 0 | 0 | 0 | 0 | 0 | 0 | 0 | 0 | 0 | 0 |
| HT141 | 0 | 0 | 0 | 0 | 0 | 0 | 0 | 1 | 0 | 0 | 0 | 0 | 0 | 0 | 0 | 0 | 0 | 0 | 0 | 0 | 0 | 0 | 0 | 0 | 0 | 0 | 0 |
| HT142 | 0 | 0 | 0 | 0 | 0 | 0 | 0 | 0 | 0 | 0 | 0 | 0 | 1 | 0 | 0 | 0 | 0 | 0 | 0 | 0 | 0 | 0 | 0 | 0 | 0 | 0 | 0 |
| HT143 | 0 | 0 | 0 | 0 | 0 | 0 | 0 | 0 | 0 | 1 | 0 | 0 | 0 | 0 | 0 | 0 | 0 | 0 | 0 | 0 | 0 | 0 | 0 | 0 | 0 | 0 | 0 |
| HT144 | 0 | 0 | 0 | 0 | 0 | 0 | 0 | 0 | 0 | 1 | 0 | 0 | 0 | 0 | 0 | 0 | 0 | 0 | 0 | 0 | 0 | 0 | 0 | 0 | 0 | 0 | 0 |
| HT145 | 0 | 0 | 0 | 0 | 0 | 0 | 0 | 0 | 0 | 1 | 0 | 0 | 0 | 0 | 0 | 0 | 0 | 0 | 0 | 0 | 0 | 0 | 0 | 0 | 0 | 0 | 0 |
| HT146 | 0 | 0 | 0 | 0 | 0 | 0 | 0 | 0 | 0 | 0 | 0 | 0 | 0 | 0 | 1 | 0 | 0 | 0 | 0 | 0 | 0 | 0 | 0 | 0 | 0 | 0 | 0 |
| HT147 | 0 | 0 | 0 | 0 | 0 | 0 | 0 | 0 | 0 | 0 | 0 | 0 | 0 | 0 | 0 | 0 | 0 | 0 | 0 | 0 | 0 | 0 | 0 | 1 | 0 | 0 | 0 |
| HT148 | 0 | 0 | 0 | 0 | 0 | 0 | 0 | 0 | 0 | 0 | 0 | 0 | 0 | 0 | 1 | 0 | 0 | 0 | 0 | 0 | 0 | 0 | 0 | 0 | 0 | 0 | 0 |
| HT149 | 0 | 0 | 0 | 0 | 0 | 0 | 0 | 0 | 0 | 0 | 0 | 0 | 0 | 0 | 1 | 0 | 0 | 0 | 0 | 0 | 0 | 0 | 0 | 0 | 0 | 0 | 0 |
| HT150 | 0 | 0 | 0 | 0 | 0 | 0 | 0 | 0 | 0 | 0 | 0 | 0 | 1 | 1 | 1 | 0 | 0 | 0 | 0 | 0 | 0 | 0 | 0 | 0 | 0 | 0 | 0 |
| HT151 | 0 | 0 | 0 | 0 | 0 | 0 | 0 | 0 | 0 | 0 | 1 | 0 | 0 | 0 | 0 | 0 | 0 | 0 | 0 | 0 | 0 | 0 | 0 | 0 | 0 | 0 | 0 |
| HT152 | 0 | 0 | 0 | 0 | 0 | 0 | 0 | 0 | 0 | 0 | 0 | 0 | 0 | 0 | 0 | 0 | 0 | 0 | 0 | 0 | 0 | 0 | 0 | 0 | 4 | 7 | 0 |
| HT153 | 0 | 0 | 0 | 0 | 0 | 0 | 0 | 0 | 0 | 0 | 0 | 0 | 0 | 0 | 0 | 0 | 0 | 0 | 0 | 0 | 0 | 0 | 0 | 0 | 0 | 13 | 0 |
| HT154 | 0 | 0 | 0 | 0 | 0 | 0 | 0 | 0 | 1 | 0 | 0 | 0 | 0 | 0 | 0 | 0 | 0 | 0 | 0 | 0 | 0 | 0 | 0 | 0 | 0 | 0 | 0 |
| HT155 | 0 | 4 | 0 | 0 | 0 | 0 | 0 | 0 | 0 | 0 | 0 | 0 | 0 | 0 | 0 | 0 | 0 | 0 | 0 | 0 | 0 | 0 | 0 | 0 | 0 | 0 | 0 |
| HT156 | 0 | 2 | 0 | 0 | 0 | 0 | 0 | 0 | 0 | 0 | 0 | 0 | 0 | 0 | 0 | 0 | 0 | 0 | 0 | 0 | 0 | 0 | 0 | 0 | 0 | 0 | 0 |
| HT157 | 0 | 1 | 0 | 0 | 0 | 0 | 0 | 0 | 0 | 0 | 0 | 0 | 0 | 0 | 0 | 0 | 0 | 0 | 0 | 0 | 0 | 0 | 0 | 0 | 0 | 0 | 0 |
| HT158 | 0 | 2 | 0 | 0 | 0 | 0 | 0 | 0 | 0 | 0 | 0 | 0 | 0 | 0 | 0 | 0 | 0 | 0 | 0 | 0 | 0 | 0 | 0 | 0 | 0 | 0 | 0 |
| HT159 | 0 | 1 | 0 | 0 | 0 | 0 | 0 | 0 | 0 | 0 | 0 | 0 | 0 | 0 | 0 | 0 | 0 | 0 | 0 | 0 | 0 | 0 | 0 | 0 | 0 | 0 | 0 |

Table 56: Haplotype counts per Location according to pegas *(continued)*

| | GOC | COR | SIL | SAF | RUN | SEY | ARS | SRL | TAI | IND | PNG | FZR | VIR | DAR | ADR | SAR | EAR | BMB | ROR | TOR | WER | TRI | CLR | SYH | URR | OKI | FIJ |
|---|---|---|---|---|---|---|---|---|---|---|---|---|---|---|---|---|---|---|---|---|---|---|---|---|---|---|---|
| HT160 | 0 | 1 | 0 | 0 | 0 | 0 | 0 | 0 | 0 | 0 | 0 | 0 | 0 | 0 | 0 | 0 | 0 | 0 | 0 | 0 | 0 | 0 | 0 | 0 | 0 | 0 | 0 |
| HT161 | 0 | 1 | 0 | 0 | 0 | 0 | 0 | 0 | 0 | 0 | 0 | 0 | 0 | 0 | 0 | 0 | 0 | 0 | 0 | 0 | 0 | 0 | 0 | 0 | 0 | 0 | 0 |
| HT162 | 1 | 0 | 0 | 0 | 0 | 0 | 0 | 0 | 0 | 0 | 0 | 0 | 0 | 0 | 0 | 0 | 0 | 0 | 0 | 0 | 0 | 0 | 0 | 0 | 0 | 0 | 0 |
| HT163 | 0 | 2 | 0 | 0 | 0 | 0 | 0 | 0 | 0 | 0 | 0 | 0 | 0 | 0 | 0 | 0 | 0 | 0 | 0 | 0 | 0 | 0 | 0 | 0 | 0 | 0 | 0 |
| HT164 | 0 | 1 | 0 | 0 | 0 | 0 | 0 | 0 | 0 | 0 | 0 | 0 | 0 | 0 | 0 | 0 | 0 | 0 | 0 | 0 | 0 | 0 | 0 | 0 | 0 | 0 | 0 |
| HT165 | 0 | 0 | 1 | 0 | 0 | 0 | 0 | 0 | 0 | 0 | 0 | 0 | 0 | 0 | 0 | 0 | 0 | 0 | 0 | 0 | 0 | 0 | 0 | 0 | 0 | 0 | 0 |

```r
strata <- as.character(BS.gt@schemes$Site)
names(strata) <- as.character(BS.gt@schemes$id)
strataG::setStrata(BS.gt) <- strata
BS.gt.AUS <- BS.gt[,,"Australia", drop = TRUE]


schemes.AUS <- BS.gt.AUS@schemes[BS.gt.AUS@schemes$Site == "Australia",]
strata <- as.character(schemes.AUS$Location)
names(strata) <- as.character(schemes.AUS$id)
strataG::setStrata(BS.gt.AUS) <- strata
BS.gt.AUS@schemes <- schemes.AUS

group.levels <- c("Fitzroy River", "Victoria River", "Daly River","Adelaide River",
                  "South Alligator River","East Alligator River","Blue mud Bay",
                  "Roper River","Towns River","Wenlock River","Trinity inlet",
                  "Clarence River","Sydney Harbour")
BS.gt.AUS@schemes$Location <- factor(BS.gt.AUS@schemes$Location,
                                     levels = group.levels)

ind.names <- names(BS.dna) %in% BS.gt.AUS@data$id
BS.dna.AUS <- as.dna(BS.dna[ind.names, , drop = TRUE])
BS.bin.AUS <- subset(BS.bin, names(BS.bin) %in% BS.gt.AUS@data$id)
BS.hap.aus <- strataG::labelHaplotypes(BS.bin.AUS, prefix = "HT",
                                       use.indels = TRUE)
haps <- BS.hap.aus$haps
HTs <- unique(haps[names(haps) %in% names(BS.bin.AUS)])
BS.h.AUS <- pegas::haplotype(BS.bin.AUS, HTs)



hapcount <- pegas::haploFreq(BS.dna.AUS@sequence,
                             fac = BS.gt.AUS@schemes$Location,
                             split = NULL, what = 1, haplo = NULL)
row.names(hapcount) <- paste0("HT", sprintf('%0.3d', 1:85))
shortnames <- c("FZR", "VIR", "DAR", "ADR","SAR", "EAR","BMB", "ROR","TOR",
                "WER","TRI","CLR", "SYH")

kableExtra::kbl(hapcount, col.names = shortnames,
  caption = "Haplotype counts per Site according to pegas",
  longtable = TRUE, booktabs = TRUE) %>%
  kableExtra::kable_styling(full_width = FALSE,font_size = 5,
                            latex_options = c("repeat_header", "hold_position"))

group.names <- BS.gt.AUS@schemes$Location
BS.hap.aus <- haplotypes::haplotype(BS.dna.AUS,indels = "sic")
HTs <- paste0("HT", sprintf('%0.3d', 1:85))
df <- data.frame(group = NULL,nind = NULL, hap = NULL, nhap = NULL,
                 hapfreq = NULL, stringsAsFactors = FALSE)
for (k in 1:length(group.levels)) {
  group.inds <- BS.gt.AUS@schemes$id[group.names == group.levels[k]]
  for (i in 1:85) {
    group <- group.levels[k]
    nind <- length(group.inds)
    hap <- HTs[i]
    nhap <- sum(group.inds %in% BS.hap.aus@haplist[[i]])
    # nhap <- sum(group.inds %in% names(BS.hap.aus$haps)[BS.hap.aus$haps == hap])
    hapfreq <- nhap/nind
```

```
    df <- plyr::rbind.fill(df, data.frame(group = group, nind = nind,
                                           hap = hap,
                                           nhap = nhap, hapfreq = hapfreq,
                                           stringsAsFactors = FALSE))
  }
}

df$group <- factor(df$group, levels = group.levels)

HT.plot <- ggplot2::ggplot(data = df) +
  ggplot2::geom_bar(ggplot2::aes(x = hap, y = nhap, fill = group),
                    stat = "identity",position = "stack") +
  ggplot2::labs(x = "Haplotypes", y = "Number of individuals") +
  ggplot2::scale_fill_manual(values = colours.13) +
  ggplot2::theme(
    axis.text = ggplot2::element_text(angle = 45, hjust = 1, size = 5),
    axis.title.x = ggplot2::element_text(size = 10),
    axis.title.y = ggplot2::element_text(size = 10),
    legend.text = ggplot2::element_text(size = 7))
print(HT.plot)
```

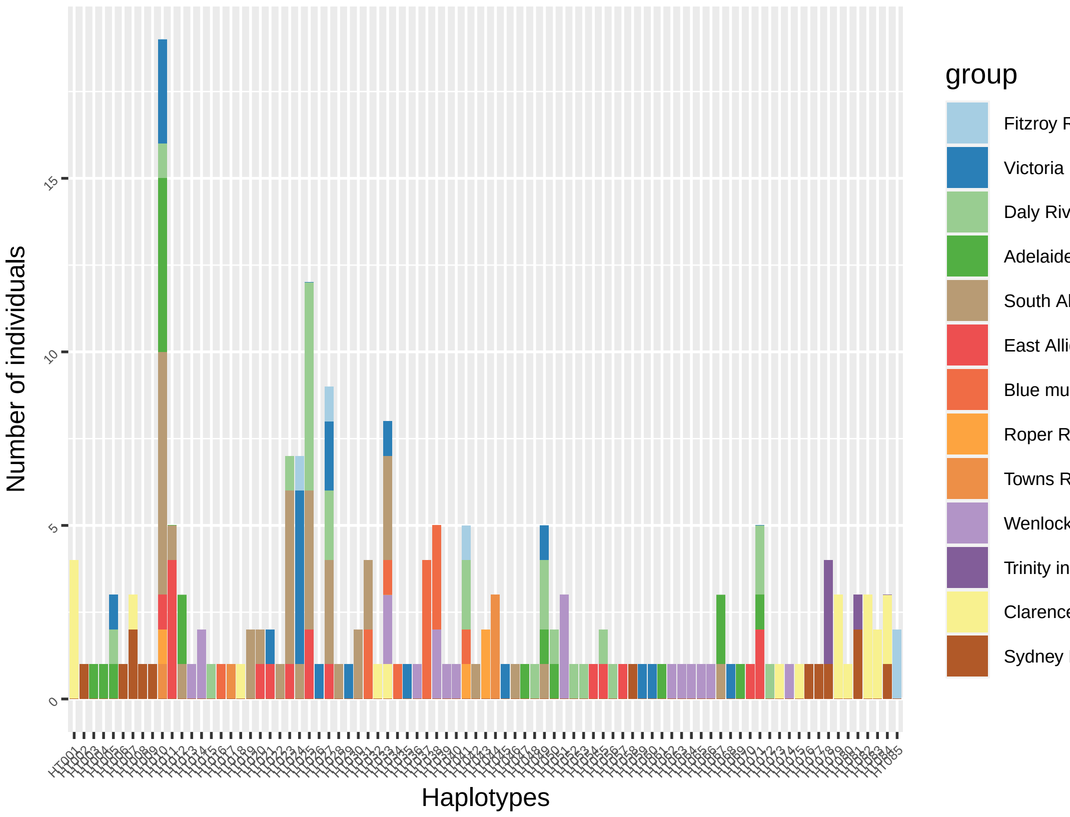


#### 13.3.2.1 Within AUS

```
ggplot2::ggsave(HT.plot, filename = "HT_barplot_per_Location_AUS.png",
                width = 30, height = 15, units = "cm")
```

Table 57: Haplotype counts per Site according to pegas

| | FZR | VIR | DAR | ADR | SAR | EAR | BMB | ROR | TOR | WER | TRI | CLR | SYH |
|---|---|---|---|---|---|---|---|---|---|---|---|---|---|
| HT001 | 2 | 0 | 0 | 0 | 0 | 0 | 0 | 0 | 0 | 0 | 0 | 0 | 0 |
| HT002 | 0 | 0 | 0 | 0 | 0 | 0 | 0 | 0 | 0 | 0 | 1 | 0 | 2 |
| HT003 | 0 | 0 | 0 | 0 | 0 | 0 | 0 | 0 | 0 | 0 | 3 | 0 | 1 |
| HT004 | 0 | 0 | 1 | 0 | 0 | 0 | 0 | 0 | 0 | 0 | 0 | 0 | 0 |
| HT005 | 0 | 0 | 0 | 0 | 0 | 0 | 0 | 0 | 0 | 0 | 0 | 0 | 1 |
| HT006 | 0 | 0 | 0 | 0 | 0 | 0 | 0 | 0 | 0 | 1 | 0 | 0 | 0 |
| HT007 | 0 | 0 | 0 | 0 | 2 | 0 | 0 | 0 | 0 | 0 | 0 | 0 | 0 |
| HT008 | 0 | 0 | 0 | 0 | 1 | 4 | 0 | 0 | 0 | 0 | 0 | 0 | 0 |

Table 57: Haplotype counts per Site according to pegas *(continued)*

| | FZR | VIR | DAR | ADR | SAR | EAR | BMB | ROR | TOR | WER | TRI | CLR | SYH |
|---|---|---|---|---|---|---|---|---|---|---|---|---|---|
| HT009 | 0 | 0 | 0 | 2 | 1 | 0 | 0 | 0 | 0 | 0 | 0 | 0 | 0 |
| HT010 | 0 | 1 | 0 | 0 | 0 | 0 | 0 | 0 | 0 | 0 | 0 | 0 | 0 |
| HT011 | 1 | 5 | 0 | 0 | 1 | 0 | 0 | 0 | 0 | 0 | 0 | 0 | 0 |
| HT012 | 0 | 1 | 0 | 0 | 0 | 0 | 0 | 0 | 0 | 0 | 0 | 0 | 0 |
| HT013 | 0 | 0 | 0 | 0 | 0 | 0 | 0 | 0 | 3 | 0 | 0 | 0 | 0 |
| HT014 | 0 | 0 | 0 | 0 | 0 | 0 | 0 | 2 | 0 | 0 | 0 | 0 | 0 |
| HT015 | 0 | 0 | 0 | 0 | 1 | 1 | 0 | 0 | 0 | 0 | 0 | 0 | 0 |
| HT016 | 0 | 0 | 0 | 0 | 2 | 0 | 0 | 0 | 0 | 0 | 0 | 0 | 0 |
| HT017 | 0 | 1 | 0 | 0 | 0 | 1 | 0 | 0 | 0 | 0 | 0 | 0 | 0 |
| HT018 | 0 | 0 | 0 | 0 | 1 | 0 | 0 | 0 | 0 | 0 | 0 | 0 | 0 |
| HT019 | 0 | 0 | 0 | 0 | 1 | 0 | 0 | 0 | 0 | 0 | 0 | 0 | 0 |
| HT020 | 0 | 0 | 0 | 0 | 1 | 0 | 0 | 0 | 0 | 0 | 0 | 0 | 0 |
| HT021 | 1 | 0 | 2 | 0 | 0 | 0 | 1 | 1 | 0 | 0 | 0 | 0 | 0 |
| HT022 | 0 | 0 | 0 | 0 | 2 | 0 | 2 | 0 | 0 | 0 | 0 | 0 | 0 |
| HT023 | 1 | 2 | 2 | 0 | 3 | 1 | 0 | 0 | 0 | 0 | 0 | 0 | 0 |
| HT024 | 0 | 1 | 0 | 0 | 0 | 0 | 0 | 0 | 0 | 0 | 0 | 0 | 0 |
| HT025 | 0 | 0 | 0 | 1 | 0 | 0 | 0 | 0 | 0 | 0 | 0 | 0 | 0 |
| HT026 | 0 | 0 | 0 | 0 | 1 | 0 | 0 | 0 | 0 | 0 | 0 | 0 | 0 |
| HT027 | 0 | 0 | 6 | 0 | 4 | 2 | 0 | 0 | 0 | 0 | 0 | 0 | 0 |
| HT028 | 0 | 0 | 1 | 0 | 5 | 1 | 0 | 0 | 0 | 0 | 0 | 0 | 0 |
| HT029 | 0 | 0 | 1 | 0 | 0 | 0 | 0 | 0 | 0 | 0 | 0 | 0 | 0 |
| HT030 | 0 | 0 | 0 | 0 | 0 | 0 | 1 | 0 | 0 | 0 | 0 | 0 | 0 |
| HT031 | 0 | 0 | 0 | 0 | 0 | 0 | 0 | 0 | 0 | 1 | 0 | 0 | 0 |
| HT032 | 0 | 3 | 1 | 5 | 7 | 1 | 0 | 1 | 1 | 0 | 0 | 0 | 0 |
| HT033 | 0 | 1 | 0 | 0 | 3 | 0 | 1 | 0 | 0 | 2 | 0 | 1 | 0 |
| HT034 | 0 | 0 | 0 | 0 | 0 | 0 | 0 | 0 | 0 | 1 | 0 | 0 | 0 |
| HT035 | 0 | 0 | 0 | 0 | 0 | 0 | 0 | 0 | 0 | 1 | 0 | 0 | 0 |
| HT036 | 0 | 1 | 0 | 0 | 0 | 0 | 0 | 0 | 0 | 0 | 0 | 0 | 0 |
| HT037 | 0 | 0 | 0 | 0 | 0 | 0 | 0 | 0 | 0 | 2 | 0 | 0 | 0 |
| HT038 | 0 | 0 | 0 | 0 | 0 | 0 | 0 | 0 | 0 | 1 | 0 | 0 | 0 |
| HT039 | 0 | 0 | 0 | 0 | 0 | 0 | 0 | 0 | 0 | 1 | 0 | 0 | 0 |
| HT040 | 0 | 0 | 0 | 0 | 0 | 0 | 0 | 0 | 0 | 1 | 0 | 0 | 0 |
| HT041 | 0 | 0 | 0 | 0 | 0 | 0 | 0 | 0 | 0 | 1 | 0 | 0 | 0 |
| HT042 | 0 | 0 | 0 | 0 | 0 | 0 | 0 | 0 | 0 | 3 | 0 | 0 | 0 |
| HT043 | 0 | 0 | 1 | 0 | 0 | 0 | 0 | 0 | 0 | 0 | 0 | 0 | 0 |
| HT044 | 0 | 0 | 2 | 1 | 0 | 2 | 0 | 0 | 0 | 0 | 0 | 0 | 0 |
| HT045 | 0 | 0 | 0 | 0 | 0 | 0 | 0 | 0 | 0 | 1 | 0 | 0 | 0 |
| HT046 | 0 | 0 | 0 | 0 | 0 | 0 | 0 | 0 | 0 | 1 | 0 | 0 | 0 |
| HT047 | 0 | 0 | 0 | 0 | 0 | 0 | 0 | 0 | 0 | 0 | 0 | 1 | 0 |
| HT048 | 0 | 0 | 0 | 0 | 0 | 0 | 0 | 0 | 0 | 0 | 0 | 2 | 1 |
| HT049 | 0 | 0 | 0 | 0 | 0 | 0 | 0 | 0 | 0 | 0 | 0 | 3 | 0 |
| HT050 | 0 | 0 | 0 | 0 | 0 | 0 | 0 | 0 | 0 | 0 | 0 | 2 | 0 |
| HT051 | 0 | 0 | 0 | 0 | 0 | 0 | 0 | 0 | 0 | 0 | 0 | 0 | 1 |
| HT052 | 0 | 0 | 0 | 0 | 0 | 0 | 0 | 0 | 0 | 0 | 0 | 1 | 0 |
| HT053 | 0 | 0 | 0 | 0 | 0 | 0 | 0 | 0 | 0 | 0 | 0 | 4 | 0 |
| HT054 | 0 | 0 | 0 | 0 | 0 | 0 | 0 | 0 | 0 | 0 | 0 | 3 | 0 |
| HT055 | 0 | 0 | 0 | 0 | 0 | 0 | 0 | 0 | 0 | 0 | 0 | 1 | 0 |
| HT056 | 0 | 0 | 0 | 0 | 0 | 0 | 0 | 0 | 0 | 0 | 0 | 0 | 1 |
| HT057 | 0 | 0 | 0 | 0 | 0 | 1 | 0 | 0 | 0 | 0 | 0 | 0 | 0 |
| HT058 | 0 | 0 | 1 | 0 | 0 | 1 | 0 | 0 | 0 | 0 | 0 | 0 | 0 |
| HT059 | 0 | 0 | 1 | 0 | 0 | 0 | 0 | 0 | 0 | 0 | 0 | 0 | 0 |
| HT060 | 0 | 0 | 0 | 0 | 0 | 1 | 0 | 0 | 0 | 0 | 0 | 0 | 0 |
| HT061 | 0 | 1 | 2 | 1 | 1 | 0 | 0 | 0 | 0 | 0 | 0 | 0 | 0 |
| HT062 | 0 | 0 | 1 | 1 | 0 | 0 | 0 | 0 | 0 | 0 | 0 | 0 | 0 |
| HT063 | 0 | 0 | 0 | 0 | 0 | 0 | 0 | 0 | 0 | 0 | 0 | 1 | 0 |
| HT064 | 0 | 0 | 0 | 0 | 0 | 0 | 0 | 0 | 0 | 0 | 0 | 0 | 1 |
| HT065 | 0 | 0 | 0 | 0 | 0 | 0 | 0 | 0 | 0 | 0 | 0 | 0 | 1 |
| HT066 | 0 | 0 | 0 | 0 | 0 | 0 | 0 | 0 | 0 | 0 | 0 | 1 | 2 |
| HT067 | 0 | 0 | 0 | 0 | 0 | 0 | 0 | 0 | 0 | 0 | 0 | 0 | 1 |
| HT068 | 0 | 0 | 0 | 0 | 0 | 0 | 0 | 0 | 1 | 0 | 0 | 0 | 0 |
| HT069 | 0 | 0 | 0 | 0 | 0 | 0 | 0 | 0 | 0 | 0 | 0 | 1 | 0 |
| HT070 | 0 | 0 | 0 | 0 | 0 | 0 | 1 | 0 | 0 | 0 | 0 | 0 | 0 |
| HT071 | 0 | 0 | 0 | 0 | 0 | 0 | 3 | 0 | 0 | 2 | 0 | 0 | 0 |
| HT072 | 0 | 0 | 0 | 0 | 0 | 0 | 4 | 0 | 0 | 0 | 0 | 0 | 0 |
| HT073 | 0 | 0 | 1 | 0 | 0 | 0 | 0 | 0 | 0 | 0 | 0 | 0 | 0 |
| HT074 | 0 | 0 | 1 | 0 | 0 | 0 | 0 | 0 | 0 | 0 | 0 | 0 | 0 |
| HT075 | 0 | 1 | 0 | 0 | 0 | 0 | 0 | 0 | 0 | 0 | 0 | 0 | 0 |
| HT076 | 0 | 1 | 0 | 0 | 0 | 0 | 0 | 0 | 0 | 0 | 0 | 0 | 0 |
| HT077 | 0 | 0 | 0 | 2 | 1 | 0 | 0 | 0 | 0 | 0 | 0 | 0 | 0 |
| HT078 | 0 | 0 | 0 | 0 | 0 | 1 | 0 | 0 | 0 | 0 | 0 | 0 | 0 |
| HT079 | 0 | 0 | 0 | 1 | 0 | 0 | 0 | 0 | 0 | 0 | 0 | 0 | 0 |
| HT080 | 0 | 1 | 0 | 0 | 0 | 0 | 0 | 0 | 0 | 0 | 0 | 0 | 0 |
| HT081 | 0 | 0 | 0 | 1 | 0 | 0 | 0 | 0 | 0 | 0 | 0 | 0 | 0 |
| HT082 | 0 | 0 | 0 | 0 | 0 | 0 | 0 | 0 | 0 | 0 | 0 | 0 | 1 |
| HT083 | 0 | 0 | 0 | 1 | 0 | 0 | 0 | 0 | 0 | 0 | 0 | 0 | 0 |
| HT084 | 0 | 0 | 0 | 1 | 0 | 0 | 0 | 0 | 0 | 0 | 0 | 0 | 0 |
| HT085 | 0 | 1 | 1 | 1 | 0 | 0 | 0 | 0 | 0 | 0 | 0 | 0 | 0 |

### 13.3.3 Diversity per Site

```
strata <- as.character(BS.gt@schemes$Site)
names(strata) <- as.character(BS.gt@schemes$id)
strataG::setStrata(BS.gt) <- strata

divers.Site <- data.frame(Site = character(), pi.strataG = numeric(),
                          pi.pegas = numeric(), pi.pegas.var = numeric(),
                          HT.richness.strataG = numeric(), h.pegas = numeric(),
                          h.pegas.var = numeric())
for (Site in levels(BS.gt@schemes$Site)) {
  print(Site)
  BS.gt.sub <- BS.gt[,,Site, drop = TRUE]
  BS.bin.sub <- subset(BS.bin, names(BS.bin) %in% BS.gt.sub@data$id)
  BS.h.sub <- strataG::labelHaplotypes(BS.bin.sub, prefix = "HT",
                                        use.indels = TRUE)
  BS.dna.sub <- as.dna(BS.dna[names(BS.dna) %in% BS.gt.sub@data$id, ,
                               drop = TRUE])
  bases <- haplotypes::basecomp(BS.dna.sub)
  print(paste0("Mitogenome base composition:"))
  print(colMeans(bases))

  pi.gt <-
    strataG::nucleotideDiversity(BS.gt.sub,bases = c("a", "c", "g", "t"),
    simplify = TRUE)
  pi.peg <- pegas::nuc.div(BS.bin.sub, variance = TRUE,
                          pairwise.deletion = FALSE)
  h.peg <- pegas::hap.div(BS.bin.sub, variance = TRUE, method = "Nei")
  res <- data.frame(Site, mean(pi.gt), pi.peg[1], pi.peg[2],
                    length(unique(BS.h.sub$haps)),
                    h.peg[1], h.peg[2])
  colnames(res) <- c("Site", "pi.strataG", "pi.pegas", "pi.pegas.var",
                     "HT.richness.strataG", "h.pegas", "h.pegas.var")
  divers.Site <- rbind(divers.Site, res)
}

knitr::kable(
  divers.Site,
  caption = "Nucleotide and haplotype diversity per Site",
  # format = "html",
  digits = 6
  ) %>%
  kableExtra::kable_styling(full_width = FALSE,font_size = 5,
                            latex_options = c( "hold_position"))


dA.gt <- strataG::nucleotideDivergence(BS.gt,
                                       probs = c(0, 0.025, 0.5, 0.975, 1),
                                       model = "RAW")
knitr::kable(
  dA.gt$within[order(dA.gt$within$mean, decreasing = TRUE), -1],
  # format = "html",
  caption = "Nucleotide divergence within each Site",
  digits = 6
  ) %>%
  kableExtra::kable_styling(full_width = FALSE,font_size = 5)
knitr::kable(
  dA.gt$between[order(dA.gt$between$mean, decreasing = TRUE), -1],
```

```r
  # format = "html",
  caption = "Nucleotide divergence between each Site",
  digits = 6
  ) %>%
  kableExtra::kable_styling(full_width = FALSE, font_size = 5)
```

```r
hapcount <- pegas::haploFreq(BS.dna@sequence, fac = BS.gt@schemes$Site,
                             split = NULL, what = 1, haplo = NULL)
row.names(hapcount) <- paste0("HT", sprintf('%0.2d', 1:BS.hap@nhap))
shortnames <- c("GOC", "COR","SIL", "SAF", "RUN", "SEY", "ARS", "SRL", "TAI",
                "IND", "PNG", "AUS", "JAP","FIJ")

kableExtra::kbl(hapcount, col.names = shortnames,
  caption = "Haplotype counts per Site according to pegas",
  longtable = TRUE, booktabs = TRUE) %>%
  kableExtra::kable_styling(full_width = FALSE,font_size = 5,
                            latex_options = c("repeat_header", "hold_position"))

group.levels <- levels(BS.gi$other$ind.meta$Site)
group.names <- BS.gi$other$ind.meta$Site

HTs <- paste0("HT", sprintf('%0.3d', 1:BS.hap@nhap))
df <- data.frame(group = NULL,nind = NULL, hap = NULL, nhap = NULL,
                 hapfreq = NULL, stringsAsFactors = FALSE)
for (k in 1:length(group.levels)) {
  group.inds <- adegenet::indNames(BS.gi)[group.names == group.levels[k]]
  for (i in 1:BS.hap@nhap) {
    group <- group.levels[k]
    nind <- length(group.inds)
    hap <- HTs[i]
    nhap <- sum(group.inds %in% BS.hap@haplist[[i]])
    hapfreq <- nhap/nind
    df <- plyr::rbind.fill(df, data.frame(group = group, nind = nind, hap = hap,
                                          nhap = nhap, hapfreq = hapfreq,
                                          stringsAsFactors = FALSE))
  }
}

df$group <- factor(df$group, levels = group.levels)

haplotype.labels <- unique(df$hap)
haplotype.labels2 <- haplotype.labels[seq(1, length(haplotype.labels), by = 5)]

HT.plot <- ggplot2::ggplot(data = df) +
  ggplot2::geom_bar(ggplot2::aes(x = hap, y = nhap, fill = group),
                    stat = "identity",position = "stack") +
  ggplot2::labs(x = "Haplotypes", y = "Number of individuals") +
  ggplot2::scale_fill_manual(values = funky(15)) +
  ggplot2::scale_x_discrete(breaks = haplotype.labels2) +
  ggplot2::theme(
      axis.text = ggplot2::element_text(angle = 45, hjust = 1, size = 5),
      axis.title.x = ggplot2::element_text(size = 10),
      axis.title.y = ggplot2::element_text(size = 10),
      legend.text = ggplot2::element_text(size = 7))
print(HT.plot)
```

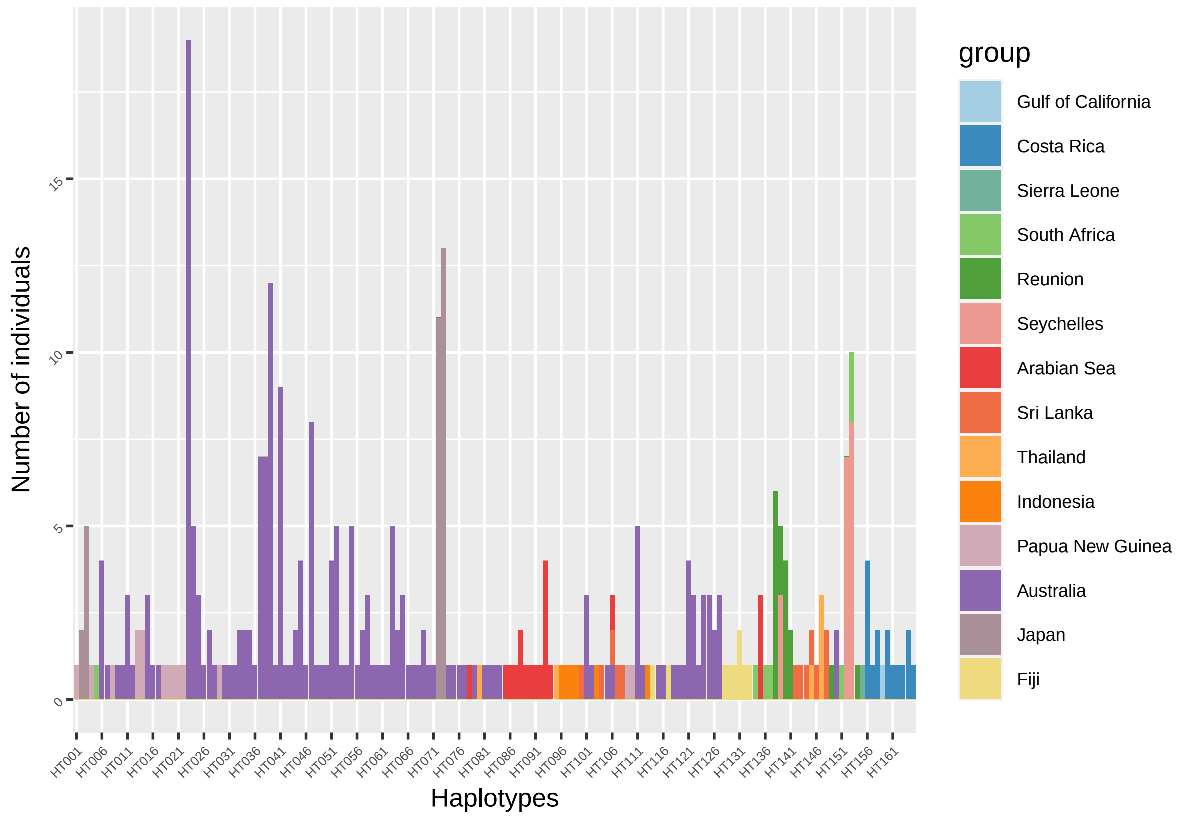


```r
ggplot2::ggsave(HT.plot, filename = "HT_barplot_per_Sites.png", width = 30,
                height = 15, units = "cm")
```

```
## [1] "Gulf of California"
## [1] "Mitogenome base composition:"
##         A         T         G         C
## 0.3151972 0.2423535 0.1307236 0.3117256
## [1] "Costa Rica"
## [1] "Mitogenome base composition:"
##         A         T         G         C
## 0.3151516 0.2424022 0.1307582 0.3116879
## [1] "Sierra Leone"
## [1] "Mitogenome base composition:"
##         A         T         G         C
## 0.3145576 0.2423680 0.1312702 0.3118041
## [1] "South Africa"
## [1] "Mitogenome base composition:"
##         A         T         G         C
## 0.3148254 0.2429858 0.1309720 0.3112168
## [1] "Reunion"
## [1] "Mitogenome base composition:"
##         A         T         G         C
## 0.3148585 0.2431229 0.1309273 0.3110912
## [1] "Seychelles"
## [1] "Mitogenome base composition:"
##         A         T         G         C
## 0.3149296 0.2427989 0.1309067 0.3113648
## [1] "Arabian Sea"
## [1] "Mitogenome base composition:"
##         A         T         G         C
```

```
## 0.3146861 0.2433287 0.1310578 0.3109275
## [1] "Sri Lanka"
## [1] "Mitogenome base composition:"
##         A         T         G         C
## 0.3147039 0.2430626 0.1310614 0.3111722
## [1] "Thailand"
## [1] "Mitogenome base composition:"
##         A         T         G         C
## 0.3147902 0.2430688 0.1310021 0.3111388
## [1] "Indonesia"
## [1] "Mitogenome base composition:"
##         A         T         G         C
## 0.3145539 0.2432308 0.1311531 0.3110621
## [1] "Papua New Guinea"
## [1] "Mitogenome base composition:"
##         A         T         G         C
## 0.3145063 0.2429284 0.1312395 0.3113257
## [1] "Australia"
## [1] "Mitogenome base composition:"
##         A         T         G         C
## 0.3145112 0.2430699 0.1312364 0.3111825
## [1] "Japan"
## [1] "Mitogenome base composition:"
##         A         T         G         C
## 0.3146686 0.2430748 0.1311079 0.3111486
## [1] "Fiji"
## [1] "Mitogenome base composition:"
##         A         T         G         C
## 0.3146038 0.2429049 0.1311531 0.3113381
```

Table 58: Nucleotide and haplotype diversity per Site

| | Site | pi.strataG | pi.pegas | pi.pegas.var | HT.richness.strataG | h.pegas | h.pegas.var |
|---|---|---|---|---|---|---|---|
| 1 | Gulf of California | NaN | NaN | NaN | 1 | NaN | NaN |
| 11 | Costa Rica | 0.000269 | 0.000269 | 0e+00 | 9 | 0.914286 | 0.002208 |
| 12 | Sierra Leone | NaN | NaN | NaN | 1 | NaN | NaN |
| 13 | South Africa | NaN | 0.001950 | 1e-06 | 6 | 0.952381 | 0.005950 |
| 14 | Reunion | 0.000289 | 0.000289 | 0e+00 | 6 | 0.808333 | 0.004289 |
| 15 | Seychelles | 0.000299 | 0.000299 | 0e+00 | 3 | 0.660131 | 0.002867 |
| 16 | Arabian Sea | 0.001792 | 0.001792 | 1e-06 | 13 | 0.941520 | 0.001170 |
| 17 | Sri Lanka | 0.003036 | 0.003037 | 3e-06 | 11 | 0.984848 | 0.000986 |
| 18 | Thailand | NaN | 0.002925 | 3e-06 | 4 | 0.800000 | 0.025926 |
| 19 | Indonesia | 0.000447 | 0.000447 | 0e+00 | 6 | 1.000000 | 0.004630 |
| 110 | Papua New Guinea | 0.000630 | 0.000631 | 0e+00 | 13 | 0.980952 | 0.000600 |
| 111 | Australia | 0.000629 | 0.000630 | 0e+00 | 85 | 0.977711 | 0.000014 |
| 112 | Japan | 0.000389 | 0.000389 | 0e+00 | 4 | 0.690323 | 0.001877 |
| 113 | Fiji | NaN | 0.001374 | 1e-06 | 8 | 0.972222 | 0.002578 |

Table 59: Nucleotide divergence within each Site

| | stratum | mean | q.0 | q.0.025 | q.0.5 | q.0.975 | q.1 |
|---|---|---|---|---|---|---|---|
| 13 | Sri Lanka | 0.003038 | 0 | 3.7e-05 | 0.005210 | 0.005569 | 0.005629 |
| 14 | Thailand | 0.002926 | 0 | 0.0e+00 | 0.005269 | 0.005428 | 0.005449 |
| 12 | South Africa | 0.001950 | 0 | 6.0e-05 | 0.001138 | 0.005150 | 0.005150 |
| 1 | Arabian Sea | 0.001793 | 0 | 0.0e+00 | 0.000240 | 0.006048 | 0.006108 |
| 4 | Fiji | 0.001374 | 0 | 0.0e+00 | 0.000240 | 0.005509 | 0.005509 |
| 8 | Papua New Guinea | 0.000631 | 0 | 0.0e+00 | 0.000778 | 0.001257 | 0.001377 |
| 2 | Australia | 0.000630 | 0 | 0.0e+00 | 0.000599 | 0.001257 | 0.005569 |
| 6 | Indonesia | 0.000447 | 0 | 0.0e+00 | 0.000240 | 0.001078 | 0.001078 |
| 7 | Japan | 0.000389 | 0 | 0.0e+00 | 0.000000 | 0.001078 | 0.001078 |
| 10 | Seychelles | 0.000299 | 0 | 0.0e+00 | 0.000000 | 0.001018 | 0.001018 |
| 9 | Reunion | 0.000289 | 0 | 0.0e+00 | 0.000120 | 0.001078 | 0.001078 |
| 3 | Costa Rica | 0.000269 | 0 | 0.0e+00 | 0.000299 | 0.000419 | 0.000419 |
| 5 | Gulf of California | NA | NA | NA | NA | NA | NA |
| 11 | Sierra Leone | NA | NA | NA | NA | NA | NA |

Table 60: Nucleotide divergence between each Site

| | strata.1 | strata.2 | dA | mean | q.0 | q.0.025 | q.0.5 | q.0.975 | q.1 |
|---|---|---|---|---|---|---|---|---|---|
| 86 | Sierra Leone | South Africa | 0.008195 | 0.009170 | 0.008982 | 0.008982 | 0.009281 | 0.009281 | 0.009281 |
| 53 | Gulf of California | South Africa | 0.008178 | 0.009153 | 0.008982 | 0.008982 | 0.009162 | 0.009383 | 0.009401 |
| 50 | Gulf of California | Reunion | 0.008965 | 0.009109 | 0.009042 | 0.009042 | 0.009162 | 0.009162 | 0.009162 |
| 78 | Reunion | Sierra Leone | 0.008965 | 0.009109 | 0.009042 | 0.009042 | 0.009162 | 0.009162 | 0.009162 |
| 34 | Costa Rica | South Africa | 0.007999 | 0.009109 | 0.008862 | 0.008862 | 0.009102 | 0.009401 | 0.009401 |
| 4 | Arabian Sea | Gulf of California | 0.008208 | 0.009105 | 0.008922 | 0.008976 | 0.009102 | 0.009254 | 0.009281 |
| 47 | Gulf of California | Indonesia | 0.008858 | 0.009082 | 0.008982 | 0.008997 | 0.009102 | 0.009102 | 0.009102 |
| 48 | Gulf of California | Japan | 0.008874 | 0.009069 | 0.009042 | 0.009042 | 0.009042 | 0.009162 | 0.009162 |
| 49 | Gulf of California | Papua New Guinea | 0.008750 | 0.009066 | 0.008922 | 0.008922 | 0.009102 | 0.009201 | 0.009222 |
| 31 | Costa Rica | Reunion | 0.008786 | 0.009065 | 0.008922 | 0.008922 | 0.009042 | 0.009162 | 0.009162 |
| 2 | Arabian Sea | Costa Rica | 0.008030 | 0.009061 | 0.008802 | 0.008868 | 0.009042 | 0.009222 | 0.009281 |
| 28 | Costa Rica | Indonesia | 0.008680 | 0.009038 | 0.008862 | 0.008876 | 0.009042 | 0.009102 | 0.009102 |
| 55 | Gulf of California | Thailand | 0.007569 | 0.009032 | 0.008922 | 0.008930 | 0.009042 | 0.009147 | 0.009162 |
| 29 | Costa Rica | Japan | 0.008696 | 0.009025 | 0.008922 | 0.008922 | 0.009042 | 0.009162 | 0.009162 |
| 10 | Arabian Sea | Sierra Leone | 0.008127 | 0.009023 | 0.008802 | 0.008856 | 0.008982 | 0.009281 | 0.009281 |
| 30 | Costa Rica | Papua New Guinea | 0.008572 | 0.009022 | 0.008802 | 0.008838 | 0.009042 | 0.009222 | 0.009222 |
| 37 | Fiji | Gulf of California | 0.008335 | 0.009022 | 0.008982 | 0.008982 | 0.009042 | 0.009042 | 0.009042 |
| 51 | Gulf of California | Seychelles | 0.008862 | 0.009012 | 0.008982 | 0.008982 | 0.008982 | 0.009162 | 0.009162 |
| 54 | Gulf of California | Sri Lanka | 0.007493 | 0.009012 | 0.008922 | 0.008922 | 0.008982 | 0.009145 | 0.009162 |
| 82 | Seychelles | Sierra Leone | 0.008862 | 0.009012 | 0.008982 | 0.008982 | 0.008982 | 0.009162 | 0.009162 |
| 88 | Sierra Leone | Thailand | 0.007529 | 0.008992 | 0.008802 | 0.008825 | 0.009042 | 0.009042 | 0.009042 |
| 36 | Costa Rica | Thailand | 0.007390 | 0.008988 | 0.008802 | 0.008816 | 0.008982 | 0.009162 | 0.009162 |
| 26 | Costa Rica | Fiji | 0.008156 | 0.008978 | 0.008862 | 0.008862 | 0.008982 | 0.009042 | 0.009042 |
| 32 | Costa Rica | Seychelles | 0.008684 | 0.008968 | 0.008862 | 0.008862 | 0.008982 | 0.009162 | 0.009162 |
| 35 | Costa Rica | Sri Lanka | 0.007315 | 0.008968 | 0.008802 | 0.008802 | 0.008982 | 0.009162 | 0.009162 |
| 16 | Australia | Gulf of California | 0.008652 | 0.008966 | 0.008743 | 0.008743 | 0.008922 | 0.009222 | 0.009281 |
| 60 | Indonesia | Sierra Leone | 0.008739 | 0.008962 | 0.008862 | 0.008877 | 0.008982 | 0.008982 | 0.008982 |
| 87 | Sierra Leone | Sri Lanka | 0.007443 | 0.008962 | 0.008802 | 0.008802 | 0.008982 | 0.009145 | 0.009162 |
| 67 | Japan | Sierra Leone | 0.008754 | 0.008949 | 0.008922 | 0.008922 | 0.008922 | 0.009042 | 0.009042 |
| 73 | Papua New Guinea | Sierra Leone | 0.008631 | 0.008946 | 0.008802 | 0.008802 | 0.008982 | 0.009081 | 0.009102 |
| 14 | Australia | Costa Rica | 0.008473 | 0.008922 | 0.008623 | 0.008683 | 0.008922 | 0.009162 | 0.009281 |
| 43 | Fiji | Sierra Leone | 0.008215 | 0.008902 | 0.008862 | 0.008862 | 0.008922 | 0.008922 | 0.008922 |
| 22 | Australia | Sierra Leone | 0.008528 | 0.008843 | 0.008623 | 0.008626 | 0.008862 | 0.009102 | 0.009162 |
| 52 | Gulf of California | Sierra Leone | 0.006347 | 0.006347 | 0.006347 | 0.006347 | 0.006347 | 0.006347 | 0.006347 |
| 33 | Costa Rica | Sierra Leone | 0.006169 | 0.006303 | 0.006228 | 0.006228 | 0.006287 | 0.006347 | 0.006347 |
| 65 | Japan | Reunion | 0.005288 | 0.005628 | 0.005269 | 0.005269 | 0.005629 | 0.005988 | 0.005988 |
| 71 | Papua New Guinea | Reunion | 0.005129 | 0.005589 | 0.005210 | 0.005328 | 0.005569 | 0.005988 | 0.005988 |
| 58 | Indonesia | Reunion | 0.005198 | 0.005566 | 0.005210 | 0.005254 | 0.005569 | 0.005808 | 0.005808 |
| 20 | Australia | Reunion | 0.005076 | 0.005535 | 0.000240 | 0.005210 | 0.005569 | 0.005868 | 0.005928 |
| 66 | Japan | Seychelles | 0.005016 | 0.005361 | 0.005210 | 0.005210 | 0.005210 | 0.005988 | 0.005988 |
| 72 | Papua New Guinea | Seychelles | 0.004874 | 0.005339 | 0.005150 | 0.005150 | 0.005269 | 0.005689 | 0.005988 |
| 59 | Indonesia | Seychelles | 0.004926 | 0.005299 | 0.005150 | 0.005150 | 0.005269 | 0.005728 | 0.005808 |
| 21 | Australia | Seychelles | 0.004802 | 0.005267 | 0.000180 | 0.005030 | 0.005269 | 0.005749 | 0.005928 |
| 41 | Fiji | Reunion | 0.004197 | 0.005029 | 0.000359 | 0.000958 | 0.005509 | 0.005629 | 0.005629 |
| 68 | Japan | South Africa | 0.003843 | 0.005013 | 0.001557 | 0.001557 | 0.005569 | 0.006108 | 0.006108 |
| 61 | Indonesia | South Africa | 0.003760 | 0.004959 | 0.001497 | 0.001617 | 0.005389 | 0.005928 | 0.005928 |
| 74 | Papua New Guinea | South Africa | 0.003614 | 0.004905 | 0.000659 | 0.000778 | 0.005389 | 0.006000 | 0.006108 |
| 23 | Australia | South Africa | 0.003602 | 0.004892 | 0.000180 | 0.001198 | 0.005389 | 0.005988 | 0.006048 |
| 8 | Arabian Sea | Reunion | 0.003841 | 0.004882 | 0.000240 | 0.000240 | 0.005689 | 0.005954 | 0.005988 |
| 42 | Fiji | Seychelles | 0.003973 | 0.004809 | 0.000299 | 0.000299 | 0.005329 | 0.005629 | 0.005629 |
| 9 | Arabian Sea | Seychelles | 0.003704 | 0.004750 | 0.000359 | 0.000768 | 0.005389 | 0.005837 | 0.005988 |
| 44 | Fiji | South Africa | 0.002900 | 0.004562 | 0.000299 | 0.000365 | 0.005389 | 0.005749 | 0.005749 |
| 11 | Arabian Sea | South Africa | 0.002625 | 0.004496 | 0.000120 | 0.000120 | 0.005449 | 0.006048 | 0.006108 |
| 76 | Papua New Guinea | Thailand | 0.002145 | 0.003923 | 0.000240 | 0.000359 | 0.005329 | 0.005735 | 0.005749 |
| 25 | Australia | Thailand | 0.002064 | 0.003842 | 0.000180 | 0.000419 | 0.005329 | 0.005569 | 0.005629 |
| 70 | Japan | Thailand | 0.002138 | 0.003795 | 0.000359 | 0.000359 | 0.005269 | 0.005749 | 0.005749 |
| 63 | Indonesia | Thailand | 0.002059 | 0.003746 | 0.000299 | 0.000404 | 0.005210 | 0.005569 | 0.005569 |
| 46 | Fiji | Thailand | 0.001570 | 0.003720 | 0.000060 | 0.000359 | 0.005419 | 0.005509 | 0.005509 |
| 13 | Arabian Sea | Thailand | 0.001218 | 0.003578 | 0.000120 | 0.000120 | 0.005389 | 0.005769 | 0.005868 |
| 75 | Papua New Guinea | Sri Lanka | 0.001691 | 0.003525 | 0.000240 | 0.000299 | 0.005329 | 0.005689 | 0.005868 |
| 24 | Australia | Sri Lanka | 0.001608 | 0.003442 | 0.000000 | 0.000299 | 0.005269 | 0.005629 | 0.005808 |
| 45 | Fiji | Sri Lanka | 0.001195 | 0.003401 | 0.000060 | 0.000340 | 0.005269 | 0.005509 | 0.005509 |
| 69 | Japan | Sri Lanka | 0.001680 | 0.003394 | 0.000359 | 0.000359 | 0.005210 | 0.005808 | 0.005868 |
| 12 | Arabian Sea | Sri Lanka | 0.000915 | 0.003330 | 0.000000 | 0.000359 | 0.005329 | 0.005749 | 0.005808 |
| 62 | Indonesia | Sri Lanka | 0.001544 | 0.003286 | 0.000120 | 0.000166 | 0.005180 | 0.005582 | 0.005689 |
| 89 | South Africa | Sri Lanka | 0.000372 | 0.002866 | 0.000240 | 0.000240 | 0.001317 | 0.005864 | 0.005868 |
| 80 | Reunion | Sri Lanka | 0.001188 | 0.002852 | 0.000299 | 0.000406 | 0.001018 | 0.005749 | 0.005749 |
| 91 | Sri Lanka | Thailand | -0.000246 | 0.002736 | 0.000000 | 0.000060 | 0.000958 | 0.005509 | 0.005569 |
| 90 | South Africa | Thailand | 0.000109 | 0.002548 | 0.000240 | 0.000241 | 0.001198 | 0.005868 | 0.005868 |
| 84 | Seychelles | Sri Lanka | 0.000860 | 0.002528 | 0.000240 | 0.000240 | 0.000928 | 0.005689 | 0.005749 |
| 81 | Reunion | Thailand | 0.000885 | 0.002493 | 0.000299 | 0.000359 | 0.001078 | 0.005749 | 0.005749 |
| 3 | Arabian Sea | Fiji | 0.000483 | 0.002066 | 0.000719 | 0.000838 | 0.001018 | 0.005749 | 0.005749 |
| 85 | Seychelles | Thailand | 0.000443 | 0.002056 | 0.000240 | 0.000240 | 0.000299 | 0.005668 | 0.005749 |
| 7 | Arabian Sea | Papua New Guinea | 0.000524 | 0.001735 | 0.000240 | 0.000359 | 0.001078 | 0.005808 | 0.006108 |
| 1 | Arabian Sea | Australia | 0.000441 | 0.001652 | 0.000000 | 0.000359 | 0.000898 | 0.005868 | 0.006048 |
| 6 | Arabian Sea | Japan | 0.000391 | 0.001482 | 0.000359 | 0.000359 | 0.000539 | 0.006108 | 0.006108 |
| 39 | Fiji | Japan | 0.000596 | 0.001477 | 0.000838 | 0.000838 | 0.000958 | 0.005407 | 0.005749 |
| 40 | Fiji | Papua New Guinea | 0.000446 | 0.001448 | 0.000599 | 0.000659 | 0.000958 | 0.005449 | 0.005749 |
| 5 | Arabian Sea | Indonesia | 0.000316 | 0.001436 | 0.000120 | 0.000240 | 0.000599 | 0.005731 | 0.005928 |
| 38 | Fiji | Indonesia | 0.000511 | 0.001422 | 0.000719 | 0.000719 | 0.000958 | 0.005449 | 0.005569 |
| 15 | Australia | Fiji | 0.000337 | 0.001339 | 0.000180 | 0.000240 | 0.000838 | 0.005509 | 0.005689 |
| 79 | Reunion | South Africa | 0.000134 | 0.001254 | 0.000060 | 0.000153 | 0.000898 | 0.005030 | 0.005030 |
| 83 | Seychelles | South Africa | 0.000063 | 0.001188 | 0.000000 | 0.000000 | 0.001078 | 0.004611 | 0.005030 |
| 57 | Indonesia | Papua New Guinea | 0.000438 | 0.000977 | 0.000299 | 0.000419 | 0.001078 | 0.001198 | 0.001257 |
| 64 | Japan | Papua New Guinea | 0.000450 | 0.000960 | 0.000359 | 0.000359 | 0.001018 | 0.001257 | 0.001377 |
| 18 | Australia | Japan | 0.000401 | 0.000910 | 0.000299 | 0.000359 | 0.000898 | 0.001257 | 0.005629 |
| 17 | Australia | Indonesia | 0.000342 | 0.000880 | 0.000060 | 0.000265 | 0.000898 | 0.001257 | 0.005449 |
| 19 | Australia | Papua New Guinea | 0.000176 | 0.000807 | 0.000120 | 0.000240 | 0.000719 | 0.001198 | 0.005629 |
| 77 | Reunion | Seychelles | 0.000453 | 0.000747 | 0.000000 | 0.000000 | 0.000898 | 0.001018 | 0.001078 |
| 56 | Indonesia | Japan | 0.000271 | 0.000690 | 0.000419 | 0.000419 | 0.000539 | 0.001257 | 0.001257 |
| 27 | Costa Rica | Gulf of California | 0.000173 | 0.000307 | 0.000240 | 0.000240 | 0.000299 | 0.000419 | 0.000419 |

Table 61: Haplotype counts per Site according to pegas

| | GOC | COR | SIL | SAF | RUN | SEY | ARS | SRL | TAI | IND | PNG | AUS | JAP | FIJ |
|---|---|---|---|---|---|---|---|---|---|---|---|---|---|---|
| HT01 | 0 | 0 | 0 | 1 | 0 | 0 | 0 | 0 | 0 | 0 | 0 | 0 | 0 | 0 |
| HT02 | 0 | 0 | 0 | 1 | 0 | 0 | 0 | 0 | 0 | 0 | 0 | 0 | 0 | 0 |
| HT03 | 0 | 0 | 0 | 1 | 0 | 0 | 0 | 0 | 0 | 0 | 0 | 0 | 0 | 0 |
| HT04 | 0 | 0 | 0 | 0 | 1 | 0 | 0 | 0 | 0 | 0 | 0 | 0 | 0 | 0 |
| HT05 | 0 | 0 | 0 | 0 | 0 | 0 | 0 | 0 | 3 | 0 | 0 | 0 | 0 | 0 |
| HT06 | 0 | 0 | 0 | 0 | 0 | 0 | 0 | 2 | 0 | 0 | 0 | 0 | 0 | 0 |
| HT07 | 0 | 0 | 0 | 0 | 0 | 0 | 0 | 1 | 0 | 0 | 0 | 0 | 0 | 0 |
| HT08 | 0 | 0 | 0 | 0 | 1 | 0 | 0 | 0 | 0 | 0 | 0 | 0 | 0 | 0 |
| HT09 | 0 | 0 | 0 | 1 | 0 | 0 | 0 | 0 | 0 | 0 | 0 | 0 | 0 | 0 |
| HT10 | 0 | 0 | 0 | 2 | 0 | 8 | 0 | 0 | 0 | 0 | 0 | 0 | 0 | 0 |
| HT11 | 0 | 0 | 0 | 0 | 0 | 7 | 0 | 0 | 0 | 0 | 0 | 0 | 0 | 0 |
| HT12 | 0 | 0 | 0 | 0 | 0 | 0 | 0 | 0 | 0 | 0 | 0 | 2 | 0 | 0 |
| HT13 | 0 | 0 | 0 | 0 | 0 | 0 | 0 | 1 | 0 | 0 | 0 | 0 | 0 | 0 |
| HT14 | 0 | 0 | 0 | 0 | 0 | 0 | 0 | 1 | 0 | 0 | 0 | 0 | 0 | 0 |
| HT15 | 0 | 0 | 0 | 0 | 6 | 0 | 0 | 0 | 0 | 0 | 0 | 0 | 0 | 0 |
| HT16 | 0 | 0 | 0 | 0 | 2 | 3 | 0 | 0 | 0 | 0 | 0 | 0 | 0 | 0 |
| HT17 | 0 | 0 | 0 | 0 | 2 | 0 | 0 | 0 | 0 | 0 | 0 | 0 | 0 | 0 |
| HT18 | 0 | 0 | 0 | 0 | 4 | 0 | 0 | 0 | 0 | 0 | 0 | 0 | 0 | 0 |
| HT19 | 0 | 0 | 0 | 0 | 0 | 0 | 0 | 0 | 0 | 0 | 0 | 0 | 0 | 1 |
| HT20 | 0 | 0 | 0 | 0 | 0 | 0 | 0 | 1 | 0 | 0 | 0 | 0 | 0 | 0 |
| HT21 | 0 | 0 | 0 | 0 | 0 | 0 | 0 | 1 | 1 | 0 | 0 | 0 | 0 | 0 |
| HT22 | 0 | 0 | 0 | 0 | 0 | 0 | 3 | 0 | 0 | 0 | 0 | 0 | 0 | 0 |
| HT23 | 0 | 0 | 0 | 0 | 0 | 0 | 0 | 0 | 0 | 0 | 0 | 3 | 0 | 0 |
| HT24 | 0 | 0 | 0 | 0 | 0 | 0 | 0 | 0 | 0 | 0 | 0 | 4 | 0 | 0 |
| HT25 | 0 | 0 | 0 | 0 | 0 | 0 | 0 | 0 | 0 | 0 | 0 | 1 | 0 | 0 |
| HT26 | 0 | 0 | 0 | 0 | 0 | 0 | 0 | 0 | 0 | 0 | 0 | 1 | 0 | 0 |
| HT27 | 0 | 0 | 0 | 0 | 0 | 0 | 0 | 0 | 0 | 0 | 0 | 1 | 0 | 0 |
| HT28 | 0 | 0 | 0 | 0 | 0 | 0 | 0 | 0 | 0 | 1 | 0 | 0 | 0 | 0 |
| HT29 | 0 | 0 | 0 | 0 | 0 | 0 | 0 | 0 | 0 | 0 | 0 | 2 | 0 | 0 |
| HT30 | 0 | 0 | 0 | 0 | 0 | 0 | 0 | 0 | 0 | 0 | 0 | 5 | 0 | 0 |
| HT31 | 0 | 0 | 0 | 0 | 0 | 0 | 0 | 0 | 0 | 0 | 0 | 3 | 0 | 0 |
| HT32 | 0 | 0 | 0 | 0 | 0 | 0 | 0 | 0 | 0 | 0 | 1 | 0 | 0 | 0 |
| HT33 | 0 | 0 | 0 | 0 | 0 | 0 | 0 | 0 | 0 | 0 | 0 | 1 | 0 | 0 |
| HT34 | 0 | 0 | 0 | 0 | 0 | 0 | 0 | 0 | 0 | 0 | 0 | 7 | 0 | 0 |
| HT35 | 0 | 0 | 0 | 0 | 0 | 0 | 0 | 0 | 0 | 0 | 0 | 1 | 0 | 0 |
| HT36 | 0 | 0 | 0 | 0 | 0 | 0 | 0 | 0 | 0 | 0 | 0 | 3 | 0 | 0 |
| HT37 | 0 | 0 | 0 | 0 | 0 | 0 | 0 | 0 | 0 | 0 | 0 | 2 | 0 | 0 |
| HT38 | 0 | 0 | 0 | 0 | 0 | 0 | 0 | 0 | 0 | 0 | 0 | 2 | 0 | 0 |
| HT39 | 0 | 0 | 0 | 0 | 0 | 0 | 0 | 0 | 0 | 0 | 0 | 2 | 0 | 0 |
| HT40 | 0 | 0 | 0 | 0 | 0 | 0 | 0 | 0 | 0 | 0 | 0 | 2 | 0 | 0 |
| HT41 | 0 | 0 | 0 | 0 | 0 | 0 | 0 | 0 | 0 | 0 | 0 | 1 | 0 | 0 |
| HT42 | 0 | 0 | 0 | 0 | 0 | 0 | 0 | 0 | 0 | 0 | 0 | 1 | 0 | 0 |
| HT43 | 0 | 0 | 0 | 0 | 0 | 0 | 0 | 0 | 0 | 0 | 0 | 1 | 0 | 0 |
| HT44 | 0 | 0 | 0 | 0 | 0 | 0 | 0 | 0 | 0 | 0 | 0 | 5 | 0 | 0 |
| HT45 | 0 | 0 | 0 | 0 | 0 | 0 | 0 | 0 | 0 | 0 | 0 | 4 | 0 | 0 |
| HT46 | 0 | 0 | 0 | 0 | 0 | 0 | 0 | 0 | 0 | 0 | 0 | 9 | 0 | 0 |
| HT47 | 0 | 0 | 0 | 0 | 0 | 0 | 0 | 0 | 0 | 0 | 0 | 1 | 0 | 0 |
| HT48 | 0 | 0 | 0 | 0 | 0 | 0 | 0 | 0 | 0 | 0 | 0 | 1 | 0 | 0 |
| HT49 | 0 | 0 | 0 | 0 | 0 | 0 | 0 | 0 | 0 | 0 | 0 | 1 | 0 | 0 |
| HT50 | 0 | 0 | 0 | 0 | 0 | 0 | 0 | 0 | 0 | 0 | 0 | 12 | 0 | 0 |
| HT51 | 0 | 0 | 0 | 0 | 0 | 0 | 0 | 0 | 0 | 0 | 0 | 7 | 0 | 0 |
| HT52 | 0 | 0 | 0 | 0 | 0 | 0 | 0 | 0 | 0 | 0 | 0 | 1 | 0 | 0 |
| HT53 | 0 | 0 | 0 | 0 | 0 | 0 | 0 | 0 | 0 | 0 | 0 | 1 | 0 | 0 |
| HT54 | 0 | 0 | 0 | 0 | 0 | 0 | 0 | 0 | 0 | 0 | 0 | 1 | 0 | 0 |
| HT55 | 0 | 0 | 0 | 0 | 0 | 0 | 0 | 0 | 0 | 0 | 0 | 19 | 0 | 0 |
| HT56 | 0 | 0 | 0 | 0 | 0 | 0 | 0 | 0 | 0 | 0 | 0 | 8 | 0 | 0 |
| HT57 | 0 | 0 | 0 | 0 | 0 | 0 | 0 | 0 | 0 | 0 | 0 | 1 | 0 | 0 |
| HT58 | 0 | 0 | 0 | 0 | 0 | 0 | 0 | 0 | 0 | 0 | 0 | 1 | 0 | 0 |
| HT59 | 0 | 0 | 0 | 0 | 0 | 0 | 0 | 0 | 0 | 0 | 0 | 1 | 0 | 0 |
| HT60 | 0 | 0 | 0 | 0 | 0 | 0 | 0 | 0 | 0 | 0 | 0 | 2 | 0 | 0 |
| HT61 | 0 | 0 | 0 | 0 | 0 | 0 | 0 | 0 | 0 | 0 | 0 | 1 | 0 | 0 |
| HT62 | 0 | 0 | 0 | 0 | 0 | 0 | 0 | 0 | 0 | 0 | 0 | 1 | 0 | 0 |
| HT63 | 0 | 0 | 0 | 0 | 0 | 0 | 0 | 0 | 0 | 0 | 0 | 1 | 0 | 0 |
| HT64 | 0 | 0 | 0 | 0 | 0 | 0 | 1 | 0 | 0 | 0 | 0 | 0 | 0 | 0 |
| HT65 | 0 | 0 | 0 | 0 | 0 | 0 | 1 | 0 | 0 | 0 | 0 | 0 | 0 | 0 |
| HT66 | 0 | 0 | 0 | 0 | 0 | 0 | 0 | 0 | 0 | 0 | 0 | 1 | 0 | 0 |
| HT67 | 0 | 0 | 0 | 0 | 0 | 0 | 1 | 0 | 0 | 0 | 0 | 0 | 0 | 0 |
| HT68 | 0 | 0 | 0 | 0 | 0 | 0 | 0 | 0 | 0 | 0 | 0 | 3 | 0 | 0 |
| HT69 | 0 | 0 | 0 | 0 | 0 | 0 | 2 | 0 | 0 | 0 | 0 | 0 | 0 | 0 |
| HT70 | 0 | 0 | 0 | 0 | 0 | 0 | 1 | 0 | 0 | 0 | 0 | 0 | 0 | 0 |
| HT71 | 0 | 0 | 0 | 0 | 0 | 0 | 1 | 0 | 0 | 0 | 0 | 0 | 0 | 0 |
| HT72 | 0 | 0 | 0 | 0 | 0 | 0 | 1 | 0 | 0 | 0 | 0 | 0 | 0 | 0 |
| HT73 | 0 | 0 | 0 | 0 | 0 | 0 | 0 | 0 | 1 | 0 | 0 | 0 | 0 | 0 |
| HT74 | 0 | 0 | 0 | 0 | 0 | 0 | 0 | 0 | 0 | 0 | 1 | 0 | 0 | 0 |
| HT75 | 0 | 0 | 0 | 0 | 0 | 0 | 0 | 0 | 0 | 0 | 1 | 0 | 0 | 0 |
| HT76 | 0 | 0 | 0 | 0 | 0 | 0 | 0 | 0 | 0 | 0 | 0 | 1 | 0 | 0 |
| HT77 | 0 | 0 | 0 | 0 | 0 | 0 | 0 | 0 | 0 | 0 | 0 | 5 | 0 | 0 |
| HT78 | 0 | 0 | 0 | 0 | 0 | 0 | 1 | 0 | 0 | 0 | 0 | 0 | 0 | 0 |
| HT79 | 0 | 0 | 0 | 0 | 0 | 0 | 4 | 0 | 0 | 0 | 0 | 0 | 0 | 0 |
| HT80 | 0 | 0 | 0 | 0 | 0 | 0 | 1 | 0 | 0 | 0 | 0 | 0 | 0 | 0 |
| HT81 | 0 | 0 | 0 | 0 | 0 | 0 | 0 | 0 | 0 | 0 | 0 | 1 | 0 | 0 |
| HT82 | 0 | 0 | 0 | 0 | 0 | 0 | 0 | 0 | 0 | 0 | 0 | 1 | 0 | 0 |
| HT83 | 0 | 0 | 0 | 0 | 0 | 0 | 1 | 0 | 0 | 0 | 0 | 0 | 0 | 0 |
| HT84 | 0 | 0 | 0 | 0 | 0 | 0 | 0 | 0 | 0 | 0 | 0 | 1 | 0 | 0 |

Table 61: Haplotype counts per Site according to pegas *(continued)*

| | GOC | COR | SIL | SAF | RUN | SEY | ARS | SRL | TAI | IND | PNG | AUS | JAP | FIJ |
|---|---|---|---|---|---|---|---|---|---|---|---|---|---|---|
| HT85 | 0 | 0 | 0 | 0 | 0 | 0 | 0 | 0 | 0 | 0 | 0 | 3 | 0 | 0 |
| HT86 | 0 | 0 | 0 | 0 | 0 | 0 | 0 | 0 | 0 | 0 | 0 | 3 | 0 | 0 |
| HT87 | 0 | 0 | 0 | 0 | 0 | 0 | 0 | 0 | 0 | 0 | 0 | 2 | 0 | 0 |
| HT88 | 0 | 0 | 0 | 0 | 0 | 0 | 0 | 0 | 0 | 0 | 0 | 1 | 0 | 0 |
| HT89 | 0 | 0 | 0 | 0 | 0 | 0 | 0 | 0 | 0 | 0 | 0 | 0 | 0 | 1 |
| HT90 | 0 | 0 | 0 | 0 | 0 | 0 | 0 | 0 | 0 | 0 | 0 | 0 | 0 | 1 |
| HT91 | 0 | 0 | 0 | 0 | 0 | 0 | 0 | 0 | 0 | 0 | 0 | 0 | 0 | 1 |
| HT92 | 0 | 0 | 0 | 0 | 0 | 0 | 0 | 0 | 0 | 0 | 0 | 1 | 0 | 0 |
| HT93 | 0 | 0 | 0 | 0 | 0 | 0 | 0 | 0 | 0 | 0 | 0 | 4 | 0 | 0 |
| HT94 | 0 | 0 | 0 | 0 | 0 | 0 | 0 | 0 | 0 | 0 | 0 | 3 | 0 | 0 |
| HT95 | 0 | 0 | 0 | 0 | 0 | 0 | 0 | 0 | 0 | 0 | 0 | 1 | 0 | 0 |
| HT96 | 0 | 0 | 0 | 0 | 0 | 0 | 0 | 0 | 0 | 0 | 0 | 1 | 0 | 0 |
| HT97 | 0 | 0 | 0 | 0 | 0 | 0 | 0 | 0 | 0 | 0 | 0 | 0 | 0 | 1 |
| HT98 | 0 | 0 | 0 | 0 | 0 | 0 | 0 | 0 | 0 | 0 | 0 | 0 | 0 | 2 |
| HT99 | 0 | 0 | 0 | 0 | 0 | 0 | 0 | 0 | 0 | 0 | 0 | 0 | 0 | 1 |
| HT100 | 0 | 0 | 0 | 0 | 0 | 0 | 0 | 0 | 0 | 0 | 0 | 0 | 0 | 1 |
| HT101 | 0 | 0 | 0 | 0 | 0 | 0 | 0 | 0 | 0 | 0 | 0 | 1 | 0 | 0 |
| HT102 | 0 | 0 | 0 | 0 | 0 | 0 | 0 | 0 | 0 | 0 | 0 | 2 | 0 | 0 |
| HT103 | 0 | 0 | 0 | 0 | 0 | 0 | 0 | 0 | 0 | 0 | 0 | 1 | 0 | 0 |
| HT104 | 0 | 0 | 0 | 0 | 0 | 0 | 0 | 0 | 0 | 0 | 0 | 1 | 0 | 0 |
| HT105 | 0 | 0 | 0 | 0 | 0 | 0 | 0 | 0 | 0 | 0 | 0 | 5 | 0 | 0 |
| HT106 | 0 | 0 | 0 | 0 | 0 | 0 | 0 | 0 | 0 | 0 | 0 | 2 | 0 | 0 |
| HT107 | 0 | 0 | 0 | 0 | 0 | 0 | 0 | 0 | 0 | 0 | 0 | 1 | 0 | 0 |
| HT108 | 0 | 0 | 0 | 1 | 0 | 0 | 0 | 0 | 0 | 0 | 0 | 0 | 0 | 0 |
| HT109 | 0 | 0 | 0 | 0 | 0 | 0 | 0 | 0 | 0 | 0 | 0 | 1 | 0 | 0 |
| HT110 | 0 | 0 | 0 | 0 | 0 | 0 | 0 | 0 | 0 | 0 | 1 | 0 | 0 | 0 |
| HT111 | 0 | 0 | 0 | 0 | 0 | 0 | 0 | 0 | 0 | 0 | 1 | 0 | 0 | 0 |
| HT112 | 0 | 0 | 0 | 0 | 0 | 0 | 0 | 0 | 0 | 0 | 0 | 1 | 0 | 0 |
| HT113 | 0 | 0 | 0 | 0 | 0 | 0 | 0 | 0 | 0 | 0 | 1 | 0 | 0 | 0 |
| HT114 | 0 | 0 | 0 | 0 | 0 | 0 | 0 | 0 | 0 | 0 | 1 | 0 | 0 | 0 |
| HT115 | 0 | 0 | 0 | 0 | 0 | 0 | 0 | 0 | 0 | 0 | 2 | 0 | 0 | 0 |
| HT116 | 0 | 0 | 0 | 0 | 0 | 0 | 0 | 0 | 0 | 0 | 2 | 0 | 0 | 0 |
| HT117 | 0 | 0 | 0 | 0 | 0 | 0 | 0 | 0 | 0 | 0 | 0 | 3 | 0 | 0 |
| HT118 | 0 | 0 | 0 | 0 | 0 | 0 | 0 | 0 | 0 | 0 | 0 | 1 | 0 | 0 |
| HT119 | 0 | 0 | 0 | 0 | 0 | 0 | 0 | 0 | 0 | 0 | 1 | 0 | 0 | 0 |
| HT120 | 0 | 0 | 0 | 0 | 0 | 0 | 0 | 0 | 0 | 0 | 0 | 1 | 0 | 0 |
| HT121 | 0 | 0 | 0 | 0 | 0 | 0 | 0 | 0 | 0 | 0 | 0 | 1 | 0 | 0 |
| HT122 | 0 | 0 | 0 | 0 | 0 | 0 | 0 | 0 | 0 | 0 | 1 | 0 | 0 | 0 |
| HT123 | 0 | 0 | 0 | 0 | 0 | 0 | 0 | 0 | 0 | 0 | 1 | 0 | 0 | 0 |
| HT124 | 0 | 0 | 0 | 0 | 0 | 0 | 0 | 0 | 0 | 0 | 0 | 0 | 5 | 0 |
| HT125 | 0 | 0 | 0 | 0 | 0 | 0 | 0 | 0 | 0 | 0 | 0 | 0 | 2 | 0 |
| HT126 | 0 | 0 | 0 | 0 | 0 | 0 | 0 | 0 | 0 | 0 | 0 | 1 | 0 | 0 |
| HT127 | 0 | 0 | 0 | 0 | 0 | 0 | 0 | 0 | 0 | 0 | 0 | 5 | 0 | 0 |
| HT128 | 0 | 0 | 0 | 0 | 0 | 0 | 0 | 0 | 0 | 0 | 0 | 4 | 0 | 0 |
| HT129 | 0 | 0 | 0 | 0 | 0 | 0 | 0 | 0 | 0 | 0 | 0 | 1 | 0 | 0 |
| HT130 | 0 | 0 | 0 | 0 | 0 | 0 | 0 | 0 | 0 | 0 | 0 | 1 | 0 | 0 |
| HT131 | 0 | 0 | 0 | 0 | 0 | 0 | 0 | 0 | 0 | 1 | 0 | 0 | 0 | 0 |
| HT132 | 0 | 0 | 0 | 0 | 0 | 0 | 0 | 0 | 0 | 1 | 0 | 0 | 0 | 0 |
| HT133 | 0 | 0 | 0 | 0 | 0 | 0 | 0 | 1 | 0 | 0 | 0 | 0 | 0 | 0 |
| HT134 | 0 | 0 | 0 | 0 | 0 | 0 | 0 | 0 | 0 | 0 | 0 | 1 | 0 | 0 |
| HT135 | 0 | 0 | 0 | 0 | 0 | 0 | 0 | 0 | 0 | 0 | 0 | 1 | 0 | 0 |
| HT136 | 0 | 0 | 0 | 0 | 0 | 0 | 0 | 0 | 0 | 0 | 0 | 3 | 0 | 0 |
| HT137 | 0 | 0 | 0 | 0 | 0 | 0 | 1 | 1 | 0 | 0 | 0 | 1 | 0 | 0 |
| HT138 | 0 | 0 | 0 | 0 | 0 | 0 | 0 | 1 | 0 | 0 | 0 | 0 | 0 | 0 |
| HT139 | 0 | 0 | 0 | 0 | 0 | 0 | 0 | 0 | 0 | 0 | 0 | 1 | 0 | 0 |
| HT140 | 0 | 0 | 0 | 0 | 0 | 0 | 0 | 1 | 0 | 0 | 0 | 0 | 0 | 0 |
| HT141 | 0 | 0 | 0 | 0 | 0 | 0 | 0 | 1 | 0 | 0 | 0 | 0 | 0 | 0 |
| HT142 | 0 | 0 | 0 | 0 | 0 | 0 | 0 | 0 | 0 | 0 | 0 | 1 | 0 | 0 |
| HT143 | 0 | 0 | 0 | 0 | 0 | 0 | 0 | 0 | 0 | 1 | 0 | 0 | 0 | 0 |
| HT144 | 0 | 0 | 0 | 0 | 0 | 0 | 0 | 0 | 0 | 1 | 0 | 0 | 0 | 0 |
| HT145 | 0 | 0 | 0 | 0 | 0 | 0 | 0 | 0 | 0 | 1 | 0 | 0 | 0 | 0 |
| HT146 | 0 | 0 | 0 | 0 | 0 | 0 | 0 | 0 | 0 | 0 | 0 | 1 | 0 | 0 |
| HT147 | 0 | 0 | 0 | 0 | 0 | 0 | 0 | 0 | 0 | 0 | 0 | 1 | 0 | 0 |
| HT148 | 0 | 0 | 0 | 0 | 0 | 0 | 0 | 0 | 0 | 0 | 0 | 1 | 0 | 0 |
| HT149 | 0 | 0 | 0 | 0 | 0 | 0 | 0 | 0 | 0 | 0 | 0 | 1 | 0 | 0 |
| HT150 | 0 | 0 | 0 | 0 | 0 | 0 | 0 | 0 | 0 | 0 | 0 | 3 | 0 | 0 |
| HT151 | 0 | 0 | 0 | 0 | 0 | 0 | 0 | 0 | 0 | 0 | 1 | 0 | 0 | 0 |
| HT152 | 0 | 0 | 0 | 0 | 0 | 0 | 0 | 0 | 0 | 0 | 0 | 0 | 11 | 0 |
| HT153 | 0 | 0 | 0 | 0 | 0 | 0 | 0 | 0 | 0 | 0 | 0 | 0 | 13 | 0 |
| HT154 | 0 | 0 | 0 | 0 | 0 | 0 | 0 | 0 | 1 | 0 | 0 | 0 | 0 | 0 |
| HT155 | 0 | 4 | 0 | 0 | 0 | 0 | 0 | 0 | 0 | 0 | 0 | 0 | 0 | 0 |
| HT156 | 0 | 2 | 0 | 0 | 0 | 0 | 0 | 0 | 0 | 0 | 0 | 0 | 0 | 0 |
| HT157 | 0 | 1 | 0 | 0 | 0 | 0 | 0 | 0 | 0 | 0 | 0 | 0 | 0 | 0 |
| HT158 | 0 | 2 | 0 | 0 | 0 | 0 | 0 | 0 | 0 | 0 | 0 | 0 | 0 | 0 |
| HT159 | 0 | 1 | 0 | 0 | 0 | 0 | 0 | 0 | 0 | 0 | 0 | 0 | 0 | 0 |
| HT160 | 0 | 1 | 0 | 0 | 0 | 0 | 0 | 0 | 0 | 0 | 0 | 0 | 0 | 0 |
| HT161 | 0 | 1 | 0 | 0 | 0 | 0 | 0 | 0 | 0 | 0 | 0 | 0 | 0 | 0 |
| HT162 | 1 | 0 | 0 | 0 | 0 | 0 | 0 | 0 | 0 | 0 | 0 | 0 | 0 | 0 |
| HT163 | 0 | 2 | 0 | 0 | 0 | 0 | 0 | 0 | 0 | 0 | 0 | 0 | 0 | 0 |
| HT164 | 0 | 1 | 0 | 0 | 0 | 0 | 0 | 0 | 0 | 0 | 0 | 0 | 0 | 0 |
| HT165 | 0 | 0 | 1 | 0 | 0 | 0 | 0 | 0 | 0 | 0 | 0 | 0 | 0 | 0 |

### 13.3.4 Diversity per region

```r
strata <- as.character(BS.gt@schemes$Region)
strata[strata %in% c("W-IO", "N-IO", "E-IO", "W-PAC")] <- "IWP"
names(strata) <- as.character(BS.gt@schemes$id)
strataG::setStrata(BS.gt) <- strata

divers.region <- data.frame(Region = character(), pi.strataG = numeric(),
                            pi.pegas = numeric(), pi.pegas.var = numeric(),
                            HT.richness.strataG = numeric(), h.pegas = numeric(),
                            h.pegas.var = numeric())
strat.levels <- c("E-PAC", "E-ATL", "IWP","Japan", "Fiji")
for (Region in strat.levels) {
  print(Region)
  BS.gt.sub <- BS.gt[,,Region, drop = TRUE]
  BS.bin.sub <- subset(BS.bin, names(BS.bin) %in% BS.gt.sub@data$id)
  BS.h.sub <- strataG::labelHaplotypes(BS.bin.sub, prefix = "HT",
                                        use.indels = TRUE)
  BS.dna.sub <- as.dna(BS.dna[names(BS.dna) %in% BS.gt.sub@data$id, ,
                              drop = TRUE])

  bases <- haplotypes::basecomp(BS.dna.sub)
  print(paste0("Mitogenome base composition:"))
  print(colMeans(bases))

  pi.gt <- strataG::nucleotideDiversity(BS.gt.sub,
                                         bases = c("a", "c", "g", "t"),
                                         simplify = TRUE)
  pi.peg <- pegas::nuc.div(BS.bin.sub, variance = TRUE,
                           pairwise.deletion = FALSE)
  h.peg <- pegas::hap.div(BS.bin.sub, variance = TRUE, method = "Nei")
  res <- data.frame(Region, mean(pi.gt), pi.peg[1], pi.peg[2],
                    length(unique(BS.h.sub$haps)), h.peg[1], h.peg[2])
  colnames(res) <- c("Region", "pi.strataG", "pi.pegas", "pi.pegas.var",
                     "HT.richness.strataG", "h.pegas", "h.pegas.var")
  divers.region <- rbind(divers.region, res)
}

knitr::kable(
  divers.region,
  caption = "Nucleotide and haplotype diversity per region",
  # format = "html",
  digits = 6
  ) %>%
  kableExtra::kable_styling(full_width = FALSE,font_size = 5)


dA.gt <- strataG::nucleotideDivergence(BS.gt,
                                       probs = c(0, 0.025, 0.5, 0.975, 1),
                                       model = "RAW")
knitr::kable(
  dA.gt$within[order(dA.gt$within$mean, decreasing = TRUE), -1],
  # format = "html",
  caption = "Nucleotide divergence within each region"
  ) %>%
  kableExtra::kable_styling(full_width = FALSE,font_size = 5)
knitr::kable(
  dA.gt$between[order(dA.gt$between$mean, decreasing = TRUE), -1],
```

```r
  # format = "html",
  caption = "Nucleotide divergence between each region"
  ) %>%
  kableExtra::kable_styling(full_width = FALSE, font_size = 5)



hapcount <- pegas::haploFreq(BS.dna@sequence, fac = BS.gt@schemes$Region,
                             split = NULL, what = 1, haplo = NULL)
row.names(hapcount) <- paste0("HT", sprintf('%0.2d', 1:BS.hap@nhap))

kableExtra::kbl(hapcount,
  caption = "Haplotype counts per region according to pegas",
  longtable = TRUE, booktabs = TRUE) %>%
  kableExtra::kable_styling(full_width = FALSE,font_size = 5,
                            latex_options = c("repeat_header", "hold_position"))

group.levels <- levels(BS.gi$other$ind.meta$Region)
group.names <- BS.gi$other$ind.meta$Region

HTs <- paste0("HT", sprintf('%0.3d', 1:BS.hap@nhap))
df <- data.frame(group = NULL,nind = NULL, hap = NULL, nhap = NULL,
                 hapfreq = NULL, stringsAsFactors = FALSE)
for (k in 1:length(group.levels)) {
  group.inds <- adegenet::indNames(BS.gi)[group.names == group.levels[k]]
  for (i in 1:BS.hap@nhap) {
    group <- group.levels[k]
    nind <- length(group.inds)
    hap <- HTs[i]
    nhap <- sum(group.inds %in% BS.hap@haplist[[i]])
    hapfreq <- nhap/nind
    df <- plyr::rbind.fill(df, data.frame(group = group, nind = nind, hap = hap,
                                          nhap = nhap, hapfreq = hapfreq,
                                          stringsAsFactors = FALSE))
  }
}
df$group <- factor(df$group, levels = group.levels)

haplotype.labels <- unique(df$hap)
haplotype.labels2 <- haplotype.labels[seq(1, length(haplotype.labels), by = 5)]

HT.plot <- ggplot2::ggplot(data = df) +
  ggplot2::geom_bar(ggplot2::aes(x = hap, y = nhap, fill = group),
                    stat = "identity", position = "stack") +
  ggplot2::labs(x = "Haplotypes", y = "Number of individuals") +
  ggplot2::scale_fill_manual(values = colours.8) +
  ggplot2::scale_x_discrete(breaks = haplotype.labels2) +
  ggplot2::theme(
      axis.text = ggplot2::element_text(angle = 45, hjust = 1, size = 5),
      axis.title.x = ggplot2::element_text(size = 10),
      axis.title.y = ggplot2::element_text(size = 10),
      legend.text = ggplot2::element_text(size = 7))
print(HT.plot)
```

Table 62: Nucleotide and haplotype diversity per region

| | Region | pi.strataG | pi.pegas | pi.pegas.var | HT.richness.strataG | h.pegas | h.pegas.var |
|---|---|---|---|---|---|---|---|
| 1 | E-PAC | 0.000274 | 0.000274 | 0e+00 | 10 | 0.925000 | 0.001807 |
| 11 | E-ATL | NaN | NaN | NaN | 1 | NaN | NaN |
| 12 | IWP | 0.002089 | 0.002090 | 1e-06 | 142 | 0.987349 | 0.000003 |
| 13 | Japan | 0.000389 | 0.000389 | 0e+00 | 4 | 0.690323 | 0.001877 |
| 14 | Fiji | NaN | 0.001374 | 1e-06 | 8 | 0.972222 | 0.002578 |

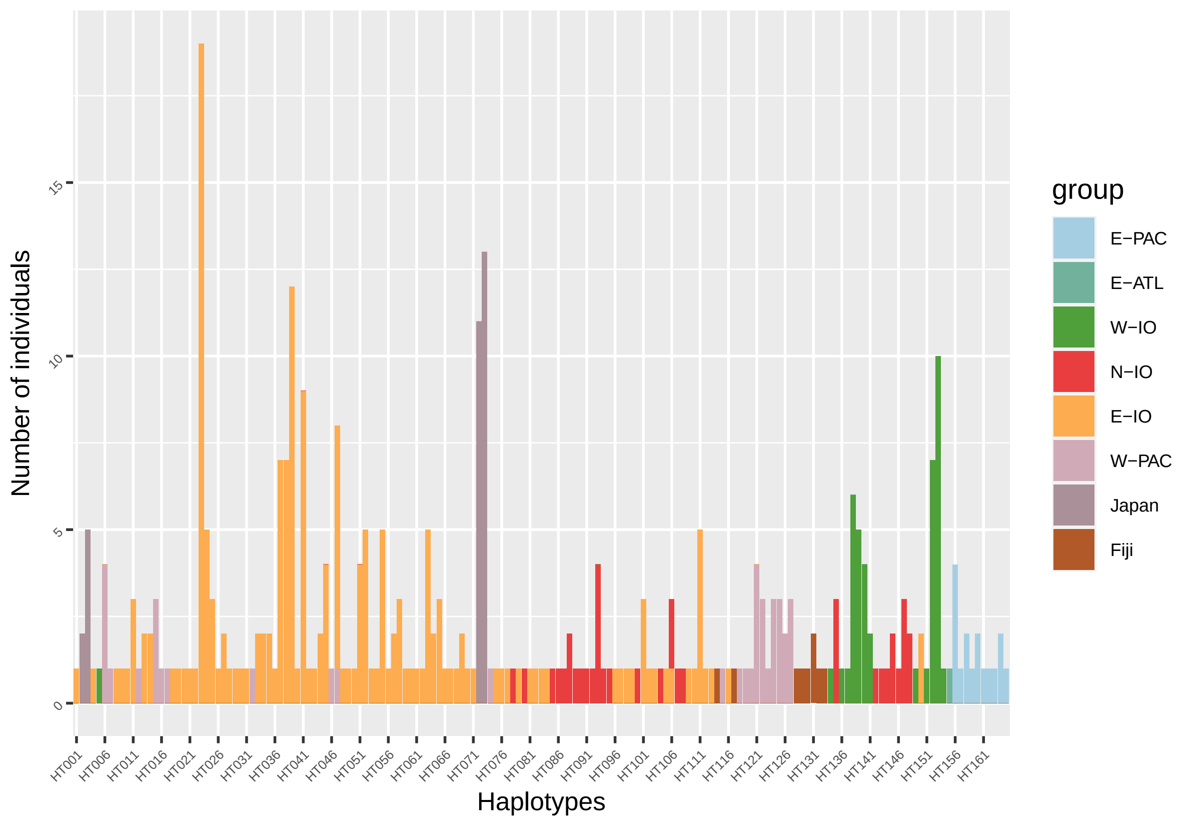


```r
ggplot2::ggsave(HT.plot, filename = "HT_barplot_per_Region.png", width = 30,
                height = 15, units = "cm")
```

```
## [1] "E-PAC"
## [1] "Mitogenome base composition:"
##         A         T         G         C
## 0.3151545 0.2423992 0.1307560 0.3116903
## [1] "E-ATL"
## [1] "Mitogenome base composition:"
##         A         T         G         C
## 0.3145576 0.2423680 0.1312702 0.3118041
## [1] "IWP"
## [1] "Mitogenome base composition:"
##         A         T         G         C
## 0.3145866 0.2430667 0.1311699 0.3111767
## [1] "Japan"
## [1] "Mitogenome base composition:"
##         A         T         G         C
## 0.3146686 0.2430748 0.1311079 0.3111486
## [1] "Fiji"
## [1] "Mitogenome base composition:"
##         A         T         G         C
## 0.3146038 0.2429049 0.1311531 0.3113381
```

## Table 63: Nucleotide divergence within each region

| | stratum | mean | q.0 | q.0.025 | q.0.5 | q.0.975 | q.1 |
|---|---|---|---|---|---|---|---|
| 4 | IWP | 0.0020900 | 0 | 5.99e-05 | 0.0008982 | 0.0058084 | 0.0061078 |
| 3 | Fiji | 0.0013739 | 0 | 0.00e+00 | 0.0002395 | 0.0055090 | 0.0055090 |
| 5 | Japan | 0.0003894 | 0 | 0.00e+00 | 0.0000000 | 0.0010778 | 0.0010778 |
| 2 | E-PAC | 0.0002740 | 0 | 0.00e+00 | 0.0002994 | 0.0004192 | 0.0004192 |
| 1 | E-ATL | NA | NA | NA | NA | NA | NA |

## Table 64: Nucleotide divergence between each region

| | strata.1 | strata.2 | dA | mean | q.0 | q.0.025 | q.0.5 | q.0.975 | q.1 |
|---|---|---|---|---|---|---|---|---|---|
| 7 | E-PAC | Japan | 0.0086961 | 0.0090278 | 0.0089222 | 0.0089222 | 0.0090419 | 0.0091617 | 0.0091617 |
| 5 | E-PAC | Fiji | 0.0081569 | 0.0089808 | 0.0088623 | 0.0088623 | 0.0089820 | 0.0090419 | 0.0090419 |
| 6 | E-PAC | IWP | 0.0077769 | 0.0089588 | 0.0086228 | 0.0087425 | 0.0089222 | 0.0092216 | 0.0094012 |
| 4 | E-ATL | Japan | 0.0087545 | 0.0089492 | 0.0089222 | 0.0089222 | 0.0089222 | 0.0090419 | 0.0090419 |
| 2 | E-ATL | Fiji | 0.0082152 | 0.0089022 | 0.0088623 | 0.0088623 | 0.0089222 | 0.0089222 | 0.0089222 |
| 3 | E-ATL | IWP | 0.0078560 | 0.0089009 | 0.0086228 | 0.0086826 | 0.0088623 | 0.0091617 | 0.0092814 |
| 1 | E-ATL | E-PAC | 0.0061692 | 0.0063061 | 0.0062275 | 0.0062275 | 0.0063174 | 0.0063473 | 0.0063473 |
| 8 | Fiji | IWP | 0.0002662 | 0.0019981 | 0.0000599 | 0.0002395 | 0.0008982 | 0.0056287 | 0.0057485 |
| 10 | IWP | Japan | 0.0004710 | 0.0017107 | 0.0002994 | 0.0003593 | 0.0008982 | 0.0057485 | 0.0061078 |
| 9 | Fiji | Japan | 0.0005956 | 0.0014773 | 0.0008383 | 0.0008383 | 0.0009581 | 0.0054072 | 0.0057485 |

Table 65: Haplotype counts per region according to pegas

| | E-PAC | E-ATL | W-IO | N-IO | E-IO | W-PAC | Japan | Fiji |
|---|---|---|---|---|---|---|---|---|
| HT01 | 0 | 0 | 1 | 0 | 0 | 0 | 0 | 0 |
| HT02 | 0 | 0 | 1 | 0 | 0 | 0 | 0 | 0 |
| HT03 | 0 | 0 | 1 | 0 | 0 | 0 | 0 | 0 |
| HT04 | 0 | 0 | 1 | 0 | 0 | 0 | 0 | 0 |
| HT05 | 0 | 0 | 0 | 3 | 0 | 0 | 0 | 0 |
| HT06 | 0 | 0 | 0 | 2 | 0 | 0 | 0 | 0 |
| HT07 | 0 | 0 | 0 | 1 | 0 | 0 | 0 | 0 |
| HT08 | 0 | 0 | 1 | 0 | 0 | 0 | 0 | 0 |
| HT09 | 0 | 0 | 1 | 0 | 0 | 0 | 0 | 0 |
| HT10 | 0 | 0 | 10 | 0 | 0 | 0 | 0 | 0 |
| HT11 | 0 | 0 | 7 | 0 | 0 | 0 | 0 | 0 |
| HT12 | 0 | 0 | 0 | 0 | 2 | 0 | 0 | 0 |
| HT13 | 0 | 0 | 0 | 1 | 0 | 0 | 0 | 0 |
| HT14 | 0 | 0 | 0 | 1 | 0 | 0 | 0 | 0 |
| HT15 | 0 | 0 | 6 | 0 | 0 | 0 | 0 | 0 |
| HT16 | 0 | 0 | 5 | 0 | 0 | 0 | 0 | 0 |
| HT17 | 0 | 0 | 2 | 0 | 0 | 0 | 0 | 0 |
| HT18 | 0 | 0 | 4 | 0 | 0 | 0 | 0 | 0 |
| HT19 | 0 | 0 | 0 | 0 | 0 | 0 | 0 | 1 |
| HT20 | 0 | 0 | 0 | 1 | 0 | 0 | 0 | 0 |
| HT21 | 0 | 0 | 0 | 2 | 0 | 0 | 0 | 0 |
| HT22 | 0 | 0 | 0 | 3 | 0 | 0 | 0 | 0 |
| HT23 | 0 | 0 | 0 | 0 | 0 | 3 | 0 | 0 |
| HT24 | 0 | 0 | 0 | 0 | 0 | 4 | 0 | 0 |
| HT25 | 0 | 0 | 0 | 0 | 1 | 0 | 0 | 0 |
| HT26 | 0 | 0 | 0 | 0 | 0 | 1 | 0 | 0 |
| HT27 | 0 | 0 | 0 | 0 | 1 | 0 | 0 | 0 |
| HT28 | 0 | 0 | 0 | 0 | 1 | 0 | 0 | 0 |
| HT29 | 0 | 0 | 0 | 0 | 2 | 0 | 0 | 0 |
| HT30 | 0 | 0 | 0 | 0 | 5 | 0 | 0 | 0 |
| HT31 | 0 | 0 | 0 | 0 | 3 | 0 | 0 | 0 |
| HT32 | 0 | 0 | 0 | 0 | 1 | 0 | 0 | 0 |
| HT33 | 0 | 0 | 0 | 0 | 1 | 0 | 0 | 0 |
| HT34 | 0 | 0 | 0 | 0 | 7 | 0 | 0 | 0 |
| HT35 | 0 | 0 | 0 | 0 | 1 | 0 | 0 | 0 |
| HT36 | 0 | 0 | 0 | 0 | 3 | 0 | 0 | 0 |
| HT37 | 0 | 0 | 0 | 0 | 2 | 0 | 0 | 0 |
| HT38 | 0 | 0 | 0 | 0 | 2 | 0 | 0 | 0 |
| HT39 | 0 | 0 | 0 | 0 | 2 | 0 | 0 | 0 |
| HT40 | 0 | 0 | 0 | 0 | 2 | 0 | 0 | 0 |
| HT41 | 0 | 0 | 0 | 0 | 1 | 0 | 0 | 0 |
| HT42 | 0 | 0 | 0 | 0 | 1 | 0 | 0 | 0 |
| HT43 | 0 | 0 | 0 | 0 | 1 | 0 | 0 | 0 |
| HT44 | 0 | 0 | 0 | 0 | 5 | 0 | 0 | 0 |
| HT45 | 0 | 0 | 0 | 0 | 4 | 0 | 0 | 0 |
| HT46 | 0 | 0 | 0 | 0 | 9 | 0 | 0 | 0 |
| HT47 | 0 | 0 | 0 | 0 | 1 | 0 | 0 | 0 |
| HT48 | 0 | 0 | 0 | 0 | 1 | 0 | 0 | 0 |
| HT49 | 0 | 0 | 0 | 0 | 1 | 0 | 0 | 0 |
| HT50 | 0 | 0 | 0 | 0 | 12 | 0 | 0 | 0 |
| HT51 | 0 | 0 | 0 | 0 | 7 | 0 | 0 | 0 |
| HT52 | 0 | 0 | 0 | 0 | 1 | 0 | 0 | 0 |
| HT53 | 0 | 0 | 0 | 0 | 1 | 0 | 0 | 0 |
| HT54 | 0 | 0 | 0 | 0 | 1 | 0 | 0 | 0 |
| HT55 | 0 | 0 | 0 | 0 | 19 | 0 | 0 | 0 |
| HT56 | 0 | 0 | 0 | 0 | 7 | 1 | 0 | 0 |
| HT57 | 0 | 0 | 0 | 0 | 1 | 0 | 0 | 0 |
| HT58 | 0 | 0 | 0 | 0 | 1 | 0 | 0 | 0 |

Table 65: Haplotype counts per region according to pegas *(continued)*

| | E-PAC | E-ATL | W-IO | N-IO | E-IO | W-PAC | Japan | Fiji |
|---|---|---|---|---|---|---|---|---|
| HT59 | 0 | 0 | 0 | 0 | 1 | 0 | 0 | 0 |
| HT60 | 0 | 0 | 0 | 0 | 2 | 0 | 0 | 0 |
| HT61 | 0 | 0 | 0 | 0 | 1 | 0 | 0 | 0 |
| HT62 | 0 | 0 | 0 | 0 | 1 | 0 | 0 | 0 |
| HT63 | 0 | 0 | 0 | 0 | 1 | 0 | 0 | 0 |
| HT64 | 0 | 0 | 0 | 1 | 0 | 0 | 0 | 0 |
| HT65 | 0 | 0 | 0 | 1 | 0 | 0 | 0 | 0 |
| HT66 | 0 | 0 | 0 | 0 | 1 | 0 | 0 | 0 |
| HT67 | 0 | 0 | 0 | 1 | 0 | 0 | 0 | 0 |
| HT68 | 0 | 0 | 0 | 0 | 3 | 0 | 0 | 0 |
| HT69 | 0 | 0 | 0 | 2 | 0 | 0 | 0 | 0 |
| HT70 | 0 | 0 | 0 | 1 | 0 | 0 | 0 | 0 |
| HT71 | 0 | 0 | 0 | 1 | 0 | 0 | 0 | 0 |
| HT72 | 0 | 0 | 0 | 1 | 0 | 0 | 0 | 0 |
| HT73 | 0 | 0 | 0 | 1 | 0 | 0 | 0 | 0 |
| HT74 | 0 | 0 | 0 | 0 | 1 | 0 | 0 | 0 |
| HT75 | 0 | 0 | 0 | 0 | 1 | 0 | 0 | 0 |
| HT76 | 0 | 0 | 0 | 0 | 1 | 0 | 0 | 0 |
| HT77 | 0 | 0 | 0 | 0 | 5 | 0 | 0 | 0 |
| HT78 | 0 | 0 | 0 | 1 | 0 | 0 | 0 | 0 |
| HT79 | 0 | 0 | 0 | 4 | 0 | 0 | 0 | 0 |
| HT80 | 0 | 0 | 0 | 1 | 0 | 0 | 0 | 0 |
| HT81 | 0 | 0 | 0 | 0 | 1 | 0 | 0 | 0 |
| HT82 | 0 | 0 | 0 | 0 | 1 | 0 | 0 | 0 |
| HT83 | 0 | 0 | 0 | 1 | 0 | 0 | 0 | 0 |
| HT84 | 0 | 0 | 0 | 0 | 0 | 1 | 0 | 0 |
| HT85 | 0 | 0 | 0 | 0 | 0 | 3 | 0 | 0 |
| HT86 | 0 | 0 | 0 | 0 | 0 | 3 | 0 | 0 |
| HT87 | 0 | 0 | 0 | 0 | 0 | 2 | 0 | 0 |
| HT88 | 0 | 0 | 0 | 0 | 0 | 1 | 0 | 0 |
| HT89 | 0 | 0 | 0 | 0 | 0 | 0 | 0 | 1 |
| HT90 | 0 | 0 | 0 | 0 | 0 | 0 | 0 | 1 |
| HT91 | 0 | 0 | 0 | 0 | 0 | 0 | 0 | 1 |
| HT92 | 0 | 0 | 0 | 0 | 0 | 1 | 0 | 0 |
| HT93 | 0 | 0 | 0 | 0 | 0 | 4 | 0 | 0 |
| HT94 | 0 | 0 | 0 | 0 | 0 | 3 | 0 | 0 |
| HT95 | 0 | 0 | 0 | 0 | 0 | 1 | 0 | 0 |
| HT96 | 0 | 0 | 0 | 0 | 0 | 1 | 0 | 0 |
| HT97 | 0 | 0 | 0 | 0 | 0 | 0 | 0 | 1 |
| HT98 | 0 | 0 | 0 | 0 | 0 | 0 | 0 | 2 |
| HT99 | 0 | 0 | 0 | 0 | 0 | 0 | 0 | 1 |
| HT100 | 0 | 0 | 0 | 0 | 0 | 0 | 0 | 1 |
| HT101 | 0 | 0 | 0 | 0 | 1 | 0 | 0 | 0 |
| HT102 | 0 | 0 | 0 | 0 | 2 | 0 | 0 | 0 |
| HT103 | 0 | 0 | 0 | 0 | 1 | 0 | 0 | 0 |
| HT104 | 0 | 0 | 0 | 0 | 1 | 0 | 0 | 0 |
| HT105 | 0 | 0 | 0 | 0 | 5 | 0 | 0 | 0 |
| HT106 | 0 | 0 | 0 | 0 | 2 | 0 | 0 | 0 |
| HT107 | 0 | 0 | 0 | 0 | 0 | 1 | 0 | 0 |
| HT108 | 0 | 0 | 1 | 0 | 0 | 0 | 0 | 0 |
| HT109 | 0 | 0 | 0 | 0 | 0 | 1 | 0 | 0 |
| HT110 | 0 | 0 | 0 | 0 | 1 | 0 | 0 | 0 |
| HT111 | 0 | 0 | 0 | 0 | 1 | 0 | 0 | 0 |
| HT112 | 0 | 0 | 0 | 0 | 0 | 1 | 0 | 0 |
| HT113 | 0 | 0 | 0 | 0 | 1 | 0 | 0 | 0 |
| HT114 | 0 | 0 | 0 | 0 | 1 | 0 | 0 | 0 |
| HT115 | 0 | 0 | 0 | 0 | 2 | 0 | 0 | 0 |
| HT116 | 0 | 0 | 0 | 0 | 2 | 0 | 0 | 0 |
| HT117 | 0 | 0 | 0 | 0 | 0 | 3 | 0 | 0 |
| HT118 | 0 | 0 | 0 | 0 | 0 | 1 | 0 | 0 |
| HT119 | 0 | 0 | 0 | 0 | 1 | 0 | 0 | 0 |
| HT120 | 0 | 0 | 0 | 0 | 1 | 0 | 0 | 0 |
| HT121 | 0 | 0 | 0 | 0 | 0 | 1 | 0 | 0 |
| HT122 | 0 | 0 | 0 | 0 | 1 | 0 | 0 | 0 |
| HT123 | 0 | 0 | 0 | 0 | 1 | 0 | 0 | 0 |
| HT124 | 0 | 0 | 0 | 0 | 0 | 0 | 5 | 0 |
| HT125 | 0 | 0 | 0 | 0 | 0 | 0 | 2 | 0 |
| HT126 | 0 | 0 | 0 | 0 | 1 | 0 | 0 | 0 |
| HT127 | 0 | 0 | 0 | 0 | 5 | 0 | 0 | 0 |
| HT128 | 0 | 0 | 0 | 0 | 4 | 0 | 0 | 0 |
| HT129 | 0 | 0 | 0 | 0 | 1 | 0 | 0 | 0 |
| HT130 | 0 | 0 | 0 | 0 | 1 | 0 | 0 | 0 |
| HT131 | 0 | 0 | 0 | 0 | 1 | 0 | 0 | 0 |
| HT132 | 0 | 0 | 0 | 0 | 1 | 0 | 0 | 0 |
| HT133 | 0 | 0 | 0 | 1 | 0 | 0 | 0 | 0 |
| HT134 | 0 | 0 | 0 | 0 | 1 | 0 | 0 | 0 |
| HT135 | 0 | 0 | 0 | 0 | 1 | 0 | 0 | 0 |
| HT136 | 0 | 0 | 0 | 0 | 3 | 0 | 0 | 0 |
| HT137 | 0 | 0 | 0 | 2 | 1 | 0 | 0 | 0 |
| HT138 | 0 | 0 | 0 | 1 | 0 | 0 | 0 | 0 |
| HT139 | 0 | 0 | 0 | 0 | 1 | 0 | 0 | 0 |
| HT140 | 0 | 0 | 0 | 1 | 0 | 0 | 0 | 0 |
| HT141 | 0 | 0 | 0 | 1 | 0 | 0 | 0 | 0 |
| HT142 | 0 | 0 | 0 | 0 | 1 | 0 | 0 | 0 |

Table 65: Haplotype counts per region according to pegas *(continued)*

| | E-PAC | E-ATL | W-IO | N-IO | E-IO | W-PAC | Japan | Fiji |
|---|---|---|---|---|---|---|---|---|
| HT143 | 0 | 0 | 0 | 0 | 1 | 0 | 0 | 0 |
| HT144 | 0 | 0 | 0 | 0 | 1 | 0 | 0 | 0 |
| HT145 | 0 | 0 | 0 | 0 | 1 | 0 | 0 | 0 |
| HT146 | 0 | 0 | 0 | 0 | 1 | 0 | 0 | 0 |
| HT147 | 0 | 0 | 0 | 0 | 0 | 1 | 0 | 0 |
| HT148 | 0 | 0 | 0 | 0 | 1 | 0 | 0 | 0 |
| HT149 | 0 | 0 | 0 | 0 | 1 | 0 | 0 | 0 |
| HT150 | 0 | 0 | 0 | 0 | 3 | 0 | 0 | 0 |
| HT151 | 0 | 0 | 0 | 0 | 1 | 0 | 0 | 0 |
| HT152 | 0 | 0 | 0 | 0 | 0 | 0 | 11 | 0 |
| HT153 | 0 | 0 | 0 | 0 | 0 | 0 | 13 | 0 |
| HT154 | 0 | 0 | 0 | 1 | 0 | 0 | 0 | 0 |
| HT155 | 4 | 0 | 0 | 0 | 0 | 0 | 0 | 0 |
| HT156 | 2 | 0 | 0 | 0 | 0 | 0 | 0 | 0 |
| HT157 | 1 | 0 | 0 | 0 | 0 | 0 | 0 | 0 |
| HT158 | 2 | 0 | 0 | 0 | 0 | 0 | 0 | 0 |
| HT159 | 1 | 0 | 0 | 0 | 0 | 0 | 0 | 0 |
| HT160 | 1 | 0 | 0 | 0 | 0 | 0 | 0 | 0 |
| HT161 | 1 | 0 | 0 | 0 | 0 | 0 | 0 | 0 |
| HT162 | 1 | 0 | 0 | 0 | 0 | 0 | 0 | 0 |
| HT163 | 2 | 0 | 0 | 0 | 0 | 0 | 0 | 0 |
| HT164 | 1 | 0 | 0 | 0 | 0 | 0 | 0 | 0 |
| HT165 | 0 | 1 | 0 | 0 | 0 | 0 | 0 | 0 |

## 13.4 Haplotype network

### 13.4.1 Rarefaction

```
hapcount <- pegas::haploFreq(BS.dna@sequence, fac = BS.gt@schemes$Site,
                             split = NULL, what = 1, haplo = NULL)
BCI <- t(hapcount)

S <- vegan::specnumber(BCI) ## observed number of HTs
(raremax <- min(rowSums(BCI)))
Srare <- vegan::rarefy(BCI, raremax, se = FALSE)

plot(S, Srare, xlab = "Observed No. of HTs", ylab = "Rarefied No. of HTs",
     xlim = c(0,8),ylim = c(0,5))
abline(0, 1)
```

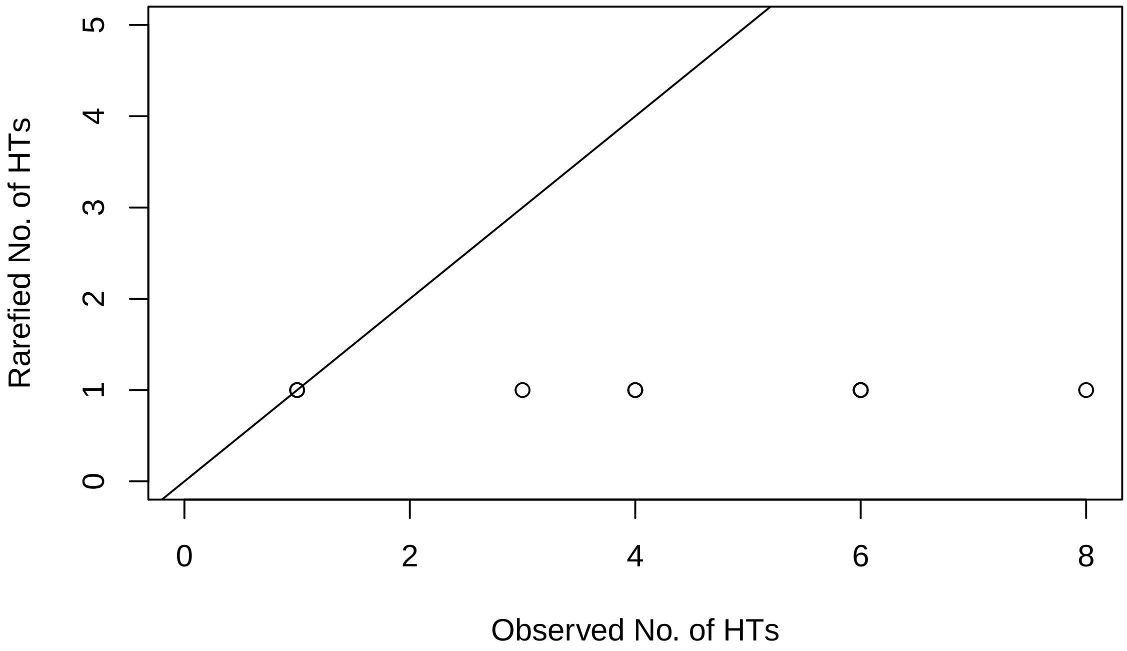


```
# vegan::rarecurve(BCI, step = 5, sample = raremax, col = "blue", cex = 0.6)

## HT accumulation curve based on observed HTs
sp1 <- vegan::specaccum(BCI)
sp2 <- vegan::specaccum(BCI, "random")
plot(sp1, ci.type = "poly", col = "blue", lwd = 2, ci.lty = 0,
     ci.col = "lightblue")
boxplot(sp2, col = "yellow", add = TRUE, pch = "+")
```

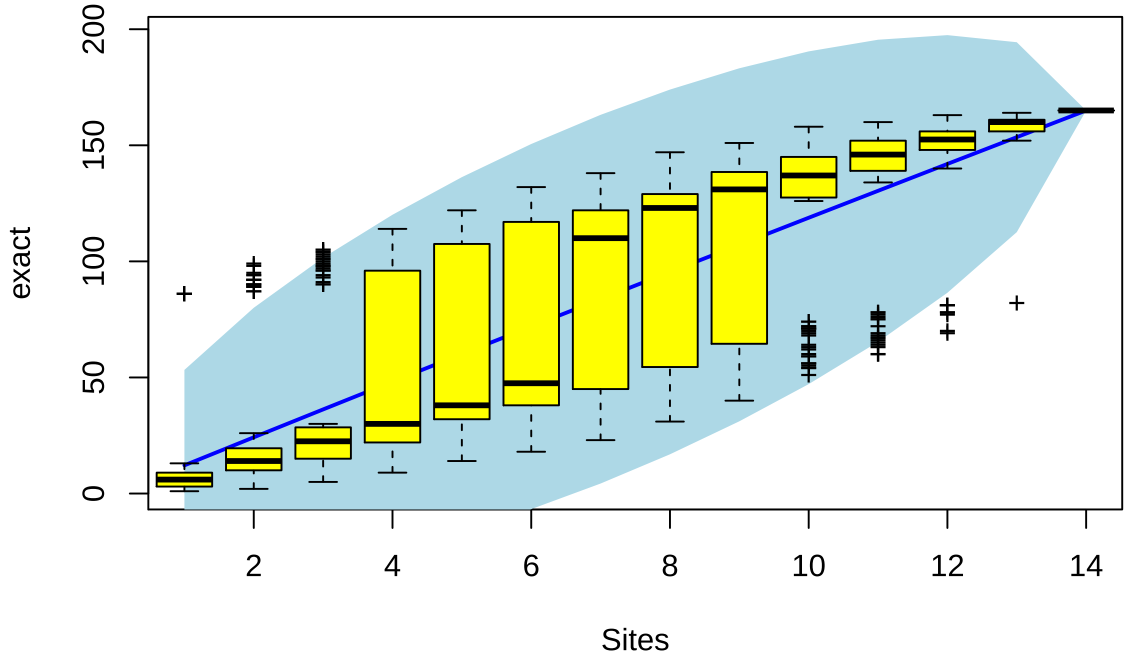


```r
## Extrapolated HT richness based on unobserved HTs
pool <- vegan::poolaccum(BCI)
knitr::kable(pool$means,
             # format = "html",
             caption = "Observed and extrapolated HT richness based on resampling") %>%
  kableExtra::kable_styling(full_width = FALSE,font_size = 5)
plot(pool)
```

Table 66: Observed and extrapolated HT richness based on resampling

| N | S | Chao | Jackknife 1 | Jackknife 2 | Bootstrap |
|---|---|---|---|---|---|
| 3 | 38.53 | 907.120 | 64.08333 | 76.82833 | 49.89407 |
| 4 | 50.61 | 1466.492 | 88.30500 | 113.32167 | 66.53387 |
| 5 | 60.89 | 1987.694 | 109.14600 | 145.09950 | 80.69728 |
| 6 | 74.24 | 2619.667 | 135.33167 | 183.74100 | 98.86869 |
| 7 | 83.42 | 3050.469 | 153.93714 | 212.07048 | 111.48729 |
| 8 | 95.70 | 3860.636 | 178.16000 | 248.01714 | 128.21404 |
| 9 | 107.10 | 4479.339 | 200.70000 | 281.56556 | 143.74568 |
| 10 | 116.44 | 4786.255 | 219.26500 | 309.37078 | 156.48238 |
| 11 | 128.68 | 5240.840 | 243.35273 | 345.08545 | 173.12754 |
| 12 | 140.01 | 4773.671 | 265.47417 | 377.63098 | 188.48911 |
| 13 | 154.95 | 4540.759 | 294.63923 | 420.50814 | 208.75565 |
| 14 | 165.00 | 4176.583 | 314.50000 | 450.12637 | 222.42879 |

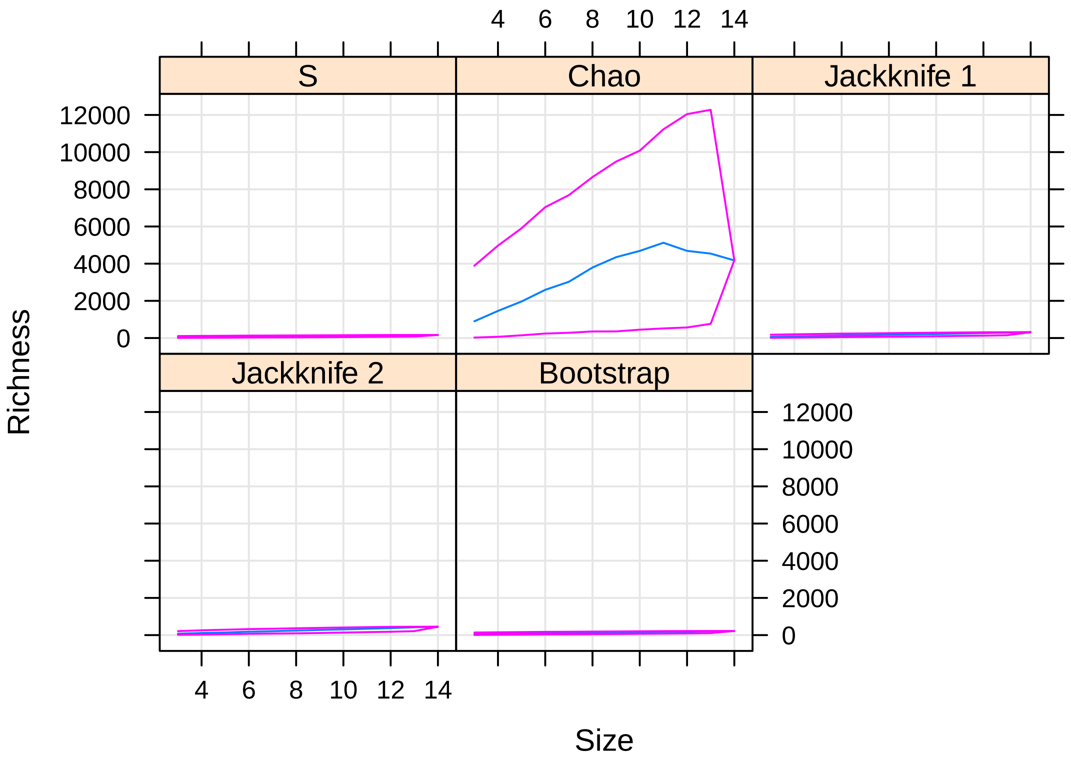


```
## [1] 1
```

### 13.4.2 Network

#### 13.4.2.1 Global

```
strata <- as.character(BS.gt@schemes$Site)
names(strata) <- as.character(BS.gt@schemes$id)
strataG::setStrata(BS.gt) <- strata

net <- pegas::haploNet(BS.h)
ind.hap <- with(
    stack(setNames(attr(BS.h, "index"), rownames(BS.h))),
    table(hap = ind, pop = strata[values]))

size.new <- attr(net, "freq")
size.new <- sqrt(size.new)
```

```r
# colours.14 <- adegenet::funky(14)
colours.14 <- c("#a6cee3", "#66b6db", "#84bf96", "#f57c7c", "#e42622",  "#ffdbb6",
                "#ffb666", "#fe8d19", "#de9e83", "#b6b6db", "#666690", "#977899",
                "#f3e587", "#b15928")

names(colours.14) <- c("Gulf of California","Costa Rica", "Sierra Leone",
                       "South Africa","Reunion","Seychelles","Arabian Sea",
                       "Sri Lanka","Thailand","Indonesia","Papua New Guinea",
                       "Australia","Japan","Fiji")
colours.14.new <- colours.14[order(names(colours.14))]


#### SHAPES #####
df <- as.data.frame(ind.hap)
df2 <- select(df, hap, pop, Freq) %>%
  reshape(., idvar = "hap", timevar = "pop", direction = "wide")
df.rownames <- df2$hap
row.names(df2) <- df.rownames
df2$hap <- NULL
df2 <- sapply(df2, as.integer)
row.names(df2) <- df.rownames
colnames(df2) <- stringr::str_replace_all(string = colnames(df2),
                                          pattern = "Freq.", replacement = "")
res <- colnames(df2)[max.col(df2)]
res2 <- data.frame(HT = row.names(df2), site = res)
res3 <- res2[order(res2$HT),]
shape <- res3$site

tes <- rowSums(df2 > 0)
tes2 <- df2[tes > 1,]
# tes2[,colSums(as.data.frame(tes2) > 0) > 0]

#       Arabian Sea Australia Reunion Seychelles South Africa Sri Lanka Thailand
# HT106           1         1       0          0            0         1        0
# HT139           0         0       2          3            0         0        0
# HT145           0         0       0          0            0         1        1
# HT153           0         0       0          8            2         0        0

shape[shape == "Gulf of California"] <- "square"
shape[shape == "Costa Rica"] <- "circle"
shape[shape == "Sierra Leone"] <- "diamond"
shape[shape == "South Africa"] <- "square"
shape[shape == "Reunion"] <- "square"
shape[shape == "Seychelles"] <- "square"
shape[shape == "Arabian Sea"] <- "circle"
shape[shape == "Sri Lanka"] <- "circle"
shape[shape == "Thailand"] <- "circle"
shape[shape == "Indonesia"] <- "square"
shape[shape == "Papua New Guinea"] <- "diamond"
shape[shape == "Australia"] <- "circle"
shape[shape == "Japan"] <- "square"
shape[shape == "Fiji"] <- "diamond"

names(shape) <- res3$site
shape.leg <- shape[unique(names(shape))]
shape.leg <- shape.leg[order(match(names(shape.leg), names(colours.14)))]
shape.leg[shape.leg == "circle"] <- 16
```

```r
shape.leg[shape.leg == "square"] <- 15
shape.leg[shape.leg == "diamond"] <- 18
shape.leg2 <- shape.leg[order(names(shape.leg))]


par(mar = c(1,1,1,1))
plot(net, size = size.new, scale.ratio = 0.2, pie = ind.hap, labels = FALSE,
     shape = shape,
     show.mutation = 0,threshold = 0,  srt = 1, cex = 0.75, bg = colours.14.new)
# legend(5, 60, names(colours.14), col = colours.14, pch = 19, ncol = 2,
#        x.intersp = 0.5, y.intersp = 0.5, pt.cex = 2)
legend(5, 60, names(colours.14), col = colours.14, pch = as.numeric(shape.leg),
       ncol = 2, x.intersp = 0.5, y.intersp = 0.5, pt.cex = 2)
title(paste0("HT network of all individuals per Site"))
```

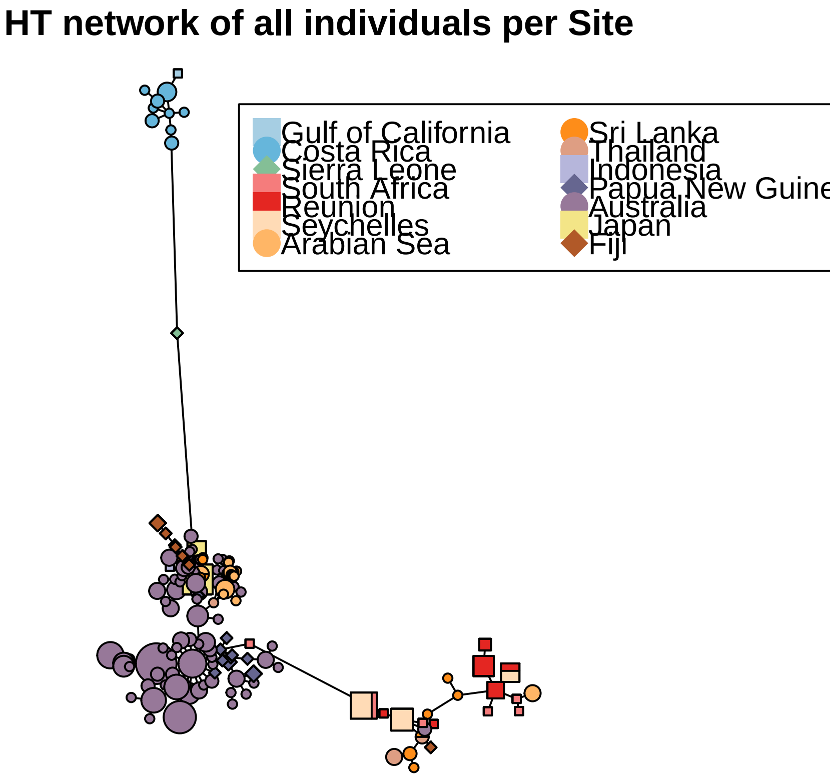


#### 13.4.2.1.1 Per Site

```r
dev.print(
device = png,
file = "Global_per_Site_HT_network2.png",
res = 600,
width = 30,
height = 30,
units = "cm")



# xy <- replot()
# plot(net, xy = xy, size = size.new, scale.ratio = 0.2, pie = ind.hap,
#      labels = FALSE,
#      shape = shape,
#      show.mutation = 0,threshold = 0,  srt = 1, cex = 0.75, bg = colours.14.new)
# # legend(5, 60, names(colours.14), col = colours.14, pch = 19, ncol = 2,
```

```r
# #        x.intersp = 0.5, y.intersp = 0.5, pt.cex = 2)
# legend(5, 60, names(colours.14), col = colours.14, pch = as.numeric(shape.leg),
#        ncol = 2, x.intersp = 0.5, y.intersp = 0.5, pt.cex = 2)
# title(paste0("HT network of all individuals per Site"))
#
#
# dev.print(
# device = png,
# file = "Global_per_Site_HT_network3.png",
# res = 600,
# width = 30,
# height = 30,
# units = "cm")

###############################################################
clust.names <- unlist(BS.hap@haplist[1:132])
strata <- as.character(BS.gt@schemes$Site)
names(strata) <- as.character(BS.gt@schemes$id)
strataG::setStrata(BS.gt) <- strata
BS.gt.clust <- BS.gt[clust.names,,, drop = TRUE]

schemes.clust  <- BS.gt.clust@schemes[BS.gt.clust@schemes$id %in% clust.names,]
strata <- as.character(schemes.clust$Site)
names(strata) <- as.character(schemes.clust$id)
strataG::setStrata(BS.gt.clust) <- strata

ind.names <- names(BS.dna) %in% BS.gt.clust@data$id
BS.dna.clust <- as.dna(BS.dna[ind.names, , drop = TRUE])
BS.bin.clust <- subset(BS.bin, names(BS.bin) %in% BS.gt.clust@data$id)
BS.haps <- strataG::labelHaplotypes(BS.bin.clust, prefix = "HT",
                                    use.indels = TRUE)
haps <- BS.haps$haps
BS.hap.clust <- haplotypes::haplotype(BS.dna.clust,indels = "sic")


HTs <- unique(haps[names(haps) %in% names(BS.bin.clust)])
BS.h.clust <- pegas::haplotype(BS.bin.clust, HTs)

net <- pegas::haploNet(BS.h.clust)
ind.hap <- with(
    stack(setNames(attr(BS.h.clust, "index"), rownames(BS.h.clust))),
    table(hap = ind, pop = strata[values]))

size.new <- attr(net, "freq")
size.new <- sqrt(size.new)


colours <- colours.14[-c(1:3,5,6)]
colours.new <- colours[order(names(colours))]


### SHAPES
df <- as.data.frame(ind.hap)
df2 <- select(df, hap, pop, Freq) %>%
  reshape(., idvar = "hap", timevar = "pop", direction = "wide")
df.rownames <- df2$hap
row.names(df2) <- df.rownames
```

```r
df2$hap <- NULL
df2 <- sapply(df2, as.integer)
row.names(df2) <- df.rownames
colnames(df2) <- stringr::str_replace_all(string = colnames(df2),
                                          pattern = "Freq.", replacement = "")
res <- colnames(df2)[max.col(df2)]
res2 <- data.frame(HT = row.names(df2), site = res)
res3 <- res2[order(res2$HT),]
shape <- res2$site

tes <- rowSums(df2 > 0)
tes2 <- df2[tes > 1,]
#       Arabian Sea         Australia          Sri Lanka
#HT026      1                 1                1

shape[shape == "South Africa"] <- "square"
shape[shape == "Arabian Sea"] <- "circle"
shape[shape == "Sri Lanka"] <- "circle"
shape[shape == "Thailand"] <- "circle"
shape[shape == "Indonesia"] <- "square"
shape[shape == "Papua New Guinea"] <- "diamond"
shape[shape == "Australia"] <- "circle"
shape[shape == "Japan"] <- "square"
shape[shape == "Fiji"] <- "diamond"


names(shape) <- res2$site
shape.leg <- shape[unique(names(shape))]
shape.leg <- shape.leg[order(match(names(shape.leg), names(colours)))]
shape.leg[shape.leg == "circle"] <- 16
shape.leg[shape.leg == "square"] <- 15
shape.leg[shape.leg == "diamond"] <- 18



par(mar = c(1,1,1,1))
plot(net, size = size.new, scale.ratio = 0.6, pie = ind.hap, labels = FALSE,
     asp = 1, shape = shape,
     show.mutation = 1,threshold = 0,  srt = 1, cex = 0.75, bg = colours.new,
     fast = FALSE)
# legend(-40, 70, names(colours), col = colours, pch = 19, ncol = 2,
#        x.intersp = 0.5, y.intersp = 0.5, pt.cex = 2)
legend(-25, 25, names(colours), col = colours, pch = as.numeric(shape.leg),
       ncol = 2, x.intersp = 0.5, y.intersp = 0.5, pt.cex = 2)
title(paste0("HT network of all E-IO/W-PAC individuals per Site"))
```

**HT network of all E−IO/W−PAC individuals per Site**

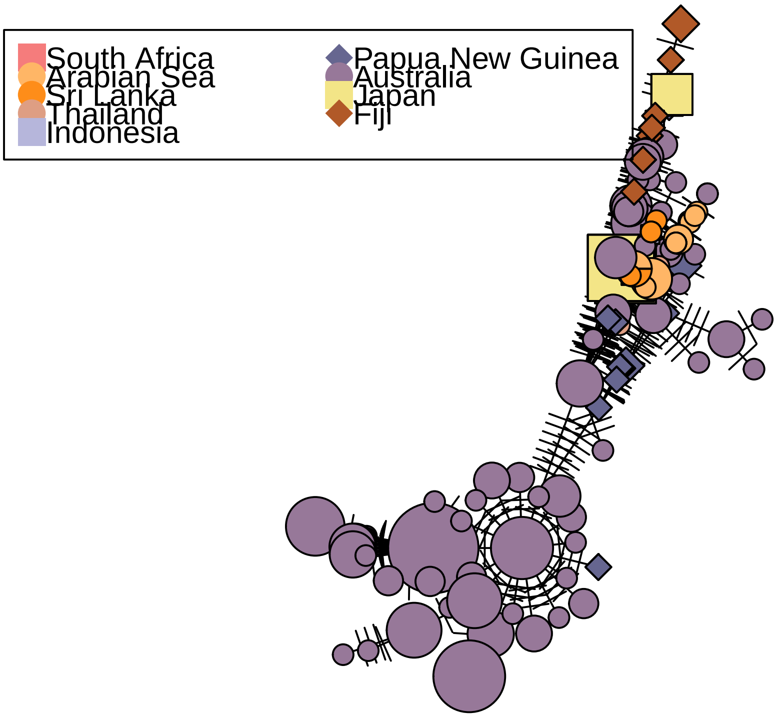


```
dev.print(
device = png,
file = "E-IO-W-PAC_cluster_HT_network.png",
res = 600,
width = 30,
height = 30,
units = "cm")


# xy <- replot()
# save(xy, file = "HT_network_replot_by_E-IO_cluster.Rdata")
load("HT_network_replot_by_E-IO_cluster.Rdata")
par(mar = c(1,1,1,1))
plot(net, xy = xy, size = size.new, scale.ratio = 0.2, pie = ind.hap,
     labels = FALSE, asp = 1, shape = shape,
     show.mutation = 2,threshold = 0,  srt = 1, cex = 0.75, bg = colours.new,
     fast = FALSE)
# legend(-40, 70, names(colours), col = colours, pch = 19, ncol = 2,
#        x.intersp = 0.5, y.intersp = 0.5, pt.cex = 2)
# legend(-20, 35, names(colours), col = colours, pch = as.numeric(shape.leg),
#        ncol = 2, x.intersp = 0.5, y.intersp = 0.5, pt.cex = 2)
title(paste0("HT network of all E-IO/W-PAC individuals per Site"))
```

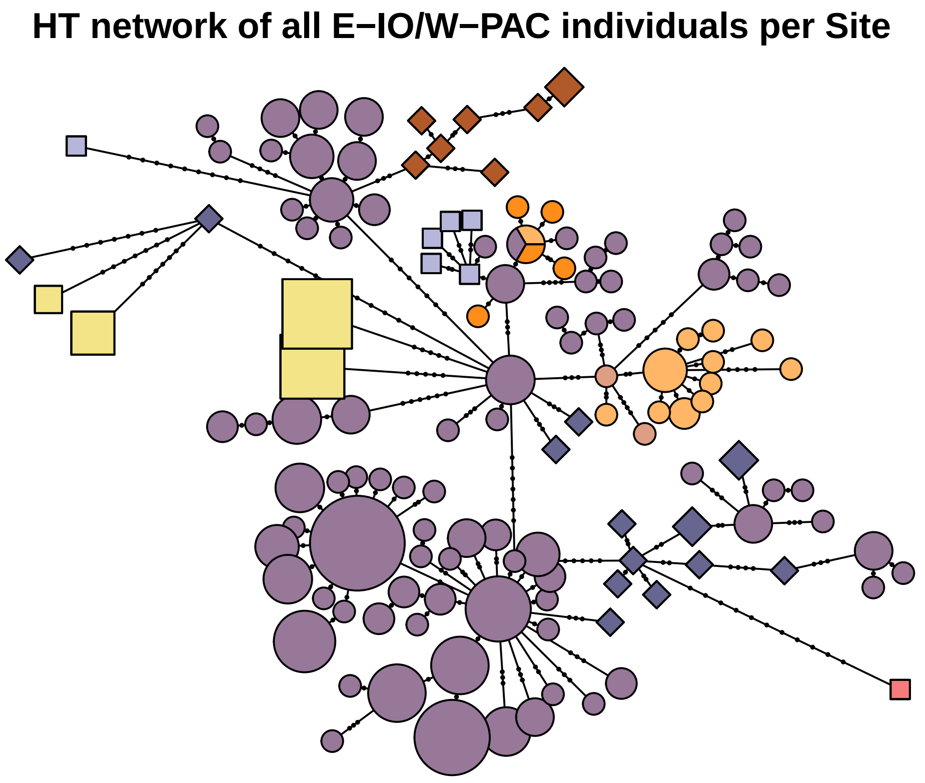


```
dev.print(
device = png,
file = "E-IO-W-PAC_cluster_HT_network3.png",
res = 600,
width = 30,
height = 30,
units = "cm")
```

```
## pdf
##   2
## pdf
##   2
## pdf
##   2
```

HT network per Site

```
strata <- as.character(BS.gt@schemes$Site)
names(strata) <- as.character(BS.gt@schemes$id)
strataG::setStrata(BS.gt) <- strata

BS.haps <- strataG::labelHaplotypes(BS.bin, prefix = "HT", use.indels = FALSE)
haps <- BS.haps$haps

for (Site in levels(BS.gt@schemes$Site)) {
  BS.gt.sub <- BS.gt[,,Site, drop = TRUE]
  if (length(BS.gt.sub@data$id) > 1) {
    ind.names <- names(BS.dna) %in% BS.gt.sub@data$id
    BS.dna.sub <- as.dna(BS.dna[ind.names, , drop = TRUE])
    BS.bin.sub <- subset(BS.bin, names(BS.bin) %in% BS.gt.sub@data$id)

    HTs <- unique(haps[names(haps) %in% names(BS.bin.sub)])
    BS.h.sub <- pegas::haplotype(BS.bin.sub)
```

```
    BS.h.sub <- sort(BS.h.sub, what = "label")
    net <- pegas::haploNet(BS.h.sub)
    ind.hap <- with(
      stack(setNames(attr(BS.h.sub, "index"), rownames(BS.h.sub))),
      table(hap = ind, pop = BS.gt.sub@data$stratum[values]))

     size.new <- attr(net, "freq")
     size.new <- sqrt(size.new)

    attr(net, "labels") <- paste0(attr(net, "labels"),"\nn=",
                                  attr(net, "freq"))
    plot(net, size = size.new, scale.ratio = 6, pie = NULL,
      show.mutation = 1,threshold = 0,  srt = 1, cex = 0.75)
    title(paste0(Site))
  }
}
```

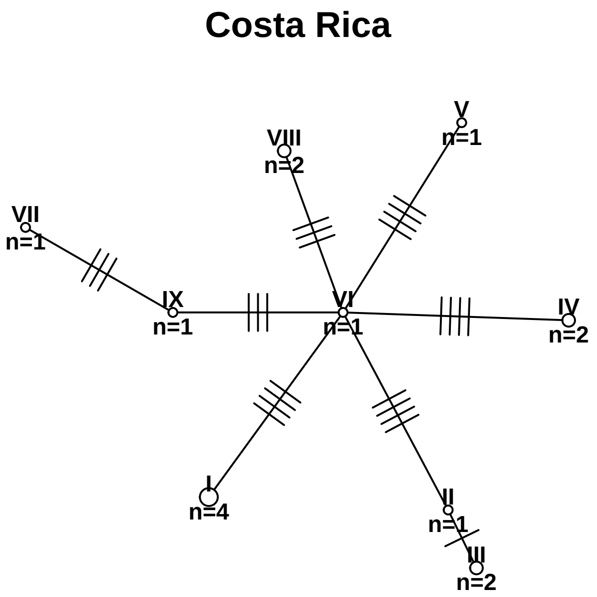

## South Africa

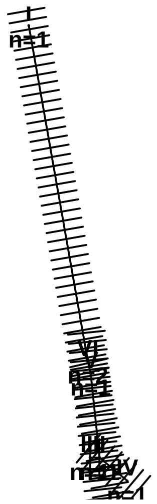


## Reunion

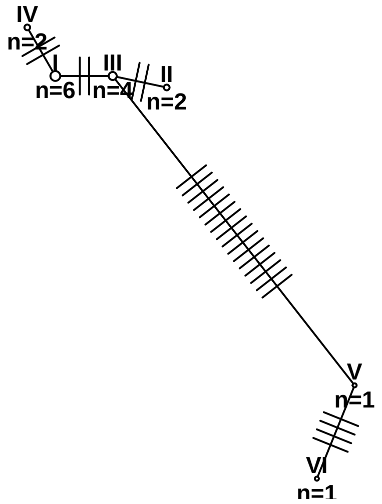

## Seychelles

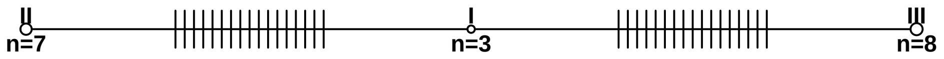


## Arabian Sea

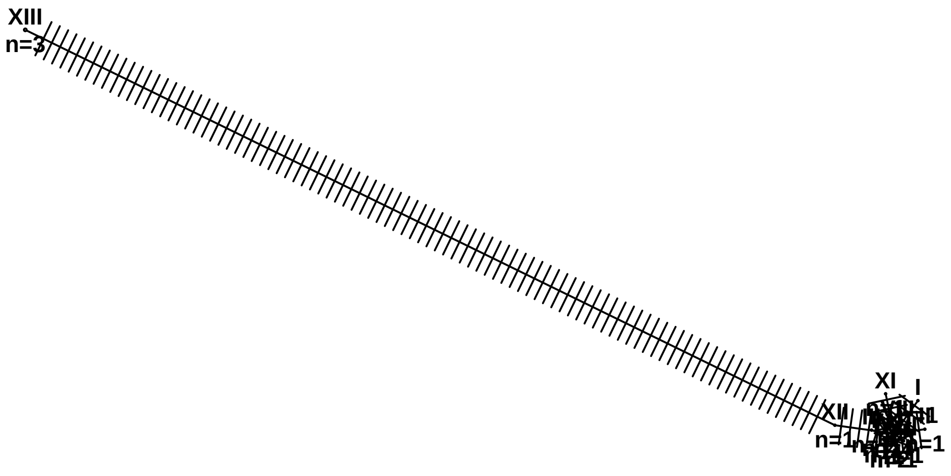

Sri Lanka

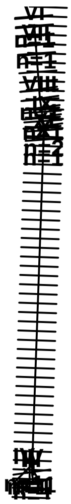

Thailand

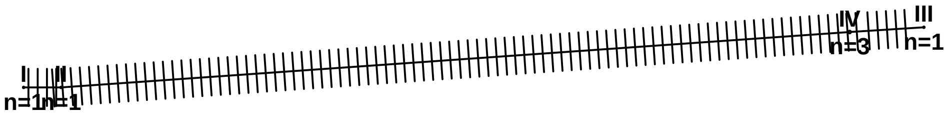

# Indonesia

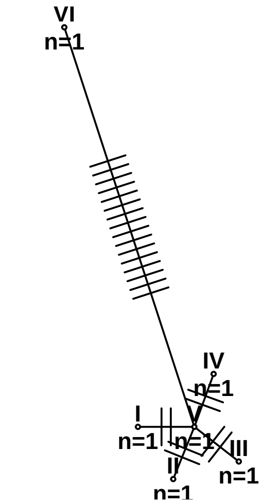


# Papua New Guinea

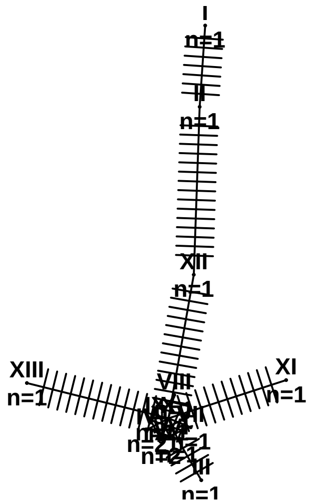

## Australia

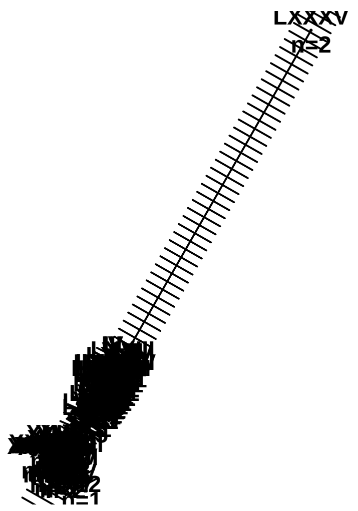


## Japan

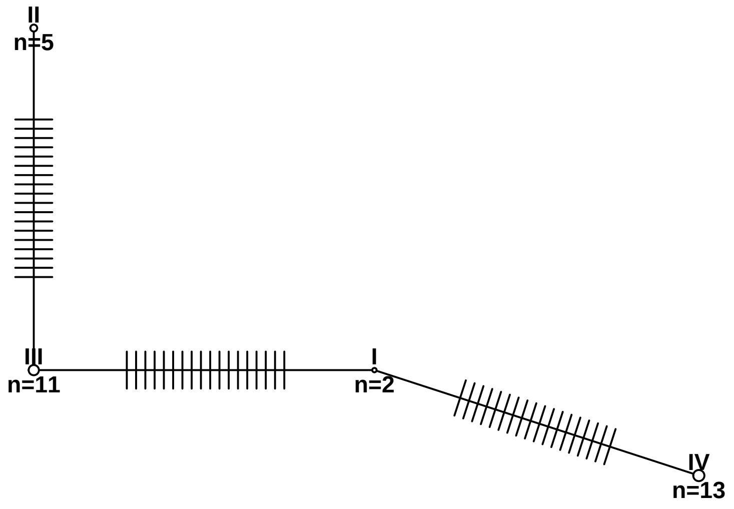

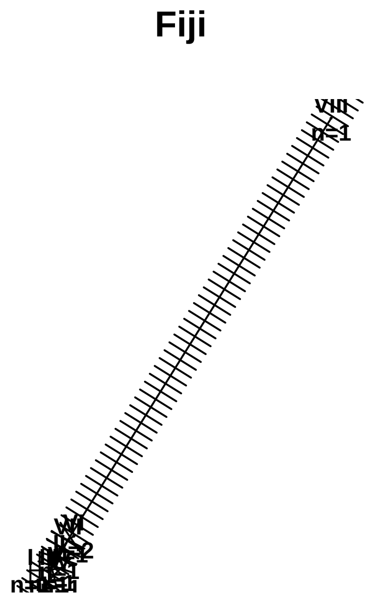


```
strata <- as.character(BS.gt@schemes$Region)
names(strata) <- as.character(BS.gt@schemes$id)
strataG::setStrata(BS.gt) <- strata

net <- pegas::haploNet(BS.h)
ind.hap <- with(
    stack(setNames(attr(BS.h, "index"), rownames(BS.h))),
    table(hap = ind, pop = BS.gt@schemes$Region[values]))

size.new <- attr(net, "freq")
size.new <- sqrt(size.new)
colours.8.new <- colours.8[order(names(colours.8))]
par(mar = c(1,1,1,1))
plot(net, size = size.new, scale.ratio = 0.2, pie = ind.hap, labels = FALSE,
     show.mutation = 0,threshold = 0,  srt = 1, cex = 0.75, bg = colours.8)
legend(-30, 50, names(colours.8), col = colours.8, pch = 19, ncol = 2,
       x.intersp = 0.5, y.intersp = 1, pt.cex = 2)
title(paste0("HT network of all individuals per region"))
```

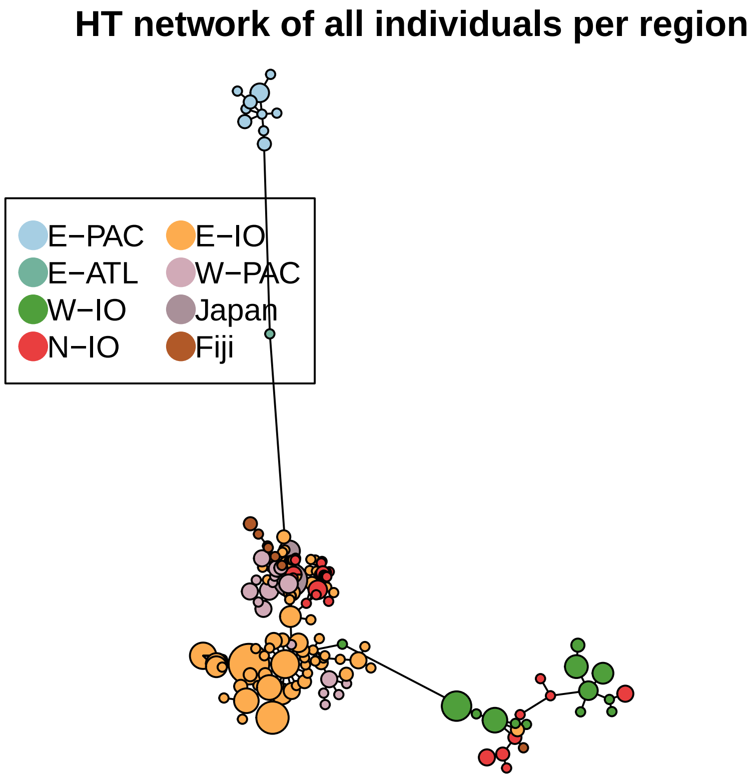


#### 13.4.2.1.2 Per region

HT network per region

```
strata <- as.character(BS.gt@schemes$Region)
names(strata) <- as.character(BS.gt@schemes$id)
strataG::setStrata(BS.gt) <- strata

BS.haps <- strataG::labelHaplotypes(BS.bin, prefix = "HT", use.indels = FALSE)
haps <- BS.haps$haps

for (pop in levels(BS.gt@schemes$Region)) {
  BS.gt.sub <- BS.gt[,,pop, drop = TRUE]
    if (length(BS.gt.sub@data$id) > 1) {
      ind.names <- names(BS.dna) %in% BS.gt.sub@data$id
      BS.dna.sub <- as.dna(BS.dna[ind.names, , drop = TRUE])
      BS.bin.sub <- subset(BS.bin, names(BS.bin) %in% BS.gt.sub@data$id)

      HTs <- unique(haps[names(haps) %in% names(BS.bin.sub)])
      # BS.h.sub <- pegas::haplotype(BS.bin.sub, HTs)
      BS.h.sub <- pegas::haplotype(BS.bin.sub)

      BS.h.sub <- sort(BS.h.sub, what = "label")
      net <- pegas::haploNet(BS.h.sub)
      ind.hap <- with(
        stack(setNames(attr(BS.h.sub, "index"), rownames(BS.h.sub))),
        table(hap = ind, pop = BS.gt.sub@data$stratum[values]))

      size.new <- attr(net, "freq")
      size.new <- sqrt(size.new)

      attr(net, "labels") <- paste0(attr(net, "labels"),"\nn=",
                                    attr(net, "freq"))
```

```
      plot(net, size = size.new, scale.ratio = 6, pie = NULL,
        show.mutation = 1,threshold = 0,  srt = 1, cex = 0.75)
      title(paste0(pop))
    }
}
```

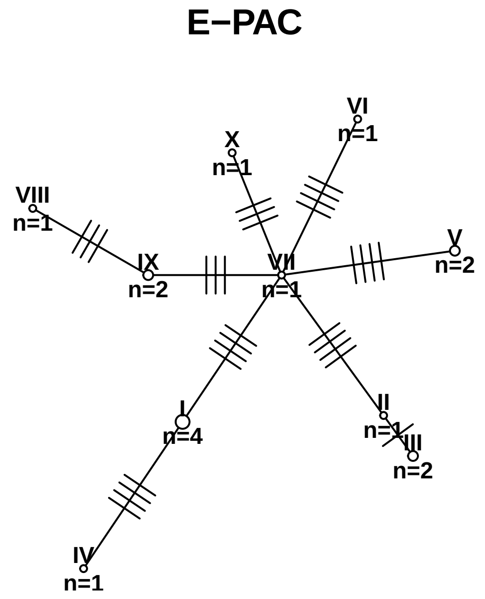

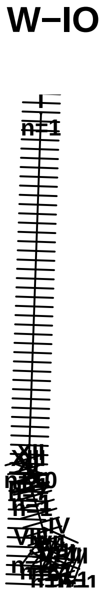
W−IO
I
n=1

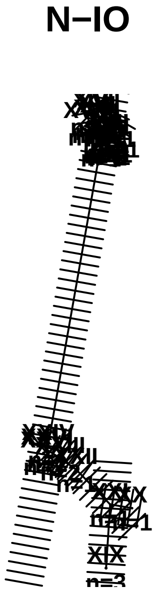
N−IO
XIX
n=3

## E−IO

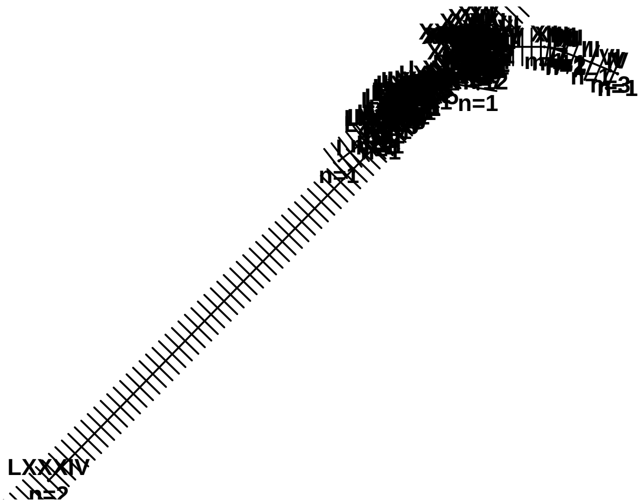

## W−PAC

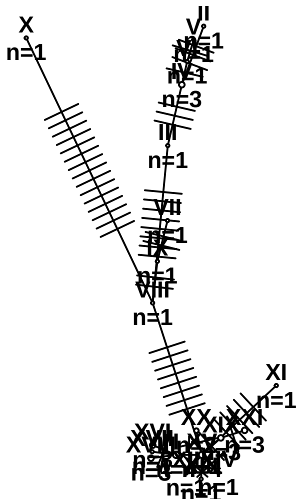

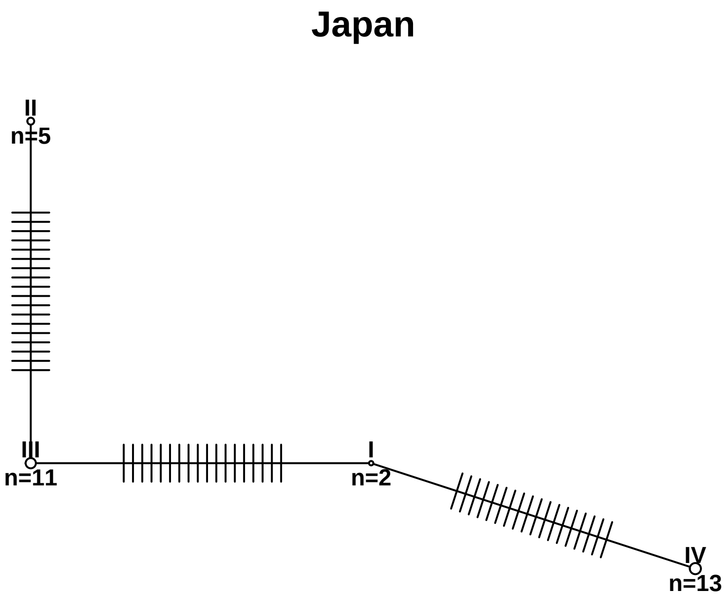


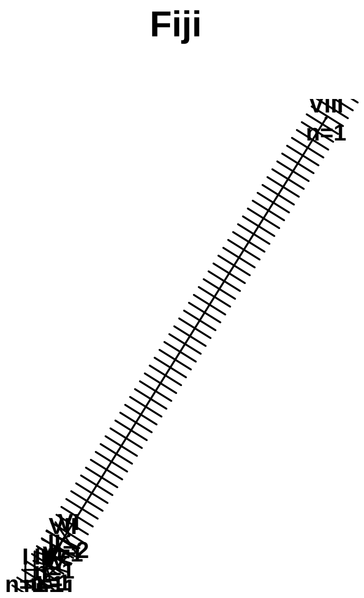


```r
strata <- as.character(BS.gt@schemes$Site)
names(strata) <- as.character(BS.gt@schemes$id)
strataG::setStrata(BS.gt) <- strata
```

```r
BS.gt.IO <- BS.gt[BS.gt@schemes$id[!BS.gt@schemes$Site %in%
                                    c("Gulf of California","Costa Rica",
                                      "Sierra Leone")],,, drop = TRUE]


schemes.IO <- BS.gt.IO@schemes[!BS.gt.IO@schemes$Site %in%
                                c("Gulf of California","Costa Rica", "Sierra Leone"),]
strata <- as.character(schemes.IO$Site)
names(strata) <- as.character(schemes.IO$id)
strataG::setStrata(BS.gt.IO) <- strata

ind.names <- names(BS.dna) %in% BS.gt.IO@data$id
BS.dna.IO <- as.dna(BS.dna[ind.names, , drop = TRUE])
BS.bin.IO <- subset(BS.bin, names(BS.bin) %in% BS.gt.IO@data$id)

BS.haps <- strataG::labelHaplotypes(BS.bin.IO, prefix = "HT", use.indels = TRUE)
haps <- BS.haps$haps

HTs <- unique(haps[names(haps) %in% names(BS.bin.IO)])
BS.h.IO <- pegas::haplotype(BS.bin.IO, HTs)

net <- pegas::haploNet(BS.h.IO)
ind.hap <- with(
    stack(setNames(attr(BS.h.IO, "index"), rownames(BS.h.IO))),
    table(hap = ind, pop = strata[values]))

size.new <- attr(net, "freq")
size.new <- sqrt(size.new)

colours <- colours.14[-c(1:3)]
colours.new <- colours[order(names(colours))]

par(mar = c(1,1,1,1))
plot(net, size = size.new, scale.ratio = 0.5, pie = ind.hap, labels = FALSE, asp = 1,
     show.mutation = 0,threshold = 0,  srt = 1, cex = 0.75, bg = colours.new)
legend(-40, 70, names(colours), col = colours, pch = 19, ncol = 2,
       x.intersp = 0.5, y.intersp = 0.5, pt.cex = 2)
title(paste0("HT network of all IWP individuals per Site"))
```

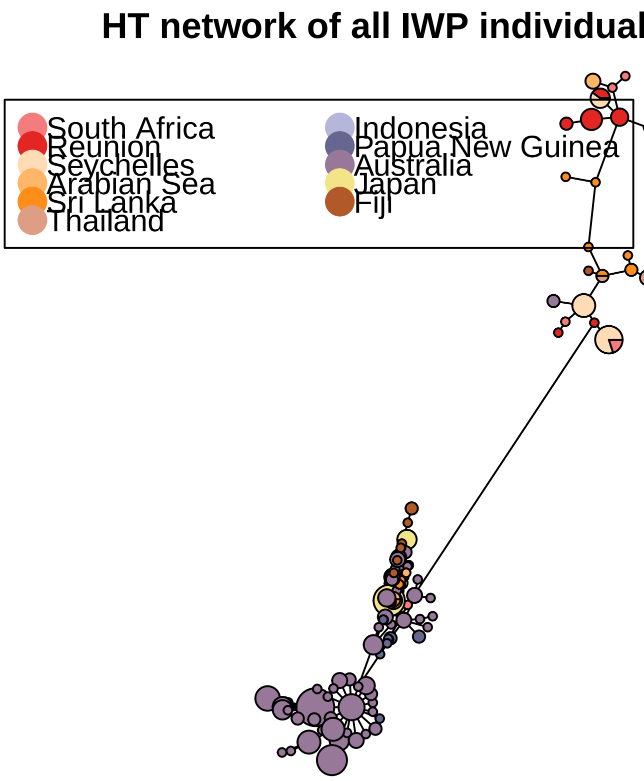


#### 13.4.2.2 Within Indo - West Pacific per Site

```
colours <- colours.27[-c(1:3)]
colours.new <- colours[order(names(colours))]


dev.print(
device = png,
file = "Indian_Ocean_HT_network.png",
res = 600,
width = 30,
height = 30,
units = "cm")
```

```
## pdf
##   2
```

```

strata <- as.character(BS.gt@schemes$Site)
names(strata) <- as.character(BS.gt@schemes$id)
strataG::setStrata(BS.gt) <- strata
AUS.names <- BS.gt@schemes$id[BS.gt@schemes$Site == "Australia" &
                                !BS.gt@schemes$id %in% c("CLF012",
                                                         "CLF014")] # 2 samples with high divergence
BS.gt.AUS <- BS.gt[AUS.names,,, drop = TRUE]

schemes.AUS <- BS.gt.AUS@schemes[BS.gt.AUS@schemes$id %in% AUS.names,]
strata <- as.character(schemes.AUS$Location)
names(strata) <- as.character(schemes.AUS$id)
strataG::setStrata(BS.gt.AUS) <- strata
BS.gt.AUS@schemes <- schemes.AUS
```

```
ind.names <- names(BS.dna) %in% AUS.names
BS.dna.AUS <- as.dna(BS.dna[ind.names, , drop = TRUE])
BS.bin.AUS <- subset(BS.bin, names(BS.bin) %in% BS.gt.AUS@data$id)
BS.haps <- strataG::labelHaplotypes(BS.bin.AUS, prefix = "HT", use.indels = TRUE)
haps <- BS.haps$haps
HTs <- unique(haps[names(haps) %in% names(BS.bin.AUS)])
BS.h.AUS <- pegas::haplotype(BS.bin.AUS, HTs)

net <- pegas::haploNet(BS.h.AUS)
ind.hap <- with(
    stack(setNames(attr(BS.h.AUS, "index"), rownames(BS.h.AUS))),
    table(hap = ind, pop = strata[values]))

colours.13.new <- colours.13[order(names(colours.13))]

par(mar = c(1,1,1,1))
plot(net, size = size.new, scale.ratio = 0.5, pie = ind.hap, labels = FALSE, asp = 1,
     show.mutation = 2,threshold = 0,  srt = 1, cex = 0.75, bg = colours.13.new)
legend(12, 7, names(colours.13), col = colours.13, pch = 19, ncol = 1,
       x.intersp = 0.2, y.intersp = 0.5, pt.cex = 2)
title(paste0("HT network of all Australian individuals per river"))
```

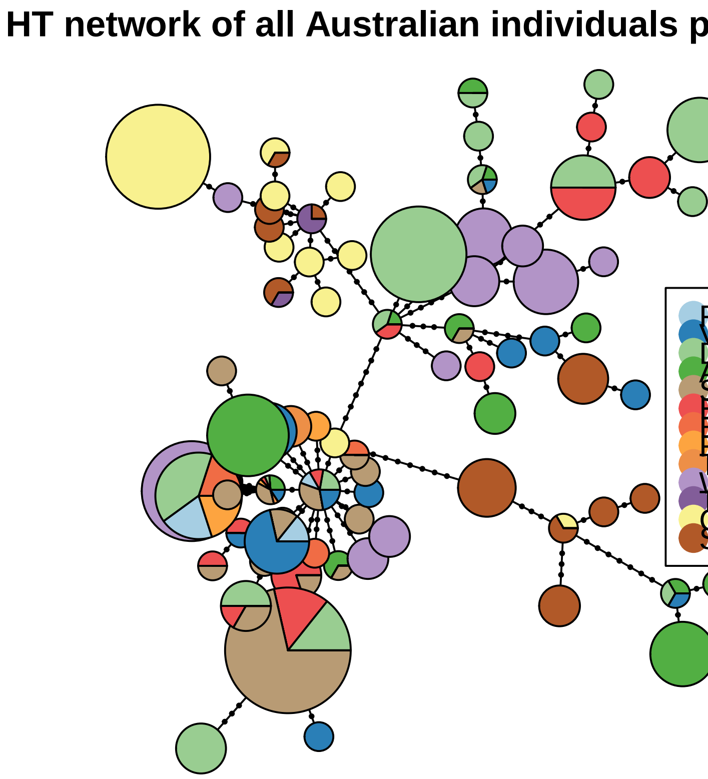


#### 13.4.2.3 Within Australia per River

```
dev.print(
device = png,
file = "Australia_HT_network4.png",
res = 600,
width = 30,
height = 30,
units = "cm")

## pdf
```

```
## 2
```

```r
strata <- as.character(BS.gt.AUS@schemes$Location)
names(strata) <- as.character(BS.gt.AUS@schemes$id)
strataG::setStrata(BS.gt.AUS) <- strata

pop.levels <- c("Fitzroy River","Victoria River","Daly River",
                "Adelaide River","South Alligator River", "East Alligator River",
                "Blue mud Bay","Roper River","Towns River","Wenlock River",
                "Trinity inlet","Clarence River","Sydney Harbour")
BS.gt.AUS@schemes$Location <- factor(BS.gt.AUS@schemes$Location,
                                     levels = pop.levels)

BS.haps <- strataG::labelHaplotypes(BS.bin.AUS, prefix = "HT",
                                    use.indels = FALSE)
haps <- BS.haps$haps

for (Location in levels(BS.gt.AUS@schemes$Location)) {
  BS.gt.sub <- BS.gt.AUS[,,Location, drop = TRUE]
  if (length(BS.gt.sub@data$id) > 1) {
    ind.names <- names(BS.dna) %in% BS.gt.sub@data$id
    BS.dna.sub <- as.dna(BS.dna[ind.names, , drop = TRUE])
    BS.bin.sub <- subset(BS.bin, names(BS.bin) %in% BS.gt.sub@data$id)

    HTs <- unique(haps[names(haps) %in% names(BS.bin.sub)])
    BS.h.sub <- pegas::haplotype(BS.bin.sub)
     if (length(HTs) > 1) {
       BS.h.sub <- sort(BS.h.sub, what = "label")
       net <- pegas::haploNet(BS.h.sub)
        ind.hap <- with(
          stack(setNames(attr(BS.h.sub, "index"), rownames(BS.h.sub))),
          table(hap = ind, pop = BS.gt.sub@data$stratum[values]))

       size.new <- attr(net, "freq")
       size.new <- sqrt(size.new)

       attr(net, "labels") <- paste0(attr(net, "labels"),"\nn=",
                                     attr(net, "freq"))
       plot(net, size = size.new, scale.ratio = 6, pie = NULL,
            show.mutation = 1,threshold = 0,  srt = 1, cex = 0.75)
       title(paste0(Location))
     }
  }
}
```

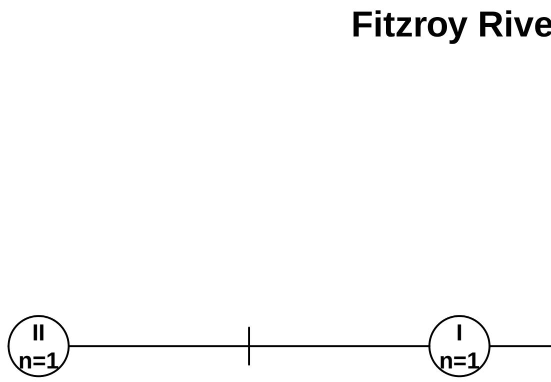


#### 13.4.2.3.1 HT network per Location in Australia

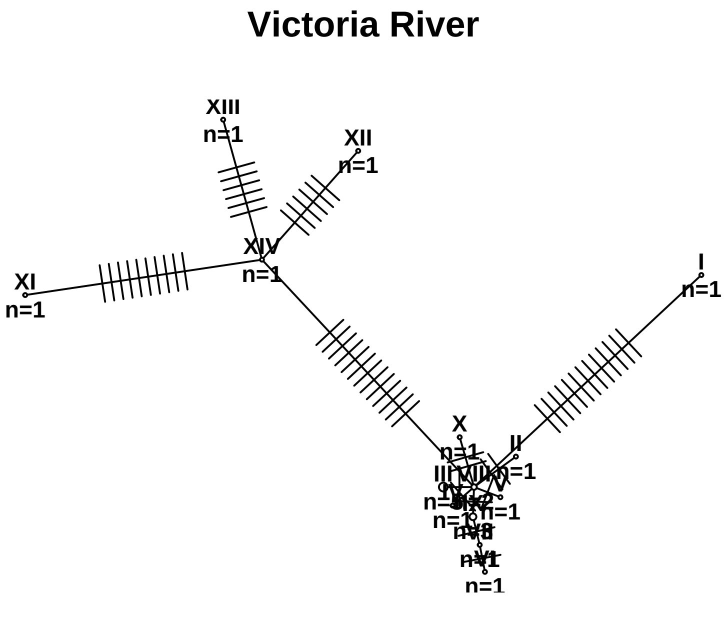

## Daly River

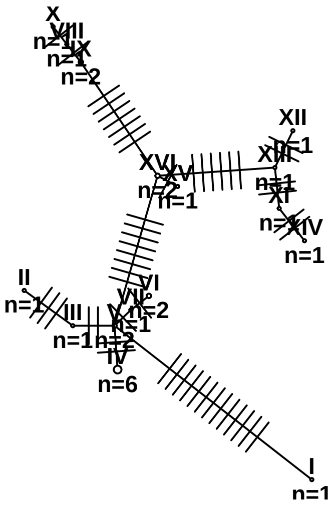


## Adelaide River

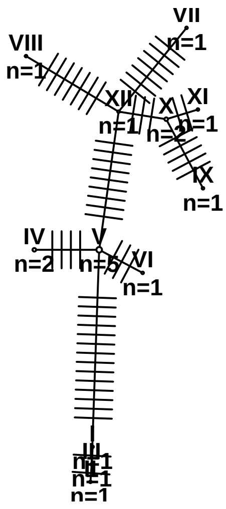

## South Alligator River

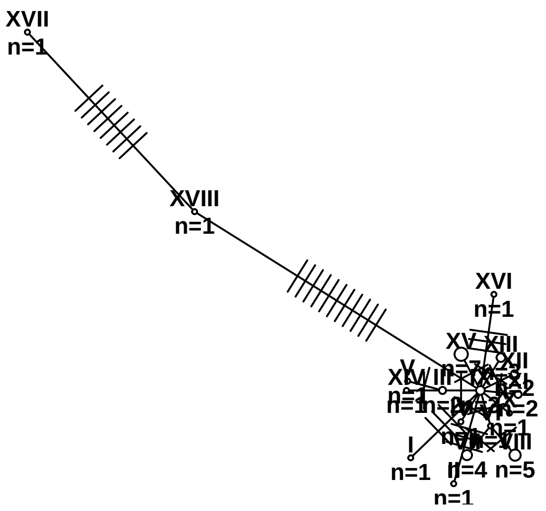


## East Alligator River

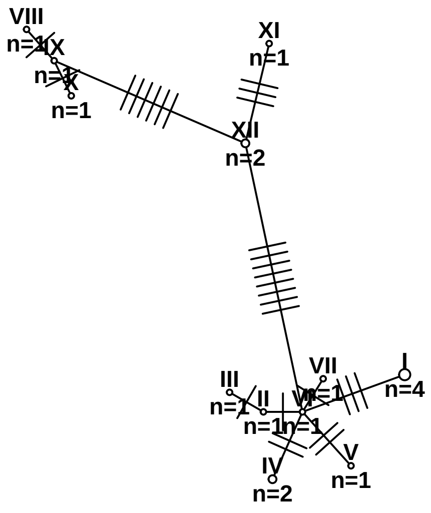

## Blue mud Bay

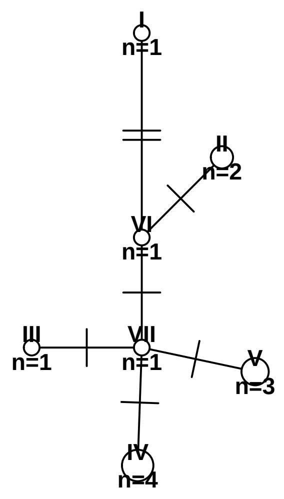


## Roper River

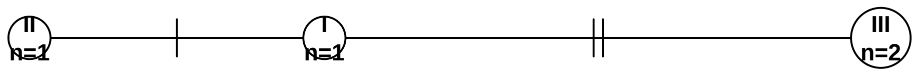

## Towns River

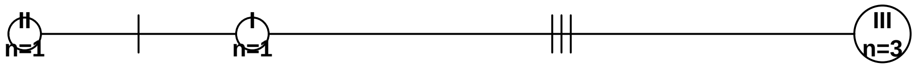


## Wenlock River

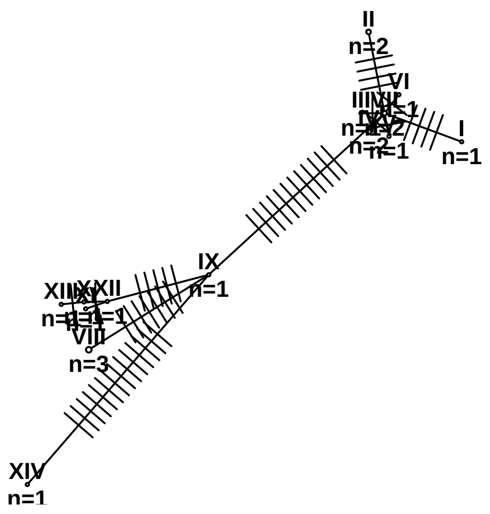

## Clarence River

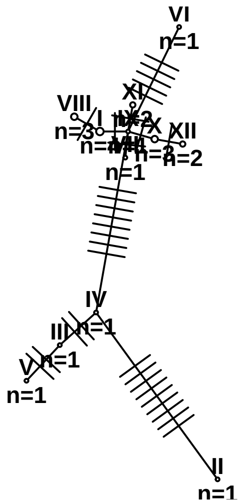


## Sydney Harbour

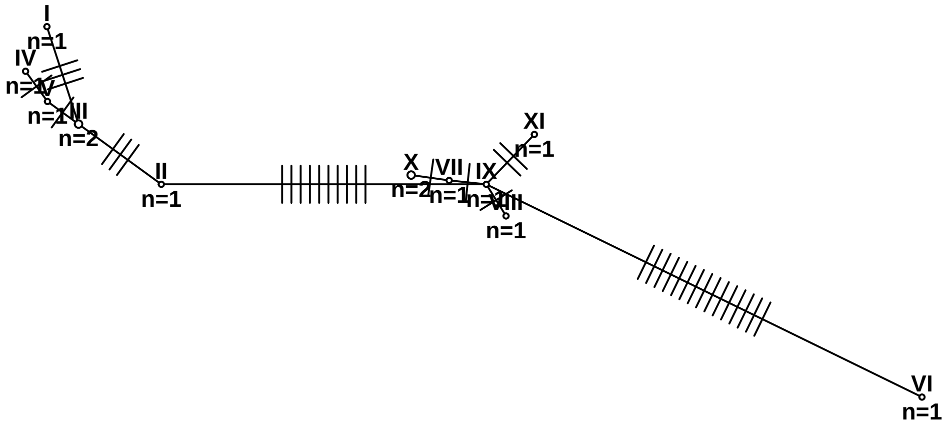

## 13.5 Genetic differentiation

### 13.5.1 PHIst per Location

```
strata <- as.character(BS.gt@schemes$Location)
names(strata) <- as.character(BS.gt@schemes$id)
strataG::setStrata(BS.gt) <- strata

popstr.mt.Location <-
  strataG::popStructTest(
    BS.gt,
    nrep = 1000,
    stats = "all",
    type = "both",
    keep.null = FALSE,
    quietly = TRUE,
    max.cores = parallel::detectCores()-1,
    write.output = TRUE
  )
save(popstr.mt.Location, file = "Mitogenome_ALL_Locations_phist.Rdata")
readr::write_tsv(popstr.mt.Location$pairwise$result,
                 path = "Mitogenome_ALL_Locations_phist.txt")
```

```
load("Mitogenome_ALL_Locations_phist.Rdata")
knitr::kable(
  popstr.mt.Location$overall$result,
  digits = 4,
  # format = "html",
  caption = "Overall mtDNA differentiation between Sites"
  ) %>%
  kableExtra::kable_styling(full_width = FALSE,font_size = 5)

kableExtra::kbl(
  popstr.mt.Location$pairwise$result[order(popstr.mt.Location$pairwise$result$PHIst,
                                      decreasing  = TRUE), -c(1:8)],
  caption = "Pairwise mtDNA differentiation between Sites",
  longtable = TRUE, booktabs = TRUE) %>%
  kableExtra::kable_styling(full_width = FALSE,font_size = 5,
                            latex_options = c("repeat_header", "hold_position"))


pop.levels1 <- c("Gulf of California","Costa Rica","Sierra Leone","South Africa",
                 "Reunion","Seychelles","Arabian Sea","Sri Lanka","Thailand",
                 "Indonesia","Papua New Guinea","Fitzroy River","Victoria River",
                 "Daly River","Adelaide River","South Alligator River",
                 "East Alligator River","Blue mud Bay","Roper River","Towns River",
                 "Wenlock River","Trinity inlet","Clarence River","Sydney Harbour",
                 "Okinawa","Urauchi River","Fiji")

m2 <- popstr.mt.Location$pairwise$result[order(match(
  popstr.mt.Location$pairwise$result$strata.1,pop.levels1)),c(1,2,9)]
Gdf <- igraph::graph.data.frame(m2, directed = FALSE)
m2.1 <- igraph::as_adjacency_matrix(Gdf, names = TRUE, sparse = FALSE,
                                    attr = "PHIst", type = 'upper')

m2 <- popstr.mt.Location$pairwise$result[order(match(
  popstr.mt.Location$pairwise$result$strata.1,pop.levels1)),c(1,2,10)]
Gdf <- igraph::graph.data.frame(m2, directed = FALSE)
m2.2 <- igraph::as_adjacency_matrix(Gdf, names = TRUE, sparse = FALSE,
```

Table 67: Overall mtDNA differentiation between Sites

| | estimate | p.val |
|---|---|---|
| Chi2 | 9334.0000 | 0.001 |
| Fst | 0.0000 | 1.000 |
| PHIst | 0.7537 | 0.001 |

```
                                    attr = "PHIst.p.val", type = 'upper')
m2$PHIst.p.adj <- p.adjust(m2$PHIst.p.val, "bonferroni")
Gdf <- igraph::graph.data.frame(m2, directed = FALSE)
m2.3 <- igraph::as_adjacency_matrix(Gdf, names = TRUE, sparse = FALSE,
                                    attr = "PHIst.p.adj", type = 'upper')
write.csv(m2.1, file = "Mitogenome_ALL_phist_Locations.csv")
write.csv(m2.2, file = "Mitogenome_ALL_phist_Pval_Locations.csv")
write.csv(m2.3, file = "Mitogenome_ALL_phist_Pval_Bonferroni_Locations.csv")
```

Table 68: Pairwise mtDNA differentiation between Sites

| | PHIst | PHIst.p.val |
|---|---|---|
| Gulf of California (1) v. Trinity inlet (4) | 1.0000000 | 0.2047952 |
| Sierra Leone (1) v. Trinity inlet (4) | 1.0000000 | 0.2147852 |
| Gulf of California (1) v. Urauchi River (21) | 0.9887413 | 0.0519481 |
| Sierra Leone (1) v. Urauchi River (21) | 0.9885876 | 0.0439560 |
| Blue mud Bay (13) v. Gulf of California (1) | 0.9884460 | 0.0709291 |
| Blue mud Bay (13) v. Sierra Leone (1) | 0.9882860 | 0.0969031 |
| Gulf of California (1) v. Roper River (4) | 0.9878215 | 0.2077922 |
| Roper River (4) v. Sierra Leone (1) | 0.9876525 | 0.2037962 |
| Gulf of California (1) v. Towns River (5) | 0.9854394 | 0.1518482 |
| Sierra Leone (1) v. Towns River (5) | 0.9852380 | 0.1718282 |
| Costa Rica (15) v. Urauchi River (21) | 0.9811117 | 0.0009990 |
| Blue mud Bay (13) v. Costa Rica (15) | 0.9785333 | 0.0009990 |
| Gulf of California (1) v. South Alligator River (38) | 0.9769065 | 0.0269730 |
| Sierra Leone (1) v. South Alligator River (38) | 0.9765779 | 0.0219780 |
| Costa Rica (15) v. Trinity inlet (4) | 0.9750733 | 0.0009990 |
| Costa Rica (15) v. South Alligator River (38) | 0.9748986 | 0.0009990 |
| Costa Rica (15) v. Towns River (5) | 0.9735751 | 0.0009990 |
| Costa Rica (15) v. Roper River (4) | 0.9732309 | 0.0019980 |
| Costa Rica (15) v. Reunion (16) | 0.9694116 | 0.0009990 |
| Gulf of California (1) v. Reunion (16) | 0.9684873 | 0.0679321 |
| Reunion (16) v. Sierra Leone (1) | 0.9684819 | 0.0529471 |
| Costa Rica (15) v. Seychelles (18) | 0.9684040 | 0.0009990 |
| Clarence River (21) v. Costa Rica (15) | 0.9676359 | 0.0009990 |
| Reunion (16) v. Urauchi River (21) | 0.9672442 | 0.0009990 |
| Gulf of California (1) v. Seychelles (18) | 0.9670436 | 0.0499500 |
| Seychelles (18) v. Sierra Leone (1) | 0.9670379 | 0.0569431 |
| Clarence River (21) v. Gulf of California (1) | 0.9662884 | 0.0499500 |
| Clarence River (21) v. Sierra Leone (1) | 0.9658398 | 0.0399600 |
| Costa Rica (15) v. Indonesia (6) | 0.9652015 | 0.0009990 |
| Seychelles (18) v. Urauchi River (21) | 0.9636613 | 0.0009990 |
| Blue mud Bay (13) v. Reunion (16) | 0.9631304 | 0.0009990 |
| Blue mud Bay (13) v. Seychelles (18) | 0.9591600 | 0.0009990 |
| Costa Rica (15) v. Victoria River (21) | 0.9587297 | 0.0009990 |
| Reunion (16) v. South Alligator River (38) | 0.9584980 | 0.0009990 |
| Costa Rica (15) v. Sierra Leone (1) | 0.9575420 | 0.0629371 |
| Costa Rica (15) v. Okinawa (10) | 0.9573514 | 0.0009990 |
| Reunion (16) v. Trinity inlet (4) | 0.9563031 | 0.0009990 |
| Costa Rica (15) v. East Alligator River (17) | 0.9559540 | 0.0009990 |
| Seychelles (18) v. South Alligator River (38) | 0.9553989 | 0.0009990 |
| Reunion (16) v. Towns River (5) | 0.9541431 | 0.0009990 |
| Costa Rica (15) v. Sydney Harbour (13) | 0.9541290 | 0.0009990 |
| Reunion (16) v. Roper River (4) | 0.9534421 | 0.0009990 |
| Seychelles (18) v. Trinity inlet (4) | 0.9515810 | 0.0009990 |
| Gulf of California (1) v. Indonesia (6) | 0.9511629 | 0.1458541 |
| Gulf of California (1) v. Victoria River (21) | 0.9509015 | 0.0439560 |
| Costa Rica (15) v. Papua New Guinea (15) | 0.9505300 | 0.0009990 |
| Indonesia (6) v. Sierra Leone (1) | 0.9504958 | 0.1268731 |
| Seychelles (18) v. Towns River (5) | 0.9502713 | 0.0009990 |
| Sierra Leone (1) v. Victoria River (21) | 0.9501907 | 0.0499500 |
| Roper River (4) v. Seychelles (18) | 0.9494001 | 0.0009990 |
| Costa Rica (15) v. Wenlock River (19) | 0.9489106 | 0.0009990 |
| Costa Rica (15) v. Daly River (25) | 0.9472102 | 0.0009990 |
| Clarence River (21) v. Reunion (16) | 0.9463779 | 0.0009990 |
| Adelaide River (18) v. Costa Rica (15) | 0.9443990 | 0.0009990 |
| East Alligator River (17) v. Gulf of California (1) | 0.9437660 | 0.0589411 |
| East Alligator River (17) v. Sierra Leone (1) | 0.9429891 | 0.0619381 |
| Clarence River (21) v. Seychelles (18) | 0.9428199 | 0.0009990 |
| Indonesia (6) v. Reunion (16) | 0.9408742 | 0.0009990 |
| Gulf of California (1) v. Okinawa (10) | 0.9374170 | 0.0909091 |
| Indonesia (6) v. Seychelles (18) | 0.9370692 | 0.0009990 |

Table 68: Pairwise mtDNA differentiation between Sites *(continued)*

| | PHIst | PHIst.p.val |
|---|---|---|
| Okinawa (10) v. Sierra Leone (1) | 0.9365667 | 0.0829171 |
| Gulf of California (1) v. Sydney Harbour (13) | 0.9358266 | 0.0799201 |
| Sierra Leone (1) v. Sydney Harbour (13) | 0.9349417 | 0.0589411 |
| Daly River (25) v. Gulf of California (1) | 0.9337473 | 0.0419580 |
| Reunion (16) v. Victoria River (21) | 0.9327623 | 0.0009990 |
| Daly River (25) v. Sierra Leone (1) | 0.9326885 | 0.0319680 |
| Gulf of California (1) v. Wenlock River (19) | 0.9324838 | 0.0469530 |
| Sierra Leone (1) v. Wenlock River (19) | 0.9314555 | 0.0559441 |
| Okinawa (10) v. Reunion (16) | 0.9311654 | 0.0009990 |
| Gulf of California (1) v. Papua New Guinea (15) | 0.9309788 | 0.0739261 |
| Papua New Guinea (15) v. Sierra Leone (1) | 0.9300344 | 0.0529471 |
| Seychelles (18) v. Victoria River (21) | 0.9294086 | 0.0009990 |
| East Alligator River (17) v. Reunion (16) | 0.9285024 | 0.0009990 |
| Okinawa (10) v. Seychelles (18) | 0.9280175 | 0.0009990 |
| East Alligator River (17) v. Seychelles (18) | 0.9251465 | 0.0009990 |
| Reunion (16) v. Sydney Harbour (13) | 0.9249207 | 0.0009990 |
| Costa Rica (15) v. Fiji (9) | 0.9246048 | 0.0009990 |
| Adelaide River (18) v. Gulf of California (1) | 0.9232640 | 0.0589411 |
| Adelaide River (18) v. Sierra Leone (1) | 0.9220914 | 0.0579421 |
| Seychelles (18) v. Sydney Harbour (13) | 0.9215933 | 0.0009990 |
| Papua New Guinea (15) v. Reunion (16) | 0.9190447 | 0.0009990 |
| Reunion (16) v. Wenlock River (19) | 0.9179158 | 0.0009990 |
| Papua New Guinea (15) v. Seychelles (18) | 0.9160519 | 0.0009990 |
| Daly River (25) v. Reunion (16) | 0.9152573 | 0.0009990 |
| Seychelles (18) v. Wenlock River (19) | 0.9146460 | 0.0009990 |
| Costa Rica (15) v. South Africa (7) | 0.9130404 | 0.0019980 |
| Daly River (25) v. Seychelles (18) | 0.9118562 | 0.0009990 |
| Roper River (4) v. Trinity inlet (4) | 0.9106151 | 0.0339660 |
| Adelaide River (18) v. Reunion (16) | 0.9105991 | 0.0009990 |
| Adelaide River (18) v. Seychelles (18) | 0.9073780 | 0.0009990 |
| South Africa (7) v. South Alligator River (38) | 0.8974237 | 0.0009990 |
| Costa Rica (15) v. Fitzroy River (5) | 0.8910443 | 0.0009990 |
| Costa Rica (15) v. Thailand (6) | 0.8866068 | 0.0009990 |
| Towns River (5) v. Trinity inlet (4) | 0.8846628 | 0.0039960 |
| South Africa (7) v. Urauchi River (21) | 0.8838649 | 0.0009990 |
| Blue mud Bay (13) v. Urauchi River (21) | 0.8834663 | 0.0009990 |
| Trinity inlet (4) v. Urauchi River (21) | 0.8820587 | 0.0009990 |
| Roper River (4) v. Urauchi River (21) | 0.8799386 | 0.0009990 |
| Towns River (5) v. Urauchi River (21) | 0.8792479 | 0.0009990 |
| Arabian Sea (19) v. Costa Rica (15) | 0.8773310 | 0.0009990 |
| Blue mud Bay (13) v. Trinity inlet (4) | 0.8729977 | 0.0019980 |
| Fiji (9) v. Reunion (16) | 0.8639407 | 0.0009990 |
| Fiji (9) v. Seychelles (18) | 0.8618021 | 0.0009990 |
| Clarence River (21) v. South Africa (7) | 0.8488479 | 0.0009990 |
| Fiji (9) v. Gulf of California (1) | 0.8483095 | 0.0889111 |
| Blue mud Bay (13) v. South Africa (7) | 0.8464529 | 0.0009990 |
| Fiji (9) v. Sierra Leone (1) | 0.8462238 | 0.0929071 |
| Costa Rica (15) v. Sri Lanka (12) | 0.8322144 | 0.0009990 |
| South Africa (7) v. Victoria River (21) | 0.8282895 | 0.0009990 |
| South Alligator River (38) v. Thailand (6) | 0.8250337 | 0.0009990 |
| Daly River (25) v. South Africa (7) | 0.8128966 | 0.0009990 |
| East Alligator River (17) v. South Africa (7) | 0.8081566 | 0.0009990 |
| Arabian Sea (19) v. Gulf of California (1) | 0.8037655 | 0.0559441 |
| South Alligator River (38) v. Urauchi River (21) | 0.8021153 | 0.0009990 |
| Arabian Sea (19) v. Sierra Leone (1) | 0.8019334 | 0.0469530 |
| South Africa (7) v. Wenlock River (19) | 0.7998318 | 0.0009990 |
| Sierra Leone (1) v. South Africa (7) | 0.7883953 | 0.1148851 |
| Gulf of California (1) v. South Africa (7) | 0.7880327 | 0.1358641 |
| Adelaide River (18) v. South Africa (7) | 0.7839000 | 0.0009990 |
| Papua New Guinea (15) v. South Africa (7) | 0.7819128 | 0.0009990 |
| South Africa (7) v. Sydney Harbour (13) | 0.7806569 | 0.0009990 |
| Thailand (6) v. Urauchi River (21) | 0.7801245 | 0.0009990 |
| Okinawa (10) v. South Africa (7) | 0.7771304 | 0.0009990 |
| Arabian Sea (19) v. Seychelles (18) | 0.7762695 | 0.0009990 |
| Arabian Sea (19) v. Reunion (16) | 0.7756795 | 0.0009990 |
| Blue mud Bay (13) v. Indonesia (6) | 0.7616878 | 0.0009990 |
| South Africa (7) v. Towns River (5) | 0.7565992 | 0.0009990 |
| Clarence River (21) v. Urauchi River (21) | 0.7503802 | 0.0009990 |
| Indonesia (6) v. South Africa (7) | 0.7474610 | 0.0019980 |
| Clarence River (21) v. Thailand (6) | 0.7412919 | 0.0009990 |
| South Africa (7) v. Trinity inlet (4) | 0.7407161 | 0.0049950 |
| Roper River (4) v. South Africa (7) | 0.7363390 | 0.0039960 |
| Blue mud Bay (13) v. Thailand (6) | 0.7294146 | 0.0009990 |
| South Alligator River (38) v. Trinity inlet (4) | 0.7202121 | 0.0009990 |
| Thailand (6) v. Victoria River (21) | 0.7132750 | 0.0009990 |
| Fitzroy River (5) v. Reunion (16) | 0.7125173 | 0.0009990 |
| Indonesia (6) v. South Alligator River (38) | 0.7053679 | 0.0009990 |
| South Alligator River (38) v. Sri Lanka (12) | 0.6920817 | 0.0009990 |
| Daly River (25) v. Thailand (6) | 0.6913071 | 0.0009990 |
| Blue mud Bay (13) v. Okinawa (10) | 0.6912234 | 0.0009990 |
| Okinawa (10) v. South Alligator River (38) | 0.6873925 | 0.0009990 |
| Fitzroy River (5) v. Seychelles (18) | 0.6784070 | 0.0049950 |
| Gulf of California (1) v. Thailand (6) | 0.6772203 | 0.1418581 |
| East Alligator River (17) v. Thailand (6) | 0.6765307 | 0.0009990 |

Table 68: Pairwise mtDNA differentiation between Sites *(continued)*

| | PHIst | PHIst.p.val |
|---|---|---|
| Sierra Leone (1) v. Thailand (6) | 0.6757185 | 0.1658342 |
| Indonesia (6) v. Towns River (5) | 0.6727657 | 0.0059940 |
| Urauchi River (21) v. Victoria River (21) | 0.6671736 | 0.0009990 |
| Thailand (6) v. Wenlock River (19) | 0.6658557 | 0.0009990 |
| Sydney Harbour (13) v. Urauchi River (21) | 0.6656183 | 0.0009990 |
| Fitzroy River (5) v. Urauchi River (21) | 0.6655974 | 0.0019980 |
| Gulf of California (1) v. Sri Lanka (12) | 0.6642238 | 0.0689311 |
| Sierra Leone (1) v. Sri Lanka (12) | 0.6622797 | 0.0749251 |
| Indonesia (6) v. Urauchi River (21) | 0.6598200 | 0.0009990 |
| Indonesia (6) v. Roper River (4) | 0.6513836 | 0.0079920 |
| Fitzroy River (5) v. Gulf of California (1) | 0.6491786 | 0.1648352 |
| Papua New Guinea (15) v. Thailand (6) | 0.6477381 | 0.0009990 |
| Blue mud Bay (13) v. Clarence River (21) | 0.6463417 | 0.0009990 |
| Fitzroy River (5) v. Sierra Leone (1) | 0.6462521 | 0.1728272 |
| Adelaide River (18) v. Thailand (6) | 0.6456381 | 0.0009990 |
| Fiji (9) v. South Africa (7) | 0.6425882 | 0.0029970 |
| Papua New Guinea (15) v. Urauchi River (21) | 0.6385181 | 0.0009990 |
| Sydney Harbour (13) v. Thailand (6) | 0.6381324 | 0.0009990 |
| Indonesia (6) v. Trinity inlet (4) | 0.6291095 | 0.0049950 |
| Okinawa (10) v. Thailand (6) | 0.6211585 | 0.0009990 |
| East Alligator River (17) v. Urauchi River (21) | 0.6152912 | 0.0009990 |
| Fitzroy River (5) v. South Alligator River (38) | 0.6109927 | 0.0069930 |
| Fiji (9) v. Urauchi River (21) | 0.6107598 | 0.0009990 |
| Sri Lanka (12) v. Urauchi River (21) | 0.6102716 | 0.0009990 |
| Reunion (16) v. Seychelles (18) | 0.6057351 | 0.0009990 |
| Clarence River (21) v. South Alligator River (38) | 0.6028123 | 0.0009990 |
| Okinawa (10) v. Towns River (5) | 0.5981897 | 0.0009990 |
| Clarence River (21) v. Towns River (5) | 0.5971462 | 0.0009990 |
| Fiji (9) v. South Alligator River (38) | 0.5935288 | 0.0009990 |
| Arabian Sea (19) v. South Africa (7) | 0.5897958 | 0.0019980 |
| Clarence River (21) v. Sri Lanka (12) | 0.5847829 | 0.0009990 |
| Thailand (6) v. Towns River (5) | 0.5803960 | 0.0249750 |
| Clarence River (21) v. Roper River (4) | 0.5782384 | 0.0009990 |
| Okinawa (10) v. Roper River (4) | 0.5735961 | 0.0049950 |
| Clarence River (21) v. Indonesia (6) | 0.5712502 | 0.0009990 |
| Clarence River (21) v. Okinawa (10) | 0.5695569 | 0.0009990 |
| Sri Lanka (12) v. Victoria River (21) | 0.5633338 | 0.0009990 |
| Blue mud Bay (13) v. Sri Lanka (12) | 0.5611878 | 0.0009990 |
| Okinawa (10) v. Urauchi River (21) | 0.5547461 | 0.0029970 |
| Daly River (25) v. Sri Lanka (12) | 0.5521694 | 0.0009990 |
| Okinawa (10) v. Trinity inlet (4) | 0.5505012 | 0.0029970 |
| Indonesia (6) v. Thailand (6) | 0.5499141 | 0.0139860 |
| Roper River (4) v. Thailand (6) | 0.5435733 | 0.0069930 |
| Clarence River (21) v. Fitzroy River (5) | 0.5430071 | 0.0009990 |
| Thailand (6) v. Trinity inlet (4) | 0.5393582 | 0.0069930 |
| Urauchi River (21) v. Wenlock River (19) | 0.5292057 | 0.0009990 |
| Blue mud Bay (13) v. Sydney Harbour (13) | 0.5284805 | 0.0009990 |
| South Alligator River (38) v. Sydney Harbour (13) | 0.5270156 | 0.0009990 |
| East Alligator River (17) v. Sri Lanka (12) | 0.5241172 | 0.0009990 |
| Sri Lanka (12) v. Wenlock River (19) | 0.5204537 | 0.0009990 |
| Reunion (16) v. Thailand (6) | 0.5202350 | 0.0009990 |
| Daly River (25) v. Urauchi River (21) | 0.5144132 | 0.0009990 |
| Adelaide River (18) v. Urauchi River (21) | 0.5143602 | 0.0009990 |
| Blue mud Bay (13) v. Fiji (9) | 0.5119905 | 0.0009990 |
| Papua New Guinea (15) v. Sri Lanka (12) | 0.5035981 | 0.0009990 |
| Trinity inlet (4) v. Victoria River (21) | 0.5021163 | 0.0009990 |
| Okinawa (10) v. Victoria River (21) | 0.5005285 | 0.0009990 |
| Adelaide River (18) v. Sri Lanka (12) | 0.5004452 | 0.0009990 |
| Sri Lanka (12) v. Sydney Harbour (13) | 0.4898373 | 0.0009990 |
| Papua New Guinea (15) v. South Alligator River (38) | 0.4858569 | 0.0009990 |
| Arabian Sea (19) v. South Alligator River (38) | 0.4830534 | 0.0009990 |
| Okinawa (10) v. Sri Lanka (12) | 0.4758559 | 0.0009990 |
| Blue mud Bay (13) v. Papua New Guinea (15) | 0.4748028 | 0.0009990 |
| Blue mud Bay (13) v. Fitzroy River (5) | 0.4691471 | 0.0049950 |
| Indonesia (6) v. Victoria River (21) | 0.4628799 | 0.0019980 |
| Reunion (16) v. Sri Lanka (12) | 0.4561799 | 0.0009990 |
| Fiji (9) v. Thailand (6) | 0.4506479 | 0.0019980 |
| East Alligator River (17) v. Trinity inlet (4) | 0.4491245 | 0.0009990 |
| Okinawa (10) v. Sydney Harbour (13) | 0.4461679 | 0.0009990 |
| Clarence River (21) v. Victoria River (21) | 0.4404689 | 0.0009990 |
| Fitzroy River (5) v. Victoria River (21) | 0.4389129 | 0.0269730 |
| East Alligator River (17) v. Okinawa (10) | 0.4353099 | 0.0009990 |
| Sri Lanka (12) v. Towns River (5) | 0.4340831 | 0.0059940 |
| Daly River (25) v. Fitzroy River (5) | 0.4331509 | 0.0079920 |
| Fitzroy River (5) v. Okinawa (10) | 0.4312088 | 0.0009990 |
| Papua New Guinea (15) v. Trinity inlet (4) | 0.4287198 | 0.0009990 |
| Sydney Harbour (13) v. Towns River (5) | 0.4276393 | 0.0019980 |
| Clarence River (21) v. Papua New Guinea (15) | 0.4271172 | 0.0009990 |
| Indonesia (6) v. Okinawa (10) | 0.4246671 | 0.0129870 |
| Indonesia (6) v. Papua New Guinea (15) | 0.4237095 | 0.0009990 |
| Okinawa (10) v. Papua New Guinea (15) | 0.4109033 | 0.0009990 |
| Clarence River (21) v. East Alligator River (17) | 0.4107713 | 0.0009990 |
| Fiji (9) v. Victoria River (21) | 0.4101916 | 0.0009990 |
| Indonesia (6) v. Sydney Harbour (13) | 0.4093502 | 0.0009990 |

Table 68: Pairwise mtDNA differentiation between Sites *(continued)*

| | PHIst | PHIst.p.val |
|---|---|---|
| Fitzroy River (5) v. Papua New Guinea (15) | 0.4048663 | 0.0039960 |
| Fitzroy River (5) v. Wenlock River (19) | 0.4047859 | 0.0089910 |
| East Alligator River (17) v. Fitzroy River (5) | 0.4039457 | 0.0289710 |
| Roper River (4) v. Sri Lanka (12) | 0.4038690 | 0.0229770 |
| Seychelles (18) v. Thailand (6) | 0.3993580 | 0.0009990 |
| Fitzroy River (5) v. Sydney Harbour (13) | 0.3990371 | 0.0009990 |
| Seychelles (18) v. Sri Lanka (12) | 0.3954333 | 0.0009990 |
| Roper River (4) v. Sydney Harbour (13) | 0.3935885 | 0.0009990 |
| Sri Lanka (12) v. Trinity inlet (4) | 0.3926234 | 0.0169830 |
| Arabian Sea (19) v. Blue mud Bay (13) | 0.3921366 | 0.0009990 |
| Indonesia (6) v. Sri Lanka (12) | 0.3909716 | 0.0339660 |
| East Alligator River (17) v. Indonesia (6) | 0.3893668 | 0.0009990 |
| Fiji (9) v. Okinawa (10) | 0.3891665 | 0.0009990 |
| Arabian Sea (19) v. Thailand (6) | 0.3865570 | 0.0059940 |
| Daly River (25) v. Okinawa (10) | 0.3861823 | 0.0009990 |
| Papua New Guinea (15) v. Towns River (5) | 0.3796237 | 0.0019980 |
| Okinawa (10) v. Wenlock River (19) | 0.3779682 | 0.0009990 |
| Daly River (25) v. Trinity inlet (4) | 0.3768918 | 0.0009990 |
| Adelaide River (18) v. Fitzroy River (5) | 0.3750436 | 0.0219780 |
| Fiji (9) v. Towns River (5) | 0.3671478 | 0.0009990 |
| Clarence River (21) v. Daly River (25) | 0.3659189 | 0.0009990 |
| East Alligator River (17) v. Fiji (9) | 0.3631261 | 0.0009990 |
| Arabian Sea (19) v. Clarence River (21) | 0.3615792 | 0.0009990 |
| Daly River (25) v. Fiji (9) | 0.3583998 | 0.0009990 |
| Trinity inlet (4) v. Wenlock River (19) | 0.3573103 | 0.0009990 |
| Adelaide River (18) v. Okinawa (10) | 0.3564518 | 0.0009990 |
| Clarence River (21) v. Wenlock River (19) | 0.3479436 | 0.0009990 |
| Fitzroy River (5) v. Indonesia (6) | 0.3454361 | 0.0019980 |
| Papua New Guinea (15) v. Roper River (4) | 0.3425044 | 0.0049950 |
| Fiji (9) v. Papua New Guinea (15) | 0.3391077 | 0.0009990 |
| Adelaide River (18) v. Clarence River (21) | 0.3375021 | 0.0009990 |
| Adelaide River (18) v. Trinity inlet (4) | 0.3346570 | 0.0019980 |
| Fiji (9) v. Sri Lanka (12) | 0.3337432 | 0.0039960 |
| Blue mud Bay (13) v. Towns River (5) | 0.3311125 | 0.0069930 |
| Fiji (9) v. Wenlock River (19) | 0.3279948 | 0.0009990 |
| Fiji (9) v. Roper River (4) | 0.3264978 | 0.0059940 |
| Arabian Sea (19) v. Urauchi River (21) | 0.3259254 | 0.0009990 |
| Arabian Sea (19) v. Victoria River (21) | 0.3223922 | 0.0009990 |
| Fiji (9) v. Indonesia (6) | 0.3212353 | 0.0009990 |
| Daly River (25) v. Indonesia (6) | 0.3204187 | 0.0009990 |
| Sydney Harbour (13) v. Victoria River (21) | 0.3169697 | 0.0009990 |
| Adelaide River (18) v. Fiji (9) | 0.3118569 | 0.0009990 |
| Indonesia (6) v. Wenlock River (19) | 0.3078026 | 0.0009990 |
| Blue mud Bay (13) v. Roper River (4) | 0.2955472 | 0.0169830 |
| East Alligator River (17) v. Sydney Harbour (13) | 0.2940437 | 0.0009990 |
| Arabian Sea (19) v. Sri Lanka (12) | 0.2920615 | 0.0019980 |
| Papua New Guinea (15) v. Victoria River (21) | 0.2898890 | 0.0009990 |
| Fitzroy River (5) v. Trinity inlet (4) | 0.2881883 | 0.1938062 |
| Arabian Sea (19) v. Towns River (5) | 0.2872795 | 0.0189810 |
| Arabian Sea (19) v. Papua New Guinea (15) | 0.2857166 | 0.0009990 |
| East Alligator River (17) v. Papua New Guinea (15) | 0.2770597 | 0.0009990 |
| Fitzroy River (5) v. South Africa (7) | 0.2758936 | 0.0859141 |
| Arabian Sea (19) v. Sydney Harbour (13) | 0.2715370 | 0.0009990 |
| Blue mud Bay (13) v. Daly River (25) | 0.2681789 | 0.0039960 |
| Daly River (25) v. Sydney Harbour (13) | 0.2588976 | 0.0009990 |
| Papua New Guinea (15) v. Wenlock River (19) | 0.2565555 | 0.0009990 |
| Arabian Sea (19) v. Roper River (4) | 0.2562320 | 0.0759241 |
| Arabian Sea (19) v. Okinawa (10) | 0.2557410 | 0.0049950 |
| Arabian Sea (19) v. East Alligator River (17) | 0.2555895 | 0.0009990 |
| Fitzroy River (5) v. Towns River (5) | 0.2555059 | 0.0039960 |
| Adelaide River (18) v. Indonesia (6) | 0.2553233 | 0.0109890 |
| Sydney Harbour (13) v. Wenlock River (19) | 0.2551439 | 0.0009990 |
| Arabian Sea (19) v. Daly River (25) | 0.2519896 | 0.0009990 |
| Daly River (25) v. Papua New Guinea (15) | 0.2452140 | 0.0009990 |
| Blue mud Bay (13) v. East Alligator River (17) | 0.2450737 | 0.0029970 |
| Blue mud Bay (13) v. Wenlock River (19) | 0.2355891 | 0.0029970 |
| Adelaide River (18) v. Blue mud Bay (13) | 0.2343158 | 0.0019980 |
| South Alligator River (38) v. Wenlock River (19) | 0.2331459 | 0.0009990 |
| Arabian Sea (19) v. Trinity inlet (4) | 0.2277714 | 0.0589411 |
| Arabian Sea (19) v. Wenlock River (19) | 0.2265136 | 0.0009990 |
| Adelaide River (18) v. Arabian Sea (19) | 0.2244192 | 0.0009990 |
| Adelaide River (18) v. South Alligator River (38) | 0.2225027 | 0.0009990 |
| Arabian Sea (19) v. Fiji (9) | 0.2193626 | 0.0109890 |
| Reunion (16) v. South Africa (7) | 0.2107817 | 0.0049950 |
| Fiji (9) v. Fitzroy River (5) | 0.2099277 | 0.0299700 |
| Daly River (25) v. South Alligator River (38) | 0.2060549 | 0.0009990 |
| Arabian Sea (19) v. Fitzroy River (5) | 0.2025943 | 0.0419580 |
| Fitzroy River (5) v. Roper River (4) | 0.1973826 | 0.2187812 |
| Daly River (25) v. Towns River (5) | 0.1944328 | 0.0269730 |
| Clarence River (21) v. Sydney Harbour (13) | 0.1879409 | 0.0049950 |
| Sydney Harbour (13) v. Trinity inlet (4) | 0.1864946 | 0.1348651 |
| Adelaide River (18) v. Sydney Harbour (13) | 0.1842047 | 0.0019980 |
| Clarence River (21) v. Fiji (9) | 0.1784404 | 0.0029970 |
| Fiji (9) v. Sydney Harbour (13) | 0.1783542 | 0.0019980 |

Table 68: Pairwise mtDNA differentiation between Sites *(continued)*

| | PHIst | PHIst.p.val |
|---|---|---|
| Towns River (5) v. Wenlock River (19) | 0.1748255 | 0.0489510 |
| East Alligator River (17) v. Towns River (5) | 0.1626439 | 0.0699301 |
| Seychelles (18) v. South Africa (7) | 0.1586276 | 0.0279720 |
| Adelaide River (18) v. Towns River (5) | 0.1562088 | 0.0269730 |
| Adelaide River (18) v. Papua New Guinea (15) | 0.1528138 | 0.0069930 |
| South Alligator River (38) v. Towns River (5) | 0.1480850 | 0.0289710 |
| East Alligator River (17) v. South Alligator River (38) | 0.1477612 | 0.0029970 |
| Blue mud Bay (13) v. South Alligator River (38) | 0.1468654 | 0.0019980 |
| Daly River (25) v. Roper River (4) | 0.1454959 | 0.0799201 |
| Arabian Sea (19) v. Indonesia (6) | 0.1349157 | 0.1158841 |
| Blue mud Bay (13) v. Victoria River (21) | 0.1326374 | 0.0089910 |
| Roper River (4) v. Wenlock River (19) | 0.1319979 | 0.1258741 |
| Costa Rica (15) v. Gulf of California (1) | 0.1242849 | 0.1818182 |
| Papua New Guinea (15) v. Sydney Harbour (13) | 0.1162714 | 0.0229770 |
| South Africa (7) v. Sri Lanka (12) | 0.1128273 | 0.1288711 |
| Adelaide River (18) v. Roper River (4) | 0.1096056 | 0.1418581 |
| East Alligator River (17) v. Roper River (4) | 0.1027974 | 0.1088911 |
| Towns River (5) v. Victoria River (21) | 0.0734100 | 0.0749251 |
| Victoria River (21) v. Wenlock River (19) | 0.0692990 | 0.0379620 |
| Daly River (25) v. Victoria River (21) | 0.0634446 | 0.0489510 |
| Roper River (4) v. South Alligator River (38) | 0.0616557 | 0.2297702 |
| South Africa (7) v. Thailand (6) | 0.0484505 | 0.1698302 |
| East Alligator River (17) v. Wenlock River (19) | 0.0435785 | 0.1158841 |
| South Alligator River (38) v. Victoria River (21) | 0.0423776 | 0.0409590 |
| Adelaide River (18) v. Victoria River (21) | 0.0387443 | 0.1238761 |
| Fitzroy River (5) v. Thailand (6) | 0.0298249 | 0.2457542 |
| East Alligator River (17) v. Victoria River (21) | 0.0297948 | 0.1508492 |
| Daly River (25) v. Wenlock River (19) | 0.0290248 | 0.1338661 |
| Adelaide River (18) v. East Alligator River (17) | 0.0281864 | 0.1558442 |
| Adelaide River (18) v. Daly River (25) | 0.0249065 | 0.1658342 |
| Adelaide River (18) v. Wenlock River (19) | 0.0178644 | 0.1848152 |
| Roper River (4) v. Victoria River (21) | 0.0085205 | 0.2727273 |
| Daly River (25) v. East Alligator River (17) | 0.0045904 | 0.2987013 |
| Fitzroy River (5) v. Sri Lanka (12) | -0.0031832 | 0.2847153 |
| Fiji (9) v. Trinity inlet (4) | -0.0134690 | 0.3066933 |
| Clarence River (21) v. Trinity inlet (4) | -0.0463369 | 0.4815185 |
| Sri Lanka (12) v. Thailand (6) | -0.0912389 | 0.6243756 |
| Roper River (4) v. Towns River (5) | -0.2264393 | 0.9950050 |
| Gulf of California (1) v. Sierra Leone (1) | NA | NA |

### 13.5.2 PHIst per Site

```
strata <- as.character(BS.gt@schemes$Site)
names(strata) <- as.character(BS.gt@schemes$id)
strataG::setStrata(BS.gt) <- strata

fxdiff <- strataG::fixedDifferences(BS.gt, count.indels = TRUE,
                                    consec.indels.as.one = TRUE,
  bases = c("a", "c", "g", "t", "-"))
knitr::kable(
  fxdiff$num.fixed[order(fxdiff$num.fixed$num.fixed, decreasing = TRUE),],
  # format = "html",
  caption = "Fixed mtDNA differences between Sites"
  ) %>%
  kableExtra::kable_styling(full_width = FALSE,font_size = 5)
```

```
popstr.mt.Site <-
  strataG::popStructTest(
    BS.gt,
    nrep = 1000,
    stats = "all",
    type = "both",
    keep.null = FALSE,
    quietly = TRUE,
    max.cores = parallel::detectCores()-1,
    write.output = TRUE
  )
save(popstr.mt.Site, file = "Mitogenome_ALL_sites_phist.Rdata")
```

Table 69: Fixed mtDNA differences between Sites

| | strata.1 | strata.2 | num.fixed |
|---|---|---|---|
| 50 | Gulf of California | Reunion | 146 |
| 51 | Gulf of California | Seychelles | 146 |
| 78 | Reunion | Sierra Leone | 145 |
| 82 | Seychelles | Sierra Leone | 145 |
| 47 | Gulf of California | Indonesia | 144 |
| 48 | Gulf of California | Japan | 143 |
| 31 | Costa Rica | Reunion | 142 |
| 32 | Costa Rica | Seychelles | 142 |
| 49 | Gulf of California | Papua New Guinea | 142 |
| 60 | Indonesia | Sierra Leone | 141 |
| 67 | Japan | Sierra Leone | 141 |
| 28 | Costa Rica | Indonesia | 140 |
| 29 | Costa Rica | Japan | 139 |
| 73 | Papua New Guinea | Sierra Leone | 139 |
| 30 | Costa Rica | Papua New Guinea | 138 |
| 53 | Gulf of California | South Africa | 113 |
| 86 | Sierra Leone | South Africa | 112 |
| 34 | Costa Rica | South Africa | 109 |
| 52 | Gulf of California | Sierra Leone | 107 |
| 55 | Gulf of California | Thailand | 107 |
| 37 | Fiji | Gulf of California | 106 |
| 54 | Gulf of California | Sri Lanka | 106 |
| 16 | Australia | Gulf of California | 105 |
| 88 | Sierra Leone | Thailand | 105 |
| 4 | Arabian Sea | Gulf of California | 104 |
| 22 | Australia | Sierra Leone | 104 |
| 87 | Sierra Leone | Sri Lanka | 104 |
| 10 | Arabian Sea | Sierra Leone | 103 |
| 33 | Costa Rica | Sierra Leone | 103 |
| 43 | Fiji | Sierra Leone | 103 |
| 26 | Costa Rica | Fiji | 102 |
| 35 | Costa Rica | Sri Lanka | 102 |
| 36 | Costa Rica | Thailand | 102 |
| 14 | Australia | Costa Rica | 101 |
| 2 | Arabian Sea | Costa Rica | 100 |
| 65 | Japan | Reunion | 78 |
| 66 | Japan | Seychelles | 78 |
| 58 | Indonesia | Reunion | 76 |
| 59 | Indonesia | Seychelles | 76 |
| 71 | Papua New Guinea | Reunion | 75 |
| 72 | Papua New Guinea | Seychelles | 75 |
| 68 | Japan | South Africa | 12 |
| 61 | Indonesia | South Africa | 11 |
| 74 | Papua New Guinea | South Africa | 10 |
| 27 | Costa Rica | Gulf of California | 2 |
| 20 | Australia | Reunion | 1 |
| 21 | Australia | Seychelles | 1 |
| 39 | Fiji | Japan | 1 |
| 1 | Arabian Sea | Australia | 0 |
| 3 | Arabian Sea | Fiji | 0 |
| 5 | Arabian Sea | Indonesia | 0 |
| 6 | Arabian Sea | Japan | 0 |
| 7 | Arabian Sea | Papua New Guinea | 0 |
| 8 | Arabian Sea | Reunion | 0 |
| 9 | Arabian Sea | Seychelles | 0 |
| 11 | Arabian Sea | South Africa | 0 |
| 12 | Arabian Sea | Sri Lanka | 0 |
| 13 | Arabian Sea | Thailand | 0 |
| 15 | Australia | Fiji | 0 |
| 17 | Australia | Indonesia | 0 |
| 18 | Australia | Japan | 0 |
| 19 | Australia | Papua New Guinea | 0 |
| 23 | Australia | South Africa | 0 |
| 24 | Australia | Sri Lanka | 0 |
| 25 | Australia | Thailand | 0 |
| 38 | Fiji | Indonesia | 0 |
| 40 | Fiji | Papua New Guinea | 0 |
| 41 | Fiji | Reunion | 0 |
| 42 | Fiji | Seychelles | 0 |
| 44 | Fiji | South Africa | 0 |
| 45 | Fiji | Sri Lanka | 0 |
| 46 | Fiji | Thailand | 0 |
| 56 | Indonesia | Japan | 0 |
| 57 | Indonesia | Papua New Guinea | 0 |
| 62 | Indonesia | Sri Lanka | 0 |
| 63 | Indonesia | Thailand | 0 |
| 64 | Japan | Papua New Guinea | 0 |
| 69 | Japan | Sri Lanka | 0 |
| 70 | Japan | Thailand | 0 |
| 75 | Papua New Guinea | Sri Lanka | 0 |
| 76 | Papua New Guinea | Thailand | 0 |
| 77 | Reunion | Seychelles | 0 |
| 79 | Reunion | South Africa | 0 |
| 80 | Reunion | Sri Lanka | 0 |
| 81 | Reunion | Thailand | 0 |
| 83 | Seychelles | South Africa | 0 |
| 84 | Seychelles | Sri Lanka | 0 |
| 85 | Seychelles | Thailand | 0 |
| 89 | South Africa | Sri Lanka | 0 |
| 90 | South Africa | Thailand | 0 |
| 91 | Sri Lanka | Thailand | 0 |

Table 70: Overall mtDNA differentiation between Sites

| | estimate | p.val |
|---|---|---|
| Chi2 | 4667.0000 | 0.987 |
| Fst | 0.0000 | 1.000 |
| PHIst | 0.7858 | 0.001 |

```
readr::write_tsv(popstr.mt.Site$pairwise$result,
                 path = "Mitogenome_ALL_sites_phist.txt")
```

```
load("Mitogenome_ALL_sites_phist.Rdata")
knitr::kable(
  popstr.mt.Site$overall$result,
  digits = 4,
  # format = "html",
  caption = "Overall mtDNA differentiation between Sites"
  ) %>%
  kableExtra::kable_styling(full_width = FALSE,font_size = 5)
knitr::kable(
  popstr.mt.Site$pairwise$result[order(popstr.mt.Site$pairwise$result$PHIst,
                                        decreasing  = TRUE), -c(1:8)],
  # format = "html",
  digits = 4,
  caption = "Pairwise mtDNA differentiation between Sites"
  ) %>%
  kableExtra::kable_styling(full_width = FALSE,font_size = 5)
```

### 13.5.3 PHIst per region

```
strata <- as.character(BS.gt@schemes$Region)
names(strata) <- as.character(BS.gt@schemes$id)
strataG::setStrata(BS.gt) <- strata

fxdiff <- strataG::fixedDifferences(BS.gt, count.indels = TRUE,
                                    consec.indels.as.one = TRUE,
                                    bases = c("a", "c", "g", "t", "-"))
knitr::kable(
  fxdiff$num.fixed[order(fxdiff$num.fixed$num.fixed, decreasing = TRUE), ],
  # format = "html",
  caption = "Fixed mtDNA differences between regions"
  ) %>%
  kableExtra::kable_styling(full_width = FALSE,font_size = 5)
```

```
popstr.mt.pop <-
  strataG::popStructTest(
    BS.gt,
    nrep = 10000,
    stats = "all",
    type = "both",
    keep.null = FALSE,
    quietly = TRUE,
    max.cores = parallel::detectCores()-1,
    write.output = TRUE
  )
save(popstr.mt.pop, file = "Mitogenome_ALL_region_phist.Rdata")
readr::write_tsv(popstr.mt.Site$pairwise$result,
                 path = "Mitogenome_ALL_region_phist.txt")
```

## Table 71: Pairwise mtDNA differentiation between Sites

| | PHIst | PHIst.p.val |
|---|---:|---:|
| Costa Rica (15) v. Reunion (16) | 0.9694 | 0.0010 |
| Gulf of California (1) v. Reunion (16) | 0.9685 | 0.0699 |
| Reunion (16) v. Sierra Leone (1) | 0.9685 | 0.0679 |
| Costa Rica (15) v. Seychelles (18) | 0.9684 | 0.0010 |
| Gulf of California (1) v. Seychelles (18) | 0.9670 | 0.0480 |
| Seychelles (18) v. Sierra Leone (1) | 0.9670 | 0.0559 |
| Costa Rica (15) v. Indonesia (6) | 0.9652 | 0.0010 |
| Costa Rica (15) v. Japan (31) | 0.9615 | 0.0010 |
| Costa Rica (15) v. Sierra Leone (1) | 0.9575 | 0.0470 |
| Gulf of California (1) v. Japan (31) | 0.9574 | 0.0310 |
| Japan (31) v. Sierra Leone (1) | 0.9568 | 0.0410 |
| Gulf of California (1) v. Indonesia (6) | 0.9512 | 0.1598 |
| Costa Rica (15) v. Papua New Guinea (15) | 0.9505 | 0.0010 |
| Indonesia (6) v. Sierra Leone (1) | 0.9505 | 0.1449 |
| Indonesia (6) v. Reunion (16) | 0.9409 | 0.0010 |
| Japan (31) v. Reunion (16) | 0.9372 | 0.0010 |
| Indonesia (6) v. Seychelles (18) | 0.9371 | 0.0010 |
| Japan (31) v. Seychelles (18) | 0.9339 | 0.0010 |
| Australia (203) v. Costa Rica (15) | 0.9336 | 0.0010 |
| Gulf of California (1) v. Papua New Guinea (15) | 0.9310 | 0.0689 |
| Australia (203) v. Gulf of California (1) | 0.9303 | 0.0020 |
| Papua New Guinea (15) v. Sierra Leone (1) | 0.9300 | 0.0729 |
| Australia (203) v. Sierra Leone (1) | 0.9293 | 0.0050 |
| Costa Rica (15) v. Fiji (9) | 0.9246 | 0.0010 |
| Papua New Guinea (15) v. Reunion (16) | 0.9190 | 0.0010 |
| Papua New Guinea (15) v. Seychelles (18) | 0.9161 | 0.0010 |
| Costa Rica (15) v. South Africa (7) | 0.9130 | 0.0010 |
| Australia (203) v. Reunion (16) | 0.8936 | 0.0010 |
| Australia (203) v. Seychelles (18) | 0.8885 | 0.0010 |
| Costa Rica (15) v. Thailand (6) | 0.8866 | 0.0010 |
| Arabian Sea (19) v. Costa Rica (15) | 0.8773 | 0.0010 |
| Fiji (9) v. Reunion (16) | 0.8639 | 0.0010 |
| Fiji (9) v. Seychelles (18) | 0.8618 | 0.0010 |
| Japan (31) v. South Africa (7) | 0.8587 | 0.0010 |
| Fiji (9) v. Gulf of California (1) | 0.8483 | 0.1069 |
| Australia (203) v. South Africa (7) | 0.8474 | 0.0010 |
| Fiji (9) v. Sierra Leone (1) | 0.8462 | 0.0969 |
| Costa Rica (15) v. Sri Lanka (12) | 0.8322 | 0.0010 |
| Arabian Sea (19) v. Gulf of California (1) | 0.8038 | 0.0529 |
| Arabian Sea (19) v. Sierra Leone (1) | 0.8019 | 0.0529 |
| Sierra Leone (1) v. South Africa (7) | 0.7884 | 0.1469 |
| Gulf of California (1) v. South Africa (7) | 0.7880 | 0.1349 |
| Papua New Guinea (15) v. South Africa (7) | 0.7819 | 0.0010 |
| Arabian Sea (19) v. Seychelles (18) | 0.7763 | 0.0010 |
| Arabian Sea (19) v. Reunion (16) | 0.7757 | 0.0010 |
| Australia (203) v. Thailand (6) | 0.7673 | 0.0010 |
| Japan (31) v. Thailand (6) | 0.7551 | 0.0010 |
| Indonesia (6) v. South Africa (7) | 0.7475 | 0.0020 |
| Australia (203) v. Sri Lanka (12) | 0.6938 | 0.0010 |
| Gulf of California (1) v. Thailand (6) | 0.6772 | 0.1499 |
| Sierra Leone (1) v. Thailand (6) | 0.6757 | 0.1588 |
| Gulf of California (1) v. Sri Lanka (12) | 0.6642 | 0.0669 |
| Sierra Leone (1) v. Sri Lanka (12) | 0.6623 | 0.0839 |
| Papua New Guinea (15) v. Thailand (6) | 0.6477 | 0.0010 |
| Fiji (9) v. South Africa (7) | 0.6426 | 0.0010 |
| Japan (31) v. Sri Lanka (12) | 0.6144 | 0.0010 |
| Reunion (16) v. Seychelles (18) | 0.6057 | 0.0010 |
| Arabian Sea (19) v. South Africa (7) | 0.5898 | 0.0010 |
| Indonesia (6) v. Thailand (6) | 0.5499 | 0.0220 |
| Reunion (16) v. Thailand (6) | 0.5202 | 0.0010 |
| Fiji (9) v. Japan (31) | 0.5153 | 0.0010 |
| Papua New Guinea (15) v. Sri Lanka (12) | 0.5036 | 0.0010 |
| Japan (31) v. Papua New Guinea (15) | 0.4933 | 0.0010 |
| Reunion (16) v. Sri Lanka (12) | 0.4562 | 0.0010 |
| Fiji (9) v. Thailand (6) | 0.4506 | 0.0030 |
| Indonesia (6) v. Papua New Guinea (15) | 0.4237 | 0.0010 |
| Indonesia (6) v. Japan (31) | 0.4090 | 0.0050 |
| Seychelles (18) v. Thailand (6) | 0.3994 | 0.0020 |
| Australia (203) v. Japan (31) | 0.3989 | 0.0010 |
| Seychelles (18) v. Sri Lanka (12) | 0.3954 | 0.0010 |
| Arabian Sea (19) v. Australia (203) | 0.3925 | 0.0010 |
| Indonesia (6) v. Sri Lanka (12) | 0.3910 | 0.0290 |
| Arabian Sea (19) v. Thailand (6) | 0.3866 | 0.0090 |
| Australia (203) v. Fiji (9) | 0.3641 | 0.0010 |
| Australia (203) v. Indonesia (6) | 0.3433 | 0.0030 |
| Fiji (9) v. Papua New Guinea (15) | 0.3391 | 0.0010 |
| Fiji (9) v. Sri Lanka (12) | 0.3337 | 0.0060 |
| Fiji (9) v. Indonesia (6) | 0.3212 | 0.0020 |
| Arabian Sea (19) v. Japan (31) | 0.3063 | 0.0010 |
| Arabian Sea (19) v. Sri Lanka (12) | 0.2921 | 0.0060 |
| Arabian Sea (19) v. Papua New Guinea (15) | 0.2857 | 0.0010 |
| Arabian Sea (19) v. Fiji (9) | 0.2194 | 0.0090 |
| Australia (203) v. Papua New Guinea (15) | 0.2187 | 0.0010 |
| Reunion (16) v. South Africa (7) | 0.2108 | 0.0060 |
| Seychelles (18) v. South Africa (7) | 0.1586 | 0.0230 |
| Arabian Sea (19) v. Indonesia (6) | 0.1349 | 0.1379 |
| Costa Rica (15) v. Gulf of California (1) | 0.1243 | 0.1588 |
| South Africa (7) v. Sri Lanka (12) | 0.1128 | 0.1209 |
| South Africa (7) v. Thailand (6) | 0.0485 | 0.1928 |
| Sri Lanka (12) v. Thailand (6) | -0.0912 | 0.6553 |
| Gulf of California (1) v. Sierra Leone (1) | NA | NA |

Table 72: Fixed mtDNA differences between regions

| | strata.1 | strata.2 | num.fixed |
|---|---|---|---|
| 4 | E-ATL | Japan | 141 |
| 7 | E-ATL | W-PAC | 140 |
| 15 | E-PAC | Japan | 139 |
| 18 | E-PAC | W-PAC | 138 |
| 6 | E-ATL | W-IO | 112 |
| 17 | E-PAC | W-IO | 109 |
| 1 | E-ATL | E-IO | 103 |
| 2 | E-ATL | E-PAC | 103 |
| 3 | E-ATL | Fiji | 103 |
| 5 | E-ATL | N-IO | 103 |
| 14 | E-PAC | Fiji | 102 |
| 8 | E-IO | E-PAC | 101 |
| 16 | E-PAC | N-IO | 100 |
| 24 | Japan | W-IO | 12 |
| 28 | W-IO | W-PAC | 9 |
| 19 | Fiji | Japan | 1 |
| 9 | E-IO | Fiji | 0 |
| 10 | E-IO | Japan | 0 |
| 11 | E-IO | N-IO | 0 |
| 12 | E-IO | W-IO | 0 |
| 13 | E-IO | W-PAC | 0 |
| 20 | Fiji | N-IO | 0 |
| 21 | Fiji | W-IO | 0 |
| 22 | Fiji | W-PAC | 0 |
| 23 | Japan | N-IO | 0 |
| 25 | Japan | W-PAC | 0 |
| 26 | N-IO | W-IO | 0 |
| 27 | N-IO | W-PAC | 0 |

Table 73: Overall mtDNA differentiation between regions

| | estimate | p.val |
|---|---|---|
| Chi2 | 2513.0000 | 0.3916 |
| Fst | 0.0000 | 1.0000 |
| PHIst | 0.7576 | 0.0001 |

```r
load("Mitogenome_ALL_region_phist.Rdata")
knitr::kable(
  popstr.mt.pop$overall$result,
  digits = 4,
  # format = "html",
  caption = "Overall mtDNA differentiation between regions"
  ) %>%
  kableExtra::kable_styling(full_width = FALSE,font_size = 5)
knitr::kable(
  popstr.mt.pop$pairwise$result[order(popstr.mt.pop$pairwise$result$PHIst,
  decreasing = TRUE), -c(1:8)],
  # format = "html",
  digits = 4,
  caption = "Pairwise mtDNA differentiation between regions"
  ) %>%
  kableExtra::kable_styling(full_width = FALSE,font_size = 5)
```

Table 74: Pairwise mtDNA differentiation between regions

| | PHIst | PHIst.p.val |
|---|---|---|
| E-PAC (16) v. Japan (31) | 0.9615 | 0.0001 |
| E-PAC (16) v. W-PAC (38) | 0.9583 | 0.0001 |
| E-ATL (1) v. Japan (31) | 0.9568 | 0.0305 |
| E-ATL (1) v. E-PAC (16) | 0.9568 | 0.0582 |
| E-ATL (1) v. W-PAC (38) | 0.9532 | 0.0228 |
| E-IO (186) v. E-PAC (16) | 0.9334 | 0.0001 |
| E-PAC (16) v. W-IO (41) | 0.9314 | 0.0001 |
| E-ATL (1) v. E-IO (186) | 0.9286 | 0.0057 |
| E-PAC (16) v. Fiji (9) | 0.9261 | 0.0001 |
| E-ATL (1) v. W-IO (41) | 0.9163 | 0.0233 |
| Japan (31) v. W-IO (41) | 0.8892 | 0.0001 |
| W-IO (41) v. W-PAC (38) | 0.8878 | 0.0001 |
| E-IO (186) v. W-IO (41) | 0.8754 | 0.0001 |
| E-ATL (1) v. Fiji (9) | 0.8462 | 0.0993 |
| Fiji (9) v. W-IO (41) | 0.8153 | 0.0001 |
| E-PAC (16) v. N-IO (37) | 0.7789 | 0.0001 |
| E-ATL (1) v. N-IO (37) | 0.6815 | 0.0266 |
| Japan (31) v. W-PAC (38) | 0.5398 | 0.0001 |
| N-IO (37) v. W-IO (41) | 0.5162 | 0.0001 |
| Fiji (9) v. Japan (31) | 0.5153 | 0.0001 |
| E-IO (186) v. N-IO (37) | 0.4620 | 0.0001 |
| E-IO (186) v. Fiji (9) | 0.4045 | 0.0002 |
| E-IO (186) v. Japan (31) | 0.3972 | 0.0001 |
| N-IO (37) v. W-PAC (38) | 0.3628 | 0.0001 |
| Japan (31) v. N-IO (37) | 0.3244 | 0.0001 |
| E-IO (186) v. W-PAC (38) | 0.2738 | 0.0001 |
| Fiji (9) v. W-PAC (38) | 0.1954 | 0.0018 |
| Fiji (9) v. N-IO (37) | 0.1814 | 0.0427 |

## 13.6 PCA

```
BS.haps <- strataG::labelHaplotypes(x = BS.bin, prefix = "HT",
                                    use.indels = TRUE)
BS.hapdist <- dist(BS.haps$hap.seqs)
table(BS.haps$haps)
x <- ape::pcoa(BS.hapdist, correction = "none", rn = NULL)
biplot(x, Y = NULL, plot.axes = c(1,2), dir.axis1 = 1,
       dir.axis2 = 1, rn = NULL, main = NULL)
```

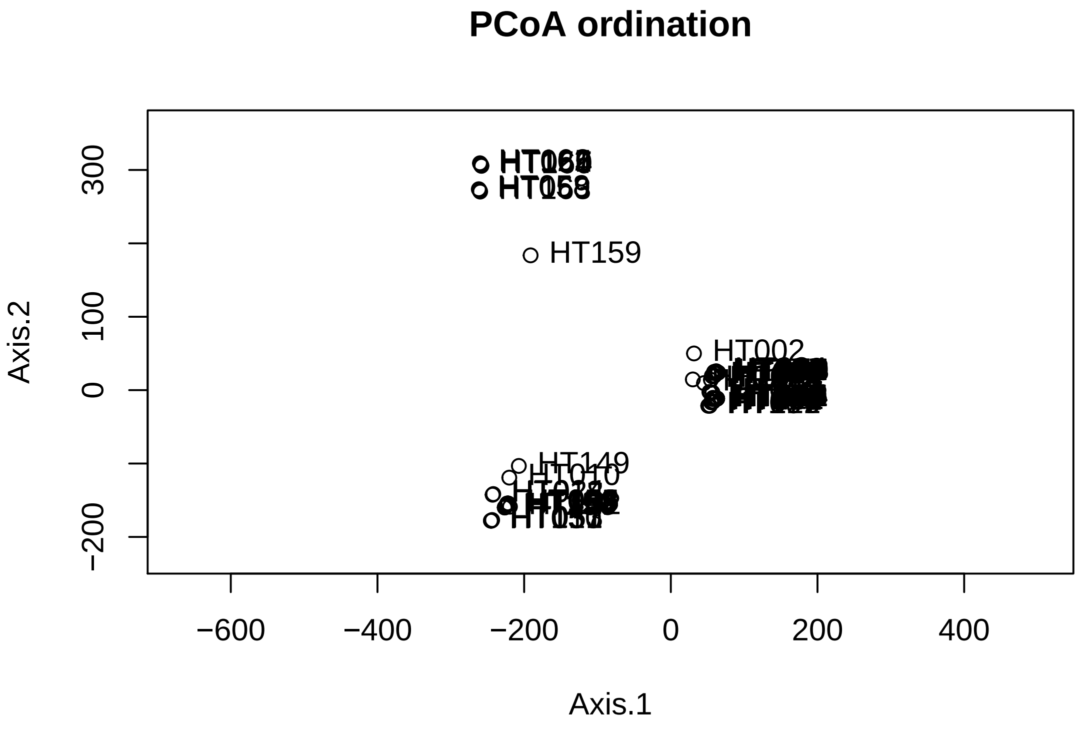

```r
pco1 <- dudi.pco(BS.hapdist, scannf = FALSE, nf = 2)
scatter(pco1, posi = "bottomleft")
```

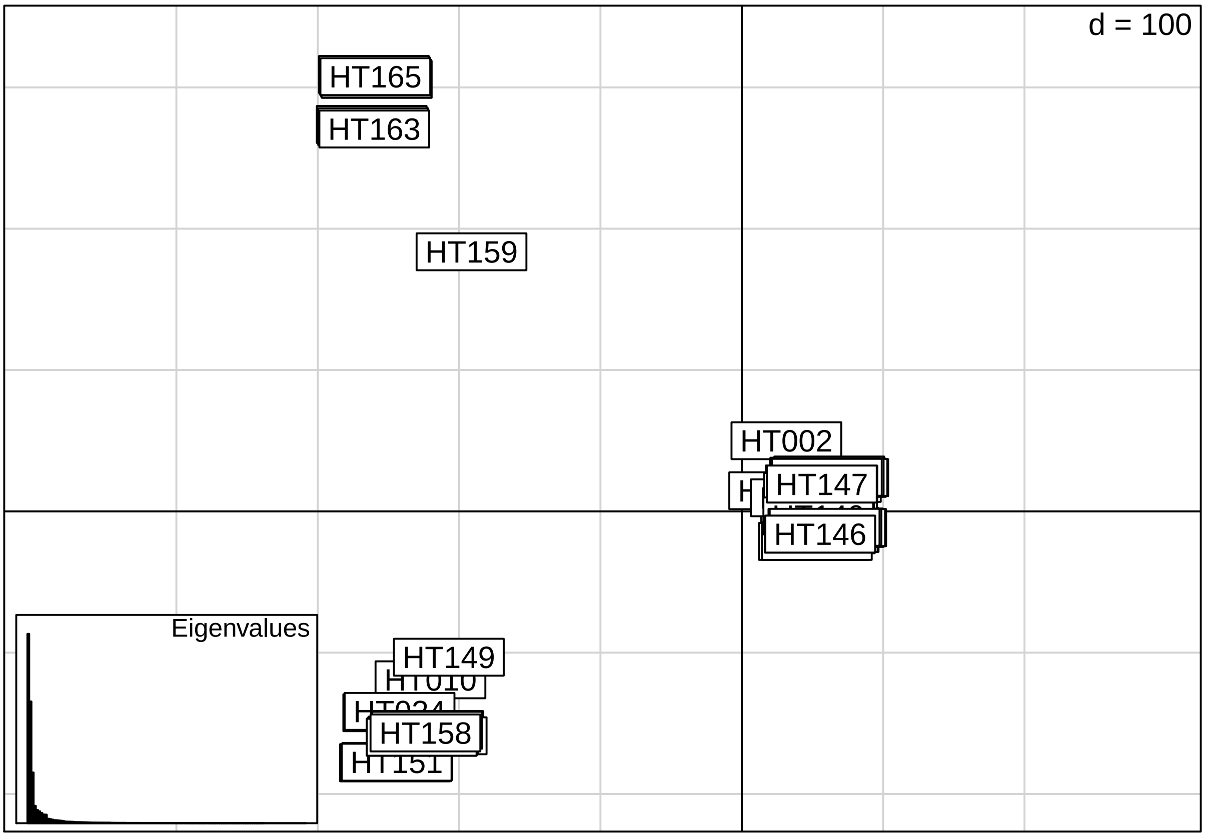


```
##
## HT001 HT002 HT003 HT004 HT005 HT006 HT007 HT008 HT009 HT010 HT011 HT012 HT013
##    19    13    12    11    10     9     8     7     7     7     6     5     5
## HT014 HT015 HT016 HT017 HT018 HT019 HT020 HT021 HT022 HT023 HT024 HT025 HT026
##     5     5     5     5     5     4     4     4     4     4     4     4     3
## HT027 HT028 HT029 HT030 HT031 HT032 HT033 HT034 HT035 HT036 HT037 HT038 HT039
##     3     3     3     3     3     3     3     3     3     3     3     3     2
## HT040 HT041 HT042 HT043 HT044 HT045 HT046 HT047 HT048 HT049 HT050 HT051 HT052
##     2     2     2     2     2     2     2     2     2     2     2     2     2
## HT053 HT054 HT055 HT056 HT057 HT058 HT059 HT060 HT061 HT062 HT063 HT064 HT065
##     2     2     2     2     2     2     2     1     1     1     1     1     1
## HT066 HT067 HT068 HT069 HT070 HT071 HT072 HT073 HT074 HT075 HT076 HT077 HT078
##     1     1     1     1     1     1     1     1     1     1     1     1     1
## HT079 HT080 HT081 HT082 HT083 HT084 HT085 HT086 HT087 HT088 HT089 HT090 HT091
##     1     1     1     1     1     1     1     1     1     1     1     1     1
## HT092 HT093 HT094 HT095 HT096 HT097 HT098 HT099 HT100 HT101 HT102 HT103 HT104
##     1     1     1     1     1     1     1     1     1     1     1     1     1
## HT105 HT106 HT107 HT108 HT109 HT110 HT111 HT112 HT113 HT114 HT115 HT116 HT117
##     1     1     1     1     1     1     1     1     1     1     1     1     1
## HT118 HT119 HT120 HT121 HT122 HT123 HT124 HT125 HT126 HT127 HT128 HT129 HT130
##     1     1     1     1     1     1     1     1     1     1     1     1     1
## HT131 HT132 HT133 HT134 HT135 HT136 HT137 HT138 HT139 HT140 HT141 HT142 HT143
##     1     1     1     1     1     1     1     1     1     1     1     1     1
## HT144 HT145 HT146 HT147 HT148 HT149 HT150 HT151 HT152 HT153 HT154 HT155 HT156
##     1     1     1     1     1     1     1     1     1     1     1     1     1
## HT157 HT158 HT159 HT160 HT161 HT162 HT163 HT164 HT165
##     1     1     1     1     1     1     1     1     1
```

# 14 References

Benestan, L., Moore, J. S., Sutherland, B. J. G., Le Luyer, J., Maaroufi, H., Rougeux, C., . . . Bernatchez, L. (2017). Sex matters in massive parallel sequencing: Evidence for biases in genetic parameter estimation and investigation of sex determination systems. Molecular Ecology, 26(24), 6767–6783. doi:10.1111/mec.14217

Devloo-Delva, F., Maes, G. E., Hernández, S. I., McAllister, J. D., Gunasekera, R. M., Grewe, P. M., . . . Feutry, P. (2019). Accounting for kin sampling reveals genetic connectivity in Tasmanian and New Zealand school sharks, Galeorhinus galeus. Ecology and Evolution, 9, 4465-4472. doi:10.1002/ece3.5012

Devloo-Delva, F., Kyne, P. M., Marthick, J. R., Grant, M. I., Gunasekera, R. M., Johnson, G. J., . . . Feutry, P. (under review). Sex-specific dispersal patterns of the threatened Northern River Shark. Heredity

Devloo-Delva, F., Gosselin, T., Butcher, P. A., Grewe, P. M., Huveneers, C., Thomson, R. B., . . . Feutry, P. (2022 preprint). An R-based tool for identifying sex-linked markers from Restriction Site-Associated DNA sequencing with applications to elasmobranch conservation. doi:Preprint at https://doi.org/10.21203/rs.3.rs-1797792/v1

Feutry, P., Berry, O., Kyne, P. M., Pillans, R. D., Hillary, R. M., Grewe, P. M., . . . Bravington, M. (2017). Inferring contemporary and historical genetic connectivity from juveniles. Molecular Ecology, 26(2), 444-456. doi:10.1111/mec.13929

Feutry, P., Devloo-Delva, F., Tran Lu Y, A., Mona, S., Gunasekera, R. M., Johnson, G., . . . Kyne, P. M. (2020). One panel to rule them all: DArTcap genotyping for population structure, historical demography, and kinship analyses, and its application to a threatened shark. Molecular Ecology Resources, 20, 1470–1485.

Foster, S. D., Feutry, P., Grewe, P. M., Berry, O., Hui, F. K., & Davies, C. R. (2018). Reliably discriminating stock structure with genetic markers: Mixture models with robust and fast computation. Molecular Ecology Resources, 1–16.

Puechmaille, S. J. (2016). The program structure does not reliably recover the correct population structure when sampling is uneven: subsampling and new estimators alleviate the problem. Molecular Ecology Resources, 16(3), 608-627.

Ritland, K. (1996). Estimators for pairwise relatedness and individual inbreeding coefficients. Genetics Research, 67(2), 175-185.

Tillett, B. J., Meekan, M. G., Field, I. C., Hua, Q., & Bradshaw, C. J. A. (2011). Similar life history traits in bull (Carcharhinus leucas) and pig-eye (C. amboinensis) sharks. Marine and Freshwater Research, 62(7), 850-860.

# 15 Citations for packages

```
packages <- c(
"radiator",
"ggplot2",
"dplyr",
"tidyverse",
"ade4",
"adegenet",
"ape",
"apex",
"assigner",
"assignPOP",
"dartR",
"haplotypes",
"data.table",
"ggplot2",
"grid",
```

```r
"gridExtra",
"gtools",
"kableExtra",
"knitr",
"magrittr",
"marmap",
"mvbutils",
"OutFLANK",
"pegas",
"qvalue",
"rubias",
"strataG",
"vegan"
)
for (P in packages) {
p <- utils::citation(P)
print(attr(unclass(p)[[1]], "textVersion"))
}
```

```
## [1] "Gosselin, T. (2020). radiator: RADseq Data Exploration, Manipulation and Visualization using
## [1] "H. Wickham. ggplot2: Elegant Graphics for Data Analysis. Springer-Verlag New York, 2016."
## [1] "Hadley Wickham, Romain François, Lionel Henry and Kirill Müller (2021). dplyr: A Grammar of
## [1] "Wickham et al., (2019). Welcome to the tidyverse. Journal of Open Source Software, 4(43), 16
## NULL
## [1] "Jombart, T. (2008) adegenet: a R package for the multivariate analysis of genetic markers. B
## [1] "Paradis E. & Schliep K. 2019. ape 5.0: an environment for modern phylogenetics and evolution
## [1] "Klaus Schliep, Thibaut Jombart, Zhian Namir Kamvar, Eric Archer and Rebecca Harris (2020). a
## [1] "Gosselin, T, Anderson, E. C., Bradbury, I. (2020). assigner: Assignment Analysis with GBS/RA
## [1] "Kuan-Yu Chen, Elizabeth A. Marschall, Michael G. Sovic, Anthony C. Fries, H. Lisle Gibbs and
## [1] "Gruber, B, Unmack, PJ, Berry, OF, Georges, A. dartr: An r package to facilitate analysis of
## [1] "Caner Aktas (2020). haplotypes: Manipulating DNA Sequences and Estimating Unambiguous Haplot
## [1] "Matt Dowle and Arun Srinivasan (2021). data.table: Extension of `data.frame`. R package vers
## [1] "H. Wickham. ggplot2: Elegant Graphics for Data Analysis. Springer-Verlag New York, 2016."
## [1] "R Core Team (2020). R: A language and environment for statistical computing. R Foundation fo
## [1] "Baptiste Auguie (2017). gridExtra: Miscellaneous Functions for \"Grid\" Graphics. R package
## [1] "Gregory R. Warnes, Ben Bolker and Thomas Lumley (2020). gtools: Various R Programming Tools.
## [1] "Hao Zhu (2021). kableExtra: Construct Complex Table with 'kable' and Pipe Syntax. R package
## [1] "Yihui Xie (2021). knitr: A General-Purpose Package for Dynamic Report Generation in R. R pac
## [1] "Stefan Milton Bache and Hadley Wickham (2020). magrittr: A Forward-Pipe Operator for R. R pa
## [1] "Eric Pante, Benoit Simon-Bouhet (2013) marmap: A Package for Importing, Plotting and Analyzi
## [1] "Mark V. Bravington (2018). mvbutils: Workspace Organization, Code and Documentation Editing,
## [1] "Michael C. Whitlock and Katie Lotterhos (2014). OutFLANK: Fst outliers with trimming. R pack
## [1] "Paradis E. 2010. pegas: an R package for population genetics with an integrated-modular appr
## [1] "John D. Storey, Andrew J. Bass, Alan Dabney and David Robinson (2020). qvalue: Q-value estim
## [1] "Moran, BM and Anderson, EC. 2018. Bayesian inference from the conditional genetic stock iden
## [1] "Archer, F. I., Adams, P. E. and Schneiders, B. B. (2016) strataG: An R package for manipulat
## [1] "Jari Oksanen, F. Guillaume Blanchet, Michael Friendly, Roeland Kindt, Pierre Legendre, Dan M
```

# 16 Session Info

```
sessioninfo::session_info()
```

```
## - Session info ---------------------------------------------------------------
##  setting  value
##  version  R version 4.0.2 (2020-06-22)
##  os       Ubuntu 18.04.1 LTS
##  system   x86_64, linux-gnu
##  ui       X11
##  language (EN)
##  collate  en_AU.UTF-8
##  ctype    en_AU.UTF-8
##  tz       Australia/Canberra
##  date     2022-07-11
##
## - Packages -------------------------------------------------------------------
##  package        * version    date       lib
##  ade4           * 1.7-15     2020-02-13 [2]
##  adegenet       * 2.1.3      2020-05-10 [2]
##  adehabitatMA     0.3.14     2020-01-13 [1]
##  ape            * 5.6-1      2022-01-07 [1]
##  apex           * 1.0.4      2020-04-11 [1]
##  assertthat       0.2.1      2019-03-21 [2]
##  assigner       * 0.5.8      2020-10-30 [1]
##  assignPOP      * 1.2.2      2020-11-17 [1]
##  backports        1.4.1      2021-12-13 [1]
##  bit              4.0.3      2020-07-30 [2]
##  bit64            0.9-7.1    2020-07-15 [2]
##  blob             1.2.1      2020-01-20 [2]
##  boot             1.3-25     2020-04-26 [2]
##  broom            0.7.6      2021-04-05 [1]
##  cachem           1.0.5      2021-05-15 [1]
##  calibrate        1.7.7      2020-06-19 [1]
##  callr            3.7.0      2021-04-20 [1]
##  caret            6.0-86     2020-03-20 [2]
##  cellranger       1.1.0      2016-07-27 [2]
##  class            7.3-17     2020-04-26 [2]
##  classInt         0.4-3      2020-04-07 [2]
##  cli              3.3.0      2022-04-25 [1]
##  cluster          2.1.0      2019-06-19 [2]
##  coda             0.19-4     2020-09-30 [1]
##  codetools        0.2-16     2018-12-24 [2]
##  colorspace       2.0-2      2021-06-24 [1]
##  combinat         0.0-8      2012-10-29 [2]
##  crayon           1.4.2      2021-10-29 [1]
##  dartR          * 1.9.9.1    2022-01-13 [1]
##  data.table     * 1.14.2     2021-09-27 [1]
##  DBI              1.1.2      2021-12-20 [1]
##  dbplyr           2.1.1      2021-04-06 [1]
##  deldir           1.0-6      2021-10-23 [1]
##  DEoptimR         1.0-8      2016-11-19 [2]
##  desc             1.3.0      2021-03-05 [2]
##  devtools         2.4.1      2021-05-05 [1]
##  digest           0.6.27     2020-10-24 [2]
##  dismo            1.3-3      2020-11-17 [1]
##  doParallel       1.0.15     2019-08-02 [2]
##  dplyr          * 1.0.6      2021-05-05 [1]
```

```
##  e1071           1.7-9      2021-09-16 [1]
##  ellipsis        0.3.2      2021-04-29 [1]
##  evaluate        0.14       2019-05-28 [2]
##  expm            0.999-6    2021-01-13 [1]
##  fansi           1.0.0      2022-01-10 [1]
##  farver          2.1.0      2021-02-28 [1]
##  fastmap         1.1.0      2021-01-25 [1]
##  fastmatch       1.1-0      2017-01-28 [2]
##  forcats       * 0.5.1      2021-01-27 [1]
##  foreach         1.5.0      2020-03-30 [2]
##  foreign       * 0.8-80     2020-05-24 [2]
##  formatR       * 1.7        2019-06-11 [2]
##  fossil        * 0.4.0      2020-03-23 [1]
##  fs              1.5.2      2021-12-08 [1]
##  gap             1.2.3-1    2021-04-21 [1]
##  gdata           2.18.0     2017-06-06 [2]
##  gdistance       1.3-6      2020-06-29 [1]
##  gdsfmt          1.26.1     2020-12-22 [1]
##  generics        0.1.1      2021-10-25 [1]
##  genetics        1.3.8.1.3  2021-03-01 [1]
##  GGally          2.0.0      2020-06-06 [2]
##  ggplot2       * 3.3.3      2020-12-30 [1]
##  glue            1.6.0      2021-12-17 [1]
##  gmodels         2.18.1     2018-06-25 [2]
##  gower           0.2.2      2020-06-23 [2]
##  gridExtra     * 2.3        2017-09-09 [2]
##  gtable          0.3.0      2019-03-25 [2]
##  gtools        * 3.8.2      2020-03-31 [2]
##  haplotypes    * 1.1.2      2020-02-28 [1]
##  haven           2.4.1      2021-04-23 [1]
##  hierfstat       0.5-10     2021-11-17 [1]
##  hms             1.1.0      2021-05-17 [1]
##  htmltools       0.5.1.1    2021-01-22 [1]
##  httpuv          1.5.4      2020-06-06 [2]
##  httr            1.4.2      2020-07-20 [2]
##  igraph          1.2.11     2022-01-04 [1]
##  ipred           0.9-9      2019-04-28 [2]
##  iterators       1.0.12     2019-07-26 [2]
##  jsonlite        1.7.2      2020-12-09 [1]
##  kableExtra    * 1.3.4      2021-02-20 [1]
##  KernSmooth      2.23-17    2020-04-26 [2]
##  knitr         * 1.33       2021-04-24 [1]
##  labeling        0.4.2      2020-10-20 [1]
##  later           1.1.0.1    2020-06-05 [2]
##  lattice       * 0.20-41    2020-04-02 [2]
##  lava            1.6.7      2020-03-05 [2]
##  LearnBayes      2.15.1     2018-03-18 [2]
##  lifecycle       1.0.0      2021-02-15 [1]
##  lubridate       1.7.10     2021-02-26 [1]
##  magrittr      * 2.0.1      2020-11-17 [1]
##  mapdata         2.3.0      2018-03-30 [2]
##  maps          * 3.3.0      2018-04-03 [2]
##  marmap        * 1.0.5      2020-11-19 [1]
##  MASS            7.3-51.6   2020-04-26 [2]
##  Matrix          1.2-18     2019-11-27 [2]
##  memoise         2.0.0      2021-01-26 [2]
##  mgcv            1.8-31     2019-11-09 [2]
##  mime            0.12       2021-09-28 [1]
```

```
##   mmod                1.3.3      2017-04-06 [1]
##   ModelMetrics        1.2.2.2    2020-03-17 [2]
##   modelr              0.1.8      2020-05-19 [2]
##   munsell             0.5.0      2018-06-12 [2]
##   mvbutils          * 2.8.372    2021-05-18 [1]
##   mvtnorm             1.1-1      2020-06-09 [2]
##   ncdf4               1.17       2019-10-23 [2]
##   network             1.16.0     2019-12-01 [2]
##   nlme                3.1-148    2020-05-24 [2]
##   nnet                7.3-14     2020-04-26 [2]
##   OutFLANK          * 0.2        2020-10-30 [1]
##   pegas             * 1.0-1      2021-05-17 [1]
##   permute           * 0.9-5      2019-03-12 [2]
##   phangorn          * 2.5.5      2019-06-19 [2]
##   pillar              1.6.1      2021-05-16 [1]
##   pkgbuild            1.2.0      2020-12-15 [2]
##   pkgconfig           2.0.3      2019-09-22 [2]
##   pkgload             1.2.1      2021-04-06 [2]
##   plotrix             3.8-2      2021-09-08 [1]
##   plyr                1.8.6      2020-03-03 [2]
##   png                 0.1-7      2013-12-03 [2]
##   PopGenReport        3.0.4      2019-02-04 [1]
##   prettyunits         1.1.1      2020-01-24 [2]
##   pROC                1.16.2     2020-03-19 [2]
##   processx            3.5.2      2021-04-30 [1]
##   prodlim             2019.11.13 2019-11-17 [2]
##   promises            1.2.0.1    2021-02-11 [1]
##   proxy               0.4-26     2021-06-07 [1]
##   ps                  1.6.0      2021-02-28 [1]
##   purrr             * 0.3.4      2020-04-17 [2]
##   quadprog            1.5-8      2019-11-20 [2]
##   qvalue            * 2.20.0     2020-04-27 [2]
##   R.cache             0.15.0     2021-04-30 [1]
##   R.methodsS3         1.8.1      2020-08-26 [1]
##   R.oo                1.24.0     2020-08-26 [1]
##   R.utils             2.11.0     2021-09-26 [1]
##   R6                  2.5.1      2021-08-19 [1]
##   radiator          * 1.1.9      2021-05-18 [1]
##   randomForest        4.6-14     2018-03-25 [2]
##   raster              3.4-10     2021-05-03 [1]
##   RColorBrewer        1.1-2      2014-12-07 [2]
##   Rcpp                1.0.7      2021-07-07 [1]
##   RcppParallel        5.0.2      2020-06-24 [2]
##   readr             * 1.4.0      2020-10-05 [1]
##   readxl              1.3.1      2019-03-13 [2]
##   recipes             0.1.13     2020-06-23 [2]
##   remotes             2.3.0      2021-04-01 [1]
##   reprex              2.0.0      2021-04-02 [1]
##   reshape             0.8.8      2018-10-23 [2]
##   reshape2            1.4.4      2020-04-09 [2]
##   rgdal               1.5-28     2021-12-15 [1]
##   RgoogleMaps         1.4.5.3    2020-02-12 [1]
##   rlang               0.4.11     2021-04-30 [1]
##   rmarkdown           2.8        2021-05-07 [1]
##   robustbase          0.93-6     2020-03-23 [2]
##   rpart               4.1-15     2019-04-12 [2]
##   rprojroot           2.0.2      2020-11-15 [1]
##   RSQLite             2.2.0      2020-01-07 [2]
```

```
##  rstudioapi       0.13       2020-11-12 [1]
##  rubias         * 0.3.2      2021-01-15 [1]
##  rvest            1.0.0      2021-03-09 [1]
##  scales           1.1.1      2020-05-11 [2]
##  seqinr           3.6-1      2019-09-07 [2]
##  sessioninfo      1.1.1      2018-11-05 [2]
##  sf               1.0-3      2021-10-07 [1]
##  shape            1.4.4      2018-02-07 [2]
##  shapefiles     * 0.7        2013-01-26 [2]
##  shiny            1.5.0      2020-06-23 [2]
##  sna              2.5        2019-12-10 [2]
##  SNPRelate        1.22.0     2020-04-27 [1]
##  sp             * 1.4-5      2021-01-10 [1]
##  spData           0.3.8      2020-07-03 [2]
##  spdep            1.1-5      2020-06-29 [2]
##  StAMPP           1.6.2      2021-04-26 [1]
##  statnet.common   4.3.0      2019-06-02 [2]
##  strataG        * 2.5.01     2021-03-23 [1]
##  stringi          1.6.2      2021-05-17 [1]
##  stringr        * 1.4.0      2019-02-10 [2]
##  styler         * 1.7.0      2022-03-13 [1]
##  survival         3.1-12     2020-04-10 [2]
##  svglite          2.0.0      2021-02-20 [1]
##  swfscMisc        1.4.3      2021-03-15 [1]
##  systemfonts      1.0.2      2021-05-11 [1]
##  testthat         3.0.2      2021-02-14 [2]
##  tibble         * 3.1.2      2021-05-16 [1]
##  tidyr          * 1.1.3      2021-03-03 [1]
##  tidyselect       1.1.1      2021-04-30 [1]
##  tidyverse      * 1.3.1      2021-04-15 [1]
##  timeDate         3043.102   2018-02-21 [2]
##  tree             1.0-40     2019-04-26 [2]
##  units            0.7-2      2021-06-08 [1]
##  usethis          2.0.1      2021-02-10 [2]
##  utf8             1.2.1      2021-03-12 [1]
##  vctrs            0.3.8      2021-04-29 [1]
##  vegan          * 2.5-7      2020-11-28 [1]
##  viridisLite      0.4.0      2021-04-13 [1]
##  webshot          0.5.2      2019-11-22 [1]
##  withr            2.4.2      2021-04-18 [1]
##  xfun             0.31       2022-05-10 [1]
##  xml2             1.3.2      2020-04-23 [2]
##  xtable           1.8-4      2019-04-21 [2]
##  yaml             2.2.1      2020-02-01 [2]
##  source
##  CRAN (R 4.0.2)
##  CRAN (R 4.0.2)
##  CRAN (R 4.0.2)
##  CRAN (R 4.0.2)
##  CRAN (R 4.0.2)
##  CRAN (R 4.0.2)
##  Github (thierrygosselin/assigner@d39a692)
##  CRAN (R 4.0.2)
##  CRAN (R 4.0.2)
##  CRAN (R 4.0.2)
##  CRAN (R 4.0.2)
##  CRAN (R 4.0.2)
##  CRAN (R 4.0.2)
```

```
##  CRAN (R 4.0.2)
##  CRAN (R 4.0.2)
##  CRAN (R 4.0.2)
##  CRAN (R 4.0.2)
##  CRAN (R 4.0.2)
##  CRAN (R 4.0.2)
##  CRAN (R 4.0.2)
##  CRAN (R 4.0.2)
##  CRAN (R 4.0.2)
##  CRAN (R 4.0.2)
##  CRAN (R 4.0.2)
##  CRAN (R 4.0.2)
##  CRAN (R 4.0.2)
##  CRAN (R 4.0.2)
##  CRAN (R 4.0.2)
##  Github (green-striped-gecko/dartR@3022577)
##  CRAN (R 4.0.2)
##  CRAN (R 4.0.2)
##  CRAN (R 4.0.2)
##  CRAN (R 4.0.2)
##  CRAN (R 4.0.2)
##  CRAN (R 4.0.2)
##  CRAN (R 4.0.2)
##  CRAN (R 4.0.2)
##  CRAN (R 4.0.2)
##  CRAN (R 4.0.2)
##  CRAN (R 4.0.2)
##  CRAN (R 4.0.2)
##  CRAN (R 4.0.2)
##  CRAN (R 4.0.2)
##  CRAN (R 4.0.2)
##  CRAN (R 4.0.2)
##  CRAN (R 4.0.2)
##  CRAN (R 4.0.2)
##  CRAN (R 4.0.2)
##  CRAN (R 4.0.2)
##  CRAN (R 4.0.2)
##  CRAN (R 4.0.2)
##  CRAN (R 4.0.2)
##  CRAN (R 4.0.2)
##  CRAN (R 4.0.2)
##  CRAN (R 4.0.2)
##  CRAN (R 4.0.2)
##  CRAN (R 4.0.2)
##  Bioconductor
##  CRAN (R 4.0.2)
##  CRAN (R 4.0.2)
##  CRAN (R 4.0.2)
##  CRAN (R 4.0.2)
##  CRAN (R 4.0.2)
##  CRAN (R 4.0.2)
##  CRAN (R 4.0.2)
##  CRAN (R 4.0.2)
##  CRAN (R 4.0.2)
##  CRAN (R 4.0.2)
##  CRAN (R 4.0.2)
##  CRAN (R 4.0.2)
##  CRAN (R 4.0.2)
```

```
##  CRAN (R 4.0.2)
##  CRAN (R 4.0.2)
##  CRAN (R 4.0.2)
##  CRAN (R 4.0.2)
##  CRAN (R 4.0.2)
##  CRAN (R 4.0.2)
##  CRAN (R 4.0.2)
##  CRAN (R 4.0.2)
##  CRAN (R 4.0.2)
##  CRAN (R 4.0.2)
##  CRAN (R 4.0.2)
##  CRAN (R 4.0.2)
##  CRAN (R 4.0.2)
##  CRAN (R 4.0.2)
##  CRAN (R 4.0.2)
##  CRAN (R 4.0.2)
##  CRAN (R 4.0.2)
##  CRAN (R 4.0.2)
##  CRAN (R 4.0.2)
##  CRAN (R 4.0.2)
##  CRAN (R 4.0.2)
##  CRAN (R 4.0.2)
##  CRAN (R 4.0.2)
##  CRAN (R 4.0.2)
##  CRAN (R 4.0.2)
##  CRAN (R 4.0.2)
##  CRAN (R 4.0.2)
##  CRAN (R 4.0.2)
##  CRAN (R 4.0.2)
##  CRAN (R 4.0.2)
##  CRAN (R 4.0.2)
##  local
##  CRAN (R 4.0.2)
##  CRAN (R 4.0.2)
##  CRAN (R 4.0.2)
##  CRAN (R 4.0.2)
##  CRAN (R 4.0.2)
##  Github (whitlock/OutFLANK@e502e82)
##  CRAN (R 4.0.2)
##  CRAN (R 4.0.2)
##  CRAN (R 4.0.2)
##  CRAN (R 4.0.2)
##  CRAN (R 4.0.2)
##  CRAN (R 4.0.2)
##  CRAN (R 4.0.2)
##  CRAN (R 4.0.2)
##  CRAN (R 4.0.2)
##  CRAN (R 4.0.2)
##  CRAN (R 4.0.2)
##  CRAN (R 4.0.2)
##  CRAN (R 4.0.2)
##  CRAN (R 4.0.2)
##  CRAN (R 4.0.2)
##  CRAN (R 4.0.2)
##  CRAN (R 4.0.2)
##  CRAN (R 4.0.2)
##  CRAN (R 4.0.2)
##  CRAN (R 4.0.2)
```

```
##   Bioconductor
##   CRAN (R 4.0.2)
##   CRAN (R 4.0.2)
##   CRAN (R 4.0.2)
##   CRAN (R 4.0.2)
##   CRAN (R 4.0.2)
##   Github (thierrygosselin/radiator@5c6b865)
##   CRAN (R 4.0.2)
##   CRAN (R 4.0.2)
##   CRAN (R 4.0.2)
##   CRAN (R 4.0.2)
##   CRAN (R 4.0.2)
##   CRAN (R 4.0.2)
##   CRAN (R 4.0.2)
##   CRAN (R 4.0.2)
##   CRAN (R 4.0.2)
##   CRAN (R 4.0.2)
##   CRAN (R 4.0.2)
##   CRAN (R 4.0.2)
##   CRAN (R 4.0.2)
##   CRAN (R 4.0.2)
##   CRAN (R 4.0.2)
##   CRAN (R 4.0.2)
##   CRAN (R 4.0.2)
##   CRAN (R 4.0.2)
##   CRAN (R 4.0.2)
##   CRAN (R 4.0.2)
##   CRAN (R 4.0.2)
##   CRAN (R 4.0.2)
##   CRAN (R 4.0.2)
##   CRAN (R 4.0.2)
##   CRAN (R 4.0.2)
##   CRAN (R 4.0.2)
##   CRAN (R 4.0.2)
##   CRAN (R 4.0.2)
##   CRAN (R 4.0.2)
##   CRAN (R 4.0.2)
##   CRAN (R 4.0.2)
##   Bioconductor
##   CRAN (R 4.0.2)
##   CRAN (R 4.0.2)
##   CRAN (R 4.0.2)
##   CRAN (R 4.0.2)
##   CRAN (R 4.0.2)
##   Github (ericarcher/strataG@0b78e7c)
##   CRAN (R 4.0.2)
##   CRAN (R 4.0.2)
##   CRAN (R 4.0.2)
##   CRAN (R 4.0.2)
##   CRAN (R 4.0.2)
##   CRAN (R 4.0.2)
##   CRAN (R 4.0.2)
##   CRAN (R 4.0.2)
##   CRAN (R 4.0.2)
##   CRAN (R 4.0.2)
##   CRAN (R 4.0.2)
##   CRAN (R 4.0.2)
##   CRAN (R 4.0.2)
```

```
##  CRAN (R 4.0.2)
##  CRAN (R 4.0.2)
##  CRAN (R 4.0.2)
##  CRAN (R 4.0.2)
##  CRAN (R 4.0.2)
##  CRAN (R 4.0.2)
##  CRAN (R 4.0.2)
##  CRAN (R 4.0.2)
##  CRAN (R 4.0.2)
##  CRAN (R 4.0.2)
##  CRAN (R 4.0.2)
##  CRAN (R 4.0.2)
##  CRAN (R 4.0.2)
##
## [1] /home/dev093/R/x86_64-pc-linux-gnu-library/4.0
## [2] /apps/R/4.0.2/lib/R/library
```
